# Systematic Analysis and Identification of Molecular Subtypes of TRP-Related Genes and Prognosis Prediction in Lung Adenocarcinoma

**DOI:** 10.1155/2022/5388283

**Published:** 2022-08-31

**Authors:** Yang Guo, Ning Liu

**Affiliations:** Shenyang Tenth People's Hospital (Shenyang Chest Hospital), No 11 Beihai Street, Dadong District, Shenyang 110044, Liaoning, China

## Abstract

**Background:**

Transient receptor potential channel (TRP) is a superfamily of nonselective cation channels, which is a member of calcium ion channels with a vital role in different calcium ion signal transduction pathways. TRP channel expression is often changed in the tumor, although the role of TRP proteins in lung cancer is unknown.

**Methods:**

Molecular Signatures Database (MsigDB) provided the TRP gene set. Univariate Cox regression analysis was performed on The Cancer Genome Atlas Lung Adenocarcinoma (TCGA-LUAD) data collection set employing the coxph function of R package survival to find prognosis-related genes. The R package ConsumusClusterPlus was employed for doing the consistency cluster analysis of TCGA-LUAD samples according to the prognosis-related TRP gene. The R-package limma was utilized for investigating the differential expression of TRP subtypes. According to the differentially expressed genes between subtypes, the least absolute shrinkage and selection operator (LASSO) regression was employed to find the major genes and develop the risk model. CIBERPORT algorithm, R package maftools, gene set variation analysis (GSVA), and pRRophetic of R-package were employed for measuring the proportion of immune cells among subtypes, genomic mutation difference, pathway enrichment score, and drug sensitivity analysis.

**Results:**

A total of 15 TRP-related genes associated with the prognosis of lung adenocarcinoma were found. According to the expression value of 15 genes, lung adenocarcinoma can be sorted into two subcategories. The prognosis of cluster1 is considerably better in comparison with that of cluster2. There were 123 differentially expressed genes between C1 and C2 subtypes, including 6 up- and 117 downregulated genes. There were major variations in the tumor microenvironment between C1 and C2 subtypes. The proportion of CD8 T cells in the C1 subtype was considerably enhanced in comparison with that in the C2 subtype. We further discovered 123 differentially expressed genes among subtypes, and 8 key genes were obtained at the end. The risk score (RS) model developed by the 8-gene signature had good strength in the TCGA validation set, overall set, and Gene Expression Omnibus (GEO) external dataset. There were major variations in immune checkpoint gene expression, patient sensitivity to immunotherapeutic drugs, immune infiltration, and genomic mutations between high and low groups on the basis of RS.

**Conclusions:**

The risk model developed on the basis of TRP-related genes can help in predicting the prognosis of patients suffering from lung adenocarcinoma and guide immunotherapy.

## 1. Introduction

Lung cancer is a widely known clinical malignancy and the primary cause of cancer-related deaths around the world. It is categorized into small cell lung cancer (SCLC) and non-small cell lung cancer (NSCLC). The most widely known subtype of NSCLC is lung adenocarcinoma [1]. The symptoms of lung adenocarcinoma at the start of the disease are not prominent and can be ignored easily. Most patients are diagnosed after they have reached the advanced stage of the disease, and the five-year survival rate is usually only 20%–30% [2]. The advanced treatment of lung adenocarcinoma includes chemotherapy, immune checkpoint inhibitor therapy, radiotherapy, and molecular targeted therapy. Although the emergence of molecularly targeted drugs such as aletinib and gefitinib greatly prolonged the survival rate of lung adenocarcinoma patients, many of them indicated varying degrees of drug resistance due to the high heterogeneity of tumors [3, 4]. Hence, finding the molecular targets and prognostic markers for various subtypes of lung adenocarcinoma has great importance for the prognosis prediction and clinical monitoring of lung adenocarcinoma.

The transient receptor potential (TRP) channel has many physiological functions. It can work as a receptor for different intracellular and external environmental signals. After being activated by temperature or corresponding ligands, it mediates Ca^2+^ and other cations into cells to regulate physiological activities [5]. Moreover, TRP channels are key players in regulating mineral absorption, intestinal peristalsis, body fluid balance, blood circulation, bladder and airway hypersensitivity, cell growth, and survival [6]. Recently, in the field of tumor therapy, TRP protein has become a kind of “Star” therapeutic target [7]. Numerous studies have revealed that TRP channels can interfere with major tumor signal transduction pathways through Ca^2+^ signal transduction dysfunction, thus affecting tumor cell proliferation, apoptosis, angiogenesis, gene transcription, etc. [8–10]. However, the mechanism of TRP protein in lung adenocarcinoma is yet to be understood.

In this report, researchers used a variety of commonly known bioinformatics analysis tools to investigate the relationship between TRP gene expression disturbance and lung adenocarcinoma prognosis on multiple levels by collecting lung adenocarcinoma samples from TCGA and GEO datasets, aiming at the expression and mutation of TRP gene set in the samples, combined with survival information and other clinical data. Finally, an RS model for evaluating lung cancer prognosis was developed, and the model's good and stable evaluation efficiency was proven.

## 2. Materials and Methods

### 2.1. Dataset Source and Preprocessing

We used the R-package TCGAbiolinks to download the expression profile data (FPKM value) and clinical information of LUAD in TCGA. The FPKM value was log2-converted, and the unified survival time unit—day, was adopted when processing survival information at the same time. The expression data and clinical data of GSE72094 and GSE68465 were taken from GEO (https://www.ncbi.nlm.nih.gov/geo/) database and processed as follows: (1) samples without information regarding clinical follow-up were excluded; (2) samples with unknown survival time or those with less than 0 days and no survival status were excluded, and days was set as the unified survival time unit; (3) the probe was converted to gene symbol; (4) the probe corresponded to multiple genes was excluded; (5) the expression with multiple gene symbols was considered the median value. The expression profile, survival, and response data of the IMvigor210 immunotherapy cohort (bladder cancer) were provided by the R package IMvigor210CoreBiologies. TRP-related gene sets were from the REACTOME_TRP_CHANNELS pathway in the MsigDB database and the inflammatory mediator regulation of TRP channel pathway in the Kyoto Encyclopedia of Genes and Genomes (KEGG) database, and the gene union of the two pathways was considered the TRP gene set.

### 2.2. Unsupervised Clustering of TRP-Related Genes

We carried out a univariate Cox analysis of the TRP gene with the coxph function of R package survival to find (*P* < 0.05) prognosis-related genes to prognosis. Then, based on the TRP-related genes related to prognosis, the consistency cluster analysis of LUAD samples was carried out using R package ConsumusClusterPlus. To achieve classification stability, an 80% resampling rate was used and 1000 repetitions were performed. Afterward, using R package survival, the KM survival curve of the classified patients was constructed, and we determined the importance of the prognostic difference between the classification utilizing the log-rank test. Finally, TRP molecular subtypes were chosen based on the clustering outcomes that had a good clustering effect and significant variations in survival among subtypes.

### 2.3. Differential Expression Analysis of TRP Molecular Subtypes

Based on the consistency cluster analysis, LUAD samples were categorized into two molecular subtypes: C1 and C2. We assessed the differential expression of TRP subtypes using R package limma, and the *P*-value corrected by Benjamin–Hochberg (FDR) was adj.*P*-value <0.05 and ｜log2FC｜>1 were considered thresholds to find differentially expressed genes.

### 2.4. Construction of Prognostic Risk Model and Analysis of Survival Differences

The signature related to lung adenocarcinoma's prognosis was identified (*P* < 0.05) using a univariate Cox analysis of differentially expressed genes among subtypes. Then, the major prognostic genes were then identified using the LASSO regression of R package glmnet, and the prognostic model was made. The tumor samples were sorted into high- and low-risk groups using the median RS as the threshold point. The prognostic analytic survival curve was created using the Kaplan–Meier method, and the importance of the variation was found by the log-rank test. The ROC (receiver operating characteristic) curve was then generated using the R package timeROC for evaluating the disturbance scoring model's scoring prediction; the R package ggplot2 was employed for drawing the scatter diagram of survival time and state, as well as the scatter diagram of sample score. An expression heat map for the model gene was created using the R package pheatmap. The risk value in the model was the sum of each candidate gene expression value multiplied by the weight. The formula is as follows:

(1) $$\begin{array}{c} \text{RiskScore}=\sum_{i=0}^{n}\text{coef}\left(i\right)*\text{Exp}\left(i\right). \end{array}$$

### 2.5. Estimation of Proportion of Immune Infiltrating Cells and Immune Score

The CIBERPORT algorithm of R package IOBR was used according to the expression profile of the TCGA-LUAD dataset for measuring the proportion of immune infiltrating cells. The CIBERPORT algorithm [11] was a method to characterize the composition of cells based on the gene expression profile of complex tissues. We used the leukocyte characteristic gene matrix LM22 composed of 547 genes to differentiate 22 immune cell types, including myeloid subsets, plasma cells, naive and memory B cells, natural killer (NK) cells, and seven T cell types. CIBERPORT in combination with the LM22 characteristic matrix was employed for estimating the proportion of 22 cell phenotypes in the sample. The sum of the proportions of all immune cell types in each sample was equal to 1. Moreover, the ESTIMATE algorithm was employed for measuring the tumor purity, immune score, matrix score, and estimate score of the tumor.

### 2.6. Genomic Mutation Analysis

A waterfall diagram was drawn using the R Package maftools and clinical grouping information to depict the variation distribution of genes with high somatic mutation frequency in lung cancer samples, and the data were sorted using model grouping information. To investigate the association between model grouping and tumor TMB, the tumor mutation burden (TMB) of each sample was assessed at the same time.

### 2.7. Enrichment Analysis of GSVA HALLMARKER Pathway

The ssGSEA algorithm of R package GSVA was employed for computing the enrichment scores of 50 HALLMARKER pathways for each sample according to the gene expression of lung cancer samples. The enrichment score difference across subtypes and model groups was obtained using a statistical test, and the enrichment score heat map was created using the R package pheatmap and the clinical features of the data.

### 2.8. Drug Sensitivity Analysis

The R package pRRophetic and model gene expression data were employed to predict the sensitivity (IC50 value) of 138 medications in the Genomics of Drug Sensitivity in Cancer (GDSC) database [12]. The IC50 number was used to determine how sensitive lung adenocarcinoma patients were to medication treatment. The Wilcoxon test examined the variations in IC50 values between high- and low-risk groups, and medications with significant variations between the two groups were discovered.

### 2.9. Statistical Test

The Wilcoxon test was used in the significance labeling for comparing the differences between the two groups of samples, and Kruskal–Wallis helped in comparing the differences between multiple groups of samples, where ns indicates *P* > 0.05, ^∗^ indicates *P* ≤ 0.05, ^∗∗^ indicates *P* ≤ 0.01, ^∗∗∗^ indicates *P* ≤ 0.001, and ^∗∗∗∗^ indicates *P* ≤ 0.0001. Among them, *P* < 0.05 was significant, and the difference was statistically significant.

## 3. Results

### 3.1. Identification of TRP-Related Genes Linked with LUAD Prognosis

**Figure 1:**
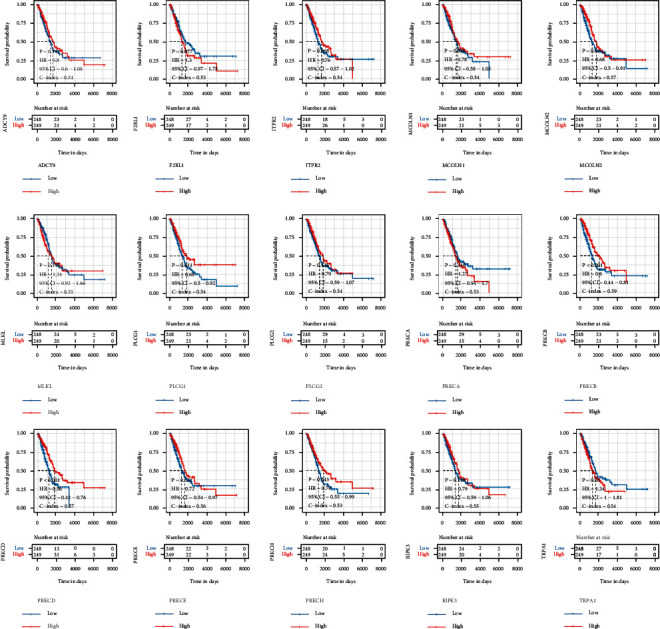
Survival curves of 15 TRP genes linked with LUAD prognosis. Red represents the high expression group, and blue represents the low expression group.

TRP genes were studied using a univariate Cox analysis, and 15 TRP genes (Table S1) were shown to be linked with the prognosis of lung cancer (*P* < 0.05). The median gene expression was then used as a cutoff point, the lung cancer samples were separated into high and low groups, and a Kaplan–Meier survival curve was created.  Figure 1 shows that high expression of genes MCOLN2, PLCG1, PRKCB, PRKCD, PRKCE, and PRKCH was linked with a better prognosis, while low expression of TRPA1 was associated with a worse prognosis.

### 3.2. Molecular Subtypes of TRP-Related Genes

**Figure 2:**
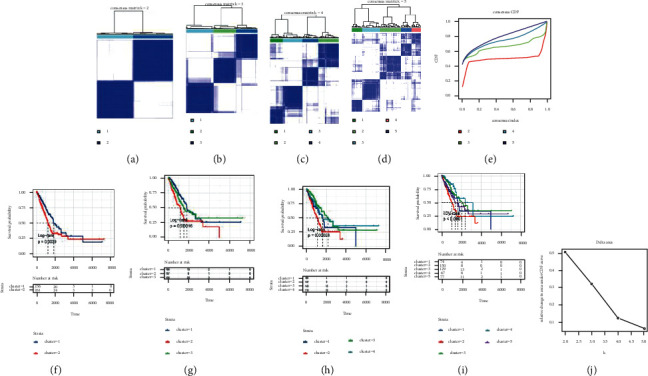
TRP molecular subtype recognition outcomes and survival differences among subtypes. (a)–(d): Clustering outcomes when the classification number *k* = 2, *k* = 3, *k* = 4, and *k* = 5; (f)–(i): survival curve when classification number *k* = 2, *k* = 3, *k* = 4, and *k* = 5; (e) CDF curve distribution of consistent clustering; (j) the area distribution under the CDF curve of consistent clustering.

We performed the consistency clustering analysis according to the expression values of 15 TRP-associated genes in each tumor sample of the TCGA-LUAD dataset. The clustering effect was best when the clustering algorithm was KM and the distance was Euclidean, *t*: best*K* = 2 (Figure 2(a)).  Table S2 shows the clustering outcomes of samples. The cumulative distribution function (CDF) of consistency clustering was illustrated in Figure 2(e), which indicated the cumulative distribution function when K was different values. Figure 2(j) highlights the change of area under the CDF curve when *K* is relative to *K *−* *1. Finally, two independent TRP subtypes with major survival variations were identified, and the prognosis of cluster1 was much better than that of cluster2, as demonstrated in Figure 2(f).

### 3.3. Differential Analysis of TRP Molecular Subtypes

#### 3.3.1. Identification of Differentially Expressed Genes

**Figure 3:**
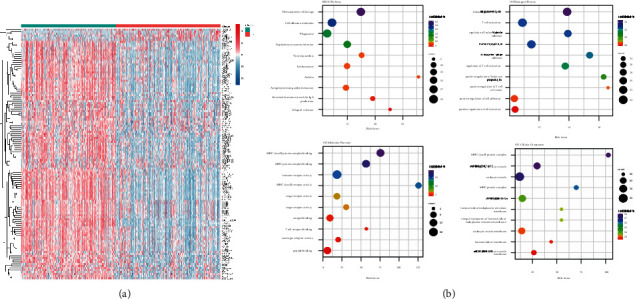
Gene identification and functional enrichment analysis of differential gene expression in TRP subtypes. (a) Heat map of differentially expressed genes in TRP subtypes; (b) bubble diagram of enrichment pathway of KEGG, BP (biological process), MF (molecular function), and CC (cell component) of differentially expressed genes. The size of the dot indicated the number of genes enriched to the difference, and the color represents the significance of enrichment.

The differential expression of TRP subtypes was investigated using the TCGA-LUAD dataset's consistent clustering results (Table S3). Finally, 123 genes were found to be significantly differentially expressed, with 6 differentially expressed upregulated genes and 117 differentially expressed downregulated genes.  Figure 3(a) shows a heat map of the expression distribution of differentially expressed genes across subtypes. The top 10 entries with substantial enrichment results were selected to build bubble charts, and KEGG enrichment analysis and carried out Gene Ontology (GO) functional enrichment analysis for the differentially expressed genes among the identified subtypes.  Figure 3(b) depicts the outcomes.

#### 3.3.2. Differences in Pathway Enrichment and Immune Infiltrating Cells among TRP Subtypes

**Figure 4:**
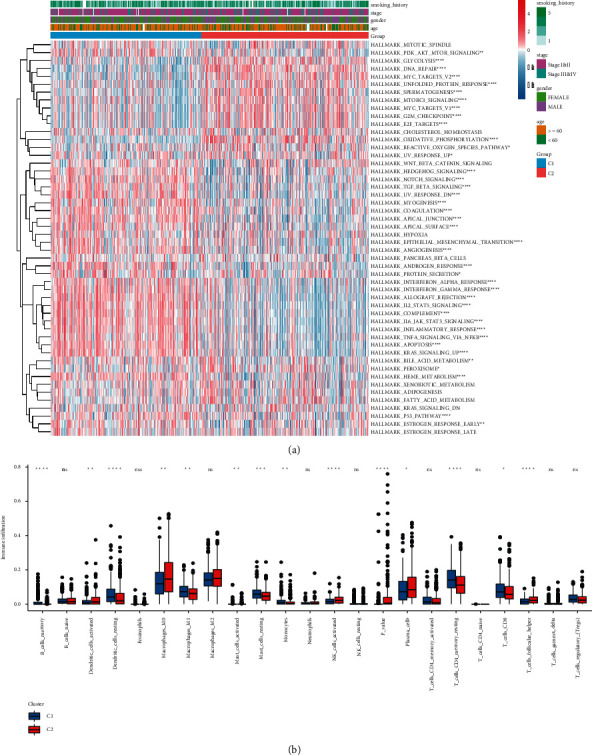
TRP subtype HALLMARKER pathway enrichment difference and immune infiltrating cell difference. (a) HALLMARKER pathway enrichment score heatmap; (b) box diagram of difference in immune infiltration of TRP subtypes.

To study the biological importance of TRP disorder, we used GSVA to estimate the HALLMARKER pathway enrichment score of each tumor sample in the TRP subtype (Table S4). Afterward, combined with the grouping data of clinical features, we drew the heat map of pathway enrichment scores, and the pathways with major variations in TRP subtype enrichment scores were marked with “^∗^”. The outcomes are highlighted in Figure 4(a): 40 HALLMARKER pathways had considerable enrichment variations among TRP subtypes. Meanwhile, the difference in tumor immune microenvironment between TRP subtypes was found by estimating the proportion of immune cell infiltration (Table S5).  Figure 4(b) illustrated the outcomes.

#### 3.3.3. Model construction and validation of differential genes among TRP subtypes

**Figure 5:**
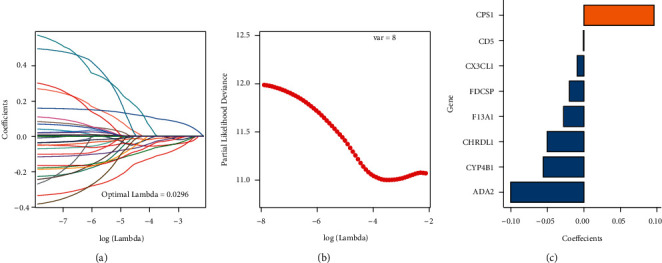
LASSO result diagram of TCGA training set. (a) The changing track of the LASSO regressed independent variable, the abscissa representing the logarithm of the independent variable lambda, and the ordinate representing the coefficient of the independent variable; (b) the confidence interval under each Lambda of LASSO; (c) LASSO regression coefficient of the key prognostic gene.

To begin, the entire TCGA-LUAD dataset (*n* = 497) was categorized into a training set (*n* = 249) and a test set (*n* = 248) in a 1 : 1 ratio. The differentially expressed genes of 123 TRP subtypes were discovered in the training set by univariate Cox analysis (*P* < 0.05), and finally, 32 genes linked to lung adenocarcinoma prognosis were obtained (Table S6). Then, after removing duplicated genes with LASSO linear regression, seed = 2110, construct TRPRS was built, and 8 prognosis-associated signatures were discovered. Figures 5(a) and 5(b) show the LASSO results, while Table S6 shows the gene symbol and weight coefficient.

**Figure 6:**
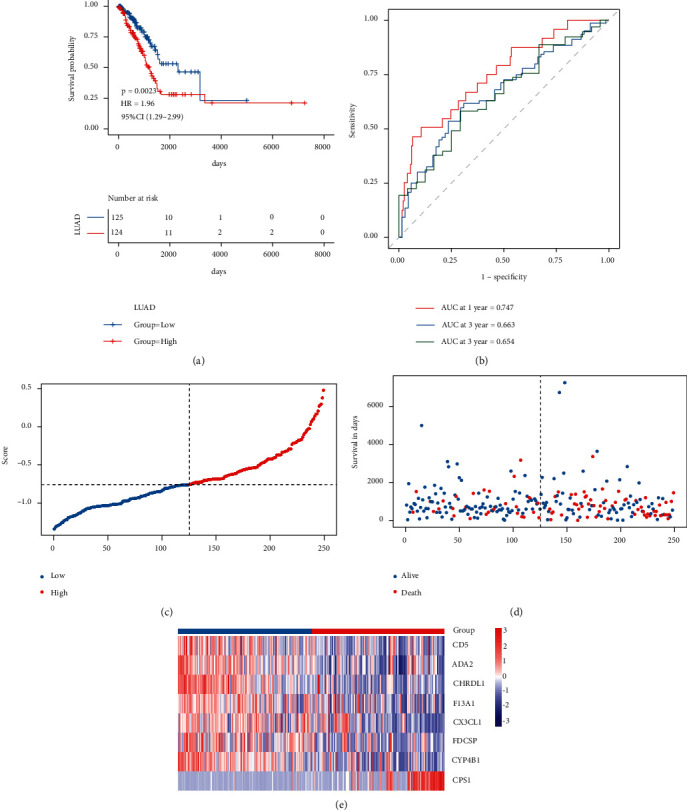
The verified prognostic efficacy of the model in the TCGA training set. (a) KM curve of TCGA training set; (b) ROC curve; (c)–(e): risk triple plot, including risk dispersion plot, survival time scatter plot, and heat map of model gene expression in RS grouping. Red represents the high-risk group and blue represents the low-risk group.

Afterward, we assessed the impact of the model scores developed by the eight signatures on the overall survival of the training set. Initially, the median of RS was taken as the critical value, the samples were sorted into high- and low-risk groups (Table S6), and the KM curve was drawn. The outcomes are illustrated in Figure 6(a): samples in the high-risk group had a worse prognosis, and the KM curve of the high-risk group was *p* = 0.0028, showing that there were major variations in the prognosis of the two groups. According to the developed risk model, the ROC curve of prognostic signature was drawn, as illustrated in Figure 6(b): the AUC values of 1/3/5 years were 0.747/0.663/0.654, respectively, showing good prediction efficiency of the model score. The scatter plot of survival time and survival state and the scatter plot of sample RS were drawn simultaneously. Combined with these two scatter plots, the relationship between survival and score could be observed. The outcomes are illustrated in Figures 6(c) and 6(d). Finally, the expression heat map of model genes and the expression differences of model genes in the model groups of the training set are highlighted in Figure 6(e).

**Figure 7:**
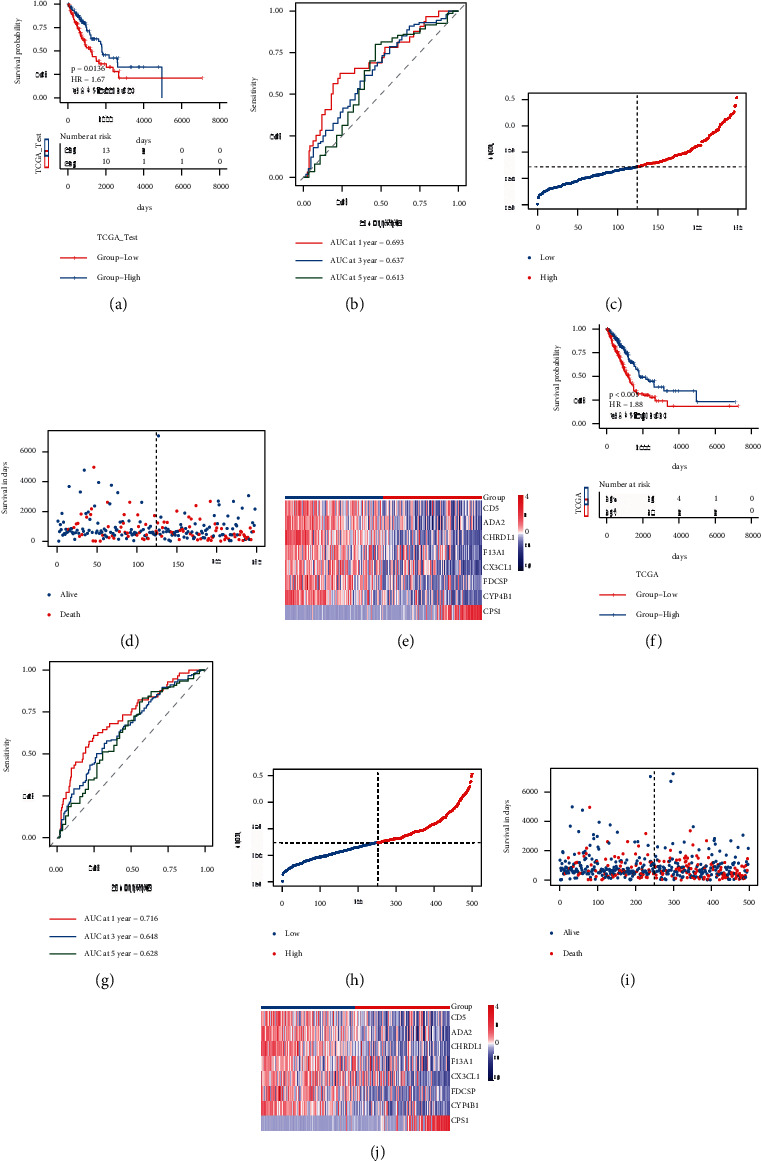
TCGA validation set and whole set validation model prognostic efficacy. (a)-(b): KM curve and ROC curve of TCGA verification set; (c)–(e): risk triple connection diagram of TCGA verification set; (f)-(g): KM curve and ROC curve of TCGA overall set; (h)–(j): risk triple connection diagram of TCGA overall set.

The capacity of RS to predict overall survival was then tested using the test set and overall set of TCGA-LUAD. In the test set and overall set, the samples were sorted into high-risk and low-risk groups (Table S7) using the same technique as the TCGA training set. The prognosis of the high-risk group was worse, as indicated in Figures 7(a) and 7(f), and there were substantial disparities in the prognosis of the high-risk and low-risk groups. As shown in Figures 7(b) and 7(g), the AUC of 1/3/5 years in the TCGA-LUAD test set was 0.693/0.637/0.613, whereas the AUC of 1/3/5 years in the whole dataset of TCGA-LUAD was 0.716/0.648/0.628. The scatter diagrams of RS, survival time, and survival state of the two datasets are shown in Figures 7(c), 7(d), and 7(h) and 7(i), respectively, to show the distribution of RS in the sample. The expression heat maps of model genes in the matching dataset, shown in Figures 7(e) and 7(j), revealed the expression distribution of genes in the dataset samples. The validation findings of the TCGA validation set and overall set revealed that the model score had strong and stable efficiency for survival prediction in both sets.

**Figure 8:**
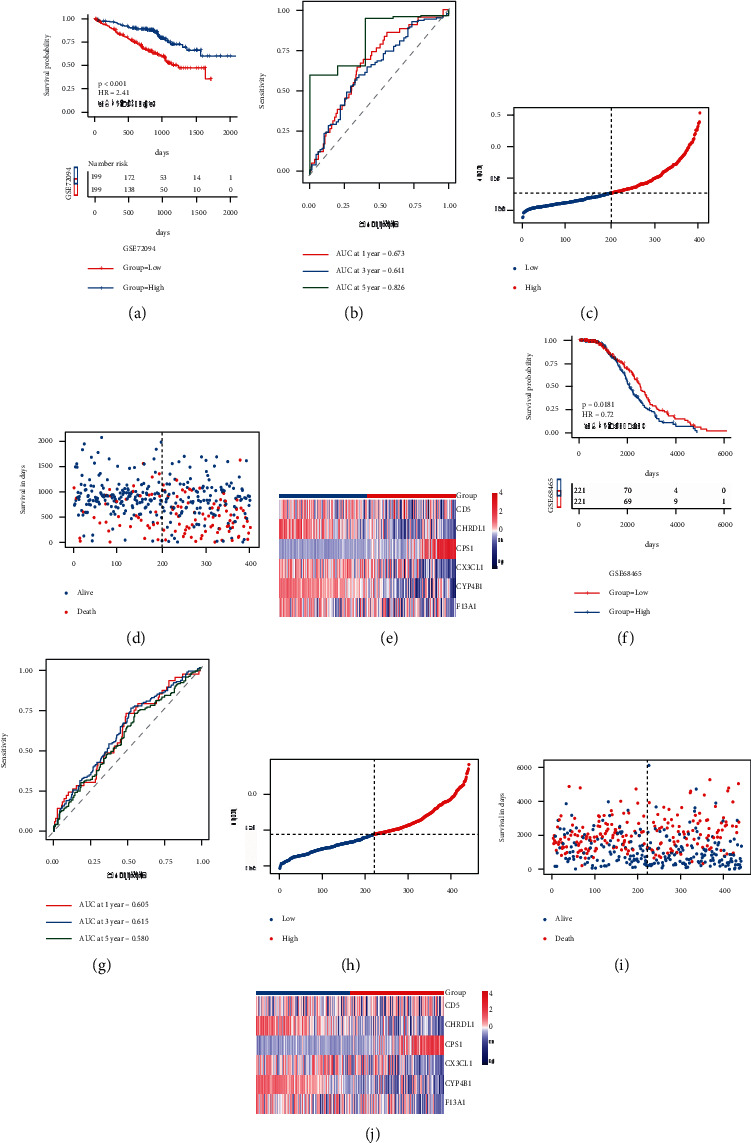
The GEO dataset was used for the verification of the model's prognostic efficacy. (a)-(b): KM curve and ROC curve of verification set GSE72094; (c)-(e): risk triple connection diagram of verification set GSE72094; (f)-(g): KM curve and ROC curve of verification set GSE68465; (h)-(j): risk triple connection diagram of verification set GSE68465.

For further verification of model score strength in predicting the overall survival of lung adenocarcinoma patients, we chose two GEO external datasets for the same analysis and verification (Table S7). The outcomes are demonstrated in Figure 8, in which Figures A-E were the verification outcomes of the verification set GSE72094: as highlighted in Figure A, there were major variations in the KM curve of high- and low-risk groups, and the high-risk group had a worse prognosis. Figure B was the ROC curve, 1/3/5-year AUC was 0.673/0.646/0.826, respectively; C and D were the scatter diagram of sample RS and the scatter diagram of survival time and survival state, respectively; and Figure E was the expression heat map of model genes in this dataset. F-J was the verification result of GSE68465: Figure F highlights that there were major variations in KM curves between high- and low-risk groups, with a worse prognosis in the high-risk group. In Figure G, the AUC of 1/3/5 years was 0.605/0.615/0.580, respectively; Figures C and D are the scatter diagram of RS and the scatter diagram of survival time and survival state respectively, and Figure E is the expression heat map of model genes in the GSE68465 dataset. In the two GEO validation sets, the prognostic efficacy of the model was good.

### 3.4. Correlation of Multiple Tumor Features of TRP-RS

#### 3.4.1. Correlation of LUAD Clinical Features in TRP-RS

**Figure 9:**
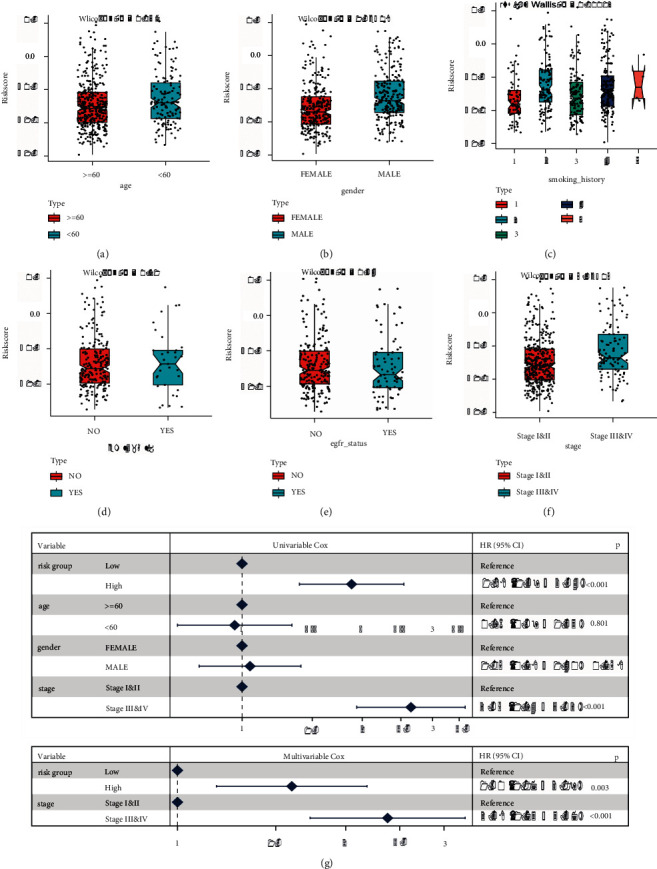
Clinical characteristics were related to TRPRS. (a)-(f): the distribution of RS in the clinical feature group. The corresponding clinical features were gender, age, smoking history, EML4 rearrangement, EGFR mutation status, and clinical grade, respectively. (g) Single- and multi-factor cox forest map.

The distribution disparities of RS among groups with distinct clinical characteristics were shown based on the clinical characteristics of the TCGA-LUAD dataset (Table S8).  Figure 9(a)–9(f) illustrates this. Age, gender, smoking history, and clinical classification were all significant variances in RS. Furthermore, univariate and multivariate Cox regression analysis might be done to see whether RS can work as an independent prognostic factor in the presence of other clinical variables. This study was integrated with the clinical parameters of LUAD samples, such as age, gender, and stage. After performing univariate Cox analysis, independent prognostic factors were chosen for multivariate Cox analysis.  Figure 9(g) shows the results: there were substantial differences in prognostic model grouping and clinical-grade in univariate and multivariate Cox regression compared to the reference, proving that they were independent prognostic factors.

### 3.5. The Difference in the Proportion of Immune Infiltrating Cells

**Figure 10:**
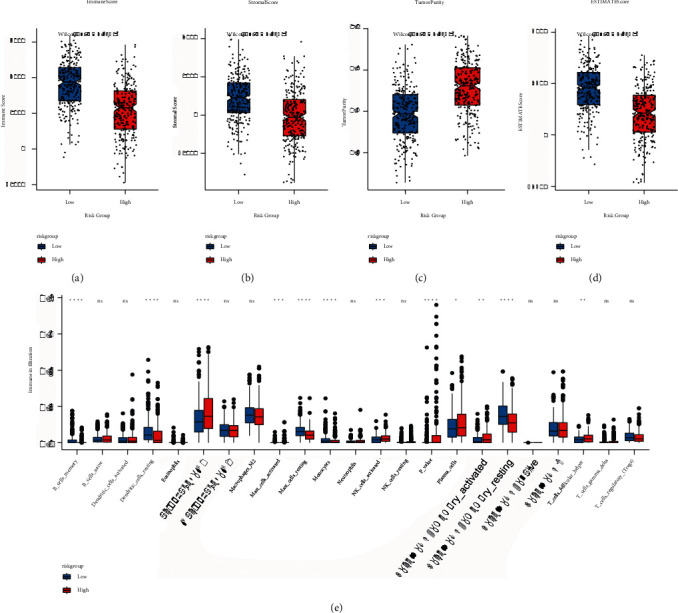
The model groups had a different proportion of immune infiltrating cells. (a)-(d): box diagram of the immune score, matrix score, tumor purity, and ESTIMATE score of high- and low-risk groups, respectively. Red represents the high-risk group and blue was for the low-risk group; (e) box plot of the proportion of immune infiltrating cells in high-risk and low-risk groups. Red indicates the high-risk group, and blue indicates the low-risk group.

Immune cells and stromal cells were two key categories of nontumor components in the tumor microenvironment, and they had been postulated to be useful for tumor diagnosis and evaluating the prognosis. Immune score, matrix score, tumor purity, and ESTIMATE score were all calculated in this study (Table S5).  Figure 10(a)–10(d) shows the results: the high-risk group's immune score, matrix score, and ESTIMATE score were considerably decreased in comparison with that of the low-risk group, while the tumor purity was higher; at the same time, the difference in the proportion of immune cells in the high-risk and low-risk groups was measured. The outcomes are demonstrated in Figure 10(e): there were major variations in the proportion of infiltrating cells of 12 kinds of immune cells in the high- and low-risk groups.

### 3.6. Differential Expression of Immune Checkpoint

**Figure 11:**
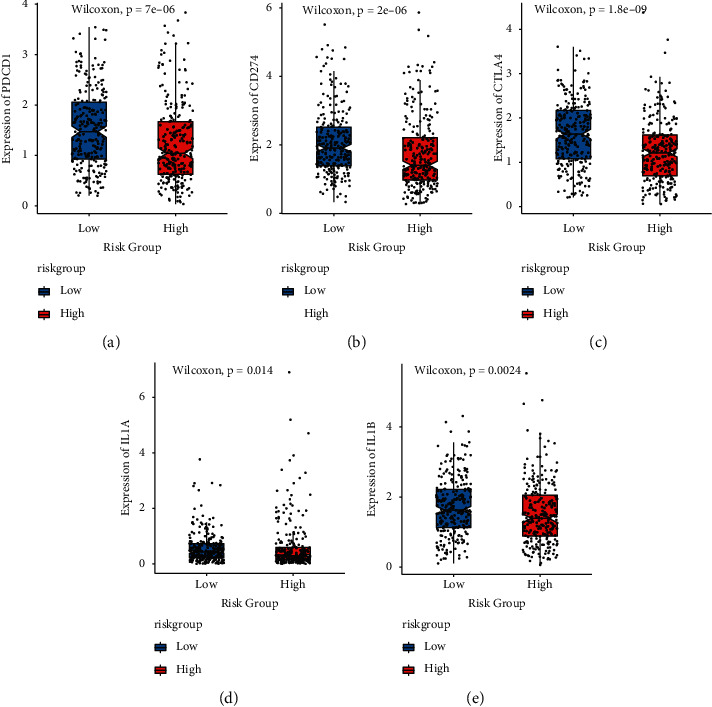
The expression of immune checkpoints varied between model groups. (a)-(h): box diagram indicating the expression difference of immune checkpoints PDCD1, CD274, CTLA4, IL1A, IL1B, IDO1, CXCL8, and IL18 in high- and low-risk groups, respectively, in which red represents the high-risk group and blue represents the low-risk group.

Immune checkpoints were a group of chemicals found in immune cells that controlled how active the immune system was. They were important in the development of human autoimmunity. In this study, 23 immunological checkpoints were studied, and 8 immune checkpoints that showed different expressions in high- and low-risk groups were visualized.  Figure 11 depicts the outcomes.

### 3.7. Mutational Differences in the Genome

**Figure 12:**
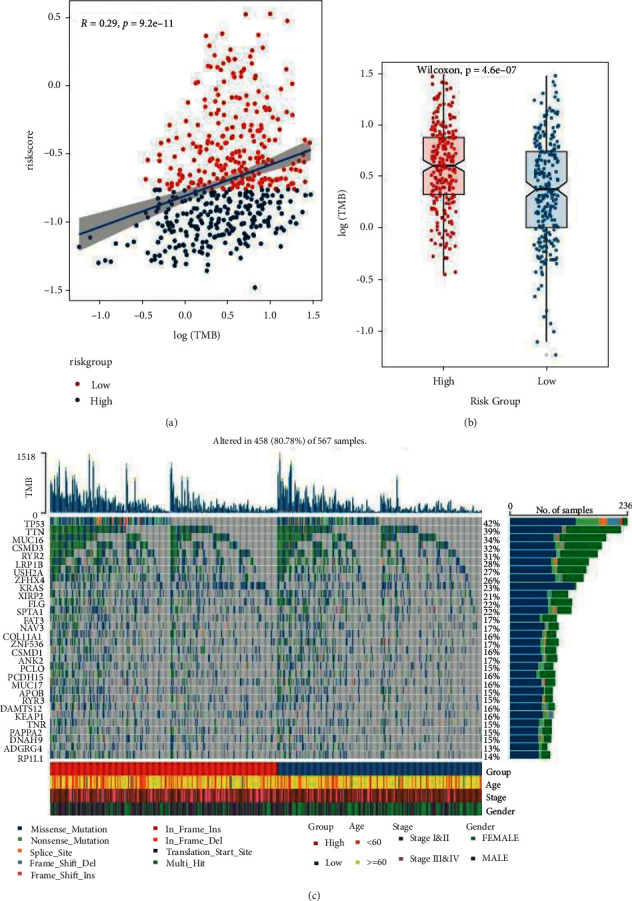
Genomic mutation variations between model groups. (a) RS and TMB correlation scatter plot, R was the correlation coefficient, P was the significant *P*-value of the statistical test; (b) box diagram of TMB value of high- and low-risk groups; (c) SNV waterfall of top 30 (mutation frequency) genes in the LUAD cohort.

A gene mutation might either promote and cause cancer, or it could coordinate and drive cancer's malignant progression. The research and development of tumor-targeted medications and innovative tumor therapies relied heavily on the knowledge of genome-level mutation. The first step was to conduct a correlation analysis between RS and TMB.  Figure 12(a) shows that the RS was positively linked with TMB. The variation in TMB between high- and low-risk groups proved useful in determining how patients responded to immunotherapy. As demonstrated in Figure 12(b), the TMB of the high-risk group was considerably higher in comparison with that of the low-risk group. To show the distribution of somatic variation in each sample between high- and low-risk groups, and the distribution of gene mutation among samples with various clinical features, the top 30 genes with the highest mutation frequency were selected to draw a waterfall diagram, as illustrated in Figure 12(c).

### 3.8. Enrichment Differences in the HALLMARKER Pathway

**Figure 13:**
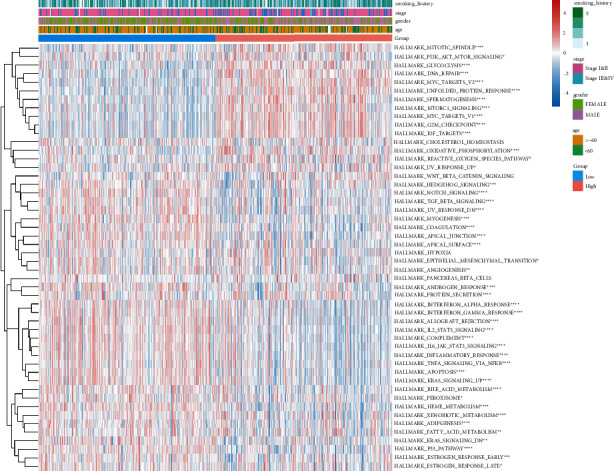
Variations in HALLMARKER pathway enrichment scores between model groups.

The pathway enrichment variations between high-risk and low-risk groups were assessed using the HALLMARKER pathway enrichment score of lung adenocarcinoma samples calculated in 3.2.2, combined with the model grouping information (Table S4), which was useful in studying the relationship between cancer characteristic pathways and prognosis.  Figure 13 displays the outcomes, which revealed that 45 pathway enrichment scores differed significantly between model groups.

### 3.9. Prediction of TRP-RS on the Therapeutic Efficacy of Patients

#### 3.9.1. Evaluation of Chemotherapeutic Drug Resistance

**Figure 14:**
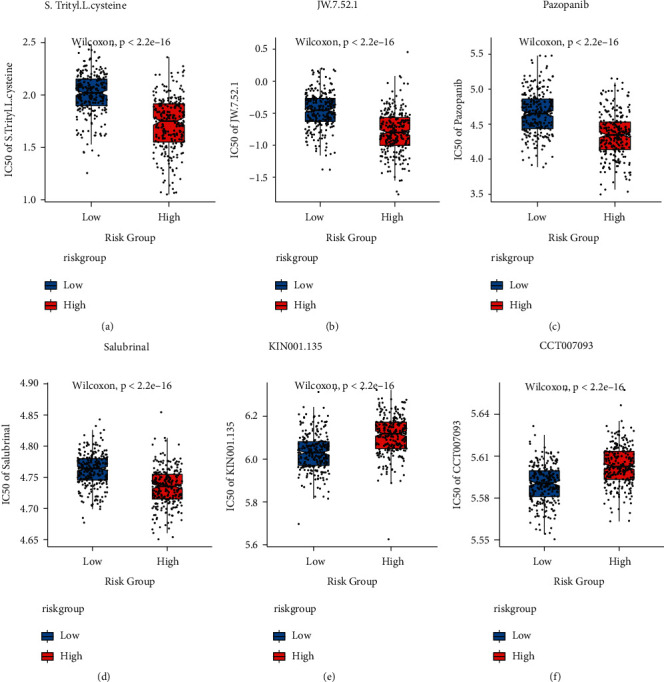
Variations in drug sensitivity between model groups. (a)-(f): IC50 box diagram of the first six drugs with the most significant difference in drug sensitivity in the high-risk and low-risk groups, respectively, in which red indicates the high-risk group and blue indicates the low-risk group.

According to the expression profile data from TCGA-LUAD (Table S9), the sensitivity IC50 value of 138 drugs in the Genomics of Drug Sensitivity in Cancer (GDSC) database was predicted, of which 97 drugs had significant differences between high-risk and low-risk groups, and the top 6 having the most major variation were selected for display, as shown in Figure 14.

#### 3.9.2. Prediction of Immunotherapy Efficacy

**Figure 15:**
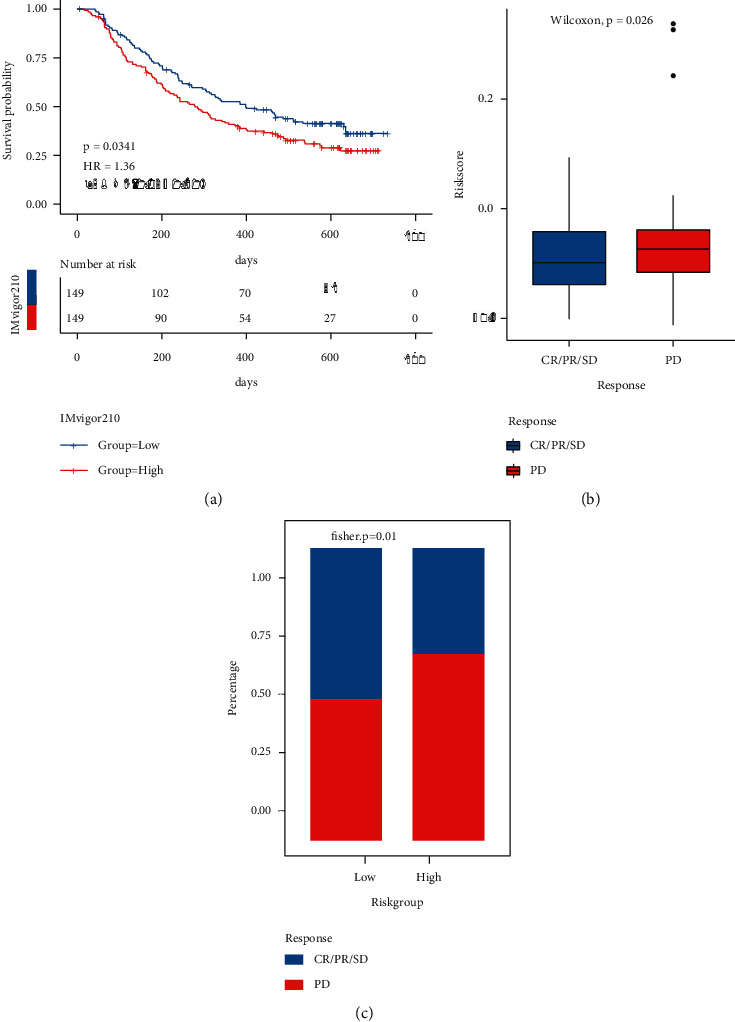
TRPRS predicted immunotherapy effect. (a) KM curve of immunotherapy cohort; (b) box diagram of RS distribution in different immunotherapy response groups; (c) bar chart of proportion distribution of immunotherapy response of samples in high-risk and low-risk groups. Blue indicates the reactive group and red indicates the nonreactive group.

To study whether the model gene could work as a marker of immunotherapy response, the RS calculation method was verified with the model in 3.4 using the IMvigor210 dataset. The dataset was sorted into high- and low-risk groups, and the KM curve was drawn for comparing their survival differences (Table S10). The outcomes are illustrated in Figure 15(a): there were major variations in the prognosis of high- and low-risk groups in the immunotherapy cohort. After receiving immunotherapy, they were grouped based on the response data, and then, we compared the model score variations between various immunotherapy response groups. The outcomes are highlighted in Figure 15(b): the RS of the immunotherapy nonresponse group (PD) was considerably higher in comparison with that of the response group. Meanwhile, the objective response rate of immunotherapy in the low-RS group was considerably higher than that in the high-risk group. The outcomes are demonstrated in Figure 15(c).

## 4. Discussion

TRP channels are cation channels found on the surface of cell membranes that can penetrate Ca^2+^, Mg^2+^, Na^+^, K^+^, and other cations. The TRP superfamily is categorized into seven subfamilies on the basis of the amino acid sequence homology, which is as follows: TRPM, TRPML, TRPA, TRPC, TRPN, TRPP, and TRPV [13, 14]. In tumors, the signal pathway imbalance can regulate the expression level of some TRP channels, thus altering the sensitivity and adaptability of cells to the external environment [15, 16]. Currently, a variety of drug trials that block or interfere with the TRP channel have been performed domestically and abroad, indicating the prospect of the TRP channel in tumor molecular targeted therapy. Recently, some studies have proposed the prognostic value of TRP protein in Pan-cancer [17]. However, reports on the role of TRP protein in lung adenocarcinoma are insufficient. Existing literature shows that TRPA1 is increasingly expressed in non-small cell lung cancer patients [18]. In Lewis lung cancer cells, the expression of TRPA1 and TRPM8 is linked with autophagy, tumor cell metastasis, and energy metabolism [19]. Thus, it is very important to study the mechanism and potential prognostic value of TRP protein in lung adenocarcinoma for its molecular targeted therapy.

In this research, TRP-related genes linked with prognosis were found in lung adenocarcinoma samples, yielding a total of 15 genes including TRPA1. The outcomes of survival analysis revealed that patients with high expression of TRPA1 had a poor prognosis. TRPA1 is an ankyrin first found by Story in 2003 [20]. A large amount of evidence supports that TRPA1 has a significant role in pain generation and the pathological process of high pain sensitivity [21], and can act as a novel target for the treatment of pain. According to recent research, TRPA1 is also involved in tumorigenesis, development, and chemoresistance. For instance, in oral squamous cell carcinoma, the TRPA1 channel is overexpressed [22], and the activation of TRPA1 can promote the metastasis and proliferation of prostate cancer cells [23]. Additionally, TRPA1 can be activated by O_2_, H_2_O_2_, and platinum drugs such as carboplatin and oxaliplatin, which are widely known clinically used cytotoxic drugs, and promote the cellular oxidative stress defense process resulting from tumor cells escaping ROS [24]. TRPA1 and FGFR2 binding events are carcinogenic drivers in lung adenocarcinoma [25]. TRPA1 may have predictive relevance in lung cancer, according to our findings. Following that, lung adenocarcinoma samples were classified into two subgroups according to the expression of 15 TRP-related genes. Cluster 1 had a much better prognosis than Cluster 2. When the amount of immune cell infiltration between the two subtypes was calculated, it was discovered that the proportion of CD8+ T cells in the C1 subtype was much larger in comparison with that in the C2 subtype. Because CD8^+^ T cells were the primary effector cells of antitumor immunity [26–28], it was plausible to hypothesize that the C1 subtype could play an antitumor role by controlling immune cells in the tumor microenvironment.

Afterward, 8 gene signatures—CX3CL1, FDCSP, F13A1, CPS1, CD5, CHRDL1, CYP4B1, and ADA2—were developed according to the differentially expressed genes among subtypes, and the RS model was constructed. Except for ADA2 and FDCSP in the above eight genes had not been examined in lung cancer, others had been reported in the literature such as carbamoyl phosphate synthase 1 (CPS1) was the rate-limiting enzyme of the urea cycle. The study revealed that CPS1 knockdown can induce ammonia accumulation and inhibit the nucleic acid synthesis pathway resulting in the inhibition of tumor cells' growth [29]. CPS1 was upregulated in many types of tumors, including lung adenocarcinoma [30], glioblastoma multiforme [31], and gastric cancer [32], and indicated a poor prognosis. CX3C motif chemokine ligand 1 (CX3CL1) was considerably overexpressed in lung cancer [33] and has the ability to promote the invasion and migration of lung cancer cells through the Src/focal adhesion kinase signaling pathway [34]. Bidirectional crosstalk between macrophages and tumor cells through CCR2 and CX3CR1 signal transduction might be the driving mechanism of lung cancer [35]. Transglutaminase F13A1 was a primary indicator of venous thrombosis features such as thrombus size, stability, and erythrocyte retention [36], which might be involved in VTE formation and lung cancer progression [37]. CHRDL1 inhibited bone morphogenetic proteins (BMPs). Its low expression predicted a poor prognosis of lung cancer [38], and it might improve immunotherapy efficacy by controlling immune cell infiltration [39]. Cytochrome P4504B1 (CYP4B1) expression was dramatically reduced in lung cancer [40, 41]. Although the mechanism of a single gene in lung adenocarcinoma had been steadily established, the prognosis of patients with the same stage and differentiation was extremely variable due to tumor cell heterogeneity; thus, the prognosis model of gene TRP-panel had more clinical application value.

The RS model's effectiveness was confirmed further through the validation of the TCGA training set, validation set, overall set, and GEO external validation set. The results demonstrated that the model's effectiveness was consistent. Cox analysis, both univariate and multivariate, indicated that RS was an independent predictive factor in several clinical indications of lung cancer. To investigate the clinical application value of RS, patients were categorized into high- and low-risk groups according to their RS median. We assessed the number of immune infiltrating cells, immunological checkpoint expression differences, and the genetic mutation variations between the two groups. The findings revealed that there were considerable variances.

The occurrence and progression of lung cancer was a multistage and multistep process driven mostly by aberrant gene expression in the cell pathway. EGFR mutation frequency was higher in Chinese patients with lung cancer compared to TCGA data, while KRAS, BRAF, TP53, and KEAP1 mutation frequency was decreased [42]. Our findings revealed that the TP53 mutation frequency was higher in lung cancer samples, which could be attributed to ethnic variations. The efficacy of the model was validated by an immunotherapy dataset to investigate the utility of RS in treatment. The findings revealed that there were substantial disparities in survival and immunotherapy response between model groups. However, this study still has some limitations. First, we only applied the LUAD samples in the TCGA database, which lacked the validation of real clinical data. Second, the roles of the relevant signaling pathways identified in the conclusions are unclear, so more work needs to be done to strengthen and validate the stability of the risk signature.

## 5. Conclusions

In this research, we used the TRP gene associated with prognosis to categorize patients with lung adenocarcinoma. According to the differentially expressed genes among subtypes, the 8-gene signature and RS model were developed. The RS model could predict patients' prognosis with lung adenocarcinoma and help in its clinical diagnosis and treatment.

## Data Availability

The data used to support the findings of this study are available from the corresponding author upon reasonable request.

## Supplementary Materials

Supplementary Materials Table S1. TRP gene expression and cox results. Table S2. Consensus clustering. Table S3. TRP subtype differential expression analysis. Table S4. Enrichment difference of HALLMARKER pathway. Table S5. Immune infiltrating cell ratio analysis. Table S6. TCGA training set risk model construction. Table S7. Validation results of TCGA and GEO datasets. Table S8. Univariate and multivariate cox validation of risk score as independent prognostic. Table S9. Drug sensitivity prediction. Table S10. Validation of IMvigor210 cohort for immunotherapy.
