# Supplementary material for: Systematic Analysis and Identification of Molecular Subtypes of TRP-Related Genes and Prognosis Prediction in Lung Adenocarcinoma
Source: J Oncol. 2022 Aug 31;2022:5388283. doi: 10.1155/2022/5388283 (PMC9452946; doi:10.1155/2022/5388283)
Supplement: Supplementary Materials — Table S1. TRP gene expression and cox results. Table S2. Consensus clustering. Table S3. TRP subtype differential expression analysis. Table S4. Enrichment difference of HALLMARKER pathway. Table S5. Immune infiltrating cell ratio analysis. Table S6. TCGA training set risk model construction. Table S7. Validation results of TCGA and GEO datasets. Table S8. Univariate and multivariate cox validation of risk score as independent prognostic. Table S9. Drug sensitivity prediction. Table S10. Validation of IMvigor210 cohort for immunotherapy. [file 5388283.f1.pdf]

Table S1.TRP gene expression and cox results

gene	coef	p.value	Hazard_Ratio	lower_.95	upper_.95	logrank_pvalue	wald_pvalue	Likelihood_pvalue	HR
ADCY9	-0.315314435	0.002346927	0.729559439	0.595446009	0.893879492	0.002381706	0.002346927	0.001989208	0.73(0.60-0.89)
F2RL1	0.210052621	0.002120733	1.233742979	1.079039449	1.410626591	0.002128047	0.002120733	0.002099449	1.23(1.08-1.41)
ITPR2	-0.210541082	0.04531635	0.810145772	0.659224132	0.995619154	0.045248597	0.04531635	0.044566613	0.81(0.66-1.00)
MCOLN1	-0.326296386	0.039083153	0.721591286	0.529273824	0.983789413	0.039420916	0.039083153	0.039177426	0.72(0.53-0.98)
MCOLN2	-0.308976317	0.010010384	0.734198158	0.580357484	0.928818789	0.009858143	0.010010384	0.009028116	0.73(0.58-0.93)
MLKL	0.259710212	0.043535221	1.296554307	1.007564799	1.66843172	0.043707569	0.043535221	0.04523551	1.30(1.01-1.67)
PLCG1	-0.251413169	0.028926863	0.777700983	0.620650931	0.974491118	0.029294193	0.028926863	0.028958628	0.78(0.62-0.97)
PLCG2	-0.319934214	0.015856593	0.726196809	0.55996129	0.94178261	0.015718661	0.015856593	0.014437245	0.73(0.56-0.94)
PRKCA	0.182504114	0.038566241	1.200219089	1.009646225	1.426762984	0.038491835	0.038566241	0.0413649	1.20(1.01-1.43)
PRKCB	-0.329747426	0.006053652	0.719105338	0.568244732	0.910017212	0.005968719	0.006053652	0.005114199	0.72(0.57-0.91)
PRKCD	-0.529282856	1.17172E-05	0.589027235	0.464879975	0.746328305	1.22385E-05	1.17172E-05	2.29654E-05	0.59(0.46-0.75)
PRKCE	-0.577340491	0.002094928	0.561389403	0.388618119	0.810971097	0.002065763	0.002094928	0.001777048	0.56(0.39-0.81)
PRKCH	-0.35558144	0.021628943	0.700765872	0.517362028	0.949186024	0.02156301	0.021628943	0.021468116	0.70(0.52-0.95)
RIPK3	-0.310800838	0.009864945	0.732859819	0.578764	0.927983625	0.009837927	0.009864945	0.010362436	0.73(0.58-0.93)
TRPA1	1.141587358	9.54897E-09	3.131735609	2.120571884	4.625057983	3.73174E-09	9.54897E-09	2.93322E-06	3.13(2.12-4.63)

Table.S2. Consensus Clustering.

sample	group
TCGA-05-4249-01A	C1
TCGA-05-4250-01A	C2
TCGA-05-4382-01A	C1
TCGA-05-4384-01A	C1
TCGA-05-4389-01A	C2
TCGA-05-4390-01A	C2
TCGA-05-4396-01A	C2
TCGA-05-4397-01A	C2
TCGA-05-4398-01A	C1
TCGA-05-4402-01A	C2
TCGA-05-4403-01A	C2
TCGA-05-4405-01A	C1
TCGA-05-4415-01A	C2
TCGA-05-4417-01A	C1
TCGA-05-4418-01A	C2
TCGA-05-4420-01A	C2
TCGA-05-4422-01A	C2
TCGA-05-4424-01A	C1
TCGA-05-4425-01A	C1
TCGA-05-4426-01A	C2
TCGA-05-4427-01A	C1
TCGA-05-4430-01A	C2
TCGA-05-4432-01A	C1
TCGA-05-4433-01A	C1
TCGA-05-4434-01A	C1
TCGA-05-5420-01A	C2
TCGA-05-5423-01A	C2
TCGA-05-5425-01A	C2
TCGA-05-5428-01A	C2
TCGA-05-5429-01A	C2
TCGA-05-5715-01A	C2

TCGA-35-3615-01A C2

TCGA-35-4122-01A C2

TCGA-35-4123-01A C2

TCGA-35-5375-01A C2

TCGA-38-4625-01A C2

TCGA-38-4626-01A C1

TCGA-38-4627-01A C2

TCGA-38-4628-01A C2

TCGA-38-4629-01A C2

TCGA-38-4630-01A C2

TCGA-38-4631-01A C2

TCGA-38-4632-01A C2

TCGA-38-6178-01A C2

TCGA-38-7271-01A C1

TCGA-38-A44F-01AC1

TCGA-44-2655-01A C2

TCGA-44-2656-01A C1

TCGA-44-2657-01A C1

TCGA-44-2659-01A C1

TCGA-44-2661-01A C1

TCGA-44-2662-01A C1

TCGA-44-2665-01A C2

TCGA-44-2666-01A C1

TCGA-44-2668-01A C1

TCGA-44-3396-01A C1

TCGA-44-3398-01A C2

TCGA-44-3917-01A C2

TCGA-44-3918-01A C1

TCGA-44-3919-01A C1

TCGA-44-4112-01A C2

TCGA-44-5643-01A C2

TCGA-44-5644-01A C2

TCGA-44-5645-01A C1

TCGA-44-6145-01A C1

TCGA-44-6146-01A C1

TCGA-44-6147-01A C1

TCGA-44-6148-01A C1

TCGA-44-6774-01A C1

TCGA-44-6775-01A C1

TCGA-44-6776-01A C2

TCGA-44-6777-01A C1

TCGA-44-6778-01A C1

TCGA-44-6779-01A C2

TCGA-44-7659-01A C1

TCGA-44-7660-01A C2

TCGA-44-7661-01A C1

TCGA-44-7662-01A C2

TCGA-44-7667-01A C2

TCGA-44-7669-01A C1

TCGA-44-7670-01A C2

TCGA-44-7671-01A C2

TCGA-44-7672-01A C1

TCGA-44-8117-01A C2

TCGA-44-8119-01A C2

TCGA-44-8120-01A C1

TCGA-44-A479-01A C1

TCGA-44-A47A-01A C1

TCGA-44-A47B-01A C2

TCGA-44-A47G-01A C1

TCGA-44-A4SS-01A C2

TCGA-44-A4SU-01A C1

TCGA-49-4486-01A C2

TCGA-49-4487-01A C1

TCGA-49-4488-01A C2

TCGA-49-4490-01A C2

TCGA-49-4494-01A C2

TCGA-49-4501-01A C2

TCGA-49-4505-01A C2

TCGA-49-4506-01A C2

TCGA-49-4507-01A C2

TCGA-49-4510-01A C2

TCGA-49-4512-01A C2

TCGA-49-4514-01A C2

TCGA-49-6742-01A C2

TCGA-49-6743-01A C2

TCGA-49-6744-01A C1

TCGA-49-6745-01A C1

TCGA-49-6761-01A C2

TCGA-49-6767-01A C2

TCGA-49-AAQV-01A C2

TCGA-49-AAR0-01A C2

TCGA-49-AAR2-01A C2

TCGA-49-AAR3-01A C2

TCGA-49-AAR4-01A C1

TCGA-49-AAR9-01A C2

TCGA-49-AARE-01A C2

TCGA-49-AARN-01A C1

TCGA-49-AARO-01A C1

TCGA-49-AARQ-01A C2

TCGA-49-AARR-01A C1

TCGA-4B-A93V-01A C2

TCGA-50-5044-01A C2

TCGA-50-5045-01A C1

TCGA-50-5049-01A C1

TCGA-50-5051-01A C2

TCGA-50-5055-01A C1

TCGA-50-5066-01A C2

TCGA-50-5068-01A C2

TCGA-50-5072-01A C2

TCGA-50-5930-01A C2

TCGA-50-5931-01A C2

TCGA-50-5932-01A C2

TCGA-50-5933-01A C1

TCGA-50-5935-01A C1

TCGA-50-5936-01A C2

TCGA-50-5939-01A C2

TCGA-50-5941-01A C1

TCGA-50-5942-01A C1

TCGA-50-5944-01A C1

TCGA-50-5946-01A C2

TCGA-50-6590-01A C1

TCGA-50-6591-01A C2

TCGA-50-6592-01A C2

TCGA-50-6593-01A C1

TCGA-50-6594-01A C2

TCGA-50-6595-01A C2

TCGA-50-6597-01A C2

TCGA-50-6673-01A C1

TCGA-50-7109-01A C2

TCGA-50-8457-01A C1

TCGA-50-8459-01A C1

TCGA-50-8460-01A C1

TCGA-53-7624-01A C2

TCGA-53-7626-01A C1

TCGA-53-7813-01A C2

TCGA-53-A4EZ-01A C2

TCGA-55-1592-01A C1

TCGA-55-1594-01A C1

TCGA-55-1596-01A C2

TCGA-55-5899-01A C1

TCGA-55-6543-01A C2

TCGA-55-6642-01A C1

TCGA-55-6712-01A C1
TCGA-55-6968-01A C2
TCGA-55-6970-01A C1
TCGA-55-6971-01A C1
TCGA-55-6972-01A C2
TCGA-55-6975-01A C2
TCGA-55-6978-01A C1
TCGA-55-6979-01A C1
TCGA-55-6980-01A C1
TCGA-55-6981-01A C2
TCGA-55-6982-01A C2
TCGA-55-6983-01A C1
TCGA-55-6984-01A C2
TCGA-55-6985-01A C1
TCGA-55-6986-01A C2
TCGA-55-6987-01A C1
TCGA-55-7227-01A C1
TCGA-55-7281-01A C2
TCGA-55-7283-01A C1
TCGA-55-7570-01A C2
TCGA-55-7573-01A C1
TCGA-55-7574-01A C1
TCGA-55-7576-01A C1
TCGA-55-7724-01A C1
TCGA-55-7725-01A C1
TCGA-55-7726-01A C2
TCGA-55-7727-01A C1
TCGA-55-7728-01A C1
TCGA-55-7815-01A C1
TCGA-55-7816-01A C1
TCGA-55-7903-01A C1
TCGA-55-7907-01A C1
TCGA-55-7910-01A C2

TCGA-55-7911-01A C1
TCGA-55-7914-01A C1
TCGA-55-7994-01A C1
TCGA-55-7995-01A C1
TCGA-55-8085-01A C2
TCGA-55-8087-01A C1
TCGA-55-8089-01A C1
TCGA-55-8090-01A C2
TCGA-55-8091-01A C1
TCGA-55-8092-01A C1
TCGA-55-8094-01A C2
TCGA-55-8096-01A C1
TCGA-55-8097-01A C1
TCGA-55-8203-01A C1
TCGA-55-8204-01A C2
TCGA-55-8205-01A C1
TCGA-55-8206-01A C1
TCGA-55-8207-01A C1
TCGA-55-8208-01A C1
TCGA-55-8299-01A C1
TCGA-55-8301-01A C1
TCGA-55-8302-01A C2
TCGA-55-8505-01A C2
TCGA-55-8506-01A C2
TCGA-55-8507-01A C2
TCGA-55-8508-01A C2
TCGA-55-8510-01A C1
TCGA-55-8511-01A C1
TCGA-55-8512-01A C2
TCGA-55-8513-01A C1
TCGA-55-8514-01A C2
TCGA-55-8614-01A C2
TCGA-55-8615-01A C2

TCGA-55-8616-01A C1

TCGA-55-8619-01A C1

TCGA-55-8620-01A C2

TCGA-55-8621-01A C1

TCGA-55-A48X-01A C1

TCGA-55-A48Y-01A C2

TCGA-55-A48Z-01A C2

TCGA-55-A490-01A C1

TCGA-55-A491-01A C2

TCGA-55-A492-01A C2

TCGA-55-A493-01A C1

TCGA-55-A494-01A C2

TCGA-55-A4DF-01A C1

TCGA-55-A4DG-01A C1

TCGA-55-A57B-01A C1

TCGA-62-8394-01A C2

TCGA-62-8395-01A C1

TCGA-62-8397-01A C2

TCGA-62-8398-01A C1

TCGA-62-8399-01A C1

TCGA-62-8402-01A C1

TCGA-62-A46O-01A C2

TCGA-62-A46P-01A C2

TCGA-62-A46R-01A C1

TCGA-62-A46S-01A C2

TCGA-62-A46V-01A C2

TCGA-62-A46Y-01A C1

TCGA-62-A470-01A C2

TCGA-62-A471-01A C2

TCGA-62-A472-01A C1

TCGA-64-1676-01A C2

TCGA-64-1677-01A C2

TCGA-64-1678-01A C2

TCGA-64-1679-01A C2

TCGA-64-1680-01A C2

TCGA-64-1681-01A C1

TCGA-64-5774-01A C2

TCGA-64-5775-01A C2

TCGA-64-5778-01A C2

TCGA-64-5779-01A C1

TCGA-64-5781-01A C2

TCGA-64-5815-01A C2

TCGA-67-3770-01A C2

TCGA-67-3771-01A C1

TCGA-67-3772-01A C2

TCGA-67-3773-01A C2

TCGA-67-3774-01A C1

TCGA-67-6215-01A C2

TCGA-67-6216-01A C2

TCGA-67-6217-01A C1

TCGA-69-7760-01A C2

TCGA-69-7761-01A C1

TCGA-69-7763-01A C2

TCGA-69-7764-01A C1

TCGA-69-7765-01A C1

TCGA-69-7973-01A C2

TCGA-69-7974-01A C1

TCGA-69-7978-01A C1

TCGA-69-7979-01A C2

TCGA-69-7980-01A C2

TCGA-69-8253-01A C2

TCGA-69-8254-01A C1

TCGA-69-8255-01A C2

TCGA-69-8453-01A C1

TCGA-69-A59K-01A C1

TCGA-71-6725-01A C2

TCGA-71-8520-01A C2
TCGA-73-4658-01A C1
TCGA-73-4659-01A C2
TCGA-73-4662-01A C1
TCGA-73-4666-01A C1
TCGA-73-4668-01A C2
TCGA-73-4670-01A C2
TCGA-73-4675-01A C2
TCGA-73-4676-01A C2
TCGA-73-4677-01A C2
TCGA-73-7498-01A C1
TCGA-73-7499-01A C2
TCGA-73-A9RS-01A C2
TCGA-75-5125-01A C1
TCGA-75-5146-01A C2
TCGA-75-5147-01A C2
TCGA-75-6206-01A C2
TCGA-75-6212-01A C1
TCGA-75-6214-01A C2
TCGA-75-7025-01A C1
TCGA-75-7027-01A C2
TCGA-78-7143-01A C1
TCGA-78-7145-01A C2
TCGA-78-7146-01A C2
TCGA-78-7147-01A C2
TCGA-78-7148-01A C2
TCGA-78-7149-01A C2
TCGA-78-7150-01A C2
TCGA-78-7152-01A C2
TCGA-78-7153-01A C2
TCGA-78-7154-01A C2
TCGA-78-7155-01A C2
TCGA-78-7156-01A C2

TCGA-78-7158-01A C2

TCGA-78-7159-01A C2

TCGA-78-7160-01A C1

TCGA-78-7161-01A C2

TCGA-78-7162-01A C1

TCGA-78-7163-01A C2

TCGA-78-7166-01A C2

TCGA-78-7167-01A C2

TCGA-78-7220-01A C2

TCGA-78-7535-01A C2

TCGA-78-7536-01A C2

TCGA-78-7537-01A C2

TCGA-78-7539-01A C1

TCGA-78-7540-01A C2

TCGA-78-7542-01A C2

TCGA-78-7633-01A C2

TCGA-78-8640-01A C1

TCGA-78-8648-01A C1

TCGA-78-8655-01A C1

TCGA-78-8660-01A C1

TCGA-78-8662-01A C2

TCGA-80-5608-01A C2

TCGA-80-5611-01A C2

TCGA-83-5908-01A C1

TCGA-86-6562-01A C1

TCGA-86-6851-01A C1

TCGA-86-7701-01A C1

TCGA-86-7711-01A C1

TCGA-86-7713-01A C2

TCGA-86-7714-01A C1

TCGA-86-7953-01A C1

TCGA-86-7954-01A C1

TCGA-86-7955-01A C2

TCGA-86-8054-01A C2

TCGA-86-8055-01A C2

TCGA-86-8056-01A C1

TCGA-86-8073-01A C2

TCGA-86-8074-01A C2

TCGA-86-8075-01A C2

TCGA-86-8076-01A C1

TCGA-86-8278-01A C2

TCGA-86-8279-01A C2

TCGA-86-8280-01A C1

TCGA-86-8358-01A C2

TCGA-86-8359-01A C2

TCGA-86-8585-01A C2

TCGA-86-8668-01A C1

TCGA-86-8669-01A C1

TCGA-86-8671-01A C1

TCGA-86-8672-01A C2

TCGA-86-8673-01A C2

TCGA-86-8674-01A C2

TCGA-86-A456-01A C1

TCGA-86-A4D0-01A C2

TCGA-86-A4JF-01A C2

TCGA-86-A4P7-01A C1

TCGA-86-A4P8-01A C1

TCGA-91-6828-01A C1

TCGA-91-6829-01A C2

TCGA-91-6830-01A C1

TCGA-91-6831-01A C2

TCGA-91-6835-01A C1

TCGA-91-6836-01A C2

TCGA-91-6840-01A C2

TCGA-91-6847-01A C2

TCGA-91-6848-01A C1

TCGA-91-6849-01A C2
TCGA-91-7771-01A C1
TCGA-91-8496-01A C2
TCGA-91-8497-01A C1
TCGA-91-8499-01A C2
TCGA-91-A4BC-01A C1
TCGA-91-A4BD-01A C2
TCGA-93-7347-01A C1
TCGA-93-7348-01A C1
TCGA-93-8067-01A C2
TCGA-93-A4JN-01A C1
TCGA-93-A4JO-01A C1
TCGA-93-A4JP-01A C1
TCGA-93-A4JQ-01A C1
TCGA-95-7039-01A C2
TCGA-95-7043-01A C2
TCGA-95-7562-01A C2
TCGA-95-7567-01A C2
TCGA-95-7944-01A C2
TCGA-95-7947-01A C2
TCGA-95-7948-01A C2
TCGA-95-8039-01A C1
TCGA-95-8494-01A C1
TCGA-95-A4VK-01A C2
TCGA-95-A4VN-01A C1
TCGA-95-A4VP-01A C1
TCGA-97-7546-01A C1
TCGA-97-7547-01A C1
TCGA-97-7552-01A C1
TCGA-97-7553-01A C1
TCGA-97-7554-01A C1
TCGA-97-7937-01A C2
TCGA-97-7938-01A C1

TCGA-97-7941-01A	C1
TCGA-97-8171-01A	C2
TCGA-97-8172-01A	C1
TCGA-97-8174-01A	C1
TCGA-97-8175-01A	C2
TCGA-97-8176-01A	C2
TCGA-97-8177-01A	C2
TCGA-97-8179-01A	C1
TCGA-97-8547-01A	C2
TCGA-97-8552-01A	C1
TCGA-97-A4LX-01A	C1
TCGA-97-A4M0-01A	C1
TCGA-97-A4M1-01A	C1
TCGA-97-A4M2-01A	C1
TCGA-97-A4M3-01A	C2
TCGA-97-A4M5-01A	C1
TCGA-97-A4M6-01A	C1
TCGA-97-A4M7-01A	C1
TCGA-99-7458-01A	C1
TCGA-99-8025-01A	C1
TCGA-99-8028-01A	C1
TCGA-99-8032-01A	C2
TCGA-99-8033-01A	C2
TCGA-99-AA5R-01A	C1
TCGA-J2-8192-01A	C1
TCGA-J2-8194-01A	C2
TCGA-J2-A4AD-01A	C2
TCGA-J2-A4AE-01A	C1
TCGA-J2-A4AG-01A	C1
TCGA-L4-A4E5-01A	C2
TCGA-L4-A4E6-01A	C1
TCGA-L9-A443-01A	C1
TCGA-L9-A444-01A	C1

TCGA-L9-A50W-01A	C2
TCGA-L9-A5IP-01A	C2
TCGA-L9-A743-01A	C1
TCGA-L9-A7SV-01A	C1
TCGA-L9-A8F4-01A	C1
TCGA-MN-A4N1-01A	C2
TCGA-MN-A4N4-01A	C2
TCGA-MN-A4N5-01A	C2
TCGA-MP-A4SV-01A	C1
TCGA-MP-A4SW-01A	C1
TCGA-MP-A4SY-01A	C2
TCGA-MP-A4T4-01A	C1
TCGA-MP-A4T6-01A	C1
TCGA-MP-A4T7-01A	C2
TCGA-MP-A4T8-01A	C2
TCGA-MP-A4T9-01A	C1
TCGA-MP-A4TA-01A	C2
TCGA-MP-A4TC-01A	C2
TCGA-MP-A4TD-01A	C1
TCGA-MP-A4TE-01A	C2
TCGA-MP-A4TF-01A	C2
TCGA-MP-A4TH-01A	C1
TCGA-MP-A4TI-01A	C1
TCGA-MP-A4TJ-01A	C1
TCGA-MP-A4TK-01A	C1
TCGA-MP-A5C7-01A	C2
TCGA-NJ-A4YF-01A	C2
TCGA-NJ-A4YG-01A	C2
TCGA-NJ-A4YI-01A	C1
TCGA-NJ-A4YP-01A	C2
TCGA-NJ-A4YQ-01A	C1
TCGA-NJ-A55A-01A	C1
TCGA-NJ-A55O-01A	C2

TCGA-NJ-A55R-01A	C1
TCGA-NJ-A7XG-01A	C2
TCGA-O1-A52J-01A	C1
TCGA-S2-AA1A-01A	C1

Table S3. TRP subtype differential expression analysis

gene	log2FC	pvalue	adjusted_pvalue
DOCK2	-1.017198229	1.77785E-74	1.73678E-70
IKZF1	-1.000879604	3.45669E-74	2.25123E-70
ARHGAP30	-1.147778298	3.26147E-72	1.36004E-68
SPN	-1.129203151	2.68025E-70	8.72779E-67
ITGAL	-1.263480027	2.01034E-66	4.36423E-63
NCKAP1L	-1.095377454	9.30523E-64	1.81806E-60
CSF2RB	-1.12165856	3.52275E-61	4.30171E-58
PTPRC	-1.355162341	5.71221E-61	6.56501E-58
RASSF2	-1.03307305	7.19298E-59	5.62146E-56
PPP1R16B	-1.019006305	3.53602E-58	2.55877E-55
ABI3BP	-1.10700469	4.35302E-58	3.03748E-55
CD84	-1.009024928	4.78746E-57	3.01734E-54
IL10RA	-1.051625732	5.74766E-54	2.80744E-51
CIITA	-1.08338323	1.53407E-53	6.6606E-51
IRF8	-1.093972316	3.04991E-53	1.26786E-50
CD5	-1.071042317	1.40443E-52	5.48795E-50
SASH3	-1.130648488	4.02534E-51	1.37977E-48
CD37	-1.151573213	1.53317E-50	4.99253E-48
EVI2B	-1.148233774	2.63976E-50	8.45504E-48
CD4	-1.146721682	2.12773E-49	6.02487E-47
MPEG1	-1.146523009	4.55512E-48	1.20268E-45
IL7R	-1.294400174	7.2486E-48	1.88831E-45
CCR7	-1.098665814	3.90046E-46	9.29356E-44
SLCO2B1	-1.153969491	9.33594E-46	2.14595E-43
FCMR	-1.090656375	2.18232E-45	4.73757E-43

SLAMF6	-1.003505365	8.91952E-45	1.81531E-42
SELPLG	-1.050447556	1.33242E-44	2.6564E-42
CYBB	-1.404591696	1.37073E-44	2.70518E-42
GIMAP7	-1.016462634	4.37215E-44	8.21376E-42
HLA-DOA	-1.388837011	1.46899E-42	2.41185E-40
A2M	-1.279946826	3.08957E-42	4.98876E-40
FGL2	-1.194981702	8.0637E-42	1.25039E-39
PLEK	-1.148366552	4.24206E-41	6.32681E-39
LCP1	-1.17645646	6.24712E-41	9.10867E-39
C7	-1.789476727	5.38302E-39	6.74189E-37
CD53	-1.097910447	8.04775E-39	9.70599E-37
CSF1R	-1.104592915	1.95052E-38	2.28199E-36
CLEC10A	-1.016506802	5.2239E-38	5.83226E-36
MNDA	-1.088427111	3.31307E-37	3.49896E-35
ADA2	-1.024406067	2.05708E-36	2.04016E-34
CCL19	-1.823831437	2.60767E-36	2.56024E-34
SELL	-1.164514456	3.87368E-36	3.71E-34
CHRDL1	-1.315397303	4.11455E-36	3.92147E-34
CD3E	-1.084385097	6.90311E-36	6.51561E-34
ITGAM	-1.009073185	8.7416E-36	8.05629E-34
PTGDS	-1.495846651	1.07553E-35	9.81947E-34
MRC1	-1.454591997	1.32938E-35	1.19693E-33
MS4A1	-1.250867997	7.05796E-35	5.96963E-33
CD2	-1.101952248	1.47993E-33	1.10785E-31
AOC3	-1.070763768	1.18851E-32	8.47485E-31
HLA-DQA1	-1.353145701	1.26872E-32	8.98123E-31
FMO2	-1.024827879	1.29724E-32	9.14997E-31
GZMK	-1.040062208	2.23814E-32	1.54519E-30
ITGB2	-1.131794679	9.75498E-32	6.33199E-30
ALOX5	-1.007838556	1.62595E-31	1.02808E-29
ADH1B	-1.520343946	3.45985E-31	2.11245E-29
FPR3	-1.005005283	9.37257E-31	5.53236E-29
LTBP2	-1.05954317	1.17394E-30	6.90856E-29

IL2RG	-1.041600577	1.93213E-30	1.11029E-28
CD27	-1.062941085	3.85662E-30	2.14064E-28
MFAP4	-1.397915792	9.23754E-30	4.93379E-28
COLEC12	-1.0157115	1.14684E-29	6.03962E-28
HLA-DPA1	-1.231185771	1.4945E-29	7.80738E-28
SPOCK2	-1.014826504	4.32498E-29	2.13928E-27
HLA-DPB1	-1.169312227	7.65549E-29	3.73933E-27
LYZ	-1.546479563	1.12224E-27	5.07555E-26
VWF	-1.01789673	5.82106E-27	2.46707E-25
PLA2G2D	-1.168723514	1.59507E-26	6.53344E-25
CD163	-1.104580459	4.54328E-26	1.77533E-24
COL14A1	-1.024098375	1.56749E-25	5.86698E-24
F13A1	-1.180762331	2.03191E-25	7.47971E-24
CD74	-1.086538108	8.25239E-25	2.8792E-23
CD52	-1.04022744	2.63094E-24	8.71243E-23
INMT	-1.127061194	6.77576E-24	2.1561E-22
CPA3	-1.243289016	1.05238E-23	3.2741E-22
ACKR1	-1.078774805	1.96768E-23	5.94198E-22
LTB	-1.017607752	3.3894E-23	1.00212E-21
HLA-DRA	-1.091717703	4.90745E-23	1.42894E-21
CHIT1	-1.388895756	1.34532E-21	3.3916E-20
HLA-DRB1	-1.07826629	8.05828E-21	1.88329E-19
ROS1	-1.190674249	1.354E-20	3.0761E-19
HLA-DRB5	-1.263234696	7.29966E-20	1.51402E-18
ATP13A4	-1.114079223	1.12905E-19	2.29784E-18
LRRK2	-1.115885583	1.35206E-19	2.72617E-18
C1QB	-1.007075974	2.59074E-19	5.04665E-18
CD79A	-1.201111111	1.56533E-18	2.80765E-17
MARCO	-1.328984332	1.79908E-18	3.20131E-17
HLA-DQB1	-1.08086836	2.75619E-18	4.7995E-17
CX3CL1	-1.067365899	2.10766E-17	3.35064E-16
CXCL9	-1.318535218	1.7858E-16	2.53017E-15
FDCSP	-1.171290746	7.24335E-16	9.63381E-15

SFTPC	-2.469675218	1.96319E-15	2.47943E-14
ABCA3	-1.115064195	3.88097E-15	4.75103E-14
DPP4	-1.164470381	5.89562E-15	7.05445E-14
HLA-DQB2	-1.12475405	1.3544E-14	1.55843E-13
PIGR	-1.799682404	2.10455E-14	2.35772E-13
SLC34A2	-1.318168693	3.06173E-14	3.36445E-13
CXCL13	-1.175302828	3.24763E-14	3.55474E-13
HLA-DQA2	-1.238467311	3.59433E-14	3.91667E-13
AGER	-1.280221488	5.08209E-14	5.40817E-13
CCL18	-1.086818708	1.02843E-13	1.05831E-12
CLDN18	-1.383450961	2.6881E-13	2.61685E-12
JCHAIN	-1.113080697	4.2003E-12	3.52212E-11
S100P	1.736920934	8.98128E-12	7.25108E-11
SFTPA1	-1.957858295	1.13609E-11	9.03415E-11
SCGB1A1	-2.037026882	1.26001E-11	9.98704E-11
SFTPA2	-1.908520892	2.1827E-11	1.67041E-10
LTF	-1.194921858	4.82544E-11	3.4751E-10
SUSD2	-1.125969264	6.26004E-11	4.42666E-10
AQP1	-1.032261804	8.39249E-11	5.82082E-10
DMBT1	-1.178624799	9.76761E-10	5.88647E-09
CYP4B1	-1.069987387	1.24881E-08	6.41916E-08
AKR1C2	1.446035169	1.41645E-08	7.2295E-08
C4BPA	-1.094625396	4.72352E-08	2.22649E-07
SFTPD	-1.143893819	6.79478E-08	3.14365E-07
UCHL1	1.008897079	1.0873E-07	4.87275E-07
CPS1	1.163240593	1.14452E-07	5.1054E-07
SCGB3A1	-1.444265368	1.45643E-07	6.37822E-07
NAPSA	-1.060793481	1.02221E-06	3.93459E-06
CTSE	-1.037467138	3.48936E-06	1.23573E-05
SFTPB	-1.096968326	1.37228E-05	4.46637E-05
GPX2	1.123207015	2.96484E-05	9.17148E-05
FGA	1.076657803	4.95084E-05	0.000147927

Table S4. Enrichment difference of HALLMARKER pathway

sample HALLMARK_TNFA_SIGNALING_VIA_NFKB HALLMARK_HYPOXIA
HALLMARK_CHOLESTEROL_HOMEOSTASIS HALLMARK_MITOTIC_SPINDLE
HALLMARK_WNT_BETA_CATENIN_SIGNALING
HALLMARK_TGF_BETA_SIGNALING HALLMARK_IL6_JAK_STAT3_SIGNALING
HALLMARK_DNA_REPAIR HALLMARK_G2M_CHECKPOINT
HALLMARK_APOPTOSIS HALLMARK_NOTCH_SIGNALING
HALLMARK_ADIPOGENESIS HALLMARK_ESTROGEN_RESPONSE_EARLY
HALLMARK_ESTROGEN_RESPONSE_LATE
HALLMARK_ANDROGEN_RESPONSE HALLMARK_MYOGENESIS
HALLMARK_PROTEIN_SECRETION
HALLMARK_INTERFERON_ALPHA_RESPONSE
HALLMARK_INTERFERON_GAMMA_RESPONSE HALLMARK_APICAL_JUNCTION
HALLMARK_APICAL_SURFACE HALLMARK_HEDGEHOG_SIGNALING
HALLMARK_COMPLEMENT HALLMARK_UNFOLDED_PROTEIN_RESPONSE
HALLMARK_PI3K_AKT_MTOR_SIGNALING HALLMARK_MTORC1_SIGNALING
HALLMARK_E2F_TARGETS HALLMARK_MYC_TARGETS_V1
HALLMARK_MYC_TARGETS_V2
HALLMARK_EPITHELIAL_MESENCHYMAL_TRANSITION
HALLMARK_INFLAMMATORY_RESPONSE
HALLMARK_XENOBIOTIC_METABOLISM
HALLMARK_FATTY_ACID_METABOLISM
HALLMARK_OXIDATIVE_PHOSPHORYLATION HALLMARK_GLYCOLYSIS
HALLMARK_REACTIVE_OXYGEN_SPECIES_PATHWAY
HALLMARK_P53_PATHWAY HALLMARK_UV_RESPONSE_UP
HALLMARK_UV_RESPONSE_DN HALLMARK_ANGIOGENESIS
HALLMARK_HEME_METABOLISM HALLMARK_COAGULATION
HALLMARK_IL2_STAT5_SIGNALING HALLMARK_BILE_ACID_METABOLISM
HALLMARK_PEROXISOME HALLMARK_ALLOGRAFT_REJECTION
HALLMARK_SPERMATOGENESIS HALLMARK_KRAS_SIGNALING_UP
HALLMARK_KRAS_SIGNALING_DN HALLMARK_PANCREAS_BETA_CELLS

TCGA-05-4249-01A 0.422022728 0.365877464 0.447071671 0.333061922
0.24849845 0.512850191 0.3718381170.449989516 0.320081555 0.477228178

0.43543467 0.448692756 0.33765236 0.341454609 0.473681792 0.181589638
0.549653808 0.48974571 0.436775891 0.3359141 0.136455752 0.182692373
0.347663731 0.528514549 0.41952001 0.508034189 0.325796799
0.653453871 0.436162463 0.3831160050.263938041 0.292033712
0.398293357 0.590964142 0.407907163 0.541373236 0.440813018
0.409385648 0.344375421 0.383238098 0.329681797 0.304695176
0.343740351 0.142660752 0.378169906 0.323342046 -0.119662166
0.266016315 -0.144235164 -0.060973908
TCGA-05-4250-01A 0.539034398 0.447715407 0.459048001 0.401835554
0.222612039 0.5512110060.384200725 0.482999169 0.45741984 0.515420116
0.405648413 0.469142371 0.326682295 0.35741081 0.466359662
0.181716224 0.538500494 0.5211659940.473259237 0.350890879
0.237459819 0.175375625 0.379550153 0.559635899 0.411543346
0.578669603 0.4919511930.704365319 0.518035512 0.497641725
0.322442659 0.294055785 0.402781862 0.6112754490.45807045 0.541884416
0.459241745 0.448905453 0.337251432 0.478531731 0.308892575
0.335759888 0.375244092 0.122365421 0.370149857 0.364392871 -
0.062679431 0.325471706 -0.181661511 -0.049786102
TCGA-05-4382-01A 0.489335896 0.471005653 0.490726852 0.352998169
0.26369074 0.522980319 0.432691778 0.455047427 0.363850656
0.501946652 0.448520514 0.456333232 0.336924041 0.343858125
0.444521391 0.240505088 0.534224778 0.482752095 0.460933671
0.351763158 0.206570746 0.149187692 0.37999274 0.540805302
0.418002483 0.567598548 0.376470316 0.657219509 0.416259657
0.556534478 0.331918551 0.308225297 0.412897745 0.598504191
0.448600788 0.549983748 0.444460191 0.423206406 0.330573368
0.469744014 0.321206108 0.358214966 0.352651589 0.116977885
0.352572294 0.372161842 -0.080665577 0.30574501 -0.186309918 -
0.103909292
TCGA-05-4384-01A 0.435058432 0.389773276 0.448862786 0.32354839
0.3011580650.530021741 0.323794833 0.45360094 0.257072839 0.467410577
0.413342166 0.502587128 0.365127596 0.359564735 0.466558982
0.227033763 0.54965933 0.424796206 0.369313668 0.316886514

0.147699786 0.151994641 0.34394623 0.500997496 0.404476486
0.502924002 0.271934394 0.640606694 0.412463164 0.36280098
0.245063353 0.341739718 0.466126162 0.613778086 0.381859119
0.574493532 0.457640235 0.4114233240.35713753 0.351329328 0.349939503
0.336969702 0.326103579 0.181080019 0.384001573 0.250590901 -
0.131623125 0.25138436 -0.137049548 -0.039988646
TCGA-05-4389-01A 0.382227508 0.348934007 0.4602411050.329254531
0.3211342070.447507595 0.390654637 0.489595274 0.398418133
0.4611077030.369376002 0.493207212 0.342826227 0.359896166
0.445032554 0.176942349 0.529289505 0.497999575 0.462047822
0.291390142 0.130095538 0.09995863 0.366676039 0.539607323
0.432154393 0.567474907 0.44197115 0.689975402 0.489443399
0.344589322 0.262156325 0.360501396 0.461939576 0.640617845
0.412886706 0.594880789 0.445392092 0.417434982 0.250364534
0.329862066 0.3356118810.315538121 0.353652719 0.19586219 0.40853982
0.373553809 -0.049316642 0.256366208 -0.150016034 -0.026486818
TCGA-05-4390-01A 0.402017453 0.427108304 0.498455971 0.347944085
0.271682255 0.486071561 0.332004448 0.487071482 0.466013344
0.437712816 0.370122187 0.464674087 0.352808668 0.372323653
0.47215572 0.196354283 0.520631593 0.37577561 0.332894474 0.291029918
0.137547218 0.093908505 0.313486808 0.54321288 0.405079516
0.572102103 0.495677507 0.703273034 0.515635538 0.466147266
0.231325997 0.2949062110.419562542 0.629655506 0.452020513
0.530767789 0.4115896620.441219769 0.298986656 0.420766223
0.3080551180.338198084 0.299965353 0.138848768 0.38427342 0.244804584
-0.041482256 0.252667184 -0.173683201 0.050693155
TCGA-05-4396-01A 0.391341246 0.371829035 0.485451801 0.347500516
0.256323595 0.520874312 0.30647792 0.431886299 0.343876874 0.43690303
0.381316822 0.468806275 0.357189247 0.357836598 0.479668794
0.163668613 0.574914257 0.456235991 0.377421715 0.277914082
0.108169236 0.194350192 0.326674241 0.521663779 0.432493262
0.5114278820.338208187 0.645186823 0.391046685 0.33684584 0.181295262
0.332016381 0.466694149 0.624203999 0.404280672 0.498181098

0.409066298 0.423752563 0.345902957 0.394389409 0.345862059
0.332443988 0.302061923 0.197585903 0.408725347 0.217377771 -
0.099320743 0.227115483-0.157657662 0.021084995
TCGA-05-4397-01A 0.347767546 0.388824531 0.485100269 0.395466317
0.18349984 0.395529761 0.353793301 0.509683539 0.528488816
0.423047729 0.321910885 0.494151689 0.275622483 0.322571047
0.420353688 0.175754416 0.517106363 0.573971766 0.469700591
0.262184052 0.185862672 0.1113935680.327623948 0.539260165
0.407958369 0.582454497 0.58124052 0.7109121190.460048191 0.330095237
0.209871742 0.319475615 0.450319401 0.672343871 0.433084036
0.568485655 0.402449519 0.447029882 0.224781075 0.277869952
0.327053876 0.275756965 0.273603255 0.17806108 0.394659999
0.255166881 -0.011558182 0.155239088 -0.16364511 -0.124742589
TCGA-05-4398-01A 0.526969337 0.4502110530.47635141 0.370976401 0.221076688
0.5011829020.454562646 0.48294059 0.419475292 0.487747187 0.377615606
0.467363822 0.326084279 0.343941312 0.435050239 0.195218982
0.520201784 0.557819012 0.5111371810.324703147 0.22547232 0.159668546
0.370069224 0.56517108 0.431955283 0.570010428 0.455539549
0.6951150420.520227565 0.470388902 0.354039434 0.280396742
0.395282633 0.593098866 0.443173687 0.559617288 0.432244398
0.434655098 0.297442927 0.422270601 0.315965814 0.317691414
0.367326414 0.1132775090.362619995 0.383811834-0.07265914 0.294260818 -
0.160082078 -0.096775087
TCGA-05-4402-01A 0.409468878 0.418591391 0.501710034 0.335003481
0.255246456 0.527853223 0.3502411730.466682867 0.37519703 0.490365381
0.423712131 0.4859392 0.325777965 0.346533571 0.465022347
0.213928831 0.547566349 0.572404629 0.480643325 0.337389024
0.168305635 0.130926001 0.3611834840.523142721 0.429939808
0.576300802 0.389419648 0.668837001 0.429583887 0.472966369
0.27315484 0.304813425 0.438666976 0.605360262 0.466994848 0.53201741
0.432669755 0.4077411950.343124617 0.454299063 0.333044276 0.33021267
0.326006864 0.1691731190.405883445 0.307006901 -0.078768799
0.269588511-0.117000605 -0.081350697

TCGA-05-4403-01A 0.458349992 0.421032908 0.513014966 0.29698282 0.239659638 0.529552781 0.378881855 0.453472495 0.293210258 0.499718727 0.431972731 0.494892155 0.383533345 0.427281331 0.488995728 0.20034233 0.536182189 0.54833003 0.45845659 0.328972006 0.237743104 0.146096537 0.402529547 0.494449855 0.397531967 0.527662342 0.289064592 0.632840799 0.384267668 0.424850036 0.305909819 0.347678129 0.439734885 0.625959715 0.443346537 0.542678672 0.470273807 0.422918123 0.341040594 0.393731048 0.334948661 0.376992925 0.359224322 0.197391606 0.392268783 0.329931096 -0.141250358 0.331477395 -0.122821022 -0.002818706

TCGA-05-4405-01A 0.436131274 0.383106328 0.472322098 0.359787188 0.272209074 0.546290645 0.33167222 0.421285709 0.320061873 0.485265829 0.46364676 0.455090394 0.357497629 0.352566631 0.482166079 0.248397908 0.531293873 0.489152735 0.42948888 0.362082889 0.196408579 0.2112402360.3394311920.533428487 0.406717888 0.517935029 0.333169766 0.64988978 0.450522715 0.494801909 0.2697011330.276719132 0.382609584 0.577057397 0.409662444 0.513623107 0.435610322 0.407880938 0.366458761 0.413296069 0.3285111170.3420961150.322827294 0.167658184 0.37111698 0.299172824 -0.098165093 0.280492636 -0.148871825 -0.055657581

TCGA-05-4415-01A 0.454231449 0.39418858 0.507001907 0.436919404 0.257733585 0.492790399 0.327331701 0.506281546 0.528444233 0.453521032 0.316367223 0.469685417 0.289048953 0.326207951 0.449937826 0.1150780220.544148133 0.506553619 0.44496748 0.271217058 0.174153552 0.086155992 0.342878985 0.578903806 0.43332852 0.622096 0.55165338 0.747915132 0.60967479 0.386229332 0.242896633 0.288050333 0.404181647 0.637272548 0.432679384 0.581843028 0.375552791 0.422254886 0.355081481 0.404720457 0.302723913 0.26062238 0.326090414 0.143306557 0.380929739 0.278653265 0.005154377 0.209045755 -0.18523234 -0.066734706

TCGA-05-4417-01A 0.524844759 0.4295731150.447939152 0.34939096 0.267179275 0.537506843 0.421992674 0.431409095 0.349557686 0.499655015 0.418815578 0.4793864 0.360510217 0.3724451110.498628701 0.244427402

0.568090221 0.493879574 0.468817932 0.336194844 0.213558353
0.162770941 0.400221326 0.543654396 0.42808773 0.546095189
0.353365057 0.661046517 0.436120778 0.5372058110.335257326
0.301808274 0.419450939 0.617058699 0.409453144 0.535668471
0.425704887 0.433909067 0.354669462 0.491329705 0.332737062
0.36637719 0.391882854 0.146697405 0.372307721 0.388559821 -
0.096286074 0.329827458 -0.161428604 -0.009395082
TCGA-05-4418-01A 0.498334753 0.439056732 0.459356502 0.321012181
0.239461506 0.483035721 0.38327161 0.505135949 0.384603632
0.487227968 0.329360943 0.504806617 0.344166439 0.375103692
0.4198114760.158268726 0.528390303 0.591031256 0.515284802
0.292348665 0.209661395 0.048575508 0.380195013 0.565773899
0.438920061 0.583734279 0.42354053 0.695360879 0.501799722
0.435621746 0.306940324 0.377840366 0.450190005 0.683629358
0.457646418 0.603247612 0.458095933 0.437513656 0.275853232
0.3501182340.328873821 0.328275109 0.343966715 0.149498007
0.378745972 0.35663371 -0.069020927 0.292793249 -0.161819589 -
0.116756264
TCGA-05-4420-01A 0.360053083 0.362903573 0.444694573 0.340956245
0.206337687 0.441698669 0.283203676 0.496858232 0.463457742
0.4168281190.349849289 0.476777907 0.294204918 0.338998712
0.434360747 0.167719585 0.534493888 0.35320004 0.30481531 0.283641054
0.1152711330.04042802 0.294658394 0.558567863 0.408390747 0.566948454
0.489350856 0.699194231 0.483224856 0.403325086 0.202498953
0.272051201 0.414446878 0.640076876 0.447105762 0.499383935
0.389642053 0.441854171 0.272906379 0.38002064 0.306809182
0.304175693 0.28915109 0.148050998 0.367471782 0.198237869 -
0.006930828 0.189648796 -0.190708572 -0.139492122
TCGA-05-4422-01A 0.396991369 0.348881059 0.432024814 0.319257059
0.263153809 0.421575723 0.361343315 0.474601928 0.354966381
0.448070559 0.35045483 0.489289222 0.321422165 0.3375611320.455433998
0.170344781 0.536750663 0.481245363 0.443189969 0.262395538
0.085826887 0.11059411 0.345096269 0.542756692 0.420477978

0.537527278 0.382897168 0.672451029 0.485380866 0.249696971
0.237509773 0.333625935 0.465859069 0.651295033 0.383987489
0.563129813 0.439155386 0.42391333 0.252081831 0.347610283 0.33028092
0.320586233 0.319915966 0.198479075 0.397632491 0.343088081 -
0.108215741 0.231666579 -0.15856498 -0.077436211
TCGA-05-4424-01A 0.444668966 0.422541265 0.524763604 0.407191846
0.269635453 0.537700437 0.35361761 0.443126209 0.435348067
0.495543824 0.449088162 0.48296992 0.3468811860.366193316 0.520819162
0.212541644 0.547002897 0.514617666 0.436869703 0.341406814
0.206692287 0.1496311550.36515834 0.544152053 0.419856373 0.572598744
0.458614136 0.686145996 0.453478875 0.449987603 0.286898623
0.309083073 0.438649731 0.60266623 0.435909329 0.527264203
0.4177711350.435708053 0.369399968 0.444273807 0.334408105
0.330396419 0.345816585 0.171551294 0.397392693 0.311573283-
0.03553363 0.293932369 -0.127721991 -0.076417008
TCGA-05-4425-01A 0.5076311840.420519558 0.5011124040.334007495 0.255881753
0.528361778 0.419225966 0.467667715 0.366315258 0.516947703
0.442962587 0.479476469 0.353752198 0.385955714 0.469645396
0.210199007 0.5411408150.5614111550.499787591 0.339977662 0.211637514
0.19783264 0.390223652 0.5203661110.410187305 0.552234361 0.395640056
0.663772396 0.437842965 0.498930817 0.344644779 0.344219271
0.442176606 0.618288291 0.424876579 0.55832308 0.475912548
0.4361108540.329282366 0.416046249 0.322269515 0.379824089
0.378748862 0.212875957 0.408950562 0.362293336 -0.089330173
0.338315745 -0.119048257 -0.033568243
TCGA-05-4426-01A 0.446363248 0.448287802 0.442297225 0.349135216
0.22445503 0.506092939 0.3811815640.488758407 0.344690242 0.48882259
0.382743131 0.47256731 0.327549441 0.332713686 0.425240562
0.191035257 0.539671747 0.49772445 0.42541025 0.317906583 0.181032197
0.137626941 0.360902955 0.530192934 0.398657266 0.531564256
0.381669859 0.666079832 0.441731347 0.419380494 0.282922867
0.2789386 0.397175619 0.60531539 0.46347596 0.53883627 0.472790646
0.433410868 0.344086441 0.405009209 0.336935913 0.339935134

0.317086917 0.168774154 0.393648473 0.298951502 -0.096224443
0.267185148 -0.177774883 -0.073714427
TCGA-05-4427-01A 0.476394273 0.4023051110.440786613 0.445156827
0.220952744 0.54643967 0.353924498 0.456023495 0.463837919
0.493478141 0.353154265 0.454556862 0.306524729 0.307452165
0.441916025 0.170434155 0.576079477 0.502037644 0.442648339
0.314800366 0.177972682 0.1489711610.352945586 0.528798459
0.437545947 0.533722288 0.475169187 0.684630238 0.436826807
0.435343202 0.28567761 0.247587601 0.384431033 0.575007146
0.387199571 0.517463818 0.416596583 0.4221197840.355692278
0.408722937 0.338126455 0.291701742 0.335001465 0.135065768
0.352912024 0.31740251 -0.050779311 0.260032592 -0.217779725 -
0.101560069
TCGA-05-4430-01A 0.464776558 0.408180834 0.49255898 0.3115479550.25798341
0.528609495 0.390622772 0.459326905 0.3325711470.485161054
0.443896357 0.477301645 0.331532961 0.3598087110.462679903
0.235730265 0.522474461 0.512808268 0.463053264 0.373791179
0.201318097 0.141632535 0.380266584 0.539227284 0.416540969
0.534361803 0.355657088 0.664906307 0.428774554 0.535286218
0.312258443 0.294561796 0.405854455 0.612141521 0.424032388
0.522004438 0.432710374 0.437055481 0.35477435 0.47719461 0.327299378
0.3795753110.348435447 0.164714947 0.374985251 0.337045215 -
0.100834839 0.319864216 -0.141986182 -0.058769195
TCGA-05-4432-01A 0.434229916 0.426903306 0.501912823 0.362179345
0.253920593 0.519015218 0.340897467 0.466862643 0.417826336
0.462035121 0.388619251 0.468337926 0.350232553 0.367754251
0.438740256 0.206512489 0.544670858 0.437334282 0.404620161
0.304420968 0.1362174 0.1196254650.335151563 0.540309859 0.427707464
0.562805306 0.443142818 0.679846444 0.458245043 0.429336658
0.254504529 0.280581869 0.409017407 0.628300202 0.430270019
0.53563093 0.414786034 0.443764292 0.29268036 0.412662059 0.321636832
0.300939248 0.330127564 0.158189154 0.381384129 0.308854215 -
0.055214448 0.253394185 -0.158788468 0.019704577

TCGA-05-4433-01A 0.476629676 0.385526172 0.502263917 0.38963076
0.222031435 0.534095107 0.407335528 0.436285392 0.382667821
0.500770796 0.414421974 0.474245776 0.38180397 0.407443191
0.486303364 0.17574426 0.571087928 0.580180924 0.496573254
0.323212997 0.207743294 0.155381036 0.409387753 0.525061555
0.420625274 0.545227333 0.3637118710.651699241 0.414446413 0.34451217
0.299161697 0.342784655 0.436309846 0.583377888 0.41043672 0.54926369
0.460544315 0.430448332 0.370929566 0.342175021 0.33655742
0.340682585 0.372639121 0.179615786 0.38369087 0.349458115-0.111126201
0.309950376 -0.145003139 -0.009629786
TCGA-05-4434-01A 0.50085847 0.460913338 0.4611901030.363194352 0.25494886
0.51825567 0.42802527 0.479104842 0.416542461 0.519545139 0.373919395
0.472497842 0.382792048 0.4024630110.471024144 0.214766553
0.517793653 0.596168088 0.538557231 0.349892415 0.240467088
0.197230876 0.405665391 0.551481288 0.4171172050.586986153
0.439283256 0.695869844 0.492708845 0.504656065 0.334582695
0.338696422 0.434997734 0.61145436 0.45282002 0.575235871 0.478760244
0.446652557 0.338171847 0.39159908 0.3312801160.354764586 0.374377177
0.163802341 0.3874117090.399472626 -0.108247228 0.309438598 -
0.135980201 -0.073887604
TCGA-05-5420-01A 0.467323465 0.381222658 0.501546836 0.256359987
0.169161595 0.409592321 0.468266788 0.507179293 0.375476461
0.490262799 0.278361384 0.482441231 0.246772897 0.318538921
0.454757707 0.1118615140.529871827 0.578466388 0.55710051 0.238539607
0.126173041 -0.007111607 0.42415136 0.548413619 0.424029926
0.586907652 0.427819635 0.693774579 0.4892694110.378180316
0.356477307 0.366050451 0.466006102 0.679663321 0.403370271
0.562634015 0.410069405 0.440053422 0.233007494 0.360968763
0.301479493 0.340547294 0.366630783 0.185542768 0.396807518
0.458269526 -0.061731882 0.30136979 -0.190721849 -0.050922199
TCGA-05-5423-01A 0.452647577 0.366312095 0.493994219 0.245724195
0.149509677 0.435802832 0.389819905 0.482157272 0.290065307
0.486204148 0.3211468290.490191669 0.303302418 0.360020654 0.45866015

0.182414403 0.547810993 0.537012518 0.483303189 0.282266596
0.10503874 0.077685929 0.373182667 0.514969929 0.409129884
0.538876094 0.355453288 0.657149483 0.413419496 0.361830968
0.317397395 0.319596905 0.462418104 0.666050502 0.394611898
0.524431783 0.43204621 0.435786398 0.266755563 0.339848753
0.324275163 0.3121169370.344938061 0.232396072 0.419865697
0.363747748 -0.093446245 0.307010464 -0.169767521 -0.089992799
TCGA-05-5425-01A 0.514008275 0.404353442 0.507068139 0.343508612
0.220806153 0.497402554 0.424547302 0.486098607 0.42140894 0.49969221
0.338034715 0.494754654 0.341497576 0.3814118820.464436286
0.174146537 0.534389925 0.548947447 0.508401023 0.312735957
0.188146225 0.080839032 0.407191553 0.55452107 0.451958691
0.568964036 0.446384873 0.697969124 0.491948444 0.423841706
0.340669505 0.350631006 0.455993514 0.660238309 0.425720515
0.54581545 0.439179547 0.465347713 0.298342521 0.410866226
0.318381079 0.357154248 0.366054886 0.179840883 0.395978966
0.390150292 -0.073027076 0.312620788 -0.156947985 -0.038215004
TCGA-05-5428-01A 0.399829107 0.401600128 0.472659758 0.281864723
0.154775876 0.4309041170.342847173 0.507386237 0.396617012
0.453697083 0.347227524 0.495193737 0.2731561160.328027546
0.430423224 0.138695526 0.517663459 0.433152913 0.398331237
0.258947882 0.173331938 -0.001540884 0.326435049 0.530044451
0.390364664 0.576332616 0.464126976 0.709042239 0.506115304
0.391003822 0.244704858 0.320627375 0.445179938 0.671825688
0.450716023 0.583257793 0.408549516 0.420652741 0.247680092
0.310940961 0.322024635 0.271082544 0.301413759 0.167608752
0.400689071 0.281850855 -0.027554836 0.237275013 -0.17113405 -
0.099546933
TCGA-05-5429-01A 0.482843805 0.417804508 0.55322567 0.329401914
0.183723613 0.50829173 0.294252538 0.482956701 0.379871082 0.49173215
0.401246406 0.481855863 0.353142687 0.38804159 0.447830716
0.165171016 0.538701758 0.481973389 0.384555126 0.293202977
0.159351747 0.072865188 0.33563419 0.505247285 0.378769251

0.5522118360.4115339860.680628747 0.453506514 0.454167627 0.228742632
0.352024614 0.483823517 0.650641523 0.457456243 0.510571405
0.464064658 0.435787966 0.308053887 0.353654808 0.300407316
0.329029541 0.295099854 0.196726797 0.419631059 0.223612273 -
0.090919652 0.291871645 -0.156331685 -0.008626277
TCGA-05-5715-01A 0.383175361 0.3729087 0.519757322 0.329377091
0.224058299 0.482589686 0.324909009 0.456774166 0.351937251
0.46616177 0.416534134 0.481851057 0.345686977 0.372961881
0.480635377 0.196157288 0.562587734 0.466518854 0.405637204
0.324388702 0.192479582 0.1243597110.371025618 0.519569638
0.405353787 0.550400193 0.3731193850.651825486 0.450338471
0.437016314 0.246844963 0.31840931 0.428683745 0.615780469
0.421362797 0.525306479 0.431492689 0.4085211820.318955203
0.383142204 0.322785294 0.3733611710.319955778 0.167202159
0.403289665 0.269113499-0.087141521 0.289059735 -0.149959529 -
0.050125518
TCGA-35-3615-01A 0.396202094 0.326239814 0.436355618 0.267586868
0.214150072 0.463641247 0.326205069 0.485204236 0.258835973
0.441203378 0.371558064 0.495877691 0.322243057 0.341133883
0.432794632 0.155761445 0.548365661 0.437247226 0.394304561
0.262527981 0.069965791 0.101018012 0.307963428 0.530848746
0.3972110680.506786274 0.304164851 0.673034076 0.462816071
0.329897262 0.209521485 0.3276152 0.461629469 0.664564624
0.368903785 0.590109603 0.456332194 0.420122888 0.288263299
0.335554135 0.344479638 0.278833994 0.284027985 0.192321253
0.393891821 0.267653048 -0.143220982 0.215741465 -0.154356457 -
0.030926752
TCGA-35-4122-01A 0.525692927 0.428305833 0.473642587 0.269723581
0.1581024110.477039683 0.449004379 0.494659468 0.402144625
0.504783199 0.331928573 0.480379345 0.284022385 0.341844547
0.442752106 0.19060072 0.525323173 0.570451273 0.535279127 0.30891438
0.174373069 0.050974172 0.403332496 0.543689513 0.424412527
0.593206222 0.457874872 0.703084514 0.495629428 0.467537964

0.380723501 0.314705882 0.433903051 0.650707123 0.457397858
0.55724952 0.429816654 0.458261687 0.277745164 0.402804283
0.308166217 0.315084168 0.386050873 0.171896554 0.390689047
0.430487891 -0.04000621 0.335048317 -0.179054643 -0.042008745
TCGA-35-4123-01A 0.540061708 0.432319586 0.459296037 0.314562437
0.202284817 0.51556438 0.449316998 0.506547494 0.409295855
0.530271831 0.367089279 0.469087491 0.2753881190.334738571
0.417552971 0.170568563 0.516576549 0.588484881 0.542770078
0.324988726 0.186836896 0.1018641 0.387589843 0.526347492
0.431777477 0.570979277 0.451216072 0.695338824 0.47950212
0.488833388 0.3664695 0.27765307 0.40264409 0.638036959 0.458312144
0.551083689 0.440549935 0.442238898 0.304824308 0.402700656
0.310399841 0.301057836 0.389755655 0.133443254 0.373043668
0.45119548 -0.020572906 0.296087825 -0.187235424 -0.068053124
TCGA-35-5375-01A 0.390107392 0.4111247670.457044206 0.284433421
0.2118558280.406766928 0.327781482 0.521398696 0.431770596
0.448574349 0.380047886 0.45374246 0.253265047 0.313489974 0.42723171
0.143775491 0.516275637 0.452708035 0.415950989 0.242010543
0.187793423 0.082959964 0.334666706 0.53030019 0.398750843
0.599320712 0.51523419 0.715804143 0.489984562 0.429721343
0.2391167820.338162022 0.460277097 0.667221017 0.45884131 0.5688363
0.433773529 0.423835073 0.273419955 0.354955866 0.301191374
0.259158247 0.298998928 0.166406287 0.385134414 0.332381636
0.019882088 0.223926459 -0.137177113 -0.082080251
TCGA-38-4625-01A 0.467906717 0.392280734 0.471613876 0.38844648
0.229891576 0.431640946 0.387776252 0.525858164 0.497022042
0.488156926 0.345892508 0.49186111 0.296073147 0.338141421 0.4346613
0.155194496 0.53582096 0.609494744 0.52238127 0.320391731 0.176423486
0.088077553 0.345817723 0.559871288 0.420910323 0.602899686
0.555298361 0.734677479 0.582400261 0.413174939 0.310144486
0.302514579 0.427418904 0.636483812 0.432296938 0.569702351
0.41240698 0.44775302 0.29788818 0.361672106 0.314950244 0.305775944
0.334184489 0.155293878 0.384750293 0.344131677 -0.028890224

0.246124997 -0.192344921 -0.1230414
TCGA-38-4626-01A 0.556004255 0.435348334 0.549584142 0.329081609
0.246537402 0.539448214 0.466953205 0.43305648 0.297467185
0.502673239 0.395607799 0.525272354 0.352044747 0.368279373
0.500404162 0.237823046 0.543722232 0.537424613 0.502757919
0.35181592 0.21362681 0.138956209 0.413933149 0.532046215 0.418931861
0.563139092 0.298146213 0.637467892 0.40303856 0.493034081
0.393969165 0.340661744 0.446125898 0.619357611 0.391704021
0.562644049 0.470675517 0.446336654 0.379129294 0.472712451
0.348079392 0.369174189 0.389438829 0.198980352 0.3949087 0.35978757
-0.125528325 0.331591706 -0.163944909 -0.040423883
TCGA-38-4627-01A 0.479099632 0.452786464 0.47052502 0.301126708 0.259673059
0.547561223 0.379389209 0.441544751 0.295233525 0.503777698
0.45069467 0.482239155 0.333603257 0.34050562 0.501086337 0.277429744
0.541634897 0.496063269 0.45818364 0.386056309 0.225181666 0.2095045
0.389387656 0.526513251 0.400381053 0.551533283 0.313081436
0.64604864 0.37273972 0.641527095 0.334721154 0.326684796 0.415159012
0.604098384 0.434273536 0.552067415 0.447542506 0.415943173
0.391399038 0.495510254 0.329100826 0.382385151 0.366483622
0.170643559 0.378896395 0.338988265 -0.11524471 0.352773778 -
0.145361218 -0.025716792
TCGA-38-4628-01A 0.35734425 0.345661441 0.493615074 0.284083168
0.195542683 0.472884002 0.299403129 0.504011193 0.344335133
0.457404939 0.389203781 0.499546161 0.306742701 0.344802979
0.457154083 0.21213201 0.530488073 0.419130223 0.360647891
0.308288964 0.148571416 0.071040284 0.334822509 0.514022067
0.41174278 0.539907092 0.415743865 0.679142136 0.444651076
0.453767644 0.232820613 0.310220703 0.433743908 0.654356247
0.418105768 0.515329269 0.428917011 0.422712849 0.288033657
0.403897753 0.317699478 0.311426768 0.300751238 0.196454311 0.404809529
0.254407725 -0.04856095 0.258717901 -0.139519117 -0.101672806
TCGA-38-4629-01A 0.520611765 0.47466112 0.511139783 0.354626341 0.245790861
0.530216197 0.428703539 0.486291615 0.421384695 0.515239975

0.3955117380.476294057 0.315789549 0.350402059 0.477041605
0.2101981120.541495393 0.550147537 0.516824496 0.369216722
0.2369621170.148036637 0.396398612 0.545489509 0.421401805
0.601783317 0.464524129 0.702630902 0.494205366 0.550795423
0.372930358 0.305980872 0.422420832 0.637715158 0.482285076
0.554032047 0.43592112 0.437237188 0.34211018 0.478017421 0.305405676
0.340170628 0.371692165 0.151902404 0.383298398 0.406035246 -
0.081442293 0.339396895 -0.211558878 -0.079890134
TCGA-38-4630-01A 0.322523137 0.362845698 0.4711391580.432817041
0.270522443 0.440636615 0.252313222 0.506161012 0.52676979
0.415066824 0.349275751 0.417545536 0.227773759 0.262439557
0.375051044 0.175530827 0.48621725 0.471437752 0.366573665
0.264900093 0.132823521 0.176745788 0.245784762 0.57443899
0.415706735 0.578687014 0.577878258 0.727679552 0.57256017
0.422467872 0.152451029 0.229407875 0.378607432 0.607712415
0.402707616 0.492853133 0.35645705 0.4114209310.283429525 0.32049561
0.279372244 0.216546772 0.255754472 0.104613369 0.372907765
0.207548139 -0.002708703 0.151883994 -0.113356054 -0.055975843
TCGA-38-4631-01A 0.462393385 0.384716831 0.4883060110.325935166
0.160780191 0.455479179 0.325788702 0.523899237 0.475979447
0.477140951 0.3126361160.461902321 0.263726944 0.324084665
0.418579841 0.134259721 0.526604656 0.520502175 0.433772004
0.272472675 0.122320079 0.05236718 0.347464228 0.544300436
0.426619241 0.598773997 0.537599964 0.727464032 0.525795898
0.395833654 0.247990014 0.329959809 0.424861046 0.654747075
0.451699663 0.585855104 0.426420463 0.455560263 0.234276799
0.288504958 0.300566343 0.302244477 0.301630546 0.141088116
0.388673817 0.259754381 0.001802715 0.225273982 -0.170597623 -
0.058634367
TCGA-38-4632-01A 0.499353545 0.404364703 0.495302886 0.322266929
0.191414077 0.474557154 0.42206216 0.494291253 0.436293936 0.49693922
0.364167075 0.494530779 0.325471436 0.372400024 0.446442811
0.179013851 0.530428426 0.4811262610.447486616 0.31988491 0.19442527

0.085507856 0.363251622 0.554745836 0.4154512 0.586453419
0.487028058 0.702359175 0.497395656 0.438088582 0.341212892
0.3115217380.425932505 0.654561309 0.45139439 0.559616802 0.426980303
0.450540672 0.275897189 0.413318274 0.316272252 0.330925833
0.382903252 0.183020016 0.421403336 0.364759202 -0.042298771
0.310820265 -0.168838053 -0.092794326

TCGA-38-6178-01A 0.419615462 0.416610225 0.43458857 0.359589553 0.29629825
0.5337027 0.332722999 0.448084505 0.344645664 0.485254843
0.437890327 0.462610827 0.345786507 0.349006849 0.483240365
0.218693638 0.548162016 0.541885595 0.462760095 0.365898365
0.191225053 0.190234598 0.3411424970.545697344 0.417933593
0.545291899 0.35649679 0.667000608 0.487308777 0.5108115560.243358279
0.290406147 0.39115538 0.581739353 0.444884265 0.518855704
0.432527073 0.424273171 0.365595934 0.421634875 0.32861075
0.337604832 0.318052622 0.127546956 0.382898308 0.272581451 -
0.1148988120.259204929 -0.134017818 -0.060367422

TCGA-38-7271-01A 0.5527645110.412033228 0.47168865 0.334935866 0.2792549
0.549253899 0.446551058 0.43738611 0.324960695 0.528646409 0.44232431
0.475829577 0.355496462 0.366077348 0.500989763 0.219281818
0.522810589 0.492383632 0.501023143 0.362662437 0.239863951
0.2081136670.42693395 0.530109253 0.428221332 0.527145631 0.335361639
0.654598253 0.428397678 0.492330194 0.367380849 0.307535194
0.399745017 0.594778267 0.376547166 0.54247352 0.453087958
0.430883828 0.382772719 0.430790323 0.323413731 0.366008975
0.419541864 0.168045877 0.371888248 0.464309145 -0.124014952
0.337469819 -0.133732488 -0.016240752

TCGA-38-A44F-01A0.534042399 0.419000608 0.463035024 0.279911441
0.275909228 0.535193872 0.444135421 0.452025231 0.241229424
0.517582588 0.419056839 0.475719282 0.350846976 0.389802205
0.441769817 0.229944701 0.504218406 0.579509754 0.508843493
0.342672999 0.206752426 0.1195645560.398991275 0.494204467
0.382236781 0.49817458 0.256186443 0.623758906 0.367246929 0.48417557
0.329175717 0.330482096 0.421413884 0.602722571 0.406261772

0.537349363	0.486379234	0.43196349 0.329585562	0.434521895	0.32165671
0.378465102	0.383073331	0.164725498	0.372825085	0.380264449	-
0.131333853	0.348020673	-0.121793441	-0.049496977
TCGA-44-2655-01A 0.44807328 0.372779449	0.463702225	0.284241823
0.223666802	0.51952661 0.338755052	0.47554501 0.266581899	0.487093491
0.351812597	0.514761068	0.341965996	0.36553759 0.444426864
0.196127726	0.546003746	0.474852122	0.417761626	0.296793217
0.150473203	0.090528576	0.350635154	0.512553121	0.394354368
0.527958146	0.304156388	0.667348885	0.423760743	0.379854831
0.251514018	0.355163263	0.478981749	0.650815845	0.399360343
0.592373282	0.475485725	0.431445862	0.318106269	0.330386648
0.34857813 0.331079739	0.325034537	0.195978913	0.413905919
0.296112569-0.128014174	0.26227629 -0.14974397-0.084016183
TCGA-44-2656-01A 0.49502481 0.4204611550.49563063 0.347657412	0.192115713
0.507048482	0.413887104	0.468882201	0.381742271	0.518474931
0.397731865	0.490877866	0.329250445	0.350371776	0.495720841
0.175022457	0.566074055	0.584257257	0.536581796	0.334007002
0.191315016	0.092449787	0.402661308	0.5511193130.437203969
0.587233904	0.402710235	0.680894977	0.467501601	0.429975971
0.333554848	0.324580708	0.436861341	0.628259413	0.440405878
0.573017822	0.438705252	0.433682498	0.343420444	0.405766328
0.3530741130.32452447 0.374304464	0.173921875	0.407531095	0.421336028
-0.105591466	0.309716292	-0.194416404	-0.085609933
TCGA-44-2657-01A 0.439213437	0.391986516	0.441040807	0.307851045
0.2117183090.495773909	0.423143517	0.462466609	0.3343953	0.503718864
0.402762347	0.468635987	0.321350524	0.360871478	0.461817942
0.207093718	0.523756359	0.54980489 0.520408427	0.3267111250.169507242
0.1187333640.389970371	0.533394982	0.433659248	0.526594094
0.364366415	0.65790215 0.413715261	0.435606928	0.301073831
0.320009401	0.425657035	0.6156256 0.405622206	0.532921923
0.451728894	0.431320694	0.317503688	0.394601538	0.323413476
0.315409633	0.374046386	0.157108699	0.378391279	0.446272195	-
0.105098697	0.307085298	-0.131687339	-0.069274319

TCGA-44-2659-01A 0.402329615 0.37681002 0.446059433 0.320659165
0.250708678 0.527174037 0.348684899 0.452916245 0.312787663
0.491398987 0.449896301 0.465710676 0.326542564 0.318883434
0.482730324 0.204777394 0.569641928 0.521256305 0.468885754
0.335026431 0.181284505 0.144035098 0.350391379 0.553424517
0.42212896 0.526589121 0.319056704 0.652792412 0.43036335 0.447428065
0.258262071 0.306580059 0.414303854 0.608766242 0.387539698
0.530275064 0.422127791 0.419099906 0.365730736 0.411210276
0.345634314 0.302896571 0.341987128 0.174658544 0.389748243
0.348966563 -0.11444128 0.273809883 -0.180458003 -0.037344836
TCGA-44-2661-01A 0.470388369 0.3711897720.457326505 0.251323073 0.16605585
0.4804211930.457982626 0.472319592 0.270552515 0.507882125
0.377123151 0.490690679 0.3074110290.334232272 0.4508115460.192117394
0.539962748 0.565406209 0.52462686 0.313339928 0.149367596
0.066703194 0.403312579 0.505453281 0.408090604 0.519819794
0.314291544 0.650503463 0.378981829 0.452769404 0.344207167
0.329444319 0.440837332 0.644620772 0.381720741 0.536155169
0.451857247 0.429335719 0.295000576 0.3811375140.329424673
0.3411326430.391360205 0.18512254 0.391930629 0.44444531 -0.114564026
0.325432742 -0.165431534 -0.077077132
TCGA-44-2662-01A 0.5062686 0.47130036 0.510410613 0.446563383 0.213809023
0.520346547 0.413996435 0.461708915 0.473152767 0.497093456
0.418416655 0.455747988 0.319276179 0.3040411790.494724831
0.150521292 0.59631401 0.464717402 0.440072596 0.324997592
0.151379127 0.139045085 0.390453769 0.55611828 0.4413425110.612301881
0.493239948 0.689528849 0.445070864 0.503699088 0.359815026
0.302890461 0.396363935 0.599906188 0.487471685 0.548567623
0.425281539 0.419039227 0.363202092 0.446924483 0.32722705
0.302535579 0.362204136 0.160431255 0.3863117560.347559297 -
0.033882812 0.324329309 -0.205162641 -0.131962581
TCGA-44-2665-01A 0.476232935 0.448450344 0.429326458 0.311956941
0.257765433 0.5322390110.39505481 0.456782144 0.307828048 0.499227547
0.422053548 0.443281869 0.308322223 0.325465956 0.469226492

0.2116889050.560458689 0.503197104 0.469666647 0.359820641 0.23174139
0.199027275 0.377087195 0.552100033 0.389848939 0.535422182
0.330627036 0.659260004 0.413970261 0.603085661 0.317068351
0.296022484 0.375375224 0.588044193 0.427391064 0.536320118
0.432017248 0.413517042 0.355975426 0.443691275 0.313318853
0.356045142 0.35349594 0.128958655 0.363505649 0.337548799 -
0.14857395 0.342642399 -0.193026877 -0.0181186
TCGA-44-2666-01A 0.42462368 0.383847968 0.485650665 0.356808437
0.250624842 0.526087713 0.270448523 0.455173714 0.348500439
0.456540379 0.3736382 0.482095995 0.331248536 0.34337535 0.494079351
0.14679902 0.587414976 0.539616761 0.4336687110.277733392 0.097430989
0.129779795 0.316608584 0.532993008 0.399170561 0.527235642
0.393315273 0.683718514 0.471343413 0.363222279 0.228889354
0.277553995 0.443967459 0.629723418 0.419376143 0.496039917
0.456266377 0.418733382 0.378759589 0.348935802 0.312406078
0.289107372 0.30889926 0.197225381 0.394270723 0.216110971-0.073069834
0.235323082 -0.130029105 -0.095808069
TCGA-44-2668-01A 0.607103012 0.466536709 0.42225776 0.476160623
0.227995994 0.5506113290.514006219 0.440014293 0.508550665
0.537366624 0.393769543 0.432421572 0.30174117 0.3182922 0.476611157
0.155715656 0.58008198 0.597460762 0.55243583 0.355887689 0.222822017
0.149552453 0.43048905 0.554369655 0.459320451 0.579050363 0.50332928
0.690724293 0.447179501 0.560466437 0.442197127 0.269862482
0.361979681 0.569024825 0.445993452 0.538001673 0.43498648
0.439258041 0.382358417 0.478181662 0.321925468 0.337518745
0.403249897 0.1169210590.344002423 0.406577849 -0.058828804
0.358256766 -0.203946787 -0.103843458
TCGA-44-3396-01A 0.579366808 0.437760788 0.460507971 0.351518038
0.22397058 0.4971119130.482608208 0.490006463 0.396869094 0.519234357
0.399658532 0.464523928 0.306212489 0.323497205 0.45210927
0.190282871 0.527381709 0.520786064 0.509578018 0.339018813
0.224087259 0.140218654 0.399666134 0.537992808 0.410197535
0.567281408 0.443520376 0.689224376 0.457421345 0.547442538

0.384645419 0.288044797 0.392455904 0.601541607 0.438081248
0.5255961110.436827027 0.443323885 0.323827324 0.4594101130.313336105
0.34216969 0.381518454 0.171972893 0.371943847 0.406751396 -
0.097291874 0.32212864 -0.207804923 -0.121553426
TCGA-44-3398-01A 0.50282275 0.474479584 0.461520947 0.243184128
0.178170842 0.518263373 0.45044271 0.481010169 0.279964376
0.510997859 0.403780954 0.503614994 0.306767067 0.351402043
0.446018636 0.2131041 0.55759543 0.479617935 0.47099379 0.331516749
0.186773635 0.0941183720.417099195 0.52576592 0.399273419 0.55407411
0.322990109 0.657074236 0.370651915 0.55862763 0.351895245
0.3481130150.449253906 0.646282332 0.461226415 0.542928522
0.464362269 0.436669006 0.341528156 0.45484357 0.331201412
0.380249563 0.38205997 0.181697182 0.401293734 0.377506402 -
0.1188984720.370087781 -0.169846496 -0.054639322
TCGA-44-3917-01A 0.348962827 0.325460758 0.420840504 0.376341306
0.1421181870.464894635 0.323243762 0.482612729 0.485699868
0.434904469 0.29078197 0.45754584 0.248105835 0.293138281 0.430336384
0.1100430780.576092188 0.452051371 0.428503843 0.232654804
0.038566592 0.070064043 0.339651925 0.563088528 0.403221609
0.584018893 0.529941272 0.722031763 0.480344737 0.397761183
0.243205255 0.281646513 0.415178 0.653269988 0.417035814
0.530704978 0.343466679 0.4098353 0.298102661 0.3428584 0.304915387
0.2748301 0.283544607 0.188172105 0.391898304 0.308852469 -
0.002356196 0.216201254 -0.193281032 0.056629425
TCGA-44-3918-01A 0.507882691 0.426969569 0.438683271 0.280216483
0.186924271 0.506541361 0.456568507 0.480713663 0.353106981
0.517360803 0.400332136 0.45596 0.306874707 0.335108008 0.440426351
0.18230053 0.536886161 0.573587698 0.545146065 0.306323449
0.213171521 0.0992416 0.4072753 0.548856206 0.419144865 0.551391602
0.38719198 0.683853168 0.414045889 0.512438581 0.369839002
0.282234392 0.395425493 0.61939113 0.4311790860.528482366 0.427153934
0.4421194970.308206997 0.401986858 0.31878142 0.328500703 0.378936298
0.12968188 0.35985756 0.458973198 -0.056324532 0.318000979 -0.17078069 -

0.104002647
TCGA-44-3919-01A 0.445803546 0.408739385 0.45310307 0.355560691
0.233187187 0.522596924 0.422046654 0.468621448 0.37868066
0.503146765 0.424684429 0.460995514 0.337887072 0.370476765
0.453257185 0.188771253 0.535322163 0.602324278 0.527125061
0.322551427 0.198294779 0.132267726 0.382790385 0.52221636
0.432755328 0.556099909 0.419957295 0.676963354 0.411948309
0.456822033 0.313078541 0.300754997 0.406229505 0.593857659
0.436236674 0.52235813 0.444845295 0.435829383 0.337705321
0.387590619 0.339878322 0.347186601 0.370868657 0.16451827
0.382533265 0.391197909-0.100654503 0.290856355 -0.147073381 -
0.084436736
TCGA-44-4112-01A 0.464890349 0.4428509110.45048522 0.312718814 0.229257078
0.515734555 0.3191148530.493190858 0.382209198 0.489879666
0.402478179 0.47533723 0.327460916 0.344029949 0.458002318
0.205970753 0.557910718 0.451361533 0.423187129 0.313895304
0.162947374 0.127351577 0.331876256 0.540143838 0.425510058
0.543773503 0.413381639 0.692402638 0.449356315 0.509781218
0.277304155 0.296712523 0.410361281 0.634392552 0.449126224
0.563061288 0.44427577 0.449806254 0.334558095 0.418762559
0.337892662 0.340280022 0.327535093 0.150781585 0.38913415 0.29128569
-0.07443375 0.290651686 -0.175785694 -0.074201017
TCGA-44-5643-01A 0.420389735 0.3641138660.475151745 0.407936266
0.328859833 0.434606434 0.304020587 0.4876911680.49972099 0.457798874
0.389606598 0.449105664 0.348061343 0.362763208 0.431981061
0.131593666 0.515184094 0.504933319 0.427983514 0.286315418
0.271588844 0.13483542 0.283683537 0.556220035 0.457944496
0.596823716 0.52848273 0.737095628 0.582970697 0.281616737
0.213770304 0.267826868 0.41272293 0.645624433 0.401454838
0.539019181 0.466308056 0.4362811750.285788237 0.291394133 0.29578124
0.174142331 0.303746509 0.143476137 0.372737627 0.286503251 -
0.036766491 0.175620028 -0.082498992 -0.158537981
TCGA-44-5644-01A 0.394008193 0.418234026 0.416407772 0.356258554

0.224368713 0.496765767 0.244261496 0.48647077 0.436895442
0.425050343 0.299663682 0.417536725 0.32970567 0.342995785
0.421276737 0.15752365 0.541695975 0.31465598 0.265373281 0.239375512
0.072982721 0.209457469 0.309537796 0.590893644 0.408321444
0.557215249 0.467224938 0.688988364 0.506064599 0.384117538
0.138191037 0.275645562 0.366278659 0.622834596 0.416482419
0.491986682 0.373572691 0.434693624 0.269734892 0.344561018
0.298107815 0.299144739 0.263656512 0.102582917 0.33532837
0.166287791 -0.002945128 0.19979279 -0.176978843 0.220621101
TCGA-44-5645-01A 0.42376821 0.339624439 0.413157647 0.329475223
0.253082727 0.521265354 0.382440417 0.445437217 0.275650165
0.499952156 0.423438249 0.46369401 0.3165501180.31767846 0.470120909
0.1840548 0.563127343 0.465942401 0.432497208 0.327313468
0.174931095 0.172263167 0.358142846 0.491655406 0.421505965
0.489120967 0.302738258 0.63798976 0.370788082 0.393382302
0.278506802 0.310494718 0.421829176 0.599949289 0.356864628
0.521872477 0.459501496 0.401439232 0.358167618 0.366838796
0.334789973 0.284076741 0.344317213 0.187263903 0.383189089
0.376209736 -0.116646626 0.282765048 -0.154321205 -0.085278949
TCGA-44-6145-01A 0.492708604 0.426108395 0.442155375 0.396186949
0.239885335 0.546340347 0.405691774 0.46257266 0.395945348
0.507355882 0.34643798 0.469259064 0.363348989 0.382999566
0.474220452 0.189784705 0.558350892 0.552392276 0.520594158
0.336959553 0.24025695 0.146595615 0.418899294 0.510636515
0.439414952 0.548271498 0.415453464 0.664968853 0.390861101
0.496535582 0.313148537 0.34607314 0.415209554 0.585060064 0.42840188
0.58073813 0.448542205 0.42224665 0.369072263 0.372849007 0.34339572
0.36662628 0.367192344 0.170956484 0.38605385 0.40176693 -0.098658961
0.315632149 -0.166892399 -0.070078811
TCGA-44-6146-01A 0.433784556 0.369490843 0.475815346 0.380846695
0.210358389 0.547606543 0.28170758 0.409793777 0.297996726
0.468450256 0.394803027 0.465699439 0.371772899 0.381145806
0.5112203420.141570647 0.606158499 0.477706136 0.366688366

0.288189177 0.164445653 0.174016729 0.311115673 0.512740871
0.414914041 0.503047831 0.278036547 0.618284543 0.361507036
0.331816165 0.202487266 0.312371827 0.430795444 0.575869065
0.392217062 0.492201718 0.449216299 0.390535022 0.395955226
0.325449241 0.34274044 0.27723082 0.30851326 0.187511418 0.377537847
0.173301754 -0.113795755 0.236939808 -0.159866404 -0.087583447
TCGA-44-6147-01A 0.438046304 0.420059846 0.467302465 0.321326854
0.255712977 0.522592987 0.353470008 0.44189758 0.294352666
0.475510394 0.418705033 0.465105128 0.371191302 0.373790324
0.510764517 0.222968508 0.532784348 0.519164562 0.456668479
0.349397587 0.197749158 0.17137487 0.352766781 0.533605816
0.398772145 0.517706591 0.315123921 0.647219357 0.43545187
0.483125707 0.263253618 0.300466881 0.394127911 0.598327424
0.393340254 0.519289876 0.44747341 0.409707213 0.375485453
0.466216957 0.329799012 0.326690222 0.350921904 0.168278151
0.391740785 0.306860103 -0.093415766 0.271203449 -0.119818459 -
0.038743057
TCGA-44-6148-01A 0.421639664 0.388603429 0.419796446 0.299457597 0.30636
0.526745223 0.333905467 0.424925248 0.211539876 0.480550636
0.456072075 0.464984529 0.355424045 0.357192395 0.478342631
0.214829366 0.525125371 0.412357681 0.355859892 0.337404348
0.2328272 0.188844736 0.30757012 0.474485771 0.376292226 0.450484858
0.221895858 0.604087775 0.327197184 0.41612045 0.220665802
0.325871524 0.421192584 0.590637604 0.366470428 0.49343017 0.464060808
0.390970583 0.406435173 0.410732712 0.328104212 0.30965321
0.329919386 0.198424528 0.37693232 0.248031176 -0.107705192 0.273385496
-0.093542311 0.008838404
TCGA-44-6774-01A 0.51104301 0.445695109 0.458265685 0.372355413
0.314552879 0.579706807 0.404970091 0.427559539 0.367600616
0.496493047 0.483890442 0.457967941 0.372941518 0.366741321
0.495818334 0.263955128 0.552312389 0.466356349 0.447647118
0.399098337 0.217501802 0.220373328 0.37459656 0.554287952
0.406131607 0.547573878 0.372898708 0.664398785 0.450285302

0.647481047 0.329504757 0.291323298 0.380836783 0.586594861
0.421539376 0.500767429 0.424656154 0.435601881 0.399469865
0.499560774 0.310840168 0.359285843 0.367410984 0.139785133
0.363527248 0.341525317 -0.106540379 0.330564716 -0.158390111 -
0.005733327
TCGA-44-6775-01A 0.469232493 0.423138241 0.512406457 0.385124654
0.238819531 0.544391267 0.389765477 0.445144941 0.403083851
0.505595531 0.434514401 0.457366385 0.346854228 0.354445668
0.487297678 0.202028499 0.554722077 0.5334011790.483777437
0.354966672 0.215535002 0.158186456 0.382385462 0.513219325
0.420690827 0.568754627 0.437500828 0.665092469 0.403811338
0.574610716 0.329841533 0.304801082 0.408949404 0.581563262
0.44686608 0.528098473 0.4332267110.430986547 0.383700579 0.479328818
0.325644883 0.359632673 0.363877496 0.169540621 0.388261642
0.356998167 -0.091163918 0.346403808 -0.156625186 -0.078541789
TCGA-44-6776-01A 0.365584866 0.355154277 0.480626529 0.315695823
0.26438906 0.505721692 0.256480269 0.446426313 0.271780049
0.452960744 0.419551484 0.505481418 0.369532498 0.362041387
0.47822317 0.187693585 0.562312085 0.348071201 0.300025368
0.305154857 0.129992267 0.142837169 0.281606369 0.509431844
0.404768872 0.508095163 0.2907981 0.642678042 0.427712369
0.325003321 0.15900743 0.330399369 0.472185585 0.64920983 0.395636915
0.554353213 0.4428037 0.413716696 0.332948273 0.328375655
0.355633337 0.262868043 0.283004374 0.189303388 0.415820001
0.19323801 -0.136854229 0.166637186 -0.106964555 -0.031321692
TCGA-44-6777-01A 0.488560478 0.471429043 0.476807085 0.357765149
0.272366684 0.555414073 0.442030918 0.428824741 0.325707057
0.515173533 0.472292552 0.491301684 0.347403394 0.343231489
0.510993024 0.25480101 0.5705118730.514324781 0.49942888 0.377521969
0.227271381 0.19590738 0.425980031 0.536238504 0.420910767
0.554728051 0.333868995 0.639246809 0.37377238 0.574481548
0.374408338 0.315283819 0.416023454 0.595173639 0.438488099
0.553524172 0.432214312 0.424035005 0.3883116460.503286766

0.350394852 0.382886257 0.405954615 0.176442795 0.381809417
0.424979092 -0.107212547 0.365913153 -0.170346284 -0.020498278
TCGA-44-6778-01A 0.5383152 0.408403872 0.462661578 0.402436123
0.270258044 0.546462747 0.458372464 0.450409195 0.457034938
0.532108903 0.379471425 0.464562197 0.314989073 0.339130716
0.470854007 0.185569741 0.556020526 0.521958544 0.513708859
0.311817811 0.156996051 0.195822842 0.4098047 0.540163521 0.437591517
0.572672803 0.467961531 0.68815679 0.439704797 0.413222627
0.368654592 0.300537222 0.411328758 0.594297341 0.356835641
0.556493633 0.440293746 0.457675466 0.361123113 0.400556422
0.337764237 0.305295323 0.394169513 0.153818261 0.357932999
0.438674039 -0.021841786 0.318602546 -0.166719027 0.146052711
TCGA-44-6779-01A 0.509226136 0.406240695 0.438692348 0.430303687
0.271087377 0.540623974 0.411558188 0.455946084 0.461667673
0.516934639 0.401718356 0.477734512 0.379934951 0.379823912
0.492187669 0.175687555 0.569273749 0.584984198 0.542175158
0.364498773 0.263290768 0.184791821 0.409252333 0.505761951
0.446815603 0.558301033 0.464299039 0.672877686 0.407267169
0.490492665 0.336641378 0.319123539 0.404578162 0.571913045
0.432517997 0.560148726 0.447942595 0.413285849 0.368951248
0.40167539 0.341106401 0.314937808 0.353900878 0.143784231 0.372612619
0.411159344 -0.081121148 0.306223587 -0.149871767 -0.100809239
TCGA-44-7659-01A 0.458771629 0.351511214 0.464214462 0.305655157 0.21158099
0.501254815 0.324798699 0.470080371 0.282507978 0.474863823
0.441941196 0.459966039 0.340977413 0.337229248 0.446091912
0.159762648 0.533293035 0.520130223 0.429938592 0.273264911
0.109868569 0.112795944 0.313059145 0.53685637 0.40186952 0.497882007
0.303279646 0.652359686 0.440890256 0.340619351 0.249305456
0.313208439 0.432214764 0.627858142 0.37436016 0.520368123
0.459439465 0.423233596 0.324832106 0.34057294 0.332573004
0.305596049 0.308842696 0.173757798 0.390025054 0.263579709 -
0.133374559 0.220646979 -0.156128655 -0.069530425
TCGA-44-7660-01A 0.356540869 0.379560937 0.463126708 0.385668747

0.286174675 0.469484546 0.27712738 0.5113968490.500914822 0.448624965
0.424947909 0.43266487 0.314429065 0.354998178 0.429489377
0.183478961 0.498435006 0.413197419 0.344143004 0.271643326
0.184054883 0.1358011520.294597221 0.537347493 0.426190492
0.596888732 0.536509203 0.729478428 0.556663609 0.393222847
0.144004543 0.312096516 0.406065528 0.617143031 0.440794402
0.582703541 0.433656832 0.417357064 0.28110046 0.336540998 0.29462988
0.227080715 0.275825393 0.1146261670.36033776 0.236496653 -0.010179903
0.178380517 -0.091267418 -0.144103593
TCGA-44-7661-01A 0.5522653 0.470563509 0.476268358 0.434943125
0.271433509 0.570313649 0.459610331 0.467780883 0.466353476
0.534523696 0.44500994 0.472314146 0.330578213 0.3585531150.468307183
0.210842297 0.548956588 0.582083462 0.527462855 0.392402853
0.255745597 0.201800272 0.404073529 0.535615306 0.428394381
0.575088272 0.488787784 0.698722213 0.488021448 0.554988788
0.37797373 0.283755464 0.393527133 0.5789311070.45282181 0.544219946
0.451680422 0.422390563 0.378997603 0.509644423 0.314902592
0.353555616 0.399835645 0.1198344520.339349854 0.394550199 -
0.085808589 0.37143479 -0.185540473 -0.030313175
TCGA-44-7662-01A 0.497883573 0.424593018 0.489941661 0.418217905
0.281506994 0.538679867 0.394699012 0.483318085 0.467748336
0.505376381 0.473013533 0.440465917 0.329155552 0.338390527
0.450767018 0.21125446 0.521821983 0.5496113740.4970408 0.38214795
0.209131617 0.152847526 0.371443238 0.550274128 0.413067661
0.575612983 0.491865158 0.688191239 0.490040931 0.58130475
0.310554529 0.279473177 0.37983213 0.593301685 0.449498945
0.530371547 0.42883096 0.422744683 0.357704234 0.469631495
0.284912738 0.328448861 0.345059039 0.11358872 0.342334579
0.349136005 -0.071992267 0.296604404 -0.171443062 -0.093583219
TCGA-44-7667-01A 0.3107493110.332126739 0.44548407 0.41929301 0.246292259
0.418853888 0.239101692 0.520060163 0.560968733 0.388591065
0.359793609 0.434905254 0.264759786 0.317301083 0.370577839
0.189765945 0.498277057 0.365049151 0.316929953 0.257985942

0.1149024420.044317766 0.25682685 0.555876958 0.426536173 0.589663807
0.6010843 0.748814076 0.612961971 0.367765506 0.138751367
0.250608339 0.384946271 0.6311122640.395412605 0.513991247
0.367137966 0.420188159 0.244798981 0.31619541 0.28284697 0.199836636
0.242241093 0.106806956 0.36214631 0.197219754 0.019134522
0.163759504 -0.165592601 0.048343233
TCGA-44-7669-01A 0.427879445 0.416465188 0.495919751 0.440179438
0.2561411390.505215656 0.317934559 0.495507977 0.55208661 0.47863856
0.37407925 0.45788468 0.326992472 0.336393994 0.437500859 0.162882472
0.565025043 0.50140321 0.434281962 0.276042737 0.099132596
0.177709078 0.335261445 0.577952266 0.450002692 0.630301034
0.585830719 0.738908128 0.537642014 0.398971856 0.236537129
0.271014143 0.407496246 0.6251141560.405047815 0.570081231
0.401300735 0.445242236 0.330863904 0.386897935 0.309906515
0.258427099 0.325279338 0.133373768 0.355149789 0.298104067 -
0.007543605 0.234720258 -0.163244678 0.033028554
TCGA-44-7670-01A 0.473465055 0.405075245 0.480617089 0.418635719
0.295185782 0.510662853 0.304624176 0.487441808 0.498162213
0.472476658 0.386086912 0.445074824 0.342095826 0.359738521
0.452194065 0.17043193 0.526590562 0.427338047 0.359864899
0.314677505 0.189614663 0.176830262 0.332704796 0.555864033
0.41698569 0.594658478 0.515785662 0.707725162 0.534434875 0.40707288
0.216292147 0.307463295 0.390282422 0.597144819 0.425667775
0.551859457 0.447774461 0.462937944 0.280517192 0.420357846
0.327763003 0.288725995 0.323038726 0.162307246 0.389782982
0.223749502 0.006375424 0.216963346 -0.124630405 -0.038601778
TCGA-44-7671-01A 0.423022793 0.404422422 0.469732891 0.340494112
0.313920883 0.540447309 0.244358759 0.453616015 0.307796534
0.45960936 0.42254672 0.512267695 0.387082682 0.389075196 0.484801771
0.217023838 0.559606751 0.393237548 0.33457225 0.327489071
0.147246321 0.167791033 0.322622676 0.532936207 0.393010425
0.5360011930.319605312 0.657780519 0.464259669 0.452604522 0.18775722
0.354780362 0.458718602 0.629991414 0.425613286 0.596637607

0.464458541 0.414241983 0.354399186 0.433093355 0.346948792
0.357642961 0.295504431 0.200889644 0.400214724 0.197834947 -
0.1118785420.222769723 -0.138521882 0.004157944
TCGA-44-7672-01A 0.4941147640.422246152 0.5121261120.367304124 0.246058447
0.516648213 0.426462519 0.469698659 0.404290091 0.510012269
0.423180177 0.463226316 0.35211867 0.368696688 0.471041743
0.208815009 0.522225213 0.551093094 0.52165084 0.348760433
0.221046555 0.155841458 0.410020823 0.541979413 0.420159466
0.558449984 0.422082849 0.671260689 0.447140357 0.533886998
0.348957517 0.300264138 0.40400559 0.599415767 0.433564885
0.555535361 0.435737956 0.41875649 0.337404645 0.462617874
0.314808875 0.376609196 0.389367199 0.145160083 0.377768493
0.420007082 -0.089276662 0.340283326 -0.16488704 -0.028961377
TCGA-44-8117-01A 0.428107151 0.402705905 0.467542951 0.382567061
0.214749151 0.541843541 0.327744669 0.4511754280.367650365
0.473959463 0.436405004 0.462970589 0.336933434 0.32176002
0.450327187 0.2171188350.564050613 0.364207076 0.346450827 0.352363
0.1632511320.145698967 0.330932571 0.518687405 0.414886686
0.522683826 0.382042726 0.658422752 0.441671959 0.462071651
0.234709004 0.290065937 0.398064436 0.599361558 0.427759443
0.520061634 0.4004211180.402180866 0.3551291130.405593855 0.308039488
0.321764326 0.299313988 0.148665691 0.359486535 0.266372181 -
0.078325013 0.225530373 -0.186651486 -0.146020127
TCGA-44-8119-01A 0.505532341 0.479214147 0.492423697 0.383874747
0.2978470110.5821188130.363135405 0.473471666 0.449658396 0.505747248
0.449437509 0.444702064 0.3545622110.378858524 0.485429456 0.21849781
0.5623665 0.393974092 0.398054922 0.317330928 0.197785296
0.193596246 0.349187715 0.557857908 0.436977996 0.58024499
0.465074913 0.6911945450.451956276 0.574473064 0.2811357 0.271315574
0.383229907 0.609315914 0.427943255 0.5241110110.420327653
0.4545411840.384659395 0.472378505 0.289222182 0.333732644 0.36513017
0.152444677 0.363829729 0.319574394 -0.037831408 0.308424698 -
0.171578316 -0.040879493

TCGA-44-8120-01A 0.452531424 0.398847942 0.499332498 0.336389286
0.288647875 0.554281599 0.3153968110.435935824 0.292953977
0.480305521 0.435385009 0.477744656 0.344400054 0.343476677
0.468187555 0.233050184 0.530027769 0.394516059 0.350728576
0.367777536 0.176954518 0.193024317 0.340347839 0.507404031
0.409677632 0.517495662 0.3107118150.643597945 0.4043358110.503675611
0.24288922 0.300219372 0.414655546 0.609534587 0.40867623 0.528296906
0.431664132 0.425772592 0.381382155 0.438355389 0.313927698
0.350870178 0.307959366 0.169967736 0.383796774 0.268818263 -
0.108100378 0.26613701 -0.175987243 -0.073156308
TCGA-44-A479-01A 0.4442912110.385145684 0.452198522 0.361530955
0.231286394 0.522689801 0.421778803 0.44708688 0.401012245
0.498684258 0.378450192 0.438736155 0.315300094 0.336890429
0.4811542140.167001305 0.575070592 0.550907816 0.524897146
0.295332688 0.158191292 0.123332647 0.392521843 0.55181284
0.440277001 0.563681111 0.417049569 0.685067802 0.422723036
0.473986506 0.296501034 0.2928117020.402891005 0.59775874 0.39264169
0.538224714 0.40625017 0.415491328 0.324521955 0.425938444
0.303495373 0.335920827 0.361883834 0.154554349 0.364332054
0.424044022 -0.074278555 0.29860765 -0.18546314 0.032456658
TCGA-44-A47A-01A 0.407550044 0.367043281 0.465994805 0.351038115
0.2578271170.442893807 0.388090957 0.48388065 0.39163635 0.489525683
0.375955333 0.478498743 0.314450917 0.346875936 0.437498653
0.1749118370.512059962 0.572962845 0.476121438 0.300636843
0.130907702 0.162190624 0.353260893 0.556019703 0.419118455
0.553245633 0.402722303 0.676585975 0.504244958 0.371642895
0.243546982 0.315132444 0.4127849 0.616120007 0.406274557
0.557890131 0.448761344 0.415497206 0.26032716 0.356009466
0.322524814 0.3121271140.35962242 0.1787118150.386158531 0.334118849-
0.105154026 0.240658886 -0.160587145 -0.091851548
TCGA-44-A47B-01A 0.460750454 0.4067003110.510745003 0.367300618
0.213790473 0.555754962 0.350534946 0.457630567 0.374801297
0.461389924 0.369976674 0.446644398 0.331617737 0.3471110640.44665234

0.193674183 0.540427366 0.467794163 0.396014794 0.313820554
0.159194242 0.1546311150.320905275 0.5118794660.435803108 0.526649858
0.375637686 0.6611864950.407815938 0.433556888 0.267924922
0.261697579 0.37497393 0.585302885 0.402147943 0.51991666 0.437765336
0.414909673 0.344724639 0.415050855 0.332429163 0.319510865
0.329217409 0.154377045 0.378817309 0.294097143 -0.111691548
0.221866718 -0.146922126 -0.061882246
TCGA-44-A47G-01A 0.548789186 0.452977968 0.445064574 0.359449411
0.225323849 0.524399424 0.468558093 0.445588968 0.344210561
0.524877198 0.394144335 0.482556315 0.329956273 0.353653126
0.456857989 0.212555924 0.530736779 0.561662056 0.532799052
0.348997338 0.213502664 0.135679897 0.403022123 0.519400864
0.409062835 0.544474243 0.372987875 0.669327803 0.443065365
0.507850753 0.381388934 0.323712306 0.4119613750.594500387
0.440955385 0.582837873 0.464620815 0.430261976 0.341972718
0.437391223 0.325868645 0.373293291 0.406460474 0.155277669
0.383732391 0.431700756 -0.115167534 0.351654242 -0.169389394 -
0.080321901
TCGA-44-A4SS-01A 0.518608546 0.405685894 0.464957755 0.391372857
0.2411668 0.529727281 0.404914891 0.441994989 0.374929431
0.502807361 0.402006585 0.471050874 0.348715923 0.372696373
0.434178315 0.186580035 0.566449467 0.559185296 0.508639033
0.353140634 0.253121859 0.170171549 0.389136026 0.511593143
0.438423467 0.565952077 0.389729708 0.663867531 0.442807026
0.485872919 0.319846533 0.356470722 0.426893247 0.592043669
0.424600827 0.589472874 0.468303409 0.427174951 0.355907367
0.425322838 0.316378347 0.356306538 0.347733798 0.179093196
0.393766786 0.378424336 -0.111293474 0.314554602 -0.181398608 -
0.099316026
TCGA-44-A4SU-01A 0.412774164 0.3978514 0.452834658 0.319006792
0.25565534 0.495896809 0.349949256 0.452460926 0.306225427
0.458979401 0.416337222 0.4391198160.317412496 0.334058356
0.457314414 0.197603108 0.516727285 0.462777215 0.412783559

0.332223318	0.172350388	0.1711394180.32473768 0.517580627	0.40019407
0.51012326 0.324401637	0.647458479	0.438271899	0.450868432
0.236464561	0.26630543 0.403487259	0.581276881	0.450174694
0.510058013	0.426377231	0.4111840140.314148557	0.399185673
0.319205145	0.329159348	0.313143746	0.136240586	0.388802709
0.288511505-0.125878496	0.247097945	-0.134957423	-0.078040764
TCGA-49-4486-01A 0.348076013	0.346438318	0.495835398	0.254004125
0.225194293	0.444824513	0.230151863	0.49510797 0.265126006
0.422145189	0.333802796	0.507995975	0.343861595	0.367940705
0.441308781	0.152973079	0.520993645	0.3888257 0.318144316
0.223775013	0.096746376	0.045133877	0.277883278	0.504275644
0.387060401	0.508414775	0.31831692 0.668955297	0.479980947
0.203838795	0.141715254	0.344552713	0.504431029	0.690050235
0.390754388	0.578101628	0.456452183	0.418770509	0.261209943
0.24445974 0.33143942 0.259828147	0.252375941	0.214755596	0.417518889
0.199357909	-0.108154899	0.13408276 -0.129400722	-0.022909999
TCGA-49-4487-01A 0.487148929	0.434640897	0.514548586	0.397076668
0.275200043	0.529335853	0.400017908	0.4851146650.480434225
0.487842596	0.341517415	0.461452505	0.335301979	0.374777214
0.44585259 0.198945368	0.508713238	0.492193762	0.46303424 0.315408413
0.20863085 0.121092096	0.376398023	0.538337836	0.443509132	0.55883182
0.505916331	0.705192906	0.512632566	0.499847061	0.304151592
0.272417021	0.404397304	0.62322832 0.414181994	0.545710074
0.436086477	0.436916758	0.348373668	0.44091106 0.307769392
0.328831649	0.39534819 0.123563217	0.368991253	0.406282327	-
0.053170207	0.297615539	-0.151503428	-0.041431128
TCGA-49-4488-01A 0.4531011030.400533008	0.462339007	0.301834522
0.197059244	0.4812116210.358834601	0.487581716	0.381906688
0.479839189	0.385219768	0.475690982	0.313418213	0.352970953
0.435561266	0.180729129	0.529914133	0.488843198	0.439787444
0.298240814	0.127885999	0.098033553	0.339865747	0.552947348
0.414779578	0.563143388	0.416124706	0.690050312	0.490118026
0.393824601	0.285315165	0.303177632	0.4239251160.637101111 0.44459701

0.589358791 0.448585093 0.447961013 0.279660838 0.335031199
0.330960276 0.292793566 0.330542862 0.179467501 0.416780668
0.343828809 -0.053428566 0.243278442 -0.148148114 -0.076064408
TCGA-49-4490-01A 0.41207731 0.4110378180.42146178 0.308109023 0.243576629
0.521767187 0.298107259 0.45953613 0.292592156 0.468529474
0.424120435 0.470060412 0.3391565 0.357829084 0.424445271
0.251963325 0.506921959 0.461355147 0.403184831 0.349266851
0.188565677 0.169306687 0.328836187 0.514446995 0.390916719
0.520631478 0.305852751 0.6537241160.482962464 0.479581684
0.225175477 0.289705514 0.409228687 0.605482287 0.459979475
0.514321694 0.434183331 0.409726855 0.3262855 0.406315102 0.30252268
0.356122893 0.313206732 0.1189565280.385758561 0.269532508 -
0.121708323 0.283548343 -0.095710379 -0.097243547
TCGA-49-4494-01A 0.445862774 0.415712177 0.518854234 0.307859898
0.225886787 0.467273812 0.368443098 0.49906081 0.407575591
0.486991536 0.400357546 0.485701925 0.330984471 0.369566833
0.435915027 0.16626163 0.510335544 0.569933718 0.48655469 0.290810642
0.171661881 0.097721837 0.361079757 0.538971054 0.407654517
0.590920968 0.44982439 0.690036184 0.49683471 0.405185232 0.283207791
0.304390089 0.428257474 0.633739481 0.472808988 0.562720494
0.4476119710.427015839 0.26268782 0.398452555 0.313504019 0.334274926
0.329977045 0.161041285 0.396057465 0.309410916 -0.069755761
0.250286905 -0.13220198 -0.106233482
TCGA-49-4501-01A 0.450768255 0.393700722 0.502344238 0.284314522
0.21343478 0.518189754 0.351797944 0.464150764 0.277192008
0.4879117080.378908397 0.493102307 0.341827467 0.357330961 0.46862281
0.224485515 0.554315605 0.513889495 0.455064405 0.338936518
0.170968643 0.131776474 0.360048757 0.533541425 0.410296253
0.54713612 0.314035027 0.6530536110.409313807 0.471920544 0.272561715
0.3202701150.436673782 0.644286776 0.428004838 0.527247706
0.465299766 0.438137051 0.328420767 0.363632767 0.331098399 0.3351
0.348662967 0.197180681 0.423198426 0.334862143 -0.116427129
0.298427811-0.164440994 -0.054183782

TCGA-49-4505-01A 0.533336871 0.43101675 0.450175467 0.27531085 0.223685826 0.525340216 0.415731945 0.460127025 0.277893402 0.508113033 0.398302538 0.483575427 0.32561206 0.350035872 0.474838222 0.217592008 0.525750288 0.543343773 0.504773826 0.3258548 0.190864464 0.11171055 0.394573764 0.528106841 0.4189864110.526057289 0.303840004 0.641058888 0.423870661 0.457462799 0.338954022 0.295034936 0.407194223 0.614305763 0.448812126 0.541967082 0.4544601140.441658544 0.34137714 0.416610739 0.340536205 0.356996162 0.380809149 0.15038822 0.37862522 0.378104514 -0.117742954 0.331622287 -0.154212979 -0.05871824

TCGA-49-4506-01A 0.479826662 0.408139017 0.425328075 0.316091991 0.222573069 0.454714538 0.359616355 0.5115769650.409310523 0.468419663 0.320806693 0.486896404 0.314783454 0.34991577 0.417957744 0.134824756 0.527526237 0.48716074 0.450448435 0.256780303 0.175475809 0.0724110720.348648158 0.57788737 0.409219343 0.584406352 0.452689299 0.715870452 0.550516994 0.390782263 0.281318963 0.3467131170.428573847 0.679324312 0.455082743 0.634875175 0.458900465 0.446639847 0.253590623 0.390173892 0.296838571 0.267846144 0.315887652 0.155850927 0.383603362 0.352407969 -0.069024436 0.239201211-0.205246408 -0.089111402

TCGA-49-4507-01A 0.485680924 0.403231696 0.417315951 0.33505417 0.251890876 0.492265398 0.354409517 0.5022427110.448691979 0.487340346 0.316291641 0.462946326 0.339499271 0.39411914 0.417327002 0.169051531 0.486818771 0.5284511270.477256746 0.289549136 0.220456418 0.051869034 0.367909223 0.540091494 0.430712003 0.564499522 0.481982079 0.701461425 0.526133441 0.424944509 0.278856333 0.330902294 0.401788882 0.636710593 0.436177013 0.589201879 0.438718588 0.433639809 0.280711022 0.390215601 0.292263745 0.320536512 0.330540699 0.131333685 0.367792635 0.371803559 -0.056488075 0.238994895 -0.175387757 -
0.154442782

TCGA-49-4510-01A 0.406853708 0.406170363 0.428227871 0.255299407 0.246002455 0.528741341 0.301402341 0.468562848 0.236185628

0.45698674 0.366272222 0.502190393 0.329565703 0.338028143 0.43050292
0.218709173 0.508801853 0.374083861 0.332856443 0.285162512
0.17653453 0.08304751 0.300026366 0.51055594 0.400958523 0.481456637
0.270367402 0.63693961 0.3997179110.394980356 0.200814171 0.313152864
0.4407115290.663849765 0.394221252 0.520745627 0.452288522
0.423518763 0.325798101 0.372422191 0.324019779 0.337148673
0.309378753 0.149431684 0.379464067 0.224401471 -0.111332996
0.234373528 -0.134314943 -0.046398824
TCGA-49-4512-01A 0.416853595 0.386137657 0.487460006 0.314880425
0.261627959 0.494461982 0.319198927 0.47206328 0.317778218
0.463021977 0.4162611050.458979022 0.316638667 0.329753285
0.431907188 0.237268574 0.49765389 0.477856329 0.412185632
0.344788814 0.181285702 0.187323991 0.332416331 0.524512947
0.397698059 0.5114288910.348134826 0.645496348 0.463331502
0.499429386 0.239623823 0.294873622 0.404195631 0.615824541
0.416192322 0.537665503 0.449226017 0.425928887 0.311854838
0.431619881 0.2961114220.335854126 0.315348356 0.149573657
0.389505384 0.29775469 -0.115761006 0.262640039 -0.16320877 -0.061316742
TCGA-49-4514-01A 0.386899122 0.361061846 0.4118288310.343608606
0.241056793 0.423485926 0.333197694 0.492186424 0.445197647
0.443903274 0.380319748 0.487929913 0.326560918 0.375050078
0.412031969 0.170382684 0.496997332 0.450172402 0.392719947
0.273874647 0.16226403 0.071486941 0.339681028 0.555320394 0.41866339
0.554996642 0.4919216110.705932535 0.562494757 0.328402145
0.208405354 0.362267757 0.470723215 0.659844421 0.430063189
0.599091977 0.4341120090.448142206 0.2469251110.329381525 0.312403965
0.319665873 0.31944863 0.154298129 0.39562579 0.31352842 -0.048363881
0.22995324 -0.153301062 -0.063059642
TCGA-49-6742-01A 0.427650975 0.394777161 0.469037327 0.378269854
0.259883038 0.506087894 0.2815119920.491514425 0.44589783 0.47686659
0.370951028 0.494973267 0.358439988 0.3966951180.457566207
0.177610789 0.540962851 0.527435879 0.415908329 0.274722942
0.150626802 0.073021448 0.331866572 0.535965643 0.397949795

0.541883032 0.4561311340.696456983 0.50959473 0.336018559 0.191970584
0.358547178 0.455029685 0.625252763 0.44164938 0.568298876 0.44862633
0.437880095 0.305577075 0.3141092 0.314990008 0.331345139
0.297121316 0.162417505 0.408501248 0.198422636 -0.042539604
0.211728309-0.124740403 -0.042625393
TCGA-49-6743-01A 0.4643975110.424922561 0.501926759 0.415576929
0.244827333 0.534169573 0.364565166 0.467599964 0.478855525
0.48166214 0.402725974 0.466698671 0.337013287 0.348422831
0.486938592 0.204391724 0.554247419 0.436691217 0.424081058
0.326530042 0.155372041 0.10365326 0.357271389 0.544845949
0.438025559 0.594764536 0.497587889 0.690838042 0.446425207
0.502441276 0.290792487 0.32695761 0.403017836 0.585906197
0.445441651 0.573731831 0.422926667 0.416847519 0.347979384
0.366108595 0.335058856 0.327617277 0.327171503 0.173310506
0.378495763 0.304884801 -0.028865709 0.274794633 -0.152055963 -
0.128912363
TCGA-49-6744-01A 0.499664185 0.4227021110.462268666 0.346571445
0.255888923 0.533934133 0.432055916 0.446617042 0.324717412
0.504930787 0.432756138 0.495454256 0.350764131 0.373016833
0.49850815 0.218135238 0.552514693 0.569181817 0.520007925
0.358601753 0.229207506 0.146837052 0.404347944 0.528270274
0.41341526 0.530005943 0.351332213 0.659246486 0.451377639
0.529032627 0.341540161 0.326233904 0.416094279 0.6111229890.41596233
0.55817111 0.449052089 0.434498212 0.386417325 0.459021895
0.340531378 0.374717394 0.386186982 0.147048712 0.379553415
0.39903937 -0.106478708 0.331330472 -0.142131412 -0.037519868
TCGA-49-6745-01A 0.501958972 0.427034388 0.443101701 0.410048599
0.2584898 0.541745923 0.412545237 0.45669957 0.42626282 0.530078318
0.432866269 0.497088748 0.361735882 0.387395556 0.479574674
0.203020549 0.570158941 0.577471433 0.523714686 0.368233648
0.25139673 0.165999748 0.397151958 0.528718369 0.432587351
0.570641506 0.448071468 0.693167713 0.482336202 0.516942267
0.3501102680.304220376 0.407207063 0.599617947 0.445194973

0.537015327 0.460972837 0.427643528 0.38207135 0.476269736
0.330043799 0.354394021 0.3845007110.1411315810.39006679 0.386976506 -
0.088106912 0.334040288 -0.178615948 -0.077052966
TCGA-49-6761-01A 0.516867265 0.430738709 0.422630464 0.360202689
0.257087937 0.509589864 0.389737944 0.485764475 0.416462845
0.484521937 0.352075665 0.440812696 0.329882134 0.339544132
0.4118266980.210980376 0.533007084 0.513318893 0.483620121
0.313153861 0.194696196 0.136329868 0.368203189 0.572816042
0.432898736 0.544670146 0.42897941 0.702309337 0.53806527 0.441755584
0.300681634 0.256581249 0.362640715 0.602213261 0.42727751
0.529085674 0.446237524 0.43575735 0.303826184 0.427707249
0.290710034 0.313266132 0.355927808 0.10151099 0.351890769
0.365796639 -0.060314662 0.287911447-0.144507629 -0.102313005
TCGA-49-6767-01A 0.449155051 0.381078675 0.44358331 0.410313002
0.226940582 0.516201368 0.360864596 0.494269587 0.488427933
0.488091724 0.36480311 0.455437178 0.309098552 0.326034275
0.4110822450.166842751 0.535447044 0.507245851 0.45273549 0.349095975
0.232081686 0.137687457 0.340204279 0.539059107 0.434538157
0.572252997 0.496352674 0.717008698 0.520536132 0.434057977
0.27372351 0.263420428 0.384298636 0.615931833 0.421654431
0.537361578 0.409482892 0.42946608 0.312149221 0.342096739
0.293650731 0.286422908 0.3367811190.0955751120.35846041 0.353402921 -
0.049298155 0.244197374 -0.195043974 -0.115210744
TCGA-49-AAQV-01A 0.426350514 0.396854575 0.457284854 0.330030315
0.199087456 0.428838047 0.338836707 0.48385376 0.383858151
0.454137145 0.359373435 0.471683205 0.324743566 0.327852018
0.427965074 0.186442322 0.509981848 0.538683225 0.460836802
0.288325204 0.139502124 0.108565543 0.339333778 0.550980765
0.408085289 0.553214854 0.424807256 0.689361974 0.51359608
0.365052991 0.238347273 0.293372922 0.402159318 0.636917896
0.450105028 0.548420467 0.424469854 0.437028257 0.257729519
0.3170119690.308044802 0.326260955 0.31339225 0.173039048 0.414479397
0.312321786 -0.06817403 0.232437876 -0.145085282 -0.089743804

TCGA-49-AAR0-01A 0.447771017 0.38599282 0.4947472 0.316435947
0.265001382 0.470885383 0.355923932 0.491234536 0.382511326
0.466735105 0.420679369 0.480063862 0.335690713 0.389327591
0.409838877 0.232759444 0.471375156 0.462254785 0.400351533
0.3122116960.16630879 0.160593531 0.3352150110.529016574 0.400526948
0.550945052 0.419679222 0.676455381 0.498739266 0.393523666
0.231795825 0.33547468 0.441996391 0.62833108 0.403558492 0.615084717
0.455568755 0.470878421 0.284922898 0.349310439 0.314588982
0.3118346420.318684689 0.164787692 0.37721799 0.314701166-0.044714458
0.234615104 -0.128868684 0.175736759
TCGA-49-AAR2-01A 0.332562169 0.33414426 0.43843397 0.319229356
0.247282753 0.378632399 0.321023284 0.4911638860.399420546
0.419751022 0.291653543 0.448857651 0.243837736 0.302447419
0.333757823 0.188640477 0.429724449 0.534585249 0.427026378
0.249625541 0.091019514 0.016697184 0.286377349 0.494960244
0.38737914 0.491037664 0.42666777 0.646908243 0.496946102 0.302225757
0.152461395 0.303641401 0.414988062 0.622252916 0.377179449
0.555710238 0.4287647110.395535897 0.207072526 0.297324909
0.287277858 0.295773936 0.268967232 0.160299708 0.365657869
0.270083959 -0.042856403 0.166383704 -0.147440197 -0.115908768
TCGA-49-AAR3-01A 0.516192688 0.37645457 0.439672751 0.392885459
0.270497328 0.506510757 0.459008096 0.486325054 0.464113101
0.513957458 0.379508019 0.423795035 0.285642349 0.310050306
0.410152882 0.195484736 0.496454425 0.586195084 0.54256626
0.357625533 0.25626929 0.1196369290.398908813 0.531092688 0.426710006
0.568025237 0.499363124 0.6985391180.52566536 0.521958601 0.352340845
0.2933861150.388413573 0.588959056 0.417291567 0.580791336
0.445733146 0.41912663 0.301710531 0.442744029 0.2821108750.308955454
0.375028216 0.10085695 0.334801213 0.439879707 -0.085309468
0.290724829 -0.180500061 -0.121069256
TCGA-49-AAR4-01A 0.480322973 0.394244852 0.490623783 0.371117088
0.248800648 0.48729594 0.385358739 0.478636981 0.392561293
0.516763571 0.399396462 0.430382264 0.330838563 0.356064576

0.412243097	0.210462238	0.47608419 0.541435637	0.48516445 0.336380291
0.213900072	0.096681948	0.372484274	0.513165732	0.412985933
0.524605664	0.409770594	0.659788528	0.444803875	0.464033277
0.282818436	0.2728061160.375240893	0.58573055 0.412694013	0.52960166
0.44732469 0.426279678	0.300937228	0.40830518 0.298063595	0.335868502
0.358858909	0.126548985	0.358485916	0.396137373	-0.064539236
0.274770257	-0.149728928	-0.130842806
TCGA-49-AAR9-01A	0.429680937	0.405673125	0.530632445	0.436390872
0.268266368	0.552404541	0.297323002	0.48687241 0.479221201
0.471403061	0.416663027	0.448232284	0.319648032	0.336231178
0.436300286	0.1554111230.528879303	0.426164766	0.356634694
0.341314256	0.203225519	0.15332577 0.3057827	0.518752909	0.441169461
0.580095676	0.492228327	0.697999452	0.479288254	0.434860318
0.18741455 0.272214379	0.382285939	0.606404164	0.46247118 0.545569985
0.47323154 0.398427413	0.32199635 0.383064157	0.278132669	0.269974571
0.286034358	0.109159392	0.375292759	0.217145253	-0.016183678
0.225956094	-0.165269968	-0.105092448
TCGA-49-AARE-01A	0.390837869	0.3836149	0.502029873	0.36293614
0.208901455	0.433914194	0.318210928	0.495280187	0.421247684
0.456749196	0.347353292	0.450615248	0.3099118750.332810853
0.410981648	0.202913061	0.488864716	0.463239752	0.400330985
0.29312008 0.169588227	0.058145006	0.29742058 0.557698249	0.405598716
0.581868196	0.452283944	0.690446075	0.522984637	0.417238059
0.187722846	0.295971316	0.378718363	0.601898794	0.429880791
0.568450078	0.43862435 0.412039471	0.244162571	0.276732097
0.300978896	0.287254353	0.299823377	0.141765079	0.373433598
0.270388622	-0.059327946	0.201672386	-0.133924974	-0.156489438
TCGA-49-AARN-01A	0.43458322 0.384128954	0.452638671	0.323914796
0.26814645 0.496143617	0.353217518	0.474930219	0.337660237
0.480304576	0.438072021	0.448486105	0.35608059 0.375384017
0.421223484	0.189582272	0.512094771	0.582918776	0.480021883
0.336605058	0.1683511230.176733026	0.346517892	0.534857686	0.39984746
0.513860152	0.36124112 0.66007531 0.460326308	0.443075723	0.231330946

0.31761994 0.397941856 0.601020213 0.422714494 0.524801421 0.46296644
0.420662808 0.308621027 0.38975407 0.322546697 0.3527118110.326876331
0.146092874 0.393324972 0.292974941 -0.115516791 0.277539275 -
0.126293718 0.145532299
TCGA-49-AARO-01A 0.509833575 0.435227358 0.443397396 0.338516565
0.285707169 0.532663765 0.429856392 0.460875574 0.341650151
0.51149274 0.401790546 0.454991071 0.320856544 0.344542835 0.40821796
0.220656152 0.489944538 0.5229113280.474677178 0.364187137
0.221805739 0.145084702 0.381824402 0.509250334 0.404594002
0.521272497 0.370269001 0.642941851 0.419229688 0.517471128
0.329624432 0.288863347 0.385692935 0.577378714 0.431664005
0.531842559 0.452335185 0.415083497 0.316572406 0.45815108
0.301745905 0.35281154 0.371698195 0.1144785220.356802263 0.382556727
-0.088012469 0.327005764 -0.155649181 -0.097189278
TCGA-49-AARQ-01A 0.352854841 0.339875286 0.4751115250.34890928 0.1842404
0.355783376 0.334972174 0.496981293 0.455205042 0.430521074
0.254832921 0.426097866 0.26234293 0.321063428 0.360019299 0.17133905
0.459524808 0.547761809 0.453573921 0.250669426 0.171455002
0.099961539 0.286688659 0.5112322070.4252911130.573233474 0.503823547
0.667655643 0.473876927 0.296612032 0.163858527 0.282297182
0.412886482 0.612207836 0.418215769 0.544675069 0.427051565
0.435033074 0.226650634 0.246395193 0.285829279 0.213558724
0.284053726 0.147805472 0.38046301 0.305895596 -0.02077942 0.163298405
-0.141850093 -0.101829928
TCGA-49-AARR-01A 0.496290737 0.418389276 0.460272621 0.297632529
0.281929202 0.521583247 0.390129304 0.4135071160.224507701
0.488673255 0.401876291 0.469608279 0.362242181 0.371038399
0.472499371 0.278051547 0.4923893 0.470246627 0.422905108
0.340523908 0.214771245 0.155149453 0.353980578 0.476686574
0.390101484 0.474570654 0.228349682 0.594754071 0.344547152
0.46477172 0.288409686 0.316667209 0.404978757 0.5711081790.359874072
0.528844084 0.452338512 0.416467791 0.3674521130.444769359
0.316708629 0.365924207 0.345615757 0.172297005 0.374529353

0.299213844 -0.092811978 0.254557708 -0.124474481 -0.099279327
TCGA-4B-A93V-01A 0.445921814 0.432357398 0.482280946 0.330952727
0.268748747 0.468361735 0.295725728 0.499520139 0.402881059
0.436150615 0.370730994 0.445607431 0.3118499660.313735775
0.408082227 0.171784702 0.47527959 0.345196426 0.350893173
0.223913603 0.096826249 0.168219779 0.302370163 0.564726641
0.393197521 0.568188356 0.436050729 0.699088729 0.563639681
0.344648346 0.194522069 0.304877559 0.418008623 0.647175635
0.434339142 0.582203686 0.428131291 0.446868765 0.255700563
0.402102712 0.299645885 0.24635328 0.31430068 0.1696311180.365691096
0.273939823 -0.058884162 0.194307461 -0.187370984 -0.137543277
TCGA-50-5044-01A 0.469583405 0.440172931 0.435384232 0.369114957
0.292779982 0.543382064 0.383571242 0.501306353 0.431753512
0.497624952 0.397024318 0.469583602 0.305979397 0.353862016
0.4214931120.208876361 0.497192288 0.537001218 0.459529765 0.35514603
0.183837761 0.165276666 0.352717122 0.547775699 0.405819334
0.550158981 0.453212043 0.69710859 0.507551088 0.539526051
0.254934285 0.28212838 0.38821851 0.623097172 0.452122954 0.565791945
0.487844667 0.441982287 0.3311207 0.471550725 0.305106045
0.343722841 0.355688447 0.121837321 0.364492066 0.332723665 -
0.082916186 0.282710089 -0.186389025 -0.066768035
TCGA-50-5045-01A 0.544834836 0.427125102 0.476518697 0.286045124
0.231907471 0.521819102 0.4597951160.4911183250.331598378 0.522138551
0.413679846 0.49317107 0.302060669 0.351937049 0.442966443
0.237865163 0.506505653 0.551757615 0.522198819 0.333621102
0.208447667 0.081534884 0.426689169 0.528980096 0.407802602
0.552532042 0.378745776 0.674765522 0.447987588 0.543663543
0.369375729 0.3118920290.42844934 0.64297335 0.425906143 0.553749335
0.456359396 0.443281825 0.3093573 0.456997593 0.316609167
0.373051352 0.400722518 0.157164436 0.388184407 0.435672533 -
0.097369059 0.359048842 -0.150590047 -0.073944045
TCGA-50-5049-01A 0.505631329 0.424557271 0.466416168 0.324240793
0.228782543 0.49813923 0.445523588 0.474890124 0.373670864

0.515741997 0.376564184 0.468281575 0.313796497 0.347911568
0.456007724 0.196323977 0.526714368 0.551419005 0.535636219
0.336613217 0.199461099 0.1175910910.415877209 0.534817551
0.425378547 0.556668734 0.412100484 0.681749224 0.452830587
0.536874274 0.36135954 0.298233435 0.4131182720.626655422 0.41406418
0.550093771 0.432198713 0.436906732 0.33463534 0.420630523
0.312032027 0.337593955 0.388316044 0.150587058 0.37928913
0.468477221 -0.089317343 0.330877366 -0.161517221 -0.064880878
TCGA-50-5051-01A 0.413654216 0.405909004 0.439739562 0.350457663
0.292236925 0.497832069 0.256792537 0.478819766 0.413396086
0.446338173 0.398840894 0.48616771 0.343707178 0.37692796 0.42693523
0.204470486 0.498910995 0.375595343 0.32062071 0.280248785
0.151872385 0.17077556 0.308300685 0.540642212 0.400860587
0.563540427 0.430845139 0.687325866 0.509604508 0.388495377
0.156552829 0.3099346110.44045707 0.647351898 0.423727342 0.571944234
0.442529285 0.453092779 0.290320242 0.389324481 0.334160669
0.307961261 0.290842552 0.149474697 0.405399368 0.207130592 -
0.064904344 0.209673956 -0.121488775 -0.035999486
TCGA-50-5055-01A 0.522799932 0.39762857 0.447590202 0.280382077
0.264154546 0.510338861 0.447437281 0.47286655 0.317492258
0.5211951420.394545179 0.477226416 0.316924782 0.355568191
0.445760815 0.216959258 0.503760224 0.555153292 0.525695602
0.339279055 0.20898883 0.093002804 0.41195526 0.520486913 0.417673991
0.530000735 0.357099478 0.669192445 0.443883187 0.528429139
0.350812802 0.309328768 0.403720337 0.6311176840.400173839
0.552383451 0.458037128 0.430484104 0.328159328 0.430907924
0.299389168 0.363282529 0.399435416 0.136841412 0.372647809
0.454647427 -0.104456151 0.358455338 -0.142832703 -0.090841523
TCGA-50-5066-01A 0.508438625 0.431875399 0.469004485 0.27675707
0.160775282 0.461329681 0.414230044 0.513780186 0.404455436
0.50618063 0.308002233 0.481565018 0.254082865 0.330776958
0.418587613 0.174247144 0.5119297930.504648825 0.48882947 0.270387653
0.1694866 0.034104343 0.393189502 0.541023179 0.402133862

0.595587909 0.481801032 0.710706937 0.470768709 0.502841575
0.339593389 0.324029871 0.4416711960.6635437110.439047341 0.573610413
0.436079812 0.45203873 0.29181948 0.367907739 0.302322102 0.310115191
0.369975634 0.146423595 0.372735606 0.425994202 -0.019497068
0.31055181 -0.171319531 -0.10558728
TCGA-50-5068-01A 0.472509361 0.384694818 0.471286529 0.226130115
0.147310152 0.455159644 0.415154246 0.496888172 0.324577114
0.492751305 0.329439227 0.477938544 0.260389769 0.316623717
0.438035015 0.153696951 0.519556255 0.569090538 0.518413148
0.254313505 0.094649621 0.034537073 0.381058032 0.503565019
0.403037929 0.545593921 0.393136783 0.674613337 0.4261154820.40956955
0.30626544 0.303648069 0.451327273 0.6659338 0.4119392890.543758278
0.424474035 0.434364309 0.278438469 0.325644403 0.311809892
0.2911477010.335641787 0.198860143 0.4111726930.410238647 -0.066010537
0.306345873 -0.176653095 -0.063932432
TCGA-50-5072-01A 0.465507405 0.423273048 0.476948685 0.403673873
0.24223135 0.504260413 0.32209732 0.498779265 0.515106062 0.483481518
0.389580539 0.488588552 0.368724438 0.422498416 0.459161227
0.203121547 0.545066913 0.493023365 0.419747129 0.315968893
0.235681012 0.10489323 0.352699767 0.569782818 0.429454406
0.604925446 0.540342003 0.726636309 0.541984566 0.485462014
0.249100981 0.342179207 0.430470581 0.629373226 0.466654371
0.575477461 0.445567295 0.448538219 0.324038348 0.420629484
0.305452801 0.352251979 0.324760983 0.146817192 0.381974801
0.244403467 -0.011272062 0.265181823 -0.144582184 -0.039345588
TCGA-50-5930-01A 0.463918614 0.461254377 0.484813052 0.346470922
0.285601833 0.532533756 0.344322304 0.455694368 0.369394154
0.484107361 0.3982114060.475898852 0.380722965 0.375494006
0.503691682 0.201365806 0.55590347 0.405331805 0.395458685
0.328140359 0.226802664 0.1100441910.360647181 0.564265725
0.417958618 0.549442083 0.381493501 0.664814746 0.439637879
0.52011827 0.274537439 0.327859082 0.406829089 0.624162963
0.453739999 0.543420375 0.425475609 0.428520365 0.367877785

0.419689337 0.332236526 0.341339867 0.335587858 0.158646644
0.385378964 0.320132266 -0.07588811 0.276386574 -0.135626915 -
0.099109691
TCGA-50-5931-01A 0.393687323 0.421850955 0.466900515 0.425358275
0.305026516 0.520150964 0.19761884 0.517952643 0.51848188 0.464584199
0.371778485 0.437416359 0.294616046 0.327644856 0.411144413
0.186673688 0.52085121 0.380861871 0.292622531 0.312702944
0.168507296 0.210859054 0.261906275 0.571810742 0.418907212
0.563678313 0.551927881 0.736092593 0.556522095 0.461441207
0.1141185950.231535385 0.383724406 0.62148688 0.429438485 0.532199166
0.444815565 0.431435455 0.316099915 0.368939983 0.271274238
0.239222278 0.275041401 0.079925395 0.374438401 0.143597175 -
0.012359264 0.179845577 -0.101285111 -0.071625451
TCGA-50-5932-01A 0.352452966 0.349023338 0.448558771 0.368927758
0.270371303 0.503125488 0.243227904 0.465727598 0.348027452
0.449704451 0.420589068 0.470267252 0.337998805 0.320695825
0.469336554 0.168748687 0.575784988 0.436656626 0.347349187
0.304639537 0.109068942 0.149720708 0.294740553 0.522662827
0.407398362 0.506749947 0.359096424 0.65921671 0.4117142290.326669903
0.166523468 0.283155096 0.425896507 0.625469379 0.391838505
0.513662866 0.41798861 0.397618906 0.346574355 0.321218907
0.321014191 0.277665461 0.264564706 0.153961661 0.388372578
0.194765014 -0.118014722 0.194941618 -0.168278217 -0.07056825
TCGA-50-5933-01A 0.513810365 0.457377317 0.4799611240.4112481460.266953323
0.552289189 0.397613002 0.4589481110.442560465 0.514189992
0.446733509 0.468659095 0.327881243 0.339894499 0.496636161
0.230535237 0.588937482 0.4911285490.470292383 0.388078266
0.236162293 0.175278716 0.388701091 0.574918531 0.428210385
0.593297709 0.460718847 0.692416276 0.506271801 0.574621931
0.344516352 0.284336365 0.392681555 0.590441653 0.453056729
0.557155722 0.428068175 0.421771962 0.389130232 0.497528189
0.3119320710.338809512 0.359910638 0.133193571 0.359754542 0.36684734
-0.078305568 0.328327692 -0.202423221 -0.095588778

TCGA-50-5935-01A 0.42864488 0.4068029110.480329338 0.314472841 0.2625532
0.52951715 0.346969902 0.449627158 0.3021131350.485021824 0.468506132
0.464894044 0.360297453 0.363072682 0.483438697 0.204999323
0.549596534 0.50282396 0.447364295 0.3262078 0.193378847 0.176095843
0.365303054 0.518133486 0.41869757 0.52910497 0.32216007 0.647025956
0.404204794 0.422104925 0.266059607 0.3110793220.422442281
0.620410665 0.427124249 0.531238504 0.445396351 0.418483752
0.352720816 0.408796618 0.3291515110.350165023 0.345863662
0.1944432110.398244621 0.313272171 -0.130234871 0.289914721 -
0.144213844 0.009830073
TCGA-50-5936-01A 0.447872355 0.426053416 0.451650425 0.389418308
0.293876265 0.517532783 0.355492051 0.471525089 0.406768276
0.466238191 0.393991563 0.48245492 0.354265962 0.364931795
0.478075597 0.179893735 0.557952372 0.408773002 0.370483321
0.33487397 0.199009005 0.128170761 0.3607241180.533530147 0.416631054
0.562788526 0.424328494 0.69453163 0.499513768 0.49823972 0.27203422
0.335319304 0.429159479 0.620967045 0.4577539 0.585060712
0.449898359 0.408101441 0.354208323 0.396978121 0.327608143
0.34791333 0.337371794 0.168428078 0.378149235 0.299299302 -
0.092048578 0.28838458 -0.198610139 -0.061334668
TCGA-50-5939-01A 0.569967754 0.472375052 0.502393221 0.393479687
0.310839615 0.560447402 0.423529316 0.450314138 0.412114586
0.519719829 0.368842375 0.486860942 0.375218846 0.380540098
0.492591603 0.203272079 0.5548011670.486236946 0.461426861
0.358255309 0.228193534 0.157649031 0.399785984 0.542671869
0.426319565 0.590000695 0.413165966 0.676865428 0.454119371
0.518844949 0.344266667 0.348341642 0.413124655 0.586194856
0.45833695 0.5775911040.472730818 0.4435251140.385303014 0.460584552
0.337787809 0.35280574 0.36925383 0.144487305 0.370770355 0.359320359
-0.069151693 0.322855026 -0.178089866 -0.10754519
TCGA-50-5941-01A 0.493329826 0.398280476 0.4773561150.382360678 0.26045909
0.531448579 0.441086509 0.473981497 0.40760518 0.5110637950.372614568
0.4651181590.323887591 0.356545028 0.469380864 0.1917016110.542435477

0.604122152	0.554199644	0.34853726 0.232206752	0.14594449 0.413659952
0.537350583	0.437701629	0.557822621	0.427648097	0.678666852
0.461286566	0.46286157 0.343262357	0.296476641	0.407957648
0.602135516	0.430012794	0.561506126	0.4311159740.424628595
0.337041919	0.4311962680.315251329	0.329815427	0.382308739
0.137315922	0.389301255	0.432596785	-0.09317822 0.307131619	-
0.169374743	-0.058792657
TCGA-50-5942-01A 0.409671264	0.3726110560.420053427	0.335331046
0.274381261	0.562788155	0.3196611940.441665172	0.267710078
0.465569938	0.470945313	0.472955703	0.343601231	0.338612117
0.497913388	0.204157806	0.552893001	0.454441876	0.385727371
0.332243482	0.185349987	0.164303195	0.319153369	0.491742697
0.384653856	0.468202907	0.270628091	0.625791004	0.376801592
0.432846848	0.231938082	0.303408499	0.437080352	0.596862562
0.371826821	0.493628642	0.442228509	0.399749225	0.401486025
0.401684926	0.337694493	0.348608436	0.329196345	0.202044119
0.378343359	0.271211925-0.114565402	0.257475846	-0.119758503	-
0.034897786
TCGA-50-5944-01A 0.387522783	0.376259587	0.468628382	0.362582971
0.251037773	0.561220519	0.284970283	0.430553949	0.329598162
0.493109135	0.441454373	0.495247383	0.3411729390.338328109
0.495309401	0.243770909	0.577284672	0.458213658	0.390310773
0.348389999	0.163697087	0.179802124	0.333515669	0.514919406
0.403134487	0.530837327	0.334433814	0.644933339	0.379716778
0.502162365	0.224382815	0.318869897	0.42773212 0.595868183
0.402765029	0.509792179	0.419156314	0.399536846	0.40952479
0.415580962	0.3473084110.337015959	0.320192305	0.169308543	0.40150218
0.254343025	-0.091744373	0.267063034	-0.165362467	-0.069931582
TCGA-50-5946-01A 0.351435467	0.37095244 0.451505326	0.459713103
0.256940185	0.497987261	0.223542959	0.50133253 0.542655431
0.440215377	0.44730388 0.436465282	0.298778198	0.326616845
0.442554976	0.1903621190.5291131690.452866912	0.369293704	0.289901188
0.101876238	0.107351727	0.260859181	0.55280113 0.422953956

0.545465704 0.556013246 0.701935036 0.494550325 0.364074344
0.139774496 0.225516216 0.375159703 0.583199883 0.415957032
0.475802618 0.3832113140.40665303 0.307180164 0.383902423 0.291189636
0.225490659 0.258948036 0.128307227 0.377619104 0.16602049
0.000520909 0.152647876 -0.128971604 -0.159540781
TCGA-50-6590-01A 0.49836741 0.424258455 0.455653354 0.41999932 0.286203049
0.536396818 0.4341171390.475570495 0.518503107 0.5112913150.392696526
0.437294492 0.307967042 0.349679562 0.436352456 0.178590749
0.512490474 0.532432051 0.501733884 0.348833844 0.275013935
0.146482674 0.387854227 0.571648603 0.432569136 0.595718896
0.530333128 0.721094018 0.55244812 0.524557159 0.324891004
0.272348014 0.373193829 0.60513975 0.424288275 0.573813948
0.442062662 0.446314013 0.3385203110.457290387 0.299029132
0.297740817 0.359051286 0.096812098 0.340977281 0.413760483 -
0.02166804 0.257383704 -0.168307418 -0.066354276
TCGA-50-6591-01A 0.308313589 0.38734771 0.447266899 0.477666759
0.361299106 0.5131365 0.14632707 0.503893901 0.572951402 0.395090502
0.40181712 0.384682241 0.22664697 0.243176422 0.453731761 0.146335953
0.521984465 0.290809065 0.223204675 0.291297891 0.126598034
0.194459396 0.196249415 0.550299701 0.41902353 0.552988484
0.601395757 0.741798521 0.538785722 0.482290981 0.073218248
0.181431062 0.336328265 0.585677432 0.420744568 0.466271364
0.339269997 0.4115231920.365899401 0.395766964 0.257203839
0.150900608 0.244008013 0.072662634 0.333310853 0.084071743
0.0093443110.161900107 -0.180884454 -0.049253394
TCGA-50-6592-01A 0.5256641180.44998116 0.48383795 0.408522188 0.241124089
0.538324573 0.442609427 0.466289589 0.450442939 0.502889721
0.37178458 0.456857324 0.330676848 0.367626059 0.481422205
0.161886006 0.560033233 0.565146965 0.515776184 0.335690501
0.200755739 0.142630153 0.4116180380.569548014 0.4351138430.607817039
0.482305266 0.703781356 0.480264818 0.497132092 0.346446708
0.351694519 0.40481344 0.58777776 0.454521072 0.576151728 0.472193276
0.451897545 0.345596585 0.38107824 0.307680967 0.348797646

0.367984319 0.142335361 0.382586965 0.398727065 -0.046257517
0.316530892 -0.15039357 -0.10101381
TCGA-50-6593-01A 0.449697411 0.405403132 0.511291259 0.380687298 0.252373935
0.536783699 0.361392604 0.4544822 0.383088285 0.493934618
0.426470123 0.495896604 0.3403905 0.352979853 0.46532674 0.246277785
0.521582887 0.432714807 0.391930992 0.373041596 0.191756673
0.161357893 0.35163922 0.522299305 0.407576474 0.559536255
0.403867411 0.667664681 0.478552331 0.52782608 0.284115659 0.318996071
0.424805774 0.607132681 0.431269804 0.554992895 0.429243127
0.423670643 0.352119828 0.460446735 0.318785056 0.351643133
0.340084547 0.175683395 0.41189571 0.310265303 -0.084580035
0.305941394 -0.160662352 -0.08261611
TCGA-50-6594-01A 0.397108209 0.380660611 0.408688667 0.382499972
0.171635915 0.452063168 0.290298116 0.491262411 0.430151173 0.478248335
0.364174409 0.483760179 0.296635222 0.302995088 0.447392612
0.182905759 0.585857389 0.465064441 0.410195915 0.318733861
0.168412838 0.091085936 0.320813896 0.574899848 0.413111582
0.564456613 0.479381689 0.69194265 0.511440268 0.426121391 0.215692208
0.293343838 0.418973203 0.647216978 0.463008569 0.539843193
0.422479623 0.414581458 0.2833652 0.390948463 0.29542247 0.287385193
0.30301396 0.134055607 0.404786967 0.273887928 -0.052581616
0.231919423 -0.185908338 -0.086443559
TCGA-50-6595-01A 0.529211734 0.456642425 0.458324576 0.404084537
0.307040595 0.574686449 0.421932301 0.48936593 0.479553592
0.519839266 0.454774317 0.440710695 0.331534675 0.348438444
0.456316101 0.209854222 0.537410151 0.638595685 0.557292541
0.39245366 0.269875308 0.22336504 0.400267712 0.563965713 0.42115114
0.585005733 0.501814847 0.715092555 0.523630981 0.607081625
0.345918947 0.27767582 0.370654483 0.593819786 0.457959356
0.536878312 0.466165441 0.429458675 0.388447171 0.496043491
0.297452187 0.349540125 0.362542412 0.090738202 0.339153671
0.372955541 -0.067076797 0.345624464 -0.172145308 -0.06333049
TCGA-50-6597-01A 0.370368705 0.366045437 0.421230005 0.262102761

0.179730076 0.435791089 0.345499305 0.45613132 0.292748446
0.461375993 0.327896808 0.474291841 0.3178117540.354506671
0.393548082 0.213772666 0.49425135 0.558151419 0.465372435
0.279369563 0.205823194 0.061716998 0.338127862 0.506845135
0.40370482 0.512493702 0.324681233 0.63519218 0.443215717 0.337909187
0.247790499 0.321256098 0.440920664 0.64469361 0.4111273620.527522389
0.447080694 0.417277722 0.248778191 0.274644546 0.299323523
0.313671733 0.34380674 0.193380383 0.41662743 0.343087861 -0.076959333
0.232969995 -0.141123496 -0.080899607
TCGA-50-6673-01A 0.481043283 0.4161196120.492641584 0.367912934
0.269433025 0.540917931 0.34722405 0.42759201 0.35497888 0.499823026
0.431957139 0.493030982 0.368052512 0.379391246 0.490333548
0.208396954 0.578362385 0.454475385 0.403456764 0.334946532
0.220018037 0.137249707 0.365479864 0.514599544 0.40955908
0.547230659 0.357136107 0.654738578 0.444881266 0.487620395
0.284697419 0.327029315 0.420304279 0.589580086 0.438589504
0.530490323 0.434166886 0.408908629 0.351763326 0.455230252
0.323092417 0.406615443 0.352038474 0.18430919 0.3951738110.284164699
-0.064376209 0.31073182 -0.123189444 0.008644811
TCGA-50-7109-01A 0.401644481 0.3806381130.461645963 0.3586110130.304454791
0.525056535 0.3451174270.480134241 0.430725969 0.450782597
0.403056474 0.4573011130.293694202 0.326279962 0.466765242
0.171573015 0.561561634 0.462862046 0.416950564 0.2721067
0.143382957 0.16805045 0.337127573 0.551807303 0.427261604
0.550609513 0.451080758 0.69382837 0.483201543 0.422191076
0.227373269 0.262972963 0.399832286 0.628996829 0.406910951
0.5300113340.401236393 0.426406382 0.319892453 0.431232234
0.328316449 0.277300869 0.318690746 0.150806986 0.369897664
0.316888904 -0.04141925 0.231825277 -0.196026597 -0.091428072
TCGA-50-8457-01A 0.469439392 0.390992412 0.475722486 0.331704271
0.27932425 0.533644905 0.375820016 0.4422112210.266052641 0.499293959
0.45073669 0.479777161 0.351280967 0.339663742 0.479476573
0.233173674 0.520283309 0.4811499850.440897695 0.34320845 0.17974338

0.171052835 0.373377621 0.5117884610.4074411580.490159079 0.27451515
0.62633565 0.414322525 0.437257325 0.28533262 0.302015846 0.403329325
0.589702373 0.371523305 0.512442223 0.46541227 0.403734413
0.367255422 0.386930513 0.333008981 0.334306735 0.36116518
0.158752251 0.368414822 0.362076697 -0.137311364 0.290310058 -
0.148991799 -0.067531596

TCGA-50-8459-01A 0.4777211640.45689606 0.441341463 0.345069881 0.343477357
0.569508703 0.394533006 0.43186212 0.272264065 0.50656056 0.506716403
0.464431883 0.355786444 0.366434641 0.464626649 0.263522417
0.510978194 0.486198172 0.450878312 0.405010196 0.251579887
0.218215264 0.402954067 0.485399066 0.398850814 0.493123152
0.267631786 0.6117025130.351338249 0.638488087 0.32437138 0.30613569
0.374727366 0.574202084 0.42059201 0.532324686 0.466644942
0.392054922 0.390765874 0.519700057 0.316258322 0.391462149
0.385714649 0.136846652 0.349690189 0.365935274 -0.139213635
0.372265169 -0.141687142 -0.054825832

TCGA-50-8460-01A 0.413280769 0.3883711320.443378276 0.313097036
0.302451496 0.473683655 0.396641708 0.463940523 0.301230198
0.500705718 0.397066522 0.5052117490.325799253 0.357913728
0.433792234 0.221642971 0.510697434 0.454325431 0.418621287
0.338058683 0.19790478 0.140616823 0.368176016 0.501879497 0.39425616
0.512966739 0.329208206 0.645513997 0.441588266 0.422686296
0.253074555 0.322175761 0.438895861 0.62580185 0.404569808 0.55519478
0.4561291150.41845236 0.320766007 0.393636015 0.33263328 0.337828381
0.356088538 0.182688251 0.395385156 0.349840156 -0.138505549
0.283289455 -0.133833245 -0.084348716

TCGA-53-7624-01A 0.436575337 0.416276702 0.477736593 0.446756905
0.202926069 0.518096498 0.340193489 0.496965658 0.537128686
0.475673963 0.287623856 0.436943139 0.340884832 0.361373102
0.457543524 0.17389229 0.545274024 0.597597954 0.490708964
0.276701485 0.213847619 0.039739128 0.336602306 0.557710486
0.431602824 0.601856853 0.557812125 0.712742 0.512362136
0.3896811220.228580321 0.267943174 0.366387976 0.600279979

0.425678647 0.547178777 0.419247387 0.428086389 0.30706915
0.334427572 0.293829189 0.230713072 0.285240433 0.123829345
0.3493021160.269686123 0.003990514 0.164928889 -0.149866134 -0.098492785
TCGA-53-7626-01A 0.562612923 0.417395774 0.449979685 0.372178877
0.255626404 0.5729511260.458539103 0.423422866 0.341762894
0.5311967420.445045228 0.477623618 0.367590915 0.370847322
0.497347977 0.235161545 0.539905767 0.53018635 0.50076322 0.350572521
0.22051731 0.214464132 0.409944258 0.509049043 0.418276031
0.506950378 0.345754612 0.643356953 0.41854952 0.4423110080.378587627
0.299017382 0.402609837 0.579313564 0.364714656 0.512845843
0.456676139 0.42523896 0.390171234 0.402982797 0.334410653
0.337723592 0.389137324 0.165672981 0.371215335 0.417976624 -0.123149551 0.331888038 -0.141059412 -0.070887701
TCGA-53-7813-01A 0.410755714 0.37592474 0.483568666 0.334890436
0.233873859 0.512952285 0.348295525 0.474743109 0.434823305
0.438213774 0.3450651130.426953927 0.298706882 0.342143909
0.464556775 0.109523318 0.5622811540.5174211410.457849837 0.226414072
0.099642472 0.141988307 0.339187704 0.563208137 0.426995434
0.571723652 0.457041533 0.707043209 0.446734258 0.357299092
0.213150081 0.249586073 0.410549098 0.63312886 0.4114946130.500226525
0.39277263 0.42605193 0.300284446 0.373837748 0.3119782710.27877833
0.30468376 0.1320587110.379016571 0.290698792 -0.054150545 0.218276287
-0.175461877 -0.028630218
TCGA-53-A4EZ-01A 0.3117355470.348929991 0.455140215 0.374719512
0.275239621 0.42105986 0.239974388 0.498012814 0.477097484
0.383818712 0.326810022 0.461427671 0.323149445 0.343536423
0.40597971 0.176704451 0.514308803 0.4115207080.329378561 0.244360031
0.094460804 0.068986939 0.280710528 0.53293968 0.408527006
0.574664093 0.5207314110.700501065 0.540389835 0.249552269
0.135215622 0.310195523 0.443283705 0.646418106 0.410017224
0.56059589 0.399899637 0.422060042 0.217864304 0.280753973
0.309008897 0.227639206 0.286930756 0.174210157 0.399059563

0.206350312	-0.030681728	0.124344101	-0.171048684	-0.101332221
TCGA-55-1592-01A 0.436885154	0.400088212	0.520937108	0.336228245
0.233422652	0.494845506	0.354683413	0.459816629	0.379670996
0.488436486	0.384563801	0.48645174 0.338535583	0.351457905
0.481901993	0.187698122	0.559505486	0.461550242	0.396820045
0.303564833	0.153924745	0.11802095 0.340822587	0.524316838	0.40292048
0.567300512	0.406959164	0.665329722	0.420718036	0.39701944
0.249697337	0.315843422	0.455181272	0.612723894	0.440443376
0.521913301	0.42666814 0.437844941	0.315544153	0.349167886
0.330971851	0.327731365	0.302516727	0.197141914	0.412772567
0.269367943	-0.078167991	0.241030162	-0.140539087	-0.107992519
TCGA-55-1594-01A 0.32754576 0.370444257	0.447826417	0.372170745
0.228762718	0.453487545	0.298532177	0.493822894	0.462903316
0.437405637	0.3662961160.453409278	0.31235483 0.320592391	0.425739312
0.1617113820.578312582	0.360489352	0.335824253	0.26471792 0.112654588
0.092171059	0.318015448	0.567932551	0.438947583	0.587820103
0.499607937	0.705942366	0.479058586	0.376590131	0.190439562
0.285732065	0.404866127	0.623712668	0.414853222	0.543206804
0.378972972	0.423779218	0.265882847	0.349403539	0.297992867
0.301803073	0.281287504	0.167903451	0.385076985	0.278788672	-
0.015175239	0.210070199	-0.148291073	-0.005826408
TCGA-55-1596-01A 0.351249015	0.342322074	0.510796273	0.347242944
0.212433628	0.373187145	0.316331651	0.492701048	0.463239538
0.449444959	0.355398999	0.4755217110.309209218	0.327868951
0.441654654	0.149219833	0.524531482	0.60212909 0.4794408	0.254390301
0.155393	0.087144889	0.322217291	0.574632139	0.395175176
0.595736321	0.515913588	0.700250952	0.506563994	0.319556705
0.185558835	0.315712712	0.437623216	0.646525063	0.440193122
0.532589869	0.387533714	0.431242722	0.220198057	0.334355203
0.303542187	0.274353146	0.2880201190.143637221	0.39482212 0.255236969
0.000605813	0.189479807	-0.140562803	-0.136344226
TCGA-55-5899-01A 0.364663091	0.376242573	0.478931827	0.372441451
0.318982835	0.477782653	0.289521741	0.483078958	0.480703866

0.45538391 0.353173088 0.482630096 0.303871828 0.338004078
0.428296758 0.182766202 0.562067552 0.400275738 0.378190057
0.294367586 0.109583954 0.201387963 0.326161209 0.547878561
0.433018753 0.602900386 0.514276334 0.710236888 0.532837583
0.458377624 0.182063714 0.345398259 0.452570266 0.663361479
0.442979404 0.580851216 0.3776224110.439230449 0.289564862
0.459606316 0.295525675 0.314451661 0.315157035 0.205469769
0.418700029 0.2812071150.004434409 0.262525332 -0.159414546
0.149907093

TCGA-55-6543-01A 0.49191281 0.42245658 0.47941811 0.323020606 0.227627595
0.550577055 0.403252761 0.459409285 0.28705772 0.488234191
0.422296761 0.48124758 0.365308616 0.347634429 0.524868227
0.187472851 0.568219967 0.467940589 0.444400981 0.331669733
0.183474078 0.153541297 0.360700198 0.551207827 0.4078678
0.546983432 0.297099079 0.644786667 0.43464166 0.4503911280.310165614
0.310647942 0.424940268 0.609688752 0.448528544 0.539332978
0.467403742 0.427718155 0.370081545 0.425686585 0.346808056
0.32998194 0.348342604 0.178886436 0.385944264 0.303231085 -
0.11375717 0.290741747 -0.142012069 -0.079007518

TCGA-55-6642-01A 0.431896106 0.453515569 0.460342966 0.388949191
0.280194309 0.541743203 0.360369804 0.430934928 0.393054724
0.492865825 0.459027906 0.459690887 0.345235141 0.337664658
0.498988724 0.223274421 0.567501543 0.469582404 0.447187667
0.358961298 0.220559509 0.204187732 0.370691679 0.569270827
0.436808974 0.560932518 0.38784034 0.668642508 0.425218249
0.572179051 0.272951828 0.261763771 0.387615383 0.589746639
0.425182277 0.496022902 0.408229093 0.415743897 0.387283261
0.472008215 0.336976437 0.342328736 0.351494039 0.137624129
0.374342818 0.344956874 -0.095203201 0.293897609 -0.168208971 -
0.054266271

TCGA-55-6712-01A 0.483771318 0.421739609 0.450484731 0.393929584
0.23932622 0.542771408 0.399320504 0.451519692 0.415729252 0.52326459
0.413475688 0.456733024 0.34593983 0.358072396 0.479621455

0.172354416 0.5731120920.544092267 0.5111067230.3574349 0.235347902
0.168083216 0.393210288 0.513948349 0.428305522 0.559275286
0.436097384 0.671794878 0.430333923 0.468758249 0.312845089
0.293661404 0.397228175 0.581997495 0.446054759 0.550206965
0.438962104 0.402921396 0.373172815 0.421314689 0.337232854
0.331010255 0.364097953 0.141399254 0.365704761 0.411679136-
0.082583952 0.31808817 -0.162431342 -0.093719933
TCGA-55-6968-01A 0.350467556 0.366501003 0.416316523 0.446970078
0.274070688 0.491504424 0.322158797 0.501364107 0.551847583
0.450396038 0.314546751 0.42740104 0.283817406 0.31152159 0.410850757
0.156155096 0.530619732 0.446823182 0.418262182 0.276749315
0.136228227 0.242516548 0.316009723 0.557286369 0.418741244
0.604980843 0.5809686110.726821949 0.4954344 0.401243988 0.188490631
0.253509202 0.37164935 0.626338551 0.406422171 0.5799110920.393028465
0.4508801110.271345009 0.336017274 0.29823021 0.212671327 0.302340832
0.1432011620.377244862 0.33579967 0.0710411120.218877729 -0.145686526
0.077427633
TCGA-55-6970-01A 0.484520343 0.415607968 0.441600329 0.389781474
0.258829201 0.524715689 0.366207256 0.443983303 0.406974618
0.474813162 0.354684132 0.509959062 0.349858577 0.350789919
0.489420322 0.16309548 0.579612604 0.504565061 0.463194599 0.30343583
0.150352649 0.124618535 0.372663286 0.556475505 0.418918239
0.552812479 0.418592771 0.687995598 0.471281076 0.387691845
0.29428065 0.340037526 0.452225507 0.646165279 0.414942172
0.5890340110.442020562 0.434799884 0.344036526 0.379567266
0.342344592 0.329713826 0.352388141 0.157906646 0.382868481
0.330965334 -0.084147265 0.26656375 -0.148680246 -0.052820285
TCGA-55-6971-01A 0.5391141090.431940837 0.463523973 0.368003633 0.26607505
0.536603944 0.457128242 0.445225674 0.388966404 0.511229651
0.372247078 0.462258619 0.33917117 0.374100903 0.471884988
0.189123726 0.548712234 0.539376766 0.533452197 0.329706418
0.195642565 0.132476781 0.413674898 0.551315832 0.433685705
0.558747992 0.403481569 0.676104821 0.43591097 0.48601563 0.376887606

0.291570732 0.385970806 0.577788397 0.413949588 0.526526561
0.448703071 0.435949555 0.361890518 0.441313865 0.319732604
0.352539807 0.4110368330.138566058 0.357192425 0.438384141 -
0.085040595 0.340361758 -0.172118587 0.003374317
TCGA-55-6972-01A 0.294058152 0.273316562 0.414346017 0.274444534
0.241796805 0.424879563 0.169394633 0.492393154 0.326187634
0.387837242 0.347582681 0.459565309 0.287377296 0.317931035
0.388710528 0.121320379 0.528852485 0.341026377 0.270734438
0.196916581 0.029454066 0.012773963 0.209551379 0.529978257
0.393834138 0.477131204 0.384769282 0.689071423 0.486564914
0.13413641 0.069756225 0.289840983 0.467371087 0.673032736
0.352920366 0.5548391130.398418386 0.397643203 0.232096767
0.141839671 0.316268999 0.180676532 0.210247759 0.157756041
0.376603898 0.116307363-0.078397857 0.075333055 -0.172810237 -
0.04872637
TCGA-55-6975-01A 0.455958154 0.4231112210.471710449 0.414079891
0.309312317 0.559296524 0.308359691 0.4711233820.486185808 0.4848262
0.443125175 0.470819732 0.365772175 0.369677642 0.483154684
0.228959932 0.545927837 0.474269773 0.413655514 0.360004536
0.236510248 0.160307288 0.34340592 0.556234215 0.425612284
0.594829841 0.48973167 0.7124311050.505422085 0.561969996 0.241033641
0.306897613 0.399225502 0.61271741 0.45078404 0.563269962 0.432578023
0.4161143510.36334627 0.423171461 0.305244461 0.344486751 0.320518931
0.121290397 0.356135843 0.263621313 -0.048901633 0.265107277 -
0.165991122-0.102882181
TCGA-55-6978-01A 0.561427461 0.43807519 0.469590864 0.432790456
0.250567231 0.571084694 0.45943365 0.460987788 0.491265687
0.534715863 0.413010782 0.444088563 0.308220538 0.324429952
0.481631745 0.1877113860.540945619 0.615828712 0.573860497
0.375180781 0.248441009 0.192603237 0.419347281 0.550044389
0.43642864 0.590299378 0.501702577 0.71265207 0.488550948 0.583113244
0.383772446 0.26961012 0.374652028 0.5800011770.4234096 0.526540117
0.430348786 0.428600243 0.395920354 0.470573277 0.297211738

0.342193758 0.398609397 0.109298102 0.362421941 0.45896959 -0.072609926 0.344259455 -0.187139515 -0.100133501

TCGA-55-6979-01A 0.51206666 0.401175 0.477489929 0.381833222 0.247101863 0.51331109 0.461202054 0.471879926 0.422613999 0.517007864 0.408305044 0.456429493 0.329467193 0.349873415 0.472907511 0.186405945 0.527781494 0.608046809 0.570064354 0.3370981 0.214773694 0.164942518 0.422496488 0.53995711 0.426390815 0.570358925 0.453688955 0.679356089 0.43808481 0.5066311340.368826844 0.303754943 0.401745844 0.602717035 0.420675706 0.539764049 0.434439482 0.440469324 0.348104396 0.466744335 0.318775322 0.350999368 0.40000646 0.132854368 0.370577271 0.452393127 -0.071729568 0.345621625 -0.162054215 -0.075246646

TCGA-55-6980-01A 0.540827124 0.456479601 0.439043353 0.312263425 0.277077234 0.572088038 0.40613417 0.431025583 0.271989218 0.5317113890.452214169 0.485496478 0.344868521 0.35860358 0.477261556 0.264789001 0.549800573 0.526812165 0.484162475 0.368914968 0.236629815 0.20670098 0.392154387 0.520777971 0.4098718110.520005809 0.274702059 0.641319373 0.413515943 0.54389505 0.333138282 0.319635539 0.412946189 0.59663918 0.454337929 0.54459286 0.468799076 0.445590686 0.37242934 0.473590345 0.331852929 0.392827623 0.385844204 0.158502477 0.40306429 0.371038028 -0.119882963 0.341324092 -0.127052389 -0.079977411

TCGA-55-6981-01A 0.435873371 0.407231423 0.529144445 0.365505273 0.287676413 0.530481417 0.329023658 0.461516221 0.381258476 0.493715109 0.439995587 0.475533395 0.347030058 0.344570454 0.485349169 0.207258091 0.554607536 0.447774248 0.405101913 0.363759305 0.196700141 0.180282648 0.349057516 0.53914256 0.418563013 0.5811649340.410837088 0.667875797 0.47295468 0.475862717 0.263892923 0.300903733 0.421203507 0.598235016 0.475186885 0.525041221 0.431994816 0.427055252 0.354492193 0.4450709 0.33573608 0.345282739 0.339251877 0.160886733 0.40309836 0.274042802 -0.067097168 0.286605099 -0.154486918 -0.114203014

TCGA-55-6982-01A 0.472904549 0.432545469 0.522981884 0.367812331

0.233800004 0.52341841 0.389209334 0.445680216 0.393714313 0.51433355
0.392828304 0.486670688 0.346038453 0.363763334 0.478174122
0.204238813 0.564204962 0.5511344640.4911273290.356018199 0.206277445
0.164480329 0.392489809 0.544263142 0.416728492 0.591739072
0.417228937 0.674494638 0.465276507 0.541831658 0.312374987
0.327077654 0.4223881170.605953812 0.461644636 0.561874883 0.45188419
0.434579293 0.349192946 0.4465112670.308493684 0.359993087
0.355094363 0.172494499 0.417055275 0.351655985 -0.09946753
0.312471779 -0.170779263 -0.060574723
TCGA-55-6983-01A 0.471608147 0.400753584 0.463771925 0.309092295
0.263637756 0.545256969 0.366124574 0.441777521 0.318896865
0.486648484 0.412005288 0.466344841 0.376015778 0.400272864
0.472182726 0.218394493 0.520783588 0.419049974 0.404145239
0.314654123 0.178526488 0.164165692 0.3611544770.545968371
0.402387015 0.541584414 0.330061263 0.663555636 0.439638942
0.4812115050.259288824 0.313538374 0.417662344 0.615983425
0.406647835 0.543206002 0.450769525 0.432319697 0.337807965
0.405969217 0.324679138 0.339014602 0.346763833 0.164313785
0.402489848 0.337125224 -0.116017716 0.305917858 -0.139177936
0.134609663
TCGA-55-6984-01A 0.51128201 0.421354448 0.518433486 0.370630785
0.275920797 0.525900648 0.342495937 0.452125454 0.375474933
0.489125763 0.407645912 0.462435956 0.375447154 0.392652362
0.46552022 0.168891492 0.555245975 0.462089984 0.389704714
0.313524912 0.207504801 0.140648878 0.360071032 0.554963025
0.401038016 0.552401483 0.379925217 0.675789242 0.488729727
0.405367778 0.254913047 0.31763934 0.41920887 0.60278729 0.45448352
0.572398929 0.462327277 0.440834789 0.338763904 0.371893943
0.316921565 0.314591994 0.338986143 0.147983007 0.393602507
0.269681113-0.104004705 0.28529543 -0.158499137 -0.043395541
TCGA-55-6985-01A 0.563131548 0.426478894 0.464235747 0.364986779
0.265175772 0.543947003 0.422312745 0.434959579 0.387933878
0.528263148 0.418678875 0.444701476 0.370020206 0.383328392

0.491037758 0.2081164370.519278027 0.52555806 0.5031128740.357684107
0.227266491 0.193724453 0.383872607 0.531700187 0.415541451
0.542099223 0.395590744 0.66279999 0.435519867 0.519951557
0.347310515 0.288673906 0.397364448 0.585729851 0.43715836
0.527880221 0.453409881 0.440335281 0.36837235 0.4411641170.326780186
0.34406175 0.381478673 0.158626598 0.3906111770.375673228 -0.067838313
0.310279054 -0.139299797 -0.068321521
TCGA-55-6986-01A 0.444576979 0.405008122 0.440176775 0.32208101
0.245126091 0.541248594 0.334918997 0.47024478 0.322943301
0.477859871 0.389860819 0.476055141 0.338879929 0.345068814
0.476058077 0.165399912 0.567424913 0.426771554 0.38817681
0.3111277940.157922364 0.137713277 0.335844409 0.57092281 0.40680713
0.547406078 0.33961919 0.693335902 0.539241273 0.396001067 0.25730345
0.288506129 0.415910626 0.625520473 0.427605005 0.549974337
0.457187042 0.430478044 0.344468279 0.34426333 0.324652335
0.325982192 0.336151729 0.138123189 0.393619196 0.297787787 -
0.143380779 0.271260204 -0.165568238 -0.062933973
TCGA-55-6987-01A 0.507712179 0.4278116760.465064254 0.353791838
0.2630011470.51854934 0.451454038 0.468622524 0.416077836 0.507914074
0.352181595 0.471090673 0.330653796 0.36395625 0.488377946
0.167389134 0.549474031 0.566798298 0.550742834 0.291759663
0.214902874 0.1170136050.42091886 0.567397701 0.449330905 0.593588592
0.431677801 0.703698059 0.510382298 0.433761225 0.354737247
0.303880172 0.414378498 0.626665425 0.425096128 0.582907514
0.4441199710.440144891 0.330433084 0.391723055 0.332943071
0.314692846 0.397100524 0.155265406 0.378054746 0.462264924 -
0.070319323 0.298007785 -0.181532344 -0.084438636
TCGA-55-7227-01A 0.478542067 0.4112165220.4471079 0.355283139 0.254062675
0.560556688 0.410857731 0.463174018 0.359078775 0.509431864
0.456532492 0.465133657 0.349652002 0.359641072 0.476075579
0.219401445 0.539005923 0.548659542 0.497472983 0.361892443
0.20403486 0.186957605 0.3830113740.525074383 0.40904943 0.535660932
0.37143626 0.672122979 0.44016156 0.5006631120.316426264 0.297937521

0.399706806 0.602544697 0.417606188 0.540542537 0.449522945
0.415184683 0.379441402 0.435988526 0.337486101 0.335744696
0.372316195 0.164583836 0.395626326 0.38355969 -0.110084133
0.318973885 -0.169515257 -0.03757535
TCGA-55-7281-01A 0.542622597 0.4223511440.478607329 0.333500043
0.2544044110.535599868 0.423985798 0.456090959 0.320577951 0.51521749
0.456456984 0.470828379 0.360031843 0.374481808 0.43801544
0.229237886 0.513445384 0.496890594 0.455766695 0.342565839
0.212120518 0.168517255 0.385912393 0.5011718820.400266067
0.5114675710.34060528 0.65390572 0.438332194 0.49324129 0.340780181
0.293750825 0.394273071 0.60232402 0.4119054570.528130024 0.468277592
0.435547162 0.349557109 0.419588303 0.31983534 0.347281045
0.371955575 0.157337328 0.385560763 0.37910299 -0.133586808
0.338271919 -0.12187002 -0.071563314
TCGA-55-7283-01A 0.436013688 0.346551837 0.465085322 0.347622936
0.228999395 0.498773097 0.328200636 0.46526553 0.360620633
0.469418008 0.384338952 0.479817427 0.342341752 0.367379334
0.467763222 0.203557952 0.528616697 0.508977703 0.438033247
0.3161190410.133035106 0.156438428 0.327770613 0.539057426
0.403273576 0.529331994 0.384890531 0.684603168 0.525121065
0.327728 0.249718485 0.287500236 0.418163977 0.614720559
0.378849536 0.5116525250.438783722 0.431552121 0.332080109
0.295344564 0.313912764 0.301893277 0.323365404 0.1822173
0.391098373 0.298949379 -0.089672264 0.237173964 -0.162527323 -
0.081289017
TCGA-55-7570-01A 0.353407682 0.327175296 0.460848186 0.430443274
0.381832493 0.47719886 0.221527127 0.518773636 0.546358019
0.444080927 0.375476263 0.458451979 0.273889855 0.319801818
0.43193217 0.1491101580.529615656 0.284817858 0.262601785 0.264574704
0.1197896610.14974211 0.260229361 0.573993252 0.419653236 0.607259739
0.589235003 0.758972547 0.599836552 0.393486358 0.088616738
0.29828765 0.409332595 0.643327246 0.418779869 0.535415659
0.391360604 0.4207831150.251429617 0.3511297120.286949916 0.213019037

0.262152585 0.124747388 0.376439324 0.183256941 0.028396547
0.177847176 -0.170599323 -0.085185134
TCGA-55-7573-01A 0.442730756 0.371266393 0.470142852 0.330680453
0.26035896 0.537166273 0.382710183 0.427378184 0.271088161
0.489297734 0.430703905 0.469555787 0.34550182 0.339914384
0.473145746 0.19474782 0.550010592 0.526821321 0.454924203
0.322962582 0.176216107 0.164295271 0.351585137 0.507179098
0.409534796 0.496456407 0.282521804 0.624428035 0.352025519
0.43470678 0.264156208 0.302233444 0.405134316 0.572852687
0.388780097 0.510568466 0.437120488 0.4067311120.3704411780.380386784
0.337182666 0.325580377 0.339380536 0.187103058 0.395471886
0.346550781 -0.123399141 0.276224664 -0.140291355 -0.066663349
TCGA-55-7574-01A 0.528258678 0.439450734 0.478933219 0.366039347
0.282858194 0.54792095 0.420935844 0.43899637 0.343178502 0.514429342
0.432644827 0.466144977 0.35339711 0.363173822 0.4911335030.230091326
0.514497706 0.499051559 0.480407052 0.369438397 0.223832511
0.167552529 0.403542261 0.532899595 0.426691491 0.521131157
0.357701433 0.659282301 0.413946933 0.526732206 0.3202110810.30029007
0.385890647 0.579747403 0.385472707 0.538062406 0.457236
0.4338311340.383018567 0.480374657 0.318428454 0.367262786
0.406602542 0.136759351 0.361209789 0.395389773 -0.132966772
0.332207759 -0.161730023 -0.06943299
TCGA-55-7576-01A 0.420775343 0.378990813 0.47653898 0.3944741150.248030939
0.523877608 0.349464741 0.468776886 0.416070326 0.488985256
0.430737306 0.468139256 0.3476713 0.357147273 0.454744062
0.203384474 0.54210942 0.522384361 0.454065433 0.339865752
0.1811688460.139649846 0.364885171 0.53692825 0.422548488 0.547639689
0.428962339 0.678896872 0.484609827 0.434812124 0.256611915
0.281830533 0.389766006 0.580886727 0.41079748 0.538950938
0.414799383 0.400964939 0.352670592 0.401065827 0.321252224
0.3239857 0.3351178220.150868827 0.379893446 0.306627464 -0.081890096
0.263727339 -0.168834561 -0.088287734
TCGA-55-7724-01A 0.471367013 0.442556652 0.476522999 0.365359545

0.244575668 0.542605085 0.378150738 0.414405418 0.414179742
0.507654237 0.408717158 0.48725752 0.358859182 0.374221408 0.5334354
0.176448245 0.597622686 0.51558613 0.479373909 0.342423945
0.283300768 0.171367294 0.404554079 0.525826638 0.425940348
0.579916556 0.420559141 0.688208409 0.353762789 0.474035409
0.328040632 0.312081984 0.437646831 0.602922147 0.409290045
0.505920765 0.438318094 0.413225631 0.4131311460.479559294
0.355172924 0.354528188 0.37375024 0.169158932 0.394363772
0.345722738 -0.04140836 0.325895147 -0.090218552 -0.06410782
TCGA-55-7725-01A 0.474760605 0.368792094 0.410258258 0.312257159
0.181233107 0.512417812 0.384236702 0.440159556 0.329603535
0.486374052 0.364027357 0.470493071 0.294167395 0.310041147
0.485524161 0.1180139370.605092407 0.545173553 0.492106916
0.2711786270.172974709 0.11386155 0.378639874 0.532107525 0.401930652
0.518785597 0.356160912 0.685303479 0.427373912 0.293213681
0.314961496 0.278418894 0.435241571 0.622029499 0.365624957
0.485883899 0.409906322 0.404685518 0.353520778 0.327425983
0.32634539 0.272463958 0.357031964 0.161385594 0.384987157
0.367859539 -0.106482811 0.294162255 -0.151725066 -0.02643599
TCGA-55-7726-01A 0.522305532 0.4368413110.428230026 0.420859316
0.320364465 0.594739789 0.362246216 0.471392939 0.493818624
0.520025991 0.487457075 0.423887829 0.354010264 0.367422793
0.4881469 0.195932317 0.573428161 0.423148024 0.418039279
0.368361447 0.274967435 0.236788413 0.355554496 0.556696436
0.428193661 0.576905409 0.489529763 0.70638614 0.45742154 0.612033615
0.3196187 0.264515832 0.364307617 0.568668455 0.432577603
0.501855595 0.473044673 0.416661827 0.413730549 0.494545611
0.310138925 0.343145665 0.349403426 0.121376998 0.335141301
0.311837513-0.059864009 0.344699433 -0.122812847 -0.062137013
TCGA-55-7727-01A 0.410074273 0.365573175 0.466822331 0.378593677
0.1706151140.471647041 0.357605238 0.451915589 0.442228866
0.488570037 0.35356726 0.465648559 0.324967299 0.331387207
0.500740092 0.1109116850.59699826 0.5382734 0.48492446 0.2696094

0.155872052	0.1119665040.364525951	0.5441112030.420405106	0.559739625
0.469849681	0.698024858	0.3758118490.310208037	0.26832075	0.270720584
0.417887788	0.602215023	0.406413928	0.517277894	0.400545596
0.405469522	0.345628377	0.332329558	0.338176652	0.297294628
0.349263681	0.169261073	0.379921982	0.347752217	-0.030703821
0.271823129	-0.19021785	-0.077119389
TCGA-55-7728-01A	0.489071342	0.386848516	0.491313985	0.328189822
0.210078546	0.488029938	0.422712993	0.456238401	0.286399233
0.479051228	0.37718368	0.45325148	0.336348284	0.383706571	0.409063203
0.193635602	0.471030506	0.572896522	0.49260487	0.319022379
0.190983252	0.126649773	0.413904226	0.462786537	0.402630218
0.489778093	0.281782071	0.599298231	0.380958878	0.339623071
0.321013674	0.324675603	0.401620462	0.5871224	0.390266561
0.547894936	0.485754263	0.4114011140.283840253	0.339308977
0.321280385	0.354543277	0.360172318	0.157984282	0.358358958
0.388556437	-0.156125514	0.298136212	-0.13236205	-0.058626158
TCGA-55-7815-01A	0.434897208	0.410428709	0.465456422	0.353857282
0.228513421	0.518770057	0.386232212	0.396843774	0.396537247
0.45892601	0.332047053	0.444569515	0.33221904	0.333153331	0.535133753
0.1145198660.62940337	0.466877983	0.454532034	0.26175859	0.175568854
0.1478462	0.40251165	0.529666222	0.416087888	0.579997217	0.406176292
0.670213033	0.362197641	0.477279492	0.296776647	0.299414838
0.4054481170.594289765	0.39565722	0.49531066	0.368263078	0.376660088
0.395701536	0.435492536	0.330395666	0.35190842	0.38075791	0.203936514
0.374288888	0.345078695	-0.038759388	0.324531953	-0.183498023
0.048489946
TCGA-55-7816-01A	0.414915743	0.383530537	0.397990486	0.346390456
0.238476868	0.513629592	0.433884489	0.423648101	0.278790038
0.4872211720.402547053	0.489290519	0.324706354	0.324474178
0.470887802	0.204657404	0.561679958	0.586010863	0.529415228
0.379165191	0.214235543	0.158277944	0.3818611430.497133151
0.401761717	0.489776075	0.28850075	0.641324984	0.348578574
0.522635028	0.315183049	0.30010826	0.417392398	0.608132887

0.347474944 0.519924389 0.398024149 0.390199774 0.403997352
0.440068267 0.334702156 0.329344141 0.334846228 0.190337905
0.362307939 0.37956843 -0.131750619 0.292870049 -0.160905839 -
0.064281204
TCGA-55-7903-01A 0.444280936 0.396120443 0.4681512 0.383270842
0.245057084 0.482496654 0.373376051 0.496753087 0.435858891
0.487247424 0.400678588 0.462853934 0.324712444 0.342021712
0.428371299 0.174416803 0.533207252 0.560263904 0.493289422
0.317454552 0.164149007 0.149174597 0.355045879 0.546924059
0.426274301 0.559487354 0.467156078 0.701695921 0.505723388
0.332762868 0.2661197950.282649668 0.40272806 0.61128281 0.430605149
0.549392913 0.438269963 0.424094087 0.303507271 0.302067482
0.323634808 0.277851451 0.322180798 0.143631286 0.380629684
0.335620178 -0.088413012 0.241028983 -0.207765174 -0.102227894
TCGA-55-7907-01A 0.42927902 0.387247548 0.468189182 0.371942756 0.26125274
0.544239193 0.3622857110.46334945 0.373675719 0.492041927 0.426783354
0.4503983110.321505334 0.352591735 0.467363544 0.2111944540.556799794
0.454208947 0.442641327 0.338679074 0.20215783 0.12901405 0.381343383
0.5115891760.395875009 0.541839539 0.394172237 0.669217892
0.435929809 0.46260206 0.276161046 0.289592202 0.429881771
0.603633907 0.405437714 0.530821395 0.416733355 0.425379475
0.343655797 0.410380386 0.314635976 0.327916852 0.350805151
0.147941206 0.4111803540.347703131 -0.074663783 0.299263989 -
0.139413789 -0.116336382
TCGA-55-7910-01A 0.456145279 0.427864598 0.484674286 0.392420888
0.270626512 0.53838766 0.336744959 0.478448287 0.470738901
0.462855109 0.378954041 0.456336148 0.341654092 0.360172454
0.442101685 0.17329494 0.528981098 0.392526804 0.360463993
0.256703785 0.094350426 0.162039964 0.319543233 0.576142553
0.423646343 0.575555873 0.497825124 0.700808142 0.482630969
0.417514774 0.219598655 0.252281622 0.3760802110.637725044
0.432649429 0.526434541 0.402991037 0.433650513 0.321640658
0.4278011580.312824222 0.27605402 0.32380974 0.136139261 0.381818892

0.232865359 -0.030206496 0.233445056 -0.177871659 -0.057636248
TCGA-55-7911-01A 0.466433639 0.359994471 0.471632644 0.384097753
0.212709507 0.477395804 0.407007207 0.489657108 0.435726756
0.5118181360.3911408860.459741482 0.313106479 0.347974097 0.432652448
0.137861452 0.518258508 0.67387037 0.575505763 0.297659403 0.13992993
0.091497405 0.373171672 0.550837289 0.421286966 0.554652226
0.478773577 0.7040986 0.479298558 0.3445963110.308499171 0.280163532
0.410683045 0.613798941 0.385141231 0.527679109 0.42761632 0.42770465
0.293091385 0.358026836 0.313005767 0.294850079 0.334552501
0.1711159380.389605947 0.39067051 -0.095423847 0.251214599 -0.168153995
-0.09906772
TCGA-55-7914-01A 0.409076492 0.375754494 0.414081835 0.335153451
0.220907459 0.530588668 0.365893802 0.458753377 0.364994941
0.460187312 0.355372727 0.471870533 0.321391045 0.329628425
0.445486126 0.189848323 0.530656712 0.49921915 0.450505544
0.281276625 0.141460425 0.127623708 0.329966727 0.546257728
0.390451506 0.534662202 0.38122598 0.687026752 0.493819247
0.398782664 0.236424258 0.300363929 0.415433109 0.620157041
0.405083703 0.536431757 0.416248254 0.415202509 0.297345751
0.357474852 0.329328073 0.295290529 0.327840378 0.146683539
0.369977247 0.327480544 -0.088479287 0.259120963 -0.186523329 -
0.069107076
TCGA-55-7994-01A 0.445481345 0.386529732 0.478944217 0.42769322
0.242477596 0.506733001 0.412059867 0.473582091 0.467400911
0.479204569 0.390560089 0.452459789 0.322212749 0.332564487
0.459586534 0.146451051 0.561327502 0.550968857 0.494640862
0.327801557 0.190437678 0.101481267 0.378928128 0.513481079
0.434194975 0.574820539 0.493936085 0.679495094 0.483455064
0.339358574 0.308531897 0.272860566 0.409733603 0.566891624
0.42980796 0.52043823 0.416371739 0.417123381 0.315067703 0.349335594
0.333746895 0.279589186 0.35657045 0.174963024 0.391680565
0.395314065 -0.071809778 0.244722679 -0.20340444 -0.107405177
TCGA-55-7995-01A 0.4011169370.409028863 0.497194539 0.406779229

0.264560126 0.468494414 0.39145138 0.477424126 0.4546625110.479142525
0.4027119340.446640962 0.318875789 0.344613829 0.434763486
0.178282295 0.515878378 0.537400216 0.504635278 0.296928807
0.202457016 0.137021337 0.370638639 0.513235018 0.451543211
0.604328686 0.487845635 0.674635366 0.439264626 0.371099792
0.267856212 0.328362375 0.44101861 0.5811186540.441014451 0.598846486
0.433598841 0.421207573 0.304962496 0.339200813 0.320158594
0.264161473 0.352567568 0.175491599 0.380045805 0.395772995 -
0.054291032 0.236908347 -0.159530835 -0.158342722
TCGA-55-8085-01A 0.439435317 0.393092643 0.452786658 0.358765169
0.268328598 0.469058478 0.369741797 0.491613201 0.438943477
0.459100821 0.322687815 0.472720284 0.281768742 0.324911012
0.423201019 0.178952147 0.515508825 0.510264952 0.473474976
0.288084829 0.149164283 0.086577133 0.362427709 0.559977178
0.421746568 0.5711484790.46937613 0.709661776 0.5176717110.384218116
0.278680897 0.32443937 0.43696478 0.621593199 0.41352452 0.592821647
0.434720496 0.4438693110.281947705 0.390194552 0.312880621
0.298237509 0.328876894 0.148956473 0.38680633 0.36716529 -0.064325326
0.228772422 -0.160094299 -0.074795346
TCGA-55-8087-01A 0.328059103 0.328245509 0.506975136 0.314981078
0.244027359 0.523456968 0.284283868 0.443397676 0.254869302
0.439468437 0.456417434 0.458765328 0.308552959 0.316031636
0.466974607 0.19012393 0.526788055 0.367133216 0.292101658
0.315183188 0.136329583 0.158641707 0.281814352 0.506949566
0.395655078 0.483743344 0.267894341 0.624802681 0.374103156
0.357566825 0.169235356 0.272159382 0.417401729 0.596433704
0.384712492 0.49994467 0.427929404 0.384712409 0.351302544
0.335905236 0.328320072 0.266762585 0.291879389 0.153230205
0.375261762 0.216201989 -0.136855983 0.189201459 -0.147247862 -
0.089888701
TCGA-55-8089-01A 0.5115008950.405400437 0.448251491 0.41223541 0.234642031
0.527864942 0.46534552 0.473349069 0.457862637 0.525356559
0.368400753 0.466423502 0.3406092 0.362660841 0.463477766

0.159856887 0.5691621130.608637589 0.5711499940.354606783 0.265882357
0.154984247 0.421063668 0.53461589 0.442335654 0.576050986
0.479837839 0.690538078 0.450724697 0.459126135 0.361033378
0.331532182 0.404422277 0.595061994 0.41730469 0.589805214
0.468873806 0.406522337 0.363926219 0.383635244 0.322237966
0.307399194 0.375072801 0.146952068 0.363886941 0.462201715 -
0.081496921 0.332696419 -0.16958808 -0.106837058
TCGA-55-8090-01A 0.520153349 0.440673865 0.45443045 0.378484479
0.289572985 0.583953283 0.373795387 0.471423964 0.370588417
0.509666879 0.445523134 0.454399452 0.340004923 0.362779614
0.454825303 0.216157608 0.528836934 0.496140481 0.44121708
0.356024085 0.177359008 0.2113870060.356915131 0.519067392
0.409442749 0.537507839 0.375802083 0.660329882 0.41163931
0.484031942 0.296493381 0.272037236 0.394297294 0.589924321
0.439225123 0.541926212 0.476842296 0.432683343 0.358990269
0.436414642 0.337693298 0.346662034 0.360675923 0.152527574
0.376265186 0.290784609 -0.112547128 0.305804077 -0.152445228 -
0.085607105
TCGA-55-8091-01A 0.545295055 0.451600305 0.523958447 0.339048605
0.253602846 0.5622211060.423749951 0.443591386 0.33046931 0.527645669
0.450899802 0.472054404 0.338375496 0.356594454 0.516669237
0.215239469 0.550784008 0.522171743 0.504313145 0.353307037
0.223652133 0.16083594 0.38860308 0.533767997 0.419519793 0.567184242
0.3262845110.650475465 0.375203943 0.563247217 0.35428012 0.325019322
0.426383538 0.592725534 0.43169873 0.564757232 0.472545862 0.43699203
0.3901097 0.483254732 0.3479119420.364932864 0.377789265 0.160443176
0.377595869 0.356042752 -0.117665796 0.361795244 -0.164938665 -
0.040000727
TCGA-55-8092-01A 0.428722359 0.391685657 0.501425302 0.347138919
0.254904587 0.492803955 0.356088947 0.479342286 0.375775844
0.477333962 0.328957978 0.459408952 0.307138246 0.319906809
0.429042165 0.164041776 0.565079496 0.546216547 0.508929629
0.302972956 0.186307602 0.086749864 0.357899826 0.571697283

0.4113769020.5575041 0.387877815 0.67731837 0.46313578 0.439581404
0.242720652 0.301957065 0.423644358 0.631389413 0.412968815
0.523875312 0.407218605 0.4211827790.321583867 0.351025645
0.309253376 0.294818003 0.313047773 0.142657337 0.357455296
0.369467844 -0.111055774 0.268167232 -0.189826043 -0.126770392
TCGA-55-8094-01A 0.365534956 0.388632037 0.510676068 0.351934871
0.1811603520.3867119160.200714717 0.489509657 0.434094038 0.40736328
0.285131566 0.456897526 0.299549063 0.352191424 0.450572365
0.150218292 0.543871258 0.47161516 0.327057655 0.248286715
0.067144979 0.137234673 0.324126271 0.586674413 0.389948915
0.585648693 0.466310619 0.697618041 0.504391781 0.265842127
0.12192658 0.312555108 0.427699836 0.636765404 0.4491491170.504493136
0.367124372 0.443761936 0.263996205 0.313206874 0.302223476
0.33789902 0.25772596 0.205290525 0.405750879 0.1174241150.003825969
0.172704016 -0.133696536 0.176641945
TCGA-55-8096-01A 0.4251121990.432603462 0.480333965 0.334158522
0.268905229 0.532259102 0.357680319 0.452016383 0.311250129
0.483537731 0.457708084 0.47473688 0.317946788 0.325028709 0.47927131
0.249526997 0.554303924 0.425813505 0.407659171 0.355552145
0.191278026 0.164551283 0.370490631 0.519973187 0.409070013
0.54239207 0.314120976 0.64405698 0.433719257 0.542974942 0.290983027
0.302548318 0.416766057 0.595103313 0.44933086 0.526964316
0.436817012 0.410482601 0.342389016 0.479401495 0.3112897220.36411908
0.332567882 0.163035059 0.375001356 0.318163594 -0.108575872
0.327829941 -0.166155882 -0.045671613
TCGA-55-8097-01A 0.419232671 0.345537791 0.481582852 0.320679475
0.261238313 0.553598216 0.329393421 0.455088536 0.274079714
0.46809637 0.443939139 0.463783588 0.32543911 0.329883832 0.4600386
0.231965228 0.520353071 0.402197652 0.342186003 0.3424704
0.145405601 0.164376868 0.305596916 0.500483432 0.396485923
0.471062475 0.285620604 0.637885646 0.4116089630.408199019
0.2196881120.282376409 0.407527297 0.593855272 0.361608878
0.498675239 0.456223143 0.417445706 0.357131242 0.364508643

0.310254725 0.303925017 0.303551355 0.153964247 0.359808466
0.275140697 -0.146066666 0.217109818 -0.126285977 -0.091241199
TCGA-55-8203-01A 0.43336585 0.37770566 0.463446646 0.3491105390.245422118
0.495996704 0.376989489 0.467049736 0.398049817 0.46558747
0.390345374 0.458846158 0.326037234 0.357575718 0.454080992
0.166019109 0.535525313 0.461644363 0.430889838 0.296343609
0.150485272 0.133686492 0.3511198270.558028487 0.4127811 0.559524609
0.407449901 0.687617253 0.464577721 0.430425704 0.235505484
0.279579977 0.41638675 0.619429394 0.420409723 0.531344564
0.424780554 0.414800947 0.292590068 0.396007554 0.326315954
0.319699401 0.334314696 0.139234727 0.3816958 0.339766779 -
0.09141961 0.250644328 -0.17828795 0.008813721
TCGA-55-8204-01A 0.484190099 0.419999578 0.428331716 0.370965204
0.312823049 0.5151150380.357474232 0.468056454 0.415907023
0.506692064 0.409000198 0.4501969 0.34628259 0.363717275 0.484395519
0.195523202 0.52721798 0.46894906 0.439013694 0.339309564 0.256136071
0.167494347 0.352673836 0.5573381160.403822173 0.571692837
0.438843034 0.70308336 0.500553393 0.512194267 0.283727887
0.302419358 0.397158878 0.609050506 0.434277395 0.531728511
0.477404196 0.421087727 0.348736084 0.432844044 0.314324397
0.332412622 0.337184634 0.122101502 0.390133258 0.301819888 -
0.059349891 0.277757713 -0.093294615 -0.121820412
TCGA-55-8205-01A 0.5277721190.395012576 0.455563614 0.438910357
0.2221101590.519688549 0.475746376 0.492156896 0.502371698
0.525494088 0.355746629 0.461852228 0.3103265 0.325894918
0.450866785 0.156647475 0.538619998 0.604643491 0.562306886
0.338849294 0.235533875 0.128524441 0.40794462 0.5411052690.442290156
0.580916352 0.541019534 0.715846323 0.540001743 0.439322006
0.373955026 0.283986895 0.398593232 0.602279028 0.414884174
0.547291626 0.4266622 0.432708986 0.342163125 0.388104048
0.3114796520.284000266 0.3801135370.140686278 0.370034004 0.449688401
-0.062216996 0.305844907 -0.225258004 -0.099292804
TCGA-55-8206-01A 0.472451525 0.365299652 0.435002576 0.338463686

0.240512472 0.536579862 0.398765784 0.445889672 0.277421526
0.492212253 0.447739729 0.486975947 0.321942412 0.323546222
0.494018731 0.201662984 0.559984672 0.429693153 0.393144598
0.320356733 0.167290275 0.154226591 0.367060481 0.499808613
0.402698626 0.49049933 0.3020711050.62743519 0.382202175 0.401214826
0.29458461 0.338157399 0.425481515 0.595702434 0.354354273
0.520534454 0.462264104 0.423291888 0.359298759 0.406912756
0.330202133 0.309569733 0.356385944 0.173106887 0.369694431
0.337136756 -0.139315955 0.284911247-0.156397471 -0.086179747
TCGA-55-8207-01A 0.511141811 0.42706609 0.44916728 0.327924873 0.25594837
0.556204179 0.367701576 0.439023695 0.262103022 0.497507209
0.496788779 0.47321716 0.353291476 0.353554564 0.477236808
0.262979469 0.551094782 0.430262695 0.394143032 0.358272288
0.17648479 0.179626024 0.376846815 0.513031204 0.399418982
0.499015788 0.280597562 0.640240531 0.395931766 0.535294381
0.307234191 0.3136811820.414665055 0.600193165 0.400456184
0.520760338 0.454865734 0.415985896 0.3911359490.456028323 0.32723001
0.376486849 0.357874599 0.156379458 0.373554187 0.296160038 -
0.140164173 0.333343204 -0.143948198 -0.026925963
TCGA-55-8208-01A 0.510784071 0.427797466 0.478775483 0.351221096
0.250263266 0.51066811 0.472702533 0.465470204 0.382558386 0.5203376
0.392419962 0.460967893 0.320734489 0.348336935 0.457441231
0.199878479 0.531230327 0.594270905 0.556227844 0.340503832
0.2119908040.134144859 0.422663561 0.545997033 0.427158778
0.579608967 0.415356964 0.675391267 0.436716955 0.503797937
0.390802691 0.338776109 0.415969984 0.595766577 0.41005445
0.595351449 0.464371667 0.431509698 0.331261924 0.461250722
0.329476317 0.353886733 0.400013135 0.152409903 0.370822179
0.455016118-0.101398612 0.340596783 -0.148426798 -0.002128048
TCGA-55-8299-01A 0.54259264 0.433014559 0.445917205 0.370561312
0.290592339 0.552416256 0.469243337 0.456069305 0.394896191
0.51438348 0.407564996 0.481992962 0.347890937 0.375634572
0.479213948 0.215210017 0.523190471 0.579770451 0.545000861

0.360554243	0.248340098	0.171619445	0.420517689	0.55874173
0.421328388	0.559273935	0.404902717	0.678028353	0.461170351
0.544188907	0.366305559	0.328169221	0.404513892	0.610077727
0.417130674	0.564355282	0.454394689	0.452674589	0.365870811
0.449333955	0.320887218	0.372388433	0.391279901	0.142507936
0.360628787	0.436297038	-0.099218141	0.331036592	-0.187938355	-
0.07185066
TCGA-55-8301-01A 0.426015385	0.4119325680.489176487	0.369374303
0.273484086	0.479488331	0.4161256	0.482929638	0.42451033	0.504476475
0.382337599	0.470667089	0.335597972	0.35741342	0.43840191	0.210942835
0.512869236	0.646476584	0.580424928	0.332472646	0.174181282
0.138072943	0.383920537	0.565484238	0.435167679	0.578871114
0.456796797	0.697859981	0.5098110440.456460199	0.301797358	0.28152839
0.4132119110.621555429	0.430545284	0.567550239	0.437683245
0.437342207	0.317666451	0.392296976	0.332457007	0.314698387
0.361881447	0.154943797	0.381584688	0.439500618	-0.098440991
0.266882458	-0.165207113	-0.036311018
TCGA-55-8302-01A 0.440260407	0.412005672	0.447889421	0.379741843
0.229222997	0.497630821	0.352352005	0.491446002	0.403361989
0.480438881	0.3867114070.464452404	0.34379984	0.357047534	0.407235007
0.189199225	0.51264016	0.598539355	0.503802075	0.334836355
0.194379733	0.129827877	0.350263354	0.542482323	0.412586986
0.55111226	0.433963523	0.674200994	0.4932331110.400634791	0.250027152
0.304405564	0.397665539	0.588876434	0.454594043	0.57688458
0.4489311420.420192832	0.298609947	0.326709938	0.306416976
0.315740031	0.338971717	0.11495073	0.358925015	0.317226989	-
0.100745458	0.242307268	-0.119415211	-0.109924269
TCGA-55-8505-01A 0.462047163	0.461216515	0.531817952	0.385994142
0.307429031	0.524504978	0.30762172	0.455558757	0.393173526
0.485204186	0.449928641	0.4913715110.390239264	0.400063905
0.456626683	0.217033125	0.541485061	0.409329822	0.35566558
0.330542876	0.204173733	0.1555612	0.347522827	0.564566055
0.412289807	0.59790096	0.396969164	0.669140657	0.469828712

0.544436128 0.217887559 0.369440956 0.4394111490.630689882
0.490556271 0.5955118530.46548081 0.433665609 0.343340366 0.430443627
0.317601258 0.374910291 0.297673161 0.1658279 0.394077707
0.211325661-0.084850376 0.257772516 -0.130953798 -0.067635352
TCGA-55-8506-01A 0.452676105 0.399906524 0.450088605 0.375925415
0.208708252 0.500896844 0.372096618 0.459836399 0.393942954
0.47053515 0.39552663 0.469951487 0.336133661 0.34846615 0.435819327
0.21859662 0.54051034 0.520210692 0.454897469 0.329004733 0.16544366
0.131254645 0.343524924 0.527161303 0.424392599 0.525495608
0.397606293 0.635734434 0.428399689 0.423681823 0.267121395
0.268079405 0.392130186 0.591836027 0.426963973 0.521396269
0.415315218 0.4113531650.309973758 0.35695299 0.332804153 0.31241481
0.31566869 0.157247506 0.372890843 0.290290707 -0.063709041
0.226897453 -0.134753529 -0.091984532
TCGA-55-8507-01A 0.443467402 0.39041297 0.495206317 0.374575757
0.279430643 0.524914431 0.344666276 0.466521331 0.419073983
0.464769543 0.39063733 0.473485621 0.340361632 0.382761643 0.42781463
0.218664979 0.4908253 0.4607058110.3790411950.296664297 0.153754008
0.141613637 0.334025931 0.537335785 0.4060117810.538292342
0.435282932 0.674703754 0.487269027 0.4200683110.244158384
0.291277783 0.415534705 0.609120972 0.403671409 0.508830533
0.414850214 0.437184316 0.304786279 0.398145994 0.31208618
0.334126192 0.307272362 0.154262159 0.3826073110.246781001 -
0.077459521 0.235685748 -0.159604867 -0.009283557
TCGA-55-8508-01A 0.44042523 0.43687597 0.451642472 0.33988637 0.265981752
0.530438182 0.361942255 0.456097619 0.372156192 0.468398417
0.399763569 0.44004888 0.359449584 0.364770677 0.441489167 0.19405895
0.515422153 0.416309832 0.3911941920.308274712 0.1860213110.176246997
0.3370461120.547757896 0.412447096 0.54365998 0.38198022 0.664498078
0.456746836 0.472056254 0.223804868 0.290476675 0.401164032
0.605484652 0.43846803 0.525423926 0.424053819 0.4141156130.300456251
0.385122865 0.32004367 0.319538714 0.314169102 0.140741752
0.367272519 0.28909063 -0.099079903 0.247767566 -0.147401005

0.114774403
TCGA-55-8510-01A 0.460709621 0.396667552 0.473050217 0.361100083
0.261272376 0.521565702 0.42030523 0.45858726 0.3559110630.502550943
0.41477362 0.473079853 0.329909788 0.3460315 0.43113776 0.222020575
0.51139066 0.519639436 0.467594505 0.367089086 0.194523149
0.144287938 0.379271238 0.512925642 0.414234466 0.53420548
0.389346405 0.654564302 0.446043658 0.469454864 0.303778727
0.342488654 0.428145233 0.5913116860.409303884 0.584686929
0.452138574 0.426894053 0.339600365 0.407319597 0.323636558
0.344938674 0.3528201180.167994025 0.387133588 0.395235891 -
0.1102233530.293190204 -0.159600569 -0.101939471
TCGA-55-8511-01A 0.540886621 0.406312154 0.483451303 0.393091261
0.2929479 0.510663004 0.401712215 0.457007121 0.424307597
0.488378867 0.428183327 0.447203167 0.340638563 0.333895051
0.468614606 0.184046764 0.523664939 0.456823327 0.458952588
0.359933095 0.186361801 0.12490997 0.380332274 0.550836159
0.426426228 0.5503608 0.435436426 0.670239729 0.498109887
0.487272068 0.315100649 0.291903195 0.399027094 0.587728406
0.416327353 0.544738189 0.44424539 0.437786552 0.318025508
0.4117827440.322417232 0.339086179 0.351660094 0.131558186 0.36035508
0.36411769 -0.077153008 0.269165581 -0.180656026 -0.144505218
TCGA-55-8512-01A 0.49440812 0.384393277 0.50852671 0.297292257 0.269011117
0.491373585 0.335843328 0.430669953 0.224540381 0.482145768
0.393764699 0.470973829 0.380232727 0.3828112940.466294058
0.235439372 0.499105565 0.440996 0.369239407 0.3139641110.171975926
0.149199834 0.304839527 0.489529724 0.378962389 0.489147404
0.239392236 0.605577025 0.383910236 0.364619185 0.230310619
0.3411342040.444064148 0.589679413 0.368315999 0.538910278
0.479186709 0.417578403 0.336495 0.334551826 0.329558065
0.3011157370.294391903 0.194050043 0.386075372 0.207748136 -
0.1107688830.189818215 -0.099706788 -0.07941466
TCGA-55-8513-01A 0.491563129 0.40164497 0.489198827 0.309013666
0.277244188 0.529717197 0.43531551 0.418140855 0.228592181

0.485686175 0.434577159 0.479091255 0.366491328 0.392541935
0.477067309 0.222457019 0.521954379 0.626506002 0.522009315
0.333046136 0.186426522 0.134619779 0.379962069 0.502630101
0.393588459 0.496878578 0.222391733 0.596443572 0.34668816
0.416286971 0.302145171 0.33289034 0.434402689 0.57830339 0.377488122
0.54506949 0.471738186 0.427366234 0.345136886 0.399258438
0.343757589 0.350289503 0.357962797 0.171955749 0.389146948
0.330257399 -0.142989051 0.292350373 -0.104848702 -0.070188824
TCGA-55-8514-01A 0.455979158 0.367955375 0.527199781 0.283115354
0.237694724 0.463063082 0.346317132 0.469403567 0.261602691
0.454776491 0.408906513 0.471723974 0.319025436 0.303882259
0.426257775 0.1811290910.49638226 0.517299925 0.437103673 0.305496904
0.152443039 0.139143131 0.302057998 0.524647571 0.392709531
0.506203064 0.279672693 0.62223193 0.477675781 0.32566201 0.252156166
0.288081541 0.396232395 0.59390624 0.409251394 0.517068669
0.427184574 0.418776039 0.282075195 0.312960318 0.304048079
0.280452705 0.3026148 0.160343822 0.3597451130.267372984 -0.13632972
0.230779318 -0.11427032 -0.097150116
TCGA-55-8614-01A 0.531469941 0.439842628 0.455945897 0.374404454
0.388552166 0.558315756 0.350970827 0.475488459 0.417444566
0.509007421 0.474980242 0.449191515 0.359303514 0.376796076
0.465202504 0.234533124 0.509404649 0.415970569 0.380015024
0.37504479 0.2115055390.181817405 0.330631746 0.541233791 0.396718195
0.529320149 0.434324721 0.680276076 0.490696388 0.545057151
0.270301247 0.280252214 0.383273195 0.582992788 0.42675339
0.5089771130.476572107 0.450088433 0.369970076 0.479197317
0.3114851560.360095872 0.332073561 0.1154427890.373673842 0.286471565
-0.080764964 0.265922114-0.093912441 -0.109528515
TCGA-55-8615-01A 0.365832109 0.378089713 0.44794361 0.3350611890.242945707
0.492431031 0.279750503 0.462073894 0.36160804 0.414203207
0.3502121120.459381303 0.331254208 0.354494174 0.432367814
0.199783419 0.509324601 0.3669352110.297341076 0.283378004 0.13240304
0.143080395 0.294850913 0.537833704 0.401456247 0.505291243

0.371539001 0.656847468 0.472576806 0.399889738 0.160002167
0.303694125 0.404199 0.618288547 0.405575621 0.509655581
0.416178741 0.415061795 0.27453197 0.3794031130.304314658 0.330467004
0.279133215 0.14581771 0.378434885 0.187438827 -0.058685464
0.215832535 -0.152968672 0.112638636
TCGA-55-8616-01A 0.396826091 0.393490969 0.48039644 0.358265429
0.255102231 0.479635167 0.294585582 0.462167416 0.321198567
0.467833672 0.409717177 0.478598942 0.352399585 0.345164006
0.456234087 0.220934235 0.509081243 0.388606204 0.3516627110.32115259
0.149966727 0.14203604 0.305774325 0.5115608410.393903775 0.514360153
0.350873431 0.647251046 0.488879594 0.3906119040.190057191
0.300842573 0.412886849 0.615848174 0.419417978 0.517527446
0.436963889 0.399368915 0.319894981 0.354513847 0.311852436
0.295361392 0.296759643 0.167972361 0.395121658 0.25078714 -
0.1153493810.205453486 -0.121448065 -0.071805999
TCGA-55-8619-01A 0.498619817 0.4173351110.475718987 0.340909064
0.297638487 0.532380243 0.449617901 0.415415151 0.268985278
0.503439476 0.423662768 0.493653948 0.3653114610.393667242
0.456314982 0.244842686 0.531341272 0.531623269 0.479802731
0.359951944 0.208548275 0.181237032 0.40640443 0.495169892
0.400572093 0.500707013 0.261512642 0.607262441 0.393279153
0.449755106 0.32976673 0.344426393 0.431884387 0.586864563
0.395782546 0.555744018 0.468248758 0.427036914 0.360313562
0.419476134 0.337983186 0.377589051 0.387104923 0.174216182
0.375031389 0.385178741 -0.161553819 0.339987394 -0.136895087 -
0.031431344
TCGA-55-8620-01A 0.299066472 0.353108804 0.472884249 0.395918279
0.287363781 0.407276651 0.257647858 0.499342146 0.49532441
0.415500596 0.319562159 0.432140236 0.281883273 0.309961169
0.394458178 0.155147144 0.503200953 0.400876973 0.341003395
0.257725665 0.077516493 0.206878994 0.309252161 0.521793775
0.415300213 0.586451677 0.54521446 0.707187465 0.529541993
0.287589368 0.122214662 0.286524554 0.403174067 0.621416975

0.414380226	0.555663682	0.372746072	0.420326471	0.232873083
0.315045794	0.309914609	0.236403316	0.271593375	0.181788437
0.361381044	0.265523456	0.017629927	0.172175471	-0.174571107
0.144401208
TCGA-55-8621-01A 0.476236484	0.425183706	0.505589775	0.3412029
0.299439962	0.531613923	0.429050342	0.439658586	0.30233543
0.499719481	0.435867561	0.502229663	0.351738633	0.375648252
0.47091299 0.264350548	0.524253264	0.576386628	0.514766996
0.371364632	0.252699126	0.171034674	0.394127777	0.527424715
0.414745435	0.538846439	0.312262357	0.639440521	0.399672232
0.479635028	0.332099959	0.329898352	0.443986484	0.596755703
0.383848151	0.553029035	0.456854439	0.429353141	0.372495656
0.457733982	0.33843892 0.357358059	0.373063366	0.187627024
0.392531248	0.381815071	-0.120580305	0.326802268	-0.138304415	-
0.043168199
TCGA-55-A48X-01A	0.445191607	0.374623287	0.479443638	0.349969934
0.257150877	0.500232226	0.377242976	0.444183991	0.326605739
0.4952131150.427279612	0.44654901 0.350220712	0.351636869	0.460695772
0.203003836	0.523201937	0.489169971	0.449862371	0.352340276
0.1985276110.146593532	0.371828755	0.507545316	0.406847208
0.515124615	0.341685419	0.645952078	0.397361436	0.428001022
0.25769586 0.294153291	0.402754183	0.576820744	0.400554293	0.52566328
0.445745466	0.402433272	0.337892799	0.392334328	0.321881384
0.342188331	0.331755871	0.165576896	0.38997663 0.356894003	-
0.1179532820.262635843	-0.155336833	-0.104037306
TCGA-55-A48Y-01A	0.4381166780.399189626	0.495447572	0.361087993
0.274051661	0.506586928	0.336627044	0.469050151	0.409598631
0.458094634	0.398371537	0.478140061	0.342254622	0.358397342
0.461083693	0.182696234	0.554546448	0.386078246	0.383567942
0.31753963 0.192619442	0.1511308570.342867539	0.57200197 0.424354636
0.579727853	0.4311980140.701619122	0.497914464	0.498218869
0.255251624	0.322535432	0.43910443 0.623365182	0.423990145
0.561361442	0.434379085	0.442369947	0.342618054	0.468959215

0.325971341	0.303160655	0.316453438	0.144575003	0.376971148
0.286481701	-0.098608174	0.264362339	-0.174514432	-0.07792553
TCGA-55-A48Z-01A	0.427096617	0.429568457	0.476702204	0.359540596
0.283408303	0.496163822	0.359783732	0.47008157	0.408468055
0.481168601	0.435299833	0.430094263	0.325997748	0.340549462
0.447873081	0.205639534	0.513987705	0.538786851	0.480050067
0.334229598	0.212222626	0.16282689	0.350272525	0.528032668
0.420048744	0.546421265	0.441217655	0.663566215	0.444634541
0.513793247	0.254467629	0.27557251	0.382296727	0.58423277	0.448711074
0.530687358	0.438471195	0.431117298	0.319089484	0.421523791	0.307320594
0.338036706	0.340242541	0.125717468	0.362144964	0.326837509	-
0.071765678	0.282373484	-0.169409727	-0.020471809
TCGA-55-A490-01A	0.429073507	0.412650989	0.448391655	0.406742897
0.30679699	0.505071832	0.332632879	0.468076552	0.435498332
0.451516784	0.419149415	0.435842621	0.337312684	0.328618518
0.435528559	0.22820235	0.514705549	0.361706354	0.348617224
0.345149195	0.231774009	0.149173386	0.33095921	0.546082821
0.437400058	0.520469873	0.435681636	0.665730533	0.480649238
0.465761921	0.256217072	0.275670273	0.3585471	0.561306271
0.417558266	0.524272818	0.415529966	0.406722812	0.33442336
0.441416747	0.31256198	0.312550018	0.326579077	0.097596664
0.348735767	0.280111781	-0.046699578	0.24288333	-0.14924729	-0.104503749
TCGA-55-A491-01A	0.410557822	0.407221061	0.495622551	0.324937953
0.271239315	0.480326333	0.364415984	0.475107854	0.343178423
0.478523647	0.415968544	0.459053139	0.335738496	0.323169181
0.446151318	0.227843905	0.50267572	0.487729508	0.438074931
0.334637887	0.187807693	0.146903475	0.37149084	0.544805523
0.427390552	0.561336366	0.402968644	0.662448325	0.452286883
0.445961987	0.280844589	0.30119489	0.405276632	0.610166569	0.44632875
0.529426892	0.433282196	0.427156383	0.287604046	0.393110372
0.297119601	0.345664013	0.349199718	0.136508765	0.364195945
0.348774946	-0.052338492	0.293975999	-0.132432326	-0.044661215
TCGA-55-A492-01A	0.328593168	0.341709529	0.460262905	0.250862629

0.259392298 0.413236484 0.249389864 0.477767893 0.236550287
0.420858966 0.367248028 0.506247814 0.316478839 0.336378719
0.400559815 0.214498134 0.504903568 0.42904631 0.340722466
0.266943712 0.09831461 0.107039069 0.294741435 0.499883141
0.371886328 0.497802967 0.285745692 0.643060949 0.446999346
0.273318777 0.13936313 0.341358124 0.492029751 0.658323787
0.396072622 0.592049282 0.438715376 0.402772583 0.265244596
0.3119006650.324839326 0.295606394 0.273291568 0.191539268
0.384870269 0.212254129 -0.129882752 0.155162072 -0.129269403 -0.009156457
TCGA-55-A493-01A0.517446231 0.445574424 0.452636089 0.440796568
0.24580797 0.52603149 0.452818813 0.479565332 0.498170509 0.511828942
0.379363836 0.443614401 0.3113080350.331629602 0.428123069
0.197417471 0.528094479 0.549042304 0.5111037810.37088843 0.23401522
0.169653231 0.37282573 0.560163868 0.431738088 0.582317863
0.528386762 0.702599569 0.551853045 0.496898245 0.347752484
0.264894151 0.362255599 0.57603694 0.463055843 0.557871989
0.449895771 0.457248586 0.315726241 0.440755149 0.303039645
0.302864437 0.387626166 0.082404418 0.3384118140.412699076 -0.040012331 0.3032674 -0.177791413 -0.089020651
TCGA-55-A494-01A0.361891502 0.350834517 0.495673607 0.371175926
0.236855514 0.500021302 0.195996353 0.464209254 0.395782245
0.425470981 0.428928258 0.447089167 0.340102565 0.331018831
0.458079197 0.172164328 0.504869598 0.337531408 0.265417816
0.296251724 0.156813878 0.15887564 0.233177321 0.5361139190.388197073
0.520793012 0.413496785 0.665752485 0.478247058 0.327661329
0.1199751610.269983656 0.39366001 0.596142259 0.417938484 0.488539828
0.404969592 0.41809939 0.280862608 0.372170459 0.306945903
0.257022002 0.271268815 0.145921724 0.382687463 0.123808228 -0.05790025 0.162347221 -0.151079917 -0.104444442
TCGA-55-A4DF-01A 0.442481017 0.428171289 0.489150087 0.396925739
0.242483482 0.477088544 0.3710211420.495432845 0.513987814
0.471310774 0.355750746 0.429335563 0.287594789 0.35199398

0.441410357 0.16208007 0.530524409 0.566704316 0.508820062
0.268762648 0.15515502 0.1108006950.349655504 0.554350695 0.421827165
0.6114846540.563968962 0.712853197 0.4539627110.375060067 0.265093668
0.298461221 0.424679823 0.597934217 0.431712464 0.562311572
0.428867216 0.449629022 0.288288686 0.35037903 0.32551372 0.24456646
0.337378294 0.133580467 0.3770211650.349402923 0.002288933
0.204402517 -0.147718924 -0.073700152
TCGA-55-A4DG-01A 0.424946528 0.36189035 0.474025167 0.325723217
0.291732674 0.50665839 0.341835981 0.436737637 0.286926834 0.47370462
0.434677898 0.460420519 0.330073424 0.335429668 0.480808598
0.200841858 0.537081576 0.417990907 0.374613882 0.301437186
0.151995012 0.1170452170.319509078 0.497133844 0.390624043
0.473595251 0.305869363 0.625323735 0.3903117890.367292855
0.220551674 0.298323865 0.434857272 0.595645571 0.376090996
0.491432625 0.426680869 0.4105581110.350348328 0.316764018
0.324861993 0.304416159 0.312156036 0.180912812 0.383412664
0.295544425 -0.111255337 0.230397201 -0.137550406 -0.137052668
TCGA-55-A57B-01A 0.480754172 0.389862514 0.447916735 0.367316408
0.29253526 0.544651609 0.3602851180.441796356 0.304046484 0.509484147
0.4601301 0.477123378 0.335839977 0.325540054 0.427125132
0.229900784 0.536675306 0.546900827 0.45911265 0.352169435
0.142777694 0.171351291 0.341490481 0.507652047 0.402090644
0.499878598 0.320470263 0.638520131 0.387704791 0.451654529
0.276031029 0.304937035 0.409350688 0.577350918 0.386641808
0.5211237790.45569698 0.423357372 0.360786793 0.413706202 0.335140851
0.347928563 0.328269268 0.172978374 0.380984584 0.300308335 -
0.129530455 0.280765378 -0.125155899 -0.084224012
TCGA-62-8394-01A 0.45166709 0.412195289 0.492575562 0.387404924
0.243023005 0.497855282 0.343580383 0.490216865 0.464176606
0.482461517 0.402075272 0.461204456 0.33859513 0.37338648 0.432961374
0.20216513 0.512253806 0.605418708 0.489376773 0.319645377
0.236386085 0.1176175880.346557952 0.522104459 0.414848357
0.568563431 0.513845764 0.685108362 0.465784208 0.420004131

0.251525957 0.294454871 0.422208832 0.593150566 0.451370026
0.523124394 0.453147423 0.458348808 0.290097402 0.369345408
0.300651833 0.328458981 0.33832227 0.137502173 0.390552519 0.27356201
-0.044754373 0.276365789 -0.115467657 -0.111726779
TCGA-62-8395-01A 0.407319937 0.3911319830.47472881 0.322554982 0.31425336
0.53379255 0.31685144 0.430466089 0.26627627 0.461341784 0.417623303
0.494251576 0.372326742 0.377458261 0.474104707 0.237582432
0.525198825 0.403483852 0.351943883 0.343520279 0.157798208
0.163748463 0.31909636 0.509338952 0.413927506 0.5113501920.285056943
0.63044849 0.430554332 0.423157967 0.224770423 0.352582803
0.458935924 0.603820918 0.389466046 0.548840279 0.450711429
0.417371223 0.34877488 0.434694302 0.339074429 0.340983808
0.3119684540.183302808 0.3811291860.257325801 -0.136302964 0.236535022
-0.134864176 -0.02544933
TCGA-62-8397-01A 0.378886043 0.391354845 0.465916845 0.324768701
0.251043085 0.497408224 0.314339919 0.455918481 0.29084168
0.473774481 0.398502363 0.500962876 0.338180378 0.349055576
0.446722943 0.221702516 0.5394370110.393845743 0.342456602
0.328096635 0.19025133 0.155730847 0.329761738 0.489631096
0.403701013 0.521752807 0.299263336 0.622045196 0.411111942
0.376995262 0.210561996 0.338573088 0.446235431 0.633428282
0.431048579 0.550319721 0.440803626 0.415276815 0.306186942
0.385181594 0.330136264 0.318316608 0.325893349 0.187936418
0.404666712 0.259139665 -0.126722728 0.254154482 -0.154612171 -
0.07511062
TCGA-62-8398-01A 0.468874039 0.435362221 0.516067812 0.404562832
0.295233862 0.507918097 0.359034941 0.471863458 0.438908355
0.489530405 0.412156313 0.488326313 0.36859641 0.394105814
0.468884388 0.184883244 0.543648225 0.456889334 0.418992332
0.335294929 0.204700808 0.145963812 0.375981208 0.554682542
0.4118799550.590141685 0.452930856 0.689378286 0.506034904
0.453760603 0.268607899 0.355129965 0.4510509110.628685378
0.476327467 0.6046911940.460184565 0.421767339 0.295856839

0.392629893 0.312141495 0.3211360420.342435642 0.177039043
0.401059448 0.304236581 -0.105868463 0.285915578 -0.172349845 -
0.062606978
TCGA-62-8399-01A 0.5115135620.403896873 0.4811926340.395140527 0.269684826
0.540732915 0.338268387 0.475471252 0.407709432 0.485777773
0.3941641140.463860196 0.357522066 0.354730408 0.456065134
0.202549793 0.512712871 0.40250595 0.379672273 0.337220662
0.166359772 0.100888154 0.314752371 0.53344257 0.429777975
0.5117763540.423969561 0.674540013 0.4883143 0.450668706 0.27324977
0.288061305 0.406035356 0.582704847 0.39660752 0.4974626110.420882557
0.415666228 0.363298259 0.412253694 0.303004241 0.317812798
0.305144516 0.132824932 0.3651153050.248644429 -0.056013016
0.242469529 -0.158200668 -0.094435477
TCGA-62-8402-01A 0.431591246 0.350574514 0.490153298 0.376442271
0.282572141 0.477287043 0.388597674 0.491891726 0.468732646
0.468842123 0.385130417 0.426026398 0.315648344 0.314652718
0.443063745 0.1379596110.53033844 0.634746648 0.548254253 0.285968816
0.11496889 0.133743931 0.361244173 0.574372886 0.446935101
0.589917902 0.485924342 0.707898979 0.506815433 0.32442605 0.27397943
0.264397456 0.400885803 0.606684672 0.41267101 0.503786055
0.405874473 0.423142268 0.251085491 0.354529781 0.271446055
0.267462488 0.328270056 0.1181852040.384775623 0.375041028 -
0.058728558 0.220600778 -0.144808041 -0.02572627
TCGA-62-A46O-01A 0.341554273 0.364719547 0.454542703 0.4091503
0.221562131 0.500443923 0.186971526 0.4928990110.521947955
0.414535123 0.282133996 0.458106994 0.33412962 0.359693092
0.458817547 0.125763551 0.6114657070.439630458 0.323859595
0.221704197 0.167001375 0.02869577 0.25492978 0.561836842 0.44014298
0.582632063 0.549701571 0.729484162 0.488082513 0.286759662
0.11624205 0.281389751 0.396003158 0.619676542 0.4214670110.566626218
0.371396689 0.402631506 0.289885193 0.28518971 0.31031559 0.21679844
0.239983556 0.1128829850.364266179 0.1192911930.02322746 0.105230104 -
0.178913366 -0.138456191

TCGA-62-A46P-01A0.388857564 0.372557582 0.480020931 0.267855057
0.245826643 0.451205071 0.315677633 0.471920954 0.279268572
0.424817423 0.357166244 0.477225629 0.331530504 0.364905689
0.419306292 0.194207873 0.517989684 0.444812154 0.356732246
0.256033575 0.096481231 0.137214585 0.299754471 0.539276764
0.384417504 0.513264443 0.309818285 0.6426112720.4608411230.310133371
0.182245745 0.290761594 0.433884582 0.642970971 0.405815299
0.542053637 0.4411684030.438788419 0.261080975 0.390457362
0.3110138840.297509143 0.28847748 0.1575419110.39303292 0.220388332 -
0.120178412 0.209226751 -0.141450224 -0.060336107
TCGA-62-A46R-01A 0.40304242 0.355540077 0.4211407760.363292846 0.2142944
0.45910975 0.356777566 0.469654356 0.37474693 0.471557926 0.408964305
0.468418003 0.3113133460.316649169 0.440531957 0.167430055
0.576868346 0.554359484 0.462567294 0.314333565 0.161741699
0.1566046 0.337939748 0.531400068 0.414675675 0.525736047
0.418787613 0.673672256 0.434737388 0.364356952 0.250914823
0.2971197220.404486971 0.608385057 0.417725624 0.52850405 0.400334193
0.399736624 0.316458906 0.414446945 0.3117338180.270335973
0.325959821 0.151240861 0.382730071 0.3209078 -0.081583752
0.249949975 -0.177618525 -0.126815737
TCGA-62-A46S-01A0.457262943 0.399980695 0.488771564 0.30531291
0.242675869 0.52361956 0.352548274 0.449184188 0.283908759
0.453198794 0.387758813 0.480616166 0.331353418 0.331857596
0.499490039 0.187481054 0.557013031 0.4487767110.39622539 0.285665053
0.103772272 0.1591311960.340241456 0.539878898 0.392765044
0.508888657 0.290624851 0.644936868 0.415163587 0.350257403
0.24484463 0.301970759 0.449732642 0.622206332 0.404694487
0.5201136840.43309424 0.437893828 0.346244149 0.395684617 0.330180365
0.326984462 0.330138378 0.174293543 0.386025251 0.276264297 -
0.101854025 0.273273053 -0.152899251 -0.009428145
TCGA-62-A46V-01A 0.373553314 0.367987926 0.444342335 0.344298367
0.231836646 0.459066687 0.28223673 0.481658721 0.3511682250.457954657
0.4002811340.485714025 0.289217657 0.315904399 0.430345429

0.217200257 0.534764376 0.427332818 0.356288109 0.333834818
0.147681374 0.12792629 0.316305334 0.540279318 0.384196009 0.52114073
0.382532346 0.668050436 0.437722331 0.460399759 0.189354855
0.308469854 0.440459223 0.624813368 0.41915458 0.523969843
0.423105199 0.422387504 0.286790026 0.388160549 0.313773845
0.343597063 0.283568393 0.163362757 0.393786973 0.220893863 -
0.091729036 0.231152536-0.173470608 -0.091712446
TCGA-62-A46Y-01A 0.447766727 0.397197041 0.465958971 0.334489492
0.21163229 0.466536707 0.395023591 0.485469748 0.34472818 0.486900863
0.398161604 0.490200577 0.310379394 0.332369624 0.449654709
0.165790524 0.546898814 0.538641763 0.472105096 0.313463823
0.149104572 0.104322174 0.371844799 0.5292411360.403669803
0.550735172 0.38647242 0.678782747 0.451378505 0.352949287
0.286866126 0.308273185 0.4303111670.634399852 0.439365364
0.546064832 0.430369779 0.419432577 0.304358253 0.372968134
0.3261123610.302541952 0.334502003 0.171500734 0.40903454 0.350694556
-0.094636516 0.27379351 -0.166435335 -0.085177777
TCGA-62-A470-01A0.423155379 0.392986354 0.489526855 0.325880375
0.273030976 0.488381093 0.278422488 0.463253205 0.370505805
0.434784194 0.387838849 0.500945746 0.307878761 0.355699342
0.445095634 0.1915054110.540965817 0.391339368 0.335722401 0.24396717
0.085734033 0.127628481 0.321499995 0.54092394 0.410558676
0.560322207 0.39817538 0.67430897 0.47050498 0.304355241 0.208973579
0.338166914 0.462225259 0.639381204 0.415531866 0.559471716
0.434008624 0.435492351 0.291916074 0.352616337 0.324138471
0.281632016 0.299532934 0.164378653 0.386557412 0.207910761 -
0.086304762 0.21543538 -0.174199416 -0.075372766
TCGA-62-A471-01A0.44744335 0.421762641 0.473959579 0.401685772
0.264269212 0.465194056 0.297366023 0.495683032 0.486814196
0.466659597 0.340226402 0.47371077 0.354589367 0.3890211360.443424007
0.159723149 0.517362352 0.512158849 0.4118301270.277130165
0.193577995 0.100060352 0.345286641 0.563043609 0.431667818
0.584905852 0.508994325 0.713919806 0.53762102 0.352746246

0.192290489 0.354745766 0.4469114920.6611569380.4697336 0.61529266
0.449449572 0.432180877 0.264050139 0.325843421 0.312523312
0.305351023 0.284626243 0.134193641 0.372489061 0.223477871 -
0.058485883 0.193700037 -0.146473842 -0.09084011
TCGA-62-A472-01A 0.426483417 0.377752517 0.432140013 0.367918751
0.22343919 0.480377358 0.357009652 0.480739075 0.417929787
0.493769415 0.378809205 0.468876753 0.320149833 0.361589243
0.442153878 0.198061382 0.543353099 0.575065352 0.488394808
0.312098301 0.132279893 0.139807667 0.350132155 0.538937374
0.420521887 0.553285737 0.452827865 0.7071138770.505153187
0.347703285 0.259665416 0.280702272 0.414770866 0.618000172
0.413478704 0.540735028 0.421732148 0.431848466 0.295651494
0.356346925 0.325069109 0.2994811280.334691091 0.151283804
0.380444169 0.331376914 -0.079060653 0.236378761 -0.181684646 -
0.092999308
TCGA-64-1676-01A 0.485675176 0.418191702 0.4439734 0.278353171 0.16480838
0.49152924 0.405582931 0.495764073 0.372097535 0.506318792
0.365889264 0.484344419 0.2930111310.350913873 0.444067348
0.168822595 0.526542477 0.497476663 0.470072271 0.272191683
0.18302534 0.072852959 0.3968911520.521415894 0.408202936 0.562522896
0.427050738 0.685607837 0.4611556550.456965665 0.319246037
0.306036826 0.427892259 0.663666429 0.460206673 0.570439483
0.432898779 0.436485365 0.289794466 0.419233745 0.310006152
0.327652494 0.337359056 0.158956272 0.39945098 0.385288128 -
0.076944778 0.325262918 -0.209806274 -0.070003357
TCGA-64-1677-01A 0.453528644 0.399849778 0.506533483 0.31014196
0.203310613 0.450241738 0.369421042 0.532553282 0.447069526
0.472588967 0.339710805 0.44970771 0.2957744110.358108435 0.426563453
0.137483804 0.501345588 0.504632719 0.463444287 0.242848885
0.139777021 0.056704827 0.3520233110.572901237 0.418723262
0.592131429 0.506031086 0.732849122 0.534807808 0.364077871
0.258688558 0.270655579 0.419822808 0.6653687110.454027507
0.528891964 0.427881751 0.444021493 0.254443344 0.357493662

0.302523085 0.283284396 0.327602719 0.124591781 0.379643879
0.328439808 -0.043699074 0.221856832 -0.169819896 -0.124330054
TCGA-64-1678-01A 0.323508777 0.387625394 0.45792732 0.302343541
0.156003977 0.450937348 0.2115292370.517044768 0.510551036 0.39465974
0.309483503 0.443840598 0.291425543 0.355404075 0.432449272
0.131217032 0.509236383 0.308428828 0.279278124 0.173342221
0.081742578 0.059477788 0.260293133 0.568553488 0.40531564
0.602331829 0.548677186 0.734060781 0.535223594 0.33643154
0.107655542 0.263422041 0.432062863 0.672630455 0.434166749
0.540332891 0.38527918 0.436277812 0.227243487 0.29962187 0.279994067
0.243048902 0.274915234 0.152109267 0.391630517 0.15278101
0.023435976 0.146354192 -0.140096676 -0.001504143
TCGA-64-1679-01A 0.473192172 0.466889273 0.466642365 0.390258997
0.285291781 0.549433279 0.383688372 0.443596736 0.395917648
0.506817479 0.464835237 0.45867469 0.371524817 0.365343069
0.479255261 0.240467812 0.549201913 0.473076148 0.434048856
0.393890142 0.250573945 0.192046731 0.365984398 0.521491382
0.4237594110.548786275 0.394060866 0.654660389 0.4021144920.59280394
0.32368547 0.279815031 0.377174999 0.5644371120.4694054 0.525591891
0.435189185 0.421021862 0.408277999 0.477703485 0.322396145
0.372597322 0.340360661 0.132196886 0.369699815 0.309961416 -
0.060029389 0.31723967 -0.119952818 -0.076872379
TCGA-64-1680-01A 0.412359296 0.36739045 0.464617948 0.277371288
0.179233522 0.469668219 0.316780139 0.487327555 0.308780187
0.483723355 0.357333586 0.488766194 0.3244341 0.361019617
0.441647055 0.14656661 0.533742519 0.434020953 0.383619559
0.263782567 0.131361596 0.073667437 0.3099981120.522510975
0.385744863 0.526055485 0.357640479 0.672524216 0.461133821
0.330186952 0.225917245 0.299812573 0.434790291 0.646602182
0.421821495 0.520241984 0.452540913 0.4137371110.297454751
0.260164591 0.3192811960.302143503 0.285990336 0.18175639 0.413810738
0.245512731 -0.097434607 0.238568784 -0.142346642 -0.074484828
TCGA-64-1681-01A 0.474030059 0.412866951 0.480933209 0.312893912

0.231925683	0.547949314	0.377792151	0.445774926	0.315561012
0.490560961	0.3949611480.489082104	0.3351148720.353559982	0.465013424
0.232810052	0.527653349	0.588677892	0.494247049	0.330920877
0.166234022	0.141221488	0.350146771	0.509772957	0.402744023
0.52783614 0.341558085	0.650264664	0.41079566 0.44921849 0.280366664
0.319014158	0.429419601	0.616192224	0.415974001	0.532907204
0.460128024	0.425368826	0.336143798	0.417003338	0.329267401
0.340363916	0.347013125	0.215153937	0.422156572	0.305388647	-
0.101312949	0.273187404	-0.130933113	-0.075528862
TCGA-64-5774-01A 0.420994639	0.430656916	0.428859161	0.392079959
0.27848088 0.499528557	0.285739895	0.474871543	0.48856753 0.426263918
0.366479209	0.445217441	0.316351916	0.329459482	0.426871517
0.163695435	0.540217164	0.403673501	0.3502451	0.273491404	0.17697197
0.107596017	0.28813554 0.567379763	0.417856623	0.565505796
0.502809178	0.699201822	0.493755231	0.421028752	0.1853552
0.251873708	0.398650337	0.630519134	0.439305078	0.490015123
0.4011803980.43103307 0.284599944	0.396456663	0.295451374	0.268634288
0.304592412	0.1129673	0.379508291	0.206337698	-0.008201715
0.211452014-0.180524787	-0.041315611
TCGA-64-5775-01A 0.467445253	0.428417998	0.449389966	0.428235797
0.366046258	0.57195022 0.35287607 0.503808627	0.501847543	0.48764583
0.3481151110.452556425	0.223303399	0.247538136	0.447975577
0.166323109	0.531461993	0.462582474	0.393214449	0.319977814
0.177220596	0.207043268	0.340165892	0.566047803	0.411557079
0.576940172	0.524036891	0.729028416	0.555060603	0.533016619
0.259392892	0.244049228	0.386054724	0.603729013	0.41411183
0.504857715	0.416613875	0.397672957	0.398703267	0.457696301
0.282779594	0.308430845	0.329244925	0.098497694	0.339442523
0.273958133	-0.045334372	0.269331661	-0.241027453	-0.067242484
TCGA-64-5778-01A 0.433961642	0.363632056	0.440045019	0.301782958
0.1881156940.440028204	0.364501986	0.503435941	0.360438604
0.473902514	0.330557774	0.461064202	0.297955446	0.34954593
0.403582126	0.159904328	0.503289574	0.60578746 0.520959138	0.27746995

0.155355269 0.014126076 0.363958681 0.517208263 0.398070724
0.518863877 0.429271239 0.687002171 0.476264261 0.323571393
0.267607362 0.293060435 0.423694491 0.650200674 0.41889155
0.5411998310.429312178 0.42448886 0.259127968 0.261734056 0.291638579
0.299954597 0.343235606 0.1511580730.410700531 0.395819027 -
0.061275421 0.270552358 -0.160627942 -0.110222811
TCGA-64-5779-01A 0.44966028 0.4221106750.464720107 0.398889252 0.258488029
0.548697206 0.318867828 0.455723187 0.454785405 0.482459647
0.436389933 0.42836815 0.330677204 0.326866328 0.469106554
0.2081190620.545106945 0.419996668 0.394532353 0.316130322
0.158599409 0.156026673 0.340094138 0.557830502 0.408466195
0.550160619 0.476148583 0.698841038 0.466165251 0.501020962
0.219797753 0.23808081 0.378558767 0.610996005 0.402737319
0.485737683 0.393856597 0.43208913 0.336638025 0.466790332
0.297234615 0.284259722 0.329804989 0.1188553850.363038083
0.291704678 -0.003523124 0.274975435 -0.156399838 -0.003215061
TCGA-64-5781-01A 0.533161382 0.429725035 0.461091872 0.323249309
0.23375916 0.516026378 0.38608108 0.500153423 0.398096801 0.510774027
0.348105354 0.475031076 0.348739332 0.394002108 0.419090628
0.192462258 0.519283382 0.516231399 0.484405008 0.322013876
0.237451926 0.098672503 0.372415262 0.5532311430.421213081
0.557678151 0.429218407 0.681445468 0.490881376 0.530121175
0.280536537 0.32340171 0.39893238 0.651439785 0.449836371 0.580506488
0.467824234 0.465272415 0.321358727 0.395554981 0.313668081
0.34475334 0.32926803 0.156598626 0.394266856 0.390491452 -0.067779698
0.273363126 -0.159888323 -0.105851225
TCGA-64-5815-01A 0.525347612 0.488168491 0.485926435 0.3657337
0.275699813 0.562917678 0.392860065 0.4411822990.350500894
0.516564299 0.455397623 0.482802854 0.366560051 0.386152063
0.472052196 0.242541216 0.541025307 0.520802698 0.478029724
0.37921449 0.236592309 0.208358423 0.402855386 0.525248635
0.407624225 0.546567023 0.348044033 0.644377053 0.402807015
0.603219443 0.351595425 0.319531467 0.403795936 0.586989667

0.453892184 0.534582382 0.473860072 0.431024767 0.361470898
0.507921094 0.326939015 0.400655396 0.384014958 0.159601597
0.368158602 0.355468668 -0.11978851 0.373189476 -0.146693109 -
0.038216221
TCGA-67-3770-01A 0.417089453 0.3925991130.493946104 0.24999921 0.151370483
0.487023895 0.326134651 0.46685415 0.270597163 0.498370907 0.32201928
0.512273637 0.322318477 0.346715162 0.446632107 0.210321998
0.53837197 0.507644515 0.454524294 0.280494993 0.080299086
0.046634454 0.348195317 0.512356822 0.407691244 0.551575194
0.3258261120.646226234 0.394715393 0.374907462 0.2755749 0.351235945
0.479478123 0.664786306 0.43452613 0.532647794 0.458522499
0.440522986 0.2811636270.355550025 0.338308149 0.323826151
0.332171796 0.240045345 0.432726469 0.311405246-0.100783025
0.273089034 -0.126820204 -0.09182628
TCGA-67-3771-01A 0.397140143 0.420033579 0.478488798 0.342888616
0.219768757 0.487968487 0.340124318 0.451341858 0.396248598
0.466145696 0.386358021 0.459492472 0.344820717 0.363916697
0.458867594 0.204546536 0.551232724 0.451500962 0.427114649
0.306267295 0.150164235 0.158080461 0.346724734 0.554276802
0.4112999950.589256886 0.400788821 0.678182058 0.463532519
0.422809349 0.266780283 0.3319500110.422735404 0.606837046
0.448929418 0.5757580110.437927233 0.439379551 0.296303035
0.325230618 0.33431482 0.294157755 0.322823692 0.133548145
0.362712141 0.322655065 0.0171106450.239259878 -0.098102107
0.022253307
TCGA-67-3772-01A 0.459521857 0.41689592 0.4942171150.265501922 0.180074255
0.510460121 0.359615801 0.457665139 0.286409196 0.489712327
0.398879689 0.502349477 0.338963927 0.35717844 0.48932914 0.20284907
0.549207193 0.470803761 0.445370939 0.306620425 0.166584696
0.129419077 0.373983679 0.514627359 0.414289866 0.546082629
0.323428676 0.648402186 0.376306773 0.495314743 0.289845069
0.33487833 0.457310428 0.63782968 0.44292866 0.537352799 0.459207503
0.413843001 0.322223213 0.410248622 0.32365342 0.340100969

0.341039154 0.193744161 0.39970025 0.328885117-0.120129631 0.316115672-
0.141979105 -0.057936246
TCGA-67-3773-01A 0.49797194 0.393609829 0.45837309 0.251352367 0.211083154
0.491265175 0.410791381 0.484533462 0.280969909 0.501408771
0.375714687 0.495469402 0.290267655 0.321839518 0.439588803
0.194463292 0.532568314 0.536199255 0.481244595 0.284494568
0.121577105 0.108286891 0.370721448 0.49444417 0.404537962
0.527838326 0.313269868 0.649288672 0.398627715 0.417661558
0.332032091 0.324635748 0.449956214 0.661741413 0.394078264
0.531548343 0.465835935 0.438960904 0.301423665 0.361513238
0.3362801 0.35299518 0.352494845 0.1961126560.392035657 0.371119118-
0.11965086 0.294635126 -0.167649287 -0.057708516
TCGA-67-3774-01A 0.48320063 0.41314975 0.499032307 0.317877308 0.240859685
0.549001035 0.380803657 0.4616185110.295291518 0.492360329
0.355835079 0.486607305 0.364457951 0.373619279 0.480127418
0.214270667 0.546506448 0.4571100390.427828508 0.317963774
0.162586253 0.129810997 0.3643811440.535281813 0.423247055
0.523131606 0.312706572 0.655482463 0.403693716 0.412848665
0.286602845 0.336680604 0.436927678 0.61523923 0.399223457
0.548596534 0.469230466 0.444359864 0.355755332 0.404782038
0.339142647 0.36081263 0.352963051 0.169209447 0.389226031
0.322854797 -0.097827612 0.28796479 -0.129967087 -0.010459016
TCGA-67-6215-01A 0.387749279 0.367778328 0.52199928 0.370568058
0.241250856 0.479069521 0.328973127 0.469937294 0.38739724
0.468429761 0.394753089 0.482056988 0.344446379 0.354393141
0.473222728 0.164813658 0.554991487 0.432608305 0.358759027
0.315304731 0.138273883 0.144013674 0.312690717 0.534522199
0.413369883 0.560939766 0.402743469 0.677976488 0.471446987
0.308272197 0.237947655 0.306438365 0.428291028 0.620037375
0.425820161 0.545131207 0.440873589 0.418980464 0.310391971
0.353509158 0.34492629 0.287828531 0.324351961 0.15951585 0.388427879
0.254315177 -0.119685848 0.232358306 -0.133075458 -0.084407371
TCGA-67-6216-01A 0.474371226 0.406696719 0.417425443 0.343846715

0.243812851 0.501582664 0.4119394210.481488637 0.336013741
0.501914793 0.39279682 0.473138772 0.335873007 0.345017014
0.445036456 0.171578253 0.537433662 0.528758795 0.463425642
0.3131162270.1723114310.147644461 0.373405361 0.514868544 0.415254109
0.524516187 0.370273532 0.663760775 0.453372281 0.390846873
0.3011926010.307858699 0.4118353120.613525486 0.435629585 0.544155293
0.468154264 0.430162154 0.325594096 0.344667575 0.323540547
0.328546883 0.349516756 0.148755425 0.390522343 0.344263533 -
0.137590515 0.286671101-0.146588213 -0.056665926
TCGA-67-6217-01A 0.430821259 0.370858787 0.494675242 0.322753351
0.2110760630.501957646 0.357036923 0.451501772 0.262342407
0.480254548 0.372309631 0.467473075 0.343160282 0.326282482
0.469414529 0.2139065 0.566363299 0.485504708 0.444688408 0.32981802
0.11346365 0.141496674 0.336241824 0.533977249 0.407723287
0.519896135 0.285375633 0.638756959 0.385020312 0.38000378
0.265967849 0.306371634 0.414345163 0.612097887 0.405636477
0.514294298 0.439665173 0.403295409 0.341885703 0.327201163
0.322428124 0.313438318 0.339162083 0.215867731 0.399625085
0.334955114-0.128490371 0.242924271 -0.178999421 -0.091648311
TCGA-69-7760-01A 0.424722056 0.394960204 0.449649266 0.344925897
0.248244337 0.530558305 0.250222079 0.480544856 0.415708153
0.470652353 0.405365471 0.450733149 0.327964333 0.326806868
0.471246905 0.165179089 0.581477458 0.450645991 0.391555368
0.287161827 0.141784394 0.18304822 0.3052251 0.584605127 0.424727179
0.586652575 0.459206071 0.708854741 0.5118417810.408100152
0.193389041 0.259140622 0.399033804 0.625385575 0.446504737
0.515528841 0.423437505 0.436925128 0.331429531 0.35322366 0.31069764
0.272790148 0.28605389 0.162366373 0.377756106 0.200724339 -
0.071492896 0.23348724 -0.181242935 -0.033881785
TCGA-69-7761-01A 0.527281047 0.410197376 0.476974293 0.422313031
0.240868259 0.567081501 0.439307545 0.42903547 0.412156001
0.5211484560.434705021 0.452697392 0.342996762 0.365796437
0.5112787840.158982712 0.585679892 0.604220704 0.5538118680.351217394

0.248796052 0.182494572 0.420910619 0.526888825 0.426294132
0.558685724 0.396716779 0.6615381170.39603392 0.490189072 0.343876663
0.310133435 0.408661003 0.58683774 0.424131814 0.530993242
0.429876658 0.414359187 0.370557157 0.439240895 0.322705595
0.3513098110.369751215 0.142857249 0.386786937 0.407698134 -
0.102096847 0.340513043 -0.158381739 -0.051117588
TCGA-69-7763-01A 0.482841307 0.422985498 0.4641156460.341377268
0.296887602 0.578730017 0.322509997 0.436647592 0.29064682
0.482437927 0.446724603 0.49175111 0.372324507 0.368009736
0.471241396 0.220632498 0.537145027 0.507368021 0.436447783
0.354588219 0.186752104 0.177445791 0.336370302 0.530076998
0.39549839 0.490332159 0.300170521 0.646319253 0.409622753
0.496333432 0.258139015 0.2981140630.4118927740.6118414640.392086096
0.513533713 0.449048487 0.419496791 0.397165238 0.449621936
0.324880292 0.351508544 0.338569883 0.136263967 0.369125152
0.265652209 -0.131778932 0.28169225 -0.125733187 -0.082247291
TCGA-69-7764-01A 0.3911346830.359386746 0.469321259 0.345806636
0.276580264 0.541372408 0.295644949 0.468144181 0.359200633
0.453965157 0.394953177 0.462477816 0.318543554 0.332126039
0.476948058 0.200830265 0.523819256 0.450472686 0.38604036
0.303020243 0.1120264910.165018152 0.307591779 0.512194039
0.426966583 0.488847636 0.374405831 0.66457227 0.438879829
0.388003825 0.195545458 0.284436818 0.420760727 0.605846329
0.382795038 0.512232654 0.423783433 0.398908322 0.347290502
0.375854858 0.322696765 0.3113095520.301859268 0.161834998
0.395582613 0.253908454 -0.103755389 0.196874767 -0.149016666 -
0.036173484
TCGA-69-7765-01A 0.479081735 0.45097676 0.5093711220.354773498 0.236650815
0.535298279 0.387199372 0.43270172 0.359919783 0.504024515
0.438316244 0.477670238 0.375169766 0.3607116190.497388315
0.216853182 0.5798482110.492725423 0.461756562 0.33717223 0.231741067
0.209685402 0.382742891 0.550215359 0.407367067 0.55957067
0.351044797 0.667460588 0.425040342 0.545083263 0.304808225

0.281972777 0.395740892 0.591072618 0.438100416 0.542936884
0.4116884540.409408493 0.401488619 0.463120822 0.332036378
0.360748613 0.36078393 0.159134465 0.38692147 0.349465626 -0.075718425
0.312441301 -0.177339746 -0.035885936
TCGA-69-7973-01A 0.372527558 0.403149145 0.442128821 0.409293178
0.268275071 0.500565357 0.290318619 0.469015817 0.474493102
0.431601502 0.380715427 0.465082496 0.366922142 0.378699777
0.476503096 0.186418023 0.542265937 0.421427083 0.360325642
0.289506087 0.153993662 0.1199590240.306261385 0.578617507
0.408377582 0.582319538 0.500443803 0.694847351 0.514692026
0.423558457 0.186578303 0.266027181 0.412299675 0.6110328910.42577228
0.503408558 0.406128449 0.42466748 0.313162056 0.399647596 0.30960389
0.303061328 0.315921421 0.131731215 0.402945028 0.214773576 -
0.046158324 0.22266573 -0.180451937 0.008865393
TCGA-69-7974-01A 0.532539079 0.432036544 0.47582342 0.420668852
0.229673351 0.552653398 0.4376356110.448958335 0.420445575
0.537547578 0.408327028 0.476141522 0.351994014 0.362680452
0.47493841 0.198299896 0.5541189370.543621565 0.496710776 0.350139327
0.19144956 0.131915508 0.384670396 0.522989534 0.421421299
0.564613331 0.441273956 0.670199652 0.464274616 0.433088461
0.349291671 0.305044805 0.4117179830.58518564 0.427778773 0.554650702
0.45418499 0.427550476 0.341992658 0.456580917 0.327006656
0.334423091 0.378063017 0.153842536 0.385774595 0.372907031 -
0.064566762 0.311730566-0.163449979 -0.060037887
TCGA-69-7978-01A 0.521509187 0.449682447 0.430573313 0.395777711
0.269427909 0.550123904 0.473355323 0.440218595 0.396085321
0.510191085 0.433955154 0.449195459 0.329004216 0.331948761
0.4565742110.188315629 0.551368406 0.557962258 0.532654476
0.366919371 0.251386335 0.158468526 0.424920158 0.555842269
0.436461298 0.557679575 0.402330946 0.662566969 0.441397703
0.527209797 0.368423305 0.3011125370.373149816 0.550607642
0.4401196690.556703481 0.437778019 0.424542461 0.378250141
0.470900882 0.327961422 0.361224676 0.400542037 0.121129091

0.358782751 0.432506234 -0.081661716 0.331518883 -0.17921116 -
0.06353287
TCGA-69-7979-01A 0.367837264 0.379145741 0.532125641 0.375031335
0.266367689 0.464923669 0.262283886 0.476316598 0.421029278
0.444157952 0.403583966 0.436449108 0.352026209 0.337380936
0.435697733 0.221005707 0.493221559 0.383895886 0.341001976
0.324418755 0.163276622 0.1136608820.275291218 0.547646722
0.395204886 0.549854395 0.438775507 0.675709235 0.496696599
0.452655076 0.171282223 0.284532152 0.404706141 0.604654253
0.429469582 0.577838823 0.406448858 0.425687773 0.312394392
0.377107385 0.307138907 0.293252424 0.268677615 0.151618306
0.375623279 0.206125187 -0.04697913 0.154691319 -0.130224682 -0.1586373
TCGA-69-7980-01A 0.482319517 0.385782966 0.4129311320.364890705
0.230719901 0.552842716 0.366084062 0.458012565 0.378202643
0.487147763 0.4119784290.439468572 0.324857776 0.341407676
0.420936439 0.210918269 0.515186635 0.563391396 0.485163114
0.339445294 0.173621206 0.1421195040.365192843 0.520694904
0.429670633 0.499975836 0.38826023 0.659035352 0.429343959
0.454021885 0.264610208 0.285556575 0.371467203 0.581643736
0.403980829 0.5120911830.430285382 0.420413636 0.333721381
0.3773840110.306851024 0.340635169 0.329560519 0.1110380240.371268311
0.318360524 -0.115375087 0.274140361 -0.141528815 -0.080638533
TCGA-69-8253-01A 0.437984037 0.420381446 0.506043779 0.313299241
0.2911326090.508130418 0.30616907 0.46384327 0.284174719 0.467216665
0.3927114060.493645324 0.353391965 0.368685259 0.449630757
0.190492323 0.529752362 0.400516933 0.356842091 0.298976445
0.162792443 0.1176131330.326520659 0.548477253 0.380463655
0.539440099 0.297135883 0.6605511470.489830421 0.371376732 0.22026092
0.322999784 0.437747887 0.636074392 0.432260864 0.599247126
0.455303978 0.422727548 0.3311569290.410685089 0.33722576 0.323540213
0.322149045 0.152687486 0.382774469 0.235221245 -0.139362588
0.232945369 -0.126871181 -0.06914579
TCGA-69-8254-01A 0.528513648 0.406221761 0.481844248 0.320960834

0.253824612 0.531543193 0.40246806 0.444520047 0.278828135
0.499809395 0.419502694 0.471790587 0.378343895 0.383823103
0.5030811630.177388179 0.544092414 0.538878149 0.475072388
0.326882504 0.185384734 0.146797498 0.379216848 0.502338409
0.39307105 0.52011008 0.2834011040.621536333 0.413204645 0.378721857
0.302940053 0.344817109 0.445552856 0.602745162 0.419075005
0.553048287 0.494339435 0.427829537 0.343710621 0.383146025
0.330717891 0.353301826 0.374409032 0.208091249 0.411294689
0.336255508 -0.127132603 0.320668893 -0.086006954 -0.055819683
TCGA-69-8255-01A 0.389943933 0.349736427 0.516056232 0.380804666
0.19183003 0.388357328 0.352849859 0.525200756 0.5011556880.449846982
0.282622887 0.483221019 0.229487366 0.310513669 0.41581401
0.1193026740.505496595 0.516999425 0.470626726 0.232105841
0.122857633 0.04376196 0.369291938 0.604285584 0.42878937 0.622123489
0.554645476 0.735298086 0.61790817 0.263236795 0.233914065
0.414564821 0.451629546 0.663905476 0.422306598 0.578532342
0.38606665 0.459466037 0.219440416 0.282362702 0.278099628
0.367591067 0.333458515 0.246731328 0.404613633 0.376981067
0.002242103 0.213873419 -0.182506715 -0.087658508
TCGA-69-8453-01A 0.560538903 0.419928348 0.526801008 0.346969543
0.238341239 0.532021572 0.468360016 0.45537185 0.334252001
0.517955931 0.408189341 0.496693272 0.390450008 0.428705833
0.478962039 0.231684359 0.513163506 0.569762645 0.504286807
0.344918538 0.234728548 0.140088534 0.418198126 0.515363133
0.406528803 0.537149142 0.338974331 0.64967968 0.435775917
0.435247463 0.358371221 0.345293449 0.433962184 0.602498649
0.399185717 0.559196873 0.4944191130.437727325 0.345138338
0.409542357 0.323539286 0.372749678 0.397781447 0.182112731
0.379933921 0.387527303 -0.118735317 0.35909163 -0.106700293 -
0.034096377
TCGA-69-A59K-01A 0.430149513 0.40218249 0.416800475 0.357186472
0.21387335 0.478051004 0.340835813 0.463368834 0.407686435
0.482824525 0.409299686 0.455035386 0.308644644 0.320758013

0.408098456 0.183742669 0.542940039 0.457412669 0.415996816
0.30642164 0.169425385 0.1132021340.327934905 0.556698935 0.423258264
0.571895006 0.447348364 0.676975284 0.472410859 0.386053957
0.236874832 0.273628395 0.374452435 0.59254605 0.432872836
0.577537306 0.413819437 0.439184202 0.287169958 0.353466006
0.318570819 0.264760772 0.313483581 0.149551287 0.3675699
0.309309499 -0.061477887 0.23660728 -0.165300586 -0.121900316
TCGA-71-6725-01A 0.380166024 0.32368593 0.470753901 0.329575399
0.228329996 0.470369939 0.291440149 0.464827507 0.336940503
0.443009543 0.426439064 0.451820997 0.327039991 0.306099801
0.443248166 0.1665931170.538277243 0.431092644 0.374500757
0.304809967 0.165644449 0.145283314 0.278530506 0.550095638
0.396493336 0.527556491 0.354428177 0.643625275 0.463041481
0.292053925 0.191034998 0.269141842 0.381701526 0.601871324
0.37238701 0.524103191 0.404785896 0.398301276 0.315648181
0.345590242 0.325190555 0.224378499 0.251265955 0.169692395
0.382334234 0.211313533-0.090482266 0.156192834 -0.165517964 -
0.104678694
TCGA-71-8520-01A 0.438991897 0.426740538 0.500824754 0.382858482
0.2551661190.534178204 0.31940757 0.444698104 0.384540622 0.483394611
0.436109538 0.472529338 0.336884894 0.345051266 0.44254637
0.219622713 0.547138974 0.559676076 0.463797586 0.355586181
0.201774923 0.233701937 0.347943335 0.509139878 0.408663209
0.546356747 0.412849924 0.6466991190.426906468 0.542058561
0.261380908 0.292027563 0.410368529 0.569883078 0.460055445
0.517405047 0.4392136 0.420558891 0.331770519 0.459269943 0.32204357
0.371552345 0.329202957 0.171498979 0.387438761 0.25833033 -
0.087242445 0.283213873 -0.136078281 -0.084930915
TCGA-73-4658-01A 0.566799825 0.45653017 0.4750701170.330659652 0.270397457
0.555923453 0.4381161180.450281459 0.332990921 0.5257891140.464065654
0.484441401 0.348503237 0.349362576 0.499325336 0.2421211750.54379222
0.463993655 0.460979299 0.379673191 0.204504641 0.183524454
0.403793665 0.528500916 0.416783597 0.550815818 0.353165241

0.660408996 0.443289369 0.564685307 0.370802294 0.32023505
0.410394408 0.613395754 0.439649316 0.560674876 0.465164834
0.447590457 0.37219587 0.4981536 0.338846123 0.384538835 0.409149271
0.166943362 0.3854561120.394099041 -0.126307828 0.35556367 -0.172697256
-0.070704665
TCGA-73-4659-01A 0.449677301 0.423330088 0.501468294 0.356467724
0.249261093 0.509735608 0.330105377 0.468554428 0.411093257
0.465337658 0.394470062 0.503935249 0.373241974 0.400483433
0.490159101 0.207386137 0.543082712 0.394476596 0.372202287
0.315663186 0.16464057 0.129757952 0.351256623 0.559364927
0.4112621350.591878172 0.43155767 0.68314401 0.4971187580.460711353
0.263476181 0.33651045 0.439272457 0.629978034 0.435728721 0.57329016
0.446887693 0.446720049 0.322710172 0.44727215 0.340998222
0.362289173 0.338483533 0.179593769 0.4032731150.256011147-0.06820676
0.273953783 -0.168002924 -0.032851218
TCGA-73-4662-01A 0.459916088 0.37224996 0.490100967 0.351768488
0.236319632 0.478447206 0.348615285 0.459006483 0.331608898
0.487587147 0.418259912 0.474866527 0.346520527 0.349847504
0.448293222 0.191385144 0.549943301 0.475094872 0.426271661
0.332540889 0.151438424 0.131923736 0.355858557 0.516008397
0.43129694 0.521648319 0.359600817 0.65776772 0.424702016 0.403628946
0.273160729 0.299762912 0.410451405 0.613914461 0.386974259
0.515151909 0.440707601 0.416968794 0.347616816 0.375935122
0.326308273 0.302981262 0.3471903 0.191931724 0.387321617
0.345126072 -0.121287251 0.263957229 -0.182352895 -0.079245302
TCGA-73-4666-01A 0.46120128 0.399929089 0.478769322 0.412413213 0.23144623
0.459984756 0.389581005 0.514134613 0.509904388 0.488066139
0.392167191 0.459404027 0.316854408 0.352872759 0.432080828
0.151498212 0.527165037 0.557839151 0.513967258 0.326306489
0.2111348130.121472976 0.396403329 0.54348576 0.446783172 0.605802837
0.570506292 0.713193497 0.559161738 0.379580742 0.299617965
0.314671076 0.419694732 0.618881409 0.448124841 0.584106219
0.432660064 0.42354825 0.300127141 0.298718199 0.313894932

0.289499371 0.346548697 0.147018756 0.370875023 0.398824699 -
0.049731294 0.258370092 -0.174177852 -0.107496711
TCGA-73-4668-01A 0.464020734 0.443232267 0.49225048 0.388125779
0.267228403 0.557393325 0.323017207 0.469280832 0.410349986
0.4940826110.422125264 0.464547072 0.361716476 0.35622511 0.479939541
0.2113814860.552391406 0.352108754 0.329871666 0.341895159
0.194080468 0.137694725 0.3429112110.557229144 0.412734136
0.557077252 0.424751485 0.691884074 0.500014255 0.502362133
0.248358044 0.295210784 0.395024345 0.601025462 0.457486399
0.5302119780.44423689 0.427494263 0.376198571 0.436966633 0.324147366
0.339880442 0.337655058 0.148857285 0.370212176 0.246424424 -
0.084907263 0.264729574 -0.145637321 -0.095136198
TCGA-73-4670-01A 0.480971912 0.465055255 0.4511567090.372329742
0.247537495 0.504276528 0.324259551 0.484662758 0.468201682
0.470277154 0.366838753 0.490028783 0.350245005 0.37818483
0.4655911780.181005727 0.55258697 0.391323424 0.360737337 0.295329156
0.199651204 0.130059154 0.328098553 0.574990155 0.411593926
0.595437318 0.494415301 0.71264358 0.537008677 0.486228338
0.248992204 0.325244937 0.4177821150.658299378 0.4777244110.577687481
0.433761553 0.457097642 0.310627599 0.415187616 0.309210979
0.296806168 0.298022493 0.121726272 0.379792155 0.232992295 -
0.058657625 0.250187291 -0.189117709 -0.132889588
TCGA-73-4675-01A 0.397535149 0.4219163110.467628851 0.301991455
0.303066595 0.492526278 0.291847862 0.459535002 0.287122662
0.450463664 0.410915095 0.506512649 0.332072783 0.351714595
0.422780432 0.232518821 0.541048919 0.366468065 0.332245969
0.317799725 0.153891562 0.184731218 0.307331756 0.525798268
0.38709176 0.520425413 0.320396059 0.640505192 0.4500119790.456752002
0.213744442 0.33498289 0.442764769 0.623175405 0.450333032
0.577533562 0.463173873 0.426646555 0.302268335 0.425783873
0.336974586 0.306053345 0.29418471 0.155533863 0.369785996
0.245557331 -0.152159749 0.243416587 -0.12500353 -0.07592046
TCGA-73-4676-01A 0.471277486 0.436904623 0.501389839 0.388014725

0.22341093 0.513651301 0.325727821 0.480560684 0.45364932 0.482975908
0.372135866 0.495707059 0.341232964 0.349585663 0.470697314
0.17605417 0.565740443 0.410410445 0.381867409 0.332004301
0.196504217 0.139308614 0.347352313 0.546407251 0.404039912
0.598598705 0.50810051 0.716813601 0.5119089510.424277719 0.25011777
0.339707593 0.451581875 0.64438332 0.476821314 0.585618714
0.434478432 0.434428013 0.32182698 0.400181438 0.333106223
0.315874091 0.339050898 0.19762098 0.410565474 0.275869925 -
0.058803128 0.275681313 -0.172670447 -0.098892523
TCGA-73-4677-01A 0.423723391 0.38942771 0.507271409 0.30178516 0.267549068
0.48849924 0.3119541450.44810381 0.27379534 0.468202329 0.390974959
0.509418827 0.369364866 0.36625031 0.48021158 0.16946561 0.564732053
0.394784128 0.357931972 0.292252178 0.148022793 0.126716796
0.336666923 0.521983844 0.39779915 0.520026909 0.288141265
0.640375952 0.413604337 0.3379691120.24033863 0.339730925 0.474118776
0.639767707 0.400653787 0.585878918 0.456569206 0.420326361
0.341269043 0.396014518 0.357583638 0.319268206 0.318441749
0.197716815 0.398465503 0.274290285 -0.133637736 0.230287858 -
0.121453342 0.002369497
TCGA-73-7498-01A 0.358436767 0.348288362 0.453958235 0.317431623
0.323023434 0.513809632 0.319669733 0.437906148 0.244973719
0.462929581 0.448837693 0.500435172 0.335923848 0.331927133
0.442644338 0.23196485 0.519416487 0.403338592 0.354939048
0.344194475 0.142299241 0.197567578 0.314946487 0.487943661
0.398717683 0.47838415 0.255573146 0.620261737 0.389222328 0.38942206
0.197933575 0.320921747 0.446926696 0.609937778 0.379576753
0.538046255 0.4347123110.393753027 0.354935643 0.407373806 0.32660143
0.317813488 0.313605299 0.17038177 0.375617693 0.264842009 -
0.152709287 0.218999095 -0.150261621 -0.031111859
TCGA-73-7499-01A 0.39377984 0.357038761 0.485496666 0.303918961
0.173728767 0.413445385 0.361845413 0.502826604 0.447505168
0.471234751 0.331765562 0.462819752 0.279522343 0.33586184
0.435270778 0.209966785 0.505745936 0.558410644 0.494811375

0.268560979 0.145659513 0.05162871 0.367356198 0.535400407
0.418303956 0.576120648 0.510331862 0.706629683 0.457874548
0.388575034 0.250687076 0.299628376 0.444710449 0.653238372
0.424710463 0.532009175 0.420742222 0.43598067 0.254372747
0.336806498 0.29677661 0.281063335 0.317516198 0.166446663
0.415226278 0.38699744 -0.038817361 0.241846508 -0.144691374 -
0.094707756
TCGA-73-A9RS-01A 0.362662144 0.359780464 0.506166329 0.392763096
0.165437493 0.398467954 0.2437248 0.500915591 0.48574554 0.414423145
0.320926026 0.459258357 0.305645845 0.337489477 0.458058766
0.136671495 0.543395944 0.4301977110.35244585 0.208430477 0.095013951
0.012525342 0.2711939650.594679701 0.422315104 0.596148463 0.51608706
0.704565625 0.508197687 0.262419401 0.131366643 0.262167926
0.402186464 0.656108429 0.413077977 0.547904936 0.393190213
0.415791845 0.25861226 0.227612293 0.285927463 0.2311940370.236792627
0.137233761 0.377015175 0.189646876 0.004393183 0.132227489 -
0.196255277 -0.173598994
TCGA-75-5125-01A 0.451231929 0.40736326 0.426168252 0.419022441
0.240577097 0.478024473 0.420724863 0.494224927 0.485239363
0.497008489 0.343056098 0.464875905 0.296722838 0.332182307
0.428467778 0.168731803 0.567646127 0.545124275 0.494336818
0.326752414 0.212820122 0.095504259 0.373540815 0.55041287
0.435545345 0.576500934 0.517183432 0.716589181 0.513367457
0.429423488 0.31933218 0.267172027 0.401763086 0.606650565
0.438281393 0.557858477 0.406906483 0.427191372 0.310975232
0.387417999 0.323746341 0.295559726 0.359241626 0.135672973
0.387487837 0.388923775 -0.039421692 0.270259998 -0.169808939 -
0.095303882
TCGA-75-5146-01A 0.397509683 0.374776849 0.500717443 0.261486398
0.199529353 0.5051121690.338412732 0.4697324 0.281666004 0.473501517
0.345160046 0.5021102850.335739519 0.366053273 0.475921788
0.180297855 0.538570047 0.441203776 0.391377929 0.271938682
0.1101700490.068587814 0.331398484 0.533392936 0.414682086

0.534245287 0.335632848 0.657129012 0.406518327 0.342667839
0.240579877 0.319976948 0.4511818060.648883704 0.398601803
0.518043565 0.433733519 0.42514646 0.302482146 0.31221833 0.341863727
0.288297158 0.313715958 0.184864233 0.403782108 0.310797911-
0.101995021 0.24413651 -0.150851536 -0.05549627
TCGA-75-5147-01A 0.435921767 0.39330329 0.502375768 0.301685281
0.173128658 0.464306105 0.371275858 0.5110184990.41422356 0.480055702
0.330096936 0.475779034 0.284302001 0.355124258 0.456311569
0.165432549 0.520829382 0.588737583 0.502445421 0.2865685
0.129172944 0.059154815 0.369243774 0.550899879 0.419985679
0.58355145 0.4891198430.713987815 0.487715966 0.43793886 0.292084615
0.314351699 0.446064098 0.656965008 0.445146586 0.509948865
0.434638593 0.449623431 0.270449948 0.36879873 0.299340321 0.34012957
0.333012541 0.199018076 0.435155133 0.342722942 -0.031650134
0.302235174 -0.160431505 -0.015492519
TCGA-75-6206-01A 0.439743039 0.3916011080.448382751 0.323250433
0.276627725 0.562326555 0.322610095 0.432131555 0.249840661
0.463165392 0.39001602 0.482728726 0.356769563 0.327682351
0.446596369 0.213932833 0.558505399 0.451645819 0.396614123
0.3141111740.13806462 0.188642095 0.329983294 0.523685681 0.411000623
0.478731523 0.264223065 0.635315468 0.428681633 0.400441921
0.254566558 0.341216626 0.428144826 0.596487245 0.377540973
0.533793803 0.447329995 0.419330712 0.361718467 0.433718983
0.337566845 0.326965141 0.317167282 0.164517046 0.359347111
0.274201121-0.138083884 0.237453432 -0.152851549 -0.010757299
TCGA-75-6212-01A 0.448780922 0.446549717 0.4651135250.326647226 0.2297418
0.491820571 0.37141453 0.456664355 0.320457865 0.505675107
0.423380416 0.519844735 0.335928775 0.354272478 0.48558517
0.203795291 0.562219147 0.465695601 0.417935467 0.320460987
0.181321714 0.165207155 0.3743882110.508609366 0.412197345
0.555801242 0.33969326 0.65977879 0.459061252 0.414768949 0.297359255
0.333686814 0.463612864 0.640171637 0.455046612 0.563867819
0.479841625 0.420483408 0.335890259 0.435319505 0.350872847

0.355279068 0.365037199 0.2027903110.402760929 0.348699527 -
0.100321948 0.304126188 -0.138071543 -0.04786291
TCGA-75-6214-01A 0.420223953 0.388251568 0.419367065 0.406214509
0.282180829 0.514074268 0.283315581 0.476628342 0.467563455
0.479080388 0.41163234 0.438672523 0.333120396 0.369913386
0.426921564 0.153757706 0.540676891 0.468906187 0.389759902
0.329715353 0.2116659110.077280028 0.320815714 0.53016004 0.421147507
0.543840232 0.482197781 0.692594446 0.515922069 0.398985237
0.197486781 0.26817393 0.389939027 0.597282488 0.436381931
0.519393976 0.464105972 0.40888417 0.319738938 0.351940408
0.300550014 0.271623675 0.292385764 0.123164278 0.373453145
0.200906104 -0.045111409 0.214402922 -0.087901735 -0.097850374
TCGA-75-7025-01A 0.439458465 0.374254576 0.402564608 0.31061494
0.274319632 0.541955657 0.367439784 0.439094519 0.23141124
0.495510245 0.422021614 0.452471901 0.321466556 0.33086394
0.450258402 0.241260206 0.537475198 0.509743163 0.449860793
0.338708533 0.172254467 0.1911886230.347605713 0.49125106 0.39120439
0.470400438 0.25587211 0.62251756 0.356147003 0.475347428 0.244522433
0.315879122 0.4113796290.583676357 0.3811072460.516122462 0.470017772
0.404330159 0.351767072 0.398565898 0.320976573 0.32968225
0.328182858 0.151925204 0.381049985 0.32961482 -0.12304792 0.279946071
-0.134978099 -0.069759933
TCGA-75-7027-01A 0.4481137760.423040032 0.465958677 0.42409457 0.312279587
0.534980076 0.263703454 0.466084862 0.477456288 0.466384186
0.412989395 0.472490563 0.338351769 0.358509107 0.474547701
0.198353292 0.546807025 0.359453505 0.336795351 0.325559466
0.162809359 0.192775281 0.322365603 0.563812862 0.417199798
0.605489141 0.483602577 0.709494777 0.506910791 0.533429311
0.197626192 0.321971041 0.42437065 0.63189277 0.446237916 0.583383317
0.439134258 0.430465373 0.321884595 0.418930274 0.31240506
0.313306045 0.3151389 0.139715325 0.383021287 0.218337276 -
0.081690918 0.260954161 -0.180952202 -0.06887241
TCGA-78-7143-01A 0.429944076 0.372538751 0.455155659 0.398698728

0.221472541 0.508869161 0.321604359 0.485501351 0.442891894
0.498781238 0.4151169730.454580673 0.333145389 0.354692632
0.460595751 0.166813615 0.548281032 0.571077904 0.466362062
0.295815355 0.151015026 0.105538375 0.337068201 0.539829677
0.433294041 0.552506781 0.493302522 0.691644713 0.446000456
0.338344232 0.256858406 0.283435125 0.412297992 0.595111775
0.433382268 0.522534158 0.4541114090.43984372 0.32578047 0.297258592
0.33360323 0.287768652 0.331778309 0.15472108 0.3931183930.295857453 -
0.047373858 0.254253389 -0.154395328 -0.11149106
TCGA-78-7145-01A 0.489167136 0.435752318 0.4876118 0.392753293
0.275506899 0.556447084 0.330484749 0.472539793 0.448241317
0.497769061 0.397369954 0.48989502 0.313437882 0.351280924
0.488653083 0.19779913 0.575826864 0.500064268 0.419372018
0.320864053 0.164700049 0.1811597130.362059679 0.563412908
0.418899178 0.579319787 0.469751664 0.703232141 0.503567286
0.474834243 0.286187276 0.307883368 0.42095038 0.617031335
0.469750739 0.540399538 0.439673682 0.443737223 0.3627331
0.4458751150.330197487 0.315883198 0.343838926 0.152230374
0.412901001 0.274927807 -0.042989835 0.306216971 -0.183981858 -
0.077522325
TCGA-78-7146-01A 0.469464261 0.434884266 0.457805976 0.424198436
0.235709461 0.53861542 0.331857332 0.469237928 0.5113308780.479129491
0.342609786 0.455327364 0.329447688 0.339123712 0.460696248
0.18169862 0.566977127 0.518369333 0.432390707 0.32394167 0.182497736
0.1178819640.338206163 0.585615659 0.435241859 0.593263045
0.537202418 0.72841036 0.564731216 0.394481788 0.2540702 0.275712289
0.3911247890.586071599 0.462434668 0.569138832 0.428983207 0.44728134
0.321069531 0.3768112020.328916316 0.263078163 0.323906413
0.122759343 0.359256523 0.28361463 -0.039310188 0.217473142 -
0.189671357 -0.105850597
TCGA-78-7147-01A 0.356499639 0.360524564 0.444259047 0.34784294
0.193753779 0.453479176 0.303415577 0.495273813 0.411808937
0.453435467 0.373776501 0.464741807 0.317994867 0.348119591

0.4284801140.1811650320.517368393 0.530915238 0.440792889 0.275621665
0.156774091 0.093139964 0.316834199 0.541675393 0.413336618
0.563216281 0.463754508 0.688953207 0.452740044 0.324262972
0.194266273 0.30023541 0.423576786 0.618180661 0.443249108
0.538989521 0.432742496 0.422334678 0.263381898 0.289518694
0.3114105280.274187207 0.281445635 0.162880139 0.408759228
0.272116287-0.057319226 0.199987469 -0.149554024 -0.104983126
TCGA-78-7148-01A 0.415905083 0.422256678 0.480499705 0.36825722 0.2597137
0.519423001 0.318719422 0.467473926 0.412183128 0.482700446
0.405927495 0.492639461 0.371053779 0.38003044 0.500090353
0.189697441 0.56353405 0.47783166 0.41304965 0.299424573 0.166448524
0.138303377 0.345278824 0.5411955220.4173826110.566499895 0.427093704
0.68785582 0.465870442 0.380858764 0.22508536 0.349576897 0.465043872
0.629810899 0.46709725 0.5835113970.436673182 0.414740022 0.333350944
0.38012007 0.337585806 0.326794249 0.328087454 0.1804843 0.391875977
0.28374009 -0.095678068 0.254188051 -0.139480691 -0.022493302
TCGA-78-7149-01A 0.357775645 0.370132178 0.430754706 0.297681455
0.327198437 0.491641201 0.26193115 0.461526486 0.270968799
0.440469294 0.390278156 0.514325308 0.323470561 0.342190947
0.404698609 0.222179781 0.5110279 0.373351537 0.319905632
0.287904881 0.1190681690.131996989 0.292350562 0.520768475
0.383208445 0.498225514 0.296746578 0.647721905 0.47480197
0.385145152 0.15043726 0.337177965 0.470594007 0.642779834
0.399543175 0.559885681 0.455225895 0.402027089 0.31272351
0.417272697 0.333332997 0.333171057 0.293514881 0.151274122
0.38984561 0.215284846 -0.125738132 0.202354071 -0.128410044 -
0.058856629
TCGA-78-7150-01A 0.431756174 0.44983829 0.516198277 0.404424456
0.252934974 0.533541042 0.294309756 0.472325138 0.479710764
0.48353681 0.397046109 0.489052494 0.3640725 0.396382798 0.470775937
0.18507786 0.57253371 0.489456566 0.405957832 0.3011665470.202838977
0.100797413 0.336704766 0.572629318 0.429157026 0.60621584
0.495104773 0.710767923 0.514832961 0.515735443 0.20931444

0.354985135 0.443681064 0.639083496 0.475555909 0.580893015
0.419069199 0.425905508 0.366103605 0.382920918 0.30306021
0.328509572 0.306145189 0.151843103 0.395630582 0.216596862 -
0.019620252 0.233319376 -0.148193033 -0.058288214
TCGA-78-7152-01A 0.425492608 0.376060714 0.472502595 0.341451663
0.277254523 0.507176635 0.341936778 0.469957737 0.337375136
0.479857081 0.356426659 0.492656698 0.355862343 0.37431685
0.437243448 0.184033335 0.523547325 0.48277568 0.429548795 0.29588374
0.130559523 0.122829073 0.344595961 0.539553941 0.424772607
0.52715679 0.3677117970.677650655 0.492855904 0.363498163 0.249102933
0.30058925 0.416348507 0.632205796 0.396970173 0.546826063
0.463490091 0.431535446 0.319670515 0.371214799 0.32609504
0.305087015 0.326379937 0.149768134 0.385187965 0.326437018 -
0.126501092 0.236245017 -0.14772901 -0.061725037
TCGA-78-7153-01A 0.3470971160.369646447 0.458751495 0.317250066 0.26653516
0.486529252 0.247610707 0.480670244 0.371968504 0.431786344
0.369852864 0.490207921 0.334773159 0.360215076 0.461677784
0.183427645 0.5223981110.390838253 0.324420269 0.262567986
0.092531785 0.131358816 0.2901211730.546398946 0.391348518
0.533824612 0.387610474 0.695561974 0.548379709 0.35300723
0.163961261 0.321914142 0.472643269 0.661717681 0.412054956
0.545400537 0.43344117 0.4360521120.29315699 0.376770841 0.316013853
0.307158527 0.273463392 0.199453075 0.405696092 0.228367407 -
0.098849348 0.197998504 -0.195264672 -0.012104713
TCGA-78-7154-01A 0.430874241 0.405844336 0.532893679 0.390632048
0.210078133 0.476701088 0.309502875 0.472854441 0.48905469
0.473022986 0.402868344 0.480713685 0.378722769 0.382757901
0.486403943 0.156591385 0.561001566 0.410661868 0.363712589
0.300708193 0.194901596 0.231377982 0.33707909 0.580849705
0.407538558 0.644015769 0.520134154 0.72746175 0.552163407
0.430688948 0.224282307 0.348319539 0.452053616 0.647518713
0.466287458 0.585175718 0.421523895 0.444946453 0.32580956
0.435149255 0.331818799 0.312156888 0.326551563 0.169687622

0.40346895 0.241869351 -0.031996391 0.256427497 -0.169025694 -
0.02549741
TCGA-78-7155-01A 0.22561145 0.322973291 0.431865865 0.4704381 0.313197132
0.4655927 0.184331084 0.492439457 0.563997798 0.373332903
0.369636574 0.423830907 0.205648239 0.213018319 0.420510636
0.107283513 0.568239627 0.195374648 0.189861687 0.265016656
0.08814634 0.166839205 0.229015193 0.555392443 0.390760876
0.575651974 0.614551638 0.750231519 0.552914667 0.290974678
0.088988369 0.173452945 0.349181336 0.592604814 0.405241666
0.407656468 0.3112481980.386992517 0.31518385 0.270905155 0.254888152
0.14183932 0.230840074 0.082322158 0.342039138 0.158845072
0.080698933 0.105952034 -0.165718651 -0.12429047
TCGA-78-7156-01A 0.331388263 0.335014634 0.439476461 0.316359641
0.29951669 0.485025835 0.242063889 0.468146177 0.27710648 0.440137734
0.382408093 0.506194665 0.320254739 0.307151515 0.434325806
0.173917567 0.565991817 0.397675567 0.331440321 0.261626469
0.0771176420.121974882 0.2861930110.500240053 0.406091787 0.485470699
0.299349996 0.65928178 0.392522048 0.249216993 0.136931642
0.310389072 0.505033266 0.659142764 0.355959569 0.565090526
0.415693603 0.386678093 0.32553739 0.307226306 0.337300942 0.27520069
0.256292213 0.197386918 0.368573381 0.185642681 -0.110403285
0.151381722 -0.149291347 0.012606639
TCGA-78-7158-01A 0.347019664 0.309461531 0.43232848 0.333025158 0.32457943
0.480375504 0.250480252 0.482755535 0.372261426 0.436874434
0.415508676 0.470704434 0.296228729 0.3311284020.458341837
0.151424218 0.527223997 0.416083064 0.332708222 0.286116217
0.1231147730.132540103 0.27871869 0.519938144 0.383657992 0.513993947
0.397601961 0.704754351 0.533198129 0.337087136 0.171328746
0.285780347 0.43738685 0.626218064 0.382876478 0.512451805 0.42531371
0.400836828 0.2898011840.401080824 0.301582638 0.279806746
0.290959643 0.147617609 0.402871279 0.226877251 -0.107517512
0.19730547 -0.176235868 -0.051529253
TCGA-78-7159-01A 0.42499256 0.406746505 0.451686385 0.374327927 0.27047072

0.518218967 0.364774981 0.47901123 0.437629128 0.442785774
0.367761247 0.463785396 0.325921313 0.34972739 0.457372682
0.174799487 0.55194007 0.457553746 0.4041511590.291475122 0.13628385
0.137518077 0.333304591 0.567063149 0.418802504 0.577285359
0.459724017 0.696473089 0.467694867 0.438244724 0.230935248
0.256034549 0.394013631 0.619744991 0.42931039 0.518023081
0.412032432 0.42753891 0.316998504 0.444420579 0.310838141
0.307709234 0.3169211240.130597982 0.378075813 0.278948213 -
0.049728138 0.266503796 -0.179257182 -0.060219727
TCGA-78-7160-01A 0.503667002 0.416514634 0.4704182 0.381834034
0.274922648 0.560724197 0.380679845 0.4247611510.391785669
0.499286679 0.407382615 0.498666508 0.374919498 0.379222426
0.515284483 0.205349212 0.571017696 0.494569412 0.471611585
0.337893594 0.190037462 0.162930081 0.391772747 0.527402284
0.410036776 0.558144139 0.386973513 0.672274416 0.456081088
0.472434436 0.316254223 0.35175109 0.45771202 0.614509075 0.423575614
0.590542777 0.44793475 0.427148229 0.3804561190.455645904 0.337404377
0.350961824 0.372840791 0.176661977 0.396281683 0.370255775 -
0.1108375070.312264327 -0.158446405 -0.056276403
TCGA-78-7161-01A 0.425423867 0.387924217 0.460539944 0.317108382
0.281003234 0.553362858 0.263971214 0.444324369 0.305094276
0.431635306 0.378322694 0.457252419 0.33056277 0.335776458
0.464943697 0.193241204 0.529128427 0.430126907 0.365491095
0.286062859 0.1144318970.157725685 0.301812062 0.527730727
0.382362962 0.501756791 0.308493144 0.655804289 0.434484183
0.421888184 0.169098772 0.2861124590.39868679 0.600661762 0.396496828
0.484627197 0.409263399 0.403661493 0.338167174 0.429675664
0.309865336 0.336835977 0.297108784 0.146409446 0.371080975
0.162491603 -0.104026213 0.201431194-0.167551733 -0.064268872
TCGA-78-7162-01A 0.414282299 0.382512723 0.484845515 0.326352752
0.279694366 0.541026682 0.354673974 0.44566033 0.296492841 0.48303619
0.426160671 0.508223395 0.359439988 0.3678809110.483902559
0.229236156 0.555124806 0.454160624 0.412625089 0.342142158

0.200915032	0.161698058	0.35886557 0.517763243	0.421942804
0.529245069	0.315666748	0.653245366	0.426949157	0.38936627
0.258868228	0.351586823	0.477129631	0.623621438	0.379367615
0.578205915	0.445795779	0.419576172	0.374977609	0.401381259
0.351000265	0.328497157	0.33979772 0.194414795	0.393099005
0.324040697	-0.093794441	0.257064919	-0.132172611	-0.001607016
TCGA-78-7163-01A 0.363768363	0.384756734	0.451322352	0.294099319
0.196165929	0.456305218	0.257354518	0.468563722	0.273545072
0.427481675	0.37203977 0.468492365	0.325459374	0.310895984
0.505255947	0.109874926	0.564817506	0.4329235110.383377236
0.258695017	0.156195788	0.1136767970.272665401	0.545382038
0.416257206	0.543889847	0.305067235	0.64421326 0.424931269
0.202488176	0.163786525	0.312655178	0.445160393	0.645959732
0.454305591	0.547350518	0.454416452	0.413349448	0.268245744
0.231912887	0.347746486	0.210889267	0.2841615110.168007702
0.375827002	0.208840257	-0.092508529	0.189149014	-0.167260207	-
0.093254774
TCGA-78-7166-01A 0.438985188	0.423454922	0.474046202	0.347984905
0.284080565	0.469121491	0.307329671	0.500899218	0.415503651
0.458954857	0.37582923 0.470920212	0.324482031	0.351838249
0.417104731	0.169640568	0.509981263	0.446345092	0.384668782
0.259867088	0.152219649	0.07725704 0.3271081190.528560121	0.393353266
0.547807676	0.44001075 0.691889643	0.514755132	0.356827822
0.177848532	0.339720995	0.457031655	0.648989065	0.465014582
0.610392196	0.454518026	0.409306421	0.270658586	0.300111026
0.309472391	0.29835314 0.301023429	0.157839659	0.412633837
0.248188174	-0.088956626	0.206599936	-0.134072983	-0.12151329
TCGA-78-7167-01A 0.321550598	0.3391150560.406273647	0.313306175
0.3087112550.534678873	0.224223846	0.456594414	0.248520518
0.427660161	0.428957351	0.488490431	0.301403108	0.302679881
0.415065747	0.183341401	0.5411746480.355210254	0.280806013
0.279315576	0.107599919	0.141532549	0.280215181	0.49448205
0.392243154	0.466039518	0.261703358	0.642354766	0.401495764

0.345865057 0.107213245 0.29448621 0.452413652 0.61836712 0.376480329
0.534648571 0.422796819 0.375982591 0.330593854 0.364887651
0.334906394 0.284758592 0.2614414 0.131093749 0.371278128 0.16663694
-0.136850079 0.163025589 -0.145681118 -0.038993948
TCGA-78-7220-01A 0.3777471180.390318845 0.464639019 0.423391696
0.251747395 0.502815584 0.269296521 0.483165538 0.491345808
0.470564431 0.339673949 0.489063883 0.356280825 0.392295341
0.476088654 0.163893449 0.572100789 0.445047844 0.3600291140.26109248
0.209738681 0.064923925 0.336827538 0.57198385 0.437568452
0.5816321130.518928039 0.715335707 0.513196183 0.366431539
0.176910423 0.35247631 0.449120249 0.660349564 0.431388067 0.59679334
0.401935501 0.439526406 0.30610713 0.334531946 0.3114750250.311069002
0.282296487 0.174404138 0.390905161 0.20178264 -0.002891104
0.209669887 -0.188803618 -0.012748485
TCGA-78-7535-01A 0.426536999 0.391093783 0.493437685 0.342405638
0.218893467 0.469046816 0.335968974 0.48417623 0.393150813
0.474844553 0.393352503 0.456337382 0.324040588 0.351344363
0.3913119410.19467117 0.457430293 0.42491632 0.382759904 0.318472451
0.189027554 0.143653189 0.314547039 0.48483477 0.393604123
0.516832916 0.429031517 0.640600189 0.444068583 0.377406839
0.238197872 0.274930253 0.378623871 0.596148594 0.436080489
0.5591139490.4391116730.406894158 0.304282366 0.341978792 0.297992097
0.300377512 0.287413671 0.151836936 0.382440889 0.292977512 -
0.099172887 0.238618438 -0.17464844 -0.095670265
TCGA-78-7536-01A 0.391805381 0.368006318 0.45649335 0.421024385
0.2551105820.484931306 0.3185711420.502480348 0.531859379 0.433538569
0.412944739 0.425733072 0.312627053 0.3412049110.468879674
0.1130991580.544586169 0.433961215 0.386848986 0.236309757
0.135531014 0.076592903 0.291548759 0.555010605 0.44551004
0.5886811980.573682907 0.727993801 0.529790922 0.314350845
0.216742638 0.23862495 0.419382967 0.626482044 0.423150086
0.508400138 0.36003579 0.421362409 0.274826536 0.289633174
0.297213514 0.19948019 0.286701247 0.129049531 0.362439098

0.2811993020.000660988 0.17861494 -0.185826575 -0.08436433
TCGA-78-7537-01A 0.3643411510.367520733 0.447854014 0.29445309 0.275016196
0.50498976 0.310794355 0.468332991 0.265957523 0.449890818
0.382923952 0.48831276 0.347620672 0.349367357 0.4305405110.206145628
0.5411872230.412249676 0.344636046 0.300092307 0.155165397
0.1676682110.291569051 0.543950269 0.39365184 0.497712057 0.281292814
0.640658703 0.423161687 0.423138381 0.201914142 0.305268358
0.426734947 0.638078433 0.406187954 0.523052024 0.449270891
0.413786312 0.329692498 0.408262638 0.337156409 0.295655706
0.304319262 0.160640563 0.360074275 0.263818114-0.14164836 0.22673027 -
0.164288024 -0.042352974
TCGA-78-7539-01A 0.456572913 0.356817764 0.455275639 0.307264797
0.263741708 0.4832113790.371790889 0.448053325 0.292438641 0.48573371
0.375804076 0.463014187 0.350626771 0.352735277 0.422716522
0.1911899180.528583871 0.55682701 0.486257583 0.3036114770.12352984
0.10279709 0.34891121 0.517529077 0.4271182390.508177401 0.315708118
0.649196928 0.436047838 0.3395116870.276403561 0.29911185 0.414541928
0.625636932 0.389100154 0.523159481 0.447957429 0.420289004
0.292161787 0.315604324 0.315400428 0.280573416 0.354916914
0.176388204 0.368338206 0.365185672 -0.128011165 0.230186819 -
0.146252248 -0.080305286
TCGA-78-7540-01A 0.429220278 0.398372808 0.492252949 0.319734092
0.217495575 0.539288551 0.320108759 0.443964852 0.270115157
0.474236356 0.424779529 0.462986338 0.375730434 0.405035228
0.469397709 0.182195815 0.561042448 0.520529587 0.407266984
0.30559039 0.196731091 0.128589362 0.35921168 0.495709627 0.389700935
0.508053821 0.2604941180.6116149080.354130916 0.345753583 0.229700389
0.342059845 0.428083364 0.59431249 0.4283311150.535579398 0.46237711
0.406084372 0.328454995 0.329040507 0.331988038 0.337223456
0.321514415 0.185207754 0.395102366 0.237378029 -0.154726988
0.270758375 -0.142348461 -0.043375336
TCGA-78-7542-01A 0.471381285 0.412999798 0.508192208 0.444274021
0.226198828 0.504175085 0.355089645 0.495774315 0.514565748

0.48707552 0.353837023 0.438952207 0.329689393 0.346669387
0.452420182 0.146325071 0.571677799 0.620038155 0.506231168
0.327957963 0.207784283 0.1149679650.347978336 0.555423883
0.435409689 0.587894814 0.540573607 0.7119164950.497687559
0.371336925 0.25683963 0.254396439 0.401416647 0.599232623 0.45926877
0.550704338 0.447100353 0.391950091 0.326724638 0.362053444
0.310148838 0.261266367 0.312927686 0.108436656 0.365034275
0.289727309 -0.072445011 0.246858755 -0.168370069 -0.149606058
TCGA-78-7633-01A 0.358526413 0.364015901 0.489531899 0.347005516
0.320140167 0.502741701 0.244032488 0.454580754 0.308261105
0.429640618 0.427437675 0.508465362 0.34245386 0.349468491
0.446597438 0.200795379 0.560323865 0.362762279 0.304616901
0.301296823 0.139078449 0.134950405 0.30427448 0.519964355
0.398359892 0.512642446 0.329931959 0.64951921 0.403316914
0.327578908 0.168241406 0.329330377 0.457251495 0.631771961
0.393236818 0.562497545 0.43221928 0.427038599 0.361574059
0.354794233 0.343920059 0.301753199 0.282855383 0.184237273
0.368302075 0.175459053 -0.105955325 0.197066776 -0.129506085
0.008730584
TCGA-78-8640-01A 0.363760543 0.377231913 0.487153741 0.380877561
0.19709546 0.430300243 0.29720316 0.493132626 0.477222276 0.446272315
0.319299165 0.458237235 0.31586711 0.310132483 0.425458256
0.165052321 0.532521388 0.439455128 0.388767189 0.269693716
0.120740837 0.049769347 0.297212967 0.573540638 0.420870896
0.601945183 0.501960835 0.714918688 0.57165354 0.309555631
0.190183969 0.306898195 0.418936561 0.622336187 0.444884226
0.567681243 0.430997552 0.405854915 0.251028541 0.247408952
0.313732731 0.256189054 0.28340053 0.130436289 0.380121737
0.271971036 -0.054450053 0.180380529 -0.182228313 -0.146788895
TCGA-78-8648-01A 0.562482439 0.471833789 0.455322212 0.365121073
0.283496048 0.545318081 0.446505029 0.444331057 0.349347967
0.5251120870.44709405 0.484034328 0.334295899 0.350271059 0.475440174
0.251067332 0.508305758 0.483002608 0.493196307 0.376368194

0.236232541 0.184208921 0.418269884 0.5381120470.415348967
0.532683076 0.363855884 0.638919892 0.401952559 0.594751633
0.370551889 0.317159745 0.404824829 0.58957243 0.403856722
0.540977629 0.465434257 0.450683992 0.383250946 0.507066486
0.333342258 0.369074352 0.409575665 0.169233505 0.356703974
0.436042391 -0.109906672 0.333901873 -0.183033977 -0.056362819
TCGA-78-8655-01A 0.439335903 0.36152434 0.414470189 0.335383443
0.2571165490.540048491 0.384445038 0.477870768 0.3257116590.480569835
0.431901034 0.46325354 0.318437535 0.334243044 0.443414515
0.186389365 0.51872831 0.517791422 0.465028235 0.338785978
0.175593317 0.134565458 0.366226176 0.50079677 0.407591085
0.496215121 0.336997147 0.656792308 0.451988685 0.411297317
0.270404172 0.288320287 0.406480831 0.601474867 0.416905416
0.514901785 0.44345258 0.4095086110.310399898 0.375859633 0.322620421
0.334305221 0.338309973 0.142763975 0.358261468 0.340539266 -
0.124202788 0.291989376 -0.158607795 -0.096279217
TCGA-78-8660-01A 0.455553133 0.393962857 0.442622162 0.389667817
0.26821398 0.490691757 0.401007407 0.47722619 0.451453243 0.48599208
0.365305992 0.472982597 0.318596888 0.354903094 0.440814485
0.185428194 0.507751484 0.474244815 0.44203653 0.312860968
0.1722503110.077990775 0.376768592 0.562254573 0.418137355
0.578604877 0.485564216 0.7011401090.548470944 0.387066409 0.28728288
0.321656747 0.425324078 0.595516555 0.435831852 0.594075185
0.453072578 0.439918925 0.277784248 0.321779626 0.307072508
0.315500731 0.359099218 0.1439411560.376071515 0.386285545 -
0.071368815 0.259999796 -0.175485976 -0.124485705
TCGA-78-8662-01A 0.36710551 0.362057207 0.519346092 0.3743110660.260002984
0.430216196 0.25443996 0.493895205 0.432886896 0.43425794 0.376244084
0.4682858 0.285196637 0.300489805 0.404999347 0.189835519
0.530029733 0.319756049 0.285364081 0.315169054 0.143945414
0.1152557390.26044773 0.554296162 0.415387345 0.558941561 0.469323477
0.684360801 0.521078377 0.321605858 0.145955587 0.281416011
0.408897226 0.631557828 0.424224477 0.522880729 0.414242959

0.429925623 0.295045124 0.318946148 0.284688642 0.237033209
0.250317605 0.124087282 0.377409609 0.179511929-0.066231016
0.120130737 -0.134664286 -0.208248084
TCGA-80-5608-01A 0.417004852 0.416451697 0.520522625 0.358313297
0.235667277 0.495727129 0.329882635 0.466197192 0.409289455
0.444482561 0.366860484 0.470903312 0.379445884 0.418096652
0.489035059 0.183966184 0.53309461 0.458535057 0.398981291
0.274183168 0.14521888 0.14608879 0.341516396 0.539519414 0.401143015
0.555881984 0.420058771 0.667825357 0.476436533 0.358014832
0.203559006 0.305784705 0.439316226 0.631579187 0.434106137
0.536009473 0.435948958 0.436226872 0.301331741 0.352981112
0.324713094 0.330221382 0.320176539 0.157200005 0.406866695
0.244488261 -0.086057337 0.225805714 -0.130706826 0.042847678
TCGA-80-5611-01A 0.427525482 0.40060955 0.492055694 0.38929939 0.221328212
0.442764165 0.393620731 0.492764377 0.488106856 0.473317829
0.322060041 0.461224029 0.293418454 0.330848158 0.450328002
0.142527149 0.52746672 0.554628254 0.49058079 0.260598472 0.128591778
0.091333286 0.358064564 0.535718044 0.436922746 0.591703578
0.5327749110.707676238 0.485263935 0.334603733 0.271461517
0.337809288 0.433722695 0.643584389 0.451869303 0.563908956
0.434368075 0.40845982 0.28664667 0.312033701 0.310256284 0.272295989
0.3127119070.145432474 0.355487718 0.356498574 -0.004072141
0.222984085 -0.170488071 -0.161171677
TCGA-83-5908-01A 0.5511130190.394041005 0.432331675 0.457552333
0.250690322 0.544534252 0.420997251 0.486886816 0.53888323
0.528778496 0.381916604 0.425225701 0.301235439 0.321007524
0.440136013 0.143691487 0.525089892 0.625321929 0.569009303
0.356567945 0.209167863 0.150990549 0.406204964 0.565259367
0.455282915 0.573201886 0.565030133 0.725792102 0.523038789
0.474636302 0.342906088 0.259747917 0.360196379 0.584182678
0.417540148 0.53006129 0.447298817 0.433683271 0.355558038
0.408866014 0.2837771110.314388209 0.381622657 0.090754407
0.355179478 0.428254618 -0.054640058 0.309315254 -0.212719178 -

0.092239729
TCGA-86-6562-01A 0.473772329 0.439668314 0.510209997 0.374618882 0.271884276 0.5517237 0.336048579 0.438368664 0.377127625 0.499607709 0.479940315 0.482209929 0.36173982 0.37362248 0.499201524 0.230770377 0.561230972 0.459818814 0.416871435 0.36125426 0.188902283 0.192992944 0.351791325 0.515349829 0.404894758 0.54426792 0.383028199 0.650359522 0.408808665 0.521778057 0.274241995 0.291155050.409931617 0.588551831 0.445890262 0.509602434 0.440974789 0.410448543 0.371815504 0.445555601 0.344742072 0.354900141 0.33670091 0.157177486 0.375800368 0.268890783 -0.098039853 0.282579635 -0.144926856 -0.091343629
TCGA-86-6851-01A 0.438268423 0.386306283 0.419001668 0.387219191 0.2565113640.492309591 0.427622799 0.450600718 0.377408778 0.501548879 0.385426409 0.42122004 0.33960104 0.337725656 0.448296677 0.164174447 0.533634418 0.56249977 0.528510076 0.322840182 0.21474854 0.09775666 0.377664693 0.551454221 0.438515015 0.53726038 0.38733531 0.656665801 0.407686374 0.378782131 0.291893085 0.263287675 0.392046014 0.568334506 0.395228478 0.509444735 0.427562657 0.406550991 0.303360962 0.351048 0.332552291 0.285905998 0.378468986 0.125918534 0.354380395 0.433375917 -0.091242242 0.274018547 -0.150690205 -0.101617326
TCGA-86-7701-01A 0.453760909 0.419614596 0.454172551 0.375850932 0.26145086 0.547346894 0.399021263 0.447001612 0.479791522 0.488742759 0.404449422 0.450063757 0.347218528 0.385502064 0.49517658 0.161044904 0.572381736 0.487829314 0.480872933 0.284378514 0.182523235 0.1411765550.394034371 0.56813927 0.437682659 0.58358173 0.478065388 0.700418104 0.463010409 0.481607572 0.279560464 0.26939266 0.401345544 0.6141140010.418223953 0.520548405 0.394356064 0.444937964 0.351749099 0.4550802110.318747372 0.316342502 0.384797555 0.131564145 0.383009033 0.391457474 -
0.051648013 0.295478594 -0.160171317 0.072872781
TCGA-86-7711-01A 0.515035176 0.427174583 0.493729159 0.399426321 0.247509705 0.513888237 0.440581205 0.439708605 0.463331843

0.49352559 0.386162234 0.48966596 0.333295637 0.336532688 0.479881845
0.179584823 0.547199436 0.457509216 0.436047093 0.298545106
0.195527029 0.153562047 0.374948243 0.556061537 0.458488142
0.613141043 0.46907197 0.691344831 0.502483874 0.492160032
0.344196723 0.325027705 0.405075994 0.60809209 0.441574432
0.588121467 0.44384285 0.433337654 0.334910187 0.413366071
0.324381836 0.3200411910.326271305 0.160210522 0.373952587
0.341887321 -0.04239103 0.272337781 -0.176927609 -0.131987996
TCGA-86-7713-01A 0.363008864 0.365591727 0.448954904 0.413408207
0.285307788 0.4934010110.280288398 0.465294258 0.451472064
0.414994016 0.385508623 0.454385553 0.308942485 0.336508329
0.4511119590.136974019 0.5394611870.445891981 0.353325404 0.272634722
0.131043372 0.170740162 0.28223757 0.556997347 0.425310805 0.5430399
0.464091788 0.697039672 0.536122978 0.314737229 0.19653324
0.240172575 0.396673551 0.588245281 0.414180412 0.485426612
0.388401048 0.407314332 0.319316813 0.386637461 0.292356091
0.252270677 0.280293856 0.143435065 0.368036129 0.187079538 -
0.052580956 0.201207924 -0.156511383 -0.048542706
TCGA-86-7714-01A 0.388188468 0.371553652 0.43128581 0.355467716
0.254309414 0.520606301 0.340580307 0.456957862 0.306645467
0.481541754 0.448692986 0.470195018 0.32531294 0.320319382
0.495476918 0.155551229 0.575392865 0.505677462 0.430295466
0.317184826 0.173127424 0.157435349 0.33382208 0.484961834
0.416675782 0.50280832 0.321405879 0.641467541 0.379883809
0.416821522 0.241868048 0.316157346 0.428537965 0.59708423
0.402004681 0.5220512 0.463780326 0.384974948 0.362601325
0.372231507 0.337330896 0.32257649 0.31462004 0.186961652 0.390509087
0.280650946 -0.113279022 0.269397918 -0.122655486 -0.040180663
TCGA-86-7953-01A 0.52036569 0.423893332 0.465031072 0.418464814
0.233952036 0.538918864 0.385373004 0.456039268 0.4740376
0.495487756 0.410973334 0.457984967 0.325842689 0.338610114
0.469303934 0.172335287 0.549399579 0.45812544 0.430354974
0.308720366 0.151773932 0.190063981 0.357285925 0.54577506

0.446377393 0.576399971 0.491082446 0.682638965 0.49832374
0.444616369 0.323947087 0.274937843 0.399581612 0.620924277
0.425895225 0.547583336 0.428616415 0.459225748 0.335613469
0.43436035 0.331515245 0.282557377 0.362588836 0.163761983
0.384002364 0.353398013 -0.022077714 0.286447233 -0.188277032 -
0.145486839
TCGA-86-7954-01A 0.430141621 0.356003009 0.423964056 0.380097485
0.244951284 0.483768659 0.439040679 0.465734235 0.400777354
0.485986515 0.429134389 0.454587875 0.281608339 0.313794881
0.478603268 0.162142642 0.548999288 0.664332729 0.573769446
0.300877406 0.184998315 0.162056304 0.390702963 0.545413423
0.437665743 0.548121851 0.424644338 0.668925704 0.445101235
0.39880154 0.307244185 0.295636975 0.395833777 0.590031875
0.4058363110.546210912 0.4202112780.42998944 0.305783164 0.404421589
0.313414465 0.313130413 0.351612734 0.15187258 0.376185967
0.389248177 -0.068538645 0.273955056 -0.166471358 -0.059459884
TCGA-86-7955-01A 0.331240428 0.377478491 0.506903525 0.393819 0.23349353
0.426323558 0.202416829 0.488126064 0.457720475 0.400377289
0.2736611110.468535413 0.30450252 0.317474201 0.462465751 0.117335869
0.551966619 0.394672683 0.318268137 0.231329508 0.108803623
0.1188350350.2336303110.560679639 0.413138637 0.589334181 0.49629551
0.708732421 0.557577903 0.252874307 0.122045982 0.263393971
0.407381788 0.655433552 0.457044242 0.520647445 0.37419767
0.369517193 0.257371996 0.218060722 0.300530058 0.195983135
0.227002943 0.158000049 0.385588077 0.147630825 -0.012494152
0.088275702 -0.202777478 -0.192699917
TCGA-86-8054-01A 0.354471826 0.415926683 0.44549429 0.413305542
0.263675687 0.50476545 0.223451444 0.5005811060.542575446 0.418895815
0.372675957 0.449252309 0.308962527 0.342297809 0.438483114
0.152750935 0.569722229 0.286120059 0.261856337 0.264050052
0.1163772340.182986213 0.274417497 0.581345258 0.4251182570.599076874
0.564332465 0.735839371 0.502579749 0.40125074 0.153084747
0.236219601 0.385950768 0.652599208 0.432214019 0.487754625

0.395687354	0.436560609	0.330417656	0.358740365	0.280999042
0.25517605	0.27352662	0.1162839850.361942755	0.17218027	0.012349885
0.227855176	-0.190011455	0.211508012
TCGA-86-8055-01A	0.486258601	0.469476315	0.488971225	0.390499175
0.277994357	0.562945637	0.380307976	0.439847088	0.389779004
0.504593709	0.462923379	0.476019133	0.345528496	0.344314278
0.499353726	0.23699266	0.582677291	0.503244689	0.465613821
0.385020412	0.218690256	0.219279483	0.383532996	0.549478337
0.414328405	0.583936307	0.390569143	0.663276918	0.413404902
0.610841253	0.334031967	0.291312238	0.396832828	0.575965403
0.480243229	0.533165408	0.44266211	0.415403863	0.388046428
0.506639563	0.3181196470.364453574	0.365943566	0.146402963
0.364505729	0.332164667	-0.068992284	0.360852964	-0.156961897	-
0.077309287
TCGA-86-8056-01A	0.481770001	0.379933471	0.476238525	0.345665643
0.23316951	0.54128484	0.367913497	0.415475787	0.284823925	0.489638335
0.365009908	0.469986457	0.3519225	0.333741747	0.486499686
0.1909131190.590367808	0.457263373	0.403623557	0.313957762
0.139546504	0.154570204	0.342583486	0.528378173	0.428699972
0.50659789	0.288128587	0.630586714	0.396579995	0.365849541	0.28353428
0.291995203	0.419822076	0.591359026	0.381405234	0.52850469
0.448623248	0.429535915	0.356120444	0.346605818	0.333627726
0.321540209	0.327461609	0.172889358	0.37169255	0.299886058	-
0.1137329490.267466394	-0.146091141	-0.094389313
TCGA-86-8073-01A	0.400816202	0.384270871	0.495479652	0.377723728
0.249396446	0.537208949	0.316342813	0.426887192	0.362418183
0.466749353	0.427778642	0.468393677	0.3411202210.349344259
0.494688856	0.20334981	0.573247354	0.458577242	0.399319274
0.336326663	0.154852012	0.147258994	0.330414803	0.53601721
0.405638363	0.54685017	0.365349872	0.666152077	0.4272621170.446246999
0.233274378	0.321934916	0.427905886	0.587452319	0.402098498
0.576302409	0.428834	0.413894065	0.363446105	0.382998588
0.344693395	0.328092625	0.305978986	0.167041807	0.402836631

0.275382086 -0.052121042 0.225029406 -0.155540599 -0.115279176
TCGA-86-8074-01A 0.490328321 0.436866134 0.494231785 0.394101578
0.288869533 0.542156012 0.384608774 0.468687218 0.431135147
0.502037942 0.38334723 0.450772866 0.326437369 0.3408892 0.472117868
0.215467981 0.555166197 0.553624936 0.482385492 0.351836939
0.196190875 0.150960035 0.355886141 0.536236121 0.428991392
0.57148365 0.462740964 0.6882115330.445221262 0.546718293 0.315493794
0.293751822 0.404413362 0.583984059 0.456268962 0.526593585
0.453694483 0.448748896 0.381381735 0.437975012 0.318525479
0.3469011030.35039857 0.144517932 0.375297993 0.330551774 -0.072537127
0.29852948 -0.177661636 -0.077427547
TCGA-86-8075-01A 0.565310026 0.459132895 0.495106674 0.394140506
0.270774127 0.573844082 0.394933242 0.453707513 0.403312359
0.528649459 0.458132902 0.450726957 0.36471502 0.362274521
0.492050129 0.229936147 0.566050228 0.5465908110.495021446
0.386381793 0.225314745 0.214196012 0.375321076 0.540592544
0.419624306 0.580280909 0.427067081 0.670969835 0.425268567
0.59236761 0.3457116220.290003821 0.3911757190.567069901 0.470334063
0.536519488 0.461809351 0.450389201 0.392198225 0.456750011
0.3115767640.346487186 0.368276509 0.147086708 0.372529498
0.323255594 -0.072684599 0.353730898 -0.135562437 -0.102288997
TCGA-86-8076-01A 0.486470897 0.404894281 0.422622222 0.311738622
0.255232521 0.510976034 0.405787816 0.453399683 0.284665661
0.49656213 0.3611733490.4861129860.3411061380.354707435 0.456423048
0.1847182110.539318541 0.50083972 0.478901688 0.303887776 0.159574497
0.1114587010.380472829 0.54066046 0.417586229 0.517804917 0.302197313
0.667295156 0.459980654 0.414687992 0.297712241 0.319172252
0.43123472 0.635398433 0.397413047 0.551541482 0.455050656
0.439523848 0.3296511810.377018438 0.338690816 0.318038709
0.364388305 0.147300284 0.377371753 0.399139673 -0.123731519
0.278918469 -0.165708767 -0.07612248
TCGA-86-8278-01A 0.435879304 0.409936732 0.468758517 0.337767038
0.222602728 0.503714785 0.388508359 0.4611558330.330459629

0.486421741 0.418626419 0.451035852 0.343913684 0.345463765
0.45790912 0.204002322 0.544280364 0.528208895 0.459067254
0.327573304 0.218398479 0.158547121 0.34244758 0.547319082
0.414052437 0.539129429 0.345746896 0.647592397 0.433721257
0.5143114730.26584722 0.2908113060.396875868 0.570370592 0.438689441
0.528355004 0.441228534 0.412009453 0.350069396 0.408445521
0.321759078 0.32601987 0.318918966 0.130940253 0.371355633
0.303566628 -0.138457765 0.27905145 -0.166417514 -0.072351276
TCGA-86-8279-01A 0.393796066 0.39803148 0.48184285 0.348656858 0.256383049
0.5064110250.317059376 0.476239064 0.366580077 0.466759372
0.424151814 0.465727243 0.318265309 0.322761718 0.446609588
0.217078086 0.518785489 0.394172934 0.349276906 0.334570127
0.164563371 0.1598311490.30783263 0.5468911180.419850222 0.538372898
0.41055211 0.664964866 0.467164867 0.477176018 0.193622493
0.288540017 0.387356497 0.603473047 0.418726823 0.522635257
0.423408556 0.416089236 0.326910159 0.363095932 0.305618664
0.304851041 0.27412932 0.155752816 0.373955547 0.242996518 -
0.088542613 0.22466222 -0.13584017 -0.100803183
TCGA-86-8280-01A 0.449096491 0.408514302 0.469280322 0.329606172
0.26541739 0.515495444 0.388581769 0.453955229 0.323487992
0.504500551 0.413213218 0.479470352 0.330533134 0.349460298
0.474128358 0.231996883 0.536233422 0.5115956510.465301787
0.361351348 0.190374717 0.172133715 0.377423692 0.515330798
0.415441627 0.526977092 0.348968086 0.65153692 0.41862145 0.48262256
0.280864941 0.314645235 0.439704854 0.603541063 0.427677745
0.549483885 0.440467725 0.43396009 0.343629739 0.418046138
0.323452045 0.35103515 0.361968241 0.166480498 0.392507981
0.363540509 -0.095742773 0.298671844 -0.154680255 -0.076236012
TCGA-86-8358-01A 0.328406498 0.38119049 0.416964435 0.462831403
0.248450988 0.460689865 0.237659968 0.4947281140.5911394460.423485275
0.347995488 0.416072416 0.299632949 0.318246857 0.407379473
0.165225641 0.5211443470.384005795 0.336991501 0.282423072
0.056789431 0.230721088 0.281616555 0.55732228 0.423206158

0.6068511310.635036713 0.73221454 0.504382423 0.387769807 0.100488389
0.246046907 0.360657539 0.609578966 0.402456183 0.552477035
0.387526156 0.453366799 0.293320953 0.389383587 0.30137783
0.193049076 0.258555163 0.1164326950.362508984 0.217450517
0.086868465 0.178975913 -0.151761989 0.207219806
TCGA-86-8359-01A 0.4987275110.417533644 0.457590936 0.325164981
0.234013426 0.5187119890.387897027 0.444801551 0.33038922 0.471234888
0.387600329 0.457307943 0.347365672 0.353777479 0.432938734
0.166776731 0.52721127 0.4921676110.448284798 0.302758872 0.196846647
0.186689769 0.367195254 0.543534444 0.425917374 0.531996534
0.338503078 0.650289797 0.461521753 0.454023009 0.298939901
0.3011430230.395295789 0.600652303 0.428587223 0.527869286
0.454935716 0.440256945 0.314371776 0.399650344 0.317673795
0.335793357 0.357862398 0.108128295 0.361906536 0.341482985 -
0.138415517 0.269465959 -0.161346907 -0.094455756
TCGA-86-8585-01A 0.449792481 0.430386266 0.46354884 0.361567422 0.2337974
0.465108842 0.364579942 0.4893110490.419123246 0.463618165
0.308573678 0.456690755 0.3267911830.356799709 0.408649665
0.179453734 0.50151747 0.444471427 0.410424253 0.276225678
0.198377329 0.077500829 0.344572089 0.522546729 0.4040446
0.534338287 0.45508069 0.676828468 0.484079798 0.4117582890.253827842
0.290669263 0.405018401 0.62934326 0.425996367 0.535036796 0.43869183
0.436051973 0.251447919 0.289879267 0.293735275 0.311905126
0.3114744850.141605576 0.375300651 0.361297272 -0.062842751
0.218587883 -0.12606434 -0.163350004
TCGA-86-8668-01A 0.421810395 0.396893662 0.427076141 0.350715966
0.270327262 0.528317433 0.36889987 0.426643002 0.282578825
0.484412974 0.463617722 0.470734671 0.335605164 0.337153222
0.456623863 0.219685515 0.547318269 0.456473626 0.419410581
0.358671453 0.168000805 0.162232185 0.350685512 0.50268274
0.403684192 0.495932103 0.306036646 0.614953595 0.378980638
0.494518608 0.263925829 0.307653518 0.406883196 0.559856188
0.403478598 0.495771924 0.457510674 0.4112943920.379912289

0.472368166 0.324239389 0.367028687 0.338594687 0.17579725
0.358214101 0.336892344 -0.131410982 0.310174627 -0.127446131 -
0.069548892
TCGA-86-8669-01A 0.426944362 0.411118675 0.476109775 0.333836238
0.305390494 0.481580482 0.320862884 0.444634495 0.338951842
0.450818733 0.371809241 0.479996436 0.36095931 0.369932299
0.468774363 0.193631003 0.543964328 0.413019669 0.360301179
0.275946137 0.141471976 0.148475032 0.347300694 0.553491354
0.42292679 0.559896771 0.354316558 0.660468331 0.471887902
0.353788052 0.240176507 0.317546094 0.446095236 0.613338565
0.402635207 0.544399462 0.442123298 0.444534418 0.29094151
0.368966952 0.336971327 0.2963114920.352477022 0.186130864
0.393403313 0.286077882 -0.09490865 0.248781183-0.181570673 -0.029124537
TCGA-86-8671-01A 0.485564848 0.389048516 0.475889828 0.330898966
0.291936564 0.498848413 0.482973376 0.440929539 0.31127357
0.519592095 0.424807846 0.462538439 0.318158309 0.344083353
0.457122155 0.2321179380.486816409 0.619674287 0.57459587 0.361465693
0.23087289 0.178217109 0.421377495 0.497127229 0.425792349
0.515506306 0.320714235 0.629424925 0.409562044 0.490479333
0.360584522 0.309792833 0.403816192 0.577890626 0.364927239
0.559576722 0.459038647 0.419200075 0.33545718 0.422143925
0.316231718 0.353880685 0.409316786 0.167688256 0.377515405
0.467760776 -0.149382494 0.329301657 -0.141423709 -0.059546904
TCGA-86-8672-01A 0.475123712 0.418595067 0.486250335 0.354163722
0.254607085 0.503843409 0.380390339 0.48092815 0.4118956480.482275417
0.359647599 0.458123779 0.3424211060.36158489 0.434137737 0.195718824
0.498663326 0.514570633 0.4711478670.3191175320.224001976 0.115073893
0.367479217 0.5514114130.423183393 0.593061931 0.445041552
0.6911676430.534092681 0.5117679120.273382696 0.322227776 0.407119011
0.610129301 0.443510544 0.593059463 0.46393654 0.440471992
0.304475973 0.381372723 0.301596808 0.314953974 0.337174786
0.133596425 0.36818161 0.365059135 -0.106027685 0.253140812 -
0.17963389 -0.120020528

TCGA-86-8673-01A 0.41575395 0.413180164 0.450808739 0.3447872110.285392453
0.4781811150.352953728 0.481797654 0.398816297 0.439294792
0.361406998 0.453535583 0.320514954 0.347952282 0.403189185
0.231368362 0.505448539 0.448690263 0.394064084 0.310165331
0.138859463 0.144300142 0.319515952 0.543618685 0.395468734
0.528301923 0.439004 0.678737147 0.5038494 0.45314402 0.218192097
0.271243739 0.380726851 0.615512666 0.438264909 0.545343078
0.414128637 0.463079844 0.284976077 0.417564931 0.311832859
0.318544569 0.301753805 0.132792352 0.363043379 0.298853275 -
0.054396435 0.218884305 -0.172217001 -0.136275851
TCGA-86-8674-01A 0.423038402 0.369998171 0.483756707 0.314870334
0.298607575 0.468567444 0.2594711010.476868561 0.33668223 0.441570063
0.396583657 0.490802069 0.312061098 0.348448234 0.412640927
0.170838854 0.507830793 0.377613387 0.326567321 0.258423078
0.141495402 0.099393866 0.294701815 0.548057389 0.385468404
0.520380641 0.365325712 0.668946241 0.499999712 0.343785905
0.155906931 0.334530784 0.461717787 0.645634589 0.410778543
0.585787312 0.46399791 0.440069795 0.260188509 0.34729338 0.329184176
0.310586221 0.264977254 0.14064848 0.381680365 0.177669562 -
0.102319159 0.162524912 -0.131565117 -0.029736957
TCGA-86-A456-01A 0.569008613 0.398078789 0.524176695 0.297455265
0.231770008 0.501733428 0.412144089 0.453275886 0.282053244
0.518723002 0.3374113240.486768506 0.345235363 0.356731724
0.470121984 0.220704188 0.525246566 0.636551625 0.545943146
0.320632217 0.155630406 0.130248649 0.385463356 0.556160702
0.401510986 0.548628765 0.314306634 0.651643287 0.454563311
0.405392557 0.336080752 0.334841435 0.43609587 0.602830312
0.397516708 0.561062662 0.472771414 0.455883472 0.322588597
0.409985913 0.330533649 0.363995391 0.35272895 0.17767112 0.391180521
0.335866079 -0.12372671 0.283200395 -0.167164052 -0.08586682
TCGA-86-A4D0-01A 0.349770895 0.362382361 0.465286297 0.390648658
0.277944503 0.453136422 0.242060475 0.510699863 0.531732101
0.425228644 0.3621177410.456005891 0.312672447 0.341204387

0.439431923 0.141503885 0.530816326 0.415080413 0.349103868
0.222027796 0.063750309 0.104973548 0.300855649 0.596470335
0.415196938 0.620571393 0.570770344 0.745583528 0.579896078
0.300174193 0.128019618 0.298442571 0.430820552 0.654544957
0.421298682 0.558562478 0.389730929 0.46267124 0.240599373
0.340025991 0.31011034 0.256519418 0.272830871 0.170904892
0.404429356 0.209605944 0.003554418 0.170800749 -0.159904982
0.076627627
TCGA-86-A4JF-01A 0.497180433 0.411722463 0.491355432 0.373513852
0.230507663 0.474122979 0.39703915 0.492639867 0.465348612 0.47875523
0.37856304 0.473443266 0.343038498 0.352430602 0.472734265
0.202082966 0.528996163 0.626687241 0.542257288 0.301544574
0.17845938 0.134734585 0.35682808 0.563840498 0.437426427 0.575578378
0.486694942 0.696599135 0.517571968 0.372814811 0.298072031
0.293805828 0.406870207 0.616017736 0.442541861 0.557255267
0.423813465 0.430073647 0.291215218 0.379056146 0.31270123
0.306528283 0.355378687 0.140453878 0.372380991 0.346789204 -
0.081468544 0.243902592 -0.16101182 -0.076193572
TCGA-86-A4P7-01A 0.492157802 0.400357327 0.481404802 0.342029683
0.27787832 0.545992449 0.404192842 0.451602229 0.323144743
0.500847554 0.416545182 0.476390598 0.329326548 0.357913795
0.465021454 0.238378336 0.521694387 0.524075498 0.476252314
0.348905378 0.151395567 0.186630647 0.377072553 0.540817
0.404120735 0.516531233 0.342928738 0.640275416 0.43632351
0.426898373 0.294913212 0.312439924 0.402923735 0.585612621
0.388451844 0.521374007 0.455423835 0.441177897 0.350793684
0.437983417 0.316769469 0.340823883 0.362253551 0.157037838
0.374353158 0.353002287 -0.13587075 0.287412616 -0.149876962 -
0.085862319
TCGA-86-A4P8-01A 0.522901921 0.399172698 0.470321421 0.3385694
0.353907155 0.570344993 0.447173541 0.41605518 0.227520374
0.513327679 0.449448625 0.500688757 0.314941064 0.331599965
0.454994581 0.251906416 0.521932764 0.56836621 0.514972349

0.353509595 0.190489556 0.152818151 0.4034114310.466557264
0.409750875 0.462143986 0.22466539 0.589161866 0.331769174
0.415628927 0.343728987 0.313123878 0.433816638 0.584052362
0.307070185 0.525947266 0.4581179980.430127394 0.384009283
0.395889638 0.338923658 0.329208458 0.386794751 0.178830288
0.363606309 0.389984629 -0.156551494 0.298754418 -0.154464654 -
0.083586173
TCGA-91-6828-01A 0.512838103 0.4113715410.500562027 0.384785863
0.243649667 0.568709535 0.417057098 0.428673838 0.398843007
0.49808773 0.401492919 0.46373616 0.3641103830.374150945 0.525918676
0.177488813 0.569923622 0.497103635 0.4571123130.318717375 0.18071195
0.178078205 0.3902111560.5401171070.431746732 0.560778679 0.395214576
0.662414281 0.424918217 0.429818443 0.34477674 0.304008557
0.429580465 0.581044531 0.4007780110.524140325 0.436188389 0.43116024
0.372965882 0.448455956 0.339063767 0.332710868 0.38299969
0.174355733 0.377477139 0.371524083 -0.07722108 0.294195767 -
0.173567751 -0.051905024
TCGA-91-6829-01A 0.391972087 0.40289662 0.475605458 0.407137241
0.281237896 0.555964448 0.298046007 0.442686778 0.424249498
0.4744611250.437793494 0.457428263 0.335147274 0.320883319
0.476939319 0.2251901110.565028174 0.461376354 0.403773514
0.371481217 0.189858962 0.162372416 0.33589311 0.53930132 0.412495906
0.554160291 0.4458061180.681950037 0.4286117710.582664031 0.24340081
0.301203238 0.399851792 0.5857106110.426259092 0.509984678
0.407103212 0.417354623 0.415437479 0.444655302 0.309869512
0.3371153950.315285443 0.155174614 0.374405198 0.257355051 -
0.047204913 0.27983599 -0.160839951 -0.040611573
TCGA-91-6830-01A 0.492444633 0.443898394 0.46876008 0.357332574
0.236554526 0.550601942 0.402706632 0.446310154 0.340889824
0.503900565 0.406363822 0.493564873 0.381282802 0.381233426
0.474906906 0.257972293 0.577373394 0.559773403 0.491372865
0.345262935 0.2421109560.137894849 0.39333815 0.552541504 0.406504752
0.563029407 0.35890508 0.6633154110.398810861 0.490701969 0.301836692

0.340551017 0.416715902 0.595979664 0.413964495 0.57191149
0.458148916 0.426058446 0.382504158 0.463061956 0.336944898
0.371589716 0.355821265 0.15169141 0.375357098 0.32879369 -0.097388446
0.315720337 -0.132405427 -0.060539009
TCGA-91-6831-01A 0.415661329 0.417771055 0.470781642 0.41440841
0.263905643 0.5243611490.341390486 0.49037395 0.5232112050.460477688
0.4025860110.4491118810.3113714380.3473358110.454056712 0.18012482 0.51831641
0.43177463 0.41379079 0.303858653 0.145262088 0.106532724 0.326950743
0.567055494 0.424174898 0.592175189 0.55342351 0.721768745
0.537754532 0.521450232 0.249554401 0.254264413 0.397143989
0.635709623 0.424951671 0.532993084 0.390718422 0.437519077
0.319302762 0.424704045 0.294079421 0.282095362 0.323226306
0.143208824 0.377379274 0.296973684 -0.013581658 0.237694874 -
0.196591853 -0.046588891
TCGA-91-6835-01A 0.491892205 0.385076637 0.474950194 0.411186612
0.265577407 0.530287083 0.430949252 0.444310068 0.388346618
0.508189166 0.412257364 0.446038108 0.342448418 0.340291081
0.474445745 0.193122552 0.541936072 0.58483556 0.53549269 0.369234434
0.231770581 0.199693044 0.410872681 0.513990139 0.435893939
0.53691664 0.415499189 0.658930341 0.408988998 0.461763853
0.356627733 0.278377543 0.379629367 0.562257394 0.386347717
0.50941714 0.441348804 0.425832363 0.39064497 0.421491499 0.323791109
0.326306274 0.405876326 0.151758687 0.378021345 0.444307366 -
0.079068589 0.333135742 -0.139618706 -0.087823643
TCGA-91-6836-01A 0.43366129 0.418067724 0.4895803110.464924417 0.262606566
0.48369246 0.298462923 0.493513216 0.556753081 0.446050891
0.353079535 0.430840972 0.302553892 0.296849277 0.43093066 0.16099378
0.543945948 0.497843014 0.425835762 0.289404696 0.159045498
0.121280253 0.321626295 0.569598741 0.452689084 0.588839411
0.576802547 0.7314611260.54077964 0.365690474 0.21908847 0.232891755
0.36909694 0.604055362 0.457137941 0.559330345 0.398539608
0.419678743 0.309254282 0.3455119540.294482855 0.227669031
0.303238639 0.085890969 0.340486437 0.24141642 0.00074147 0.20194659 -

0.189109226 -0.121826157
TCGA-91-6840-01A 0.369542392 0.346519556 0.453993085 0.400357985
0.246245657 0.523060794 0.320378156 0.48641746 0.436093075
0.451658564 0.403677167 0.433654253 0.278618063 0.319290854
0.419927423 0.193679778 0.515124042 0.577610743 0.462097136
0.340904984 0.144104355 0.181662929 0.319980041 0.514531708
0.420088607 0.508862292 0.457558956 0.684431498 0.460144247
0.379704994 0.213009675 0.258634463 0.38452059 0.592929259
0.381856529 0.484781275 0.413631034 0.443378164 0.324480416
0.450664461 0.297124263 0.309167141 0.297179586 0.097924752
0.361322358 0.300991269 -0.045968876 0.209642582 -0.141338722 -
0.085895352
TCGA-91-6847-01A 0.339454466 0.366986657 0.451684722 0.458573165
0.307305526 0.520664733 0.231887059 0.50670584 0.519734697
0.423972212 0.38984499 0.400910617 0.304126427 0.302866714
0.452541709 0.146054397 0.535869833 0.442235536 0.332104219
0.28902331 0.194783433 0.14565959 0.220994897 0.525364746 0.426926167
0.5711853540.558404723 0.731816888 0.514209001 0.358496881
0.121371939 0.215006237 0.369643846 0.601964286 0.454584809
0.499578464 0.404661913 0.3976081180.30455229 0.297461588 0.276848518
0.168905485 0.278806125 0.10622589 0.340684255 0.152482586 -
0.033679463 0.174427527 -0.162577573 -0.121092073
TCGA-91-6848-01A 0.53587472 0.423304604 0.405204045 0.445075476
0.305744664 0.521345085 0.434787574 0.4911413390.530166271
0.525457254 0.388724893 0.421272156 0.25117495 0.264710146
0.443328048 0.184380948 0.527492914 0.449512857 0.455463928
0.346289217 0.2068511610.163756635 0.383984109 0.593290992
0.426984066 0.612342973 0.558776878 0.739778995 0.572331932
0.602312571 0.365207323 0.25680644 0.350087373 0.584903071 0.4037539
0.527547284 0.410984532 0.446188175 0.369285428 0.444639684
0.292322677 0.313543937 0.392414968 0.072530576 0.337722811
0.412566208 -0.000311173 0.342782868 -0.204111663 -0.077365332
TCGA-91-6849-01A 0.532708354 0.416785749 0.539023046 0.297167103

0.256575908 0.551047958 0.363957941 0.459748905 0.250149885
0.488982379 0.417497002 0.51264001 0.370245977 0.360250801
0.497236793 0.192557253 0.551331884 0.395593361 0.405955614
0.2916311910.160812739 0.1166685450.379181214 0.51673548 0.404731511
0.5434899 0.272017544 0.642691506 0.431683164 0.389094219
0.296175847 0.377293134 0.475941673 0.642622082 0.399502872
0.589063645 0.480617691 0.446594019 0.355940781 0.40420636
0.358636484 0.353544961 0.348669126 0.217979251 0.411151162
0.332285955 -0.131963583 0.273638495 -0.130421027 -0.016574466
TCGA-91-7771-01A 0.510318145 0.425852876 0.461078659 0.34070936 0.24579817
0.544415758 0.393551059 0.416653799 0.324634394 0.496946395
0.378250432 0.498792854 0.353231492 0.363078491 0.514792027
0.196625053 0.568377487 0.529386624 0.486593595 0.314959547
0.172514799 0.162061251 0.3944711840.534086134 0.416065854
0.526526554 0.313885099 0.658103642 0.423064302 0.43717029
0.317539371 0.328249522 0.442401388 0.614231519 0.381845317
0.532820588 0.451825321 0.431614426 0.373939028 0.422458983
0.32694987 0.350886667 0.378545853 0.1711206760.39813611 0.383417902 -
0.131620377 0.319751263 -0.149383173 -0.046587115
TCGA-91-8496-01A 0.4301158580.372022262 0.485413583 0.249382352 0.18768294
0.493914743 0.364699387 0.480829715 0.242030224 0.475576769
0.383001427 0.5195377 0.300690788 0.343775878 0.447801239 0.20438735
0.532202221 0.457926957 0.4118191750.289169498 0.136105087
0.1134038070.358676685 0.490764193 0.405600746 0.520256952
0.302745089 0.64502565 0.4114746250.353808628 0.285203703 0.336307092
0.482506674 0.659742524 0.394843751 0.5365046110.462678473
0.430649022 0.290086753 0.365091791 0.332903271 0.307562783
0.327651497 0.206582848 0.415421743 0.320793815 -0.135671513
0.286987509 -0.153555869 -0.070668268
TCGA-91-8497-01A 0.436290695 0.383068078 0.48947721 0.300321691
0.313153846 0.518440473 0.388501386 0.41937135 0.246136095
0.484179842 0.428475297 0.497083181 0.373496528 0.389342903
0.466056607 0.250331667 0.50904758 0.455157308 0.419357343

0.333774071 0.188228696 0.2099119030.359107639 0.508429467
0.409942876 0.502156419 0.249204314 0.615448337 0.408390467
0.414830352 0.265673244 0.327925362 0.440162343 0.625024759
0.373424541 0.535445123 0.448798975 0.4328401170.3463937 0.405515228
0.324787669 0.34504197 0.366916574 0.19014444 0.3869474 0.352668762 -0.1197738480.308241551 -0.146476421 0.108623567
TCGA-91-8499-01A 0.328912873 0.370708147 0.458361041 0.370927969
0.270027708 0.432813636 0.257740987 0.540102736 0.517118501
0.427648433 0.31915673 0.428040813 0.232605359 0.284867868
0.407898038 0.141014765 0.502439027 0.344064027 0.33967055
0.246764374 0.098904294 0.093291035 0.291277751 0.538907219
0.395910333 0.587145917 0.592906265 0.744443788 0.543947345
0.364996685 0.147972373 0.230530652 0.408708647 0.647961841
0.41173142 0.50900013 0.3651195110.43134193 0.27254895 0.348893065
0.276045926 0.200391257 0.275143247 0.128925475 0.381481253
0.257026942 0.025094307 0.194190498 -0.192873766 -0.102792755
TCGA-91-A4BC-01A 0.443180461 0.3903959 0.39533017 0.371524062
0.264088968 0.472177994 0.430084366 0.480249832 0.428560018
0.483233881 0.36731958 0.431308529 0.27422029 0.310939436 0.399064284
0.1811415510.4752598110.541297361 0.49312487 0.30859409 0.162298229
0.106966926 0.374560566 0.556920669 0.4141996 0.54367841 0.462520737
0.684041414 0.49661463 0.487724843 0.2745701190.282356537 0.368011245
0.60107194 0.392483255 0.516250794 0.406890827 0.439059493
0.293992845 0.4119603050.26781969 0.325051373 0.334939671 0.102711887
0.346161388 0.419417483 -0.069574415 0.282650419 -0.202781862
0.047706481
TCGA-91-A4BD-01A 0.429454906 0.369171628 0.48520453 0.252802712
0.206556012 0.419966039 0.372254236 0.499271656 0.269742359
0.463486329 0.374604279 0.487182087 0.301701972 0.346825045
0.435751976 0.173332778 0.507826166 0.55370968 0.4573944110.261628884
0.142508615 0.063775462 0.338607195 0.510440579 0.391918996
0.5115609880.310077883 0.6556511020.4381344 0.264996843 0.269647604
0.320471271 0.46283927 0.659683548 0.396212838 0.55326255 0.455046783

0.418769473 0.257394948 0.278607865 0.320973504 0.285028925
0.330713685 0.196969059 0.410318436 0.297342612 -0.10245284
0.252780568 -0.144231708 -0.046481062
TCGA-93-7347-01A 0.463701958 0.382768618 0.476279736 0.325144172
0.272410721 0.51582691 0.427525075 0.447428639 0.308208576
0.518582959 0.427009934 0.486000111 0.350032447 0.365118519 0.471073976
0.231490613 0.53510263 0.532315084 0.49257406 0.355152921 0.217755661
0.18243603 0.393677411 0.51016134 0.425475352 0.533153036 0.326674717
0.646013533 0.419803937 0.486352541 0.341714844 0.337236724
0.426146968 0.616544995 0.384871158 0.553745691 0.457936738
0.429250485 0.339148893 0.418959571 0.333928587 0.368414648
0.390797215 0.187437819 0.381292317 0.432133425 -0.130379136
0.339981686 -0.15888258 -0.039200875
TCGA-93-7348-01A 0.538731804 0.420786169 0.50034244 0.313470352
0.274863204 0.530799237 0.383719084 0.439985229 0.275489634
0.501569099 0.463693511 0.458261017 0.360033726 0.363282214
0.501215732 0.210052865 0.53585304 0.431574743 0.408565523
0.339061749 0.18337298 0.168390754 0.357270579 0.525271062
0.399551063 0.526851863 0.300372648 0.623575006 0.382115775
0.543608919 0.308165386 0.303166252 0.422697968 0.597945476
0.432082557 0.51716916 0.464499724 0.439927294 0.360595828
0.455554369 0.339431436 0.354956947 0.361009644 0.163342343
0.399311932 0.317186355 -0.119666883 0.324238338 -0.1517685 -0.065738654
TCGA-93-8067-01A 0.480514729 0.412518147 0.484183812 0.402779831
0.277663497 0.545389663 0.293458175 0.463330503 0.451108484
0.466194825 0.379821842 0.461755246 0.360830235 0.371766331
0.463595786 0.178980843 0.551253445 0.500233443 0.421058106
0.300135126 0.154269289 0.15185573 0.348147971 0.560102814 0.43660894
0.575332212 0.456470702 0.691640238 0.502580557 0.403386585
0.235818211 0.325585499 0.436706188 0.596261507 0.429836586
0.585439203 0.463763811 0.456502358 0.335416283 0.403239172
0.332569929 0.315141568 0.318563564 0.137457554 0.384890186
0.23585581 -0.08603595 0.22808558 -0.156604515 -0.095784614

TCGA-93-A4JN-01A 0.462204341 0.396314566 0.451421819 0.394820575
0.283913504 0.550780914 0.386062453 0.481275322 0.42810684
0.493660558 0.4116199980.415595347 0.335143756 0.359262054
0.420191668 0.230999957 0.480918319 0.596059457 0.496928358
0.37413063 0.1788761130.143649546 0.3416830110.51435188 0.410147065
0.513150528 0.470452256 0.66437815 0.417891692 0.510789058
0.249841666 0.27954094 0.361582902 0.541656944 0.404208602
0.512041528 0.458803948 0.436004396 0.354297704 0.41297029
0.308719014 0.344912615 0.344387859 0.1190266310.351991579
0.303704101 -0.083834794 0.266308499 -0.120709223 -0.108022566
TCGA-93-A4JO-01A 0.466751339 0.37797683 0.492008283 0.3232533
0.242707613 0.486783124 0.419742589 0.4763114570.327632388
0.486330779 0.396450642 0.458467918 0.3191163020.319470742
0.425195033 0.191270183 0.487400634 0.532248509 0.484646102
0.343552169 0.171046995 0.108293608 0.364395896 0.524211547
0.408242016 0.517097058 0.367166129 0.656454055 0.482562466
0.416087021 0.305172432 0.289736688 0.383958031 0.583437516
0.398884174 0.53114321 0.452659417 0.434889635 0.313682758
0.403080603 0.306532127 0.312268733 0.339314645 0.140831254
0.372014991 0.390247611-0.119880543 0.275057255 -0.136970938 -
0.122783348
TCGA-93-A4JP-01A 0.474620928 0.396125152 0.498990882 0.363629622
0.306578618 0.53109411 0.394574807 0.438720281 0.349554139
0.491645369 0.457724323 0.506744977 0.357726013 0.378390636
0.5011347640.246284217 0.544772103 0.594887092 0.4901164950.349898521
0.210764267 0.163361995 0.360015346 0.503974775 0.405351433
0.523881794 0.364549373 0.637901422 0.376523865 0.413951795
0.275637353 0.3260456 0.450569518 0.593375669 0.377580426
0.527053108 0.447185032 0.443903783 0.386869689 0.408192759
0.326380565 0.318209364 0.339831098 0.200526299 0.395935747
0.281552714 -0.122490557 0.27748586 -0.11152883 -0.123820727
TCGA-93-A4JQ-01A 0.521740702 0.408938461 0.446354817 0.379872008
0.283372153 0.533418249 0.424694789 0.466331355 0.390712889

0.519464659 0.452959294 0.453968871 0.318176569 0.354244592
0.431953293 0.2115277560.503788451 0.59591901 0.535360248 0.380411673
0.230847659 0.138646338 0.395612459 0.533900708 0.421276426
0.542432171 0.407477879 0.669538369 0.474971049 0.562395561
0.336323535 0.279331612 0.378980288 0.577720556 0.415857968
0.541310364 0.472042362 0.427633229 0.345771723 0.432526828
0.305895577 0.362401288 0.374930156 0.103533774 0.344729258
0.411022204-0.111274521 0.319713743 -0.163051451 -0.071003098
TCGA-95-7039-01A 0.471412647 0.40853606 0.476655063 0.37168144 0.273857011
0.540651685 0.33290999 0.462800826 0.402430093 0.494355266 0.40316936
0.473834363 0.347990884 0.354501437 0.466813436 0.212446595
0.508171403 0.46275857 0.408435949 0.3406821120.190214948 0.176835658
0.340895745 0.528819155 0.410763647 0.543210844 0.435767158
0.67202835 0.493302104 0.442909221 0.217872679 0.294108439
0.420618972 0.6092326 0.439496304 0.549134393 0.434269946
0.428990772 0.357874982 0.399309967 0.320837186 0.328796195
0.329144607 0.138052604 0.378212692 0.266014679 -0.069743838
0.233901795 -0.164808627 -0.061632519
TCGA-95-7043-01A 0.2658533110.337133804 0.454864386 0.371953038
0.303274053 0.472210727 0.222795731 0.5211920610.435848086
0.403877162 0.413182768 0.479448143 0.327268754 0.331841207
0.422281891 0.166669666 0.538716451 0.321430975 0.282043442
0.257478939 0.126792357 0.078906176 0.271895902 0.558454204
0.389814186 0.57286168 0.474459945 0.69890694 0.509856931 0.334720296
0.108965205 0.32599928 0.447430053 0.637166004 0.415635534
0.590904707 0.408368031 0.426715475 0.276025877 0.366104356
0.319151833 0.219409982 0.278646673 0.162623092 0.374982852
0.173446989 -0.035273016 0.140815383 -0.167255687 -0.111716231
TCGA-95-7562-01A 0.403068323 0.406475467 0.500569047 0.382919467
0.263318758 0.466328126 0.321379246 0.499230156 0.491696289
0.460479761 0.377545948 0.451329021 0.310273824 0.338049938
0.474036147 0.137416696 0.53218623 0.512915755 0.444864094
0.290908092 0.180797632 0.168167692 0.341778322 0.548127603

0.439526527	0.605747693	0.528251914	0.718172745	0.51295855
0.430806933	0.250199002	0.25480843 0.417159519	0.622523589
0.473083868	0.543914505	0.41134 0.423463983	0.315158892	0.361085937
0.323812294	0.292851887	0.329861839	0.151076297	0.415619414
0.302808319	-0.024388975	0.227260341	-0.164770713	-0.091649134
TCGA-95-7567-01A 0.42644834 0.370504915	0.482026641	0.3763051170.238483099
0.476162582	0.353599785	0.478561593	0.42155333 0.477627256
0.428714756	0.460956645	0.338615742	0.344172455	0.423593014
0.198265627	0.519757528	0.56313436 0.479621086	0.316126363
0.152939826	0.128964775	0.329268832	0.557912465	0.41772152 0.54928255
0.435308143	0.683481805	0.512497884	0.405850385	0.254555348
0.268592618	0.374657121	0.5968723 0.409825787	0.532020997	0.42954778
0.410770807	0.30966748 0.384699404	0.307330008	0.285150143
0.327333406	0.133020216	0.371873586	0.328364298	-0.077875826
0.217493732	-0.151188089	-0.117432507
TCGA-95-7944-01A 0.477513322	0.371828843	0.404047502	0.374801524
0.277846784	0.513846072	0.432405469	0.499216312	0.450415669
0.48136267 0.300607215	0.45242248 0.302928357	0.344945218	0.400161943
0.162307551	0.513046636	0.595251476	0.539361733	0.325351423
0.241967248	0.075941809	0.380928462	0.543971347	0.42415641
0.525763929	0.471524097	0.706175847	0.552679863	0.454676438
0.293504181	0.309223459	0.365981294	0.615774071	0.3895422110.51654252
0.419781454	0.416181245	0.307906816	0.386812377	0.275779897
0.320028631	0.353509223	0.109087979	0.32911791 0.426212269	-
0.074523686	0.259446636	-0.166306245	-0.118570312
TCGA-95-7947-01A 0.363806066	0.359379721	0.467314046	0.402064842
0.27384159 0.453084514	0.317000299	0.4811336160.459857996	0.456055839
0.353717465	0.459529497	0.326807644	0.347195997	0.462086546
0.144496945	0.545343858	0.538304316	0.455163933	0.295120321
0.16398232 0.108308876	0.338287688	0.532553566	0.42851209 0.565639997
0.49149162 0.676752433	0.440830028	0.283378488	0.2126911880.275649683
0.415522174	0.61588501 0.431819292	0.523705405	0.388530406	0.42460668
0.316651229	0.295138723	0.329453204	0.246362761	0.312280955

0.16800389 0.394526254 0.285656552 -0.045244004 0.204772775 -
0.163168781 -0.186714937
TCGA-95-7948-01A 0.347398362 0.34658788 0.417762393 0.293448786
0.259982878 0.4911895510.270026316 0.4811073150.320790255 0.433838451
0.409904967 0.4621761 0.296413205 0.339553489 0.42748391 0.181361707
0.509235054 0.443904541 0.381029295 0.280134824 0.158928657
0.1172987240.26676976 0.539945438 0.416092682 0.499050466 0.362385889
0.679451684 0.513429697 0.350258364 0.163812536 0.265404885
0.383222159 0.628902819 0.381647871 0.516430287 0.417022461
0.424518049 0.31241455 0.316922463 0.312382886 0.227694965
0.252786279 0.142920776 0.360478533 0.216532046 -0.12996956
0.172248169 -0.135042155 -0.142762101
TCGA-95-8039-01A 0.449715144 0.38097298 0.428448449 0.352707821
0.214784067 0.519416089 0.4116553780.464766045 0.344127695
0.494890598 0.41484424 0.482269863 0.345253477 0.361206247
0.441525173 0.209314904 0.53047898 0.565536399 0.493399322
0.334419346 0.158986183 0.143152359 0.378851057 0.513776818
0.4115077790.513077267 0.367966313 0.665151383 0.445208053
0.421432441 0.309605836 0.308357214 0.410469389 0.613099245
0.415100588 0.536277646 0.44701802 0.410973772 0.337982008
0.376970776 0.331017525 0.350256483 0.366403898 0.156459631
0.401487854 0.352417047 -0.111540595 0.299723325 -0.158334327 -
0.113495431
TCGA-95-8494-01A 0.473408865 0.438940989 0.454527008 0.3906533 0.20849138
0.48196389 0.324122393 0.495978763 0.46248511 0.487448594 0.39148351
0.460215189 0.300770688 0.322072249 0.410135717 0.191106169
0.5321811730.53574971 0.453560419 0.320323398 0.17232232 0.1026142
0.317762643 0.566310987 0.428423588 0.576458705 0.506899789
0.699148326 0.542090769 0.387920937 0.27486796 0.270589708
0.3916112710.603275756 0.481308419 0.568152375 0.433536146
0.439014651 0.269919969 0.414668907 0.309737617 0.266086309
0.316124475 0.122869381 0.360434024 0.296628032 -0.074815603
0.246617784 -0.173963914 -0.125816919

TCGA-95-A4VK-01A 0.429093459 0.38152257 0.487601459 0.363915157
0.313609156 0.519057481 0.3402114980.471034962 0.349286323
0.467686795 0.420148041 0.459141219 0.335963599 0.347169536
0.448785673 0.189544421 0.514275746 0.500168097 0.417970721
0.302990952 0.161753528 0.103457015 0.316184445 0.503398483
0.410197585 0.502379961 0.373176293 0.654045108 0.437732276
0.387249535 0.209803478 0.322464071 0.422314072 0.614359919
0.404480183 0.521003766 0.446298408 0.392571639 0.326823103
0.295720755 0.304846215 0.319289027 0.313227963 0.145565242
0.379593244 0.272997138 -0.111265875 0.234094 -0.125644946 -
0.121913463
TCGA-95-A4VN-01A 0.547027427 0.423596706 0.450742358 0.416239902
0.251986168 0.49540284 0.447509306 0.485417236 0.456734985
0.521561053 0.417551539 0.464335871 0.322094086 0.354999193
0.433737884 0.183810725 0.532375614 0.59515134 0.539655976
0.341486438 0.192870148 0.133334226 0.384992807 0.558163615
0.443144793 0.563627507 0.480129059 0.705735442 0.522705811
0.415582329 0.348381531 0.295893828 0.383608217 0.584846055
0.421858648 0.573734562 0.456709003 0.450013614 0.295767378
0.43116312 0.309102524 0.317483447 0.377078717 0.135505965
0.383031708 0.402833898 -0.076580947 0.299799238 -0.173480048 -
0.103445044
TCGA-95-A4VP-01A 0.485984478 0.42178822 0.465865241 0.32965173 0.27833358
0.48438131 0.399990632 0.46691572 0.3607035110.467732922 0.398161639
0.476193858 0.333446374 0.376145963 0.409422208 0.251277153
0.476301563 0.456441752 0.409012166 0.308367105 0.158744861
0.135997172 0.35890141 0.534694981 0.4110323060.514475061 0.370930109
0.652721817 0.499253562 0.443703481 0.269201723 0.312911521
0.396975966 0.619386781 0.392710805 0.544887516 0.443699974
0.452879887 0.287403588 0.425244156 0.297139663 0.363257986
0.36174968 0.141852937 0.361421426 0.322661508 -0.110897456
0.255860953 -0.151292321 0.025045875
TCGA-97-7546-01A 0.518277292 0.392571401 0.482741398 0.357872169

0.249258656 0.592576007 0.405605504 0.426223321 0.339177753
0.510809358 0.432180552 0.4779380110.365039864 0.362430822
0.528222233 0.210757385 0.566800947 0.460137571 0.44367874
0.335203804 0.177476809 0.170903188 0.375420857 0.508470386
0.416189592 0.522163098 0.343484057 0.656771514 0.402144909
0.475555685 0.329559716 0.29295011 0.399922002 0.584499586
0.368947597 0.49277472 0.446613739 0.422296603 0.423285412
0.409127674 0.334736223 0.337819067 0.385917262 0.187499927
0.367554478 0.361072679 -0.099053533 0.304313281 -0.171158986 -
0.030633166
TCGA-97-7547-01A 0.400766633 0.381370698 0.479904049 0.325696761
0.253453455 0.519233416 0.3117701720.406447178 0.2540911930.476538306
0.426856757 0.480397187 0.370094025 0.332650676 0.519162735
0.218929563 0.560920577 0.4019448 0.376457392 0.342041431
0.183307937 0.19756051 0.326482022 0.522661469 0.393828754 0.51977597
0.246857396 0.620230801 0.4110582180.3911357640.23222918 0.30041407
0.425206254 0.596776968 0.419487903 0.547948983 0.427986271
0.409540396 0.374104297 0.329948307 0.341313835 0.296310899
0.322969819 0.1972951120.415799394 0.281971855 -0.150245863
0.267112397-0.155479798 -0.042493457
TCGA-97-7552-01A 0.470304251 0.389822687 0.448159018 0.354754335
0.264541856 0.533345988 0.42426459 0.405201993 0.293419929
0.513748545 0.423299534 0.465902871 0.371305331 0.389607914
0.504907513 0.185915388 0.569788548 0.598216579 0.543056598
0.340980827 0.240350538 0.176697375 0.42679986 0.493474097
0.419942626 0.5117418740.276256127 0.618260615 0.343228449
0.398946007 0.32621315 0.321923955 0.428948378 0.580983477
0.386047729 0.526564892 0.449016434 0.395022894 0.389063247
0.350641851 0.342910336 0.326192781 0.394876025 0.17892176
0.371750269 0.43832674 -0.127751551 0.335289175 -0.140753893 -
0.021369594
TCGA-97-7553-01A 0.554397352 0.406264363 0.497440382 0.376328906
0.26084699 0.554206204 0.491601432 0.43933096 0.377378073 0.531129998

0.413507378	0.494751424	0.350512373	0.3648114630.498587315
0.208506285	0.555297906	0.571494322	0.533764281	0.35715883
0.224489883	0.148717003	0.422276225	0.507248091	0.426578781
0.541955931	0.390396187	0.652253812	0.391351089	0.456364959
0.3943118560.323449958	0.436672043	0.593871792	0.376858178
0.550301929	0.452556531	0.450041681	0.378220161	0.454189485
0.341690833	0.360260146	0.3978224110.182103056	0.393332947
0.421695593	-0.099421582	0.349098936	-0.154230665	-0.074904357
TCGA-97-7554-01A 0.466410972	0.422092036	0.473491728	0.380764442
0.240730823	0.542595432	0.361715157	0.449987291	0.364871779
0.504364403	0.445389714	0.455818202	0.349145551	0.344362037
0.487388955	0.208372246	0.565017226	0.544786203	0.490503284
0.370430014	0.206037094	0.200257055	0.367857597	0.541045826
0.417498294	0.548283404	0.379248805	0.660751828	0.432333032
0.531015014	0.303158249	0.284651902	0.386733954	0.581105806
0.437789294	0.503593575	0.41816694 0.413421087	0.37811305 0.443798087
0.337659892	0.362878685	0.344225416	0.134663437	0.357975291
0.339312829	-0.084318496	0.295085863	-0.182685723	-0.056903558
TCGA-97-7937-01A 0.373917067	0.364986851	0.479178001	0.362987777
0.228509618	0.518375241	0.295333089	0.469556143	0.389636664
0.460008023	0.432515224	0.4536991180.321808175	0.327758229
0.466864924	0.184513095	0.5511869270.387663178	0.363433846
0.306216252	0.147469577	0.146747549	0.288523551	0.537165565
0.402046652	0.534586528	0.4297682	0.68288714 0.458625765	0.413463141
0.2071174860.265800285	0.388243056	0.615829847	0.41705155 0.521796544
0.400296637	0.403163877	0.344905261	0.376226852	0.311825998
0.273099898	0.265278003	0.165061288	0.370869885	0.237954416	-
0.0804111820.182919346	-0.179067509	-0.084938169
TCGA-97-7938-01A 0.549270294	0.400620951	0.4811318970.335654468
0.232519923	0.584445188	0.365460363	0.414083768	0.290712872
0.510927586	0.445278632	0.484158377	0.362016751	0.356078601
0.520818829	0.207004454	0.579386236	0.438082512	0.409805011
0.332883524	0.16677278 0.157483338	0.35467928 0.519608879	0.407863525

0.51075959 0.298347139 0.65016648 0.384716536 0.441911 0.302675089
0.32400753 0.4300115530.596818542 0.381707287 0.485993217 0.462814727
0.426148854 0.396900904 0.423472519 0.334262714 0.346906316
0.349632169 0.1811806170.398760219 0.286606933 -0.124620228 0.28059672
-0.136605688 -0.051275992

TCGA-97-7941-01A 0.416336777 0.399882387 0.441728699 0.300775247
0.2758874110.502886831 0.359809887 0.449134725 0.24087451 0.475624299
0.447043146 0.482842809 0.373221504 0.385595397 0.437491458
0.214327298 0.5111559130.52177709 0.418530998 0.339714608 0.197925803
0.140185073 0.352357301 0.479662845 0.372359942 0.480307772
0.252599143 0.623317177 0.430641809 0.4164750110.23528793 0.349839516
0.45176832 0.607220946 0.40838109 0.558471366 0.481281659 0.398636094
0.34691308 0.387918849 0.31288778 0.3621965110.341926401 0.179905785
0.395384249 0.289042873 -0.141562872 0.283792885 -0.100406924 -
0.037132558

TCGA-97-8171-01A 0.305323867 0.323280042 0.46180437 0.325634744
0.3232761140.426253218 0.226641266 0.494792716 0.377438699
0.400463854 0.349742055 0.492443003 0.280697146 0.305818785
0.460205708 0.148376179 0.496955049 0.40474545 0.303772346 0.26036489
0.132061814 0.13049459 0.24504744 0.521232447 0.391395799 0.541998338
0.409448399 0.699220207 0.58092193 0.26875975 0.121356781 0.297583928
0.478961019 0.654630187 0.403350132 0.5011840970.431265264
0.412836173 0.256940743 0.299525228 0.30583583 0.239837586
0.265865858 0.179771744 0.402063805 0.177005561 -0.076922878
0.130905369 -0.188096664 -0.124230149

TCGA-97-8172-01A 0.445231717 0.361466812 0.50030636 0.336837306
0.293536845 0.560243921 0.38512587 0.427101061 0.2756657110.487652766
0.452286205 0.466958158 0.341368077 0.359366927 0.485222234
0.243476376 0.532367852 0.453718633 0.4123673110.368740941
0.205651575 0.199751955 0.35197921 0.4833677 0.414200145 0.482861931
0.271207607 0.621424774 0.38178974 0.452429427 0.290183797
0.293627589 0.412458251 0.587306268 0.354181323 0.527957347
0.451998713 0.405066743 0.380568677 0.422412223 0.336468991

0.312663606	0.36106297	0.157138232	0.3612711910.365843042	-0.154776584
0.290717687	-0.153378464	-0.064636522
TCGA-97-8174-01A 0.388631615	0.387426474	0.390590614	0.340252465
0.319983964	0.538599859	0.364674147	0.424705438	0.259770423
0.467164477	0.439231729	0.49451976 0.329607575	0.317904122	0.46875586
0.215426554	0.540926257	0.440646083	0.39730802 0.349985512
0.176514333	0.209753136	0.347538353	0.4911615070.410513972
0.454443196	0.266816859	0.619010476	0.359090494	0.445388554
0.2438211070.321906683	0.44582686 0.60244459 0.362398949	0.524434332
0.438696237	0.405004949	0.379285724	0.432840744	0.340272286
0.344416807	0.328785646	0.159709296	0.353793897	0.309446326	-
0.143320351	0.267576646	-0.135152707	-0.002151681
TCGA-97-8175-01A 0.499253323	0.44689086 0.48923144 0.337026466	0.237678801
0.513709088	0.398156514	0.47586292 0.364144899	0.501571659
0.386191772	0.456080558	0.329924337	0.3511131790.438515605	0.15106691
0.5611764010.559545483	0.479843925	0.316061678	0.177749085
0.131849631	0.362950336	0.518753998	0.4118339450.555855716
0.384423108	0.670074541	0.415520194	0.397198879	0.333003789
0.299026696	0.394020159	0.605656614	0.460559985	0.550798403
0.453636152	0.427961597	0.308349731	0.421346676	0.324275332
0.339950439	0.365843851	0.140792999	0.351799623	0.31980001 -
0.091032461	0.306244978	-0.175593623	-0.104519584
TCGA-97-8176-01A 0.402483669	0.376210125	0.457080817	0.385374768
0.259541567	0.466656357	0.307788346	0.492958638	0.447737445
0.458032621	0.384005229	0.476050342	0.35785612 0.382590001
0.4372119250.174879215	0.520424208	0.395017864	0.352762516
0.293944515	0.163892884	0.094053562	0.334734715	0.5671188370.41250033
0.575427809	0.481822376	0.7112151210.537336805	0.374623412
0.212750238	0.35929436 0.439886613	0.6240110540.445282535	0.615361058
0.457435052	0.418880522	0.290151894	0.3475013 0.315480383
0.320480233	0.294881018	0.160866457	0.387728309	0.239745235	-
0.089576334	0.237371139-0.16513372 -0.088750161
TCGA-97-8177-01A 0.50849906 0.435049859	0.508558739	0.28601293 0.207702841

0.493840407 0.408897086 0.473059407 0.279851725 0.512272557
0.413793628 0.503312814 0.33269807 0.362785525 0.472529258
0.237499883 0.53216187 0.4979401190.456302241 0.325984306 0.172132628
0.132289781 0.390581967 0.530324617 0.404730891 0.555839
0.322482536 0.654235829 0.450305979 0.495425161 0.320923502
0.348554889 0.449032914 0.643964232 0.436208264 0.557421748
0.472588664 0.451554008 0.309949908 0.416106778 0.319531069
0.373022727 0.361592781 0.193762734 0.417837955 0.374902505 -
0.11638443 0.332041894 -0.131515337 -0.058074186
TCGA-97-8179-01A 0.3782470110.350348055 0.466279063 0.368694754
0.294825461 0.514069602 0.280655626 0.4614911630.3644114360.453410697
0.413791856 0.48932168 0.314400166 0.328772109 0.4462117950.211671065
0.537977143 0.423677509 0.363407517 0.330964464 0.121997449
0.144333984 0.321357745 0.513214344 0.3870821170.527267575
0.403007107 0.673703186 0.484914282 0.407316891 0.162864177
0.335899084 0.461733708 0.625053467 0.3811159170.585956308 0.41128875
0.415426582 0.32832913 0.394128092 0.322455129 0.327641075
0.277538326 0.176490887 0.397581637 0.204525955 -0.106863932
0.200387183 -0.158171951 -0.04383206
TCGA-97-8547-01A 0.4344305110.407232983 0.472259705 0.339922191
0.247919581 0.50893493 0.354981584 0.459489662 0.323884657 0.47653496
0.451382486 0.476102567 0.338842646 0.344741658 0.434156619
0.239359756 0.526362573 0.4786071170.431246358 0.363818371
0.195009773 0.184231345 0.345282466 0.529668148 0.386336202
0.543676956 0.34716878 0.643667497 0.444790091 0.562491778 0.27639791
0.309581938 0.398166125 0.5881166030.453319607 0.536967644
0.451427555 0.419882977 0.336757195 0.468020312 0.311348835
0.365148971 0.326991756 0.1681131020.378296092 0.289061086 -
0.13075376 0.309473715 -0.167023092 -0.041074208
TCGA-97-8552-01A 0.420040312 0.370955836 0.458388655 0.27992345
0.244202131 0.490187484 0.375294199 0.450779846 0.217607921
0.470358659 0.401074359 0.482138734 0.341648061 0.369135723
0.439350464 0.216698593 0.505912571 0.425280564 0.387513387

0.323769854 0.149705863 0.121861508 0.337530008 0.491121997
0.388305757 0.470385463 0.256029501 0.615753887 0.382549805
0.401435944 0.251004584 0.333658325 0.431215729 0.612649327
0.379111703 0.522530109 0.453465493 0.413481676 0.318626718
0.378290586 0.324219575 0.328974365 0.321854651 0.205090971
0.395223988 0.299865812 -0.109371177 0.267657604 -0.13367613 -0.095032686
TCGA-97-A4LX-01A 0.469776389 0.398962417 0.469815393 0.351578162
0.257080976 0.499229141 0.452855562 0.452981054 0.354927871
0.507557371 0.391207672 0.461863552 0.29574544 0.328539684
0.441989025 0.212056739 0.540812369 0.577207126 0.536610335
0.344565 0.176689754 0.144285664 0.407694227 0.535413734
0.425785804 0.542680427 0.376161905 0.656388396 0.437248421
0.487601284 0.335049902 0.297913599 0.403217783 0.586622822
0.39996419 0.556942488 0.430061798 0.424658899 0.321638876
0.472495908 0.331306912 0.346801937 0.378296149 0.142295983
0.392531876 0.446372344 -0.118704558 0.294077494 -0.171475524 -0.062802736
TCGA-97-A4M0-01A 0.447122095 0.360688986 0.414215409 0.33687431
0.244925028 0.468213654 0.381163199 0.456107509 0.297904604
0.472635409 0.422751739 0.452809952 0.311332392 0.326020181
0.448338069 0.190168886 0.52671212 0.589212626 0.50034335 0.30936192
0.152606762 0.156033138 0.34387069 0.503652232 0.420431531
0.476934197 0.329814996 0.642003641 0.44413087 0.315180977 0.28218938
0.286435683 0.384497581 0.582371842 0.389060024 0.548466341
0.427014163 0.412330779 0.30249691 0.348504633 0.325698697
0.262909297 0.359467413 0.132237033 0.375529861 0.352150073 -0.143628869 0.261024099 -0.143362817 -0.074185963
TCGA-97-A4M1-01A 0.388191019 0.341169253 0.496143353 0.299929345
0.244080817 0.499271911 0.335209344 0.462921067 0.240040235
0.459050493 0.421430663 0.496648227 0.312362736 0.334968158
0.434860705 0.240996906 0.508216729 0.475390668 0.38711583
0.325570514 0.150204147 0.11085349 0.31829817 0.476665479 0.378522783

0.476050056 0.268639393 0.621767802 0.403133305 0.348448561
0.215494186 0.323650628 0.455452576 0.617448156 0.360776624
0.520093498 0.447662671 0.415468537 0.3179967 0.361280714
0.3190114160.318539937 0.293608053 0.187200885 0.391355756
0.284565949 -0.148035716 0.229853604 -0.139167379 -0.077339544
TCGA-97-A4M2-01A 0.455618363 0.357375076 0.48204766 0.302782888
0.281365476 0.493405507 0.424412155 0.447775435 0.240970856
0.484773797 0.40504907 0.501017756 0.328805915 0.350658201
0.453747689 0.238205602 0.5065661170.509308929 0.469292666
0.327383941 0.158018032 0.103989431 0.364286632 0.502205234
0.404223196 0.500155956 0.260706779 0.623370578 0.414439269
0.36378683 0.299797293 0.339017559 0.462663431 0.613826029
0.342723792 0.564904139 0.463352468 0.431210771 0.323176567
0.374705204 0.32886549 0.312127195 0.340744515 0.175717878
0.390362653 0.370077446 -0.160417046 0.267025816 -0.15543491 -
0.082144536
TCGA-97-A4M3-01A 0.514531801 0.408033328 0.424786622 0.330362263
0.276973755 0.5461193720.313925195 0.450348275 0.291285598
0.465079761 0.421629964 0.497968365 0.352133884 0.349853222
0.413296212 0.212278247 0.516922923 0.38020992 0.344834288
0.294132366 0.140562757 0.177540086 0.320194681 0.528415307
0.396648716 0.5011923230.314597934 0.658137714 0.495767284
0.441216156 0.233493124 0.343498289 0.443834174 0.635049183
0.405083576 0.584759999 0.470075672 0.439513639 0.337891126
0.426238406 0.3311680140.353213562 0.319944175 0.137783222
0.344439649 0.220292702 -0.134660979 0.215631441 -0.123626074 -
0.007322658
TCGA-97-A4M5-01A 0.438330148 0.385465882 0.465302282 0.334136599
0.292199124 0.521655706 0.365008213 0.441895415 0.29093088 0.47014536
0.413847214 0.496920942 0.350345793 0.363787497 0.446519192
0.242126203 0.527434247 0.515512205 0.4474861 0.337981263
0.164490368 0.194644618 0.345510359 0.515046695 0.40714982
0.493578144 0.297808286 0.642465175 0.464083824 0.439602273

0.255168079	0.319043479	0.429629204	0.618539416	0.379893891
0.532225083	0.45553808 0.419014978	0.351480391	0.441732881
0.3215755110.347389783	0.329902218	0.148697184	0.37715733 0.301336628
-0.1275433 0.259339458	-0.121338425	-0.056455936
TCGA-97-A4M6-01A	0.457014526	0.391618554	0.447702338	0.325026305
0.233746789	0.505339229	0.419491456	0.45949683 0.29732543 0.509535898
0.39598003 0.470326193	0.327498479	0.329024449	0.465402078
0.206448732	0.524693196	0.603247648	0.534679471	0.323724892
0.199768397	0.162089588	0.382388462	0.508398538	0.417709675
0.519187585	0.305844127	0.634179329	0.394755629	0.429147215
0.289258697	0.340532778	0.418337581	0.581989968	0.413133129
0.569793769	0.4497112890.408918187	0.325210894	0.358283918
0.324476852	0.322964794	0.362505816	0.161975806	0.397433404
0.374932874	-0.14990876 0.27791107 -0.151228726	-0.080373002
TCGA-97-A4M7-01A	0.487294946	0.403477525	0.452222376	0.337554471
0.248222561	0.510612094	0.425852874	0.451705885	0.302579952
0.499372402	0.40194122 0.455077303	0.316368324	0.340803591	0.4422921
0.220021447	0.528980314	0.502166208	0.473724597	0.344105889
0.163846038	0.158536095	0.385520791	0.513953036	0.411226878
0.519839324	0.328604848	0.630359509	0.3741311860.4453701110.304431696
0.327526949	0.404863669	0.574000122	0.402861458	0.522877254
0.480343815	0.418459437	0.320136774	0.456709703	0.316963984
0.370974488	0.373831824	0.1611079930.370415626	0.378099837	-
0.130932277	0.314062896	-0.127911994	-0.070254954
TCGA-99-7458-01A 0.468347677	0.404577743	0.461300186	0.356086855
0.235208663	0.550575233	0.4051116690.423664999	0.31638012 0.510868322
0.436582606	0.481427338	0.350041375	0.354013948	0.494556716
0.204706132	0.584560813	0.5477221	0.490538676	0.356134589
0.2016114950.197515193	0.394484172	0.519398666	0.402210628
0.533757562	0.326056934	0.657854142	0.407857769	0.483050992
0.3145181130.306532253	0.416476132	0.590624528	0.418790351
0.547016701	0.423987723	0.405470402	0.381475954	0.439709021
0.352513947	0.350881602	0.378776512	0.178870443	0.404519988

0.397009701 -0.124462087 0.33480485 -0.174106366 0.002744483
TCGA-99-8025-01A 0.417373516 0.403139129 0.496964567 0.347653189
0.265832702 0.516970935 0.294740712 0.469342667 0.363435531
0.471963525 0.451422621 0.469702738 0.336620824 0.323620892
0.430522414 0.218472849 0.502187447 0.448995704 0.378307872
0.32735826 0.180493631 0.127407883 0.310040491 0.538327582
0.396216778 0.536051705 0.384739678 0.670343614 0.4838205
0.480203402 0.215406905 0.304436467 0.399990371 0.612391592
0.410610674 0.549046594 0.425494199 0.426133332 0.337141513
0.441114523 0.316278857 0.313524155 0.303196562 0.144749008
0.385347952 0.229253962 -0.081895153 0.225896365 -0.166391988 -0.120787862
TCGA-99-8028-01A 0.582948238 0.452132224 0.453734471 0.307709821
0.273178015 0.522424236 0.475996007 0.456783467 0.310485823
0.540477077 0.395748208 0.469228622 0.33019972 0.347861492 0.44380925
0.233837109 0.517046023 0.566686671 0.554281096 0.347986806
0.241938505 0.148274487 0.427510635 0.539051519 0.413766255
0.536739481 0.334001685 0.650179631 0.407306102 0.5387395
0.393846529 0.304090653 0.403697855 0.61503133 0.408901881
0.557604779 0.472998464 0.445125928 0.327064025 0.484121311
0.319285581 0.374548254 0.418319644 0.150510828 0.37366029
0.469599533 -0.095466645 0.382198766 -0.165613473 -0.054464607
TCGA-99-8032-01A 0.444026785 0.444731376 0.482346169 0.337925962
0.260350276 0.532293749 0.323362364 0.463279553 0.387931128
0.467358238 0.404357681 0.470721616 0.345006907 0.371638896
0.450772232 0.224039388 0.534609355 0.38554201 0.348064046
0.314103776 0.15496195 0.174813208 0.338062152 0.572833609
0.409610125 0.571529408 0.406671628 0.686979036 0.492769437
0.525336572 0.222585574 0.284103122 0.403560553 0.635399599
0.433748219 0.538492108 0.428325672 0.443249213 0.334325952
0.461093031 0.29687454 0.372265716 0.331093968 0.144187803
0.389107032 0.268644497 -0.077289129 0.280889406 -0.161424264
0.108930406

TCGA-99-8033-01A 0.510520718 0.439244408 0.483383497 0.38521906
0.244088019 0.49975817 0.385262737 0.499141714 0.45965756 0.499323349
0.34902288 0.46941942 0.334659301 0.373618326 0.427871063 0.171252123
0.522423213 0.523002092 0.497317775 0.300164213 0.194663938
0.121950317 0.376818765 0.56137479 0.432884465 0.596422855
0.487396994 0.710013297 0.532034399 0.444404578 0.289478215
0.333204156 0.413082082 0.613694212 0.446754352 0.584502149
0.445548343 0.456093451 0.320883915 0.37158781 0.312526241
0.299829785 0.334438175 0.141068609 0.367452128 0.373869174 -
0.070455168 0.245141896 -0.16150075 -0.13182603
TCGA-99-AA5R-01A 0.547826032 0.425840413 0.510457425 0.325048569
0.33317013 0.557529667 0.45054804 0.441486101 0.26274199 0.514937458
0.4445971170.498585619 0.335870839 0.361263512 0.457171627
0.282921949 0.487029609 0.522779918 0.486259724 0.366916405
0.240948743 0.179787545 0.4111872630.500149925 0.410316334
0.491366136 0.271421657 0.615922204 0.375458774 0.458710667
0.341399805 0.312964807 0.422401712 0.59523147 0.346528885
0.545056685 0.460916916 0.435937392 0.375639838 0.465478357
0.329295237 0.365204052 0.392132949 0.16237727 0.355001214
0.403582241 -0.158643223 0.32386008 -0.16324758 -0.083119131
TCGA-J2-8192-01A 0.527044632 0.456663569 0.459198683 0.348770255
0.247817821 0.558052034 0.396409434 0.416964794 0.292058241
0.529617136 0.461009852 0.476249242 0.3431555 0.340289535
0.506865814 0.222155379 0.575322921 0.519522608 0.491579997
0.375737401 0.232970266 0.221998692 0.388272612 0.506187245
0.41245694 0.533273384 0.303668599 0.630876468 0.350602995
0.593221318 0.350350418 0.3112574050.418602726 0.575432635
0.442012091 0.525397808 0.466122961 0.4115964450.4114904970.507093427
0.336169743 0.383227694 0.370253151 0.156893873 0.371240983
0.35847139 -0.139603007 0.362614952 -0.140529996 -0.086578281
TCGA-J2-8194-01A 0.494713702 0.422446866 0.435263609 0.321941517
0.283043471 0.543849168 0.374284655 0.426338494 0.268294205
0.48246454 0.36812012 0.503323275 0.338570992 0.331892676 0.462371034

0.18565662 0.5508722110.406942606 0.391889799 0.315397189 0.137095837
0.153737298 0.36042252 0.533105641 0.40680883 0.533562609 0.28762886
0.664730502 0.464331082 0.450335518 0.28024494 0.338318436
0.440730674 0.622844909 0.402928798 0.569782513 0.446531944
0.436945956 0.345251658 0.442038191 0.327865158 0.338349704
0.340002787 0.174716832 0.395251777 0.274645334 -0.133129021
0.276355217 -0.162645811 -0.022935618
TCGA-J2-A4AD-01A 0.394806319 0.399835971 0.469923206 0.375830171
0.217584065 0.503541929 0.227691616 0.463651689 0.398720907
0.469691505 0.387039659 0.467054739 0.320543819 0.324292983
0.460048122 0.203922802 0.559142166 0.407274126 0.337770244
0.325472027 0.171722348 0.109863136 0.292829585 0.52647546
0.390491864 0.544615616 0.42502927 0.674480775 0.418062294
0.446210002 0.153240081 0.300384367 0.428412489 0.60155022
0.450304468 0.549560431 0.429233545 0.412002579 0.33543813
0.340006287 0.316189938 0.305602441 0.259751054 0.149032645
0.373074359 0.185737056 -0.06444229 0.196125018 -0.122246476 -
0.199526522
TCGA-J2-A4AE-01A 0.52787054 0.399054004 0.467600054 0.333888153
0.213362286 0.525165396 0.391078373 0.480067513 0.351214321
0.5112199530.356266268 0.470380803 0.362132409 0.372003249
0.490349638 0.193027521 0.534551519 0.509215957 0.460073828
0.2816242110.135001434 0.087461849 0.333699601 0.548877459
0.423995006 0.540746505 0.400632748 0.671067336 0.419302686
0.319862545 0.300828659 0.332168742 0.449341853 0.613918332
0.39200526 0.501003145 0.484344184 0.458759631 0.317300745
0.3116893970.329012441 0.303828802 0.368740384 0.191426416
0.3866011140.33118169 -0.104383373 0.264848381 -0.13764226 -0.102877475
TCGA-J2-A4AG-01A 0.583470467 0.419947734 0.455067959 0.326850885
0.256302309 0.550541684 0.422850855 0.451766914 0.2902223
0.524235705 0.4078118910.469308633 0.364214717 0.350828271
0.470768101 0.201715151 0.537304359 0.502057395 0.474564643
0.336005196 0.170394147 0.178470405 0.379251503 0.52803731

0.427201528 0.509429865 0.320298481 0.642741021 0.426210831
0.455424346 0.336004444 0.288910907 0.390512944 0.5858824
0.408703909 0.519206906 0.46720832 0.429579278 0.365896279
0.430180834 0.339499767 0.326780062 0.386885798 0.160668947
0.379477181 0.368256432 -0.125881841 0.31190978 -0.145338969 -
0.080933304
TCGA-L4-A4E5-01A 0.3861511590.3615461110.463051731 0.405073099
0.240203007 0.445655481 0.292332982 0.500971001 0.464319395
0.441748934 0.369122532 0.454164473 0.310324433 0.322215002
0.408464103 0.179992856 0.5166119460.493132178 0.416975802
0.321392667 0.178534026 0.122283415 0.29322861 0.547277455
0.432697576 0.557973819 0.51541176 0.685370945 0.506848076
0.378738445 0.185381582 0.262008383 0.384038347 0.585316064
0.435085548 0.5112664680.4321463 0.414757852 0.286180152 0.336760641
0.314876915 0.259722145 0.281418643 0.139352547 0.36671138
0.249120475 -0.029540967 0.180677213 -0.165249221 -0.160350455
TCGA-L4-A4E6-01A 0.529320934 0.399750695 0.455573476 0.319060992
0.279808768 0.544484772 0.458521392 0.431014682 0.246649418
0.509962608 0.417069982 0.519245085 0.3160841110.342306348
0.452891603 0.241920698 0.527261534 0.526212495 0.479366832
0.336730473 0.185174878 0.130661876 0.397464196 0.485757157
0.406728375 0.48977373 0.25248861 0.606418956 0.368686028 0.430906581
0.363877463 0.320136973 0.43761224 0.610467791 0.339825699
0.547187482 0.464852708 0.434260368 0.353060578 0.437293411
0.344538537 0.350946835 0.386638061 0.187263646 0.38436615
0.373773945 -0.151919829 0.311604709-0.167914503 -0.056035383
TCGA-L9-A443-01A 0.483286793 0.39922833 0.463085744 0.306628556
0.275479418 0.539639719 0.337244089 0.460428726 0.300805889
0.484492904 0.415923571 0.458503856 0.335995952 0.346448261
0.435016456 0.22623117 0.519915778 0.483282853 0.418129174
0.316124891 0.198396407 0.1174845140.338650005 0.49319382 0.407515866
0.513108796 0.332232138 0.651014804 0.4115462380.487290613
0.248720513 0.285309813 0.406827768 0.599388345 0.405754816

0.5243909110.461668626 0.416134695 0.331297617 0.445301037 0.31023651
0.347272727 0.340718179 0.144568466 0.389782531 0.26287431 -
0.071540428 0.278723836 -0.119478999 -0.02645603
TCGA-L9-A444-01A 0.467008544 0.394826256 0.508872199 0.342935962
0.285272587 0.474734604 0.422429057 0.461697916 0.345112303
0.501586087 0.4011937760.4665117060.307436409 0.330290149 0.435580249
0.208053728 0.505085646 0.565045045 0.533424903 0.340416811
0.189328789 0.130967693 0.386532971 0.531830886 0.432846107
0.532449749 0.368789275 0.645282818 0.437030846 0.435882222
0.313192991 0.301231623 0.407449296 0.602492548 0.402370994
0.532720681 0.446842029 0.420153257 0.315373377 0.410376389
0.316032424 0.3112362160.371843991 0.159626933 0.37657317 0.438553935
-0.109963251 0.279036659 -0.169664656 -0.070114578
TCGA-L9-A50W-01A 0.408848839 0.380868584 0.4804141190.302853005
0.270672377 0.510386064 0.35601744 0.456457929 0.282198299
0.463305844 0.405005885 0.4589011070.322234846 0.346667416
0.440424605 0.216861962 0.4831185510.469343697 0.402194528
0.321087713 0.149861517 0.184250962 0.3290437110.492359724
0.390506556 0.50753079 0.299489645 0.613672043 0.391221205
0.378070873 0.233954072 0.3241133830.414612742 0.574276821
0.399029454 0.528489227 0.442240232 0.421435148 0.287046255
0.398796745 0.3112378090.350923823 0.330365726 0.196204467
0.407629908 0.284001665 -0.1191395 0.255899095 -0.137297136 -
0.078018591
TCGA-L9-A5IP-01A0.540883218 0.451666627 0.4456700110.39032518 0.28234141
0.550928454 0.323846565 0.476682792 0.430343106 0.481539017
0.378347635 0.483240061 0.374704006 0.379369186 0.446284929
0.159100715 0.543502555 0.461773152 0.410381512 0.305481846
0.227315918 0.155194453 0.370014805 0.57126626 0.41547179 0.568940223
0.441302716 0.69757608 0.515099866 0.432273254 0.2588411920.325829216
0.413712387 0.633234362 0.446898042 0.603813845 0.479073124
0.459044861 0.323052381 0.40354571 0.298784701 0.306821868
0.301824377 0.161643329 0.380521341 0.265753528 -0.096204356

0.244415173 -0.139388178 -0.130752505
TCGA-L9-A743-01A 0.582976742 0.437451583 0.501233132 0.362827175
0.251290261 0.516190012 0.464890686 0.452870179 0.375349231
0.535735044 0.405228091 0.47536619 0.369095822 0.385845777
0.477758621 0.207267471 0.516359723 0.551763599 0.523997853
0.353187964 0.236235254 0.134706556 0.414003251 0.547437058
0.416246124 0.551887481 0.398536212 0.673387489 0.475280331
0.503675652 0.397968574 0.312818474 0.40552131 0.599869433 0.42028898
0.544954827 0.475722756 0.448066834 0.34382658 0.479245837
0.318547054 0.380918421 0.420501282 0.156534109 0.384200132
0.419436598 -0.096846692 0.354374822 -0.154880442 -0.07243892
TCGA-L9-A7SV-01A 0.339796858 0.326383188 0.453739466 0.396699383
0.232429339 0.444310591 0.237701461 0.4977118030.427681726
0.4073119740.390487561 0.461812109 0.289417002 0.279601581
0.390068469 0.163668612 0.493553269 0.377967218 0.3027117620.28589069
0.048531204 0.077447267 0.232055966 0.5275588 0.412861021
0.507504289 0.466731761 0.686124173 0.508063829 0.27517148 0.12246603
0.260509238 0.389985619 0.613357329 0.367912852 0.47278018
0.390519781 0.390176863 0.287638866 0.29702681 0.310423874
0.191988253 0.252053394 0.152063175 0.375136061 0.17682823 -
0.054957634 0.104209849 -0.177294704 -0.149097523
TCGA-L9-A8F4-01A 0.428226104 0.390462757 0.47296249 0.377456604
0.266075779 0.481588828 0.358126905 0.476354984 0.434036096
0.473651298 0.374061674 0.435906871 0.324373549 0.34237988
0.4185691110.2282110410.488230334 0.420152097 0.399756855 0.314312368
0.172721792 0.11236508 0.3315117280.55441119 0.416034747 0.539909614
0.454382937 0.699723029 0.513582045 0.430543052 0.24634601
0.268243549 0.374488994 0.594763639 0.393586634 0.53976146
0.434751401 0.429982071 0.300552883 0.340581448 0.290430958
0.307293715 0.328524026 0.1117745080.358763757 0.368962734 -
0.07015613 0.216828592 -0.099043278 -0.173844791
TCGA-MN-A4N1-01A 0.372869802 0.364308419 0.47334542 0.375266717
0.221740659 0.480071363 0.232084197 0.493705297 0.422536707

0.439397047 0.380928242 0.446816287 0.32556299 0.344355617
0.415584796 0.209053047 0.499637472 0.340024001 0.312578656
0.315730651 0.149973271 0.149835098 0.270912054 0.529364322
0.393978526 0.536363966 0.454708584 0.66568136 0.488481375
0.430427968 0.166108216 0.269168024 0.388329971 0.615471223
0.436264228 0.521593007 0.416908216 0.406018801 0.273957678
0.347159246 0.287274319 0.305371765 0.262037896 0.13926087
0.366664935 0.193127697 -0.035656539 0.19194108 -0.153009866 -
0.158114063
TCGA-MN-A4N4-01A 0.437204084 0.43662746 0.444183536 0.323764577
0.2861115880.531425662 0.30897322 0.443661836 0.333993258 0.473339672
0.40452771 0.429664569 0.350428126 0.342999504 0.4348077110.239500707
0.510131326 0.384843732 0.367994204 0.355992882 0.198646464
0.166797001 0.327530588 0.532695048 0.387635772 0.511538643
0.357137499 0.644819302 0.460286538 0.525438069 0.218846996
0.279377753 0.346161658 0.557736249 0.433652665 0.517465572
0.446159171 0.419744225 0.309152478 0.3911262260.300406873
0.326053506 0.284150205 0.1145810750.335564977 0.270447852 -
0.081742909 0.251400375 -0.168230156 -0.153422461
TCGA-MN-A4N5-01A 0.448079549 0.384976223 0.461008917 0.372755899
0.224073922 0.489666207 0.382530143 0.464551915 0.391501259
0.4732781180.373062321 0.445724926 0.330893946 0.349232071
0.3974119270.1988113590.498330735 0.569249209 0.491463493 0.331329941
0.196584612 0.159902099 0.336190704 0.516931807 0.415122075
0.521386398 0.413969325 0.668095322 0.469817232 0.360260951
0.248056789 0.273081355 0.375967663 0.575157429 0.408312775
0.513144766 0.4401141540.4277739 0.299406546 0.342950689 0.295771521
0.288499531 0.327448819 0.150656159 0.386445931 0.339924157 -
0.089081802 0.236085554 -0.15820514 -0.081261914
TCGA-MP-A4SV-01A 0.4691891150.393564889 0.483868569 0.403795512
0.27522897 0.490637263 0.378095007 0.488480414 0.469269523
0.499125945 0.381779661 0.454690494 0.332545743 0.365050261
0.458984453 0.176622528 0.520807781 0.563261071 0.486756589

0.31903418 0.195162633 0.126819474 0.352675735 0.568173378
0.441415822 0.569586674 0.490565158 0.7114594280.542274506 0.43055226
0.278294965 0.274779363 0.402487474 0.605125907 0.426904264
0.532476226 0.438652903 0.438715636 0.319149148 0.393745017
0.312869301 0.314394997 0.351584296 0.13857687 0.385790913
0.356005004 -0.074580405 0.255955768 -0.171802656 -0.098693167
TCGA-MP-A4SW-01A 0.460460582 0.39322388 0.506554142 0.341427796
0.218690523 0.459184881 0.385365432 0.464586999 0.35280174
0.489334389 0.383820461 0.484305043 0.314934558 0.344081922
0.463559046 0.205128633 0.512327133 0.524829783 0.453697736
0.3233136110.138890758 0.134149868 0.347599136 0.526535485
0.421314774 0.56253437 0.377919489 0.642318175 0.446153786
0.404874213 0.289329792 0.330864398 0.442784533 0.605735716
0.4326446 0.557547446 0.465588473 0.435808131 0.294822832 0.40866312
0.323667589 0.326240961 0.339418291 0.180704783 0.410332945
0.348626043 -0.103998468 0.261500421 -0.153292575 -0.105971417
TCGA-MP-A4SY-01A 0.463057584 0.430234441 0.444123724 0.352753448
0.27415729 0.526159306 0.314548037 0.469377584 0.359389438
0.490964192 0.415103952 0.462426177 0.34231986 0.334917985
0.438082802 0.209482023 0.555436186 0.471609185 0.413962476
0.335392459 0.177076635 0.180129292 0.334142026 0.554370538
0.397914039 0.556412171 0.383582747 0.680775802 0.495114211
0.544274586 0.254569533 0.303999063 0.398943075 0.588089288
0.443904343 0.550016879 0.472561278 0.43600486 0.341033078
0.499521301 0.310225446 0.359816568 0.316548712 0.141766164
0.38848013 0.257087068 -0.127054987 0.30609109 -0.178110587 -0.062152672
TCGA-MP-A4T4-01A 0.49935136 0.41830115 0.468764549 0.386041262
0.251755931 0.520754261 0.412994764 0.468658386 0.410177196
0.521696721 0.39505477 0.434977542 0.318999944 0.340637232
0.446607052 0.212234631 0.5187011080.612551698 0.556270274
0.359162955 0.214480465 0.144714536 0.3849110290.561252299
0.427642129 0.547550334 0.431237229 0.679730064 0.477307288
0.514578233 0.326553607 0.275451521 0.384242232 0.584632209

0.434935977	0.547267073	0.456174731	0.439069351	0.333882977
0.427728621	0.3052548110.340076397	0.379514607	0.1141252440.365669037
0.42965915 -0.066195255	0.309965518	-0.162807087	-0.11940962
TCGA-MP-A4T6-01A	0.374889405	0.313666756	0.434185923	0.289953195
0.183185374	0.433074895	0.3321825110.477320279	0.268828135
0.437758469	0.378242996	0.42322059 0.297014304	0.317491977
0.381557154	0.156394604	0.476000538	0.514494314	0.436629684
0.275500134	0.099207256	0.136144617	0.303106673	0.492944821
0.391293469	0.464290489	0.303947283	0.60810516 0.410505341
0.251885037	0.204471077	0.267543096	0.392944702	0.567591985
0.375032625	0.493570467	0.433087413	0.392812044	0.242205952
0.231474362	0.300553668	0.263864025	0.300364967	0.147984987
0.36926569 0.269777277	-0.121662904	0.187391019	-0.132256354	-
0.133602469
TCGA-MP-A4T7-01A	0.503597927	0.437808158	0.542550276	0.364841331
0.283858481	0.49430815 0.346609932	0.476534254	0.384303439
0.481392651	0.387282525	0.505151607	0.37325567 0.396856958
0.4544313110.199606902	0.509724974	0.456587966	0.398207354
0.304124596	0.136926602	0.107205391	0.330900529	0.538887604
0.413983959	0.552701808	0.400888994	0.666166985	0.490021786
0.365524583	0.259181682	0.354479467	0.451218863	0.648465386
0.433050149	0.578671515	0.482409024	0.440382631	0.324169537
0.35049164 0.31337407 0.3203249	0.342266705	0.171530377	0.388940006
0.282874778	-0.120029212	0.256160367	-0.152653075	-0.09209179
TCGA-MP-A4T8-01A	0.43593278 0.414160376	0.438833185	0.363916282
0.313921817	0.514065719	0.257824248	0.454790839	0.409428565
0.447512463	0.385966008	0.468968598	0.373436478	0.375067501
0.445469719	0.21207173 0.5131169220.461851777	0.377562649	0.317220994
0.191961512	0.193542927	0.2936781140.557267563	0.412233471
0.541629047	0.413731572	0.697133337	0.53489024 0.486334864
0.189830382	0.284800777	0.394952284	0.613027132	0.436980537
0.521640275	0.412426059	0.421714379	0.30873577 0.399171527
0.308217074	0.331943158	0.293944682	0.120425568	0.386142275

0.191865333 -0.098554653 0.196704974 -0.178354936 -0.052153478
TCGA-MP-A4T9-01A 0.504705617 0.451854832 0.486816423 0.370314812
0.257547058 0.558381804 0.381062036 0.448051224 0.350877993
0.498525499 0.42590177 0.487954723 0.353887543 0.374183764
0.463805817 0.247588174 0.52590435 0.448179874 0.41913744 0.383023194
0.225590064 0.188330302 0.37134 0.531560049 0.406690621 0.549950557
0.362894462 0.653694753 0.436184422 0.503575764 0.31055441
0.301428168 0.413593906 0.589976328 0.445601838 0.504905483
0.456742127 0.442617297 0.365037219 0.454975858 0.324216
0.390646249 0.367549235 0.141054728 0.387617092 0.310696414 -
0.1102728720.334322204 -0.142463196 -0.096549865
TCGA-MP-A4TA-01A 0.469950293 0.365534683 0.430539015 0.375499082
0.232719165 0.43057997 0.339920697 0.510000995 0.456018566
0.480784351 0.338687153 0.4614117170.3146204 0.345121769 0.41381258
0.15464581 0.487914238 0.570208957 0.493763204 0.286953146
0.161705346 0.035365316 0.368377945 0.561824367 0.405325714
0.56319069 0.4990114310.701962007 0.5511941910.397202922 0.229930013
0.338667636 0.41391922 0.633952024 0.428906076 0.562829252 0.43655888
0.425684466 0.255072673 0.362967266 0.285371671 0.324727304
0.290531405 0.156300635 0.3711979920.309422875 -0.050838572
0.216023984 -0.17345281 -0.126377314
TCGA-MP-A4TC-01A 0.507406789 0.448051088 0.48382253 0.387168663
0.289550414 0.525435999 0.410584643 0.465957255 0.423392595
0.509025713 0.431095742 0.450627363 0.338220026 0.3527896
0.455644539 0.219035028 0.518289749 0.554451994 0.49973839
0.369841086 0.221509688 0.146153936 0.377349992 0.54495823 0.4044477
0.561885054 0.44927668 0.678279152 0.490983286 0.575452835
0.3254116380.289725992 0.386214048 0.5924925110.454541247 0.54104421
0.4501152850.4311812180.362071892 0.437271301 0.296749879 0.348777612
0.359217388 0.140570485 0.362631613 0.362279569 -0.099853717
0.291890822 -0.172916176 -0.089635311
TCGA-MP-A4TD-01A 0.487695431 0.419056273 0.501038275 0.319486936
0.302844299 0.494529821 0.3168246 0.460878259 0.323463244

0.476683546 0.430171464 0.4736841160.35688741 0.373025091 0.44882328
0.251775049 0.477278299 0.398235617 0.3731117270.338072073 0.19698896
0.192714972 0.345362576 0.532919231 0.3946281190.533508902
0.351748594 0.648162166 0.487830742 0.517127364 0.23853856
0.338140553 0.437105294 0.617758128 0.402002002 0.588357096
0.455886599 0.443656508 0.300847658 0.448748855 0.324264962
0.36137924 0.33220682 0.161628195 0.378355844 0.287998866 -0.130825944
0.276304151 -0.15910591 -0.003390501

TCGA-MP-A4TE-01A 0.364299202 0.379526786 0.467596962 0.329681576
0.279079812 0.465331898 0.23874321 0.462228504 0.388543567
0.419631945 0.394176559 0.469989638 0.370318296 0.3949494
0.422320318 0.194079545 0.523858137 0.304125004 0.262983509
0.247478248 0.105040472 0.128473541 0.300189618 0.54995408
0.410149488 0.564388856 0.405768615 0.676525999 0.527416495
0.341433406 0.140321825 0.310865236 0.443645518 0.640383445
0.426799241 0.570451621 0.418968822 0.449267068 0.243179892
0.389021538 0.320420007 0.309687127 0.2717261160.162068815
0.403472847 0.155553029 -0.054463095 0.191394401 -0.161319944
0.1252217

TCGA-MP-A4TF-01A 0.466876294 0.431415906 0.450813035 0.38493202
0.295998506 0.559620662 0.313894473 0.501018796 0.431295553
0.46549093 0.359545456 0.428360517 0.312095576 0.335715009
0.441610585 0.13603124 0.565966737 0.508606712 0.436962137
0.301857468 0.1721199230.1176216960.332796895 0.574460052 0.423787544
0.560270455 0.440804264 0.691726205 0.468978342 0.421183879
0.229368607 0.259208837 0.363063206 0.59302461 0.459831758
0.503084502 0.426729289 0.426091833 0.318761684 0.397002981
0.300765404 0.276639644 0.300732007 0.123232692 0.366863148
0.244152333 -0.087150297 0.241818836 -0.186868311 -0.100684669

TCGA-MP-A4TH-01A 0.4494115350.353106412 0.469589163 0.334814434
0.270755575 0.531793074 0.399829864 0.442759814 0.272377225
0.47928004 0.415784369 0.456882286 0.330791247 0.322801216 0.4670256
0.21684393 0.529517845 0.499133145 0.460171573 0.337286321

0.175013273 0.156287591 0.35967081 0.519296487 0.418224278
0.488768671 0.278078784 0.62952977 0.384774477 0.383414714 0.27804903
0.285033012 0.406882963 0.5861482110.366668841 0.527194146
0.446057368 0.406635182 0.334852287 0.34095624 0.326508943
0.304384108 0.369224802 0.144285904 0.362133442 0.38379941 -
0.157856581 0.266732053 -0.144695673 -0.083753839
TCGA-MP-A4TI-01A 0.555923141 0.431028969 0.432248849 0.401612389
0.27796872 0.540204922 0.50515555 0.48238046 0.454934175 0.544026593
0.421621872 0.455592767 0.29971678 0.342548843 0.426749632
0.218658704 0.493706497 0.651655067 0.593364752 0.372703211
0.257682595 0.183408016 0.42997239 0.529314974 0.426430522
0.539942414 0.476830482 0.689489752 0.497499963 0.53565634
0.390285834 0.285289745 0.372757567 0.585024771 0.413557125
0.55814712 0.471290474 0.428473153 0.3394113350.4681179630.29374519
0.357291094 0.4112256740.121096019 0.349752545 0.4737352 -0.115369615
0.33958104 -0.183897484 -0.102097923
TCGA-MP-A4TJ-01A 0.594993586 0.436701782 0.4711525910.346475304
0.246972984 0.516903567 0.49408307 0.450767446 0.3566174110.53008745
0.377541306 0.445674841 0.362130303 0.389272859 0.477666624
0.18419023 0.533300536 0.576288183 0.559467714 0.327064657
0.204934888 0.134172294 0.403137461 0.538220278 0.418557093
0.550333936 0.371683392 0.664564845 0.445415633 0.495787867
0.387179008 0.299379441 0.394171753 0.590207428 0.389453774
0.526960821 0.467378406 0.433703981 0.348427517 0.407356506
0.3110987980.344772988 0.4135881130.161904555 0.371372486 0.460508686
-0.08184077 0.330519629 -0.144595747 -0.067860113
TCGA-MP-A4TK-01A 0.489952895 0.478066961 0.4718117840.384850664
0.289964319 0.57541277 0.386822205 0.469588703 0.394408044
0.505335632 0.440043886 0.468907308 0.332108832 0.352088464
0.472481315 0.255169452 0.530288636 0.436840606 0.42055655
0.390818852 0.252796439 0.204550722 0.379425899 0.539379655
0.406914035 0.53888863 0.416738129 0.660652972 0.454528595
0.602857286 0.315062231 0.308799071 0.386387567 0.582241965

0.457195761 0.521394077 0.446041943 0.425239398 0.379215357
0.477078097 0.306174996 0.363402105 0.375084722 0.125738304
0.360128801 0.356246522 -0.090818267 0.341389511-0.152870638 -
0.085251337
TCGA-MP-A5C7-01A 0.3511481190.336247214 0.457256387 0.31689435
0.279034082 0.49971347 0.227883624 0.453775479 0.3088114990.406690059
0.362308763 0.473276863 0.320750201 0.336605436 0.430958483
0.17627354 0.534713687 0.35401884 0.287402403 0.257299736 0.086289103
0.133472826 0.260305297 0.540852334 0.392420734 0.510127804
0.330880694 0.649124822 0.432884277 0.279614044 0.131889226
0.31764136 0.456073216 0.606440093 0.373838717 0.537881665
0.419405803 0.418499383 0.290794393 0.326165681 0.333316302
0.257454255 0.258260237 0.162073743 0.373248063 0.158589793 -
0.1115899620.157482636 -0.143641338 -0.088326555
TCGA-NJ-A4YF-01A 0.33850444 0.418263786 0.449616979 0.338783323
0.1994111280.420935875 0.25384282 0.492673375 0.392496257 0.402299802
0.296741575 0.463439335 0.325479623 0.314392697 0.404006293
0.186602045 0.49850447 0.325595715 0.29339387 0.26243987 0.165864776
0.072791505 0.26583111 0.547366142 0.397202008 0.555627442
0.423806789 0.680607171 0.509107223 0.35257601 0.147798518
0.258989942 0.400604915 0.698274979 0.430880367 0.530985082
0.36976336 0.436962034 0.234408799 0.360550306 0.30011138 0.253284578
0.279276034 0.1337731190.357398854 0.185293948 -0.044946268
0.132257233 -0.16267257 -0.155655745
TCGA-NJ-A4YG-01A 0.453552703 0.365343131 0.477774366 0.297642183
0.246176713 0.473635399 0.3510805110.472946368 0.302271886
0.484459278 0.4063554110.468597688 0.335375204 0.360093898
0.441551308 0.198238215 0.504576978 0.516264445 0.434099562
0.30471567 0.148530174 0.156656133 0.350627225 0.5333979110.393480139
0.5157011790.317551849 0.658046561 0.490356238 0.436399056 0.25919966
0.304465652 0.414334764 0.6110935420.389883597 0.556640297 0.45917354
0.42473996 0.313437939 0.440986779 0.324368642 0.360035433
0.3351114910.165544081 0.392730602 0.304785076 -0.150774002

0.265164478	-0.151103617	-0.054137092
TCGA-NJ-A4YI-01A	0.409400747	0.337867341	0.450814979	0.320623789	0.246784937	0.517959765	0.332241045	0.46769534	0.28776573	0.475361432	0.431325869	0.468829019	0.293053569	0.331206445	0.414140713	0.193411151	0.507465529	0.479990804	0.403572189	0.340341294	0.121202375	0.117545447	0.324169403	0.508392282	0.388134369	0.491190081	0.31960353	0.639277907	0.44662239	0.419757776	0.225399147	0.300057609	0.395903435	0.588871462	0.379075798	0.536623016	0.442269159	0.408565357	0.285339938	0.435059004	0.297510248	0.362212556	0.298310525	0.150961113	0.377674292	0.249305227	-0.132090746	0.233755224	-0.157015803	-0.064307575
TCGA-NJ-A4YP-01A	0.468213353	0.406618618	0.509034264	0.346133075	0.29638249	0.489694215	0.389279712	0.469406305	0.374691038	0.475605398	0.444707898	0.475687825	0.344837252	0.364344014	0.419440987	0.182016773	0.500670755	0.475973187	0.430487455	0.331626166	0.196867694	0.128445207	0.343581957	0.550044277	0.400090355	0.541026589	0.388909773	0.684903833	0.527405904	0.515271719	0.262929082	0.283035252	0.402451058	0.617843307	0.4238266	0.532377912	0.45491638	0.434906495	0.331398769	0.440674394	0.292537023	0.329898586	0.329709599	0.124070376	0.373183179	0.302549729	-0.141953115	0.251174453	-0.160872523	-0.094776026
TCGA-NJ-A4YQ-01A	0.400238643	0.374897969	0.472472027	0.339900855	0.231437561	0.430726109	0.384212093	0.486828488	0.387425883	0.474070191	0.341382305	0.456065266	0.320744291	0.314308327	0.436342499	0.155771705	0.528015772	0.516481	0.473723892	0.290807637	0.182095315	0.058617713	0.359744906	0.563445382	0.435163786	0.572109137	0.397728518	0.672916157	0.460395699	0.347536586	0.242248649	0.30559205	0.411059851	0.611372121	0.416952952	0.583987537	0.414797253	0.399191777	0.264594904	0.284844204	0.311880617	0.269827456	0.33465904	0.168489956	0.381669259	0.400177706	-0.096834303	0.229430722	-0.175133255	-0.166930699
TCGA-NJ-A55A-01A	0.44594054	0.367786806	0.422417319	0.31372883	0.284326333	0.507113024	0.376172343	0.44780465	0.268252525	0.482178129

0.4119436380.447307202 0.340065972 0.34496635 0.446733805 0.225685898
0.50576995 0.484222282 0.44105053 0.338432466 0.180713477 0.151675686
0.331033831 0.493167431 0.392974753 0.479475496 0.286370165
0.640670367 0.387290714 0.433154374 0.252585521 0.304985717
0.41036936 0.58109624 0.352956236 0.512596628 0.448413756 0.398471636
0.360262087 0.35992461 0.318281536 0.318777047 0.334621709
0.176356883 0.380167748 0.343948792 -0.096836681 0.2458988 -0.118991671-0.041921363

TCGA-NJ-A55O-01A 0.461449602 0.398071881 0.493084145 0.338003703
0.270943457 0.489660328 0.350627739 0.445646918 0.314490489
0.46653666 0.394933564 0.490498688 0.36842742 0.385139064 0.466763034
0.217103989 0.538525334 0.492887707 0.414061817 0.314288026
0.154391668 0.138795021 0.347414531 0.514384796 0.397481692
0.516460623 0.315950654 0.638827203 0.44230552 0.362237213
0.261272709 0.317088652 0.444537349 0.63933257 0.412386985
0.547032986 0.448021697 0.436489613 0.336916809 0.358228301
0.331343583 0.331054203 0.349103652 0.181439766 0.380689562
0.281660955 -0.131936227 0.272634793 -0.145601694 -0.04128963

TCGA-NJ-A55R-01A 0.346858628 0.392317098 0.435508438 0.325193077
0.281369061 0.469935151 0.220964826 0.445703159 0.330480787
0.450505843 0.361326776 0.482622667 0.330277027 0.360689211
0.419256333 0.215705588 0.509295963 0.333351615 0.295509515
0.303177936 0.0984991120.19137246 0.30501536 0.531070292 0.388948984
0.539220087 0.353009869 0.652253586 0.442952172 0.423639008
0.1179404120.356928949 0.461395258 0.645881849 0.428789034
0.579273102 0.428058046 0.432585068 0.283649431 0.443339085
0.337200397 0.337892074 0.293666286 0.2102547 0.417500915
0.184703567 -0.038189236 0.230867651 -0.126529066 0.225714034

TCGA-NJ-A7XG-01A 0.403712398 0.36706609 0.472596898 0.312271997
0.290129668 0.485953162 0.27320194 0.468617297 0.276804199 0.45968764
0.394645387 0.4591171010.343605591 0.341224028 0.459027229
0.181613776 0.5184114260.43245096 0.360938859 0.293605483 0.128434744
0.165133767 0.271023138 0.520952342 0.391057913 0.504307492

0.291561723 0.645217187 0.4675511410.275219926 0.160864328 0.2857778
0.419707246 0.618193366 0.419343186 0.518954823 0.455151647
0.419506359 0.315381876 0.293882991 0.325440581 0.282210952
0.288264163 0.155129307 0.404693718 0.187140825 -0.144564317
0.190162513 -0.131795739 -0.107269346
TCGA-O1-A52J-01A 0.427160197 0.393166687 0.472821748 0.332673734
0.189568382 0.471889425 0.365617347 0.438631 0.2926118950.484544171
0.379260741 0.487926481 0.32540111 0.323214298 0.444628093
0.220793154 0.525947487 0.49920808 0.432235162 0.337520437
0.141675403 0.141749826 0.343382258 0.489323035 0.408594188
0.525372094 0.314335054 0.621627946 0.405002697 0.375955653
0.273816579 0.333949584 0.442215477 0.601403007 0.407247304
0.533342314 0.453002843 0.421472783 0.310322317 0.354482186
0.321626367 0.315808502 0.33206182 0.21392868 0.38805648 0.310763966 -
0.1150166630.254236246 -0.122027509 -0.104973183
TCGA-S2-AA1A-01A 0.520782776 0.40745905 0.475088894 0.301006383
0.2581171150.537736573 0.422591871 0.448821348 0.24438002 0.503832188
0.4142115620.448639131 0.335006207 0.342233656 0.457427268
0.220516081 0.525098529 0.491699642 0.469176476 0.352690785
0.209671898 0.182975057 0.380306309 0.524763503 0.384015702
0.512790844 0.257870661 0.615406128 0.383498508 0.500037925
0.303640713 0.300034595 0.4058147110.583208535 0.402176293
0.528828017 0.470228491 0.433638176 0.355292322 0.433665671
0.32159447 0.365459901 0.40229895 0.151049159 0.34782999 0.394639048 -
0.1311078770.335848002 -0.131651523 -0.071736148

Table S5. Immune infiltrating cell ratio analysis

sample B_cells_naive B_cells_memory Plasma_cellsT_cells_CD8 T_cells_CD4_naive
T_cells_CD4_memory_resting T_cells_CD4_memory_activated T_cells_follicular_helper
T_cells_regulatory_(Tregs) T_cells_gamma_delta NK_cells_restingNK_cells_activated
Monocytes Macrophages_M0 Macrophages_M1 Macrophages_M2
Dendritic_cells_resting Dendritic_cells_activated Mast_cells_resting Mast_cells_activated

Eosinophils Neutrophils P_value Correlation RMSE

TCGA-05-4249-01A 0.001625858 0.0229511170.086562315 0.031896769 0
0.087610438 0 0.034167194 0.029264314 0 0 0.007037527
0.010705382 0.005483606 0.040342645 0.11973396 0.344667414
0.010612958 0.14337803 0 0 0.023960473 0 0.456465678 0.890636198

TCGA-05-4250-01A 0.022547204 0 0.030851659 0.150543782 0 0.145657926
0.0745112060.052876025 0 0 0 0.062654187 0.0059031180.0539129
0.1007549110.205370604 0.048156033 0 0.032475363 0 0.002612367
0.0111727140 0.350850272 0.939528979

TCGA-05-4382-01A 0.002240718 0 0.129303319 0.041833925 0 0.096367568
0.040325686 0.000520316 0.043434433 0 0.002865619 0 0
0.227329381 0.077270744 0.303815887 0.016432878 0 0 0 0
0.018259526 0 0.468934954 0.882817862

TCGA-05-4384-01A 0 0 0.037452991 0.04405186 0 0.160195612 0.001735026
0.010917036 0 0 0.024036661 0.00485699 0.008741313 0.161352359
0.029956626 0.375234512 0.068968124 0.018103424 0.029086285 0 0
0.02531118 0 0.353974334 0.943831402

TCGA-05-4389-01A 0.03429236 0 0.005409859 0.225946369 0 0.023338972
0.079331043 0.0117583660.058105451 0 0.010665731 0 0 0.098155184
0.1135329680.1317211780.131078092 0.003222859 0.068806891 0 0
0.004634678 0 0.421413895 0.905971332

TCGA-05-4390-01A 0.002592391 0 0.179164136 0.005793595 0 0.095898085
0.0235171140 0.016663078 0 0.065327258 0 0 0.403093995
0.030344148 0.155870719 0.002992017 0 0 0.01164378 0 0.007099686
0 0.337386142 0.959388823

TCGA-05-4396-01A 0.033120627 0 0.097076224 0.047402552 0 0.214233689
0.026995619 0.028469768 0.032595541 0 0.009002352 0.010277708 0
0.136213703 0.082919558 0.2011566960.014584797 0.0102711070.044357009
0 0 0.01132305 0.110.121241921 1.032392946

TCGA-05-4397-01A 0 0 0 0.002774093 0 0.123810688 0 0 0.028732278
0 0.00584881 0.002869443 0.009436415 0.160730524 0.170162329
0.370273577 0.030654273 0 0.094707572 0 0 0 0.005 0.294744997
0.986435792

TCGA-05-4398-01A 0 0 0.144298183 0.030006587 0 0.094776328
0.005056516 0.028404804 0.017220176 0 0.021860477 0 0.048635447
0.1173487820.1118847580.249015406 0.095955647 0 0 0.02198187
0.008708505 0.004846515 0 0.439776301 0.89741441
TCGA-05-4402-01A 0 0.0109681140.131954876 0.04969179 0 0.077238972 0
0.030390142 0.038248293 0 0 0.023501266 0 0.104351675
0.059901429 0.288082396 0.027667174 0.046315971 0.081227377 0 0
0.030460526 0 0.394100984 0.918440847
TCGA-05-4403-01A 0.01657071 0 0.052968913 0.034712856 0 0.135198517
0.004084983 0.012389028 0.017497164 0 0.010409356 0.00175744
0.020702051 0.285072536 0.096839834 0.179402706 0.059176774 0
0.066902217 0 0 0.006314915 0 0.409723315 0.914437637
TCGA-05-4405-01A 0 0.030413242 0.150417636 0.060128394 0 0.062707139
0.00311772 0.016216751 0.023200491 0 3.7484E-05 0.002565617 0
0.375673197 0.023196283 0.165144425 0.0228067 0 0.062307976 0 0
0.002066945 0 0.46797336 0.884671923
TCGA-05-4415-01A 0.032798275 0 0.1301134720.200559602 0 0.072135733
0.092277027 0.049330025 0.000522109 0 0.086222649 0.018546296
0.002491978 0.07473524 0.08447066 0.080425621 0 0.026682673
0.030390373 0 0 0.018298266 0.025 0.226049539 0.994173615
TCGA-05-4417-01A 0.035822383 0 0.080814826 0.04345825 0 0.212347553
0.078168804 0.010184928 0.033014971 0 0 0.034483762 0
0.141225564 0.064824277 0.140317934 0.044142941 0.017295099
0.057376634 0 0 0.006522074 0 0.43644109 0.900176976
TCGA-05-4418-01A 0.033900882 0 0.171926508 0.008147324 0 0.127668169
0.080687618 0.007797971 0.017248906 0.040663943 0 0.034974263 0
0.1178092690.1001149480.170431454 0.003228455 0.00779147 0.067427987 0
0 0.010180833 0 0.384572356 0.922485646
TCGA-05-4420-01A 0.064583574 0 0.200772819 0.0390811270 0.202407754 0
0 0.027078833 0 0 0.01187354 0 0.048143161 0.014383489 0.18590839
0 4.92088E-050 0.024882569 0 0.180835536 0.055 0.163900967
1.016881752
TCGA-05-4422-01A 0.016568682 0.001079235 0.235797402 0.260864338 0

0.0693829110.014455825 0.05100053 0.050014571 0 0 0.017314066
0.03326839 0.045510234 0.055287196 0.127752635 0.017935959 0
0.003768024 0 0 0 0 0.327443834 0.947337047
TCGA-05-4424-01A 0.026056913 0 0.0378952 0.08764358 0 0.193139872
0.025546955 0.01076522 0.006200366 0 0.007685788 0 0.043722711
0.146840224 0.106400657 0.218341988 0.0268436 0.008184363
0.047726704 0 0 0.007005858 0 0.4011467990.915906014
TCGA-05-4425-01A 0.010637001 0 0 0.005591485 0 0.170685172
0.008412993 0 0.051361527 0 0.012267514 0 0 0.225399541
0.072388489 0.227586307 0.158282542 0 0.054949247 0 0
0.002438182 0 0.657449614 0.772544746
TCGA-05-4426-01A 0.029730895 0 0.002920085 0.044872607 0 0.225013541
0 0 0.055038021 0 0 0.017622422 0.032999538 0.318115094
0.037400627 0.171347906 0.002363215 0 0.026672859 0 0 0.03590319
0.005 0.291924621 0.978784795
TCGA-05-4427-01A 0.015386285 0 0.067934487 0.0996113560 0.103894973
0.070817427 0.024891494 0.008232136 0 0 0.008870533 0.049853857
0.067906063 0.0866751140.172996021 0.143251357 0.005291427
0.063230398 0 0.010035735 0.0011213360 0.319246934 0.949282644
TCGA-05-4430-01A 0.014128046 0 0.030982566 0.068858881 0 0.019378221
0.031200891 0.030279363 0.029717225 0.004269017 0 0.005955275 0
0.252631306 0.073586845 0.185208499 0.1150843070.039859199 0.0856442
0 0 0.013216158 0 0.562863073 0.830490837
TCGA-05-4432-01A 0.010178755 0 0.160241686 0.067830581 0 0.154352358
0.006380308 0.0112456310.013966486 0 0 0.037539928 0 0.259812929
0.092089253 0.132771045 0.00755302 0 0.026169821 0 0 0.0198682 0
0.402897715 0.915600012
TCGA-05-4433-01A 0.008312885 0.002100838 0.002696193 0.091320298 0
0.1142722080.016396375 0.019933314 0.013471545 0 0 0.020916521 0
0.328299794 0.1086075110.156002385 0.024901556 0 0.070725821 0 0
0.022042756 0 0.395769398 0.925339619
TCGA-05-4434-01A 0.023547053 0 0.021394831 0.03934776 0 0.201867644
0.071018923 0 0.026298032 0 0.029704138 0 0.006853957 0.16627284

0.15546003 0.180292025 0.036552866 0 0.038309455 0 0 0.003080446 0 0.487348099 0.872586943

TCGA-05-5420-01A 0 0 0.120597398 0.032507893 0 0.126803156 0.03934407 0.012182456 0 0.019755218 0 0.034783955 0 0.161940917 0.136414598 0.191484121 0.0561481120 0.055002466 0 0 0.013035639 0 0.5411271030.846066899

TCGA-05-5423-01A 0.012105295 0.000836813 0.028658949 0.034598666 0 0.101798724 0.02580574 0.019073645 0.034299049 0.023137654 0 0.0135649110.008324019 0.008188865 0.006154525 0.197079073 0.258187706 0.051970496 0.075532737 0 0.0586119980.0420711340 0.484356487 0.877532465

TCGA-05-5425-01A 0.024458668 0 0.06050387 0.203703681 0 0.062927163 0.07499555 0.009471398 0.016241843 0 0 0.002581666 0.003394967 0.078684361 0.149817794 0.184065078 0.013904055 0.022741138 0.051637447 0 0.0110574 0.029813921 0 0.412005827 0.910896891

TCGA-05-5428-01A 0.0115945920 0.033090564 0.0490531120 0.064460185 0 0 0.034192042 0.004497425 0 0.029604968 0 0.124955433 0.021290384 0.409295556 0.067564478 0 0.123967099 0 0.001283548 0.025150614 0 0.324539553 0.957848188

TCGA-05-5429-01A 0.022016045 0 0.145968764 0.02038783 0 0.277558632 0 0.000940627 0.033525974 0.037167684 0 0.01261437 0 0.076870159 0.039996515 0.210736538 0.044296205 0.003049378 0.045857012 0.006923891 0 0.022090375 0.105 0.125248975 1.024946904

TCGA-05-5715-01A 0 0.0163005 0.019182398 0.036265047 0 0.07503285 0.002327822 0.005628749 0.007722801 0 0 0.009361295 0 0.48252378 0.009059134 0.239129468 0 0.026178587 0.067956238 0 0 0.003331331 0 0.532245228 0.851960523

TCGA-35-3615-01A 0.026959587 0 0.1126881660.025574577 0 0.152594463 0.012550757 0.025451516 0.010847423 0 0.005060986 0 0 0.154597926 0.035350142 0.302962063 0.013080936 0.024397598 0.091435961 0 0 0.006447898 0 0.37220166 0.929141198

TCGA-35-4122-01A 0.0114522820 0.124207321 0.1183706110 0.038660166 0.055903927 0.041521571 0 0.039955596 0 0.00646586 0.010471868

0.044693667 0.085927302 0.295407529 0.061879636 0.007765245
0.024095133 0 0.007369455 0.02585283 0 0.464627496 0.886797391
TCGA-35-4123-01A 0 0.0015687 0.006642146 0.351563164 0 0 0.068362844
0.034199747 0.030170533 0.005420302 0 0.031773604 0 0.154154789
0.0421193030.202420796 0.032787786 0 0.022971668 0.008648896 0
0.007195723 0 0.483960859 0.874244958
TCGA-35-5375-01A 0.00580819 0 0.199024266 0.103092039 0 0.002485271
0.0419611170.032055845 0.007901606 0.085373578 0 0 0 0.080636955
0.139990443 0.157469518 0.041313989 0 0.08881037 0 0 0.014076813
0 0.470703604 0.88386785
TCGA-38-4625-01A 0.016664515 0 0.078009996 0.079541587 0 0.099357509
0.042380863 0 0 0.024255102 0 0.012817998 0 0.227949783
0.1145319830.215967967 0 0.023185042 0.053203003 0 0.001782983
0.010351669 0 0.319019487 0.958634365
TCGA-38-4626-01A 0.009217389 0 0.021817278 0.027501013 0 0.183301395
0 0 0 0 0.02341325 0.021035843 0.078745738 0.223151298
0.016795077 0.255813285 0.013840626 0.017522903 0.072479926 0
0.002341395 0.033023583 0.015 0.270933869 0.982474047
TCGA-38-4627-01A 0.014069676 0 0.060026975 0.016216495 0 0.230004484
0 0 0.015608744 0.000123044 0 0.015663399 0.025004127 0.11059142
0.042743309 0.340214678 0.034536824 0 0.069295837 0 0
0.025900987 0 0.534235291 0.85036888
TCGA-38-4628-01A 0 0 0.028924069 0.0131156960 0.041506398 0
0.016873595 0.016303309 0 0 0.013240549 0.08092493 0.164980424
0.006159905 0.410292241 0.098563101 0.00536069 0.094449155 0 0
0.009305939 0 0.362954593 0.94532402
TCGA-38-4629-01A 0.021589821 0 0.099569071 0.0116299730 0.095102247
0.017605228 0.002985306 0.020303582 0.042320764 0 0.066662009 0
0.169099309 0.096158232 0.283278536 0.013752132 0 0.033584426 0
0.005210216 0.0211491490 0.471404792 0.88116192
TCGA-38-4630-01A 0.065386203 0 0.02224706 0.0977333110 0.240824685 0 0
0.0371268110 0.004721056 0.004946374 0 0.26857248 0.087554578
0.1382025110.017905482 0.014779448 0 0 0 0 0.03 0.215161034

1.012266049

TCGA-38-4631-01A 0 0 0.317302826 0.1052115840 0.005039868 0.015978039 0.030792343 0 0 0 0.030381491 0 0.192174024 0.093595035 0.133485298 0 0 0 0 0.00473348 0.071306012 0.04 0.19222034 1.007088282

TCGA-38-4632-01A 0.017359886 0.00934543 0 0.00062142 0 0.125649969 0.06693431 0.014922342 0.028138889 0.0522114540.01585302 0 0.0112337660 0.0768529110.278209065 0.1811133260.044171067 0.064831925 0 0 0.01255122 0 0.336797457 0.944461137

TCGA-38-6178-01A 0.021445473 0 0.090828255 0.027057594 0 0.229025789 0 0.008601854 0.0171153030 0.001849456 0.013726928 0.016244087 0.092168673 0.065131882 0.182246266 0 0.077213326 0.154987204 0 0 0.002357909 0.02 0.262216406 0.973866715

TCGA-38-7271-01A 0 0.1590379 0.063007448 0.129493412 0 0.146023304 0.012733173 0 0.076688256 0.0585735110 0.0117907560 0.122865621 0.023259751 0.11575784 0.025482954 0.01311762 0.039613317 0 0 0.002555138 0 0.325193898 0.948482346

TCGA-38-A44F-01A0.047023381 0.024546136 0.120693703 0.04716116 0 0.131938417 0 0.048652034 0.063006209 0 0 0.028424801 0.010259368 0.08810587 0.128418531 0.087761602 0.097972362 0.0024961140.071316495 0 0 0.002223816 0 0.434943536 0.900612144

TCGA-44-2655-01A 0.042641614 0.021244252 0.10552599 0.018426457 0 0.230148898 0 0.03414572 0.024330345 0 0 0.024097571 0.010181335 0 0.0187247110.193505429 0.173813295 0.027196424 0.057757647 0 0.008497182 0.00976313 0.01 0.28418681 0.962137059

TCGA-44-2656-01A 0.016202459 0 0.134710149 0.2569392110 0.025742644 0.0511656950.019585905 0.004822575 0 0 0 0.023497732 0.036758433 0.123499554 0.166348716 0.067773339 0 0.058240095 0 0.009615781 0.005097712 0 0.535706309 0.85374888

TCGA-44-2657-01A 0.0915947110 0.214398408 0.179867436 0 0.041316123 0.03875908 0.046862582 0.047383793 0.017324102 0 0.013692591 0.0020110410.057352978 0.055646868 0.103271013 0.0286511770.001239633 0.056256323 0 0 0.004372142 0 0.457782833 0.893607201

TCGA-44-2659-01A 0.004418938 0 0.171863984 0.13018277 0 0.21560176 0
0.02346351 0.055567814 0 0 0.038505634 0.005009763 0.015370224
0.092428633 0.1109191510.032386158 0 0.098849878 0 0 0.005431783
0 0.351650683 0.936827566
TCGA-44-2661-01A 0 0.005342103 0.1087008 0.085400075 0 0.121908261
0.012006722 0 0.018703988 0.04350594 0 0.050712418 0.030604614 0
0.07114668 0.177783182 0.1114327640 0.1167462760 0.040229994
0.005776182 0 0.543884223 0.854352124
TCGA-44-2662-01A 0.005718894 0 0.07062901 0 0 0.079313134 0.033126691
0.000686099 0 0.024210202 0 0.015474933 0.108071756 0.266250239
0.012822976 0.1131339240.100697456 0.021235767 0.060775567 0
0.024502246 0.0633511070 0.341941494 0.947848964
TCGA-44-2665-01A 0 0 0.006852044 0.019964884 0 0.206468914
0.012486549 0 0.006863372 0 0.006346956 0 0.009365458
0.241943183 0.009323827 0.22656842 0.13576463 0.055983732 0.060570679
0 0 0.001497352 0.025 0.24144692 1.000751813
TCGA-44-2666-01A 0.005120091 0 0.1341847110.059996221 0 0.166746426 0
0.013676817 0 0 0 0.051835783 0.0042882110.025991967 0
0.071465562 0.050917362 0.081446842 0.246869134 0 0.083217483
0.00424339 0.02 0.261541372 0.973832998
TCGA-44-2668-01A 0.003999868 0 0.016106408 0.074957466 0 0.109968077
0.121864595 0 0 0 0 0.035906509 0.027302856 0.335179483
0.082339251 0.050133993 0 0 0 0.11396348 0.007999103 0.020278912
0 0.462475555 0.887517447
TCGA-44-3396-01A 0.02055868 0 0.154828306 0.070765148 0 0.169574062
0.035697686 0 0 0 0 0.001381555 0.006043374 0.241964603
0.092407271 0.12689577 0.015077389 0 0 0.035600287 0 0.02920587 0
0.436216332 0.899249063
TCGA-44-3398-01A 0.003748654 0 0.068440182 0.035534779 0 0.120323838
0.015533404 0 0.008552325 0 0 0.042798804 0.013569083
0.019716524 0.012654417 0.053634749 0.391746021 0.086514896
0.0821181770 0.034534663 0.010579484 0.04 0.184465613 1.01388032
TCGA-44-3917-01A 0.038448936 0 0.130557791 0.068975016 0 0.011328586

0.189774483 0.0027754 0 0.049942027 0 0.010979446 0.004415846 0
0.07482169 0.215862814 0.1104413570 0.057507383 0 0.012395538
0.021773688 0 0.35339956 0.934963504
TCGA-44-3918-01A 0.0039947110 0.155487821 0.149624508 0 0.074208069
0.098797358 0.020504154 0 0 0.028673177 0 0 0.138999735
0.1001134850.18272906 0.008397184 0 0.027412608 0 0 0.0110581310
0.543425508 0.850114448
TCGA-44-3919-01A 0.025632196 0 0.098183939 0.070678004 0 0.109804994
0.004255678 0.032988921 0.046483688 0.003571025 0 0.034415044 0
0.081331749 0.135209276 0.126682835 0.123623129 0 0.103049825 0
0 0.004089696 0 0.602951835 0.820473343
TCGA-44-4112-01A 0.004789635 0 0.215353403 0 0 0.124559008 0 0
0.016454609 0.076226848 0 0.041261571 0.005964658 0.064321168
0.037169854 0.219456382 0.071454215 0.0261179840.072676279 0 0
0.024194385 0 0.364542071 0.930731222
TCGA-44-5643-01A 0.058459312 0 0.045077928 0.079845441 0 0.123938153
0.106229857 0.024858023 0.00929248 0.022291259 0 0 0.008615504
0.158433545 0.10019751 0.1333119640.050355761 0.0275384110.051554853 0
0 0 0 0.417646795 0.907860886
TCGA-44-5644-01A 0.071995157 0 0.2118522470.082384803 0 0.033886612
0.083601202 0.037347359 0.014579233 0 0 0.025432385 0
0.220876247 0.020977103 0.09554785 0 0.046743864 0.01543384 0 0
0.039342099 0.405 0.053643525 1.051911546
TCGA-44-5645-01A 0.010936126 0.047572854 0.022105821 0.039221209 0
0.186616257 0.01672893 0.02219411 0.070555221 0 0 0.024942064
0.024097807 0.098233365 0.032526382 0.1103390040.150891786
0.057782541 0.08168213 0 0 0.003574394 0 0.409015903 0.91209625
TCGA-44-6145-01A 0.010561977 0.0039112550.124888195 0.251760033 0
0.0419114640.107002301 0 0.00502968 0 0 0.022414179 0.000524701
0.079185904 0.1199625450.1112987570.002269673 0.028845835 0.065377331
0 0 0.02505617 0 0.471465092 0.882300828
TCGA-44-6146-01A 0.040830773 0 0.073459904 0.067069586 0 0.349586493
0.03489591 0 0 0.031834722 0 0.020186885 0.013982269 0.020675691

0.072667321 0.121099745 0.05009526 0 0.070434988 0 0 0.033180453 0.525 0.035700493 1.06709816

TCGA-44-6147-01A 0.106121415 0 0.153185198 0.049517655 0 0.148306719 0.039903043 0 0.035901757 0.01394788 0.012493476 0 0 0.208767852 0.061616918 0.10729198 0 0.007286741 0.055659367 0 0 0 0 0.367258031 0.930056558

TCGA-44-6148-01A 0.031395375 0.010883947 0.191632742 0.049848277 0 0.079328503 0 0.028596964 0.0110954630 0 0.010031328 0.072501053 0.042410109 0.008826622 0.243430627 0.045256879 0.009897902 0.160328821 0 0 0.004535389 0 0.333474671 0.943451612

TCGA-44-6774-01A 0.002738569 0 0.103296488 0.001287442 0 0.140171786 0.008609418 0 0.053870295 0.002080728 0.005512177 0 0.022103236 0.295923225 0.072370468 0.222039198 0.019157139 0 0.029174277 0 0 0.021665554 0 0.510667599 0.859607826

TCGA-44-6775-01A 0.001081285 0 0.05424682 0.024927481 0 0.073102982 0.0116571730 0.059389241 0 0 0.022571476 0.020755808 0.290630571 0.040556449 0.163069564 0.138596129 0.020728463 0.065172908 0 0.003688578 0.009825073 0 0.529962412 0.84886766

TCGA-44-6776-01A 0.049321696 0 0.1461704 0.1191667270 0.309123666 0 0.019203901 0.004429713 0 0.015264213 0 0.057198149 0 0.002552132 0.093786078 0.026322598 0.080014342 0.069382549 0 0 0.008063836 0.095 0.13434568 1.02408478

TCGA-44-6777-01A 0.008152814 0 0.1117677120.019209933 0 0.064373284 0.0011847630 0.036257457 0 0 0.026888052 0.016367266 0.21451803 0.047636308 0.238032814 0.1164690740 0.084996474 0 0 0.014146019 0 0.563456972 0.833180416

TCGA-44-6778-01A 0.013142891 0 0.095865647 0.019184727 0 0.257862777 0.026161733 0.060128878 0 0.023222832 0.004536141 0.002840688 0.003847177 0.1120358390.126867639 0.152582342 0.061412582 0 0.027868454 0 6.49054E-060.012433161 0 0.430128948 0.901983091

TCGA-44-6779-01A 0.003429133 0 0.0113256090.202274618 0 0.09109092 0.056727379 0.015864643 0.0511397620 0.093231516 0 0 0.086428456 0.159199938 0.168838674 0.012480288 0 0.040410265 0 0

0.007558797 0 0.507410344 0.861143009
TCGA-44-7659-01A 0.017798924 0.054037135 0.125080526 0.059913718 0 0.166904921 0 0.033450124 0.047143712 0 0 0.027779361 0.009142367 0.063123627 0.031344415 0.160256017 0.084593331 0.044683007 0.0699144110 0 0.004834405 0.045 0.181058391 0.999213952
TCGA-44-7660-01A 0.005566708 0.007255266 0.084256449 0.069920058 0 0.0911660270.004350807 0.020220026 0.015126312 0 0.025995916 0 0.002762505 0.1191032130.158638735 0.343536049 0.045331214 0 0 0.006770714 0 0 0 0.34399106 0.950518985
TCGA-44-7661-01A 0.034242202 0 0 0.055259121 0 0.133660401 0.101928538 0 0.012037613 0 0.003059109 0.036701875 0.054873984 0.050314742 0.07664889 0.173978321 0.0711078860.100734479 0.086080709 0 0 0.00937213 0 0.376283796 0.92624707
TCGA-44-7662-01A 0.014353366 0 0.120007902 0.048496827 0 0.116809806 0.0313811290.002814432 0.03284946 0.015885828 0.000989769 0.009001625 0 0.254339819 0.068708455 0.244689235 0.004722533 0 0.01503394 0.003732274 0 0.016183601 0 0.522658567 0.854044899
TCGA-44-7667-01A 0.002867348 0 0.1103163670.057101491 0 0.055951456 0.0276115120 0.015329155 0 0 0.055452708 0 0.431422591 0.083643241 0.142806648 0.003823618 0 0 0.006982252 0 0.0066916110 0.453232236 0.897494172
TCGA-44-7669-01A 0.068218204 0 0.057703107 0.094103172 0 0.114184943 0.06911412 0 0.02322484 0 0.027864659 0 0.007145166 0.114588966 0.123317307 0.21649878 0.029479687 0 0.04930798 0 0 0.005249069 0 0.362671536 0.932833681
TCGA-44-7670-01A 0.010800657 0.004278047 0.063050682 0.152367541 0 0.066801408 0.046423568 0.043036278 0.012434399 0 0 0.039786283 0.006120644 0.267373659 0.11117486 0.077344653 0.059019578 0 0.025896719 0 0.0117179440.002373081 0.03 0.212441895 1.008231605
TCGA-44-7671-01A 0.003324599 0 0.145935987 0.051615741 0 0.1131304320 0.066944101 0.01482541 0 0 0.0151105790 0.218430147 0.001161997 0.203090662 0 0.084282756 0.07972436 0 0.000401634 0.002021596

0.055 0.163336507 1.019103935
TCGA-44-7672-01A 0.03031229 0 0.168872947 0.076657079 0 0.116780638 0.053802296 0 0.04751303 0 0 0.06698193 0.016299864 0.119600511 0.062202417 0.122108607 0.032289584 0 0.083557463 0 0 0.003021342 0 0.417066201 0.909492209
TCGA-44-8117-01A 0.041531803 0 0.049068327 0.019879734 0 0.15967064 0.013649194 0.041346615 0.012068436 0.014325588 0 0.020737186 0 0.308177601 0.075338737 0.17856952 0 0 0.061906233 0 0 0.003730385 0 0.510871824 0.859620794
TCGA-44-8119-01A 0.039574952 0 0.170966764 0 0 0.055181871 0.04078037 0.026891537 0.001290672 0.039939704 0 0.028788488 0 0.35830395 0.052477883 0.149359005 0 0 0.028754027 0 0 0.007690777 0 0.320514313 0.961149564
TCGA-44-8120-01A 0.035909815 0 0.107615228 0.08284779 0 0.143843867 0 0.014365343 0.024660273 0 0 0.009353572 0.021773434 0.058756215 0.01124737 0.166516342 0.108925251 0.037188828 0.158289594 0 0 0.018707078 0.02 0.243583905 0.977179989
TCGA-44-A479-01A 0.035905966 0 0.174594767 0.060707012 0 0.206978293 0.017695929 0.003552787 0.020312389 0.051600224 0 0.042493725 0 0.067394594 0.1131560730.108407355 0.03951205 0 0.057688836 0 0 0 0 0.517504173 0.862753805
TCGA-44-A47A-01A 0 0.010612848 0 0.028000264 0 0.13140999 0.0011099220.032144401 0.077527037 0 0 0.014720405 0.002002947 0.130076099 0.015791673 0.077618574 0.315498625 0.086337256 0.07714996 0 0 0 0 0.520266629 0.856433525
TCGA-44-A47B-01A 0.008985432 0 0.021952051 0.158993828 0 0.036521309 0.0311273260.042755968 0.092561558 0 0 0.044769685 0 0.184316158 0.050070652 0.151041747 0.085003994 0.004367503 0.08753279 0 0 0 0 0.36814334 0.930931495
TCGA-44-A47G-01A 0.01770085 0.002556147 0.133833544 0.121543324 0 0.165608154 0.058910184 0 0.0354941160.013843032 0 0.00443725 0.007136304 0.124306389 0.059194092 0.127536658 0.059356359 0.013386372 0.053945098 0 0 0.001212126 0 0.448459001

0.895983229
TCGA-44-A4SS-01A 0.032037367 0 0.001841759 0.084474442 0
0.236350031 0.067147494 0 0 0.029269859 0 0.041828802 5.95609E-05 0.10908497 0.094496146 0.21669397 0.008487393 0.009369996 0.06413731 0
0.001067297 0.003653602 0 0.391251998 0.920953862
TCGA-44-A4SU-01A 0.008726764 0.015374839 0.271465079 0 0
0.188994032 0 0.060076732 0.056365571 0.020228601 0 0 0
0.151520475 0.1133948620.056806694 0.025175588 0 0.027504158
0.002816037 0 0.00155057 0 0.385613589 0.922466215
TCGA-49-4486-01A 0.004798802 0 0.303899645 0.014066366 0 0.224727178
0 0.032894467 0 0 0 0.037180528 0.010725457 0.004818325
0.067303472 0.141550081 0.042009423 0.022301754 0.0911925360
0.002160305 0.00037166 0.245 0.078137268 1.034332008
TCGA-49-4487-01A 0.041091896 0.084123853 0.067216217 0.206555051 0 0
0.053753395 0.013648536 0.090699871 0.027065348 0 0 0
0.220558358 0.063332383 0.105127662 0 0.010917833 0.015909597 0
0 0 0 0.526090295 0.855376251
TCGA-49-4488-01A 0.003365232 0.007867908 0.06588007 0.167958212 0
0.057057878 0.054829991 0.022998366 0.044278772 0.027122379 0 0
0.000897595 0.156169121 0.094691336 0.1145902660.157777824 0
0.02451505 0 0 0 0.03 0.216888423 0.997060827
TCGA-49-4490-01A 0.050302415 0 0.016857158 0.076580286 0 0.141229231
0 0.012784567 0.156816784 0 0 0.028633277 0.017537526
0.277244019 0.079337292 0.06494481 0 0.0113589940.066373641 0 0 0
0.025 0.24029575 0.994389196
TCGA-49-4494-01A 0.026026066 0 0.034657053 0.037952822 0 0.012494047
0.0272502110.067739287 0.004368647 0.059059372 0 0.038861805 0
0.189124322 0.095339217 0.191919593 0.1206276110.0116811160.080816047
0 0.00019998 0.001882804 0 0.429164359 0.902586209
TCGA-49-4501-01A 0.033109815 0.021042001 0.059503624 0.062509272 0
0.20780159 0 0.027071253 0.067055767 0.040874609 0 0.04067785
0.0101981160.070991704 0.035082043 0.123952447 0.042040185
0.067506297 0.082120056 0 0 0.008463373 0.005 0.305159571

0.955915428
TCGA-49-4505-01A 0.014332487 0 0.068069456 0.030923816 0 0.127918968 0.01148874 0.028255806 0.0441800110.035227244 0 0.043734897 0.019391657 0.033939349 0.091730344 0.181614965 0.095399483 0.036048191 0.129333441 0 0 0.0084111450 0.471975372 0.884106798
TCGA-49-4506-01A 0.040924969 0 0.072894472 0.353242391 0 0 0.06690577 0.041642497 0.010717842 0.030810446 0 0.029797787 0 0.087394129 0.081650108 0.12261594 0.057897799 0 0 0 0 0.003505849 0.025 0.231600197 0.998848106
TCGA-49-4507-01A 0 0 0.079924591 0.342243776 0 0 0.122968177 0.01318846 0.036922565 0.018984988 0 0.0851138990 0.071336877 0.054027313 0.104880814 0.003768309 0 0.066640232 0 0 0 0 0.431996128 0.902387617
TCGA-49-4510-01A 0.023690419 0 0.370891359 0.069206262 0 0.101488816 0 0.050101454 0 0 0 0.043315573 0.0052774 0.0110122710.027499741 0.195493553 0.024124893 0.025636852 0.04593169 0 0 0.006329716 0.04 0.188097176 0.995972239
TCGA-49-4512-01A 0 0.037624843 0.02406183 0.006391362 0 0.1179404030 0.002599014 0.037366751 0 0 0.0115614970.035064391 0.172116621 0.01394836 0.3171711840.089469498 0.049730609 0.084953637 0 0 0 0 0.466567815 0.884096111
TCGA-49-4514-01A 0.051785188 0 0.108214397 0.189777463 0 0.041443806 0.072886086 0.050883077 0.075680573 0 0.006004645 0.017234079 0 0.094028816 0.088930656 0.095267896 0 0.063480833 0.044382486 0 0 0 0.005 0.289880435 0.963790205
TCGA-49-6742-01A 0.008590994 0 0.076043858 0.060084654 0 0.140655657 0.008717292 0.023821682 0 0 0 0.085504665 0.015404845 0.038748015 0.030242715 0.301041719 0 0.099588782 0.103582515 0 0 0.007972606 0.045 0.179330007 1.010866887
TCGA-49-6743-01A 0.054051788 0 0.133038563 0.068535669 0 0.159370158 0.044666949 0.016640215 0.026088103 0 0.016267795 0 0.000460495 0.22043049 0.083024284 0.142997305 0.00258809 0 0.028292639 0 0 0.003547454 0.005 0.301007175 0.960035931

TCGA-49-6744-01A 0.021216141 0 0.099071365 0.045452381 0 0.222193231 0.020885502 0 0.033106226 0.031231324 0 0 0.00880018 0.103283234 0.04803058 0.121482821 0.120518903 0.054284591 0.064132755 0 0 0.006310764 0 0.483169824 0.880765363

TCGA-49-6745-01A 0 0 0.0171167610.15540285 0 0.09389761 0.063647541 0.027205678 0.017012652 0 0.005033602 0 0.09610275 0.089810052 0.122328689 0.107604268 0.073141093 0.057183108 0.074513346 0 0 0 0 0.402107494 0.915091157

TCGA-49-6761-01A 0 0.015894081 0.01219463 0 0 0.123366284 0.200065674 0.017567131 0 0.026829016 0 0.080366641 0 0.157513131 0.120980935 0.14327237 0.026643897 0 0.071012883 0 0 0.004293328 0 0.439949155 0.897306287

TCGA-49-6767-01A 0.009893351 0 0.004076042 0.161024609 0 0.058127604 0.055127737 0.01850634 0.042853125 0 0.002577592 0.033459143 0 0.383986681 0.084788721 0.108134075 0.007219109 0 0.030225872 0 0 0 0 0.413683468 0.917230287

TCGA-49-AAQV-01A 0.069944575 0.004473585 0.063698753 0.085188894 0 0.180025363 0 0.020597159 0.139052974 0 0 0.002081356 0.006838822 0.242546988 0.068205093 0.080489524 0.014020863 0.001015052 0.021821 0 0 0 0 0.388716471 0.922265154

TCGA-49-AAR0-01A 0.062063985 0 0.04385851 0.06348749 0 0.120375272 0 0.087852058 0.090156235 0 0 0.027307394 0.015153806 0.115217083 0.086645175 0.1901164240.038038131 0 0.059728437 0 0 0 0 0.418793575 0.90727568

TCGA-49-AAR2-01A 0 0.038648078 0.35902493 0.099100927 0 0.086227591 0 0.016776355 0.102934605 0 0 0.016276668 0.0027113330.098410457 0.05944882 0.092689123 0 0 0.018894051 0 0 0.00885706 0.01 0.286103279 0.962059891

TCGA-49-AAR3-01A 0.0238461160 0.0700521110.166176248 0 0.020604532 0.090407235 0.024350131 0.027859132 0.006916329 0 0.0407117 0 0.206193707 0.090522056 0.16879389 0.015300907 0 0.040706776 0 0.007559129 0 0 0.444686909 0.89490097

TCGA-49-AAR4-01A 0.009962813 0 0.1114905860.388531033 0 0

0.041365415 0.052774547 0.031628037 0.095106653 0 0 0
0.104330247 0.049456393 0.093018073 0.006097868 0 0.007692866
0.00738243 0 0.0011630390 0.55067905 0.83593192
TCGA-49-AAR9-01A 0 0 0.034800179 0.00310523 0 0 0 0.124008722
0.018770587 0.127527258 0 0.027234437 0 0.483382039 0.078612228
0.046955967 0 0.055603354 0 0 0 0 0 0.387098596 0.943194135
TCGA-49-AARE-01A 0.019062009 0 0.081782234 0.10775406 0 0.004835846
0.016035557 0.034734943 0.055336256 0 0.001730015 0.017672844 0
0.323435006 0.161805551 0.125508594 0 0 0.050307085 0 0 0 0
0.507014933 0.861168833
TCGA-49-AARN-01A 0 0.104812645 0.196466315 0.03531554 0 0.158009922
0 0.002827748 0.1168497470 0 0.037831084 0 0.090964861
0.048657835 0.071036327 0.0568110930.016107879 0.062672417 0 0
0.001636587 0.01 0.280600471 0.962624565
TCGA-49-AARO-01A 0.052934185 0 0.055233186 0.091277049 0
0.067370036 1.30718E-050.0211903930.061993354 0 0.009934163 0
0.015262873 0.2951147180.0489626110.154723049 0.072869823 0.043575878
0.006850676 0 0 0.002694933 0 0.501626144 0.86483581
TCGA-49-AARQ-01A 0.099222626 0 0.042382072 0.090155564 0
0.036980358 0.0211008410.07465485 0.088446388 0 0.02410343 0.016225794
0.0037111880.155876709 0.239143266 0.076555601 0.007653086 0
0.023788228 0 0 0 0 0.359385255 0.948024369
TCGA-49-AARR-01A 0.087866989 0 0.056276343 0.11731773 0 0.149390742
0 0.017834567 0.048024856 0 0.016045768 0.018304682 0.003643616
0.045712707 0.017282216 0.273851606 0.001901463 0.014316395
0.102404075 0 0 0.029826247 0.005 0.299352485 0.958270499
TCGA-4B-A93V-01A 0.015343621 0 0.219001708 0.074653057 0
0.058692168 0.031565093 0.033541404 0.073148571 0 0.000107983
0.038625398 0 0.350302147 0.038166602 0.054678292 0 0
0.012173955 0 0 0 0.03 0.217828667 1.004119957
TCGA-50-5044-01A 0 0 0.085956905 0.134239731 0 0.064296162
0.047684464 0.010899326 0.068845475 0 0 0.05847942 0.002510832
0.108263039 0.059959571 0.179048369 0.063501889 0.031072089

0.081526977 0 0 0.00371575 0.025 0.239317932 0.983387333
TCGA-50-5045-01A 0.046617672 0 0.13539511 0.08446451 0 0.116957811
0.032032093 0.05447148 0.0421142480.027531464 0 0 0.005734717
0.026228209 0.07796753 0.135675649 0.127272047 0.019782344
0.060013842 0 0 0.007741276 0 0.480193758 0.884243771
TCGA-50-5049-01A 0.018455529 0 0.128309227 0.148297205 0 0.070930209
0.032321816 0.038395951 0.027093144 0.029704389 0 0.002909892 0
0.216197634 0.079141394 0.168024721 0.018420989 0 0.013325215 0
0.005950693 0.002521989 0 0.568573992 0.833351014
TCGA-50-5051-01A 0.050259583 0 0.191567606 0.07635275 0 0.10443296 0
0.037524949 0.063054569 0 0.016056814 0 0.012333221 0.207448094
0 0.094391672 0.00659826 0.096175367 0.025073833 0.01392901 0
0.004801313 0.03 0.22293819 0.988819049
TCGA-50-5055-01A 0.11745151 0.02735416 0.1561185830.1114180040 0.107616062
4.6362E-05 0.026915661 0.056078649 0.03317429 0 0.003973353 0
0.127342024 0.047590334 0.065420768 0.06615455 0.015389584
0.037083198 0 0 0.000872907 0 0.315807397 0.950061748
TCGA-50-5066-01A 0 0 0.2020632110.171082857 0 0 0.127139057 0 0
0.039983897 0 0.000244448 0 0.040275142 0.08877209 0.217032718
0.0619675 0 0.047689039 0 0 0.003750041 0 0.539173738
0.857619146
TCGA-50-5068-01A 0.000822968 0.04393164 0.161996507 0.1146954390
0.049993887 0 0.046156214 0.003869569 0.060455328 0 0.043372597
0.002531403 0.005417894 0.134624493 0.1495011310.159494804 0
0.014376491 0.008759635 0 0 0 0.477792126 0.879574759
TCGA-50-5072-01A 0.064710619 0 0.158952731 0.010220673 0 0.140934361
0 0.018979429 0.0463891110 0 0.013566615 0.008301328 0.200813224
0.064444568 0.144975947 0.006049635 0.044881993 0.050800491 0 0
0.025979275 0.005 0.298429235 0.959415435
TCGA-50-5930-01A 0.013920829 0 0.245271066 0.096156066 0 0.085305686
0.074703033 0 0.008178822 0.0011065830.012605843 0 0 0.242780868
0.076584256 0.122161744 0.009158129 0 0.008244769 0 0
0.003822305 0 0.429400991 0.903304128

TCGA-50-5931-01A 0.067724622 0 0.060838636 0.074599676 0 0.059065333 0 0.092974959 0 0 0 0 0 0.33733138 0.069315461 0.166132041 0 0.056205517 0.015812375 0 0 0 0.08 0.145424235 1.050925202
TCGA-50-5932-01A 0 0.003145955 0.1162468840.042165932 0 0.18045167 0 0.040409347 7.7239E-05 0 0 0.030261679 0.037174136 0.145011842 0.0378119720.26534577 0.000679206 0.018642971 0.067559581 0 0 0.015015817 0.075 0.150409945 1.022093788
TCGA-50-5933-01A 0 0 0.021258516 0.029593834 0 0.140678371 0.001568097 0 0.002042718 0.004884648 0 0.002039323 0.006679181 0.339085358 0.062318617 0.351774712 0.004674015 0 0.024990947 0 0 0.0084116620 0.487084191 0.877918185
TCGA-50-5935-01A 0.007375152 1.92188E-050.061731805 0 0 0.270600398 0.020550639 0 0.079574079 0.02696195 0.003762382 0 0 0.193641965 0.057331866 0.129505046 0.055698427 0.062904572 0.029356041 0 0 0.000986459 0 0.388439045 0.921880125
TCGA-50-5936-01A 0.022935198 0 0.042819652 0.0113153160 0.096004322 0.0111072410.034924301 0.040202302 0 0.006442564 0 0 0.521264344 0.006675364 0.127331059 0 0.052584381 0.022329818 0 0 0.004064139 0 0.612123703 0.790822001
TCGA-50-5939-01A 0 0.016537412 0.199492086 0.007225385 0 0.193638664 0.02771129 0 0.008480903 0 0.001832284 0.005496623 0.006127216 0.272889131 0.062570795 0.170527534 0 0.002372327 0.00022159 0.009237363 0 0.015639396 0 0.409236721 0.913200383
TCGA-50-5941-01A 0.029166197 0 0.045542651 0.154835751 0 0.068162136 0.001418771 0.061677095 0.062158702 0 0 0.052598873 0.007481811 0.133142657 0.1481106860.133363032 0.032467291 0 0.063268464 0 0 0.00660588 0 0.553228324 0.837876018
TCGA-50-5942-01A 0.050718252 0 0.175686944 0.042863414 0 0.229587223 0.00534554 0 0.042437037 0 0 0.052298247 0.022925195 0 0.026488466 0.090292367 0.054170562 0.013823451 0.193206073 0 0 0.000157228 0.015 0.276469918 0.965557783
TCGA-50-5944-01A 0.002494901 0 0.016825419 0.036878905 0 0.144022003 0 0 0.0116690980 0 0.044415941 0.085897885 0.09011354 0.025389916

0.289090744 0.021016252 0.014226647 0.217958748 0 0 0 0.005
0.3076115820.96020401
TCGA-50-5946-01A 0.032601467 0 0.104591925 0.13138532 0 0.105069353
0.016054965 0.074286151 0.030133634 0 0 0.037973295 0.004308529
0.136480428 0.071762875 0.139239823 0.027327893 0.026578655
0.056833967 0 0 0.005371721 0.25 0.076049875 1.047701524
TCGA-50-6590-01A 0 0.005685539 0.1195008260.103417309 0 0.093148537
0.069285612 0 0.023466185 0 0.000510696 0.027573828 0
0.255804342 0.1164355410.150538176 0 0 0.034633408 0 0 0 0
0.464326872 0.885000774
TCGA-50-6591-01A 0 0 0.0112492210.039199337 0 0.3488021150 0.017700159
0.012157099 0 0 0 0 0.50217723 0 0 0.004629268 0 0
0.063667298 0 0.000418274 0.08 0.146222138 1.08916176
TCGA-50-6592-01A 0.012576625 0 0.0705942110.089609918 0 0.136958043
0.096982051 9.44616E-050 0 0.033040619 0.047580021 0 0.223056338
0.096938774 0.139515578 0.005413613 0 0.0353105110 0 0.012329237
0 0.446496082 0.893989933
TCGA-50-6593-01A 0 0.022709199 0.031457393 0.014993851 0 0.053412932
0.009439926 0.017292675 0.016287605 0 0 0.002152059 0.055692196
0.330604029 0.022005057 0.12196629 0.169979435 0.016012523 0.09392393
0 0 0.022070898 0 0.497290819 0.867544214
TCGA-50-6594-01A 0 0.002196809 0.060633769 0 0 0.1135655810 0
0.048850318 0.001433364 0.001377638 0.019812166 0.029744485
0.23137958 0 0.290197854 0.088166157 0.089896014 0.0172379 0 0
0.005508365 0.025 0.239208088 1.004107033
TCGA-50-6595-01A 0.012652972 0 0.024546971 0.056766001 0 0.111704906
0.033166106 0 0.009081581 0 0.024995264 0.02900216 0 0.222845072
0.196128936 0.222820315 0 0.026572035 0.022074561 0 0 0.00764312
0 0.587177959 0.809630626
TCGA-50-6597-01A 0 0.019845818 0.1582911260.070267867 0 0.128154507 0
0 0.189904699 0.046791042 0 0.012992235 0 0.043594719
0.127173851 0.059454972 0.0902319 0.021638277 0.031658987 0 0 0
0 0.4015981180.915140065

TCGA-50-6673-01A 0.053876145 0 0.165999944 0.021864769 0 0.161904295
0.006487019 0.035873728 0.03243128 0 0 0.017163417 0.01348783
0.146165922 0.040337065 0.16842765 0.063515913 0.039700319
0.017639206 0 0 0.015125497 0.06 0.159772919 1.011516858
TCGA-50-7109-01A 0.027912865 0 0.220287096 0.121416199 0 0.099048978
0.058478722 0.03558616 0.020216858 0 0 0.060718553 0 0.055279072
0.106461291 0.1110634660.023447359 0 0.056829049 0 0 0.003254333
0 0.313259059 0.952023754
TCGA-50-8457-01A 0.002864932 0.051048497 0.22165044 0.1106805990
0.166310017 0 0.012895608 0.039168629 0.017013305 0 0.020350165
0.048356893 0.025401273 0.069281669 0.08889905 0.05532027 0
0.070758655 0 0 0 0 0.400094889 0.917050697
TCGA-50-8459-01A 0.003831338 0.020413187 0.078685247 0.071096359 0
0.088736459 0.018758357 0.005810923 0.0332117390 0 0.00486761
0.055033328 0.129188443 0.026808269 0.208836194 0.154465711
0.002195395 0.079213363 0 0 0.018848078 0 0.512462477
0.865039853
TCGA-50-8460-01A 0 0.028641669 0.170450583 0.080546441 0 0.108210214
0.007236303 0.033236823 0.065727562 0 0 0.029354451 0.082453027
0.020977097 0 0.074229481 0.1128533690.067944048 0.11813893 0 0 0
0.525 0.035712804 1.04438711
TCGA-53-7624-01A 0.088359625 0 0.037021646 0.103881637 0 0.039161501
0 0.053201055 0.075355016 0 0 0.02883744 0 0.230871521 0.18442801
0.1102904250 0.039024693 0 0 0.009567432 0 0 0.369156626
0.940485123
TCGA-53-7626-01A 0.028015724 0 0.0982116650.065591761 0 0.276474128 0
0.0487411880.0181651130.006348712 0 0 0.023824098 0.086971438
0.082290341 0.156601378 0.03138114 0.008280067 0.027662995 0.02323912
0 0.0182011320 0.352804989 0.936459601
TCGA-53-7813-01A 0.04875998 0 0.392156527 0.205720799 0 0.012250113
0.1142010880.018531395 0.014635412 0 0 0.022866328 0.006327507
0.047230687 0.046130616 0.020858879 0 0.025550346 0.024780322 0
0 0 0 0.364475727 0.931872432

TCGA-53-A4EZ-01A 0.089143757 0 0.039016526 0.130489747 0 0.199272865 0 0.054864668 0.028081514 0 0 0.033543705 0.017057519 0.070248642 0.062796165 0.100990949 0.121536832 0 0.048064874 0 0 0.004892237 0.17 0.100457878 1.040130687

TCGA-55-1592-01A 0.04301393 0 0.079640044 0.037978934 0 0.1198323380 0.028691485 0.010630143 0 0.001667421 0.001969682 0.068589021 0.223707988 0.073737519 0.180780101 0.071670847 0 0.045623873 0 0 0.012466676 0 0.364481857 0.933682423

TCGA-55-1594-01A 0.068739496 0 0.157836593 0.095047254 0 0.128320992 0.043503801 0.012321208 0.02781526 0 0.006925022 0.0100515110 0.1115240420.098656993 0.128928761 0.05182241 0 0.029849388 0 0 0.028657269 0.02 0.252995888 0.975191772

TCGA-55-1596-01A 0.075617936 0 0.0931162940.009399023 0 0.107566081 0.001016909 0.029541257 0.042676619 0.020395767 0.018067745 0 0 0.266235484 0.133842243 0.146625002 0 0.0411831430.009937699 0.004778797 0 0 0 0.353337139 0.943636114

TCGA-55-5899-01A 0.021092569 0 0.0564113490.1056711240 0.083484042 0.063024723 0.0111576470.017384081 0.027198862 0 0.00288718 0 0.289963724 0.062805989 0.217363746 0.000948031 0 0.040606932 0 0 0 0 0.336683875 0.95147485

TCGA-55-6543-01A 0.014578526 0 0.066858213 0.033861093 0 0.103245954 0.017909327 0.000384721 0.006007361 0 0 0.005380325 0.05366109 0.201362249 0.059688657 0.296983639 0.006587336 0.043260535 0.056405229 0 0 0.033825746 0 0.321306533 0.958312049

TCGA-55-6642-01A 0 0 0.358214441 0.11649773 0 0.086277028 0.088577329 0 0.005473575 0 0.028861617 0 0 0.144278131 0.028891334 0.092289789 0 0 0.044948549 0 0 0.005690479 0 0.450600853 0.898451436

TCGA-55-6712-01A 0 0.0208270110.05431206 0.230571055 0 0.124423397 0.084691536 0 0.012237892 0.001887105 0.002756349 0 0.003671963 0.1580441190.139148989 0.077984388 0.01977432 0.009569264 0.060100551 0 0 0 0 0.539331767 0.845248293

TCGA-55-6968-01A 0.004612977 0 0.160219097 0.008912885 0 0.121383205

0.028589592 0.024746862 0.014395333 0.0605514 0 0.032259607 0
0.31363047 0.1122934330.092366815 0.014186198 0 0.0118521260 0 0 0
0.554925682 0.835188547
TCGA-55-6970-01A 0.03102635 0 0.153737613 0.01120555 0 0.114783447
0.0138211750.034330575 0.01220962 0 0 0.015098307 0.02689541
0.140078951 0.074340477 0.179944234 0 0.082393261 0.1101350290 0
0 0 0.442397616 0.897489419
TCGA-55-6971-01A 0 0.037006605 0.139586922 0.162720486 0 0.08976035
0.012900679 0.019875873 0.057827656 0 0 0.03898164 0.012732646
0.1711520610.104625483 0.091757591 0 0 0.053004958 0 0 0.00806705
0 0.409946899 0.911320963
TCGA-55-6972-01A 0.042076993 0 0.279253325 0.22213475 0 0.025205702 0
0.050770986 0 0 0.030037371 0.070542667 0.038673893 0.023812323
0.06314487 0.076103099 0 0.022222702 0.053958485 0 0.002062835 0
0.59 0.027910421 1.067147205
TCGA-55-6975-01A 0.024641605 0 0.288498537 0 0 0.140409317 0
0.029891945 0.022032587 0.017844563 0 0.040689393 0.003398793
0.099350679 0 0.131723712 0 0.157529133 0.035797322 0 0
0.008192414 0 0.398236619 0.917641728
TCGA-55-6978-01A 0.0087011630 0.024626495 0.2444434110 0.086645542
0.102739395 0.01541963 0.026925376 0 0.027697532 0 0.01306518
0.095572643 0.1156978590.12250566 0.085982776 0 0.029977338 0 0 0
0 0.4911825090.871434858
TCGA-55-6979-01A 0.004583739 0 0.105777645 0.092159672 0 0.100639861
0.040397028 0.029990256 0.049952841 0 0 0.0161126380.004900271
0.183654071 0.157721956 0.157350089 0.014875188 0 0.034195019 0
0 0.007689727 0 0.632045315 0.793284649
TCGA-55-6980-01A 0.025635155 0 0.026004383 0.0401138180 0.226427003
0.004205397 0.034174175 0.028439834 0 0 0.024533564 0.10918368
0.031591412 0.053034427 0.038487179 0.22192368 0.066660361
0.068733028 0 0.000852902 0 0.005 0.29460152 0.960416682
TCGA-55-6981-01A 0.04807799 0.0144697 0.066007721 0.006346069 0
0.163922551 0.009650792 0.014975545 0.028249991 0 0 0.009766726

0.015654459 0.1783357110 0.096376629 0.153805666 0.090168766
0.091847787 0 0 0.012343897 0.005 0.292551023 0.962185071
TCGA-55-6982-01A 0.017335031 0 0.125485295 0 0 0.065726548
0.012274728 0 0.03193035 0.02186681 2.65385E-050 0 0.289881128
0.177512283 0.180636626 0.061999643 0.006128324 0.006050473 0 0
0.003146223 0 0.492880997 0.870673185
TCGA-55-6983-01A 0.044368693 0.1187560930.047868661 0.1178274490
0.178609919 1.1033E-05 0.021617614 0.036910087 0 0 0.043375372 0
0.198517715 0.028058088 0.086587656 0.019782338 0 0.057709283 0
0 0 0.005 0.294158652 0.963541185
TCGA-55-6984-01A 0.028462982 0.080156031 0.11858308 0.075618688 0
0.27085025 0 0 0.0686501130 0 0.018408192 0.00247351 0.066891276
0.00244549 0.075471649 0.044671953 0.045497042 0 0.064643347 0
0.037176397 0.04 0.18827994 1.002679339
TCGA-55-6985-01A 0.016853356 0 0.11846953 0.006182452 0 0.233155363
0.060452324 0 0.020557852 0 0.0073115190.017579956 0 0.168260661
0.105977205 0.133988186 0.022179461 0 0.080589543 0 0
0.008442591 0 0.404363974 0.91403306
TCGA-55-6986-01A 0.01518341 0 0.00944537 0.1107427020 0.236875849 0
0.004880291 0.000769913 0 0 0.000595739 0.029640831 0.019601469
0.016420684 0.355155261 0.1168350190.018905561 0.064947901 0 0 0
0 0.347754756 0.941510344
TCGA-55-6987-01A 0.001788517 0.049283639 0.1124395920.233271589 0 0
0.09547476 0.033002794 0.021655312 0 0.043855074 0 0.00942892
0.153649928 0.131524025 0.066920125 0.003449057 0 0.036519859 0
0 0.0077368110 0.507065464 0.864228924
TCGA-55-7227-01A 0.006161398 0 0.0114304440.054635775 0 0.184209856
0.001698218 0 0.040835967 0 0 0.026681778 0.029182535
0.128798836 0.091048572 0.082214257 0.213625135 0.029194517
0.095935751 0 0 0.004346961 0 0.4861186690.874984606
TCGA-55-7281-01A 0 0.009424372 0.022883628 0.075699171 0 0.123072828
0.005315425 0.022814627 0.0247211720 0 0.023278431 0.056358944
0.126618923 0.07334082 0.08689426 0.242618652 0.03880274 0.052715037 0

0.01520808 0.000232889 0 0.395055968 0.918387548
TCGA-55-7283-01A 0.007923921 0 0 0.098973968 0 0.237662373
0.001655233 0.016702354 0.009375571 0 0 0.048079214 0.01780405
0.03185157 0.09566956 0.189990333 0.08047948 0.015258922 0.144859192 0
0 0.003714261 0 0.3110693120.956936643
TCGA-55-7570-01A 0 0 0.46570447 0.101962236 0 0.03044541 0.016479074
0.046815407 0.028371281 0 0 0.036698305 0.006304374 0.082103376
0.042894256 0.0712511360.060797657 0 0.006910845 0.003225414
3.67585E-050 0.01 0.280183181 0.96527344
TCGA-55-7573-01A 0.040085657 0.060908369 0.074559833 0.0621011470
0.208446321 0 0.039326048 0.039226059 0 0 0.026670957
0.0118487650.0441163470.065625862 0.044918876 0.185748715 0.020986522
0.071048176 0 0 0.004382346 0.04 0.194801826 0.997437667
TCGA-55-7574-01A 0.103483034 0.036176985 0.064313509 0.083935486 0
0.275428978 0.048090193 0 0.017912565 0 0 0 0.005149042
0.128565915 0.055479278 0.1139588230 0 0.066598487 0 0
0.000907704 0 0.407686225 0.912663738
TCGA-55-7576-01A 0.027250608 0 0.028008326 0.0883707 0 0.181931636
0.028048125 0 0.055881281 0.001456698 0 0.021539815 0.022639144
0.088840312 0.073887757 0.170374612 0.089990296 0.031100735
0.084194976 0 0 0.006484978 0 0.327525702 0.9469874
TCGA-55-7724-01A 0.029749729 0 0.06791291 0.069685461 0 0.089269265
0.037208066 0 0 0.042303267 0.001030047 0 0 0.153260469
0.098921454 0.163693651 0.07908887 0.066278991 0.058706367 0 0
0.042891452 0 0.537874713 0.848627981
TCGA-55-7725-01A 0.083480887 0 0.0073001130.054741575 0 0.180793627
0.046668303 0.040857409 0 0.086151205 0 0 0 0.112421029
0.1198405820.100489309 0.054624484 0.015621568 0.093521392 0 0
0.003488518 0 0.3773118820.926438815
TCGA-55-7726-01A 0 0 0.157518922 0.015879807 0 0.187100987
0.032121489 0 0.003424017 0 0 0.0027440110.00074825 0.103788845
0.077870085 0.099431753 0.240582786 0.012092145 0 0.042918332
0.01090251 0.012876061 0 0.387765807 0.921453712

TCGA-55-7727-01A 0.0594911830.054378805 0.055246529 0.094615192 0
0.163135993 0.062544396 0.003066863 0 0.0390411680 0 0
0.053321332 0.1119178540.175789451 0.0531199320.019949332 0.054288894
0 0 9.30739E-050 0.341437483 0.94108499
TCGA-55-7728-01A 0.013823032 0.032475472 0 0.043109553 0 0.05556789 0
0.001881302 0.061573566 0 0 0.009655954 0 0.500672573
0.033942828 0.212179664 0.008979724 0 0.024849248 0 0.001289193
0 0 0.7884691140.634596357
TCGA-55-7815-01A 0.0598981110 0.0251144340.033388398 0 0.244396084
0.06903359 0 0.00979038 0.048764629 0 0 0 0.108734842 0.037948265
0.207406629 0.094940807 0.020466681 0.028338565 0 0 0.011778583
0.04 0.185051522 1.007142085
TCGA-55-7816-01A 0.015781024 0 0.018521623 0.121995183 0 0.056027593
0.036526997 0 0.013044214 0.004242913 0 0 0.128978812 0
0.145992131 0.3606334110.034870293 0 0.059429129 0 0 0.003956677
0 0.464175578 0.884917233
TCGA-55-7903-01A 0.073659091 0 0.017916669 0.174526595 0 0.051525702
0.048743058 0.021379385 0.049285777 0 0.054828208 0 0.018054605
0.152830568 0.126295734 0.1054252110.056382675 0.027215251
0.019691818 0.001982837 0 0.000256817 0 0.341437352 0.945137853
TCGA-55-7907-01A 0.009557559 0 0.06817392 0.122018269 0 0.124814993 0
0.005044888 0.024706633 0.053296899 0 0.049198809 0 0.149477835
0.129247819 0.15185677 0.0111084960 0.10149711 0 0 0 0 0.474130629
0.87982481
TCGA-55-7910-01A 0.043818452 0 0.334158947 0.016736162 0 0.190247191
0.008714289 0.033971349 0.036692423 0 0.025088702 0 0.003310048
0.134148787 0.026733716 0.030285637 0 0.087326636 0 0.009465689
0 0.019301972 0.33 0.060656063 1.045175805
TCGA-55-7911-01A 0.0311154740 0.035885405 0.137214789 0 0.055810576
0.1606041110.045922927 0.009650695 0 0.085134448 0 0.035774161
0.01716335 0.066192613 0.125035705 0 0.159297863 0.035197883 0 0
0 0 0.374095564 0.9286234
TCGA-55-7914-01A 0.002277976 0 0.316334379 0.142788252 0 0.162323646

0.01307261 9.95215E-050.059720855 0.013013255 0 0 0 0.065548981
0.1117495040.074676787 0 0 0.038394234 0 0 0 0 0.363593076
0.932040416
TCGA-55-7994-01A 0.021617447 0 0.021279984 0.180978634 0 0
0.058853264 0.037645848 0.041915084 0.003260514 0.058796657
0.009125078 0 0.146695484 0.131676582 0.179276732 0.031442567
0.019541842 0.057894284 0 0 0 0 0.461087741 0.886495316
TCGA-55-7995-01A 0.026879802 0.003655799 0.001721328 0.167875783 0
0.091840753 0.027885328 0.018414054 0.053293019 0.026315231
0.006628152 0.00552129 0.010728336 0.335710721 0.128530796 0.07817308
0 0 0.016826527 0 0 0 0 0.655723693 0.765756558
TCGA-55-8085-01A 0.041233136 0 0.073170664 0.180453166 0 0.007359797
0.055304803 0.041914986 0.10259069 0 0 0.030639208 0.032323756
0.180965626 0.102165954 0.092549863 0.02014562 0.027620725
0.007251637 0 0 0.00431037 0 0.315516562 0.955421185
TCGA-55-8087-01A 0 0.001612889 0 0.021397295 0 0.122478385 0
0.006137627 0.026950895 0 0 0.0411442780.020846463 0 0.018434228
0.108433181 0.457043463 0 0.172213567 0 0 0.003307728 0
0.367800408 0.93603981
TCGA-55-8089-01A 0.014546934 0 0.033428523 0.195983733 0 0
0.078371455 0.02317339 0.012562782 0.009976535 0.046181805
0.037717921 0.002719487 0.1149152350.188525204 0.129902828
0.016985878 0.007153439 0.087854853 0 0 0 0 0.609036136
0.800077537
TCGA-55-8090-01A 0.001468672 0.016250923 0.034852616 0.035925543 0
0.210322834 0.032751659 0.05381296 0.012140131 0 0 0.017274846
0.044382171 0.107029086 0.068426777 0.184293343 0.1187829810
0.052812985 0 0 0.009472474 0.04 0.191409757 1.003624193
TCGA-55-8091-01A 0.014491634 0 0.046984867 0.024480036 0 0.177218875
0.004441558 0 0.041334432 0 0.014684269 0.031950649 0.005790415
0.104384893 0.105158737 0.143761461 0.171988794 0.037632597
0.0621101 0 0 0.013586681 0 0.448362178 0.8932641
TCGA-55-8092-01A 0 0.029703956 0.403974206 0.209506181 0 0

0.046345079 0.007780465 0.064368851 0 0 0.003982335 0 0.0382442
0.103036636 0.075453468 0 0 0.017604623 0 0 0 0 0.574961799
0.837667587
TCGA-55-8094-01A 0.1177523740 0.087825863 0.092139768 0 0.054913281
0.031641415 0.081956996 0 0 0 0.037319315 0 0.1179742190
0.09929129 0 0.237193377 0.04041304 0 0 0.001579062 0.53
0.034026715 1.071435308
TCGA-55-8096-01A 0 0 0.174678131 0 0.000138908 0.089271519 0 0
0.012977532 0.002100683 0.037042878 0 0.01375016 0.32586046
0.049893072 0.250513894 0.015550751 0 0.0282220110 0 0 0
0.429035958 0.906670253
TCGA-55-8097-01A 0.010006292 0.030882666 0.086536649 0.0527664 0
0.244053933 0.0071197160.005797481 0.05430154 0 0 0.019589751
0.014502601 0.0116140070.029772855 0.167756375 0.141082032
0.0182227110.105897828 0 0 9.71645E-050 0.322404833 0.94753884
TCGA-55-8203-01A 0 0.073774075 0.27097899 0.1177654670 0.043459848
0.048869577 0.023503715 0.070633089 0 0.006883188 0 0
0.137908452 0.078477931 0.064661947 0.031269381 0.000335305
0.022242522 0 0 0.009236513 0 0.431843948 0.904465362
TCGA-55-8204-01A 0.006856228 0 0.297241798 0.009853657 0 0.135558883
0.038701877 0 0 0 0 0.033762258 0.00916056 0.046908224
0.147079577 0.065838834 0.060576705 0 0.127958348 0 0
0.020503051 0 0.396930528 0.917414164
TCGA-55-8205-01A 0.014320405 0 0.065226513 0.10103763 0 0.116480374
0.088948192 0.016410376 0 0 0 0.038168642 0.025933219
0.050906612 0.092147986 0.272336866 0.094154633 0 0.012528321 0
0.006552262 0.004847968 0 0.43635262 0.899167299
TCGA-55-8206-01A 0 0.00949419 0.064757154 0.018559995 0 0.122634074
0.014822515 0.0111025270.017506131 0 0 0.0081254110.244609668 0
0.00312522 0.094569507 0.215506041 0.018970937 0.155694255 0
0.000522374 0 0 0.521099832 0.861185263
TCGA-55-8207-01A 0.038976132 0 0.008823451 0.070204142 0 0.170613838
0.002461969 0.0096644110.022954468 0.000171413 0 0.003081907

0.042074188 0.148159081 0.016100016 0.20028922 0.136238356 0.023280075 0.09383883 0 0 0.013068502 0 0.325033703 0.95025645
TCGA-55-8208-01A 0.00218637 0.006473273 0.091495376 0.02517101 0 0.1167936140.019557889 0.043051758 0.039486642 0.031839913 0 0.036824619 0 0.219204537 0.130225402 0.1642311320.009389802 0 0.049935827 0 0 0.014132836 0 0.531500787 0.849546811
TCGA-55-8299-01A 0.019629512 0.000615909 1.74619E-050.206276849 0 0.00130381 0.027380247 0.03838569 0.038459991 0 0.069705845 0.018021391 0.021499055 0.0411683840.10868717 0.088971406 0 0.240005948 0.074592154 0 0 0.005279176 0 0.648939727 0.770322539
TCGA-55-8301-01A 0 0.033163921 0.132772374 0.1397841170 0.043928433 0.010958829 0.023070438 0.050210831 0.001222444 0.009928398 0.024963449 0 0.179492681 0.228146228 0.078466422 0.004237177 0 0.039654258 0 0 0 0 0.71632751 0.725209357
TCGA-55-8302-01A 0.01490504 0 0.1111057640.048066525 0 0.070470476 0.007294508 0.055325327 0.006483127 0 0.00261359 0.027764364 0.022325644 0.16332411 0.182635276 0.157394194 0.019735164 0 0.1105568920 0 0 0 0.362644789 0.936846465
TCGA-55-8505-01A 0.025527482 0 0.124421027 0.002579227 0 0.146536265 0 0 0.075220062 0 0 0.030526517 0 0.372918366 0.028456174 0.1362067110 0 0.046142315 0 0 0.0114658540 0.419728881 0.910449856
TCGA-55-8506-01A 0 0 0.0909251160.086561627 0 0 0.032128525 0.033418963 0.024188496 0 0.001435465 0.018005433 0 0.418210824 0.060032757 0.185568684 0.003121696 0 0.045122718 0 0 0.001279696 0 0.5090891130.861338338
TCGA-55-8507-01A 0 0 0.062325959 0.065565736 0 0.096207596 0 0.04746733 0.002048184 0 0 0.047269982 0.014830837 0.3193588 0.046506595 0.207058413 0 0.036879636 0.01626424 0.010143759 0 0.028072935 0.015 0.271542139 0.990293242
TCGA-55-8508-01A 0 0.006795721 0.476134159 0.054552271 0 0.023789886 0.001494297 0.007050915 0.047809697 0 0 0.017641088 0

0.279540587 0.019300588 0.036035728 0 0 0.009962566 0 0
0.019892496 0 0.435464747 0.902171785
TCGA-55-8510-01A 0 0.023030758 0.032152485 0.1168725270 0.046588237
0.028862775 0.04047214 0.053491775 0 0 0.027020325 0.013017749
0.108423184 0.096171308 0.239304749 0.065033576 0.000167812
0.095778677 0 0 0.0136119210 0.531334697 0.85367231
TCGA-55-8511-01A 0.028994271 0 0.068160961 0.125419867 0 0.1341409
0.052965419 0.038435221 0.045243981 0 0 0.026522317 0.009772811
0.1361928 0.131631663 0.1405115320.009232602 0 0.048330338 0 0
0.004445318 0 0.43090602 0.901515957
TCGA-55-8512-01A 0.02207849 0 0.052508481 0.091401946 0 0.167866312 0
0.005826837 0 0 0.036990478 0.039433243 0.074936631 0.11686338 0
0.226927676 0 0.051251884 0.043864082 0 0 0.070050561 0.155
0.105350281 1.044435527
TCGA-55-8513-01A 0.009143842 0.049626337 0.052005339 0.017605765 0
0.092169943 0 0.013687853 0.024340677 0 0.002758248 0.012818929
0.003050302 0.207584097 0.023829974 0.295672774 0.089450701
0.024386061 0.067702469 0 0 0.014166689 0 0.508835439
0.861497216
TCGA-55-8514-01A 0.013444069 0.000985702 0.076534572 0.026498094 0
0.122271764 0 0.054046835 0.028150494 0 0.005468194 0.009798857
0.099622161 0.1188263620.044193197 0.246632265 0.0111583210.074145963
0.058995328 0 0.005624617 0.003603206 0.04 0.189665424
1.005565388
TCGA-55-8614-01A 0 0 0.108530894 0.050942628 0 0.1148835360.018781208
0.034573908 0.016079487 0.018447177 0 0.04167776 0 0.259525956
0.099899667 0.11519103 0.0097503110.005371998 0.091270591 0 0
0.015073851 0.03 0.219032631 1.000779489
TCGA-55-8615-01A 0.009334292 0 0.00635646 0.001774679 0 0.045747128 0
0.064773782 0.097673401 0.0117282670 0.019551848 0.024823627
0.488852145 0.012315122 0.123924881 0 0.065049967 0.028094401 0
0 0 0.035 0.200320517 1.055308217
TCGA-55-8616-01A 0.098791805 0.003140458 0 0.06913149 0 0.292043624 0

0 0.12072796 0.01757889 0 0 0.013571264 0.067576393 0.0599136 0.132271069 0.00974974 0 0.1155037070 0 0 0.035 0.208629701 1.001892278

TCGA-55-8619-01A 0.043789101 0.033542008 0.068195265 0.133953469 0 0.1183765930 0.034525917 0.044107342 0 0 0.023143244 0.006252717 0.1152063980.037787582 0.223752762 0.020865894 0.004496601 0.057767912 0 0 0.034237195 0 0.344705021 0.939622299

TCGA-55-8620-01A 0.016600504 0 0.212146683 0.074305689 0 0.049980959 0.016723331 0.017486625 0.0113613890.036201897 0 0.056222133 0 0.213045651 0.109313614 0.109090876 0.007745717 0 0.069774933 0 0 0 0 0.452677498 0.891538562

TCGA-55-8621-01A 0.014752045 0 0.091420829 0.0731147970 0.158346999 0 0 0.017421412 0 0 0.046647326 0.023452381 0.107614737 0.091534985 0.166131919 0.077981371 0 0.1173915280 0 0.01418967 0 0.335539619 0.943311021

TCGA-55-A48X-01A 0.038779587 0.17579053 0.088227283 0.082327983 0 0.196661425 0 0.000470562 0.073504067 0 0 0.031518177 0.000150292 0.094457532 0.042206044 0.090259624 0.023587666 0 0.062059228 0 0 0 0 0.407709454 0.912779343

TCGA-55-A48Y-01A 0.064177943 0 0.171797463 0.016014937 0 0.087870464 0.035308598 0.0224112630.020593215 0.036073555 0 0.012237755 0 0.336606205 0.047552755 0.089958269 0 0 0.040987343 0 0 0.018410235 0 0.335489463 0.948538911

TCGA-55-A48Z-01A 0.000907645 0 0.169188426 0 0 0.107580143 0 0.022526923 0.062267868 0.008452996 0 0.030219623 0 0.382162819 0.062413867 0.125138368 0.00542402 0 0.023717302 0 0 0 0 0.530924156 0.847926704

TCGA-55-A490-01A0 0.0011370250.098071997 0.074177196 0 0.105893129 0.015572688 0 0.1151283490 0 0 0.033310956 0.239733951 0.030925498 0.192702086 0.08048627 0 0.01249679 0 0 0.000364065 0 0.3165921110.957043079

TCGA-55-A491-01A0.040187781 0 0.13977824 0.031478506 0 0.077766146 0 0.016579989 0.063746792 0 0 0.063172241 0.015937303 0.304159063

0.124722328 0.080125534 0.000915538 0 0.041430538 0 0 0 0
0.452622821 0.891098632
TCGA-55-A492-01A 0.021929721 0 0.364193842 0.056868388 0 0.0711782790
0.044862622 0.035093519 0 0.012567803 0.015223996 0.007414592
0.065313542 0.032452621 0.1855835110.007651785 0.022756604
0.056909173 0 0 0 0.035 0.200976291 0.991436409
TCGA-55-A493-01A 0.069959269 0 0.06644842 0.069298579 0 0.118095895
0.107014667 0.019571254 0.015591737 0 0 0.071338856 0
0.129610155 0.093216934 0.196123227 0.02731924 0 0.014473417 0 0
0.001938349 0 0.465543412 0.885174838
TCGA-55-A494-01A 0 0 0.133726332 0.1180610730 0.073155623 0
0.084493566 0.1113588850 0 0.067714249 0.00809931 0.067693179 0
0.125926469 0.151483658 0.058287655 0 0 0 0 0.76 0.001199216
1.077394491
TCGA-55-A4DF-01A 0.005332687 0 0.089942704 0.018305337 0 0.21788389
0.040569198 0.013254874 0 0.015912205 0 0.020645822 0
0.251569734 0.196166292 0.080748471 0.00196952 0 0.047487556 0 0
0.00021171 0 0.444920365 0.89699535
TCGA-55-A4DG-01A 0.101898 0.020716516 0.184494399 0.1199217870
0.22741451 0.018696876 0.012160353 0 0 0 0.00174356 0.010627123
0.063330556 0.066982099 0.1190502630.00607239 0.009375755 0.035662051
0 0 0.001853761 0.025 0.237752248 0.98159375
TCGA-55-A57B-01A 0.0119824110 0 0.026784517 0 0.185270086 0
0.0113210810.039538498 0 0 0.042701602 0.072976568 0.049520491
0.017573741 0.147732779 0.224529262 0.059677492 0.103089487 0 0
0.007301984 0.015 0.276189767 0.969570603
TCGA-62-8394-01A 0.048216899 0 0.035686927 0.03811245 0 0.15174933 0
0.034447537 0.042607192 0 0.012638225 0 0.003802915 0.282251301
0.075694885 0.129194968 0 0.1101505840.035446788 0 0 0 0
0.371527164 0.934193201
TCGA-62-8395-01A 0.042010997 0 0.143755415 0.064030845 0 0.103178729
0 0.062934477 0.018842026 0.000405627 0 0.0364481170.007156144
0.102530478 0.052868208 0.214898464 0.108180678 0 0.042514993 0

0	0.000244801	0.005	0.298910234	0.957445173
TCGA-62-8397-01A	0	0.004622326	0.107168637	0.027282504	0	0.161818907	0	0.002226712	0.027814572	0	0	0.018137891	0.101595292	0.062052507	0	0.148555082	0.178319125	0.012781789	0.147624656	0	0	0	0.02	0.245884796	0.978391565
TCGA-62-8398-01A	0.017831667	0	0.031658748	0.042762224	0	0.171650885	0.024921315	0.018382309	0.082344389	0	0.031612664	0.018637584	0.007540494	0.206683587	0.127512043	0.1381956	0.062008939	0	0.0111727110	0	0.007084841	0	0.341888945	0.946670369
TCGA-62-8399-01A	0.016815724	0	0.082759548	0	0	0.256124133	0.037500421	0.022916158	0.005284	0.050590388	0	0.037054594	0.073093619	0.0031148230.032999272	0.127683502	0	0.097001546	0.102246638	0	0.024278036	0.030537596	0.245	0.0792114641.041063533
TCGA-62-8402-01A	0.046285264	0	0.030709299	0.1173953160	0.052102098	0.076370832	0.002791462	0.08451062	0	0.028622269	0.013931047	0	0.263666123	0.219108353	0.034068184	0.005624541	0	0.024814592	0	0	0	0	0.629497565	0.781419568
TCGA-62-A46O-01A	0.147072491	0	0.314946679	0.0236249	0	0.030202589	0.055277159	0.041374065	0.017790239	0	0.004925468	0.030275724	0	0.141895973	0	0.053653061	0	0.080106092	0.058855561	0	0	0	0.345	0.05900746	1.040605029
TCGA-62-A46P-01A0.02609336	0	0.154247334	0.081083675	0	0.081332752	0	0.033517683	0.041700636	0	0	0.047632888	0	0.146390166	0.021604148	0.261416925	0	0.032628798	0.054833693	0	0	0.017517942	0.055	0.162359314	1.014929596
TCGA-62-A46R-01A	0.0301165030	0.020829008	0.050699166	0	0.223388583	0.008381607	0	0.054577984	0	0	0.025757219	0.063518529	0.059093452	0.024866153	0.17582446	0.037753494	0.089438977	0.135754865	0	0	0	0.02	0.245824642	0.979912769
TCGA-62-A46S-01A0.014400591	0	0.195169767	0.077802176	0	0.167006215	0.014577293	0.009729192	0.036169259	0	0	0.018683248	0.017238524	0.0801211880.088396396	0.107177305	0.084786957	0.005728449	0.05537067	0	0.0091172960.018525477	0.035	0.198483737	0.993668549

TCGA-62-A46V-01A	0.0268754110	0.015036835	0.005751038	0	0.149512694
0	0.001056214	0.000530512	0	0	0.02212448	0.027290651	0.099712256
0	0.215259696	0.194581615	0.1171748520.125093744	0	0	0	0.005
0.304907282	0.9612681
TCGA-62-A46Y-01A	0.01334893	0.006894729	0.0336011190.089452759	0
0.104409393	0	0.041887351	0.02724226	0	0	0.030009288	0.061908748
0.074877739	0.067334204	0.178983972	0.189804655	0	0.077382884	0
0	0.002861968	0.115	0.12088248	1.031484067
TCGA-62-A470-01A0.042800228	0	0.050370061	0.034220774	0	0.141908046
0	0.012407251	0.005957832	0	0	0.0484691130.004141382	0.154605119
0.0554395110.308131076	0.069637769	0	0.062499063	0	0	0.009412775
0.085	0.142012163	1.035021163
TCGA-62-A471-01A0.0335118920	0.136635429	0.136680279	0	0.040352745
0.010967943	0.151844087	0.04794961	0	0.046508816	0.051460162	0
0.145612217	0.050104874	0.03680688	0	0.052578571	0.0441531	0	0
0.014833395	0.085	0.141300673	1.026895872
TCGA-62-A472-01A0.008525767	0	0.040049254	0.164751056	0	0.087482883
0.024131889	0.038316283	0.046196347	0	0	0.071513153	0.008403747
0.037490673	0.094736896	0.145660738	0.1136615840.006601297
0.1110038220	0	0.001474612	0	0.322840379	0.950521246
TCGA-64-1676-01A 0	0	0.095594087	0.101509705	0	0.02674916	0.047980332
0.006596601	0.021217144	0	0	0.041751553	0.08597389	0.10435909
0.043641976	0.280538106	0.0904114430.0114078120.042269101	0	0	0
0.01	0.279414086	0.969458746
TCGA-64-1677-01A 0.03658933	0.019745528	0.187416597	0.105669848	0
0.007698416	0.165175991	0.04423022	0.015166137	0.045958153	0
0.016595343	0	0.126190076	0.157408308	0.047063739	0.011431933
0.001774308	0.006312288	0	0	0.005573786	0.005	0.305701577
0.95938162
TCGA-64-1678-01A 0.006449104	0	0.449007721	0.066376586	0	0.071913465
0.138371349	0.020517201	0.029205234	0.017416767	0.008971403	0	0
0.043144985	0.00647717	0.089945333	0.005934652	0.026972029	0.00889193
0	0	0.01040507	0.08	0.142879289	1.013980443

TCGA-64-1679-01A 0 0.000790453 0.058214097 0 0 0.072200948 0 0.010664361 0.019237489 0.006867073 0 0 0 0.418244836 0.044477278 0.1186474260.133709047 0.015674457 0.048542022 0.019713938 0 0.033016574 0 0.479425749 0.881955635

TCGA-64-1680-01A 0.007781652 0 0.07498315 0 0 0.218585616 0.003586836 0.007921525 0 0.032249937 0 0.01699735 0 0.016887051 0.060208289 0.276544725 0 0.032395173 0.245702843 0 0 0.006155854 0.05 0.174508061 1.014334687

TCGA-64-1681-01A 0.018765106 0.0119828480.04398653 0.0116694870 0.168614294 0 0.014572184 0.04842622 0 0 0 0.007605344 0.122788481 0.13689536 0.067619822 0.218008972 0.026187592 0.096745576 0 0 0.006132185 0 0.425720759 0.904086881

TCGA-64-5774-01A 0.061430601 0 0.236409307 0.063138537 0 0.169801096 0 0.041262164 0.109707945 0 0 0.015353319 0 0.156804253 0.007839267 0.100177023 0 0 0 0.02756459 0 0.0105118990.28 0.070188038 1.047217301

TCGA-64-5775-01A 0.006557641 0 0.009999357 0.045278866 0 0.20878811 0 0 0.018994387 0 0 0.052061224 0.01022418 0.327394951 0.021385717 0.267436845 0 0 0 0.025753346 0 0.006125377 0.075 0.151236293 1.062589679

TCGA-64-5778-01A 0.019308057 0.025389621 0.085646985 0.264583659 0 0 0.041763883 0.036908395 0.083151071 0.045459893 0 0.000103104 0 0.096531555 0.166568916 0.097179349 0.01023015 0 0.027175362 0 0 0 0 0.542769139 0.841669082

TCGA-64-5779-01A 0.031806631 0 0.389757865 0.1154354030 0.202861695 0.051023607 0 0.0227711210.008062491 0 0 0 0.091887247 0.051709584 0.017052883 0.013217091 0 0.004414383 0 0 0 0 0.323001013 0.949426932

TCGA-64-5781-01A 0 0 0.157175934 0.305644843 0 0 0.116930129 0.046093667 0.044381727 0.0311597110 0.072240496 0 0.078942828 0.038805855 0.065076071 0 0.022316003 0.017884643 0 0.003348093 0 0 0.399458201 0.918301986

TCGA-64-5815-01A 0.000981317 0 0.055195887 0.029770853 0 0.096808828

0.005985541	0.022544615	0.033271561	0	0.003393177	3.56193E-050
0.372546077	0.008889354	0.135823884	0.063363221	0.103960109
0.063486978	0	0	0.003942978	0	0.635661016	0.779906458
TCGA-67-3770-01A 0.015016803	0	0.024529178	0.045209265	0	0.213071375
0.018650797	0.032131666	0	0.026397498	0	0.037727514	0.006844929
0.003887627	0.047569187	0.259923225	0.0443111890.0545034110.154260976
0	0	0.015965359	0	0.3827091110.923323738
TCGA-67-3771-01A 0.010221713	0	0.176635058	0.035688719	0	0.101098465
0.024644301	0	0.0117648310.022782328	0	0.027824856	0	0.201490669
0.136813631	0.171434748	0.01854923 0	0.056578791	0	0	0.004472658
0	0.59820016 0.815461186
TCGA-67-3772-01A 0.032376197	0	0.190726782	0.059906329	0	0.123535895
0	0	0.023545964	0.017697877	0	0.001537884	0.032329325
0.046249343	0.021205948	0.141884488	0.20163595 0.031522925
0.070712587	0	0	0.005132505	0	0.3717522110.928424345
TCGA-67-3773-01A 0.036821253	0	0.049454067	0.083714288	0	0.185682277
0.010772808	0.026874954	0.026377203	0	0	0.022448433	0.003054644
0	0.083190853	0.161685932	0.1739491130	0.121582108	0	0
0.014392069	0.02	0.254702557	0.975594467
TCGA-67-3774-01A 0	0.017135371	0.104935491	0.076533547	0	0.198553712
0.017745802	0.024142966	0.003543416	0.005784533	0	0.055412448
0.002449332	0.089763982	0.106264558	0.132692232	0.016996322
0.023242367	0.097540333	0	0	0.027263587	0.005	0.291120027
0.963025557
TCGA-67-6215-01A 0.004207207	0	0	0.0422081170	0.158043541	0.001618478
0	0.01854608 0	0	0.017083877	0.026072498	0.095966258	0.004565208
0.232599329	0.20262556 0.025893058	0.164139045	0	0	0.006431743
0.04	0.1850992	1.014560358
TCGA-67-6216-01A 0	0.066486817	0.045937066	0.089095743	0	0.124645301
0	0.018185498	0.050947071	0	0	0.064240622	0.074326566
0.015709456	0.025023328	0.106526085	0.1708114840.025362632
0.1178014750	0	0.004900857	0.25	0.07549645 1.040776319
TCGA-67-6217-01A 0.006713372	0	0.120697285	0.0211666760	0.20375809

0.018618763 0.000892459 0.022766584 0.003647915 0 0 0.003901894
0.0171144240.072763045 0.226272979 0.1123489470.032624063 0.135907391
0 0 0.0008061130 0.333252054 0.943485978
TCGA-69-7760-01A 0.048641415 0 0.041741806 0 0 0.108598225 0
0.034879331 0.006657027 0 0 0.026003572 0.004535417 0.197868314
0 0.101757709 0 0.375558671 0.052885359 0 0 0.000873153 0.05
0.168208894 1.054395002
TCGA-69-7761-01A 0 0.009333323 0.231950815 0.146868218 0 0.168790755
0.069384723 0 0.009603266 0 0 0 0 0.1168912320.092199171
0.071786272 0.0231151130.0011383850.043942687 0 0 0.014996041 0
0.461720825 0.890235727
TCGA-69-7763-01A 0 0.0118722040.185267335 0.065861099 0 0.214270171
0.014845252 0.0091131810 0 0.008004594 0 0.000778978 0.1089681
0.01634983 0.259450633 0.00104752 0.048574878 0.043055836 0 0
0.012540389 0.005 0.309485647 0.95415871
TCGA-69-7764-01A 0.087317724 0 0.19259095 0.0795288110 0.32829826 0 0
0.008845978 0.031204749 0 0.023004014 0.003485221 0.076209623
0.073680489 0.061457313 0 0.01361878 0.020758088 0 0 0 0.02
0.256380763 0.979515083
TCGA-69-7765-01A 0.001658784 0 0.159350737 0 0 0.210014473
0.020424463 0 0.013635221 0 0.013436318 0.013335027 0
0.121543553 0.0775911910.124688979 0.165859368 0.013556765
0.062678541 0 0 0.00222658 0 0.362757847 0.931819934
TCGA-69-7973-01A 0.05639667 0 0.073249243 0.129820246 0 0.184730933
0.037366358 0 0.050181063 0 0 0.022179138 0.016642409 0.0527804
0.048301339 0.150415839 0.047567942 0.02365203 0.0951160630 0
0.0116003270.045 0.180848992 1.003123294
TCGA-69-7974-01A 0.023705932 0 0.001552658 0.038807846 0 0.1130130930
0.025851662 0.009008889 0 0 0.1190562970.010288606 0.030841907
0.063152136 0.287402538 0.155792922 0.028468919 0.083076106 0
0.000283686 0.009696803 0 0.408903416 0.912560652
TCGA-69-7978-01A 0.00807489 0 0.0772261120.067600924 0 0.219712552
0.031954363 0 0.0312438110.028298832 0 0.03846947 0.002360655

0.071090002 0.102182036 0.15565541 0.096275583 0 0.063459077 0 0
0.006396283 0 0.463308414 0.887001828
TCGA-69-7979-01A 0.031318667 0 0.1176609660.061848086 0 0.064494224
0.018090969 0.048797164 0 0 0.026887257 0.00143413 0 0.237165201
0.129648816 0.187759622 0.005283317 0.003879656 0.054957151 0 0
0.010774774 0.025 0.225789909 1.001128526
TCGA-69-7980-01A 0.017598883 0 0 0.120336435 0 0.238939327
0.008381692 0.041382376 0.019869701 0 0 0.046960772 0.006431457
0.040844499 0.149513682 0.207454532 0.032394448 0 0.067572069 0
0 0.002320128 0 0.42176901 0.907340167
TCGA-69-8253-01A 0.007280483 0.020804867 0.244468698 0.051361643 0
0.158623101 0 0.0119635050.002054595 0 0 0.057184709 0.011288856
0.09311689 0 0.1148396740.067126065 0.085467526 0.051280207 0 0
0.023139179 0.095 0.134635774 1.014916857
TCGA-69-8254-01A 0.02472711 0 0.141778976 0 0 0.178736597 0.007995912
0 0.009269799 0.039949648 0 0.004380655 0.0146511860.103612967
0.123328807 0.15619976 0.056573727 0.013738352 0.125056503 0 0 0
0.025 0.238563121 0.983716272
TCGA-69-8255-01A 0.035049546 0 0.0911648690.3161128360 0 0.164250435
0.028458632 0 0.0413116630 0.061587938 0.001404072 0.038045758
0.069668467 0.1161295690.002428767 0 0.034387447 0 0 0 0
0.394550699 0.921025883
TCGA-69-8453-01A 0.0160747110.003884595 0 0.055005681 0 0.121982653 0
0.031573661 0.014891473 0.001445229 0 0.012783064 0.001815963
0.391466839 0.073715816 0.1832701160.04124739 0 0.046880756 0 0
0.003962055 0 0.526135493 0.850161098
TCGA-69-A59K-01A 0 0 0.384313549 0 0 0.098671606 0.026245261
0.01186116 0 0.0225453110.0021955110.012124646 0 0.042431064
0.095179525 0.183821993 0.041526169 0.008021507 0.068310671 0
0.001506644 0.001245383 0.05 0.171557826 1.005437676
TCGA-71-6725-01A 0 0.033238972 0.092107078 0 0 0.033864676 0
0.016720896 0.000894646 0.002985887 0 0.015030884 0.136218423
0.05527842 0.020749829 0.340497991 0.094403675 0.088281085

0.029274754 0 0.024846146 0.015606637 0.08 0.144372923
1.036967087
TCGA-71-8520-01A 0 0.002754294 0.042450455 0.005388018 0 0.19428951 0
0.023637856 0.043954883 0 0.022197082 0 0.003779735 0.28999382
0.054450716 0.174068341 0.040471929 0.031375676 0.0711876830 0 0
0 0.325387217 0.956552907
TCGA-73-4658-01A 0.014351734 0 0.006429838 0.0393011130 0.105260225
0.07951451 0.033076351 0.015374997 0.040005099 0 0 0.012014653 0
0.086678352 0.218215215 0.27072488 0 0.063654602 0 0.003403368
0.0119950630 0.454573202 0.890498622
TCGA-73-4659-01A 0.002092038 0 0.046102475 0.026401381 0 0.154674205
0.004386773 0 0.007448013 0 0.036994525 0 0.037196422
0.048538575 0.005039123 0.419360583 0.043764607 0.017094326
0.122699777 0 0.001550913 0.026656264 0 0.318966592 0.955910916
TCGA-73-4662-01A 0.027598369 0.000692276 0.103892638 0.048342056 0
0.231324642 0.012385516 0.017419527 0.025337146 0.024095024 0 0
0.019004944 0.124350933 0.025192505 0.177080488 0.086890991
0.034492034 0.039035355 0 0 0.002865554 0 0.332132463
0.944749581
TCGA-73-4666-01A 0.002476471 0 0.007469084 0.251802684 0 0.023138015
0.1591150860.01214256 0.000553174 0 0.077428436 0 0.012426301
0.059703134 0.123286993 0.148671368 0.05293954 0.015482427
0.048905749 0 0 0.004458977 0 0.422997454 0.906020046
TCGA-73-4668-01A 0 0 0.18376161 0.04600895 0 0.1251139920.009636464
0.0071164920.036457946 0 0 0.006418532 0 0.331510707 0.033439134
0.106788689 0.031832745 0.017946329 0.0639684110 0 0 0
0.324610926 0.954850916
TCGA-73-4670-01A 0 0.030446371 0.135488075 0 0 0.13719243 0.004977677
0.01979157 0.004849094 0.028918064 0 0 0 0.2118360570 0.149339651
0 0.176730328 0 0.061834517 0 0.038596164 0.145 0.108154632
1.049303389
TCGA-73-4675-01A 0.003847158 0.017534521 0.04102216 0.048684814 0
0.029244124 0.015048885 0.005790177 0.037423944 0 0 0.028037299

0.01681348 0.347216447 0 0.144022232 0.123336198 0.041223102 0.089561332 0 0 0.0111941260 0.31638548 0.968047719

TCGA-73-4676-01A 0.010947512 0 0.016574772 0.050188676 0 0.076039552 0.032962134 0.01265804 0.006408814 0 0 0 0.059604464 0.146061095 0 0.361675757 0 0.08004383 0.123614402 0 0 0.023220953 0 0.315315239 0.960875707

TCGA-73-4677-01A 0.0116453850 0.162417102 0.05641656 0 0.319476758 0 0.014870633 0 0 0.005399729 0.031245072 0.035884887 0.00651361 0.027362685 0.153846917 0.074568001 0.027987792 0.061592768 0 0 0.010772103 0.04 0.19609412 0.996614263

TCGA-73-7498-01A 8.3262E-05 0.000473048 0.145100414 0.050372843 0 0.294057863 0.006710522 0 0.058777828 0 0 0.017992161 0.051210347 0.017494987 0.030997928 0.1789688 0.044639449 0.018636606 0.084483943 0 0 0 0.015 0.270685874 0.968058914

TCGA-73-7499-01A 0.013677001 0 0.099097873 0.254220356 0 0 0.058907024 0.037834298 0.069404657 0.016597974 0 0.002394316 0 0.094351265 0.173319131 0.097187062 0.039275405 0 0.043733639 0 0 0 0 0.618732722 0.799801119

TCGA-73-A9RS-01A 0.001094518 0 0.215047216 0.100998381 0 0.12765769 0.007670105 0.071253193 0.012336506 0 0 0.049941993 0.012003514 0.038459344 0.090786473 0.146427376 0.006423107 0.005131174 0.1105548710 0 0.00421454 0.1 0.129002634 1.022035892

TCGA-75-5125-01A 0.007837475 0 0.06482218 0.102154434 0 0.161176716 0.036215835 0.022986888 0.012818485 0 0.019550387 0.01641296 0.0117456350.013730815 0.1411992180.171414076 0.120638644 0.023117555 0.059793784 0 0 0.014384913 0 0.559744468 0.838320066

TCGA-75-5146-01A 0.007176144 0.067234517 0.220778402 0.085442415 0 0.202973041 0.000746598 0.019628481 0.009202655 0 0 0.026291107 0.019918825 0.010249871 0.052496485 0.165996615 0.03354578 0.017195217 0.046418323 0 0 0.014705524 0 0.355966547 0.934184742

TCGA-75-5147-01A 0.024796933 0 0.1107672950.017378924 0 0.237410258 0.0129372110 0.049222415 0.017524653 0.00214024 0 0.034561513

0.054803845 0.086773136 0.151363579 0.14749309 0.00683019 0.029581586
0 0.002563522 0.01385161 0 0.470754799 0.885708592
TCGA-75-6206-01A 0.033544532 0 0.025808662 0.030816541 0 0.04289802
0.008416509 0.042212351 0.015525076 0 0 0.0110219850 0.441168255
0.033489419 0.251438334 0 0.001225778 0.051828366 0 0
0.010606173 0 0.405638247 0.933530617
TCGA-75-6212-01A 0.095902623 0 0.085461497 0.019976262 0 0.13000327 0
0.007531384 0.039736305 0 0 0.014707818 0 0.042591865
0.010232855 0.171312646 0.222984986 0.015889608 0.1260201150 0
0.017648766 0.055 0.159965109 1.010783786
TCGA-75-6214-01A 0.014662897 0 0.007043576 0.090137826 0 0.133199123
0.022310076 0.042034574 0.0209811990 0 0.019048515 0 0.314359254
0.078277354 0.150001303 0.032231717 0.0199734 0.037379622 0 0
0.018359563 0.045 0.180305695 1.032796388
TCGA-75-7025-01A 0.025632018 0.068860907 0.064030873 0.103241723 0
0.157728338 0 0.010592008 0.053606199 0 0 0.051493493
0.048905745 0 0.0113734610.078059957 0.170697828 0.059438079
0.089303181 0 0 0.00703619 0 0.34015756 0.940345268
TCGA-75-7027-01A 0.006529481 0 0.132127461 0.051459763 0 0.179914421
0 0.014079717 0.037793927 0 0 0.02158293 0.009102979 0.153090171
0.068654093 0.155959989 0.037958316 0.000621213 0.1283785110 0
0.002747027 0.025 0.231775762 0.985187595
TCGA-78-7143-01A 0.0512133110 0 0.030221791 0 0.192336815 0
0.049199063 0.039094377 0 0 0.014187599 0.023739505 0.092978638
0.007576598 0.133688562 0.139179769 0.161899101 0.063529101 0 0
0.00115577 0 0.420759772 0.906384013
TCGA-78-7145-01A 0 0.005548847 0.013624221 0.00809839 0 0.12178836
0.026469464 0.004264337 0.013408315 0 0 0.008789236 0.032881314
0.249439029 0.016470765 0.160681491 0.1475481 0.063553327
0.125039231 0 0.001512057 0.000883516 0.035 0.205304705 1.01376694
TCGA-78-7146-01A 0.006094644 0 0.007141077 0.121996217 0 0.190645193
0.094604763 0.0100311280 0 0.003245509 0.019710078 0.040403083
0.206863413 0.065789778 0.073996377 0.034099756 0.104475656

0.0194421170 0 0.0014612110.035 0.209170446 1.00793305

TCGA-78-7147-01A 0.031937099 0 0.026625105 0.127964869 0 0.132735753 0 0.02012948 0.032633444 0 0 0.041835408 0.010813698 0.071145274 0.188847936 0.141719441 0.056533082 0.0112095060.105869905 0 0 0 0 0.393148162 0.922689702

TCGA-78-7148-01A 0.038080728 0 0.058568148 0.258097202 0 0.033792773 0 0.0303511550.082599087 0 0 0.044098228 0.014263947 0.06933305 0.069878419 0.150050501 0.087906775 0 0.062979987 0 0 0 0.04 0.195421924 1.004929354

TCGA-78-7149-01A 0.016895088 0 0.31799127 0.079740366 0 0.141646783 0 0.0274411750.035817663 0 0 0.043793214 0.0085113610.076638472 0.081390139 0.074368376 0.014759554 0.013910864 0.067095676 0 0 0 0.13 0.1121803331.026112023

TCGA-78-7150-01A 0.0861223 0 0.085248241 0.097550555 0 0.077639099 0.032940413 0.048656443 0.018640086 0.003572538 0 0.041748363 0 0.233176475 0.066028663 0.122382463 0 0.0591114560.024530716 0 0 0.002652188 0.05 0.168231463 1.01952537

TCGA-78-7152-01A 0.0054375110.07101123 0.071037983 0.140588423 0 0.169485187 0.010138724 0.026991765 0.074683987 0 0 0.037143034 0 0.129760674 0.078244594 0.078618154 0.059466088 0 0.040714518 0 0 0.006678127 0 0.331377837 0.946374419

TCGA-78-7153-01A 0.0111189350.006593694 0.206721972 0.17104477 0 0.127836477 0.002666265 0.029372037 0.024781587 0 0 0.076971925 0.003697877 0.023451974 0.036679089 0.138995133 0.030527428 0.020060203 0.089480632 0 0 0 0.095 0.133665701 1.022815016

TCGA-78-7154-01A 0.062362383 0 0.015151281 0.048001502 0 0.355974649 0 0.001397651 0.003545364 0 0.007260727 0.023714446 0.019766009 0.032445197 0.142767166 0.14793102 0.057837736 0.014261616 0.067583254 0 0 0 0.005 0.289385197 0.970328765

TCGA-78-7155-01A 0.082747816 0 0.010996444 0.229131733 0 0.095078852 0.077682703 0.044449534 0 0.017035407 0 0.013064631 0.009246304 0.072109161 0.095255875 0.157067157 0.08512793 0 0.0110064520 0 0 0.05 0.170299717 1.019515883

TCGA-78-7156-01A 0.064666342 0 0.068874389 0.109570794 0 0.218169178 0 0.047704909 0.002599981 0.02895417 0 0.046071725 0.02826843 0.017701333 0.1511023950.099300616 0.015448283 0.007206296 0.0943611560 0 0 0.245 0.081359732 1.063100184

TCGA-78-7158-01A 0 0.000847509 0.038272657 0.032378426 0 0.217117 0 0.006957689 0.018853799 0 0 0.0380633 0.031602254 0.029881558 0.052243306 0.259610019 0.12755362 0.030199765 0.107785104 0 0 0.008633992 0.045 0.179296097 1.008769743

TCGA-78-7159-01A 0.044913484 0 0.233061479 0.089908524 0 0.1102465220 0.000202515 0.060664534 0 0 0.036004179 0 0.15851156 0.067040388 0.128958937 0 0.029037088 0.008072821 0 0 0.033377971 0.02 0.261676223 0.973206831

TCGA-78-7160-01A 0.008205782 0 0.03592424 0.106779191 0 0.301141905 0.01102187 0.01530396 0.018179406 0.001921515 0 0.010187784 0.012537018 0.0831922110.089577348 0.189991617 0.04329192 0.018789255 0.05175933 0 0 0.002195647 0 0.473041714 0.8809466

TCGA-78-7161-01A 0.002719643 0 0.186021515 0 0 0.324991955 0 0 0.094561893 0 0 0.015578613 0.007576228 0.179607759 0.05731213 0.077204789 0 0.036594691 0 0.004842906 0 0.012987879 0.245 0.079833317 1.05206624

TCGA-78-7162-01A 0.009406042 0.025415759 0.068086128 0.131045421 0 0.259426776 0 0.026741423 0.001910746 0.010897503 0 0.0193051150 0.046748927 0.066758022 0.144575927 0.053496655 0.043505493 0.092680063 0 0 0 0 0.332281307 0.945721471

TCGA-78-7163-01A 0.057538095 0 0.16036796 0.24142596 0 0.271098775 0.002128381 0.016507136 0.007039439 0.022082778 0 0.022083922 0.006920387 0.01676208 0.038229874 0.05365574 0.022080449 0.023989935 0.036960713 0 0 0.0011283770.225 0.085437194 1.063948986

TCGA-78-7166-01A 0.051733549 0 0.250939581 0.097254961 0 0.042125236 0 0.04840353 0.016500257 0.022509129 0 0.044953757 0 0.24609783 0.129101291 0.021709456 0.002621632 0 0.02604979 0 0 0 0.03 0.216743324 0.999991006

TCGA-78-7167-01A 0.045837572 0 0.1130249750.098484523 0 0.144104916 0

0.044201973 0.039285442 0 0 0.043485296 0.039887664 0.057845401 0.075982536 0.163419653 0.048795369 0 0.08564468 0 0 0 0.465 0.043814961 1.052649569

TCGA-78-7220-01A 0.0393115510 0.019314099 0.18243983 0 0.11213155 0.071883105 0.1118436190.000122096 0 0 0.029048648 0.010156623 0.1197788030.059739298 0.142990592 0.044265203 0 0.051924585 0 0 0.005050399 0.055 0.16203491 1.019826262

TCGA-78-7535-01A 0 0.038430405 0.02453995 0.041244379 0 0.074554587 0 0.000272592 0.008031356 0 0.02065635 0.006542004 0.00170119 0.346195626 0.009365699 0.241562092 0.183857245 0 0.003046526 0 0 0 0.005 0.292510037 0.99520727

TCGA-78-7536-01A 0.004929533 0 0.1167583620.037925832 0 0.122941779 0.012284602 0.018185171 0.002013698 0.024134228 0 0.101168976 0.007263659 0.065955423 0.142660953 0.251097502 0.046085443 0 0.040804661 0 0 0.005790178 0 0.35668487 0.938585773

TCGA-78-7537-01A 0 0 0.296618357 0.1133553610 0.059446906 0 0.003490354 0.067087015 0 0 0.047562783 0.000989967 0.059822114 0.008183013 0.155512288 0.083535816 0.033649648 0.070746378 0 0 0 0.04 0.188727604 0.995468321

TCGA-78-7539-01A 0.001445774 0.003344081 0.031361081 0.1859113350 0.1337119040.052201635 0.028510897 0.0502311540 0 0.031166826 0.012810904 0.053253822 0.067125423 0.150699206 0.088073295 0.040508923 0.066495802 0 0 0.00314794 0 0.324442744 0.949479357

TCGA-78-7540-01A 0 0.029477059 0.013421988 0.063156302 0 0.101498853 0 0 0.053888314 0 0 0.081571041 0 0.247583218 0.023619926 0.2688116 0.055209013 0 0.061762686 0 0 0 0.06 0.159304516 1.040187573

TCGA-78-7542-01A 0.0110644540 0.020432855 0.049014854 0 0.12970335 0.058966364 0.073206033 0.023003143 0 0.005320631 0.034356292 0 0.145952236 0.147871633 0.092510509 0.050610442 0.10848205 0.049260574 0 0.00024458 0 0.04 0.184075886 1.026211841

TCGA-78-7633-01A 0.036223974 0 0.006517714 0.03195469 0 0.257602014 0 0.047557227 0 0 0 0.045343605 0.065919866 0.080658171

0.019243297 0.239029236 0.012440085 0.086914277 0.070029828 0 0
0.000566016 0.33 0.061426976 1.059057041
TCGA-78-8640-01A 0.0075781150 0.128742666 0 0 0.1363852 0.027984125
0.00870155 0.00507124 0 0.0241190360.023728764 0 0.326020974
0.126282839 0.165703423 0.0071186980 0.012563371 0 0 0 0
0.392472815 0.926421754
TCGA-78-8648-01A 0 0.010082076 0.1011002810.1184925550 0.047185322
0.033328989 0.008278938 0.022420833 0 0.016193957 0.002604616 0
0.444647767 0.039127138 0.120848136 0 0 0.035689392 0 0 0 0
0.72925004 0.709578494
TCGA-78-8655-01A 0.020480099 0 0.016237791 0.1105937580 0.095978185
0.0113062170.009446172 0.037313065 0 0 0.018358393 0.011598911
0.297452491 0.076929459 0.18436413 0.026376468 0 0.0832494 0 0
0.00031546 0 0.508832956 0.860423677
TCGA-78-8660-01A 0.01369469 0 0.050256868 0.127662464 0 0.08075131
0.040648769 0.062847393 0.0532119250 0.006841024 0.046013465 0
0.22999801 0.076841548 0.151594203 0.0115569090 0.047178956 0 0
0.000902465 0 0.372235317 0.9304226
TCGA-78-8662-01A 0.055058899 0 0.027457022 0.126639322 0 0.14269325 0
0.124320321 0.01430031 0 0 0.0311188840.0035603 0.154589858
0.132467257 0.161400535 0.017823703 0 0.007082924 0.001487416 0
0 0.195 0.09194996 1.063330681
TCGA-80-5608-01A 0.013082359 0 0.05290271 0.141488153 0 0.179119702
0.016669785 0.01026856 0.057977734 0 0.06176842 0.005848604 0
0.097698712 0.0811674760.132805986 0.032649815 0.024506861
0.046488366 0 0 0.045556757 0.04 0.188468787 1.009597322
TCGA-80-5611-01A 0 0.0241193870 0.039772779 0 0.079798616 0.012216239
0.03457983 0.044589258 0 0 0.005903706 0.05056263 0.441804315
0.091423633 0.131884762 0.016370566 0 0.026974279 0 0 0 0
0.522497838 0.853580307
TCGA-83-5908-01A 0.018982028 0 0.016825014 0.321463584 0 0
0.189834632 0.040261856 0.025372922 0.007458684 0.043575779 0
0.010946928 0.051038941 0.121531592 0.069640307 0.065624524 0

0.017443209 0 0 0 0 0.458689213 0.887938239
TCGA-86-6562-01A 0.0115836130 0.09323658 0.052936689 0 0.151552957 0.0041109560 0.058551553 0 0 0 0 0.23224222 0.035765142 0.26118303 0.00723536 0.017932326 0.010308933 0.041287661 0 0.022072981 0 0.391635232 0.9224322
TCGA-86-6851-01A 0 0 0.241559915 0.122024923 0 0.056751913 0.05827529 0.048529688 0.061338812 0 0.0801173960 3.74993E-050.101050311 0.084957376 0.101329365 0.01268423 0 0.031343284 0 0 0 0 0.51754244 0.865373531
TCGA-86-7701-01A 0.056713178 0 0.21134447 0.031018478 0 0.207081879 0.082682237 0.048705917 0.02132987 0.045760951 0.023753874 0 0 0.0355501150.044781648 0.099141432 0.039345646 0.0211230120.023960014 0 0 0.00770728 0 0.336387301 0.941863231
TCGA-86-7711-01A 0.006364687 0 0.047793583 0.001050165 0 0.196438013 0 0 0 0 0.077989631 0 0.004156625 0.3521117510.03837251 0.133387432 0 0 0 0.1115996310 0.03073597 0.04 0.189279315 1.037916626
TCGA-86-7713-01A 0.035981988 0 0.195229484 0.033177202 0 0.172250578 0.031941305 0.034194077 0.016006038 0 0.009263405 0.005520616 0.0119124610.1139090250.090453519 0.089097689 0.027706667 0.078036156 0.036807575 0 0 0.018512214 0.165 0.103302592 1.032838035
TCGA-86-7714-01A 0.001435025 0 0 0.059494509 0 0.230188398 0 0 0.015662291 0 0.006783677 0.006244097 0.029923269 0 0.012540702 0.130334656 0.336518474 0.044332152 0.1120562110 0 0.014486541 0 0.323678238 0.951956199
TCGA-86-7953-01A 0.0118422670 0.009027706 0.042624737 0 0.320317742 0.04845521 0.01883616 0.0505113560 0 0.012995972 0.065932785 0.105096992 0.041873004 0.167121314 0.04169962 0.03983011 0 0.000971753 0 0.022863272 0 0.35189603 0.938886684
TCGA-86-7954-01A 0.027235959 0 0 0.023769733 0 0.185370077 0 0.034877629 0.0115420070.024052124 0 0.057583066 0.002715539 0.035239354 0.199224077 0.208566175 0.02227895 0.059626432 0.105860289 0 0 0.002058588 0 0.596783161 0.806207031

TCGA-86-7955-01A 0.062847375 0 0.05370663 0.135105342 0 0.124364752 0.0062114040.030475908 0.067655728 0 0 0.089770514 0 0.123332279 0.082531839 0.085586555 0 0.017504835 0.120906839 0 0 0 0.415 0.050354822 1.068893447

TCGA-86-8054-01A 0.064208026 0 0.035804741 0.049970831 0 0.217868957 0.063327219 0.012455149 0 0 0.02066571 0 0.008329156 0.14481148 0.04267511 0.136153947 0.1161651080.010798809 0.050425954 0 0 0.026339804 0.075 0.148600954 1.018673036

TCGA-86-8055-01A 0.00429687 0 0.142015432 0 0 0.165320637 0.008232871 0 0.039208898 0 0.000437975 0 0.002559458 0.278499392 0.0736206 0.226764204 0.035104233 0.005406647 0.002730453 0.005438347 0 0.010363983 0 0.437648557 0.899928244

TCGA-86-8056-01A 0.026458248 0 0.030762264 0.056738177 0 0.212146783 0.021629816 0 0.016347519 0 0 0.0156411210.056254648 0.100326739 0.051682538 0.24076234 0.076227818 0.008985073 0.075962962 0 0.001234259 0.008839697 0.01 0.28149791 0.967314461

TCGA-86-8073-01A 0.0169524 0 0.032040046 0.065122039 0 0.14189744 0 0.032924752 0.022240696 0 0 0.021416442 0.1145761520.149249353 0.064646746 0.215808496 0.06060639 0 0.062519048 0 0 0 0.015 0.271458306 0.974046669

TCGA-86-8074-01A 0.018127536 0 0.059254531 0.040081364 0 0.078205239 0.040707905 0.026801462 0.043099555 0 0 0.026659722 0.010842239 0.178836087 0.071035242 0.209137563 0.0961159970.020554457 0.03909527 0 0.017214544 0.024231289 0 0.335706925 0.946028181

TCGA-86-8075-01A 0.000148479 0 0.160533121 0.0403211160 0.111106243 0.007769662 0.020776986 0.032847449 0 0 0 0.0118456080.101086387 0.059872527 0.193696556 0.080149704 0.095780092 0 0.059350473 0.018948696 0.0057669 0 0.333148277 0.944957984

TCGA-86-8076-01A 0.003980935 0.0112356690.1842213110.1867112460 0.037945661 0.038856948 0.028776683 0.073642048 0 0 0.00459883 0 0.212341502 0.062537382 0.06795657 0.043549672 0 0.035170108 0 0 0.008475434 0 0.475433256 0.882706888

TCGA-86-8278-01A 0 0 0.135679929 0.050143777 0 0.248176468 0

0.009701961 0.00589992 0 0.008523463 0.018470729 0.005209847 0.090685252 0.076594573 0.120883096 0.101486851 0.049492423 0.072249948 0 0 0.006801765 0.095 0.132184458 1.023098639

TCGA-86-8279-01A 0 0.000307415 0.0755339110.014319081 0 0.048705766 0 0.0119282040.017801071 0 0.003407415 0.000882475 0 0.361695953 0.035270885 0.10711468 0.307123188 0.000304595 0.01560536 0 0 0 0 0.331081831 0.973676161

TCGA-86-8280-01A 0.008570181 0 0.094369086 0.047292635 0 0.205443861 0 0.040181787 0.038810643 0 0 0.031262065 0 0.120530788 0.097930927 0.133500441 0.072416973 0 0.106869999 0 0 0.002820615 0 0.544243974 0.850490392

TCGA-86-8358-01A 0.008052805 0 0.1483794110 0 0.079305964 0 0.019986175 0 0.059314879 0 0.072309345 0 0.285185401 0.103642408 0.198978189 0.013550861 0 0 0 0.0112945620 0 0.417479737 0.911385524

TCGA-86-8359-01A 0 0 0.085755541 0.318567736 0 0.064554912 0.045515498 0.082898966 0.1113305720 0 0.022232441 0 0.151804309 0.051967782 0.019605396 0 0 0 0.00725104 0 0.038515806 0.02 0.259661983 0.991156553

TCGA-86-8585-01A 0.023014479 0.013678725 0.050422969 0.137847845 0 0.072781874 0.099166924 0 0.064352185 0 0 0.009917653 0 0.332140589 0.058488716 0.088145034 0.000882147 0 0.04916086 0 0 0 0 0.538960944 0.845049772

TCGA-86-8668-01A 0.006454778 0.029568123 0.135614477 0.048717152 0 0.163159367 0 0.028551388 0.042863482 0 0 0.0311895130.003293359 0.146128649 0.085145044 0.134094357 0.055169345 0 0.086489048 0 0 0.003561918 0 0.322727032 0.94804279

TCGA-86-8669-01A 0.0008643110 0.148704676 0.12087739 0 0.210467891 0.002791748 0 0.0603728 0 0 0.008317824 0.037674191 0.12379856 0.054208547 0.129300954 0.058685236 0.029150107 0 0.014785765 0 0 0.015 0.275234739 0.968977552

TCGA-86-8671-01A 0.009847723 0.05822275 0.032515798 0.1147274290 0.126942167 0 0.028160405 0.104843689 0.0119598350 0.024610738 0

0.197524391 0.12387165 0.083273244 0.038433356 0 0.045066825 0 0
0 0 0.652407717 0.783525386
TCGA-86-8672-01A 0 0 0.1176229110.206355198 0 0.0301140690.092552398
0.03318449 0.049674534 0 0.040228554 0.002663406 0.006487958
0.150095432 0.061468207 0.18456749 0.0114134940 0.013571859 0 0 0
0 0.452828524 0.891710061
TCGA-86-8673-01A 0 0.00923864 0.1511378820.037434837 0 0.084585852
0.000106335 0.002201644 0.077228776 0 0 0.091373019 0
0.315240186 0.032273415 0.149523303 0 0 0.036039431 0 0
0.01361668 0.035 0.204342877 1.013983977
TCGA-86-8674-01A 0.050414396 0 0.180909859 0.047976094 0 0.051985832
0 0.13446541 0.040205671 0 0 0.02768773 0 0.2922115440.058801824
0.046476987 0 0.05040417 0.018460483 0 0 0 0.055 0.162477329
1.02203858
TCGA-86-A456-01A0.030027954 0 0.010670183 0.035551881 0 0.121377663
0 0.039304918 0.014249225 0.021775253 0 0.013658982 0
0.195250083 0.073694062 0.3442110380 0.071053875 0.006334601
0.021224812 0 0.001615468 0.005 0.306640256 0.971329735
TCGA-86-A4D0-01A 0.050521476 0 0.083698256 0.084541446 0 0.15140551
0.126287014 0.051579404 0.012369663 0 0 0.054925224 0.002911524
0.038687308 0.050635908 0.120708673 0.145654412 0.003385179
0.0195361130 0 0.003152889 0.04 0.190357969 1.00037622
TCGA-86-A4JF-01A 0.009679163 0 0.08589442 0 0 0.137902928 0.012115895
0.049353256 0 0.049700872 0 0.08720969 0 0.155253733 0.09853531
0.141748288 0.0111555650.029381354 0.1235852 0 0 0.008484324 0.015
0.274053973 0.973484564
TCGA-86-A4P7-01A0.00806184 0 0.209439909 0.104726601 0 0.082598443
0.00911859 0.058767238 0.046764474 0 0 0.017794996 0.007806378
0.132477357 0.025314634 0.174746467 0.014174495 0.00935846
0.096883553 0 0 0.001966564 0.005 0.304258072 0.954658492
TCGA-86-A4P8-01A0.023271616 0 0 0.091289255 0 0.313028173 0 0 0
0 0.04327034 0.0117477020.015012498 0.157791563 0.056733447
0.2115396110.025105851 0 0.051209943 0 0 0 0 0.406672164

0.914179778

TCGA-91-6828-01A 0.002650793 0 0.095435739 0.138665104 0 0.188593083 0.00603502 0 0 0 0 0.05410214 0.040152751 0.002365017 0.0877887 0.244629903 0.070858247 0 0.046688744 0 0 0.02203476 0 0.398482989 0.9164006

TCGA-91-6829-01A 0.087574496 0 0.052855156 0.084169444 0 0.065624215 0.037050085 0.001384524 0.032346364 0 0.005971926 0 0 0.203212026 0.022543789 0.251755303 0.02170117 0.018587928 0.101361385 0 0 0.013862189 0.005 0.303898095 0.960371895

TCGA-91-6830-01A 0.05428342 0 0.049763135 0.098762169 0 0.142114424 0.022975906 0.001523901 0 0 0.021737861 0.009842109 0.005351118 0.201730841 0.12474398 0.138198061 0.004795495 0.006529629 0.078997737 0 0.007775214 0.030875001 0 0.34305644 0.943991307

TCGA-91-6831-01A 0 0 0.137089888 0.092877681 0 0.08039575 0.006992321 0 0.051310322 0 0 0.036685678 0 0.252081522 0.072587741 0.23397148 0.004724554 0 0.012832654 0 0 0.018450407 0 0.476673544 0.87850879

TCGA-91-6835-01A 0.025260084 0.028462782 0 0.073326389 0 0.242403235 0.018078407 0.008527868 0.070725883 0.044989814 0 0 0 0.205095909 0.08177973 0.153162513 0.0116073030 0.036580082 0 0 0 0 0.581588213 0.821925387

TCGA-91-6836-01A 0.002032008 0 0.158278466 0.09679782 0 0.131500457 0.1189066260.018218074 0.003286574 0 0.003838299 0.028188579 0.026275932 0.146581637 0.032550732 0.109953434 0.036083843 0.0453111150 0.022502191 0 0.019694214 0.12 0.1178947931.027133233

TCGA-91-6840-01A 0.083701019 0 0.08912225 0.015852243 0 0.230171746 0 0.017015894 0 0.017137476 0 0.059755743 0 0.176902826 0.149209463 0.088878762 0.004173074 0 0.068079503 0 0 0 0 0.380291529 0.926527658

TCGA-91-6847-01A 0.092962491 0 0.00231632 0.097356842 0 0.1117975970 0.027749391 0.034669227 0 0 0.154505269 0.009800697 0.185823214 0.070053783 0.045188509 0.061695365 0 0.096945867 0 0.009135429 0 0.695 0.0111210881.098332824

TCGA-91-6848-01A 0.002216979 0 0.053449982 0.11307324 0 0.168249012 0.080532201 0 0 0 0.005272023 0.027169875 0.031772785 0.1741178860.123641703 0.194970803 0.013019403 0 0.00852504 0 0 0.003989068 0 0.347971324 0.9433223

TCGA-91-6849-01A 0.0149871120.027468762 0.186742483 0.094753015 0 0 0.028450592 0.024215781 0.020900944 0 0.008985374 0 0 0.3979894 0 0.1173702130.012305129 0.039636758 0.018421989 0 0 0.007772448 0 0.398475894 0.92310228

TCGA-91-7771-01A 0.009527609 0 0.082257309 0.10226887 0 0.39278724 0.003950779 0.000178181 0 0 0 0.02132495 0.008179406 0.032088477 0.082206455 0.16463857 0.030123423 0.009438334 0.056060159 0 0 0.004970237 0 0.418380637 0.907777875

TCGA-91-8496-01A 0.000670798 0.000535228 0 0.04797427 0 0.098013808 0 0.003304913 0.012891891 0 0 0.028536392 0.079255925 0.142794754 0.0113704780.281573269 0.108047643 0 0.139279996 0 0.012503128 0.033247506 0 0.423079447 0.90637851

TCGA-91-8497-01A 0.012366938 0.077481618 0.264151693 0.065264355 0 0.108445928 0.000923069 0.024310368 0.058493505 0 0 0.02489827 0.01176061 0.030503402 0.013459594 0.1178464540.103650675 0.00986316 0.0670311370 0 0.009549221 0 0.392689992 0.921993469

TCGA-91-8499-01A 0.026623129 0 0.0928306 0.105633749 0 0.141111544 0.02873692 0.028800628 0.006727495 0.05296855 0 0.00189233 0 0.1158261520.10871741 0.165084992 0.096464021 0 0.022979055 0 0.005514739 8.86845E-050 0.372224873 0.928392367

TCGA-91-A4BC-01A 0.0079061130 0.141624171 0.392643027 0 0 0.129420222 0.021712884 0.012515269 0.024790799 0 0.039271888 0 0.045653677 0.0430115660.080975852 0.004790329 0 0.053245183 0 0 0.00243902 0 0.52862547 0.850441817

TCGA-91-A4BD-01A 0 0 0.083789336 0.017579536 0 0.129791813 0 0.019229924 0.025623867 0 0 0.025501958 0.171733234 0.060173376 0.016073347 0.2115861580.037241709 0.034705349 0.166970394 0 0 0 0 0.396161378 0.917898111

TCGA-93-7347-01A 0.013431871 0 0.017442837 0.1143510770 0.095507641 0

0.00536684 0.122198008 0.052396593 0 0.055625338 0 0.232687214
0.051951287 0.125473209 0.017522255 0.008285783 0.085028815 0 0
0.002731231 0 0.565959872 0.830426865
TCGA-93-7348-01A 0.0011843060 0.184202062 0.016230582 0 0.189126104
0.012679616 0 0.081889736 0 0.007216424 0 0 0.314927373
0.058042314 0.087017009 0.0181131060 0 0.023755412 0 0.005615957
0 0.328463018 0.952593964
TCGA-93-8067-01A 0.024064283 0 0.104497029 0.057528205 0 0.109420143
0.003252189 0.062449037 0.016823558 0 0.022231668 0.043790145
0.002277874 0.188814018 0.073102356 0.171967225 0.073363614
0.003578151 0.042840504 0 0 0 0.01 0.281014404 0.968728017
TCGA-93-A4JN-01A 0.095374961 0 0.180471817 0.019996585 0
0.182566649 0 0 0.060660049 0 0 0.037126382 0.03074508
0.121604638 0.050164189 0.126759332 0 0.020480401 0.070617348 0
0 0.003432568 0 0.338558384 0.940890453
TCGA-93-A4JO-01A 0.021062097 0.00141888 0.080121619 0.133728693 0
0.187947282 0.000232983 0.014794786 0.059448293 0 0 0.010591646
0.007557817 0.156300932 0.09951035 0.169409376 0.01428451 0
0.043590735 0 0 0 0 0.538364593 0.848460854
TCGA-93-A4JP-01A 0.034256922 0 0.013530647 0.032027095 0 0.0616112380
0.031539824 0 0 0 0.016989407 0.050069949 0.136741891
0.088297435 0.303496192 0.021407027 0.073342088 0.130708229 0 0
0.005982055 0 0.364686126 0.93619221
TCGA-93-A4JQ-01A 0.045370793 0.134709135 0.019321914 0.047623852 0
0.237968765 0 0.068434892 0.062616001 0 0 0.021537297 0
0.172135501 0.092769951 0.0562681160.026872524 0.000769506
0.013601753 0 0 0 0 0.581832968 0.824521677
TCGA-95-7039-01A 0.050415354 0 0.017158546 0.15704461 0 0.100753893
0.088546077 0.036067087 0.02128589 0.001983844 0 0 0 0.181940903
0.088347322 0.17009771 0.015757871 0.01661953 0.053981364 0 0 0
0.04 0.194449065 1.008569917
TCGA-95-7043-01A 0.1115256130 0.180638405 0.261359128 0 0.00414091 0
0.050717644 0 0 0 0.004852212 0.013090132 0.0411973970.044212166

0.161205937 0 0.0118201160.1152403380 0 0 0.655 0.018265562
1.060262312
TCGA-95-7562-01A 0.0244032110 0.037085106 0.088659151 0 0.097940217
0.039610197 0 0.058657853 0 0.006699009 0 0.000527563
0.225457981 0.129616462 0.226230466 0.008858065 0.007080973
0.047077619 0 0 0.002096126 0 0.503066755 0.863718645
TCGA-95-7567-01A 0.006235909 0 0.183331001 0.030164375 0 0.129387094
0.033650934 0.024571992 0.057884095 0.035359718 0 0.0300146110
0.054746607 0.095879592 0.246150887 0.0287114480.0246096 0.017526934
0 0 0.001775203 0 0.31219554 0.953090929
TCGA-95-7944-01A 0.004237938 0 0.012489691 0.305305622 0 0
0.102019982 0.026697891 0.02166744 0 0.044609124 0.089723638 0
0.1171783220.140880864 0.087283131 0.006623577 0.010579542
0.030703238 0 0 0 0 0.499444927 0.866693639
TCGA-95-7947-01A 0.009527777 0 0.068378771 0 0 0.334256306 0
0.010541737 0.043224778 0.043990621 0.008697008 0 0 0.152147397
0.156101527 0.150608453 0.022525626 0 0 0 0 0 0 0.43731349
0.899434007
TCGA-95-7948-01A 0.004523599 0 0.134798961 0.1299596 0 0.131301599
0.016501972 0.027595941 0 0.005047938 0 0.02990322 0.066781061 0
0.02636853 0.187498372 0.108145932 0.064990144 0.066583131 0 0 0
0.175 0.098034755 1.030295555
TCGA-95-8039-01A 0.00756837 0.0190486 0 0.029613336 0 0.245380576
0.023461583 0.0234506 0.070171015 0 0 0.022097955 0.0351113650
0.045666463 0.1136990090.206354214 0.066901848 0.081972595 0 0
0.009502471 0 0.402353021 0.914692471
TCGA-95-8494-01A 0 0.025307251 0.0031127980.020729716 0 0.076579945
0.041693083 0.033010844 0.008940037 0 0 0.029309181 0.161413672
0.056796191 0 0.1197381020.203974144 0.123429735 0.082191974 0
0.0007115440.013061782 0.02 0.252051633 0.980924899
TCGA-95-A4VK-01A 0.109421682 0.038247424 0.064383063 0.146164578 0
0.174174616 0 0.036680132 0.066797012 0 0 0.027659356 0
0.1270117880.090349103 0.059208019 0 0.018638479 0.041264746 0 0

0 0.03 0.223510799 0.996073953
TCGA-95-A4VN-01A 0.022374423 0 0.0112478380.163672812 0 0.144177734
0.078877979 0.034613644 0.003204886 0 0 0.028371296 0.000768726
0.121014242 0.103563435 0.21971094 0.04353365 0.005457417 0.014917787
0 0 0.00449319 0 0.49540864 0.868845915
TCGA-95-A4VP-01A 0.156441076 0 0.05981728 0.133639952 0 0.1089711650
0.056042656 0.123838581 0 0.001090639 0.01819962 0.014382197
0.0834163 0.045936387 0.131832587 0.023631312 0.014854533
0.024598782 0 0 0.003306933 0.025 0.236907185 0.985898034
TCGA-97-7546-01A 0.052620185 0 0.075309187 0.08887977 0 0.326100764
0.010672926 0 0.006516088 0 0.006328398 0.004469261 0.032642049
0.026123895 0.042441924 0.144297784 0.051510864 0.025246902
0.099669731 0 0 0.007170272 0.005 0.292952992 0.960812768
TCGA-97-7547-01A 0.040181927 0 0.1196105780.056041276 0 0.204026309 0
0.02107762 0.074127357 0 0 0.00803208 0.030618289 0.117006613
0.048177071 0.184628322 0.024907821 0.0118523520.059543186 0 0
0.000169199 0.02 0.253943234 0.975529124
TCGA-97-7552-01A 0.019967235 0.047869924 0.108917486 0.245226777 0
0.102244029 0 0.013566259 0.037249473 0.01878936 0 0.03081727 0
0.13784058 0.106872533 0.044835257 0.0361164720 0.049687345 0 0 0
0 0.544163636 0.844646836
TCGA-97-7553-01A 0.049612026 0 0.012357439 0.08987785 0 0.19357253
0.017637733 0 0 0 0.029292371 0 0.012959174 0.196800293
0.055510979 0.183683282 0.068489447 0 0.05150053 0 0 0.038706345
0.005 0.306997753 0.960139814
TCGA-97-7554-01A 0.022485913 0 0.218123683 0.023530032 0 0.179803017
0.014794284 0.013422206 0.00092475 0 0.004049708 0.008582041 0
0.195651074 0.08456615 0.169362866 0.010749002 0 0.049945234 0 0
0.004010039 0 0.364101387 0.932051531
TCGA-97-7937-01A 0.029856618 0 0.066043796 0.030904128 0 0.050719459
0 0.026038933 0.043582639 0.015172499 0.017602919 0 0
0.335378413 0.069447568 0.169652899 0.041025797 0 0.041559445
0.061879629 0 0.0011352580.025 0.232972253 1.009924862

TCGA-97-7938-01A 0.088559749 0 0.0516721130.143763273 0 0.130991365 0.027250926 0.024935067 0.00212956 0 0 0.0119874740 0.132774511 0.050126286 0.1123702590.089483504 0.0115665850.076145019 0 0.02884043 0.017403877 0.12 0.1149649561.029146538

TCGA-97-7941-01A 0.02026342 0.050435023 0 0.025599849 0 0.123007147 0.003583815 0.029014981 0.077449998 0 0 0 0.005532257 0.063613171 0.052969533 0.278010531 0.079923522 0.01280311 0.174794694 0 0 0.002998947 0 0.374019869 0.927570088

TCGA-97-8171-01A 0.007701516 0.008129598 0.049624957 0.010663887 0 0.108794964 0 0.024590097 0.001574251 0 0 0.016683233 0 0.412971497 0.015779893 0.268084375 0.000952874 0.020860521 0.053588338 0 0 0 0.02 0.250792102 1.02259523

TCGA-97-8172-01A 0.002493078 0.136224527 0.092825693 0.07444553 0 0.218757516 0 0.002122323 0.022954013 0 0 0.034762021 0.016184026 0.072897964 0.029231713 0.133808615 0.040907041 0.005847387 0.1165385530 0 0 0 0.483708983 0.882073758

TCGA-97-8174-01A 0.036548199 0 0.1130607820.068915648 0 0.301462437 0.009015889 0 0.0110828680 0 0.009588245 0.016829233 0 0.109879073 0.20622407 0.01225053 0.0116619750.087720938 0 0 0.0057601150 0.389015644 0.920652192

TCGA-97-8175-01A 0.005382203 0.007919365 0 0.022020387 0 0.105230688 0 0 0.077812355 0 0 0.020365564 0.024226541 0.204836307 0.029169357 0.1106961040.330469651 0.020577349 0.021970905 0 0.002072782 0.017250443 0.005 0.300582902 0.972325907

TCGA-97-8176-01A 0.042021461 0 0.06244892 0.034968476 0 0.072057875 0 0 0.002122307 0 0.020703195 0.002574055 0.016327171 0.256323664 0.027077225 0.307629188 0.017184131 0 0.10348426 0 0 0.035078073 0 0.346692655 0.949209154

TCGA-97-8177-01A 0.020104833 0.0115420890.052756775 0.055726313 0 0.097247833 0 0.046803174 0.038096775 0 0 0.034120947 0.02514733 0.088021392 0.013520809 0.19361399 0.222759844 0.032339635 0.038026751 0.014035319 0 0.016136193 0 0.478368845 0.88005163

TCGA-97-8179-01A 0.0268801160 0.0091163380.079925258 0 0.081964436 0

0.070697233 0.033565921 0 0 0.044344219 0.034285172 0.129630452
0.0511155750.184465295 0.1176053850.01325843 0.074333257 0 0.042535754
0.006277159 0.065 0.157852407 1.018712097

TCGA-97-8547-01A 0.00846137 0 0 0.006408993 0 0.058800049 0
0.047739281 0.061277231 0 0 0 0 0.517147548 0 0.154164784
0.012412198 0.1075111750.025803832 0 0 0.000273538 0 0.513291087
0.867078892

TCGA-97-8552-01A 0 0.071883236 0.022141903 0.04204184 0 0.191532621 0
0.01936755 0.017601934 0 0.01671312 0.007231357 0.055389269
0.0311638840.027271426 0.2191871190.159154423 0.022769489 0.088837391
0 0 0.007713439 0 0.409905771 0.911803985

TCGA-97-A4LX-01A 0.074228547 0 0.058367369 0.126451207 0
0.133907153 0.018157959 0.031282643 0.048672481 0 0 0.027045937
0.014364948 0.073646149 0.145222362 0.130033259 0.049397632 0
0.067520342 0 0 0.0017020110 0.539646393 0.848342669

TCGA-97-A4M0-01A 0 0.022760534 0.00184678 0.1310999110 0.267836922
0.014968533 0.012500041 0.067235665 0 0 0.038164926 0.045313375
0 0.069373342 0.095704244 0.123134556 0.027683728 0.076312545 0
0 0.006064897 0 0.336049427 0.945954479

TCGA-97-A4M1-01A 0.003021331 0 0 0.061491971 0 0.12815172 0
0.008092548 0.003461372 0 0 0.056225529 0.084641082 0.095662767
0.023643049 0.30531932 0.037294091 0.0113988670.181596352 0 0 0 0
0.345736595 0.942957498

TCGA-97-A4M2-01A 0.009063851 0.013927584 0.076923039 0.094879614 0
0.085956959 0 0.0686556110.016653904 0 0 0.032858349 0.017792367
0.207163028 0.043727621 0.176648994 0.052961058 0.007973487
0.085490169 0 0 0.009324365 0 0.458241343 0.889115973

TCGA-97-A4M3-01A 0.009610283 0 0.0772119640.072262404 0 0.188376695
0 0.066724613 0.035916189 0 0 0.056324839 0.05871093 0.028214049
0.02902239 0.251471067 0.037515682 0.030629206 0.029109202
0.009535727 0.015229018 0.004135742 0.425 0.048629758 1.058380885

TCGA-97-A4M5-01A 0.012862782 0.01031157 0.022472958 0.058771714 0
0.172026195 0 0.005538975 0.01298901 0 0.058751095 0.004783232

0.009463651 0.166974419 0.056874897 0.274048544 0.002985643 0.017833441 0.104648935 0 0 0.00866294 0 0.383797773 0.92451823
TCGA-97-A4M6-01A 0.0113094650.02983197 0.006037919 0.058467373 0 0.223704078 0 0.030423222 0.076888235 0 0 0.02181292 0.005893919 0.081372784 0.105568857 0.128493828 0.109678024 0.02357012 0.07071942 0 0 0.016227867 0 0.386031953 0.922319784
TCGA-97-A4M7-01A 0.03903884 0 0.058102973 0.053068496 0 0.192092618 0 0.054788565 0.072249249 0 0 0.009074504 0.035590612 0.023809526 0.0513554 0.106181065 0.174532568 0.035794933 0.076696186 0 0 0.017624464 0 0.437924792 0.900221735
TCGA-99-7458-01A 0 0.016736037 0.00440712 0.1195733750 0.23658061 0.001087812 0.050196752 0.051481793 0 0 0.0365355110.004827011 0.1129710540.066264717 0.08805938 0.1150604440.024755256 0.07146313 0 0 0 0 0.361853532 0.9341897
TCGA-99-8025-01A 0.050166087 0 0.149389398 0.029803046 0 0.138964749 0 0 0.064349689 0 0 0.0119507490 0.265399497 0.078555782 0.165038705 0 0 0.043522196 0 0 0.0028601 0.005 0.299856507 0.963702117
TCGA-99-8028-01A 0.012543895 0 0.087523588 0.139236662 0 0.131055073 0.0759011210 0.047484136 0.028943367 0 0.010767772 0 0.165719614 0.097102302 0.145791852 0.012955951 0 0.04050351 0 0 0.0044711570 0.557138267 0.840212729
TCGA-99-8032-01A 0.018789572 0 0.339383253 0.061287071 0 0.1163570810 0 0.102523656 0 0 0.033752875 0.000794179 0.151036051 0.018481291 0.092222956 0.000699779 0.01958597 0.038896165 0 0 0.006190101 0 0.390221637 0.921615829
TCGA-99-8033-01A 0 0 0.084469833 0.197438972 0 0 0.15435585 0.074731287 0.025356627 0 0.015790484 0.067407928 0.002620181 0.141505877 0.084335493 0.06087117 0.007757333 0.057450058 0.005684067 0 0 0.020224838 0 0.353812276 0.939650646
TCGA-99-AA5R-01A 0.006052619 0.1117859170.045381741 0.131373534 0 0.280219618 0 0.007764829 0 0 0 0.05940825 0.007984587 0.138781258 0.049527341 0.099504735 0.026657725 0 0.03487763 0 0

0.000680217 0 0.434762047 0.899836254

TCGA-J2-8192-01A 0.0118182840 0 0.045766133 0 0.218643924 0.00444017 0 0.017251812 0 0.002366812 0 0.0119694920.266400133 0.035025445 0.190307919 0.088873478 0.0183674110.084698546 0 0 0.004070441 0 0.514854555 0.857603585

TCGA-J2-8194-01A 0.0161199820 0.043553365 0.023169763 0 0.20562458 0.045520092 0 0 0 0 0.02441545 0.056929044 0.047461703 0.087027501 0.173690151 0.127092412 0 0.07878471 0 0 0.070611248 0.07 0.15424925 1.017240765

TCGA-J2-A4AD-01A 0 0 0.11315966 0.042537552 0 0.199428199 0 0.008035642 0.01830736 0 0 0.034997733 0.025789247 0.168831368 0 0.203508204 0.003796991 0.089926019 0.090669294 0 0.00101273 0 0.05 0.169207693 1.013736394

TCGA-J2-A4AE-01A 0.082325749 0 0.183521685 0.0791194770 0.266443764 8.4326E-05 0.028422252 0 0 0 0.00711458 0 0.031337821 0.075014015 0.129894345 0.052305198 0.024100032 0.033719184 0 0 0.006597572 0.04 0.190709099 0.999559624

TCGA-J2-A4AG-01A 0.029460573 0.1011533260.030552079 0.067643631 0 0.2116463180.056038156 0 0.045713429 0 0 0.013850047 0.023589685 0.059349444 0.033082748 0.099764923 0.1196241070.057693184 0.04103558 0 0 0.00980277 0.015 0.269914881 0.968465009

TCGA-L4-A4E5-01A 0.002772909 0 0.1970474110.032831095 0 0.086759716 0.001638089 0 0.071397672 0.014738935 0 0.07292585 0 0.24637544 0.075428415 0.150071641 0.016409967 0 0.03160286 0 0 0 0.015 0.276465976 0.973579418

TCGA-L4-A4E6-01A 0.013461792 0 0.007133785 0.025534998 0 0.1463611970.007716235 0.010753647 0 0 0.015918787 0.006899433 0.016299684 0.222757526 0.044371276 0.327094006 0.082907296 0 0.068467786 0 0 0.004322553 0.02 0.2542281130.99722301

TCGA-L9-A443-01A 0.017770419 0 0.047933225 0.036140094 0 0.305277131 0 0.03720374 0 0 0.034975468 0 0.020125821 0.145388669 0.1621180910.1419385 0.0412559110 0 0.009872931 0 0 0.01 0.283153626 0.977694994

TCGA-L9-A444-01A 0.019653937 0 0.1222126 0.255173587 0 0.114059347 0.06353608 0.008700465 0.056642696 0 0.051429127 0 0.005528859 0.06715399 0.1108910810.081913055 0.009512037 0 0.03359314 0 0 0 0 0.51917351 0.856742999

TCGA-L9-A50W-01A 0.033415944 0 0.130706345 0.022089854 0 0.22173799 0 0.003539351 0.0367112640 0 0.047994631 0.009684413 0.041061805 0.070236012 0.049120287 0.210626758 0 0.1192637130 0 0.003811633 0.01 0.282925663 0.962631086

TCGA-L9-A5IP-01A0 0 0.209784321 0.170067983 0 0 0.105410107 0.061029532 0.053781376 0 0 0.053163709 3.89667E-050.16133156 0.034982396 0.023153452 0.001812829 0.008162805 0.055749202 0 0.017695958 0.043835802 0.095 0.131326589 1.022738324

TCGA-L9-A743-01A 0.036185155 0 0.090718688 0.083352608 0 0.20143454 0.068913439 0 0.023102122 0 0.00461803 0 0.01339819 0.079360005 0.052462507 0.145509332 0.062872884 0.09034705 0.036497642 0 0 0.0112278090 0.428824087 0.903714631

TCGA-L9-A7SV-01A 0.085334265 0 0.081543145 0.1183736460 0.222068227 0.017159058 0.090948818 0 0 0 0.014620816 0.008574439 0.020863291 0.094629842 0.181647753 0.027920856 0 0.022447469 0 0.013868373 0 0.425 0.048662257 1.063686

TCGA-L9-A8F4-01A 0.020875349 0 0.1105916220.152307618 0 0.086751271 0.082384824 0.013746445 0.0671123160 0 0.039966671 0 0.170890378 0.07815427 0.13639655 0.008618027 0 0.032204659 0 0 0 0 0.555226476 0.842613912

TCGA-MN-A4N1-01A 0.008831082 0 0.047857343 0.019557507 0 0.051221359 0 0.019348447 0.093778832 0 0.0113181630 0.020565078 0.500874645 0.006976468 0.156639934 0.007783327 0.050823849 0.004423968 0 0 0 0 0.3114324130.996292008

TCGA-MN-A4N4-01A 0.00402142 0 0.097855535 0.042996595 0 0.061997968 0.027400363 0 0.01478401 0 0 0.0115719490 0.527128985 0.037358661 0.103827659 0 0 0.0700121150 0 0.00104474 0 0.674206732 0.742563693

TCGA-MN-A4N5-01A 0.014999674 0 0.02399527 0.056692602 0 0.237995132

0.002185082 0.080190745 0.006786459 0 0 0.1174128690 0.133076664
0.135722601 0.132673745 0 0 0.058269157 0 0 0 0.02
0.2611421690.990654449
TCGA-MP-A4SV-01A 0.021309912 0 0.097249607 0.029457203 0
0.303835536 0.03278684 0.010904087 0.010629727 0.007884721 0
0.034464024 0 0.1178214070.108573158 0.132457161 0.019741824
0.019629082 0.053255709 0 0 0 0 0.455669922 0.890034565
TCGA-MP-A4SW-01A 0.015103748 0.035572347 0.149739658 0.053429386 0
0.120799044 0 0.028458377 0.073758626 0 0 0.02535862 0.016831593
0.17431022 0.028784728 0.197648478 0.024242853 0.013641583
0.040509873 0 0 0.001810866 0 0.424667354 0.905661421
TCGA-MP-A4SY-01A 0.025938921 0 0.021486758 0.007697876 0
0.098708293 0 0.006433897 0.033031481 0 0 0.016564132 0
0.389925777 0.0451142 0.175745878 0.1146671210 0.060388934 0 0
0.004296734 0 0.395099323 0.931670525
TCGA-MP-A4T4-01A 0.008563899 0 0.125296368 0.1139014540 0.137868795
0.015577058 0.036269688 0.022833027 0.009238869 0 0.046769831 0
0.138127715 0.121360995 0.140277695 0.009000836 0 0.071913768 0
0 0.003000003 0 0.581267709 0.827922246
TCGA-MP-A4T6-01A 0.029795178 0 0.016251386 0.0521148110 0.132670878
0 0.04308805 0.13042972 0.009188688 0 0 0 0.362558644 0.080398195
0.101893289 0.005989643 0.013491428 0.021679608 0 0 0.000450483
0 0.403932091 0.920403162
TCGA-MP-A4T7-01A 0.009683255 0.005018258 0.022035455 0.057691824 0
0.164546085 0 0.079581606 0.0195621130 0.003370328 0.03103244
0.008416884 0.140758233 0.013460857 0.216837255 0 0.171334646
0.038891221 0 0 0.017779541 0.005 0.29759723 0.965480128
TCGA-MP-A4T8-01A 0.030596153 0 0.068577046 0.012800952 0 0 0
0.030025164 0.1152746690.0091183130 0.027027553 0 0.409022389 0
0.079479447 0 0.168412947 0.049665367 0 0 0 0 0.354750005
0.951386918
TCGA-MP-A4T9-01A 0.021761259 0 0.008452547 0.091894932 0
0.173989038 0.000633324 0.015460973 0.00097944 0 0 0.021934925

0.020224673 0.1664068 0.076632692 0.249509432 0.042164356
0.027324666 0.065929579 0 0.001536757 0.015164608 0 0.3356555
0.948364527
TCGA-MP-A4TA-01A 0.031436157 0 0.099783047 0.140388868 0
0.134808492 0.0145118040.067714262 0.052828747 0 0.031214735
0.014745106 0.0104119820.142826018 0.088334312 0.1133770240.000846483
0.0321193420.02465362 0 0 0 0 0.341274422 0.942806076
TCGA-MP-A4TC-01A 0.002641654 0 0.026303137 0.080372985 0
0.061526572 0.053223206 0.018303956 0.044244935 0 0.01903315
0.007326143 0 0.340834404 0.098242336 0.172959195 0.03261339 0 0
0.042374938 0 0 0 0.55740653 0.830048798
TCGA-MP-A4TD-01A 0.024927881 0.070229849 0.204526331 0.04564936 0
0.120406945 0 0.022053717 0.055536474 0 0 0.020904915 0
0.25242425 0.013776858 0.1189726910.006248077 0.008975996 0.035366657
0 0 0 0 0.475355071 0.883092842
TCGA-MP-A4TE-01A 0 0 0.413758769 0.037361794 0 0.0469323 0
0.022493327 0.039357922 0.007203383 0 0 0 0.2034211750
0.099184031 0 0 0 0.061910971 0 0.068376328 0.175 0.098549766
1.042597268
TCGA-MP-A4TF-01A 0 0 0.248670628 0.090478364 0 0.070961266
0.047961938 0.013692809 0 0 0.07523173 0.003476051 0 0.205449917
0.06415919 0.077739781 0 0.0792926110 0.00781068 0 0.015075036 0.02
0.259079912 0.977379361
TCGA-MP-A4TH-01A 0.018684539 0.1509428 0.167515247 0.1128239180
0.147186012 0 0.03900704 0.064395325 0 0 0.010887122 0.005835544
0.066944909 0.028649203 0.096310851 0.047199724 0.018035387
0.025582379 0 0 0 0 0.393921725 0.919237457
TCGA-MP-A4TI-01A 0.014080799 0 0.001292157 0.087157571 0
0.085010052 0.061509063 0.021357633 0.079882644 0.0115593060
0.028164478 0.004926428 0.215038531 0.124235038 0.207767737
0.014483517 0.02156089 0.021974155 0 0 0 0 0.670385789
0.763901624
TCGA-MP-A4TJ-01A 0.029010084 0.008003944 0.089180727 0.121345199 0

0.176388739 0.1169590980.040905905 0.03515564 0 0.03113891 0.002851237
0.002136395 0.131021992 0.082232598 0.07991292 0.036861384 0
0.0124064 0 0 0.004488827 0 0.471269547 0.8833888
TCGA-MP-A4TK-01A 0.0116043880 0.161379876 0.0306235110 0.141155335
0.0132611850 0.0306954110.007543456 0.007949347 0 0 0.316391571
0.070088035 0.179324649 0.022190997 0 0.004957623 0 0
0.002834616 0 0.643716535 0.782579635
TCGA-MP-A5C7-01A 0 0 0.089859519 0.015953956 0 0.286569561 0
0.051935367 0.001904902 0 0.016242863 0.052844493 0.011396394
0.02150071 0.067299626 0.197319284 0.087725727 0.039728503
0.059545607 0 0.000173489 0 0.225 0.085301244 1.044487175
TCGA-NJ-A4YF-01A 0.00311313 0 0.105980824 0.046000784 0 0.145148653
0 0.016380955 0.043621908 0 0.014220489 0.003645363 0
0.387198765 0.059436548 0.121832697 0.0115364740 0 0.03533527 0
0.00654814 0 0.395285571 0.926142294
TCGA-NJ-A4YG-01A 0 0.008283125 0.046717271 0.038133367 0
0.167749045 0.013683807 0.044930202 0.034498494 0 0 0.031019332
0.010519772 0.095701909 0.036368974 0.175597826 0.173348851
0.026360013 0.077768444 0 0 0.019319568 0 0.3501157010.937313565
TCGA-NJ-A4YI-01A 0 0.003282853 0.027560068 0.02176071 0 0.082097057
0 0.030846865 0.027587741 0 0.013775422 0.006639181 0.127736612
0.145065832 0.017480801 0.276109633 0.145564694 0.0031170720.0446435
0 0.012298325 0.014433634 0.02 0.249579079 0.991778252
TCGA-NJ-A4YP-01A 0 0 0.109795253 0.1114737760 0.100612398
0.017516842 0.03470832 0.041010872 0 0 0.070150178 0 0.200126368
0.07797663 0.1582521 0.006061531 0.044309064 0 0.012962486 0
0.01504418 0.005 0.2997521180.964790506
TCGA-NJ-A4YQ-01A 0.018461845 0.034714895 0.131051629 0.147327494 0
0.027515045 0.017079477 0.022170103 0.052731354 0.050138808 0
0.0411467610 0.232327413 0.083443531 0.087987445 0.000879634 0
0.053024565 0 0 0 0 0.598200483 0.8183902
TCGA-NJ-A55A-01A 0.029929998 0.090575094 0.057810313 0.073640235 0
0.180204646 0.01351971 0.01515021 0.052525865 0.051302988 0

0.002870359 0 0.04412951 0.076058685 0.091666893 0.073164179 0 0.1449112940 0 0.002540021 0 0.383078409 0.922979721
TCGA-NJ-A55O-01A 0.001051793 0.018383382 0 0.033955485 0 0.207063956 0 0.027257625 0.034921493 0 0 0.020750825 0.001330313 0.095433561 0.088867253 0.351957825 0.025877491 0.007698091 0.077908451 0 0 0.007542456 0 0.355429176 0.939376188
TCGA-NJ-A55R-01A 0.045429031 0.066025442 0.308385981 0.021609572 0 0.062085592 0.003900685 0.002978466 0.066798705 0 0 0.025053695 0 0.244762637 0.01044518 0.099032483 0 0 0.04349253 0 0 0 0 0.313164699 0.954073431
TCGA-NJ-A7XG-01A 0 0.031063327 0.061417482 0.037883953 0 0.261822779 0 0.016422726 0.057846017 0 0 0.076883287 0.008777849 0 0.014286553 0.104381953 0.1089309 0.089945765 0.129968106 0 0 0.000369303 0.245 0.078455971 1.047042056
TCGA-O1-A52J-01A 0.00057989 0 0 0.031033894 0 0.14121864 0 0 0.04640316 0 0 0.0304118670.173304487 0.056564329 0.009037241 0.3118724370.084130896 0.04376836 0.062158717 0 0 0.009516082 0.025 0.225602482 0.998554045
TCGA-S2-AA1A-01A 0.055552407 0.018466875 0.22407353 0.066342251 0 0.214043729 0.018419126 0.012064701 0.069369761 0 0 0.015351483 0.0044298 0.105072993 0.034763897 0.055929363 0.055619273 0 0.050500812 0 0 0 0 0.508362309 0.873676314

Table S6. TCGA training set risk model construction

gene coefp.value Hazard_Ratio lower_.95 upper_.95 logrank_pvalue wald_pvalue Likelihood_pvalue HR
SPN -0.295293881 0.027157505 0.744312816 0.572771744 0.967229221 0.026645399 0.027157505 0.025539969 0.74(0.57-0.97)
RASSF2 -0.285294369 0.019932452 0.751792917 0.591244424 0.955937288 0.019503919 0.019932452 0.020860721 0.75(0.59-0.96)
CD84 -0.273599966 0.041022648 0.760636295 0.585059913 0.98890312

0.040396183 0.041022648 0.040139138 0.76(0.59-0.99)

CIITA -0.277364737 0.028435141 0.757778057 0.591285721 0.971150771

0.027873015 0.028435141 0.028380074 0.76(0.59-0.97)

CD5 -0.321317491 0.014122771 0.725192972 0.561055945 0.93734832

0.013859983 0.014122771 0.0116321270.73(0.56-0.94)

SASH3 -0.219632689 0.044361322 0.802813626 0.648088782 0.99447751

0.043944425 0.044361322 0.045556616 0.80(0.65-0.99)

HLA-DOA -0.197023282 0.022440657 0.8211715150.693376983 0.972519529

0.022046314 0.022440657 0.024032957 0.82(0.69-0.97)

ADA2 -0.24824743 0.018435554 0.780166883 0.634638731 0.959065898

0.018140613 0.018435554 0.019488626 0.78(0.63-0.96)

CHRDL1 -0.234461349 0.009861453 0.790996806 0.66197258 0.945168979

0.009597265 0.009861453 0.00735569 0.79(0.66-0.95)

CD2 -0.204489086 0.038423792 0.815063638 0.671608619 0.989160524

0.037902984 0.038423792 0.036835697 0.82(0.67-0.99)

FMO2 -0.21304167 0.042969023 0.808122462 0.657479341 0.993281268

0.042422726 0.042969023 0.040049766 0.81(0.66-0.99)

MFAP4 -0.173144003 0.017857993 0.841016497 0.728753393 0.970573522

0.017448744 0.017857993 0.017758186 0.84(0.73-0.97)

HLA-DPA1 -0.169404962 0.025949701 0.844166979 0.727239605 0.979894224

0.025439522 0.025949701 0.030154967 0.84(0.73-0.98)

HLA-DPB1 -0.165735964 0.037553164 0.847269914 0.724748933 0.990503434

0.037332014 0.037553164 0.043988569 0.85(0.72-0.99)

F13A1 -0.188299985 0.019953823 0.828366173 0.706886689 0.970722078

0.019574398 0.019953823 0.0191115710.83(0.71-0.97)

CD74 -0.160926889 0.032701595 0.851354312 0.734467171 0.986843516

0.032394909 0.032701595 0.039105359 0.85(0.73-0.99)

CPA3 -0.160489035 0.02163393 0.851727163 0.742713022 0.976742211

0.0211239680.02163393 0.021307148 0.85(0.74-0.98)

HLA-DRA -0.167484799 0.0301176510.845789474 0.726976968 0.984019943

0.029927355 0.0301176510.035032187 0.85(0.73-0.98)

CHIT1 -0.155680686 0.040268725 0.855832426 0.737526965 0.993115067

0.039564917 0.040268725 0.036485127 0.86(0.74-0.99)

HLA-DRB5 -0.146098656 0.021067658 0.864072453 0.763202061 0.978274617 0.021005446 0.021067658 0.023561634 0.86(0.76-0.98)

ATP13A4 -0.171848494 0.039402478 0.842106748 0.715084121 0.991692801 0.038681008 0.039402478 0.036037538 0.84(0.72-0.99)

HLA-DQB1 -0.14940593 0.038229904 0.861219449 0.74773453 0.991928164 0.038015703 0.038229904 0.043140671 0.86(0.75-0.99)

CX3CL1 -0.185977751 0.0115284820.830292068 0.718732421 0.959167693 0.0110663690.0115284820.010464295 0.83(0.72-0.96)

FDCSP -0.129849834 0.049842131 0.878227301 0.771352251 0.99991047 0.048799443 0.049842131 0.044068447 0.88(0.77-1.00)

HLA-DQB2 -0.157669694 0.014607021 0.85413186 0.752602962 0.969357379 0.014398363 0.014607021 0.014687294 0.85(0.75-0.97)

PIGR -0.079331878 0.045991062 0.923733306 0.854488725 0.998589211 0.045187141 0.045991062 0.047202906 0.92(0.85-1.00)

SLC34A2 -0.112297585 0.024595787 0.893778243 0.810406039 0.985727536 0.024131381 0.024595787 0.031863556 0.89(0.81-0.99)

S100P 0.078389282 0.042823957 1.081543603 1.002536647 1.166776863 0.041992896 0.042823957 0.041734907 1.08(1.00-1.17)

SUSD2 -0.121488044 0.040336284 0.885601642 0.788499121 0.994662196 0.039747425 0.040336284 0.03756259 0.89(0.79-0.99)

CYP4B1 -0.124695416 0.016393403 0.882765738 0.797298171 0.977395128 0.015803373 0.016393403 0.0151437 0.88(0.80-0.98)

CPS1 0.135497123 0.00045257 1.145105901 1.06160237 1.235177654 0.000324025 0.00045257 0.001096213 1.15(1.06-1.24)

FGA 0.085753231 0.014736744 1.089537431 1.016978961 1.167272737 0.014050712 0.014736744 0.0195116871.09(1.02-1.17)

Table S7. Validation results of TCGA and GEO datasets

sample Score Group

TCGA-05-4250-01A -0.68292281 High

TCGA-05-4382-01A -0.8001949 Low

TCGA-05-4384-01A -0.783432862 Low

TCGA-05-4390-01A -0.087887778 High

TCGA-05-4397-01A	-0.682381521	High
TCGA-05-4402-01A	-0.979772708	Low
TCGA-05-4403-01A	-0.803197223	Low
TCGA-05-4405-01A	-0.799513097	Low
TCGA-05-4417-01A	-0.292053418	High
TCGA-05-4422-01A	-0.40584261	High
TCGA-05-4426-01A	-0.823594277	Low
TCGA-05-4430-01A	-0.935901902	Low
TCGA-05-4432-01A	-0.765863149	High
TCGA-05-4434-01A	-1.139203806	Low
TCGA-05-5420-01A	-0.291799732	High
TCGA-05-5429-01A	-0.424025985	High
TCGA-35-4122-01A	-0.62923141	High
TCGA-35-4123-01A	-0.566834214	High
TCGA-35-5375-01A	-0.562030189	High
TCGA-38-4626-01A	-1.198601791	Low
TCGA-38-4628-01A	-0.537352672	High
TCGA-38-4631-01A	-0.249758351	High
TCGA-38-7271-01A	-1.215862977	Low
TCGA-38-A44F-01A	-1.17158674	Low
TCGA-44-2655-01A	-0.913460004	Low
TCGA-44-2657-01A	-1.480800489	Low
TCGA-44-2659-01A	-0.884258027	Low
TCGA-44-3917-01A	-0.376416825	High
TCGA-44-4112-01A	-0.661655716	High
TCGA-44-5643-01A	-0.80394571	Low
TCGA-44-5644-01A	-0.196378991	High
TCGA-44-5645-01A	-1.185473587	Low
TCGA-44-6146-01A	-0.508456283	High
TCGA-44-6147-01A	-1.189267506	Low
TCGA-44-6774-01A	-0.70038907	High
TCGA-44-6776-01A	-0.831372843	Low
TCGA-44-6777-01A	-0.986299415	Low

TCGA-44-6778-01A	-1.100955393	Low
TCGA-44-6779-01A	-0.707667846	High
TCGA-44-7659-01A	-1.046492661	Low
TCGA-44-7661-01A	-0.888201414	Low
TCGA-44-7670-01A	-0.719319008	High
TCGA-44-7672-01A	-0.987166436	Low
TCGA-44-8117-01A	-0.687405337	High
TCGA-44-A479-01A	-0.790842352	Low
TCGA-44-A47B-01A	-0.652080893	High
TCGA-44-A4SS-01A	-0.708041982	High
TCGA-44-A4SU-01A	-1.001142721	Low
TCGA-49-4487-01A	-0.29249732	High
TCGA-49-4507-01A	0.182314577	High
TCGA-49-4510-01A	-0.385992504	High
TCGA-49-4514-01A	-0.181952446	High
TCGA-49-6742-01A	-0.094924403	High
TCGA-49-6743-01A	-0.759304249	High
TCGA-49-6767-01A	-0.529058328	High
TCGA-49-AAQV-01A	-0.657173715	High
TCGA-49-AAR0-01A	-1.114161764	Low
TCGA-49-AAR2-01A	0.068592864	High
TCGA-49-AAR3-01A	-0.651401112	High
TCGA-49-AAR9-01A	-0.236487238	High
TCGA-49-AARN-01A	-0.813363027	Low
TCGA-49-AARO-01A	-0.967964929	Low
TCGA-50-5044-01A	-0.708910055	High
TCGA-50-5045-01A	-1.086656898	Low
TCGA-50-5055-01A	-1.147030538	Low
TCGA-50-5931-01A	-0.810632983	Low
TCGA-50-5932-01A	-0.777803448	High
TCGA-50-5933-01A	-0.559099539	High
TCGA-50-5935-01A	-0.925756315	Low
TCGA-50-5936-01A	0.142236827	High

TCGA-50-5941-01A	-1.093991588	Low
TCGA-50-6591-01A	-0.262641502	High
TCGA-50-6593-01A	-0.888104855	Low
TCGA-50-6594-01A	-0.575284186	High
TCGA-50-6595-01A	-0.481067192	High
TCGA-50-6597-01A	-0.71788682	High
TCGA-50-6673-01A	-1.059441941	Low
TCGA-50-7109-01A	-0.088584808	High
TCGA-50-8457-01A	-1.30974037	Low
TCGA-50-8460-01A	-1.117349571	Low
TCGA-53-7626-01A	-1.177371251	Low
TCGA-53-7813-01A	-0.70098984	High
TCGA-53-A4EZ-01A	-0.521853136	High
TCGA-55-6642-01A	-0.372624361	High
TCGA-55-6968-01A	-0.741513194	High
TCGA-55-6972-01A	-0.617742721	High
TCGA-55-6975-01A	0.015123987	High
TCGA-55-6978-01A	-0.82847041	Low
TCGA-55-6982-01A	-0.842002749	Low
TCGA-55-6986-01A	-0.934521532	Low
TCGA-55-7227-01A	-0.921048969	Low
TCGA-55-7283-01A	-0.968043474	Low
TCGA-55-7576-01A	-0.691942462	High
TCGA-55-7725-01A	-1.110157228	Low
TCGA-55-7728-01A	-1.008624848	Low
TCGA-55-7907-01A	-0.758859434	High
TCGA-55-7910-01A	-0.07619433	High
TCGA-55-7914-01A	-0.809781235	Low
TCGA-55-7994-01A	-0.804958332	Low
TCGA-55-7995-01A	-0.805612251	Low
TCGA-55-8092-01A	-0.180016675	High
TCGA-55-8097-01A	-1.241118561	Low
TCGA-55-8203-01A	-0.852981845	Low

TCGA-55-8208-01A	-1.114381225	Low
TCGA-55-8299-01A	-0.54775604	High
TCGA-55-8301-01A	-1.014652985	Low
TCGA-55-8302-01A	-0.759637566	High
TCGA-55-8505-01A	-0.029244373	High
TCGA-55-8510-01A	-1.09924454	Low
TCGA-55-8513-01A	-1.301581646	Low
TCGA-55-8614-01A	-0.633479294	High
TCGA-55-8615-01A	-0.157812413	High
TCGA-55-8616-01A	-0.858778721	Low
TCGA-55-8619-01A	-1.284218956	Low
TCGA-55-8621-01A	-1.243304014	Low
TCGA-55-A48Y-01A	0.255442589	High
TCGA-55-A48Z-01A	-0.62016975	High
TCGA-55-A490-01A	-0.551245496	High
TCGA-55-A491-01A	-0.744259079	High
TCGA-55-A4DF-01A	-0.898753705	Low
TCGA-55-A4DG-01A	-1.181339643	Low
TCGA-62-8395-01A	-1.03645244	Low
TCGA-62-8397-01A	-0.923677925	Low
TCGA-62-8398-01A	0.023884396	High
TCGA-62-8399-01A	-0.375387874	High
TCGA-62-8402-01A	-0.607501505	High
TCGA-62-A46R-01A	-0.784444857	Low
TCGA-62-A46Y-01A	-0.849962337	Low
TCGA-62-A471-01A	0.362498809	High
TCGA-62-A472-01A	-0.663724915	High
TCGA-64-1676-01A	-0.403220029	High
TCGA-64-1680-01A	-0.677478405	High
TCGA-64-1681-01A	-1.168963143	Low
TCGA-64-5774-01A	-0.118253945	High
TCGA-64-5775-01A	-0.432252255	High
TCGA-64-5778-01A	-0.8323966	Low

TCGA-64-5779-01A	-0.634986132	High
TCGA-64-5781-01A	0.011135602	High
TCGA-67-3770-01A	-1.231562173	Low
TCGA-67-3771-01A	-1.001364205	Low
TCGA-67-3772-01A	-1.054051034	Low
TCGA-67-3773-01A	-0.871176406	Low
TCGA-67-3774-01A	-0.816746835	Low
TCGA-67-6216-01A	-0.932076038	Low
TCGA-69-7761-01A	-0.893386251	Low
TCGA-69-7763-01A	-0.832116133	Low
TCGA-69-7765-01A	-0.730494058	High
TCGA-69-7973-01A	-0.518963455	High
TCGA-69-7974-01A	-0.99629816	Low
TCGA-69-7980-01A	-0.939688747	Low
TCGA-69-8253-01A	-0.917090222	Low
TCGA-69-8255-01A	0.119978957	High
TCGA-69-8453-01A	-1.239676006	Low
TCGA-69-A59K-01A	-0.88093105	Low
TCGA-71-6725-01A	-0.521886576	High
TCGA-71-8520-01A	-0.946185877	Low
TCGA-73-4659-01A	-0.386251732	High
TCGA-73-4662-01A	-0.978507103	Low
TCGA-73-4668-01A	-0.492086887	High
TCGA-73-4670-01A	0.211447728	High
TCGA-73-4675-01A	-0.746927837	High
TCGA-73-A9RS-01A	0.117573796	High
TCGA-75-5125-01A	-0.693644904	High
TCGA-75-5146-01A	-1.063258017	Low
TCGA-75-7025-01A	-1.149916757	Low
TCGA-75-7027-01A	0.191928601	High
TCGA-78-7143-01A	-1.063783244	Low
TCGA-78-7146-01A	-0.534507025	High
TCGA-78-7148-01A	0.060903957	High

TCGA-78-7149-01A	-1.02236822	Low
TCGA-78-7150-01A	0.05151142	High
TCGA-78-7152-01A	-0.908100771	Low
TCGA-78-7154-01A	-0.707813545	High
TCGA-78-7155-01A	-0.401552817	High
TCGA-78-7156-01A	-0.613896009	High
TCGA-78-7167-01A	-0.451883761	High
TCGA-78-7537-01A	-1.100215561	Low
TCGA-78-7539-01A	-0.879174485	Low
TCGA-78-8640-01A	-0.772429737	High
TCGA-78-8648-01A	-1.159752758	Low
TCGA-83-5908-01A	-0.489376116	High
TCGA-86-6562-01A	-0.953450393	Low
TCGA-86-7713-01A	-0.278358732	High
TCGA-86-7955-01A	0.518751509	High
TCGA-86-8055-01A	-0.818418489	Low
TCGA-86-8074-01A	-0.712698823	High
TCGA-86-8075-01A	-0.782281748	Low
TCGA-86-8279-01A	-0.452889669	High
TCGA-86-8280-01A	-1.19073143	Low
TCGA-86-8358-01A	-0.718467689	High
TCGA-86-8359-01A	0.112559478	High
TCGA-86-8669-01A	-0.936976695	Low
TCGA-86-8672-01A	-0.305625222	High
TCGA-86-8674-01A	-0.716222269	High
TCGA-86-A4D0-01A	0.229887302	High
TCGA-86-A4P7-01A	-0.98831809	Low
TCGA-91-6847-01A	-0.126582935	High
TCGA-91-6848-01A	-0.810632819	Low
TCGA-93-7348-01A	-0.948513117	Low
TCGA-93-A4JO-01A	-1.075202262	Low
TCGA-93-A4JP-01A	-1.169310105	Low
TCGA-93-A4JQ-01A	-0.893476585	Low

TCGA-95-7043-01A	-0.613048464	High
TCGA-95-7944-01A	-0.308467582	High
TCGA-95-7947-01A	-0.863067634	Low
TCGA-95-7948-01A	-0.548986822	High
TCGA-95-8039-01A	-0.990236553	Low
TCGA-95-A4VK-01A	-0.714203751	High
TCGA-97-7546-01A	-1.266025419	Low
TCGA-97-7547-01A	-1.0966029	Low
TCGA-97-7553-01A	-1.294499271	Low
TCGA-97-7554-01A	-0.935529447	Low
TCGA-97-7937-01A	-0.523657537	High
TCGA-97-7938-01A	-1.011140787	Low
TCGA-97-8172-01A	-1.15139049	Low
TCGA-97-8175-01A	-0.552262907	High
TCGA-97-8179-01A	-0.128705849	High
TCGA-97-8552-01A	-1.228726789	Low
TCGA-97-A4M1-01A	-1.075376738	Low
TCGA-97-A4M5-01A	-0.594444355	High
TCGA-99-7458-01A	-1.160555507	Low
TCGA-99-8032-01A	-0.135222507	High
TCGA-99-8033-01A	0.37872866	High
TCGA-99-AA5R-01A	-1.321431107	Low
TCGA-J2-A4AD-01A	-0.778564491	High
TCGA-L9-A444-01A	-0.986871798	Low
TCGA-L9-A5IP-01A	0.111254747	High
TCGA-L9-A7SV-01A	-0.706418536	High
TCGA-L9-A8F4-01A	-0.856305485	Low
TCGA-MN-A4N4-01A	-0.561364405	High
TCGA-MN-A4N5-01A	-0.714744482	High
TCGA-MP-A4SV-01A	-0.881201752	Low
TCGA-MP-A4SW-01A	-0.844541007	Low
TCGA-MP-A4T4-01A	-0.986387521	Low
TCGA-MP-A4T6-01A	-0.902992127	Low

TCGA-MP-A4T8-01A	0.135407172	High
TCGA-MP-A4TC-01A	-0.484418915	High
TCGA-MP-A4TD-01A	-0.427052869	High
TCGA-MP-A4TE-01A	0.219379873	High
TCGA-MP-A4TH-01A	-1.095973415	Low
TCGA-MP-A4TJ-01A	-1.150649519	Low
TCGA-MP-A4TK-01A	-0.834599574	Low
TCGA-NJ-A4YF-01A	0.522087633	High
TCGA-NJ-A4YP-01A	-0.16153514	High
TCGA-NJ-A55A-01A	-1.35950745	Low
TCGA-NJ-A55R-01A	-0.85646083	Low
TCGA-O1-A52J-01A	-1.093013542	Low
TCGA-S2-AA1A-01A	-1.179582062	Low

Table.S8. Univariate and multivariate cox validation of riskscore as independent prognostic factor

sample	riskscore	riskgroup	status	time	age	gender	smoking_history	eml4_Alk	egfr_status	stage
TCGA-05-4249-01A	-0.820647704	Low	0	1523	>=60	MALE	3			Stage I&II
TCGA-05-4250-01A	-0.68292281	High	1	121	>=60	FEMALE	4			Stage III&IV
TCGA-05-4382-01A	-0.8001949	Low	0	607	>=60	MALE	4			Stage I&II
TCGA-05-4384-01A	-0.783432862	Low	0	426	>=60	MALE	3			Stage III&IV
TCGA-05-4389-01A	-0.754116411	High	0	1369	>=60	MALE	3			Stage I&II
TCGA-05-4390-01A	-0.087887778	High	0	1126	<60	FEMALE	4			Stage I&II
TCGA-05-4396-01A	0.262060501	High	1	303	>=60	MALE	3			Stage III&IV
TCGA-05-4397-01A	-0.682381521	High	1	731	>=60	MALE	2			Stage I&II

TCGA-05-4398-01A	-0.689758724	High	0	1431	<60	FEMALE	2	Stage III&IV
TCGA-05-4402-01A	-0.979772708	Low	1	244	<60	FEMALE	1	Stage III&IV
TCGA-05-4403-01A	-0.803197223	Low	0	578	>=60	MALE	3	Stage I&II
TCGA-05-4405-01A	-0.799513097	Low	0	610	>=60	FEMALE	4	Stage I&II
TCGA-05-4415-01A	0.198598595	High	1	91	<60	MALE	4	Stage III&IV
TCGA-05-4417-01A	-0.292053418	High	0	455	<60	FEMALE	4	Stage I&II
TCGA-05-4418-01A	0.064372754	High	1	274	>=60	MALE	2	Stage III&IV
TCGA-05-4420-01A	-0.374638376	High	0	912	<60	MALE	2	Stage I&II
TCGA-05-4422-01A	-0.40584261	High	0	365	>=60	MALE	4	Stage I&II
TCGA-05-4424-01A	-1.081381777	Low	0	913	>=60	MALE	4	Stage I&II
TCGA-05-4425-01A	-0.737724089	High	0	669	>=60	FEMALE	3	Stage III&IV
TCGA-05-4426-01A	-0.823594277	Low	0	791	>=60	MALE	3	Stage I&II
TCGA-05-4427-01A	-0.737333858	High	0	791	>=60	FEMALE	4	Stage I&II
TCGA-05-4430-01A	-0.935901902	Low	0	761	<60	FEMALE	4	Stage I&II
TCGA-05-4432-01A	-0.765863149	Low	0	761	>=60	MALE	2	Stage I&II
TCGA-05-4433-01A	-0.968399043	Low	0	730	>=60	MALE	3	Stage I&II
TCGA-05-4434-01A	-1.139203806	Low	1	457	>=60	FEMALE	2	Stage III&IV
TCGA-05-5420-01A	-0.291799732	High	0	457	>=60	MALE	4	Stage III&IV
TCGA-05-5423-01A	-0.978791321	Low	0	151	>=60	MALE	3	Stage I&II
TCGA-05-5425-01A	-0.16849861	High	0	882	>=60	MALE	3	Stage I&II
TCGA-05-5428-01A	-0.371829996	High	0	670	<60	MALE	3	Stage I&II
TCGA-05-5429-01A	-0.424025985	High	1	275	>=60	MALE	1	Stage III&IV
TCGA-05-5715-01A	-0.865196894	Low	0	62	>=60	FEMALE	1	Stage I&II

TCGA-35-3615-01A -0.863464652 Low 0 14 <60 MALE 1 NO NO Stage I&II
TCGA-35-4122-01A -0.62923141 High 0 225 >=60 MALE 4 NO NO Stage I&II
TCGA-35-4123-01A -0.566834214 High 0 182 <60 MALE 4 NO NO Stage I&II
TCGA-35-5375-01A -0.562030189 High 0 264 >=60 MALE 4 Stage III&IV
TCGA-38-4625-01A -1.029801605 Low 0 2973 >=60 FEMALE 2 NO NO Stage I&II
TCGA-38-4626-01A -1.198601791 Low 0 3674 <60 FEMALE 2
TCGA-38-4627-01A -0.766075182 Low 1 1147 >=60 FEMALE 4 Stage I&II
TCGA-38-4628-01A -0.537352672 High 1 1492 >=60 FEMALE 1 NO NO Stage I&II
TCGA-38-4629-01A -0.594147062 High 1 864 >=60 MALE 4 NO NO Stage I&II
TCGA-38-4630-01A -0.408301921 High 1 1073 >=60 FEMALE 1 NO NO Stage I&II
TCGA-38-4631-01A -0.249758351 High 1 354 >=60 FEMALE 4 NO NO Stage I&II
TCGA-38-4632-01A -0.681879643 High 1 1357 <60 MALE 4 NO NO Stage III&IV
TCGA-38-6178-01A -0.851105948 Low 0 448 >=60 FEMALE 1 NO YES Stage III&IV
TCGA-38-7271-01A -1.215862977 Low 1 800 >=60 FEMALE 4 NO NO Stage I&II
TCGA-38-A44F-01A-1.17158674 Low 0 133 >=60 MALE 3 YES NO Stage I&II
TCGA-44-2655-01A -0.913460004 Low 0 1324 >=60 FEMALE 3 Stage I&II
TCGA-44-2656-01A -1.050772625 Low 0 1429 <60 MALE 4 Stage I&II
TCGA-44-2657-01A -1.480800489 Low 0 1351 >=60 FEMALE 4 Stage I&II
TCGA-44-2659-01A -0.884258027 Low 0 1367 >=60 FEMALE 4 Stage I&II
TCGA-44-2661-01A -1.216028015 Low 0 1159 >=60 FEMALE 1 Stage I&II

TCGA-44-2662-01A	-0.371703142	High	0	1280	>=60	MALE	3	Stage I&II
TCGA-44-2665-01A	-0.699193452	High	0	1301	<60	FEMALE	1	Stage I&II
TCGA-44-2666-01A	-1.008607178	Low	1	97	<60	MALE	2	Stage I&II
TCGA-44-2668-01A	-0.479460719	High	1	761	<60	MALE	4	Stage I&II
TCGA-44-3396-01A	-0.761785874	Low	0	1130	>=60	FEMALE	2	Stage III&IV
TCGA-44-3398-01A	-0.889865332	Low	0	1163	>=60	FEMALE	4	Stage I&II
TCGA-44-3917-01A	-0.376416825	High	0	1183	<60	FEMALE	2	Stage I&II
TCGA-44-3918-01A	-0.760543575	Low	0	1036	>=60	FEMALE	3	Stage I&II
TCGA-44-3919-01A	-1.054377413	Low	1	1026	>=60	FEMALE	1	Stage I&II
TCGA-44-4112-01A	-0.661655716	High	1	808	>=60	FEMALE	3	Stage I&II
TCGA-44-5643-01A	-0.80394571	Low	0	1013	<60	MALE	2	Stage III&IV
TCGA-44-5644-01A	-0.196378991	High	0	863	<60	FEMALE	2	Stage I&II
TCGA-44-5645-01A	-1.185473587	Low	0	852	>=60	FEMALE	3	Stage I&II
TCGA-44-6145-01A	-0.758756998	High	0	595	>=60	FEMALE	4	Stage I&II
TCGA-44-6146-01A	-0.508456283	High	0	728	>=60	MALE	3	Stage I&II
TCGA-44-6147-01A	-1.189267506	Low	0	845	>=60	FEMALE	3	Stage I&II
TCGA-44-6148-01A	-1.284093143	Low	0	704	>=60	MALE	3	Stage I&II
TCGA-44-6774-01A	-0.70038907	High	0	658	<60	FEMALE	2	Stage III&IV
TCGA-44-6775-01A	-0.942491955	Low	0	705	>=60	FEMALE	4	Stage I&II
TCGA-44-6776-01A	-0.831372843	Low	0	2616	>=60	FEMALE	4	Stage I&II

TCGA-44-6777-01A	-0.986299415	Low	1	987	>=60	FEMALE	4			Stage I&II
TCGA-44-6778-01A	-1.100955393	Low	0	1864	<60	MALE	4			Stage I&II
TCGA-44-6779-01A	-0.707667846	High	1	500	<60	FEMALE	4			Stage I&II
TCGA-44-7659-01A	-1.046492661	Low	0	691	>=60	MALE	2			Stage I&II
TCGA-44-7660-01A	-0.781626662	Low	0	592	>=60	MALE	2			Stage I&II
TCGA-44-7661-01A	-0.888201414	Low	1	557	>=60	FEMALE	3	YES	YES	Stage I&II
TCGA-44-7662-01A	-0.543009236	High	0	218	>=60	MALE	2			Stage I&II
TCGA-44-7667-01A	-0.294011251	High	0	1097	<60	FEMALE	2			Stage I&II
TCGA-44-7669-01A	-0.315369019	High	1	574	<60	MALE	2			Stage I&II
TCGA-44-7670-01A	-0.719319008	High	0	882	<60	FEMALE	2			Stage I&II
TCGA-44-7671-01A	-0.628264646	High	0	889	>=60	MALE	3			Stage I&II
TCGA-44-7672-01A	-0.987166436	Low	0	719	<60	FEMALE	2			Stage I&II
TCGA-44-8117-01A	-0.687405337	High	0	385	<60	FEMALE	4			Stage I&II
TCGA-44-8119-01A	-0.551740406	High	0	285	>=60	MALE	2			Stage I&II
TCGA-44-8120-01A	-0.855066136	Low	0	260	<60	MALE	5			Stage I&II
TCGA-44-A479-01A	-0.790842352	Low	0	486	>=60	FEMALE	4	NO	YES	Stage I&II
TCGA-44-A47A-01A	-0.730994316	High	0	466	>=60	FEMALE	4	NO	NO	Stage I&II
TCGA-44-A47B-01A	-0.652080893	High	0	287	>=60	MALE	3	NO	NO	Stage I&II
TCGA-44-A47G-01A	-0.962887865	Low	0	351	>=60	FEMALE	4	NO	NO	Stage I&II
TCGA-44-A4SS-01A	-0.708041982	High	0	415	>=60	MALE	4			Stage I&II
TCGA-44-A4SU-01A	-1.001142721	Low	1	409	>=60	FEMALE	4			Stage I&II
TCGA-49-4486-01A	-0.827859306	Low	1	2318	>=60	MALE	3			Stage I&II
TCGA-49-4487-01A	-0.29249732	High	1	855	>=60	FEMALE	4			Stage I&II

TCGA-49-4488-01A	-0.67499668	High	1	869	>=60	FEMALE	3		Stage I&II
TCGA-49-4490-01A	-0.723708995	High	1	385	<60	FEMALE	3		Stage III&IV
TCGA-49-4494-01A	-0.583108867	High	1	1081	>=60	MALE	3		Stage III&IV
TCGA-49-4501-01A	-0.994730057	Low	1	1421	>=60	FEMALE	1		Stage I&II
TCGA-49-4505-01A	-0.763505019	Low	1	428	>=60	FEMALE	4		Stage I&II
TCGA-49-4506-01A	0.29023841	High	1	999	>=60	FEMALE	2		Stage I&II
TCGA-49-4507-01A	0.182314577	High	1	268	>=60	FEMALE	4		Stage III&IV
TCGA-49-4510-01A	-0.385992504	High	1	896	<60	FEMALE	2		Stage I&II
TCGA-49-4512-01A	-0.759316715	High	1	905	>=60	FEMALE	1		Stage III&IV
TCGA-49-4514-01A	-0.181952446	High	0	1700	>=60	FEMALE	3		Stage I&II
TCGA-49-6742-01A	-0.094924403	High	1	488	>=60	MALE	3		Stage I&II
TCGA-49-6743-01A	-0.759304249	High	0	1621	>=60	FEMALE	4		Stage III&IV
TCGA-49-6744-01A	-1.065212589	Low	0	1683	>=60	FEMALE	4		Stage I&II
TCGA-49-6745-01A	-0.925825178	Low	0	522	>=60	MALE	4	YES	Stage III&IV
TCGA-49-6761-01A	-0.679517762	High	0	354	>=60	FEMALE	4		Stage III&IV
TCGA-49-6767-01A	-0.529058328	High	0	677	<60	FEMALE	2		Stage I&II
TCGA-49-AAQV-01A	-0.657173715	High	1	677	>=60	FEMALE	1	YES	YES
TCGA-49-AAR0-01A	-1.114161764	Low	0	4765	<60	MALE	2		Stage I&II
TCGA-49-AAR2-01A	0.068592864	High	0	2224	>=60	MALE	4		Stage I&II

TCGA-49-AAR3-01A -0.651401112 High 0 1893 >=60 MALE Stage I&II

TCGA-49-AAR4-01A -0.85828961 Low 1 879 <60 MALE 2 Stage III&IV

TCGA-49-AAR9-01A -0.236487238 High 1 260 >=60 MALE 2 Stage I&II

TCGA-49-AARE-01A -0.617748136 High 1 1229 <60 FEMALE 4 Stage I&II

TCGA-49-AARN-01A -0.813363027 Low 1 1135 <60 FEMALE 5 Stage I&II

TCGA-49-AARO-01A -0.967964929 Low 0 3759 <60 FEMALE 2 Stage I&II

TCGA-49-AARQ-01A -0.693440492 High 0 6732 <60 FEMALE 4 Stage I&II

TCGA-49-AARR-01A -1.184409103 Low 0 4992 >=60 MALE 3 Stage I&II

TCGA-4B-A93V-01A 0.132885088 High 1 300 <60 FEMALE 4 Stage I&II

TCGA-50-5044-01A -0.708910055 High 1 624 >=60 FEMALE Stage III&IV

TCGA-50-5045-01A -1.086656898 Low 1 2174 <60 FEMALE

TCGA-50-5049-01A -1.043890163 Low 0 3094 >=60 MALE YES Stage I&II

TCGA-50-5051-01A -0.105558974 High 1 478 <60 FEMALE 4 YES Stage III&IV

TCGA-50-5055-01A -1.147030538 Low 1 1830 >=60 FEMALE YES Stage I&II

TCGA-50-5066-01A -0.801757676 Low 0 1442 >=60 MALE 1 YES YES Stage I&II

TCGA-50-5068-01A -0.698216127 High 1 1499 <60 FEMALE Stage I&II

TCGA-50-5072-01A 0.02635474 High 1 250 >=60 MALE 3 YES YES Stage III&IV

TCGA-50-5930-01A -0.218501167 High 1 282 <60 MALE Stage III&IV

TCGA-50-5931-01A -0.810632983 Low 1 434 >=60 FEMALE 4 Stage I&II

TCGA-50-5932-01A -0.777803448 Low 1 1235 >=60 MALE Stage I&II

TCGA-50-5933-01A -0.559099539 High 1 2393 >=60 MALE Stage III&IV

TCGA-50-5935-01A -0.925756315 Low 1 653 >=60 FEMALE YES Stage I&II

TCGA-50-5936-01A 0.142236827 High 1 257 <60 MALE YES Stage III&IV

TCGA-50-5939-01A -0.547162524 High 1 460 >=60 MALE 3 Stage I&II

TCGA-50-5941-01A -1.093991588 Low 0 1474 <60 FEMALE 2 Stage III&IV

TCGA-50-5942-01A -1.100980976 Low 0 1847 >=60 FEMALE 4 Stage I&II

TCGA-50-5944-01A -1.173404495 Low 0 1750 >=60 FEMALE Stage I&II

TCGA-50-5946-01A -0.561070888 High 0 1617 >=60 MALE 4 Stage I&II

TCGA-50-6590-01A -0.616252005 High 1 1288 >=60 FEMALE 4 YES Stage I&II

TCGA-50-6591-01A -0.262641502 High 1 119 >=60 FEMALE 1 Stage III&IV

TCGA-50-6592-01A -0.566610793 High 1 777 >=60 FEMALE 4 Stage I&II

TCGA-50-6593-01A -0.888104855 Low 1 336 <60 FEMALE 4 YES YES Stage III&IV

TCGA-50-6594-01A -0.575284186 High 1 370 >=60 FEMALE 4 YES Stage III&IV

TCGA-50-6595-01A -0.481067192 High 1 189 >=60 FEMALE 3 YES YES Stage III&IV

TCGA-50-6597-01A -0.71788682 High 1 1268 >=60 FEMALE 1 YES Stage I&II

TCGA-50-6673-01A	-1.059441941	Low	1	22	>=60	FEMALE	1			Stage I&II
TCGA-50-7109-01A	-0.088584808	High	1	308	>=60	MALE	2			Stage I&II
TCGA-50-8457-01A	-1.30974037	Low	0	1125	>=60	FEMALE	4	YES	YES	Stage I&II
TCGA-50-8459-01A	-0.83833333	Low	0	1119	>=60	MALE	2	YES		Stage I&II
TCGA-50-8460-01A	-1.117349571	Low	0	829	>=60	MALE		YES		Stage I&II
TCGA-53-7624-01A	-0.324287354	High	1	1043	<60	FEMALE	2	NO	NO	Stage III&IV
TCGA-53-7626-01A	-1.177371251	Low	1	929	>=60	FEMALE	4	NO	NO	Stage I&II
TCGA-53-7813-01A	-0.70098984	High	0	424	<60	FEMALE	4	NO	NO	Stage III&IV
TCGA-53-A4EZ-01A	-0.521853136	High	0	1071	>=60	MALE	3	YES	YES	Stage I&II
TCGA-55-1592-01A	-0.905678605	Low	1	701	>=60	MALE	4			Stage I&II
TCGA-55-1594-01A	-0.683140906	High	0	1178	>=60	MALE	2			Stage III&IV
TCGA-55-1596-01A	-0.532772432	High	0	2065	<60	MALE	2			Stage I&II
TCGA-55-5899-01A	-0.539704361	High	0	930	<60	MALE	2			
TCGA-55-6543-01A	-0.967224148	Low	0	435	>=60	FEMALE	4			Stage I&II
TCGA-55-6642-01A	-0.372624361	High	0	2449	>=60	MALE	2			Stage I&II
TCGA-55-6712-01A	-0.784764011	Low	1	171	>=60	MALE	4			Stage I&II
TCGA-55-6968-01A	-0.741513194	High	1	1293	>=60	MALE	2			Stage III&IV
TCGA-55-6970-01A	-0.218953832	High	1	464	>=60	FEMALE	4	NO	NO	Stage III&IV
TCGA-55-6971-01A	-1.099127592	Low	0	1400	<60	FEMALE	4	NO	NO	Stage I&II
TCGA-55-6972-01A	-0.617742721	High	1	1632	>=60	MALE	2	NO	NO	Stage

I&II

TCGA-55-6975-01A 0.015123987 High 1 118 >=60 MALE 4 NO NO Stage I&II

TCGA-55-6978-01A -0.82847041 Low 1 176 >=60 MALE 1 NO NO Stage I&II

TCGA-55-6979-01A -1.033436057 Low 1 237 <60 FEMALE 3 NO NO Stage I&II

TCGA-55-6980-01A -1.026352297 Low 0 2109 <60 MALE 1 NO NO Stage I&II

TCGA-55-6981-01A -0.656290477 High 1 1379 <60 FEMALE 3 NO NO Stage III&IV

TCGA-55-6982-01A -0.842002749 Low 1 995 >=60 FEMALE 1 NO NO Stage I&II

TCGA-55-6983-01A -1.042586174 Low 0 2823 >=60 MALE 4 NO NO Stage I&II

TCGA-55-6984-01A -0.545726949 High 1 760 >=60 FEMALE NO NO Stage I&II

TCGA-55-6985-01A -0.937608591 Low 0 1233 <60 FEMALE 4 NO NO Stage I&II

TCGA-55-6986-01A -0.934521532 Low 0 3261 >=60 FEMALE 1 NO NO Stage I&II

TCGA-55-6987-01A -0.424111343 High 0 2137 >=60 MALE 4 NO NO Stage I&II

TCGA-55-7227-01A -0.921048969 Low 1 952 >=60 MALE 3 Stage III&IV

TCGA-55-7281-01A -0.941521451 Low 0 872 >=60 FEMALE 4 Stage I&II

TCGA-55-7283-01A -0.968043474 Low 0 609 >=60 FEMALE 3 NO NO Stage III&IV

TCGA-55-7570-01A -0.411255814 High 0 824 >=60 MALE 2 Stage I&II

TCGA-55-7573-01A -1.173519056 Low 0 487 >=60 FEMALE 3 NO NO Stage I&II

TCGA-55-7574-01A -1.135431097 Low 1 995 >=60 FEMALE 4 Stage I&II

TCGA-55-7576-01A -0.691942462 High 0 670 <60 MALE 2 Stage I&II

TCGA-55-7724-01A -1.050768136 Low 0 705 >=60 FEMALE 4 Stage I&II

TCGA-55-7725-01A	-1.110157228	Low	0	442	>=60	FEMALE	3	NO	NO	Stage I&II
TCGA-55-7726-01A	-0.470385718	High	0	652	>=60	FEMALE	3			Stage I&II
TCGA-55-7727-01A	-0.966667435	Low	0	119	>=60	MALE	2			Stage III&IV
TCGA-55-7728-01A	-1.008624848	Low	0	704	>=60	FEMALE	4	NO	NO	Stage I&II
TCGA-55-7815-01A	-0.580063233	High	0	773	>=60	MALE	1			Stage I&II
TCGA-55-7816-01A	-1.115648446	Low	1	468	<60	FEMALE	1			Stage III&IV
TCGA-55-7903-01A	-0.588161343	High	0	567	>=60	MALE	2	NO	NO	Stage I&II
TCGA-55-7907-01A	-0.758859434	High	1	343	>=60	MALE	3			Stage I&II
TCGA-55-7910-01A	-0.07619433	High	0	1040	<60	FEMALE	4			Stage I&II
TCGA-55-7911-01A	-0.680668892	High	0	537	>=60	FEMALE	3	NO	NO	Stage I&II
TCGA-55-7914-01A	-0.809781235	Low	1	187	>=60	FEMALE	2	YES	YES	Stage I&II
TCGA-55-7994-01A	-0.804958332	Low	0	603	>=60	MALE	2	NO	NO	Stage I&II
TCGA-55-7995-01A	-0.805612251	Low	0	889	>=60	FEMALE	4	NO	NO	Stage I&II
TCGA-55-8085-01A	-0.7453244	High	0	904	>=60	MALE	2			Stage I&II
TCGA-55-8087-01A	-0.901085869	Low	0	462	<60	FEMALE	1	NO	NO	Stage I&II
TCGA-55-8089-01A	-1.012353558	Low	1	702	<60	MALE	2	YES	YES	Stage I&II
TCGA-55-8090-01A	-0.727269755	High	1	598	>=60	MALE	2			Stage I&II
TCGA-55-8091-01A	-0.76455859	Low	0	600	>=60	MALE	3	NO	NO	Stage I&II
TCGA-55-8092-01A	-0.180016675	High	1	154	>=60	MALE	4			Stage I&II
TCGA-55-8094-01A	0.36988789	High	0	541	<60	MALE	4	YES	YES	Stage III&IV
TCGA-55-8096-01A	-0.806308225	Low	1	719	>=60	FEMALE	2	NO	NO	Stage I&II
TCGA-55-8097-01A	-1.241118561	Low	0	476	>=60	FEMALE	4	NO	NO	Stage

I&II

TCGA-55-8203-01A -0.852981845 Low 0 547 >=60 FEMALE 2 NO NO Stage

I&II

TCGA-55-8204-01A -0.846464211 Low 0 515 >=60 FEMALE 3 Stage

I&II

TCGA-55-8205-01A -1.007380836 Low 0 599 >=60 FEMALE 4 NO YES

Stage I&II

TCGA-55-8206-01A -1.250955602 Low 0 888 <60 MALE 1 Stage I&II

TCGA-55-8207-01A -1.125209707 Low 0 977 >=60 MALE 3 Stage I&II

TCGA-55-8208-01A -1.114381225 Low 0 674 >=60 FEMALE 2 Stage

I&II

TCGA-55-8299-01A -0.54775604 High 1 469 >=60 FEMALE 4 Stage I&II

TCGA-55-8301-01A -1.014652985 Low 0 534 <60 MALE 2 Stage I&II

TCGA-55-8302-01A -0.759637566 High 0 478 <60 MALE 2 Stage I&II

TCGA-55-8505-01A -0.029244373 High 0 440 >=60 MALE 1 Stage

III&IV

TCGA-55-8506-01A -0.421699506 High 0 11 >=60 FEMALE 2 Stage

I&II

TCGA-55-8507-01A -0.849947438 Low 0 418 <60 MALE 2 Stage I&II

TCGA-55-8508-01A -0.422187398 High 0 617 >=60 FEMALE 2 Stage

I&II

TCGA-55-8510-01A -1.09924454 Low 0 539 <60 FEMALE 2 Stage I&II

TCGA-55-8511-01A -0.804183373 Low 0 552 >=60 FEMALE 2 Stage

I&II

TCGA-55-8512-01A -1.068959424 Low 1 607 <60 MALE 2 Stage III&IV

TCGA-55-8513-01A -1.301581646 Low 0 791 >=60 FEMALE 1 YES YES

Stage I&II

TCGA-55-8514-01A -1.017621317 Low 0 520 >=60 FEMALE 4 Stage

I&II

TCGA-55-8614-01A -0.633479294 High 0 536 >=60 MALE 3 Stage I&II

TCGA-55-8615-01A -0.157812413 High 0 446 >=60 MALE 2 Stage

III&IV

TCGA-55-8616-01A -0.858778721 Low 0 48 <60 FEMALE 4 Stage I&II

TCGA-55-8619-01A	-1.284218956	Low	0	416	>=60	FEMALE	1			Stage I&II
TCGA-55-8620-01A	-0.804693984	Low	1	375	>=60	MALE	4	NO	NO	Stage III&IV
TCGA-55-8621-01A	-1.243304014	Low	0	515	>=60	FEMALE	4			Stage I&II
TCGA-55-A48X-01A	-1.036938613	Low	0	689	>=60	FEMALE	4			Stage I&II
TCGA-55-A48Y-01A	0.255442589	High	0	630	>=60	MALE	2			Stage I&II
TCGA-55-A48Z-01A	-0.62016975	High	0	651	>=60	FEMALE	4	YES	YES	Stage III&IV
TCGA-55-A490-01A	-0.551245496	High	1	99	>=60	MALE	3	YES	YES	Stage I&II
TCGA-55-A491-01A	-0.744259079	High	0	626	>=60	FEMALE	3	NO	NO	Stage I&II
TCGA-55-A492-01A	-0.141083698	High	0	596	>=60	FEMALE	3	NO	NO	Stage I&II
TCGA-55-A493-01A	-0.686503502	High	0	28	<60	FEMALE	2	NO	NO	Stage I&II
TCGA-55-A494-01A	-0.233668102	High	0	481	>=60	FEMALE	3	YES	YES	Stage I&II
TCGA-55-A4DF-01A	-0.898753705	Low	1	614	>=60	MALE	3			Stage I&II
TCGA-55-A4DG-01A	-1.181339643	Low	0	608	>=60	MALE	4			Stage I&II
TCGA-55-A57B-01A	-0.870105489	Low	0	546	>=60	FEMALE	1	NO	NO	Stage I&II
TCGA-62-8394-01A	-0.758768545	High	1	139	>=60	FEMALE	1	NO	NO	Stage III&IV
TCGA-62-8395-01A	-1.03645244	Low	0	1216	>=60	FEMALE	1	NO	YES	Stage I&II
TCGA-62-8397-01A	-0.923677925	Low	0	1289	>=60	FEMALE	1	NO	NO	Stage I&II

TCGA-62-8398-01A	0.023884396	High	1	444	<60	MALE	4	NO	NO	Stage III&IV
TCGA-62-8399-01A	-0.375387874	High	0	2696	>=60	MALE	4	NO	NO	Stage III&IV
TCGA-62-8402-01A	-0.607501505	High	1	1498	>=60	FEMALE	1	NO	YES	Stage III&IV
TCGA-62-A46O-01A	0.470429504	High	1	1454	>=60	FEMALE	2	NO	NO	Stage I&II
TCGA-62-A46P-01A	-0.637684142	High	1	594	>=60	MALE	4	NO	NO	Stage I&II
TCGA-62-A46R-01A	-0.784444857	Low	1	1725	<60	FEMALE	4	NO	NO	Stage I&II
TCGA-62-A46S-01A	-0.540944609	High	1	1653	>=60	MALE	4	NO	NO	Stage I&II
TCGA-62-A46V-01A	-0.714726483	High	0	2199	>=60	FEMALE	3	NO	NO	Stage I&II
TCGA-62-A46Y-01A	-0.849962337	Low	1	414	>=60	FEMALE	1	NO	NO	Stage III&IV
TCGA-62-A470-01A	-0.211437007	High	1	1194	>=60	MALE	3	NO	NO	Stage I&II
TCGA-62-A471-01A	0.362498809	High	0	1246	>=60	MALE	4	NO	NO	Stage I&II
TCGA-62-A472-01A	-0.663724915	High	0	910	>=60	MALE	2	NO	NO	Stage I&II
TCGA-64-1676-01A	-0.403220029	High	0	1728	<60	MALE	2			Stage I&II
TCGA-64-1677-01A	-0.538058359	High	1	628	>=60	FEMALE	2			Stage III&IV
TCGA-64-1678-01A	-0.032896553	High	0	1189	>=60	FEMALE	3			
TCGA-64-1679-01A	-0.687966933	High	0	2488	<60	FEMALE	2			Stage III&IV
TCGA-64-1680-01A	-0.677478405	High	0	1126	>=60	MALE	3			Stage III&IV
TCGA-64-1681-01A	-1.168963143	Low	1	1167	>=60	FEMALE	3		YES	Stage I&II
TCGA-64-5774-01A	-0.118253945	High	0	2676	>=60	MALE	4			Stage I&II

TCGA-64-5775-01A -0.432252255 High 1 62 >=60 MALE 2 Stage III&IV
TCGA-64-5778-01A -0.8323966 Low 0 1305 >=60 MALE 4 Stage I&II
TCGA-64-5779-01A -0.634986132 High 0 864 >=60 MALE 4 Stage III&IV
TCGA-64-5781-01A 0.011135602 High 0 1559 <60 FEMALE 4 Stage I&II
TCGA-64-5815-01A -0.75956243 High 0 866 >=60 MALE 3 YES Stage I&II
TCGA-67-3770-01A -1.231562173 Low 0 610 >=60 FEMALE 3 NO NO Stage I&II
TCGA-67-3771-01A -1.001364205 Low 0 610 >=60 FEMALE 4 NO NO Stage I&II
TCGA-67-3772-01A -1.054051034 Low 0 573 >=60 FEMALE 1 NO NO Stage I&II
TCGA-67-3773-01A -0.871176406 Low 0 427 >=60 FEMALE 3 NO NO Stage I&II
TCGA-67-3774-01A -0.816746835 Low 0 385 >=60 FEMALE 3 NO NO Stage I&II
TCGA-67-6215-01A -0.634086353 High 0 174 <60 FEMALE 1 NO NO Stage I&II
TCGA-67-6216-01A -0.932076038 Low 0 141 <60 FEMALE 1 NO NO Stage I&II
TCGA-67-6217-01A -1.08624107 Low 0 422 >=60 FEMALE 3 YES YES Stage I&II
TCGA-69-7760-01A -0.366086881 High 0 202 >=60 MALE 1 NO YES Stage I&II
TCGA-69-7761-01A -0.893386251 Low 0 186 >=60 MALE 3 YES Stage I&II
TCGA-69-7763-01A -0.832116133 Low 0 690 >=60 MALE 4 NO NO Stage I&II
TCGA-69-7764-01A -0.451096856 High 0 414 >=60 MALE 3 NO YES Stage I&II
TCGA-69-7765-01A -0.730494058 High 0 165 <60 MALE 4 NO NO
TCGA-69-7973-01A -0.518963455 High 0 230 <60 FEMALE 4 YES Stage I&II
TCGA-69-7974-01A -0.99629816 Low 0 184 <60 FEMALE 4 YES Stage III&IV

TCGA-69-7978-01A -0.686324446 High 0 134 <60 MALE 2 YES Stage I&II
TCGA-69-7979-01A -0.510081408 High 0 408 >=60 FEMALE 2 NO NO Stage I&II
TCGA-69-7980-01A -0.939688747 Low 0 411 >=60 FEMALE 4 NO YES Stage I&II
TCGA-69-8253-01A -0.917090222 Low 0 426 <60 FEMALE 4 NO NO Stage I&II
TCGA-69-8254-01A -1.048284626 Low 0 409 >=60 MALE 3 NO YES
TCGA-69-8255-01A 0.119978957 High 0 129 >=60 MALE 2 NO YES Stage I&II
TCGA-69-8453-01A -1.239676006 Low 0 813 >=60 MALE 3 Stage I&II
TCGA-69-A59K-01A -0.88093105 Low 0 591 >=60 FEMALE 4 NO YES Stage I&II
TCGA-71-6725-01A -0.521886576 High 0 256 <60 FEMALE 2 Stage I&II
TCGA-71-8520-01A -0.946185877 Low 1 210 >=60 FEMALE 1 Stage I&II
TCGA-73-4658-01A -0.939651899 Low 1 1600 >=60 FEMALE 3 NO Stage I&II
TCGA-73-4659-01A -0.386251732 High 1 711 >=60 MALE 3 NO NO Stage III&IV
TCGA-73-4662-01A -0.978507103 Low 0 2515 >=60 FEMALE 3 NO NO Stage I&II
TCGA-73-4666-01A -0.883049559 Low 0 800 <60 FEMALE 4 NO NO Stage III&IV
TCGA-73-4668-01A -0.492086887 High 0 467 >=60 FEMALE 4 NO NO Stage I&II
TCGA-73-4670-01A 0.211447728 High 0 131 >=60 FEMALE 4 NO NO Stage III&IV
TCGA-73-4675-01A -0.746927837 High 1 922 <60 MALE 4 NO NO Stage III&IV
TCGA-73-4676-01A -0.689955824 High 1 281 <60 MALE 4 NO NO Stage I&II
TCGA-73-4677-01A -0.724334273 High 1 38 >=60 MALE 3 NO NO
TCGA-73-7498-01A -1.146750747 Low 0 1189 <60 FEMALE 4 NO NO Stage I&II
TCGA-73-7499-01A -0.929302705 Low 1 1531 >=60 FEMALE 1 NO NO Stage I&II

TCGA-73-A9RS-01A	0.117573796	High	1	340	<60	MALE	2	YES	YES	Stage I&II
TCGA-75-5125-01A	-0.693644904	High	1	2027		MALE	3	NO	NO	Stage I&II
TCGA-75-5146-01A	-1.063258017	Low	0	2368		MALE	3	NO	NO	Stage I&II
TCGA-75-5147-01A	-1.030186579	Low	0	1333		FEMALE	1	NO	NO	Stage I&II
TCGA-75-6206-01A	-0.624698149	High	0	2590		MALE	3			Stage I&II
TCGA-75-6212-01A	-1.228147831	Low	1	1516		FEMALE	1			Stage I&II
TCGA-75-6214-01A	-0.402163209	High	1	1115		FEMALE	2			Stage III&IV
TCGA-75-7025-01A	-1.149916757	Low	0	3305		MALE	3			Stage I&II
TCGA-75-7027-01A	0.191928601	High	0	3059		MALE	2			Stage I&II
TCGA-78-7143-01A	-1.063783244	Low	1	4961	>=60	FEMALE	1	NO	NO	Stage I&II
TCGA-78-7145-01A	-0.648941012	High	1	826	<60	FEMALE	4	NO	NO	Stage III&IV
TCGA-78-7146-01A	-0.534507025	High	1	173	>=60	FEMALE	4	NO	NO	Stage III&IV
TCGA-78-7147-01A	-0.907668062	Low	1	586	>=60	FEMALE	2	NO	NO	Stage I&II
TCGA-78-7148-01A	0.060903957	High	1	626	>=60	MALE	2	NO	NO	Stage I&II
TCGA-78-7149-01A	-1.02236822	Low	0	3940	>=60	MALE	2	NO	NO	Stage III&IV
TCGA-78-7150-01A	0.05151142	High	1	666	<60	MALE	4	NO	NO	Stage I&II
TCGA-78-7152-01A	-0.908100771	Low	1	1215	>=60	MALE	4	NO	NO	Stage I&II
TCGA-78-7153-01A	-0.553572628	High	0	3635	>=60	FEMALE	4	NO	NO	Stage I&II
TCGA-78-7154-01A	-0.707813545	High	1	593	>=60	MALE	4	NO	NO	Stage III&IV
TCGA-78-7155-01A	-0.401552817	High	1	1171	>=60	MALE	4	NO	NO	Stage I&II

TCGA-78-7156-01A	-0.613896009	High	1	976	>=60	MALE	3	NO	NO	Stage III&IV
TCGA-78-7158-01A	-0.560148966	High	1	179	<60	FEMALE	4	NO	NO	Stage III&IV
TCGA-78-7159-01A	-0.294309289	High	0	1974	>=60	FEMALE	4	NO	NO	Stage I&II
TCGA-78-7160-01A	-0.292671992	High	1	697	>=60	MALE	4	NO	NO	Stage III&IV
TCGA-78-7161-01A	0.160593117	High	1	291	>=60	FEMALE	4	NO	YES	Stage I&II
TCGA-78-7162-01A	-0.79606639	Low	1	3169	>=60	MALE	2	NO	NO	Stage I&II
TCGA-78-7163-01A	-0.687470777	High	0	7248	>=60	MALE	4	NO	NO	Stage I&II
TCGA-78-7166-01A	-0.089507277	High	1	258	>=60	MALE	3	NO	NO	Stage I&II
TCGA-78-7167-01A	-0.451883761	High	1	2681	>=60	MALE	2	NO	NO	Stage III&IV
TCGA-78-7220-01A	-0.027773076	High	1	807	<60	FEMALE	2	NO	NO	Stage III&IV
TCGA-78-7535-01A	-0.4584317	High	1	949	<60	MALE	2	NO	NO	Stage I&II
TCGA-78-7536-01A	-0.18027169	High	1	244	>=60	MALE	4	NO	NO	Stage III&IV
TCGA-78-7537-01A	-1.100215561	Low	1	1622	>=60	MALE	3	NO	NO	Stage I&II
TCGA-78-7539-01A	-0.879174485	Low	0	791	>=60	FEMALE	3	NO	NO	Stage I&II
TCGA-78-7540-01A	-0.766642724	Low	1	1197	>=60	FEMALE	1	NO	NO	Stage I&II
TCGA-78-7542-01A	-0.331979123	High	1	321	<60	MALE	2	NO	NO	Stage I&II
TCGA-78-7633-01A	-0.425255245	High	1	1528	>=60	MALE	4	NO	NO	Stage I&II
TCGA-78-8640-01A	-0.772429737	Low	0	7062	<60	MALE	2	NO	NO	Stage I&II
TCGA-78-8648-01A	-1.159752758	Low	1	1209	<60	FEMALE	2	NO	NO	Stage I&II
TCGA-78-8655-01A	-0.783940421	Low	0	2360	>=60	FEMALE	4	NO	NO	

Stage I&II

TCGA-78-8660-01A -0.938577595 Low 1 321 >=60 MALE 4 NO NO Stage I&II

TCGA-78-8662-01A -0.574126814 High 1 3361 <60 FEMALE 2 NO NO Stage I&II

TCGA-80-5608-01A -0.37517666 High 0 2832 FEMALE 2 Stage I&II

TCGA-80-5611-01A -0.847659172 Low 0 2595 MALE 4 Stage I&II

TCGA-83-5908-01A -0.489376116 High 0 824 <60 FEMALE 4 NO NO Stage I&II

TCGA-86-6562-01A -0.953450393 Low 1 376 <60 MALE 1 Stage I&II

TCGA-86-6851-01A -1.061924942 Low 0 179 >=60 FEMALE 2 Stage I&II

TCGA-86-7701-01A -0.320992409 High 0 947 >=60 MALE 1 Stage III&IV

TCGA-86-7711-01A -0.537513062 High 1 1046 >=60 MALE 2 Stage I&II

TCGA-86-7713-01A -0.278358732 High 0 1157 >=60 MALE 1 Stage I&II

TCGA-86-7714-01A -1.034791356 Low 1 625 >=60 FEMALE 2 NO Stage III&IV

TCGA-86-7953-01A -0.699490592 High 0 997 >=60 FEMALE 1 NO Stage I&II

TCGA-86-7954-01A -0.852735269 Low 0 605 >=60 FEMALE 3 NO Stage I&II

TCGA-86-7955-01A 0.518751509 High 0 1072 >=60 MALE 1 NO Stage I&II

TCGA-86-8054-01A 0.086696557 High 0 1148 >=60 MALE 2 NO NO Stage I&II

TCGA-86-8055-01A -0.818418489 Low 1 124 >=60 MALE 2 NO Stage I&II

TCGA-86-8056-01A -1.159875936 Low 0 139 >=60 FEMALE 3 NO NO Stage III&IV

TCGA-86-8073-01A -0.928737392 Low 0 740 <60 MALE 2 NO NO Stage I&II

TCGA-86-8074-01A -0.712698823 High 0 24 >=60 FEMALE 2 NO NO Stage I&II

TCGA-86-8075-01A -0.782281748 Low 1 694 >=60 FEMALE 1 NO NO Stage

I&II

TCGA-86-8076-01A -0.770317878 Low 0 993 <60 MALE 1 NO NO Stage I&II

TCGA-86-8278-01A -0.731425532 High 0 944 >=60 FEMALE 1 Stage I&II

TCGA-86-8279-01A -0.452889669 High 0 949 <60 MALE 1 Stage I&II

TCGA-86-8280-01A -1.19073143 Low 0 701 <60 FEMALE 1 Stage I&II

TCGA-86-8358-01A -0.718467689 High 0 653 <60 MALE 2 Stage I&II

TCGA-86-8359-01A 0.112559478High 1 444 <60 MALE 2 Stage III&IV

TCGA-86-8585-01A -0.174862263 High 0 353 <60 MALE 1 NO Stage I&II

TCGA-86-8668-01A -1.067543551 Low 0 423 >=60 FEMALE 1 Stage I&II

TCGA-86-8669-01A -0.936976695 Low 0 938 >=60 MALE 2 Stage I&II

TCGA-86-8671-01A -1.247776074 Low 0 839 >=60 FEMALE 1 Stage I&II

TCGA-86-8672-01A -0.305625222 High 1 19 <60 MALE 1 Stage I&II

TCGA-86-8673-01A -0.690713412 High 0 862 >=60 MALE 2 Stage I&II

TCGA-86-8674-01A -0.716222269 High 0 806 <60 MALE 4 Stage I&II

TCGA-86-A456-01A-1.037133026 Low 0 896 >=60 FEMALE 4 Stage I&II

TCGA-86-A4D0-01A 0.229887302 High 1 116 <60 MALE 2 NO NO Stage I&II

TCGA-86-A4JF-01A -0.626204522 High 1 737 <60 MALE 4 NO NO Stage I&II

TCGA-86-A4P7-01A-0.98831809 Low 0 415 >=60 FEMALE 1 NO NO Stage I&II

TCGA-86-A4P8-01A-1.333380097 Low 0 805 <60 FEMALE 1 YES YES Stage III&IV

TCGA-91-6828-01A -1.010838496 Low 0 323 >=60 MALE 3 Stage I&II

TCGA-91-6829-01A -0.700979071 High 1 1258 >=60 MALE 4 Stage I&II

TCGA-91-6830-01A -1.035938117 Low 0 60 >=60 FEMALE 4 Stage I&II

TCGA-91-6831-01A 0.096299268 High 0 310 >=60 MALE 2 Stage I&II

TCGA-91-6835-01A -1.2129052 Low 0 79 >=60 FEMALE 3 NO Stage I&II

TCGA-91-6836-01A -0.33441255 High 0 417 <60 FEMALE 2 NO Stage I&II

TCGA-91-6840-01A -0.787464452 Low 0 372 <60 FEMALE 3 Stage I&II

TCGA-91-6847-01A -0.126582935 High 0 842 >=60 FEMALE 3 Stage I&II
TCGA-91-6848-01A -0.810632819 Low 0 224 <60 MALE 4 Stage III&IV
TCGA-91-6849-01A -1.316130833 Low 0 35 >=60 FEMALE 3 YES Stage III&IV
TCGA-91-7771-01A -1.017934049 Low 0 492 >=60 MALE 4 NO Stage I&II
TCGA-91-8496-01A -0.90789676 Low 0 505 >=60 FEMALE 1 NO NO Stage I&II
TCGA-91-8497-01A -1.268002778 Low 1 434 >=60 FEMALE 1 Stage I&II
TCGA-91-8499-01A -0.731114303 High 0 36 >=60 FEMALE 2 YES Stage I&II
TCGA-91-A4BC-01A -0.045228888 High 0 44 <60 MALE 4 NO Stage I&II
TCGA-91-A4BD-01A -1.005677543 Low 0 603 >=60 MALE 3 NO Stage I&II
TCGA-93-7347-01A -1.18582943 Low 0 683 >=60 FEMALE 3 NO NO Stage I&II
TCGA-93-7348-01A -0.948513117 Low 0 531 >=60 FEMALE 4 NO NO Stage I&II
TCGA-93-8067-01A 0.276549116 High 0 186 >=60 MALE 4 NO NO Stage I&II
TCGA-93-A4JN-01A -0.901753772 Low 0 718 >=60 MALE 3 YES YES Stage III&IV
TCGA-93-A4JO-01A -1.075202262 Low 1 33 >=60 MALE 4 NO NO Stage I&II
TCGA-93-A4JP-01A -1.169310105 Low 0 578 >=60 MALE 1 NO YES Stage III&IV
TCGA-93-A4JQ-01A -0.893476585 Low 0 526 <60 MALE 3 NO NO Stage I&II
TCGA-95-7039-01A -0.770531969 Low 0 1272 <60 FEMALE 2 Stage I&II
TCGA-95-7043-01A -0.613048464 High 1 503 >=60 FEMALE 4 Stage I&II
TCGA-95-7562-01A -0.530924436 High 1 87 >=60 MALE 3 Stage I&II
TCGA-95-7567-01A -0.683447145 High 0 568 >=60 MALE 4 Stage I&II
TCGA-95-7944-01A -0.308467582 High 0 377 >=60 MALE 2 Stage I&II
TCGA-95-7947-01A -0.863067634 Low 0 477 >=60 MALE 4 Stage I&II

TCGA-95-7948-01A	-0.548986822	High	0	476	<60	FEMALE	3			Stage I&II
TCGA-95-8039-01A	-0.990236553	Low	0	830	>=60	MALE	1			Stage I&II
TCGA-95-8494-01A	-0.490982039	High	0	84	>=60	MALE	5	NO	NO	Stage I&II
TCGA-95-A4VK-01A	-0.714203751	High	0	651	>=60	FEMALE	3	NO	YES	Stage III&IV
TCGA-95-A4VN-01A	-0.886791384	Low	0	553	>=60	FEMALE	4	NO	YES	Stage I&II
TCGA-95-A4VP-01A	-0.422223751	High	0	605	>=60	FEMALE	4	NO	NO	Stage III&IV
TCGA-97-7546-01A	-1.266025419	Low	0	1285	>=60	FEMALE	3			Stage I&II
TCGA-97-7547-01A	-1.0966029	Low	0	1965	>=60	FEMALE	3			Stage I&II
TCGA-97-7552-01A	-1.298533704	Low	0	1932	>=60	MALE	3			Stage I&II
TCGA-97-7553-01A	-1.294499271	Low	0	1870	<60	FEMALE	2			Stage I&II
TCGA-97-7554-01A	-0.935529447	Low	0	775	>=60	FEMALE	3			Stage III&IV
TCGA-97-7937-01A	-0.523657537	High	0	564	>=60	MALE	3	YES	YES	Stage I&II
TCGA-97-7938-01A	-1.011140787	Low	1	18	>=60	FEMALE	3	NO	YES	Stage I&II
TCGA-97-7941-01A	-1.219785917	Low	0	484	>=60	FEMALE	3	NO	YES	Stage I&II
TCGA-97-8171-01A	-0.46940623	High	0	568	>=60	MALE	2	NO	YES	Stage III&IV
TCGA-97-8172-01A	-1.15139049	Low	0	545	>=60	FEMALE	3	NO	YES	Stage I&II
TCGA-97-8174-01A	-0.609008382	High	1	164	>=60	MALE	3	YES	YES	Stage I&II
TCGA-97-8175-01A	-0.552262907	High	0	551	<60	FEMALE	3	YES	YES	Stage I&II

TCGA-97-8176-01A	-0.070207356	High	1	468	>=60	MALE	4	NO	YES	Stage III&IV
TCGA-97-8177-01A	-1.026378812	Low	0	499	<60	FEMALE	1	NO	YES	Stage I&II
TCGA-97-8179-01A	-0.128705849	High	0	435	>=60	MALE	3	YES	YES	Stage I&II
TCGA-97-8547-01A	-0.939395462	Low	0	657	>=60	FEMALE	1	NO	YES	Stage III&IV
TCGA-97-8552-01A	-1.228726789	Low	0	626	<60	FEMALE	1	NO	YES	Stage I&II
TCGA-97-A4LX-01A	-1.154410854	Low	0	614	>=60	MALE	3	NO	NO	Stage I&II
TCGA-97-A4M0-01A	-0.965629445	Low	0	652	>=60	FEMALE	4	NO	NO	Stage I&II
TCGA-97-A4M1-01A	-1.075376738	Low	0	601	<60	FEMALE	3	NO	NO	Stage I&II
TCGA-97-A4M2-01A	-1.276386504	Low	0	624	>=60	MALE	2	NO	NO	Stage I&II
TCGA-97-A4M3-01A	-0.502582704	High	0	540	>=60	FEMALE	3	NO	NO	Stage I&II
TCGA-97-A4M5-01A	-0.594444355	High	0	634	>=60	MALE	3	NO	NO	Stage I&II
TCGA-97-A4M6-01A	-1.012824586	Low	0	568	<60	FEMALE	1	NO	YES	Stage I&II
TCGA-97-A4M7-01A	-1.161312506	Low	0	629	>=60	MALE	3	NO	YES	Stage I&II
TCGA-99-7458-01A	-1.160555507	Low	0	747	>=60	FEMALE	4	YES	YES	Stage III&IV
TCGA-99-8025-01A	-0.766039535	Low	0	1060	>=60	FEMALE	4	YES	YES	Stage III&IV
TCGA-99-8028-01A	-1.028951767	Low	0	1118	<60	FEMALE	4	YES	YES	Stage I&II
TCGA-99-8032-01A	-0.135222507	High	0	44	>=60	MALE	2	NO	YES	Stage

I&II
TCGA-99-8033-01A 0.37872866 High 1 656 >=60 FEMALE 1 NO YES Stage III&IV
TCGA-99-AA5R-01A -1.321431107 Low 0 658 >=60 FEMALE 2 YES YES Stage I&II
TCGA-J2-8192-01A -1.012063049 Low 0 739 >=60 FEMALE 1 NO YES Stage I&II
TCGA-J2-8194-01A -0.181242353 High 0 724 >=60 FEMALE 3 Stage I&II
TCGA-J2-A4AD-01A -0.778564491 Low 1 550 >=60 FEMALE 2 NO NO Stage I&II
TCGA-J2-A4AE-01A -1.176889078 Low 0 1079 >=60 FEMALE 1 NO NO Stage I&II
TCGA-J2-A4AG-01A -1.127596037 Low 0 988 >=60 FEMALE 3 NO NO Stage I&II
TCGA-L4-A4E5-01A -0.684789325 High 0 578 <60 FEMALE 4 NO NO Stage I&II
TCGA-L4-A4E6-01A -1.292737612 Low 0 435 >=60 MALE 3 NO NO Stage I&II
TCGA-L9-A443-01A -0.792337552 Low 1 193 >=60 FEMALE 4 NO NO Stage I&II
TCGA-L9-A444-01A -0.986871798 Low 0 307 >=60 FEMALE 2 NO NO Stage I&II
TCGA-L9-A50W-01A -0.723339445 High 1 442 >=60 MALE 3 YES YES Stage I&II
TCGA-L9-A5IP-01A 0.111254747 High 1 58 <60 FEMALE 4 YES YES Stage III&IV
TCGA-L9-A743-01A -1.009458244 Low 0 664 <60 MALE 2 NO NO Stage I&II
TCGA-L9-A7SV-01A -0.706418536 High 0 565 >=60 MALE 3 NO NO Stage I&II
TCGA-L9-A8F4-01A -0.856305485 Low 0 476 >=60 FEMALE 2 NO NO Stage I&II
TCGA-MN-A4N1-01A -0.334087562 High 0 827 >=60 MALE 2 YES YES

Stage I&II

TCGA-MN-A4N4-01A -0.561364405 High 0 1175 <60 MALE 2 NO NO Stage I&II

TCGA-MN-A4N5-01A -0.714744482 High 0 84 >=60 MALE 4 YES YES Stage I&II

TCGA-MP-A4SV-01A -0.881201752 Low 1 2620 >=60 MALE 2 NO NO Stage I&II

TCGA-MP-A4SW-01A -0.844541007 Low 1 1778 <60 MALE 3 NO NO Stage I&II

TCGA-MP-A4SY-01A -0.620582826 High 1 1501 >=60 MALE 4 NO NO Stage I&II

TCGA-MP-A4T4-01A -0.986387521 Low 1 2617 >=60 FEMALE 2 NO NO Stage I&II

TCGA-MP-A4T6-01A -0.902992127 Low 1 1790 >=60 FEMALE 3 NO NO Stage III&IV

TCGA-MP-A4T7-01A -0.20660731 High 1 167 >=60 FEMALE 4 NO NO Stage III&IV

TCGA-MP-A4T8-01A 0.135407172 High 1 161 >=60 MALE 3 NO NO Stage III&IV

TCGA-MP-A4T9-01A -1.029821076 Low 1 1265 <60 FEMALE 4 NO NO Stage III&IV

TCGA-MP-A4TA-01A 0.160143469 High 1 950 >=60 FEMALE 4 NO NO Stage I&II

TCGA-MP-A4TC-01A -0.484418915 High 1 74 >=60 MALE 3 NO NO Stage III&IV

TCGA-MP-A4TD-01A -0.427052869 High 1 307 >=60 MALE 3 NO NO Stage III&IV

TCGA-MP-A4TE-01A 0.219379873 High 1 896 <60 MALE 4 NO NO Stage I&II

TCGA-MP-A4TF-01A -0.193699101 High 1 336 <60 FEMALE 4 NO NO Stage I&II

TCGA-MP-A4TH-01A -1.095973415 Low 0 741 >=60 FEMALE 3 NO NO Stage I&II

TCGA-MP-A4TI-01A -0.932046517 Low 1 429 >=60 MALE 3 NO YES

Stage I&II

TCGA-MP-A4TJ-01A -1.150649519 Low 1 339 >=60 FEMALE 2 NO NO Stage I&II

TCGA-MP-A4TK-01A -0.834599574 Low 1 582 <60 FEMALE 4 NO NO Stage I&II

TCGA-MP-A5C7-01A -1.02775178 Low 0 2248 >=60 FEMALE 3 NO NO Stage I&II

TCGA-NJ-A4YF-01A 0.522087633 High 0 2161 <60 FEMALE 3 NO NO Stage I&II

TCGA-NJ-A4YG-01A -0.752870033 High 0 2261 >=60 MALE 4 Stage I&II

TCGA-NJ-A4YI-01A -0.645593749 High 1 4 >=60 FEMALE 3 Stage III&IV

TCGA-NJ-A4YP-01A -0.16153514 High 0 50 <60 MALE 5 NO NO Stage I&II

TCGA-NJ-A4YQ-01A -0.966409421 Low 0 1432 >=60 FEMALE 4 NO NO Stage I&II

TCGA-NJ-A55A-01A -1.35950745 Low 0 15 >=60 FEMALE 3 NO NO Stage I&II

TCGA-NJ-A55O-01A -0.435189534 High 0 13 <60 FEMALE 4 NO NO Stage I&II

TCGA-NJ-A55R-01A -0.85646083 Low 0 603 >=60 MALE 3 NO NO Stage I&II

TCGA-NJ-A7XG-01A -0.870096992 Low 0 617 <60 MALE 3 YES Stage III&IV

TCGA-O1-A52J-01A -1.093013542 Low 1 1798 >=60 FEMALE 3 NO NO Stage I&II

TCGA-S2-AA1A-01A -1.179582062 Low 0 513 >=60 FEMALE 3 NO NO Stage I&II

Table.S9. Drug sensitivity prediction

sample A.443654 A.770041 ABT.263 ABT.888 AG.014699 AICAR AKT.inhibitor.VIII AMG.706 AP.24534 AS601245 ATRA AUY922 Axitinib

AZ628 AZD.0530 AZD.2281 AZD6244 AZD6482 AZD7762 AZD8055
BAY.61.3606 Bexarotene BI.2536 BIBW2992 Bicalutamide BI.D1870 BIRB.0796
Bleomycin BMS.509744 BMS.536924 BMS.708163 BMS.754807 Bortezomib
Bosutinib Bryostatin.1 BX.795 Camptothecin CCT007093 CCT018159 CEP.701
CGP.082996 CGP.60474 CHIR.99021 CI.1040 Cisplatin CMK Cyclopamine
Cytarabine Dasatinib DMOG Docetaxel Doxorubicin EHT.1864 Elesclomol
Embelin Epothilone.B Erlotinib Etoposide FH535 FTI.277 GDC.0449 GDC0941
Gefitinib Gemcitabine GNF.2 GSK269962A GSK.650394 GW.441756
GW843682X Imatinib IPA.3 JNJ.26854165 JNK.9L JNK.Inhibitor.VIII JW.7.52.1
KIN001.135 KU.55933 Lapatinib Lenalidomide LFM.A13 Metformin
Methotrexate MG.132 Midostaurin Mitomycin.C MK.2206 MS.275 Nilotinib
NSC.87877 NU.7441 Nutlin.3a NVP.BEZ235 NVP.TAE684 Obatoclax.Mesylate
OSI.906 PAC.1 Paclitaxel Parthenolide Pazopanib PD.0325901 PD.0332991 PD.173074
PF.02341066 PF.4708671 PF.562271 PHA.665752 PLX4720 Pyrimethamine
QS11 Rapamycin RDEA119 RO.3306 Roscovitine Salubrinal SB.216763
SB590885 Shikonin SL.0101.1 Sorafenib S.Trityl.L.cysteine Sunitinib
Temsirolimus Thapsigargin Tipifarnib TW.37 Vinblastine Vinorelbine Vorinostat
VX.680 VX.702 WH.4.023 WO2009093972 WZ.1.84 X17.AAG X681640
XMD8.85 Z.LLNle.CHO ZM.447439

TCGA-05-4249-01A -0.892148543 4.601543512 2.5839811355.373989974
4.404038328 8.247961296 3.324584774 4.266504865 1.705799323
3.053913993 5.232907091 -2.391321327 3.252062198 4.600456886
4.598423824 4.925834351 3.841810181 3.799305199 0.53704989 0.37603396
2.654680124 5.340322021 -1.424800999 2.063869649 4.548036523
2.897147905 5.650931356 1.649267056 4.449465067 3.350026255
4.883639348 0.865888422 -3.724598733 3.31350909 -1.094133591
2.787858357 -3.70349636 5.587892628 3.872396145 0.879526358
4.694161527 -1.313172361 4.942341804 3.162025656 3.936853595
4.243022548 6.81542988 1.486394145 3.735719296 6.655784284 -
4.975079937 -1.900785992 4.556814488 -2.418232831 3.331391603 -
5.083517042 5.005788499 1.883272677 2.137331999 4.008942905 5.99649299
2.54691117 2.066936528 -2.971581804 4.610369663 3.936932974
3.742458344 4.363116632-1.776207231 5.077294415 6.296874453

2.944677102 -0.114624883 5.995830722 -0.718520051 6.067928416
5.154818168 4.5682111 5.488470131 6.41140242410.17859591 0.690356709
2.626264703 0.774668642 -0.975873633 3.269773849 0.701957291
4.306285488 6.782805214 3.599842494 4.734967149 -1.990453377
2.062994201 -0.42232056 3.252299685 3.585975244 -2.49166391 5.360838753
4.640309503 0.998729925 2.954336742 4.084021852 4.9177401154.74870327
3.2861102095.335264896 4.888892085 4.824238254 3.991409974
0.458924644 3.196996342 4.437918949 4.692514665 4.739310777
5.552545913 5.053667494 0.706623051 5.703897504 3.93471286 1.79122809
3.863127902 -0.431844741 -4.218276959 2.608891053 -0.380774217 -
3.48337006 -4.080793685 1.481699504 2.659995346 4.266395777 5.268545644
3.409723703 6.068324634 0.229096258 3.134047682 4.370048452
3.628791753 2.677280399
TCGA-05-4250-01A -1.255583265 4.629647615 2.027859944 5.53208393
4.009175158 8.234958924 3.3968611414.349913928 1.480686567
3.406127336 5.677892987 -2.653566393 3.706473668 4.596075195
4.677634832 4.82406852 3.177125887 3.628642856 0.274033067
0.275762709 2.361513544 4.693549335 -1.634126566 1.965097596
4.499613706 2.401773964 5.502889446 0.781682652 4.499178486
4.095357995 4.842447645 0.966984395 -3.832287753 3.186370071 -
0.849842784 1.958285252 -3.680720046 5.620633318 3.037321863 -
0.201526969 4.718140307 -1.142646618 4.801383551 2.170390827
3.879781674 4.1658511836.968162739 0.75327219 3.139082371 6.575779614
-5.154432623 -2.316369579 5.194370712 -2.393665594 3.076219858 -
5.042736313 4.915222948 1.498227298 2.310654387 3.519432618
5.847972374 2.423436984 2.251632931 -2.739980663 4.536298943
3.941367327 3.572700593 4.462512669 -2.203817116 5.088671905
6.130765491 2.712636261 -0.004847546 5.655520969 -0.973121302
6.065372289 4.970846207 4.615371813 5.524219354 6.233989551
10.8752564 0.728096604 2.6071295 0.45169887 -0.725994815 2.942969346
1.0115994234.384527051 6.761219862 3.313461704 4.798435728 -
2.4563911152.28850407 -0.558800024 3.28812353 3.624243731 -3.078268249
5.338455438 4.156644652 0.043410804 2.74165182 4.074153313

4.983893802 4.845304265 2.780339797 5.335550133 4.413482847
4.869124463 3.813931016 0.47393328 1.23253524 4.266800129 4.752848876
4.7598811845.447298782 4.632866209 0.607460459 5.27013441 3.928905829
1.720518499 3.785484323 -0.911395987 -4.6796532 2.371268879 -
0.451943452 -3.654059983 -3.365608619 1.45763351 2.46011156 4.137558834
5.01254622 3.326827507 5.762936406 -1.340370111 2.53853926 4.247546955
3.443220956 2.907680166
TCGA-05-4382-01A -0.609869442 4.614972654 2.672343774 5.279172179
4.209598996 8.293094341 3.388760227 4.254397925 1.697678878
3.403890176 5.571005648 -2.560328576 3.461479312 4.620756982
4.775283281 5.042846006 4.038375712 3.579985892 -0.035313991
0.475012077 2.707215034 5.199880499 -1.268805086 2.710097519
4.533556953 2.97703932 5.604392648 1.292640962 4.551977398
3.652310291 4.956100443 1.155470623 -3.673544698 3.272110815-
1.006295879 2.1759729 -3.271644103 5.5891178563.538693668 0.676790739
4.711517963-1.239211012 4.931632319 3.094501294 3.95757742 4.214617533
6.9511606671.285663546 3.4041102796.797545616 -4.968629808 -1.92739331
4.803432745 -2.91065763 3.354738275 -4.846763867 5.036670278
1.657157733 2.099337154 3.868597978 6.104649363 2.86107575
2.262827292 -2.153231115 4.639714072 3.936898987 3.837981923
4.247607791 -1.539461772 5.0911284926.349794394 2.794634597 -
0.129520597 5.772363482 -0.536373794 6.031359087 5.095449284
4.614456371 5.47374629 6.335323955 10.7040462 1.186937838 2.640519294
0.233914203 -0.743129596 3.38714019 0.961207761 4.286064331
6.872323835 3.623929722 4.770206986 -2.018728901 2.212733031 -
0.451242252 3.299513968 3.749911467-2.313017368 5.352201093 4.449274694
1.502731896 3.144207093 4.166018105 4.966620435 4.654707972
2.988659805 5.335488602 4.962369139 4.938496915 4.02503468
0.687476623 3.401448866 4.584634086 4.805750549 4.75040465
5.693698346 5.245895869 0.831550271 5.737497749 3.943783507
2.055165212 3.830147876 -0.676480711 -3.858900617 2.657713445 -
0.415128416 -3.652991304 -3.755591242 1.497166675 2.8114519784.306492938
5.073033203 3.382696657 5.994968078 -0.132244299 3.221104522

4.406156519 3.6113703582.892049981

TCGA-05-4384-01A -0.53218107 4.722805089 3.613922441 5.398918479 4.15516931 8.26094445 3.443865895 4.28678635 1.757955071 3.020903554 5.036298224 -2.455261746 3.175170722 4.441520705 4.621524483 4.965621033 3.597263093 4.066671799 0.467225999 0.38713981 2.6558117415.087407304 -1.284069593 2.072782322 4.547028227 3.248719459 5.756475416 2.023524521 4.4381973 3.144475249 4.8715513 0.755434274 -3.801891294 3.28397545 -1.49082538 2.892038158 -3.255829462 5.583043314 3.170682377 1.044913304 4.70473762 -1.246683303 4.985464607 3.244462578 4.047514532 4.247409777 6.915818599 1.534007539 3.789129283 6.518805647 -4.983526483 -1.866384464 4.637625938 -2.50596028 3.249284939 -5.104290215 4.99823723 1.820834551 2.089603492 3.909623684 6.002031634 2.695758039 2.025227324 -2.29501437 4.632757018 3.936560854 3.988883415 4.170600286 -1.67736255 5.083495604 6.30416414 2.995503731 -0.222158156 6.05350976 -0.6710311 6.0480178115.173106809 4.577630182 5.4911596776.385181449 10.26410188 0.687627559 2.460834294 0.582021282 -1.02802951 3.309443387 0.821435454 4.3113566236.871603166 3.732042418 4.559472758 -2.037107215 2.033425775 -0.388713832 3.233775971 3.715678673 -2.085228606 5.363139047 4.6711187680.936961514 2.692967268 4.188357095 4.976995939 4.737670503 3.259843796 5.335385799 5.1656025 4.800903674 4.080462142 0.543007987 3.212505542 4.668490901 4.769357402 4.7411021495.71628446 5.307444677 0.8511811665.727547185 4.327907982 1.988725908 3.882048446 -0.55622575 -4.055910833 2.7513048 -0.346521969 -3.498728133 -4.219081416 1.608527538 2.916990712 4.397979223 5.199393324 3.345866544 6.230368968 -0.816822979 3.067755679 4.463269236 3.6621292112.592696399

TCGA-05-4389-01A -0.848347951 4.987972752 2.91929389 5.442604355 4.392303918 8.256787475 3.4061141294.272841083 1.782389555 3.41536821 5.742260068 -2.209921341 3.655454831 4.658839929 4.985951014 5.207681722 3.984947686 4.217323345 -0.028700179 0.316938289 2.820268667 4.610981247 -1.359592228 2.854792344 4.550815406 2.940122761 5.7097435 2.446374486 4.600769196 4.392204128

4.956287796 1.10698362 -3.514379548 3.194967995 -1.084862258
2.284808598 -3.689609283 5.613577023 3.077003368 0.169034807
4.696923728 -1.165648324 5.313827414 3.166235492 3.875661246
4.324721552 6.995955557 1.007104155 3.753973872 6.635245693 -
5.231091606 -1.919887789 4.777201495 -2.600062997 3.416462776 -
4.762245777 5.020043347 1.809810246 1.791866759 3.873340782
6.086574356 2.810724097 2.701870116-1.933304052 4.52097103 3.941104129
4.180928648 4.174683754 -1.919970116 5.09606599 6.372973259
2.920722543 -0.127257155 5.903344075 -0.811739183 6.149965902
5.015410901 4.656268038 5.54958189 6.215694696 10.40188826
1.412953363 2.719035622 0.668525027 -0.937216901 3.167141035
0.944462023 4.373890039 6.8481186043.779209967 4.9721956 -2.223786567
2.671520429 -0.371292681 3.352364958 3.84369369 -2.455864511
5.363897444 4.058878319 1.024959433 2.965308599 3.982456703
5.045891793 4.590036764 3.30185116 5.335592574 5.0407110444.869220887
4.565716496 0.446380632 3.487146992 4.573277542 4.726619934
4.72873286 5.96072604 5.364060673 1.253147868 5.657053475 4.125824866
1.808963652 3.816636678 -1.244707605 -4.348005207 2.511745449-
0.364839243 -3.828402203 -3.674067543 1.639234793 2.901475481
4.5116188275.432717847 3.699997335 6.079677161 -0.934412586
2.585052229 4.542723353 3.72671649 2.748620328
TCGA-05-4390-01A -1.29380224 4.801946877 2.049932493 5.316132745
4.279468656 8.36114524 3.69495465 4.252624883 2.146244282 3.649948075
5.534974601 -1.981942436 4.031627565 4.641741686 4.881307568
4.852612322 3.657068759 4.486778732 0.921721633 0.613899095
2.701869026 5.372684779 -1.558448679 2.86248203 4.635817306
2.6571117165.776149072 2.762694652 4.653028068 4.694599387
4.792976434 1.381851534 -3.600701576 3.598743512 -0.639562662
2.658974633 -3.287779571 5.6027826113.1308768 0.934745128 4.711449415-
1.170040289 5.290822359 3.127264888 3.870298986 4.356343025
7.071843159 1.620590795 3.940289274 6.859825033 -4.588202925 -
1.74842666 4.711107571-2.068361175 3.629176826 -4.166789438 5.155909037
2.200982313 2.324777793 4.056281724 5.942864868 2.918812208

2.413795517	-1.793621158	4.581302666	3.937981504	4.529025137
4.331608762	-2.607589517	5.078829509	6.618851681	2.927478252
0.1186689885.717831555	-0.81336644 6.043371324	5.018778366	4.959816326
5.460179414	6.529127406	10.45867978	1.33205297 2.895838275	1.26085643
-0.698142268	3.294923376	0.767227875	4.32361747 6.895454869
3.628634061	5.012236142	-1.870428068	2.498063021	0.136079379
3.360061863	4.02446803 -2.832306756	5.340543717	4.43076245 1.12845358
3.3114075893.74968961 5.015066335	4.634338643	3.586598545	5.335581459
4.910389778	4.89873604 4.430903666	0.470382852	3.240413688
4.568936274	4.567802389	4.730894094	5.846316629	4.781306656
1.425851309	5.355613725	3.919635575	1.585706716	3.921692507	-
0.922652005	-3.422409215	3.390689832	-0.203283075	-3.355665589	-
2.9511336451.469963291	2.55273918 4.259858134	5.4437771193.4266641186.22701639
-0.112067449	2.317903852	4.578682176	3.754832982	2.736365458
TCGA-05-4396-01A -0.955436203	4.680576096	2.5765849115.476687429
4.3113734328.36124049 3.05449812 4.259805505	1.757956581	3.059619638
5.342510179	-2.418322214	3.17783631 4.728582522	4.739603157
5.159056494	3.776304048	3.454810319	0.12379098 0.452412085
2.681065413	4.974452937	-1.243137581	2.30452415 4.521269032
2.9006401135.423506515	2.432820729	4.364522892	2.74821519 4.637147411
0.584616196	-4.019770688	3.491690715	-1.251190355	2.284581112-
3.375824976	5.58996894 3.719891793	0.078581955	4.710647142	-1.306962332
4.976770779	3.273738381	4.057737352	4.330954313	7.200853
1.3453947114.033693605	6.453075556	-4.99855633 -2.006442066	4.698802915
-2.674125417	3.162264501	-4.776822296	4.998244172	1.662135146
2.13530905 4.043044427	6.0318273 2.833321013	2.361414284	-2.509222809
4.499121854	3.944608456	4.104054589	4.172504684	-1.002669788
5.070944292	6.229999901	3.054044737	-0.374234734	5.966749492	-
0.854089134	6.074018136	5.33747186 4.535044932	5.53317866 6.438904201
10.34041795	0.798473381	2.484758188	0.567180448	-1.102375484
3.032858167	0.802628106	4.326000147	6.804213329	3.89048912
4.812669814	-2.050522316	1.981493377	0.019053787	3.220816986
3.437266757	-1.928083057	5.366821819	4.452649909	1.100739818

2.877698724 4.332281963 4.936743974 4.75111237 2.908170374
5.335143902 5.131755662 4.7726119264.172553779 0.461798071
3.435655766 4.586636788 4.609821035 4.742608728 5.780477635
5.1170140430.714222534 5.990378299 4.671821328 1.953186449
3.932213795 -0.659362466 -3.536557353 2.793966379 -0.113542949 -
3.370312183 -3.890546038 1.51855062 2.894610863 4.450771822 5.448120753
3.514618686 6.157511486-0.640923138 2.809409354 4.536639715
3.612436327 2.263498003
TCGA-05-4397-01A -0.311785237 4.598312581 1.844480475 5.304207986
4.290960516 8.180891814 3.658482194 4.1181620022.021433549
3.814437326 5.049000208 -1.973662237 3.344519944 4.923422299
5.092392426 4.764264143 4.358730895 4.524981846 -0.795033002
0.221320634 3.055917925 5.320369699 -0.959291017 3.42347961 4.68879563
2.340419771 5.179664167 2.62635048 4.712306614 4.379338025
5.023747726 1.545407235 -3.603750387 2.715063648 -0.548483739
2.386085325 -4.390776419 5.594653647 2.882418143 0.444971824
4.69636739 -1.294421731 5.407256429 3.178664339 3.565981491
4.205044197 6.966056683 1.082802226 4.5711700657.453090249 -
5.48502749 -1.832902346 4.649285009 -2.986313981 4.251319196 -4.280937572
5.043552914 2.2011733463.900612893 4.045862551 5.946790583
2.3060551162.580183599 -1.597770358 4.638033475 3.936298731
4.709748997 4.102358982 -0.878892644 5.069485937 6.523017146
2.757898993 0.21885278 4.97499996 -0.709924418 6.026487044 4.868219809
4.960420643 5.461470541 6.479854824 10.03702481 0.331490324
3.098844142 1.569013174 -0.40998188 2.916959044 0.856948353
4.226495551 6.8495851113.244609069 5.166246654 -2.420895266 2.54297868
0.669982753 3.443266202 4.027982196 -1.262663765 5.318133077
4.344233794 0.946295552 3.387369077 3.38461837 5.028491005
4.750272587 3.812145383 5.335442082 4.4561991145.230235769
4.628912135 1.028239037 3.852494499 4.598972732 4.911632543
4.765790871 5.9400381155.147413636 1.0118931854.746141345 3.981453504
1.9508110533.872097315 -1.646123546 -2.560100673 3.397557192 -
0.638943797 -4.353105449 -2.677732666 1.1184571712.794146228 4.513126019

5.795354128 3.437076945 6.608689952 -0.790045431 1.903174686
4.7913611173.687832638 3.210953948
TCGA-05-4398-01A 0.022864615 4.958324489 2.958134551 5.45383647
4.169007374 8.185291575 3.29855234 4.328818518 1.618470642
3.492620508 5.642423467 -2.586407809 3.969138132 4.439699352
4.856624023 5.2311869883.743473741 3.808320301 0.422893978
0.500695014 2.631461884 5.013799924 -1.00810079 1.9142627 4.549253465
2.705716137 5.671328724 1.628299945 4.6211865484.1168957444.850741304
1.095537061 -3.631925621 3.26345755 -0.87811079 2.446690344 -2.797529999
5.61539407 3.126178895 0.567575852 4.702397163 -1.160009699
4.878385725 2.7830112744.045885591 4.176634506 6.916952712
1.516127817 3.700329775 7.071556973 -5.043173632 -2.09169686
5.117704281-2.201469266 3.148525122 -5.124618243 5.185886129
1.999656973 2.401395056 3.629550397 6.047614316 2.472266421
2.182197329 -2.180266614 4.696356983 3.934951415 3.853331216
4.627061553 -0.598017931 5.0911810346.283625573 2.737453314 -
0.193026157 5.724035059 -0.490553739 6.177097265 4.965161559
4.823157896 5.58626306 6.256320682 10.67244879 0.795588274
2.631797247 0.482351814 -0.542640292 3.514197386 1.061586731
4.385206924 6.829296182 3.455689036 5.038979519 -1.851619208
2.626591531 -0.590391032 3.27164734 3.731038892 -1.993304255
5.356346038 4.183540196 0.90061464 2.932825736 3.863665498
5.003547844 4.868338319 2.842974515 5.335467801 4.666628373
4.903897886 4.0431099 0.9114904032.140717539 4.555245396 4.876990429
4.740694066 5.528176549 4.849920929 0.820704525 5.58811789
3.880026686 2.043838934 3.875952972 -0.090377345 -4.315667877
2.361804715 -0.216403674 -3.653305875 -3.672119479 1.555374024
2.836269529 4.4551884115.220800808 3.179413382 6.190444532 -
0.628752033 2.953229607 4.438697513 3.544391435 3.144658508
TCGA-05-4402-01A -0.705034795 4.617219251 2.6049919 5.385888957
4.5102115478.287570332 3.350150167 4.228607446 1.701356461
3.323102859 5.575400344 -2.491765283 3.653972608 4.902503509
4.861445449 5.1877097 4.152622864 3.844581359 -0.018994222

0.508891234 2.817586833 5.071320498 -1.17790556 2.529318243
4.518343197 3.037797689 5.620296751 2.058041216 4.625451965
3.8178211914.985013577 1.267516553 -3.617457824 3.33234744 -1.108330662
2.688275135 -3.392434987 5.608177685 3.852946167 0.92559761
4.718929766 -1.142900609 5.21119142 3.084001673 3.936269752
4.221853852 7.182927324 1.339783197 3.663894525 6.633030585 -
4.940417036 -1.898981634 5.058953965 -2.791878016 3.32838428 -4.461600823
5.15010241 2.028940335 2.408919842 3.755507505 6.090406561
2.769691564 2.131101183-1.905735341 4.579009475 3.944552341 4.06660656
4.211407152-1.270796566 5.081955488 6.279030525 2.867559605
0.009618155 5.778028045 -0.530791941 6.056316363 5.137231682
4.689199643 5.479327344 6.247337237 10.56097323 1.102203714
2.773803907 0.755255743 -0.748655723 3.059124562 0.991181857
4.329980408 6.881685251 3.758852546 4.978403233 -1.947827971
2.426080603 -0.255755374 3.313903792 3.5500714 -2.319542494
5.361803005 4.048737999 1.022017089 3.050182802 4.150175446
4.9771193954.805219397 3.277925385 5.335362014 4.821732104
4.964055493 4.396152988 0.68884141 3.372088407 4.52169488 4.821026322
4.75603914 5.834084285 5.302098535 0.980071997 5.674765413
4.141645021 2.050727904 3.908305115-0.798592121 -3.865187848
2.893949333 0.076952143 -3.549809727 -3.686410135 1.7204036
2.798396808 4.638524326 5.295612934 3.698030451 6.232334508
0.083063828 2.99828186 4.741359422 3.714332523 3.163786333
TCGA-05-4403-01A -0.811347521 4.82146936 3.07632583 5.53373852 4.147818421
8.351618698 3.023829782 4.429155306 1.555422829 2.832585271
5.998576717 -2.716159413 4.255824123 4.200298589 4.547904606
5.374357074 3.333627536 3.676612238 0.478988775 0.605130405
2.527706545 4.597469773 -1.116343593 2.410188165 4.345785672
3.084612121 5.808816366 2.294502236 4.517261023 2.794233045
4.838815544 0.600785618 -3.717148557 3.522597116-1.529998237
2.682333119-2.569433403 5.596055222 3.309854531 0.383500308
4.735447672 -1.168765263 4.86877407 2.1147054364.1711722134.357927952
7.137945129 1.180167623 3.189188784 6.449965874 -4.612927272 -

2.1163012274.7122859 -2.553390765 2.987630797 -4.859695446 5.012817704
1.907189604 1.825589376 3.670575199 6.305086078 2.788329131
2.091453076 -2.49640449 4.567917087 3.933803172 3.85730045 4.275162723
-1.558805196 5.087260405 6.249574141 2.984745259 -0.367254618
6.326468616 -0.306781827 6.00371822 5.27520087 4.372276514 5.60173362
5.886735446 10.58820822 1.80753183 2.435919209 0.336292712 -
0.605046598 3.243212783 1.11774608 4.463325553 6.771343596 3.842097185
4.723825865 -2.248181169 1.911326274-0.720099357 3.218054992
3.422436863 -2.790828063 5.355397205 4.253080224 0.135774348
2.932433862 4.761344546 4.962298739 4.7115738062.426876041
5.335332931 5.275678125 4.777894091 4.300072256 0.552985874
1.3710148 4.597981935 4.7160848 4.760906999 5.867128879 5.294106723
0.875989894 5.829065607 4.5656011422.076620058 3.861329811-0.696642357
-4.491525704 2.161973982 0.073753007 -3.191396012 -3.092524824
2.39609166 2.994704564 5.023357471 4.879428365 3.218376438
5.797305912 -0.193330697 3.477320464 4.522401476 3.715990374
3.335881997
TCGA-05-4405-01A -0.403641869 4.660793391 2.526361111 5.483696222
4.2102113688.299353081 3.397821215 4.296341516 1.813150268
3.307436129 5.481138342-2.398018558 3.561817899 4.513337404
4.807383424 5.07414573 3.651999579 3.812673491 0.619920542
0.600150328 2.699442679 5.337201213 -1.101671037 2.461768968
4.579498277 3.194623075 5.563547854 1.617467944 4.553131622
3.43604577 4.942465567 1.153212149 -3.598487622 3.686055537 -
0.938567463 2.585708865 -3.075997151 5.585821298 3.755810307
1.392452438 4.704918714 -1.228422629 5.171266563 2.985138461
4.005064322 4.298403589 6.840384869 1.596071223 3.385768817
6.916568303 -4.759566617 -1.885458015 4.608607317 -2.497375067
3.367705447 -4.776931543 5.102439835 1.899160535 2.250028732
3.997494773 6.108576216 2.65685644 2.210169248 -2.221922484
4.693774588 3.936806875 3.958768376 4.369487335 -1.126608212
5.080853233 6.352070164 2.815096141 -0.079629839 5.944030302 -
0.44346139 6.087251691 5.097027989 4.6615286 5.543900435 6.458665253

10.58294296	0.821206184	2.727125431	0.71639678	-0.776971756
3.306830568	1.03513912	4.341087471	6.866362618	3.566456919
4.797633562	-1.766940254	2.271638207	-0.459260216	3.288360518
3.74620407	-1.932510812	5.347545159	4.734797199	1.0858110593.054467458
4.068770737	4.967907258	4.666709638	3.037214268	5.335446511
5.008472378	4.985941263	4.0600114030.784224284	3.290040126
4.651696504	4.786945214	4.736027692	5.605438028	5.13528341
0.845850508	5.817472693	4.149787708	2.032727133	3.84250003	-
0.227659824	-3.817786606	2.895225465	-0.308839881	-3.394501237	-
3.783251641	1.669203077	2.865684256	4.565966122	5.093567595
3.100413857	6.088306327	0.387789943	3.324758607	4.288218346
3.641791244	2.723463803
TCGA-05-4415-01A	-1.390377376	4.630737718	2.983345009	5.425453125
3.613910385	8.389664058	3.1673679114.251974619	1.940439365
3.883455828	5.534651951	-2.52056492 2.526489131	4.855685928
4.6211732294.25795003	3.74561964	3.845292956	0.137655362	0.069105349
2.420675655	4.760133691	-2.050924793	3.175130419	4.651549512
2.325416181	5.843022866	0.756004676	4.245175599	3.554246516
4.704927005	1.314767536	-4.20603924 3.085671259	-0.960680778
1.394390115-4.049752604	5.59965919	2.292728697	0.254855003	4.682551813
-1.459137589	5.190855948	2.886002536	3.640908647	4.128862061
6.616965021	0.196361539	3.323759741	6.673646331	-5.96427734 -
1.928419578	5.276536554	-3.05775937 3.595190966	-5.434279472	4.912527733
0.844328193	2.0780115883.483575387	5.659749009	2.1196802372.939794178
-2.587764789	4.579185539	3.946084175	3.867260276	4.059992173	-
3.225769564	5.084423172	6.302233034	2.696949408	-0.020805267
5.135105997	-1.723364262	6.176159028	4.8443113774.765520764
5.416567328	6.62317124	10.42383148	0.992599192	2.544776934
0.545924097	-0.558778903	2.799895278	1.219876665	4.219563401
6.8029568	3.094849672	4.732633162	-2.566094157	2.190282816	-
0.684901735	3.341242139	4.33214706	-2.892053467	5.3235011984.427565762	-
0.010836656	2.56518973	3.623500665	5.032086334	4.557356281	3.164331474
5.335569703	4.845590187	4.995370729	3.784598659	0.373182696

1.526421854 4.645496417 4.792306142 4.742591698 5.601483884
4.427422857 0.834430168 5.273808432 4.028874762 1.197384656
3.594207582 -1.439235765 -4.487046828 2.643499263 -1.380361502 -
4.26812159 -4.516164796 0.855859481 2.259583247 3.80855302 5.00492132
3.068669469 5.94142372 -2.098149485 2.2114391944.307055134 3.225598864
2.272190106
TCGA-05-4417-01A -0.935074084 4.300356826 2.312571221 5.487642191
4.088145102 8.432668164 3.505625657 4.333830534 1.722895363
3.566090736 5.682826759 -2.396231202 3.621656709 4.331134364
4.6211372895.089490193 3.602732321 4.042648783 0.687391731
0.531321526 2.593460209 5.20631618 -1.515042165 3.197197578
4.532982567 2.829735139 5.693212027 1.914879341 4.472084015
3.470458593 4.733350546 0.955645676 -3.813678392 4.05351054 -
0.948218048 2.32737228 -3.045711555 5.58732009 3.615387862 0.525202493
4.704257472 -1.287526383 5.492359802 2.929846502 4.031753302
4.36090219 6.874580625 1.415465262 3.137199373 6.597527023 -
4.888349576 -1.832805749 4.427371958 -2.447605198 3.295348247 -
4.670559619 4.910498708 2.00542819 2.049063369 3.976417913 6.222084091
2.96232353 2.784814133 -2.157196365 4.61061196 3.93547918 3.947567424
4.325754611-2.451574917 5.084889926 6.3911254072.858562502 -0.136112516
6.164664246 -0.631797448 5.952401943 5.231342038 4.537273717
5.571338703 6.42481131310.56957345 1.387438495 2.521423539 0.82833401
-0.854158936 3.687500523 0.921213267 4.32685948 6.802046105
3.789887714 4.719961307 -1.946228657 2.156137618 -0.400684576
3.317901347 3.842409835 -2.715267579 5.340646609 4.803595997
1.285513977 3.330777491 4.159025742 4.93281504 4.692426762
2.961685648 5.3354182115.261531744 4.9261135224.235901245 0.507404714
3.14166165 4.470792554 4.650254064 4.740534279 5.789816805
5.228714807 0.934615905 5.76250807 3.836559197 1.868796687
3.760482636 -0.16847589-4.052649795 3.061001998 -0.332033301 -
3.353808754 -3.315852809 1.675103548 2.742504447 4.523566543
4.822786445 3.24108858 5.854294339 -0.171291918 2.893695813
4.4116585753.673653718 2.828395642

TCGA-05-4418-01A -1.1488286 4.892777055 3.795846413 5.521748258
3.917153685 8.33012584 3.175390353 4.360700146 1.733748623
3.219184965 5.841785751 -2.543411292 3.7256591144.452548722
4.746318015 4.999318257 3.510285444 3.9010114870.007219409
0.367241297 2.535145306 4.561753004 -1.583814906 2.628984158
4.4924700112.816785037 5.838066107 2.637570043 4.464410195 3.62250267
4.718898403 0.772816828 -4.012987201 3.057004775 -1.458890446
2.071523248 -3.051917628 5.606968647 2.383891211-0.822404141
4.718949507 -1.214258101 4.960659486 2.333499572 4.020284249
4.215121299 7.122035189 0.561000858 3.347551839 6.513848206 -
5.533808241 -2.074143436 5.176821798 -2.882868967 3.261423912 -
5.187374928 4.98968365 1.501581045 1.856786426 3.54775192 5.994356863
2.607080364 2.516488461 -2.055564216 4.491408362 3.941894333
4.079787274 4.165877435 -2.802045506 5.093728355 6.23312252
2.911534183-0.283880678 5.793642675 -1.136157083 6.1751102625.058897617
4.615314018 5.555658474 6.205593073 10.36915394 1.743208104
2.4169187110.312553312 -0.529138232 2.985371451 1.225894269
4.416005185 6.837130031 3.601665244 4.671482154 -2.393773006
2.2431627 -0.630841558 3.256854754 3.97459814 -3.108260638 5.351074935
3.983935952 -0.367987754 2.5211730644.269622204 5.039590482
4.675139977 2.760676395 5.335503353 5.203377816 4.793637746
4.376628787 0.41881124 1.525198705 4.733263153 4.7477761154.735409262
5.764159131 4.904721078 0.94944521 5.49760531 4.416885688 1.513256343
3.807396827 -1.329751949 -4.532128813 2.269055231 -0.652617561 -
3.877399861 -3.678681457 1.68170351 2.808745893 4.592438406 5.120328372
3.2832011775.881042177 -2.07067269 2.844701859 4.638073363 3.553637209
2.928629785
TCGA-05-4420-01A -0.930660278 4.930066874 2.612441361 5.494379518
4.268383239 8.265400981 3.734255897 4.237244338 2.28963024
3.708717758 5.72340389 -2.013102811 3.880026834 4.837390422
5.080690942 4.903695759 3.970466423 4.478568239 -0.099451252
0.421591439 2.8311051095.331432245 -1.36639175 2.674788266 4.623764606
2.442396597 5.627144373 3.34803965 4.59528406 4.409057649 4.614903377

1.35623587 -3.633176213 3.099545527 -0.822578154 1.977865775 -
3.539461928 5.598664628 2.96234001 -0.344697661 4.704753936 -1.149174616
5.383731076 2.92274739 3.892737543 4.267454957 7.231262576
1.204616441 4.265842247 7.090192654 -5.437424873 -1.780684584
4.839340287 -2.571799442 3.856136474 -4.369567793 5.214489562
2.248381769 2.6876591113.964203141 5.988455639 2.699768029
2.547400288 -1.860631676 4.551672638 3.944542574 4.546704255
4.365199725 -1.908369076 5.072464256 6.373061004 2.901023182
0.143909735 5.745625722 -0.887331557 6.190697341 5.05263829
4.996441327 5.551500759 6.52343123 10.29562622 0.470822298
2.951820851 1.198040224 -0.563552593 3.157790819 0.879357627
4.373652414 6.898170029 3.642666408 5.194243871 -2.02080107
2.759338886 -0.016677741 3.342719851 3.986505174 -2.500264845
5.349591843 4.309615517 0.788582176 3.0138811844.1103099745.062691666
4.728439527 3.150814341 5.335394627 4.863363323 4.873592172
4.383047785 0.709370897 2.601995649 4.593089884 4.657043577
4.715257771 5.7041106164.82485491 1.395873601 5.559840001 3.95497552
1.583342061 3.940694155 -0.900595873 -3.59248374 3.125656674 -
0.319781482 -3.913600664 -3.212901768 1.249258637 2.590087879
4.382935817 5.655433992 3.487520318 6.377484144 -0.790099451
2.206255712 4.782066235 3.710270017 2.669892526
TCGA-05-4422-01A -0.693957315 4.975067149 3.500822479 5.351083602
4.002070603 8.22105696 3.36477263 4.290093596 1.883634844 3.087216957
5.183910751 -2.228232109 3.277025155 4.437880357 4.779075702
4.864310958 3.586562272 3.933142074 0.245696267 0.329443179
2.629273357 4.769924699 -1.442657463 1.856596336 4.560644301
2.967217349 5.791539668 2.503961839 4.442731749 3.591743166
4.658157004 0.663158742 -3.758125446 3.093139682 -1.261311488
2.335792279 -3.588693978 5.589888088 2.712002463 -0.037865021
4.702456229 -1.087884206 4.902350015 3.015495064 4.000162616
4.335553954 7.029293837 1.237187707 4.173717678 6.598583883 -
5.255267421 -1.974976202 4.413393452 -2.554715389 3.293538809 -
5.233390092 4.957867709 1.735779824 1.995920805 4.025310787

5.937993218 2.67710101 2.069755802 -2.426832459 4.540549073
3.9411326594.043747098 4.257261658 -2.034690053 5.083018184
6.393184485 3.023431243 -0.317933494 6.138301681 -0.747181452
6.122889475 5.098896609 4.614095573 5.54181361 6.312522857
9.797297286 0.907518227 2.500827728 0.511971062-1.070821084
3.030395378 0.780777545 4.33541933 6.88405334 3.7011663944.6977802 -
2.192903813 2.333793336 -0.339090005 3.2388394 3.778247037 -2.54437866
5.375100352 4.468395042 0.817968959 2.73260311 4.189717378
5.012086169 4.563443618 3.04316875 5.335415502 5.125230471
4.725898358 4.23394963 0.470499366 3.1191800444.621691929 4.665467953
4.737165293 5.793995227 5.12297091 1.022770718 5.718334717 4.35424169
1.675305819 3.906131421 -1.393273938 -4.31440055 2.451065404 -
0.510118355-3.688283181 -4.179162726 1.424530941 2.873341476 4.304350963
5.610501288 3.232955724 6.180639696 -1.481103183 2.897267878
4.522192936 3.752318354 2.161415916
TCGA-05-4424-01A -1.089931104 4.667573388 2.09704745 5.492297781
4.368042355 8.32644363 3.509443957 4.212779392 1.85913386 3.948467296
5.471830052 -2.091173257 3.2738950114.90241187 4.771785197 4.832145737
4.162699629 3.825607395 0.456028707 0.5141108992.8114880035.39639785 -
1.510096543 2.801262791 4.577541233 2.8817561115.623478321 1.476166897
4.651340047 4.322195437 4.92630617 1.326084637 -3.731071666
3.424770964 -0.785501613 2.771550601 -3.611123829 5.591385203
3.87037669 1.152308785 4.701543651 -1.202904947 5.084464497
3.482727384 3.8411635614.2311497456.944891648 1.339441859 3.426099266
6.85063911 -5.082883728 -1.770854544 4.684135765 -2.820822075
3.723110955-4.419099655 5.081602158 1.7321142842.230070577 3.923091474
6.024766726 2.857443448 2.461105953-1.763034857 4.600222469
3.935076728 4.017986516 4.281302947 -2.07825297 5.088000744
6.4135114422.776908378 0.12882116 5.841349365 -0.571645721 5.964024848
5.139836254 4.635877816 5.437074667 6.399563049 10.67302244
1.108254336 2.712638344 0.657713291 -0.600887655 3.400648187
0.85749108 4.253602624 6.868774827 3.660799106 4.845551513 -
1.955848343 2.246849229 0.023910549 3.354323401 3.773156955 -

2.421217646 5.338412713 4.650156353 1.533231305 3.2552545 4.244793945
4.9026051174.592885716 3.352623151 5.335365897 5.121458338
4.970751978 4.462752375 0.507539898 3.907477679 4.462399012
4.687928608 4.782084575 5.777285006 5.301774727 0.910851099
5.778757472 4.027159244 1.93923525 3.776591297 -0.769333875 -
3.86047574 3.371393936 -0.491882679 -3.687527664 -4.025196454 1.501916444
2.499166932 4.347643448 5.037489312 3.457177915 6.113254883
0.3131110432.975183794 4.459703463 3.7113173512.894271468
TCGA-05-4425-01A -0.847599275 4.719945722 2.523593643 5.44957228
4.236000575 8.291895733 3.276729628 4.313749403 1.64263435
3.366236082 5.569188279 -2.379234443 3.773925625 4.581464804
4.827454217 5.070487713 3.330223134 3.619471219 0.512485452
0.459864826 2.564091535 5.083417148 -1.413515757 2.495128055
4.502332422 2.894695321 5.701814587 2.317406847 4.52671399 3.53522111
4.852504782 0.872567872 -3.681063954 3.316543458 -0.977692874
2.679517853 -3.101792455 5.59668722 3.3641145440.673127406 4.722641516
-1.107362847 5.177349833 2.493784416 3.97779694 4.273019955
7.036381636 1.245734018 3.322287239 6.693551347 -4.840752539 -
1.9652594 4.718806614 -2.61605773 3.411696502-4.62998816 4.98107087 2.009006798
2.56413689 3.893098153 6.147094747 2.484507185 2.055405147 -
1.83057296 4.592171667 3.941925585 4.014085002 4.199790368 -2.153954184
5.085105818 6.298982639 2.877553107 -0.097553181 5.997723179 -
0.559993656 6.038934295 5.076674249 4.527738354 5.520832541
6.23381511510.67307053 1.153403789 2.7818119190.550137304 -0.56020457
3.120607445 1.1270840114.34424449 6.850476531 3.463376378 4.753117376-
2.210291688 2.186217452 -0.175733581 3.262385061 3.750912636 -
2.770608174 5.336855201 4.682559849 0.616566463 2.978235326
4.212889191 4.958417422 4.7761183462.819154983 5.335447092
4.946874999 4.967104061 4.336026547 0.507598313 2.165030796
4.535806213 4.770774126 4.778835427 5.621754771 4.946654385
0.886276844 5.580704659 4.669738544 1.902696128 3.851789966 -
0.881305791 -4.070408493 2.973640202 -0.058971031 -3.467986062 -
3.279385295 1.8351126422.810566565 4.644353563 5.088069246 3.072348872

5.940106668 -0.308467482 2.926437191 4.481223062 3.69380527
3.546642298
TCGA-05-4426-01A -0.85430911 4.614731882 2.83868883 5.416533198 4.147753228
8.184123706 3.413805925 4.283173233 1.793776095 3.127303907
5.156208002 -2.619898007 3.214624295 4.568863247 4.846881641
4.680559597 3.149764046 3.957922534 -0.014511893 0.287039196
2.519369553 5.209191203 -1.354404917 2.051313218 4.518338736
2.961013147 5.47277936 2.306042357 4.495734635 3.341886081
4.984429056 1.068734698 -3.788843582 3.058683867 -0.93957326
2.706227372 -3.433813946 5.609644653 3.448922867 0.813649138
4.727427363 -1.240590411 5.135615833 2.377125282 3.940862054
4.064530127 7.152267406 1.150766812 3.670409762 6.547115434-
4.856870062 -1.987243372 5.048940201 -2.629323218 3.303825574 -
4.703144385 5.055015071 1.847829995 3.12885139 3.749525642 5.909282895
2.214283111 1.723978938 -2.272797164 4.62282866 3.941366715 4.006890904
4.154945761 -1.600732826 5.088839826 6.32033258 2.841070057 -
0.04915038 5.59528578 -0.806362002 6.042778149 5.028641541 4.770607711
5.494064417 6.285488426 10.87595613 0.349542821 2.643338915
0.879527964 -0.626958584 2.648223351 0.954225849 4.368215773
6.864765314 3.295908857 4.611753928-2.439813875 2.058862366 -
0.09799551 3.293684423 3.744997033 -2.575441461 5.337045413 4.682909958
0.017742913 2.65167615 3.81550481 5.003064567 4.996784167 3.048038168
5.335488826 4.538094406 4.935668689 4.093698183 0.516529947
1.992735132 4.415850096 4.840022792 4.764582976 5.44868608
4.661841575 0.717943097 5.3711671944.384515071 2.007145223
3.902886057 -0.838849264 -3.76075546 3.059591146-0.075066312 -3.600046419
-3.429432047 1.521412147 2.806430722 4.304635034 5.206728963
3.145875664 6.061476598 -0.564763872 2.892780105 4.451852448
3.588459332 3.172399965
TCGA-05-4427-01A -1.103953934 4.332062042 2.19986186 5.403903523
4.366027524 8.287718428 3.568941347 4.279186442 1.540367302
3.332162055 5.121085255 -2.466078969 3.219063056 5.072057971
4.739369493 4.83783575 3.985676017 3.586352434 0.429762553

0.391049683 2.530683073 5.415954954 -1.551927722 2.528941768
4.620341642 2.488588072 5.381517989 0.282066564 4.401105802
3.569424056 4.780313682 1.016282724 -3.791052835 3.399951665 -
0.646816319 2.336679024 -4.02125166 5.604463444 4.394252502 0.997549777
4.685945433 -1.437888292 4.966544913 3.2237545 3.827835231 4.13395198
6.732043128 1.317616326 3.737039412 6.779108673 -4.944076399 -
1.93583294 5.088399559 -2.277298587 3.34429494 -5.060060905 4.957062484
1.439920523 2.632043572 3.834814486 5.84842769 2.597415236
2.357538549 -2.952385474 4.640754144 3.942378132 3.559376122
4.557765697 -1.491141426 5.075786035 6.144294012 2.773140033 -
0.023541231 5.580698616 -0.80563184 6.047955419 5.051678958 4.602238486
5.449413007 6.479739446 10.70563717 -0.444263463 2.902160937
0.708723007 -0.941312162 3.603816887 0.753553722 4.259464021
6.731895641 3.373730964 4.991276346 -1.879922124 2.180363226 -
0.249158081 3.255816424 3.518763308 -2.27909578 5.345033351 4.611681321
1.246994464 3.144409057 3.760617005 4.897935731 4.967270193
3.090207393 5.335276734 4.520699172 4.963634426 3.920846842
0.470660156 3.021312178 4.438251832 4.66803046 4.753383035
5.414366127 4.8411738640.608176456 5.708571765 4.057610784 1.87851996
3.77071289 -0.159360881 -4.019924824 2.762737956 -0.196714432 -
3.602688006 -4.332193841 1.334626418 2.42628486 3.963682901 5.127530496
3.530328134 6.088629328 -0.215492109 2.510004175 4.236417896
3.458866663 2.785456111
TCGA-05-4430-01A -0.78731651 4.517897576 2.565580668 5.546177948
4.132364725 8.321612182 3.3985114754.349639929 1.690909221
3.509618865 5.712453779 -2.402203259 3.783943552 4.275666013
4.701361502 4.9821103793.587050445 4.084554801 -0.167241151
0.428860638 2.703330829 5.280140566 -1.370575495 2.823882823
4.472710947 2.824226138 5.629698981 1.713880641 4.568858855
3.603623282 4.900473366 1.115329698-3.738096987 3.599763975 -
0.926599841 2.18852176 -3.504549675 5.587660532 3.194314174 -0.129299253
4.713535104 -1.19800265 5.333208134 2.582952325 3.977107782
4.262266677 6.936721441 1.151998895 3.134149127 6.790109544 -

5.270328314	-2.022928037	4.429491814	-2.850787651	3.43008003	-4.747658269
4.975666101	1.846208694	2.196861687	3.827895008	6.19055188
2.735403653	2.70191941	-2.276441977	4.615288484	3.932875336
4.006372039	4.245484528	-2.257084423	5.089437993	6.373287768
2.776379128	0.00494078	6.045643214	-0.647298388	5.974144433
5.1103442944.587244776	5.575034833	6.378509452	10.45828314
1.348749807	2.449570883	0.759683968	-0.601712133	3.405524219
0.969120459	4.356692067	6.839834583	3.588948236	4.849717117-
2.089979439	2.070753894	-0.435404322	3.323589244	3.848033354	-
2.669869128	5.342884918	4.6361103170.771537816	3.256329745	4.099673487
4.9811201964.6372853	2.914221261	5.335417801	4.948439948	4.975286873
4.286809945	0.622337589	2.542428436	4.569245549	4.7900778
4.746152368	5.695704158	5.149504727	0.939578667	5.768567473
3.864252266	1.960860869	3.815168714	-0.387788446	-4.345230198
2.934669641	-0.476877294	-3.778714932	-3.529386111	1.5327888
2.705244103	4.591820398	4.917352345	3.10440561	5.912800837	-
0.322156903	2.926197727	4.452030214	3.596847075	2.699654913
TCGA-05-4432-01A	-0.785507293	4.762810913	2.23181637	5.270481723
4.176689643	8.285953921	3.742835193	4.157854134	2.214395732
3.726080185	5.605499624	-2.066270576	3.590834791	5.051219453
5.08045453	4.848821254	4.346155929	4.246409764	0.3950214	0.465238971
3.028680958	5.426467247	-1.506024905	2.903945415	4.642174098
2.781964681	5.682427758	3.314491927	4.616886352	4.331354345
4.858312983	1.41620727	-3.546822666	3.308739418	-0.991229449
2.495714782	-3.830315197	5.579573365	3.391325815	0.385906396
4.707592833	-1.125324155	5.206653422	3.53233745	3.861077224
4.296646963	7.098270886	1.516922179	4.14861342	7.087972557	-
5.035397946	-1.590871741	4.606245335	-2.560430883	3.852331432	-
4.277108258	5.22952456	2.304528053	2.18613227	4.093237597	6.005683988
2.933784652	2.567745595	-1.615045714	4.556686392	3.940873461
4.516437423	4.373104805	-2.5670211	5.074870335	6.516577036
2.899436664	0.21554029	5.765860452	-0.622581495	6.11259319	5.089954937
4.960898493	5.493398421	6.53111289210.12179477	1.02021284	2.913672516

1.197707922 -0.666284106 3.525819786 0.757233058 4.293989003
6.920523845 3.657675953 4.97346973 -1.856541068 2.527892249
0.171852554 3.371925647 4.009849987 -2.672470597 5.356833333
4.414377805 1.49358681 3.284058961 4.059862736 5.0266832 4.524607865
3.604559891 5.335544142 5.066838101 4.956271469 4.491929494
0.634789526 4.368680646 4.645624674 4.725046899 4.718208819
5.803130714 5.058022086 1.244426401 5.482204234 3.900417006
1.628060425 3.981619611-0.780088011 -3.035472454 3.428354052 -
0.39362146 -3.746983009 -3.465989297 1.266551432 2.732018552 4.261066864
5.701669255 3.377861406 6.303857718 -0.025362711 2.739918978
4.735085693 3.818258381 2.720891652
TCGA-05-4433-01A -0.548297385 4.819560591 2.685146978 5.30037141 4.2960855
8.341654787 2.995307377 4.4030811531.515669534 2.981713143
5.651728897 -2.676810923 3.657832493 4.443705183 4.571748238
5.369621485 3.771739028 3.371286432 0.569944544 0.597687284
2.616027944 4.716818743 -1.058466162 2.415505741 4.398951126
3.134304036 5.794637532 1.751022746 4.484550781 2.631177988
4.884395572 0.5547822 -3.689973554 3.391074922 -1.373813368
2.667376255 -2.805893447 5.596897546 3.810691404 1.227129113
4.717719896 -1.170915038 4.6935585112.685725482 4.144996833
4.313200674 7.073109828 1.385794045 3.705553401 6.423288202 -
4.351104208-2.045396368 5.064055893 -2.395955806 2.970951695 -4.988215467
5.061418757 1.647003434 1.612813092 3.784614751 6.223432738
2.81281793 2.055843719 -2.463929001 4.6803511473.935656971 3.617893235
4.302499534 -0.817177485 5.0876265 6.177947743 2.976343419 -
0.403973987 6.142284984 -0.20249716 6.010609964 5.286772549 4.385504389
5.477946504 6.019857932 10.71662276 1.301396407 2.508945384
0.112207982-0.763622463 3.645530574 0.955388235 4.3116408416.747898862
3.8124971184.803793962 -1.964049645 1.896334638 -0.447704943
3.188128804 3.312034002 -2.247844246 5.365868219 4.332095605
0.866887245 3.152002744 4.57385051 4.921073502 4.798598707
2.597837881 5.335314613 5.142790702 4.778125703 3.90884586
0.664068357 2.22382317 4.57236855 4.762149931 4.77270112 5.76104932

5.372164603 0.652149601 5.922730359 4.388503489 2.157092754
3.887876289 -0.126256217 -4.264455876 2.202878818 0.055000869 -
3.134760468 -3.903497438 2.08514928 3.00991439 4.686909573 5.114108747
3.573999529 6.093826214 -0.014528075 3.359994024 4.46731116
3.695283607 3.426992312
TCGA-05-4434-01A -0.4492755 4.804848143 3.0113094415.42850115 3.930714874
8.171802864 3.595123349 4.291494385 1.658192354 3.41889557
5.360753829 -2.575284044 3.463205644 4.365628079 4.737551935
4.67549404 3.661548235 3.934172219 0.174605648 0.358674084
2.532869625 4.960955301 -1.347929109 2.161401475 4.507749821
2.768424196 5.739916305 1.500292753 4.476455545 3.433296462
4.833879604 0.921290443 -3.780133643 2.973669651 -1.144232718
2.326692579 -3.342379136 5.595754548 2.721222478 0.408838988
4.702400799 -1.187995136 4.899987165 2.571668984 3.989658121
4.20781615 6.89695639 1.087702473 3.405961278 6.801556987 -5.520328986
-2.071403703 4.813072018 -2.689807752 3.282607797 -5.145767528
5.00224214 1.785081773 2.433734096 3.842538501 5.979992129 2.338163
1.913301566 -2.201702708 4.679194086 3.931304255 3.793763958
4.368400644 -1.847573305 5.090798125 6.349385585 2.778650214 -
0.052575172 5.880316831 -0.554443145 6.030533325 5.000557015
4.512028739 5.525454715 6.12878766 10.5524751 0.6083711772.518676827
0.555716748 -0.518017499 3.317471388 1.05874701 4.290705208
6.845347185 3.346254638 4.729972549 -2.254521845 2.129196239 -
0.8131130773.243427307 3.844127148 -2.378845167 5.342871707 4.472849052
0.7504361132.97987797 4.002892485 4.940612683 4.748722068 2.746797478
5.335316508 4.66659519 4.917580995 4.038936291 0.730482528
1.824696447 4.504698716 4.824504704 4.77334897 5.581830259 4.82311189
0.847450782 5.421216836 4.208517094 1.954214539 3.748841377 -
0.717055877 -4.580119212 2.666157938 -0.561828373 -3.862823905 -
3.609373564 1.49996246 2.752385186 4.440171927 5.1177496553.0145634
6.013076541 -1.275122416 2.792008047 4.24688725 3.630661698
3.174865208
TCGA-05-5420-01A -1.017087121 4.633024623 3.364619027 5.559200713

3.990171534 8.412990753 3.485181629 4.42500394 1.705629673
3.680424152 5.845717129 -2.339734741 4.070924063 4.458910182
4.756813266 4.993284056 3.262770009 4.091636593 0.11823686
0.424140759 2.454562619 4.954470876 -1.695229331 3.062748177
4.5411880052.531490467 6.022780859 1.565078737 4.417395455
3.854293356 4.315881018 0.788402432 -4.099201498 3.254288981 -
1.029756782 1.887114988-3.265834639 5.587967701 2.09107842 -1.046871459
4.711232447-1.201103909 5.420048861 2.312691094 3.968797448
4.400779096 6.997810158 0.698999958 3.1177476466.675678764 -
5.495803536 -2.106633052 4.496564094 -2.673747863 3.839278426 -
4.820990974 4.754485462 1.666264793 2.347652074 4.0206119 6.165376
2.949044214 2.743327953 -3.106834893 4.465826617 3.941540935
4.045385059 4.261747666 -3.013381891 5.0930117576.203500523
2.883970366 -0.155426706 6.169666983 -1.000105485 6.022708821
5.187841053 4.233716933 5.558194478 6.320171777 10.4898926
1.531306436 2.462293132 0.654341784 -0.818452453 3.384210866
1.099986919 4.351564691 6.768961753 3.739243192 4.801351704 -
2.407367786 2.377215062 -0.34234756 3.30349657 4.070183161 -3.150369386
5.345001486 4.667685929 0.129644171 3.073950954 4.349658085
4.993754287 4.563018137 2.741542168 5.335249648 5.422590647
4.786509475 4.628585104 0.359597695 1.551315625 4.642206834
4.592106884 4.788938088 5.777184635 5.065454309 1.048149947
5.602522214 4.3626116981.657124907 3.697192098 -1.215929392 -
4.464057608 2.73924513 -0.62180104 -3.828793072 -2.823360585 1.596328372
2.494231814 4.550746564 4.951934275 3.412532939 5.740958723 -
1.841222715 2.446521224 4.729834386 3.602828687 3.132906713
TCGA-05-5423-01A -0.594458299 4.602166752 3.271564737 5.664883975
4.315507648 8.282955344 3.403561032 4.378482855 1.747176567
3.462932751 5.404645072 -2.3197002 4.247787063 4.584880725
4.866584736 5.0397116553.140464726 4.180631507 0.34986862 0.464474749
2.641491801 5.194317307 -1.177258349 2.463761618 4.517384401
2.936294334 5.707083354 2.470006297 4.497295906 3.793776791
4.577924932 1.033377297 -3.947497349 3.450609235 -0.9665354

2.837709757 -3.146296332 5.591657072 2.564551203 0.378271318
4.724976404 -1.193988421 5.507602858 2.556421954 3.970253774
4.313158074 7.106027257 1.210810748 3.4119109686.61271945 -5.121287696
-2.010710545 4.390369219 -2.590616155 3.630146906 -4.286834492
4.834884276 2.032937904 3.203086102 3.97246791 6.146643376
2.837387187 2.436133106 -2.426138894 4.457247417 3.945077131
4.32947798 4.323719018 -1.376807159 5.081868233 6.189536354 2.88466238
0.019512355 6.108204598 -0.614595166 5.964020502 5.182527069
4.380866801 5.566580156 6.357932922 10.56316839 1.243178192
2.58638822 1.037111366-0.770161515 3.240615244 1.076976829 4.417967477
6.81819646 3.770656261 4.856886348 -2.310077367 2.409050699
0.140459953 3.330067336 3.787879352 -2.331288563 5.349901422
4.7511892690.188872531 2.982532341 4.384583405 4.995561208
4.710972072 3.026324441 5.335193636 5.296398859 4.898461741
4.781822404 0.54538996 2.051426386 4.720857861 4.687248934
4.786646419 5.78186772 5.171713035 0.954419692 5.602440172
4.521921816 2.1180122843.875829485 -0.895926955 -3.614211586
3.208460396 0.014226555 -3.588844222 -2.694774901 1.782398889
2.781408244 4.770027659 5.153846798 3.194560552 5.927408913 -
0.775595506 2.890578626 4.784724214 3.589937346 3.448389393
TCGA-05-5425-01A -0.759045844 4.925628321 3.257968937 5.516220642
4.063591041 8.330234372 3.1103157294.333801435 1.675910155 3.33160056
5.856171625 -2.361977656 3.897875293 4.455980023 4.807698178
5.1911530833.987558802 3.827599565 0.085679074 0.506022099 2.64694307
4.845143121 -1.423082469 2.55150184 4.501414792 2.770042421
5.785632291 2.39018411 4.484899397 3.743767787 4.672485567
0.960649326 -3.909023968 3.19835564 -1.148037694 2.181224061 -
2.897440434 5.594762951 2.534252922 -0.214884954 4.7067805 -1.186571883
4.943856751 2.849531323 4.005473931 4.274478937 7.0411929531.17969676
3.424375598 6.802167055 -5.264482588 -2.204219274 4.859716124 -
2.760024978 3.351169476-4.924118951 5.023592263 1.77776971 2.028700595
3.840838945 6.120700745 2.752796051 2.464915078 -2.189026068
4.570544488 3.935273248 4.056041809 4.219350433 -2.131161077

5.085031469 6.298147696 2.84005596 -0.211505978 5.909648296 -
0.819267794 6.129889888 5.122073585 4.560058864 5.536638274 6.28028275
10.26520972 1.556490552 2.483220923 0.317452468 -0.628040308
3.271770033 1.035510523 4.380057652 6.852004356 3.681282235
4.962070068 -2.063604433 2.227924105 -0.439846897 3.273621316
3.878146383 -2.681553487 5.353682956 4.17490677 0.738217725
3.090396034 4.517741291 4.9636115054.565903656 2.719512446
5.335242054 5.067873709 4.77617019 4.163760029 0.631326921
2.543279738 4.624859249 4.729376457 4.743399224 5.70707422 5.32662256
1.008404031 5.721542352 4.193435641 1.723282613 3.787697157 -
0.777329445 -4.237657473 2.242254869 -0.605631346 -3.778200381 -
3.36221439 1.69211101 2.713534214 4.6434985 5.148018812 3.326815447
5.973085762 -0.9720633 3.0532117054.556848183 3.604992363 3.176461193
TCGA-05-5428-01A -1.264292913 4.548683235 2.830699835 5.730635819
4.473961385 8.312914997 3.372926409 4.350867177 1.814387257
3.685774656 5.820644497 -2.024755797 4.060080877 4.883693635
4.781785216 5.131578362 3.228325942 4.2382711540.0469391170.427048521
2.452349613 5.236218092 -1.644902628 2.34285273 4.5821081 2.524000497
5.433616787 1.2471149684.447459649 4.152969467 4.517965079
1.283257922 -4.205841902 3.06697856 -0.720695205 1.964566554 -
3.255631732 5.621585921 2.391374912 -0.028217297 4.705490867 -
1.315750721 5.15806039 2.56754918 3.801899743 4.244559199 7.039417765
0.522214486 2.967697772 6.90037422 -5.561296693 -1.841438347
5.242974806 -2.654395289 3.791547921 -4.097695519 4.841157647
1.585326197 3.335493867 3.755214482 5.90715295 2.663727877
2.457777584 -2.024729615 4.319830021 3.94937338 4.234890389
4.167759777 -2.144945016 5.070280407 6.130460968 2.758878955
0.164903977 5.764296623 -1.294865552 6.068577366 5.013545966
4.442920259 5.509671887 6.38954987 10.87020031 1.237350163
2.753072825 0.672906917 -0.826303989 2.5283161110.960152743
4.412806023 6.840319414 3.624152856 5.015125232 -2.47303448 2.53043218
-0.030590148 3.383876132 3.900350969 -2.707387004 5.330748417
4.371706553 -0.364965551 2.682803426 4.013932014 4.963941247

4.779723402 3.124008593 5.334948191 4.818035918 4.780058175
4.889614777 0.224888775 1.107525809 4.477935783 4.496919783
4.769774892 5.644597601 4.812898514 1.221642244 5.521078581
4.204089477 1.800503357 3.706114169-0.999441634 -4.499312164
3.087650259 -0.23124039 -3.752891359 -2.762457625 1.646816469 2.16389787
4.325910003 4.92085481 3.476366657 5.823244552 -2.219719677
2.233613149 4.682041356 3.52720644 2.987039268
TCGA-05-5429-01A -1.546308503 4.7336867112.272712428 5.604108158
4.1611460538.338448445 2.98885495 4.4096674 1.524302233 3.090305801
6.057610288 -2.520299553 4.05791615 4.430324316 4.365465246
5.267298389 3.407789991 3.399067546 0.466236352 0.365534382
2.441274153 4.830542149 -1.530483744 1.664194559 4.429148666
2.403672938 5.688893907 1.669895225 4.443040439 3.171264016
4.562142329 0.785230228 -4.216913709 3.359508641 -1.119912093
2.287980194 -3.017533917 5.617991228 2.932078467 -0.562492094
4.721947713 -1.202373147 4.577219752 2.097310994 4.013838362
4.40893769 7.12704266 0.759651561 2.752609418 6.419859865 -5.101602898
-2.161183663 5.092255286 -2.467073348 2.993404928 -4.722930286
4.871088273 1.625736883 2.010576597 3.637522767 6.054937783
2.429768256 1.867228212 -2.818377296 4.326051003 3.93870161
3.868810149 4.269702302 -2.065643362 5.073536964 6.194806295
3.010255288 -0.286507219 6.092995785 -1.194920611 6.09068185 5.22723896
4.277074048 5.565659176 5.922228213 10.6532834 1.986403061
2.442873639 0.34512797 -0.64989535 2.298916201 0.964897229 4.446224489
6.739071295 3.672198129 4.854718004 -2.632925661 2.002120461 -
0.702915525 3.244532466 3.329196233 -3.134985195 5.342577446
3.958098207 -0.259369849 2.909922593 4.554548842 4.910285013
4.776266056 2.368309027 5.334997838 5.107420291 4.699967465
4.476014386 0.131909041 0.571284569 4.241091645 4.560719138
4.767042955 5.820415723 4.98023574 0.799844883 5.573184612
4.502535767 1.650899058 3.811191072-1.287065568 -4.452339473
2.295014124 0.14033218 -3.36835195 -3.117730216 1.849690357 2.379656133
4.587229109 4.950173244 3.390496225 5.548751381 -1.025460912

2.889907673	4.521514929	3.616401861	3.157999282
TCGA-05-5715-01A -0.520538333	4.790395056	2.430935806	5.468072089
4.488988345	8.280069788	3.256394728	4.266221343	1.922564859
3.241972884	5.504289914	-2.17096419 4.033410574	4.534236882
4.690326427	5.377601743	3.690286514	3.994648089	0.720822602
0.626483635	2.722652664	4.903563931	-1.036199629	2.436741068
4.492800304	3.249091305	5.582077918	3.582935941	4.568214881
3.61757525 4.972103502	1.113338358-3.593541227	3.66612761 -1.123493016
2.950895577	-2.838096028	5.606615202	3.508404904	0.872505295
4.714924831	-1.192126152	5.36002929 2.904258835	4.04416704 4.438118813
7.09332042 1.555343426	3.473015367	6.843173524	-4.579034957	-
1.923581978	4.759221522	-2.44824735 3.354955671	-4.37398354 5.034508068
2.1899411592.348797235	3.995359638	6.180705215	2.932318051
2.065685813	-2.013101601	4.517383756	3.935496024	4.134713216
4.291031536	-1.034787352	5.080369825	6.271492105	2.918783365	-
0.081382208	5.941052512	-0.387495478	6.061593651	5.193771612
4.621085347	5.497032461	6.232603864	10.5952457 1.690526059
2.663053634	0.883578411-0.850237059	3.389880173	1.071096329
4.3822114326.794785269	3.885596523	4.846260796	-1.945269764
2.416151561	-0.161565435	3.3086756113.604496971	-2.167543413
5.365126589	4.323543455	0.934185243	2.973663399	4.114694086
4.997993562	4.650026865	3.14828948 5.335376813	5.104801084
4.922482281	4.459158518	0.568240747	3.35597302 4.667617642
4.690020483	4.746007171	5.859769501	5.393359735	0.999336295
5.6685531144.353931438	2.078495433	3.948051887	-0.864554808	-
3.648722045	2.928916771	0.052657402	-3.241097929	-2.837736323
2.057496414	2.906567303	4.750640753	5.2090117083.653313896
5.940446504	0.255600705	3.233980874	4.594777147	3.768649237
2.983510548
TCGA-35-3615-01A -1.345528068	4.64000889 3.666348668	5.688289067
4.202385069	8.32521699 3.301648785	4.3868752111.839772714	2.919998666
5.522316139	-2.192948755	3.528701416	4.345441416	4.524281618
5.074847624	3.226603562	4.274422762	0.271066768	0.317524432

2.471334092 4.848255765 -1.782584509 2.048991258 4.540622495
3.075955574 5.810955267 3.297413636 4.366956957 3.346939134
4.765298803 0.828854446 -4.115994329 3.720543455 -1.247926712
2.664545796 -3.098448295 5.60300567 2.546721032 0.061607208
4.704065263 -1.337970786 5.18548821 2.764940375 4.004520067 4.23293748
6.896308654 1.13272458 3.454532142 6.504590267 -5.194797002 -
1.921565221 4.512949558 -2.57597372 3.333834258 -5.038174026 4.855581565
1.757678057 2.366111516 3.79352938 5.997658735 2.57065189 2.301981388 -
2.676497129 4.440654303 3.941316374 4.235516661 3.999892598 -
3.009630747 5.080335779 6.284453305 2.989541433 -0.22792806 6.143600359
-1.381808506 6.13256467 5.134141223 4.559483282 5.566890592
6.336338781 10.2428765 1.251243529 2.380048894 0.689999627 -
0.977754143 2.477070283 0.886319109 4.461094255 6.87382954 3.765567514
4.640408826 -2.386699455 2.165124812 -0.450442373 3.294102655
3.86629453 -3.038925797 5.347444559 4.476272089 -0.086557207
2.515038191 3.991061403 5.011238883 4.675289311 3.223979981 5.33534534
5.205610032 4.775610146 4.44481862 0.160612482 2.428800571
4.612077676 4.627462758 4.718406445 5.822778648 5.281928278
1.07891993 5.708189993 4.341512533 1.470208703 3.826580567 -
1.24464145 -4.589265707 2.542730505 -0.420919986 -3.607130305 -3.805573916
1.695108329 2.622572249 4.431342172 5.012060025 3.190267148
5.934000991 -1.243712768 3.080998946 4.561299663 3.568127621
2.388231145
TCGA-35-4122-01A -1.011403547 4.54970949 2.971348087 5.69097892 4.034956375
8.359744587 3.374706987 4.504344207 1.472837139 3.831490661
6.150343088 -2.458174814 4.462852212 4.652902967 4.920249126
4.993963652 3.250440946 3.916942966 -0.025531967 0.424026058
2.425802079 4.80154121 -1.547656457 3.183199389 4.436244754
2.396339785 5.804087604 1.424827086 4.566456608 4.344855053
4.506672686 1.282774942 -3.901856397 3.522255003 -0.767563681
1.488896521 -3.601832592 5.616456077 2.288676281 -0.783421635
4.724445243 -1.182214302 5.271030933 1.968046226 3.859881274
4.275653514 6.969489203 0.284744613 2.731475768 6.698586113-

5.578872901 -2.174218354 4.931134037-2.684873781 3.52718304 -4.3292269
4.822888588 1.6110115342.677337181 3.556130622 6.169963357
2.795883033 2.985509733 -2.516216729 4.459354175 3.943344683
3.902916935 4.475942933 -2.576580409 5.095187106 6.149558277
2.63039228 0.078228334 6.066325855 -0.91693431 6.026053175 5.037167048
4.395564965 5.588774686 6.230839013 10.92814547 1.626449841
2.670600963 0.587540663 -0.509224004 3.189183605 1.235994772
4.45504235 6.796695458 3.59927161 5.012784185 -2.588391328 2.582283874
-0.294009096 3.399895975 3.935155482 -3.093535412 5.332376745
4.184053325 -0.262317209 3.095547179 4.266104375 5.002024452
4.68052009 2.582754639 5.335394898 4.930163276 4.895130993
4.662616331 0.492298262 0.601966221 4.428377661 4.685618015
4.775747455 5.6691391 4.912710516 1.09177954 5.510640697 4.169020738
1.938258475 3.671276853 -0.789833139 -4.609009117 2.925057846 -
0.485527248 -3.881945858 -2.256516974 1.656868593 2.423415506
4.563250566 4.663804375 3.098097233 5.682352122 -1.904954467
2.426186221 4.602899714 3.55493612 3.276141279
TCGA-35-4123-01A -0.837885587 4.585863771 2.502527905 5.546065559
4.0728431128.309107292 3.626827012 4.380586742 1.541623979
3.633869149 5.783215726 -2.642236834 4.300545812 4.670248535
5.035425258 4.798551806 3.139220947 3.95699012 0.149370556
0.408370534 2.395064267 5.025076317 -1.484980525 2.752053641
4.508959788 2.460309131 5.624026254 0.764445747 4.525222997
4.268019666 4.634358944 1.037959449 -3.788670775 3.402677771 -
0.691813188 1.769253405 -3.684050374 5.612804132 2.633075758
0.060421841 4.720364336 -1.178252366 5.275444903 2.015295514
3.842302168 4.195078556 6.926488987 0.685684564 2.831262862
6.861848816 -5.324491491 -2.211437241 5.231588134 -2.517279449
3.459734256 -4.544721255 4.935974305 1.74447444 2.73976714 3.595471056
5.992808744 2.663421395 2.601777608 -2.542766826 4.540376233
3.943436405 3.823235935 4.530130758 -2.213734802 5.088466498
6.143793785 2.658195272 0.182819676 5.766607226 -0.938404607
6.049203722 4.943660077 4.543626162 5.524390814 6.352294563

11.122434021.00129185 2.888555854 0.745603953 -0.610915618 3.324281881
1.182386252 4.401279476 6.819253495 3.368219598 5.025686766 -
2.324440757 2.533924177 -0.400613642 3.306134822 3.910046351 -
2.780474867 5.329525028 4.300092969 -0.10959947 3.013853976 3.831001104
5.0105111234.854214826 2.732356486 5.335401351 4.568333523
5.003617519 4.294197129 0.550701278 0.8892711194.458539652
4.7891167534.780138198 5.496966037 4.389398521 0.962647646
5.415321293 4.263644401 1.983334953 3.774403939 -0.617238124 -
4.517695934 2.810235708 -0.196382126 -3.752885639 -2.632670801
1.471961457 2.484226173 4.378813122 4.952369817 3.140186986
5.807060678 -1.269192398 2.328908568 4.465640701 3.470122793
3.337499866

TCGA-35-5375-01A -1.639468984 4.588092632 1.626645734 5.605290852
4.447890222 8.345819737 3.945755266 4.181303984 2.555171111
4.644546894 5.38509518 -1.502110578 3.664274895 4.912860608 4.83872009
4.670565156 3.867820384 4.896793224 0.367885425 0.338312607
2.894627528 5.645965096 -1.795053903 2.969681055 4.72426072
2.1116920175.486799585 3.871537922 4.7251146285.236215701 4.609806925
1.957587031 -4.136192989 3.072853892 -0.505665715 2.262151406 -
3.978692917 5.612858845 2.438748479 -0.021916172 4.704881637 -
1.326986983 5.567158315 2.7001142223.533168019 4.249537369 7.197849045
0.8511081753.488974832 7.278708126 -5.545779671 -1.272857948
4.505402428 -2.85498398 4.557591319 -3.573976458 4.870321047
2.0376841174.237367145 3.937670922 5.84249431 2.739764767 2.040300998
-0.959895539 4.298764089 3.934715727 4.789456873 4.08902675 -
2.452243752 5.078066848 6.605946479 2.774551889 0.457098878
5.296288958 -1.233868727 5.954357875 5.053536546 4.685121933
5.354080209 6.438459422 10.42458091 1.627552798 2.913903224
1.606756963 -0.361031439 2.341557084 0.7194110274.3711463366.886893223
3.579392033 4.909382975 -2.646107269 2.537830973 0.518212247
3.562951367 4.474057468 -3.023597583 5.293356434 4.83660144
0.040196771 3.065802896 3.856686601 4.902488537 4.580659514
3.688348322 5.33501413 4.797456895 4.932879051 5.502706932

0.169479482 2.51732268 4.271409245 4.4286511914.81320303 5.762547305
4.998151947 1.550938759 4.899406514 3.591058138 1.559893341
3.745612725 -1.772375768 -3.801589018 3.9309085 -0.844996869 -
4.073700016 -1.830122794 1.422634569 1.904761583 3.966439947
5.280630045 3.509474392 5.828014072 -1.048716585 1.975148999
4.830142734 3.604189192 2.849612529
TCGA-38-4625-01A -0.833401581 4.683738153 2.407474645 5.730368732
4.198966286 8.1865848113.236836219 4.356917875 1.406760491
3.713347916 5.780199646 -2.302395172 3.893782093 4.438761477
4.700437154 4.968662363 3.854737739 3.965378275 -0.38043247
0.368668333 2.461762721 4.91665283 -1.530618882 2.883454319
4.539032105 2.410885667 5.456386391 1.017018302 4.506643861
4.469285098 4.872496695 1.423349878 -3.701584556 3.187806835 -
0.628515388 1.961041519 -3.886544298 5.631608169 2.77549357 -0.28572844
4.690021243 -1.30743837 4.9395829112.569813604 3.757218163 4.190426291
6.64805443 0.764012697 3.320797899 7.046942438 -5.659054149 -
2.20626671 5.216543329 -2.397415061 3.347859856 -5.0943188 4.881721505
1.5933851112.526293007 3.512955237 5.975204837 2.167286598 2.93968884
-2.67285082 4.666714287 3.931390941 3.717988237 4.394712467 -
2.5112193415.092438595 6.30950389 2.603695024 -0.004085244 5.407088985 -
1.1166710396.1959154 4.842549582 4.613126158 5.554346943 6.254439051
11.035655 0.695236943 2.863120506 0.736182178 -0.435487434
3.002586248 1.152685532 4.404834772 6.776582296 3.334285918
5.125768855 -2.296277587 2.467148709 -0.640497728 3.359412717
3.946027117-2.592871986 5.334567705 3.91622239 0.2866846 2.916192371
3.572068023 5.009040531 4.900582447 2.641539429 5.335537023
4.4507968 4.92342329 4.124981904 0.72177194 1.578767044 4.442298979
4.761453652 4.740397909 5.623395008 4.769479451 1.00164225
4.987883845 3.871508969 1.620014318 3.613049858 -0.686474638 -
4.734714875 2.356089292 -0.547380947 -4.216868122 -3.375278776
1.369804299 2.518203895 4.649136109 4.830013576 3.07547567
6.025617529 -1.152394822 2.152251732 4.215265712 3.530119042
3.129510214

TCGA-38-4626-01A -0.319001835 4.581272777 3.204640724 5.456346314
4.139103334 8.395693157 3.297591979 4.381033838 1.576536648
3.542713776 5.401394314 -2.361349459 3.687950037 4.174818873
4.666857174 5.184056184 3.414392086 3.749893256 0.593690267
0.573266602 2.669212168 5.32659675 -1.130839224 3.189193983
4.483525148 3.242725678 5.785062568 2.386068004 4.512001985
2.929823654 4.9117221040.965153142 -3.645240589 3.871805915 -
1.105930588 2.589283672 -2.784409086 5.583164706 3.585395588
1.251059598 4.718243906 -1.27441309 5.218813814 2.744391862
4.103333683 4.304000669 6.846194904 1.494853066 3.097346306
6.733679331 -4.68302975 -1.802420442 4.483448403 -2.642133351
3.391882656 -4.464099931 4.968002498 1.862527269 2.389211395
3.926170733 6.319272888 2.81218782 2.488840554 -1.767169335
4.747478549 3.92986093 4.029597101 4.15034274 -1.567891341 5.098133233
6.500355914 2.818857816 -0.225865915 6.171227086 -0.265924589
5.944986173 5.205737599 4.429940539 5.5119846956.290560017
10.84814574 1.277936137 2.580048985 0.461611961-0.580932594
3.705576106 1.084848413 4.333597636 6.842221271 3.639067251
4.526324665 -2.023273575 1.909574756 -0.142077286 3.274253077
3.862315672 -2.121102472 5.33634672 5.102690839 1.056843932 3.11256357
4.202997441 4.966805938 4.732875151 2.736388597 5.335529839
5.403260622 4.967587139 4.27582457 0.672464742 2.542912937
4.717739103 4.824986831 4.780786588 5.6921157055.45457975 0.963296859
5.73487794 4.442051025 2.346152366 3.749148556 -0.239432582 -3.7380396
3.053550435 -0.406680651 -3.370424462 -3.182765134 2.027439856
3.042701243 4.782075692 4.584721845 2.995184369 5.930526871 -
0.174146823 3.286847103 4.283645893 3.68924594 3.409832384
TCGA-38-4627-01A -1.104786391 4.052141984 2.498288429 5.620012424
3.798865323 8.451532263 3.553394991 4.369066018 1.551766694
3.783317683 5.692946341 -2.498367049 3.649623724 4.066130579
4.537157996 5.160398633 3.379637803 4.146894888 0.2124077
0.415451586 2.666854761 5.257648662 -1.558174762 3.433724176
4.460705628 2.798829453 5.552541047 2.3113001754.542966041

3.160217558	4.849229419	1.283393001	-4.007606056	4.122301316	-
1.066244824	2.255011223-3.182347584	5.5877567113.250050316	0.270992454
4.735798253	-1.273181016	5.35840666 2.449385741	3.947510308
4.256740336	7.0052229111.174216018	2.360804104	6.726817988	-
5.063774559	-1.832094493	4.31837442 -3.007064633	3.313273012	-4.129687739
4.784126002	2.078795142	2.703900096	3.773513141	6.291021208
2.81423439 2.855061779	-1.820632249	4.631798987	3.93694604 4.264795073
4.091256767	-2.650097029	5.098240396	6.491602199	2.713456277
0.006666855	6.08673187 -0.800182811	5.814991712	5.217910874
4.434362194	5.550564039	6.31115613211.146104011.742093021	2.309036741
0.79494944 -0.498541665	3.312481099	1.041901235	4.366257244
6.839179426	3.690701528	4.543551556	-2.402544266	1.789789349	-
0.303027896	3.408408398	4.056373184	-2.947991534	5.315932671	5.05436311
0.871990278	3.263140547	4.135856624	4.923807534	4.735877232
2.783482577	5.335504052	5.198157373	5.026294505	4.497513989
0.560725285	2.344959635	4.343546287	4.7431109964.784346533
5.610948661	5.337298668	0.943845499	5.604827308	3.776968872
2.064482719	3.727638807	-0.447958353	-4.258042344	3.462192704	-
0.468223677	-3.579444068	-2.5537017 1.8131158412.685066107	4.633889843
4.246660654	2.798642145	5.634063642	-0.236458949	3.036869957
4.404428856	3.557880845	3.118113172
TCGA-38-4628-01A -0.9989474 4.596876058	2.5708114345.539846466	4.161712174
8.360446909	3.4410161144.321995216	1.751383852	3.509400971
5.895183455	-2.236084041	4.076249366	4.767167885	4.894241061
5.033013973	3.810669908	4.209197664	-0.055478382	0.477119162
2.817331416	5.035345674	-1.376298047	2.841323093	4.502651384
2.818525387	5.532096244	2.252908257	4.630083415	4.388292169
4.873180159	1.458772576	-3.753073443	3.261363835	-0.886055553
2.556990516	-3.74597162 5.597295247	3.283250245	0.161319749
4.723767958	-1.206746342	5.251087891	2.705169323	3.829514659
4.197717968	7.089261003	0.841783207	3.314506443	7.013771326	-
5.234986034	-2.076637767	4.486038446	-3.068305802	3.728279673	-
3.916172161	5.025095716	1.976150129	2.741579907	3.7731189836.114086533

2.969865572 2.779097437 -1.948620854 4.455761466 3.94188675
4.485127568 4.203826689 -2.105454132 5.081588145 6.327032499
2.745325102 0.225137476 5.86898933 -0.872734774 6.066668384
5.0911369134.744285581 5.505204343 6.328085022 10.30887145
1.785321758 2.792093133 0.93309925 -0.659615248 3.065693141
1.036524813 4.415850861 6.870013694 3.783340617 5.006704275 -
2.202272477 2.541292871 -0.068628652 3.405392012 3.820440331 -
2.732458854 5.342647246 4.106195138 0.273627688 3.098126802
4.216337449 5.032554 4.545866383 3.457542395 5.335358731
5.276923587 4.948383062 4.93268614 0.594699809 2.777091705
4.695103391 4.725785896 4.737349813 5.909612579 5.311238818
1.156560227 5.729988651 4.1157164571.91716565 3.878863924 -1.325325972
-3.707297853 3.054172335 -0.3509448 -3.826843729 -2.775325474 1.62689544
2.567182312 4.608258872 5.007675894 3.263677231 6.02975844 -
0.06485278 2.935317744 4.768120035 3.699084425 2.883466543
TCGA-38-4629-01A -0.545280688 4.41792123 2.385570391 5.665015622
4.173548589 8.256817625 3.18518179 4.359179488 1.365789673
3.546607359 5.712962031 -2.685516652 4.076872798 4.359852773
4.568143101 5.151366188 3.11117656 3.894435525 -0.197196803
0.373949642 2.432659451 4.805278288 -1.356710175 3.028758676
4.452562254 2.702236812 5.373158689 1.67839441 4.533963534
3.966467258 5.016332856 1.108391472 -3.765096583 3.476664605 -
0.679689359 1.939891041 -3.44059293 5.627717741 2.98628131 -0.220881288
4.729948393 -1.236147198 5.17758208 2.1190228023.887324903 4.121948427
7.014021918 0.684880022 2.816197694 6.895649108 -5.300760421 -
2.256255457 5.349773361 -2.710984851 3.221049628 -4.686658717
4.869890448 1.854093403 2.827532088 3.283347394 6.112544325
2.402729016 2.771227511-2.078568572 4.592109875 3.93818938 3.854655151
4.306849091 -2.065265654 5.091992068 6.229700936 2.598128587
0.024373874 5.614583496 -0.704098208 6.009433566 4.939521191
4.630018674 5.542666669 6.198855653 11.503919621.163587712
2.639330836 0.532284607 -0.420617324 3.132099873 1.257552624
4.401441312 6.791090552 3.31560541 4.826237096 -2.458986846

2.160054225 -0.319153003 3.329650475 3.813867704 -2.720563528
5.329516231 4.21212896 0.107348489 2.813518079 3.596315356
5.038959671 4.950067351 2.650525454 5.335547464 4.377445348
5.065948351 4.298807712 0.614240097 1.36681123 4.47488998 4.889827203
4.776641779 5.5587115224.719846046 0.729509483 5.249322335 4.15862524
2.087378903 3.768803308 -0.736431924 -4.500091681 2.794658048 -
0.311489581-3.865425454 -2.649654549 1.715270237 2.706227178 4.694579236
4.620171705 3.085997991 5.803149336 -1.092770245 2.509797156
4.39677518 3.470925332 3.357217464
TCGA-38-4630-01A -1.747906076 4.812486568 0.42228056 5.169212939
4.0184117758.092864844 4.643704124 3.943289045 2.61600136 4.29374453
4.994754303 -1.541538987 2.40631217 5.227225957 4.9962461173.656979472
4.953877928 4.797464745 0.066872133 0.1162006772.944959476 5.76587191
-2.068976098 2.284826964 4.763398321 1.903823761 5.541309367
2.293042354 4.717100384 5.204222309 4.923635835 1.774635604 -
3.588506361 2.949037324 -0.6140213 2.313515232 -4.891976336 5.583580557
3.489428491 0.31521902 4.670827661 -1.085642949 4.995329321
3.949562037 3.459162924 4.1770411256.83350493 1.462833293 4.34070566
7.616368027 -5.730886394 -1.308241446 4.624293178 -2.604438112
4.269939762 -4.346965912 5.31019824 2.247448552 2.6756421194.120212687
5.4236711332.465429603 2.032893336 -2.028004584 4.602070682 3.92367016
4.318418703 4.445480759 -3.208325618 5.076213952 6.636200581
2.809484962 0.924159913 5.139974245 -1.370513343 6.14340547
4.888609631 5.262860392 5.270520272 6.564715307 9.987754675 -
0.504885414 3.1681147051.822057426 -0.488576272 2.783734739 0.346331973
4.163356998 6.937262742 3.193235446 5.060418378 -2.011741668
2.4568656 -0.248523259 3.416544771 4.210133894 -2.991835317
5.317845561 4.326378215 1.907680521 3.359541239 3.15251959
4.9511653364.650554731 4.109736693 5.335594482 4.383804284
4.965659918 4.136591502 0.493189334 4.561009124 4.125091387
4.627323945 4.728553499 5.553482086 4.67434418 1.544799952
4.461860945 3.14938894 1.164607904 3.852099586 -1.697888629 -
4.065370141 3.557841785 -0.77523344-4.243432402 -3.805932921 0.651374077

1.697230549	3.102386248	5.681082173	3.695733515	6.410203143
0.092612616	1.915826366	4.401895227	3.773385999	2.090944543
TCGA-38-4631-01A -1.413336068	4.852085231	2.612806692	5.45976513	4.21831363
8.331622984	3.56777054	4.271742928	2.030051276	3.891294887
5.750740944	-2.284555547	3.935867791	4.905183631	4.882089995
5.0110348133.984610768	4.158410321	-0.606626421	0.339260042
2.861818283	4.61535932	-1.439460484	2.749700133	4.528631445
2.248535196	5.452029493	1.395396808	4.574971233	4.312149876
4.5772561111.258184093	-3.916478864	2.800512447	-1.065641988
1.533343385	-4.027741915	5.613729288	2.244898044	-0.975495471
4.71657488	-1.032613115	4.82903471	2.576381334	3.755587645	4.291327685
7.275653316	0.357221461	3.693090216	7.146033056	-5.660868327	-
2.040696723	5.169875938	-3.014060136	3.762676228	-4.528108005
5.027675417	1.586167926	2.493209818	3.5451109735.8929517112.858982061
2.807485149	-2.667119682	4.32994996	3.936523837	4.276559972
4.397858594	-1.912801413	5.083624614	6.234460124	2.8129485
0.1189001045.447103163	-1.369106573	6.187804383	5.048516664
4.700746813	5.528075206	6.353628817	10.63481231	1.557554353
2.759062289	0.511411275-0.771676574	3.125919103	0.955956306
4.358702482	6.769321557	3.749593892	5.088195378	-2.52249831
2.601605543	0.078242744	3.380104967	4.007579221	-2.73243811 5.3428225
3.815061203	0.399245544	2.899149243	4.423825154	5.044596248
4.583207753	3.33911363	5.335363664	4.958243879	4.819596122
4.628393622	0.570732968	2.007684263	4.562854683	4.701762715
4.758544801	5.871759476	5.0114449431.249649166	5.372279615
4.1161026751.625708853	3.864613964	-1.950462711	-4.034752646
3.051208227	-0.643463238	-4.045473303	-2.800522876	1.399250443
2.270628793	4.477371451	5.465596808	3.581313568	6.01963044	-
2.133194797	2.123543961	4.83182003	3.577153785	2.698341645
TCGA-38-4632-01A -0.651755708	4.677333945	3.134164875	5.653334584
4.168874581	8.345965005	3.348426216	4.379054564	1.656526562
3.640708892	5.909386421	-2.266163453	4.3494966114.65606605	4.929460623
5.055994222	3.1381129534.123492683	0.0823414110.4915651122.604477248

5.027225847 -1.347690755 2.64904612 4.513888096 2.67648702 5.409349719
2.144630392 4.52513982 4.307906105 4.692771796 1.229726314 -
3.79864149 3.279557377 -0.739920816 2.000194371 -3.254457084 5.620394107
2.627356522 -0.123191419 4.715714284 -1.168162198 5.183742621
2.270901329 3.900370346 4.230635232 6.928851246 0.682453769
3.05090225 6.975952569 -5.203228688 -2.034194779 4.954614673 -2.5597595
3.664612918 -4.402671936 4.964407362 1.899557267 2.786324727
3.659375501 6.066507373 2.673510291 2.857684392 -1.904104522
4.506735126 3.948193755 4.346459294 4.388303298 -2.116626117
5.084881794 6.216637842 2.707348794 0.04944104 5.678994811-0.899800561
6.128797834 4.92649155 4.616141804 5.6115383316.38724582 10.85691154
1.443457352 2.725647371 0.656159526 -0.526890466 3.335092402
1.274255408 4.441241377 6.812445253 3.4829114545.02160309 -2.292836015
2.70202089 0.1184832393.366624556 4.001699893 -2.664666982 5.341274946
4.352809564 -0.03003039 2.727734298 4.0441321195.078209297 4.714317861
2.796809883 5.3354776114.797653167 4.96342914 4.815761428 0.666148042
1.4106112514.6049421194.777359684 4.739245453 5.559797108 4.656764758
1.07343873 5.535183623 4.174754145 1.837052143 3.749811355-0.847258452
-4.056833832 3.046349735 -0.366327185 -3.778684026 -2.769646584
1.609474163 2.650912781 4.565793016 4.991963947 3.144116719
5.927747551 -1.632310023 2.418039339 4.585863978 3.51188167
3.259031384

TCGA-38-6178-01A -0.531131003 4.730935024 2.5826845 5.463458553
4.358907857 8.228087668 3.391009201 4.236035484 1.840505907
3.182307666 5.275728525 -2.58216519 3.416942386 4.616530343
4.867105784 5.09724652 4.173571828 3.929203351 0.806101017
0.603237187 2.693330033 5.132874764 -1.31443834 2.5308586 4.545003749
3.032792868 5.607522508 2.682003319 4.546890326 3.735709974
5.006873513 1.259641477 -3.670566508 3.757309998 -1.056614508
3.058711408-2.982505529 5.599837731 3.68419744 1.817637264 4.70571174 -
1.209272944 5.180355941 3.446768457 3.944157987 4.182622834
6.919777149 1.832223265 3.8611941446.80936258 -4.745269276 -1.871465265
4.851633202 -2.46515833 3.121524296 -4.865953046 5.161629947

2.167867019	2.320741433	3.851994312	5.977784478	2.649874163
1.936205906	-2.730507378	4.702700377	3.940197515	3.994533574
4.440229062	-1.789121045	5.078949653	6.356139199	2.826824175	-
0.049339935	5.662337405	-0.690036836	6.13427994 5.151049828	4.748733287
5.464215468	6.387655951	10.85357877	0.508291282	2.765584704
0.981137533-0.661830955	3.268909537	0.987143371	4.332008267
6.924793446	3.670447859	4.923513468	-1.720673606	2.369463585	-
0.589658386	3.310322263	3.758887864	-2.216525651	5.354162774
4.276314769	1.285755814	3.10837756 3.890246351	4.973094267
4.827879487	3.204146052	5.335459831	4.747179904	4.900987935
3.935864345	0.758897395	3.832490547	4.46378754 4.8114394834.740741489
5.603449084	5.130621754	0.886845079	5.5645911224.077735967
1.858636237	3.885988474	-0.104797224	-3.956152889	2.459701333	-
0.123982049	-3.414363174	-3.704221779	1.638228087	2.857077129
4.469279343	5.231328724	3.439424101	6.33486699 0.841716359
3.344995497	4.413558923	3.6211611512.977458448
TCGA-38-7271-01A -0.55755489 4.654094173	2.526031014	5.452242887	4.16298111
8.350557314	3.567189697	4.354182958	1.836915225	3.662468936
5.484271592	-2.318394789	3.524912125	4.308732859	4.804405585
4.947936989	3.615138671	3.949196095	0.65089977 0.617209499
2.613996516	5.305158788	-1.335310731	2.916880906	4.534273145
3.049533214	5.839723178	1.694325213	4.561318422	3.449606505
4.854966433	1.047179503	-3.608628295	3.82831995 -0.969699293
2.497304328	-3.019877977	5.575803079	3.42569055 0.961940388
4.710875357	-1.135219274	5.444403471	2.8432113394.048202739
4.401499429	6.829283152	1.481309135	3.194669983	6.766799273	-
4.891214721	-1.716344981	4.312769679	-2.626644035	3.432493178	-
4.616781004	4.995199182	1.990384079	2.109935592	4.069667589
6.212973534	2.931241657	2.39015185 -2.10798157 4.717701765	3.932275948
3.899785993	4.339682477	-2.029935435	5.102205662	6.411577506
2.843024129	-0.051295101	6.225268277	-0.281307879	5.961974134
5.169750738	4.433942344	5.493105008	6.336190514	10.78065446
1.168779953	2.577190783	0.742920089	-0.519026889	3.80550079

1.030732548 4.271616665 6.851528621 3.640834131 4.655787986 -
1.8415911892.08355152 -0.49616933 3.290496345 3.940709842 -2.465320587
5.342071709 5.027448958 1.37191378 3.42174494 4.1595112834.951091404
4.660708857 2.796395076 5.33562099 5.166072977 4.956607677
4.166803321 0.620136401 2.998139566 4.553299127 4.73086135
4.778450996 5.618976941 5.187878046 0.977687913 5.775467386
4.174508202 2.093723395 3.769144979 -0.169741785 -4.243047953
3.151758582 -0.475015929 -3.501159619 -3.390015699 1.763238597
2.872431793 4.584859086 4.844647298 3.09738027 5.860704272 0.43468311
3.145454272 4.3311502173.694799401 3.005377858
TCGA-38-A44F-01A-0.156762512 4.995421837 2.980152991 5.259706237
3.9089156118.257271235 3.710053092 4.333660067 1.806566689
3.264203193 5.38223081 -2.528347486 3.837582641 4.144707436
4.853480301 4.879036402 3.419974331 3.930240088 0.328546181
0.475644828 2.678915055 5.283026584 -1.000988886 1.793578388
4.503649728 3.014131944 6.028563264 1.566653351 4.591578177
3.059664093 4.731837237 0.671354214 -3.536307908 3.09375759 -
1.497276981 2.756391808 -3.117171836 5.576578441 2.885525859
0.146271398 4.732153516 -0.992190526 4.89856799 2.279864724
4.127076639 4.35300118 7.099828044 1.389491042 3.563271768
6.748006078 -4.694568613 -1.862450701 4.530273357 -2.413674926
3.363202224 -4.854043506 5.132252454 1.96495538 2.0227978113.972724961
6.158306307 2.73541738 1.439762188 -1.970565148 4.722656596
3.926804163 3.944487723 4.292528502 -1.278228992 5.103316163
6.371396736 3.053719874 -0.178201883 6.223873361 -0.002675311
5.960698212 5.214832239 4.533092074 5.4934281136.106299479
10.29243423 1.1121406262.533054689 0.548106795 -0.637526281
3.447387357 0.958215464 4.319122068 6.870213857 3.627014622
4.571270724 -2.101748267 1.91124991 -0.573341961 3.200362551
3.712580765 -2.403815672 5.342850237 4.770792553 0.878714176
3.059959753 4.425716709 4.97967991 4.745587936 2.699106754
5.335542728 5.22032138 4.840549499 4.22509397 0.768368548 1.843428174
4.63430254 4.851030192 4.7949669 5.666771957 5.162708099 0.968235277

5.456258566 4.372158923 2.24013215 3.905668808 -0.715809393 -3.905967479 2.940712288 -0.210924284 -3.429884815 -3.353735299 1.689083375 2.97974693 4.483452403 5.217895624 3.272000605 6.017492823 -0.606633736 3.1131445964.538702403 3.812859158 3.360639042

TCGA-44-2655-01A -0.793581579 4.475198175 3.795544037 5.676677374 4.157368706 8.280994036 3.304513933 4.354564014 1.615941226 3.085398239 5.54075783 -2.401591765 3.784131876 4.394470723 4.534078161 5.109281909 3.3576402 4.208993768 0.296147782 0.369325951 2.579055085 4.903761059 -1.424321055 2.360055661 4.482783 3.218914818 5.613014424 2.978175662 4.343827783 3.143016874 4.749194014 0.810299164 -3.995339823 3.506837004 -1.357618501 2.71960703 -3.268188141 5.598347713 2.75507025 0.432913964 4.710500605 -1.356246926 5.19568669 2.746879692 4.022765456 4.2552089 6.975376471 1.053737449 3.3355112556.485539608 -5.258788561 -1.990270541 4.701128105-2.642926238 3.223877813 -4.821503237 4.769721399 1.807648233 2.349763104 3.78654809 6.080808252 2.703222313 2.56165451 -2.129193915 4.492627912 3.94505181 4.149658878 4.076681826 -2.038924576 5.078190514 6.15830783 2.918661285 -0.174601743 6.15258038 -0.973002227 6.033233449 5.162008307 4.359925682 5.596037717 6.29721156110.48988433 1.137015929 2.418844598 0.607363185 -0.939738242 2.950169563 1.0117870674.439216262 6.796901366 3.837471958 4.730770799 -2.349474848 2.125369716 -0.353108605 3.278119844 3.637170322 -2.368240615 5.361431515 4.437408381 0.048569846 2.633196058 4.330554896 4.985770573 4.724135753 3.012162342 5.33523559 5.286948798 4.803208475 4.445582094 0.423294453 2.187279825 4.721578594 4.693321604 4.735236029 5.839419484 5.349052948 0.863886355 5.745443788 4.340462994 1.893459898 3.821192693-0.743655191 -4.252082734 2.739913245 -0.200679867 -3.597155194 -3.645456467 1.759257105 2.853771298 4.749661966 5.018841716 3.260384169 5.994404791 -1.226662767 2.94853683 4.563619423 3.57888119 2.987480686

TCGA-44-2656-01A -0.339279511 4.655672285 3.043439487 5.557750076

4.509664058 8.277895643 3.232765452 4.353915103 1.565416222
3.106096919 5.434465732 -2.566674543 3.812905512 4.624751958
4.7795241185.162040542 3.302381934 3.688289721 0.603702878
0.630816275 2.409421215 4.900914416 -1.25739766 2.657500374
4.5111337023.143270841 5.561596753 1.899628009 4.42734651 3.523198717
4.812253472 0.744505411-3.686410895 3.663208277 -0.896283722
2.634238392 -2.929833606 5.605472253 3.397255592 1.341125861
4.708370537 -1.25873791 5.473103213 2.760649708 4.045467393
4.273148724 6.888691409 1.333827732 3.308657513 6.534818489 -
4.849717973 -2.140668092 4.819931874 -2.471325234 3.202358703 -
4.915434149 4.93351704 1.76604205 2.369026671 3.801089599 6.108687868
2.777410182 2.38614784 -2.85909998 4.604017063 3.945589653 3.681125353
4.416862088 -1.45623742 5.0911635316.124401273 2.811960724-0.156090147
5.974616885 -0.493077022 6.061098006 5.101868989 4.345860767
5.532182579 6.241824317 11.045822390.937656223 2.699250393
0.585365809 -0.882531027 3.533796447 1.221498939 4.339857693
6.812595569 3.6451135534.830331369 -1.874682987 2.4023179 -0.441946306
3.21882286 3.620973847 -2.235188866 5.3566561144.485995572 0.654787698
2.945087555 3.963916402 4.992031619 4.853707841 2.728923426
5.335415755 4.91500753 4.943727061 4.219157069 0.571025082
2.641250877 4.590544679 4.770068555 4.771763662 5.6661348
5.022813859 0.725358382 5.772578333 4.595259543 2.134425713
3.790385024 -0.326096482 -4.289804987 2.756277867 0.015945145 -
3.416652683 -3.594665811 1.894865614 2.95650928 4.738550772 5.010572166
3.373874136 5.956782889 -0.200659596 2.9511683164.417450482 3.56961071
3.261087228
TCGA-44-2657-01A -0.445439063 4.774256042 2.558555656 5.282187896
4.307821655 8.244595051 3.839415013 4.221403018 2.04760518
3.616278107 5.293083697 -2.181236503 3.704024322 4.53829564
5.099453543 5.051947715 3.982336338 4.316242499 0.378342628
0.621424279 2.888442333 5.327278366 -1.277567405 2.713907621
4.578104021 3.126583586 5.8507329 2.203629453 4.652969613 3.66058439
4.898667624 1.179316189 -3.503730775 3.384057451 -1.110267086

3.088581877 -2.949529679 5.574744942 3.1901129611.4012295 4.716605545
-1.009504559 5.316524819 3.037665236 3.993196 4.280900644
7.017082442 1.803694653 3.902333762 7.021375961 -4.688291303 -
1.614605459 4.41298395 -2.561985189 3.719779999 -4.385704668 5.200616233
2.312421887 2.459275909 4.173874539 6.149332544 2.970157587
1.846050398 -1.946698866 4.7592099113.935295025 4.219962088
4.285904462 -2.137898333 5.092889589 6.438339046 2.934408972
0.124730086 5.949451844 -0.280902228 6.008023618 5.13729159
4.656100898 5.439033377 6.267872969 10.41270832 0.734577208
2.769440571 1.024426973 -0.55705925 3.83536664 0.920619057 4.283047052
6.943632808 3.653224907 4.848133917 -1.708275372 2.267666752 -
0.139465787 3.306854312 3.968311508-2.414755835 5.348046449 4.821426176
1.331566287 3.357635077 4.176030073 4.984931527 4.696447882
3.214952437 5.335556421 5.098047013 4.945944159 4.343297788
0.861561527 3.495483391 4.639963983 4.808608892 4.76988493
5.645278544 5.3411184641.267777492 5.468546617 4.218520374
2.024571876 3.89588171 -0.505084497 -3.724972596 3.167050174 -
0.209719745 -3.542656452 -3.235809679 1.684147575 2.877994706 4.49799393
5.290280233 3.454208275 6.330658303 0.4011030983.079288799
4.614183396 3.807593679 3.573555257
TCGA-44-2659-01A -0.714959272 4.583933171 2.565198456 5.41625065
4.358272487 8.3281154473.343007405 4.279386661 1.804609207
3.305829805 5.370610845 -2.434060162 3.473721913 4.618033468
4.845228283 5.169134477 3.8811281283.765006172 0.773549934
0.562374501 2.620216049 5.222967754 -1.45243843 2.864229728
4.5472851123.121328158 5.680745526 1.670080793 4.47494628 3.213794899
4.936092922 1.072757956 -3.752166062 3.986180451 -0.907257366
2.546548753 -3.020756714 5.593172057 3.752480645 1.96011259
4.711273547-1.23584806 5.133701914 3.374745884 3.96362847 4.27332023
6.905679041 1.69887392 3.692920169 6.522834572 -4.52519637 -1.792563272
4.657322396 -2.448125885 3.134170257 -4.78562925 5.042995845
1.967789578 2.249222506 3.972356426 6.137635967 2.668688554
2.023066127 -2.318008484 4.692803869 3.945659021 3.891422849

4.19858135 -2.030524925 5.085243046 6.34341653 2.885002545 -0.12475803
6.015340357 -0.656729604 6.014895972 5.1141758784.516566515
5.485926388 6.340744012 10.9815909 1.064694065 2.61880708 0.686293965
-0.881370387 3.218605026 0.885842248 4.299108238 6.908761488
3.606750178 4.815962586 -1.822655506 2.169720787 -0.22681494
3.275468483 3.777516111-2.346036974 5.354927424 4.74285962 1.463126287
3.240653288 3.949643698 4.97278531 4.775549832 2.9742681195.335487872
4.872168655 4.93612325 4.001898792 0.567735099 3.4738110494.491112983
4.727591747 4.755967278 5.650803471 5.203369066 1.051282853
5.593595546 4.339905863 1.992457694 3.851789934 -0.380712232 -
4.054424521 2.811392681-0.114245359 -3.24784353-3.707170912 1.747119307
2.88038568 4.578226203 5.065835729 3.228144518 6.168216931
0.333049907 3.19905493 4.398483127 3.576705873 3.02619647
TCGA-44-2661-01A -0.513427145 4.656803247 2.985809034 5.495625952
4.176859886 8.357640678 3.639709609 4.385793502 1.717517853
3.537615869 5.495609394 -2.222136057 3.89597665 4.427492529
4.8927114354.902925809 3.248836744 4.054590382 0.408628941
0.487083496 2.573546168 5.448768187 -1.252130412 2.66914722
4.537150994 2.961481244 5.902836216 1.674814023 4.531972577
3.394666566 4.6346115080.902491456 -3.735170726 3.466179599 -
1.008546188 2.530417273 -3.287105533 5.576243073 2.882290969
0.339679732 4.724206222 -1.169932237 5.43133676 2.454941922
3.994978343 4.374685321 7.048441544 1.167500554 3.209003019
6.750003703 -4.985194956 -1.848564463 4.332984415 -2.693309171
3.808845249 -4.304486361 4.897600545 1.974055357 2.812429782
4.177704586 6.213094232 2.805154479 2.145349407 -2.115063419
4.633361966 3.938863406 4.154597625 4.19348731 -1.795793372
5.094399546 6.3345949 2.922800196 0.008913904 6.299286025 -
0.3294994115.904750618 5.189582574 4.365284895 5.518156993 6.324702757
10.53701992 1.161657448 2.633883581 0.755023561 -0.713691991
3.336431872 1.008623039 4.345493237 6.853853738 3.652683189
4.65867274 -2.238127511 2.087153223 -0.163019527 3.285589828
3.874088826 -2.418706587 5.338648887 5.1172414170.7117597133.221996615

4.306376725 4.95781731 4.695607154 2.908609143 5.335370269
5.337358199 4.908869698 4.501595608 0.550737293 2.013551802
4.582066543 4.699571383 4.80559814 5.655848989 5.22432626 1.03914619
5.654860248 4.526518255 2.234987249 3.790093123 -0.774716459 -
4.013330174 3.367001296 -0.318776884 -3.526537256 -2.826743474
1.710898565 2.76048966 4.556496557 4.916131698 3.132989861
5.881833047 -0.543129608 2.980871897 4.57985305 3.714047836
3.496244259
TCGA-44-2662-01A -0.753339626 4.433618348 2.448208735 5.478573016
4.320594594 8.297076295 2.907094677 4.346534749 1.549725819
3.564200702 5.502543768 -2.720303387 3.386429425 5.065254584
4.503332565 5.235306805 3.640434517 3.1109886590.702429681
0.437271582 2.434727555 5.09810321 -1.563208179 2.457731487
4.591050194 2.50644415 5.442409615 1.238485371 4.410984442
3.165647816 4.784791545 1.041860213 -4.04105036 3.512095011-0.745608048
2.325448397 -3.186011914 5.619900562 4.0177454 1.276128143
4.703498762 -1.336260182 4.979606096 3.074178601 3.931459485
4.145978471 6.903891041 1.143178478 3.248656405 6.619507446 -
4.924924558 -2.048229929 5.603417445 -2.327498251 3.222240092 -
4.798605808 4.938885236 1.483607677 2.764823102 3.557716304
5.987947276 2.4258481162.313749588 -3.202972926 4.605038498
3.951991753 3.4595845 4.462747786 -1.618187274 5.080589329
6.017078447 2.711820219-0.162995494 5.580054556 -0.728564855
6.040880162 5.144730203 4.464574124 5.424362702 6.263626735
11.324815010.707207565 2.717985759 0.436976186 -0.789399445
3.080916228 1.067991473 4.206985839 6.730346372 3.434179729
4.962278974 -2.175574593 2.22506188 -0.196065476 3.259044189
3.526400628 -2.636870849 5.334744645 4.521756966 0.918246821
2.875422004 3.792002865 4.907875497 5.02552105 2.680909157 5.33513394
4.665225526 4.977100854 3.820613764 0.429310235 2.27519612
4.317606846 4.743185838 4.8023111855.221972256 4.759229273
0.314428659 5.615679876 4.264503492 1.896856251 3.732431457
0.188961959 -4.012558725 2.74585654 0.058722126 -3.51905187-3.531116933

1.602620361 2.465013257 4.170613987 4.817283884 3.581876515
5.967599681 -0.441990709 2.93101996 4.459550174 3.501093718
3.371276185
TCGA-44-2665-01A -1.047330628 4.131306689 2.602940532 5.516570197
3.868180708 8.303872036 3.368505496 4.347104224 1.458945986
3.398518572 5.3317526 -2.74934877 3.42847635 4.1188175354.469126944
4.975572942 3.310109143 3.87860499 0.150503388 0.3168114972.460088006
5.284039648 -1.581994147 2.870019331 4.496821037 2.60782174
5.471837179 1.683624052 4.415029291 3.07987578 4.782018775
0.945094231 -4.152999874 3.784356552 -0.991468899 2.389220986 -
3.075483646 5.591001842 3.2113057350.352909079 4.715256649 -1.372415147
5.218968992 2.521354982 3.971984234 4.193203721 6.819305902
1.274714232 2.908375561 6.413298666 -5.150474672 -2.014842721
4.666900862 -2.592228283 3.117079544-4.978769473 4.77750232 1.909359082
2.509528696 3.8411974516.107817621 2.438514277 2.502377777 -
2.951550507 4.644164639 3.938332856 3.900444627 4.185537809 -
2.310605768 5.088208963 6.3195232112.796389927 -0.170408006 5.852730659
-0.976717777 5.899244276 5.127790997 4.378300276 5.542049991
6.389261663 11.153029420.865397765 2.270357016 0.871080176 -
0.671305508 2.956780262 0.9452319 4.297717346 6.780544039 3.471914179
4.697037334 -2.363610701 1.870521538 -0.556100018 3.307401857
3.837838684 -2.644189466 5.331990022 4.904814685 0.625490696
3.158221068 3.728745878 4.922935333 4.961364609 2.701123208
5.335377905 4.790066681 4.940620958 3.957443099 0.5496533
2.142308555 4.331893525 4.740933741 4.763038271 5.410531146
4.869831351 0.63440069 5.442457189 3.7063079 1.918287407 3.760561913
-0.166616928 -4.374138 2.777999183 -0.317570135 -3.602949236 -
3.281869078 1.587445537 2.64959758 4.326324552 4.654089763 3.026210556
5.799594712 -0.700638485 2.757320484 4.259962863 3.403419943
2.927633975
TCGA-44-2666-01A -1.365461988 4.537691585 2.449850072 5.526567217
4.19164055 8.33038043 3.424080292 4.310083858 1.993038294 3.12135702
5.13578433 -2.120023113 2.913527745 4.844996969 4.497525688

4.722998238 3.276778284 3.693736517 0.847555225 0.43933647
2.307371902 5.304497377 -1.925207123 1.996348317 4.671632121
2.7118479315.681500961 1.248059673 4.273051418 2.917365063
4.551957738 0.760022906 -3.965310792 3.544027473 -0.7261624 2.581494
-3.793889592 5.589836044 3.20674193 1.334468733 4.698922804 -
1.268234683 5.310998631 3.062243414 3.837280916 4.261381694
6.836933172 1.209986666 3.512892638 6.539581861 -4.909500038 -
1.786809493 4.692686164 -2.366677715 3.562970931 -4.753955338
4.786633559 1.552216242 3.000124545 4.041854568 5.826326816
2.479731614 1.776066859 -2.820157076 4.53786841 3.951203229
3.753633939 4.128028565 -2.867052494 5.071274169 6.192026566
2.942011095-0.042389447 5.983027565 -0.997305386 5.97992848 5.138538724
4.333002087 5.393886655 6.492695442 10.41003829 0.509194529
2.826040646 0.82118708 -1.091683016 2.521269627 0.84504681 4.246254359
6.83425814 3.489541506 4.701601959 -2.248431605 1.91642234 0.048661225
3.214355773 3.738726613 -2.994761262 5.331574535 5.125439197
0.463364912 2.81575302 4.146275509 4.8813919114.82346405 3.080159185
5.335130713 5.063777255 4.863907477 4.039170413 0.054772 2.3632293
4.354343551 4.561586864 4.80211533 5.551788972 4.931686545
0.754224276 5.556267662 4.841661442 1.613433858 3.741436957 -
0.715395718 -4.070168799 3.030393025 -0.17894765 -3.467266941 -3.824690243
1.368952432 2.402899647 4.065373864 4.950796731 3.04897957
5.994392036 -0.319212511 2.894549394 4.455870274 3.59966131
2.754522808
TCGA-44-2668-01A -1.151033048 4.023653486 1.518512937 5.547016619
4.215425948 8.31791958 2.900542132 4.338759096 1.049835042
3.502310433 5.796600291 -2.979139791 3.214132523 4.538001691
4.240120152 5.081642549 3.600127093 2.936290996 0.251365217
0.380685076 2.178222978 4.806490004 -1.68458319 2.792231801
4.509243668 2.352935748 5.67945319 -0.915815939 4.358727561
3.448235273 4.766295259 0.810999315 -4.022848564 3.619239164 -
0.622799152 1.92067333 -3.586082189 5.625456631 3.99921157 0.353585549
4.68735034 -1.437593403 5.34676716 2.628400996 3.883205707 4.161002409

6.705814349	0.975330887	2.544167442	6.379891281	-5.246836023	-
2.47188868	5.557073572	-2.55164204	2.755159684	-5.378554148	4.618607544
1.222630199	2.363576417	3.614982829	6.060867537	2.405806633
2.665549181	-3.426363124	4.57389875	3.94431668	3.011056579	4.388505479	-
1.878284997	5.085641373	6.03918288	2.576043019	-0.225064559	5.666789031
-1.014522398	5.95205751	5.085256871	4.304030051	5.510949095
6.240395334	11.52290327	0.913296562	2.5713219	0.376769512	-0.861091981
3.152734393	1.051589729	4.282426606	6.658271726	3.309495921
4.964723953	-2.231154431	2.09410083	-0.901586952	3.222467746
3.361411384	-2.808372336	5.334398771	4.399719454	0.627348575	3.21088673
3.766900771	4.827346531	5.071204169	2.404429655	5.335088468
4.478967011	5.020734719	3.360610752	0.335936895	1.579747173
4.233193067	4.688757822	4.78566704	5.502666101	4.687904187
0.133261268	5.609317854	3.930132491	1.896382171	3.609893321
0.425458893	-5.137584188	2.14776341	-0.154238689	-3.612127959	-
3.729161784	1.538282714	2.343949022	4.358531357	4.487358825
3.289371262	5.54833457	-0.472508363	2.530307429	4.053680168
3.315296553	3.187935247
TCGA-44-3396-01A	-0.465444407	4.616988104	2.51162642	5.387942737
4.118916949	8.27876023	3.508472471	4.331931265	1.620311849	3.635882634
5.479050633	-2.505639632	3.916220083	4.533935474	4.789202286
5.144224702	3.513412167	3.817550395	0.081750605	0.410100262
2.615259835	5.178525246	-1.196671063	2.54911434	4.57805207	2.61859884
5.732516833	1.153307225	4.653336278	3.817675571	4.876249338
1.120281334	-3.700652654	3.290499786	-0.878691307	2.23758843	-
3.42120372	5.599504809	3.303103371	-0.004717872	4.724025815	-1.102497086
5.168255569	2.585676247	3.990539126	4.23160733	6.985777458
1.206023804	3.637124899	6.943258601	-5.171726	-1.885754934
4.975236918	-2.562781728	3.489492251	-4.774346772	5.113129776	2.05752537
2.394861672	3.783053805	6.119072078	2.554308896	2.173176973	-
2.345196877	4.734017028	3.938777658	3.891481204	4.388445678	-
1.782232459	5.091780821	6.297527949	2.79234675	-0.017373386	5.880613035
-0.453614683	6.062339052	5.067938102	4.684265835	5.530287563

6.319788806	10.7054863	0.566413354	2.6654119840.651752768	-0.533988283
3.309656573	1.034819239	4.313898445	6.842592985	3.475047482
4.856525595	-2.072587188	2.364921288	-0.498915116	3.292610484
3.770415559	-2.483743927	5.349264172	4.516205331	0.856556603
2.95452656	3.819877479	5.024917005	4.845174466	2.955519725
5.335582616	4.827534836	5.0045811934.092562915	0.832553609	2.21348659
4.491246409	4.899309475	4.76687112	5.549537205	4.988748268
0.917424795	5.480844627	4.152392433	1.98338035	3.873340502	-
0.557497588	-4.182197993	3.010670737	-0.274545233	-3.746975771	-
3.332597916	1.5705011912.809753395	4.493103106	5.007860289	3.327087186
6.233908392	-0.675615089	2.645421538	4.574917649	3.623092483
2.9248136

TCGA-44-3398-01A -1.035352269	4.387907347	2.991360137	5.623944016
4.063329565	8.40831647	3.649941033	4.418878792	1.709571687	3.61588745
5.607947575	-2.385460498	4.000489283	4.336484258	4.696514999
4.977632887	2.887102528	4.088079494	0.557174915	0.501645883
2.430635967	5.418189011-1.42029501 2.728606055	4.500054006	2.720404408
5.693571733	1.482445863	4.465213081	3.301352129	4.486287362
0.976550742	-4.102524647	3.647423639	-0.885605186	2.40255491	-
3.067866022	5.5911339062.696742776	0.30396401	4.729768738	-1.30412519
5.490389567	2.190005458	4.004127724	4.348377397	7.1246618
0.946407383	2.681697376	6.607927368	-4.974097664	-1.901949715
4.573177027	-2.602969026	3.595447347	-4.077724184	4.679013033
1.76481009	3.197047516	3.924546504	6.170029774	2.774261544
2.319410026	-2.115295646	4.5091136223.943914904	4.19993229	4.137326908
-1.904412547	5.09123372	6.237905628	2.828684601	0.024176694
6.187242882	-0.676519967	5.824614016	5.202493258	4.208296223
5.520959177	6.337749575	10.89904442	1.350875452	2.476373355
0.819566389	-0.684670339	3.103061226	1.092912047	4.349615967
6.784030969	3.655846078	4.626372347	-2.449653858	1.979677441	-
0.025022524	3.3131107293.87606137	-2.693614147	5.32058267	5.19087129
0.185802683	3.157067412	4.244683845	4.920636817	4.83492055
2.660678226	5.335205364	5.202484446	4.868534894	4.673049492

0.286576654 1.182372398 4.573718592 4.568443812 4.820995157
5.554149774 5.102325816 0.893215408 5.544030746 4.350130261
2.157639261 3.712522435 -0.42217698 -3.931067914 3.448110586 -0.24124949 -
3.400018463 -2.085573546 1.867301771 2.603978974 4.49479084 4.683186736
3.075472425 5.55036314 -1.17220375 2.798440831 4.546388029 3.540029947
3.67208885
TCGA-44-3917-01A -1.33546292 4.37591194 2.201321418 5.467892314 4.269108379
8.406501245 3.947074971 4.213817595 2.224135911 4.010769992
5.369555959 -1.759539924 3.51973974 5.365170119 4.914601321 4.540039382
3.72479208 4.271588409 1.158993788 0.430137562 2.448568629
5.637793627 -2.088499451 2.889345805 4.814197229 2.230922163
5.838488511 1.602940789 4.311918998 4.38280531 4.250473405 1.356775701 -
4.046525279 3.575402119 -0.604934059 2.1030487 -3.933181251 5.589114079
2.666558805 0.940002582 4.683083293 -1.336331682 5.59074735
3.663634304 3.662057151 4.36230326 6.762631007 1.292901926
3.626923068 6.811299938 -5.322525355 -1.579628728 5.010520267 -
2.260002636 4.242172359 -4.168265768 4.793806125 1.821709876
3.126216258 4.2015958 5.750075779 2.967814662 2.337071344 -
2.980566196 4.443977331 3.964545227 4.248158642 4.313517986 -
2.986205568 5.064915838 6.1594783 2.833902352 0.347125358 5.749147685
-1.163820717 6.011187864 5.073892553 4.333025686 5.318755166
6.797335637 10.6844866 0.647417122 3.057469151 1.393228635 -
1.127192401 2.997243079 0.884419682 4.140226097 6.853117535 3.601361223
5.148332999 -2.146621422 2.718835625 0.247479327 3.342518942
4.22361059 -2.816268131 5.341583894 4.976366662 1.171465922 3.20828795
3.828822086 4.941707111 4.667300161 3.385130037 5.335078976
4.926281759 4.953602704 4.452647533 0.09077165 3.243495391
4.387322379 4.422815642 4.802566821 5.504812942 4.62875549
1.213193744 5.305547034 4.296164772 1.50178891 3.705959133 -
0.706756245 -3.546299347 3.804283399 -0.201516613 -3.676645765 -
3.124417953 1.111309301 1.935980696 3.654871802 5.380684123 3.730028486
6.06385087 -0.965509555 1.862335503 4.532361062 3.503761592
2.651670664

TCGA-44-3918-01A -0.976595453 4.527347009 2.749346714 5.550194154
4.250782221 8.3568511423.631498686 4.358382576 1.70639941 3.500601307
5.598744329 -2.328811167 3.89570366 4.35724566 4.841008622 5.050687971
3.479619255 4.188775622 0.528875775 0.470076663 2.519684272
5.261887432 -1.451735924 2.765558125 4.5538479 2.732819684
5.686777549 1.686077 4.537700876 4.130784903 4.686167612
0.976055849 -3.956542624 3.720545664 -0.758754836 2.381206754 -
3.2604411295.599037373 3.023949438 0.253491826 4.712889524 -1.267041572
5.582270156 2.499381973 3.975448303 4.285574489 6.966761948
1.24015353 3.059603604 6.707771437 -5.033366727 -2.008787666
4.644646454 -2.604694041 3.494471041 -4.561680984 4.845911175
1.857121601 2.433041938 3.941260914 6.108382033 2.824613574
2.515663917 -2.275075047 4.552737609 3.941351883 4.039180925
4.344245622 -2.036179679 5.095837903 6.249063305 2.822739339
0.0116622576.007890727 -0.832480198 5.984705203 5.102522767
4.368372525 5.538957564 6.413456293 10.86127156 0.967337024
2.542164135 0.898478972 -0.770485373 3.308755934 1.025339097
4.386831932 6.804331589 3.66631624 4.869413731 -2.075302344 2.42669447
-0.330938614 3.305647459 3.887702701 -2.587755436 5.342358949
4.748687929 0.428067039 3.316450068 3.940091506 4.946216601
4.8022611682.97898141 5.335368508 5.020092775 4.9010694 4.474252797
0.409461324 2.104509151 4.544155657 4.672310917 4.780035887
5.697558381 5.1133587160.892515798 5.70841984 4.219682869 1.999900251
3.82842009 -0.440977023 -4.290930061 3.073967505 -0.414981418 -
3.588818978 -3.136727174 1.572818479 2.727316161 4.509033088
4.9611056733.3198561125.812782819 -0.725476692 2.773670081 4.433724983
3.524720703 3.157987134
TCGA-44-3919-01A -0.687296038 4.624640689 2.106789672 5.300165888
4.400524651 8.306686737 3.595662626 4.275702002 1.872189794
3.497533159 5.247238941 -2.328618813 3.582514058 4.888976541
4.9905068114.9499211843.805813573 3.968635001 0.412179434 0.457499402
2.7082281115.418261132-1.280265428 2.394978498 4.574518706 2.780589043
5.635603423 1.9761176714.624503533 3.646563137 4.949293026 1.1213481

-3.59650582 3.398284086 -0.844579391 2.770752407 -3.480446891
5.594240408 3.824597708 1.09814905 4.717293342 -1.139800523
5.248525928 2.879392208 3.959042008 4.236960742 7.05574746
1.486196005 3.818724556 6.844383168 -4.735415297 -1.775258023
4.808386119-2.445825599 3.618604992 -4.45543973 5.140465747 2.024405766
2.7761169333.964786309 6.0508723112.647428916 1.961395372 -1.836039378
4.64673587 3.938727307 4.017500554 4.307113943-1.597170127 5.085650558
6.335476789 2.906862321 0.1142442055.843724646 -0.277485506
5.967150917 5.079953809 4.701897084 5.459144838 6.329379689
10.39162022 0.624632699 2.896704897 0.954608286 -0.661417877
3.404819551 0.851443149 4.26622789 6.864026958 3.462915203
4.896144532 -1.923104684 2.245104391 0.037988918 3.295267445
3.687300306 -2.450868067 5.336792215 4.663054214 1.138564428
3.359542593 3.782627851 4.942066124 4.86731537 3.320505973
5.335414724 4.719941863 5.0300391154.297417443 0.618651246 2.92890742
4.507000996 4.769090627 4.781567266 5.5993536 5.0041156330.937900134
5.545217658 4.299808306 2.109202078 3.932042833 -0.424773063 -
3.629342972 3.273624091 -0.076655675 -3.516855857 -3.486872941
1.604556713 2.720350987 4.309748179 5.285039695 3.444339576
6.169546492 -0.162249782 2.819864619 4.6321187363.744574945 3.33318699
TCGA-44-4112-01A -1.261073822 4.4988931172.877743541 5.540212048
4.072940665 8.350081558 3.573195539 4.372914564 1.651624701
3.353837314 5.544756898 -2.347728197 3.658370448 4.587288467
4.744455434 4.8075186113.405915408 4.132127828 0.31113817 0.307478579
2.584771926 5.359638499 -1.538933751 2.564854441 4.545194513
2.618862307 5.605435918 2.213407146 4.496220197 3.780109235
4.72904599 1.174307226 -4.144953003 3.622142916 -0.835989298
2.530091006 -3.470348988 5.60149808 3.252003285 0.324802979
4.714319434 -1.336251476 5.149649252 2.689482437 3.91751381
4.235678389 6.90637475 1.020974109 3.147732261 6.616506783 -
5.21716985 -1.907509013 4.792978419 -2.664179075 3.418587753 -4.243021549
4.890258895 1.938540732 2.546460185 3.866648333 5.949321729
2.633424125 2.581730584 -2.433880706 4.497571094 3.947643337

4.282966392 4.245397075 -2.133062727 5.08046923 6.320570422
2.820488253 0.058104938 5.872508815 -1.193303897 6.047450171
5.096722263 4.480605568 5.513183863 6.464134256 10.51918483
1.123222038 2.578668965 1.078050028 -0.632900061 2.84599377
0.962660214 4.357988537 6.813533005 3.578512287 4.843278301 -
2.333958776 2.295700197 -0.251726123 3.334380691 3.836404079 -
2.574835421 5.341482575 4.514851271 0.196955684 3.042077156
3.704448298 4.97029946 4.816279977 3.182607261 5.335307485
5.037345161 4.904602164 4.486821699 0.327605715 2.176169231
4.556104989 4.683881332 4.758043842 5.691678919 4.904205303
0.826994248 5.6569021114.193240681 1.810448579 3.848984669 -
0.724152478 -3.848099785 2.986179831 -0.261167737 -3.677171251 -
3.194020878 1.4971431182.609690603 4.312981084 4.963216946 3.291885167
5.905834034 -0.681285612 2.830530321 4.41676196 3.466963602
2.697393326
TCGA-44-5643-01A -1.236191581 5.265008135 1.765375639 5.488029677
4.131524573 8.087134307 3.990503081 4.1175446282.313288278
4.296801275 5.46879273 -1.925469142 2.429276156 4.713302148
4.845763688 4.126051939 4.437896725 4.198291492 -0.257079457
0.096859954 2.915305801 5.111201744-1.472853763 2.508210378 4.69193818
1.901422831 5.712944454 2.407523673 4.7731129075.3118945564.86276062
1.302745956 -3.519089812 2.44735208 -0.71852067 1.779416103 -5.268325559
5.588958513 3.832672987 -0.832311235 4.669798198 -1.098872422
5.024217437 3.04317792 3.575352278 4.2161182076.978331614 0.631968453
4.74466074 7.458185728 -6.590547497 -1.527864362 4.873118082-2.977647743
3.570825493 -4.907784051 5.093171294 1.853714898 2.400002014
3.86856727 5.698930599 2.36987373 2.10940762 -1.997035273 4.571439968
3.93041823 4.098794146 4.496342611-0.996301189 5.09500489 6.485084052
2.723993542 0.217710671 5.70099907 -0.834376573 6.239901806
4.9704114124.79827603 5.3667429116.31149620510.01493206 -0.164149315
2.943646844 1.399193612 -0.376099998 2.6141181210.720888666 4.24241575
6.810469355 3.248552754 5.07442704 -2.367397846 2.909745439 -
0.678670334 3.349396522 3.990719654 -2.504027591 5.354428356

4.289226805	0.586849477	2.879560737	3.88135442	4.977549132
4.656019126	3.153745582	5.335438981	4.45930822	4.812967897
4.293413977	0.453482064	2.753258563	4.275531366	4.670535008
4.788382574	5.722569908	4.398862536	1.183353771	4.745500651
3.24232885	1.421909877	3.792251677	-1.598253383	-4.804843704
2.481761889	-1.214563808	-4.711774191	-4.054479311	0.916120442
2.335653948	3.949969927	5.986005326	3.659453818	6.227680725	-
0.994989648	1.945439526	4.362528106	3.616655785	1.464608026
TCGA-44-5644-01A	-1.045399649	4.922722858	1.647907538	5.084904705
4.437503172	8.333830767	4.173323672	4.09987384	2.741402403
3.504058826	5.895485151	-2.209772229	3.525765296	5.304839306
5.261304304	5.15068062	4.617552919	4.808391407	0.722801203
0.7911875493.033240768	5.361577333	-1.618020005	3.094646467
4.735225151	2.28104521	5.547486937	3.961235509	4.6118175234.279888491
4.619451451	1.395328905	-3.401611188	3.751843424	-0.953193298
2.227750423	-2.7358513	5.600356955	3.39016039	0.618325143	4.697735901
-1.04168173	5.279279514	3.8811371483.980626003	4.385729423	7.259977659
2.1163598285.046129973	6.825600357	-4.274419419	-1.326798965
4.582608785	-1.591875175	3.584982816	-4.344154104	5.425971699
3.003064517	2.238496612	4.21242021	5.893392925	3.496536921
2.324826318	-1.998167697	4.532851406	3.944130784	4.706193484
4.576578803	-2.586775132	5.063101941	6.570005904	3.085747437	-
0.059182148	5.497723014	-0.59232411	6.184633449	5.167738443	5.183063395
5.491490298	6.571803524	10.47350863	0.890522525	3.165318828
1.831982309	-0.784327891	3.559274322	0.555637099	4.273560478
6.892348172	3.821723048	5.14912964	-1.413893271	3.016432755
0.054551677	3.353247595	4.072373029	-2.771601511	5.35228541
4.138746963	1.786691663	3.494218016	4.248879722	5.064350075
4.7118002323.800508928	5.335535088	4.831854727	4.89397464	4.275058015
0.824639796	4.951323401	4.324765536	4.5152827	4.681350905
5.784699161	4.779757574	1.367138021	5.297498275	3.580785016
1.309878545	4.054725238	-0.407480385	-2.936489735	2.844599692	-
0.004710655	-3.240405612	-2.610377751	1.473497902	2.638294905

4.065441504 6.137795883 3.991731261 6.636582777 0.12978975
2.419865236 4.949441329 3.930373468 2.401608555
TCGA-44-5645-01A -0.143919321 4.72933378 2.65843128 5.346913216 4.212725636
8.256727228 3.627805085 4.246619493 1.952382963 3.374214406
4.918303178 -2.419770555 3.129990802 4.835258531 4.970898854
4.749443006 3.6465816 3.899648013 0.382583932 0.43944236 2.75764613
5.538790626 -1.198124078 2.345007621 4.598301734 2.938106509
5.743640288 2.002838346 4.521005776 3.064438714 4.839241277
1.010501838 -3.662420438 3.299849765 -1.195922497 2.714532346 -
3.639124413 5.567142374 3.5961838 1.061456802 4.716267034 -1.114839173
5.096889456 3.155735176 3.947676299 4.290665376 7.060736314
1.551575636 4.006904528 6.734681654 -5.036318496 -1.651110493
4.372280283 -2.61008225 3.509228761 -4.469390475 5.137919067
2.191441809 2.649154978 4.1175903266.028439696 2.633236698
1.721973324 -2.365018988 4.74151393 3.943434254 4.081769629
4.286681134-1.271815823 5.089471046 6.367838285 2.95408583 -0.000432904
6.027092797 -0.18346571 6.000470042 5.216743782 4.549857322
5.440776455 6.400322253 10.40744406 0.283083729 2.736342727
1.090022944 -0.774381391 3.257169929 0.868665577 4.222175638
6.887096177 3.549152654 4.654770129 -1.985335781 2.099539384 -
0.154001419 3.26437244 3.755419224 -1.967701279 5.356308017 5.013803407
1.344409978 3.096925895 4.1187555274.969146208 4.763919785
3.138327491 5.335437092 5.021218282 4.9611678344.2256506110.714696616
3.350724709 4.460395592 4.804488513 4.801940189 5.514242993
5.0356211040.875581698 5.527830139 4.510861414 2.187206632
3.877907723 -0.582037096 -3.580595343 3.390024336 -0.283362739 -
3.601837889 -3.732940847 1.44449933 2.904565105 4.318267762 5.312618012
3.314949599 6.316344155 -0.010793551 2.9691177684.60072543 3.765257072
2.822321859
TCGA-44-6145-01A -0.737730699 4.55221184 2.572392246 5.399326027
4.062718883 8.289505883 2.904740435 4.353568077 1.431989151
3.512321275 5.411411601-2.690102795 3.445151715 4.560236925
4.562070329 5.173423174 4.0112513493.274192516 0.439482108

0.376849344	2.665862486	4.806054603	-1.341428596	2.532620562
4.496396265	2.791657921	5.584407359	2.586158059	4.410666393
2.604018712	4.820466041	0.846445923	-4.117860575	3.305159177	-1.352472583	2.290149226	-3.013378441	5.590881794	3.203482638
0.486136321	4.694122413	-1.357562113	4.940830568	2.921674827
4.035341645	4.243503174	6.914409335	1.277263722	3.340459967
6.606622474	-5.161335307	-1.973608086	4.791151639-2.692261573
3.062095226	-5.171430047	4.964483094	1.635384481	2.391393734
3.725046582	6.154188189	2.53404221 2.265015444	-2.299286744
4.607027883	3.9297115653.740613883	4.371139341-1.411514916	5.079206104
6.246448814	2.845522253	-0.324804158	5.77589626 -0.647893258
6.015756335	5.179579412	4.490429848	5.50866217 6.136380581
10.69715608	1.123136497	2.318477076	0.194235047	-0.526557367
3.744837945	0.9516951154.291506133	6.79699262 3.521350623	4.854458714
-2.144978794	1.858633162	-0.532660367	3.271268833	3.68514042 -2.359239176	5.336528716	4.400751853	1.179390162	3.123717725	4.09343963
4.869873712	4.719140517	2.583628033	5.335073742	4.784232401
4.9311626493.969373474	0.575548063	2.691012215	4.573994802
4.750874543	4.757027409	5.437828444	5.150963467	0.604796213
5.876301633	4.1107123381.873277159	3.767899519	-0.042338852	-4.1119146132.366328001	-0.368607034	-3.650819542	-3.966916717	1.666668992
2.745196286	4.382396996	4.988207583	3.045277794	5.864992214	-0.660514072	3.052387412	4.408730213	3.522555923	3.09957673
TCGA-44-6146-01A -1.213393834	4.436377042	2.30535719 5.532803878
4.4611458518.413765968	2.751305151	4.319976962	1.627283322
2.862074879	5.595439338	-2.619890771	3.236770261	4.889347739
4.142795253	5.462169225	3.9901186713.007363819	1.19237348 0.594593963
2.51190549 4.759833801	-1.483020297	1.998312299	4.500623422
3.009573723	5.370078493	1.919835783	4.249712431	1.987672012
4.70636994 0.455634551	-4.133370582	3.781786329	-1.2763171 2.54251597 -3.128603561	5.604431569	4.631510427	1.35086184 4.699869207	-1.393096591
4.755989449	3.148170443	4.060289895	4.287012707	7.010497566
1.364755048	3.558686916	6.342568466	-4.480053689	-1.99352566

5.182782568 -2.364080341 2.893832812 -5.054275817 4.84447832
1.392587268 2.129496448 3.808846826 6.09724179 2.756787659
1.870204194 -3.08053002 4.469769279 3.94448866 3.49567481 4.345638241 -
1.41803742 5.061299129 5.974429848 3.059779032 -0.456650682 6.004925304
-0.807091466 6.026483981 5.41763176 4.20931779 5.480093355 6.088651826
10.81884953 1.3379711982.496418194 0.115584937-1.238631974 3.023996785
0.794002894 4.353814188 6.690139182 3.930551552 4.752797129 -
2.03150958 1.748202879 -0.236006831 3.158948933 3.091506971 -2.647188814
5.35768317 4.398262625 0.794218127 2.831240093 4.682371017
4.834890883 4.856667697 2.517420856 5.334822073 5.227065157
4.687471051 3.879719458 0.172619641 2.671536451 4.321490696
4.528651543 4.761916924 5.684265198 5.313602958 0.434001069
5.970052108 4.706240304 1.796269418 3.789663491 -0.112510196 -
4.144815551 2.183988506 0.161712798 -2.976345518 -4.101399701
1.860628796 2.660498631 4.520141641 4.926384736 3.577141992
5.925300533 -0.105053277 3.546940838 4.534026677 3.610525477
2.948596951
TCGA-44-6147-01A -0.68777647 4.789023948 2.298600443 5.3154116284.255126041
8.281643661 3.653817415 4.189057754 1.955320426 3.413103409
5.144066289 -2.2644429 3.476203633 4.4475311044.857900946 4.780944822
3.890000189 4.082025282 0.829770592 0.5287211292.712062399
5.393052042 -1.365587237 2.178520661 4.586107146 2.881421952
5.614176975 2.183275581 4.56330069 3.591598935 4.897561379 1.04686885
-3.768980333 3.493346151 -1.115358827 3.016209367 -3.192074748
5.578473816 3.434810107 1.334887441 4.707666917 -1.146122786
5.191872413 3.237691233 3.972480025 4.293278719 6.909623215
1.919779417 3.604218903 6.837481483 -4.716352684 -1.740886654
4.372449598 -2.453986196 3.463034616 -4.637133602 5.110555765
2.163549801 2.275843388 4.13637733 6.007962054 2.783652384
1.580314452 -2.002883812 4.670813015 3.930826558 4.053086167
4.297662591 -2.000153481 5.087892394 6.443302798 2.941835939
0.047892328 5.836588624 -0.433197403 6.003124429 5.150696263
4.591207456 5.4073361116.400120913 10.4827379 0.910206719 2.586369809

1.023681385 -0.739119472 3.452060062 0.786671263 4.250120733
6.928422172 3.588609184 4.755965721 -1.785047594 2.096246228 -
0.2011140253.286136563 3.861105203-2.325289926 5.342560016 4.814799872
1.417571843 3.264616671 3.9941162394.915537093 4.692594054 3.20877066
5.335391736 4.964682909 4.914903053 4.197507808 0.630741101
3.518229707 4.484650759 4.753816766 4.771021051 5.650276017
5.105023729 1.01510663 5.435638297 4.2765102 1.97769097 3.873616019 -
0.423295909 -3.994123188 3.051022129 -0.38991768 -3.44231728 -3.567195823
1.437277815 2.755647761 4.248565898 5.180782267 3.300544519
6.142896543 0.09964155 3.0336856 4.412025844 3.738218718 3.013916263
TCGA-44-6148-01A -0.707984131 4.720321633 2.369783665 5.27005189
3.959760791 8.307782545 3.964123015 4.165316025 2.255771379
3.607473383 4.611527181-2.092106357 2.82361719 4.545982155 4.86175276
4.468188191 3.633472443 4.269774947 0.367385497 0.342015926
2.904489148 5.716866674 -1.438781522 2.27969371 4.628465729
2.821988053 5.638432812 2.770619877 4.562306134 3.238598437
4.856852776 1.05568991 -3.668474797 3.298526608 -1.262242578
2.714966173 -3.974210555 5.557089219 3.829600295 0.615705777
4.722864935 -1.054079591 5.206474014 2.969499825 3.905118756
4.243141648 7.028212587 1.5420886113.874355768 6.934558876 -
4.987315098 -1.377772005 4.13545395 -2.801223514 3.769474252 -4.230568762
5.065906424 2.148171668 2.591040817 4.20610257 5.96643059 2.784469439
1.481018518 -1.440805674 4.697747152 3.932034159 4.335903234
4.103574408 -2.037479575 5.096388573 6.566228782 3.027670859
0.127287235 5.980622352 -0.188799624 5.883294166 5.246175255
4.643598161 5.332229458 6.467719921 10.07236505 0.533720345
2.6225711281.197652118-0.714033168 3.018715756 0.663162026 4.196547563
6.94290898 3.594263738 4.383530493 -2.068286709 1.795954744
0.100906941 3.292383189 3.968323667 -2.44533064 5.332299305
5.329139659 1.328958716 3.0987911454.1145517394.93213688 4.687687689
3.379277985 5.335577498 5.239844831 4.928698198 4.44546414
0.606177506 3.557935392 4.382672714 4.762050766 4.803511591
5.550684822 5.171801201 1.063109126 5.380139292 4.368822688

2.1156412553.882493259 -1.058460846 -3.607986835 3.650512406 -
0.746856239 -3.663026296 -3.557722495 1.2582921112.745292569 3.88765524
5.161677104 3.343166557 6.146618906 -0.175632166 2.90786664
4.533223762 3.825563466 2.460014631
TCGA-44-6774-01A -0.67088301 4.203700391 1.850451888 5.521360652
3.859824095 8.446128325 3.725820042 4.265845079 1.732026449
3.925219084 5.51918631 -2.313625976 3.266958613 4.514949751
4.620736618 5.029063442 3.964126181 3.988302165 0.661116011
0.567065982 2.699562444 5.511782888-1.603167982 3.380166305
4.551422705 2.8483820115.501362385 2.229903685 4.502266644
3.340887199 4.929672969 1.383285263 -3.759048163 4.157411594-
0.910707532 2.164911563-3.106114007 5.584038286 4.004912642 0.971120765
4.713287116-1.298324621 5.234037485 3.165810846 3.948280531
4.245985254 6.863352806 1.515750376 2.892243397 6.950419845 -
4.897897519 -1.583992401 4.626896072 -2.7572479 3.42275282 -4.039614123
4.963027365 2.263864092 2.419708934 3.958436799 6.190843256
2.905050569 2.73440027 -1.418290109 4.750639347 3.93583192 4.161444073
4.246192948 -2.727804981 5.08847318 6.53191138 2.687364002 0.103609973
5.904266313 -0.553515392 5.897935539 5.203581288 4.675218192
5.468495614 6.456142851 11.428734271.081309321 2.622946109
0.791228072 -0.454644953 3.780059491 0.975709517 4.281309427
6.868910765 3.607455738 4.630182118-1.913500871 1.935733513 -
0.249040383 3.362650359 4.012463923 -2.586245912 5.327067105
4.979982373 1.784634197 3.393620066 3.895668894 4.943259544
4.754125817 2.898765309 5.335538035 5.099164421 5.070125817
4.29488075 0.681568421 3.41995715 4.332053484 4.737290847 4.760892536
5.45641175 5.201283505 1.006750229 5.665480537 3.91383379 2.034237816
3.703332367 -0.037628923 -3.941173871 3.671849797 -0.423906355 -
3.466321079 -2.912100909 1.669602776 2.665305603 4.289400869 4.40457477
2.907067079 5.971781094 0.356334746 3.078963777 4.239216566
3.676917899 3.003121852
TCGA-44-6775-01A -0.331699671 4.499850831 1.68790063 5.305867144
4.400973447 8.333212158 3.447525941 4.285776503 1.705916827

3.580754103	5.346372922	-2.365921743	3.446963495	4.91303797	4.87535504
5.005797698	4.012159539	3.6598111090.489599729	0.510737336
2.683573577	5.585505515	-1.180099956	2.757265756	4.562409837
2.833845482	5.463849454	1.586378445	4.559799395	3.160367219
4.943801478	1.188596684	-3.702164145	3.621673429	-0.76593651
2.541429842	-3.347374836	5.588778062	3.895606163	1.554303283
4.709691625	-1.216290928	5.1404818113.130655505	3.939597451	4.33354781
6.885686058	1.425233013	3.40551713	6.816158509	-4.827645598	-
1.743202201	4.738835226	-2.669278765	3.671535505	-4.277056035
5.086210566	2.002585675	2.791087699	4.082255277	6.143267068
2.586133419	2.088292412	-1.848973102	4.71280799	3.940154629
4.000843398	4.222343466	-1.401019462	5.077247199	6.372930725
2.818320021	0.024929938	5.809179673	-0.250084846	5.934155263
5.1109152234.509179109	5.4375113296.345194168	10.92580976	0.798867175
2.880094345	0.730337932	-0.551032816	3.412066772	1.032077382
4.1825462	6.8253115543.445028276	4.887496206	-2.017208185	2.142043133
0.086328678	3.301393584	3.727623855	-1.8446711	5.331860477
4.820789125	1.578102028	3.582574721	3.844086343	4.916381815
4.808412106	2.99095623	5.335292279	4.802724027	5.158769839
4.254814324	0.775540776	3.16129554	4.454099017	4.814775865
4.787827897	5.615377493	5.1311662930.726131908	5.612510798
4.498526354	2.254486099	3.811419572-0.188538375	-3.357392495
3.433675272	-0.017584717	-3.511379045	-3.265637195	1.561235225
2.810065618	4.481381666	4.984468101	3.237334372	6.213438334
0.25340555	2.913967981	4.422432557	3.7255511413.512501267
TCGA-44-6776-01A -1.133584909	4.692719252	3.057099457	5.442061463
4.477107803	8.331252576	3.128644086	4.255805594	2.030745355
3.008881616	5.252121839	-2.088439471	3.466303187	4.465322233
4.394401589	5.305092097	3.81202026	4.140307936	0.800370522
0.507635493	2.775055819	4.844406848	-1.449818569	1.993265568
4.545124403	3.377964577	5.462016281	4.527030077	4.440298031
3.123139973	5.036008027	1.027674314	-3.937401197	3.488720566	-
1.391728408	3.126133428	-2.775640042	5.603177388	3.475274243

1.338101356 4.703846745 -1.302487066 5.1121385343.242964437
4.033769604 4.268728498 7.0147117251.667735475 3.773352583
6.769377907 -4.55855385 -1.572696171 4.629234242 -2.581781093
3.331545818 -4.511248856 5.021464194 2.351227741 2.578787004
3.851092681 6.062233638 2.797034553 1.68018434 -1.826941483 4.49286721
3.935057876 4.331429577 4.016568845 -2.18333109 5.06924466 6.411623617
3.023346678 -0.161247818 5.786463632 -0.923314807 6.120821499
5.199172028 4.734714928 5.44598961 6.299847981 10.2774293 1.566383178
2.455561376 0.693852973 -0.859162726 3.0097611530.851788235
4.393574225 6.883874565 3.880409073 4.6507274 -2.028549393
2.136770644 0.101419109 3.305515256 3.794370587 -2.597496684
5.358989582 4.365690038 0.718027606 2.565383786 4.094325489
4.980654841 4.635069421 3.500619133 5.33528366 5.086794276
4.846139085 4.508807544 0.377356537 3.802196452 4.675394066
4.68496155 4.70683282 5.8611708475.50981708 1.15121285 5.677776248
4.402995945 1.629578434 3.928943481 -1.029075422 -3.818557423
2.662882929 -0.207720161 -3.269280551 -3.494105067 2.01953637
2.818702361 4.527409612 5.1156757463.42238895 6.117738449-0.152832881
3.361889932 4.654245431 3.6924069112.552460163
TCGA-44-6777-01A -0.416305428 4.491967278 2.490928663 5.589184983
4.053953 8.406777462 3.423778468 4.362043389 1.715445358
3.806597671 5.611542232-2.371200541 3.437978717 4.475995698
4.894047962 5.0867886113.510729451 3.788441397 0.703736704
0.665423419 2.63604451 5.465429742 -1.339049295 3.465828282
4.492670391 3.06261756 5.534090516 2.376644325 4.539198881
3.406577994 4.863241236 1.23229514 -3.632191618 4.158533368 -
0.823039757 2.308667773 -2.614830525 5.5869211743.839830668 1.35278062
4.722486573 -1.24068907 5.432393432 2.754264415 4.05571971 4.309016417
6.909960931 1.435237353 2.797989288 6.673016136 -4.635917061 -
1.764813379 4.474286397 -2.697742358 3.383137479 -4.043179801
4.9369028112.003697607 2.542667602 3.932618727 6.292812173 2.92718037
2.752040061 -1.4331471 4.703202015 3.939688286 4.082266569
4.261077719 -1.857997698 5.105875335 6.423775979 2.733214534 -

0.095174333 6.077867959 -0.306157261 5.905646782 5.175227012
4.418991919 5.515663927 6.354000456 11.479121211.456778407
2.648213191 0.726980811-0.441253651 3.72096203 1.173525143 4.290953731
6.847437049 3.620983615 4.610825767 -1.966809184 2.126878798 -
0.013586415 3.344104597 3.969748448 -2.373188911 5.328248143
5.137745478 1.434918377 3.450671694 4.141816729 4.951498871
4.797349056 2.586176244 5.335638882 5.157748627 5.023994853
4.4718603110.563189414 2.91603907 4.4807117894.738181653 4.80218715
5.507834051 5.120669532 0.8621146695.789913007 4.307830319
2.353936058 3.693852892 0.007468023 -3.698942029 3.523565325 -
0.340003854 -3.355000182 -2.821549072 1.94317881 2.922076636 4.634006363
4.562516903 2.946125817 5.740450895 0.450631698 3.289052121
4.280038797 3.681443499 3.421773748
TCGA-44-6778-01A -0.453018488 4.835322846 1.319177827 5.0621166834.18854148
8.272683924 4.323506288 4.180147498 2.509869874 4.474113807
4.759697211-1.812273042 3.282262759 5.261073374 5.198582326
4.240662341 4.175386441 4.428984087 0.891360363 0.418036503
2.833129409 6.112311677-1.576137409 3.279942105 4.728212241
2.394172724 6.039374986 3.040895622 4.644228265 4.172949371
4.689943995 1.397215571 -3.412079586 3.600026079 -0.67070899
2.177626461 -4.039422441 5.554294129 3.289735018 1.105788232
4.693785463 -1.043226697 5.512240823 3.744754078 3.799266224
4.475037642 6.903706593 1.916995747 4.353383949 7.059175799 -
5.038630149 -1.15796143 4.264791452 -2.175194799 4.418846489 -4.200773118
5.180236624 2.253729227 2.85770177 4.417947955 6.030158829
3.070451744 2.030640811-1.559329894 4.704301924 3.934809355
4.210902458 4.47593878 -2.49728754 5.079718368 6.593545475 2.877061799
0.302412791 5.932732209 0.043134219 5.901673209 5.118368972
4.839250783 5.27996879 6.708958194 10.37710815 0.1142919583.130679808
1.576172424 -0.506263804 4.217995588 0.621331384 4.020601566
6.8993110023.344458576 4.868712865 -1.768703455 2.390061577
0.496013278 3.35161847 4.290022603 -2.334737149 5.331799473
5.471822522 2.435184243 3.84319283 3.60745001 4.962716518 4.605253455

3.4529411045.335557634 4.8463395 5.154648182 4.209320199 0.611974682
4.368128217 4.5811541164.671844553 4.803165527 5.518401958
4.783446905 1.247906464 5.227712334 4.147015738 1.898780408
3.908560497 -0.370176272 -3.053166854 4.058602698 -0.433616574 -
3.768947963 -3.313179114 1.077272904 2.449793512 3.6791127155.646142552
3.44804713 6.28905021 0.650178124 2.251038078 4.612150999 3.836023728
2.665802525
TCGA-44-6779-01A -0.758980006 4.351810877 2.404845744 5.461477843
4.272984359 8.310608642 2.513995579 4.421714686 1.182595552
3.31261069 5.586521875 -2.998605156 3.467326863 4.489560684
4.194216793 5.274075478 3.58027226 3.049233905 0.15047044 0.499088419
2.435524341 4.434071079 -1.217940146 2.354992148 4.384109483
3.058172673 5.58594578 2.201972643 4.416158951 2.7644133114.953058746
0.81002891 -3.939948235 3.155433041 -1.269413945 2.395556142 -
2.659007501 5.6161077113.270135603 0.733554667 4.699003264 -1.396349353
5.106953421 2.446687298 4.088623792 4.189936649 6.963746198
1.068830508 2.922689336 6.306438564 -4.974435418 -2.293369542
5.306648392 -2.883935213 2.926850831 -5.287383456 4.793423248
1.739400889 2.586397038 3.433635779 6.20504727 2.334852797 2.19649991
-2.814937886 4.561936568 3.936404042 3.495854889 4.230154968 -
1.180232881 5.080280752 6.1503112772.723912132 -0.284408115 5.650369198
-0.687361727 5.996655518 5.129941995 4.436147839 5.506245427
6.054666409 11.580746571.297469058 2.428001736 0.027538681 -
0.482562028 3.307690481 1.268713734 4.280885043 6.749550633
3.5091152194.832805554 -2.257794792 1.938627243 -0.725420409
3.221547819 3.440874177 -2.223145766 5.339953313 4.108633347
0.6119278072.91250144 3.893258698 4.902170602 4.890142399 2.576266573
5.335128971 4.49970754 5.087221021 3.724053327 0.628459923
1.945844672 4.573410092 4.818748844 4.750362081 5.567070352
4.961564188 0.367825582 5.903080776 4.205279374 1.998827094
3.739865628 -0.158083951 -4.771081387 2.304345594 0.14483526 -
3.438699724 -3.616752757 2.069485502 2.797374875 4.75793448 4.754602909
2.98355905 5.817480329 -0.46896978 3.051286132 4.250492308 3.463310016

3.297899785

TCGA-44-7659-01A -0.70254689 4.878111256 3.131498687 5.393773995 4.32045426 8.254112036 3.655969219 4.272399309 1.953819518 2.967327693 5.058833122 -2.202517845 3.296848661 4.546105935 4.786038431 4.928908702 3.376263886 4.066055563 0.337819427 0.432929971 2.621980103 5.27657675 -1.30801758 1.836345114 4.609543598 2.98743865 5.678226264 2.303755895 4.46364352 3.399663412 4.846186458 0.864809729 -3.676410913 3.261809934 -1.074436283 2.839392193 -3.334267573 5.5926331 3.450631467 0.747230062 4.707968648 -1.178836893 5.050454734 3.038050819 4.006055357 4.224767665 6.937632144 1.445459126 4.129300755 6.686608356 -4.792195718 -1.681503538 4.747842727 -2.319999133 3.329600159 -4.639262719 5.046904644 1.998877361 2.428801104 4.061608507 5.934973975 2.688935862 1.719806997 -2.205432061 4.61445281 3.939048424 4.123854985 4.198219963 -1.469786809 5.083492574 6.302721751 3.014763697 -0.122941667 5.977952399 -0.612368118 6.103282718 5.132557746 4.623495666 5.456945581 6.449221155 10.37841127 0.394679954 2.745064392 0.866738877 -0.954062002 2.938762118 0.809110185 4.307976573 6.88525092 3.642498604 4.609499712 -1.995830028 2.260342061 -0.100793521 3.221677858 3.691952126 -2.199621339 5.356399422 4.735055873 0.76471702 2.696064853 4.083824391 5.003952518 4.86441474 3.350883832 5.335459281 5.042823832 4.814577924 4.216959643 0.550800238 2.86126394 4.556511617 4.749315223 4.749664272 5.671993827 5.103665969 0.944772735 5.509806361 4.47858946 1.942936984 3.93252601 -0.843253729 -3.809723451 2.917678734 -0.208153987 -3.497025127 -3.728121166 1.576185771 2.872345418 4.313211877 5.371395254 3.500065909 6.245284268 -0.879480524 2.94368364 4.44102609 3.686570328 2.545514844

TCGA-44-7660-01A -1.078681696 5.047547412 1.067576589 5.272380063 4.31343344 8.104173167 3.955786828 4.053335944 2.54722017 4.20040661 5.15634047 -1.549544585 2.706875214 5.01495648 4.878185438 4.47982687 4.860280835 4.353420961 0.074909759 0.221071589 2.920266635 5.734809361 -1.554084469 1.915533816 4.792110101 2.083592684 5.936867769 2.30424113 4.608975178 4.290565591 4.911542088 1.900364211-

3.676560682	2.744086418	-0.570885119	1.976169329	-4.553543305	5.59824257
3.090262928	0.020264239	4.660645792	-1.198463062	5.132025239
3.88059949	3.616331967	4.37322339	6.893363343	1.361837085	4.321063986
7.357155189	-5.847014709	-1.078989973	4.971714717	-2.499560527
4.120906861	-4.111945654	5.276497904	1.8726343	3.133965251
4.273131387	5.773206505	2.308461431	1.615404603	-1.400896884
4.560659407	3.91787508	4.26218044	4.245015839	-1.542299229	5.068328227
6.648687433	2.912459563	0.310617452	5.663950141	-0.748187438
6.165167296	4.930773265	5.078987094	5.263383278	6.340238659
10.0070546	0.401374738	3.0081132571.407276647	-0.474036563	2.616327791
0.497534188	4.163859261	6.896526147	3.186346129	5.245903953	-
2.034178334	2.453484371	-0.102661668	3.38612973	4.080796002	-2.375529178
5.329405457	4.420158505	1.821893602	3.489275866	3.302176936
4.91892538	4.63578421	3.596423143	5.335049529	4.292191539	4.953360681
4.457805574	0.387868224	3.340544687	4.346654037	4.564766277
4.745873101	5.57725666	4.720126139	1.541422853	4.988623313
3.887933681	1.528669138	3.874036564	-1.17440835 -3.701293093
3.656965043	-0.580450536	-4.247252239	-3.587552753	0.994331924
2.010066381	3.744022457	5.762252345	3.568227217	6.204391263	-
0.687716402	2.132914741	4.487372459	3.851264968	2.27496808
TCGA-44-7661-01A -0.755343966	4.541835889	1.799052207	5.454883304
3.939451935	8.273017533	3.423077745	4.366751557	1.555680456
3.68345962	5.53098538	-2.615465581	3.55274422	4.586998765	4.704736683
4.742271736	3.426821005	3.535474814	0.301754492	0.349608265
2.381246873	5.176925421	-1.539581644	2.528131353	4.537272346
2.548794016	5.824916601	0.807793935	4.483194933	3.433760473
4.883647859	0.99199715	-3.686654669	3.506351053	-0.744157399
1.851609667	-3.532140301	5.604989694	3.542397626	0.529857046
4.715398366	-1.153265702	4.951403294	2.365846179	3.924630092
4.199546024	6.85168029	1.089468529	3.095454131	6.832853828	-
5.057652157	-2.01974575 5.163432767	-2.464505125	3.214836598	-4.827578141
5.0112567961.834493729	2.51318247	3.676815659	6.020889143	2.269861118
2.211574451-2.107777374	4.727763043	3.939557332	3.543847465

4.367859828 -2.116702345 5.092568631 6.26778383 2.654449899 -
0.019552285 5.847794856 -0.531919374 5.993262311 4.975243927 4.647764006
5.493620293 6.279293734 11.0614738 0.471127476 2.747232509 0.582781046
-0.447334226 3.352846114 1.103865178 4.28915197 6.786838774 3.118051391
4.842232382 -2.215607608 2.135435128 -0.505717712 3.256966177
3.765831364 -2.808159379 5.329396175 4.650181265 0.798347539
3.118560314 3.660889913 4.952051987 4.911523314 2.635622691 5.335580211
4.417662696 5.077931251 3.75727582 0.635047822 1.490382737
4.419234216 4.850971278 4.78978478 5.379200244 4.549466132
0.684560269 5.373875915 4.157816047 1.925355615 3.783312974 -
0.347322098 -4.368936028 2.833995776 -0.268136932 -3.61169378 -3.35298559
1.491441043 2.608543116 4.275158572 4.801007184 2.956623551
5.820404337 -0.303219195 2.604761097 4.289150986 3.51239001
3.257039504
TCGA-44-7662-01A -0.312059198 4.518599324 1.74290705 5.481768585
4.322183505 8.233887349 3.423803463 4.232409869 1.706066979
3.582690675 5.458524898 -2.419785632 3.871967767 4.530758735
4.566262177 5.25337795 4.084017733 4.018137284 0.167644616
0.438942832 2.663001794 5.300921934 -1.248577663 2.213863265
4.58115721 2.801803805 5.527813472 2.177709599 4.566597598
4.063015918 5.086143368 1.404217396 -3.661325938 3.368501008 -
0.739231838 2.488337802 -3.107759423 5.618613346 3.55112831 0.676853738
4.702783248 -1.24661739 5.235712724 2.918151071 3.921981272
4.157492529 6.846507205 1.585870885 3.403632627 7.125426892 -
4.880311504 -1.87712071 5.28573088 -2.549176181 3.425624195 -4.656112766
5.091580173 2.341065534 2.787470711 3.763888596 6.066987449
2.545909742 2.185092651 -1.615531413 4.694498588 3.929612029
3.961994381 4.35880696 -1.437883461 5.077629022 6.436935013
2.673117624 0.08486575 5.470553665 -0.508605379 6.065431076 4.978323298
4.900651939 5.485121616 6.320563084 11.14633846 0.615584708
2.777215708 0.720338258 -0.432859988 3.369615085 0.984360153
4.359781031 6.845213623 3.431181245 4.982545206 -1.882676603
2.343482748 -0.390271784 3.306503659 3.792457301 -2.254794256

5.337338971	4.324157349	1.284658688	3.165182548	3.601682777
4.978690568	4.892061566	3.077217331	5.335426085	4.474022609
5.1099211583.928804405	0.929059046	3.00403872	4.4651120454.891347938
4.727032281	5.653465003	5.0793006110.81326708	5.403564462	3.797603465
1.942716799	3.883042517	-0.445601974	-3.831214403	2.806489475
0.042936405	-3.691034917	-3.135964502	1.609478674	2.720017313
4.503170142	4.920841021	3.375417548	6.108973607	-0.070288351
2.76801902	4.358442513	3.52197234	3.176261507
TCGA-44-7667-01A -1.090998953	5.053887329	1.319864158	5.323983243
4.215708839	8.191937335	4.786930745	4.087383995	2.958869086
4.679335882	5.271484652	-1.120255671	3.429306406	5.306804656
5.129001858	4.256193269	4.561940426	5.556833183	0.379199756
0.368645755	2.978089882	5.969884256	-1.874619813	2.706256725
4.814849623	1.812907397	5.7897411813.637005043	4.876586319
6.294296252	4.976557683	2.190862878	-3.211019075	3.370395464	-
0.276368288	2.179579025	-4.358370481	5.60591932	3.062385827	-0.047357626
4.678309597	-0.88852493	5.256785173	3.975816304	3.5735481164.273082619
6.854621403	1.637183753	5.188431937	7.813493624	-5.412844902	-
0.898031764	4.745808619	-1.876806936	4.681352482	-4.147074672
5.440370746	2.759428429	3.148340431	4.14172629	5.602364473
2.933060465	2.528659679	-0.769384825	4.66603033	3.929936157
4.855710391	4.499539358	-3.392470185	5.065706716	6.787084655
2.754215316	0.653958769	5.363805067	-0.653395517	6.229034881
4.8009050115.546661387	5.356232493	6.817566746	10.02548126	-
0.134641462	3.399338622	2.373130612	-0.337168971	3.30298714	0.419517498
4.194991878	6.97158119	3.315872268	5.184793438	-1.693213467
3.396149676	0.577520302	3.5113300084.542047734	-3.011253928
5.343138532	4.35844946	1.780406735	3.25588868	3.010938837	5.132546015
4.625305392	4.381724242	5.3359311	4.3061511625.016454745	4.645128781
0.871663885	4.448163152	4.402479978	4.666786931	4.688438269
5.6511749524.408232963	1.912994695	4.4117301692.879877225	1.065081905
3.975197689	-1.372324156	-3.616728116	3.714354753	-0.698777004	-
4.220870439	-3.039889344	0.866492337	1.969946489	3.449265429	6.01465151

3.881677042 6.796644713 -0.670396362 1.591582362 4.853943598
3.8931130761.525037739
TCGA-44-7669-01A -0.963167671 4.989158971 0.529975668 5.069785166
4.266324851 8.271419375 4.529531527 4.1176193162.721747805
4.510790378 5.197812942 -1.434930556 3.146654466 5.749090161
5.237128304 4.375969715 4.927146203 4.740824258 0.668447698
0.263405746 2.933712746 5.770655339 -1.864977756 3.152669082
4.814901573 1.767815731 6.041550949 3.238489216 4.689957025
4.864777792 4.734447381 1.859388073 -3.373052424 3.568529962 -
0.589042721 1.836280215 -4.294913984 5.580788536 3.884713066
0.481391651 4.673190778 -0.98106854 5.256054684 4.532758816
3.697242748 4.463644504 6.824477784 1.824274208 4.868338243
7.057130853 -5.331148893 -0.949164089 4.712218997 -1.790659223
4.321053661 -3.926703039 5.374235037 2.41249991 2.45938625 4.370829969
5.848577191 3.073861533 2.543289942 -1.543213002 4.611707669
3.935688141 4.304095879 4.544945121 -2.951903113 5.061230453
6.708829053 2.91154164 0.248730646 5.784886107 -0.270719679
6.099225124 5.037661991 5.155221207 5.261267053 6.636724985
10.00541796 -0.117999885 3.479149262 2.083050951 -0.592793474
3.989562602 0.410948888 4.022938588 6.916750237 3.411190083
5.238874187 -1.761534602 2.896314435 0.334156617 3.423321574
4.243463 -2.771156666 5.325932898 4.614962733 2.792031476
3.9112649833.221333313 5.003249062 4.65389669 3.749738855 5.335532844
4.674441071 5.09321909 4.249343044 0.539691274 4.967312197
4.360353538 4.568894779 4.747258217 5.653121669 4.741494405
1.538579876 5.060988152 3.962687331 1.447039787 3.933488705 -
0.857511773-2.885221799 3.755121091 -0.06150263 -3.996141684 -3.358682273
0.953433324 2.070764943 3.381828354 5.845803963 4.034034118
6.517450396 0.245513185 1.712015713 4.669103183 3.951136367
1.958545328
TCGA-44-7670-01A -1.230293844 4.892420021 1.748774149 5.278579952
4.096939072 8.198174341 3.9622661 4.141072746 2.263462217 4.01957242
5.393223573 -1.999252489 3.323131896 4.904332466 4.860992877

4.681627413 4.518273895 4.340823929 -0.150881652 0.23162241
3.045268564 5.181439114-1.510040136 2.721882223 4.60768579 2.311241915
5.603674215 2.716655901 4.672239617 4.627341248 4.990652901
1.532405785 -3.361126004 3.102343075 -0.862820478 1.960996562 -
4.494885177 5.6004776113.586785987 -0.151210354 4.686757697 -1.151064784
5.217233093 3.337988162 3.725069474 4.23628825 7.0611274131.212233645
4.169828984 7.214202423 -5.319908232 -1.797291953 4.649770004 -
2.531613847 3.847109162 -4.549478335 5.128326475 1.899984462
2.381641953 3.8839209115.889148052 2.783066352 2.627824749 -
1.579451652 4.539652294 3.92925898 4.095143379 4.428715741 -2.056697299
5.082210034 6.452748868 2.774120929 0.228892152 5.533997234 -
0.871889507 6.126821051 4.992603829 5.000253252 5.4611497016.411986662
10.05754945 0.5322311013.002961556 1.183826381 -0.705406817
3.214020026 0.633160407 4.267847378 6.806143575 3.52948797
5.075022477 -2.062240171 2.476513423 0.1511167623.381253085
3.820484204 -2.602132167 5.338657464 4.161848102 1.616770641
3.228940423 3.885470049 4.984077012 4.595364979 3.74793442
5.335516247 4.738360714 4.921718053 4.307348689 0.5791911223.82449024
4.533187217 4.724755525 4.7181133125.831635071 5.193491964
1.208637521 5.275186127 3.560862974 1.620144558 3.895340912 -
1.318183263 -3.705640033 2.960828353 -0.561483622 -4.03161437-3.352653164
1.2111441232.450561015 4.007945797 5.565991923 3.682145408
6.270396969 -0.331400364 2.27236446 4.51698364 3.757656604 2.254531489
TCGA-44-7671-01A -1.248581568 4.521785231 3.363247569 5.582558462
4.124624152 8.347013953 3.1377118844.319630754 1.791666788
3.193872471 5.549890609 -2.148555349 3.542621499 4.341472499
4.360594948 5.281016877 3.852773817 4.178429842 0.586697333
0.428936962 2.720513102 4.78574476 -1.656699634 2.386675391
4.4917951143.164048864 5.408799547 3.8461482 4.412278028 3.19267046
4.962595847 1.206788659 -4.007628586 3.677872563 -1.456505538
2.584671198-2.721745826 5.603530745 3.17151995 0.873070631 4.707852438
-1.319770002 4.770416499 3.153579852 4.057050452 4.200860257
6.892363879 1.441063069 3.430351905 6.567776226 -4.931295349 -

1.679448746 4.678926788 -2.577705351 3.237928859 -4.567238785
4.9889411852.16305236 2.160654054 3.680004898 6.087880133 2.750195843
2.30632724 -1.664719601 4.543175417 3.9361132964.261344094 4.06396313 -
2.829260055 5.075386679 6.431568124 2.89544244 -0.198620309 5.950117896-
1.065287743 6.1147100465.138763077 4.627633966 5.5357116056.310358174
10.59262173 1.339853749 2.33562295 0.547296811-0.690998216 3.112506825
0.878933585 4.399924655 6.87947964 3.833183013 4.686260106 -
2.146987297 2.013855678 -0.053771257 3.347849923 3.872945816 -
2.862667061 5.353380676 4.272186017 0.826726638 2.677568459
4.213549671 4.959432053 4.660616087 3.217531972 5.335372399
5.029093997 4.786502296 4.479974363 0.464797761 3.200368494
4.599076209 4.676062589 4.693374235 5.751683845 5.476543582
1.168123296 5.72928187 4.11036292 1.580930832 3.791763701 -0.777350569
-4.151191604 2.725684188 -0.424551964 -3.441847533 -3.595502876
1.959651795 2.699249158 4.539362014 4.771041931 3.171384527
6.093714979 -0.968113835 3.156210178 4.421695426 3.656509587
2.55004733
TCGA-44-7672-01A -0.473581823 4.653261965 2.4259534 5.402612391
3.982349949 8.329315077 3.552042981 4.343194471 1.699927177
3.617415875 5.620344148 -2.45767463 3.835739925 4.367684128
4.862033243 5.062459548 3.665129422 4.108554551 0.204310197
0.514335591 2.753787645 5.176380536 -1.26190866 2.858588041
4.507493416 2.818730291 5.764307887 2.01830443 4.599202559
3.657168457 4.961847604 1.09175913 -3.542706985 3.560918394 -
0.981279822 2.334224804 -3.120111156 5.596436608 3.266809099
0.288484176 4.719110551-1.133085095 5.192986246 2.532181666 4.03185514
4.253546472 7.020824606 1.396963425 3.437137239 6.925616148 -
4.796368541 -1.918535508 4.67073263 -2.540425784 3.423970454 -4.573158102
5.100775871 2.267916031 2.25901048 3.879812882 6.199388009
2.7479811262.52052019 -1.675781662 4.74184227 3.934335877 4.085391403
4.313731279 -2.05981456 5.096245226 6.435831602 2.79043662 -0.060434984
5.938871821 -0.348540959 6.001363532 5.068017294 4.740071868
5.551466403 6.307333305 10.73594928 1.232640826 2.652123968

0.816099665	-0.47622803 3.77941936 1.077560991	4.330821202	6.817565847
3.516191513	4.809908393	-1.961843208	2.26308455 -0.279501642
3.303625402	3.867628919	-2.581562534	5.341762301	4.629815183
1.061980285	3.266355389	4.062431993	5.004386917	4.730136545
2.988569049	5.335637767	4.926244475	5.051067048	4.317384575
0.85080075 2.586788079	4.604756559	4.856564194	4.753457384	5.64129362
5.037126604	0.9561162375.632733346	4.009643668	1.951699793
3.868655657	-0.376115985	-4.033680525	3.045140255	-0.322514482	-
3.585347586	-3.048481543	1.76102956 2.93297213 4.609257459	5.0427326
3.177030735	6.076689424	-0.302702157	2.898510095	4.497429215
3.693105553	3.263720924

TCGA-44-8117-01A -0.714893188	4.753608727	2.220459133	5.383670514
4.675049992	8.202600274	3.13143645 4.175228196	1.850405339
3.158145888	5.466079754	-2.392134596	3.38608207 4.865191676
4.806598606	5.206752109	3.779096451	3.604514807	0.243717035
0.632554896	2.669122791	5.321263889	-1.393401004	2.164831546
4.6131717 3.3113966685.339418291	1.968410908	4.443524059	3.666990661
5.018397492	1.204599326	-3.586022421	3.070721031	-0.872943778
2.844361839	-2.99808054 5.602973239	3.712345515	1.625463713
4.709841488	-1.282195698	5.336362105	3.1144420153.944201516
4.173560343	7.057189174	1.633873907	3.628248196	6.841156612-
4.402659753	-1.826158014	5.155841508	-2.546326647	3.440749704	-
4.536230077	5.135606677	1.970090097	2.668895206	3.8052374 5.968663287
2.741896302	1.852274995	-1.991668661	4.591240647	3.940877613
3.977165624	4.195769379	-1.60390929 5.082246414	6.253737334	2.85842621
0.1168975765.436826618	-0.709357632	6.1126635265.0086934 4.820539376
5.4008511316.488633492	11.158335251.019476346	2.909072481	0.51504333 -
0.722003841	3.1311739311.025346845	4.294939959	6.910809533	3.509017333
4.893309145	-1.743869556	2.28152217 -0.038625972	3.246898271
3.732504299	-2.399897171	5.348154657	4.418398096	1.02240012 2.92255479
4.0315110264.991288956	4.83592585 3.307950846	5.33540905 4.60978732
4.990722614	4.145463999	0.562032558	3.6116743774.66417752 4.753505293
4.748168206	5.614822129	4.9137118020.825804289	5.6302749114.412641476

1.877486639 3.919349826 -0.378736435 -3.458102895 2.911099289
0.288139171 -3.401332866 -3.807541891 1.619392377 2.740158318
4.380354835 5.266368358 3.353789406 6.089733866 0.592212167
3.047408057 4.486338237 3.602100353 3.340927744
TCGA-44-8119-01A -1.007406304 4.451072554 1.042559974 5.169129443
4.156758825 8.388133174 3.95220713 4.183660226 2.288060482
4.1186603915.278203968 -1.937995457 3.257189674 5.004283618
4.898767338 4.712870774 4.063207369 4.343674982 0.975605988
0.55868037 2.751650928 5.835298848 -1.813922442 3.084433088
4.681339815 2.404992025 5.587485696 3.099015609 4.506789771
3.886474618 4.779895363 1.452265909 -3.696866826 3.625478692 -
0.708814418 2.337008454 -3.391428963 5.58509666 3.798125619 0.989201815
4.696296604 -1.259956755 5.386572267 3.436140907 3.881703298
4.3113152957.040800833 1.760653073 3.947177912 6.954688828 -
4.662893385 -1.252553401 4.672224257 -2.16499016 3.8888542 -3.857587271
5.11812351 2.350816 2.689012084 4.235000663 5.995863381 2.884081663
2.269535921 -1.283925359 4.665958725 3.938348531 4.366815545
4.295347858 -3.025325294 5.075085142 6.617833639 2.865123758
0.250033535 5.623307799 -0.520027974 5.963171848 5.138745619
4.957173325 5.33226315 6.647518384 10.7427309 0.549432047 2.998647017
1.45047161 -0.586427157 3.696503528 0.676138509 4.145067221
6.871814397 3.523476489 4.807336124 -1.776855868 2.234599203
0.2658811523.359315101 4.139654822 -2.935574784 5.316958609
4.9811831951.776302691 3.5117281643.585634912 4.927457494 4.767350071
3.401495926 5.33544047 4.729944442 5.006402916 4.321828194 0.54062499
3.706180963 4.345563376 4.538027331 4.752941961 5.484782036
4.6977616 1.188514476 5.254168677 3.70485357 1.574719569 3.837326375
-0.056466995 -3.35790352 3.869076729 -0.377507465 -3.440280911 -
2.737378555 1.296760681 2.419756699 3.656267523 5.346595531
3.4115824356.169415712 -0.094722548 2.389910942 4.508328094
3.802902477 2.959556548
TCGA-44-8120-01A -0.906064198 4.410961452 2.594500449 5.399352285
4.223373408 8.303253497 3.155815816 4.217521858 1.573652835

3.17612342 5.526328 -2.484082836 3.433887804 4.504750648 4.613234229
5.0475011133.66972659 3.589678614 0.163096834 0.442629123 2.736036122
5.075004433 -1.482122331 2.343370198 4.545094485 3.205989367
5.481274054 2.098171221 4.464590376 3.347691521 5.040917536
1.143735339 -3.762807354 3.402745575 -1.202902083 2.718200727 -
3.134100504 5.587405156 3.872902221 0.758914324 4.718277108 -
1.221997525 5.102617089 3.046304302 3.999862432 4.1117664976.956746369
1.558688406 3.3831115276.703332283 -4.748390991 -1.97298666 4.427087467
-2.879715199 3.290367011-4.731796487 5.100395925 2.031045479
2.196506397 3.780849895 6.060245943 2.720306908 2.256020279 -
2.190767026 4.633784625 3.935578325 4.078195812 4.209998624 -
2.14319888 5.083873459 6.31827657 2.874587945 -0.003630149 5.729785449 -
0.872260242 6.065184619 5.1122037834.753281327 5.512962682 6.399817468
10.85468389 0.912530616 2.460325169 0.441475452 -0.72593857
3.103566886 0.903427757 4.35526874 6.9311502173.622540169 4.707810197
-1.958729744 1.924730537 -0.343326555 3.292107288 3.762559923 -
2.649568321 5.346032496 4.377774437 1.105554588 2.796861531
4.102200108 4.982983631 4.674734594 3.256923863 5.335505499
5.045705003 4.93025549 4.058173579 0.642163278 3.437433783
4.584893694 4.876338732 4.7272161165.654920214 5.23870557 0.813666535
5.759722984 4.0835134111.880321517 3.860534596 -0.836347938 -
3.639215663 2.816192408 -0.22066987-3.461073271 -3.886536837 1.494136578
2.722935444 4.4432493 4.9311170933.05034136 6.1155450260.407654053
3.165915439 4.3607191183.562646529 2.776934217
TCGA-44-A479-01A-0.594475066 4.693525517 1.885808806 5.137437579
4.254120601 8.352451506 4.016189855 4.206140351 2.088785742
3.83368357 5.305095188 -2.187214539 3.430454062 4.97430619 4.932238312
4.896051819 4.250441381 4.250464543 1.036042002 0.551488496
2.807774294 5.502154139 -1.431021354 2.956067805 4.682208096
2.577975691 5.819634344 2.260558231 4.559603921 4.096748512
4.621470728 1.124455004 -3.491763409 3.405741753 -0.844203795
2.555700702 -3.506676702 5.5769116833.424398351 0.995035152
4.693674918 -1.185664428 5.50275263 3.650304368 3.885915657

4.398339562 7.045834371 1.783751929 4.029760279 6.773736283 -
4.775429997 -1.557668295 4.537306058 -2.259161752 3.782662577 -
4.546213015 5.053798175 2.365658683 2.330273699 4.286360987
6.101437949 3.060201347 2.160359189 -1.993909814 4.67517845
3.942375739 4.159618009 4.405678743 -2.090386369 5.07922702
6.410565602 2.971591722 0.032919071 5.872782131 -0.285222572
5.966709467 5.191399337 4.694884017 5.397848562 6.530717026
10.53393489 0.875134175 2.94916493 1.075354017 -0.827065672
3.904597644 0.833379952 4.205865475 6.837487565 3.737769594
4.87884584 -1.815750831 2.477614223 -0.120860597 3.299446289 4.01833103
-2.347177307 5.347685673 4.97280148 1.880714416 3.415186315 4.24657304
4.934898923 4.68645858 3.278513038 5.335371236 5.159518309
4.991560737 4.213704579 0.642586461 4.02567104 4.402894807
4.660794419 4.774346213 5.643614247 5.202069107 1.044749691
5.597183056 3.971233237 1.824710813 3.855534494 -0.331280204 -
3.533985329 3.392890516 -0.23007179 -3.506875515 -3.319762226 1.325601631
2.657571585 4.137787535 5.512495883 3.551783368 6.28652678
0.6115993392.678340314 4.640108324 3.756316847 3.120364984
TCGA-44-A47A-01A -0.186985777 5.149902966 2.766831822 5.356438362
4.200052172 8.155601564 3.670252268 4.280249355 1.901404601
3.277088487 5.414990724 -2.254259969 3.584261329 4.476321391
5.060629781 5.006065212 3.763799388 4.1199540180.539386707
0.493279047 2.726840422 5.208587568 -0.992083454 2.110459195
4.593398464 2.969291837 5.918950303 2.328859595 4.651145735
3.987022004 4.935614636 0.916997697 -3.315090629 3.218281788 -
1.1198078892.943254442 -3.008433308 5.59027215 3.224509668 0.916544841
4.704786561 -1.00362121 4.989782922 2.980999491 4.03399563 4.346819917
6.813194124 1.649313908 4.156100546 6.922104363 -4.868646922 -
1.723883482 4.769526061 -2.142481962 3.347355746 -4.709080759
5.192314675 2.376675865 2.068455603 4.02371265 5.999047558
2.558130315 1.887115103-1.926556762 4.716333966 3.937044996
4.172632483 4.352593771 -1.172806006 5.089375315 6.418173315
2.940172091 -0.161064738 6.011140916-0.321081345 6.160868948

5.032779558 4.719607187 5.508060566 6.277553908 10.36702773
0.651985601 2.953663758 0.966919699 -0.546373261 3.372574028
0.968564101 4.318668412 6.8829937 3.5115781664.844679376 -1.879579798
2.682913434 -0.510818493 3.248508807 3.722575198 -1.890587121
5.365391628 4.455771438 1.043390075 2.968485772 3.93329549
5.064693891 4.800901835 3.170433804 5.335723555 5.008623981
4.919277743 4.1711376150.795661058 2.981296296 4.642434304
4.845632426 4.748826259 5.668993769 4.922430202 1.123816477
5.465141201 4.332405551 1.963200183 3.945327612 -0.568337374 -
3.9005954 2.678024057 -0.041802145 -3.532392238 -3.620218496 1.67610732
3.063062741 4.536651336 5.584390127 3.379538558 6.336543558
0.032520315 2.8715073114.514499968 3.780927646 3.063971241
TCGA-44-A47B-01A -1.252184122 4.566106243 2.373395848 5.218692447
4.501089453 8.369106206 3.573301694 4.294618528 1.925996009
3.307169577 5.252033111-2.213148464 3.231885839 4.834131973
4.7012623114.753827525 3.649059143 3.862424449 0.685879249
0.397023934 2.658323199 5.643000131 -1.443877063 2.466996002
4.59394031 2.820714733 5.654123984 2.073417172 4.53175244 3.68882086
4.861663592 0.974499421 -3.717961836 3.39694088 -0.702088488
2.928043336 -3.591708406 5.594861632 4.255270182 1.224424067
4.70326616 -1.321688365 5.332098024 3.000019029 3.932837699
4.2118322546.8880211161.413560959 3.824334142 6.536314661 -4.548765518
-1.593657582 4.824152259 -2.338409456 3.64124924 -4.511443247
5.015294517 1.961739965 2.569519909 4.033330269 5.967038386
2.705386255 2.017775415 -2.225092581 4.559500948 3.936705567
3.984758449 4.315606738 -1.912751004 5.078130094 6.385773597
2.988284947 0.087227783 5.838138631 -0.690317827 5.967987443
5.129069361 4.688579567 5.413882649 6.500415288 10.27743639
0.866187378 2.886605045 0.962939123 -0.756835097 3.156370259
0.716154494 4.279288369 6.794032759 3.517341329 4.658462581 -
2.022059912 2.164671383 0.182919086 3.25559224 3.720422413 -2.720424637
5.335336394 4.853899578 0.746212345 3.092120243 3.925078406
4.916365156 4.829293925 3.589779051 5.33529739 4.905097488

4.951000194 4.248345493 0.328403398 2.988259338 4.439614674
4.676900979 4.75781313 5.675945763 4.931019415 0.838999839
5.645524654 4.230476913 1.822474032 3.923717465 -0.505005157 -
3.447078253 3.271165011 -0.078882716 -3.337538763 -3.662475857 1.545349689
2.617598831 4.129932654 5.295843608 3.503159628 6.066220484 -
0.306794726 3.027451798 4.492169182 3.636190627 2.875936472
TCGA-44-A47G-01A -0.356027616 4.696180152 2.894256967 5.332689933
4.086105131 8.264065041 3.529686103 4.366675232 1.649490108
3.418021536 5.176087867 -2.516826276 3.633281425 4.335841417
4.792228781 4.906961745 3.275687778 3.7378519 0.530194702
0.450018993 2.568135407 5.254022666 -1.124702453 2.161512901
4.53120405 2.94929003 5.677489802 1.525299619 4.542686443 3.316368292
4.834571332 0.920064918 -3.644404638 3.329804547 -1.033713018
2.628961224 -3.011737581 5.594656974 3.4115421650.943819286
4.712596255 -1.167707917 5.069566237 2.49504395 4.078978455
4.254369997 6.846542708 1.315625382 3.331226127 6.745320459 -
4.803808552 -1.857735679 4.777394659 -2.354457476 3.293771233 -
4.770916485 5.016106008 1.821969176 2.402535988 3.889673873
6.050060486 2.592109276 1.944230786 -2.132840728 4.703722644
3.935076582 3.832782247 4.403485012 -1.145885809 5.094735222
6.296584253 2.864737279 -0.146632776 5.931666373 -0.324436339
6.0119553975.092461071 4.508762428 5.502909818 6.251829353
10.66185064 0.534510339 2.684309849 0.549272939 -0.636549453
3.552925065 1.020702057 4.27345519 6.834368327 3.469133826
4.648962176 -2.081774633 2.163240766 -0.461672742 3.260424657
3.681064515 -2.13950009 5.338212084 4.789479997 0.952859876
2.984220325 3.924534034 4.972331725 4.858651965 2.788947295
5.3355116614.922504304 4.92366451 4.127478436 0.699593777 2.069497329
4.5745395114.834741239 4.772287099 5.470466659 4.953323538
0.794583403 5.655685347 4.156502793 2.210731937 3.802754974 -
0.232036735 -3.987333506 2.872138692 -0.180303236 -3.452852806 -
3.508747888 1.705144875 2.892919964 4.3114930174.98069496 3.244624998
5.993750468 -0.672641002 2.9117602324.282550364 3.665410523 3.25717387

TCGA-44-A4SS-01A	-0.449022395	4.646518026	2.715249121	5.341658032
4.135582924	8.3211919683.208137827	4.34533653	1.469896749	3.368495324
5.34239941	-2.674944182	3.464782055	4.443508274	4.425521228
5.429335524	4.062802938	3.4011505170.2009118110.4110069932.750301963
4.979776215	-1.120860358	2.406058714	4.525881031	2.952043288
5.466171426	1.134319216	4.544897354	3.1552688	4.998261872
1.017318471	-3.838403936	3.233959307	-1.358434932	2.2227204	-
3.238928722	5.615400485	3.562265275	0.357102244	4.70690107	-1.262682557
4.684178638	2.896020923	4.0311727464.200871578	6.936891367
1.055884351	3.335844837	6.670654502	-4.971635818	-1.916445498
5.28614491	-2.619628904	3.078383668	-5.229533127	5.036343081
1.473030153	1.8061134033.586459849	6.11203793	2.598860075	2.104441347
-2.355260554	4.691275605	3.931709149	3.617980883	4.2683451	-
1.141284789	5.089216721	6.292734725	2.887978174	-0.327701164
5.664878866	-0.496837743	6.068700794	5.175692386	4.502228628
5.486085264	6.063354547	10.95248056	1.184959607	2.411250553
0.016540103	-0.809773802	3.327315543	0.982632095	4.314750778
6.786419897	3.682334843	4.620822943	-2.174451899	2.067559191	-
0.7254191193.270808659	3.554439999	-2.128990353	5.359166897	4.210702061
1.160175735	2.781368593	4.192589093	4.965276965	4.767250954
2.622591894	5.335310924	4.986764138	4.89918994	4.099971503
0.8041193412.818652719	4.417184715	4.879309055	4.74993936	5.605130081
5.475551573	0.688974195	5.71079711	3.988602169	2.061572096
3.749520195	-0.503297947	-4.2978936	2.410716169	-0.382367254	-
3.602644544	-3.991816104	1.848576987	2.833857664	4.403920289
4.839932059	3.658383616	6.125460331	-1.033216977	3.074590924
4.358725698	3.628581869	2.868111141
TCGA-44-A4SU-01A	-0.392851596	4.914842949	2.535439438	5.180245129
4.45913255	8.189364837	3.776277039	4.153164715	2.222124785
3.017969072	4.746251608	-2.351885962	3.602490873	4.53594749
4.956958342	5.034440856	3.697142558	4.379285023	0.540976033
0.56321908	2.810295806	5.484978534	-1.033520701	1.567718899
4.639616536	3.066959158	5.473006305	2.719505084	4.682677931

3.800539544 5.051409623 1.13981568 -3.48693213 3.122975932 -1.022658407
3.601742226 -3.009222663 5.597184682 3.3569118161.577821259
4.721920264 -1.012026567 5.34110286 3.09860523 4.01166233 4.212167926
7.132032734 2.042147383 4.318610875 6.895773723 -4.389856178 -
1.7111364524.704795191 -2.196925696 3.517568737 -4.572796909 5.295140609
2.572046852 2.751509518 4.061754004 5.877240993 2.625395337
1.072125747 -2.055037956 4.706181872 3.935038017 4.29739712
4.296883765 -1.159111922 5.083387417 6.463486536 3.069612244
0.1045001115.470181489 -0.362993306 6.082243572 5.096805274
4.934858495 5.420096288 6.355007828 10.33802751 0.289774014
2.844528961 1.262439859 -0.824847523 3.003906133 0.751861012
4.304542135 6.955328568 3.630800093 4.722632889 -1.698717258
2.362396729 -0.120849009 3.260290167 3.718797675 -2.079731747
5.356324242 4.602748139 1.096530864 2.9421211723.822883161
5.023567532 4.898521475 3.641241892 5.335552105 4.792462313
4.92419162 4.1179452470.892205606 3.669943041 4.387564157 4.860466909
4.745684596 5.619772906 5.109876161 1.072059594 5.341137379
4.138576763 2.015916657 4.081099927 -0.581377512 -3.386653674
3.134816882 0.020442344 -3.366812926 -3.49171724 1.507026214
2.964869736 4.244498213 5.562848331 3.67465811 6.512367484
0.070435778 2.942093315 4.689950345 3.793570332 3.08127699
TCGA-49-4486-01A -1.160080605 4.722899173 3.364760473 5.609564643
4.361344796 8.315593651 3.385358304 4.298166963 2.080081252
3.235262341 5.461132809-1.912376368 3.7511905134.539164923 4.534443474
5.0114174943.323726427 4.5601122810.219098086 0.370230853 2.739517028
4.878202449 -1.490668305 1.927040372 4.53207562 3.088107613
5.423895045 4.343889865 4.467331891 3.775420904 4.821439262
1.136605993 -3.92909446 3.17420768 -1.248383938 2.807095888 -3.268443371
5.612840865 2.885823193 0.260044352 4.718653344 -1.22943767
5.305553696 2.752703374 3.934586664 4.225724814 7.112705511
0.969096293 3.888430525 6.586487716 -5.056304945 -1.569220876
4.539753732 -2.700798352 3.620243496 -4.196094053 4.937909642
2.266936158 3.124887781 3.814250978 5.975251425 2.867839912

1.986961263 -1.413168979 4.318934656 3.936744376 4.620462895
3.955675666 -2.088436034 5.075194922 6.395192656 2.984887506 -0.029770809 5.917328029 -1.031545136 6.093465361 5.130408334
4.718795269 5.509985691 6.318467307 10.08590712 1.603935466
2.481933393 0.970361429 -0.753548701 2.363178399 0.798163853
4.493367252 6.905174675 3.900848136 4.642109155 -2.469535872
2.28098913 0.41049176 3.371633017 3.862925155 -2.800806964 5.355742901
4.258896014 -0.384712064 2.416963636 4.288754443 5.020523808
4.688143429 3.643812008 5.335291907 5.179830443 4.74247809
5.103644151 0.276901454 2.5484469 4.577376765 4.612651967
4.718938134 6.056790396 5.308038748 1.3511255485.444210307
4.442492903 1.766346061 3.918616021 -1.79514461 -4.03480247 3.103950401
-0.176080293 -3.604843453 -3.005006178 1.862434921 2.591043687
4.578965033 5.206920292 3.325950012 6.084149796 -1.476929618
2.746062225 4.877008745 3.714734628 2.380043028
TCGA-49-4487-01A -0.963684498 4.810980323 2.157347533 5.400706071
3.975695399 8.313533499 3.650801356 4.268109002 2.046403763
3.938418952 5.650428205 -1.981444836 3.618148472 4.705722355
4.942991034 4.58118131 4.247617198 4.165633316 0.388127966
0.443430464 2.754192457 5.474639906 -1.501584701 2.789827023
4.626575761 2.54848071 5.861783549 2.294232181 4.602495776
4.223837398 4.813559933 1.591145258-3.730560924 3.392189266 -0.750435142 2.353983296 -3.367478714 5.580756105 3.071258882
0.693445637 4.692227539 -1.240314174 5.204665475 3.170229529
3.843441262 4.297120364 6.750462061 1.430016005 3.55376886
7.131918781 -5.23112865 -1.613060825 4.574516887 -2.752386943
3.812433976 -4.393017047 5.143365163 1.977852288 2.631346613
4.045604642 6.047287277 2.689906149 2.491426747 -1.377236998
4.62718943 3.92642447 4.286484644 4.302352203 -2.091156303 5.086142441
6.571518507 2.749913164 0.200167479 5.797930057 -0.660546118
6.062308444 4.977374027 4.79554978 5.444752349 6.473722367
10.25402763 1.039137732 2.817612448 0.940294528 -0.263109782
3.414076916 0.867466531 4.287773458 6.91379083 3.383101244

5.042587374	-1.958398873	2.324875357	-0.237471613	3.364815023
4.131297326	-2.521778275	5.320134182	4.609819816	1.310089789
3.541377176	3.8942116974.93452382	4.551926324	3.223666517	5.335435359
4.934021571	5.000146355	4.229181278	0.613249579	3.01727968
4.635007579	4.679723753	4.749109088	5.720907591	5.062700327
1.250158557	5.581782825	3.906997265	1.747212156	3.806050642	-
0.601624408	-3.664100356	3.148792858	-0.580344489	-3.942841112	-
3.451344633	1.328512868	2.410279322	4.286300958	5.1875011582.974946106
5.973699131	-0.0463297	2.788757054	4.365091837	3.609516392
2.864041205
TCGA-49-4488-01A -1.052410242	4.613229152	3.057179755	5.624472572
4.643259921	8.321757725	3.296371772	4.3445251151.6795781193.139835242
5.831983754	-2.404692314	4.050603251	4.500142761	4.828102528
5.283258084	3.41891663	4.345724754	0.147747415	0.531982685
2.578044748	4.892676634	-1.318094892	2.587419935	4.482768654
2.939012741	5.387297759	2.035091785	4.513186535	4.118864443
4.930059471	1.095078678	-3.829450256	3.377421541	-0.882682505
2.528348276	-3.107015479	5.626244034	3.198266874	1.045633142
4.714819079	-1.298176706	5.281250215	2.652878352	3.938493325
4.185427912	6.947966073	1.095161431	3.287096349	6.625369213	-
4.92645477	-2.104672272	4.925899458	-2.636905151	3.421006757	-4.761175125
4.914218718	1.949198921	2.556074374	3.6734421186.041746568
2.670030518	2.436135028	-2.493286935	4.509930759	3.949397597
4.068778142	4.189472441	-2.020225709	5.081213979	6.26222236
2.793958451	-0.04020218 5.720366871	-1.069493072	6.121465372
4.995363243	4.497109306	5.597804048	6.308093402	10.78022851
1.460155396	2.700474442	0.782672259	-0.816063394	2.888544535
1.11310071	4.502021568	6.84533782	3.670069048	4.983583242	-2.100191623
2.603929065	-0.272068779	3.329925615	3.763288417	-2.439525914
5.358556436	4.223232924	-0.267249052	2.753369419	3.982510825
5.03498665	4.827895176	3.177093952	5.335415676	4.940204451
4.896617984	4.551530285	0.612571943	2.027604798	4.586488569
4.7235611564.720030891	5.877934338	5.002880426	1.135100333

5.651343062 4.29356646 1.845837862 3.828919498 -0.830673397 -
4.447755007 2.572453715 -0.074074766 -3.46215685 -3.305426283 1.866004878
2.831475105 4.791281654 4.988980677 3.468352531 6.088808012 -
0.515958405 2.678905585 4.59146099 3.494424634 3.172611549
TCGA-49-4490-01A -0.251228385 4.916928947 3.048350928 5.455966844
4.215799643 8.164718306 3.450597855 4.256518342 2.031480462
3.203578057 5.463475573 -2.435504934 3.92630096 4.309884504
4.786236789 5.23945228 3.843532561 4.361376242 0.473829035
0.617715044 2.870766498 4.988824791 -0.98337002 1.766986721
4.498491907 3.228478754 5.51446885 3.323557837 4.707146297
3.905189288 5.086774816 1.415246323 -3.566321117 3.379945082 -
1.243761735 3.240874471 -2.602589297 5.609670774 3.081593398
0.979161296 4.727387956 -0.966202233 5.012159541 2.879402285
4.07997978 4.17588763 7.075943914 1.599322206 3.995809649 6.969815868
-4.69222502 -1.723073103 4.797716203 -2.534964535 3.261150124-4.474117886
5.268360599 2.382828624 2.4744384113.734682173 6.0285754112.736459873
1.717005891 -1.785883231 4.632675132 3.931565517 4.426783226
4.415953793 -1.377649058 5.084255489 6.354639844 2.904021755 -
0.009644961 5.757654517 -0.44535548 6.131903662 5.1338671184.899321586
5.516939786 6.187157206 10.695872 1.083776784 2.484274473
0.825379929 -0.412470413 3.1140728650.967990219 4.394325304
6.936978939 3.848100031 4.771339464 -1.866994247 2.42192373 -
0.362547246 3.344408771 3.733234789 -2.281955315 5.360249889 3.96397956
0.836838074 2.755425607 4.225268164 5.067196897 4.750781711
3.341222027 5.33560217 4.958080377 4.82915969 4.36230561 0.990605391
3.050271201 4.536926742 4.89998922 4.713787462 5.72005154 5.283136999
1.089935748 5.547691767 3.800628859 1.959154899 3.986047657 -
0.665530228 -3.958368254 2.689755659 -0.115396895 -3.4339887 -3.203645357
1.809191384 2.977339754 4.683608182 5.306469874 3.392436968
6.394737381 0.244221715 3.448917445 4.720914004 3.742260215
2.965431797
TCGA-49-4494-01A -0.639445063 4.996980443 2.544849276 5.677260171
4.257090351 8.299072372 3.199208545 4.425129668 1.882734495

3.304136131 5.972310263 -2.267959285 4.679918755 4.505209996
4.87447121 5.302144188 2.816151394 4.248901841 -0.14738119 0.480785947
2.590956063 4.760389591 -0.890352204 2.060433093 4.493453398
2.775843323 5.806812622 3.088492005 4.736433251 4.338620267
4.896910972 1.232807144 -3.62835192 3.405031799 -0.820057223
2.175859594 -3.132509156 5.617264616 2.526195822 -0.477010456
4.745434564 -0.995897559 5.099413793 2.031708659 3.996374431
4.343369322 7.239938237 0.948786405 3.626527703 7.073943728 -
5.087885423 -2.130420624 4.989111846-2.473133101 3.437594215 -4.145605389
5.172576893 2.261696394 2.756681003 3.57075716 6.159892207
2.555421434 2.287845507 -1.883836644 4.47786534 3.940269472
4.393072492 4.23934887 -0.977706293 5.08300503 6.324929647 2.7767737
0.008283177 6.046122669 -0.508473046 6.1579451115.008272894
4.863754985 5.632864922 6.232150122 10.26995033 1.654709732
2.740458988 0.915399752 -0.596861867 2.545934215 1.235948119
4.546952585 6.857861533 3.617557613 5.030103645 -2.326703266
2.720083156 -0.059926345 3.352534237 3.715581039 -2.521695438
5.353924439 3.945167119-0.495968434 2.72055721 3.838534998 5.11492944
4.682380387 3.171495572 5.3356116274.760028507 4.970784512
4.735369226 0.741297681 1.274829094 4.692702047 4.844438031
4.744980256 5.886296404 4.941549477 1.338092549 5.48595384
4.462676759 1.968386277 4.02398589 -1.305143234 -3.741049406
3.181391043 -0.054944205 -3.598452188 -2.248607796 1.897221357
2.886220937 4.896480885 5.315435345 3.3011930886.001050389 -
1.194750273 2.638507563 4.886702777 3.621245499 2.601011295
TCGA-49-4501-01A -0.666032028 4.503714941 3.065880638 5.521909197
4.235428635 8.307638613 3.434137591 4.314277276 1.644250469
3.319560242 5.652752705 -2.467055581 3.766969129 4.72805507
4.928318062 5.100728873 3.855297259 3.91372123 0.683348199
0.563464182 2.764185854 5.121462573 -1.430015353 2.880193641
4.486566433 3.120554688 5.573958249 2.872151608 4.505915836
3.398416218 4.883746966 1.144072705 -3.768708495 3.883509712 -
1.157487884 2.985441205 -3.073847697 5.596507862 3.392092173

0.986648593 4.727702732 -1.140070208 5.228461624 2.96521603
4.000979756 4.2119487227.103954405 1.337432206 3.387550462
6.639967347 -4.86206055 -1.907823152 4.559516323 -2.651460836
3.357552172 -4.335723678 5.051336076 1.943305932 2.392920749
3.888217315 6.1110933972.896940764 2.524421902 -2.156362067
4.5580191143.945077245 4.246519278 4.296431462 -2.327767253
5.0811091016.271661797 2.881378658 -0.016059218 6.030623436 -
0.642945771 6.030074084 5.1837571194.541040761 5.531456937 6.272784836
10.88823628 1.432022388 2.697387885 0.831250311-0.577054051
3.413874534 1.028245719 4.372667465 6.895680055 3.762343394
4.825924543 -2.043265235 2.17869447 -0.205063195 3.3379611453.764245367
-2.660236505 5.347914606 4.327364559 0.798267249 3.169188083
4.293609714 4.986765574 4.71831583 3.054083838 5.335353717 5.2988357
4.916346473 4.6065112240.617441412 3.056895296 4.641628661
4.768431327 4.762449979 5.668852152 5.2611085120.856248369
5.761859021 4.346422346 1.9998652 3.881804003 -0.564873834 -
3.883230805 2.903634856 -0.107692515 -3.466265158 -3.329362271
1.795630537 2.73902905 4.605721262 5.091378847 3.120949686
6.086984185 0.367865913 3.394368378 4.705362457 3.730935085
3.560368575
TCGA-49-4505-01A -0.640540926 4.657627657 2.899336323 5.53518514
4.388898806 8.278282972 3.342430142 4.353430681 1.769822917
3.233557155 5.528005921 -2.615859711 4.181053285 4.292319867
4.867674171 5.300682896 3.259356596 4.176714664 0.297304869
0.553394123 2.663956806 4.871472085 -1.085323575 2.536682799
4.434742906 3.067802933 5.596910857 2.5351149954.598737596
3.769508938 4.899079049 1.115352549-3.644128709 3.695173563 -
1.041287535 2.736653565 -2.903403862 5.6097711973.313865706 0.482003674
4.731394278 -1.104807856 5.491943987 2.428969587 4.076716254
4.238024269 7.130192695 1.26809433 3.345427712 6.516414857 -
4.827397764 -2.064555049 4.866963779 -2.590023525 3.110154169-4.587210728
5.041082402 1.935068403 2.369894875 3.753866049 6.17785999
2.853242156 2.32163709 -2.545455077 4.545371651 3.940075039

4.090373072 4.439137879 -1.44505458 5.094939098 6.233118662 2.891100421-
0.069842804 5.963899068 -0.593008396 6.050018769 5.177715296
4.556082075 5.577169683 6.235438249 10.92727966 1.264541863
2.527934103 0.899929842 -0.717328172 3.359088325 1.075691838
4.442006239 6.85879208 3.817642804 4.805945114 -2.050745613 2.338127647
-0.442295175 3.31273604 3.638569213 -2.447378597 5.356749466
4.319988029 0.349772268 2.911459527 4.130273624 5.04065875 4.879176215
2.932610987 5.335548838 5.013579356 4.850185382 4.349855937
0.636013318 2.144918719 4.54163548 4.794343021 4.744063702
5.732729013 5.116378405 0.991035897 5.745085447 4.174856756
2.196038788 3.914724764 -0.447035362 -4.159678767 2.78744309 0.07048694
-3.409694522 -3.00708991 1.828660225 2.972574164 4.758250052
5.072837372 3.440556149 5.980318531 -0.128226777 3.086269883
4.61754875 3.623087515 3.127208029
TCGA-49-4506-01A -1.014101524 4.983947881 4.001782603 5.701021295
3.781135108 8.340731285 3.229202319 4.448188537 2.214974906
3.540564305 6.145793788 -1.818589341 3.877448969 3.973229921
4.42715709 4.954530355 3.261343624 4.556186247 1.025993011 0.375069852
2.368733529 4.89748928 -1.689527022 2.424864741 4.560303686
2.806219721 5.922279823 4.779207307 4.40298021 3.606726513
4.610760406 1.009201864 -4.097587013 3.549795305 -1.068205552
2.170132821 -2.318284268 5.613434317 1.926261289 -0.374291151
4.692078772 -1.302853081 5.414952467 2.262870111 4.053668686
4.357500844 6.74676274 0.717911573 3.358171828 6.657605201 -5.478667961
-1.642824351 5.043845921 -2.39748111 3.639227498 -4.773248938
4.801119459 2.096631883 2.488306723 3.761026058 6.047319428
2.500315971 2.475680545 -1.243614612 4.458742203 3.93892037
4.446901217 4.103909802 -2.834313965 5.089175544 6.39341331
2.883931471 -0.288408024 6.053431274 -1.268277951 6.208657092
4.985346172 4.549413776 5.53088114 6.293897607 10.64301197 1.691950472
2.319669758 0.722765776 -0.170804789 2.521185177 1.15525327 4.415249538
6.81750738 3.630759837 4.623726272 -2.602197927 2.398128365 -
0.390467849 3.309607426 4.291539926 -3.01717276 5.342530699 4.496050712

-0.677820593	2.645236305	4.014848419	5.048824534	4.657203962
2.660519927	5.335439944	5.1126545654.737919323	4.469010582
0.252372894	0.799445354	4.601369798	4.566461395	4.714314121
5.740859203	4.90744772	1.240504536	5.333418792	4.01251204	1.344043142
3.72378187	-0.805578621	-4.749697223	2.514466347	-0.683128768	-
3.88954873	-2.67848158	1.7663112372.650234278	4.567495851	4.953937868
3.065781734	5.756233033	-2.401454336	2.8759115444.466533725
3.536669859	2.509731557
TCGA-49-4507-01A	-1.004362252	4.745561294	3.337443329	5.486346756
3.753353101	8.3211146973.154531583	4.413589187	1.87210736	3.638756839
5.952481099	-2.399489907	4.247496169	4.016493259	4.383355827
5.028388684	3.636268719	4.294632409	0.063109584	0.269678397
2.68858927	4.419132128	-1.411342271	2.255591266	4.469404807
2.576704952	5.788329127	4.230593887	4.550298146	3.9211276074.8375143
1.15399745	-4.002082126	3.057226853	-1.288899943	2.256386753	-
2.659594054	5.61657609	2.262040234	-1.096580614	4.703733003	-1.161242801
5.203208239	2.133501788	4.027409007	4.148973284	6.903903031
0.766593226	3.458990932	6.835859451	-5.534389915	-1.931632411
5.097308594	-2.721031434	3.380541052	-4.991590679	4.981522919
2.264507296	2.625583455	3.451983075	6.135563259	2.540032484
2.557640172	-1.82246215	4.421917857	3.92200145	4.272612856	4.328932702
-2.198428366	5.086661691	6.375018759	2.779952178	-0.11802353
5.703444525	-0.992739616	6.19988799	5.003744388	4.845032198
5.546032658	6.1380724	10.32058312	1.376289365	2.178054682
0.622752727	-0.168310015	2.948452667	1.082195171	4.438062976
6.821983984	3.627147652	4.88505813	-2.474932527	2.289628074	-
0.452555024	3.341242169	4.030845414	-3.069925267	5.339319757
3.806901772	-0.37207996	2.709709788	3.923212433	5.035366316
4.668895412	2.998481609	5.335440833	4.824590332	4.89506023
4.361608985	0.673856893	1.333505303	4.647031477	4.85717612	4.67779656
5.771024561	5.1174106041.16187311	5.445407709	3.716152126	1.514758224
3.864255319	-1.069785069	-4.771520269	2.463028589	-0.61173756	-
4.066901323	-2.694708322	1.797890541	2.564408447	4.641780048

5.087387019 3.2547821125.837251785 -2.563927341 2.800088797
4.577378876 3.492668028 2.541128741
TCGA-49-4510-01A -1.177562669 4.639427387 3.463625345 5.416686581
4.144894839 8.368324744 3.655517768 4.31744241 2.046390372
3.155710493 5.513615791 -2.168134015 3.705980474 4.288449438
4.733870217 5.048128373 3.479828068 4.652809184 0.247532623
0.535397356 2.845447996 5.256953803 -1.427735285 2.552510924
4.518304133 3.1281198525.688672269 3.591723205 4.539875635
3.583159933 4.825245529 1.211775212-3.797445271 3.29671214 -1.25841718
2.994094686 -2.919604781 5.589089384 3.147608417 0.306206371
4.724462793 -1.194469793 5.190168372 2.865290156 4.043233537
4.165314508 7.138518581 1.375330319 3.795624321 6.633138465 -
4.772035002 -1.552379433 4.455774425 -2.646354839 3.427341326 -4.2461401
5.098921823 2.016206823 2.259247084 3.894354247 6.100294291
3.0011858122.260670007 -1.689049857 4.496395453 3.931374656
4.692377962 4.114583471-2.21442031 5.086736522 6.468204127 3.028606648
-0.013411284 5.977661031 -0.859280504 6.067090594 5.199829523
4.773835127 5.48696159 6.412762542 10.31304613 1.302820852
2.433458873 0.865890503 -0.666673854 3.1107975960.739048512
4.428135299 6.945050754 3.875004652 4.59236978 -2.010405553
2.061930927 -0.092571192 3.342609102 3.980010227 -2.94656146
5.349409476 4.530037581 0.3105683112.822023438 4.277678702
5.028737001 4.689404144 3.48467813 5.335474085 5.321399145
4.699599866 4.7692851180.463781397 2.774467637 4.614192275
4.668918912 4.707561775 5.778030439 5.364973952 1.334930503
5.686815202 3.805976351 1.78535721 3.957692376 -0.684661597 -
3.873170853 3.112789462-0.387849305 -3.504228241 -3.418502677 1.787876158
2.678662171 4.4311001765.26800684 3.341900937 6.055076166 -0.83441789
3.063865177 4.652705489 3.695000489 2.590786551
TCGA-49-4512-01A 0.009379427 4.831792138 2.597753135 5.413198323
4.036704348 8.230818069 3.596209412 4.289513753 1.84243261
3.422861458 5.259416241 -2.343508631 3.886787335 4.262438823
4.99764151 5.009123501 3.568563616 4.241751476 0.342096672

0.531836514	2.847609013	5.229660735	-0.895938474	2.484411941
4.533757134	3.076086545	5.584624995	3.344521727	4.691685042
3.836726759	5.073038087	1.319302739	-3.462772779	3.543104906	-
1.01966734	3.053531402	-2.77096483	5.597282307	3.248465094	0.617067483
4.728646195	-1.028263885	5.262758368	2.649588958	4.019290168
4.230404284	7.076535031	1.640128651	3.788021318	7.028463024	-
4.608114752	-1.696384282	4.432478328	-2.517760769	3.405846878	-4.233635318
5.235776278	2.480228149	2.668530389	3.890125231	6.117694359
2.734576016	2.036777663	-1.303277162	4.696095909	3.924576411
4.477322498	4.358083031	-1.066052339	5.095741058	6.538371079
2.84390903	0.042785549	5.764973328	-0.229587066	6.07170637	5.061218559
5.010809755	5.496208731	6.356070777	10.85120169	0.975350472
2.70990072	1.076432902	-0.324577847	3.467189371	1.038738759
4.370883398	6.940678239	3.558158377	4.746025469	-1.932326771
2.327803249	-0.162027212	3.351792961	3.931238753	-2.07911289
5.338465192	4.49125661	1.044065307	3.100050357	3.799460824
5.066726367	4.740584788	3.345798445	5.335729696	4.960076608
5.068283565	4.662239236	0.984166519	2.956124387	4.672688924
4.942565122	4.744797945	5.676259522	5.05633593	1.11150185	5.479195307
4.144540461	2.194109636	4.003735106	-0.840090489	-3.646661585
3.356138639	-0.083476264	-3.531669887	-2.933334028	1.816215253
3.000525831	4.536222263	5.240788754	3.0185909	6.189428838	-
0.158192098	3.056165142	4.566264583	3.746109547	3.098340454
TCGA-49-4514-01A	-0.990528259	5.02078353	2.0916204	5.524842717	4.18114516
8.208117935	3.387823656	4.304697918	2.210871894	3.825479277
5.729865461	-1.786259328	4.183282961	4.458680601	4.743760394
5.207809378	4.119084891	4.455525925	0.041902292	0.459017188
2.950550798	4.497141752	-1.427332581	2.49110702	4.521729402
2.687191499	5.653279727	4.048654552	4.627940285	4.296209526
4.892069225	1.427940656	-3.527913089	3.350313525	-0.978271845
2.063221102	-3.096074984	5.613264372	2.398660068	-0.513438605
4.704067153	-0.969420153	5.002814408	2.697387232	3.953643381
4.338244978	6.953658962	1.046526911	4.04163232	7.162308894	-5.300062969

-1.691685204 4.638196284 -2.349270607 3.58083301 -4.253249498
5.161725228 2.468184569 2.412023851 3.777809217 6.107106645
2.894926899 2.372613333 -1.400833598 4.471686942 3.923335172
4.4508112194.422058213 -2.463655434 5.082202622 6.509792663
2.832544667 0.030645885 5.85005195 -0.706313261 6.178195293
5.003224473 4.885726367 5.56254146 6.11880614310.24042551 1.20078223
2.683366061 0.954785874 -0.379558274 3.312797577 0.904662954
4.405759459 6.880203442 3.72238544 5.068274944 -2.113375087
2.617179502 0.243594205 3.397961821 3.920559051 -3.003961034
5.349991208 3.815826456 0.933473564 3.131310145 4.120194866
5.077972618 4.544432567 3.356840429 5.335557844 4.626256918
4.872342067 4.9319821130.7327451112.814938932 4.627016325 4.706710913
4.689279223 5.935294833 5.275000853 1.629209878 5.362702187
3.87005717 1.5079011923.876607431 -1.426278554 -4.088582564 2.83182248 -0.474713827 -3.868308983 -2.61518415 1.850856156 2.597445064 4.601259464
5.376070209 3.4292115336.155645665 -1.7195003 2.506955108 4.701274312
3.816475916 2.440811534

TCGA-49-6742-01A -1.312943342 4.753854424 2.7571811615.439919685
4.086664376 8.298001536 3.1593354114.27085364 1.958721797 3.254803101
5.55497536 -2.294983676 3.591814555 4.669149558 4.495421789
4.995756388 4.078143157 3.960515409 -0.114951476 0.345040945
2.799691036 4.565737839 -1.60415357 2.141885724 4.516927882
2.803192016 5.66962778 2.483012969 4.4258292 3.44111754 4.814161544
1.051502635 -3.806314222 3.025594106 -1.446546598 2.196556913 -3.353686563 5.601004217 2.684100994 -0.419896499 4.694628625 -1.181367733 4.895819081 2.723000278 3.960190608 4.281608648
6.978726371 0.913417916 3.68712699 6.776911913-5.298423216 -1.994333108
4.884237729 -2.784834299 3.502179844 -5.068662586 5.027078464
1.625673291 1.943361873 3.856629481 5.973577484 2.640241652
2.339708692 -2.485215472 4.410837136 3.928495707 4.058725382
4.232873374 -2.620100628 5.074302552 6.32267462 2.978110081-0.185474307
5.709001899 -1.104646218 6.165793424 5.088978635 4.660811761
5.560771229 6.21693115210.05564408 1.237335783 2.554188834 0.46548504

-0.785565213 3.1056114080.895726501 4.359630548 6.802302589
3.714420072 4.898769428 -2.216677905 2.137864185 -0.300295605
3.287804794 3.680059521 -2.973235998 5.352579264 3.872190537
0.601033434 2.901477189 4.330270468 4.970280817 4.566976315
3.2895438 5.335243856 5.094054125 4.793176751 4.258581567
0.501690897 2.687006654 4.701587289 4.660209614 4.702286281
5.952699794 5.298273302 1.051469009 5.743748364 4.187552912
1.436179388 3.816590886 -1.543496344 -4.26550919 2.383004798 -
0.472491532 -3.774125412 -3.883328738 1.610828972 2.417055809
4.472827067 5.32403323 3.4199321116.022191996 -1.558629528 2.757267814
4.560850974 3.708371232 2.687141337
TCGA-49-6743-01A -1.220637641 4.551717734 2.228540738 5.537799169
4.466221899 8.270404639 2.966194036 4.224019681 1.801903063
3.7724756 5.571580003 -2.368466562 3.229106367 4.8711744964.56740951
5.12036211 4.33793909 3.480155187 0.203371202 0.445057194 2.721268208
4.910792812 -1.544361759 2.888487432 4.569644924 2.870689385
5.383172777 2.425970997 4.447709467 3.543564878 4.91570526
1.256463196 -3.996659564 3.536143329 -0.823329469 2.361050366 -
3.153077751 5.600728577 3.241414584 1.066339339 4.681210223 -
1.408964757 5.399821864 3.306273628 3.903649618 4.251051436
6.9114953631.294134672 3.40656812 6.864142669 -5.245335896 -1.941453727
5.015112542-2.966874696 3.497998583 -4.995837086 4.957284256
1.351865752 2.8330110253.854139795 6.094146328 2.665393374
2.685067111-2.327560206 4.498658005 3.93212409 3.901544165 4.230301403
-1.945315045 5.074320223 6.279970879 2.751313173 -0.088732793
5.498883433 -1.015436771 6.053056915 5.056020945 4.603548831
5.508625589 6.37360121 10.76369138 1.2674737112.6081197950.315364955 -
0.6113554643.548108363 1.04207317 4.285235906 6.773521427 3.543122583
5.097968716 -2.024863817 2.278827882 -0.029061596 3.330319301
3.768983962 -2.396325861 5.333271564 4.392287318 1.168385528
3.242107895 4.152681377 4.906447886 4.645024932 3.071675613
5.335137738 4.918985436 5.030397879 4.058706859 0.449257685
3.379634852 4.803214461 4.6611746394.743630891 5.782269056

5.287530271	0.726739934	5.886889819	4.179557084	1.655299243
3.770067183	-0.570898976	-3.921636618	2.687641574	-0.427939358	-
3.814983459	-4.1777161	1.516133927	2.50575473	4.564595949	5.136257889
3.187397874	5.920189669	-0.131687935	3.141460363	4.434524509
3.512050069	3.348347345
TCGA-49-6744-01A -0.693885625	4.610891953	2.70845904	5.482227956
4.1165006098.2831148563.377715642	4.336751965	1.643264191	3.485192196
5.407785916	-2.466768234	3.58011034	4.397206828	4.757057401
4.904148731	3.753107738	3.79784194	0.515884095	0.515322315
2.623907819	5.053507087	-1.378421793	2.778360941	4.499088538
2.951678487	5.684722605	1.590342218	4.485631534	3.233034171
4.821929659	1.067579715	-3.781431292	3.672852459	-1.057778032
2.517844481	-3.120223098	5.584598695	3.317893035	0.884241043
4.707455314	-1.180791215	5.138571423	2.960599639	4.013594439
4.270744388	6.853121776	1.312433087	3.203969666	6.706964527	-
5.077676338	-1.9059316	4.48099941	-2.661114295	3.226563585	-4.76384748
4.989443553	1.809952064	2.26438719	3.941583177	6.138106363
2.676714879	2.342645115-2.457842129	4.6761115153.932353396	3.85218245
4.371323134	-2.106118107	5.092818174	6.3259751122.825795963	-
0.085540995	6.042241933	-0.539581255	5.99559595	5.1644115224.419287297
5.52897574	6.259442241	10.747346	1.01020264	2.499589593	0.598989349	-
0.643977003	3.698888325	0.982601436	4.29940572	6.853781502	3.634659339
4.742053124	-2.036284062	2.005163824	-0.524810315	3.303184333
3.791469717	-2.439522031	5.339751633	4.626773889	1.274488051
3.207141063	4.187776846	4.942367008	4.699477045	2.785345711
5.335420721	5.15237374	4.889670551	4.122694719	0.638900493
2.809699001	4.5450711534.760362018	4.768727338	5.597055623
5.281377813	0.925667051	5.756463559	4.186845849	2.029071272
3.709101354	-0.292607069	-4.233459079	2.836653459	-0.454756071	-
3.565345649	-3.545907878	1.637560637	2.772816427	4.536687984
4.817346419	3.017351458	5.936467552	0.017015669	3.178985994
4.332514423	3.659793701	3.223017139
TCGA-49-6745-01A -0.690579082	4.622192631	2.189505932	5.609888588

4.229733405	8.283413297	3.228816246	4.412027913	1.479584167
3.3729411895.576966976	-2.62854852 3.68824078 4.684102195	4.674503599
4.972900334	3.083010292	3.41285918 0.482158915	0.535666177
2.335944823	4.857411732-1.375862003	2.3886249114.496280929	2.818271802
5.708033458	1.151682415	4.483371918	3.6958311894.813925797
0.942796158	-3.616478401	3.548876989	-0.784927169	2.164484484	-
3.557125634	5.608960286	3.625360516	0.7863427 4.716977466	-1.156626649
5.17435968 2.35833479 3.971672308	4.236268489	6.9409211820.965083101
3.15276224 6.650987987	-5.138879179	-2.173371715	5.153024079	-
2.477492899	3.032312832	-4.761670669	4.999279853	1.569428966
2.4291198623.637939184	6.055840267	2.542912157	2.273617968	-
2.71823027 4.638135807	3.9430112 3.558227592	4.468490059	-1.523746113
5.089769419	6.135437407	2.674079784	-0.063389429	6.029242022	-
0.385608253	6.029926137	5.059376615	4.541207068	5.510620631
6.267624109	11.063171120.766238324	2.758935988	0.499395291	-
0.642885634	3.272776827	1.261658712	4.338716208	6.775862384
3.415636337	4.828384486	-2.135630527	2.296691897	-0.560477024
3.24126109 3.583906251	-2.633466585	5.343242374	4.4537821170.35985479
2.923510036	4.104269579	4.949316963	4.879392605	2.572232962
5.335462601	4.687538401	4.992536994	3.944409571	0.598426347
1.56251689 4.422594561	4.774435094	4.787488757	5.522964043
4.761332546	0.619717585	5.665102222	4.444416742	2.066565595
3.759917066	-0.329597769	-4.488822409	2.741895277	-0.161104917	-
3.556800092	-3.515668526	1.726319217	2.619782206	4.551346736
4.845024499	3.054125915	5.840226862	-0.067978063	2.838688075
4.337395331	3.538788182	2.995125805
TCGA-49-6761-01A -0.778578022	4.851828059	2.453320151	5.564921249
4.204530003	8.285591237	3.49193986 4.365477655	1.932442468
3.387076488	5.741222203	-2.324678533	3.862129435	4.341520327
4.81218975 4.97461186 3.584485796	4.347103081	0.7911797710.51586911
2.53286028 5.25855213 -1.360321502	2.303292624	4.557218341	2.766150626
5.582462735	1.463727437	4.583556284	4.437132478	4.938705301
1.183907255	-3.609775731	3.612536313	-0.495258929	2.487785703	-

3.183821222	5.622765218	3.428097795	0.7422311744.70371975	-1.17038639
5.328123085	2.6030185113.960863249	4.230972986	6.801496354
1.305728512	3.61553233	6.884272767	-4.818706206	-1.938525244
5.097804956	-2.137670833	3.327520114-4.742775152	5.076272784	2.04495417
2.220525859	3.819681463	6.000695184	2.548698569	2.401425077	-
2.154085126	4.60643414	3.939941985	3.97554807	4.500850481	-1.943591926
5.091613488	6.317233144	2.776255647	0.01625657	5.774761264	-
0.923054964	6.206413618	4.951803473	4.749690408	5.557751486
6.412708421	11.0037388	0.626125466	2.721076609	1.075585775	-
0.6118499583.071571799	1.013406335	4.41927888	6.79286173	3.520215536
4.919249654	-1.877346976	2.681673902	-0.327213116	3.305103134
3.834390276	-2.642762542	5.355746516	4.396458027	0.308182282
3.051975652	3.682919867	5.027021515	4.906704573	3.124842773
5.335633777	4.623988254	4.843123368	4.279561254	0.571617397
2.01626932	4.489387889	4.765231728	4.718866964	5.663195779
4.614782352	0.939628672	5.523889179	3.960052816	1.719338763
3.876609211-0.168363787	-4.445364991	2.717904211-0.217865419	-3.550905466
-3.243908581	1.580917225	2.812752436	4.562157892	5.248250576
3.345340959	6.038561068	-0.393218007	2.823200605	4.331554251
3.532323691	2.714429752
TCGA-49-6767-01A -0.185790161	4.678584501	2.45247382	5.56355838	4.283760832
8.080754386	3.420433097	4.213863562	1.49661434	3.000873499	5.5607493
-3.03780747	3.546988237	4.72021371	4.9116264634.889167434	3.539056585
3.760068896	-0.57571084	0.367988563	2.409577746	4.739988226	-
1.199418431	2.427027616	4.515828385	2.467865558	5.306688019	-
0.049495644	4.420104591	3.971742062	4.914390968	0.933711713-3.536698656
2.648569365	-0.950228967	1.629962651	-4.382593496	5.627866796
3.101924518	0.056488358	4.70796339	-1.247700794	4.741400025
2.582036141	3.7381145534.040638746	7.095313156	0.791702097
3.791404773	7.100922575	-5.573197028	-2.500152875	5.624978853	-
2.638470632	2.886226549	-5.599613338	5.142629347	1.392859846
2.432538963	3.274679765	5.772172967	2.21790104	2.238655136	-
3.5431021134.685125518	3.938558519	3.338315829	4.578875283	-0.609832961

5.086977322 5.994370593 2.612439997 0.056005236 5.330641795 -
0.702912363 6.187201598 4.891273778 4.854617771 5.536466454
6.251643931 11.081766830.33284587 2.868516763 0.501973158 -0.944972539
3.142897773 1.155408746 4.39482151 6.763095018 3.208129932
5.142116306-2.157573968 2.366664124 -1.123785594 3.222412797
3.700410098 -2.112465303 5.35567423 3.809860044 0.308200055
2.593603554 3.907878343 5.01277377 4.982736847 2.63397581 5.335390713
3.936040845 4.948990155 3.608197717 0.887332873 1.662278087
4.298425742 4.859813053 4.763755022 5.414967203 4.245526565
0.617448305 5.166178473 4.037603934 2.06185742 3.849561582 -
0.444968345 -4.800565305 2.258295184 -0.351499262 -3.964152191 -
3.945701588 1.3111769992.510821053 4.512576049 5.34262202 3.497695642
6.17393056 -1.022909579 2.007247747 4.352329022 3.409363197
2.949655324
TCGA-49-AAQV-01A 0.205800476 5.1125712013.064363589 5.365033469
4.415254093 8.074149645 3.592230602 4.208902305 2.139324
3.220578609 5.085574523 -2.146295102 3.839224671 4.5058034
5.125105362 4.991860545 3.553922775 4.537490692 0.237521552
0.57213225 2.835097826 5.025306699 -0.870765569 1.96297923 4.627920584
3.089805832 5.549757137 3.5114313394.668583662 4.291549002
5.020288703 1.30828287 -3.242416967 3.012230111-1.034000374 3.261237921
-3.062379698 5.616913258 2.570616929 0.566136535 4.713559073 -
0.969145242 5.450838294 2.981865584 3.977261762 4.30480697 7.087477558
1.64673128 4.438780809 7.123321122-4.903887805 -1.827800336 4.809616782
-2.076873613 3.57269309 -4.676375203 5.255062885 2.4346118152.605579501
3.949544104 5.903268398 2.542835831 1.832145192 -1.883491903
4.6526211 3.935719305 4.379988888 4.383267899 -1.116882257
5.088643453 6.357606519 2.9411352160.036699162 5.46196892 -0.42475163
6.233983501 4.958466175 4.969127027 5.506898983 6.402150057
10.44192783 0.509833163 2.963362851 1.391312537 -0.618565191
3.1143876061.055631082 4.3115568546.913383178 3.64264233 4.958080156 -
1.897478913 2.778661012 -0.188701772 3.329836047 3.919705145 -
1.8483331165.361286625 4.08037395 0.844323981 2.769418992 3.658084402

5.122770764 4.818015004 3.55814734 5.3357579 4.642735586 4.957828014
4.466938366 0.945229986 3.41004277 4.727337978 4.919233756
4.728780712 5.717753392 4.991836134 1.146031505 5.137817109
4.215346788 1.906602776 4.010267817 -0.708587683 -3.682952433
3.083869574 -0.170286732 -3.771575168 -3.184744672 1.539877737
3.067419831 4.613059085 5.860287405 3.528974226 6.507143885 -
0.32782193 2.608022636 4.691552246 3.869560569 2.976700326
TCGA-49-AAR0-01A -0.114382121 5.245487249 2.55804465 5.123363471
4.059451979 8.183586795 4.194742033 4.180885648 2.591937257
3.861656154 5.20251385 -1.675070513 4.086560021 4.453040596
5.233747848 4.878882013 3.955352852 5.093417893 0.218398863
0.4411329653.174317944 5.42750476 -1.053723022 2.460649249 4.648423888
2.764371999 5.908214716 4.584063715 4.840749679 4.28952935
4.988096045 1.546814734 -3.231494758 3.225616594 -1.15563835
2.767728292 -2.973914446 5.580394369 2.691668245 0.392564344
4.718475046 -0.802700709 5.06316774 3.072309259 4.015613781
4.340638099 7.083988587 2.034340378 4.715435015 7.407779648 -
4.784282028 -1.240527928 4.075709602 -2.075832733 4.041747896 -
4.00332885 5.487374029 2.949772862 2.53745459 4.1126965286.085088635
2.940316109 1.822331256 -0.67991786 4.689621813 3.91871219 4.861260223
4.386380984 -1.926485527 5.085636012 6.816281552 2.985788164
0.150232924 5.876459989 -0.107236648 6.155777163 5.014703231
5.278094406 5.468892785 6.37744242 9.6987117590.685872988 2.914482688
1.55670343 -0.330068808 3.676404855 0.6154711484.286088475 7.019943026
3.593336786 4.879094542 -1.771862812 2.555797979 0.307223391
3.397922071 4.261104949-2.415871388 5.33695429 4.518429735 1.682217314
3.226801627 3.738354281 5.11272389 4.513398314 3.888130684
5.3358113454.949612737 4.988140577 4.723523174 1.038073549
3.781460786 4.749988475 4.908303261 4.713131553 5.847894881
5.174477553 1.821926509 5.088215093 3.922738501 1.806087531
4.109787601 -1.271011722 -3.175042714 3.63040035 -0.321792868 -
3.751194463-2.628863879 1.566784417 2.843543391 4.339170348 5.843661996
3.42851728 6.556068582 -0.567831304 2.489479288 4.827838025

3.993301796 2.590193721
TCGA-49-AAR2-01A 0.141628282 5.302608954 2.606221326 5.100933909
3.823743434 8.111853053 4.037924729 4.167300458 2.561776458
3.805350845 4.867039992 -1.746223814 4.136658068 4.360881546
5.19120087 4.861225254 4.104917373 5.062718314 0.002175107
0.432930868 3.189037928 4.944191206 -0.898220076 2.200302196
4.669522883 2.893047113 5.761567237 4.780630676 4.790167725
4.636705723 4.950238228 1.521798603 -2.972574264 2.728474289 -
1.196832164 2.521192483 -3.081143938 5.593204616 2.426336958 -0.183038101
4.712658441 -0.796311685 5.294884646 2.853631955 3.988415492
4.265940002 7.054129037 1.713631015 4.924796226 7.405956479 -
4.741466057 -1.268863586 4.444867522 -2.163631694 3.953104738 -
4.058732998 5.414790853 3.067199709 2.47765349 4.092305845 5.989156302
3.067981585 1.77003232 -0.337263753 4.654207585 3.916267512
4.764070724 4.369456848 -1.298954471 5.089669242 6.706381843
3.027592394 0.071001536 5.530262782 -0.033309852 6.222951827
4.995437674 5.269220256 5.470212021 6.231721429 9.859680582
0.581832064 3.081031111 1.386517953 -0.360061583 3.740996748
0.701856327 4.366748083 7.00697861 3.786498925 4.864288695 -
1.904871257 2.854871531 0.275371748 3.38230076 4.159380671 -2.246438723
5.357365901 4.067713546 1.506215171 2.905605229 3.97989099
5.133054418 4.576583746 3.798190642 5.335886359 4.936979491
4.884946553 4.797508775 1.125445426 3.726688112 4.709084698
4.906238922 4.689586921 5.87001924 5.261444807 1.915789546
5.177617061 3.883020473 1.890675933 4.11706205 -1.631312471 -
3.072490464 3.252747924 -0.31212386 -3.879773356 -2.536253328 1.533047221
3.021908936 4.237480273 5.888248075 3.559769181 6.559592202 -
1.167676976 2.285154636 4.805325531 4.02453878 2.483160415
TCGA-49-AAR3-01A 0.181138195 4.75642149 2.170723913 5.453492201
4.012238072 8.151278853 3.717800687 4.27821643 1.857287393
3.781682622 5.409774795 -2.521346892 3.978421994 4.405678869
4.859640286 5.117152015 3.905753424 4.233784283 -0.197219099
0.405223472 2.728670834 4.976846082 -1.175180598 2.474035993

4.578723028 2.710929181 5.797087745 2.217269479 4.615182703
4.186517716 5.06143302 1.273819929 -3.287007793 3.185752246 -
0.954673681 1.991722405 -3.442868404 5.613996853 2.714061903 -
0.0942116274.703173602 -1.102824002 5.188701229 2.502137863 3.912332955
4.174940161 6.854400537 1.292745444 3.702264387 7.398942038 -
5.2175357 -1.914173923 5.172914683 -2.341632684 3.432281132-4.991108897
5.175471028 2.533259441 2.669734342 3.617514651 6.058718431
2.462523745 2.252225141 -1.708404133 4.788259066 3.926788634
3.870286304 4.500495176 -1.732678652 5.092763617 6.385773953
2.665665575 0.134152619 5.583704714 -0.290022674 6.13081622
4.885043222 4.979432841 5.531905224 6.171641525 10.92121846
0.520457376 2.948766873 0.966498322 -0.412914257 3.699819359
1.123942564 4.356466387 6.843468865 3.276038595 5.003400195 -
1.951573903 2.61608529 -0.624772477 3.298810175 3.947461264 -2.371412872
5.33938124 4.2300114451.184463864 3.004624506 3.53576565 5.067567173
4.836610649 3.013469581 5.335727219 4.247364053 5.155072482
4.001769727 1.09225126 2.586366409 4.537412973 4.992519076
4.728544923 5.5090878 4.761670153 1.102294815 5.0969114983.74230481
1.904034764 3.882459274 -0.609928419 -4.110159925 2.822181697 -
0.273705485 -3.943990733 -2.910678341 1.586363029 2.84464025 4.529604362
5.138468971 3.307585262 6.218343198 -0.93390969 2.363812713 4.41930874
3.65203646 3.091632006
TCGA-49-AAR4-01A 0.484755884 5.147894575 2.37274491 5.160608374
4.2211931268.150857412 3.760488542 4.221352671 1.96110668 3.412535409
5.020071776 -2.321960435 3.917955584 4.539376753 5.077727104
4.892722277 3.477368304 4.087468525 0.089078305 0.478511862
2.850799484 5.316610274 -0.693043281 1.570441791 4.616388015
2.957506981 5.675747965 2.741313007 4.74115936 4.017962077
5.045784445 0.991742916 -3.141096826 2.767570982 -1.055644636
2.775616177 -3.378584405 5.597166324 3.163054971 0.332102338
4.717877474 -0.902122764 5.240702573 2.476317782 4.02492529
4.293158533 7.073733768 1.59556909 4.123705441 7.216122321 -
4.768493594 -1.776261047 4.891129944-2.362134546 3.588393097 -4.694155839

5.3118372472.423368093 2.497713452 3.974361808 6.006058427
2.606366261 1.279010936 -1.247717511 4.71902125 3.92532382 4.157370165
4.421445643 -0.21005449 5.092223219 6.40825442 2.915934656 0.060412315
5.595562323 0.1366299 6.103962771 4.962729223 5.070133738
5.460647171 6.333372645 10.38437724 0.57175115 2.964379864
0.819068403 -0.481141653 3.536623992 0.963985527 4.307912644
6.920343602 3.346229073 4.823996794 -1.854662033 2.521248577 -
0.274275538 3.234030902 3.811726709-1.798312077 5.346676924 4.491987921
1.002125926 2.915028725 3.841542801 5.062109902 4.797254137
3.303414038 5.335740668 4.632248914 5.090414125 4.134658461
1.036016054 2.628692539 4.640367507 5.0134263 4.752726502
5.592037321 4.825318695 1.0038241185.324388701 4.254264556
2.232142687 4.074673833 -0.859076724 -3.558317445 3.089674668 -
0.184858322 -3.695363602 -3.346526953 1.4294099113.031390012 4.33220897
5.644959487 3.340848473 6.302855219 -0.413663517 2.64759136 4.63552341
3.804145526 3.061154302
TCGA-49-AAR9-01A -1.281256069 4.628414885 1.986512725 5.385327162
4.376172292 8.238400478 3.24446252 4.209721545 1.623275784
3.141280529 5.653550238 -2.812598006 3.507945012 4.815369294
4.417488915 5.0112101514.238157539 3.704612359 -0.199640888
0.184298003 2.745026098 4.640286468 -1.454211264 2.504798745
4.470957568 2.673187022 5.042598904 2.028024801 4.505283644
3.757056914 5.201657673 1.020280834 -3.920405937 2.598771838 -
1.1811923542.351044267 -3.722388792 5.635206794 3.811309688-0.101455803
4.697680625 -1.327894493 4.974092033 2.70387643 3.799345225
4.004995505 7.103256009 0.581083359 3.422486171 6.548964446 -
5.206248434 -2.010998812 5.686552149 -2.954526826 3.089860064 -
4.960165161 5.069241389 1.428717098 1.869260316 3.177913395 5.89017947
2.4921133552.270200376 -2.416187006 4.405966602 3.928901322
3.779803861 4.254661096 -1.519487758 5.076547327 6.1452597
2.817205972 -0.006113426 4.976216871 -1.232624929 6.161432162
5.027172775 4.872555345 5.39494901 6.11583166411.535167150.927033682
2.582281063 0.241296361 -0.551546342 2.993839996 0.986303436

4.342656012 6.7383646113.528516364 4.887563072 -2.466585108
2.140356855 -0.568313245 3.282636126 3.59554501 -2.583340792
5.338718842 3.564498367 0.270547348 2.607802891 3.980786436
4.993838758 4.808526817 3.1641113865.335206141 4.455029127
4.985916868 4.26648697 0.475773842 2.510723467 4.455272053 4.88179535
4.720473478 5.650936763 5.054229669 0.45739808 5.65301557 3.913010625
1.699812279 3.882309873 -1.064314768 -4.364622136 2.288993083 -
0.108549105 -3.884480224 -3.868066079 1.529467603 2.450556515
4.238283397 5.255028025 3.977534019 5.955149677 -1.101837982
2.539660806 4.404331541 3.441852031 2.939666377
TCGA-49-AARE-01A -0.802987102 4.968225104 2.270386042 5.226317977
4.191818944 8.177017744 3.658486846 4.154759786 2.121490393
3.483421552 5.131679201 -2.125363343 3.632501997 4.30960259
4.680888642 4.871297648 3.85259802 4.292144229 -0.031035674
0.335264591 2.9113247654.87568539 -1.074448466 1.65247247 4.596571118
2.827766093 5.263503327 3.091046885 4.67493481 4.05729059 5.101534186
1.24923014 -3.777766009 2.669620915 -1.199332355 2.547635775 -
3.169364404 5.601284399 2.8557808 0.084609315 4.695712647 -1.093145145
4.888952149 2.788466097 3.948329632 4.251971789 6.998556447
1.460881456 4.043603041 7.319745987 -5.092892238 -1.678362807
4.479218225 -2.674618356 3.713129184 -4.790046795 5.248124773
2.127410442 2.492309954 3.916172899 5.953870927 2.555802657
1.552454662 -1.900545831 4.522102696 3.912796291 4.289536098
4.331276156 -1.145342717 5.08016497 6.503166745 2.878433092
0.005770316 5.363267129 -0.776635832 6.223740888 4.965111691
5.076392694 5.487428985 6.297272297 10.1547401 0.86160334 2.497758744
0.661668965 -0.689296181 3.147483655 0.772708021 4.330631514
6.869314054 3.54886443 4.75343629 -2.132479817 2.479449955 -0.090354297
3.363146841 3.958477682 -2.223723102 5.341863175 4.194522693
1.059227585 2.7113085023.743810021 5.032241799 4.489044623
3.605225685 5.335449334 4.642426807 4.9529199 4.512277358
0.845797357 3.39625417 4.656488221 4.868091692 4.692518894
5.8118259225.137168777 1.21417944 5.342173578 3.793517018 1.659624252

3.986155179 -1.610120338 -4.139319814 3.017106854 -0.771795531 -
3.854207291 -3.434252925 1.490657499 2.618693832 4.265515762
5.554491958 3.438312688 6.190227969 -1.638325286 2.750588024
4.547433208 3.715986917 2.317868558
TCGA-49-AARN-01A -0.549680261 5.047265751 2.530619281 5.306406214
4.145366543 8.194641904 3.607907038 4.277185426 1.858317401
2.948961909 5.351769074 -2.444894666 3.67044716 4.302260692
4.923251908 4.872601287 3.470131115 4.137583959 0.438389368
0.452573868 2.677247782 5.046540521 -1.081187269 1.774623491
4.553649233 2.918117673 5.676858338 2.665274171 4.642415777
3.709841552 4.962653433 0.7892696 -3.58838495 3.172408929 -1.180015223
2.971962589 -3.13000348 5.593222616 3.091073671 0.510524393
4.721843762 -1.014788026 4.88664012 2.56830161 4.03139081 4.262947591
7.048418837 1.558674182 3.963604126 6.773728292 -4.689668987 -
1.956571219 4.774372101 -2.24026935 3.199940336 -4.867526018 5.211238595
2.269542632 1.957637834 3.954464663 5.952034421 2.525976107
1.600879371 -2.185428884 4.66066839 3.931566872 4.132936993
4.347681349 -1.334438534 5.087391785 6.342820344 3.006792322 -
0.087027613 5.845118269 -0.477017423 6.099947048 5.073053063 4.795699499
5.511571337 6.25865019 10.45650736 0.759021006 2.646974945 0.837714837
-0.606098394 3.075833144 0.863288557 4.361347811 6.902551762
3.532260486 4.752775699 -2.041265558 2.243417038 -0.512963806
3.246124628 3.680525211 -2.258036915 5.351045374 4.319130506
0.613485719 2.976021059 4.027105749 5.019921247 4.804864748
3.184525955 5.335590664 4.802812599 4.87106626 4.080060234
0.775019192 2.578095198 4.440643901 4.874514733 4.74342672
5.653604017 4.913170022 0.903984565 5.398043344 4.142376276
1.925678244 4.023668552 -0.706928856 -4.028697662 2.710552407 -
0.177186829 -3.460427699 -3.59210384 1.553177073 2.968257214 4.404238522
5.528966757 3.348805436 6.246077008 -0.297320035 2.910345655
4.524672125 3.719354241 2.979902686
TCGA-49-AARO-01A -0.01648805 4.862840715 2.419522327 5.284828661
3.967561915 8.143725891 3.725191956 4.256060537 1.742844572

3.363352131 5.029877769 -2.689968452 3.875321676 4.299610688
5.015178075 4.81411776 3.327706949 4.080652514 -0.227722693
0.397218955 2.708797844 5.145180068 -0.929703728 1.784314056
4.522515797 2.906568849 5.652929157 1.818183632 4.656197894
3.7457556114.964479134 0.912180425 -3.383858544 2.915816394 -
1.133496877 2.521620506 -3.338844285 5.591718761 2.931710506
0.052248565 4.724372983 -0.972880412 5.169265478 2.273062494
4.063895577 4.183069149 7.082918569 1.463128409 3.641223465
6.914421524 -4.910226154 -2.019703665 4.728795791 -2.463880162
3.286145737 -4.793060856 5.226293581 2.236365914 2.348748991
3.810388222 6.023754581 2.567595809 1.730039234 -1.748264332
4.737458846 3.926673832 4.020710974 4.390989763 -1.028836302
5.100307246 6.336952044 2.870485437 0.050230679 5.692695083 -
0.135468164 6.031078325 5.015156756 4.909929314 5.51041284 6.27047753
10.64874635 0.391277635 2.75522717 0.722738848 -0.575357213
3.613352049 1.001043664 4.322326617 6.892434721 3.370796226
4.751215734 -1.969091635 2.313953267 -0.493808403 3.236972129
3.714906558 -2.15193834 5.338081623 4.513745319 0.960389644 2.94331286
3.939855639 5.0411710144.846266371 3.076090194 5.335702505
4.626176821 5.044929838 4.05268909 1.0011499052.210700235 4.569202137
4.979986632 4.75230014 5.454893317 4.793468499 0.923907003
5.443938586 4.103672158 2.228474452 4.006805426 -0.594899804 -
3.904942368 3.021910345 -0.156413236 -3.68386374 -3.431814675 1.427752764
3.004046614 4.33536286 5.262562624 3.21407909 6.170657317 -0.757207328
2.570230924 4.505633028 3.720396729 3.271769315
TCGA-49-AARQ-01A 0.747616444 5.448050472 1.569148795 4.924470719
4.501031227 8.087639753 4.10340373 4.135043752 2.664781072
3.950407292 4.416971628 -1.67455258 3.932301005 5.070252042
5.513127467 4.762237613 4.086158758 4.669420548 0.32228689 0.54336876
3.169979622 5.79550607 -0.354997547 2.2497498114.758427778 2.764026033
5.769640891 3.613841905 4.997675401 4.652975951 5.106238637
1.704483141 -2.918595187 2.747665762 -0.609466508 3.119839905-
3.427979635 5.5976118363.09265799 1.55063087 4.71722038 -0.830809947

5.294888625 3.196145821 3.81682582 4.357748609 7.189406322
1.945852273 5.260813557 7.722984392 -4.356493158 -1.17900346
4.818759629 -1.923739336 4.522917764 -3.755621877 5.600310145
2.551065342 3.825318563 4.258053707 5.903876963 2.64025566
1.059658268 -0.752707757 4.7698716 3.920764512 4.803312138
4.401379402 1.078031092 5.080099948 6.797374251 2.952339994
0.220965889 5.236419828 0.456521996 6.133017569 4.917368309
5.407023981 5.300796315 6.41711005310.02422876 -0.302063462
3.562174532 1.645302842 -0.332745707 3.217606823 0.764064958
4.1799911956.996066072 3.355615349 5.007361323 -1.797482099 2.91923501
0.881647721 3.38127231 4.070349446 -0.85011662 5.328105241 4.685400237
1.698966329 3.404864122 3.194851371 5.123235316 4.795691339
4.101294184 5.335817856 4.513462483 5.216616777 4.774623573
1.253010403 3.991076076 4.824041007 4.965825542 4.773203653
5.805783709 5.030471281 1.6383016114.805696477 4.470521691
2.360469939 4.189083267 -1.236059492 -2.272966112 3.852607865
0.09616846 -3.698795787 -2.426305328 1.55984666 3.009843846 4.183883362
6.220228773 3.834073095 6.858794128 -0.479624676 2.187121244
4.954155262 4.030837105 3.070215507
TCGA-49-AARR-01A -0.06891474 4.921605459 2.635799835 5.172297032
3.824185407 8.270127433 3.928329756 4.215808071 2.141813823
3.766100634 4.716349063 -2.169205176 3.322470377 4.220251815
4.910529494 4.633250189 3.59891222 4.234307215 0.388127154
0.406649404 2.982467012 5.654220567 -1.054157204 2.123891273
4.592315475 2.922093248 5.827315395 2.747717652 4.632262886
3.089229942 4.905160368 0.989619931 -3.483866915 3.202867978 -
1.428127794 2.651576661 -3.527633631 5.558405086 3.238250793
0.367700666 4.722051197-0.979398494 5.120641076 2.768599937
4.073287962 4.332891716 7.039469193 1.74398655 3.917982568
7.079979939 -4.864158453 -1.451905115 4.156264657 -2.549866409
3.74822357 -4.36812022 5.17813766 2.25428962 2.369308871 4.227072416
6.095943401 2.809922854 1.484495525 -1.043853399 4.753990194
3.921025773 4.230479822 4.221775739 -1.480902048 5.096487235

6.631034659 3.009265295 -0.022029747 6.03358768 0.134084414
5.930913323 5.205344902 4.743646051 5.40864593 6.377608632
10.02980334 0.597083918 2.623613886 1.040311443-0.544754828
3.631815785 0.727210379 4.205248308 6.934245661 3.524550142
4.434004707 -1.902659261 1.884767495 0.102104879 3.260003742
3.945074337 -2.101846115 5.333402775 5.247441485 1.536498173
3.164005598 4.099840066 4.956747649 4.675736297 3.2538311995.33558846
5.220667915 4.968307013 4.344155877 0.843804916 3.079011026
4.6785118684.876582345 4.785229652 5.604885005 5.153861362
1.1165650075.333762925 4.376340999 2.250232554 3.96699666 -0.709411933
-3.585079743 3.530403537 -0.633329906 -3.637744779 -3.318047184
1.424694742 2.951645409 4.23924915 5.329077492 3.122230733
6.205800873 -0.69049186 2.934071496 4.509106586 3.916427996
2.758095929
TCGA-4B-A93V-01A -0.686504344 4.889330861 2.797798809 5.325563494
4.142633535 8.223370972 3.687171712 4.218260971 2.700173929
3.703062619 5.285240362 -1.72138726 4.124921293 4.1113034184.852976892
5.19886855 3.671684864 5.236431342 0.234566916 0.442326535
2.984621377 5.185113003-1.303073112 2.367574937 4.69920976 2.621591527
5.682105384 4.33301731 4.6560118094.271722522 4.8697986111.62314523 -
3.566688933 3.263957737 -0.813113376 2.733517718 -2.637895711
5.598736824 2.327964491 0.220031577 4.69403027 -1.084427837 5.33061513
3.240276553 4.017056605 4.264373792 6.878481281 1.763395806
4.763546173 7.176273577 -5.039654017 -1.162845763 4.506686525 -
1.8684118763.643625972 -4.466712286 5.27936204 2.912485788 2.372313661
3.989955636 5.941899593 2.6951109762.223048258 -1.282701888 4.60063818
3.92626413 4.899012614 4.278186204 -2.283151906 5.071369541
6.787287659 3.007557788 0.001665731 5.604513081 -0.869404494
6.263607841 4.983786473 5.149907976 5.577131349 6.531436921
9.7005621140.538421415 2.769328723 1.699093557 -0.489908258
3.049440922 0.593243724 4.402242813 6.959723216 3.697978714
4.935882081 -1.90906245 2.68126933 0.1107416093.42100415 4.224962205 -
2.439612661 5.3547849 4.421536253 0.843948318 2.801862432 3.751093997

5.1181583214.605830477 3.840068255 5.335636639 4.978325624 4.80082721
4.425942361 0.852931575 3.339104633 4.5828439114.742396529
4.6541041135.853883462 5.104476797 1.757836293 5.081784037
3.253978405 1.430163221 4.016581792 -0.836740671 -3.251205926
2.874167395 -0.384615908 -3.775937199 -2.671117648 1.398591186
2.863491723 4.328463604 5.736920421 3.549649325 6.562929767 -
1.6702091142.2976527 4.647452813 3.744029679 2.116208939
TCGA-50-5044-01A -0.423796526 4.486437898 2.43567115 5.554046697
3.751926755 8.31097786 3.597653952 4.396163546 1.702657226
3.893140319 5.784954725 -2.355888518 4.000663336 4.072962871
4.683521463 4.922833971 3.697363427 4.335053145 -0.103900092
0.339913633 2.735730508 5.096027893 -1.346686161 3.373891863
4.4671494112.704492865 5.753925237 2.866059635 4.604832 3.660425435
5.1115321851.524218271 -3.593346599 3.955588173 -0.888340472
1.830226545 -3.287030469 5.61785544 3.044888828 -0.371989375
4.721079098 -1.143674378 5.054039284 2.139268607 3.895523878
4.172567967 6.772830988 0.865467607 3.148810281 7.176422514 -
5.120729392 -1.633153365 5.090812771 -2.583198871 3.358977988 -
4.334657865 5.0114848552.395816852 2.557327864 3.453278632 6.16641183
2.461924149 2.945654874 -1.194957097 4.739537181 3.926209483
4.317833264 4.278013895 -2.484635252 5.09664506 6.536509644
2.598894896 0.035334673 5.701144289-0.642030797 6.044379251
4.954415104 4.905357339 5.504198621 6.333015262 11.27274726
0.926375925 2.606141303 0.971708916 0.0361163983.355353571 1.18313067
4.376567849 6.849913924 3.347471689 4.767050638 -2.411796528
2.130756603 -0.299305805 3.409566008 4.165993459 -2.639541816
5.319150637 4.350615531 0.512394701 3.030942029 3.465807554
5.095496605 4.829335194 2.837947227 5.33589522 4.546035256
5.161874589 4.3836671190.89389589 1.690832589 4.598063575 4.993712967
4.734492223 5.5779659114.952126051 1.142624309 5.093515502
3.657469785 1.916527241 3.780971583 -0.661650782 -4.322112737
2.925709519 -0.531554936 -3.899201535 -2.18475048 1.708417354
2.732334372 4.569569457 4.685258912 2.953650829 5.97191463 -

0.905071855	2.68290176 4.379637065	3.531019885	2.962160095
TCGA-50-5045-01A -0.433509633	4.665906593	2.894625961	5.524438054
3.958153048	8.333055091	3.747943643	4.379725619	1.797922391
3.748165581	5.677001238	-2.369398112	4.258288047	4.176476993
4.885908968	4.919778734	3.338803835	4.471765754	0.153026735
0.467504685	2.620182768	5.286639703	-1.264238697	2.864926719
4.515814872	2.687668981	5.819840012	1.698659318	4.608975864
3.912961224	4.747285805	1.178447058	-3.755359751	3.562149714	-
0.918402258	2.236075828	-3.218827075	5.591390369	2.628611574-0.123840029
4.725278893	-1.102935836	5.407208977	2.332524362	3.990002062
4.299431991	6.945385319	1.124492317	3.017703801	6.978991748	-
5.193266328	-1.914243806	4.480582913	-2.619250255	3.663061485	-
4.509630879	4.989635985	2.048186535	2.559520508	3.870770955
6.203717784	2.826539761	2.604583463	-1.928065197	4.660309818
3.933437077	4.17918578 4.329209986	-2.159953458	5.098388631
6.383433323	2.7706643110.067453872	6.055085813	-0.426087071
5.970282956	5.073662905	4.534642939	5.562649729	6.294501114
10.55795274	1.384628182	2.546175873	0.923212856	-0.48262804
3.591923391	1.1110032284.386896496	6.851871886	3.605317674
4.792024646	-2.139423115	2.274419232	-0.375115716	3.340723874
4.106007774	-2.652142702	5.333194488	4.793848392	0.614702964
3.184057724	4.038042564	5.0251111024.702145416	2.784522797
5.335542954	5.100397554	4.969207846	4.519903072	0.760705062
1.716645569	4.644367079	4.806081055	4.773290309	5.634104444
5.062975271	1.150751952	5.505625728	3.999533699	2.063153049
3.800232318	-0.496470097	-4.281215291	3.076301766	-0.47954173-
3.782179722	-2.655194303	1.602745224	2.763260896	4.617700107
4.819740428	3.135882913	5.861032958	-0.804509479	2.711956651
4.600013796	3.5949116433.241425574
TCGA-50-5049-01A -0.641284059	4.608330943	2.262494931	5.467608985
4.157213186	8.377889023	3.699036956	4.307085638	1.841006889
3.848321462	5.640846567	-2.149915149	3.772370981	4.56730586
4.912433623	5.006682531	4.008226528	4.144439744	0.317544617

0.5531116882.683645931 5.405619193 -1.419981342 2.982878019
4.585243786 2.755753992 5.798024063 1.127434851 4.616956913
3.963001775 4.766273356 1.330375951 -3.72244209 3.538422758 -
0.831574929 2.306881682 -3.426977223 5.580984121 3.085698248 0.54076403
4.713054625 -1.127068043 5.295658486 2.902978415 3.874910866
4.333638014 6.892386836 1.227382157 3.086869784 7.109035013 -
5.182307844 -1.719724835 4.482588299 -2.888699442 3.870282672 -
4.345487508 5.035399214 2.012138923 2.526621708 4.028659328
6.174332122 2.887739944 2.356950556 -1.980363586 4.681032866
3.93630553 4.1407111094.286385171 -2.426935776 5.091460163 6.433390377
2.767286267 0.138795046 5.990300562 -0.482432605 6.0118824855.11799344
4.5373411465.454985554 6.360868156 10.68505575 1.272846621
2.7611555840.831316107 -0.502564824 3.564504268 1.067591795
4.304920505 6.889457747 3.614944079 4.829753405 -2.009124492
2.283175904 -0.288438341 3.363794073 4.153760395 -2.672922865
5.331730624 4.860738316 1.235205936 3.40673025 4.141239654
4.951956314 4.619557774 2.941035636 5.33544251 5.074632061
5.000592251 4.514156374 0.697248855 2.904272401 4.518265963
4.749368644 4.786425748 5.621637738 5.228780356 1.151317424
5.535812653 4.170460083 1.927553935 3.747007022 -0.685851311 -
4.06723685 3.229995348 -0.575512646 -3.78989279 -2.917234241 1.567590425
2.618527027 4.491671266 4.824448709 3.204059409 5.955241551
0.066323214 2.955178247 4.5115494923.665041477 3.150648699
TCGA-50-5051-01A -0.995248273 4.7153112732.962734538 5.354759298
3.924942912 8.293518032 3.71112125 4.277667488 2.228012763
3.452025431 5.470930278 -2.039563133 3.631829541 4.397265462
4.742033709 4.878303382 3.440434582 4.596787271 0.230478378
0.326326964 2.880489181 5.212684414 -1.330346701 2.531282819
4.605197317 2.718889691 5.743656686 3.974828107 4.570479479
3.77106205 4.803214825 1.232436958 -3.763008489 3.3524268 -1.124729294
2.39844889 -3.338132055 5.591802256 3.056230095 -0.156641306
4.702822379 -1.176820327 5.101020531 2.864753864 3.995675697
4.3570115227.001527144 1.4368119994.259092682 6.85881741 -5.12790875 -

1.452626162	4.301965624	-2.181419143	3.556437774	-4.34954019 5.115746133
2.375805735	2.297703429	3.922876111 6.019155976	2.595296788
2.547908829	-1.505860424	4.499056642	3.932143528	4.62716701
4.264430666	-2.0805147	5.070651028	6.650897964	2.975170942	-
0.081565734	6.027975939	-0.766291401	6.121180327 5.065938721	4.977678143
5.579501399	6.505448855	9.680052208	0.818091621	2.653397478
1.371028539	-0.714632736	2.908704616	0.673571356	4.324331631
6.880047439	3.644719357	4.805144602	-2.15771877 2.450464773	-
0.042162397	3.367636728	3.973059826	-2.539357997	5.346294265
4.451964851	0.812643045	2.858470587	3.584333268	5.056090471
4.586006676	3.674454027	5.33547892 4.975789065	4.918267345
4.733326956	0.590365706	2.996079459	4.713312499	4.680207637
4.689998152	5.949034339	4.834680372	1.383185815	5.503313247
3.755723317	1.5500773	3.986704166	-1.070119963	-3.659215937
3.380281107-0.429844626	-3.651496202	-3.410693083	1.557868648
2.751922819	4.382884726	5.667562906	3.155464349	6.237250043	-
1.666128202	2.380462242	4.646337537	3.788229228	1.872935511
TCGA-50-5055-01A -0.417792322	4.721006138	2.868563527	5.464758246
3.939648874	8.351831019	3.894555611 4.373106966	1.904321615
3.720094808	5.611839217-2.340999591	3.99019631 4.346956146	4.942379958
4.835195026	3.592210328	4.330595735	0.229370906	0.540144017
2.690700441	5.335871456	-1.279650693	2.912638991	4.517185864
2.772889459	5.948569953	1.594355606	4.614021491	3.766521413
4.712960572	1.039288973	-3.600800317	3.577861873	-1.093804545
2.241385203	-3.476503936	5.577247274	2.893668323	-0.028377185
4.726923151	-1.044337708	5.337055536	2.432785533	3.974442331
4.3674376	7.038748092	1.128896034	3.234699265	6.952303842	-
5.239043898	-1.807014382	4.350973229	-2.73508091 3.690961831	-4.531690353
5.025999527	2.168133219	2.22299416 3.953752844	6.214512387
2.910070015	2.40819547 -2.020121017	4.709771379	3.936934023
4.119942186 4.356793643	-2.230271287	5.102532749	6.387747025
2.824165497	0.049320527	6.238581371	-0.273921785	5.9788486
5.159558159	4.504256289	5.518615071	6.310110585 10.54269214

1.140431795 2.622787945 0.88676997 -0.510794612 3.652414729
1.130967774 4.32412723 6.8680818 3.678004754 4.773273247 -2.052099522
2.249663201 -0.497607694 3.310338434 4.087809763 -2.656316495
5.339825265 4.898067208 0.92542682 3.335129453 4.2914811455.014206812
4.658919063 2.775347468 5.335644888 5.208450679 4.9701183414.49763454
0.784036931 2.238886451 4.597099409 4.807094446 4.787611523
5.626235795 5.161572812 1.154545191 5.509418148 4.125188622
2.0261135983.805174626 -0.660344958 -4.213094561 3.267621144-0.554163025
-3.780708928 -2.838529573 1.608474424 2.805240131 4.58989237
4.983756322 3.220655955 5.955179848 -0.17473987 2.86426111 4.594467795
3.700895042 3.089574216
TCGA-50-5066-01A -1.214038505 4.30490995 2.558521496 5.657264989
4.056683195 8.415829297 3.837379762 4.367912571 1.836371574
4.201952996 5.651362425 -2.099033397 4.199483152 4.694863963
4.851862328 4.772243344 3.404519845 4.503969334 0.224473168
0.373334015 2.487222708 5.404024536 -1.824923979 3.219725275
4.59152987 2.295429855 5.602963598 1.236298978 4.438931656
4.082978255 4.475020649 1.404775506 -4.18637159 3.622881836 -
0.622058571 1.591920923 -3.70764854 5.608255676 2.242337012 -0.105510005
4.711865454-1.325362701 5.456606935 2.343937481 3.712584447
4.254228959 6.909580199 0.60493701 2.570351252 7.010567108 -
5.481266447 -1.830699687 4.766780134 -2.743393868 4.110173413-4.114081705
4.706350644 1.613570583 3.558824655 3.808588909 6.0480292
2.798525196 2.829004081 -2.046238465 4.454648091 3.940408334
4.245337438 4.246893938 -2.918064106 5.08667058 6.325269639
2.650522586 0.251677887 5.760842213 -1.159509846 5.929626129
5.013379674 4.367355563 5.473445597 6.441670531 11.01262728
1.192605962 2.697446812 0.875383215 -0.626130973 3.111291489
1.0112358654.380749706 6.82821308 3.518455256 4.911677141-2.549496502
2.227724949 0.095442555 3.426547901 4.319390876 -3.019024338
5.30151314 4.921555748 0.224794937 3.184371623 3.9166311654.957052628
4.709413094 2.81976206 5.335197079 4.867076498 4.9487411474.859137968
0.304033972 1.248196967 4.457414353 4.560571301 4.800523594

5.548960393 4.944372638 1.26171044 5.267628342 4.157324793
1.833906524 3.619733961 -1.026059306 -4.40525292 3.495949459 -
0.653140463 -3.930827762 -2.028075712 1.450653189 2.1165405744.253788031
4.610283666 3.2111961545.620634339 -1.878081475 2.148174644
4.564507823 3.464155167 3.347550311
TCGA-50-5068-01A -1.145105846 4.361946301 2.684302932 5.642095108
4.184238447 8.387300559 3.682947637 4.377152358 1.832161013
3.891478144 5.559603108 -1.986124284 4.1150497854.796836224
4.818520652 4.791759821 3.0211136434.182314218 -0.051409603
0.388694194 2.595834861 5.417800404 -1.553388068 2.516790363
4.587742238 2.6211705115.721690256 1.594574297 4.45874957 3.659160583
4.371249505 1.096000762 -4.046399065 3.16909757 -0.847245239
2.180976108 -3.807286643 5.591848159 2.309746846 -0.819451179
4.722495906 -1.211464907 5.52974405 2.214752297 3.8113405194.378769716
7.181448863 0.5494221132.913408309 6.903905456 -5.490830414 -
1.761447824 4.603911291-2.819935643 4.237486891 -3.768769812 4.711321699
1.8611733793.542573468 4.069040049 6.0831011562.871959843 2.395092774
-2.028367639 4.362239234 3.946640873 4.409518296 4.132842036 -
2.206904841 5.080629369 6.177274034 2.839431617 0.263649468 6.06517407
-0.890488032 5.925284469 5.153895896 4.330331854 5.483605514
6.402683743 10.68687414 1.128779796 2.720756504 0.980893132 -
0.753825568 2.828829374 1.023499752 4.347861299 6.81636406 3.649496207
4.84200081 -2.58807702 2.1567442 0.37156031 3.357716067 3.991856988 -
2.846222743 5.323410725 4.926790723 -0.079168713 3.0230191164.107489614
4.957172997 4.709093776 3.0510831165.335095142 5.1127926824.927090035
5.190554223 0.255964587 1.536346699 4.657205406 4.569326043
4.808832088 5.581549121 5.049580169 1.1182892275.386630661
4.464561307 1.92048988 3.732082484 -1.188518779 -3.803375199
3.796405985 -0.205685432 -3.823461615 -1.948409408 1.599634342
2.299012158 4.409038717 5.054872764 3.281878982 5.633321668 -
1.775721702 2.321290242 4.768728143 3.625700918 3.248622152
TCGA-50-5072-01A -1.252977676 4.8115299032.979417492 5.490149792
3.862690561 8.3112688263.306557397 4.24957745 1.913648272 3.635134994

5.848176547	-2.251159112	3.4979115224.64200242	4.562254948	4.842537428
4.037655177	3.820991371	-0.012574495	0.37313102	2.670988509
4.533844497	-1.612999007	2.6811452634.548002812	2.553658004
5.725869438	2.1121063814.480552676	3.974891778	4.732296843
1.213071327	-3.884001176	3.193792773	-1.266060598	1.949483076	-
3.279673285	5.601313242	2.660608735	-0.231123425	4.688180407	-
1.209653244	5.08107948	2.884745099	3.895630202	4.286895907	6.873602931
0.844242096	3.4508094	6.743861956	-5.646498661	-2.026581943	4.98191655
-2.869632863	3.44281665	-5.035920306	5.042827121	1.445222206
1.937475425	3.725433091	5.9694998	2.741645627	2.789124052	-
2.189412025	4.497915508	3.934762203	4.000556645	4.308534912	-
2.372542349	5.084917637	6.310779587	2.786750883	-0.13761315	5.685256294
-1.078673849	6.172156378	5.001983676	4.63859473	5.527037017
6.282676157	10.211605441.2849781172.491044087	0.540677806	-0.591156219
3.18878046	1.0921192354.296953652	6.820928616	3.6135914115.002750701	-
2.175071607	2.428536557	-0.469670154	3.312068767	3.859551403	-
2.863082179	5.35138242	3.919692355	0.702815089	2.879656293	4.20017094
4.972233406	4.554543171	2.923719994	5.335345149	4.995890396
4.8512621174.149536175	0.577051294	2.516357818	4.714604877
4.687612096	4.721349838	5.899479952	5.1120487651.096540384
5.6224119523.913980764	1.459624427	3.702722529	-1.2197312	-4.469377506
2.501073168	-0.697094729	-4.034073916	-3.989994278	1.512494674
2.345142862	4.417017741	5.179420051	3.365763733	5.990148353	-
1.728998695	2.528645464	4.4103061123.578713645	2.291104572
TCGA-50-5930-01A	-1.132964124	4.566049431	2.753613664	5.463667059
4.078655521	8.370957525	3.18439464	4.244362749	1.896856731	3.54678318
5.416107393	-2.491850787	3.44895146	4.503224807	4.555659518
5.094476325	3.776479393	3.820400153	0.530296574	0.550592234
2.73501862	4.94589907	-1.564921087	2.529830655	4.518754538	2.92113877
5.305920415	3.131788326	4.42608149	3.180593507	4.838325822
1.074618835	-4.16612296	3.48548775	-1.191724186	2.508988992	-2.532029515
5.592636304	3.256975949	0.904960975	4.707978942	-1.334581937
5.068388613	3.0791160024.058659642	4.18445052	7.051008858	1.413864509

3.277291568 6.683136235 -4.919299559 -1.766755423 4.728845682 -
2.89671795 3.213642501 -4.671156095 5.03513969 1.718627879 2.335798485
3.851449159 6.059329956 2.883933912 2.276031318 -1.789320545
4.516725358 3.930626272 4.226746197 4.174605494 -2.202242561
5.080578352 6.357187845 2.88884352 -0.164234346 5.63201862 -0.893559106
6.029170707 5.191016966 4.595696795 5.4811684026.292937907
10.66681432 1.717657481 2.284830319 0.384498597 -0.56687363 3.47736261
0.918815281 4.340931935 6.856391618 3.7113292664.731416353 -
1.980279023 1.931965736 -0.220690491 3.307591076 3.885430898 -
2.662417466 5.335368741 4.478837229 1.002994535 2.944228046
4.292170803 4.9163888 4.635441898 2.876287371 5.335202639
5.144535347 4.816601747 4.24992605 0.467427627 3.094164253
4.607673036 4.640655541 4.736573451 5.600673927 5.138922931
0.93053312 5.866313069 4.1665011931.789219519 3.81745343 -0.579082446 -
4.001343572 2.717688494 -0.507391715 -3.511561615 -3.59851997 1.686160841
2.637592588 4.378327767 4.914568334 3.156904594 5.8741924 -
0.447569373 3.235147772 4.43987799 3.552987872 3.0063087
TCGA-50-5931-01A -1.453086871 4.564181814 0.615258221 5.398607942
3.926853193 8.053666849 4.43202744 3.979566576 2.486655021
4.334424572 5.008042642 -1.497751923 1.710371374 4.819240927
4.557988588 3.968969952 5.08688458 4.798251709 -0.686917692 -
0.042179046 3.067818954 5.869869236 -1.907792051 2.506239028
4.791847448 1.582267887 5.513582191 2.668662803 4.597965577
4.874256743 4.918631056 2.167231612 -3.788285131 2.804611312-
0.516951332 2.048745231 -5.159984365 5.588714914 3.670891197-1.150947627
4.653189982 -1.31835917 5.249761025 3.89570139 3.480905883 4.165493671
6.732162255 1.1139502894.587233953 7.511938275-6.152207748 -1.064977874
4.786168131 -2.816664759 4.191022095 -4.082782031 5.000866436
2.090638402 3.144532233 4.195786141 5.556525295 2.411782075
2.550502597 -1.796231288 4.578834219 3.916043622 4.543881507
4.335369986 -2.373816798 5.076338662 6.633828941 2.761692892
0.529369158 5.26418541 -1.377914244 6.174420073 4.953153977
5.030312818 5.276581401 6.714730372 10.41056048 -1.128522429

2.925569451 2.080503096 -0.308131004 2.496049362 0.325704419
4.083040192 6.857178228 3.173255227 5.188391906 -2.258067306
2.315072448 0.060021027 3.478520943 4.107328307 -2.601113202
5.325700679 4.380769218 1.718497548 3.575945902 3.053286191
4.958208221 4.844384206 3.946696358 5.335326868 4.319259276
4.89034618 4.27466353 0.37006404 4.045559875 4.264480428 4.617265844
4.719539593 5.6117766094.82694055 1.123366776 4.54310132 2.795067928
1.254566618 3.807597109 -1.325638025 -3.506551035 3.753722853 -
0.783634001 -4.7414069 -3.457708989 0.606877307 1.813929685 3.33790877
5.717787 3.68678628 6.395445531 -0.848864495 1.757364039 4.163149623
3.749759713 1.452298435

TCGA-50-5932-01A -1.079000484 4.524628941 2.907428525 5.462982723
4.362050309 8.301685222 2.966413263 4.254696095 1.640292897
2.815048305 5.238891171-2.527900253 3.0022021154.944890717 4.482437044
5.0455242113.622969867 3.562499509 0.426594075 0.426344149
2.570427159 4.9614081 -1.622568468 1.880955437 4.542994477
3.134737258 5.543007458 2.664618501 4.337268435 3.37995559
4.901340377 0.820343523 -3.856069088 3.02293744 -1.221645902
2.788143745 -3.833482701 5.607949677 3.742989383 0.853968322
4.708889631 -1.294334125 5.028312442 3.405944741 3.881113821
4.200979244 7.089310628 1.221094035 3.767363553 6.340600356 -
4.859210867 -2.07754456 5.060307951 -2.664533322 3.031294985 -4.789154149
4.895380464 1.877701593 2.171595942 3.891921735 5.858358804
2.652608531 1.862044529 -2.489169994 4.450858076 3.955737714
3.865279965 3.982597314 -1.903950171 5.065163298 6.160656717
2.946298148 -0.114968539 5.733662495 -1.012694689 6.103899339
5.2116473354.682343192 5.432354147 6.38399571110.51477847 1.275560347
2.64427318 0.583006077 -1.203558324 2.538755759 0.831985243
4.344881383 6.837429055 3.814307153 4.671746273 -2.132254588
2.170500572 -0.19508319 3.236995752 3.494802974 -2.677514216
5.381853968 4.218905284 0.599057394 2.528138753 4.336763053
4.936342672 4.794054581 3.360438 5.335262136 4.974676993
4.795883999 3.936984339 0.2641211063.573383949 4.367221759

4.660960335 4.733882062 5.686679471 5.1553758110.573944686
5.680481535 4.410007201 1.6632672113.927646962 -0.755601602 -
3.917799588 2.356385862 -0.182548576 -3.386546031 -4.009088928
1.554086636 2.754418805 4.308301314 5.305656719 3.63571548
6.030447979 0.062338723 3.02102717 4.588575048 3.5631164392.489123565
TCGA-50-5933-01A -0.751877433 4.372667124 2.255891521 5.58293455
4.177435654 8.299167675 3.204183798 4.333467917 1.393919378
3.514959602 5.634519724 -2.718976256 3.391265982 4.676576177
4.548519064 5.03278642 3.865180192 3.455918503 0.581818528 0.44688396
2.39604032 5.0410429 -1.668047361 2.900164644 4.517533416 2.722789413
5.447564291 0.877202173 4.4114505153.862807664 4.9311233531.242121162-
3.879551906 3.809572873 -0.663814408 1.991905681 -3.282899025
5.622673388 4.086309955 1.192635974 4.698333466 -1.369938986
5.053568962 2.962086739 3.900007127 4.097230777 6.662237392
1.185484583 2.929190243 6.598374937 -5.019266122 -2.14846107
5.341624778 -2.527920163 2.997685745 -5.044124427 4.829007525
1.62479429 2.320860582 3.637095022 6.025124617 2.501351557
2.683751047 -2.663770902 4.678265205 3.949525754 3.447299975
4.453398075 -2.35919349 5.085604287 6.173984207 2.606211786-0.101389148
5.589846812 -0.883930265 6.063663303 5.010092942 4.436745671
5.498026688 6.289876835 11.573279990.810969362 2.642444876
0.513303535 -0.679799083 3.271535258 1.147380003 4.324653222
6.7857451 3.391059267 4.849959875 -2.040274311 2.340588848 -
0.549828075 3.2947422 3.737063512 -2.680214987 5.349377913 4.359064762
0.997336459 3.006052567 3.756055945 4.936819419 4.955130426
2.504300959 5.335413571 4.548257473 4.97723656 3.785557155
0.554029652 2.415763198 4.347852359 4.784896377 4.757598301
5.432533337 4.738234666 0.526214356 5.5411190224.090187006
1.834507786 3.644805379 -0.038405955 -4.587188996 2.339903594 -
0.237750446 -3.579873908 -3.643157695 1.58749349 2.65576267 4.383229503
4.5860871193.188108587 5.894324387 -0.008693566 2.983287675
4.080275219 3.381288314 3.145789005
TCGA-50-5935-01A -0.811903568 4.637518255 2.7299557 5.442675935

4.266665555 8.289597522 3.376857722 4.259617937 1.936803453
3.215081827 5.096869347 -2.239243757 3.370721876 4.596948766
4.731542091 4.884221407 3.556274031 3.974918667 0.722098351
0.457012461 2.770853946 5.375383067 -1.375366733 2.306797023
4.573742383 3.130045 5.402673776 2.965794503 4.508522165
3.684642158 4.931094481 1.007816063 -3.781194287 3.403665676 -
1.01474132 2.997482215 -3.375362446 5.586332571 3.456291083 0.987159336
4.713169195 -1.22301311 5.251966497 3.1122948563.94000692 4.273851877
6.998143235 1.497886388 3.643647839 6.570183576 -4.757232023 -
1.803469547 4.281047276 -2.547500148 3.398263527 -4.361893607
4.9259119922.120792883 2.414188274 4.041771256 6.004766642
2.746342879 2.003064901 -1.950491909 4.515650419 3.940444931
4.18489635 4.150240625 -1.661968675 5.085427001 6.334447798
2.945662633 -0.024501442 5.860543964 -0.610652252 5.984495738
5.147553154 4.567390971 5.475850548 6.444677725 10.44057041
1.2117213592.504998746 0.961461204 -0.808140377 3.01701151 0.838084937
4.313217873 6.845162277 3.704740006 4.596945694 -2.119877152
2.121207387 0.133095253 3.310377133 3.76880753 -2.336782954 5.35934071
4.746506039 0.971231282 3.048385783 4.1137829934.947068816
4.656266897 3.376234126 5.335414206 5.074736593 4.889556765
4.516376186 0.369646945 3.813993238 4.527413079 4.704600634
4.76299102 5.658908733 5.262835091 0.760239035 5.625244247
4.333877575 1.991662261 3.890692142 -0.629325698 -3.40271633
3.145286164 -0.414034176 -3.45798203 -3.35715747 1.629795548 2.869411456
4.393651879 5.285268941 3.296176027 5.963610995 0.026857528
3.153701971 4.545920917 3.679741874 2.774903596
TCGA-50-5936-01A -1.376810776 4.616241756 3.091716818 5.494285845
3.8563959 8.351492591 3.137481209 4.328710459 1.724482574
3.267878776 5.687231668 -2.616287128 3.083916766 4.445586655
4.3522118364.840319182 3.62779606 3.662809307 0.080160696 0.15326246
2.542180944 4.675120763 -1.730289312 2.519297708 4.522297802
2.597451478 5.72763107 2.153504371 4.393653204 3.359707747 4.8504595
1.000350209 -4.168027488 3.152191527 -1.362244778 2.132257863 -

3.416957973 5.598871801 3.203415579 -0.221503027 4.702867761 -
1.351029244 5.096015479 2.694131841 3.93420798 4.202525272 6.885522044
0.787499734 3.26540673 6.38354347 -5.481939196 -1.993566979 4.866131762
-2.939170842 3.246082708 -5.184048001 4.932021481 1.463089298
1.902978676 3.655048868 5.975575966 2.41791425 2.587568513 -
2.70507467 4.517477476 3.936582495 3.887662502 4.157508187 -2.594630816
5.089073706 6.298596221 2.876913339 -0.193024051 5.829080721 -
1.283163276 6.11624347 5.09083578 4.599357595 5.52248629 6.302517471
10.34536628 1.376243953 2.227306403 0.50218261 -0.677298801
2.710654183 0.970288161 4.338014304 6.783938789 3.542651581
4.675954707 -2.47706346 2.021351873 -0.692963502 3.300940027
3.869195768 -3.164838325 5.34914845 4.26893919 0.299010492 2.683892296
4.06017833 4.957303571 4.657349024 3.04080953 5.335383041 5.131592817
4.805363632 4.001637803 0.313133512 2.161728247 4.521242278
4.731267178 4.731742793 5.72358581 4.978667831 0.716995256
5.710405742 3.96384447 1.44185923 3.751709373 -1.244858509 -4.433011966
2.392855725 -0.694270327 -3.862859481 -4.051035561 1.450296547
2.559014755 4.305208244 4.915612593 3.26213547 5.78724408 -1.093544494
2.937013723 4.415809989 3.476572546 2.377720814
TCGA-50-5939-01A -0.700800745 4.513426906 3.162181953 5.522511216
4.040020248 8.26894961 3.206667084 4.344845215 1.578307915
3.3511725365.570340457 -2.734875122 3.280920377 4.319534294
4.449614629 5.0211834953.704074515 3.702940184 0.372150917
0.359188239 2.533417694 4.836947046 -1.457231458 2.741734941
4.4815961162.944322672 5.597575613 2.565128333 4.392880173
2.909230785 4.9311040950.888723406 -3.896389037 3.300007423 -
1.2224631122.372722622 -3.066364481 5.604565973 3.166759395 0.620424319
4.70658429 -1.395159576 5.099013041 2.638754785 4.012960184
4.098860354 6.9119528470.9566691 3.270486567 6.70131483 -5.299045109 -
1.950996713 5.277046394 -2.765993574 3.096936876 -5.051255427 4.91123296
1.774560773 2.465932739 3.567324595 6.063779934 2.388487785
2.322235154 -2.234156778 4.670049907 3.937378748 3.863030914
4.280324994 -1.962707196 5.09061061 6.199789782 2.773475989 -

0.128351049 5.752589515 -0.818452907 6.053980766 5.104079832
4.547219806 5.502964161 6.212715675 11.1341419 0.526441309
2.450299134 0.417062327 -0.374142651 3.298350829 1.133661723
4.312320405 6.791867422 3.494125916 4.6906597 -2.319112538
1.864198678 -0.688519919 3.25335784 3.728111951-2.480179725 5.339512652
4.423470476 0.467897405 2.803409666 3.977566687 4.946877771
4.930916196 2.701728892 5.335405934 4.7621149974.912669883
3.816835457 0.545877657 1.873975705 4.556573591 4.840640603
4.75578373 5.537471646 4.978294966 0.585015288 5.6103711254.131456437
1.906813386 3.734923606 -0.124313023 -4.377564671 2.416688047 -
0.499389029 -3.756031042 -3.63900507 1.607817529 2.828504758 4.519635624
4.797936514 3.136362982 5.970287479 -0.793470722 3.043288843
4.254595956 3.517716235 3.275002254
TCGA-50-5941-01A -0.495827806 4.685382364 2.279696642 5.413565175
4.380925 8.228867979 3.2073711914.306992121 1.482992049 3.398618171
5.55654082 -2.581158785 3.665331443 4.574050978 4.813190816
5.210737574 3.98379473 3.758516241 0.308339957 0.487184561
2.5974691114.908616794 -1.301801437 2.607149556 4.5041146522.925918837
5.773553056 1.357894427 4.555073079 3.800212374 4.988097634
1.088126877 -3.586248425 3.304650285 -0.971995346 2.590087069 -
3.196661628 5.609348309 3.6112024190.930365102 4.707544243 -1.174943443
5.197308601 2.96856979 3.970756516 4.204435551 6.9311310871.412033932
3.594892406 6.682162485 -4.893900256 -2.102478092 5.035498248 -
2.555903653 3.193123647 -5.07093388 5.076651926 1.78036467 2.079702634
3.782477009 6.11871599 2.632542579 2.216287589 -2.384539148
4.706707031 3.939077454 3.564668224 4.350232185 -1.585667863
5.089392791 6.245599886 2.783731477 -0.115090581 5.81019105 -
0.424489764 6.076956732 5.095277333 4.6110380945.488294252 6.17178742
10.80919147 0.915070927 2.719480204 0.552939489 -0.685350261
3.553436178 1.044857981 4.328612526 6.835863982 3.614064725
4.947728097 -1.863935208 2.336204355 -0.713337208 3.275907393
3.638949807 -2.482322399 5.35832804 4.232906193 1.081602588
3.138343598 4.000331833 4.949271839 4.83538016 2.912446783

5.335454722 4.794446917 4.932707193 3.889975192 0.745406086
2.79401697 4.531174756 4.829249702 4.760462568 5.680437708 5.167852583
0.801752966 5.657992308 4.050087127 1.958309488 3.83652449 -
0.278740616 -4.523054972 2.344685523 -0.094026247 -3.548166726 -
3.920996822 1.733963002 2.816209149 4.552314825 5.125427404
3.525489257 6.0953671 0.346850144 2.920170161 4.43325139 3.602849581
3.369194411

TCGA-50-5942-01A -1.214105341 4.663095686 2.52489708 5.426265211 4.241361089
8.324153611 3.541811437 4.290469806 1.923501579 3.025021211 5.15216251 -
2.2688949 3.146203602 4.582930172 4.711859499 4.83353199 3.374918745
3.824479629 0.822343062 0.585178097 2.465718078 5.314486649 -
1.67869644 2.386272953 4.56135521 3.008529619 5.679060341 1.976748386
4.425062987 2.891271837 4.763580031 0.85244009 -3.716029693
3.673099281 -1.033551516 2.830939303 -3.252516209 5.57335496
3.638161414 1.425199067 4.715908586 -1.19093226 5.149371948
2.982107634 3.966250409 4.277299033 6.914325652 1.549919445
3.655012314 6.464033018 -4.623498396 -1.688192341 4.296466178 -
2.408102038 3.264938354 -4.521361526 4.990789788 1.977108145 2.56792783
4.067337611 6.013983107 2.751928092 1.583482083 -2.563740182
4.649896944 3.939320121 3.94585728 4.179345305 -2.495007457
5.087800925 6.389580772 2.98978322 -0.063767222 6.141937538 -
0.594454682 5.943231358 5.217374319 4.454418523 5.420725924
6.365382307 10.64823498 0.571487149 2.645866277 0.844314959 -
0.854723208 3.012968899 0.793541065 4.295258113 6.908968102 3.619400113
4.49386012 -2.013534079 1.861655541 -0.186850513 3.239046139
3.720261719 -2.850907282 5.340725584 5.10481547 0.965952033 3.0307372
4.326721249 4.909101018 4.795606227 3.056893147 5.335445119
5.031353166 4.822895696 4.107020801 0.333093613 2.973046287
4.270279665 4.608863775 4.78209903 5.520236075 5.105515935
1.000384515 5.666233397 4.568645991 1.910656025 3.795119205-
0.584073312 -4.159894894 3.078970384 -0.265226519 -3.238797334 -
3.723628083 1.682069635 2.660538579 4.215369051 4.910404151
3.071168041 6.026313809 0.224177949 3.128207412 4.341966636

3.7208691162.640597427

TCGA-50-5944-01A -1.034206449 4.3214801112.47544244 5.438304503 4.20986362 8.389370563 3.30994546 4.257159335 1.651469398 3.447374301 5.439709209 -2.27850128 3.352919435 4.62921619 4.635654647 5.203029892 3.902743645 3.712687643 0.449569256 0.533367231 2.757790788 5.147462523 -1.467683505 3.006498905 4.503648472 3.24666168 5.473865256 3.151865513 4.477649179 3.12666247 4.97118904 1.281972118- 3.746044669 3.781272981 -1.074696394 2.87965122 -3.116356725 5.590728135 4.160145834 1.490025678 4.715880138 -1.310960874 5.337722947 3.180931756 3.926942352 4.202880957 7.019978248 1.491931858 3.269980412 6.543866396 -4.511457915 -1.688143609 4.648729311- 2.968148185 3.332756093 -4.071532109 4.925458978 1.995272389 2.666053703 3.884914793 6.197556522 2.968254032 2.310318738 - 1.651071919 4.612059505 3.93930526 4.120922934 4.040190677 -1.811747985 5.086646655 6.373419664 2.844754685 0.043312032 5.864616163 - 0.629177157 5.9112153125.213137689 4.522817971 5.391424731 6.364087386 11.040587851.387160653 2.594895223 0.592879116-0.661228928 3.217776075 0.951879658 4.314758101 6.896137316 3.760587746 4.730627467 - 2.030896235 1.895771431 0.065344376 3.3115987123.716062292 -2.401535144 5.337659501 4.622729634 1.25470734 3.103049712 4.144128279 4.907784412 4.737163066 3.191395222 5.335388029 5.164974094 4.990469608 4.29319181 0.46963251 3.68365285 4.4397741134.726681 4.771812692 5.690094531 5.54830451 0.932224377 5.796614043 4.312020029 2.170200595 3.814518753 -0.705904598 -3.59153564 3.239936491 -0.001350153 -3.356578831 -3.361106851 1.89823437 2.674180095 4.463830136 4.775106392 3.225136498 5.971242507 0.630947947 3.318834423 4.364679566 3.598758399 3.105953164

TCGA-50-5946-01A -1.209052899 5.044628415 1.293027234 5.220521921 4.552374466 8.124012555 3.785990107 4.034453093 2.252499892 3.413924627 5.139225955 -1.971096598 3.210122777 5.250932587 5.078089432 4.425372417 4.301326638 4.2119052860.0351117280.484859738 2.78653509 5.414243684 -1.459747733 1.635475283 4.716009725 2.5011364 5.492531452 2.1120992574.652616292 4.90706354 4.997667828 1.51337946 -

3.539517748 2.619551164-0.643918202 2.666892727 -3.663973734 5.594742137
3.844345717 1.247466152 4.686822153 -1.094099526 4.8667594
3.559754098 3.729224632 4.170023337 6.967908372 1.774299082
4.4335118887.166767738 -4.944846927 -1.787815276 4.956188496 -
2.4841131583.866002266 -4.542311391 5.368295393 1.934168947 2.743225553
4.104361025 5.6391174332.667467822 1.715330585 -1.958993439
4.559570607 3.938387941 4.1911607324.323925386 -1.330888142
5.0643141196.425649147 2.833644419 0.256715994 5.19039962 -0.799740699
6.235958178 4.895585002 5.052026029 5.338471397 6.517697156
10.2586464 0.073769256 3.220962992 1.118208306-0.664334379 2.854812159
0.695205424 4.231051047 6.947639457 3.335000195 5.218787822 -
1.745461093 2.893297012 0.003941375 3.308343336 3.83550892 -2.166276337
5.3481811134.208467332 1.349274964 3.102702386 3.712286864
4.992974384 4.726480597 3.6840611735.335387555 4.300019017 4.96732074
4.10693393 0.7311507973.950835592 4.552995993 4.695540985 4.72100437
5.62514911 4.539869703 1.121301853 5.3073119233.985943216 1.550705769
4.001735374 -1.226462837 -3.4855847 2.738107014 -0.117876351 -
3.832596237 -4.084501591 1.062934976 2.364352887 3.83869762 5.841985648
3.816357367 6.461270399 0.3921156612.410491655 4.437798522
3.670503361 2.430493768
TCGA-50-6590-01A -0.284387796 4.65686191 2.03035923 5.386963696 4.041207955
8.2341100063.651226616 4.244641392 1.758882426 3.859068841
5.512613359 -2.625470698 3.151637693 4.499263777 4.727358593
4.665095257 4.085399967 4.127846651 -0.419402121 0.283144303
2.579029868 5.106911149-1.424108999 2.84939546 4.585920779 2.285911547
5.705773739 0.994987344 4.581802006 4.394328909 5.012767502
1.161192373-3.663184519 2.936159325 -0.695117538 1.596750634 -
3.936467265 5.607214629 3.027621051 -0.036896361 4.685815716 -
1.270929304 4.945896479 2.771208174 3.722428152 4.065982659 6.70144005
0.973709768 3.492875924 7.310684788 -5.820177633 -1.973992272
5.318222282 -2.94215049 3.512310706 -5.407827166 5.050712737
2.038060738 2.495058961 3.648514872 5.940322834 2.2586633
2.342817226 -2.430649157 4.8063021163.931965975 3.608739728

4.315026025 -2.159307421 5.087085479 6.407766184 2.565816752
0.12140786 5.32542556 -0.634497618 6.130308392 4.8753493114.857254838
5.422836318 6.33041169810.62215566 0.5122711492.80186363 0.719579523 -
0.34820486 3.189833191 1.099860468 4.310359717 6.84870199 3.048696077
4.956915512 -2.157205039 2.423431241 -0.968017097 3.300619113
4.142826519 -2.222264091 5.334023576 4.305731601 0.706756409
2.976809042 3.453230957 4.992066174 4.81303607 2.814944038
5.335541715 4.225345796 5.133644988 3.70301437 0.963207951
2.308175144 4.282452076 4.959065367 4.753439502 5.484092848
4.5546118860.912219904 4.958728276 3.749782963 1.757259185
3.708685906 -0.637948891 -4.881356403 2.371631469 -0.97109646 -
4.287946662 -3.508465957 1.164194817 2.5811612564.217370012 4.958385634
3.3190411156.208662972 -0.703849034 2.413358751 4.198542308
3.439406407 2.754454668
TCGA-50-6591-01A -1.39975393 4.537746152 -0.440578641 5.145922916
3.979221639 8.257922419 4.554713935 3.982707202 2.863512264
4.633686014 4.403780438 -1.405411966 2.208852289 5.598963536
4.991453364 3.616624106 5.131743225 4.810524582 0.564178443
0.168955081 3.071859584 6.662123351 -2.112602732 3.161487353
4.875414142 1.837238168 5.654577827 2.646721919 4.701788386
4.882670191 5.1186631282.22310099 -3.565134345 3.603226967 -0.311773813
2.174159208 -5.318968889 5.578649664 4.18197701 1.287927676
4.682872661 -1.180129653 5.292149609 4.290010327 3.282853163
4.240931571 6.933396772 1.748607922 4.7185745117.848388079 -
5.195735472 -0.872808733 4.90220722 -2.633840595 4.832966593 -3.516318157
5.283383992 2.485105461 3.574308934 4.360161583 5.453722853
2.406540665 2.073445167 -0.968748936 4.760203095 3.93482795
4.824418246 4.200313534 -3.087951582 5.064706001 6.895396142
2.683782782 0.992107951 4.815302448 -1.014365627 5.960743778
4.86947744 5.549565364 5.074275919 7.054166696 10.68982049 -
1.218148458 3.357937764 2.347187922 -0.287662391 2.741232978 0.38640245
3.995217407 6.937947069 2.974129548 5.150037111-1.897161308
2.241885899 0.732200162 3.482558792 4.595117194-2.477268435

5.303587339 5.148639502 2.414205406 4.068993586 2.513805605
4.953109487 4.778586995 4.584492944 5.335760661 4.145589124
5.313996072 4.16441383 0.64818066 5.240391783 4.332670276 4.657877035
4.779721313 5.396402207 4.621818607 1.182619681 4.438659607
3.298483966 1.301000713 3.944888312 -1.09232063 -2.890161065
4.315750024 -0.919604687 -4.138038967 -2.970761223 0.634415067
1.796404512 2.69047485 5.776386998 3.590603426 6.587201725
0.801703412 1.793952525 4.523098495 3.651892408 2.125709171
TCGA-50-6592-01A -1.422036248 4.596257678 2.06652871 5.29599771 4.295454255
8.356613394 3.399598414 4.259544455 1.667636564 3.660475342
5.55856556 -2.614288032 3.318623123 4.579845982 4.435937807
4.942672141 4.2193371153.572213722 0.07049494 0.282525559 2.61979922
4.850557489 -1.727318562 2.6788711784.510359801 2.480248675
5.326826932 0.326708748 4.496219926 3.955460299 4.879463579
1.016533522 -4.027530385 2.868127127 -0.96943231 2.044283744 -
3.571964355 5.6116575463.595792322 0.472764086 4.68933383 -1.379011177
4.889677961 2.798603748 3.857547837 4.101474168 6.888841394
0.736202965 3.181501612 6.623496942 -5.223112518 -1.960470148
5.160122529 -3.027636329 3.35521407 -5.27037927 4.93257408 1.241933225
1.931888038 3.679581058 5.986894194 2.67585794 2.183755962 -
3.000239387 4.536365909 3.93281688 3.556525758 4.26051424 -2.353893195
5.094651625 6.2831129372.81701737 -0.06122233 5.401941926 -1.106721686
6.0699811915.082024778 4.397758341 5.443895748 6.103690737
10.90975579 1.188707248 2.458522294 0.128723054 -0.837351355
3.287888027 0.967263519 4.295902871 6.739707881 3.548866719
4.779593476 -2.337508351 2.21366867 -0.596928434 3.290241628
3.771580012 -2.91869327 5.342606317 4.310880799 0.751301709
2.940368723 4.146274913 4.878647375 4.7113176162.864901344
5.335207029 4.818948708 4.810731695 4.131457603 0.381056866
2.470776444 4.33371676 4.709257322 4.76264977 5.725531552 5.121547252
0.78732383 5.580554575 3.80940108 1.706786434 3.652021407 -1.066261534
-4.564231881 2.293299205 -0.562788883 -3.751775647 -3.869369612
1.573876522 2.450165677 4.126717262 4.865851512 3.77099265 5.85705197

-1.329484022 2.70020187 4.175358717 3.474443992 3.091628793
TCGA-50-6593-01A -0.413420597 4.681852483 2.499714998 5.460105754
4.220155757 8.232076667 3.220605818 4.274132205 1.644932572
3.363104497 5.619168965 -2.412832852 3.630014448 4.513112428
4.764778683 5.09893496 3.755975912 3.76607709 0.093283634 0.492526133
2.708030771 5.072045813 -1.284914064 2.254617843 4.518275263
3.03661998 5.438235192 1.828025191 4.538404647 3.453145089
5.060621414 1.230485337 -3.545333916 3.298922284 -1.062529364
2.371432634 -3.224113703 5.603339132 3.621087577 0.566916776
4.716191097 -1.100166332 4.892780791 2.826732446 4.017459052
4.236598171 6.9401911971.240320353 3.34091902 6.98955351 -5.013571764 -
1.954496686 4.779433224 -2.600563138 3.389784429 -4.674576225
5.161897353 1.960913731 2.298313936 3.76848171 6.069748848
2.600173146 2.220331622 -1.84127579 4.661890013 3.93758019 3.928933994
4.289232882 -1.895089474 5.085098218 6.344420269 2.765312206 -
0.0210114915.805068234 -0.437716841 6.1110433925.031977869 4.727193991
5.538939224 6.223146641 10.65260179 1.04567715 2.668595932
0.573572764 -0.620002759 3.27750656 1.058355606 4.305386552
6.878359788 3.526173643 4.842904456 -1.948410382 2.272488503 -
0.3158011023.315233281 3.731861573 -2.528249913 5.354368378 4.303426557
1.149547922 2.931228265 3.938949516 5.0191178654.737715155
3.019175653 5.335550083 4.74360763 4.977700552 4.315867592
0.803526001 2.886414999 4.635550046 4.871008134 4.73257784
5.562445442 4.977292237 0.91734221 5.634256691 4.058837476
1.935623397 3.822056666 -0.581804608 -4.158008475 2.747621479 -
0.32609763 -3.608566086 -3.701428549 1.664552518 2.8110567914.520967834
5.045449248 3.220900156 6.059705349 -0.137732993 2.942244657
4.405227422 3.734758982 2.944816556
TCGA-50-6594-01A -0.542733368 4.778642462 3.017695915 5.684869344
4.371661078 8.128568599 3.4746468 4.269883802 1.862329866 3.46096003
5.502198055 -2.298556831 3.800292452 4.770949085 4.829770641
5.1129806873.4901411294.110359733-0.026390019 0.514608268 2.713331259
4.667045162 -1.166965605 2.442178642 4.534965354 3.010078322

5.320836564 2.834045685 4.559964708 4.442957325 4.810742942
1.426953419 -3.581409534 3.215901077 -0.757183083 2.76318751 -
3.293381946 5.628128874 3.060771708 0.56411657 4.716214564 -1.125114745
5.329975266 2.91966925 3.89724315 4.148906297 7.132471346 1.089304641
3.983576172 6.936549492 -5.067763704 -2.125178477 5.359604934 -
2.497209722 3.341879865 -4.252371267 4.976577813 1.830434039
2.809320426 3.645945058 5.940604747 2.754159676 2.599701127-
2.514423209 4.523000966 3.94713179 4.257260543 4.463504438 -0.945017212
5.088697905 6.061352858 2.667748731 0.082988191 5.473504658 -
0.941518608 6.185819366 5.026094502 4.725101258 5.565826653
6.430794878 11.385889440.453503906 2.804036345 0.958655941 -
0.74141308 3.280380821 1.195524705 4.3960881 6.770633932 3.798340287
5.026508134 -2.218224902 2.749573927 0.280168569 3.383288424
3.779777266 -2.026195819 5.3661102654.068065621 0.557386489
2.679128398 4.1137277945.080774083 4.972699026 3.138682922
5.335573939 4.810049742 4.858173147 4.565472229 0.720229388
2.762183339 4.751482066 4.83789961 4.751864344 5.683786242
5.245387643 0.771974365 5.409625727 3.982082883 1.96905456
3.856721328 -0.832421561 -3.706254701 3.166863009 -0.030034579 -
3.772942732 -2.83749858 1.602708904 2.771049833 4.632744127 5.508868719
3.490451653 6.235471309 -0.535119242 2.800427919 4.566755553
3.586181329 3.245480481
TCGA-50-6595-01A -0.642506058 4.329681788 1.675843128 5.598266136
3.953344519 8.34669459 3.420827941 4.375576541 1.575801358
3.728946166 5.815395027 -2.774664347 3.759921939 4.429847767
4.389994126 4.998459779 3.9208511553.984567715 0.106053834
0.317143934 2.453598075 5.06823725 -1.37798043 2.89050306 4.477614445
2.393470519 5.707432124 1.446230498 4.569569696 3.875147985
5.036509745 1.377469249 -3.88476558 3.493968167 -0.691719721
1.768589774 -3.563303835 5.615709459 3.249251969 0.031108
4.711334591-1.307370828 5.128792038 2.389039395 3.836088978
4.136205168 6.880778348 0.934864406 2.982579588 7.028351825 -
5.451302795 -2.004742742 5.45831599 -2.854823943 3.164635831 -4.796785473

4.898873072 2.055344987 2.709233795 3.503392269 6.135641684
2.217278414 2.426612513 -2.05269811 4.6967497 3.935246925 3.749675812
4.270582878 -1.997717611 5.08247153 6.38378407 2.529236052 0.111836663
5.633127117-0.61180144 5.991526941 5.015749134 4.73947524 5.489194736
6.227514215 11.266540680.907624555 2.60071692 0.684183528 -0.286703893
3.041580819 1.181513548 4.3748342116.792445938 3.284612925
4.898068487 -2.34345179 2.219077628 -0.865591686 3.341567934
3.849199721 -2.571531565 5.330275756 4.33037671 0.484831215
3.186336027 3.617688927 4.965745864 4.875796464 2.701683368
5.33544794 4.400161748 5.147635241 3.780717279 0.841791473 1.44301828
4.310027857 4.904083885 4.756136395 5.640177659 4.854519199
0.752455594 5.214284456 3.682565984 1.890782402 3.756085315 -
0.2908356 -4.685423585 2.64136694 -0.413408989 -3.895010843 -2.785369865
1.560440163 2.638452496 4.532469029 4.575136909 3.047433424
5.859594829 -0.728584809 2.694137989 4.371210086 3.371586539
3.091188153
TCGA-50-6597-01A -0.132066011 4.74717335 2.83941084 5.492488495 4.138742649
8.372376002 3.4891185614.363877169 2.104758899 3.482843905
5.693224944 -2.139971343 4.22320298 4.544685372 4.941560981 5.22792848
3.934017162 4.468393672 0.496021402 0.571451521 3.060368617
5.071945132 -0.949943392 2.656301439 4.437728588 3.240439588
5.532966457 4.930216017 4.732217034 4.1294441175.034430238
1.356285634 -3.432894643 3.633374635 -1.111565069 3.017455387 -
2.729927105 5.596799227 3.486266617 0.504812501 4.728336969 -
0.989990543 5.3075119312.583705055 4.0577150114.184535716 7.091916146
1.181727284 3.901397391 6.924022482 -4.515123236 -1.470454763
4.356997098 -2.8002356 3.744067749 -3.715660513 5.13976217 2.844512181
2.644169038 3.761493595 6.277346143 3.133876456 2.233395151 -
0.858547157 4.505846393 3.92570919 4.782388437 4.307431165-1.457813914
5.085665571 6.4215589 2.879191324 0.010214448 5.969713073 -
0.039055467 6.055234081 5.198132916 4.967505043 5.489237755
6.295005713 10.59716441 1.594107206 2.721785262 0.95185393 -
0.179942104 3.349770185 1.038326979 4.450215518 6.891666892

3.903741327	4.740676438	-2.071895059	2.550334015	0.414765246
3.400813963	3.855652964	-2.457787854	5.3394511624.156750099	0.71228264
3.188106105	4.316448759	5.080550889	4.542424647	3.629255054
5.335630836	5.445374352	5.052739208	5.080445976	0.899597676
3.505965009	4.671989439	4.905637758	4.717754035	5.846321086
5.608148095	1.2119920365.760712854	4.22154143	2.137349659	4.020493551
-1.201557875	-3.240305881	3.271366256	-0.204414266	-3.465237877	-
2.059027903	2.2112047982.895853943	4.672431847	5.235354992	3.345924149
6.154105145	-0.07255001 3.556988672	4.941422426	3.837529745
3.101967544
TCGA-50-6673-01A -1.01958925 4.65611816	2.495247122	5.373777243	4.219502273
8.364342242	3.220296091	4.344179903	1.687699875	3.300548107
5.77015271	-2.549026882	3.492870399	4.538971955	4.618794206
5.183959384	4.043681722	3.637720054	0.655010747	0.582333782
2.669512661	5.17796311	-1.369018574	2.684663139	4.482555805
2.955595105	5.68453145	0.645949317	4.554544312	3.525366414
4.840196043	1.084131667	-3.600396284	3.561611058-1.110795394
2.468449987	-3.254276054	5.5968671173.767087167	1.161892507	4.71446049
-1.228628697	4.999096981	2.997226426	3.98845328	4.312367487	6.99113727
1.242050842	3.404597739	6.462780683	-4.555135365	-1.922508397
4.724829088	-2.503562967	3.340989199	-4.837312322	5.007556503
1.6911250361.866706397	3.916674541	6.146584442	2.756442125
2.299440919	-2.465480655	4.6469517	3.942584387	3.793968226
4.283166348	-2.050991203	5.088951026	6.321228428	2.914553896	-
0.209331438	6.002572188	-0.578404728	6.0117029355.2059118394.453661206
5.524523533	6.26210811310.64907456	1.3893761132.642440333	0.423366991
-0.759994961	3.14950384	0.975352066	4.308429431	6.794772681	3.78331252
4.745037647	-2.005281188	2.136228046	-0.40906231 3.287325602
3.578108076	-2.652479818	5.357561812	4.531401505	1.185782687
3.172104899	4.629815258	4.913217789	4.733003888	3.024678292
5.335449235	5.23245969	4.802337707	4.081779073	0.597537897
2.872081951	4.407958257	4.670646979	4.749448269	5.762706279
5.2891146450.815690243	5.7865119613.998579138	1.878210524	3.789053533

-0.365053725	-4.231517347	2.602084257	-0.211812525	-3.275218699	-
3.691514752	1.798980036	2.791349619	4.546998519	4.969250265
3.259535662	6.080902125	0.469710573	3.244840615	4.45643192
3.705574255	3.233381202
TCGA-50-7109-01A -1.207838206	4.530396071	1.884682212	5.33236239
4.3385409118.355498893	3.429733546	4.184543369	2.088571446	3.62502978
5.30919015 -2.063307644	3.351986651	4.726672249	4.650468574
4.970992788	4.153690474	4.094464704	0.662599278	0.409747741
2.708085515	5.305765416	-1.711383415	2.694341035	4.666172849
2.640960174	5.653308806	2.227966498	4.500930198	4.223567454
4.789893419	1.238567506	-3.858339985	3.379788775	-0.78408166
2.610262874	-3.752735721	5.596383193	3.589286946	0.793783114
4.694185109	-1.321259554	5.433789856	3.540238503	3.818665262
4.2391176686.950572267	1.520089262	3.999218989	6.74271899 -4.941290431
-1.718351701	4.820765718	-2.416240646	3.66700828 -4.769836649	4.99061218
1.832982641	2.34861001 4.028222124	5.9533494	2.749162751	2.296536986
-2.261309882	4.547808781	3.940310243	4.005989676	4.242637111-
2.621500881	5.076409681	6.406237294	2.9309951170.027076763	5.697997257
-0.890352425	6.044917635	5.127967436	4.716226685	5.392146285
6.499220131	10.1745481 0.964713939	2.690264507	1.09971688 -0.983399086
3.134457297	0.656710288	4.2713711466.849792917	3.680683152
4.948057971	-1.918934469	2.240800207	0.023651961	3.329583803
3.876022884	-2.922055275	5.350005504	4.624715855	1.171952957
3.156733431	3.8111347564.92512329 4.693189105	3.38966669 5.33527204
4.946315593	4.842050763	4.185840076	0.307449273	3.779898094
4.415904722	4.582925992	4.74241889 5.723348653	5.0524343	1.116854333
5.521069622	3.874528949	1.56168233 3.858434908	-0.654893291	-
3.79565655 3.126421683	-0.434213826	-3.644750256	-3.81785557 1.261507492
2.502224935	4.100078988	5.360337695	3.659595417	6.166486421	-
0.236350864	2.565323645	4.563599236	3.629814035	2.591985976
TCGA-50-8457-01A -0.606331569	4.768225252	2.959286574	5.328183707
4.04474472 8.256958941	3.674919594	4.259839346	1.940653732
3.070424536	5.060989193	-2.334385157	3.1301123284.287172372

4.819627627	4.763127394	3.604236083	4.094422341	0.619344843
0.4686811172.613686942	5.374475523	-1.384028428	2.19895932	4.575234725
2.99203912	5.649197388	1.776637922	4.488074126	3.187918024
4.890610428	0.91872924	-3.620311225	3.473028967	-1.130360687
2.852520205	-3.229343121	5.570597057	3.620868729	1.16886214
4.704570886	-1.16713977	5.059087957	3.091656593	4.023156996
4.263179539	6.775324804	1.628998549	3.743675698	6.734271775	-
4.805156171	-1.693719164	4.237126679	-2.439684283	3.239508928	-
4.762945168	5.042437329	2.177023776	2.169997198	4.101628493	6.00025771
2.679761419	1.799226178	-2.272747253	4.729892971	3.932766409
3.981873465	4.318431561	-2.059540636	5.095559884	6.423244447
2.974187016	-0.082795217	6.063925902	-0.489237414	6.025648394
5.144300044	4.591081482	5.476987563	6.36873021110.35601343
0.578780107	2.61554117	0.899627706	-0.772773258	3.450366576
0.761371626	4.264050889	6.895239024	3.595776964	4.530021541	-
1.901622912	2.035211792-0.537445083	3.252969395	3.817674195	-2.453975866
5.344759044	4.924766683	1.430072367	3.102250638	4.10755552
4.956218182	4.770281291	3.157950299	5.3355707	5.073050839
4.822410346	4.006473601	0.582482639	3.20766938	4.401639736
4.7291139774.756822497	5.519584619	5.0628816110.968498199	5.579881751
4.171296804	1.973526784	3.831918669	-0.463030554	-4.062492065
2.890061608	-0.467494769	-3.490721077	-3.778259251	1.497840244
2.9114638244.268372917	5.038388619	3.102282248	6.101544351
0.068057221	3.194848373	4.273256839	3.749739414	2.680497008
TCGA-50-8459-01A	-0.292800336	4.586001962	2.472781371	5.346851065
3.608777224	8.375251482	3.706025563	4.336057436	1.666931929
3.613854885	5.415032011-2.667037522	3.567662249	3.96325243	4.701103999
4.886459398	3.508053913	3.920359268	0.340092639	0.465338615
2.685336588	5.404259993	-1.111180504	2.931306095	4.465518046
2.874179835	5.738807873	1.62791601	4.643160582	3.018202175
4.935400593	1.002605046	-3.613364003	3.733201489	-1.317287358
2.23782031	-2.908356048	5.576693789	3.452348988	0.242153358
4.732097191	-1.128502999	5.001860197	2.337606725	4.083956426

4.276476008 6.936108685 1.447163108 2.957589247 6.836531325 -
4.721180546-1.711896637 4.4433657 -2.680602815 3.267416587 -4.57918444
5.062780879 2.181869009 2.048450147 3.834831938 6.267246719
2.728422301 2.182677279 -1.43355455 4.821301857 3.923009767
3.991093268 4.251555207 -1.728069518 5.106242834 6.537927367
2.84036621 -0.14489935 6.040462831 -0.040776671 5.861912324 5.209161153
4.629478817 5.496969997 6.214371055 11.02100946 1.235793612
2.456108031 0.566834404 -0.327264818 3.720908952 1.011934543
4.278320102 6.856712448 3.556714211 4.394624496 -2.153177888
1.744491795 -0.68243021 3.291531509 3.960648466 -2.347249189
5.319406798 5.038226614 1.457629962 3.246837412 4.14540536
4.952556776 4.767835255 2.552583481 5.335709819 5.106277416
5.032763288 4.168704743 0.89486123 2.256516524 4.423546212
4.897755077 4.785285114 5.48147869 5.15222083 0.899539762 5.535597197
3.931763123 2.348444763 3.795135736 -0.173667654 -4.151129603
3.188438455 -0.520865513 -3.456762454 -2.931245731 1.818238128
2.967500598 4.435379482 4.547696952 2.869871252 5.887920764 -
0.311032417 3.138250209 4.243698161 3.702200978 3.099158613
TCGA-50-8460-01A -0.582264961 4.766019775 2.949465627 5.449614179
4.075096507 8.273472795 3.394121558 4.332958701 1.953957133
3.496984451 5.317873825 -2.258610362 3.758882812 4.152676638
4.773027723 5.049087028 3.276340517 4.163729593 0.301986463
0.58525674 2.786982218 5.186740851 -1.114837993 2.440954538
4.515500248 3.215451567 5.75651546 3.103316311 4.634172068 3.575098669
4.940143388 1.180139118-3.500606313 3.427991837 -1.103491145
2.937587575 -2.81390288 5.586211923 3.268662576 0.844743819 4.725671095
-1.084476309 5.260134425 2.552218856 4.066869103 4.222179242
6.901192418 1.481255275 3.71400859 6.854128895 -4.668662182 -1.671827967
4.514462285 -2.475171777 3.414239402 -4.436069653 5.066621366
2.274391759 2.521169618 3.882167084 6.157193524 2.789351456
2.143229514 -1.545505867 4.650434059 3.929675877 4.27391141
4.227829834 -1.482608563 5.098238385 6.496004626 2.913753491 -
0.076122914 6.003809131 -0.29380274 6.003848475 5.149968758 4.663644706

5.513239327 6.248200752 10.48615469 1.148012748 2.608854481
0.860958903 -0.45264512 3.260093179 0.914515719 4.3729243116.930129087
3.659474244 4.632317674 -1.970662164 2.168460152 -0.094192118
3.313965107 3.786734093 -2.482064802 5.345374899 4.782592545
0.721073306 2.960996222 4.14187315 5.019052136 4.762298751
3.193270332 5.335680527 5.166869879 4.873581271 4.414899066
0.701385614 2.669155549 4.562544822 4.808985801 4.753716312
5.645505151 5.1254465111.214120487 5.59737654 4.072984154 2.14972279
3.906761943 -0.610819417 -3.746458069 3.18360505 -0.162521877 -
3.456381227 -3.319151398 1.84550477 2.930261783 4.577795967 5.029095605
3.103781766 6.038940433 0.058713588 3.044244242 4.522413189
3.722644883 2.894729621
TCGA-53-7624-01A -0.896993262 4.727459244 1.888186792 5.116665888
4.206880002 8.188130516 3.488574647 4.095665998 1.665018126
3.5695241185.151973177 -2.539026719 2.488230062 5.316877738
4.715047509 4.567122442 4.828862647 3.687846338 -0.513365543
0.290340376 2.774041214 4.793505317 -1.430154839 2.616135728
4.569455565 2.510430166 5.312770653 1.273778249 4.513056132
3.962979551 5.024060728 1.196312341 -3.861191655 1.940244242 -
1.1128701822.036837735 -4.137947098 5.603838236 3.480967142 0.488501488
4.679488416 -1.253367934 5.012641585 3.475651983 3.641287895
4.140329034 7.036148923 0.887997217 3.963405747 6.928935238 -
5.855471298 -2.041551956 5.31980566 -3.625799093 3.769078213 -5.225768944
5.0836454 1.557456386 2.635033222 3.899334439 5.881861685
2.356566108 1.919478198 -2.241069934 4.5215143113.932897132
3.581878894 4.154549137 -1.713869275 5.059282588 6.26345948
2.699136553 0.249347764 4.934939355 -0.901543385 6.174371541
4.903705733 4.930772747 5.375926929 6.339288128 10.56720547
0.928834217 2.931427992 0.343255773 -0.509024363 3.058710287
0.973330677 4.271030271 6.82933483 3.225916151 4.943225212 -
2.247915396 2.329326133 -0.659603473 3.274334496 3.976090759 -
2.088345965 5.345595023 3.880486678 1.05188958 2.993613263 3.912329102
4.876754786 4.636127359 3.3711491175.335045304 4.471229788

5.071228074	3.788341236	0.785139892	3.455462996	4.518318506
4.810990538	4.736758979	5.768653388	4.99200553	0.666539672
5.407628674	4.530741249	1.6061100873.823622548	-1.557982615	-
4.507818976	2.48455512	-0.700793548	-4.306347823	-3.968320465	1.288258391
2.356224543	4.212537849	5.483646245	3.274000528	6.256349636	-
0.554508779	2.679849947	4.542007802	3.619466747	2.884626827
TCGA-53-7626-01A -0.665485988	4.487854248	1.995346589	5.376632422
4.399459881	8.31763836	3.396309038	4.285813402	1.697721281
3.453163902	5.322379444	-2.394412468	3.293016663	4.437760054
4.639078946	5.064578317	3.694132784	3.668778951	0.536038294
0.632275712	2.604540244	5.183428665	-1.246082371	2.569186308
4.559351914	3.107181013	5.740150132	0.8069508114.506287293
3.213644622	4.8815547	0.901472622	-3.67636977 3.481570178	-0.986183271
2.692179602	-3.295918745	5.578019102	3.794591379	1.198983782
4.702901649	-1.188504031	5.27492364	3.063843127	3.991575013
4.314997244	6.844794204	1.552297451	3.403218073	6.829585355	-
4.754534595	-1.817483712	4.603384642	-2.642536856	3.289940415	-
4.88783261	5.010607946	1.681507216	2.301664389	4.07524751	6.152510097
2.851320365	1.970611061-2.473345206	4.688142388	3.930348498
3.722227582	4.317076962	-1.391003254	5.088004212	6.294483422
2.844921305	-0.055888152	6.026294645	-0.291107582	5.963957715
5.183657576	4.472235778	5.450532889	6.374251897	10.71042564
0.922232899	2.587837351	0.546449536	-0.834388335	3.725740284
0.904176309	4.264492894	6.843192348	3.61574511	4.704019709	-
1.762295261	1.99751032	-0.412586476	3.259228387	3.70782635	-2.276935254
5.347953947	4.912480338	1.294993348	3.230376303	4.112655831
4.8988671184.723923063	2.993317199	5.335369544	4.978256061	4.95114358
3.936534682	0.650629176	3.269303675	4.561472759	4.72728424
4.771213032	5.665899156	5.226667055	0.805308759	5.73574977
4.180864366	2.0982451123.803341172-0.045218627	-4.230114094	2.97721036	-
0.36085655	-3.401971715	-3.881596404	1.672100564	2.714528675	4.49479346
4.965450961	3.227181775	5.961365053	0.301533802	3.097490586
4.339729246	3.637666161	2.857572679

TCGA-53-7813-01A -1.569889446 4.382130282 1.534368681 5.383214476
4.360908003 8.3839411783.70444696 4.198481951 2.067809875 3.858325179
5.287697537 -1.96891213 3.033473302 5.213007988 4.655301591
4.705416149 3.989941384 3.882132508 0.826622784 0.331330571
2.646429093 5.740093488 -1.908355687 2.636078971 4.682274187
2.374959856 5.677546365 2.859853347 4.394858684 4.070516391
4.532540305 1.168150215 -4.07166933 3.397752213 -0.686225771
2.482584505 -3.876254977 5.604237277 3.707798936 0.371575414
4.687203054 -1.4315337 5.462729895 3.453384107 3.733282805
4.308948668 7.150704358 1.223608347 3.867042166 6.515206648 -
4.983092519 -1.47304858 5.026756212 -2.306419857 3.705859422 -4.074274189
4.849451256 1.84053506 2.669096169 4.188026244 5.856376617
2.724651252 2.249728443 -1.687392031 4.35546364 3.943972449
4.249633289 4.130696593 -2.113566392 5.067593331 6.320720608
2.978034662 0.148571338 5.743107977 -1.076942253 5.940319314
5.199418273 4.677052796 5.340834642 6.626402819 10.93507447
0.477808406 2.843914245 1.220914302 -0.961849437 2.676009081
0.564758437 4.22425354 6.789246132 3.61212127 4.965434825 -2.197700533
2.241331091 0.255290796 3.32987941 3.730006564 -2.75887749 5.331875062
4.721435147 1.060758393 3.335613853 3.683809899 4.853811083
4.833803862 3.54705499 5.335010309 4.830512619 4.858691461
4.589839245 -0.016041645 3.3411411244.313168141 4.3992371114.772864371
5.65539444 4.912085393 0.912905957 5.4761185223.850517121 1.610866374
3.845786225 -0.674664953 -3.44387343 3.675160027 -0.181059776 -
3.471320028 -3.379325993 1.328776601 2.100083944 3.717535742
5.527836083 3.79252496 5.912909816 -0.863124732 2.142600885
4.557506355 3.6580223112.662357537
TCGA-53-A4EZ-01A -0.601089682 5.032884978 2.68136416 5.137999325
4.3008110798.225900186 4.040221833 4.145255512 2.214473757
3.255614717 5.238476494 -1.931431681 3.435792132 5.010553374
5.18890221 5.1181904434.2605746 4.551835673 0.319919881 0.510557091
3.025780181 5.176785625 -1.181756055 2.670179138 4.738566597
2.713382784 5.369356589 3.084816588 4.6941101394.442952725

4.902825229 1.343519194 -3.218347952 2.987762006 -0.974321411
2.626681398 -3.678742933 5.6098411233.227930747 1.139751345
4.685533534 -1.125379585 5.1190504533.745315242 3.756317378
4.320656863 6.977858069 1.475164138 4.616198946 7.106033265 -
4.819557516 -1.452645771 4.598002759 -2.079588806 3.692496571 -
4.512020418 5.331724874 2.35004802 2.316700932 3.974897265 5.789417839
2.868391482 2.003291516 -1.41959262 4.602406077 3.935089685
4.356367104 4.308175329 -1.384391852 5.068418264 6.58592421
3.003178962 -0.031488101 5.375582246 -0.521334885 6.239831041
4.935255686 5.1152478465.455102404 6.391405888 9.631926096
0.992791898 3.369066793 1.256110594-0.949698629 3.1115738710.64776868
4.3205996 6.952094584 3.607992004 5.028854852 -1.839146296
2.878879912 -0.141332275 3.366431452 3.988770779 -2.014915099
5.348274157 3.966180995 1.363357879 2.926168809 3.703901534
5.02350088 4.624142368 3.949454414 5.335477026 4.8657311014.933929179
4.720666531 0.745282986 4.664297662 4.399121844 4.712471818
4.7112852135.846436343 5.245577882 1.520529159 5.290322899
3.899856794 1.701928912 3.949373315 -1.346290174 -3.331239488
2.899594686 -0.290910295 -3.711050043 -3.583360098 1.529864142
2.664579304 4.057481677 5.896221661 3.70383087 6.591869674 -
0.660752836 2.263959987 4.764057295 3.983039615 2.39172032
TCGA-55-1592-01A -0.851727877 4.77299899 2.355578022 5.268926293
4.658534186 8.210478153 3.335564164 4.139620197 1.963474486
3.454590235 4.960538995 -2.206808989 3.3912113334.97954131 4.8804818
5.028703974 4.198662132 3.76296187 0.151637473 0.50009443 2.872926854
5.19661443 -1.311303453 2.444525871 4.602587328 3.15982957 5.461114361
1.976864749 4.548096306 3.754636666 4.895060433 1.181927133 -
3.725402557 2.977910742 -1.057460702 2.901943867 -3.555423933
5.586938329 3.512936364 1.57122723 4.702612328 -1.242305324
5.149612559 3.510072827 3.8680711574.243435866 7.097504077
1.602731759 4.001538014 6.829303465 -4.839687224 -1.867070584
4.554439598 -2.755647532 3.786522032 -4.579947465 5.141977077
1.692683965 2.589841218 4.044433686 5.999171987 2.720179843

1.906585668 -2.628280763 4.5814443 3.937780546 4.10084436 4.270838264
-1.531309161 5.07780598 6.304620406 2.956770968 0.017572603
5.569757977 -0.707973883 6.052002677 5.139789833 4.672394828
5.429423515 6.355975575 10.47985814 0.645982042 2.770789734
0.66015457 -0.953355469 3.457698078 0.822013497 4.234988393
6.873348554 3.713782266 4.922269862 -1.922470074 2.225998682
0.217916394 3.301362305 3.709338263 -2.135912568 5.350824719
4.550241208 1.563120423 3.065589904 4.190721535 4.953831962
4.641838965 3.367108314 5.335243988 4.977774169 4.894944359
4.26148177 0.565765472 4.186530042 4.65492121 4.715958174 4.761527473
5.774364338 5.384259067 0.969487436 5.646320574 4.381364375
1.931064458 3.880109041 -1.101620872 -3.217812348 3.021672639 -
0.151437142 -3.596793718 -3.961951324 1.452086058 2.73802982 4.408857817
5.418861844 3.572021703 6.337752224 0.376271241 2.9125862
4.619083293 3.740895783 3.228117256
TCGA-55-1594-01A -1.370728634 4.443057205 1.7011502785.319408892
4.358758823 8.260020535 4.220452453 4.108159303 2.223848209
3.912898263 5.574213566 -1.990189568 3.552560883 5.334319984
4.900249018 5.073582179 4.7035011924.565855294 0.173743227
0.334476768 3.032767465 5.199737013 -1.744749616 3.069776191
4.702535729 2.302737527 5.721901322 2.969673066 4.599890391
4.665012348 4.705269861 1.604444539 -3.500516818 3.288681658 -
0.780779582 2.56263973 -3.977640236 5.590389664 3.517712034 0.473016651
4.689815257 -1.125128456 5.521492547 3.97720611 3.758350072
4.232061392 7.122289687 1.581453374 4.624267859 6.936266128 -
5.259923443 -1.515667856 4.698057642 -2.357759463 3.82885064 -4.38256159
5.127416032 2.39441109 2.6191168784.142306331 5.977263849 3.118780087
2.704197164 -2.220543104 4.515068138 3.947954775 4.454306924
4.433162824 -2.502599718 5.063430474 6.363519261 2.931881509
0.278213532 5.730446604 -0.813310866 6.093220829 5.103857354
4.910576759 5.499640018 6.463554728 10.18448473 0.343418749
3.069155458 1.498793136 -0.900495506 3.668654648 0.592783872
4.3221105176.824692604 3.740057437 5.198739925 -1.906730217

2.811864291 0.233651752 3.396586033 3.816730192 -3.058021492
5.373863364 4.397659672 1.670403671 3.302278236 4.285463573
4.988498188 4.655150675 3.722172812 5.335344045 5.081784419
4.919654903 4.310015903 0.636026325 4.442182153 4.40752509
4.608244132 4.711325598 5.817318568 5.386836567 1.364655742
5.479414922 3.283992456 1.392791575 3.952260262 -0.972501221 -3.203526225 3.112580213 -0.066812246 -3.846172341 -3.498895545 1.292514094
2.281235694 4.022179763 5.747874224 3.963531603 6.432748376 -0.034505124 2.295328021 4.882916223 3.685469748 2.802382692
TCGA-55-1596-01A -0.804047648 4.841910847 2.684435971 5.546234818
4.380136476 8.167620996 3.213312731 4.175098443 1.871416387
3.454258029 5.648354112 -2.216887632 3.902887769 4.69391889 4.788088798
5.261571432 4.048006875 3.89101808 -0.498739591 0.366571491 2.92983709
4.364335296 -1.187026755 2.462963829 4.566308889 2.809134934
5.436935508 3.656926529 4.522157023 4.395009194 4.771345474
1.210376093 -3.655084631 2.993251335 -1.219635006 2.276267908 -3.28117485 5.61920695 2.637872918 -0.619388717 4.700843156 -1.176910727
5.30540745 2.965137021 3.904514861 4.258068137 7.260740654
1.100584181 4.286721864 6.897692156 -5.36409682 -2.184582245
5.021321011 -2.7633808 3.52556031 -4.971610816 5.061608878 1.914334641
2.448917543 3.817805392 6.01500099 2.765889473 2.798239676 -2.324693007 4.411311925 3.93860347 4.342834501 4.395175883 -1.051804656
5.077655666 6.183018207 2.893442178 -0.060321907 5.557054611 -0.951733911 6.2159601 5.055844214 4.820856873 5.612084341 6.379093153
10.33923642 1.162289337 2.714098325 0.725548494 -0.879457789
3.067081024 1.028224744 4.386432579 6.823142783 3.874706105
5.22020488 -2.215322867 2.74239809 -0.018604528 3.335266336 3.696670342
-2.183472854 5.368340682 3.534873812 0.923489104 2.774545453
4.36910207 5.052192144 4.643720126 3.3742659 5.335369271 4.884901248
4.854088753 4.354934143 0.755605322 3.456940525 4.814798284
4.724631269 4.708689174 6.08540857 5.296023805 1.048255033
5.425239791 3.843619293 1.650685228 3.96467929 -1.914917582 -3.655658543 2.605664535 -0.209130091 -3.952047477 -3.499276931

1.467884412 2.703647181 4.739224916 5.844455924 3.688012569
6.30407395 -1.370819897 2.543958197 4.752719289 3.697272663
2.882227349
TCGA-55-5899-01A -0.672198364 4.812066585 1.148031362 5.189174734
4.42799695 8.298672894 4.376501285 4.057715154 2.782956576
4.299403594 5.478934548 -1.57962326 3.784670947 5.021155241 5.106125194
5.100919563 4.227622196 5.479594147 0.548462138 0.344088131
3.138451585 5.489174378 -1.586757051 3.444354845 4.758975933
2.305810099 5.413811521 4.507534417 4.741757806 4.952920031
4.896891679 1.732094246 -3.403582022 3.635428966 -0.741007448
2.123090137 -3.313469755 5.610778071 3.294251534 0.46213778
4.705640638 -1.044843235 5.372626048 3.696332597 3.808010398
4.373168527 7.209442356 1.781787966 4.699343873 7.021457649 -
4.745861265 -1.086964332 4.521552896 -1.867243725 4.463834651 -
3.902464803 5.271553427 2.79837932 2.568733752 4.132554895 5.992095152
3.207943467 2.719109116 -0.790709547 4.595298059 3.940971257 4.78695424
4.208320788 -2.883606482 5.062199872 6.785115313 2.994543046
0.087850255 5.451749317 -0.397513164 6.069093977 5.038638755
5.127776207 5.420263621 6.545176094 10.08551927 0.956538184
3.147139173 1.963187464 -0.735374354 3.39866057 0.543082329
4.233888146 6.913212391 3.770165334 4.974499241 -1.995055229
2.773821277 0.65323926 3.50052609 4.379215336 -2.476530745 5.333703818
4.443213221 1.956075728 3.251847732 3.44747584 5.104810798
4.625715116 3.85929427 5.335643321 4.980244942 5.017379247 4.764270696
0.771797688 4.889886947 4.448989437 4.656842377 4.718554867
5.947734323 5.109413239 1.65814493 4.798662531 3.565660367
1.558728301 3.986243534 -1.611388757 -3.14975689 3.723640976 -
0.07763445 -3.716185859 -2.352967109 1.370890728 2.568545961 4.007877639
5.919843058 4.20372848 6.640982302 -0.340471586 1.801448848
4.780134057 3.945293577 2.395309021
TCGA-55-6543-01A -0.530151253 4.735229594 2.826813013 5.507131577
4.498368165 8.284633342 3.269640959 4.297287427 1.838615767
3.127075056 5.371395204 -2.502921258 3.557259288 4.479675456

4.769765055	5.251579733	3.2511866933.912389159	0.631951363
0.564770648	2.601083774	5.166917907	-1.22030453 2.205731968
4.515662796	3.19817427 5.649555273	3.093767379	4.565653542
3.382168157	5.0111926930.92417106 -3.810619539	3.58762356 -1.039835182
3.049265893	-2.919417253	5.609950108	3.453548492	1.300016676
4.736126023	-1.127822542	5.210156884	2.686000778	4.075358527
4.236701985	7.275744491	1.564202469	3.674250445	6.559687521	-
4.599819486	-1.856898955	4.879528081	-2.520356278	3.118501675-4.528750122
5.1143943362.128909099	2.667432293	3.788138973	6.091387277
2.496257668	1.657993238	-1.874839085	4.644991748	3.946507644
4.1933911474.103545334	-1.641568236	5.0852417 6.368166067	2.922637029
-0.122060223	5.956561051	-0.46209496 6.042470364	5.174099214
4.721418409	5.507905701	6.27097069 10.857476 1.183563999	2.57243141
0.762053971	-0.721599446	2.75161027 1.1112819684.406908574	6.886272351
3.637474038	4.574126366	-2.077813826	2.229000023	-0.109030973
3.262026581	3.717734926	-2.447457662	5.353037512	4.743126672
0.344028518	2.695658876	4.166627379	5.003237505	4.87386522
2.984432786	5.335486069	4.883507484	4.963993238	4.222338673
0.609557284	2.493748183	4.4789611544.814360006	4.778524825
5.6821041115.043717567	0.807080621	5.584941512	4.6155311781.977275549
3.973270019	-0.511109732	-3.823248724	3.030523274	-0.118807976	-
3.253341255	-3.295544199	1.858395499	3.105433795	4.7119620455.204724677
3.352828481	6.169049958	-0.219193803	3.2511301894.682568345
3.650948337	3.333191849
TCGA-55-6642-01A -1.085204371	4.408741886	2.1155183735.416397358
4.075966623	8.3928411613.500560675	4.25596246 1.812209803	3.526801027
5.445557033	-2.334912516	2.927990082	4.644744719	4.659732664
4.840805932	3.8470719113.874899023	0.912505823	0.55113627 2.534030446
5.390864748	-1.742895583	3.031047208	4.582626755	2.786576398
5.537380593	1.897274952	4.386950898	3.234828931	4.806043795
1.015534786	-3.847449037	3.920398686	-0.809062361	2.396823135	-
3.245250295	5.5864011613.88199617 1.094561063	4.697136117-1.347845376
5.26153606 3.390375675	3.940369123	4.29316926 6.902878447	1.60973093

3.400969756 6.673914173 -4.79362869 -1.81270867 4.653295916 -2.509856433
3.310814479 -4.70299667 4.876127725 1.860014128 2.29165885 4.072928626
6.047542642 2.760748311 2.452972286 -2.210961979 4.654514456
3.943290415 3.877958968 4.215995905 -2.649309425 5.081894491
6.396954704 2.843261025 -0.015649527 5.865090946 -0.697924738
5.93033235 5.175327437 4.612545041 5.429080961 6.526513737
10.86721104 0.996788164 2.623581686 0.8309575 -0.840876797 3.468821941
0.860864423 4.214085532 6.828425321 3.579277599 4.660343943 -
1.89087866 1.991015136 -0.339884379 3.274567443 3.874824304 -2.681917949
5.335180498 4.955093587 1.553639837 3.292891891 3.904693035
4.888205794 4.765830424 2.973811348 5.335400156 4.933902551
4.950143014 4.001724842 0.381947179 3.487336552 4.360328847
4.567216091 4.762849051 5.535488781 4.941185444 0.797527272
5.663326013 4.088747418 1.754199973 3.748653995 0.003465394 -
4.220578244 3.097897764 -0.469826252 -3.378582117 -3.694476581
1.473527617 2.607133663 4.174830242 4.939626368 3.170242682
5.921346886 0.206836056 3.069580899 4.247247746 3.62253566
2.842601666
TCGA-55-6712-01A -0.634762467 4.518471476 1.926522815 5.468231522
4.517914147 8.274707903 3.086474194 4.347608277 1.488908833
3.308122199 5.615451797 -2.806464267 3.838226043 4.824629188
4.701555668 5.183148438 3.357547162 3.391968863 0.266299348
0.611349499 2.402310252 4.881616057 -1.316172218 2.401202484 4.45840899
3.065394721 5.60519541 1.474450443 4.441105146 3.26996349 4.94912149
0.979318905 -3.621399456 3.350369827 -0.893828955 2.524289214 -
3.019565621 5.618846286 3.994397913 1.340865118 4.723052878 -1.200680137
5.297765864 2.478828493 4.02988373 4.152616078 7.071599376 1.24304588
3.229715894 6.574023058 -4.450971121 -2.114111083 5.41647606 -
2.470549941 3.096565592 -4.679435285 5.032709667 1.965360336
2.754360778 3.598110888 6.12066677 2.599136216 1.874145211 -2.569964014
4.604387605 3.94305618 3.490648461 4.403587058 -1.552082105
5.088177917 6.077334397 2.767527532 0.041586455 5.766556865 -
0.381982952 6.009437189 5.095064812 4.549161896 5.491232311 6.121278318

11.66644281 1.122109731 2.841968115 0.51684909 -0.691625133 3.416737353
1.167676026 4.338037277 6.819916115 3.471152852 4.920857732 -1.941235687
2.226236245 -0.326089038 3.227619146 3.425786284 -2.657330621
5.342257856 4.283341977 0.752493467 2.990631533 3.889304246
4.947894295 5.011334776 2.852788848 5.335364429 4.483343791
5.029869807 3.906250123 0.637897585 2.103664467 4.454062314
4.815072718 4.781115967 5.615366579 4.63630408 0.652289425 5.675050234
4.584176277 2.157914302 3.849615323 -0.264518479 -4.065079949
2.78673884 0.554043186 -3.189106421 -3.217208585 1.958919946
2.779759479 4.600325352 4.8277932 3.324088438 5.911622847 0.065934738
2.834207463 4.521436933 3.605703194 3.617676695
TCGA-55-6968-01A -0.502904266 5.016186713 -0.040000764 4.980826929
4.320765887 8.207271917 4.91477836 4.04072158 3.121528449 4.890077455
4.886388519 -1.165709738 3.214117635 5.850571702 5.442482406 3.96945374
4.772115556 5.18178545 0.815191332 0.198082035 3.182730499 6.478233662
-1.438715102 3.076633318 4.872761899 1.699339328 5.748052053
4.591465794 4.834611547 5.214241749 4.775750111 2.006478178 -3.255190683
3.291896904 -0.275128392 2.103955444 -4.694022323 5.563261968
3.57545719 0.518914365 4.677501341 -0.969198848 5.510758433
4.070491095 3.566693354 4.480083189 6.958579198 1.878103328
5.377657259 7.811044671 -5.262276349 -0.635711437 4.505781004 -
1.896415776 5.360012051 -3.444320935 5.409051283 2.758586692 4.02541181
4.552255986 5.794522557 2.954006066 2.035350268 -0.65141504
4.565441261 3.934532986 4.930973735 4.640537433 -1.751253342
5.047772529 6.749731562 2.880526638 0.768370458 5.553996042
0.06107043 6.007269275 4.951743206 5.40943176 5.223430043 6.853979539
9.956862451 -0.568855497 3.608879579 2.656154077 -0.332816466
3.839112563 0.351680046 3.937656134 6.894989456 3.151280481
5.272693441 -1.891778291 3.100071778 1.333558737 3.490780832
4.432700601 -1.938011175 5.319942469 5.154129075 2.638432859
4.095658162 2.875054714 5.039085362 4.581180648 4.384859761
5.335512547 4.274689773 5.340093003 4.747696882 0.766030491
4.902826745 4.520961911 4.677466544 4.762825923 5.642185692

4.243296672 1.4735126 4.77510838 3.82343273 1.640814668 4.103726261 -
0.991880558 -2.047197095 4.859635885 -0.290481805 -4.079971281 -
2.797044914 0.679808261 2.153721208 3.143134174 6.422092792
3.881010934 6.680032751 0.042710918 1.579832647 4.933100836
3.943629155 2.049017292

TCGA-55-6970-01A -1.411139701 4.5561195563.22260182 5.544196779 4.154934208
8.357832909 2.91242975 4.341088678 1.59920112 3.170415072 5.575027066
-2.324829277 3.1063119994.537007928 4.438373724 4.933210087
3.760230876 3.572421227 0.762141726 0.466755203 2.416327395
4.982708295 -1.85253438 2.485322823 4.556504348 3.038453475
5.863520503 1.5882261174.317747494 3.0991102924.699914364 0.758225968
-4.1772452 3.41943911 -1.26783484 2.657775309 -3.254452082 5.593344506
2.8993052 0.877516455 4.685178168 -1.442829277 5.076928867
3.2528101133.923898619 4.301867947 6.70725684 1.207688423 3.144386603
6.396537441 -5.116230568 -2.025920054 4.819220963 -2.558302357
3.362206673 -5.220859634 4.822073987 1.347353437 2.018856029
3.889036727 5.964957735 2.514230516 2.329017607 -2.764273351
4.530874147 3.940197771 3.776272619 4.077886122 -2.913886771
5.078253964 6.2573277112.903566934 -0.287448849 5.947977181 -
1.298827969 6.086807691 5.140320424 4.375850285 5.468808319
6.389515196 10.44473774 1.239935132 2.361342528 0.40072375 -
0.879846441 3.001369213 0.912228627 4.286530915 6.823256283
3.617194494 4.760114537-2.122557008 1.873780116-0.429138563 3.249558047
3.844369478 -2.884696717 5.348041394 4.598034195 0.619017338
2.929320316 4.1181646394.863749936 4.671233197 2.943983061
5.335166702 5.1100058314.767773332 3.964724059 0.098146955
2.984392286 4.626353121 4.55030561 4.74103119 5.670509323 5.164658637
0.7631209 5.730206551 4.360938431 1.448433155 3.670106109 -
0.302048582 -4.470193841 2.324335224 -0.556127545 -3.558974872 -
4.243202867 1.607831794 2.5301111764.383552437 4.8682547113.144784815
5.844659025 -0.663090987 2.969795502 4.277333595 3.537031451
2.749184839

TCGA-55-6971-01A -0.638560836 4.756985797 2.38977201 5.278694694

4.036355705	8.356024722	3.715514528	4.30308748	2.004845887
3.673867562	5.292242149	-2.389886657	3.292566957	4.611575926
5.018651465	4.782230997	3.54669811	3.997032747	0.850236195
0.575779166	2.597383645	5.535248976	-1.397083132	2.801807464
4.62740887	2.816443279	5.984853562	1.613006623	4.561604709	3.50641454
4.773943328	0.860146369	-3.638672313	3.635256985	-0.815268676
2.568982524	-3.220208976	5.5721173063.347613686	1.2091117484.709899325
-1.134513619	5.382089816	3.014121648	4.017690459	4.336037278
6.940176644	1.6115065733.629318987	6.749564475	-4.756208506	-
1.751513366	4.482436632	-2.382040761	3.498629678	-4.759332714	5.12340093
1.835460304	2.099937531	4.094851384	6.049761708	2.797552698
2.110530307-1.959079541	4.751688939	3.935709508	3.973998988
4.256954321	-1.989624526	5.096132633	6.428066764	2.923965262	-
0.057227187	6.034203617	-0.324432201	5.93123782	5.150432091	4.665106249
5.429196162	6.434453602	10.35418189	0.914808739	2.732306363
0.839566519	-0.663662825	3.681461257	0.938257106	4.245940874
6.871536609	3.472235631	4.713299893	-1.769591663	2.193390419	-
0.451595858	3.254447647	3.972605355	-2.449829223	5.334099941
5.140492624	1.177633215	3.361780355	4.0013031174.955594775
4.6717451143.050633931	5.335559908	5.159049578	4.998977749	4.03971431
0.582460764	2.932820372	4.545216554	4.728057547	4.798177064
5.53438063	4.933759498	0.968170791	5.690008916	4.276150887
2.010070504	3.87941958	-0.111367218	-3.749900121	3.253874812	-
0.51745645	-3.430140779	-3.820981788	1.452899083	2.801041066	4.224097232
5.186899565	3.325470623	6.063497428	0.291600677	2.885607372
4.497717866	3.668124607	3.061465171
TCGA-55-6972-01A	-1.450751633	4.671221826	3.147705989	5.556746991
4.381218331	8.2188388113.387844628	4.203838845	2.028935559
3.073716585	5.226274131	-2.081401057	3.409474657	4.759699212
4.659235324	4.824417628	3.860594179	4.363986843	-0.18902017
0.177062476	2.767639303	4.962542281	-1.800618206	1.834735768
4.589238554	2.840841204	5.465465787	3.274192917	4.342841044
3.56705535	4.7858114261.035750962	-4.242568475	3.112097158-1.42085306

2.580819683 -3.712617605 5.604181637 2.792462854 0.036015644
4.694321895 -1.269610222 4.816319231 3.352752323 3.819036189
4.134973328 7.03876768 1.220986559 4.250313632 6.629190488 -
5.492084381 -1.745275742 4.651669211-2.62184828 3.451506211-4.868829918
5.050713857 1.712984248 2.615067565 3.815503663 5.776108633
2.537697268 2.204056494 -2.577462762 4.302893018 3.937249908
4.434345489 4.05821887 -2.392348267 5.061300939 6.338429505
3.003514068 -0.038634117 5.732217916 -1.548330844 6.213009674
5.073412241 4.782515182 5.50778416 6.43790401 10.05754528 0.674403399
2.392294224 0.916297384 -1.147614059 2.37931319 0.525786824 4.42616927
6.925080754 3.710335275 4.933363259 -2.336069839 2.222774118
0.096775272 3.332922423 3.894554824 -2.755242976 5.363531083
4.069381539 0.186466553 2.545700838 3.948403617 5.007933196
4.669459319 3.647162094 5.3350771134.8721161384.686817231 4.641725049
0.264812378 3.238019623 4.609104545 4.59901925 4.692974602
5.836894486 5.121097826 1.201974215 5.420103764 4.082449699
1.402367918 3.933751372 -1.491658805 -4.153198264 2.515366106 -
0.399793768 -3.860147435 -4.162504918 1.316171401 2.288104834
4.142045459 5.616546955 3.489883545 6.218423267 -2.004564691
2.432824696 4.721766952 3.56211628 2.151094247
TCGA-55-6975-01A -1.279022836 4.242876369 2.221508741 5.552623488
3.875399793 8.334672574 3.204013842 4.240103659 1.534571755
3.609699535 5.664551756 -2.577200428 3.465581082 4.551378011
4.302386952 4.992157707 3.889199104 3.610666678 0.073714954
0.347150909 2.619192002 4.669158403 -1.728784601 3.007344993
4.535260399 2.684597267 5.535901601 1.774428952 4.398413307
3.240413228 4.880623821 1.113454326-4.08059418 3.325766851 -1.31380016
2.06359401 -3.527531521 5.601329531 2.767673337 -0.077793075
4.695077322 -1.375458782 5.179241407 2.824666834 3.807514453
4.216357499 6.85229905 0.932777734 2.968324203 6.780384039 -
5.569363022 -2.035370975 4.977789161 -3.036215946 3.316086921 -
5.047320757 4.906626759 1.54665038 2.185148842 3.6216626116.04089808
2.528563665 2.666169091 -2.469385701 4.556121379 3.932533887

3.849099735 4.210029853 -2.681914812 5.077426274 6.285710349
2.714782559 -0.036444574 5.53671843 -1.204683945 6.048812488 5.04224619
4.653177978 5.48693936 6.314415956 10.9748138 1.378732856 2.366715261
0.442820791 -0.52809519 3.172496862 1.07981586 4.300711661 6.804614221
3.508610531 4.86367862 -2.305572165 1.853194813 -0.701045285
3.308261914 3.921212362 -2.806316996 5.335478033 4.15641758
0.755092287 2.885243933 3.891016904 4.907079295 4.615134883
2.874955288 5.335255807 4.8575594 5.044291921 3.96835607 0.55081518
2.742808824 4.58622022 4.740947521 4.734232701 5.732079775
5.087216643 0.748379504 5.518287473 3.967681316 1.539167977
3.690989089 -0.854456063 -4.63326778 2.623427787 -0.556757035 -
3.937434001 -3.703465998 1.522259771 2.32480935 4.493590149 4.749595985
2.986046894 5.886493463 -0.869124821 2.561948793 4.355392864
3.462172532 2.614810299
TCGA-55-6978-01A -0.82632229 4.396507593 1.859829069 5.451584626
3.908263348 8.322911272 3.435154223 4.378643973 1.350937219
3.767127288 5.457121533 -2.687167338 3.462142636 4.689679072
4.685737387 4.714953389 3.544403018 3.364288526 0.10873058
0.332498258 2.379065609 5.150584045 -1.554846631 2.976215458
4.554284003 2.365809214 5.856035504 -0.087361045 4.472471104
3.664776069 4.799894728 1.07059337 -3.68881912 3.505371848 -0.68704722
1.648000084 -4.061625285 5.595506495 3.390943227 0.15480435
4.703138815 -1.262376433 5.078897276 2.551396733 3.807100656
4.231566931 6.699037252 0.975133826 2.942676422 6.832239145 -
5.406156404 -2.093130158 5.090471288 -2.627393571 3.277525755 -
4.94982652 4.93672283 1.624834013 2.403081266 3.6687653 6.042113644 2.43333237
2.545591348 -2.795706764 4.748666946 3.935114607 3.382893588
4.474232414 -2.078412467 5.090379848 6.289542815 2.602355929
0.034212626 5.836934437 -0.536840163 5.971372251 4.993432751
4.606255771 5.449758499 6.368310583 11.03269312 0.556762174
2.831463819 0.549419827 -0.585604462 3.579319636 1.119277197
4.246952287 6.786166915 3.124771012 4.908722391 -2.177677686
2.160599011 -0.707866258 3.270489866 3.863833078 -2.70154512 5.325416601

4.628567716 1.05300788 3.249531368 3.638826969 4.923558878
4.835605336 2.618569156 5.335492922 4.460293831 5.145196107
3.734858234 0.643842447 1.863764009 4.378376902 4.832301854
4.791069958 5.375071059 4.623988824 0.71300507 5.397363565
4.1166607081.942999432 3.704508689 -0.316123283 -4.370209829
2.878544119-0.41541734 -3.841417179 -3.583504666 1.392667216 2.430400679
4.218994868 4.655028146 2.948326306 5.808068912 -0.561935382
2.490577653 4.125095422 3.458946971 2.846847139
TCGA-55-6979-01A -0.167084529 4.730483158 2.182086384 5.375911225
4.284334397 8.321964453 3.433314628 4.344061636 1.777589417
3.581434829 5.430361606 -2.446006267 3.931457028 4.740252266
4.944167201 5.062987855 3.795604914 3.7441150550.76211315 0.58598739
2.60779663 5.371783644 -1.114491071 2.414655804 4.594388979
2.828909618 5.768749712 1.259686273 4.63065272 3.509716848
4.896767591 1.078113247-3.600293826 3.550218159 -0.839951549
2.566280657 -3.177025848 5.594755946 3.24753408 1.163083852
4.713595602 -1.111680371 5.238629731 2.816089535 3.992772676
4.322771019 6.917056435 1.503542078 3.567156576 7.034930667 -
4.793756579 -1.903109258 4.809710924 -2.377158739 3.61622254 -4.761990147
5.147019829 2.007566626 2.652462886 3.9876452 6.1350818 2.503369196
1.913344186 -2.113675307 4.725842436 3.935727434 3.853757274
4.3976752 -1.355307835 5.085074624 6.385382764 2.8011799650.007982045
5.883814862 -0.141273032 6.015962077 5.061990015 4.651691364
5.514162717 6.292621519 10.66860271 0.789825416 2.840062529
0.661830682 -0.599054116 3.530467073 1.082441463 4.294571979
6.8576641113.423616209 4.902946898 -1.853122252 2.311332442-0.319006553
3.276546284 3.831765447 -2.168150567 5.333772675 4.788990654
1.231595097 3.399130995 3.915418185 4.954031009 4.755859105
3.005669589 5.335438252 4.8411078435.1062110394.075239492 0.877781847
2.697990356 4.55518252 4.842039933 4.7838603115.558244866 5.104356
0.821473436 5.573220384 4.381791762 2.099505603 3.860915236 -
0.124632637 -3.898428711 3.102689652 -0.261261508 -3.488220451 -
3.249676426 1.712034999 2.821216803 4.523029845 5.120038449

3.171282379	6.123835123	-0.188708083	2.980663432	4.519014483
3.6581191713.530650231
TCGA-55-6980-01A -0.119144155	4.56082367 2.78613923 5.432192896	4.162959627
8.252018315	3.24319531 4.326635748	1.578095481	3.294731756
5.260172883	-2.832047194	3.651005321	4.345507542	4.831838585
5.263766644	3.529976704	3.675156581	0.609998942	0.623036493
2.718372547	5.052349468	-1.103343326	2.7115858	4.441827331
3.1167741635.550828278	2.307271784	4.538901824	3.004403326	4.96341823
1.101621404	-3.694109701	3.655326316	-1.252180846	2.969721936	-
2.440198598	5.599972573	3.42968205 1.384577809	4.726168488	-1.126671327
5.175194435	2.757876937	4.1198302974.214135587	7.041339718
1.645165201	3.440895453	6.520524633	-4.583719756	-1.89483445
4.703847446	-2.507103911	2.929394914	-4.684555882	5.10062911
1.916826457	2.243269729	3.761790637	6.196604137	2.613108686
2.023357877	-2.277122709	4.718020889	3.93235304 3.937001052
4.372001712	-1.492845796	5.0909110956.294274752	2.87425382 -0.156113096
5.843938472	-0.320055599	5.950401573	5.18499521 4.627164019
5.523746645	6.262280031	11.200612521.250707951	2.516509634
0.614820793	-0.532826264	3.628663132	1.070595628	4.308852772
6.882665862	3.632390982	4.742610991	-1.895462636	1.993475901	-
0.553706999	3.292135389	3.693256502	-2.177044656	5.340917344
4.418402413	1.162941909	3.129716783	4.128640977	4.97905485
4.837805998	2.847150914	5.335454446	4.992183004	4.961257186
4.1155902490.820802363	2.613879725	4.542144214	4.86750512 4.771073784
5.557997526	5.125176084	0.706763991	5.744885067	4.186234492
2.281383651	3.856672239	0.097908318	-4.108620932	2.787351865	-
0.041950044	-3.242069575	-3.431255945	1.989355096	2.920274081
4.641867572	5.00943417 3.01147748 6.13710624 0.156857593	3.326568488
4.512503062	3.693918603	3.659240094
TCGA-55-6981-01A -0.307241444	4.886907587	2.101332405	5.486952231
4.629922323	8.244379382	3.175814835	4.246430183	1.90531916
3.486779528	5.578313226	-2.454002034	3.962899265	4.69855027
4.869198007	5.451723099	3.587129534	3.8110066790.555627679

0.639887843 2.825514132 4.981634236 -0.878162841 2.124594944
4.553694498 3.186659879 5.550228886 3.182568042 4.707285823
4.1143909315.101317935 1.379751093 -3.510669145 3.590322194 -
0.900042442 2.999380533 -2.590736391 5.619412771 3.857600102
1.344495398 4.727527037 -1.108955788 5.333497048 2.861210539
4.055999954 4.256884968 7.247779097 1.669207819 3.760728594
6.933490648 -4.445707569 -1.840790625 5.252213486 -2.340759723
3.167472881 -4.143958775 5.247432074 2.272028498 2.457231817
3.724981615 6.1121850472.696303518 2.12408888 -1.626900642 4.631642569
3.940907157 4.356277996 4.351179288-0.268677636 5.087828181
6.3011822642.844535063 -0.038221711 5.656515245 -0.421809294
6.090428346 5.108184809 4.903398334 5.504999679 6.305359601
11.255707181.129922933 2.793299286 0.733247445 -0.587980079
3.174820878 1.153489005 4.384134734 6.877078013 3.709047728
5.026878065 -1.853662636 2.650913835 -0.17797761 3.332436509
3.581691085 -1.89691385 5.347463992 4.168360516 0.943484105
2.898529601 3.92846509 5.072448487 4.8551684113.246478999 5.335601089
4.789543185 5.020425058 4.322617148 0.8119984442.953914375
4.614063343 4.864279396 4.753002841 5.667535645 5.044436194
0.904142812 5.673539219 4.087934141 2.175107712 4.011465663-
0.464460221 -3.398807092 3.139740816 0.304086275 -3.269817254 -
3.1133939021.79067786 3.002235248 4.643183869 5.361287349 3.597959545
6.280767692 0.554093557 3.096563203 4.657403134 3.667064443
3.366569786
TCGA-55-6982-01A -0.506794588 4.385398695 2.1426229 5.394636817
4.458185189 8.34794744 3.186892508 4.326102818 1.563208791
3.362510497 5.757065937 -2.465744188 3.7116249454.655319427
4.680690503 5.405399744 3.786824725 3.6311100070.377499576
0.649170504 2.627484179 4.95404183 -1.239566888 3.066691407
4.452976405 3.174529088 5.355285905 2.377010184 4.51384228
3.286700033 5.00293004 1.119533369-3.542812299 3.718629871 -0.82117808
2.666176636 -2.721293412 5.613759328 3.931864733 1.490049893
4.713948241 -1.235571294 5.308540894 2.83719175 4.020552667

4.251420051	7.013135824	1.310501628	3.181823013	6.581102612-
4.414706528	-1.973982166	4.965191463	-2.590131225	3.279410806	-
4.26232229	4.985997569	2.071691588	2.416052884	3.728479518	6.239357682
2.918907781	2.391436516	-1.950329881	4.644840386	3.940859937
3.894290596	4.19534232	-1.583838506	5.0888791196.208539945	2.76273183	-
0.07384867	5.755019933	-0.465516152	6.000049758	5.135712418	4.566277455
5.453889153	6.2197075	11.400096891.349845467	2.77873992	0.511596927-
0.659680148	3.497150849	1.198085804	4.284590049	6.786736403
3.714685098	4.866901105-1.961000526	2.241492905	-0.006069868
3.309213186	3.591491055	-2.342976794	5.3448112964.3115342621.081775477
3.249926528	4.087955573	4.9798631124.832740055	2.962772074
5.335442683	4.868001491	5.056980726	4.366507979	0.691086877
3.1183811234.504861057	4.822148203	4.758847195	5.7115964215.405796999
0.772557898	5.67620575	4.175767535	2.17476398	3.787316031	-0.308295906
-3.760542864	3.031923731	0.186686029	-3.25451925	-2.830838538
2.120655961	2.853796661	4.669438188	4.830012812	3.538338978
6.048791825	0.22555932	3.09074246	4.432224516	3.71969368	3.652314651
TCGA-55-6983-01A	-0.828963968	4.759204202	2.433359551	5.416729479
4.123694544	8.324690975	3.667410794	4.313209362	2.139069175
3.412912473	5.421552413	-2.140415617	3.642003729	4.446835146
4.81597051	4.907726209	3.444329001	4.282784463	0.727434292
0.548810789	2.743522919	5.358275214	-1.381120105	2.511713262
4.582297689	2.937822071	5.780195164	3.009740378	4.55290689
3.395150017	4.7697081	0.987697295	-3.683562922	3.772383537	-
1.054152123	2.685303176	-3.259954545	5.577448244	3.05629059	0.403385218
4.710588921	-1.094844148	5.304581761	2.879671054	4.042241615
4.454722596	7.000362907	1.508836255	3.698768723	6.850568802	-
4.942283662	-1.622485818	4.28404948	-2.34374071	3.507454976	-4.396362012
5.101494474	2.223760074	2.1157214624.121065586	6.086001296
2.845409075	2.13741886	-2.171172173	4.598484223	3.934552612
4.366745298	4.314973635	-2.33107145	5.0819711866.470688377	3.007790613
-0.012131926	6.159115447-0.50597225	6.017634381	5.197173523	4.70816632
5.5118009326.437495587	10.2163984	1.100288534	2.596793323	1.153162103

-0.714127697 3.336833298 0.873152475 4.292820802 6.865441569
3.703062676 4.6859025 -1.984293254 2.16101514 -0.201480797 3.308071218
3.926352095 -2.606115671 5.344159434 4.819622177 1.079991103
3.190884885 4.044873181 4.989290297 4.594043256 3.202659432
5.335492886 5.152620793 4.920074397 4.413566995 0.499975877
3.219310353 4.585810094 4.666068035 4.7496238 5.738678865
5.057733054 1.100816759 5.615985994 4.240069883 1.760757998
3.895493259 -0.530068686 -3.695586195 3.335230527 -0.390628864 -
3.444048824 -3.188605001 1.551735144 2.787541586 4.43402528 5.330545759
3.273471203 6.075196803 -0.107375372 2.950482348 4.580591953
3.81197434 2.526229473
TCGA-55-6984-01A -1.017196609 4.880572385 2.340358184 5.460308967
4.196229849 8.26498578 2.764213643 4.406497675 1.615409187
3.007801986 5.921122207-2.644440816 3.719447846 4.166449876
4.357997577 5.367619605 3.687877038 3.380348533 0.702789195
0.484888181 2.509820643 4.665536087 -1.246930645 1.723245797
4.44719569 2.818983582 5.7742840112.515073054 4.450565268 2.801711951
4.83956329 0.719919862 -3.923453955 3.575908796 -1.215397011 2.59419923
-2.490073312 5.614886363 3.431366596 0.334539357 4.707305983 -
1.270880631 4.703673443 2.403277461 4.178143675 4.320483897
6.947433457 1.151097485 3.43403991 6.430225776 -4.600827245 -
1.94858224 5.282129745 -2.151338951 2.770126081 -5.125755889 5.072948042
1.591302203 1.51720551 3.610902076 6.093933567 2.475746835
1.950449619 -2.517715169 4.55340892 3.9280353113.741391946 4.336763582
-1.249825729 5.085807264 6.213366489 3.026526188 -0.461625022
6.067404173 -0.780337946 6.137507362 5.217151895 4.509850253
5.542605049 5.960275977 10.84396799 1.543692694 2.37347578
0.241742948 -0.49209845 2.932133104 0.92465126 4.390638418 6.747917075
3.6571114044.909039377 -2.202354005 1.992467437 -0.875601993
3.213026077 3.281375629 -2.674449748 5.348233909 3.936584697
0.303317156 2.988523968 4.278937907 4.967032105 4.84310561
2.492025136 5.33528954 4.989037362 4.71398066 3.901844684 0.392154958
1.249054398 4.477724413 4.688830373 4.734789441 5.708944241

4.938887819	0.678934337	5.723010244	4.088402633	1.777219451
3.867930426	-0.344262084	-4.401022873	1.998870292	0.088489293	-
3.223667938	-3.819511851	1.851339749	2.794632015	4.522350369
5.086504689	3.413313708	5.839967348	-0.35062699 3.233213997
4.317391257	3.627484654	3.078901488
TCGA-55-6985-01A -0.598818696	4.707784286	1.672621231	5.317590835
4.282773432	8.278707206	3.426074445	4.263575382	1.876296543
3.699608309	5.205726019	-2.380699937	3.703126669	4.51728627
4.781033916	4.936784688	3.607485307	3.821860739	0.394110255
0.507901267	2.752830282	5.321438048	-1.145991973	2.153329392
4.555072981	2.795646448	5.706898504	2.007007434	4.626393862
3.684633681	4.942770747	1.181553195	-3.552467009	3.266533054	-
0.958805663	2.467121534	-3.469695227	5.591923841	3.580853973
0.581719374	4.713162813	-1.064153819	5.315838073	2.699927798
3.97812324 4.316289826	7.053157517	1.481648626	3.522077665
6.875633312	-4.9134318 -1.837703419	4.731841883	-2.570651917
3.480590664	-4.531529072	5.144550184	2.020352284	2.489304921
4.033130492	6.086866685	2.692467589	1.725883781	-1.885808929
4.640830251	3.929164237	3.867955315	4.361913273	-1.250644436
5.085484632	6.419759183	2.839254398	0.07734098 5.821393901	-
0.243786484	6.002289792	5.1155135294.717901438	5.451356277	6.350840012
10.51060962	0.973125431	2.710519446	0.878298598	-0.606315032
3.417109187	0.902619625	4.276625821	6.865638699	3.487263837
4.864002002	-1.856025377	2.138372966	-0.200729017	3.290830601
3.717540248	-2.368979671	5.341512915	4.652433576	1.179364735
3.241330349	3.948641223	4.93067899 4.728152495	3.139127471
5.335375587	4.779761651	4.998875593	4.076139371	0.726458813
2.746104338	4.517154524	4.795069547	4.764932527	5.629769014
4.918719012	0.954848768	5.536254875	4.11460146 2.074594971
3.919860006	-0.510495775	-4.003529528	3.185419879	-0.206999207	-
3.531183572-3.330839534	1.5838511032.695448857	4.385980763	5.226859578
3.307673998	6.036401988	-0.044999801	2.743683063	4.459929305
3.706890618	2.834313682

TCGA-55-6986-01A -1.059495027 4.833282075 2.954065104 5.581278296
4.374059683 8.256101887 3.524764857 4.33837911 2.028083555
3.049763794 5.428586451 -2.255226649 3.585073685 4.240382068
4.601583235 4.985314864 3.334042092 4.219392217 0.881153949
0.524877853 2.41384967 5.068027561 -1.59904713 2.027225492 4.549248182
2.9118045725.740660477 2.727388316 4.450853583 3.556000884
4.905102214 0.898819551 -3.740428395 3.667684652 -0.906438088
2.833483423 -3.222741053 5.606927544 3.256373693 1.004507864
4.713636081 -1.220691967 5.199472285 2.767015209 4.015818643
4.2363996 6.922827002 1.377416334 3.8480118866.753496868 -4.919846047
-1.776496935 4.837675886 -2.19107875 3.207396516 -5.012923665
5.035002847 2.073662957 2.394388671 3.847330889 5.942401935
2.497058934 1.78435625 -2.804814003 4.669654944 3.93916854 3.949684055
4.30943713 -2.62259714 5.087799087 6.369961376 2.918344462 -0.067070999
6.084538807 -0.880739996 6.140146538 5.102606552 4.639795865
5.503000894 6.312028183 10.4908126 0.639948331 2.560210483
0.910882728 -0.755418673 2.795744271 0.932940993 4.421052387
6.86304238 3.599697974 4.690445507 -2.031359879 2.189682673 -
0.531090077 3.245255094 3.79417393 -2.932510286 5.356459025 4.667293538
0.205969329 2.6950529 4.022002893 5.026562458 4.857234558
3.015809868 5.335634446 4.917218952 4.7591128153.943048225
0.435829527 2.290995 4.358545862 4.701797594 4.7381194365.606476755
4.900053753 1.094979667 5.377326909 4.21900914 1.592166277
3.855934653 -0.653735332 -4.500861066 2.629421084 -0.325967877 -
3.450747495 -3.527917593 1.657663707 2.795779733 4.466568633
5.127176306 3.387824283 6.107935223 -0.187526817 3.05711062
4.397619583 3.582825934 2.680716994
TCGA-55-6987-01A -0.41303241 4.870616922 2.946345937 5.453713781
4.201012316 8.270342457 3.36125972 4.331767958 2.002881507
3.845676198 5.441349201 -2.168947886 3.421823364 4.506156138
4.962910632 4.952197422 3.832671272 3.971551692 0.624990718
0.51667315 2.641349753 5.235439043 -1.46170824 2.927701877 4.601372054
2.776594281 5.942526375 2.524735766 4.481591867 3.489919065

4.764441576 1.115614309-3.754409064 3.655979753 -0.896250803 2.26416413
-3.110935312 5.592314945 3.01216545 0.775634148 4.691595739 -
1.200926654 5.31361359 3.132690135 4.003420389 4.35675467 6.809489127
1.459886224 3.73280877 6.87372078 -5.247364096 -1.693859862 4.837762316
-2.380967175 3.428643369 -4.928024654 5.056682546 1.786424953
2.309873573 3.982839392 6.097066753 2.578584282 2.389083269 -
2.147064599 4.696641039 3.934808735 3.951653677 4.331380872 -
2.1170059915.093304125 6.426498622 2.844486733 -0.137772688 6.01395366 -
0.552309381 6.081635092 5.06120741 4.5735511385.475304223 6.390689648
10.55976281 0.846634464 2.663569556 0.8253439 -0.552390252
3.614767221 0.977933288 4.2511198426.864909897 3.461966825
4.929062811-1.904785993 2.32201536 -0.421896362 3.292073725 4.087094368
-2.392466909 5.337830907 4.793576616 1.204380667 3.285020874
3.833714734 4.982581692 4.730536823 2.739169645 5.33544807
4.906484273 4.916875475 4.097568806 0.516270522 2.712603077
4.613381783 4.669852032 4.775081676 5.5052114244.941748321
1.107348925 5.5497111034.237921408 1.834665509 3.771179136-0.080459211
-4.213285099 2.81335687 -0.557012891 -3.767847857 -3.668721422
1.507679993 2.7117751684.454562561 5.250339272 3.2693118636.040006274
-0.471484386 2.883344819 4.390716715 3.659394097 3.055540851
TCGA-55-7227-01A -0.726365649 4.601360055 2.626555931 5.4210131154.18323827
8.307258273 3.4531011664.358428142 1.623948669 3.213318008
5.339239471 -2.464890247 3.519459121 4.486003239 4.726797379
4.8624944113.099778094 3.719339066 0.686504742 0.538448804
2.417105044 5.282604267 -1.324478647 2.256152851 4.524263885
2.961826364 5.719487972 1.440916473 4.499718261 3.205786229
4.828219628 0.900082364 -3.701831912 3.542106599 -0.909501498
2.762175319 -3.166510163 5.594038151 3.536443174 1.18286954
4.718718235 -1.176579404 5.08257539 2.596318145 4.017192624
4.287086037 6.898593158 1.344208154 3.416023552 6.62755301 -
4.800501777 -1.826909391 4.69486221 -2.446556509 3.271751824 -4.576422653
5.010587373 1.985094135 2.535932831 3.941291279 6.041635526
2.55325057 1.811642975-2.412718045 4.675191824 3.941749016 3.851103628

4.329100148 -1.717346562 5.085497417 6.332989867 2.847033542 -
0.066107178 6.065646659 -0.406864605 5.981386679 5.1155891934.552364255
5.48728955 6.321202944 10.811514 0.71438533 2.700038566 0.718648758 -
0.644953589 3.186387744 1.014450915 4.293267994 6.84893322 3.490622923
4.65336101 -2.061737256 2.085143833 -0.439873746 3.245898498
3.678069721 -2.492215002 5.339918552 4.788964198 0.732514883
2.96212715 3.892307674 4.94180354 4.8426301152.936708761 5.335449843
4.85705185 4.963928989 4.08781085 0.58027574 2.126515042 4.453276139
4.742474471 4.779673787 5.560345464 4.8111890820.830223827
5.656268506 4.4488221152.046915367 3.833024986 -0.371673156 -
4.217640688 3.109099889 -0.094099599 -3.338854541 -3.49245374 1.77920897
2.742033184 4.41540629 4.977879162 3.005634773 5.987492189 -
0.148096922 3.038931 4.313660981 3.623989852 2.880699439
TCGA-55-7281-01A -0.320864505 4.628077265 2.437252415 5.450857151
4.346361006 8.27821926 3.360004762 4.356371255 1.700542099
3.291831947 5.681429037 -2.463724734 4.034458819 4.453254078
4.738661633 5.221241688 3.501278529 3.881621234 0.353372764
0.600480685 2.660368604 5.174071583 -0.981554328 2.352110059
4.466476996 3.072276515 5.546058607 2.007680908 4.60324198
3.364442089 5.017347037 1.224196737 -3.499471247 3.498418789 -
0.9750118312.664355791 -2.919295562 5.606585484 3.8110897250.583503445
4.720315431 -1.122421617 5.101544894 2.48871066 4.093620928
4.291866749 6.920667472 1.242196384 3.2815181146.871632441 -
4.731351047 -1.83928329 4.691558594 -2.437605502 3.230346226 -4.499268507
5.14509188 2.196332602 2.464632557 3.71624358 6.193387234 2.723747882
2.035909718 -1.934573006 4.610812843 3.9278116493.900681784
4.473381331 -1.01289832 5.090061446 6.296379546 2.812405062 -
0.008050468 5.966333895 -0.201484697 6.061307436 5.128091475
4.684005667 5.566028754 6.148945843 10.74866961 1.211686735
2.654324049 0.70469037 -0.504382719 3.468503769 1.0301196424.392424024
6.818353086 3.6302114454.695563467 -1.955741993 2.240462017 -
0.412620942 3.312667199 3.596618473 -2.440686164 5.346382475
4.340720079 0.847427423 3.017706728 4.097949438 4.999555454

4.78867388 3.003399638 5.33550439 4.832697727 4.962336467 4.374157405
0.753436508 2.361305436 4.461691071 4.82852954 4.740423026
5.685433371 5.134660688 0.932698292 5.659017976 4.072703639
2.208149754 3.853721352 -0.361552545 -3.908628159 2.907966044 -
0.086254321 -3.383923997 -3.012175395 2.005846143 2.839331716
4.732461761 4.89492315 3.284330579 5.908579533 -0.070964281
3.128712733 4.513359967 3.725925945 2.979124657
TCGA-55-7283-01A -0.723937354 4.882926561 2.956196643 5.570029012
4.306460037 8.227039392 3.371414308 4.334852863 1.806278649
3.155408226 5.36746978 -2.121221973 3.490456725 4.420434309 4.7849367
4.836492571 3.354648006 4.002976787 0.497555482 0.529432125
2.551506866 5.045290503 -1.376204509 1.903841957 4.533545202
3.056206323 5.717069562 2.089042722 4.477101912 3.725733723
4.881873384 1.028104452 -3.542403403 3.351858728 -0.995969672
2.699622371 -3.464277353 5.596107377 3.392236791 0.628817014
4.70511461 -1.102409765 4.957587581 2.913776421 3.975993401
4.300854842 6.830657103 1.347742204 3.732063249 6.875052377 -
5.104926453 -2.018440699 4.52449035 -2.384200548 3.324933253 -4.787973497
5.020146849 1.956061472 2.2555068 3.98259176 5.924326648 2.549355273
2.041649357 -2.671098886 4.6114844373.940794679 3.925029999
4.348086971 -1.839734002 5.084599093 6.310367783 2.825610898 -
0.045694779 6.037038093 -0.682978932 6.169133413 5.074814962
4.6241125025.537587553 6.417737427 10.24476846 0.623478068
2.679960584 0.922721403 -0.871996636 3.06696816 0.977346897
4.367278899 6.865363051 3.594368206 4.757980139 -1.942458842
2.366963045 -0.389694336 3.281338544 3.731011176-2.451135285
5.368294423 4.47804247 0.632657304 2.774667164 4.040155397
5.001317347 4.7246116153.201716479 5.335567382 4.942187406
4.7790901184.2411825110.561625937 2.75101744 4.530389868 4.711883672
4.736338996 5.598823757 4.963395744 0.980065782 5.58449102
4.261630345 1.804195768 3.832588507 -0.710877833 -4.341011248
2.68486759 -0.393345168 -3.517199308 -3.756779341 1.629236136
2.744453358 4.471305336 5.236664732 3.228515057 6.068971768 -

0.059043931 2.977688012 4.301998105 3.681887095 2.456451469
TCGA-55-7570-01A -1.289876412 4.907372821 1.757322218 5.531909359
4.016367879 8.065498712 4.079930553 3.976469742 2.496493148
4.367939768 5.464936559 -1.701818896 2.918050774 5.218021409
4.804295567 4.798873724 4.840340069 4.618125262 -0.032656128
0.06067279 3.013207645 5.0680044 -1.911719455 2.786735157 4.837621656
2.001381792 5.833471078 3.156704324 4.56100497 4.967091452
4.768577483 1.837024602 -3.650811675 3.143316704 -0.93203872
2.150225911-4.362982521 5.602153047 2.807203463 -0.439291006
4.657754496 -1.19183001 5.382253012 4.334892088 3.561845109
4.280938684 6.780626553 1.294043345 4.740592078 7.384694872 -
5.986991969 -1.201892576 4.886683781 -2.3835822 4.270375863 -4.663283035
5.182084584 1.854666669 2.5050911814.089944145 5.678734738
2.627626066 2.92451377 -1.975470601 4.568576352 3.935731782
4.395624619 4.379444291 -2.790599899 5.063803922 6.443921909
2.880288982 0.319098141 5.423926812 -1.175786493 6.215933467
4.92030439 5.098367413 5.410751744 6.526367067 10.14244137 -
0.300293843 3.037078475 1.860059379 -0.704001575 2.903320109
0.564991973 4.214421487 6.824410745 3.468601661 5.314711258-
2.078253368 2.789288634 -0.299421552 3.434427837 4.170844996 -
2.702776838 5.346348261 3.943532476 1.817581913 2.73992433 3.119598275
5.038325943 4.545434057 3.878040822 5.335415255 4.846953834
4.940553985 4.273121716 0.6067116724.47048429 4.646823002 4.613096375
4.709426215 5.762084955 4.950916762 1.395446967 5.000618749
3.284960406 1.0696511723.803331547 -1.663394622 -4.157663023
2.913870051 -0.615074844 -4.418946564 -3.91172563 0.986385725
2.005321601 3.692541286 5.849226607 3.922855871 6.538980061 -
0.859923439 1.893689615 4.602989858 3.666233252 1.714840576
TCGA-55-7573-01A -0.527740872 4.743831002 2.913347916 5.286489066
4.300469988 8.26828712 3.577109363 4.239147934 1.906743812
3.393732715 5.070148448 -2.275164423 3.257051275 4.703508057
4.887748846 4.980937886 4.009387233 3.858017914 0.586796197
0.51989055 2.875778027 5.453723777 -1.249323677 2.371203029

4.585751271 3.155969726 5.745879547 2.848207641 4.563196283
3.40490568 4.905364553 1.217759287 -3.549185199 3.409324562 -
1.300130534 3.179993735 -3.149743401 5.580586096 3.855158645
1.369564182 4.706128855 -1.179169717 5.256363976 3.348433637
3.998379012 4.291661201 6.932647695 1.792922951 3.843356019
6.674051594 -4.613144652 -1.5609416 4.605300087 -2.469126156
3.494044383 -4.429837259 5.088980969 2.28656546 2.442457547
4.146470586 6.075433718 2.860875724 1.822092664 -2.026778876
4.690794136 3.939510471 4.138567607 4.273891849 -1.410276606
5.088541907 6.364520017 2.990800579 -0.036605341 5.891711154-
0.358446308 6.022674806 5.212168981 4.571816941 5.414981368
6.374503621 10.54229859 0.732021909 2.754338464 0.988460604 -
0.681767577 3.440722949 0.849839598 4.230559943 6.895270625
3.744772759 4.731723113-1.838816344 2.197962202 -0.076451526
3.288132857 3.762587781 -2.104846307 5.357743736 4.792175236
1.618560073 3.170246221 4.168154943 4.941586019 4.794965353
3.23697583 5.335470407 5.121496817 4.913939945 4.1788110320.641957356
3.947471249 4.554506592 4.774004026 4.762931644 5.555031032
5.347937088 0.967369273 5.658612281 4.220737556 2.096501623
3.874072267 -0.316942202 -3.474927714 3.017718453 -0.055140548 -
3.436556862 -3.669004469 1.645359533 2.892740607 4.334733707 5.24801768
3.505178401 6.2228921170.633499178 3.251819086 4.439051427
3.764419453 3.046198038
TCGA-55-7574-01A -0.544669045 4.70094042 2.586772604 5.328041207
3.964896338 8.305736195 3.701813226 4.263878329 1.802231301
3.441608996 5.192643467 -2.435621207 3.303719531 4.354772736
4.85211236 4.699821323 3.632572763 3.868406853 0.740630618 0.51406126
2.60316548 5.549165774 -1.387441385 2.573015797 4.607460361
2.931585594 5.7698966111.077124618 4.49215396 3.174975682 4.822636317
0.788167031 -3.722296218 3.575256237 -1.045596979 2.683704226 -
3.122378158 5.571679657 3.377190137 1.202948694 4.704380249 -
1.221402076 5.145158337 2.943165372 3.997752936 4.2937071126.83082214
1.699895546 3.399723862 6.805609772 -4.809934959 -1.786928851

4.425037506	-2.447345646	3.406376826	-4.895884773	5.08062108
1.997140394	2.161042768	4.097267198	6.048919271	2.641805304
1.969398278	-2.076546284	4.778193864	3.9311868343.912585587
4.301543056	-1.986481184	5.092493432	6.437022371	2.897946295	-
0.086437432	5.945624719	-0.453797878	5.973718465	5.100945996
4.608966127	5.464102292	6.404910797	10.60788045	0.6519841
2.6511143690.720198842	-0.633453206	3.691081559	0.901254753
4.256237592	6.875452826	3.397194659	4.636136441	-1.85244434 1.95994371
-0.550813173	3.223207831	3.94637177 -2.30648407 5.328460525	5.130448586
1.346090519	3.280987106	4.029861771	4.928885754	4.740536189
2.872094936	5.335480361	5.014236529	4.964008466	3.951080113
0.671778784	2.977887167	4.526100772	4.760894395	4.778223071
5.456126577	4.94764655 0.8584611345.563241359	4.1911055461.985116841
3.821950889	-0.148148975	-3.962698642	3.052261734	-0.486039229	-
3.511157107-3.799266966	1.484530212	2.82054278 4.271968098	5.02836588
2.996246846	6.027894568	-0.002687321	3.005486449	4.233833388
3.653058959	3.153353885
TCGA-55-7576-01A -0.633059592	4.733639222	2.556949923	5.419382487
4.415870184	8.2951571193.3661145034.302181097	1.662654314	3.347697077
5.683343821	-2.481148715	3.59871954 4.842574313	4.867736905
5.185386401	3.998163031	3.745021729	0.442982084	0.539304406
2.719533672	5.091446868	-1.266376292	2.44928979 4.538316464
3.106616603	5.571891562	2.098690059	4.573293447	4.030647168
5.000748421	1.191435531	-3.532887312	3.319936288	-0.944702404
2.801694573	-3.223916409	5.6061146383.961070723	1.312120543
4.702295403	-1.208922299	5.185543961	3.07938698 3.949201592
4.155427431	6.934535899	1.301655871	3.615023132	6.704578622	-
4.696888778	-1.923648272	5.18042535 -2.625894453	3.385510945	-4.626661123
5.126653978	1.989132862	2.295073706	3.744687141	6.028072395
2.877638047	2.241161731-2.232912728	4.603529014	3.941905641
3.947675743	4.359397553	-1.120382141	5.08603043 6.2411366242.775953322
0.027978201	5.744696306	-0.568377635	6.143076469	5.05572436
4.696783792	5.441314727	6.382443217	11.029254860.972793166

2.858972418 0.623009941 -0.684247958 3.339644664 1.026536411
4.324749293 6.863492258 3.629968534 4.966501864 -1.844626439
2.545389461 -0.2222439 3.287205135 3.633102989 -2.18445279 5.361573044
4.19481335 0.941670507 3.091288287 4.0293951124.986720756 4.774580997
3.312749931 5.335457181 4.877496835 4.975856594 4.115757096
0.643205609 3.525331372 4.614031675 4.823575785 4.740082768
5.698297635 5.158749203 0.808718765 5.829081721 4.124139682
2.028588482 3.890779644 -0.453967592 -3.84765112 2.621856269
0.081572559 -3.498642804 -3.756599296 1.754895964 2.739529818
4.406983886 5.2111487953.545473817 6.156301013 0.213493735
3.101583225 4.433048559 3.592178896 2.996090627
TCGA-55-7724-01A -1.284262989 4.486849056 1.689421567 5.659190106
4.3831191998.383724378 3.548541652 4.251958058 1.870682949
4.153442479 5.467384 -2.261598637 3.003693003 5.090228163
4.553940695 4.529096414 3.615272226 3.385469741 1.123383825
0.462702083 2.400191034 5.575831677 -1.570249721 2.931045128
4.59853194 2.607399694 5.825555345 1.605832947 4.4512950113.613513851
4.4263541140.970396765 -4.138370108 3.587018453 -0.818615113
2.326034444 -3.720804934 5.571619981 3.5220311941.309219589 4.69111884
-1.516434537 5.654658435 3.017631408 3.753720543 4.445809555
7.1105190651.148303563 2.914961534 6.470782746 -5.386270126 -
1.702483781 4.758335505 -2.942318582 3.575854445 -4.113753862
4.536698603 1.358510368 3.276862784 4.162409153 6.090273485
2.725283678 2.037646166 -1.918630616 4.4516315113.945326394
3.885108281 4.119137081-0.826600723 5.088563024 6.173103678
2.767084262 0.10859455 6.148209634 -0.582452393 5.696678798
5.286321026 3.952204014 5.329509415 6.405390566 11.3881334
0.967825134 2.617403878 0.762313824 -0.790796365 3.046838637
0.96694997 4.202326523 6.733663509 3.492324154 4.819779147 -
2.399143019 2.027891451 -0.142091154 3.286175778 3.658734106 -
2.025677752 5.324889574 5.479499381 0.855268022 3.43864901 4.358138625
4.764696332 4.712626841 2.578751306 5.334837792 5.155106961
4.960234303 4.328424977 -0.083254062 2.28042998 4.402265261

4.393820985 4.890088744 5.645702 5.048736287 0.61692471 5.549208803
4.417033226 2.272530741 3.580543757 -0.268114455 -4.079779417
3.760756108 -0.226351353 -3.513768652 -3.197779843 1.583171661
2.322397165 4.274125674 4.926218335 3.189058049 5.487777624 -
0.265389988 2.667790624 4.2272110843.493446596 3.343191407
TCGA-55-7725-01A -1.279114113 4.651549549 2.28218483 5.589882904
4.478049699 8.325629516 3.587705048 4.330724785 1.923424653
3.402302604 4.87291362 -2.221429938 2.9411009665.1406251134.693251389
4.4508507 3.229870176 3.454664269 1.385347348 0.5651127432.117994274
5.646585283 -1.899400872 2.136877908 4.626997504 2.637476582
5.787125327 1.1145331494.226043082 3.223907427 4.255349485
0.633648389 -4.095131515 3.527695562 -0.734790992 2.489772547 -
3.723276424 5.587726371 3.225342057 1.564893058 4.684830652 -
1.465774539 5.44937679 3.029833035 3.825263854 4.434378949 6.889656505
1.1112200733.391019934 6.194225862 -5.060925208 -1.77969817 4.772352398
-2.270108128 3.408422108 -4.650182583 4.637598772 1.178484733
3.179705303 4.282252773 5.831079843 2.648662769 1.461177176-
2.962310387 4.454486884 3.949043914 3.616424964 4.240370465 -
1.928602841 5.078856343 6.076229205 2.945314752 -0.109105211
6.126378135 -0.845141033 5.875326725 5.251769244 4.064725946
5.324436956 6.469651092 10.99262401 0.261332451 2.8015131110.7614498
-1.210583428 2.869484727 0.85645881 4.22820158 6.7611728 3.526309556
4.651038922 -2.232536202 2.089114528-0.178261305 3.195792184
3.605992348 -2.487658641 5.334912097 5.415125161 0.540454669
3.133362232 4.3728380114.800454037 4.913362145 2.574012775
5.3349118085.016778857 4.734098677 4.118303829-0.222897201 1.964344355
4.213882039 4.290134245 4.842761618 5.445791445 4.772020362
0.64485116 5.560889323 4.824763998 1.940503893 3.628673068 -
0.305488818 -4.282973493 3.194115428-0.190031322 -3.307831757 -3.719443791
1.488548279 2.272662677 3.937397445 5.035071287 3.385598107
5.662767144 -0.538609949 2.631033449 4.237280345 3.590536828
3.063381152
TCGA-55-7726-01A -0.953657721 4.295153272 1.1137775425.632739876

4.150199662 8.374140133 3.345419663 4.277906449 1.63333775
4.214505471 5.972605362 -2.606800823 2.96296329 4.869874557
4.303034491 5.09678823 4.202585057 3.367275827 0.686933738
0.403786536 2.530999788 5.531227816 -1.51478874 2.814858793
4.564766342 2.276505268 5.532867273 1.334064274 4.478469944
3.479069432 4.727196044 1.342189347 -4.139680133 3.48882829 -
0.73479013 1.732062049 -3.328536442 5.60965046 3.676597237 0.590725784
4.691157787-1.456471481 4.997678616 2.810368839 3.8011397354.210979028
7.008165196 1.017779697 2.990565267 6.80557135 -5.403510411 -
1.752884086 5.632572231 -2.851515671 3.211071365-4.472862246 4.79894001
1.673338825 3.089709513 3.751833328 6.098209797 2.347009448
2.197302849 -1.437874729 4.61157732 3.939899772 3.862457938 4.21838161
-0.88216113 5.076807936 6.246094846 2.600223505 0.023178535
5.634650255 -0.724455099 5.888300164 5.142264797 4.473628535
5.383226481 6.36706211411.956575 0.880151013 2.575220803 0.436890518
-0.31565806 2.8112906190.996484842 4.241522445 6.71851602 3.299290043
4.903516537 -2.313162824 2.020543041 -0.444991966 3.312286104
3.715569189 -2.156963431 5.322016903 4.763019034 0.786514153
3.335478796 4.140352021 4.802761955 4.949222221 2.440634775
5.334939385 4.463683186 5.035032065 3.83362749 0.539966103
1.500452833 4.1106560224.615781784 4.812058435 5.465568763
4.832004505 0.42045224 5.354755723 3.581667343 1.997381299
3.6323731170.355602187 -4.483531623 2.960487832 -0.343319084 -
3.670804913 -3.082089012 1.557340335 2.248012838 4.342857482
4.702364664 3.178151063 5.822798363 -0.792911814 2.618280387
4.230598535 3.362862632 3.252095675
TCGA-55-7727-01A -1.322394581 4.357207102 1.881787195 5.545730787
4.69149085 8.362846265 3.480076738 4.269472369 1.802239591
3.630073728 5.046561784 -2.101050558 2.906404955 5.455988238
4.703102179 4.604120739 3.641914264 3.414681229 1.005781507
0.478944686 2.36866691 5.630522233 -1.788539763 2.381545517
4.640619106 2.693931001 5.521028183 1.13753682 4.278025766
3.470767534 4.49801925 0.855974635 -4.065267328 3.469517758 -

0.60151394 2.569383414 -3.939075869 5.602183772 3.874263599 1.708030529
4.680583925 -1.546967699 5.487498685 3.356003662 3.77954143 4.31759812
6.988803767 1.220188478 3.546081631 6.440065468 -4.908646805 -
1.871712689 5.151358099 -2.382239102 3.757294221 -4.406266628
4.640535466 1.176222424 3.357874705 4.249588655 5.867380946
2.674330833 2.130288446 -2.466103045 4.432551306 3.954287043
3.742193649 4.227887384 -1.539882037 5.069878141 5.997050225
2.85003406 0.037727751 5.820716531 -0.944410567 5.866088478 5.17508936
4.237429193 5.356573018 6.636335182 11.146485040.084094906
2.938969372 0.771382556 -1.225822297 3.129583075 0.784961381
4.171264877 6.702643269 3.512803576 4.962635577 -2.125966967
2.1158647850.45085561 3.24588509 3.483179686 -2.149291038 5.329690952
5.180684379 0.9912116723.329297075 3.96674012 4.78880834 4.950112114
3.09172013 5.334855643 4.884795259 4.908200948 4.223405199 -
0.071826048 2.867548248 4.4812421164.387322101 4.8259611985.551701978
4.874378127 0.410585628 5.741013946 4.616855901 1.967676948
3.682937168 -0.220327091 -3.706936115 3.482535652 0.030074038 -
3.351145327-3.752114883 1.419208506 2.232387257 3.979540868 5.222935855
3.594289097 5.878553178 -0.310082189 2.5416251124.3511921813.560831946
3.38139329
TCGA-55-7728-01A 0.624033292 5.321074208 3.193803024 5.078836608
3.936868708 8.240626698 3.61548128 4.379675658 1.940949126
3.030771498 5.202196947 -2.547905092 4.091852402 4.054563521
4.972789499 4.969568399 3.335683772 3.983371247 0.482097717
0.615218207 2.782549714 5.189326084 -0.468699439 1.829685295
4.482569624 3.22059089 6.043943742 2.351990246 4.727530226
3.126194087 4.91851857 0.634683891 -3.113127072 2.935374903 -
1.60060435 2.85211345 -2.643349923 5.57926765 3.144946179 0.375067911
4.733667175 -0.871742349 4.826005051 2.1174429214.234185138
4.404991598 7.0811536651.590096414 4.075677128 6.913398043 -
4.290739761 -1.712850164 4.462078575 -2.119682101 3.405398653 -
4.79625555 5.330589752 2.430306741 1.885473631 3.965217416 6.225620221
2.775484008 1.18297533 -1.621437687 4.788503283 3.918061475

3.9868114564.426904738 -0.150513425 5.105444385 6.492876841 3.07986002
-0.297358709 6.169843078 0.565229766 6.06330814 5.192874585
4.786591928 5.49588508 6.02381992 10.1010123 1.091222446 2.740767496
0.534246067 -0.436404425 3.722805876 0.997283398 4.303198626
6.864902532 3.678166051 4.509398847 -1.907940472 2.1250842 -
0.529289719 3.174179012 3.704221322 -1.93173747 5.35361382 4.613878637
1.072298368 3.034148757 4.377488175 5.048204046 4.713554557
2.917190931 5.335808429 5.20230727 4.904296487 4.34597476 1.060985776
2.140101946 4.708183895 4.979425545 4.765739207 5.67533024
5.170579267 1.1116266245.533440551 4.4910661112.401406791 4.019986016
-0.634904869 -3.773761223 2.776936782 -0.157837424 -3.283089994 -
3.06437267 2.088447647 3.32962864 4.6280261175.468268383 3.339792557
6.169477357 -0.45250222 3.189145883 4.634186644 3.93223782 3.224119463
TCGA-55-7815-01A -1.627893173 3.796762168 1.673303823 5.633425223
4.446149385 8.634452153 3.351630065 4.372017365 1.737095623
4.162770513 5.464876324 -2.254081885 2.859391025 5.525465289
4.458072265 4.8527671163.374437044 3.204256642 1.569076614
0.578897893 2.303384997 5.94781461 -2.09751278 3.65012049 4.622785338
2.5831173475.6572982111.529442398 4.224122236 3.126714284 4.207549654
0.946089197 -4.299069593 4.259134482 -0.535336332 2.095262084 -
3.59580494 5.593037678 3.958109343 1.688663612 4.68448717 -1.762569224
5.807855412 3.425924233 3.781295331 4.430534594 6.971821146
0.968671003 2.748846634 5.840668205 -4.768634045 -1.651998166
4.779477862 -2.328621444 3.765831194-3.968566829 4.353841331
1.013867538 3.045106199 4.186038842 6.11221628 3.077976017
3.023970319 -2.153707119 4.358149078 3.960248525 3.768324099
4.001629295 -2.320279796 5.070770417 6.092414937 2.845664709 -
0.156901684 6.099629662 -0.932511077 5.625206316 5.398608017
3.926171958 5.347167197 6.674856185 11.526038061.398894864
2.834696898 0.784936431 -1.209867289 3.105795816 0.862237493
4.148487104 6.627681035 3.756555217 4.726098422 -2.305691871
1.929340655 0.527149852 3.316572386 3.616929498 -2.575794357
5.313724061 5.630758088 1.172388867 3.606920245 4.262563033

4.713954644 4.827075529 2.770597174 5.334714194 5.454518001
4.965475158 4.491231902 -0.483003105 3.024312668 4.317920115
4.199448397 4.854338596 5.594794772 5.0945211250.396746615
5.924394044 4.371725751 2.040354438 3.526012661 0.300456491 -
3.40311909 3.988600953 -0.009657799 -3.051385095 -3.32306717 1.720568336
2.08035698 3.956152261 4.62328123 3.447959271 5.485980463 -0.072955874
2.430899256 4.3391441133.538672255 3.234455429
TCGA-55-7816-01A -0.141140461 4.262055167 2.758587314 5.432863937
3.720635501 8.460353507 4.04671537 4.329909237 1.62964272 3.78845182
4.987021078 -2.187473672 2.529163145 4.621881608 4.823984305
4.596365234 3.831804778 3.958550152 0.486371539 0.410061776
2.67025766 5.949356248 -1.470445692 3.31869493 4.617092446 2.834126563
5.570906547 2.718825507 4.41728265 2.761666824 4.755770249
1.158459313 -3.779031563 3.865022637 -1.02887904 2.392079426 -
3.581099714 5.562246527 3.781909096 1.0665801 4.7182285 -1.374703256
5.127393789 2.975227886 3.89889472 4.210540128 6.707383608
1.266157735 3.067314769 6.83973632 -5.159676927 -1.417388282
4.310640558 -3.089122842 3.786117317-3.903520505 4.851574282
2.370502532 3.1010599 4.157828312 6.1122912972.809810001 2.149806092
-1.745902545 4.838641425 3.937604153 4.2429116434.204304938 -
1.999087831 5.102472858 6.538237907 2.755944834 0.08434726 6.013674798
-0.403769982 5.824354824 5.244683778 4.342620785 5.383682903
6.5072947 11.229389170.565270662 2.648414758 0.89309368 -0.293086784
3.524309531 1.019928414 4.201054707 6.891578075 3.479462019
4.302257668 -2.214988301 1.735775642 -0.091393065 3.328648114
4.220448416 -1.943482885 5.321919485 5.479718875 1.447913296
3.402725541 3.883010553 4.907589528 4.863685459 2.950535349
5.335570806 5.231686496 5.107040145 4.520845881 0.767542781
3.220767253 4.1611332154.861870221 4.832270258 5.346558384
5.288858473 0.777279576 5.4811150874.392442731 2.434260534
3.635756985 -0.615794844 -3.299712946 4.084370795 -0.702125072 -
3.72927298 -2.898422028 1.48873696 2.77230015 4.162736731 4.397035325
2.87767813 6.014417523 0.2204641173.282742339 4.148033155 3.64458234

2.928587937
TCGA-55-7903-01A -0.877167089 4.846668167 2.563203197 5.421585255
4.479014179 8.189342368 3.338786158 4.294958263 1.749616498
3.170589644 5.160959513 -2.346079719 3.3236531164.810153234
4.879410321 4.851694929 3.428399152 3.829948465 0.274528287
0.419490692 2.543717895 5.205085137 -1.458005525 1.891032838
4.615563839 2.874344253 5.593080834 1.780580034 4.480871089
3.986291314 4.965708412 0.970108514 -3.574408749 3.179175569 -
0.725332096 2.909887624 -3.402880475 5.615442563 3.741503807
1.015129603 4.694837777 -1.249904445 5.165207307 2.785790505
3.924895899 4.12060834 6.8224808111.271995054 3.869065145 6.691513559
-4.831689602 -1.999951201 5.237854155 -2.311264168 3.356318881 -
5.047459374 5.059998678 1.6118361212.409945206 3.855309547 5.807752068
2.389382213 2.00192064 -2.624115207 4.609979599 3.943248203
3.826314099 4.335960009 -1.661465572 5.087930151 6.208313342
2.881687259 -0.030189068 5.541823922 -0.773652737 6.142288119
4.946219096 4.714879288 5.461991972 6.40839584 10.68028525
0.355836757 2.914045569 0.837459516 -0.781624755 3.010835625
0.941502751 4.323641357 6.851399968 3.267066983 4.901262696 -
1.958425712 2.469595546 -0.322469307 3.235557035 3.609533235 -
2.473998727 5.346502239 4.36549053 0.283768404 2.773267312 3.562632734
5.006905569 4.963673502 3.257549033 5.335516082 4.624455943
4.914029959 4.017685856 0.472820471 2.445288706 4.558985797
4.746422563 4.749847627 5.5111540554.616615864 0.728026543
5.512638316 4.205423921 1.775864162 3.86772575 -0.746292016 -
4.040334567 2.504089049 -0.087165232 -3.60913351 -4.164672459 1.452866346
2.672237287 4.060100846 5.38558473 3.525634605 6.136341277 -
0.237452049 2.770220866 4.353035023 3.570067934 3.044621567
TCGA-55-7907-01A -1.043162648 4.62516997 2.033237931 5.364876583
4.4239220118.317313871 3.508123974 4.236272487 1.774995745
3.419362227 5.585079713 -2.315791046 3.594429816 4.820275924
4.571700142 5.066089074 3.634917786 3.756252866 0.648522489
0.5111912142.68949032 4.971932751 -1.375504342 1.908505869 4.573598017

2.993917133 5.482089384 1.867873552 4.521259901 3.99580741 4.86139135
1.106955584 -3.69629838 3.132104168 -0.965339925 2.653138956 -
3.406328047 5.599803613 3.643642306 1.02419641 4.704900974 -1.226769779
5.259776472 2.890901412 3.910963723 4.242924859 6.97830398
1.252624652 3.392837449 6.908451524 -4.59987946 -1.873752427
4.854122722 -2.741127947 3.459436145 -4.47068267 5.01399553 1.841685269
2.505069368 3.931980281 6.001830118 3.012778157 1.828516115 -2.111732812
4.504031452 3.938012686 4.052122713 4.380274102 -1.179453338
5.082878402 6.163675178 2.857486252 0.136554252 5.724751743 -
0.61058597 6.031403729 5.13109982 4.695184445 5.442626181 6.382750539
10.78220735 1.423036312 2.643909909 0.429964128 -0.986613002
3.314731807 0.945005818 4.339757045 6.823621102 3.715906 4.761291624
-2.015802424 2.444192297 -0.215354014 3.304008164 3.637497278 -
2.537728059 5.351856097 4.455601744 0.819798344 2.790406172
4.353644318 4.944726108 4.633526629 3.455990005 5.335407549
5.030888916 4.895490997 4.239780207 0.535869713 2.990674733
4.537817085 4.708576022 4.745508079 5.822176856 5.189283391
0.801207755 5.701391954 3.876383114 1.891233042 3.863252198 -
1.019376272 -3.738116535 2.916760282 -0.180002021 -3.294453021 -
3.456318717 1.560210882 2.554010867 4.18022201 5.115615231 3.503433122
5.917042212 -0.112774035 3.112318827 4.486984775 3.602773484
2.811458641
TCGA-55-7910-01A -0.260306365 4.901912587 2.228822039 5.299602973
3.965350814 8.279987726 3.737900708 4.169543195 2.275283241
3.690836097 5.195785761 -2.100777751 3.326731674 4.877892184
5.004946085 4.842918136 4.176809806 4.118089433 0.832275117 0.527411459
2.85138246 5.509265176 -1.135410455 2.480438367 4.721085024
2.470193465 5.632463537 3.012715163 4.478501655 3.84021012
4.518311013 1.133883805 -3.669520416 3.273119743 -0.868896699 2.395753443
-3.233315003 5.572592555 3.159170102 0.581437558 4.682649404 -
1.231876495 5.169916266 3.636192791 3.910717756 4.386206286
7.039141043 1.714136214 4.496029353 7.091034647 -5.120706525 -
1.622272535 4.816171357 -2.216860886 3.499583773 -4.339628577

5.138978664 2.3134114512.710502988 4.285357654 5.901455677
2.795687373 2.215619441 -1.500159674 4.586549501 3.937398976
4.5051198274.436105242 -0.350541878 5.061370446 6.414133788
2.926960031 -0.044907436 5.754372964 -0.503292628 6.093453725
5.143058366 4.973934082 5.443701778 6.705760814 10.12815531
0.387745718 2.982186999 1.262974683 -0.728073003 3.468284972
0.702792467 4.219041002 6.840771412 3.612064695 5.021487437 -
1.854510197 2.571515614 0.023262358 3.282045705 3.872019305 -
1.499990162 5.345467824 4.665155967 1.627795597 3.313064414
4.242023771 4.956854779 4.646056457 3.368179424 5.335177558
4.958945085 4.903269236 4.1148624510.76360457 3.671813444 4.52493392
4.603144374 4.738318306 5.609548886 4.806295037 0.922570937
5.5376211513.9221679 1.873592365 3.958241036 -0.324351884 -2.881591448
3.263006155 -0.414618397 -3.631807973 -3.150753199 1.217437869
2.6611037974.1177869885.834040291 3.343549384 6.337539449 -0.579276478
2.573769437 4.552712076 3.6927011282.549040042
TCGA-55-7911-01A -0.795266379 4.789853653 2.5656953115.412680768
4.305726098 8.229913503 3.580605886 4.339656791 1.685384614
3.377160997 5.180161607 -2.161149852 3.425416722 4.813653
4.768454215 4.756122619 3.196262746 3.860242814 0.332875954
0.305570587 2.521836565 5.243745076 -1.354850576 1.785509902
4.618214461 2.826023857 5.820487858 2.232078276 4.549802555
4.19645681 4.824318316 0.879826458 -3.576718654 3.113385254-0.715395016
2.868189992 -3.537665563 5.60512636 3.358694606 0.464877241
4.700958401 -1.176170251 5.204821655 2.566150279 3.923453692
4.198326004 6.885406495 1.173661001 3.892614493 6.664841929 -
4.89232786 -1.899757895 4.988856965 -2.356939999 3.55474991 -4.65853706
4.983268802 1.978243936 2.631937938 3.908906926 5.872449163
2.566152643 1.961294736 -2.139687778 4.597569355 3.943247342
3.951013364 4.299565918 -1.209325854 5.086570745 6.167196689
2.857722624 -0.033819768 5.83157137 -0.574811548 6.055207599
4.995207526 4.69864112 5.452548232 6.404143652 10.59856948
0.283813263 2.83398447 0.798643762 -0.686039877 3.141899932

0.951119311 4.308542879 6.792312088 3.345462663 4.776857461 -
2.216332817 2.558154643 -0.067122434 3.244323246 3.619222792 -
2.331013644 5.35155355 4.538993553 0.290793257 2.774476141 3.740977555
5.006890676 4.869735934 3.340624657 5.335532386 4.744602518
4.977378572 4.228120982 0.498241241 2.301462288 4.540219063
4.806637372 4.771941648 5.560300931 4.886512169 0.693701136
5.488521127 4.215879563 1.9210043 3.892719359 -1.032891767 -3.730523177
2.994569882 -0.030843763 -3.630270125 -3.709589371 1.519853398
2.673158514 4.116235664 5.37253424 3.522683099 6.121827081 -0.627263248
2.681245713 4.428271921 3.561725744 2.874016262
TCGA-55-7914-01A -1.15167433 4.758487661 2.377741917 5.393719588
4.293178949 8.216895087 3.48832123 4.233350763 1.779854589
3.081278537 5.233406512 -2.252592654 3.14699116 4.587409667
4.712239674 4.868586297 3.623878586 3.686813836 0.540671363
0.401126845 2.468041008 5.006364103 -1.728031887 2.041588191 4.6027256
2.791155256 5.727624758 1.270020532 4.3592425 3.602035962 4.779789757
0.897582149 -3.671388646 3.192804126 -0.957502477 2.536242732 -
3.916578425 5.602051946 3.219365027 0.887620461 4.698380482 -
1.224002194 5.093888707 3.181961266 3.818177066 4.288768982
6.801812609 1.285608002 3.712724382 6.753228913 -4.970691571 -
1.95697747 5.01971163 -2.375418192 3.207257534 -5.055466007 4.906094368
1.753238423 2.117076155 4.047062942 5.858283867 2.395917555
1.598510037 -3.145943864 4.606506007 3.944287705 3.749173804
4.246291231 -2.603241716 5.085428458 6.22871233 2.936356679
0.016214643 5.85195436 -1.079720824 6.131882478 5.067691009
4.544683093 5.43340809 6.435707307 10.5600947 0.467160739 2.770396364
0.668649667 -1.106407023 2.698607993 0.866013392 4.300094123
6.86798802 3.528093471 4.836748393 -2.150810973 2.149587253 -
0.593518546 3.234351999 3.674841984 -2.744172512 5.359051093
4.522248141 0.867472745 2.780685238 4.041631824 4.952065295
4.832396424 3.120316389 5.335524611 4.795183357 4.790363125
3.822447613 0.272187015 2.834477994 4.281332155 4.662614026
4.752639732 5.626211549 4.971244273 1.025385059 5.339945109

4.498306688 1.634404601 3.794300596 -0.95769644 -4.417930728
2.757381332 -0.337317408 -3.542795897 -3.847966584 1.31831292
2.565471294 4.220390245 5.270796533 3.392604061 6.078982685 -
0.179350794 2.763100138 4.274623576 3.663744266 2.770366366
TCGA-55-7994-01A -0.532133036 4.949514319 2.366842642 5.307144197
4.653213652 8.163706474 3.222540856 4.240620194 1.761663572
3.184869397 5.290657119-2.582263705 3.4900811984.936729926 4.97843208
5.11304867 3.4698611543.391784142 0.465801476 0.551547452 2.439130719
4.814925134 -1.301463404 1.926395416 4.593764331 2.951840991
5.707396982 1.323247613 4.5118613923.892242572 4.913512024
0.872262072 -3.49903962 3.170831118-0.784288019 2.650488401 -3.123553582
5.620450162 3.772047329 1.735595522 4.711738936-1.170454775
5.2117246442.745946678 3.989934756 4.145456956 7.009638185
1.531747172 4.029426634 6.623320811-4.407549621 -2.183498546
5.426775772 -2.208417379 3.188091614 -5.18924186 5.19326423 1.608976308
2.588020843 3.75343193 5.891405788 2.580148529 1.654518175 -
2.810720809 4.702536413 3.950386335 3.472203904 4.498957811-1.051290613
5.088668419 6.036579559 2.865279954 -0.120993981 5.600026545 -
0.417984165 6.1192178565.00747091 4.656918045 5.435953223 6.173412301
10.90200429 0.408554674 2.949460998 0.542121594 -0.85377111
3.317376488 1.07408666 4.287142499 6.845133321 3.386623082
4.924115754-1.810135782 2.59627018 -0.418990841 3.189864752 3.488915749
-2.394414302 5.356441068 4.351621745 0.736750188 2.7224872
3.929665888 4.990727123 5.016373355 2.881502707 5.335517133
4.333381091 4.93164296 3.633219468 0.678270915 2.32759505 4.468301349
4.803716378 4.7811963255.493779736 4.509105586 0.699730627
5.583584262 4.512562229 1.956465638 3.924275801 -0.639415773 -
4.073707199 2.398238279 0.238643143 -3.315712575 -4.045258896
1.624083033 2.894267917 4.321692319 5.421387585 3.694862221
6.278773417 0.21541778 2.809528407 4.428718994 3.565494317
3.346328129
TCGA-55-7995-01A -0.755598459 4.886796651 1.816613517 5.080549996
4.356730392 8.150546202 3.652197429 4.170880535 2.065114539

3.896163841 4.992310748 -2.080991507 2.964860331 4.749145266
4.877099707 4.946055166 4.3560611424.02848141 -0.068416049 0.22234734
3.06871973 5.092281579 -1.048294865 2.270998277 4.664269198
2.620141877 5.695860849 2.96485369 4.775079799 4.178939659
5.043728349 1.319955925 -3.465873043 2.827780537 -0.996156122
2.769372645 -3.573046774 5.587922357 3.491471938 0.727559718
4.685208122 -1.123003778 5.15216626 3.354957536 3.858593857
4.301900994 7.040434442 1.640850522 4.296078877 7.098961242 -
5.077824552 -1.507757216 4.547844774 -2.572533417 3.638455249 -
4.560945963 5.183395695 2.308427274 2.847847853 4.0301323115.999098515
2.58931312 1.837430925 -1.410935018 4.634393372 3.924194597
4.100648393 4.293222785 -0.265088863 5.0855933 6.537598083
2.922786052 -0.028177518 5.600805991 -0.27107147 6.047708913
5.0522497114.903646504 5.40221779 6.198246017 9.852733172 0.51880215
2.916124629 0.986807514 -0.649434708 3.39804127 0.643836753
4.180746765 6.850448137 3.498644701 4.949087842 -2.142431937
2.510899104 0.007821554 3.37717211 3.774759988 -1.865025536
5.334313233 4.49092871 1.786687126 3.234927018 3.804764777
4.922893214 4.655755317 3.724484488 5.335419976 4.62793147
5.050120171 4.292923314 0.689550869 4.0573541134.520662866
4.802366987 4.76731513 5.740919159 5.357837877 1.063345137
5.4951152243.7171146571.961403924 3.946977713 -1.054962191 -3.476883521
3.186401356 -0.430602556 -3.880872894 -3.761047099 1.38213888
2.702593712 4.056894202 5.6987112793.683974034 6.292659031 -
0.365552685 2.702288971 4.54781273 3.822367225 2.852817667
TCGA-55-8085-01A -1.015703897 4.869527488 2.9118516535.393482915
4.027441682 8.155744557 3.541692127 4.246854954 1.864622704
3.456798896 5.297655406 -2.126637904 3.420283447 4.586235002
4.895825183 4.812596621 3.897335222 4.255936896 0.025372116
0.254429161 2.659679846 4.990428779 -1.602675741 2.322372136
4.596235103 2.74881993 5.862892145 2.473148745 4.476692269
3.976269799 4.785096793 1.13232144 -3.641928734 3.206798428 -
1.031224822 2.269355711-3.411585613 5.598779324 2.761775038 0.332885707

4.693249352	-1.186505705	5.078902296	3.181533699	3.907221672
4.206510447	6.897095342	1.351046955	4.123833873	6.6535694	-
5.279796197	-1.927467489	4.768232337	-2.443225049	3.408823641	-
5.016686803	5.042675977	1.719982494	2.023076976	3.903315047
5.893156696	2.525958032	2.37858077	-2.12927147	4.62398604	3.93860311
4.015775673	4.190136891	-2.225300053	5.085558697	6.394430776
2.902269806	-0.116153174	5.842197671	-0.980106394	6.118186805
4.970292376	4.699943662	5.525730036	6.370275681	10.27853972
0.340247761	2.691146882	0.808890649	-0.812953864	3.109541539
0.761392583	4.329181658	6.889885793	3.503967843	4.993336833	-
2.116965332	2.36659356	-0.399317276	3.296282188	3.832196244	-2.591210035
5.358903956	4.26092219	1.083982725	2.964294635	3.778958315
5.004519084	4.711645874	3.208427359	5.335471941	4.761894399
4.794921175	4.105422105	0.503664883	2.837406027	4.637239219
4.707929083	4.732115464	5.69446094	5.083887068	1.221438259	5.522481799
3.945349961	1.647596189	3.844048352	-1.092198994	-4.315461755
2.684691671	-0.269444141	-3.801319735	-4.17886301	1.371740937
2.568386554	4.175819537	5.525481903	3.433376137	6.273216003	-
1.000289358	2.408524693	4.361169976	3.629253482	2.574751012
TCGA-55-8087-01A	-0.876877292	4.744218313	3.072681396	5.273123574
4.371141333	8.233345226	3.668063757	4.182631813	1.991101163	2.812601258
4.96305955	-2.240394937	2.945740427	4.655055708	4.672844238
4.785370507	3.802061453	4.011935265	0.360710814	0.334785062
2.777862117	5.579771488	-1.282921895	1.788974113	4.596006279	2.939875933
5.441862277	2.623178268	4.491419266	3.223538958	4.904054145
0.830856134	-3.734663356	3.151599082	-1.234660853	2.991076621	-
3.74866468	5.576103741	3.99645764	0.943719406	4.705653139	-1.269888852
4.775828601	3.333403878	3.925175433	4.225807602	6.966355573
1.690600714	4.177213628	6.826197475	-4.884450367	-1.669851634
4.407421677	-2.558247078	3.530948002	-4.80297377	5.108619559	2.10842554
2.473777096	4.048894205	5.89739849	2.568545928	1.552301533	-
2.670574963	4.626529835	3.936607474	4.147204919	4.27704351	-1.311227646
5.07383561	6.406525305	3.062934254	-0.073354948	5.926887774	-0.6881631

6.085772581 5.180915087 4.719692968 5.43594939 6.474686159
9.968205432 0.422803294 2.676065906 0.876340531 -0.990356711
2.749229091 0.593419058 4.280421791 6.880684087 3.636298991
4.629138473 -2.002273625 2.004547927 -0.172100105 3.228730744
3.682847998 -2.132585561 5.36224431 4.785549132 1.084288869
2.771004995 3.942527607 4.958955416 4.781371538 3.543580496
5.335351856 4.969082893 4.822447231 4.163139584 0.517974649
3.7831165224.445752027 4.749768798 4.743836673 5.599610473
5.0941694110.847926968 5.606054849 4.2431161421.869014206 3.967479512
-0.839982881 -3.629690978 2.91829246 -0.312369265 -3.484624363 -
4.104863868 1.373042056 2.712120562 4.135239856 5.487805391
3.460196953 6.310636539 -0.047406133 3.143278726 4.487469261
3.713000768 2.240070895
TCGA-55-8089-01A -0.475850235 4.606496392 2.408130443 5.402783269
4.2496811228.271641395 3.234817887 4.349556937 1.515690718
3.483998626 5.491641953 -2.703550631 3.538604735 4.6096575 4.50870199
4.867053675 3.649661358 3.497180278 0.244469826 0.402515526
2.485160186 4.912254549 -1.331089705 2.473778545 4.504707403
2.819663622 5.799543933 1.049219147 4.429829235 3.24521588
4.870218663 0.850474002 -3.772373445 2.984281468 -1.118639888
2.294337763 -3.339075035 5.605083029 3.108927998 0.424004898
4.703871666 -1.248402751 5.156831356 2.551917406 3.950841773
4.210004622 6.892543602 0.951561327 3.274743413 6.653616461 -
5.176255638 -2.008864848 5.367329167 -2.78607531 3.321724314 -5.225727862
4.92651995 1.617288934 2.335717257 3.765653237 6.096877489
2.414228302 1.994316567 -2.504206148 4.670144913 3.938217858
3.569704526 4.271373194 -1.690584946 5.088862517 6.108624683
2.794001794 -0.063625888 5.742540965 -0.425992299 6.007739229
5.064454933 4.433303359 5.444524564 6.139352382 11.24005329
0.908147627 2.540648983 0.255521158-0.568417947 3.480315387
1.2116888994.268247231 6.782122426 3.362187016 4.798291183-2.250454791
2.057123151 -0.626731529 3.2221108143.738454626 -2.501996943
5.347267741 4.497931736 0.596293566 2.933751365 4.041456535

4.953473321 4.860896657 2.541813602 5.335338591 4.651944535
5.000807631 3.8927613 0.683887202 1.6211184384.548109851 4.841936651
4.780874519 5.496012324 4.885247966 0.6110598755.477199218
4.2511188661.938717893 3.730798163 -0.317248719 -4.509701797 2.65871185
-0.211989805 -3.713309102 -3.628683256 1.578260978 2.712067299
4.477643671 4.95601494 3.39822978 5.928028478 -0.844550566 2.750169857
4.328507458 3.48740719 3.609150228
TCGA-55-8090-01A -0.881064084 4.623013778 2.318067041 5.377456552
4.1166348988.283718662 3.380601294 4.382155313 1.77284991 3.14422434
5.304695448 -2.626902053 3.833240737 4.37855356 4.604063603
5.032943332 2.8088233 3.84370694 0.379556164 0.494170702 2.570434896
5.138979032 -1.118080886 1.876435639 4.496250624 2.934643676
5.71018564 2.197053296 4.576147046 3.228702059 4.932215099
0.899838343 -3.577162965 3.185133655 -1.066596052 2.670546116-
3.121756836 5.601888354 3.382909937 0.372026104 4.732393118-1.088462215
5.065571468 2.126468099 4.07947876 4.24979703 7.152186713 1.285133869
3.604812422 6.766245875 -4.64215968 -1.921876812 4.913338698 -2.2424651
3.160563937 -4.55267297 5.10861602 2.196448628 2.653178188 3.762193774
6.062297291 2.4699342 1.706622932 -2.158931643 4.591636629
3.935602021 3.990681976 4.359421217 -1.102383389 5.08538143
6.331983012 2.880887835 -0.047795859 5.940757137 -0.419505398
6.0411472325.1117027854.8191157775.544237899 6.29175093 10.56318601
0.809428124 2.689868313 0.830302696 -0.684964042 3.031501862
0.941627026 4.369661402 6.803338978 3.470926961 4.599302406 -
2.1189252862.11668364 -0.29329124 3.2490713113.568125622 -2.74151468 5.344839163
4.618860815 0.248679971 2.761206542 3.921034562 4.988613745
4.876090881 3.244449248 5.335549952 4.635132459 4.934291988
4.1166831240.642258272 1.792550407 4.512084686 4.80241386 4.750388751
5.531320094 4.669908984 0.785902266 5.555256174 4.047866133
1.94384449 3.994266778 -0.426442468 -3.888696503 3.068334332 -
0.045954983 -3.241729815 -3.019638128 1.890975228 2.815522286 4.45331705
5.227260362 3.180258162 5.898666665 -0.733350512 2.868179386
4.548824196 3.649940009 2.749446004

TCGA-55-8091-01A -0.804408661 4.421976834 2.595810267 5.372772915
4.232829449 8.348876563 3.3951130854.344298997 1.690176414
3.367663014 5.315286597 -2.590440079 3.419653381 4.228549959
4.6683271135.091437421 3.121343361 3.898053237 0.292738808
0.475965646 2.565428999 5.374734741 -1.218424665 2.912671792
4.504669728 2.877591603 5.641942472 2.265699401 4.532221645
3.038505138 4.867141352 0.892217626 -3.853012366 3.673806281 -
0.893263185 2.687485947 -2.877369805 5.594363696 3.540012321 0.89161313
4.722534166 -1.277296937 5.343345595 2.461870875 4.031508877
4.240759599 7.08010431 1.40063149 3.143269458 6.548114144-4.711979005 -
1.832350834 4.831475004 -2.609424078 3.205890024 -4.420083558
4.939934775 2.024163317 2.720544968 3.795144922 6.163810217
2.574047793 2.174049922 -2.226955369 4.665709919 3.934030386
4.032158487 4.176355837 -1.362315214 5.093848063 6.402106485
2.852308403 -0.101079727 5.893106461 -0.569336927 5.885213385
5.155079547 4.61071249 5.468495358 6.400349821 10.997612 0.970287209
2.542334472 0.800257395 -0.600513937 3.165703859 1.023444872
4.284852654 6.8118871043.479679629 4.631836782 -2.15698508 1.870596793
-0.153552586 3.2717599 3.807171218 -2.306202484 5.317551418
4.984682548 0.640445406 3.159554992 3.781790824 4.935826186
4.953728873 2.973563615 5.33540548 4.842335162 5.046814663
4.168929941 0.539049378 2.152963851 4.4746113654.79764202 4.793031279
5.578052077 4.994459599 0.706777929 5.549978528 4.226748395
2.21424912 3.864492042 -0.21222944 -3.894744124 3.339340053 -0.104537832
-3.367230506 -2.996364185 1.837557454 2.807437132 4.533796029
4.878537816 3.273645879 5.896632103 -0.558641037 2.972024677
4.382731485 3.552730784 3.269917027
TCGA-55-8092-01A -0.53115419 4.868851962 2.850241072 5.365708107
4.0338411928.2310811523.199742508 4.260435751 1.815579991 3.38366908
5.269927339 -2.426449539 3.382471759 4.507300363 4.666491037
5.141012991 4.254999312 3.772000237 0.163669605 0.345326655
2.741678704 5.167037612 -1.323019757 1.900646383 4.614943924
2.8115014475.79654086 2.298941041 4.469816419 3.439578037 4.774748204

0.995627217	-4.076296219	3.011673342-1.187590496	2.611820012-2.950158447
5.5884110943.009850807	0.194428075	4.700843775	-1.17565291 4.686051308
3.279661561	4.0113889854.223222008	6.920285052	1.6011114384.103545404
6.860502446	-5.08725165 -1.858538421	5.076594685	-2.609993665
3.352228089	-5.190105789	5.067159577	1.992788003	2.12866631
3.973436042	6.003903545	2.470439731	1.88411284 -2.334559185
4.644572766	3.935948693	4.14317915 4.222749712	-1.572383876
5.077443206	6.385130225	2.990563217	-0.264875592	5.676455901	-
0.806091016	6.16311354 5.182849583	4.67104892 5.465642853	6.309884214
10.480741160.416957109	2.432775329	0.40103852 -0.709987681	2.993236204
0.802938612	4.306545355	6.901378696	3.6490015	4.832071822	-
2.016502394	2.261847165	-0.423623385	3.262844787	3.810882246	-
2.243463342	5.362059021	4.354190891	1.257580867	2.955262786
4.102617485	4.989720702	4.771390676	2.912452966	5.335350689
4.967456444	4.7575801193.873499935	0.753827087	3.1986115894.559682716
4.792288737	4.734587997	5.633785355	5.236993106	0.905141374
5.475722627	4.0092110571.63205646 3.91491741 -0.844261745	-3.679348626
2.237874646	-0.370693748	-3.75406788 -4.000569206	1.325089006
2.908215928	4.292158482	5.4114645343.574955831	6.247735751	-
0.749853128	3.095853984	4.55710782 3.561586977	2.83004324
TCGA-55-8094-01A -1.197199564	4.780737478	2.1161833815.405017568
4.356760715	8.274382896	3.8820118544.1188910622.588212628	3.629989885
5.389674389	-1.909699531	3.979217365	4.98069877 4.993966046
5.002997924	4.13936561 4.841944382	0.929633449	0.57277995 2.903304402
5.04292424 -1.817044315	2.688749147	4.645445284	2.677552482
5.1196787615.232623016	4.452651893	4.135233743	4.802058901
1.461146192-3.801665536	3.795466992	-0.948411224	2.434487439	-
2.648715817	5.626834446	3.327413327	0.600436855	4.700928838	-
1.169972598	5.243031869	3.313032599	4.0011583924.210596859	7.103027077
1.709177186	4.674773903	6.930169502	-4.45404444 -1.521811657
4.787597367	-1.731815845	3.602230254	-4.202423798	5.228595834
2.43299318 2.330728375	3.930283625	5.826701994	3.253669263
2.324982749	-1.834077541	4.3990153113.936685307	4.637317595

4.461333638 -2.899810853 5.070092436 6.343055617 3.010616585
0.004088126 5.246387654 -1.192219056 6.238580433 5.140424089
4.984782294 5.509749121 6.412338644 10.74777439 0.980492068
2.861265566 1.573155582 -0.7991053 3.442856656 0.5811970724.403903207
6.828657674 3.930817196 4.993885365 -1.809308111 2.854339185
0.3511323373.403167182 3.90649912 -3.127936148 5.351819104 3.750690403
1.191064872 3.039629881 3.824865775 5.100031027 4.689856221
3.603704277 5.33545203 4.9211615544.677529254 4.677148045 0.515027317
4.183752135 4.549996764 4.5114799444.684931775 5.784093007
4.9525112681.37368746 5.082840478 3.5112230911.2821142593.954921054 -
1.248562849 -3.058879827 2.613685462 -0.098586456 -3.324329873 -
2.652809053 1.578408838 2.451553446 4.0151119275.796833536 4.123694397
6.312113047-0.585933928 2.403446279 4.8401197333.862973556 2.635042371
TCGA-55-8096-01A -0.233595072 4.829883434 2.606322919 5.3532712
4.287860124 8.235738546 3.609710552 4.239714403 2.116891303
3.376005872 5.323231319 -2.397482603 3.566877133 4.531054558
4.982129412 5.154558602 3.881737046 4.227088696 0.578681211
0.670827267 2.779391333 5.445664922 -1.129687238 2.482267424
4.586266383 3.036041616 5.671653913 2.1103481074.60969107 3.500144201
4.818721512 1.221072805 -3.576093691 3.644967808 -0.93570602
2.986900351 -2.519085279 5.584747161 3.188327355 1.513546072
4.719238479 -1.080253452 5.294413609 2.997907547 4.066096717
4.322495746 7.050368819 1.829889974 3.727322121 6.775971529 -
4.498563664 -1.653547545 4.475532156 -2.262116931 3.416889877 -
4.266547817 5.231283493 2.392303407 2.463442005 4.007925636
6.077446013 2.83784818 1.954133884 -1.896880946 4.728103014
3.936585454 4.373717389 4.361815898 -1.440044859 5.091314591
6.451892304 2.956549145 -0.098645822 5.878819014 -0.33353788
6.005605627 5.15797508 4.738461058 5.499519624 6.334430844
10.82352199 0.727892668 2.706512021 1.013261843 -0.56402374
3.448912188 0.979089732 4.271520223 6.883016153 3.699862875
4.747003967 -1.801016672 2.396695403 -0.102139091 3.309466643
3.871053229 -2.19810364 5.345798178 4.836056877 1.410416084

3.322719592 4.351554478 5.009943838 4.786029965 3.149670654
5.335522909 4.937143202 4.932606723 4.420845925 0.848233896
3.252306452 4.464245566 4.776573401 4.763820269 5.56067089
5.188487525 0.982613456 5.591564102 4.047492044 2.072830949
3.905498794 -0.263533044 -3.349256078 3.205237066 -0.01117245 -
3.334834559 -3.08209372 1.660352037 2.989698469 4.519404182 5.214239585
3.441799548 6.26666313 0.290746359 3.24225676 4.622632988 3.788341881
3.426023833
TCGA-55-8097-01A -0.710009367 4.75756788 2.85068107 5.179528964 4.217149576
8.259038366 3.6114702944.22383935 1.806706269 3.046701897 5.235172098
-2.285476425 3.244927478 4.384362489 4.7114767464.866410123 3.86513366
3.900555818 0.228313459 0.410806795 2.793296918 5.411699101-
1.320192926 1.915924208 4.563181039 2.97062027 5.73807855 2.05723608
4.519172405 3.201782517 4.930441212 0.938118655-3.592868002
3.184514776 -1.247257762 2.985434381 -3.359331691 5.577421044
3.919835626 0.902976224 4.710007809 -1.13473995 4.703756059
3.1741131323.998738705 4.195357261 6.915788671 1.777371295
4.130579666 6.806608061 -4.676687776 -1.643044312 4.320662977 -
2.498107918 3.367914962 -4.79340598 5.182852534 2.313892136 2.180869301
4.036181849 6.013718029 2.698309227 1.609239521 -2.359056732
4.71235985 3.9308661124.076760526 4.315248126 -1.692673159 5.085466386
6.445414205 3.007110909-0.055145225 5.99426351 -0.555440838 6.074143297
5.150156082 4.77931104 5.434764751 6.383160968 10.09543419
0.573133416 2.58114499 0.749066958 -0.738167918 3.233372638
0.647783492 4.281812686 6.909920577 3.581323265 4.687092603 -
1.839837242 1.978234621 -0.323110594 3.225323372 3.732599095 -
2.536246762 5.358488536 4.616020385 1.23653786 2.922930526 4.090123894
4.968170168 4.737582439 3.445628564 5.335474039 4.954914974
4.822017916 4.075172932 0.717963255 3.347464582 4.439221077
4.835359826 4.73939236 5.5803301175.138538679 1.012376227 5.566700193
3.912232755 1.877489745 3.952818487 -0.747635156 -3.836105351
2.79309009 -0.269552369 -3.448067276 -3.947669578 1.49722261 2.72994112
4.213065122 5.368256955 3.273490528 6.288669822 0.159251975

3.186361438 4.426124874 3.687849692 2.589697555
TCGA-55-8203-01A -0.86214731 4.737043914 2.27194352 5.286976887 4.204485912
8.248875511 3.672041132 4.241433009 2.066286028 3.498722468 5.167492612
-2.273385921 3.453157867 4.654230601 4.853862017 4.756954754
3.541640214 4.228588397 0.588260688 0.400950126 2.691425309
5.47391782 -1.518297185 2.139647965 4.64506063 2.717817916 5.785295083
2.42255226 4.521451692 3.778241157 4.78725882 0.857685335 -3.709674878
3.264701873 -0.960769803 2.565601043 -3.59140144 5.590484399
3.149408591 0.340293622 4.701006194 -1.123876823 5.05381383
3.086098582 3.956825163 4.330542605 7.03195439 1.537557764
4.063240732 6.805437826 -4.996464707 -1.768605709 4.578159745 -
2.05304496 3.530440915 -4.940516972 5.119648899 2.130349891 2.144414279
4.063875964 5.875095757 2.564209758 1.972529634 -2.273532246
4.616107012 3.938974388 4.076297297 4.281317589 -2.37598043
5.072682774 6.411598838 3.02155846 -0.077452384 5.836395266 -0.626700045
6.048194125 5.104743816 4.812264818 5.490556643 6.458120509
10.0722176 0.649849181 2.763480385 1.077589119 -0.886914759 3.22365162
0.710618816 4.267025561 6.86221571 3.518523159 4.802575208 -
1.980577941 2.328107656 -0.330393714 3.271092014 3.832807826 -
2.594735069 5.3520784 4.650422661 1.132728325 3.015995232 3.713799125
4.991179713 4.704311025 3.32689281 5.335385318 4.851375862 4.898423542
4.139668237 0.542477596 3.237401913 4.475038232 4.654113171
4.742784219 5.557854981 4.664041096 1.035867794 5.460127039
3.928390478 1.610922193 3.958900917 -0.561509576 -3.775971024
2.968627599 -0.300245008 -3.507538226 -3.89798021 1.254397202
2.659318485 4.047857384 5.562002315 3.535589682 6.250972268 -
0.297534177 2.392132881 4.620138839 3.748249168 2.556284155
TCGA-55-8204-01A -0.840325357 4.746434064 1.588706636 5.620404553
4.309879663 8.205664192 3.500359034 4.213148742 1.933713943
4.04165482 5.508390089 -2.359473733 3.450871219 4.406772235
4.470032541 4.943429303 4.078704353 3.999110152 0.299187247 0.39902808
2.721794391 5.149754957 -1.291276881 2.259333407 4.592472386
2.410857698 5.677578289 1.828251917 4.661699646 4.284800953

4.928234995 1.392283529 -3.747891416 3.072469615 -0.925794361
2.192231295 -3.824928003 5.60585755 3.126879398 -0.224851927 4.69178778
-1.265904801 5.385457395 2.660671993 3.832477775 4.27517373
6.931860034 1.085226071 3.495353771 7.121892562 -5.727452552 -
1.775244033 5.07798574 -2.745187136 3.405467884 -4.900019272 4.912542207
1.923399832 2.646240664 3.860507532 6.008255216 2.360407736
1.794189114-1.686784516 4.628219003 3.929803789 3.986442316
4.262069705 -1.54564081 5.088224092 6.414398648 2.715627715
0.079806761 5.648184741 -0.555552439 6.032500026 5.068325974
4.554473645 5.468309938 6.226551797 10.97158094 0.899404337
2.586849684 1.007500091 -0.387813961 2.928366825 0.992519274
4.32178624 6.813866964 3.356402727 4.937675843 -2.160851612
2.371346621 -0.624652419 3.337166594 3.784547952 -2.352717653
5.347779645 4.592789741 0.510359134 3.027269625 4.068176225
4.939751775 4.789557825 2.843028821 5.335291828 4.658249215
4.964251917 4.19610682 0.652853624 2.006697128 4.430899322
4.708365679 4.768775244 5.652896689 4.89756874 0.889942759
5.036595068 3.627677484 1.774335537 3.705766812 -0.556330977 -
4.649537455 2.637375243 -0.60066453 -4.003929818 -3.161297941 1.408979227
2.634454841 4.472607435 5.071528743 3.327992808 6.002875884 -
0.763564763 2.396977663 4.373898825 3.578141295 2.83617618
TCGA-55-8205-01A -0.079542707 4.780482247 2.461394407 5.440198103
4.048864479 8.212516405 3.3189901154.387428755 1.408809484
3.639235045 5.589000794 -2.628037913 3.424678351 4.815217411
4.990712072 4.775294922 3.507441776 3.295252683 -0.097850237
0.448322991 2.439175571 4.972350463 -1.316155988 2.450588654
4.549417196 2.553388318 5.928346595 0.312515135 4.517759276
3.69304097 4.799354288 0.920156385 -3.436737431 2.881633402 -
0.915623383 1.718698969 -3.953007169 5.602166678 3.180415855
0.186985215 4.710632503 -1.052877537 4.646742558 2.506842777
3.894517197 4.20792248 6.899369785 0.937561591 3.653706831
7.034779888 -5.479055242 -2.180225621 5.109824737 -2.422710044
3.349538525 -5.047186194 5.1199158211.825372554 2.43863798 3.658852539

5.954587023	2.243208881	2.280330667	-2.416371101	4.802895153
3.945524428	3.387040178	4.494378727	-1.713664933	5.088515251
6.206703512	2.609519535	-0.060511103	5.898716826	-0.266422769
6.131937787	4.9511961284.716386145	5.501828949	6.227952176
10.61476007	0.322013068	3.036748751	0.458403952	-0.543087934
3.421913857	1.1834411424.2662119686.831947438	3.141227884	4.997338698
-2.054547345	2.453545932	-0.62083188 3.23461998 3.811336964-2.432671639
5.34890033 4.34360542 0.767955692	2.921273697	3.923837188	4.984974398
4.84740227 2.68002901 5.335573292	4.394556281	5.064106852	3.813408831
0.9211560031.618101286	4.447595072	4.923131023	4.790954585
5.266512478	4.3809489110.723204762	5.389536128	4.369328592
1.942025307	3.768755963	-0.33875037 -4.459749497	2.637978958	-
0.540897078	-3.868071512	-3.744517075	1.376859869	2.709274273
4.399407454	5.129139924	2.988451786	6.170543332	-0.50477677
2.400963251	4.437382375	3.624274163	3.20706021
TCGA-55-8206-01A -0.122254453	4.788864709	2.978497502	5.211109677
4.298056304	8.293098359	3.57460184 4.22183581 1.900417734	3.247265996
4.861201104-2.298847848	3.313712232	4.481559706	4.8783641134.969769388
3.8345189113.94063235 0.999746039	0.592102801	2.728879661	5.475979897
-1.154274566	2.357421692	4.627931745	3.150291289	5.79801007
2.303627053	4.536717929	3.345636474	4.889865423	0.983916951	-
3.56977313 3.286711958-1.204391112	3.266756517	-3.116418808	5.572819594
3.463971391	1.78926206 4.707382991	-1.203192727	5.260705266
3.364717151	3.989400453	4.273825333	6.889171432	1.980323858
3.972519905	6.76474492 -4.47027603 -1.633768654	4.518274987	-2.422837614
3.499218483	-4.658546462	5.096751502	2.2161116632.536533877
4.213255434	6.0591117142.818248976	1.470282516	-2.051681249
4.802648372	3.93548807 4.081295332	4.250219653	-1.355705663
5.089829489	6.436255299	3.017833938	-0.033702456	5.890760708	-
0.150400415	5.962733281	5.182512591	4.582437419	5.359919505
6.450296885	10.44672299	0.86861916 2.71793051 0.851341026	-0.754413231
3.590046561	0.839919461	4.222763759	6.929959056	3.649033252
4.637488614	-1.732317662	2.026077527	-0.139633174	3.223970754

3.868935632 -1.927602825 5.35485076 5.040378567 1.489849756
3.1157224374.199358748 4.928337827 4.750125486 3.277989889
5.335436995 5.186530656 4.921012717 3.95829629 0.728836787
3.668510012 4.497396838 4.789593473 4.788955289 5.555140012
5.275792805 0.934013916 5.5623411764.403733216 2.196921388
3.878200786 -0.218331802 -3.526399835 2.996531923 -0.272610397 -
3.351210416 -3.674155061 1.606704926 2.970834656 4.313068849
5.200582987 3.335493109 6.32013112 0.364530372 3.142842343
4.460424231 3.693546875 3.246742379
TCGA-55-8207-01A -0.945594346 4.5094391192.500902898 5.452634692 4.19564934
8.3488116183.560280038 4.293279196 1.781642416 3.35384365 5.203163958
-2.326322658 3.318793582 4.342004105 4.685138905 4.959733055
3.235046579 3.9078151170.669489819 0.461421568 2.570744926 5.5810265
-1.471904187 2.404510798 4.551745677 2.990561795 5.6165606
2.241241224 4.510907053 3.454348762 4.909061746 1.103645671 -
3.867453277 3.77088936 -0.945336395 3.047377641 -2.805791897 5.588185195
3.844829696 1.0485648 4.716393365 -1.239568442 5.385157745 2.8001924
4.026902175 4.255510841 6.919583484 1.564838477 3.051391959
6.424579902 -4.577579035 -1.690926514 4.473360711-2.556549684
3.276910938 -4.328996752 4.937791018 1.966596257 2.37755206
4.042535931 6.056249634 2.7711605851.997280927 -1.72315057 4.616082559
3.933882723 4.090420624 4.110441967-1.909441243 5.091509729
6.387033002 2.939799094 -0.014751406 5.956864017 -0.586990409
5.908921442 5.19018884 4.571506932 5.445026425 6.428344642
10.89727838 1.092074275 2.517280695 0.856559212 -0.68795524
3.036844329 0.853253762 4.315971992 6.883603271 3.592193829
4.451652978 -2.060087855 1.964777114-0.288005791 3.298730712
3.781781044 -2.57852856 5.330500999 5.036269066 0.9511124483.09158264
4.071659553 4.92409413 4.827576306 3.078628655 5.335440959
5.203907424 4.89674667 4.202257543 0.406840224 2.722278002
4.388026126 4.701315691 4.776555749 5.556448846 5.171474629
0.803648659 5.606540709 4.073491084 2.085172145 3.815744362 -
0.497769386 -3.780306198 3.059237681 -0.198333226 -3.285313147 -

3.289997288	1.744732983	2.695716621	4.210007756	4.785405357
3.304702327	5.795568751	-0.036032294	3.188181694	4.303235774
3.629384353	3.080826945
TCGA-55-8208-01A -0.420527911	4.795141766	2.616899956	5.37778781
4.175215845	8.272802791	3.644910013	4.314900454	1.739887013
3.723834837	5.423716363	-2.320685714	3.619953253	4.445833718
4.897924622	5.021353707	3.984637026	3.98133885 0.255902763
0.464028528	2.753741723	5.206841175-1.198070275	2.868815835	4.5585888
2.843652568	5.874018915	1.629163837	4.602220656	3.708769127
4.882332929	1.087198296	-3.534241785	3.439230699	-1.026315068
2.356312613	-3.270523827	5.589184107	3.187301319	0.488840869
4.705030404	-1.179146346	5.293210779	2.867231	3.989023558
4.336393896	6.948412941	1.438866874	3.484669436	6.964728514	-
5.011162529-1.832457389	4.593484391	-2.605119445	3.506633053	-4.873482621
5.032065031	1.817729776	2.187927404	3.992870847	6.184168483
2.759103408	2.37375433 -1.914521646	4.738162078	3.93028061 3.890100543
4.322124758	-1.511848398	5.102624218	6.408932879	2.826310571	-
0.094852566	5.984135665	-0.248926738	5.989874039	5.077294676
4.586862343	5.519007189	6.265761842	10.61065998	0.859004724
2.67967786 0.568198894	-0.645140826	3.860334757	1.028453604
4.299835689	6.8110564943.543041589	4.8384305	-1.956936613	2.257234692
-0.467625846	3.298331925	3.889299665	-2.262848281	5.339540949
4.826159738	1.467438075	3.381849907	4.101601071	4.961698686
4.688016401	2.890594005	5.335570338	5.045871276	4.998100006
4.257143237	0.742243936	2.835335287	4.661703407	4.793178867
4.778688899	5.651470232	5.343590086	0.980318509	5.664313859
4.048488657	2.073025553	3.79652726 -0.519588402	-4.199679212
3.005833567	-0.478752455	-3.696871258	-3.56002583 1.658015769
2.855632679	4.504494667	5.125889844	3.387157273	5.984928319	-
0.361003424	2.921202833	4.406684922	3.685015851	3.40594414
TCGA-55-8299-01A -0.502733951	4.612957943	2.909548416	5.464390871
3.798628292	8.317554257	3.445084724	4.335036432	1.636813358
3.627774916	5.343448596	-2.384836539	3.462667541	4.320918735

4.6686479114.826057887 3.513853847 3.718451923 0.5911362430.440522486
2.559072678 5.221394633 -1.415933835 2.613232418 4.558087074
2.917583499 5.826373158 2.148840021 4.463035274 3.134274765
4.830766635 0.996880541 -3.765636525 3.490292672 -1.132914549
2.357979882 -3.030311864 5.584139395 3.067438227 0.441056089
4.707459563 -1.214626245 5.1195889492.7271148324.028882972 4.281412492
6.81240342 1.331407083 3.216906523 6.837709779 -5.156245004 -
1.828719972 4.758763188 -2.674369457 3.359573482 -4.776921979
4.9449511881.923003386 2.328424961 3.976048132 6.1117487772.498104044
2.198512064 -1.717427092 4.746179545 3.934509827 4.006124823
4.219926486 -2.169221568 5.095027902 6.412710318 2.789912325 -
0.131446337 5.949725034 -0.517578407 6.003462083 5.081908132
4.559594717 5.491898778 6.323071333 10.90162296 0.70772366
2.528792369 0.495770398 -0.416222366 3.500981231 1.110863997
4.2685119456.867707004 3.408708549 4.654378466 -2.098202233
1.981799696 -0.507278816 3.253507804 3.949683468 -2.429918214
5.336671222 4.863060256 1.058686978 3.079690007 3.905251618
4.954374568 4.768817776 2.656645997 5.335544962 5.058190593
4.987325764 4.015646252 0.686083904 2.265980241 4.635011288
4.797294172 4.769398212 5.478315312 5.082696503 0.878281227
5.526224515 4.335123618 1.9678601163.73071403 -0.313753133 -4.157244143
3.021122995-0.506335087 -3.684146421 -3.520066987 1.599794103
2.818218971 4.469666665 4.76814443 2.818422753 5.940709314 -
0.639852872 2.912159126 4.202601935 3.61723236 3.128868212
TCGA-55-8301-01A -0.132561174 4.921059083 2.648931592 5.436266248
4.0644133 8.219076483 3.670333181 4.281769789 1.821065459
3.564438126 5.330658791 -2.205540891 3.58235806 4.542928418
5.0681142624.825185762 3.86116257 4.204341412 0.1156975050.45949357
2.684289375 5.112449102-1.189572019 2.65457367 4.600559122 2.850180821
5.851958223 1.982794298 4.615929099 4.086547846 4.948710157
1.255203351 -3.481714441 3.417954303 -0.840215105 2.478714771 -
3.376264108 5.595968357 3.210512416 0.637229342 4.709425593 -
1.1011083975.1503477113.039223687 3.910722544 4.278239165 6.860858278

1.5068707113.824097938 7.081149561-5.085720524 -1.821279069 4.829615933
-2.482294486 3.375027129 -4.617532664 5.132624089 2.145558674
2.328425498 3.920045717 6.02302422 2.60386651 2.35026883 -1.668626429
4.7514116453.935328322 4.125984579 4.338400921 -1.564701698
5.096250837 6.474064852 2.770647499 0.019183767 5.864397857 -
0.397958123 6.085312912 4.977653188 4.805540866 5.4981114716.330211577
10.60499894 0.770401762 2.857867556 1.009238657 -0.534530337
3.537633204 1.014790897 4.319740264 6.928199018 3.447388072
4.928001009 -1.932828066 2.51568347 -0.439522923 3.321234032 3.98294619
-2.158609689 5.345380208 4.472780606 1.298356839 3.194289859
3.61225164 5.040184044 4.765533056 3.065404086 5.335714095 4.74545345
5.0142103 4.348653365 0.825734146 3.082175621 4.623721555
4.859683782 4.76349788 5.650619983 4.928507537 1.174743089
5.401346985 4.167608244 2.000949853 3.859325039 -0.74880751 -
3.906427763 3.056891096 -0.223852168 -3.807443789 -3.541767541
1.480051248 2.926014861 4.46776878 5.291539584 3.201437122
6.174093216 -0.164046091 2.741881497 4.440194232 3.681089902
2.899977025
TCGA-55-8302-01A -0.675147001 4.972834251 2.818338712 5.381829957
4.408810744 8.140605263 3.396928892 4.309285198 1.694432935
3.066365563 5.387410934 -2.575873955 4.231214005 4.417499148
4.749473296 5.351717152 3.36532467 4.127239071 -0.001309446
0.472785861 2.707326224 4.693502939 -0.997357112 1.907979602
4.502000235 3.156016204 5.58268435 2.906489866 4.676627701
4.225241239 5.156460891 1.102444869 -3.351212597 2.910892099 -
1.061482223 3.02908969 -2.853640071 5.630506961 3.272820993 0.687507837
4.718560485 -1.077003948 5.187929693 2.278229917 4.045706554
4.058956592 7.051776659 1.391747301 3.981020885 6.792837305 -
4.527639271 -2.032801579 5.40151988 -2.235332293 3.161740729 -4.815805363
5.235167759 2.2311574632.536232583 3.368868534 6.041336054
2.662321254 1.832642784 -2.089426096 4.616654846 3.928272687
4.003924203 4.480831477 -0.75466033 5.093016084 6.276295866
2.833468086 -0.05642408 5.476368385 -0.497566865 6.172825446

4.934225078 4.9547980115.526282071 6.140529925 11.012618090.615432437
2.761554682 0.646334899 -0.566092256 3.4114728671.057368906
4.457702848 6.835552701 3.528132349 4.878569977 -1.972288961
2.600811295-0.159597224 3.261674607 3.603630465 -2.479631508
5.358147055 3.883470436 0.209366248 2.588805292 3.875168914
5.072575255 4.9583308 3.329363962 5.33567218 4.421321612 4.942742855
4.216167902 0.820002656 2.316220232 4.683981589 4.980581968
4.719319241 5.741304831 5.073933052 0.954303779 5.440115427
4.024807606 1.966106642 4.010183615 -0.865986748 -3.825544828
2.34411515 0.28911087 -3.533071611 -3.382606651 1.98830386 2.964894531
4.630914424 5.348563851 3.664184335 6.230088674 -0.865265818
2.839852679 4.5511734593.597771467 3.080482438
TCGA-55-8505-01A -1.004414512 4.86002135 2.975424342 5.430188643
3.918246245 8.296695784 3.082332543 4.289293368 1.712413612
3.304129863 5.746028652 -2.558097581 3.484159021 4.382859429
4.52468875 5.323828835 3.871208084 3.459960429 0.136381032
0.321945563 2.788427034 4.573239221 -1.335690653 2.434873172
4.522934831 2.885406178 5.643261731 2.718941659 4.514052636
3.076344065 4.931720682 0.886744764 -3.802996082 3.287253416 -
1.481510255 2.008092802 -2.895801606 5.601665429 2.955124264 -
0.2112298884.703542111-1.258789166 4.634649759 2.696292699 4.063547811
4.247952846 7.01440197 1.077048565 3.45057531 6.729546878 -5.162395152
-1.961183168 4.972060838 -2.709088009 3.108863172 -5.173552078
5.120337482 1.7111092271.751888493 3.622302253 6.099275563
2.447706676 2.275368577 -2.139872326 4.614904299 3.928641421
4.069973555 4.271071142-1.947171639 5.087052377 6.387715202
2.951059206 -0.389110798 5.760793809 -0.901435895 6.141041217
5.087060508 4.734079975 5.596809576 6.104815657 10.48547954
1.488837725 2.401830988 0.100024254 -0.597076333 3.106564049
1.065193967 4.38215238 6.830672697 3.529794365 4.755355378 -
2.273660769 2.040503346 -0.603622816 3.250494251 3.694450551 -
2.509010823 5.348621943 4.01182605 0.834537019 2.654518976 4.231213298
5.00540348 4.596716549 2.799885539 5.335445483 5.08701342 4.904662889

4.1221142590.592362814 2.496919222 4.623478824 4.818006544
4.716870095 5.753940275 5.204853149 0.9401147245.713472819
4.275750508 1.704622559 3.814129292 -1.185733454 -3.945010592
2.395861821 -0.424288736 -3.615630053 -3.94094067 1.700991523
2.820643421 4.538323071 5.073670848 3.142709439 6.089285517 -
1.461592677 3.016328549 4.371865001 3.650699039 2.794913999
TCGA-55-8506-01A -0.154359171 4.953969875 2.156509367 5.412009401
4.649449058 8.20644077 3.365337823 4.229516478 2.028329387
3.382713267 5.388167226 -2.10624945 4.016074905 4.57967837 4.958059132
5.401843192 3.672707714 4.258303925 0.274099575 0.594867121
2.954010941 5.175285975 -0.634005945 1.972424128 4.578052213
3.205066988 5.308853817 4.318835132 4.73505383 4.157543747
5.069469041 1.260427125 -3.335862709 3.306378284 -0.696717731
3.093747631 -2.554438446 5.608447083 3.488464745 0.994959129
4.711639801-1.096193322 5.512972948 2.664627651 4.09131777 4.286965921
7.075976741 1.707335086 3.899491752 7.141837268 -4.493802899 -
1.708317861 4.912978841 -2.40265938 3.474201343 -3.882500706 5.245959632
2.937736396 3.093954906 3.928808271 6.12507102 2.879715904 1.93530271
-0.945376066 4.566177452 3.932557696 4.604485996 4.366385687
0.023149809 5.078468328 6.410939688 2.896375651 0.041530915
5.53405638 -0.35631643 6.151606632 5.036226137 5.003799313 5.543607641
6.313432287 10.63631099 1.227525235 3.001719358 1.051351867 -
0.346653842 3.4522031 1.049808034 4.437663202 6.869447262 3.662407235
4.917961735 -1.838540899 2.800320257 0.382322522 3.31685626
3.686141584 -1.656404904 5.34801524 4.291113411 0.781824872 2.958973955
3.966413855 5.055744868 4.844214276 3.480635685 5.335537715
4.721497173 5.079289984 4.52245988 0.866313498 3.1636668 4.677340996
4.901952924 4.713776318 5.787041272 5.083909256 1.07306626
5.688868634 4.371615005 2.193000945 4.090407724 -0.707138624 -
2.823786899 3.232547922 0.224068735 -3.465032984 -2.657660952
1.745476707 3.20733771 4.725295145 5.437691498 3.430932175
6.258432695 0.18001262 3.06501583 4.688862173 3.730904215 3.148345528
TCGA-55-8507-01A -0.85983327 4.805101215 2.169409093 5.218537082

4.270236585 8.210023423 3.73219091 4.13119607 2.165153402 3.53581599
5.127410883 -1.983498706 3.50487686 4.741880418 4.9269948114.967262011
4.292526978 4.334327522 0.374371926 0.435577625 2.89740625
5.399717372 -1.440716063 2.057292562 4.674364106 2.758562119
5.604674203 2.72923443 4.599491255 4.22197911 4.968399559 1.304287859
-3.396367344 3.126401852 -0.881341814 2.724823595 -3.667098249
5.59699935 3.328716717 0.51861661 4.694648126 -1.126739019 5.189904099
3.454534083 3.90165487 4.237832213 7.009900144 1.780400502
4.260849172 7.160588819 -4.897513848 -1.681684134 4.614167893 -
2.213766346 3.65101251 -4.777210462 5.252097559 2.145709939 2.505828009
4.1114577935.900285417 2.73812448 1.895396224 -1.535258335 4.626000132
3.928734813 4.20593798 4.34556254 -2.251987809 5.077721859 6.569049837
2.951810924 0.075014407 5.563583471 -0.602633636 6.15181977
5.008825064 5.058164636 5.484684363 6.386171878 9.858617463
0.600729323 2.946664137 1.128530571 -0.782878265 3.48230246 0.62208656
4.321642257 6.900612233 3.522639203 4.895932905 -1.695958383
2.401161484-0.04730526 3.307580585 3.857475329 -2.671968771 5.353170422
4.437658768 1.514689378 3.1081145553.836598022 4.99062313 4.690606263
3.617139695 5.335507313 4.783406431 4.885359509 4.231332713
0.713556472 4.021070921 4.572304791 4.71441053 4.70527858 5.69555591
5.145520094 1.321088147 5.32930336 3.791448158 1.618774312
3.943018447 -0.801874433 -3.473668444 2.915149307 -0.384617096 -
3.684744257 -3.750899012 1.327404741 2.639075635 4.103499388
5.598026619 3.463710247 6.282070741 -0.152565952 2.555137816
4.578533634 3.8162117012.783011873
TCGA-55-8508-01A -0.603319951 4.980322785 2.468152171 5.2939113614.13532646
8.241453286 3.603516759 4.216012657 2.23356443 3.483548297
5.318950494 -2.140600733 3.4611627514.5211696474.903707834 4.969404709
4.013023474 4.335728548 0.483863131 0.450213036 2.940068128
5.326704353 -1.33611016 2.199140423 4.6411842792.743742592 5.714972813
3.288348367 4.607122867 3.71417205 4.972356318 1.327729511-3.627789926
3.393105242 -1.092979124 2.744698766 -3.066133605 5.592492455
3.169254635 0.133327049 4.708188856 -1.069356507 5.026743604

3.361472653 4.053379762 4.33239283 7.031356746 1.8112750754.310720627
6.97216154 -4.943763113 -1.503196619 4.661893926 -2.187503753
3.361033523 -4.523797671 5.298198978 2.279033073 1.917771852
4.094426845 5.97382876 2.640463969 2.016330288 -1.536166803
4.629556196 3.929745047 4.520155929 4.243250766 -2.116694442
5.078779399 6.623641454 3.034909952 -0.08902851 5.767718305 -
0.551944235 6.161695754 5.1000748115.064170002 5.49763542 6.423590112
10.24453386 0.775216527 2.647555031 1.143862172 -0.599739911
3.290646603 0.72528571 4.291765627 6.939863096 3.603698687
4.769474495 -1.875547085 2.376411066-0.2826414 3.329617518 3.963352881
-2.496550532 5.352807914 4.448642312 1.415427357 3.099022434
3.882371706 5.056005021 4.702873232 3.392333864 5.335631515
4.948416402 4.86829383 4.307463305 0.716516653 3.57117646 4.548728086
4.745238996 4.703147022 5.63679868 5.012523751 1.14951185 5.479212255
3.870800358 1.61761885 4.007929407 -0.406324938 -3.433715094
2.951754449 -0.471673084 -3.63946222 -3.550936586 1.318462492 2.87814257
4.201802463 5.641763347 3.451355983 6.299390195 -0.476077895
2.827429551 4.584159285 3.825804466 2.601567849
TCGA-55-8510-01A 0.181036355 4.927553651 2.694921743 5.266776464
4.210097684 8.2114714313.429450774 4.273330836 1.85883681 3.357115261
5.28415912 -2.358431101 3.802336796 4.375820768 4.940157521
5.155132569 3.658653858 4.021395627 0.242087784 0.520281809
2.823269896 5.136741309 -0.884306077 2.204698271 4.57670078
3.237604406 5.657012199 2.566487056 4.670283144 3.714905562
5.103498579 1.1892323 -3.368609783 3.158521794 -1.097893986
2.751122756-2.815162782 5.58998756 3.206890836 1.195824417 4.716116681-
1.026753106 5.160323108 2.802033547 4.060379019 4.254144468 6.9178434
1.677476892 3.652666144 7.031084647 -4.600125452 -1.807709927
4.61628844 -2.540955177 3.461236537 -4.581177163 5.228881562
2.321598977 2.49144309 3.924002848 6.125866957 2.670827636 1.80439412
-1.54509368 4.726125213 3.928495305 4.146338704 4.28966665 -0.854059036
5.093806358 6.456377349 2.857942824 -0.027098263 5.756479962 -
0.138435183 6.071695322 5.0057116954.806346216 5.505838823 6.220781187

10.53485838	0.982468155	2.742049103	0.597980951	-0.559902359
3.518170956	1.003263031	4.335315218	6.93563335	3.501796749
4.786464173	-1.864035862	2.375929042	-0.242932773	3.29479893
3.849617342	-1.905895071	5.347130516	4.538280798	1.229020401
3.029273101	3.959922834	5.043153071	4.6813711863.248887001
5.335657792	4.897946527	5.029988479	4.174415427	0.937824259
3.092370251	4.65223422	4.957536043	4.748039496	5.650491666
5.257234168	1.107275952	5.661527957	4.290071765	2.279095411
3.937460488	-0.623451385	-3.591260304	2.947800952	-0.214615179	-
3.5355088	-3.453418848	1.69163886	3.066132903	4.569232104	5.158926895
3.189926096	6.215515276	-0.016907133	3.090220425	4.453147322
3.724914068	3.220857227
TCGA-55-8511-01A	0.014865913	4.950725231	2.72471755	5.340107353
4.543608955	8.15581856	3.1271128214.218969832	1.633314908	3.059343243
5.197350743	-2.552549395	3.628153302	4.252937703	4.790166341
5.153475827	3.661804813	3.734585783	0.068244999	0.551672208
2.589367705	4.887340211-1.028556556	2.170035536	4.526829854
3.104794556	5.193598967	1.634448848	4.547481679	3.635128078
5.14827147	0.849506622	-3.581773203	3.167739309	-0.935162973
2.685771583	-2.896060693	5.612707427	3.4471152041.010478032
4.706275856	-1.150733146	5.093963299	2.897619438	4.050924592
4.208623076	6.867357216	1.494734458	3.656103576	6.954428745	-
4.897740422	-2.089431641	5.032758151	-2.551221113	3.15300143	-5.375156271
5.191028019	1.872013376	2.08498662	3.763522161	5.990353512	2.47274683
1.930230972	-2.504952734	4.7345991	3.931959297	3.710025749
4.337725695	-1.314053873	5.088140791	6.278785607	2.836471135-
0.104201497	5.456100287	-0.522201721	6.193347283	4.940051514
4.793601714	5.547074172	6.295340781	10.84772213	0.966912194
2.6955483	0.385517564	-0.620516006	3.485083552	1.080352938
4.310205527	6.872382689	3.439046784	4.869127752	-1.751511677
2.308751627	-0.626337613	3.22263306	3.739824894	-1.908770554
5.354370239	4.192726087	1.145345128	2.84442151	3.681372174
5.039446731	4.866085069	2.895231283	5.335596584	4.564627622

4.964096552 3.772565669 0.909654534 2.938674748 4.675239087
4.920389599 4.725988715 5.587009274 4.811110878 0.76344182 5.508981863
4.315608353 1.955959087 3.900379595 -0.53408413 -4.472630981
2.083113766-0.203698401 -3.638835426 -4.014524169 1.559366228
3.072424105 4.626752878 5.156820346 3.322794344 6.25927046 -
0.1165397752.9727114274.32830213 3.6411625643.158587677
TCGA-55-8512-01A -0.139710188 5.070804708 2.913077323 5.154029018
4.062041235 8.206270767 3.709344803 4.172739123 2.152407927
3.388397697 4.603582127 -2.171073232 3.214974176 4.283479183
4.744704153 4.709134793 3.598891543 4.207707562 0.304616686
0.33730695 2.984286468 5.392261727 -0.957276968 1.584563009
4.602540305 3.017254577 5.662044582 3.5311773374.613188701
3.205736715 5.001933662 0.978543757 -3.504519615 2.867099039 -
1.530738144 2.893387405 -3.521973729 5.572010348 3.527670616
0.484742497 4.716580696 -1.018021149 5.049261436 2.931599865
4.060672601 4.272525929 7.095739144 1.708722308 4.260989569
6.878730584 -4.858028126 -1.440077598 4.411826019-2.548459836
3.562207477 -4.439837369 5.177333224 2.388219909 2.351055433
4.141930451 5.966476831 2.717866203 1.172980563 -1.127836346
4.66793751 3.924912165 4.22038731 4.154300822 -0.869050283 5.087250431
6.561804087 3.066029037 -0.044139088 5.86906392 -0.030661651
6.010932923 5.19264866 4.781022872 5.393889099 6.322839135
9.896539375 0.714135577 2.607145427 0.994986991 -0.656048462
3.24565991 0.657239484 4.247927046 6.945132492 3.599615288
4.478797469 -1.969889618 1.9986699110.086079996 3.242162805
3.771428517 -1.960141054 5.355160996 4.830389771 1.182837339
2.7611664484.1135267174.98181136 4.719323963 3.533733076 5.335451306
5.120207646 4.867304373 4.332932101 0.753329644 3.110391739
4.609476425 4.865123609 4.764034437 5.662453686 5.129691119
1.074335142 5.369034679 4.37548102 2.181394995 4.036953295 -
1.003201861 -3.673758023 3.139077079 -0.40138011 -3.606383221 -3.661744779
1.458893157 3.042402893 4.19654622 5.490757478 3.366845347
6.339361758 -0.83847711 2.896569448 4.573515847 3.868783153

2.502439597

TCGA-55-8513-01A 0.062136387 5.008833059 3.1117631985.18558846 4.066172206 8.2771126873.5382436114.289217618 1.819634842 3.1811576965.09722138 -2.460791581 3.546523081 4.241296885 4.918213082 4.944531419 3.623805846 3.845488219 0.610768171 0.550705266 2.739459993 5.259555725 -0.902214839 2.263403401 4.510846642 3.279439206 5.857222456 2.167604393 4.57658894 2.809561229 4.898476855 0.690548836 -3.499061406 3.308313277 -1.477401874 3.00786984 -2.884098957 5.567777941 3.533051601 1.197512751 4.724913527 -1.097493121 5.019801512 2.797527775 4.143650391 4.318793414 7.025412977 1.746941788 3.78773105 6.69247224 -4.433462544 -1.767261272 4.37694506 -2.450473865 3.339894594 -4.764146606 5.163141687 2.10856664 2.008924479 4.073369897 6.186687917 2.884426192 1.42837544 -1.912749976 4.777993697 3.927171585 3.897402405 4.244311184-0.808435178 5.1040348 6.42532115 3.040051461 -0.229857567 6.131294027 0.15962696 5.968185216 5.251568352 4.506464245 5.444232509 6.158025499 10.3836616 1.010342604 2.555066666 0.598520834 -0.694907281 3.681897671 0.894216078 4.267875023 6.890343809 3.681172251 4.497597857 -1.871653577 1.914726263 -0.3312974 3.1990491113.677509333 -1.90941969 5.34892256 4.954314524 1.3633711453.15017711 4.339116522 4.952913752 4.743961376 2.923582003 5.33556803 5.29543014 4.860957586 4.141813607 0.791360882 3.065737239 4.659280543 4.86325925 4.793312761 5.662658054 5.384684094 0.96874888 5.566553369 4.626889247 2.460268032 3.907417753 -0.509802891 -3.749759163 2.823683777 -0.212009315 -3.307431292 -3.575970067 1.841044358 3.168104487 4.502305108 5.13332996 3.33447891 6.136592374 0.007604847 3.3505648114.4350983113.830205825 3.262161389

TCGA-55-8514-01A 0.777430538 5.207515373 3.080679444 5.324764342 4.492414971 8.122865166 3.350283254 4.212680154 2.18533894 3.126219909 5.136107744 -2.053714045 4.061983851 4.359401836 5.053051632 5.403276886 3.909961822 4.3616111490.645475662 0.63258806 2.9166998115.353667238 -0.690579008 1.662903804 4.6114757153.487252857 5.536942559 3.902164107 4.678548507 3.772829662 5.165765039

1.353962599 -3.285346001 3.227281868 -1.100750993 3.275454661 -
2.379129933 5.605831727 3.153499238 1.364533457 4.711258088-1.093275994
5.230550376 3.0446511484.146516051 4.283724308 7.008750274
2.0118964034.414294322 7.232736835 -4.199283935 -1.581331934
4.762375823 -1.948084091 3.580325905 -4.393764459 5.324672487
2.806571736 2.620916872 4.022528085 6.088397484 2.521923549
1.466458209 -1.067679161 4.739370377 3.928134847 4.465385095
4.323590891 -0.913169552 5.087780403 6.467147555 3.006760734 -
0.057688314 5.657090778 -0.294614861 6.243250178 5.038129239
5.056274343 5.523493283 6.32497799 10.51064066 0.61628507 2.886573326
0.973022537 -0.562832722 3.360088892 0.948638789 4.384333528
6.938043452 3.726777803 4.817157947 -1.638904877 2.57533564
0.057002752 3.298976945 3.785044903 -1.584510123 5.357779521
4.335463934 1.296858961 2.809254877 3.9118231815.11481515 4.796564232
3.528176296 5.335731523 4.879173234 4.893323968 4.347694723
0.974388438 3.499212619 4.800940312 4.91148829 4.699043194
5.695878312 5.290815947 1.331658175 5.402776686 4.410205719
2.064178595 4.040916474 -0.529411455 -3.058040505 3.023741361
0.016738673 -3.333919655 -2.906132411 1.788581887 3.455609318
4.700156801 5.531298196 3.440146389 6.560255574 -0.143159623
3.205976098 4.671025337 3.904805714 3.456157181
TCGA-55-8614-01A -1.218056101 4.679725958 1.040500408 5.278159015
4.300273409 8.233358418 3.733494548 4.081964245 2.04567182
3.591322489 5.189684134 -2.383063878 3.250628013 4.269696595
4.567884965 4.943412391 4.229448968 4.212584231 0.106886311
0.392627089 2.79441162 5.130644603 -1.3170395 2.084529935 4.63162474
2.59470448 5.5909359111.831347156 4.693317849 4.243389788 5.078176279
1.459351723 -3.712268275 2.938213531 -0.872184886 2.802072345 -
3.530003959 5.593936607 3.537772619 0.904746599 4.700903493 -
1.23124847 5.39292227 3.028642584 3.788159792 4.14364562 6.930483949
1.653430991 3.687239554 7.119505205-5.010893214 -1.613048899 4.98586716
-2.837553364 3.308466907 -4.67818801 5.143462577 2.078547503
2.521074499 3.999082914 5.941001014 2.50964749 1.358358275 -

1.830956138 4.693009604 3.916641685 4.165338051 4.149801256 -
1.624893471 5.086412222 6.604576573 2.837530565 0.206322504
5.327996102 -0.842071204 6.036610612 5.040052642 4.873967674
5.376308252 6.308815433 10.60903883 0.912046858 2.687492694
0.944123622 -0.508949876 2.9869911390.702934229 4.343363893
6.924750934 3.4121611964.890647633 -1.94379793 2.121708419 -0.668456567
3.336929848 3.837412211-2.465831059 5.3328211094.528745139 0.879133715
3.14441905 3.881032079 4.927397564 4.778800018 3.429395179
5.335396355 4.755313946 4.941634976 3.940783825 0.794290467
2.983435352 4.273168564 4.813722203 4.732831988 5.806597873
5.12948213 1.187787508 5.1144464633.537041381 1.805008448 3.876139373
-0.938757134 -4.263319826 2.930593857 -0.261900468 -3.762253556 -
3.488191001 1.330295194 2.570404598 4.214708421 5.092289138
3.275145968 6.16766393 -0.016101186 2.592690149 4.300204982
3.576694629 2.890948669
TCGA-55-8615-01A -1.06899882 4.828341858 2.65622179 5.212200517 4.003511527
8.333060732 3.839662935 4.2321197092.276528371 3.121796979
5.318919338 -2.165310225 3.414926498 4.476151247 4.778104973
4.7713115113.8049811254.593865101 0.102623879 0.477956861 2.895126136
5.248799755 -1.382645655 2.376746688 4.622975732 2.860271551
5.553636583 2.749253135 4.606508775 4.1137757024.8918113581.174709865
-3.403634187 3.094924876 -1.127291864 2.448256213 -3.497851289
5.587164177 3.341362147 -0.066107856 4.704473784 -1.106658038
5.202315944 3.098575636 3.962835048 4.291098544 7.057318575
1.447829509 4.31605577 6.847842319 -4.870473873 -1.619475618
4.527211841-2.309858345 3.487838826 -4.508728716 5.188287171 2.34149949
1.8653231 4.087261235 5.925588382 2.9641180722.185600381 -1.780036525
4.553967763 3.932244258 4.523171096 4.207223095 -2.351538029
5.078243286 6.564123257 3.026948634 -0.081097933 5.695174037 -
0.78602964 6.172804871 5.08986433 5.090852353 5.501974992 6.564332906
9.907857465 1.025936408 2.82973407 1.140716754 -0.928102856
3.181816505 0.686482868 4.325137348 6.9113341933.769894975 4.70756474
-1.906008326 2.461479058 -0.170681658 3.328455396 3.953031335 -

2.718743538 5.359345995 4.390861667 1.0611420152.951652652 4.071933472
5.06022899 4.657996108 3.725958471 5.335663788 5.08024791 4.80117729
4.449969596 0.645750273 3.843965864 4.584757834 4.683696215
4.667974379 5.799819218 5.060351224 1.313329588 5.554168153
3.633745532 1.523410269 3.980722397 -0.866978234 -3.490802143
3.006110012-0.454925474 -3.608909572 -3.471304179 1.378077927
2.745845596 4.07761341 5.609257216 3.491472696 6.324852668 -
0.782785388 2.584313458 4.655697067 3.86397497 2.066005774
TCGA-55-8616-01A -0.434200103 4.936122136 2.291205882 5.305947588
4.461671259 8.1804111423.449377608 4.149915232 1.887658881
3.083495617 5.132471001 -2.352523099 3.638865544 4.667711413
4.833598994 5.062818994 3.9453814 3.956743746 0.473303947
0.594794621 2.73520899 5.103587251 -1.209820797 1.542891425 4.59271963
3.20057071 5.286274005 1.973085003 4.554381795 3.822615121
5.1170561331.073889619 -3.491888537 2.958639412 -1.037985573
2.967304331 -3.247738265 5.604878791 3.670239633 1.344297462
4.702690312 -1.125796424 5.010339396 3.173371776 3.955995258
4.215090187 6.904359174 1.634705148 3.774715241 7.135773738 -
4.63595771 -1.899311676 4.686071829 -2.391872329 3.426507445 -4.822262617
5.252977533 2.2114834312.403655634 3.909998769 5.915920439
2.706984215 1.351650558 -1.948919878 4.616689503 3.931668474
4.016532656 4.323261444 -1.569363893 5.077698706 6.404255869
2.886389018 0.048655955 5.456664974 -0.515973062 6.200092205
5.001866892 4.920104014 5.4471174326.288169978 10.59897764
0.898415025 2.88496282 0.739924708 -0.853705303 3.360487604
0.897161714 4.317476678 6.937616975 3.556959255 4.804817031 -
1.668613195 2.467458216 -0.260640399 3.267000667 3.725590415 -
2.245845591 5.355101387 4.251696234 1.300988451 2.815157412
3.828651273 5.025892823 4.731607772 3.449617957 5.33550584
4.635926965 4.953436825 4.331437933 0.77103772 3.870399643
4.6480511664.82796823 4.712650779 5.684428465 4.977943378 1.040145914
5.448136686 4.336565586 1.843058706 3.936023421 -0.942705588 -
3.89351806 2.484985779 -0.21119735 -3.477704675 -3.733763053 1.595158212

2.870203503 4.293709672 5.29365905 3.407755815 6.230169254
0.164691548 2.895786917 4.491052164 3.77546916 2.795812158
TCGA-55-8619-01A 0.264984001 4.97531244 3.082438223 5.253210124
3.924750157 8.266189795 3.337989991 4.343093143 1.788948934
3.247067894 5.391778455 -2.488889231 3.621277764 4.118961518
4.892784559 5.083638359 3.5972756 3.725254345 0.542957155
0.572228922 2.748696306 5.196988906 -0.886137378 2.37376454
4.479352971 3.298452057 5.859225688 2.065623601 4.581082267
2.644904992 4.930581293 0.780004084 -3.457532456 3.380453738 -
1.3841124132.825980397 -2.680391773 5.570976828 3.3441117181.022204904
4.726421549 -1.023950214 4.881500164 2.625615246 4.188895642
4.361673462 6.976435837 1.636738464 3.597191399 6.886131341 -
4.461641997 -1.802720288 4.308098275 -2.464237094 3.29426714 -4.807998448
5.214064256 2.089415542 2.014794044 4.018886669 6.26949971
2.676018534 1.710672009 -1.927442676 4.794624668 3.924596422
3.902377389 4.319955203 -1.150338981 5.100394729 6.489955891
2.986054127 -0.261608876 6.201782349 0.107992396 6.01278107
5.204622747 4.605259654 5.549940512 6.082247244 10.41128438
1.137209998 2.562949436 0.469798024 -0.516868393 3.781573441
0.978449273 4.299059929 6.883972153 3.615409788 4.533053442 -
1.935685773 1.889274998 -0.472340638 3.230350542 3.752391309 -
2.120734864 5.343451617 4.881067578 1.449797596 3.204082035
4.317492065 4.983963796 4.671952922 2.712718507 5.335626823
5.255888623 4.899530601 4.074013864 0.866102636 2.68509782
4.687637021 4.899989149 4.77643836 5.67264726 5.285255054 0.982994211
5.686515416 4.544053444 2.324980096 3.889364821 -0.334272323 -
3.722414516 2.811871335-0.271680464 -3.327081436 -3.489617005 1.92719597
3.2014324 4.704477062 5.073523977 2.916382986 6.063791738
0.002455626 3.519097422 4.4115937893.861074878 3.313314298
TCGA-55-8620-01A -0.510923029 5.276148193 0.164175175 4.637304398
4.618950164 8.221087727 4.825485166 4.034201518 3.288921524
4.599745915 4.850481648 -1.131948026 3.520728888 5.552370394
5.518205393 4.565956285 4.856083613 5.7080114450.364508873

0.247290034 3.482499449 5.968654551 -1.370021951 3.138549972
4.849014045 1.767475793 5.572689618 5.369266979 4.976459178
5.142601783 4.952692111 1.949313724 -3.09923824 3.044796286 -0.608817458
2.025403604 -4.042976653 5.581157299 3.840447811 0.17385286 4.697756096 -
0.759078806 5.045387919 3.992537155 3.746825477 4.425842911 7.151568679
2.287685566 5.850450736 7.589464384 -4.740571832 -0.448646016
4.149905524 -1.422059881 5.134936549 -3.752616238 5.630041404
3.524689377 3.162857092 4.419768021 5.879821563 3.246791662
1.843234025 -0.519866245 4.609113417 3.927086289 4.948690869
4.639628375 -2.409196455 5.053893286 6.989934043 3.094302307
0.241914526 5.298601249 0.169708809 6.161595845 4.992632044
5.58731092 5.290578354 6.643852737 9.291905903 0.131425396
3.567570351 2.656533988 -0.335931696 3.998803887 0.130971522
4.052056047 6.952950045 3.430681727 5.067158282 -1.766228726
3.15928205 1.34196722 3.535436446 4.57567268 -2.352181913 5.325078711
4.670080221 2.630809342 3.823535709 3.039262983 5.097504057
4.573441923 4.673651005 5.335792618 4.432587487 5.149420755
5.011116405 1.027252601 5.742408257 4.282179884 4.768531587
4.711302497 5.740560481 4.54189882 1.812965279 4.529685347 3.69805211
1.456502764 4.2562812 -1.656005278 -2.163935849 4.003135933 -
0.168523358 -3.897086123 -2.454269753 0.942327903 2.532717897 3.25633825
6.509004146 4.185748959 6.953223 0.279975576 1.560755854
5.209325822 4.212189392 1.837758862
TCGA-55-8621-01A 0.092012812 4.90648005 2.941970765 5.264626175
3.932581431 8.272557049 3.795599745 4.274248478 1.879249719
3.56329522 5.168556098 -2.284069958 3.529342016 4.392102424
5.040051242 4.792429308 3.620180685 4.104441328 0.438399215
0.521883598 2.789043667 5.418278565 -1.080143487 2.707298602
4.559481371 3.128870209 5.861194161 2.134281695 4.608048703
3.325172418 4.916554129 0.962884178 -3.379944944 3.416134983 -
1.165003572 2.664220271 -3.224576884 5.566554267 3.279767898
1.023876416 4.723147004 -1.020394997 5.119678814 2.864786952
4.037045203 4.305072993 6.973562016 1.68001372 3.690970107

7.013623651 -4.732428244 -1.714993747 4.269788468 -2.586670019
3.583147585 -4.460132025 5.16627157 2.194592317 2.3551911 4.039262569
6.165852458 2.855450657 1.928873778 -1.452952958 4.785166844
3.930095034 4.123109253 4.263852474 -1.444218779 5.101479929
6.499203721 2.879332981 -0.02762398 6.078614058 0.0668811025.964683997
5.1127893024.692162253 5.457912232 6.346382303 10.52600912
0.684000353 2.7236031110.806391828 -0.574411112 3.841661382 0.97233782
4.2488007116.914494177 3.4921135184.557682759 -1.902086022 2.131921529
-0.090397959 3.262285271 3.955296908 -2.088901358 5.340632887
5.089642519 1.489514216 3.20799629 4.0980541155.0011536 4.672080967
3.0611786745.335715402 5.1197772995.016434267 4.335976886 0.834260846
3.153757543 4.707849683 4.910471701 4.792710787 5.61671012
5.224063883 1.062625042 5.512948641 4.462542372 2.323418543
3.889031262 -0.549328944 -3.625227053 3.229193089 -0.446346511 -
3.557808102 -3.345844342 1.651567008 3.024018559 4.477916701
5.157594621 3.078590184 6.142028844 -0.105827777 3.116736521
4.449184661 3.809527909 3.113045374
TCGA-55-A48X-01A -0.34005196 4.78727285 2.550126298 5.242188255
4.3455601128.1781145013.636348243 4.224631949 1.855956913 3.098144807
5.18212783 -2.438276434 3.414448227 4.601371018 4.897313581
4.959164447 3.860397982 3.955145773 0.257865502 0.458012208
2.759427633 5.297545151 -1.134273925 1.775678609 4.581287404
3.1117850175.685086032 1.873876635 4.567753506 3.321650313
5.004277645 1.016714696 -3.518625325 3.19413875 -1.196235103
2.984217158 -3.359541335 5.587553253 3.445868092 1.09692199
4.713647123 -1.075158622 5.073003184 3.095179573 4.03479347
4.251456241 7.012570895 1.726506856 3.982485487 6.873776713 -
4.677426499 -1.818759153 4.590129118-2.455144699 3.350816428 -4.779388225
5.199397877 2.151697535 2.267463217 4.007937092 5.973352446
2.6728118461.540441796 -2.163782339 4.695488344 3.934358522
3.945325391 4.306193776 -1.216587327 5.085773679 6.350076185
2.961126443-0.016708949 5.835774518 -0.33446801 6.080441604 5.107608886
4.708972722 5.475496349 6.308506079 10.37921938 0.337215696

2.751933964 0.78179163 -0.778605531 3.409404376 0.803013267
4.271542727 6.912944923 3.570872214 4.760024313 -1.83234711
2.202848072 -0.403040865 3.24798286 3.605389435 -2.104493393 5.35790522
4.517601349 1.336515268 3.1185225553.991482549 4.979483206
4.792630384 3.338594089 5.3355111224.8559957 4.939312193 3.936454978
0.809757658 3.448480521 4.56856316 4.884514305 4.75344166 5.68967295
5.240352689 0.847599445 5.587256157 4.214623496 2.063672404
3.98620563 -0.459899685 -3.679691556 2.842265344 -0.108064449 -
3.504063233 -3.893938501 1.441241699 2.922254017 4.324154098
5.429750987 3.407440326 6.291303624 0.169705087 3.019370627
4.491573639 3.752622121 3.001572097
TCGA-55-A48Y-01A -1.325601819 4.40355596 2.281681716 5.599349319
4.017229057 8.364138375 3.169230284 4.318976634 1.754170986
3.486021387 5.724909938 -2.316949448 3.289590695 4.59802038 4.38799367
5.163652188 3.978039477 3.788868102 0.499461825 0.367781336
2.54314978 5.04406624 -1.773275829 2.666558514 4.559677082 2.662135921
5.640258304 2.208430364 4.35882661 3.073842793 4.747397268
1.032444072 -4.177181464 3.860819078 -0.961757266 2.09986242 -
2.868241978 5.59084661 3.174531289 0.375261987 4.686906522 -1.377953973
5.1123292023.180830967 3.982704889 4.270876586 6.813824978
1.2702611423.284876488 6.600354556 -5.357544834 -1.882724174
4.682919387 -2.609841367 3.29668636 -4.960233436 4.947900188
1.780140234 2.212714674 3.846251858 6.071888022 2.544581605
2.684637281 -2.396809983 4.549721639 3.935835507 4.019184418
4.231231508 -2.652615495 5.072772922 6.365052845 2.836122797 -
0.159530656 5.99278024 -1.16923046 6.04249865 5.13021531 4.607043471
5.555048632 6.365658839 10.611766240.877613862 2.353936295
0.562599965 -0.721782715 3.1188116730.85156886 4.343632063 6.808117285
3.545804453 4.910649021 -2.082848511 2.044913306 -0.507880214
3.306228822 3.791356827 -2.89381209 5.341074383 4.500727598
0.977312347 3.164167207 3.956078492 4.926293802 4.65684963
2.966776373 5.335145677 4.934965446 4.891563635 4.150200777
0.361660189 2.717951952 4.485732838 4.592425777 4.722069317

5.629904629	5.150076658	0.951572929	5.843698849	3.962559039
1.51181574 3.733495638	-0.535454059	-4.395691022	2.758586601	-
0.457890375	-3.599709842	-4.003660698	1.581317743	2.412010812
4.2991155264.79473255 3.108036128	5.954091922	-0.980282738	2.93449278
4.392204513	3.533876976	2.499167947
TCGA-55-A48Z-01A	-0.51230088 4.660740558	2.070395674	5.093281573
4.153779256	8.288002592	3.634868132	4.232861306	2.050566588
3.356901968	5.074075961	-2.187082788	3.3021649114.748307264
5.040002749	4.856449006	4.0503797114.1545081120.492498031	0.45004123
2.861332764	5.595740955	-1.145144927	2.210890397	4.65642637
2.787844621	5.444423088	1.933755369	4.669166912	3.44083133
5.065266222	1.293746954	-3.573856861	3.206326709	-0.79812474
2.881265496	-3.389286787	5.593131259	3.390829058	1.324061359
4.717616022	-1.066236328	5.030045382	3.1059591 3.895654293
4.223606622	7.004452787	1.651879485	4.186721045	7.145129323	-
4.485220901	-1.542846908	4.699995227	-2.413241907	3.772924504	-
4.17098722 5.26287309 2.44647621 2.9491151294.089249032	5.927899338
2.445320817	1.531307463	-1.507852685	4.72031025 3.934743541
4.335294827	4.224816387	-1.36601841 5.079985285	6.558063956	2.91276447
0.159508618	5.495721166-0.376116956	6.0434098 4.989971833	4.967568663
5.397750238	6.459399161	10.3350955 0.524573965	2.933072636	1.11458998
-0.517697633	3.140636447	0.764954809	4.1971186616.91515204 3.372199143
4.795891962	-1.938548371	2.216773794	0.231068973	3.335857313
3.963946369	-2.144063706	5.32792396 4.685767276	1.432785806
3.442330218	3.557801783	4.970251699	4.795650658	3.799200129
5.335569068	4.541999939	5.096327293	4.290150661	0.877901292
3.629019073	4.469420968	4.868017872	4.761463128	5.541027078
5.023992565	0.953102629	5.295330143	4.183244206	1.958368728
4.007769986	-0.554273936	-3.21218318 3.487040092	-0.3576711 -3.509560581
-3.082249508	1.466160631	2.808233922	4.129819342	5.507357381
3.237044968	6.388594814	-0.377854073	2.950721831	4.599483631
3.7791140273.234891731
TCGA-55-A490-01A-0.870861813	4.979745712	2.16367883 5.426191922

4.333122447 8.250075524 3.554314161 4.183922425 2.19304014
3.634369274 5.745492537 -2.116789615 3.583039349 4.653324109
4.989055076 4.972109691 4.1070141144.288579278 0.691405519
0.592195042 2.86802993 5.338285764 -1.427567208 2.527429361
4.583069646 2.84010017 5.427654552 4.0529113894.703371066 4.971636961
5.100596936 1.573095636 -3.359050303 3.714261492 -0.72714431 2.79107206
-2.472620719 5.615183981 4.200030056 1.035887429 4.705648194 -
1.088264534 5.305896346 3.014022235 4.012294507 4.145553169
6.963747935 1.800726121 3.970639319 6.95126407 -4.509218248 -
1.59451662 4.731026939 -2.305751741 3.392687982 -4.346313272 5.302236437
2.566099538 2.35798102 3.866514581 6.006082481 3.046853031
2.438435184 -0.910972398 4.601051823 3.931709556 4.264128378
4.522291867 -1.918275415 5.09686028 6.529217008 2.8119344070.08426083
5.531918786 -0.495944537 6.16435577 4.9788411295.074671286 5.488842523
6.381898259 10.837118561.205488409 2.871236459 1.145909689 -
0.280199731 3.42076592 0.842833013 4.376796983 6.888689998 3.625218218
4.954548373 -1.58153891 2.784863632 -0.018631062 3.360965408
3.915423888 -2.760191249 5.344966555 4.111789751 1.343341323
3.289462901 3.888731225 5.032026635 4.759605206 3.477764802
5.335832537 4.769391076 4.930935137 4.43359623 0.7111694353.658699997
4.507998422 4.761792727 4.707690521 5.738641445 4.824363044
1.167685864 5.554897495 3.653520315 1.775378717 3.9432154 -
0.592221069 -3.720111951 2.884448906 -0.28474242 -3.503868171 -3.413688583
1.64494361 2.767915391 4.300392127 5.357242541 3.536229641
6.1166099120.423744894 3.101832172 4.448691871 3.703353558
2.833608429
TCGA-55-A491-01A -0.723006166 4.886356846 2.287126496 5.348062217
4.155518752 8.315423666 3.515643801 4.284783606 1.992604296
3.706046242 5.945726746 -2.202934704 3.957019485 4.614406978
4.972532405 5.190541226 4.064124338 3.909498975 0.21405292
0.513881953 3.010946433 5.115299706-1.078584767 2.501467443
4.526826831 2.898694685 5.755861456 2.860768024 4.772550532
4.164731203 5.013774992 1.412371773 -3.291633922 3.445131382 -

1.179484637 2.471142289-3.244088199 5.602545109 3.796049482 0.183316827
4.722030788 -1.033047886 5.337021334 2.671058163 4.00413704
4.317027173 7.0975691151.435838289 3.8507116567.060520615 -4.687452381
-1.810764522 4.556474534 -2.480155674 3.656237191 -4.332297271
5.262431083 2.249009042 2.107881042 3.871404277 6.249058465
2.863177742 2.417977769 -1.344267255 4.606790732 3.927426204
4.275448579 4.388002647 -1.232405376 5.099492692 6.528167335
2.860537358 -0.024215054 5.966468084 -0.388466217 6.160125586
5.109612951 4.962500814 5.575096512 6.275966855 10.45001517
1.276182463 2.829632321 0.85018528 -0.577973338 3.173393765
0.953879888 4.391333303 6.878563738 3.761759234 4.944241868 -
1.93532967 2.588184073 -0.137814956 3.371983152 3.804423606 -2.591309107
5.344584128 4.317390529 1.217170013 3.283407362 4.262699413
5.027777213 4.638947335 3.328193618 5.335794645 5.137453908
4.970712228 4.555506147 0.873627295 3.304037378 4.664372756
4.863618497 4.716767029 5.90055173 5.395994331 1.216266638
5.578571368 3.72688435 1.901215702 3.954698103 -0.890166845 -
3.484308431 3.250036561 -0.244095942 -3.55872603 -2.933425102 1.767444535
2.82291471 4.556324914 5.447309537 3.332371864 6.094940949 -
0.088479172 3.015293933 4.664744618 3.87917053 2.831392397
TCGA-55-A492-01A -0.521817909 4.877362894 3.905540302 5.518143867
4.048222549 8.243129302 3.560040394 4.260735272 2.269516883
3.162679004 5.299758075 -1.779910255 4.016808526 4.155081828
4.723559586 5.278159315 3.559822183 5.001049227 0.192371509
0.413109992 2.97213243 4.832439958 -1.125899569 1.920074712
4.545122246 3.429030469 5.499429745 5.383234048 4.55940578 3.69498937
4.92941889 1.199440134 -3.642786972 3.194761348 -1.692203972
2.962125524 -2.657685735 5.603668193 2.491075353 0.155708297
4.723385018 -1.08962432 5.139065537 2.809069571 4.0746625 4.261327471
7.141833359 1.48484108 4.191076053 6.920541394 -4.821821319 -
1.527386496 4.412297157 -2.538795557 3.569015381 -4.166047258
5.089724325 2.729654682 2.64426444 3.804589604 6.086836428
3.007154189 2.120020579 -0.749049915 4.458169049 3.929515495

4.883650713 4.036389188 -1.459914621 5.086917853 6.50941538
3.026491927 -0.114245873 5.926593291 -0.712488025 6.17452572
5.147398937 4.95346373 5.554609565 6.278427014 10.02114021 1.400207897
2.444119521 0.973784717 -0.750334536 2.981131632 0.826661173 4.52034597
6.92895282 4.064002745 4.581576107 -2.214551271 2.383389987
0.260072932 3.370526025 4.041409902 -2.419053499 5.362726543
4.267962259 0.381116005 2.406013665 4.327987984 5.092057098
4.617053793 3.628437198 5.335631977 5.305824206 4.73138933
4.985913153 0.676216105 3.31763372 4.745426608 4.748706568
4.688705001 5.929974267 5.656020037 1.510198227 5.369927633
4.026809502 1.856708098 4.026316935 -1.624560723 -3.373656823
3.185691321 -0.274387824 -3.551432277 -2.588161333 2.060768564
2.982178974 4.698087367 5.442342821 3.533381406 6.190955646 -
1.658474247 2.919379391 4.843151202 3.815060848 2.500584288
TCGA-55-A493-01A 0.097640648 5.093956687 2.111131236 5.229628531
4.045345303 8.162776459 3.73476358 4.288283067 1.845851798
3.787786524 5.520345699 -2.567715986 3.683702816 4.790877495
5.177256561 4.901121381 3.72093223 3.862450475 0.462408864 0.50820864
2.688548099 4.99635507 -1.042596126 2.522088955 4.579328849
2.528219643 5.695043619 1.977045149 4.742961295 4.722031576
4.987954374 1.407266979 -3.265450055 3.113447614 -0.618608561
2.189410423 -3.227004909 5.617215521 3.720506846 0.928628392
4.719396113 -0.913407005 4.841660554 2.696913175 3.938800676
4.153550202 7.020807052 1.391274268 3.966250329 7.116006489-
4.71954741 -1.939558731 5.344466756 -2.123723264 3.118783702 -4.509831305
5.32660862 2.302017663 2.305569937 3.603713732 5.901622066
2.632399441 2.197138516 -1.633981158 4.772544848 3.938541737
3.891933651 4.742258702 -0.840433982 5.095289203 6.342052893
2.645072499 0.079476201 5.416143631 -0.291389366 6.16042395
4.897427103 5.01915865 5.470018759 6.302480935 11.17705491 0.460477605
3.098025815 0.887947207 -0.283362462 3.806615493 1.0806067
4.296490727 6.870390264 3.266745004 4.99344542 -1.858056366
2.946563936 -0.262372954 3.329234932 3.914812703 -2.235124008

5.346366136 4.105470309 1.183837603 3.0428663113.706437247
5.0721173144.9119795453.076381292 5.335795458 4.232357014 5.045504114
4.04283115 1.029686805 2.332124941 4.423163962 5.008559531
4.770458466 5.386636814 4.380688594 0.854340556 5.297285611
3.917867814 2.078427751 3.968752834 -0.360136714 -3.839034578
2.860613402 -0.146781716 -3.658032045 -3.147958977 1.460484845
2.822410944 4.22715437 5.472715216 3.3281372 6.312877347 -0.134719298
2.589833089 4.403706777 3.607151792 3.005945177
TCGA-55-A494-01A -0.88064297 4.983234906 2.185466566 5.223589693
4.238272717 8.196088247 3.704386857 4.156831485 2.069688864
3.006493586 5.020582109 -2.148376334 3.325099501 4.692988584
4.916029063 4.8510301143.604281597 4.11391364 0.45020598 0.320260803
2.874322343 5.215793059 -1.253716524 1.339664859 4.642521873
2.84578919 5.42952494 2.8118333384.532746747 4.067792364 4.939228476
0.996290862 -3.523386634 3.002417634 -1.039521379 2.571817863 -
3.690924044 5.603336892 3.806159936 0.273745548 4.705333398 -
1.098181743 4.630372914 3.064707961 3.935007659 4.2118135857.100554197
1.4253861184.353523286 6.963135443 -4.771947374 -1.792602495
4.795164422 -2.214073868 3.306947442 -4.389987421 5.214839044
2.22721964 1.941271407 3.977179167 5.689921415 2.571446515
1.705667674 -1.895362949 4.516692717 3.940540324 4.392585824
4.40429793 -1.159761554 5.069342878 6.383807241 3.041078267 -
0.067405429 5.604167256 -0.889244616 6.20707877 5.062255107 5.061746013
5.486573945 6.510267175 10.00779142 0.434625046 2.90000979
0.978673696 -1.039393016 2.969443761 0.704847248 4.32318305
6.875151455 3.574063293 4.802371885 -2.023308403 2.63947034 -
0.147968058 3.257524383 3.609738989 -2.251557357 5.362515016 4.11919049
0.905332356 2.5960113533.845800107 5.066371312 4.740728023
3.816768537 5.335475316 4.746368341 4.819837624 4.44283128 0.54703845
3.349973546 4.463235259 4.740387625 4.710778585 5.639418484
4.688140089 0.934760049 5.500340988 4.0371968 1.709855489
4.096082944 -1.354197643 -3.192458494 2.932117496-0.22346581 -3.391492219
-3.890623244 1.248019247 2.755492422 3.853262969 5.843218285

3.670939549	6.365785425	-0.696972281	2.644706781	4.459451491
3.772105523	2.192828087
TCGA-55-A4DF-01A	-0.854625589	4.846828003	0.860607734	5.022223437
4.469602349	8.187260717	3.913788133	4.07139881 2.315208318
4.289507133	4.502487282	-1.902255103	2.995928386	5.428941983
5.142094268	4.374516756	4.523613699	4.066773416	0.358041782
0.321887952	2.889075656	5.812614264	-1.41433599 2.288929976
4.775341801	2.197241295	5.802633639	1.630078229	4.675424664
4.488954427	4.855141545	1.515625261	-3.648325156	2.667943006	-
0.562027994	2.519417982	-4.568527301	5.586314946	3.621783582
1.679217087	4.680887946	-1.187209697	5.216842035	3.86485659
3.638991529	4.335539466	7.036991578	1.752871925	4.652479747
7.163711721-5.047093659	-1.413627458	4.841228903	-2.278893079	4.22986156
-4.107275911	5.137521231	1.887805846	3.659303222	4.33641851
5.770835655	2.535971751	1.508223393	-1.327725038	4.62244981
3.938319718	4.109019828	4.207213175	-1.222784037	5.068513138
6.565240132	2.878927648	0.299725848	5.382612537	-0.303998775
5.924126587	5.009527332	4.908690083	5.215802838	6.443695471
9.920078065	-0.005832375	3.306986084	1.413626314	-0.846029593
3.336914483	0.466907614	4.056759297	6.868388544	3.252539747
5.163677021	-1.959247899	2.5786701160.631390403	3.360954592
3.851211712-1.974255496	5.321818433	4.922695555	2.098382846
3.645993948	3.429186037	4.883382086	4.724260991	3.798679047
5.335171608	4.471714136	5.077630296	4.19803908 0.532818369
4.004875335	4.486205305	4.587354239	4.817547729	5.597600844
4.708832282	1.1999311615.065251966	4.087182928	1.85544248 3.908046836
-0.951384744	-2.967603387	3.64940555 -0.352126521	-3.830165635	-
3.457434057	0.986882128	2.443542688	3.50501292 5.888208136	3.773380922
6.381391294	0.002328365	2.012560581	4.743973619	3.807266499
3.247549553
TCGA-55-A4DG-01A	-0.405347571	4.830592256	2.599774769	5.200015937
4.604970458	8.230001454	3.557290238	4.1145652722.043953997
3.096025019	4.689131069	-2.324445057	3.212100952	4.701569176

4.758027137 4.985081906 3.870396794 3.90951055 0.784960924
0.582599573 2.744905245 5.479195495 -1.27274928 1.319643462
4.671809214 3.334454455 5.416749515 1.342553545 4.470434101
3.442404527 4.933987565 0.934250712 -3.627302238 2.801792012 -
1.06895788 3.681140392-3.171901263 5.593496995 3.755775295 2.454199648
4.699274503 -1.218410036 5.297378236 3.592774603 3.968781881
4.2071194186.915513157 1.893041315 4.0116345086.789937578 -4.304525901
-1.756981651 4.853541802 -2.270577786 3.568147204 -4.754747752
5.100430449 2.059355922 2.749454686 4.289516206 5.857754771
2.771748723 0.984934188 -2.230917751 4.630824282 3.938805605
3.998941424 4.160217634 -1.139761891 5.078959401 6.346251699
3.070373022 0.012982522 5.519162518 -0.546092006 6.064114382
5.140138392 4.590927836 5.3563556 6.32871801110.4706788 0.551552177
2.8496618110.786106658 -1.155705146 3.096222344 0.673386812
4.245563869 6.946348514 3.729250003 4.652360479 -1.665301541
2.138812192 -0.042445773 3.21272191 3.582996415 -1.964561436 5.3571537
4.857367984 1.383830347 2.925650402 4.1101870234.921369485
4.889293951 3.559645094 5.335253192 5.075373007 4.770959786
4.013700161 0.592754066 4.097266941 4.4465702 4.702371031 4.75565339
5.629517863 5.164721995 0.870318729 5.630455571 4.367058588
2.061376606 3.927446129 -0.487950315 -3.516269805 2.843384953
0.079555525 -3.292262271 -3.91649286 1.461762645 2.895659039 4.13132903
5.368676706 3.699142151 6.310812614 0.261290503 3.018022281
4.457079019 3.744271547 3.298934214
TCGA-55-A57B-01A -0.131335909 4.657347275 2.663238605 5.209590792
4.293144325 8.24376283 3.484721883 4.204520107 1.810040207
3.159261205 5.173574205 -2.450838256 3.216390342 4.624199247
4.802156164 5.043262509 3.565904083 3.822335616 0.268873719
0.51008501 2.756660338 5.271439855 -1.001966234 1.985814619
4.609738795 3.309027045 5.632036926 2.256640312 4.585468528
3.275682722 5.069920412 1.036861955 -3.456471131 3.223884792 -
1.217646469 3.147988223 -2.98405294 5.589625272 3.90435561 1.45396682
4.71525346 -1.116354685 5.237389614 3.007907459 4.030625671

4.230460337	6.974016778	1.696264033	3.757256759	6.763696146	-
4.482041324	-1.662504574	4.82581163	-2.62594834	3.349617211	-4.645291323
5.159214873	2.297288962	2.515687192	3.985734216	6.034388778
2.644640828	1.608664376	-1.729581628	4.728104392	3.939503041
4.068000106	4.157580428	-0.814190289	5.089171017	6.268861122	2.95786273
-0.04516565	5.690573928	-0.238166204	6.022605882	5.094554333	4.71548775
5.436806747	6.351208764	10.55729865	0.858948157	2.755461639
0.694783198	-0.725282261	3.070164686	0.957481789	4.274835863
6.903604628	3.564612755	4.623304563	-1.901204359	2.256120122	-
0.309531536	3.252741801	3.638423068	-1.889499708	5.354809426
4.567187094	1.058451734	2.77593858	3.928123527	5.003852157	4.82912556
3.35333754	5.335594082	4.973975761	5.040041147	3.976424453	0.829313442
3.353653629	4.540103405	4.924896272	4.759744636	5.681715641
5.192734288	0.85134621	5.512100493	4.214923891	2.220843515
3.942879366	-0.60352453	-3.68810163	3.001890124	0.066698214	-3.40388988	-
3.784145786	1.615970513	2.990695629	4.443965226	5.220260193
3.323831546	6.303264016	0.342002035	3.224997209	4.540765303
3.709891635	3.0726071
TCGA-62-8394-01A	-0.298123272	4.865543352	1.645882748	5.186580414
4.386043267	8.20103579	3.378143408	4.124754601	1.999184949
3.308095815	5.147386812	-2.319640186	3.629698575	4.933164947
5.050819223	5.027197084	4.467015683	3.961419956	0.310892628
0.531847604	2.892365638	5.058320174	-0.996054692	2.074997842
4.634046078	2.735588498	5.352253672	2.124593274	4.657569897
3.791110233	5.170848342	1.432759097	-3.440166147	3.128175454	-
0.819288152	2.934138851	-3.478949876	5.608242286	3.48895788	1.362298648
4.70254008	-1.091273839	5.215200395	3.30010426	3.837958536	4.271133786
7.031135147	1.703276774	4.179362821	7.270558088	-4.724231163	-
1.921126454	4.888039938	-2.539967064	3.464268413	-4.541282116	5.262183684
2.080981457	3.035514913	4.030303265	5.937809845	2.513308046
1.641728177	-1.553230919	4.610020855	3.929205809	4.13545617
4.262566061	-0.650204402	5.070861665	6.442835559	2.855633625
0.122993566	5.223056483	-0.320980442	6.088095136	4.940589599

5.076094509 5.420710072 6.248167028 10.07095522 1.045103163
3.030240783 0.983358625 -0.671689005 3.242588884 0.852362767
4.27326588 6.889859363 3.417149472 5.13018949 -1.789141117 2.460947612
-0.103020902 3.328828272 3.691903389 -1.978251974 5.333022638
4.073400496 1.466247217 3.435276803 3.810412797 4.973195722
4.723660192 3.613957531 5.335358179 4.526656925 5.121166861
4.1195331140.919618662 3.736871457 4.591503692 4.824332754
4.743062638 5.769603923 5.178084975 0.945729901 5.29611832
4.237292603 1.9771153013.999510787 -0.893146559 -3.588306983
2.686446438 -0.264372662 -3.714936322 -3.294838302 1.500589913
2.702395303 4.412964257 5.629917909 3.458303729 6.278909836
0.174805656 2.9544181114.714476057 3.753248637 3.494717528
TCGA-62-8395-01A -0.757029039 4.648012177 3.049827507 5.362978703
4.12680262 8.277394487 3.564206816 4.207520548 1.773101037
3.206433187 5.326080294 -2.342754752 3.380215684 4.438670775
4.778950937 5.161675201 3.775076946 4.127568592 0.306038
0.423428769 2.946867681 5.006516399 -1.299335327 2.508098142
4.560892506 3.254781489 5.540332476 2.927857247 4.547530929
3.448095859 5.023704256 1.07125192 -3.515818797 3.512696523 -
1.418974945 3.102737435 -2.978944419 5.587649138 3.632601307
1.0377261144.709958062 -1.181706215 5.174464395 3.21306414 4.050043049
4.130847099 6.948931612 1.741462094 3.9543926 6.706980803 -
4.70017338 -1.749054892 4.235070517 -2.514050621 3.365594552 -4.697789181
5.183471285 2.231333294 2.065772621 3.845836098 6.096345823
2.876275299 2.259286083 -1.817062164 4.676258679 3.932144969
4.331527648 4.206474818 -1.893216201 5.085357791 6.478111351
2.987031874 -0.167336137 5.862222039 -0.606792602 6.089330654
5.127002772 4.7840611785.53962825 6.335817984 10.38397755 0.933285013
2.584532529 0.830483887 -0.792284895 3.382824267 0.839255662
4.358924604 6.93505144 3.724599368 4.62555016 -1.909760056 2.181284344
-0.10942095 3.299351285 3.80544164 -2.32383869 5.358623336 4.488876373
1.106623698 2.744400871 4.182927971 5.013648579 4.66891527
3.472546768 5.335566972 5.226223029 4.915336553 4.44758091

0.786879361 3.973473459 4.676783422 4.883878172 4.709914808
5.818609966 5.500245678 1.054051341 5.71263177 4.074519286
1.952222178 3.902634581 -0.783671707 -3.518971323 2.748587299 -
0.260007749 -3.479655511 -3.906146311 1.710582748 2.924494854
4.4530921135.179947759 3.2711497476.406057989 -0.226451088 3.088986409
4.45571019 3.747973316 2.593835699
TCGA-62-8397-01A -0.044643944 4.872736142 3.369393234 5.353286907
4.308433096 8.260134086 3.216349301 4.341333259 1.861581605
3.064472342 5.364777173 -2.371086492 3.694718455 4.393452947
4.893470346 5.338174465 3.201500519 3.96302154 0.381701803
0.643686184 2.754686559 5.195857103 -0.850712233 2.655016139
4.467492933 3.441807525 5.662577848 4.062929804 4.598666916
3.051851297 4.998206672 1.105502539 -3.348861877 3.4465721 -
1.136817272 3.066246505 -2.382795163 5.603526038 3.727412995
1.607069365 4.730735291 -1.164501615 5.363176724 2.589780501
4.1152831154.283996346 7.163414943 1.644697888 4.018493807
6.595597669 -4.160673161 -1.614962553 4.806706996 -2.350147871
3.256075027 -4.075484823 5.157121381 2.314941613 2.604044001
3.858587751 6.232628024 2.739318089 1.995785686 -1.080965017
4.698716108 3.934938488 4.352343847 4.127196418 -0.425710751
5.088579707 6.508885609 2.980182877 -0.202170374 5.995995556 -
0.041912565 6.014798356 5.144263993 4.913923482 5.481667266
6.280792591 10.80203201 1.184651703 2.858668364 0.742115527-
0.48840275 3.061804174 1.031966095 4.348669537 6.932828254 3.703122574
4.68905347 -1.952630642 2.194279635 0.044057905 3.27443626 3.721152883-
1.898273536 5.348640957 4.635714446 0.779181653 2.983832101
4.080415867 5.028471424 4.915393649 3.157472561 5.335651027
5.032910326 4.972604856 4.487009445 0.778305547 2.507575853
4.678568203 4.839578853 4.74810611 5.774929674 5.158547548
1.096822047 5.650715341 4.477827787 2.361389369 4.010051584 -
0.442264457 -3.405113796 3.138722046 0.386552255 -3.141755361 -
3.056689185 2.220202547 3.208597068 4.71358424 5.291908739 3.151522281
6.226672738 -0.164953526 3.146587947 4.59739593 3.846562195

3.349386219
TCGA-62-8398-01A -0.9215458 4.979347846 2.97356744 5.432160692 3.861217964
8.245983672 3.215747398 4.349753181 1.915227107 3.282072316
5.611903327-2.371278635 3.562692418 4.291968784 4.4416883 5.153850147
3.599691601 3.880339555 0.224794303 0.341226339 2.66603932
4.630528054 -1.357031521 2.15081289 4.540757207 2.825107842
5.862792914 3.148482443 4.473947678 3.186198235 4.878501194
0.877339502 -3.709051344 3.146354908 -1.422511139 2.217089877 -
2.909237645 5.602578252 2.813563773 -0.249839575 4.700771373 -
1.188176083 4.801917431 2.679101618 4.078620085 4.315629531
6.961049679 1.138079503 3.864352528 6.713807045 -5.088150279 -
1.8531751195.127450707 -2.310155743 3.130027448 -5.101111684 5.038712342
2.00079741 1.77367484 3.684477751 6.025353794 2.514129401 2.250816604
-1.976660072 4.629215756 3.9345211874.03330392 4.296932527 -1.96996832
5.088443402 6.335469786 2.940882399 -0.34628028 5.874576444 -
0.799722189 6.141782827 5.099037029 4.738644797 5.542529256
6.172029197 10.34921206 1.161080997 2.443967317 0.46104057 -
0.586320221 3.245303922 1.005220823 4.332991258 6.781027974
3.6147209114.744185401 -2.248248546 2.191742031 -0.533209908
3.252404618 3.733404583 -2.738214046 5.358877345 4.18010927
0.744206189 2.587046006 4.178723306 5.034696287 4.701389293
2.930688154 5.335556988 4.901928791 4.8191211594.066788837
0.573333485 2.262646554 4.629817497 4.752480927 4.721721275
5.679135863 5.00767278 0.954591022 5.527869735 3.861771616 1.5086712
3.853997883 -0.897805651 -4.197647535 2.434624275 -0.354824676 -
3.631834483 -3.746947782 1.715492799 2.885395397 4.394881968 5.29011728
3.410037083 6.069176108 -1.341847773 2.862137522 4.484286175
3.627476053 2.539979349
TCGA-62-8399-01A -0.521171297 4.922991032 2.149569368 5.212748243
4.314070348 8.20773296 3.468612538 4.1169740092.097958867 3.424438207
4.980932385 -2.434366003 3.235089191 4.915021448 4.872376763
4.802703617 4.5418646113.905084405 0.145992997 0.499704963
2.810177674 5.44562466 -1.392645938 2.245484026 4.664594896

2.803710594 5.620072438 1.49197778 4.575591047 3.907778471
5.030502761 1.289415078 -3.564403686 2.952876549 -1.033998904
2.601869758 -3.288351086 5.572003185 3.3113970381.120318523
4.684141646 -1.21923697 5.089581993 3.703660485 3.886266246
4.131422282 6.848881492 1.902321006 4.220738702 7.259053033 -
5.008569095 -1.723843289 4.750522655 -2.694506071 3.763887913 -
5.198087037 5.363888107 1.905491853 2.51188495 3.979065495 5.897655268
2.648373958 1.728546103 -2.45748698 4.742790103 3.922892638
3.977928467 4.378496561 -2.154193055 5.073018455 6.481335041
2.854126815 0.083220148 5.248916824 -0.584574975 6.153766318
5.016132714 5.093547295 5.375660796 6.542888422 10.35067634 -
0.1159552212.870251353 0.655817435 -0.603357058 3.762355722 0.770396832
4.225065292 6.969954599 3.301661301 4.946028962 -1.529176549
2.217082414 -0.349276301 3.260992143 3.955402908 -2.212957581
5.339614845 4.488077819 1.694120386 3.091346646 3.856719706
4.993035364 4.647916215 3.465027191 5.3353991174.702154412
4.991023492 3.720234367 0.882962959 4.253288805 4.668519965
4.863723509 4.712749587 5.522699639 5.094943377 1.027449374
5.545150714 4.015282653 1.716320313 3.897689838 -0.401915916 -
3.625835968 2.504970326 -0.479685597 -3.857578914 -4.280818837 1.21329002
2.625174379 4.0076798115.33537489 3.290643792 6.487600836 0.215631516
2.972075364 4.447782469 3.623766996 2.737547866
TCGA-62-8402-01A -0.860282663 4.887729373 1.696793598 5.332465456
4.368486913 8.171804991 4.002154016 4.197474873 2.077027259
3.299986712 5.321409159 -2.217094128 3.665724201 5.083262054
5.038419576 5.000386489 4.134893462 4.331656935 -0.079750076
0.544622665 2.702932617 5.131856706 -1.40339237 2.525189603
4.639221889 2.691025661 5.894591201 1.25431952 4.63164204 4.389612306
4.864986774 1.065019487 -3.117823706 3.167027403 -0.794836254
2.526960257 -3.943484125 5.598316331 3.26481746 0.129674603 4.70548177
-0.987676276 5.287561246 3.315253345 3.873772483 4.336261522
7.171485133 1.4118892814.612927693 6.970266783 -5.021885588 -
1.9331198554.943310217 -2.153996786 3.461264997 -4.728171407 5.212734984

2.142695937	2.095737107	4.045382192	5.909387665	2.847670878
2.156639593	-2.396783983	4.668476697	3.939998226	4.023795594
4.352900728	-2.091196175	5.077162333	6.3411668982.92328868	0.02824339
5.789631447	-0.436536069	6.136644489	5.058692127	4.977576644
5.515988988	6.33853843	10.50779054	-0.157878225	3.241543074
1.249785443	-1.029620167	3.530437564	0.830105024	4.319328953
6.802968916	3.604676467	4.97886246	-1.772632276	2.758473514	-
0.231283105	3.260419309	3.587178582	-2.687610938	5.369313242
4.231844844	1.416707872	3.219548056	4.035969239	5.071670123
4.843668341	3.403808986	5.335673663	4.793024762	4.942356105
4.066997478	0.745386553	3.785161877	4.582523793	4.718503373
4.733754168	5.784742547	5.221804378	1.134452855	5.242810159
4.079595087	1.60224492	3.954738111-0.946236569	-3.66997228	2.740366392
0.04653806	-3.677316377	-3.374459733	1.5112077042.726358093	4.378206313
5.798128784	3.941652612	6.475343343	-0.234572266	2.342418366
4.704845814	3.867057783	2.824602677
TCGA-62-A46O-01A	-1.725080258	4.591280586	2.899370876	5.321099936
4.354269524	8.331894646	2.972034729	4.100665431	1.83400322
3.241538838	5.48626859	-2.32385643	2.568508728	5.436552491	4.517448414
4.865912799	4.672831257	2.994442208	0.531906212	0.452823614
2.635023729	4.691137037-2.029529961	2.436929097	4.66025589	2.531719345
5.261223899	2.492390563	4.231670981	3.357669543	4.646987584
1.083110748-4.33384896	2.836982132	-1.482794706	2.40729718	-3.261107808
5.612544102	3.081232544	0.738819032	4.667590546	-1.499250509
5.10655232	3.840557478	3.731951775	4.213083853	6.959020581
1.105582191	3.757873718	6.562906466	-5.322313636	-1.919378393
5.401447865	-2.972598354	3.513480497	-5.224521078	4.948929374
1.109063713	2.658715242	3.765151689	5.703330812	2.596228854
2.157937464	-2.783482899	4.269482527	3.938912095	3.906738821
4.192081326	-2.516879168	5.062279624	6.057020868	2.91657698	-
0.1039451144.984100105	-1.767760558	6.246274363	5.023498602	4.669914464
5.317383012	6.500777753	10.78518298	1.262138914	2.673848624
0.229940918	-0.830175637	2.832947673	0.843100854	4.217865607

6.822307448 3.486246144 5.141207028 -2.113829857 2.151733996 -
0.203869448 3.258615433 3.922481675 -2.902219939 5.334953923
3.775085018 0.857872225 2.872497953 4.1146798164.860608763
4.688342547 3.207937899 5.334883578 4.762271255 4.820815868
3.89426424 0.015177745 3.856714201 4.661583086 4.455819977
4.729280932 5.602722446 5.074229677 0.693299275 5.595853085
4.505961305 1.182582298 3.708225948 -1.142314949 -4.099279067
2.160538266 -0.673966285 -3.888760151 -4.336280237 1.396204011
1.887374044 3.938306162 5.425843231 3.429437857 5.956554679 -
1.174134268 2.77028294 4.516655522 3.525838403 2.770833791
TCGA-62-A46P-01A-0.73521848 4.936805299 2.957061619 5.259805263 4.24890881
8.312788016 3.640604352 4.26922156 2.280229962 3.086683077
5.331494494 -1.900951929 3.989349583 4.328496601 4.803438197
5.195506753 3.787652837 4.836936643 0.644370261 0.493422832
2.88853381 5.350657989 -1.21701638 2.230406052 4.564587491 3.161064564
5.548744738 4.0247603114.570224014 3.626395692 4.952337637
1.121591724 -3.576916766 3.319979501 -1.113142825 3.085444982 -
2.862701856 5.59742576 3.233407504 0.582751861 4.721957835 -1.112733658
5.179363751 2.919850844 4.089559512 4.306719182 7.1135997081.63814067
4.200458787 6.931221898 -4.471717053 -1.598905068 4.616316772 -
2.264149391 3.781645584 -4.259961461 5.194823779 2.520396597
2.201466276 4.058437015 6.084676713 2.794298755 1.897801939 -
1.51398868 4.548696102 3.933165072 4.717724375 4.102888176 -2.09441104
5.079071067 6.6711356283.099174953 -0.061206964 5.887600013 -
0.64976128 6.1609411595.156806667 4.956336944 5.50127966 6.399713321
10.0722127 1.179586108 2.712199735 1.093134367 -0.856511982 2.93051126
0.761221998 4.404131918 6.921234248 3.907363407 4.615367317 -
1.990362432 2.321400238 0.19727285 3.315084448 3.974392164 -2.502930169
5.3583081124.542539048 0.676866539 2.875287969 3.97124697 5.079713385
4.683678471 3.646878343 5.335628615 5.287648045 4.785541095
4.75362396 0.638229172 3.34894692 4.71278133 4.730369436 4.693520749
5.982683858 5.402887428 1.444218279 5.452994848 4.274692021
1.742470895 4.048069093 -1.137270046 -3.182779176 3.095165396 -

0.113018996-3.313710952 -2.779026168 1.780966775 3.037703925 4.489083486
5.493863569 3.625952071 6.300969622 -0.81742558 3.005610981
4.747037517 3.871770671 2.768665029
TCGA-62-A46R-01A -0.575642483 4.693913838 2.606738298 5.421584767
4.587442644 8.218430593 3.205971279 4.251036755 1.658762195
3.104246068 5.116566618-2.267302686 3.529681572 4.923736686
4.827593575 5.123857323 3.420144038 3.490821997 0.805452613
0.500260294 2.555501653 5.210376592 -1.251398498 2.110394214
4.617850235 3.193210993 5.362605491 2.218237525 4.408093906
3.40545417 4.851671536 0.834816795 -3.69220219 3.254515493 -0.924402902
2.860668084 -3.128624171 5.604651797 3.49273448 1.502556574
4.702107988 -1.273787245 5.201783259 2.971280016 3.939890858
4.256856887 6.905787673 1.382465544 3.6515831176.734426458 -
4.720061706 -1.906473059 5.137717834 -2.380188889 3.422942861 -
4.547690343 4.950716828 1.858833402 2.67899696 3.923289107 5.934812947
2.523092239 1.78360987 -2.21128173 4.567710837 3.948932812 3.96995533
4.340699176 -0.825460755 5.080424526 6.04952565 2.90827046 -0.048357827
5.670553001 -0.605743043 6.070436224 5.039396016 4.493484449
5.439088031 6.454277389 11.099384910.561540564 2.91846602 0.499645827
-0.936434958 3.166731607 1.07450317 4.269484879 6.8282064113.520222764
4.869516608 -2.006630533 2.370363264 -0.054524797 3.229165829
3.598963001 -1.834868307 5.352498813 4.532599104 0.883330153
2.866225104 3.829985232 4.973172493 4.889588431 3.165899748
5.335355746 4.776897644 4.984014489 4.261444779 0.443032394
2.883466148 4.652195424 4.72004561 4.780280849 5.563829545
4.940195628 0.681845465 5.578627515 4.7014251192.091219812
3.864256683 -0.577734627 -3.536859902 2.922319364 0.1032609 -
3.329961677 -3.809546915 1.620898084 2.8192854 4.341560645 5.252120872
3.418614834 6.134878448 -0.493161453 2.869351818 4.461002854
3.633038781 3.443252155
TCGA-62-A46S-01A-0.954811749 4.665206085 3.276386023 5.369987384
4.25799533 8.375023321 3.352178422 4.342418619 1.919026156
3.179570072 5.307248887 -2.132715373 3.54265843 4.32506465 4.616060067

5.170485816 3.514062344 4.092559742 0.938801982 0.532159577
2.760402647 5.274344225 -1.363360221 2.249658918 4.540511828
3.128844873 5.710674757 3.682152664 4.436480398 3.209067344
4.703955816 0.9329482 -3.959975566 3.684496663 -1.122897678
3.020155456 -2.802433107 5.593684036 3.40885859 0.9119923844.718928734
-1.209497164 5.090217563 3.078576434 4.121018319 4.297167608
7.1112631551.684671636 3.972799867 6.507682555 -4.325715811 -
1.643571213 4.542941547 -2.24371213 3.268250161 -4.491219862 4.998621242
1.995161013 2.147276532 4.050003729 6.070372404 2.952148481
2.222567961 -1.791072223 4.544205444 3.937906252 4.417170056
4.239487337 -1.711097207 5.079792081 6.417253426 3.098868822 -
0.201836615 6.100583461 -0.784636885 6.034993338 5.280106258
4.583096865 5.509677185 6.503134125 10.4213502 1.268572887
2.403948847 0.687206019 -0.882376071 3.245267177 0.722979127
4.360314242 6.85449169 3.929463171 4.655113611-1.964929412 2.06114923
0.298963376 3.2834691153.789252585 -2.478620667 5.362684488
4.741457068 0.870839576 2.999383728 4.358706991 4.970026982
4.72622345 3.295900855 5.335363937 5.425469028 4.702815596
4.417802484 0.463351461 2.998834533 4.617789853 4.667738112
4.739597012 5.817788243 5.361609362 0.95333659 5.762170173
4.051803612 1.819326123 3.983314756 -0.52602027-3.320567434
3.003299064 -0.131730498 -3.130242565 -3.601383703 1.666343713
2.920424417 4.413718658 5.408899344 3.406492623 6.118276297-
0.61680322 3.222963209 4.588214961 3.658198072 2.787110106
TCGA-62-A46V-01A -0.915883576 4.774853321 2.996527186 5.4421185
4.083548004 8.178673086 3.730924033 4.225456452 1.883236356
3.395603073 5.087667622 -2.151258262 3.522806073 4.584545095
4.873675731 4.6924821133.307365369 4.061854826 0.142145172
0.297357388 2.707946468 5.357114607-1.346483696 1.893416582
4.601450861 2.938814636 5.672451662 2.965830326 4.520158331
3.949786143 4.78161966 1.165311486-3.610052662 3.068200192 -1.053469079
2.840885364 -3.397624002 5.594985245 2.963640108 0.567989176
4.706103099 -1.286824618 5.245510714 2.71877656 3.904379226

4.173601987 6.883724686 1.4962991163.875659048 6.711699398-4.875874804
-1.870612806 4.611203942-2.368161917 3.570003702 -4.590543319
4.971594935 1.985977984 2.803432795 3.971644087 5.899883668
2.558025969 1.947171846 -2.007252762 4.606338348 3.941316284
4.288709686 4.236043323 -1.341260097 5.091290628 6.326505637
2.914186813 -0.013327905 5.702693321 -0.806384626 6.062129781
5.033058233 4.732977385 5.503335693 6.40115059810.34538984
0.479847951 2.7062701110.972095232 -0.757817155 2.889679358
0.915555249 4.375152665 6.892537975 3.462499871 4.731463608 -
2.219557229 2.373508685 -0.081292871 3.291383887 3.763449436 -
2.2409115415.351454183 4.742649497 0.505386995 2.606815013 3.982760127
5.007773761 4.834743305 3.199529948 5.335589371 4.915047963
4.871001022 4.31455267 0.536085163 2.613967339 4.58945498 4.75052712
4.744597044 5.559937306 4.954676018 1.044345309 5.346388928
4.063169441 1.9243011013.865688132 -1.121422293 -3.301096305
3.135203856 -0.079035071 -3.573525295 -3.625540865 1.403120722
2.759210309 4.077290382 5.355295928 3.271689485 6.138870993 -
0.642208146 2.626874587 4.391946362 3.610683981 2.950561543
TCGA-62-A46Y-01A -0.159257329 4.973809532 2.884233034 5.435178056
4.264617963 8.192014876 3.389583956 4.325740177 1.923842354
3.341028055 4.972734102 -2.347878654 3.794283056 4.675286547
5.016902198 4.936132166 3.250603861 3.896452932 0.579935207
0.497921574 2.619072231 5.338792055 -1.096178267 1.655102067
4.600225657 2.966922353 5.723195657 2.589837427 4.575722405
3.581715385 4.799922143 1.030383266 -3.604934367 3.012624615 -
1.062232703 2.844478749 -3.206426454 5.594505874 2.994054421
0.953400361 4.725491985 -1.071750659 5.050628479 2.612511346
4.024423595 4.248242243 7.023546093 1.452381053 4.039766978
6.888039853 -4.858357596 -1.847873671 4.737042018 -2.248630648
3.604232821 -4.595673118 5.169401066 2.031579016 2.885994247
3.951059723 5.947009542 2.472923154 1.385685666 -2.26894898
4.669697659 3.941520324 4.042337386 4.335358719 -1.063084538
5.08442917 6.324780836 2.925123802 -0.075226473 5.831845655 -

0.223140902	6.09169862	5.107714005	4.668168254	5.479325885	6.276250322
	10.35480427	0.3398811182.829502567	0.89139566	-0.655573755	3.126127972
	1.000417721	4.338827197	6.906808405	3.472151456	4.773445171	-
2.023993316	2.357906927	-0.086708929	3.265293217	3.760635923	-
2.19052163	5.353304632	4.715687333	0.59320282	2.789886166	3.980826284
	5.013364478	4.841772394	3.050613089	5.335475144	4.854276148
	4.894953922	4.281947778	0.737795808	2.312814925	4.573504032
	4.815782602	4.786946897	5.4011508484.822175652	0.956387912
	5.450840641	4.5837050112.091728437	3.929635135	-0.715140717	-
3.539478494	2.973407357	-0.139900834	-3.50550441 -3.420231333	1.619475232
	2.893256128	4.318575314	5.402302159	3.3311668116.210096667	-
0.225679773	2.802222146	4.631517245	3.686187362	3.165533111
TCGA-62-A470-01A-1.260676506	4.765766768	2.6697031125.306686255
	4.259737583	8.226645504	3.60810008	4.198039378	2.161205928	3.41368675
	5.187031938	-1.912023085	3.412519135	4.7113897564.734594552	4.99312913
	3.984844336	4.191834123	0.527057324	0.261864729	2.709522085
	5.070927436	-1.715122818	2.235203278	4.647604191	2.796485454
	5.845124831	3.487199149	4.364344762	3.380985281	4.643504023
	0.995917203	-3.74626544 3.353511337-1.18509042 2.167889878	-3.525310389
	5.597068171	3.10473375	0.39912782	4.692033053	-1.234315665	5.064674219
	3.288666218	3.967151775	4.33539772	7.009338885	1.52692322	4.14862785
	6.712899877	-5.077339302	-1.668029901	4.613644319	-2.064418764
	3.562977437	-4.695477814	5.037021877	1.909762109	2.260551581
	4.036407684	5.939024301	2.70700253	2.048477111-1.948806442	4.493359512
	3.937667783	4.099638226	4.24564737	-2.373623589	5.072293786
	6.444121471	3.07136205	-0.192136982	6.040481864	-0.921778987
	6.109152585	5.125849685	4.690802454	5.478314691	6.348394865
	9.901096121	0.669350105	2.78726759	0.854917997	-1.175054317	3.13932154
	0.616526573	4.293941078	6.851535468	3.75792258	4.872504317	-
2.10769647	2.275263875	0.000106139	3.276923938	3.830791559	-2.866704895
	5.355896859	4.514627262	1.426775499	2.925740142	3.934130171
	4.967550857	4.663932417	3.304859358	5.33526069	4.975629235
	4.775183967	4.361715062	0.285581305	3.329430173	4.607819366

4.552306521 4.725794814 5.758433552 5.172858399 1.342715365
5.580223933 4.107784786 1.509615697 3.895825054 -1.174863968 -
3.626053088 2.831293829 -0.238700224 -3.504566652 -3.797253787
1.427954845 2.548564932 4.013321689 5.556756557 3.713249975
6.076755812 -1.25833913 2.410360797 4.551308428 3.766278224
2.470424894
TCGA-62-A471-01A -1.435668531 4.752418091 2.737287125 5.447471881
4.089677438 8.205221998 3.103606099 4.193465031 2.142876329
3.434273387 5.300153174 -2.186012213 3.562993936 4.367469508
4.265450159 5.197090284 4.118138472 4.060165688 -0.164102988 0.18647714
2.838982748 4.549064544 -1.68569716 1.761001056 4.612448127
2.553814541 5.3515196 4.103528726 4.386432966 3.349026049
4.956350333 1.104162697 -4.062791618 2.719918124 -1.327573084
2.179912242 -3.051699366 5.627896094 2.625657866 -0.829786268
4.675939377 -1.368528228 5.115373596 2.591933025 3.972186819 4.11493863
6.980533836 0.966390673 3.903987031 6.985892089 -5.516394297 -
1.819710129 5.3258789 -2.543050964 3.398102449 -5.444310312 5.077640077
1.769877107 2.554834718 3.567484924 5.888412935 2.245585622
1.940317345 -1.840400497 4.439917459 3.918097296 4.109978117
4.230811256 -2.555264769 5.07593137 6.445869981 2.956912253 -0.189161604
5.139038772 -1.338473049 6.264837958 4.931663761 4.909782585
5.533960239 6.113257778 9.989395756 1.047463937 2.351035869 0.60530566
-0.570227276 2.888250324 0.826707255 4.381659033 6.805848047
3.387074087 4.970162998 -2.230717923 2.093877157 -0.267034746
3.273125307 3.874961173 -3.125188568 5.342703509 3.806860629
0.257220703 2.586796776 3.676037094 4.990419875 4.726672731
3.161224534 5.33517741 4.522201476 4.853835152 4.276657862
0.472202181 2.512751861 4.694758284 4.70935863 4.666412009
5.759875077 5.055012515 1.158969218 5.218618112 3.823921918
1.161094126 3.814052975 -1.564940252 -4.100208215 2.263660848 -
0.599171544 -4.071864831 -3.803162291 1.586712014 2.44003445 4.246756955
5.36956637 3.565649197 5.971289438 -2.931507343 2.34438888 4.483927895
3.57619608 2.503637082

TCGA-62-A472-01A -0.657859588 4.838137255 2.842333015 5.38076792
4.220551459 8.236028827 3.612011246 4.346746451 1.672683339
3.178472257 5.228990084 -2.374303552 3.482087444 4.757981355
4.930839745 4.726991123 3.004894151 3.751669399 0.681672117 0.46673428
2.486938688 5.331124251 -1.245108541 1.946174737 4.600422766
2.837108425 5.794997225 1.548874693 4.488840543 3.786738506
4.671516043 0.72246368 -3.652219199 3.032728381 -0.874028536
2.940833905 -3.464529194 5.601270973 3.393328996 1.173743997
4.705313275 -1.154748496 5.000202022 2.575848869 3.921526215
4.219248811 6.849930369 1.175332938 3.897036613 6.684140828 -
4.928362499 -1.884115808 4.965945332 -2.186269884 3.408562493 -
4.711107056 5.129646989 1.770092893 2.454568109 3.862895055 5.844494256
2.477736164 1.767754871 -2.729486172 4.609945375 3.94238947
3.979372114 4.493858584 -0.841798468 5.084242288 6.210838327
2.869992354 -0.018537297 5.905351035 -0.60921759 6.12231868 5.016449673
4.657342076 5.45218291 6.464412459 10.56684797 0.203162805
2.898771877 0.868238338 -0.776323267 2.953017387 0.962933399
4.303671333 6.850018479 3.338789426 4.859620091 -2.021255526
2.493622179 -0.334307726 3.221153512 3.694393469 -2.201386515
5.348938102 4.55037941 0.145592116 2.807158172 3.69180914 4.990447881
4.885085031 3.290194733 5.335420051 4.779491987 4.900806987
4.312258651 0.542179007 1.936751839 4.530160384 4.776569116
4.782749034 5.560849308 4.397997214 0.781656418 5.483946837
4.389330352 1.951393329 3.903807729 -0.752244457 -3.931986308
3.040290473 -0.025827897 -3.469586897 -3.935351681 1.563420013
2.573399424 4.193311847 5.517665736 3.330526284 6.171423021 -
0.813438786 2.576403982 4.411760707 3.595439678 2.734243985
TCGA-64-1676-01A -0.806800205 4.272945009 2.553275987 5.669767399
4.472877103 8.275708944 3.269259435 4.355414701 1.736986644
3.691464074 5.50859383 -2.320421638 4.368120437 4.495533499
4.537361329 5.218874249 3.062934798 4.249132423 0.448129308
0.520170727 2.515545862 5.102017541 -1.409912511 2.522460358
4.536137738 2.685702307 5.263794345 2.394239835 4.426343032

3.943187909 4.594615106 1.232694934 -4.083801308 3.292380825 -
0.585809978 2.562450789 -2.790411769 5.623990015 2.416288705
0.263656104 4.715042877 -1.326173894 5.576600465 2.18855084
3.908017319 4.198513232 6.981005585 0.727376228 2.870364641
6.753003871 -5.176722945 -1.927922592 5.109987645 -2.508404228
3.585667721 -4.25261632 4.729496105 1.981713902 3.498255948
3.711241736 6.034290379 2.728207123 2.433483608 -2.22964951 4.38755343
3.943026134 4.189254971 4.360290439 -1.631758189 5.07816122
6.065882223 2.776689658 0.168831462 5.618682314 -0.917862978
5.964624209 5.063381071 4.316696676 5.511582597 6.278933877
11.30235785 1.099580558 2.555773851 0.951661354 -0.552672391
3.015054456 1.114390232 4.420719096 6.756956319 3.640875032
4.897185963 -2.442174285 2.312221944 0.084345802 3.371641908
3.775899277 -2.656909496 5.336550651 4.463203412 -0.502563968
2.846502521 3.956506045 4.969764241 4.894927144 2.900592906
5.335035991 4.639158205 4.86813771 4.876832782 0.395345662
1.055711875 4.454425474 4.633704609 4.776189137 5.542511468 4.854244605
0.910710996 5.457553758 4.042788512 2.031845861 3.73391077 -
0.606520695 -4.080982138 3.257478794 -0.036262772 -3.607895141 -
2.058967503 1.803287553 2.449481827 4.581151284 4.836972019 3.510591144
5.756577184 -1.509381928 2.428740313 4.614862378 3.478411657
3.585402174
TCGA-64-1677-01A -1.341837982 4.840229438 1.99304627 5.538009694
4.352483311 8.257046975 3.943560516 4.272595246 2.219315788
3.654944303 5.567716906 -1.99366817 3.952355857 4.821006415
4.915988391 4.55708718 3.636195769 4.57868799 0.223625185 0.400831048
2.486123927 5.458181933 -1.76583688 2.040102 4.657381719 2.282551152
5.873876972 2.328552548 4.586924741 5.033765539 4.675431635
1.275282051 -3.749531393 3.101264852 -0.529280072 2.45692276 -
3.983104727 5.611107203 2.826013124 -0.082644608 4.705828337 -1.180412294
5.417158643 2.716203134 3.745701841 4.214985814 7.091219224
1.115821165 4.026199238 7.06842667 -5.215965735 -1.814673508 4.800530727
-2.297504169 3.93230947 -4.557105593 5.027855188 2.095914394 3.26512373

3.974520236 5.79770725 2.624219316 1.936644925 -2.204749034
4.4771129463.94054047 4.188618895 4.256241225 -2.707221909 5.075049222
6.39498644 2.819219939 0.3434723 5.770976769 -0.968427962 6.104238682
4.993725376 4.875735959 5.454889875 6.454420284 10.12830834
0.656398184 3.069774386 1.447189492 -0.727665841 2.415769217
0.76595236 4.406341989 6.877439913 3.469681506 5.024720482 -
2.18066129 2.733749529 -0.157073279 3.333239649 3.979911343-3.220702694
5.3311409194.439655799 0.104787103 2.989042015 3.714664175
5.0011867924.752221874 3.55194396 5.335354281 4.676000532 4.86722946
4.46328717 0.399366209 2.284333628 4.421386199 4.562717093 4.75827224
5.724728344 4.636278257 1.302047789 5.079739298 3.957548733
1.403957928 3.891402513 -1.280559973 -4.125782749 2.880312165 -
0.301212888 -3.792300658 -2.720640405 1.333005543 2.270326358 4.06110439
5.502456587 3.59457537 6.061596132 -0.669459678 2.243935491
4.745450437 3.617390795 2.739684774
TCGA-64-1678-01A -1.697961736 4.313352777 1.281869377 5.429525687
4.087543554 8.339073868 4.3841180534.213340276 2.575983192
4.255236693 5.509474121 -1.490684085 4.253977891 5.191579657
4.824756618 4.547313515 3.602377464 5.083087984 0.433056073
0.427063401 2.733976761 5.643719032 -1.969560571 2.940673993
4.703391385 1.883826277 5.666473162 2.932875061 4.469912012
4.358643713 4.207635808 1.602232087 -3.948101479 3.337471022 -
0.5594012111.925459685 -4.003340208 5.600062439 2.108337748 -0.547989949
4.684919315 -1.257184981 5.497332405 2.891745569 3.606092079
4.4141967117.094471556 0.89004962 4.141746339 7.168619121 -5.482839869
-1.232801684 4.451127739-2.013530796 4.453005427 -3.538631141
4.940849961 2.390879514 3.85140145 4.1140415845.841801529 2.997424461
2.533893328 -1.884361499 4.228887881 3.942658685 4.75700513
4.361684905 -2.969600693 5.0427169 6.386081608 2.875732138
0.631319959 5.681066768 -1.225117461 6.014236464 5.05012826
4.859200099 5.436096784 6.690223986 10.06514229 0.640803229
3.155305079 2.093737469 -0.809328399 2.813506971 0.52602995
4.297303852 6.798440881 3.627266814 5.137925765 -2.426585431

2.64422493 0.630430678 3.443239979 4.111315869 -3.2538122 5.318699845
4.598179106 0.462423414 3.137628515 3.739860073 4.955640014
4.584667091 3.864044618 5.334876355 4.701121715 4.90848397 5.086249555
0.152702638 2.312424076 4.399206046 4.29208129 4.738981318
5.787804377 4.608974812 1.723482466 4.889104388 3.543392843
1.250333256 3.847062835 -1.19577128 -3.666685319 3.872157663 -
0.273689235 -3.821646822 -1.580816109 1.314313175 1.586565326
3.787697253 5.683174549 3.513101454 6.093581988 -2.147381203
1.370932223 4.892004608 3.712895289 2.145219795
TCGA-64-1679-01A -0.626198529 4.537272032 2.113824263 5.498455447
4.260384928 8.342268519 3.168298863 4.281520965 1.713813695
3.716004874 5.759623594 -2.584826923 3.636207776 4.621956705
4.62740863 5.211755763 4.029987049 3.598837794 0.441136279 0.56205729
2.753969858 5.232598357 -1.174976792 2.860867152 4.489918931
2.805611696 5.310214176 2.65752409 4.640821788 3.776684461 5.016028196
1.566794777 -3.729566036 3.642814973 -0.896201921 2.456400907 -
2.727572948 5.607638608 4.148968126 1.26644359 4.713000938 -1.298227987
5.203292316 2.921859849 3.965293433 4.187119626 6.951938291
1.474113606 3.10756108 6.758789907 -4.759069783 -1.76925335 4.905980066 -
2.788930272 3.26538431 -4.287203263 5.106195141 2.268903976 2.715218721
3.680956081 6.206729512 2.779300643 2.332597251 -1.6672676
4.656674969 3.932620858 3.980241225 4.401747857 -0.97304567
5.089678763 6.379973845 2.684968098 -0.015747277 5.603449218 -
0.463883236 6.001779146 5.155426422 4.699703848 5.49304469 6.272862437
11.29372948 1.346436063 2.57687248 0.638901154 -0.278908519 3.298732307
1.051405611 4.348171896 6.831461237 3.680988811 4.8559 -2.028285673
2.217603771 -0.21730546 3.374566121 3.754926791 -2.209224203
5.336379802 4.41243128 1.252600096 3.155343506 4.264381444
4.906700963 4.813015258 2.857188914 5.335394126 4.791494601
5.00282565 4.141065488 0.760727941 2.992678778 4.293604969
4.810649795 4.754072386 5.593212946 5.224192757 0.786375673
5.784261583 3.800525562 2.219103667 3.771814564 -0.060305376 -
3.796662161 2.888592718 -0.200653033 -3.50622545 -2.964063452 1.776719835

2.738147692 4.582018033 4.648337015 3.206348057 5.982992885
0.61837965 3.306006392 4.3717855 3.573047208 3.068450987
TCGA-64-1680-01A -1.240106866 4.630698188 3.028324493 5.617525353
4.440262139 8.282522146 3.466585714 4.383249061 1.932469169
3.076878374 5.337998428 -2.151587765 3.643089281 4.615865958
4.661901666 5.012742257 3.000749764 4.196489519 0.407570272
0.453091079 2.506125217 5.104893384 -1.441607116 1.793016274
4.510857987 2.866963344 5.482086946 3.092085428 4.455635436
3.431328982 4.656691938 0.96325145 -3.920452526 3.367707747 -
0.8751151142.658602989 -3.426840271 5.608632318 3.228719413 0.225088673
4.726679148 -1.172638691 5.173762296 2.522075333 3.984137417
4.262646907 7.150365297 0.972125102 3.749556541 6.624949107 -
4.977564805 -1.848868994 4.790397814 -2.414093025 3.366764561 -
4.234329832 4.880361003 1.949007272 3.034198883 3.90036426 5.929813057
2.707988326 1.88020605 -2.411730695 4.391952484 3.949377973
4.271479793 4.234121312 -1.680274495 5.077042073 6.15719585
2.965732446 0.0071196846.058037501 -0.934621055 6.068350281
5.206544005 4.542829474 5.512926506 6.367224034 10.46514191
0.742025332 2.6262158 0.93822096 -0.901148243 2.587164675 0.879175609
4.413593164 6.823092568 3.788574904 4.665685795 -2.374192881
2.2913441120.085307414 3.307130807 3.615745787 -2.81598093 5.355308655
4.607957426 -0.152812583 2.635009633 4.202675078 4.990565236
4.930871767 3.24322157 5.335328236 4.902759278 4.710403806
4.649536994 0.232597943 1.802758299 4.37379822 4.576284558
4.758318686 5.614236218 4.984971958 0.945565257 5.598590942
4.402584668 1.837205656 3.916079545 -0.869936118 -3.977811728
3.076674881 -0.125148471 -3.393358868 -3.032813834 1.69786058
2.638435708 4.356677656 5.201904304 3.533094015 5.923845165 -
0.806932842 2.840844694 4.690344962 3.643788333 2.646605423
TCGA-64-1681-01A -0.202271336 4.695687197 2.606814861 5.397072004
4.317750357 8.295715082 3.438304694 4.3115959751.770486586
3.517489569 5.182024099 -2.396834493 3.992768566 4.721766039
4.96322074 5.092750717 3.820404287 3.947050915 0.492188903

0.575484082	2.906309656	5.393028547	-0.900105941	2.727823833
4.507461779	3.089742198	5.554571207	2.745944322	4.62049645
3.5113647224.9293225111.369343935	-3.546242468	3.469316267	-1.086007498
2.927798836	-3.17510048 5.590417337	3.424695874	1.14384 4.724313459	-
1.161616413	5.159286181	2.984545554	3.966104734	4.270173128
7.072679921	1.4452733113.732171748	6.867822922	-4.690947473	-
1.651236992	4.62798228 -2.57984359 3.410730579	-4.029598412	5.139751992
2.22890083 2.778020686	3.918038083	6.146433012	2.85948138 2.058071699
-1.594843959	4.616658478	3.931929286	4.291307582	4.388133536	-
0.667796503	5.084939638	6.369696642	2.879379686	0.087776712
5.811940994-0.146610713	5.961788316	5.190759802	4.638399838
5.481500697	6.31166023610.70627065	1.180433079	2.775160478
0.914384792	-0.505857731	3.611611196 0.961973199	4.334060153
6.879877868	3.723518196	4.808177726	-1.949390131	2.188270418
0.096832395	3.346949256	3.686824546	-1.853712296	5.345086157
4.4273119751.226913031	3.195526668	4.169642544	4.970695404
4.7307011493.317995738	5.335379655	5.073236178	4.971288688
4.646399055	0.81304036 3.206680908	4.6121188854.833705766	4.774040947
5.729205225	5.3240211131.045549763	5.598087301	4.255836151
2.410429706	3.906220431	-0.457236699	-3.239280847	3.28502463 -
0.030863997	-3.409784521	-2.978438561	1.8717466	2.8894114684.609980365
5.236791924	3.248470568	6.161690807	-0.021057309	3.099410697
4.688639721	3.730852877	3.371307976
TCGA-64-5774-01A -1.320171999	4.651267066	1.231926759	5.235172748
4.1147310098.300867483	4.1656311354.144081353	2.605137181	3.874600122
5.111235359-1.745448798	3.408332835	5.03249703 4.972877476	4.536087474
3.858591082	4.877742314	0.814700489	0.336975051	2.895221315
5.791479544	-1.630426053	2.391219171	4.767800988	2.250138669
5.620991544	3.479134445	4.639078937	4.682326417	4.761374004
1.481232283	-3.614818675	3.301216431	-0.543322184	2.573255359	-
4.0811471735.595921737	3.165521008	0.039961049	4.699542083	-1.091009113
5.400779277	3.487691683	3.763184886	4.310766851	7.195063356
1.539954445	4.533482491	7.138217562	-5.011782356	-1.376007595

4.896859148 -2.092009095 3.941170346-4.014859584 5.177840546
2.451715624 2.863197399 4.282837765 5.730513957 2.750810895
2.138881522 -1.458235859 4.477708129 3.944096688 4.784493988
4.323419969 -2.43897846 5.062624726 6.550591923 3.007138996
0.404425048 5.477278823 -0.847091281 6.072678698 5.049590812
5.173299735 5.4055415 6.780403244 10.02565087 0.39724848 3.023796399
1.852475497 -0.751085594 3.099448689 0.546531279 4.245450233
6.87292909 3.517020209 4.942474578 -2.039207761 2.540029369
0.568370785 3.383035513 4.019594783 -2.686852423 5.342722994
4.676346639 1.269002047 3.227745094 3.629386266 5.011290673
4.743829934 4.010394556 5.33549608 4.817012077 4.960913101
4.375360558 0.389293955 3.796474499 4.36908459 4.565698239
4.722825999 5.691674617 4.66713543 1.252306795 5.0921197713.743269499
1.455512418 4.069865677 -0.822572183 -2.947552123 3.874350458 -
0.365570146 -3.692193965 -3.043780917 0.954415627 2.365455986
3.707616326 5.9427281113.724313043 6.363963681 -0.585774692
2.033003986 4.773767101 3.755097282 2.352114901
TCGA-64-5775-01A -1.48254034 4.092732168 1.91027765 5.63665791 3.794338791
8.304038196 3.898533776 4.336108992 1.719450375 4.180010914
5.259009931 -2.719120082 2.786643951 4.619109496 4.58079159
4.4311540283.771986822 4.138944025 -0.241746574 0.064175346
2.427059301 5.521050821 -2.31795307 3.878484839 4.578643476
1.949301673 5.852488774 -0.197498899 4.416417653 4.28683326
4.969015064 1.467679946 -4.004216219 4.045145764 -0.487726753
1.11360739 -4.778857838 5.63047823 3.630426695 -0.022464888 4.710080179
-1.293589689 5.033553086 2.561691939 3.518819365 3.989665322
6.745823881 0.577415206 2.9116456356.930827612 -5.714376948 -
1.783953515 5.279827706 -2.664295428 3.158791055 -4.988564147
4.879313548 1.735560861 2.713358974 3.265539709 5.778731585
1.956253015 3.29781678 -2.725433806 4.795321935 3.947512743
3.815943071 4.346874638 -4.122143627 5.099033559 6.500871343
2.500851515 0.1420119255.365437624 -1.552550813 6.010732652
4.874977761 4.85829373 5.458462986 6.509619012 11.757676280.004142918

2.807524503	1.286450854	-0.420475169	2.946326932	1.055745272
4.316341044	6.776656004	2.923534455	4.704770145	-2.578955578
2.264900623	-0.663546157	3.404169234	4.462107357	-3.66524652
5.309964661	4.905237467	0.63008082 2.826771903	3.08784679 5.041798597
5.079010162	2.710310824	5.33590972 4.055718065	5.160376063
3.901297862	0.467315316	1.608082669	4.108268271	4.822233337
4.787737679	5.269937214	4.037538001	0.89326146 4.70658211 3.542860898
1.285873736	3.635936913	-0.826060831	-4.928371139	2.67504004 -
0.973990699	-4.172018521	-3.303793012	1.065722742	2.250849823
3.941523965	4.317924593	3.1110152795.944691259	-0.871153882	1.8963841
4.352061456	3.296995458	2.591125664
TCGA-64-5778-01A -0.619180393	4.829179188	3.021283541	5.540449567
4.313984903	8.302835352	3.546916396	4.355209899	1.920581605
3.339375475	5.404558586	-2.121718499	4.174032779	4.677341215
4.90965304 5.028295033	3.7020547114.410554416	0.120321569	0.439166249
2.669037396	5.216293974	-1.158926751	1.687022105	4.564538922
2.743863235	5.612943219	1.944158347	4.632251728	4.143021214
4.764882803	1.313293196	-3.869756356	3.067742006	-0.828181168
2.900385934	-3.385202903	5.6118752632.823154136	0.060927557
4.719386909	-1.091652069	5.10806997 2.675424603	3.910704823
4.202975757	7.039602637	1.060851857	3.827400229	7.032950909	-
5.046458626	-1.84604728 4.854603546	-2.521812392	3.789667379	-4.433018672
5.057933818	1.944772829	2.920366215	3.873952454	5.9547616
2.736232396	2.060773001	-1.756828033	4.50400511 3.932155843
4.255535628	4.339077285	-1.271362195	5.07513957 6.281533328
2.862396336	0.033747154	5.729842855	-0.580749874	6.105533296
5.090401552	4.793935963	5.5074211086.340912667	10.211968040.625735105
2.70571176 0.871124892-0.640714644	3.127343875	0.86392069 4.440365562
6.85219579 3.745842536	4.886582185	-2.146955833	2.43291228 0.05483715
3.373302824	3.830721949	-2.460143843	5.341334226	4.335227852
0.337700321	3.019417219	4.168402269	4.989154055	4.7777784
3.373975276	5.335266589	5.034735467	4.841201921	4.690387871
0.771304314	2.175105028	4.606817276	4.782380849	4.742054982

5.677000315 5.342837958 1.005522357 5.550536843 4.136202989
1.900782214 3.933765469 -1.022464327 -3.919033031 2.919574078 -
0.279933492 -3.787805202 -2.966821325 1.590054837 2.614538865
4.404004959 5.308984143 3.5458941 6.080007648 -1.159261374
2.902910254 4.782945195 3.614394503 3.085439643
TCGA-64-5779-01A -1.138444 4.412827011 1.184253394 5.113940036 4.07639282
8.349583961 4.267940343 4.115749541 2.316850612 3.98665485 5.220594435
-1.968828472 2.949705153 5.130364781 4.917305983 4.516766165
4.304964908 4.371201681 0.685636428 0.476125391 2.818980185
5.833388548 -1.767127439 3.044205417 4.749559851 2.28935516
5.708027975 1.323455503 4.517191507 3.9340066 4.660739949
1.429461258 -3.636522166 3.510874354 -0.623031132 2.322732093 -
3.906620411 5.575956927 3.848696675 0.821374532 4.686686361 -1.200942597
5.504147992 3.844372494 3.760168142 4.323933843 6.970799868
1.647105088 4.145666009 6.990854969 -5.010640375 -1.260457163
4.532580504 -2.193965662 3.833003986 -4.10885123 5.123160921
2.393825574 2.55892413 4.354160767 5.882952619 2.89920776 2.30863571 -
1.673641836 4.645254017 3.942069158 4.194241242 4.401124052 -2.766519854
5.069118973 6.523979929 2.919341181 0.309304616 5.710914105 -0.646244305
5.976459269 5.113481046 4.895888248 5.385434323 6.635901959
10.32476496 0.261961621 3.058477801 1.577998292 -0.793574872
3.67850539 0.539262099 4.131060876 6.897880303 3.527440416
4.901539696 -1.749242485 2.331360546 -0.095625716 3.351321548
4.004328496 -2.749147916 5.330702612 4.942412309 2.048865239
3.571499118 3.7134104 4.923926533 4.745794209 3.575978818 5.335345243
4.911822682 4.983117421 4.228856037 0.544940095 4.151110876 4.280211645
4.571312756 4.746521509 5.619201575 4.846490376 1.273788193
5.313760873 3.645207271 1.589903954 3.831334523 -0.253210692 -
3.370141377 3.801667404 -0.31202986 -3.642243037 -3.354817469 1.062919812
2.345877776 3.641448423 5.432274889 3.396611548 6.320976386
0.023799972 2.292950403 4.603954381 3.805449103 2.559012113
TCGA-64-5781-01A -1.01097662 4.63392299 2.94661295 5.505207506 3.978506228
8.326815556 3.034376218 4.375474331 1.75647362 3.574393417

5.866885969 -2.467336376 3.982392485 4.0611751134.420499129
5.178637978 3.7304119874.1136850630.191712992 0.308904101 2.665884745
4.822252929 -1.359469435 2.451077159 4.493683615 2.788032187
5.568314766 4.200863635 4.518398143 3.670143739 4.905809723
1.253902016 -4.282927129 3.256268981 -1.159939602 2.330471827 -
2.394932329 5.613723717 2.695064952 -0.433232819 4.703922216 -
1.306358173 5.253959583 2.337816449 4.026486132 4.185184622 6.93691576
0.925700779 2.973513238 6.698716268 -5.351828298 -1.851558498
5.259195644 -2.999870556 3.310700115-4.793668525 4.966308861
2.069453617 2.496956735 3.567426474 6.150555049 2.483714607
2.356329406 -1.771349087 4.454838535 3.929867265 4.43593995
4.121808806 -2.011984877 5.081715533 6.351493734 2.815189173 -
0.094625545 5.540087958 -1.174721068 6.1675456115.06119314 4.690319888
5.497089434 6.221613865 10.91063476 1.7744747 2.180735783
0.534669268 -0.135395524 2.736642318 1.147759615 4.423823206
6.8641180273.588702576 4.887393413 -2.465032548 2.22130557 -0.585693602
3.355975461 4.068734177 -2.667682214 5.334124627 3.937673902 -
0.1110648652.8361199183.743940154 5.015965274 4.72527896 2.724237733
5.335289822 4.819660927 4.898536575 4.321700975 0.519690705
1.720529988 4.665235509 4.805969529 4.702076717 5.737726068
5.061402467 1.028357241 5.452140551 3.86243132 1.635558431
3.827890361 -0.922549553 -4.214155737 2.382747422 -0.440799864 -
3.905095912 -2.93110527 1.700903772 2.698639168 4.568756603 4.87230367
3.209592376 5.77370729 -1.270846301 2.999126442 4.483625942
3.418337638 2.841358497
TCGA-64-5815-01A -0.897278038 4.513207096 2.605618733 5.533603066
4.025944128 8.36065216 3.288997105 4.397406799 1.504715371
3.340918208 5.7646797 -2.640797862 3.644681546 4.031460315
4.560465046 5.124360412 3.100214713 3.8181178710.501685245
0.4755119682.5112437275.054690888 -1.187721169 2.810512586 4.431535465
2.930592874 5.50124493 2.2711443614.571008084 3.38527095 4.918837116
0.972603951 -3.762949404 3.73895495 -0.897229375 2.586335716 -
2.638601682 5.60211497 3.592095598 0.64915822 4.727350981 -1.270122415

5.201704467 2.24142965 4.056178851 4.235790569 6.980225464
1.259392903 2.792170184 6.532296742 -4.651190634 -1.971064088
4.789318827 -2.649511281 3.005206165 -4.522413208 4.867048431
1.980670381 2.378421558 3.687472432 6.226912526 2.670345211
2.347520246 -1.844002057 4.595995395 3.93465558 3.969799673
4.245300334 -1.162385373 5.101549941 6.319579181 2.771829774 -
0.14503859 5.995519408 -0.578529134 5.940331812 5.134694329 4.494301075
5.547258478 6.239869926 11.221042741.53759263 2.412802426 0.519589255
-0.39168375 3.200888407 1.093027983 4.383450646 6.775151812
3.571474873 4.547695 -2.3102721 1.981555705 -0.461358163 3.303702304
3.725436799 -2.502121907 5.332721904 4.699215529 0.447238228
3.071899474 4.148895853 4.931612282 4.87454256 2.724846827
5.335562217 4.90078089 4.925166557 4.238600387 0.506570047
1.797342718 4.392064282 4.783960352 4.776505576 5.596025721
5.077464779 0.658768978 5.650638371 3.955226217 2.209657908
3.794081812 -0.170112267 -4.274231788 2.827526165 -0.211928148 -
3.316553796 -2.829622605 2.0112733842.884153272 4.637079975 4.720846351
2.9546024 5.57069761 -0.073064545 3.247003658 4.230900779 3.513234605
3.330445793
TCGA-67-3770-01A -1.045321057 4.36385843 3.305983565 5.521983164
4.432995527 8.361353434 3.404706288 4.306127054 1.773768188
3.428404308 5.630603335 -2.176627774 4.057603385 4.726264064
4.778332881 5.159814895 3.334158055 4.17313474 0.201720007
0.472578617 2.763534481 5.146352613 -1.422566296 2.731069764
4.459613899 3.254270103 5.508491341 3.416589618 4.529468722
3.571329777 4.797660452 1.179116746-3.832524577 3.620273569 -
1.097470643 3.03033254 -2.952130049 5.607087734 3.194588658 0.769440489
4.732319732 -1.21013794 5.547675657 2.584343808 3.978492735
4.242323485 7.240001802 1.1195750893.252239296 6.513360709 -
4.629960577 -1.791301466 4.642716482 -2.750271675 3.714262726 -
3.7831153714.862416348 2.061858806 3.21449759 3.877391063 6.18373125
3.007003647 2.535512823 -1.452146699 4.395885681 3.945941789
4.522638034 4.021130014-2.138222475 5.081000512 6.233633517

2.936219471 0.061740752 5.986246823 -0.792787249 5.914825036
5.161967877 4.498497035 5.517806805 6.307044651 10.70696096
1.577510978 2.657505141 0.855930616 -0.750700833 2.962361367
0.9752320114.385348533 6.868842547 3.868720577 4.771198973-2.345612411
2.144121914 0.58016413 3.36433867 3.71881102 -2.705421987 5.337227353
4.505107453 0.277164888 2.900505506 4.197213646 4.986957737
4.79074829 3.310328299 5.335278227 5.2514119154.908628848 4.987250495
0.331317581 2.550463782 4.7495116014.679512546 4.759358806 5.90462054
5.435448468 1.105383308 5.662363421 4.449780886 2.144090687
3.88197062 -1.076175515 -3.594236515 3.459653988 0.276161877 -
3.382569447 -2.54073209 2.032547947 2.684670015 4.738494407 5.033312165
3.346298212 5.994931728 -0.890363951 3.033523066 4.848285148
3.756205626 3.612404924
TCGA-67-3771-01A -1.268707803 4.806912664 2.696047836 5.316940961
4.47939702 8.258139346 3.796869123 4.154792179 2.130365437
3.705679134 5.670166906 -2.01303663 3.537721383 4.8325656 4.878742111
4.960642282 4.379940927 4.354942434 0.649930631 0.465002224
2.916797958 5.3531244 -1.486367032 2.793089717 4.620753016 2.97607274
5.755068857 2.370253858 4.571776264 4.027461757 4.792712256
1.116695674-3.71291584 3.225204868 -1.061252422 2.76138428 -3.423561821
5.586461698 3.20283378 1.388801728 4.689424932 -1.287545067
5.204641717 3.560238046 3.846159525 4.351237721 7.033304007
1.598623351 3.760941893 6.998423331 -4.900164583 -1.573592894
4.702098417 -2.642716029 3.844890757 -4.529981703 5.02170598
1.898664827 2.340344084 4.156409569 6.071473799 2.920998205
2.025784126 -1.970613095 4.484892533 3.943081224 4.265926367
4.200855173 -1.993771214 5.07915793 6.359625676 2.941016484
0.088961219 5.843681119-0.94636851 6.09601328 5.104912515 4.566138398
5.482261551 6.387544963 10.40521082 1.154863736 2.788432307
0.794563277 -0.937200055 3.335100469 0.814681405 4.322499975
6.818959361 3.757204574 4.909388009 -2.040982102 2.494423203 -
0.0276681 3.335174252 3.899546698 -2.389506484 5.35349875 4.718093004
1.393703568 3.09950848 4.325289751 4.92097408 4.552763499 3.483067404

5.335288021 5.153700228 4.847342325 4.4740357110.371785692
3.800774564 4.703062554 4.594738186 4.745897967 5.948762679
5.31807716 1.195169599 5.601537042 3.922000106 1.627544697
3.849317525 -0.884918726 -3.897533973 3.075074297 -0.352905543 -
3.500006725 -3.525801603 1.614898062 2.648476005 4.366920955
5.605678393 3.648649513 6.057486121 -0.055971867 3.003200028
4.485950167 3.674583227 3.241170253
TCGA-67-3772-01A -1.130276595 4.231232207 2.53644617 5.534474075
4.4232236118.333947542 3.173853581 4.304349804 1.716523495
3.364193854 5.669916395 -2.354799532 4.024755274 4.570375801
4.623470978 5.319884832 3.592299442 3.93038631 0.472716492
0.555101279 2.6540465115.054010092 -1.516778618 2.663822649
4.475387434 3.026224858 5.494401999 2.619952212 4.41401868
2.9702000114.765876562 1.125946922 -4.003247097 3.78504237 -1.100907649
2.75583556 -2.927430961 5.598951989 3.178684499 0.638498375
4.723581376 -1.214703424 5.3140701182.705951308 3.99981763 4.320325049
7.151536146 1.25373727 2.972420765 6.611282376-4.833815058 -1.875051492
4.658603062 -2.626267216 3.339439229 -4.147086722 4.864352878
2.049082987 2.8651674113.917698248 6.179892434 2.866240669
2.261414699 -2.325780594 4.433970128 3.944169361 4.150817366
4.190876095 -2.262625246 5.0761160916.191979291 2.921246648
0.005565639 6.020929792 -0.81383062 5.937968674 5.236873638 4.32942721
5.523200924 6.216185312 10.83474673 1.66924451 2.486152734
0.835002224 -0.814209015 3.189312359 0.945565522 4.35728494
6.8161184263.852857896 4.837485186 -2.212576134 1.987558585 -
0.039295816 3.3442911673.653423834 -2.937797264 5.344249429 4.477778477
0.713944812 3.123904251 4.281800243 4.918913325 4.75594766 2.98865863
5.335092016 5.041341301 4.855200482 4.591482242 0.377523292
2.559727308 4.518703356 4.595831467 4.769579071 5.681771611
5.249491986 0.978727017 5.670535721 4.149185869 1.925125267
3.803848149 -0.587435272 -3.980944663 3.017376125 0.071415686 -
3.299107653 -2.682475396 1.931673179 2.538891855 4.648025678
4.904771313 3.3483499 5.745247038 -0.240785951 3.103004178

4.734212941 3.668716798 3.538637669
TCGA-67-3773-01A -1.080668708 4.474642014 3.249349124 5.499622804
4.231554602 8.347609088 3.4631181764.407879704 1.710944668
3.238560596 5.390715346 -2.357524698 3.906779335 4.432289813
4.759217756 5.097297687 2.8461011473.896236968 0.146969493
0.473402272 2.444075731 5.269647617 -1.449761591 2.641694556
4.494306806 2.929126308 5.819603487 1.92875249 4.443896797
3.029596404 4.607314645 0.903543254 -3.83877294 3.434134305 -
0.976372832 2.465673833 -3.001503604 5.590475905 2.91361322 0.566009033
4.723477713 -1.265999168 5.378928843 2.329835722 4.012110262
4.309778703 6.992991252 1.039423543 3.353789304 6.427265593 -
4.880401933 -1.814348304 4.42755502 -2.531980363 3.430258991 -4.44234709
4.833174124 1.802812751 2.917799516 3.96912741 6.176107409
2.702077823 1.951504969 -2.426068275 4.54333125 3.941045554
4.060488681 4.081514978 -2.089450455 5.088640605 6.305666332
2.933501289 -0.138821811 6.22574305 -0.713155501 5.948095819 5.15639324
4.353144316 5.539393009 6.275822974 10.511882161.134058469
2.648515712 0.716346005 -0.755146364 2.89223248 0.960987024
4.378128958 6.852975913 3.581704559 4.642730426 -2.305653402
2.085305844 -0.158135799 3.274607351 3.743984872 -2.778290876
5.340270694 4.930930753 0.14542076 2.967464072 4.197106896
4.964431778 4.815518262 2.912777148 5.335350465 5.169650958
4.844792477 4.514814984 0.384107163 1.554418062 4.570684574
4.62815065 4.775369182 5.675036422 5.162671968 1.145472523
5.530182783 4.489065122 2.044839386 3.797217808 -1.015715196 -
4.051660828 2.970567152 -0.017762637 -3.375738059 -2.876654657
2.025199835 2.698581294 4.5411724544.891097998 3.0809171185.81421943 -
1.099145151 2.789467653 4.56128703 3.664606178 3.180282279
TCGA-67-3774-01A -0.951280189 4.569586707 3.146350415 5.434803797
4.12092287 8.32007528 3.500643369 4.315659645 1.836847967 3.37788283
5.332001172-2.268595498 3.323293161 4.476396789 4.642051626
4.930530795 3.708213225 4.05774338 0.382724589 0.451720154
2.733705847 5.268570776 -1.451057452 2.647329915 4.48498702

3.107920971 5.734790441 2.376339547 4.464322426 3.251645615
4.79376601 0.92810646 -3.79582698 3.501772408 -1.225005086 2.650241553 -3.42554017 5.581392588 3.399539588 0.53254274 4.714263575 -1.234442532
5.262376386 2.992461704 4.031245008 4.260039033 7.049045928
1.27469765 3.650042858 6.561513659 -4.893153014 -1.734710766
4.525313242 -2.707722754 3.46227239 -4.637580787 4.907155021
1.693962049 2.166589767 4.01687885 6.121409024 2.895083264
2.467724521 -1.879293082 4.59455864 3.935774563 4.1144235624.158075335
-2.349564405 5.08350784 6.337030903 2.948427226 -0.11050406 6.133004294
-0.656617801 5.946916268 5.23271775 4.533984707 5.50466928 6.458929557
10.4645505 0.842592416 2.503909136 0.637898574 -0.810553614
3.5081192440.822273893 4.314899419 6.827363729 3.790707527
4.624029841 -2.064619395 1.93420365 -0.015606541 3.285231422
3.749777327 -2.641949446 5.348388028 4.841745857 0.903523377
3.030206416 4.37733011 4.960539021 4.692388795 3.149392802 5.33539012
5.32859313 4.814819971 4.262927559 0.46804462 2.914712953 4.732720977
4.688152102 4.74555289 5.730463654 5.40397803 0.8899427115.826633457
4.224024846 1.885624581 3.872999325 -0.424934567 -3.974489332
3.11401249 -0.395959273 -3.511915906 -3.761481011 1.633308415
2.799338719 4.4720031185.152308917 3.27395654 6.084112427-0.590575726
3.16516624 4.499523737 3.700383125 2.801269447
TCGA-67-6215-01A -0.66405135 4.820093242 2.748765675 5.437515809
4.367703023 8.218871397 3.106524809 4.325820966 1.832060217
3.2096961135.223180271 -2.279455434 3.320533217 4.728584732
4.626937848 4.962876962 3.445375944 3.644784494 0.637602349
0.36747862 2.602473279 5.236646875 -1.284417015 1.714220285
4.549134228 2.894025542 5.586814598 3.06041576 4.4744114853.499665119
4.882484836 0.909217099 -3.714405945 3.35365605 -0.907210292
2.670590965 -3.230060323 5.603633613 3.77840751 0.900022626
4.702817809 -1.219970517 4.982919952 2.843428319 4.031902807
4.286479841 6.924380526 1.352905136 3.907526608 6.598627463 -4.87225968 -1.848921065 4.849339659 -2.293148328 3.331327174 -4.648727182
5.002786342 2.091789704 2.551944979 3.921249583 5.959489735

2.400846059 1.949774838 -2.211073408 4.527240992 3.944562405
3.988498096 4.278307079 -1.201407143 5.073572686 6.268216211
2.927781293 -0.228234027 5.978754957 -0.615052453 6.099893961
5.109726725 4.647674776 5.494717243 6.336731599 10.43773604
0.746539594 2.743246566 0.764621326 -0.721747447 2.792705323
0.868427102 4.295855803 6.789757481 3.53969224 4.787401819 -
2.202339524 2.36161396 -0.024854962 3.249780625 3.511983947-2.250770276
5.35962151 4.5314121190.650690263 2.78977001 3.966458196 4.986090669
4.830029106 3.139832302 5.335346481 4.813955555 4.882622386
4.145638397 0.390763135 2.519375318 4.510227313 4.706841744
4.750387312 5.556568439 4.831552123 0.715560657 5.742570163
4.448971865 1.904520366 3.93682167 -0.683765053 -3.7050374 2.796512865
-0.08733803 -3.428512101 -3.69713068 1.61017088 2.835525573 4.342980942
5.35798967 3.340972347 6.100214688 -0.081764495 3.051272517
4.481823023 3.664329676 2.68478041
TCGA-67-6216-01A -0.870161258 4.7392201152.984006446 5.461583302
4.354837275 8.243804981 3.328193368 4.379445799 1.771268309
2.955757803 5.184458115-2.505474215 3.666814899 4.432912022
4.732972721 5.060797063 3.022252274 3.85126575 0.34162481 0.537858282
2.444288607 4.979059492 -1.21946586 1.862400307 4.498686565
3.007425667 5.77015427 2.446754994 4.516568516 3.240426439
4.873297492 0.822326196 -3.657260506 3.274375176 -1.054488642
2.895824724 -2.990365624 5.605927724 3.493277087 0.815700556
4.723942041 -1.138947882 5.238364342 2.3276239 4.087918455 4.25064308
7.046002108 1.359798266 3.69363142 6.528225092 -4.768293454 -
1.97215518 4.992721446 -2.310869017 3.175546778 -4.903290307 5.038999758
1.970704417 2.714737163 3.853134949 6.029419368 2.44144022
1.581003853 -2.5073421 4.627324178 3.939095986 3.85557533 4.279998589
-1.434954426 5.085176829 6.331392242 2.915104663 -0.150290017
6.013723752 -0.471243393 6.051392229 5.1150361754.638461831
5.532068569 6.239324995 10.46716452 0.675607574 2.719069626
0.81620764 -0.771376753 2.93067595 1.018480679 4.384603498 6.867591344
3.523581421 4.717925392 -2.052742872 2.223817751 -0.347539761

3.230370735	3.620380235	-2.621384612	5.353193594	4.679503433
0.131620512	2.797802439	4.00626572	4.985189062	4.98006289	3.013568727
5.335492643	4.757392261	4.894371848	4.092413555	0.594540593
1.827313083	4.523727499	4.756630631	4.75907164	5.575869895
4.842649481	0.906312468	5.582296213	4.426310536	1.927722498
3.92150221	-0.601307738	-4.165218686	2.738068984	0.111149646	-3.309188736
-3.534972052	1.953268016	2.889692407	4.518012136	5.187601299
3.333323544	5.991027562	-0.590346741	2.901142694	4.524529791
3.641500591	3.009057536
TCGA-67-6217-01A	0.058950597	4.705790877	2.82331544	5.3305497	4.456891589
8.244686057	3.457802903	4.221140249	2.000461664	3.300710854
5.083598776	-2.282693224	3.534817564	4.788882412	4.883127361
5.097658534	3.810772795	4.011608577	0.870023881	0.587915849
2.871509137	5.415937768	-1.123226341	2.467723688	4.584711215
3.317439589	5.49472033	3.482672078	4.526813799	3.313383805
4.985283795	1.220950848	-3.541432011	3.598598231	-1.089413991
3.169529071	-2.913443905	5.590444551	3.79894387	1.975997236
4.716464771	-1.140735537	5.323793938	3.423734481	4.029026779
4.281828152	7.016627586	1.849124983	4.110451116	6.691738633	-
4.377333976	-1.531892793	4.581986823	-2.364635799	3.451368176	-
4.305031331	5.101821322	2.343768145	2.479276083	4.109688498
6.084684737	2.889335952	1.844480822	-1.686306017	4.661031572
3.943845877	4.223164468	4.244258522	-1.231377108	5.078366142
6.321318563	2.972158639	-0.068217601	5.793535139	-0.248202878
6.040453746	5.189253911	4.629033293	5.437172752	6.413857104
10.76862357	0.892739812	2.770561535	0.966185958	-0.745434708
3.493030397	0.892407883	4.273353219	6.905503984	3.783700686
4.743653807	-1.781595936	2.269560056	0.192957536	3.309914953
3.757815538	-1.945974241	5.366192459	4.688836594	1.450857528
3.026708206	4.132300857	5.014864336	4.772574454	3.276959801
5.33547275	5.117401529	4.940424232	4.310749983	0.737942743	3.927341944
4.667031049	4.829983634	4.756914906	5.657039601	5.312888497
0.933178121	5.61226065	4.279798052	2.135122031	3.962830751	-

0.319957667 -3.2879849 3.154567066 0.05812662 -3.299559044 -3.490010361
1.693960002 3.022727483 4.485359324 5.447490713 3.48603156
6.341496619 0.708437028 3.269946996 4.635971488 3.72810217
3.213919296
TCGA-69-7760-01A -1.106762932 4.44162503 2.574109052 5.666415046
4.371977455 8.317949351 3.255974957 4.307546674 1.839529626
3.505371531 5.536760791 -2.19592693 3.338329576 4.8778432 4.667998606
5.045147684 3.351795662 3.97724189 0.394078636 0.436880628
2.663304453 5.111619432 -1.503416857 2.72522556 4.556670015 2.752199157
5.356225801 3.293184871 4.434171872 4.031201671 4.812921848
1.365885493 -3.874722466 3.658552089 -0.636798736 2.55593041 -
3.326212896 5.62358973 3.975157527 0.617236742 4.705475148 -1.325467806
5.523642525 2.885195835 3.885611959 4.229545744 7.022859327
1.012951092 3.705813253 6.3700796 -4.97437986 -1.796359556 4.942071089
-2.481710076 3.217220514 -4.132349197 4.834262491 1.931746579
2.659567696 3.870450865 5.986575584 2.674162815 2.784658593 -
1.893823179 4.425025657 3.952561908 4.193960024 4.176906465 -
1.416891117 5.081297875 6.191690378 2.805439429 0.03357713 5.772506013 -
1.044517284 6.064724926 5.083189866 4.610799511 5.515899298 6.524678409
11.02108464 1.160900617 2.677959106 1.125778024 -0.823543457
2.483710972 0.992447682 4.369857531 6.755183181 3.716967916
4.915633078 -2.244533651 2.604589773 0.093793442 3.359256856
3.685864993 -2.419635468 5.351999253 4.346450186 0.299172929
2.904606179 3.99076314 5.016302849 4.85284569 3.36411868 5.335422872
4.945462927 4.91548779 4.576983029 0.348802196 2.5234786 4.403959842
4.599185729 4.741231318 5.709498203 4.908637881 0.779451083
5.600178059 4.078872309 1.810446609 3.843014208 -0.761203851 -
3.812808864 3.349810233 -0.077813438 -3.552966845 -3.187427469 1.69199534
2.633507522 4.447672947 5.273281003 3.592892165 5.955468055 -
0.682299671 2.687965507 4.462205213 3.480196881 2.689371171
TCGA-69-7761-01A -0.792117184 4.637250678 2.149425113 5.289079628
4.021775761 8.351664362 2.854746083 4.387565999 1.306865714
3.269963137 5.548702791 -2.791556907 3.191746918 4.713839992

4.491617547	5.153091773	3.948790468	2.782767424	0.681359334
0.44660082	2.451510011	4.928956592	-1.33094241	2.272024269	4.499259884
2.742389493	5.857908089	0.620799899	4.413888615	2.830362962
4.626163517	0.588462193	-3.913304299	3.36635689	-1.139167499	2.2487538
-3.314334239	5.592780309	3.729823475	0.59203269	4.708248975	-
1.284398212	4.75085027	2.774473118	4.026573951	4.324152995	6.997475069
1.08274663	3.287018611	6.223988959	-4.670105801	-2.1510199	5.343114787-
2.581087399	2.969791217	-5.129257359	4.859394171	1.239343741
1.732329134	3.854506651	6.124477071	2.615573315	1.973887927	-
3.026525541	4.642369048	3.941012309	3.332340729	4.30895117	-1.119720967
5.089582448	6.109332735	2.958799753	-0.38413052	6.048607052	-
0.431795343	5.931732687	5.310111285	4.208365913	5.421435497	6.132842504
10.99598191	1.412638052	2.520387899	-0.090114634	-0.84367213
3.307465773	1.009997572	4.211562624	6.729786122	3.646041337
4.811228102	-2.249630749	1.818790144	-0.680895399	3.168354923
3.299161337	-2.351601054	5.348934303	4.419872581	1.213897794
3.267987687	4.483054901	4.844728581	4.815685639	2.332085546
5.335151149	5.147607621	4.850534867	3.622339925	0.423657125
2.120297794	4.32363036	4.69301965	4.813437669	5.57322014	5.124027143
0.34902349	5.870219758	4.511620626	2.103301504	3.765651185	-0.305195095	-
4.093666989	2.534272786	0.059646464	-3.299793275	-4.019531481	1.66389026
2.735834765	4.242874792	4.987677788	3.449557883	5.869311005-
0.228318311	3.255880696	4.266280891	3.573877223	3.334641111
TCGA-69-7763-01A	-1.128199142	4.576889834	2.868139445	5.479923727
4.009093358	8.393360135	3.367318789	4.340488175	1.6082726
3.056210215	5.516020364	-2.397976762	3.254974037	4.29492192
4.404451942	4.931793026	3.720329101	3.920610336	0.8181756
0.458292484	2.508308114	5.203964835	-1.565772095	2.403789511	4.501988845
3.081962028	5.830994091	2.11224203	4.448026156	3.182061744	4.90969979
0.881208341	-3.968689953	3.622000173	-1.222002546	2.859892641	-
3.092890601	5.583614752	3.430191353	0.806405022	4.712093345	-
1.305864613	5.0043092	3.053764534	3.99307919	4.25486926	6.861198675
1.548034657	3.249250814	6.562273088	-4.764391428	-1.820652836

4.732062271 -2.627992154 3.19004827 -4.828897002 4.957252031
1.875260273 2.013752134 3.954862679 6.094587731 2.71268452
2.025521919 -1.981739398 4.6116907323.932047945 3.992891631
4.123614924 -2.411241612 5.079083788 6.389260639 2.910272992 -
0.0754572 6.116655645-0.764844985 5.971544194 5.202025832 4.671120571
5.454349581 6.40936537 10.60123555 1.343518058 2.337393921
0.535620272 -0.690642394 3.248584378 0.8114730374.359654957
6.877250143 3.665792674 4.556057781 -2.023099854 1.758411886-
0.514475028 3.258049832 3.827723437 -2.764795997 5.346874452
4.695834358 0.833575846 3.034415574 4.182142499 4.894850545
4.71801919 3.169295902 5.335386315 5.10487064 4.823171585 4.013114304
0.382868359 2.841587463 4.524736229 4.708398127 4.739402325
5.690189989 5.271267578 0.8119338755.730004241 4.201887579
1.795174195 3.848558703 -0.237548648 -4.287151784 2.599571175-
0.485477614 -3.349205488 -3.876107753 1.695915455 2.686307253
4.352047401 4.807269446 3.003043125 5.860185958 -0.113452381
3.447315096 4.337672571 3.566437035 2.715387239
TCGA-69-7764-01A -1.135794308 4.616699528 2.572740876 5.234554406
4.193153022 8.340107716 3.275400726 4.226207977 1.838659089
3.270315928 5.197173394 -2.351676568 3.157171486 4.860485892
4.6758611215.001562707 4.045308982 3.597306247 0.587856032
0.389213405 2.748510139 5.15126029 -1.55609117 2.086891445 4.597737719
2.989831952 5.665022328 2.425707073 4.479506588 3.447974185
4.852306801 1.088823767 -3.790515507 3.219140344 -1.190329699
2.99999722 -3.349270467 5.588639109 3.509238926 1.26863856 4.694913307
-1.310540542 5.150439101 3.604508501 3.909647768 4.200884404
6.949367564 1.630663715 4.03808144 6.512358119-4.50780329 -1.751270903
4.55306949 -2.565830319 3.35417194 -4.660577866 5.003792565 1.844298868
2.385942261 3.970152341 5.944008837 2.873884071 1.925024132 -
2.03312354 4.531287161 3.936889736 4.047122618 4.119533632-1.664498553
5.07703102 6.302527632 2.978955776 -0.152646856 5.696098618 -
0.844366303 6.004024515 5.186730264 4.7321183575.352680523 6.427166239
10.42817267 0.976144945 2.608166258 0.597546916 -0.930983897

3.236374269	0.656642201	4.256965669	6.876496184	3.709983803
4.738814799	-1.985399137	2.073347871	-0.01648994 3.283625482
3.622838239	-2.447364029	5.349904217	4.514469287	1.174126358
2.898414662	4.194351025	4.902692502	4.641551064	3.50605246
5.335254764	5.156125248	4.852651881	3.993800373	0.400319495
4.059367141	4.528815055	4.665551778	4.738084697	5.656055697
5.488739877	0.793722697	5.850772868	3.929149155	1.83810337
3.908963187	-0.587005228	-3.453658245	2.569149013	-0.174766488	-
3.379974641	-3.895127706	1.553994873	2.61468014 3.986680885	5.333867852
3.427876152	6.173216635	0.03410409 3.078268903	4.518385748
3.609223229	2.599795163
TCGA-69-7765-01A -0.714227596	4.43970441 2.177133328	5.522897567
4.346413556	8.354644424	3.340137518	4.32361194 1.725412615	3.43661383
5.259662162	-2.378750855	3.424929026	4.574804789	4.608362494
4.949638397	3.450027463	3.715862084	0.724239246	0.558427396
2.453238419	5.442828991	-1.320793857	2.787859183	4.544295452
2.90697204 5.335628046	1.54270953 4.372186503	3.140241645	4.722051586
1.004957078	-3.944009261	3.799200274	-0.699288543	2.246226274	-
3.082067962	5.591028439	3.816767302	1.361717476	4.697659546	-
1.43023577 5.357605649	3.012507054	3.958488433	4.31060468 6.870924329
1.406856205	3.099226478	6.643092599	-4.824700543	-1.882786228
4.851748591	-2.505344233	3.261515404	-4.476057566	4.810790074
1.720189196	2.729994323	4.028966532	6.087163771	2.705491807
2.347573802	-2.436914793	4.59815981 3.940965747	3.807986071
4.344040237	-1.045494259	5.081985989	6.223842897	2.788521152-
0.089325801	5.904490064	-0.691125273	5.959296196	5.173296635
4.374825464	5.479020589	6.50371187611.1674241 0.83019377 2.555639299
0.640273107	-0.905668413	3.530308882	0.939890982	4.286124265
6.775647341	3.582847234	4.777086609	-1.992274074	1.998951419	-
0.171002148	3.27838557 3.678456505	-1.955035804	5.339851912	5.004325665
1.15805023 3.205928736	3.97013721 4.89515199 4.844024541	2.729750925
5.335198389	4.874261691	4.872145098	4.08419925 0.386147709
2.799723746	4.392185491	4.610200018	4.775190066	5.445579528

4.958729949 0.691005569 5.840807029 4.202654515 2.182817022
3.7054711270.116358491-3.784901947 3.24683029 -0.159934939 -3.307268165 -3.291983421 1.608084658 2.7113619844.293157302 4.813420444 3.243943446
5.768224139 -0.050374558 2.975145059 4.196049402 3.500494782
2.966670172
TCGA-69-7973-01A -1.129056435 4.850042268 1.437374591 5.344605248
4.121442569 8.308879369 3.986803661 4.160803569 2.408116059
3.767812385 5.240517076 -1.860725427 3.265503583 4.926861472
4.96475083 4.626790286 3.947151638 4.531442334 1.041202279
0.493748154 2.786751686 5.494463886 -1.574635067 2.43356257
4.716144752 2.5307067 5.774213753 2.892170399 4.627185339
4.526341688 4.770320532 1.271447051 -3.602465032 3.637471501 -0.643366126 2.670969105 -3.885993669 5.582297289 3.3441127250.814341648
4.694833079 -1.082140931 5.315453008 3.638217972 3.812015034
4.430075513 6.993556006 1.708204029 4.224738958 7.069084493 -4.956854443 -1.596016455 4.499916916 -2.066402493 3.670530708 -4.217869378 5.1157665572.303213989 2.433801993 4.259090904 5.813943011
2.835476402 2.238294257 -1.671579385 4.546792447 3.942830145
4.541678213 4.336449452 -2.397271034 5.066463585 6.57894084
2.944970122 0.1951144755.79786403 -0.613361755 6.010004574 5.094677589
5.032002375 5.432313367 6.609310868 9.978036794 0.869125643
2.995515841 1.613386432 -0.844839484 3.247406949 0.663430813
4.25293715 6.8883611083.58305395 4.899163302 -1.899695917 2.656377262
0.1127407253.350084331 3.947634537 -2.575297225 5.340736506
4.786658929 1.569817852 3.356617767 3.754085407 4.987371177
4.600596184 3.718451842 5.335508385 4.952163966 4.988151633
4.310699627 0.445232447 4.065042147 4.412022959 4.546424197
4.74517388 5.7221188674.735147244 1.23262295 5.366805936 3.989913782
1.536590834 3.974771316 -0.753058198 -3.260097515 3.482879577 -0.452728903 -3.539989401 -3.448509107 1.086585034 2.552478183
3.885305256 5.685724634 3.442101735 6.235757087 0.220750605
2.476878073 4.650659497 3.777723617 2.284945999
TCGA-69-7974-01A -0.572046421 4.741209446 2.203308263 5.405906095

4.318504731 8.243723592 3.167151086 4.302543562 1.606379558
3.37189112 5.415975335 -2.500332 3.706932122 4.696960085 4.812054686
5.0119372483.279571663 3.372085083 0.519070158 0.438337793
2.507566472 5.145341997 -1.333907694 1.937700273 4.560255926
3.011116202 5.643631903 1.19661734 4.451566736 3.314612786 4.955620641
0.868019294 -3.567349294 3.244959109 -0.865374467 2.522600009 -
3.005651766 5.60031657 3.562963181 1.090031837 4.710285272 -1.178951299
5.018312558 2.504532016 4.056457698 4.193190798 6.916006026
1.3365681183.319630474 6.797102884 -4.607683747 -2.057997116
4.966144203 -2.398639498 3.377062771 -4.864317308 5.102330096
1.76530453 2.659273373 3.804182207 6.055205919 2.428196514
1.756547825 -2.203060788 4.659182489 3.937643769 3.672955841
4.332386909 -1.596255662 5.089120516 6.229570048 2.808156126 -
0.087253622 5.805783416 -0.387831708 6.0596811724.974321386 4.653444539
5.515546704 6.281519417 10.831132520.737089395 2.761818001
0.349765031 -0.716751814 3.518200622 1.1080038 4.31011522 6.816007045
3.190048298 4.779316475 -2.014272324 2.215082134 -0.102185408
3.213524033 3.598692352 -2.559095786 5.338199688 4.652767808
0.837463208 2.885226976 3.97957766 4.985724863 4.7885951192.937316131
5.33547611 4.670046492 5.0426811953.947226841 0.596282768 2.160315838
4.688527987 4.838189184 4.771869634 5.479917137 4.776547645
0.703250472 5.601734355 4.589817533 2.022553215 3.83378746 -
0.700475585 -3.749828802 2.859194281 -0.070410249 -3.369507525 -
3.613762972 1.764606641 2.773791234 4.398159977 5.043948496
3.091946463 5.897437824 -0.453445979 3.020443677 4.277094379
3.588479561 3.507965
TCGA-69-7978-01A -0.547067444 4.713841381 2.348109399 5.355953775
4.16893213 8.277929067 3.394033668 4.333744109 1.598689132
3.436149744 5.515937292 -2.739225009 3.4949115634.464048034
4.879746773 5.084060279 3.648018659 3.623223778 0.488195794
0.47390977 2.556206292 5.027791682 -1.253691859 2.657515942
4.5170118732.796602259 5.604161827 1.489849218 4.537196933 3.61180988
4.908197659 0.97521756 -3.685093492 3.446697049 -0.865857627

2.346351513	-3.000928386	5.60048811	3.591061045	0.954789703	4.71117404
-1.214244946	5.121828561	2.709601039	4.0112160184.172359666
6.910498055	1.3781911113.270078145	6.643564986	-4.783686527	-
2.0121160035.004033108	-2.448415654	3.062621374	-4.901468026	5.068599898
1.914002956	2.243206026	3.734174493	6.0943115672.60967741	2.140076291
-2.548813887	4.720313651	3.936518559	3.664265383	4.492057226	-
1.328470259	5.09998268	6.212642522	2.78499541	-0.100921247	5.746163601	-
0.48438688	6.04669151	5.07498173	4.585441565	5.494236087	6.271715278
11.109144390.800394857	2.612319587	0.579720956	-0.564142649	3.59318489
1.085885365	4.315133992	6.804950202	3.434730226	4.787529419	-
1.991296796	2.218914714	-0.580363462	3.265926444	3.753260032	-
2.318052049	5.340769416	4.5655116621.061004994	3.132017562	3.962330182
4.947693769	4.905197092	2.74573311	5.335551277	4.638169092
4.956632905	3.863449256	0.647922069	2.428926925	4.378215068
4.868046182	4.775459187	5.410938535	4.849302188	0.670932133
5.669445131	4.210687257	2.12845984	3.800327481	0.026782955	-
4.10818647	2.597582345	-0.283132881	-3.493321809	-3.507089009	1.611705055
2.875632479	4.390286789	4.952123515	3.27103312	5.956368107	-
0.0431115063.072721986	4.276002638	3.5278114123.313398642
TCGA-69-7979-01A	-0.373594717	4.901797465	1.887708042	5.26932135
4.603553509	8.218923927	3.803137394	4.066458939	2.283812464
3.461880895	5.073662535	-1.931779062	3.704342281	4.637272601
4.78003247	5.014945717	4.350444204	4.489594403	0.270499481
0.433263201	2.967545766	5.435971791	-1.023881978	1.828125905
4.690340243	2.803107612	5.338994458	3.206982548	4.739121415
4.342904121	5.2110081611.598204856	-3.644063894	3.229351917	-
0.7526081182.948290933	-3.437656683	5.610765759	3.777482227	1.268630095
4.698996037	-1.18463267	5.161019078	3.542634923	3.82920237	4.242373195
6.967938481	1.913314612	4.079041207	7.499033811-4.737340909	-
1.536693435	4.85536065	-2.560517771	3.820822306	-4.336715425	5.357978167
2.675359478	2.975393041	3.989884747	5.88943465	2.649522694
1.596642603	-1.326774307	4.668847816	3.925324881	4.484208607
4.284841832	-0.602682544	5.067982225	6.530304485	2.839190085

0.254683683 5.208771106-0.54014357 6.193086484 4.957587431 5.165957255
5.412220278 6.45273552 10.13238768 0.555161252 2.838351838
1.264514491 -0.619985979 3.0711470560.734484069 4.344533041
6.919645513 3.54054155 5.013220384 -1.763222704 2.527399091 0.23260841
3.372137529 3.920278444 -1.764864376 5.352109227 4.347297038
1.501281229 3.071941976 3.463995408 5.012379081 4.742337272
3.758570059 5.335413986 4.465816265 5.062699001 4.282986634
0.863428079 4.145694888 4.589769167 4.887698195 4.707637953
5.787854742 5.277334001 1.213465076 5.285902224 3.864368378
1.878883763 4.068889816 -1.075049984 -3.307048752 3.11656346 -
0.396986579 -3.746204718 -3.272734333 1.321432489 2.733700977
4.313965317 5.453776379 3.74649818 6.460927669 0.1198814152.796299918
4.528703455 3.685151557 2.533370953
TCGA-69-7980-01A -1.095562505 4.607570085 2.265999438 5.25972044
4.270448907 8.287094294 3.597357047 4.267039672 1.742172997
3.067101765 5.33275897 -2.562698081 3.659901745 4.416984153
4.620820974 4.98221355 3.878616418 3.955240149 -0.320807048
0.446230123 2.763978961 4.963711628-1.269958796 2.348399354
4.496077957 2.825139965 5.732545707 1.813788146 4.593764388
3.724848572 4.987250496 1.070050753 -3.621501129 3.17038635 -
1.128760722 2.732621141-3.498341258 5.589702842 3.8368611250.14449277
4.705999147 -1.17566015 5.208702883 2.742460664 3.97370366 4.17490207
6.973267598 1.415582675 3.828804486 6.721819196 -4.860022867 -
1.985779785 4.711043323-2.749349807 3.148679257 -4.908414405 5.139871691
2.059470157 2.047499567 3.844815716 6.060309434 2.956100673
2.036249322 -2.899981801 4.625856333 3.923636821 3.895818078
4.36537833 -1.511493197 5.085079741 6.307433079 2.88929753 0.005245553
5.728851499 -0.639003373 6.063652273 5.175034523 4.762870585
5.463372469 6.325143001 10.22728196 0.660258881 2.537515918
0.80095057 -0.787519857 3.456097427 0.797423829 4.356316408
6.835835065 3.701725379 4.842039605 -1.888364234 2.175627287 -
0.60246015 3.263908853 3.552260928 -2.608364605 5.359302787 4.157503449
0.850099163 3.069847276 4.152901068 4.974948356 4.726833273

3.361568108	5.335450635	4.817706507	4.886145918	3.872549167
0.721652792	3.270701893	4.481716231	4.847650741	4.714469001
5.753993042	5.382729788	0.938399218	5.740869126	3.725592701
1.866557098	3.94846758	-0.748241961	-3.902010455	2.411469066-0.215859866
-3.647948382	-3.66084709 1.5649136112.683977989	4.3461151245.239735622
3.771551408	6.131277644	-0.009171333	3.001232654	4.423355209
3.584105221	2.303745964
TCGA-69-8253-01A -0.894766289	4.883560629	3.550000184	5.451538093
4.229401328	8.296539166	3.419287127	4.349197406	2.003669486
2.908361389	5.471498233	-2.269510301	3.753688254	4.233546186
4.481003666	5.144230088	3.619382947	4.452143086	1.029440784
0.495085683	2.5826114415.083714902	-1.386012515	1.917473776
4.5366321153.192799324	5.663134588	3.539028883	4.48142975	3.448404148
4.952658965	0.944527713	-3.934525509	3.513814151	-1.254140413
3.022732523	-2.445947527	5.600178452	2.893553236	1.060977592
4.710791504	-1.223829261	4.85079188	2.969939178	4.123269929
4.2417611126.88168885	1.517959662	3.754865393	6.747229028	-4.759997832
-1.599554258	4.891075591	-2.269937046	3.239253789	-4.710123931
5.085842748	2.442273108	2.146609226	3.77284693	5.993806793
2.676518281	1.792588704	-1.938595665	4.54985711	3.936870403
4.363532522	4.174454667	-2.296044966	5.073148879	6.363728174
2.990271324	-0.130728622	5.955092242	-0.885661129	6.177227124
5.106519345	4.708104925	5.5063118226.318343443	10.57280531	1.07383247
2.4581114020.762654397	-0.62138112 3.102893648	0.912109297	4.414586755
6.854862993	3.725305721	4.588857006	-2.077891787	2.284390158	-
0.218454716	3.26967826	3.851452807	-2.625944388	5.36440903	4.395481336
0.329647096	2.63124956	4.063185942	5.032899826	4.729843307
3.307340025	5.335432189	4.983337006	4.762900154	4.325608922
0.465321042	2.659552297	4.662793575	4.7727126	4.702875828
5.722597534	5.223077507	1.082822016	5.598509705	4.320820713
1.640575506	3.941301322	-0.443294056	-3.954666782	2.526291539	-
0.390816675	-3.36356745 -3.341090004	1.899480415	2.933031892	4.527297986
5.125758779	3.370744247	6.145308439	-0.967429597	3.30103606

4.533597311 3.609591453 2.627481669
TCGA-69-8254-01A -0.685238038 4.945810184 2.738942145 5.373012341
4.37267947 8.324129373 3.268180163 4.346398721 1.829178116 2.917963816
5.477132192 -2.398363468 3.736990925 4.382118647 4.758653541
5.266967656 3.312474829 3.750917332 0.840839202 0.578686705
2.61333598 4.906848337 -1.058311867 2.105212848 4.490174015
3.145141365 5.812400359 2.797092513 4.517285365 3.041241578
4.812492897 0.639400365 -3.630269554 3.646971348 -1.267113368
2.899129717 -2.611647073 5.590615181 3.463874885 0.956227341
4.722059839 -1.132790048 5.130246845 2.664717118 4.168153166
4.403260993 7.04466565 1.620302415 3.642190556 6.451363033 -
4.377833394 -1.910599324 4.642278874 -2.258684634 3.074418432 -
4.672804783 5.041302994 2.050821815 1.864281372 4.025001157 6.175668194
2.871351441 1.789685875 -2.203288566 4.595528506 3.939639726
4.026699834 4.292872494 -1.07015759 5.09041077 6.244731355 3.082155312
-0.341541437 6.210786196 -0.335372645 6.028118171 5.264487115 4.415052445
5.526129174 6.198393159 10.43310929 1.375743258 2.590681463
0.594987447 -0.841100291 3.318337825 0.998433434 4.366653845
6.815774716 3.822123542 4.653465494 -1.998015187 2.185239648 -
0.346322745 3.214933106 3.44728871 -2.205631742 5.360451963 4.620333155
0.766252795 2.990339766 4.490863058 4.979098286 4.752597099
2.859046682 5.33548645 5.2353144 4.817160304 4.188716907 0.533005164
2.540183093 4.57752117 4.733363488 4.761626644 5.857580318 5.30002102
0.852250853 5.823686264 4.578399111 2.124830806 3.943387831 -
0.489484699 -3.840433469 2.614422266 0.023426732 -3.075408083 -
3.445868502 1.967053968 3.138849841 4.627014665 5.233339439
3.463437677 6.022359759 -0.152001524 3.316374813 4.521705498
3.7455652 3.018596026
TCGA-69-8255-01A -0.409256124 5.438724603 2.878396874 5.362654955
3.944727584 8.224174714 3.695991454 4.207292805 2.330750134
3.846970197 5.545764299 -1.834935979 3.403957631 4.707491019
5.301521091 4.695468262 3.901821861 4.278019137 0.174764606
0.458922302 2.744014851 4.889465892 -1.669651468 2.831389175

4.700619293 2.436502942 5.983224113 2.478836797 4.581726108
4.861257079 4.61606397 1.162477295 -3.322650201 3.021626089 -
0.87130189 2.096632605 -3.043002901 5.583598446 2.362577634 -0.226196629
4.683139476 -1.013451057 5.170342734 3.012303213 3.914388963
4.296900143 6.71321783 1.243035987 4.509888682 7.147385798 -
5.31975476 -1.810656192 4.452076605 -2.308974429 3.989948295 -4.741050964
5.257404232 2.026829071 1.503118941 4.090153137 5.816516334
2.919674438 2.693631728 -2.347199759 4.72693158 3.93521375 4.185131743
4.429939469 -2.894070548 5.102643385 6.45738362 2.885010082 -
0.143175134 5.699172972 -0.764982548 6.277377253 4.971254561
4.909122991 5.473265558 6.415578927 10.0058584 0.429150547
3.029279921 1.016747209 -0.468220185 3.706712926 0.977802412
4.246705511 6.920000864 3.481370038 5.025197943 -1.803580917
3.014605859 -0.393682372 3.303861693 4.173089666 -2.598537877
5.345877955 4.140203723 1.066913478 3.103544053 4.131053633
5.125304136 4.490254085 3.235559749 5.335859409 5.20225012
4.803504118 4.35751784 0.78420124 3.075113361 4.639018452 4.720450586
4.740826168 5.697718725 4.855769456 1.350673438 5.272244922
4.103248131 1.498728976 3.773718891 -1.579371211 -4.035841411
2.479957466 -0.778122356 -4.052905041 -3.633668037 1.305026118
2.674711775 4.073832615 5.591298588 3.629823687 6.515838832 -
1.167186486 2.208664584 4.430791536 3.846038948 2.748364407
TCGA-69-8453-01A 0.071155801 5.030403543 2.938758494 5.293273492
3.975412338 8.292927813 3.400860562 4.386668898 1.734990347
3.358090413 5.476647205 -2.480346584 3.920131764 4.185370385
4.83514404 5.027670614 3.468467625 3.818273137 0.561384637
0.589527899 2.670301583 5.13140055 -0.877290477 2.40676009 4.455260012
3.051487496 5.942409015 1.97595233 4.625055781 3.11452967 4.858791662
0.832738273 -3.447116726 3.30840767 -1.337170194 2.600289066 -2.858624
5.58127338 3.173955651 0.685123269 4.726600325 -1.006611855
4.904827944 2.390582889 4.140624451 4.353889277 6.9887966
1.398908109 3.581704586 6.790458291 -4.65342419 -1.835828532
4.547537891 -2.438921411 3.322762099 -4.734695348 5.166500916

1.883515187 1.93832903 3.882047967 6.23887277 2.76298731 1.84333589 -
1.710688725 4.747183347 3.924725871 3.875470151 4.35115808 -0.993153893
5.099789251 6.408600264 2.922578703 -0.232261528 6.194045274
0.188192968 5.987701414 5.188151944 4.558942358 5.497803015
6.095276215 10.41903845 1.308808945 2.596651662 0.43854225 -
0.440157285 3.748504481 1.047563695 4.317732479 6.842790241
3.609854155 4.622251667 -1.976697895 2.009892424 -0.455700932
3.233487675 3.721908034 -2.247278718 5.346167978 4.69699028
0.941695178 3.134339053 4.450200696 4.979708964 4.677337352
2.663053469 5.3355441175.223774525 4.884293504 4.225486444
0.863868017 1.831219855 4.663568265 4.86712366 4.78568539 5.693514189
5.21094045 0.968866636 5.633751961 4.410743299 2.327030421
3.870106199 -0.368607536 -4.146504663 2.726273452 -0.324081385 -
3.399868614 -3.385050673 1.965607267 3.0286117654.666587144 5.055178514
3.1168213566.046878877 -0.346267252 3.191633297 4.480345944
3.795851257 3.314763764
TCGA-69-A59K-01A -0.625245043 4.884561827 2.614807395 5.423561528
4.386495292 8.147045831 3.903333386 4.178368533 2.070544116
3.707756022 4.92075971 -2.139468278 3.581871799 4.840169839
5.138739793 4.924491773 3.629075578 4.157586298 0.56044074
0.335694576 2.870186925 5.379047044 -1.103033484 2.316702913
4.633827969 2.853524254 5.797697231 3.025738374 4.6504321
4.319318175 4.829029446 1.327456475 -3.720306918 3.616794976 -
0.787416303 2.998340744 -3.300608953 5.599809047 3.518493362
1.248984599 4.700632909 -1.177450903 5.103699971 3.165776369
3.925597561 4.170942005 6.857963894 1.661799736 4.067018303
6.984268137 -4.983178736 -1.649840554 4.948156247 -2.234071158
3.414747755 -4.358576882 5.233845029 1.956385852 2.606267439
3.865032174 5.912019362 2.686154463 2.026166452 -2.132568601
4.594584244 3.93580227 4.381061505 4.621665656 -0.616669997 5.08688912
6.284194469 2.886747991 0.105973062 5.761889911-0.755396628
6.1184295925.049609994 4.80423341 5.4725115386.445533853 10.55879984
0.141989598 2.848395054 1.225827791 -0.686498135 3.453399379

0.819627601 4.341561857 6.889455726 3.465006918 4.896945018 -
1.979412924 2.756501278 -0.003892662 3.342247394 3.782303318 -
1.770249571 5.341921216 4.52503226 1.170515532 2.908789053 3.581526465
5.045355898 4.828916126 3.437924426 5.335475185 4.777339455
4.959250718 4.48051328 0.596501987 3.12860059 4.64367352 4.829291357
4.767583586 5.647251302 4.866983697 1.108271975 5.364563808
4.140001738 2.098144778 3.972845681 -0.908471196 -2.995736188
3.431505382 -0.057651386 -3.614809844 -3.657557827 1.371322195
2.72186936 4.182270533 5.563761438 3.665469987 6.320286673 -
0.578351839 2.8072801144.42617693 3.606494735 2.780492703
TCGA-71-6725-01A -0.204938295 4.945210584 2.238590366 5.426589154
4.81125506 8.146521875 2.8221135634.225248222 1.809300127 2.915553834
5.367349307 -2.462616592 4.082309996 4.679576069 4.808679368
5.437873669 3.812996175 3.750819504 0.519902455 0.57889367
2.781421575 4.94055513 -0.931230373 1.741990178 4.563479459
3.357216321 5.380077713 3.47266917 4.586249857 3.890473295
5.1451114381.153947755 -3.62076481 3.181314293 -0.960644958 3.118069324-
2.967133563 5.612099083 3.525471362 1.616484742 4.71239654 -1.223346601
5.105100972 2.981237973 3.990821081 4.212714955 7.063248187
1.747602012 4.163906079 6.856242104 -4.35087173 -1.991699173
4.996350163 -2.468570166 3.246658464 -4.772393922 5.231637112
2.2117688332.685916695 3.801208079 6.057648732 2.5339811321.543540817
-2.273388953 4.558385341 3.938261712 4.197628658 4.308489804 -
0.545788738 5.065013207 6.288652957 2.935509856 -0.051060433
5.533656695 -0.477420343 6.18861488 5.078190361 4.921416055
5.474219249 6.291987385 10.7618031 0.981737614 2.775359781
0.651034258 -0.796123742 2.837051365 1.030085486 4.421662678
6.9012660113.679829034 5.010621078 -1.866902103 2.610772887 -
0.075718459 3.264357285 3.516343725 -1.814314447 5.367467946
4.034868089 0.62028649 2.663204279 4.090141229 5.047610408
4.745588781 3.350548083 5.33534809 4.615515862 5.00293884 4.018043184
0.777836145 3.302439485 4.6900221184.839813064 4.716365467 5.83901763
5.0763118470.87271502 5.662368071 4.508891286 1.915852957 4.085607548

-0.878229791 -3.484508341 2.38504357 0.181405471 -3.301036474 -
3.615522191 1.710997886 3.097043577 4.755986144 5.632894241
3.471889626 6.364582847 0.498957228 3.230547679 4.566123987
3.601560794 3.12323406
TCGA-71-8520-01A -0.376189421 4.514796244 2.231284339 5.312087421
4.299691822 8.269319284 3.265041259 4.267017178 1.821339774
3.478428733 5.40762961 -2.293486807 3.673839239 4.618102275
4.868821025 5.325806719 3.740925159 3.788020759 0.33905706
0.581367658 3.008673319 5.193136039 -0.845553645 2.483220342
4.563981539 3.205935859 5.407926948 3.024052038 4.616277044
3.304585612 5.042958374 1.483648483 -3.409649999 3.622561242 -
0.942906242 2.859130305 -2.664275967 5.602598722 3.653773423
1.271796469 4.716718925 -1.12881156 5.105525839 2.875898439
4.028777537 4.249362189 7.018274062 1.644953074 3.726655548
7.004893235 -4.394637928 -1.723594589 4.91862208 -2.376801292
3.319297596 -4.043892099 5.160934912 2.389538807 2.726740387
3.881303762 6.1155873862.675876652 2.269586957 -1.479634497
4.647923542 3.936332531 4.45403937 4.340570238 -0.230683365
5.081347753 6.379525513 2.845264604 -0.029986322 5.586872622 -
0.474927847 6.056539169 5.059329657 4.828326541 5.524820808
6.41136883710.86333599 1.007770866 2.863516031 0.819705507 -
0.499044207 3.409337861 1.008248529 4.318526135 6.871787371
3.580863916 4.93255404 -1.91494982 2.315280836 0.180635098 3.36569906
3.666459715 -1.718587658 5.3415973114.335241848 1.2589118243.172194445
3.920453399 5.021215738 4.818459379 3.402718937 5.335546634
4.807647781 5.063951797 4.366751564 0.919565792 3.385175906
4.648079061 4.886395393 4.730437758 5.607352455 5.232632976
0.925932279 5.608961996 3.815316135 2.244093152 3.949052053 -
0.4332168 -2.85117256 3.358584397 0.17742594 -3.292062889 -3.035349409
1.763677003 2.893012619 4.549556884 5.323709573 3.160400588
6.26704824 0.185303292 3.161770348 4.519320667 3.724104581
3.308831795
TCGA-73-4658-01A -0.677723179 4.493252169 2.766471377 5.640206351

4.16741438 8.363329583 3.176569964 4.427099493 1.538517409
3.403406416 5.765080865 -2.502103861 3.928068656 4.220404843
4.658846359 5.1129611 3.191719657 3.831714657 0.496109433
0.542878846 2.482692412 5.173331155-1.331797102 2.902100343
4.459033682 2.988004312 5.67689625 2.273199031 4.4937721 3.487721189
4.814445057 1.043197627 -3.8267686 3.883439442 -0.858512824
2.348314467 -2.841282271 5.595355885 3.203969172 0.64351038
4.718187525 -1.307904771 5.303588961 2.417823379 4.058220363
4.280884959 6.846510471 1.1921144632.617380933 6.584346543 -
4.922029177 -2.02074583 4.58276377 -2.601108664 3.200431841 -4.584206138
4.892444754 1.875974479 2.333671652 3.753097069 6.222235051
2.681544546 2.694888497 -2.164471142 4.633918496 3.936691888
3.94183473 4.340160889 -1.970313856 5.09575375 6.32011428 2.760752672 -
0.131818063 6.106900739 -0.620754665 5.965267397 5.120804182 4.41923675
5.584094923 6.317397241 10.98587094 1.522332196 2.475009072
0.591512716 -0.494238625 3.509352761 1.198613999 4.403014112
6.802189526 3.573100954 4.755277247 -2.14833663 2.086390331 -
0.44865839 3.305769458 3.841223153 -2.496403149 5.335896567 4.753903748
0.523906823 3.137538793 4.12558003 4.967588271 4.740896736
2.601867438 5.335479487 5.078608871 4.974760536 4.254657738
0.507539215 1.879907999 4.517794313 4.761046771 4.765807741
5.606423258 5.031330779 0.796089189 5.838365655 4.232929832
2.140767225 3.741452345 -0.07914831 -4.072739844 2.994427015 -
0.304922432 -3.46206606 -3.174853049 1.81222756 2.89369293 4.70293406
4.544301985 2.87845413 5.710507326 -0.198783296 3.128307283
4.265959962 3.539272909 3.219827018
TCGA-73-4659-01A -0.696129072 4.787880961 2.860685095 5.430914875
4.190301358 8.308574935 3.509938719 4.2864206 2.038308496
3.647630335 5.517998598 -2.104007178 3.765548281 4.419077034
4.8227211065.051339432 3.484865061 4.300226316 0.609942338
0.517195785 2.764135396 5.334502587 -1.337127493 2.742598386
4.566465178 2.8611917195.744748626 3.447717045 4.558398902
3.713815613 4.815638897 1.128835348 -3.709620633 3.642235899 -

0.992783512 2.575649805 -2.979874064 5.591505475 3.073132527
0.752617162 4.705484302 -1.176535016 5.123842398 2.995312691
4.022659598 4.3411125657.001840004 1.5750206113.764718328 6.919982352
-4.933196155 -1.701667678 4.54407983 -2.291370816 3.569018582 -
4.377338071 5.1137203692.149695909 2.539161712 3.920598979 6.097728712
2.738362744 2.537787117-1.742865011 4.6305113393.934432524 4.468617777
4.291514166 -2.069482604 5.080761729 6.579276515 2.878618125 -
0.121509766 5.989308875 -0.50740377 6.027602144 5.11120818 4.796047065
5.506838773 6.436777803 10.28538528 1.105865264 2.647115503
0.961782969 -0.601486059 3.502564789 0.888396865 4.289984046
6.869437612 3.622423206 4.835852862 -2.004852721 2.286059305
0.004172476 3.327247463 4.001448099 -2.370265104 5.33741172
4.737655875 1.126740549 3.0362115753.890848385 5.0211271294.643492469
3.128166702 5.335462525 5.016401863 4.959222199 4.429399658
0.619538658 2.973960213 4.68333856 4.686452619 4.746788303
5.707651401 4.968824047 1.237859648 5.593981212 4.050641096
1.828121735 3.871015508 -0.50808103 -3.690851621 3.286473582 -
0.388019932 -3.519376757 -3.273680875 1.649864137 2.76198972 4.458848452
5.31508798 3.223805253 6.169152925 -0.535911611 2.744214442
4.519395368 3.734653408 2.811868988
TCGA-73-4662-01A -0.512311776 4.659443859 2.884803242 5.416457896
4.404010938 8.316481742 3.388607034 4.284156138 1.886571367
3.32904511 5.300780976 -2.212048629 3.351496602 4.869847684
4.943075884 4.939977366 3.784969535 3.942268569 0.542545159
0.4835581192.753073992 5.521747938 -1.329593013 2.417239331
4.596353726 3.1039226 5.555956053 2.332396567 4.500307607
3.674899061 4.938882773 1.131871446 -3.582299203 3.402795264 -
0.915859506 2.848002568 -3.529962732 5.591908424 4.007397574
1.0621170784.707630725 -1.253016555 5.33334839 3.165193696 3.967012186
4.2304117466.934387423 1.479741451 3.816889084 6.738684815 -
4.757301359 -1.795733666 4.6250292 -2.43201249 3.615877562 -4.44813569
5.024481626 2.134431316 2.351814458 4.028350952 6.001784518
2.775725899 2.35642615 -1.837299408 4.640561949 3.946880361

4.041278848 4.274078669 -1.642610119 5.086455386 6.280316694
2.90267579 0.01952572 5.809868443 -0.506955795 6.067025529 5.11583692
4.676069128 5.482762625 6.516373608 10.461229 0.713535122 2.88326302
0.969871235 -0.787113253 3.34161557 0.934863128 4.301964847 6.82579287
3.64843871 4.721831196-1.847192669 2.376009459 0.027437264 3.284701755
3.748865843 -2.25458253 5.35610883 4.752034614 1.0548394 3.105416961
4.082013622 4.978269183 4.795920667 3.392746094 5.33551056
5.121762313 4.952138828 4.343909707 0.608983177 3.48288515
4.578142567 4.751295301 4.749413883 5.591302174 5.128975967
0.748018601 5.679897845 4.285284905 1.975618853 3.902298823 -
0.299850921 -3.348053685 3.011577088-0.201606904 -3.507878204 -3.676232768
1.539077826 2.9597409114.357602218 5.2118847563.477333018 6.176339812
0.162696123 3.043151441 4.552500042 3.690375412 3.072215636
TCGA-73-4666-01A -0.441438629 5.078333516 2.418856902 5.547959168
4.367290333 8.053304482 3.359944521 4.282535944 1.825500468
3.553276153 5.251367867 -2.487720286 3.974337665 4.748953539
4.918844028 5.1142152283.499322399 3.963066339 -0.104803251
0.318072225 2.528173342 4.491701606 -1.253467316 1.913972174
4.567653732 2.6538611325.744262478 2.936895801 4.5871171594.601046069
4.960894301 1.134703077 -3.639940634 2.987233911-0.827396352
2.122953857 -3.487470967 5.625163748 2.737303956 0.557044383
4.707120069 -1.12528269 4.976558689 2.534502761 3.893816195
4.155518883 6.963559874 1.278583776 3.934044901 7.090542178 -
5.366690391 -2.290359534 5.33098463 -2.371789249 3.1704225 -5.186283651
5.123552574 1.906067732 2.960632877 3.449288737 5.912776247
2.355739437 2.042893933 -2.670754014 4.604891654 3.943069614
3.692463063 4.557273194 -1.018373377 5.087423128 6.222036379
2.641069085 -0.009842401 5.590395066 -0.622564221 6.209400827
4.892093104 4.739126788 5.527321012 6.077334487 10.56331855
0.504496364 2.886309318 0.799912339 -0.674411414 3.228729666
1.191651683 4.385500593 6.832926486 3.334170803 5.094391615 -
2.1701193952.9499409 -0.534218631 3.2861514 3.742921947 -2.263639408
5.354175731 4.033271905 0.537213329 2.623256443 3.550990589

5.053783567	4.853981458	2.852010615	5.335569135	4.049708631
5.017299792	3.927090836	0.775343455	2.188888651	4.565611858
4.902730912	4.76894571 5.670282326	4.533188773	0.972676291
5.221215841	4.2721190081.8211223983.92498251 -1.245886386	-4.207247269
2.281337377	-0.085377423	-3.911089213	-3.543368121	1.604756444
2.903345327	4.525841099	5.464607678	3.543219517	6.146852985	-
0.8299611032.336856493	4.476972871	3.46897612 2.79234672
TCGA-73-4668-01A -0.970193353	4.532061819	1.881812778	5.458516324
4.452554655	8.240587295	3.224727983	4.234758565	1.820497702
3.585372582	5.260820633	-2.501518719	3.7289116394.616061314
4.500536272	5.066773591	3.907617929	3.804351978	0.672392371
0.469935596	2.700718201	5.242807855	-1.49634336 2.381393406
4.545870342	2.760337685	5.035406754	1.984674185	4.495826774
3.913998921	5.0242511371.339761908	-3.83890628 3.382129039	-0.723575638
2.534200257	-3.273480409	5.621659471	3.913915194	1.507858418
4.695619866	-1.331228416	5.266606987	3.1145009993.881414543
4.133473416	6.870612079	1.34783492 3.465792746	6.690722466	-
4.795628105	-1.868147715	5.101683492	-2.410559143	3.198763969	-
4.82029798 5.026233447	1.891228749	2.607005826	3.787678391	5.938412601
2.5811757542.210362506	-2.15943213 4.577496364	3.93722783 3.819880031
4.399085123	-1.907473118	5.07348353 6.2539116752.804191852	0.077282977
5.266670801	-1.018882804	6.102804773	5.051363265	4.686681594
5.4821179296.330849101	11.041774860.806121325	2.649914757	0.708207912
-0.696964367	3.200054723	0.786291986	4.369635858	6.81050213
3.593577223	4.905975586	-1.978189286	2.197891507	-0.198117348
3.328405954	3.609471035	-2.502789015	5.350405898	4.279083651
0.938091003	2.845897024	4.050975361	4.945722933	4.858600809
2.996043335	5.335285484	4.685088662	4.80973717 3.792357477	0.51600572
2.872759438	4.294485952	4.709815624	4.71655333 5.536847305
4.936734821	0.853635849	5.542806813	3.658531962	1.764689761
3.804347683	-0.264779788	-4.076752788	2.451899837	-0.19713845 -
3.52302192 -3.520143329	1.430583222	2.639299371	4.251602213	5.004039397
3.448276738	6.098406635	0.196089597	2.767517407	4.36922316

3.510854824 3.055211177
TCGA-73-4670-01A -1.163100845 4.668563905 2.9766738 5.685144957
4.097995514 8.182294319 2.902512455 4.269721097 1.662760366
3.298276385 5.613055348 -2.363965662 3.567966941 4.308044865
4.405271399 5.123006004 3.57661237 3.751710186 0.353518039
0.321861446 2.418528149 4.679547591 -1.65605709 2.105788175
4.548832181 2.595694951 5.498974965 3.621190631 4.338924819 3.46939225
4.722625107 0.918540064 -4.182838605 3.207776474 -1.132372552
2.097079104 -2.84293862 5.621176101 2.45115427 -0.377741204 4.689761058 -
1.341456732 4.817863246 2.629692278 3.972278266 4.215024212
6.947193669 1.064767507 3.512954339 6.706228383 -5.676280007 -
2.130302623 5.181131342 -2.524062646 3.062659749 -5.338143609 4.952270243
1.641307913 2.516408363 3.643676598 5.91595551 2.193801986
2.192584974 -2.169851832 4.491278932 3.933686951 3.98776934
4.248955427 -2.275787725 5.069296681 6.312677647 2.831437881 -
0.354124689 5.638380861 -1.294324105 6.207952031 5.009617996
4.700239385 5.553094176 6.255056426 10.72922055 1.046384843
2.401941432 0.356956229 -0.532193865 2.656749456 0.977386466
4.399204712 6.801234808 3.456216391 4.891751611 -2.401788295
2.204067494 -0.633730112 3.263024016 3.855830912 -2.73517601
5.346664989 3.954267623 0.092728866 2.5626099 3.991597348 4.96501616
4.750172356 2.597046839 5.335165127 4.594302058 4.792990911
3.968080379 0.497946994 1.67403034 4.462032903 4.609019868
4.710496596 5.552298382 4.78699046 0.856912111 5.347970639 4.003179154
1.373121695 3.776515617 -0.935976003 -4.568843392 2.083459237 -
0.664969925 -3.833485701 -3.707740347 1.524229647 2.485002659
4.417987689 5.163460221 3.186484768 5.907824637 -1.790994311
2.508566941 4.35704671 3.506645927 2.726722473
TCGA-73-4675-01A -0.529454445 5.028307439 3.444120061 5.412593014
4.054785681 8.271262161 3.462711068 4.289253986 2.172495334
3.388932704 5.483427196 -2.014169665 3.662872147 4.320814142
4.801559016 5.208956611 3.472126938 4.401927542 0.34133954 0.486870908
3.03425356 5.032735674 -0.96454943 2.17438098 4.530345707 3.391073248

5.482326269 4.654368047 4.695561005 3.826081795 5.049620981
1.266974266 -3.573479252 3.343807021 -1.315005671 2.753931232 -
2.618031051 5.593977686 2.999352135 0.384776447 4.723829347 -
1.050041271 4.985766795 2.596426364 4.140771821 4.272742363
7.123778789 1.349022871 3.825151657 6.93602237 -4.791330924 -
1.516806345 4.517722336 -2.662451372 3.517132282 -4.084526721
5.212952898 2.493374838 2.226246233 3.735915061 6.112808375
2.859145823 2.139631368 -0.885643472 4.558538704 3.936047518
4.816174196 4.221321017 -1.363215487 5.093537749 6.479268226
2.983849734 -0.097426443 5.914112791-0.552503231 6.14924509 5.089307906
4.853352025 5.544021382 6.296784581 10.37413511 1.787185926
2.4984113320.708380668 -0.395334538 3.154219043 1.062143543
4.3911772886.923448393 3.786639891 4.513628346 -2.128856763
2.478935122 0.155515625 3.349583914 4.001239053 -2.22866192
5.358120776 4.383037008 0.636176192 2.520979703 4.167553775
5.10160461 4.572769129 3.402898973 5.335724512 5.082397953
4.879374093 4.937841319 0.673528342 3.068383483 4.789226055
4.839921208 4.720301272 5.785255191 5.199094805 1.277634953
5.639674245 4.146106845 1.933419895 3.98003618 -1.252807306 -
3.207620206 3.17226149 -0.384431344 -3.540809526 -3.13097857 1.878839424
3.140474619 4.632300623 5.316643551 3.227495525 6.241164278-
0.857509736 3.373091042 4.740428132 3.777625287 2.470605093
TCGA-73-4676-01A -0.922254803 4.601954862 2.496336303 5.756328035
4.451946417 8.24167184 3.086560062 4.29517744 1.638726221 3.512267903
5.658681954 -2.236638344 3.878302874 4.843476855 4.763250137
5.199959279 3.5325341193.752465425 0.562030628 0.471785582
2.501306204 4.910610476 -1.519330929 2.302460931 4.591167045
2.826501906 5.224978968 2.469658874 4.405931614 3.994221012
4.858866739 1.358105575 -3.940167682 3.475563745 -0.674933419
2.251125766-3.339500377 5.631635384 2.990105326 0.812052145
4.703254946 -1.344322192 5.239840459 2.73743743 3.860427345 4.2269095
6.853255518 0.800162068 2.910795829 6.824074555 -5.169221056 -
1.941472668 5.081717743 -2.63190259 3.386488119-4.442466292 4.911958599

1.594575387	3.143446861	3.596848349	5.933097863	2.478602847
2.330657447	-2.016724515	4.451244313	3.946229043	4.040665814
4.250930819	-1.860435257	5.080922168	6.196923326	2.698861932
0.016806653	5.562115487-1.003990809	6.123390414	4.903461254
4.478915302	5.537873243	6.265497977	11.131480031.283245555
2.7116296150.552113922-0.637115519	2.905460658	1.249088044	4.428120357
6.809879906	3.382993763	4.982019578	-2.337337145	2.556687333	-
0.039238031	3.360027057	3.781781191-2.502315913	5.333747738	4.218745651
0.246885815	2.702203905	3.844976204	5.00851241 4.737978549
2.826875072	5.335313217	4.633693706	5.000857533	4.625989911
0.359962159	2.208943358	4.492134901	4.666567093	4.761292898
5.596500617	4.867730668	0.870380615	5.5111859264.544639098
1.844477615	3.701100458-1.131516125	-3.949120919	2.988307561	-
0.277178692	-3.71428885 -3.211772065	1.682455723	2.56089155 4.55152489
4.8897611343.216957053	5.766358856	-0.88941902 2.657347349	4.373848499
3.476460177	3.231987313
TCGA-73-4677-01A -1.299484952	4.524864207	3.341787262	5.597684243
4.292896683	8.346539143	3.000979579	4.325414779	1.751192049
2.986093222	5.417905488	-2.262799112	3.466700957	4.226669058
4.289059923	5.1130377883.424826738	4.106087866	0.573554706
0.355630748	2.515129557	4.803004087	-1.526186526	1.996833299
4.469536313	3.245158072	5.551570904	3.262260206	4.364744379
3.187927481	4.826838822	0.769345964	-4.144774693	3.489998289	-
1.296773821	2.725045065	-3.091782375	5.600771842	3.175357445
0.817674952	4.710548331	-1.427305789	5.178508507	2.957370245
4.028017542	4.251961959	6.981574393	1.336888849	3.419962956
6.34938822 -4.91686753 -1.975364219	4.66287667 -2.693949843	3.186066472	-
4.923257679	4.727887494	1.796943779	2.3474311563.872696712	6.063648022
2.707174014	2.181182035-2.076551651	4.434787467	3.943359668	4.0681145
3.964653067	-2.09824189 5.075200186	6.285205018	2.988008108	-
0.268397145	6.094176616	-1.11138386 6.017316766	5.215549968	4.422725238
5.549598449	6.30110485 10.27677373	1.551651082	2.240182431
0.500756801	-1.014376409	2.774845556	0.810508061	4.444622709

6.813053207	3.894143582	4.601240106	-2.314850002	1.938673197	-
0.187703262	3.274253504	3.620535076	-2.617364895	5.36091711	4.600026563
0.26568582	2.654893442	4.290097388	4.916435254	4.723028443
3.1197184645.335218994	5.196541766	4.694305237	4.243432931
0.192515791	2.637807413	4.603963044	4.579004677	4.722048196
5.890961641	5.349946229	0.907396525	5.771440715	4.335604489
1.7572372113.851034671	-0.845208281	-4.238899319	2.470774055	-
0.233949733	-3.311918991	-3.709229994	1.792047054	2.861620078
4.586870745	4.923916199	3.256573603	5.868032263	-0.755189514
3.124509208	4.467552666	3.541252758	2.62557523
TCGA-73-7498-01A -0.659428432	4.706320752	3.3106611755.348093081
4.136491632	8.24150235	3.598854642	4.223706447	1.834721615
2.835245559	5.098691437	-2.332190653	3.184680858	4.238426676
4.661328387	4.988549464	3.700572482	4.280995107	0.155320328
0.399204514	2.747240302	5.135139809	-1.36221194	1.938826264
4.556074655	3.327920009	5.697549238	2.564581435	4.469048743
3.1162526474.981941677	0.823931853	-3.655105582	3.276945278	-
1.471249462	3.078521788	-3.20113231	5.57941613	3.372753052	0.79138821
4.70913056	-1.148939631	5.03814345	3.243909993	4.044491353	4.23473693
6.9626117331.77553123	3.904727456	6.707499405	-4.8298814	-1.815514572
4.31999891	-2.560230218	3.193715424	-4.937758765	5.073673848
2.260342093	1.949457444	3.977636197	6.009975637	2.730828849
1.876996989	-2.039120015	4.666936692	3.935250818	4.181601915
4.093348814	-1.970689776	5.084125092	6.421379687	3.027748679	-
0.165789497	5.986818381	-0.670348708	6.070977823	5.168790447
4.776694422	5.502872101	6.37730621	10.18205649	0.605133857
2.478055558	0.774320571	-0.982617766	3.263536655	0.745488415
4.333079468	6.933730996	3.779696633	4.574658776	-1.908885009
2.03442153	-0.53546745	3.249314826	3.75439876	-2.361833546	5.366169062
4.624298484	1.190931991	2.812929258	4.080555537	4.996225008
4.730740725	3.413017046	5.335554222	5.105484556	4.823014163
4.146208553	0.646262164	3.686657127	4.623672454	4.810765686
4.7193114455.746642358	5.3946817	0.998882558	5.707778109	4.031727851

1.852159017 3.954840705 -0.677490389 -4.028505812 2.633905881 -
0.297702241 -3.48807867 -4.229272365 1.551026553 2.977015393 4.357892202
5.254430444 3.295707323 6.245151629 -0.1428315 3.107575809
4.464024043 3.731869978 2.46473944
TCGA-73-7499-01A -0.784043021 4.708262383 2.095634504 5.447651401
4.442445395 8.203401698 3.742892946 4.241750479 1.983351778
3.489919271 5.385380595 -1.991323807 4.007512872 4.784088056
5.053158292 4.864182174 4.07340031 4.501068294 -0.05634134 0.351947565
2.755323523 5.136067554 -1.262066484 2.449317463 4.605914274
2.539290446 5.6028132 2.508337175 4.59153801 4.277280531 4.78275368
1.422479636 -3.719472811 3.240355012 -0.784632758 2.632578301 -
3.944649774 5.612030361 2.578527149 0.000175189 4.710269499 -
1.143393895 5.468108742 2.992732104 3.774109582 4.315375145
7.031084387 1.178995515 4.059520806 7.087833886 -5.268304179 -
1.942635597 4.671815854 -2.623523106 3.96741498 -4.522735531 5.013447927
1.7119823573.101393543 4.028726058 5.966155845 2.737658159
2.352318811-2.201669509 4.477087352 3.936014099 4.307636137
4.308853147 -1.456178033 5.078301698 6.338121871 2.859662735
0.189698986 5.65229402 -0.652371625 6.070273205 5.013120545
4.646093219 5.455580361 6.4027014 10.13713586 0.637969532
2.891028299 1.307890895 -0.753642754 3.019658967 0.8649488
4.312030604 6.860997621 3.635253207 5.173002445 -2.203237685
2.518928657 0.089087084 3.385494809 3.851152465-2.300784119
5.342686163 4.187512604 0.820772388 3.432502989 3.60108325 5.00052803
4.731387522 3.461742083 5.335308485 4.732008529 4.994179107
4.720519664 0.682015741 3.07337448 4.687715451 4.715227327
4.765371618 5.949610922 5.268194067 1.09008528 5.283823539
4.138390525 1.87630911 3.925333077 -1.203676875 -3.91464914 3.162835871
-0.237513945 -4.003068448 -3.102816965 1.372200984 2.454017714
4.427828744 5.695949863 3.63673604 6.14841508 -0.854117961 2.314223439
4.723492096 3.685395157 3.015917535
TCGA-73-A9RS-01A -0.952318639 5.1196883192.482204932 5.359047153
4.323163464 8.193870484 3.595889225 4.104046079 2.229709033

3.551504941	5.045064226	-1.860670403	3.491882463	4.633995249
4.796141607	4.942916543	3.9451118464.440976885	-0.06918621 0.327184938
2.932051389	4.981231393	-1.344536083	1.813335469	4.676432581
2.542062934	5.541345957	4.153788459	4.526486397	4.364691673
4.77019002 1.143489594	-3.898643246	2.768764497	-1.170785757
2.473836764	-3.452767154	5.608127777	2.992513145	-0.307053277
4.686369679	-1.141118824	5.137609153	3.353978465	3.879778248
4.295080061	7.123230045	1.54559172 4.486437323	7.112120314-5.356947139
-1.762877168	5.087549117-2.593575645	3.555525892	-4.669410611
5.186763729	2.24201488 2.426082444	4.038623763	5.809519667
2.782377162	2.003371954	-2.136198827	4.473950164	3.933396266
4.537447198	4.244098901	-1.228739277	5.069069451	6.407104942
2.958701824	-0.048099628	5.288016166	-1.099300801	6.323280832
5.073766497	5.07128653 5.435726789	6.552138252	9.835434028
0.851515599	2.68163884 1.149682656	-0.766931267	2.950055803
0.739769577	4.361803177	6.843321857	3.70612101 4.991172691-2.005560811
2.685023609	0.076516959	3.295474734	3.93425369 -2.14631382 5.365006181
4.033326003	0.876537086	2.594212884	3.919678905	5.026561804
4.681491393	3.372496512	5.335347728	4.914964178	4.785786557
4.130412491	0.615410026	3.707551289	4.639052482	4.675920396
4.700615239	5.838782589	5.039087363	1.16454088 5.1401166214.143334818
1.407978675	4.040327385	-1.748970644	-3.711444149	2.599518433	-
0.377861442	-3.994985996	-3.410587073	1.225074566	2.525298509
4.258061531	5.876665215	4.018966807	6.33290225 -1.009687076
2.367080278	4.635223228	3.65028413 2.07894977
TCGA-75-5125-01A -0.127365573	4.913051542	2.652694957	5.476333367
4.271326893	8.220471315	3.563377746	4.296257292	1.663291746
3.510136376	5.440058108	-2.593741515	3.558354545	4.940755615
4.995548625	4.842010767	3.848530231	3.792172624	0.058520268
0.395941721	2.544432836	4.983914648	-1.109104184	2.562203202
4.561758774	2.569140406	5.682758665	1.006698518	4.57594418
4.350507328	4.885356972	1.082127534	-3.535900284	3.227698164	-
0.658181022	2.098476762	-3.849382584	5.6114221253.781464097	0.72798463

4.709388824 -1.147579765 5.05928082 2.9160979 3.869729903 4.171173165
6.992209675 1.191386361 3.941771438 6.982315398 -5.212718966 -
2.170531315 5.434145854 -2.508176267 3.297986257 -5.047875492
5.141290513 1.649354543 2.564217329 3.660821085 5.942468752
2.517902154 2.534067272 -2.339164558 4.708400497 3.943241324
3.703320547 4.578342686 -0.713478239 5.088044068 6.189686827
2.690590875 0.017570547 5.707337458 -0.349463599 6.105458734
4.953394153 4.81266451 5.483125601 6.325800809 10.80016598
0.344726082 2.942397191 0.697925533 -0.739436188 3.607330138
1.1157982924.310754789 6.820200724 3.340896053 5.099865782 -
1.940594928 2.714452916 -0.385044886 3.259287831 3.747710087 -
2.046177313 5.355746427 4.277008261 0.958735051 3.00589904 3.693290226
5.035310672 4.940794954 2.88707401 5.335563351 4.453189517
5.034097882 3.98909101 0.833982983 2.285250488 4.569603188 4.90833012
4.7764471175.554484877 4.5380101130.789925422 5.518871534 4.3360893
2.084267417 3.902055963 -0.561693234 -4.012001542 2.841885817 -
0.141557406 -3.83105288 -3.9508333 1.3681822 2.806332394 4.401215107
5.405533184 3.4861178486.223697436 -0.604350758 2.570247796
4.490300975 3.507251655 2.993879543
TCGA-75-5146-01A -1.131646707 4.460171625 2.876934106 5.493555543
4.343255425 8.38011677 3.552160029 4.25500841 1.81651838 3.441923615
5.570006999 -2.093221074 3.524124289 4.86623473 4.760668397
4.968950913 3.874277942 3.966083569 -0.045891377 0.447041229
2.752997435 5.145931159-1.551358158 2.657230016 4.527217633
2.9251145735.447271906 2.416418322 4.432643025 3.462425267
4.708286742 1.098708972 -3.866187545 3.23028467 -1.162230528
2.327783513 -3.964929983 5.594821761 3.403048191 -0.122598075
4.71303278 -1.274717456 5.316003125 2.977456133 3.869377726
4.279718356 7.145034182 0.860382354 3.490808838 6.847567733 -
5.162406877 -1.769159289 4.575586005 -2.943710462 3.768511699-4.116612999
4.862652543 1.879325657 2.909464432 3.95550586 6.084906826
3.034375745 2.472477502 -1.999859261 4.440750946 3.942187013
4.304538006 4.129085243 -2.355206685 5.081823567 6.23620546

2.909734039	0.085363661	5.947261925	-0.894180986	6.007503782
5.219487486	4.53326458	5.471693239	6.424516298	10.43470281
1.382023467	2.68271165	0.737084347	-0.928882466	3.231874288
0.879209472	4.339583353	6.8470119363.884874826	4.772101092	-
2.252476734	2.09696942	0.2598742113.335018487	3.807446625	-2.812272468
5.343363719	4.528384266	0.826230036	2.922730649	4.295390972
4.961784207	4.698469961	3.23902342	5.33524401	5.173370581	4.831615243
4.800682442	0.297396168	3.24733767	4.6171147464.595833182	4.755702572
5.770235819	5.343239735	1.084261435	5.693732385	4.368241049
1.870952522	3.788062782	-1.058867278	-3.611839814	3.227014962	-
0.364045986	-3.692648899	-3.036435132	1.562040471	2.571550174
4.432863822	5.038281445	3.443727195	5.949543961	-0.715933638
2.828246978	4.777308593	3.752192106	2.76280219
TCGA-75-5147-01A	-0.930140758	4.732981751	2.599074712	5.430113385
4.130866699	8.293791772	3.948894805	4.262087214	1.875456217
3.671453849	5.518904662	-2.245584888	3.771958935	5.018309084
5.145947906	4.709953658	3.999697448	4.2040611510.081607821
0.358643955	2.671208313	5.220725121	-1.527701251	2.686370046
4.5880041142.307463651	5.745644084	1.335330763	4.604570021
4.422585276	4.62928214	1.254386305	-3.789003412	3.358961134-0.907518069
2.407326178	-3.837499062	5.599888595	2.834220632	0.036933974
4.708163327	-1.090977413	5.340346875	3.056020907	3.817882262
4.312370736	7.02087471	1.169685054	3.803845521	6.77996561	-5.374069359
-1.96464586	4.627170282	-2.518358411	3.803131978	-4.460488896
5.036861246	1.8271106072.484299699	4.020057538	5.950838477
2.792400298	2.557569991	-2.444093564	4.547184482	3.945764339
4.188919807	4.349929571	-2.415360904	5.083634684	6.241871411
2.850794121	0.1036114525.843013953	-0.845854554	6.054620185
5.070315005	4.575690518	5.492414519	6.41969459	10.45755258
0.617650952	2.907122733	1.216194454	-0.800029468	3.284948208
0.886243263	4.250989539	6.860821372	3.637860403	5.172710959	-
2.155480912	2.673096703	-0.142495859	3.355944848	3.861098006	-
2.486408125	5.343216557	4.279070317	1.02820746	3.396045832	3.939221916

5.002466829 4.732331042 3.202602553 5.335388269 4.997723913
4.935080512 4.573723178 0.603690736 2.818789963 4.567333244
4.726100555 4.774785022 5.779023287 5.084837432 1.081339169
5.391817796 4.233274641 1.85394092 3.823614771 -0.90247631 -3.838723463
3.087082571 -0.251478468 -3.903429252 -3.222050628 1.285994215
2.4911458444.279633734 5.403032896 3.548941302 6.272221385 -
0.846831833 2.251652904 4.732329308 3.7402111933.091716536
TCGA-75-6206-01A -1.13390802 4.4679112883.223695469 5.462925351 4.176704708
8.341328197 3.088502907 4.309530923 1.478533128 2.906809468
5.492354297 -2.527448249 3.189805254 4.332415417 4.357085661
5.214641579 3.943095174 3.674337973 0.35865572 0.361989067
2.602750924 4.876395929 -1.565440324 2.597454066 4.458953768
3.208521387 5.667043605 2.300168287 4.396587987 3.181804458
4.945770631 0.891863821 -3.935120161 3.746168547 -1.42880227
2.693262882 -2.998538066 5.588994656 3.935174783 0.77918102
4.701227033 -1.384888442 4.90981677 3.353003427 4.032485366
4.159350631 6.864080854 1.500231495 3.548445073 6.250015376 -
4.874899563 -1.927073046 4.702568755 -2.68664886 2.974086132 -5.174394626
4.959748878 1.694623996 1.66353146 3.7745616116.12615114 2.773064048
2.553155261 -2.730454468 4.599041836 3.935254325 3.90523241
4.184917597 -2.286076618 5.080072454 6.331685168 2.933702086 -
0.343631464 6.085109874 -1.009950034 6.072621964 5.230472439
4.565655513 5.520917393 6.38593285 10.78778712 0.875589848 2.2886263
0.36412972 -0.990974099 3.237592697 0.786726763 4.371038021
6.873528492 3.8203314 4.685919718 -2.043192473 1.895100585 -
0.531583134 3.273198416 3.614559589 -2.617126951 5.365590024
4.380534394 1.071964439 2.814562451 4.137412087 4.94514436
4.749054499 3.05227761 5.335354612 5.129328528 4.739489566
3.946161583 0.386077957 3.487676156 4.581715543 4.730767182
4.699906429 5.75721468 5.450595854 0.835465496 5.939941277
4.002494408 1.809540595 3.82844996 -0.46582381 -4.23249998 2.30200839 -
0.153568918 -3.405123014 -4.405942243 1.731537361 2.780457531
4.420374881 4.857599959 3.284891836 6.140660233 -0.454969425

3.348859279 4.238396779 3.563392247 2.253323618
TCGA-75-6212-01A -0.477164663 4.770505426 3.025416502 5.545340821
4.281070989 8.277041864 3.391826838 4.340145834 2.024423791
3.414748939 5.310976903 -2.265120042 3.635122357 4.479772807
4.88437255 5.028835993 3.053389826 4.008127694 0.682395748
0.661710906 2.549371916 5.204026561 -1.20634652 2.515379563
4.515175004 3.098777615 5.670548991 2.792426646 4.497649902
2.986840001 4.766393836 1.017626048 -3.559299335 3.68114628 -
0.909519388 2.716612183 -2.764109173 5.596833848 3.272189529
1.618021895 4.72565074 -1.135834284 5.31082213 2.7281101374.050781948
4.32500185 7.066248899 1.429687762 3.643103314 6.663322951 -
4.698177934 -1.709661609 4.629508005 -2.291682202 3.308117849-4.265657027
5.036636903 2.099690209 2.975721902 3.929403167 6.097042076
2.625591051 1.986256906 -1.672090565 4.677628782 3.939853455
4.1191720144.21699058 -1.395828321 5.091770248 6.402423338 2.900756392
-0.124836844 6.122139865 -0.309647031 5.966060426 5.159658264
4.570937994 5.517315964 6.265149543 10.58943761 0.981507258
2.789039433 0.873672655 -0.598434111 3.078696231 1.014079101
4.35697383 6.887210299 3.642625816 4.671664748 -2.061121811
2.055399717 -0.020039314 3.289910407 3.771599335 -2.281490995
5.337571552 4.956852952 0.758637346 2.9517116844.22165206 4.966455599
4.877525487 2.844952877 5.335498946 5.078356661 4.871079904
4.351968072 0.57477195 2.136483831 4.465779922 4.685182456
4.7950611985.594075743 4.998161074 1.159333582 5.547368172
4.554757936 2.23313967 3.813540116-0.492122891 -3.922953384 3.357997779
0.042877977 -3.238841076 -2.959554929 1.924413773 2.876814337
4.667705842 5.04429207 3.044289305 6.055183713 -0.006244215
2.979703956 4.601663627 3.78618305 3.390855556
TCGA-75-6214-01A -0.912770079 4.730010922 1.738550008 5.391507101
4.487156019 8.202989477 2.980524395 4.265476902 1.577364146
3.332781988 5.599495981 -2.760687865 3.17006635 4.859192173
4.299798783 4.888184757 4.199393129 3.422584348 0.12310076
0.257256018 2.662027386 4.927574865 -1.303910096 1.87208311

4.510378761 2.518544154 5.403894007 1.039998779 4.58545806
3.713024465 5.068496231 1.167223321 -3.971449266 2.247068194 -
1.171877961 2.169471251 -3.862781064 5.61535355 4.147930288 0.315169899
4.703036639 -1.256210706 4.547124731 2.740503788 3.810761993
4.0911464497.064547546 0.863188584 3.775566946 6.793742404 -
5.506622549 -2.023008926 5.518888026 -3.027771465 3.227755117-4.932483408
5.1145935491.842875045 2.309976767 3.60910302 5.883214422 2.031555829
1.371547583 -2.524754353 4.572610329 3.937823066 3.713119759
4.219619401 -1.070976691 5.066271254 6.2657649112.711444903-0.019214658
5.278025185 -0.794387711 6.170139713 5.060016952 4.720889096
5.440789635 6.16129716 11.198220450.448296225 2.574929137 0.43609371 -
0.469295772 2.414853103 0.932698515 4.318519565 6.809674573
3.323860271 4.896957835 -2.377764539 2.245337611-0.653495262 3.29113577
3.572875591 -2.289768173 5.367598326 3.95410296 0.035826624
2.753466453 3.976864229 4.91697596 4.93163844 3.057389749 5.335076552
4.469473886 4.898141904 3.90245115 0.709349622 1.837037433
4.363462226 4.867023275 4.746769995 5.653069744 4.881465725
0.442188928 5.304985647 4.0114869471.722999121 3.820136097 -
0.608170839 -4.735361445 1.867204451 -0.325630834 -3.826787836 -
3.618789662 1.506007176 2.58816988 4.384298486 5.183153545 3.377449484
6.258984938 -0.811320609 2.558416388 4.459277017 3.536740927
2.773390766
TCGA-75-7025-01A -0.45401269 4.667973056 2.827372344 5.253061937
4.086449173 8.257925697 3.70860755 4.257343692 1.944536618 3.40728612
4.962695248 -2.387954057 3.284082422 4.3711994544.829166413
4.836366436 3.582964253 4.076079743 0.402003231 0.504493696
2.817475625 5.436337016 -1.124593737 2.246829355 4.571595504
3.1100051475.733037553 1.876054861 4.56458737 3.094743449 4.857881138
1.074249082 -3.627888625 3.277885933 -1.288354458 3.046542477 -
3.131475462 5.575530676 3.296420588 1.167682782 4.719691292 -
1.085891872 5.212907346 2.85254599 4.037307964 4.270288813 7.031911042
1.645588915 3.7211164566.629705351 -4.622782613 -1.594049459
4.464546757 -2.53128021 3.419020968 -4.462671768 5.066685832

2.084904387 2.490438843 4.109817657 6.045212505 2.800658761
1.705600106 -1.723834424 4.7116391383.933533627 4.13810659 4.233185418
-1.28360262 5.093181634 6.442958397 3.002657244 -0.06121993 5.940058189
-0.245061147 5.90690143 5.200896969 4.549336612 5.436772382
6.328384562 10.53919469 0.718753442 2.574682082 0.897110571-
0.698815004 3.4578580110.804465969 4.24101963 6.918467348 3.661734876
4.524546532 -1.950356278 1.966465382 -0.106271322 3.284428906
3.765911505-2.14877391 5.344296764 4.999056942 1.233130325 3.088958392
4.280692987 4.949322607 4.787816296 3.2110060015.335457747
5.129498474 4.900378786 4.232897538 0.774261212 3.00103211
4.534789829 4.79489872 4.781320533 5.596898547 5.200894324
0.958348454 5.615303312 4.048705732 2.198157227 3.886778555 -
0.441470162 -3.7665074 3.285773112-0.156553765 -3.40478751 -3.612175552
1.597445422 2.901588875 4.327766077 5.222691245 3.300924271
6.183200329 -0.296904601 3.054950295 4.510923678 3.745474994
3.097419633
TCGA-75-7027-01A -1.32186963 4.468698764 2.2601138735.474940187 3.790253834
8.298536104 3.469866884 4.249685207 1.734310309 3.50144707
5.514392447 -2.297254012 3.158226596 4.463418328 4.309462738
4.966334839 3.9119989563.824998385 0.106481918 0.215961748
2.625831682 4.911928251-1.767433779 2.451549961 4.599655396
2.497892251 5.555049023 2.054307747 4.340791455 3.046647294
4.763284402 0.93259359 -4.081735009 3.259703814 -1.300250581
1.760040372 -3.720043398 5.600142161 3.035994057 -0.713721566
4.682165103 -1.263379015 4.52614809 3.125321486 3.875183457
4.325851558 6.812341458 0.97889851 3.491526823 6.847010144 -
5.568337059 -1.853235808 4.947325774 -2.400145112 3.225768458 -
4.91652265 4.909921369 1.881549412 1.892507127 3.789419828 5.87544734
2.575351971 2.499526228 -2.573778473 4.544222797 3.933110042
3.954259482 4.28823524 -2.540492163 5.068101538 6.360720357
2.850754789 -0.19918568 5.68802927 -1.24903423 6.1151763095.102186249
4.718827745 5.5025617116.357104994 10.63636552 0.569220745
2.428589775 0.674210467 -0.854655335 3.152975459 0.739381294

4.274795144 6.731052219 3.547107518 4.743945419 -2.348084848
1.990022939 -0.488473009 3.294779509 3.814458717 -2.883114283
5.349866652 4.231596746 1.362996155 2.783205182 3.7198625114.9428747
4.641637308 2.987006433 5.335208545 4.6115006 4.842596188 4.05192276
0.434800179 3.158321841 4.365174208 4.61974914 4.7147716115.467525938
4.887051717 0.846138326 5.462243556 3.607558024 1.344968466
3.735491514 -0.912716328 -4.283115603 2.575823155 -0.66920155 -
3.771700324 -3.672561613 1.432300298 2.29722418 3.961743747 5.098313405
3.5356119685.915080496 -1.72401556 2.294579841 4.260593597 3.56830629
1.779930482
TCGA-78-7143-01A -0.62483185 4.857851891 1.888341721 5.270434045
4.445215035 8.239708662 3.54251487 4.246891827 2.042460013 3.32791734
4.970855391 -2.220328127 3.434892537 5.143017683 5.079444356
4.615562636 3.820036293 3.867937752 0.474231944 0.471127881
2.710010676 5.471816032 -1.087515011 1.801690002 4.634916632
2.7325163115.581799204 1.441006226 4.602329804 3.934531796
4.900505806 1.153536665 -3.542648005 3.010466748 -0.721191788
2.701573453 -3.967421555 5.596416443 3.633220933 1.3223244
4.710771314 -1.125114444 5.145137598 2.952357353 3.85647826
4.302284025 7.078498973 1.352287683 4.2071142936.916599241 -
4.830663468 -1.85793695 5.028268116-2.426462101 3.711449136-4.39574292
5.191582629 1.758885196 3.0395119494.147427775 5.836376534
2.513022147 1.627873976 -1.949515322 4.593902944 3.944597215
4.083340946 4.369254088 -0.547434314 5.077019812 6.300770438
2.900438059 0.159414019 5.662546246 -0.348917109 6.060382802
5.001386506 4.715837417 5.415670162 6.437387074 10.11993661
0.177574514 3.065590858 1.053778553 -0.823788091 3.034776992
0.894722403 4.208948191 6.857544497 3.376557059 5.061723035 -
1.905948555 2.462826458 0.310054044 3.267679566 3.575981034 -
1.862658882 5.345905585 4.569076389 0.994296977 3.370251588
3.793196756 4.95789947 4.864038267 3.493094912 5.335397699
4.524955404 5.007821875 4.275724164 0.645821801 2.897663541
4.610419236 4.737683819 4.779503616 5.708710777 4.66504479

0.844400492	5.425262592	4.621466897	2.075814957	3.983824362	-
0.753405929	-3.234170655	3.424207625	-0.00183195 -3.605469085	-3.723496686
1.32707202 2.760489089	4.271717719	5.727645706	3.575456447
6.319389275	-0.5645386 2.514777107	4.577206317	3.699282257
3.086928896
TCGA-78-7145-01A -1.189831596	4.338782984	2.646975089	5.602093925
4.629708282	8.340190971	3.25406119 4.360521455	1.588701533
3.428314691	5.584673001	-2.298855366	3.85317022 4.807510507
4.634130574	5.1162912293.673938163	3.816830289	0.865413258
0.642317784	2.577539762	5.179215779	-1.491050711	2.928993822
4.499778674	2.84803566 5.29211002 2.045882845	4.429649054	3.825078512
4.803151426	1.287785507	-3.781339791	3.884031721	-0.62198002
2.813546151	-3.083713678	5.627072377	4.055139917	1.63493003
4.697351607	-1.373575234	5.347385673	2.948842824	3.915357676
4.138470163	6.822565005	1.135047722	3.335440871	6.521233017	-
4.714067351	-1.897389212	5.228122167	-2.385891817	3.253205539	-
4.459246674	4.881725039	1.57839244 2.744749079	3.673884981	6.005159152
2.797436708	2.760819261	-2.537966932	4.51507262 3.946872564	3.90860377
4.515490444	-1.708601548	5.081852047	6.1136318152.752087776
0.089920057	5.659804544	-0.955025386	6.051063005	5.09447112 4.38813626
5.498734275	6.435667189	11.174283571.014323625	2.778540703	0.73385664
-0.725963883	3.376896681	1.022088596	4.374498214	6.774541983
3.689230643	5.033426157	-1.917368384	2.349631306	-0.047460929
3.317077096	3.587003702	-2.507827421	5.34795527 4.237664508
0.465343761	2.982370058	4.057610748	4.944534165	4.943552258
3.126312544	5.335275908	4.927324497	4.845672088	4.305690596
0.380138627	2.299554907	4.475868997	4.625001981	4.75466928 5.6525819
5.012130894	0.815636894	5.718772092	3.902084882	1.923071904
3.711170634-0.117643729	-4.054631774	2.919334355	0.081134135-3.301509212
-3.521814297	1.8323011712.467106669	4.440391012	4.974609914
3.4701137925.977704847	-0.110582305	2.917330397	4.360164958
3.478970493	3.478274752
TCGA-78-7146-01A -1.411533687	4.503871642	2.095044999	5.581775821

4.379632173 8.140741467 3.21057977 4.240507839 1.400033049
3.267925927 5.639851929 -2.689375206 3.0754511324.691497133
4.495704962 4.875908838 3.901400712 3.508665757 0.039829885
0.287195017 2.337146514 4.447967858 -1.801444874 2.253346163
4.518678203 2.425842553 5.21235432 1.169273377 4.303786349
4.095161547 4.833812825 1.054144284 -4.012868281 3.029500516 -
0.738783748 2.019618015 -3.685152986 5.646561826 3.734727268
0.642646513 4.684396355 -1.399496356 4.899281989 2.783995886
3.7764905114.037463626 6.853230164 0.865055495 3.336532601
6.558801036 -5.386123366 -2.35557725 5.584924737 -2.49671399 2.793045853
-5.351525482 4.864475009 1.456102249 2.557486966 3.363588233
5.741653138 2.280756861 2.396593488 -3.403916269 4.480301162
3.943488613 3.317948044 4.532607978 -1.85452292 5.082054659
6.012358981 2.629683973 -0.034013063 5.27285309 -1.49655049 6.218796373
4.9117747144.592749632 5.504087038 6.231764778 11.333230310.251337384
2.636647644 0.597836857 -0.905426817 2.76197035 0.994236092 4.39086053
6.708606962 3.356408837 5.092544077 -2.288906931 2.433963723 -
0.724483408 3.284835241 3.558629533 -2.913010602 5.347484973
3.714986839 0.269355903 2.622070438 3.669794961 4.899548066
5.023342851 2.818035021 5.335223267 3.91042919 4.793837055
3.776012107 0.307018166 1.961623509 4.178410517 4.670779474
4.735654202 5.497453255 4.385090257 0.5362571 5.42054733 3.856877839
1.49686931 3.70532808 -0.86451432 -4.964796658 2.1677627 -0.280166611 -
3.771511048-3.965930995 1.4273511812.28840278 4.260524019 5.151158857
3.630679777 5.813466971 -0.91553237 2.433654833 4.054658018
3.349942547 2.666202364
TCGA-78-7147-01A -0.318301987 4.88966891 2.096143699 5.33172372 4.412319149
8.153080009 3.608039645 4.14927795 2.161282522 3.314850938
5.163098158 -2.104916276 3.548043599 4.938313324 4.986797075
4.986158328 4.285361427 4.291146296-0.11219894 0.398058785 2.999743012
5.233945023 -1.115423401 1.876616877 4.630529305 2.744969332
5.445813233 3.205463194 4.664799409 4.072551946 5.10931799
1.642332034 -3.46239574 3.000653576 -0.913546349 3.122881443 -

3.588970321 5.608222191 3.50822929 0.660707598 4.710635237 -1.085265806
5.291642278 3.261639196 3.843250395 4.185453221 7.112618039
1.484013024 4.60251148 7.237026823 -4.930664256 -1.679243263
4.883906328 -2.614484935 3.764402135 -4.237960147 5.270504711
2.308807794 3.218353712 4.0063112065.915037924 2.5174110041.819570422
-1.559694988 4.591739582 3.935246672 4.525757838 4.250854889 -
1.051695754 5.072451561 6.400331042 2.916408971 0.234821493
5.458551347 -0.432381745 6.169444412 5.028284294 5.049365998
5.437815697 6.338160196 10.0266319 0.231709408 2.985993438
1.388633793 -0.636258103 2.7511028190.824769449 4.279689439
6.927928828 3.569075153 5.06959546 -1.947896832 2.5553711070.171666374
3.36895641 3.648099476 -2.219929511 5.349553276 4.02100739 1.157969561
3.096996242 3.789198455 5.039976786 4.834318278 3.802857655
5.3354442 4.583994404 4.988844682 4.553386357 0.875430714
3.726071889 4.586019429 4.864799045 4.72597113 5.825581395
5.230165017 1.081478682 5.247461273 4.019336849 1.820740257
4.028425127 -1.272840779 -3.357970218 3.211647569-0.040441981 -
3.892901494 -3.283531449 1.444944648 2.728574254 4.386061412
5.812468717 3.710809283 6.521390014 -0.250815477 2.873725452
4.796036785 3.836975234 2.997291329
TCGA-78-7148-01A -1.308397518 4.629574471 2.723131856 5.507294828
4.183843805 8.377954077 3.0411576244.363653979 1.856661204
3.222716023 5.70870089 -2.301187521 3.713313271 4.552305444
4.491599338 5.246296384 3.407709286 3.851302684 0.465418058
0.494780651 2.613082071 4.716982399 -1.534130257 2.381723854
4.495229727 2.949606967 5.7275327 3.324806528 4.405360933
3.107343632 4.752437812 0.93396194 -3.909240516 3.459374396 -
1.170935058 2.483336717 -2.874865677 5.603758042 3.015675622
0.378096444 4.708338243 -1.264770271 5.156459033 2.747648857
4.065844546 4.3198845117.027479051 1.161517002 3.572837433 6.47310245
-4.816265113 -1.886494939 4.785649311-2.442394855 3.265240573 -
4.465127187 4.979312819 2.072667609 2.346550801 3.834622713
6.105569465 2.813242742 2.278032618 -2.299606216 4.454464274

3.937392632	4.129201755	4.201253745	-2.217539874	5.078614058
6.322078878	2.937293586	-0.197671531	6.009144689	-0.983963491
6.068570385	5.201424268	4.588664451	5.544657583	6.298920841
10.31986961	1.5429665	2.459762666	0.660106427	-0.776968638
3.083573286	0.926352082	4.378557259	6.788695362	3.83042878
4.791889297	-2.179928876	2.101939267	0.034249718	3.293760219
3.646270377	-2.914948896	5.3520259	4.241678036	0.523818016
2.857590236	4.318326908	4.94734938	4.650033515	3.285633685
5.335286332	5.078348202	4.809576568	4.312474384	0.324459553
2.636350131	4.56609938	4.615061893	4.728731843	5.901915521
5.200173457	1.016667374	5.8257605	4.312510505	1.643221403
3.858306909	-0.917749049	-3.83820836	2.829337819	-0.131196697	-
3.33363928	-3.223649626	1.918085954	2.675387863	4.576953789	5.111454331
3.366590065	5.917119764	-0.85327469	2.945602712	4.612636411	3.63858168
2.574497234
TCGA-78-7149-01A	-1.043510087	4.757550836	3.087208464	5.523731856
4.048411388	8.238954992	3.576597788	4.232865366	2.138699576
3.211991885	5.320251263	-1.939709761	3.528208979	4.222599943
4.598781798	4.992302844	3.880128125	4.726668181	0.284244464
0.385301086	2.779681523	4.796672346	-1.630770059	2.061103212
4.571234217	3.220975178	5.549577626	4.28162302	4.455701419	3.79798207
4.930877773	1.284659542	-3.706952424	3.373704165	-1.349525806
2.717877418	-3.011140731	5.59775919	2.968761323	0.378234272
4.706162769	-1.185848403	5.212773716	3.056353463	4.004326375
4.19747741	6.85960796	1.437734846	3.758998241	6.743275629	-4.947559487
-1.636418402	4.506327193	-2.59717563	3.434375132	-4.51091886	4.990968539
2.30973868	2.192263308	3.901129006	5.960114619	2.937602779	2.130472153	-
1.591368763	4.507318269	3.933340599	4.476182056	4.088306187	-
2.703175268	5.084048357	6.444203055	2.977795273	-0.059645789
5.864952101	-1.01491376	6.115689297	5.133177136	4.78796912	5.49263976
6.273156956	10.4144748	1.107653905	2.422376373	0.914277854	-
0.733721769	3.039983262	0.804782133	4.437366449	6.937462383
3.942454434	4.655931785	-2.148451547	2.279620014	-0.197473977

3.35618061 3.966628278 -2.854479746 5.359586857 4.308195844
0.738703251 2.605397483 4.230932139 5.044089943 4.650397489
3.379370104 5.335577699 5.193136394 4.737793683 4.53634834 0.47382686
3.334785974 4.554787159 4.683358749 4.684840391 5.795204362
5.441026912 1.370818269 5.620392829 3.950614064 1.65313368
3.875073699 -1.375804534 -4.018915921 2.683205808 -0.348106449 -
3.595487532 -3.463779286 1.603955865 2.7769411564.330560285 5.064853554
3.45763207 6.10526073 -0.389675825 2.997960236 4.462722077 3.710142335
2.375121517
TCGA-78-7150-01A -1.510744917 4.595977659 2.397967051 5.493686619
4.157378928 8.393336072 2.901652692 4.244762817 1.7238264
3.798754131 5.762490282 -2.266791258 2.962308921 4.778987591
4.307868444 5.16648842 4.430375675 3.450197238 -0.159056517 0.30177565
2.814671457 4.748756806 -1.653677976 2.601285585 4.556232024
2.639477435 5.473531339 1.876430148 4.434605243 3.64800371
4.814747273 1.323539691 -4.183407138 3.171501116-1.0789191 1.838575968
-3.14045435 5.606585388 3.151312558 -0.076759741 4.683266443 -
1.345824574 4.774910444 3.199601701 3.85573068 4.237235197 6.949873755
0.779464807 3.086885172 6.841447521 -5.564955044 -1.871866949
5.279743311-3.214134972 3.442990216 -4.69590666 4.928787548 1.705356365
2.414886385 3.729641556 5.993826602 2.553812551 2.616445394 -
1.998643893 4.424806063 3.934967613 4.212973107 4.135761602 -
2.161560678 5.0743711736.371336556 2.789426683 -0.127201894 5.403051821
-1.399962983 6.133599527 5.062771575 4.682679829 5.493722222
6.33788611910.64621994 1.542634512 2.434198108 0.198894145 -
0.566045475 2.852832294 1.012816851 4.300678483 6.785140813
3.566605943 4.947721269 -2.331412259 2.196056763 -0.070718294
3.343091791 3.836835733 -2.653785858 5.339958007 3.974362721
1.093080155 2.983974463 4.174959761 4.910831888 4.582167596
3.194989417 5.335146974 4.879180306 4.900400651 4.239624436
0.346247444 3.085215727 4.569209904 4.663529321 4.723780026
5.844327874 5.259447024 0.976033187 5.733263602 4.145453018
1.492274899 3.679266268 -1.472208235 -3.930957261 2.680695466 -

0.657306058 -3.879264747 -3.793390962 1.528853636 2.315825854
4.373888407 4.885350803 3.274536396 5.879125841 -1.445415246
2.933409916 4.410986738 3.49702027 2.58956332
TCGA-78-7152-01A -0.885936708 4.932350193 3.063890334 5.507480689
4.26033304 8.30873157 3.437613005 4.357763015 1.9658785 3.066717601
5.61407037 -2.14582056 3.511153239 4.486023407 4.802696527 4.966122646
3.5117425 4.209507559 0.669860957 0.583831317 2.526840424
5.096844714 -1.415590495 2.11275747 4.536975409 3.062148458
5.657036778 2.911848774 4.52671222 3.654136042 4.872096064 0.922898517
-3.665474408 3.615687569 -0.934625907 2.758316733 -2.990897031
5.597992396 3.474823623 0.862189659 4.706977441 -1.120751505
5.019481577 2.823760034 4.058370177 4.326669493 6.860125555
1.359207464 3.611179018 6.778882252 -4.951279458 -1.795338657
4.682275363 -2.358681544 3.351641129 -4.612325061 5.098414521
2.344682346 2.186195037 3.9307669 5.999617368 2.70483441 2.054705091
-2.18840956 4.591719025 3.940883578 4.140980723 4.314644408 -
2.287912419 5.082815839 6.374598805 2.921638183 -0.028053165
6.074748824 -0.706497915 6.140971593 5.107814921 4.676148177
5.517692456 6.305463525 10.33663585 0.989165465 2.721902973
0.932422159 -0.660827468 2.991552054 0.975971584 4.36782092
6.880524025 3.708321989 4.73987966 -1.929779436 2.420929827 -
0.338627242 3.275640843 3.852316398 -2.688256158 5.352828671
4.443284823 0.631114502 2.884613136 4.059372498 4.997904866
4.763017257 3.160714758 5.335518685 4.879035881 4.841372426
4.362781101 0.449585666 2.646245595 4.482153435 4.685321011 4.734316277
5.722594317 4.889686026 1.149894248 5.662806802 4.4822709
1.724456172 3.873352714 -0.719959298 -4.376926056 2.738060878 -
0.309859081 -3.474389682 -3.635136202 1.743698581 2.846381407
4.579967457 5.117359944 3.264161644 6.090496867 -0.118600967
3.191406783 4.467549695 3.72567778 2.451571003
TCGA-78-7153-01A -0.924307757 4.921422867 2.754131958 5.55602422 4.11958622
8.28281063 3.571066824 4.295335556 2.199166448 3.317415131
5.697010938 -1.80487178 3.600544292 4.43885275 4.771976648 5.025319686

3.516670658 4.544342166 0.718762743 0.535738441 2.721622504
5.065551387 -1.598155472 2.3113363674.623622512 2.882933803
5.627556547 3.715865178 4.503298098 4.047615544 4.79185166
1.295943513 -3.687958843 3.5965774 -0.860855252 2.707401902 -
3.191901568 5.602696407 2.960261819 0.487260947 4.714380025 -
1.085887896 4.989817017 3.074360866 3.967260891 4.3472378116.993685811
1.359617233 4.126214232 6.864911394-4.826102176 -1.5231985 4.460646248
-2.122717763 3.451726639 -4.209556223 5.126804074 2.398673561
2.257074579 3.981795045 5.92732125 2.793442865 2.325962001 -
1.699267654 4.546021746 3.939740857 4.659529895 4.259509336 -
2.723515425 5.07889792 6.568655916 2.985558732 0.006975648 6.055157124
-0.853939739 6.168322768 5.070437722 4.791509252 5.544075157
6.38028942 10.35445617 1.132377494 2.686103635 1.230369604 -
0.654708644 2.671854985 0.803724651 4.40817371 6.921323837 3.749697919
4.771622691 -2.102139153 2.481940706 0.153490483 3.366952005
3.96612114 -2.899861409 5.360291388 4.331038683 0.640603875
2.856999998 4.067582083 5.071265172 4.624212748 3.398391392
5.335601713 5.081294599 4.742432299 4.776874579 0.492132748
3.128013676 4.409731086 4.625828341 4.715708473 5.884208084
4.980520681 1.392761489 5.404366602 4.07963739 1.502493356
3.902033788 -1.241058359 -3.725748357 3.156154263 -0.282783746 -
3.534040294 -3.156500134 1.579042012 2.7760749114.472769796 5.473684966
3.2301168836.261272862 -0.850385911 2.739174792 4.573380598
3.835064772 2.145664229
TCGA-78-7154-01A -1.062946493 4.952494561 2.250629394 5.614503047
4.324476647 8.222077362 3.391630932 4.224633819 2.115246184
3.773724676 5.604783211-1.998691559 3.6083215114.714476862 4.567765922
5.177515427 3.8511744093.904348726 0.479229082 0.331507284
2.732763533 4.778976571 -1.512000726 2.365403374 4.628442821
2.434338326 5.496591548 3.3311475874.499082515 4.086434718
4.730737188 1.24749714 -3.856618932 3.501572637 -0.947264709
2.092587152 -3.279972043 5.613458527 2.723048799 0.185290242
4.689642571 -1.177122668 4.756961495 3.003839429 3.840907461

4.3260711526.963934707 1.040489647 3.832001869 7.026237985 -
5.402599839 -1.689201773 4.947796348 -2.325630186 3.444217482 -
4.601549168 5.05547037 1.987380258 2.646333652 3.62282881 5.91367703
2.559591095 2.253651316 -2.569807778 4.496228519 3.940536203
4.176786378 4.457699333 -1.484531582 5.073517852 6.330342081
2.86134877 -0.070866944 5.750125056 -0.836435124 6.168157098
5.050797566 4.636690813 5.4858439 6.228818598 10.68200101
0.710050997 2.67416337 0.695877421 -0.665453461 2.718676323
0.969160924 4.316168507 6.787468649 3.589140468 5.010883002 -
2.443652378 2.557826423 -0.093137166 3.349459761 3.782779489 -
2.38886384 5.343896786 4.131473018 1.045818557 2.672224972 4.153280904
5.015474485 4.568971319 2.939528923 5.33531926 4.65966186 4.894744225
4.2615568 0.471873234 2.809740427 4.30048609 4.650622406 4.759369947
5.692207623 5.055374413 1.08488972 5.325288299 4.099636743
1.597957647 3.761930158 -1.500349827 -3.828213983 2.845865322 -
0.525257619 -3.830025561 -3.228980414 1.593343529 2.4196115394.261969124
5.330649815 3.697330016 6.135363242 -1.484008752 2.666769553
4.309023189 3.591043275 2.24820003
TCGA-78-7155-01A -1.649656665 4.970355968 0.52581773 5.414234908
4.437053018 8.165359754 4.366333661 3.9173644112.922273854
4.534398472 5.184699789 -1.335658699 2.313282048 6.039466811
5.092600818 3.964608492 5.335953991 4.51923757 0.457775798
0.389636391 2.915340179 6.051477301 -2.256775796 3.080653613
4.84496523 1.994915865 5.79680788 2.317103593 4.670455764 5.847704364
4.81609703 2.221395893 -3.6421675 3.018508617 -0.226750246 2.07917664 -
4.9119575865.5762092114.12840708 2.052941247 4.662103662 -1.247935863
5.362588446 4.828477924 3.347855498 4.206332051 6.801559246
1.745426206 4.791564741 7.845004064 -5.572009919 -1.293837343
4.624753338 -2.703030688 5.126644428 -3.905462216 5.360015325
2.0562949113.56894156 4.153271592 5.510899023 2.810703362 2.360402655
-2.222388145 4.626634023 3.9431162914.367725725 4.475715434 -
3.425585536 5.054193102 6.569509877 2.586745713 0.993079778
5.058110867-1.202498072 6.158979825 4.870488944 5.285848944

5.195854901 6.829563892 10.23016529 -0.922160749 3.62362123
2.054532761 -0.459561474 3.205001346 0.551334285 4.128197457
6.90603183 3.136451875 5.486554668 -1.535952502 3.079847299
0.574013348 3.4415748 4.420091834 -2.919312592 5.326371609
4.777213929 2.307764319 3.752014538 3.22542547 4.95931337 4.541944896
4.206057022 5.335361076 4.453309819 5.1135457124.236659908
0.431484791 5.439291606 4.570236507 4.518346341 4.772011902
5.668449762 4.603520837 1.460843828 5.097458431 3.898004617
1.0506413 3.81290574 -1.489071714 -2.996398639 3.368024537 -0.658311338
-4.265577485 -3.94212591 0.765086235 1.671229667 3.410740141
5.659453558 3.680139194 6.658537129 1.671820347 2.005711559
4.648237857 3.671765878 1.923912645
TCGA-78-7156-01A -1.454635215 4.4571145823.370012661 5.539893734
4.136956189 8.292501777 3.428893332 4.246732075 1.936974034
2.9506115055.137699471 -2.146421249 2.921260768 4.661892735
4.397946922 4.894546145 3.749135699 4.25635267 0.400741805
0.306879976 2.621834866 4.993200651 -1.785505225 1.983975297
4.5731131723.11873793 5.555868252 3.36499325 4.308169719 3.202529099
4.696806607 0.935665981 -4.057447675 3.406104452 -1.379030095
3.017477136 -3.300617928 5.584746095 3.22582753 0.984942023
4.696825256 -1.363839084 5.077683596 3.515990742 3.936397352
4.160597573 6.904752812 1.400434748 3.9044011736.335139304 -
5.00405869 -1.69090926 4.4402805 -2.648627395 3.239524142 -4.613169366
4.830343009 2.061292843 2.519365491 3.795390477 5.855851789
2.891031989 2.04615107 -2.463191413 4.40671153 3.942790812 4.247376747
4.036681985 -2.323830133 5.073402365 6.264930063 3.000283195 -
0.092830235 5.959287465 -1.295024861 6.050009082 5.241243213
4.565167525 5.455334498 6.421456361 10.28869884 0.803794997
2.40997949 0.87341686 -1.073114762 2.747842266 0.649464084 4.380386104
6.865419126 3.891230134 4.623168903 -2.22385206 2.045919018 0.02920926
3.29101505 3.706341691 -2.795075888 5.360468421 4.462819805
0.629988219 2.585556755 4.280087162 4.936294727 4.728271518
3.608450994 5.33520213 5.21661731 4.71549159 4.38624402 0.122064225

3.665513351 4.515203569 4.577717597 4.727937189 5.788507087
5.480446901 0.965799194 5.673791994 4.207853832 1.625856066
3.84739539 -1.20345079 -3.795068846 2.791094039 -0.144040975 -3.463473733
-4.015782819 1.613419736 2.471938132 4.171849669 5.144938344
3.450086649 6.073142298 -0.672433169 3.036924322 4.516785391
3.542232628 2.143315486
TCGA-78-7158-01A -1.238184895 4.876169273 2.504166061 5.454364405
4.247432367 8.15019199 3.606007775 4.222695484 1.9401143732.878619247
5.26663643 -2.133835505 2.756084725 4.5925118794.701826843 4.624243545
3.853859026 4.134448149 0.260359441 0.306219314 2.430747212
5.27616946 -1.812109842 1.710638364 4.6489001122.624944377 5.701620889
1.421459233 4.370053686 3.730012754 4.830202427 0.988344154 -
3.709013715 3.194508964 -0.933021708 2.629001301 -3.893002812
5.596735505 3.4354582 0.6967384 4.678847677 -1.24675951 4.855869176
3.479531967 3.805858414 4.248517717 6.607428193 1.3142831154.08360656
6.741112354-5.284957181 -1.798417264 4.762922329 -2.208444318
3.237081298 -5.091522771 5.01494169 1.8281120062.140632692 4.035528906
5.663400461 2.322388279 1.867789502 -3.207992135 4.613674955
3.940814153 3.856798365 4.212079411-2.617182543 5.077173707
6.3511742682.968359998 -0.08634076 5.857494854 -1.225082883 6.206282592
5.02185891 4.66744702 5.423954425 6.414248406 10.26088461 -0.001033497
2.810324936 1.138501347 -1.04492609 2.455297798 0.657574472
4.279637202 6.879202838 3.472713003 4.856915556 -2.081931942
2.343068885 -0.814353569 3.251065747 3.706688788 -2.707751446
5.3611903894.353531404 0.821964342 2.760862762 3.587034974 4.99163969
4.8593901113.293045261 5.335450194 4.696874292 4.716509408
3.865955632 0.295846739 3.065674894 4.272538295 4.603163948
4.722480693 5.570719197 4.706498166 1.065579125 5.330054284
4.067345892 1.440300648 3.778665443 -0.854887906 -4.557826422
2.310087577 -0.293298898 -3.72243842 -4.333027964 1.17862738 2.450889562
3.934859028 5.336576089 3.538674241 6.235735548 -0.22217966
2.589993554 4.221380658 3.678722394 1.983970172
TCGA-78-7159-01A -1.152430112 4.522388208 1.8663501125.391750487

4.236035843 8.335566503 3.76727439 4.198173003 2.081036187
3.683834997 5.363613264 -1.964977821 3.210606278 4.888636123
4.79584425 4.798688412 3.9283731194.321833724 0.875420387 0.410830409
2.70898181 5.55435046 -1.626726389 2.599600312 4.695247279 2.567460795
5.638182804 2.751232167 4.506227292 4.217897054 4.723382079
1.264184714 -3.713515491 3.515213641 -0.704377297 2.621754625 -
3.781746806 5.598159541 3.617508451 0.527574525 4.692645601 -
1.307703178 5.535814913 3.486578184 3.84358907 4.31985412 7.021100534
1.48877774 4.025394755 6.817424709 -4.948641802 -1.61401455 4.810997624
-2.24356881 3.678037957 -4.314440353 4.980120452 2.02922357 2.458548913
4.242445654 5.884589237 2.775088962 2.342425434 -1.728053103
4.546364177 3.946377031 4.33832167 4.245120281 -2.159273312 5.07463597
6.399278973 2.960675347 0.140921068 5.759680058 -0.780622581
6.018750459 5.13085035 4.834426091 5.4374118986.57677337 10.44514658
0.710268193 2.860076809 1.353690415 -0.899565699 3.082352966
0.676781537 4.263850045 6.825721972 3.643843085 4.908430607 -
1.94875227 2.439306997 -0.024876087 3.337701369 3.832908855 -2.60107395
5.347961481 4.725996631 1.23956414 3.231415604 3.74030933 4.942363246
4.773476868 3.556076871 5.335396154 5.034950616 4.886395574
4.2681189260.376343284 3.535073191 4.414842704 4.530606001
4.740238486 5.705816699 4.924773109 1.028990255 5.421174601
3.742721552 1.628155626 3.870288002 -0.509933018 -3.544516566
3.418950189 -0.332591036 -3.506278649 -3.477401026 1.219237315
2.519684039 3.94545802 5.488249498 3.590390713 6.132269968 -
0.156613849 2.384626539 4.540861635 3.717186477 2.718319166
TCGA-78-7160-01A -1.126007463 4.406142739 3.11070439 5.5281155054.091117273
8.374572941 3.034445836 4.395732708 1.652405402 3.259878203
5.443877881 -2.390485617 3.320190626 4.362139648 4.312428474
5.088456036 3.556190949 3.6375027 0.537218844 0.415400096
2.502050438 4.855760077 -1.590168227 2.614876892 4.52263396
3.049608505 5.755109197 2.226307109 4.288079183 2.623437796
4.703173401 0.769353988 -4.069596205 3.543511206-1.26421987 2.277746274
-3.167669525 5.585433126 2.94694105 0.749868995 4.682071502 -

1.439619955 5.159426421 3.034769927 4.000796484 4.342007637
6.628680465 1.17059404 3.0167425116.496650233 -5.180337107 -1.962754993
4.589105961 -2.591301865 3.272787889 -5.183787002 4.752258284
1.556481422 2.1146051683.88779901 6.1178456982.629679996 2.466604295 -
2.952405651 4.569619282 3.934945232 3.690857543 4.219581068 -
2.250033584 5.080403086 6.24726786 2.875785857 -0.285519629 6.067241245
-0.966542601 6.010338543 5.165948085 4.25396873 5.528539045
6.331058987 10.30258427 1.262709283 2.309153032 0.386142935 -
0.897538029 3.439622385 0.952173162 4.294455716 6.761449706 3.662072
4.756684006 -2.175620702 1.841009972 -0.492779765 3.263638625
3.770054438 -2.504621174 5.347574046 4.701814002 0.947589816
3.006808151 4.088568719 4.872057631 4.603801448 2.830267187
5.335145187 5.1190009134.867542876 3.96167305 0.295617518 2.909010763
4.609120562 4.619373381 4.742178674 5.699143051 5.300055956
0.760334898 5.884268852 4.2260663111.735177926 3.657915395 -
0.345246007 -4.312487916 2.517264833 -0.526206526 -3.556081979 -
4.034150474 1.703374821 2.604220612 4.478839083 4.70249448 3.088806817
5.777707952 -0.779895156 3.024525997 4.250825705 3.536739057
2.545989728
TCGA-78-7161-01A -1.07159316 4.495156521 2.636451341 5.320290196
4.107973853 8.340679409 3.559423085 4.17924668 2.100276966
3.152276149 4.970573681 -2.27932292 3.152761777 4.483376327
4.695732925 4.907616923 3.703976279 4.260342555 0.833156086
0.467779008 2.729047475 5.442238756 -1.57268597 2.148763575
4.6306811122.955676004 5.51027707 2.893435588 4.392583312 3.310708693
4.809198654 1.050384561 -3.737555865 3.490273476 -1.157943484
3.01444669 -3.237812031 5.597199927 3.29103824 0.485233819 4.698694503
-1.304003382 5.4171158333.365832294 3.985013338 4.257675341
7.080972986 1.638717447 3.916537316 6.651854367 -4.70871286 -
1.606506056 4.777986157 -2.202521907 3.354082301 -4.613242707
5.032544665 2.137322377 2.335194165 4.133990473 5.884806704
2.728617024 1.945823267 -1.873179551 4.538289645 3.935108307
4.398037527 4.163150438 -2.330845533 5.074937307 6.445587999

3.086114673-0.024774307 5.680300844 -0.895229378 6.041740585 5.19882937
4.948122014 5.445836545 6.629854248 10.45865261 0.651109933
2.662566418 1.139203282 -1.006815676 2.994590284 0.666385328
4.2696235116.878374073 3.7578285 4.632277306 -1.911131457 1.990425627
-0.177843188 3.282365268 3.850392148 -2.572812798 5.338758213
4.675186106 1.195714448 2.942044791 3.881015606 4.947043544
4.8237508 3.682661425 5.335360787 4.99449472 4.823478932 4.218799697
0.388651961 3.637374303 4.524173506 4.562079219 4.71055464
5.571830349 5.046481955 0.987415139 5.508308233 4.02893507
1.689877004 3.930413189 -0.27246474 -3.591971623 3.130660113-0.305191686
-3.317249065 -3.477002003 1.424937127 2.627988824 4.039250124
5.381253516 3.464555238 6.153569408 -0.778592101 2.824558883
4.418996216 3.774489872 2.62813798
TCGA-78-7162-01A -1.163033074 4.571036255 2.840497752 5.508233404
4.161604072 8.341305327 3.3904125 4.283890405 1.862250409
3.456910819 5.403723956 -2.112250032 3.366543553 4.553478548
4.652544638 4.995696099 3.812837523 4.027062859 0.39604115
0.436653592 2.712624337 5.111243108-1.654130849 2.804237651
4.5089081133.184713054 5.727302682 2.970246192 4.459638917
3.321536175 4.894478758 1.136732671 -3.821115823 3.701484709 -
1.178968668 2.590700982 -3.237646562 5.58246279 3.503442235 1.111687004
4.709352676 -1.248351359 5.233637722 3.195172125 3.968135703
4.261062746 6.929548009 1.47930356 3.42866163 6.604785543 -4.878750116
-1.713692333 4.290641093 -2.781640258 3.425697267 -4.455785291
4.9452421131.8523119992.338367044 3.943635267 6.162561606 2.881732314
2.367735764 -1.805251676 4.572466164 3.936279423 4.085728843
4.066475865 -2.626707328 5.086532441 6.429889044 2.889217751 -
0.078322145 6.117244182-0.764642025 5.969448366 5.181426008 4.545247193
5.4940355116.334012909 10.489311751.173007794 2.480033323 0.703472455
-0.778253259 3.362240301 0.847397575 4.344022425 6.880771273
3.739368433 4.664252276 -2.067990321 1.979600244 0.011609322
3.326804434 3.856613513 -2.803507763 5.344879052 4.784406949
1.16122863 3.059677376 4.197544971 4.928984763 4.607503956

3.156468507 5.335418952 5.232061463 4.874680146 4.449209331
0.3611897323.413978197 4.664870938 4.67823536 4.7537119325.831995157
5.497319629 1.126742935 5.75844907 4.386441232 1.862396944 3.78139428
-0.853525795 -3.939298333 2.984750565 -0.348958782 -3.467487827 -
3.721405781 1.804820452 2.69486347 4.512548048 4.859958817 3.123761622
5.954952376 0.035365653 3.205155361 4.453078551 3.685454563
2.768727779
TCGA-78-7163-01A -0.646296452 4.700381844 2.979080755 5.520206172
4.588058096 8.176475379 3.073989307 4.236671266 1.922724066
3.029683768 4.995374843 -2.682907824 3.562651893 4.838112835
4.714390713 5.342439083 3.465973884 3.99906281 0.378175787
0.380615767 2.884069309 4.816784228 -1.083892724 2.017014916
4.483654875 3.075019464 5.28878662 4.185819931 4.630235826
3.518632624 5.1121771630.959021176-3.977900932 3.346233971 -1.244202307
3.580551933 -3.15152515 5.616313048 3.697049763 1.129394406
4.738254551 -1.104319839 5.056942439 2.966126933 3.995911233
4.155433436 7.444851826 1.493051876 4.538955282 6.450799978 -
4.846588459 -1.884145391 4.751839271 -2.39610545 3.088528799 -4.42528646
5.135529906 2.667956904 2.955730652 3.632643577 5.941310555
2.453554191 1.832587649 -2.326716745 4.476041076 3.954924842
4.39963724 4.204607401 -1.185730887 5.060620222 6.2731197033.008860876
-0.146267258 5.676598445 -0.717969606 6.1207581185.236495986
4.962468601 5.532066026 6.172438861 10.55929214 0.721431362
2.591825739 1.339557209 -0.830336405 2.406508782 0.8646805
4.423372568 6.839664958 3.778456645 4.675634273 -2.272956609
2.497930169 0.281221243 3.334655461 3.407206461 -2.26842693
5.369448251 4.138056343 0.125467196 2.389676269 4.00479594
5.062146187 4.91159337 3.639555632 5.335361806 4.80548043 4.91877829
4.552351713 0.68178235 3.4319106114.509831306 4.858434544 4.736681001
5.69676526 4.992073168 0.689287879 5.412466928 4.449108688
1.769523626 4.147911161-1.176693983 -3.368667817 2.769092026
0.160389625 -3.378869425 -2.981777246 1.899038398 3.205227629
4.683743123 5.678655608 3.757592483 6.523462985 -0.397224062

3.15752227 5.13732035 3.700588444 2.91666958
TCGA-78-7166-01A -0.958753212 4.969365428 2.897818478 5.396290451
3.998682321 8.258638829 3.1455118054.3116681332.170424084 3.242470581
5.393653036 -2.155489031 3.695086904 4.342697774 4.494513211
4.990800907 3.592223851 4.205081632 0.327305648 0.445587494
2.697393161 4.791674218 -1.389054312 1.651358296 4.55397758
3.003426081 5.670101778 3.81044736 4.439408592 3.130778627
4.874576183 1.098006698 -3.858331143 3.059794233 -1.341175521
2.504340197 -2.60189687 5.613481672 2.484572275 -0.182615952
4.706048872 -1.138788432 4.946917134 2.598197482 4.07014848
4.280537902 7.081210423 1.120483735 3.846853751 6.89275782 -
4.896890848 -1.615659752 5.049525364 -2.438521986 3.445783164 -
4.608732439 5.132464826 2.171487421 2.499729479 3.778885401
5.918575156 2.568426867 1.797782706 -1.318637478 4.429578971
3.920627 4.348633078 4.209757089 -2.024819668 5.078857447
6.456371353 2.992651975 -0.176618267 5.650125195 -0.943845908
6.194618574 5.06209132 4.97376557 5.476802985 6.272765002 10.44490333
1.172175451 2.475624881 0.572751191-0.468931455 2.939595502
0.847669447 4.362670053 6.867140253 3.644449994 4.684859619 -
2.204213767 2.018389894 -0.069673776 3.29094887 3.879731727 -2.813832424
5.336215705 3.985981452 0.433622489 2.685226449 3.933564801
5.0165372 4.669430323 3.43748743 5.335351216 4.846446458 4.794099009
4.534127157 0.500882399 2.3145116474.674628915 4.672857858
4.698221591 5.744034806 5.019868438 1.1763171195.566766949
4.285124845 1.540038876 3.940475891 -1.196212732 -4.050070524
2.892705247 -0.244036687 -3.571420529 -3.01159893 1.789566579
2.597135999 4.363056779 5.347829454 3.279881756 5.980374086 -
1.923559415 2.871580279 4.640785694 3.7451511392.587782499
TCGA-78-7167-01A -1.191953664 4.589462757 3.1151307995.325049382
4.252436706 8.284050215 3.551310452 4.169896168 1.925608574
2.915672856 5.146994677 -2.238832063 2.959902386 4.622367273
4.510850082 5.128860919 3.961998604 4.2921779110.380867829
0.350532168 2.733494708 5.172140749 -1.671756905 1.951378192

4.60724948 3.090274308 5.588315239 2.9461144494.387623826 3.406640107
4.848236914 0.961118176-3.894153821 3.005971752 -1.441036164
2.861900136 -3.416700806 5.593318706 3.616536122 0.915935394
4.696708213 -1.316551695 4.821035182 3.5469522 3.933073985
4.139454038 6.95008934 1.489817683 4.015229282 6.520726517 -
4.887247724 -1.593351771 4.810420395 -2.477311839 3.251478611-4.611296494
5.036717177 2.196068777 2.139471341 3.873422479 5.851921912
2.850833558 1.753681244 -2.029716689 4.500357191 3.941432326
4.223632337 4.040953885 -2.115873396 5.068056189 6.36070906
3.048420475 -0.114971474 5.820919772 -1.09150318 6.1145292715.201059495
4.74894575 5.410883306 6.396682043 10.27778551 0.523737133
2.570195509 0.786631672 -1.143867563 2.810304801 0.547638501
4.343860245 6.914571531 3.86663413 4.603035068 -1.988092012
2.134486736 -0.217030874 3.2728089113.685091366 -2.667854878
5.368675049 4.35932039 0.881352078 2.553780235 4.098483399
4.943287206 4.805206473 3.699054314 5.335208476 4.933248689
4.656296599 4.29568922 0.313205656 3.744498482 4.368991696
4.618739314 4.703228619 5.68297075 5.301713689 1.090938276
5.694890663 3.896572051 1.659748644 3.927317802 -0.821186 -
3.929984492 2.551463482 -0.238239679 -3.423733342 -4.113758874
1.575983646 2.617151904 4.052032591 5.256778368 3.813282076
6.254787127 -0.85124531 2.76345873 4.484615129 3.657934706 2.037734579
TCGA-78-7220-01A -1.658398111 4.712888832 2.623503591 5.462714301
4.165181213 8.335614579 3.304693652 4.157642827 2.07827055
3.557982705 5.527896789 -2.12558457 3.155384833 4.947231791
4.532251646 5.10266823 4.362422681 3.795174375 0.1811089760.351666861
2.7103114094.849313462 -1.999196326 2.354947352 4.646847562
2.393014714 5.575999609 2.269375645 4.35735333 3.849446533
4.698027901 0.999686553 -4.127231786 2.906632936 -1.176248133
2.018568339 -3.515343775 5.606812879 2.91628134 -0.050377649
4.676197075 -1.368772731 4.875841664 3.268885407 3.838658726
4.230828734 6.936407948 1.08224216 3.690015836 6.827429726 -
5.467781858 -1.931251811 5.173828742 -2.659007923 3.645467851 -

5.045107098	4.903410556	1.538950729	2.309954553	3.867577549
5.8201189512.765715053	2.271814088	-2.289676937	4.427162145	3.93998942
3.961730667	4.222390752	-3.130764674	5.067181273	6.335199351
2.913153046	-0.190797626	5.393532716	-1.410390978	6.155127586
5.079988236	4.639518419	5.43454629 6.289892616	9.973127399
0.997020076	2.646848792	0.581569358	-0.876891165	2.953477737
0.821527689	4.307715559	6.75902098 3.574877264	4.957878912	-
2.0911002162.298784758	-0.021372056	3.257682952	3.847969943	-3.217593256
5.3481105484.160194223	0.815595795	2.814499215	4.27852021 4.892395155
4.573454261	3.15206566 5.335051803	4.874177022	4.8033911644.167514739
0.280713782	3.36279798 4.544033372	4.519342543	4.724380161
5.748391084	5.08738525 1.028932486	5.5068711	4.221318987	1.137119602
3.707426111-1.444993282	-3.91888696 2.264793093	-0.789117362	-3.871847274
-3.792041335	1.531291282	2.269016974	4.06083323 5.302756208
3.8690941145.943993899	-1.666541441	2.514448522	4.446374798
3.555578899	2.430485628
TCGA-78-7535-01A 0.460787369	5.1481146532.076865622	5.240579106	4.36310154
8.179942642	3.426178273	4.267020696	2.307528831	3.453227422
5.227299703	-2.059434418	4.241430247	4.721005596	5.215367492
5.150153144	3.828247542	4.432588752	0.372257133	0.64598776
2.934139017	5.366272276	-0.598380634	1.620593734	4.567033694
3.146053636	5.530704902	3.623512262	4.757107302	3.729657017
5.120717131	1.532339334	-3.278540246	3.034195952	-0.866372412
2.98199755 -2.463823192	5.597317079	3.0592112470.959197136	4.729744321
-0.892131472	5.033070061	2.592496072	4.1101343334.305520684
7.069912017	1.7116952914.270584368	7.412680946	-4.418295686	-
1.517414512	4.763816075	-2.286378041	3.939712003	-3.685455915
5.485964538	2.962321291	3.209591037	4.000773364	6.076212852
2.671026652	1.376609206	-0.951152872	4.652055423	3.927899463
4.694656864	4.41704469 -0.387639682	5.081581528	6.59185641 2.881974856
0.169133332	5.59967915 0.081217125	6.186739738	5.0113148765.140163098
5.481736032	6.253693515	10.57413186	0.486864481	3.012320813
1.219614929	-0.168210921	3.436058019	0.999029149	4.306980305

6.956544674 3.534374841 4.899232993 -1.791739768 2.641305834
0.418370382 3.349590878 3.854903817 -1.787079811 5.341689364
4.350420284 1.183837937 3.174598483 3.656397268 5.106503816
4.758538177 3.650942536 5.335718832 4.463427901 5.08680861
4.792414856 1.142644895 2.949462829 4.777839699 4.958455029
4.72104281 5.695296374 4.833691474 1.367033903 5.474354597 4.54737907
2.213599121 4.0965374 -0.802037208 -2.79494058 3.563680671 0.139939506
-3.459802615 -2.251932978 1.87569035 3.128853779 4.600380935
5.579910811 3.292000393 6.401510631 -0.365732016 2.923576247
4.793750273 3.883115013 3.08540828
TCGA-78-7536-01A -1.248169872 4.860674421 1.19948531 5.303493173
4.505024796 8.267766993 3.770029286 4.131260448 2.243338007
3.715123275 5.29797677 -1.774133735 3.089136079 5.281304053
4.970158899 4.615969348 4.384093838 4.197584558 0.385428941
0.41735834 2.691316805 5.435741337 -1.728245217 2.015495165
4.745894725 2.106425746 5.853502506 1.8357655 4.56695983 4.729830027
4.642401896 1.302906932 -3.694878524 2.942410361 -0.707131072
2.431213284 -4.399346123 5.595426304 3.403548332 0.331801571
4.675327333 -1.165623486 5.301544675 3.877380291 3.691197768 4.34714822
6.99923414 1.573465725 4.497853264 7.061141628 -5.461745294 -1.732393196
4.810393024 -2.211162906 3.894219065 -4.4906936 5.220371355
1.972062429 2.678751194 4.166267764 5.724460266 2.728771846
2.018350614 -2.259192326 4.513814862 3.939026789 4.129930271
4.365767032 -2.194313847 5.063424328 6.484570168 2.882777699
0.229780564 5.699506399 -0.793637596 6.1794992 5.05184769 5.048976072
5.338419494 6.624755684 9.822016255 0.226999656 3.153630186
1.538252522 -0.97365063 2.980381741 0.518749511 4.199386289 6.886254325
3.472250087 5.240644969 -1.807544055 2.673225043 0.04129381
3.317130051 3.884302755 -2.743305066 5.34464248 4.417795443
1.443501413 3.433049172 3.656907507 4.910984267 4.712943454
3.74049738 5.335182535 4.53838716 4.910320755 4.23935185 0.415638315
3.977331284 4.301981999 4.511310285 4.743480162 5.684989923
4.732684062 1.152969039 5.283007181 3.980527349 1.373034862

3.892971588 -0.701401929 -3.677414232 3.148305058 -0.374528516 -
3.909087439 -3.699218668 0.985627327 2.12676305 3.746076402 5.777289983
3.867253416 6.347033844 -0.290242037 1.88813882 4.685941783
3.805239724 2.138627574
TCGA-78-7537-01A -0.752971937 4.77646212 2.975602096 5.509335129
4.306803839 8.342587354 3.450616393 4.295970182 2.067135364
3.101737133 5.53975121 -2.00852019 3.669483421 4.31600131 4.688186148
5.305311417 3.643752875 4.435956658 0.625589952 0.588301764
2.804768973 5.153545688 -1.133207043 2.376335533 4.542586327
3.252747134 5.561641221 4.475640689 4.582159812 3.546348182
4.929335641 1.136640691 -3.744577019 3.745641786 -1.071814508
3.098233343 -2.502549515 5.59043957 3.482961025 0.867534634
4.720840583 -1.190117372 5.243892984 2.987895877 4.098872016
4.27968261 7.047210739 1.703402182 3.8676368 6.796812446 -4.595321128
-1.489743958 4.602737477 -2.557517713 3.3246773 -4.100538722
5.099704821 2.570350224 2.438119509 3.970073173 6.156490206
2.947482632 2.011106913 -1.351296881 4.555140707 3.934956631
4.699179231 4.10132386 -1.403534174 5.079590697 6.49000749 2.997403108
-0.047775083 6.051197725 -0.584711944 6.076835717 5.223021752
4.767346819 5.501452116 6.368191442 10.45437074 1.364383902
2.555621493 0.95425285 -0.555930295 2.930124817 0.86784861 4.462105156
6.929829944 3.887164327 4.631657872 -1.952796812 2.259939823
0.015463728 3.322965241 3.847612239 -2.289835903 5.354183043
4.597993352 0.548764446 2.835424095 4.243699064 5.015482645
4.744052763 3.352205067 5.335487375 5.243513645 4.810945426
4.587044117 0.571306433 3.173345356 4.54412584 4.715024681 4.724853092
5.826048493 5.434464029 1.225781127 5.700647304 4.217096952
1.962289244 3.983312033 -0.781156934 -3.552004182 3.043972588 -
0.128661561 -3.305542241 -3.091578887 1.904829663 2.975819866
4.634925568 5.201055285 3.337137224 6.147943445 -0.0800877
3.403080185 4.61384846 3.690849671 2.633966795
TCGA-78-7539-01A -0.285821561 4.579631948 3.327086226 5.339924394
4.288297087 8.277332551 3.332518097 4.29140758 1.702759698

2.875465322 5.399241955 -2.534336344 3.440458381 4.691010116
4.8296661155.168217505 3.872058098 3.968356142 0.125880683
0.463315539 2.7601711675.118628301-1.259406951 2.655046977 4.51615393
3.316312823 5.49217019 2.125180678 4.505644383 3.477661503
5.037993382 0.903232533 -3.459533789 3.300801745 -1.144179539
2.974098228 -3.504911638 5.599390712 3.831639447 0.951856916
4.710166701 -1.22518184 5.254686027 3.236172674 3.976503707
4.177820246 7.021686132 1.26543637 4.046422914 6.57156653 -4.704857308
-1.987557969 4.723085082 -2.604548653 3.341144601-5.019535998 5.02146039
1.938371289 2.062092867 3.861912912 6.085778245 2.789701406
2.358013634 -2.32025701 4.619123586 3.942501629 3.943455541
4.148224819 -1.802384463 5.084497485 6.245228764 2.933489327 -
0.139155392 5.86301579 -0.512361337 6.099190305 5.128969831 4.737413084
5.510051091 6.38081215 10.45930069 1.023519354 2.763964956
0.704971612 -1.02869249 3.232261874 0.90996603 4.305801855 6.839945442
3.762602951 4.709445223 -1.935787305 2.28435902 -0.19762198 3.263458682
3.618590244 -2.293411052 5.370910078 4.353327228 0.934276496
2.964774528 4.103677442 5.027741947 4.799335141 3.420157984
5.335555189 5.227649518 4.920264229 4.23047201 0.677626049
3.925500093 4.668780643 4.844617191 4.720546592 5.799162222
5.468600951 0.75500076 5.637781291 4.141489507 1.98853045 3.937032091
-0.653181087 -3.771714703 2.453742862 -0.055327405 -3.543578291 -
3.778056638 1.757761093 3.043456597 4.584720708 5.367910891
3.565239496 6.303277008 -0.11029625 3.171856185 4.655804352
3.752906188 3.032720286
TCGA-78-7540-01A -0.547854924 4.919574629 2.669321017 5.428410969
4.315545451 8.3333571192.9611694624.393594387 1.692393284 2.701953398
6.010669426 -2.756897569 4.489693614 4.366865846 4.55835006
5.738126099 3.510145197 3.581059632 1.031202785 0.694695922
2.686940968 4.670707966 -0.749485951 1.986261835 4.383776193
3.270283391 5.754165165 3.5611724284.584051685 2.7071192564.889872
0.710681524 -3.719697625 3.630767024 -1.514558287 3.157092126 -
1.917973746 5.607233435 3.620373222 1.054225459 4.73476688 -1.151814296

4.971938716 2.309377453 4.2684911714.367883624 7.249888126
1.593900546 3.63454378 6.340781636 -3.852692661 -1.964245759 4.99317223
-2.207395475 2.806310699 -4.57770556 5.173824159 2.305983566
1.8311895693.685137146 6.340602435 2.931097057 1.545373335 -
2.340746563 4.535530722 3.934521266 4.043892272 4.379181191-0.081678406
5.083435233 6.131612106 3.122808596 -0.368911099 6.145384984 -
0.219158301 6.081709201 5.346769752 4.492563556 5.57625958 5.852286327
10.93649652 1.817092312 2.40677238 0.407071187-0.597013416 3.216340714
1.059623171 4.51105534 6.750327669 3.948032949 4.832979785 -
2.01471294 2.123306633 -0.502285715 3.195668075 3.118242545-2.27877763
5.369186448 3.988497277 0.268041457 2.949837442 4.758316083
4.9803701184.80057091 2.587825196 5.335272421 5.313735586 4.758645554
4.157960319 0.619936013 1.82032402 4.56491872 4.761095491 4.751450611
5.948121503 5.494732638 0.832976074 5.8082595 4.57668047 2.23656047
4.021712821 -0.517657386 -3.639423125 2.214875863 0.645804388 -
2.776769318 -2.876533927 2.569160948 3.131761093 5.041686349
5.175932974 3.6123112646.000357144 0.16193283 3.63338675 4.665124505
3.694842961 3.555983941
TCGA-78-7542-01A -0.50818503 4.461376454 1.51170341 5.37832255 4.61746583
8.134133805 3.026190429 4.27168029 1.453243628 3.358758086
5.390673368 -2.844689728 3.9172798 4.837080662 4.170940848
5.298408462 3.943496148 3.560535059 -0.045334072 0.35470404
2.547970052 4.723207102 -1.282489676 2.129937685 4.500539036
2.65523279 5.215018032 1.849671846 4.51045638 3.549381679 5.250427681
1.242606434 -3.744742574 2.812818982 -0.781349632 2.441156715-
3.5411428935.657509537 3.852342275 0.553509381 4.707149715 -1.268637009
4.93893349 2.449107545 3.874853988 4.03982769 6.972204108 0.997302715
3.857804149 6.884007766 -4.835165769 -2.047689855 6.116383563-
2.245901486 3.111822372-4.976479799 5.021560966 2.168852758 2.966966315
3.132301541 5.948072325 2.150656308 1.817256633 -2.525142051
4.608981235 3.939164122 3.457982468 4.349310533 -1.515456469
5.070558915 6.066326073 2.651531887 0.033324621 4.879905788 -
0.651489585 6.150059863 4.947033992 4.801634475 5.392797278 5.99320148

11.622572180.319561603 2.79192575 0.714318624 -0.361458249 3.001397742
1.010688492 4.310490144 6.725623027 3.294107851 5.029826409 -
2.225176691 2.236298703 -0.243449793 3.268557503 3.327562856 -
2.561788549 5.355956074 3.4960211880.176849951 2.597744467 3.216579071
5.01906494 5.218019371 2.890130039 5.335334927 3.762750683
5.066181248 3.755389859 0.736437439 1.925267709 4.51938238
4.964765558 4.740141335 5.626131238 4.731413333 0.479881159
4.860128888 3.992948436 1.770202332 3.876404932 -0.482561914 -
4.425283725 1.977949635 0.277897696 -3.792791742 -3.114135278
1.697684412 2.672539402 4.554610704 5.075315541 3.718663811
6.248024669 -0.47026243 2.335444636 4.641231302 3.490448855
3.363162074
TCGA-78-7633-01A -1.383042139 4.575193572 2.94409475 5.589890695
4.246946296 8.33981382 3.279970072 4.23089665 1.904186868 3.216195219
5.396889325 -2.020427852 2.992503308 4.705315476 4.580195065
5.239824244 3.961738562 4.1174575560.251421035 0.334538219
2.890042461 4.966498688 -1.670321419 2.657857478 4.529117286
3.322475582 5.426769841 4.171906032 4.44712296 3.635081269
4.988938102 1.24901664 -3.896457376 3.547419135 -1.333767269
2.745755264 -3.161702457 5.5988621123.770229863 0.922360795
4.708750425 -1.325411258 5.149015918 3.502337698 3.916927858
4.178494557 7.046991054 1.33983865 3.746412964 6.348868192 -
4.815967738 -1.602175186 4.643041998 -2.921856767 3.249994227 -
4.1911314014.883979201 1.814207444 2.120263129 3.83800988 6.048637507
2.935878307 2.537960114-1.351080287 4.415082755 3.942788668
4.480064981 3.81342491 -2.151201822 5.076146429 6.370498772
2.972130857 -0.145534301 5.842789947 -1.186365962 6.045730157
5.194630762 4.670166873 5.481266712 6.457620794 10.71754695
1.281079457 2.506278917 0.642697475 -0.872000424 2.715002838
0.760434202 4.41283997 6.884918169 3.93386898 4.636587013 -2.268364084
2.179985231 0.247239194 3.361399384 3.770098174 -2.5762985
5.359493666 4.437167497 0.835388621 2.7532878114.289992044
4.967950244 4.672072582 3.507107992 5.335395972 5.324712356

4.7871170354.584156104 0.177627047 3.908300665 4.6112259414.639779432
4.723172746 5.910462232 5.680134214 0.971499837 5.811044299
4.263045817 1.779624717 3.882957945 -1.179650669 -3.599091228
2.735879252 -0.231155577 -3.478155345 -3.906156367 1.765403854
2.689085673 4.440109531 5.163994662 3.457674002 6.070665926 -
0.3231451143.217335765 4.479777919 3.6359078112.439729275
TCGA-78-8640-01A -0.812246302 5.163302144 2.859373786 5.331156604
4.583056267 8.10310459 3.43854198 4.097404032 2.251544222 3.538235295
5.249364988 -2.063853533 3.484689323 4.609956421 4.933932771
5.084658582 4.357626448 4.267328283 -0.008873106 0.434267753
2.941006913 4.892488647 -1.303188878 2.139553424 4.663284899
2.92287407 5.536462209 2.498402792 4.634063129 4.65936388 5.085605123
1.544038728 -3.532234367 2.788819074 -1.046018093 2.989315951 -
3.235910092 5.612641366 2.976589225 1.203568625 4.678888441 -
1.178923462 5.016332774 3.610290606 3.858106479 4.183465421
6.825725309 1.549822 4.366367672 7.187459751 -5.059891119 -
1.619839779 5.042977913 -2.382634037 3.586224318 -4.996601974
5.272676782 1.840741216 2.224047995 3.814845698 5.829255988
2.59161297 1.886383174 -2.739470944 4.585139454 3.927039933 4.10205883
4.321997758 -1.817153274 5.084740452 6.442432565 2.924205263 -
0.0231138755.214680411-1.052668702 6.313967718 4.921541069 4.891682084
5.4491144756.277020584 10.39831281 0.64042119 2.847121801 0.922761508
-0.696266875 3.233969563 0.800305735 4.295729633 6.901105786
3.603087865 5.068508402 -1.92316957 2.747595168 -0.190917146
3.361224444 3.917468146 -2.16170591 5.355585333 3.885472648
1.275496297 2.680319517 3.820052564 5.061904221 4.650716969
3.548151083 5.335501456 4.720325872 4.792218841 4.189671896
0.673784318 4.003665681 4.670732742 4.7811507234.704315072
5.796398058 5.171627037 1.417665707 5.2286541193.881631528
1.612231423 3.867417026 -1.29985221 -3.861710444 2.409221043 -
0.36086102 -3.959356449 -3.884518146 1.475230667 2.660228235 4.364437652
5.6642141133.870373215 6.460892625 -0.368900534 2.705741847
4.447278889 3.695067706 2.683956483

TCGA-78-8648-01A -0.341588069 4.493703097 2.548308861 5.336600019
3.696636018 8.369065741 3.6901351114.316604715 1.744053604 3.82409228
5.223432822 -2.38469587 3.389609906 4.046042184 4.907567937
4.8807711613.691901452 4.048561554 0.241020755 0.509743255
2.800748874 5.401597806 -1.251066661 3.413296729 4.537374921
2.9532133 5.695340001 1.965380658 4.5911079523.102337097 4.956972732
1.206612499 -3.564741626 3.845524437 -1.062023387 2.196877455 -
3.013310459 5.566363725 3.46650068 0.745761778 4.720593063 -1.160445579
5.245766617 2.734268512 4.029283484 4.258807951 6.796263904
1.569299376 3.2231411397.019570576 -4.879901115 -1.701462248
4.173282796 -2.840553469 3.435722148 -4.470132542 5.067531834
2.045943373 2.240042375 4.019918018 6.250441462 2.807028228
2.594021705 -1.551011348 4.856927509 3.925019822 4.146843876
4.21930787 -2.078190621 5.106872761 6.667245073 2.769801541 -
0.055349313 5.980369657 -0.263888444 5.927388385 5.132165306
4.638993576 5.501341657 6.349464471 10.73705249 1.000412843
2.603665815 0.713893796 -0.360699892 3.914794307 0.999987242
4.268719777 6.917788442 3.490515958 4.517145384 -1.946061654
1.880240794 -0.441225575 3.330154553 4.157419184 -2.29528315
5.321059005 5.187242735 1.657009012 3.438165272 3.989324757
4.97404397 4.675570947 2.770740243 5.335783208 5.18310184 5.06085799
4.183244808 0.921242271 3.099986629 4.5645911674.874174336
4.771243637 5.517725474 5.266624451 1.098830073 5.580644083
4.005293757 2.22826861 3.759921105-0.335368842 -3.914555711 3.321735756
-0.732868757 -3.653865255 -3.158436566 1.670315538 2.983271054
4.466065364 4.670977209 2.756687483 5.988501453 -0.055701294
3.035144959 4.190607518 3.708623279 3.100427186
TCGA-78-8655-01A -0.754445866 4.813977961 2.892628814 5.455165561
4.384413407 8.236006384 3.28126519 4.3306811681.826564442 2.847632096
5.454038419 -2.553110987 3.529018272 4.365876393 4.782695849
5.254337921 3.420635179 4.061695608 0.046323224 0.445072276
2.5951495114.979243189 -1.132104684 1.721408639 4.531451849
3.042693364 5.773673898 1.710433654 4.575263566 3.578830156

5.00764143 0.922529613 -3.576200728 3.159294602 -1.049909519 2.79193262
-3.353986742 5.605395712 3.369649932 0.309138348 4.718137912 -
1.090516979 5.265022526 2.740873321 4.041663433 4.256389599 7.05929852
1.342785299 3.707387201 6.677349502 -4.930557722 -2.07506712
4.851175887-2.591080031 3.121687939 -5.074034274 5.1009706111.919198212
2.153515308 3.814627851 6.028846752 2.496619666 1.762660316 -
2.70314203 4.606586451 3.939430096 3.902737234 4.205807988 -1.317252073
5.091021027 6.282979902 2.940102792 -0.126732319 5.912954942 -
0.665443434 6.155224776 5.1161176834.755797599 5.545943781 6.253999073
10.26258787 0.81789651 2.585740904 0.74377376 -0.917965634 2.842299773
1.021543333 4.42566837 6.847257133 3.648269907 4.722977866 -
1.951779922 2.381131938-0.740565728 3.242252577 3.643639291 -2.515600901
5.367076614 4.385269095 0.309464751 2.733935552 4.031829649
5.029858902 4.872829502 3.191029161 5.335595988 4.88777286
4.866774707 3.984293663 0.648724207 2.542348182 4.500785639
4.815564237 4.730869874 5.660185514 5.2401114490.89215274 5.640089056
4.201791271 1.857493122 3.972507398 -0.569796504 -4.266844451
2.455037045 -0.15590405 -3.510414069 -3.78809928 1.680667003 2.969264521
4.613795967 5.318360206 3.5691042 6.029436861 -0.018979154
3.080540074 4.541636787 3.601514904 2.710152507
TCGA-78-8660-01A -0.598868579 5.088375154 2.6114359215.360921724 4.1917081
8.153707698 3.55164232 4.285513168 1.872204706 3.5279301115.447571739
-2.224264955 3.950908451 4.462379691 4.959109007 5.121779005
3.80590935 4.152834032 0.41914796 0.484006822 2.754483476 4.661570652
-1.344988931 2.2811705564.574300042 2.954973916 5.871660239
2.559314561 4.664938654 4.525266628 5.103383078 1.147436514 -
3.314481214 3.169217827 -1.0133497 2.728663731 -3.066267948 5.607604408
3.084003608 0.964041306 4.707811669-0.981032356 4.991418953
2.754193596 3.998672099 4.210462979 6.839643542 1.535401425
3.976763727 7.02080784 -4.784126569 -1.930261069 4.8996478 -2.245485615
3.309664706 -4.914959351 5.240314914 2.162447968 2.052989114
3.790774842 6.000709691 2.677542957 1.999987074 -2.031563175
4.696248659 3.933652653 4.007590172 4.456444319 -2.168696776

5.091346857 6.408687381 2.81777427 -0.041370574 5.771678251 -
0.531438738 6.196075757 4.905196433 4.878997932 5.528503745
6.153284274 10.52840306 0.865849674 2.8358110670.837185374 -
0.571505836 3.683689731 1.024044317 4.396438551 6.887280288
3.4232751174.933777051 -1.856891281 2.791851382 -0.307089518
3.295092298 3.834519444 -2.796845795 5.361698353 4.1631791
0.835881531 2.803879481 3.963684022 5.082555597 4.655363716
3.269084935 5.335805291 4.69945711 4.922212661 4.1107518090.797071062
2.856770106 4.682948254 4.880792031 4.723837987 5.652899717
4.953877109 1.215350947 5.480705774 4.076128827 1.655954194
3.926383349 -1.080775591 -4.063693712 2.359000796 -0.272917716 -
3.619617851 -3.540204462 1.79312801 2.861205186 4.473062977 5.427286247
3.506836467 6.249625887 -0.241274846 2.867481902 4.402298266
3.679826891 3.006443017
TCGA-78-8662-01A -1.281315144 4.994888185 2.443817945 5.34409239
4.527022065 8.170135589 3.2711986344.1186500272.120296737 3.194600324
5.495498708 -1.939822246 3.260078671 4.648409127 4.541928619
4.922167501 4.349657124 4.031456302 -0.368926598 0.431841488
2.920038079 4.91187062 -1.463027576 1.750467905 4.582251531
3.064836344 5.441038103 2.873857768 4.65292568 4.853955015
5.153684417 1.521884152 -3.583021364 2.352815952 -1.058032005
2.781902234 -3.434798906 5.605195302 3.830336473 0.695571488
4.689220158 -1.189631077 5.104831429 3.254897559 3.791664773
4.144218608 6.93549966 1.438595559 4.012220206 7.158580972 -
5.044823284 -1.852435716 4.617106123 -3.226465209 3.787747536 -
4.856884352 5.16256894 1.945651304 2.418138331 3.830275698 5.890582274
2.593357309 1.87460556 -2.032878356 4.498086397 3.933648343
4.269142956 4.078985001 -2.130089002 5.077087262 6.484645134
2.77957532 0.176139242 5.271575084 -1.106718986 6.265534303 4.91847108
5.102361471 5.421569376 6.431222448 9.983505121 1.342228186
2.766902964 0.61884942 -0.628062285 2.4869110710.86561848 4.368287387
6.954174322 3.645167308 5.032010234 -1.920726151 2.616394008 -
0.417220624 3.335702132 3.928638841 -2.619521706 5.360877289 3.90153803

0.763519157 2.586834846 3.912401952 5.047153493 4.51787762
3.735071393 5.335576713 4.989702669 4.897082271 4.255261013
0.609313582 4.001606458 4.836324362 4.744147398 4.676862945
5.996213424 5.264764309 1.220986656 5.622572644 3.830178239
1.356704402 3.910107976 -1.954871095 -4.250411673 2.101826049 -
0.447800132 -3.905473248 -4.148863514 1.426385576 2.55765085 4.347410506
5.439971865 3.378131779 6.270701948 0.800150239 3.073021882
4.55606665 3.628295076 2.329070599
TCGA-80-5608-01A -1.056014518 4.756767599 2.12260476 5.344494951
4.349887609 8.33375567 3.180964567 4.271262055 2.012660151
3.454594792 5.342190086 -2.115343676 3.961776744 4.559344509
4.677941519 5.223948382 3.597045294 3.9800801130.677601955
0.531722615 2.851720532 5.023615151 -1.260600099 2.133721467
4.554635795 2.923783361 5.695998973 3.548626734 4.590018924
3.688491905 4.891263154 1.090812576 -3.707479475 3.406031119-
1.057175065 2.786086367 -3.003459937 5.608468752 3.395305546
0.280312461 4.718676508 -1.085010802 5.142964504 2.960079566
4.045718348 4.373391226 7.236473308 1.590723664 4.012818704
6.804399143 -4.558456178 -1.716641017 4.695331646 -2.134537943
3.322408651 -4.35992514 5.187271334 2.298055492 2.25255291 4.01260936
6.05832822 2.781563391 2.008936356 -1.320781151 4.477054123
3.930943424 4.39936406 4.269992165 -1.620238045 5.072080799
6.531961709 3.040461688 -0.113519553 5.833721646 -0.512264676
6.068535016 5.171036479 4.95916796 5.541681244 6.32276146 10.19311031
1.421589641 2.628615081 0.942231374 -0.786026741 3.19016688 0.72981059
4.374490815 6.861652572 3.76343995 4.834653676 -1.957284864
2.264540064 0.260659104 3.332229968 3.675150469 -2.713042579
5.35662411 4.304701346 1.061298225 3.039225957 3.982752483
4.975138971 4.657219305 3.537374894 5.335398089 5.000396059
4.85228982 4.59064419 0.555356655 3.271397981 4.638703726 4.650635823
4.720471363 5.89228167 5.1143906871.215290427 5.563021984 4.06643181
1.702416494 4.022073141 -0.868878579 -3.366500752 3.01362118 -
0.084926423 -3.203491947 -3.110778389 1.817797288 2.787531204

4.445240849 5.534732636 3.476879912 6.079687395 -0.799766946
2.636132387 4.784176124 3.83255929 2.705214841
TCGA-80-5611-01A -0.815320117 4.633063826 2.072940397 5.308208815
4.510444616 8.175887641 3.1113300174.182657912 1.663777555
3.483107601 4.770439617 -2.267844056 3.422014693 4.916415345
4.769014601 4.8510679114.014230894 3.681256263 -0.269918799
0.285303361 2.733006894 4.932487557 -1.394726631 2.328115731
4.592510268 2.792644438 5.289291259 1.487903437 4.52043098
3.884629956 5.0596211411.131479713 -3.780127452 2.628092383 -
0.947742486 2.725008126 -3.982430922 5.60706414 3.0118762520.710021207
4.69723577 -1.250461429 4.945048947 3.088733362 3.735948566 4.19823786
6.917964285 1.087840969 3.788951214 6.852174372 -5.35607867 -
2.033738373 4.938779764 -2.903267865 3.785038746 -4.899580553
5.026377678 1.355228904 2.921997851 3.857900123 5.905302494
2.329544242 1.921791191-2.619410338 4.534309341 3.933791457
3.888148179 4.066012505 -1.703996942 5.079016697 6.292457106
2.807691802 -0.02291358 5.234338496 -0.844136022 6.1153958584.927264137
4.638214171 5.389663582 6.299009651 10.32433282 0.645040136
2.806962653 0.614346093 -0.780580964 2.884355648 0.88363369
4.221966567 6.828829107 3.344658313 4.99010917 -2.305778159
2.162750197 0.225956396 3.329328977 3.703231902 -2.115979932
5.344454426 4.138583885 0.855614284 2.995770343 3.512686529
4.954160409 4.730533146 3.3917117435.335307768 4.562906231
4.995527148 4.371536045 0.546594637 3.468665394 4.679905878
4.807782004 4.765147166 5.700824366 5.13049934 0.771681482
5.228928705 4.410838158 1.868462633 3.817038903 -1.602966187 -
3.9954851142.699889177 -0.408357546 -4.062115704 -3.794941186 1.42931886
2.534880241 4.341228562 5.417962493 3.4631125636.202351696 -
0.799530398 2.470388463 4.506426688 3.684822929 3.219646681
TCGA-83-5908-01A -0.17108884 4.841533304 1.294624498 5.423181622
4.379325074 8.175795103 3.55322447 4.240560501 1.648985832
3.370271663 5.232511128-2.697944116 3.768961485 4.863488665
4.930001733 5.0406636113.914343588 3.793858599 0.539719723

0.555224208 2.335630396 5.02945848 -1.237310345 2.404165596
4.625710982 2.449791473 5.662370982 0.598247122 4.564023549
4.268392657 4.990027634 1.031431819 -3.526424966 3.176092925 -
0.585679822 2.492912819 -3.582951735 5.6162112663.332170388 0.906571811
4.695742586 -1.178211468 5.370999852 2.920799074 3.827099079
4.28402796 6.860979701 1.456439769 3.6771601156.98335144 -5.020369064 -
2.208536089 5.654043008 -2.166090354 3.126412694 -5.226986436
5.082189169 1.759321417 2.437379863 3.794019725 5.875559541
2.476721758 1.856352542 -2.729267873 4.73427113 3.936185264 3.41424974
4.479460547 -1.264864755 5.078726798 6.1968977112.668419208
0.086095383 5.336164038 -0.347098433 6.088555358 4.945366048
4.797595281 5.381674804 6.281277735 11.17791335-0.005558781 3.07776563
0.939763378 -0.692019852 3.630261862 1.094782263 4.296021742
6.799369421 3.15462574 5.142890197 -1.757517936 2.588283874 -
0.849231281 3.234214707 3.636734794 -2.110582177 5.343998264
4.265401266 0.93136599 3.26268333 3.49407517 4.987242788 4.969512076
2.752832673 5.335429157 4.102037183 5.10639371 3.5368211340.805399831
2.586640061 4.383377734 4.8271147134.77489228 5.413061983 4.610046498
0.646027303 5.171010003 4.265521331 1.966224588 3.864960843 -
0.075212804 -4.476193638 2.392093201 -0.037532084 -3.767886049 -
3.500130717 1.518235152 2.704517645 4.326222 5.269414832 3.772227526
6.106771022 -0.097960536 2.314516314 4.288547265 3.491108611
3.215164555
TCGA-86-6562-01A -0.632663276 4.605346268 1.7911114855.482516993
4.476842398 8.28186323 3.18651075 4.252834774 1.691568319 3.193800141
5.188035703 -2.475839606 3.645329803 4.606718028 4.64643623
5.133416858 3.488760977 3.790865634 0.363636818 0.541644453
2.616862708 4.991122765-1.061751602 2.049315483 4.558125141
3.135628944 5.367482473 1.613161023 4.51278511 3.36248494 4.994449579
0.943417157 -3.803963649 3.343539227 -0.96306211 2.6650034 -3.411783756
5.593965644 3.518482145 0.963266708 4.709092886 -1.266982788
5.1124948872.987013751 3.95226131 4.278877063 7.02106466 1.481753842
3.344554418 6.934591101-4.887137108 -2.083551084 4.926106378 -

2.684058684 3.167559314 -4.588440736 5.020348084 1.731214932
2.538801995 3.89293902 6.036475313 2.690825937 1.877956681 -
2.414920294 4.554880075 3.936764449 4.006859532 4.219693473 -
0.535193574 5.078066086 6.246982574 2.818387496 -0.046372215
5.682996859 -0.544315864 6.023823881 5.144219151 4.649673031
5.466579777 6.382810856 10.75810715 1.209239353 2.626980075
0.549498427 -0.954880764 3.284374488 0.969166422 4.339507585
6.829059021 3.642060799 4.771656802 -2.017296688 2.170771211-
0.324953718 3.277592066 3.534795717 -1.786575816 5.348295262 4.53481392
0.943831275 2.936257828 3.935639421 4.94548313 4.732593734
3.144720314 5.335278699 4.841038206 4.981837599 4.105579854
0.603609579 3.330946364 4.543227185 4.728415438 4.75963692
5.669706507 5.11040541 0.638471991 5.715718881 4.294609505
2.159666244 3.902721142-0.412260277 -3.819786509 2.765595591 -
0.123713077 -3.424172821 -3.513450423 1.556304218 2.820040157
4.407583962 5.07875571 3.34587741 5.952464169 0.1123645942.980149403
4.461527765 3.59029394 2.833738016
TCGA-86-6851-01A -0.125882532 4.914204852 2.260027085 5.219253537
4.399209234 8.172442391 3.553822705 4.171062326 1.754646745
3.189723925 5.105090283 -2.634695689 3.092608501 4.76580225
5.088178772 5.084109121 4.1125719473.680452919 0.290339641
0.491718334 2.699434406 5.110723066-1.120718284 2.380860498
4.6116523832.864097531 5.624009655 1.330708176 4.580066303 3.64149157
4.974678602 0.814501807 -3.492151537 3.253794494 -0.993799899
2.793120641 -3.361559028 5.593055447 4.258551621 1.325490195
4.701605287 -1.128407568 5.19293628 3.262677394 3.973068544
4.241363135 7.022439349 1.783322171 4.178472318 6.806289062 -
4.81201159 -2.022180272 4.850100971 -2.484418035 3.12129317 -5.196605853
5.223166475 1.836810177 2.17116568 4.042242453 5.990065697
2.683745878 1.78897428 -2.500352314 4.744477189 3.936283683
3.633543195 4.412338529 -0.623545565 5.085454757 6.307354296
2.940583872 -0.181100134 5.742066175 -0.216203599 6.090211682
5.094379688 4.805436053 5.462494522 6.238976781 10.42848486

0.406572699	2.89424808	0.783515017	-0.956036924	3.574890918
0.832677582	4.27298335	6.872702001	3.494354456	4.873485801	-
1.6831157822.444053529	-0.666035533	3.213452166	3.568906903	-1.968198019
5.357568093	4.514363051	1.520129798	3.224797964	3.990518068
4.948464019	4.877984522	2.97034734	5.335439858	4.746753843
4.941609591	3.725077029	0.8211438083.673167603	4.428438584	4.8070517
4.764077382	5.61307604	4.973076935	0.715436083	5.59671151	4.231090743
2.076019859	3.977440653	-0.426272257	-4.02038602	2.323698961	-
0.129216199	-3.593011339	-4.221044144	1.474445655	2.979314881
4.304023302	5.545109592	3.696365763	6.275833248	0.318610841
2.992992683	4.452218328	3.700770389	3.046900412
TCGA-86-7701-01A -0.936088609	4.517612092	1.730757195	5.334982766
4.197460504	8.361771484	3.707648794	4.238856732	2.058336104
3.708080548	5.558855053	-2.263460609	3.4867011754.955275558
4.900733074	4.868686593	3.837784124	3.98808943	0.7711895020.595410352
2.625692221	5.402252372	-1.578609431	3.1468188114.668169375
2.414997346	5.824960939	1.5743301124.4170271	3.538833645	4.471854512
1.044855913	-3.808805243	3.742751145-0.772114487	2.20661449	-3.301090371
5.576823494	3.150238085	0.669869737	4.69507034	-1.280061538
5.406095268	3.353192331	3.896534162	4.453134635	6.984199409
1.5536711333.76481761	6.760772032	-5.05356093	-1.688589916	4.663720982	-
2.249123092	3.556764921	-4.431721931	5.018191797	2.042720899	2.35457664
4.2171164826.030371353	2.947046431	2.557367501	-2.486390632
4.596942891	3.947520772	4.088506002	4.414398273	-2.082967363
5.073240283	6.356071357	2.89669853	0.010346829	5.99757512	-0.523321968
5.96774146	5.179557395	4.560854268	5.460739716	6.559703312
10.37639289	0.820708318	2.825455298	1.255109564	-0.839520699
3.619250339	0.863397332	4.186409753	6.807704607	3.597672692
5.030862275	-1.872726328	2.296524988	-0.239250893	3.296004556
3.888161903	-2.441807329	5.341513324	4.840306672	1.493300703
3.523704622	4.035670677	4.916661022	4.687651092	3.033191268
5.335256295	4.952297833	4.975174356	4.187990212	0.468158485
3.407480287	4.500164348	4.527360707	4.777776609	5.695811381

4.785892884 0.955575913 5.571573735 4.153857983 1.752413363
3.789968171 -0.138279733 -3.520625274 3.383445027 -0.262675882 -
3.494513329 -3.211266234 1.363692941 2.51497148 4.231204626 5.410464763
3.477003782 6.069830344 -0.104636363 2.4864117254.507443992
3.666662203 2.856884494
TCGA-86-7711-01A -0.868316805 4.679225483 2.597192933 5.488271098
4.4951281138.307993691 2.971987292 4.247701914 1.64050154 3.952074045
5.619052444 -2.277021547 3.012697321 4.850877974 4.54472079
5.148087475 4.471853831 3.487892991 0.065595386 0.315946336
2.798596416 4.910406412 -1.507745445 3.257921549 4.544168653
2.85371452 5.173767582 2.875729178 4.519689429 4.173683764
5.027541385 1.330131291 -3.919914764 3.14166516 -0.999429038
2.002832901 -3.358286419 5.617664358 3.799445063 0.217048549
4.684267705 -1.388593759 5.282484837 3.429452787 3.816108658
4.173491581 6.895103357 0.786800019 3.151343108 6.716177832 -
5.528400682 -1.72457312 5.385240869 -3.278801894 3.465704823 -4.766592254
4.876289252 1.412452803 2.223197044 3.674201201 6.101965254
2.725771633 2.805027025 -1.988006346 4.509293098 3.935730934
3.92460523 4.169972246 -1.866276618 5.090902339 6.268182968
2.732141739 -0.08853173 5.280390983 -0.969304933 6.129828894 5.08011187
4.592302455 5.415315381 6.351356671 11.600618311.212410921
2.553526158 0.104711647-0.457214609 3.2413931141.190392006 4.246783698
6.780471802 3.68345961 4.827110009-2.332261373 2.372039568 -0.249876841
3.366628985 3.990188211-2.188669765 5.343705731 4.235950495
1.273486376 2.856290285 3.952438894 4.953155362 4.706953265
2.8341168365.335350879 4.900023018 4.999704687 4.3974231180.451065492
3.624544504 4.519941594 4.790823614 4.746068529 5.61422911
5.382580014 0.683839198 5.768169921 4.201920476 1.838910781
3.63766821 -0.661147116 -4.413764993 2.743282252 -0.552381988 -
4.030904312 -3.718360365 1.619506947 2.6261160014.431960072 4.91394798
3.674024256 5.906511371-0.827842543 2.929858479 4.232665447
3.560395763 2.913385389
TCGA-86-7713-01A -1.08570157 4.883371219 2.428382984 5.333259743

4.301413952 8.244784396 3.539681603 4.1861785112.22454533 3.251217563
5.266292988 -2.105922132 2.928029261 5.127963969 4.831800662
4.970100003 4.342592517 4.17864877 0.691019877 0.592837935
2.680286701 5.22648973 -1.781882772 2.10300305 4.691716379 2.766214544
5.845189101 2.253019838 4.451558887 3.962296921 4.786285811
1.265795215 -3.547866892 3.284744156 -0.859186112 2.864606391 -
3.432400538 5.598466585 3.448697555 1.13747842 4.681643675 -1.148399989
5.107857131 3.952656913 3.896243404 4.19400978 6.984195886
1.706350989 4.648349989 6.853362795 -4.822544771 -1.673599858
5.146465495 -1.942312179 3.363291726 -4.964979171 5.257372183
1.687238791 2.2019311763.941278558 5.760091269 2.754002128
2.090459707 -2.356724462 4.604148432 3.938876472 4.047882259
4.337237338 -2.551153632 5.065046687 6.314283601 2.995411334
0.021274762 5.632223353 -0.894181991 6.197774013 5.088705889
5.039879385 5.374655134 6.607125305 10.301180380.121273099
3.019614923 1.019788476 -1.091476459 3.282816075 0.658297531
4.226262739 6.892776468 3.640482205 4.991322814 -1.538794509
2.582448495 -0.156098691 3.260100479 3.777732337 -2.881788311
5.356381983 4.28571828 1.274803726 2.982942644 3.97838557 5.001257674
4.78353569 3.672897878 5.33534971 4.8441362114.775212686 3.907701758
0.505221445 3.805722052 4.507792065 4.614795795 4.71700915
5.554563403 4.999057765 1.034748963 5.515995391 3.713480363
1.307439247 3.915127222 -0.232440245 -3.750044704 2.493459754 -
0.177197474 -3.531815895 -4.44853041 1.245250553 2.3590112683.878625884
5.763775468 3.802155065 6.481576284 -0.342778026 2.835070546
4.602951625 3.745463343 2.494263058
TCGA-86-7714-01A -1.186232683 4.612932406 2.612304474 5.457821592
4.4117190578.316528936 3.421091414 4.329148758 1.7597086 2.954558403
5.078758662 -2.430116094 3.122717939 4.787884098 4.684996616
4.835366259 3.202029682 3.598183447 0.62631492 0.544942496
2.383799483 5.211453837-1.506194351 2.093990244 4.539584272
2.9156711875.60319241 1.685953655 4.427101719 3.023817408 4.743565082
0.777654956 -3.771977726 3.42437327 -0.973718625 2.747638428 -

3.495355187 5.592761331 3.841450204 1.284635124 4.715424343 -
1.261331715 5.168249181 2.813188831 3.949732989 4.276948096
7.014248984 1.312455153 3.572135967 6.323260089 -4.775063531 -
1.912036988 4.694112692-2.428856716 3.215286476 -4.646214671 4.944016472
1.827550352 2.751200215 3.982566761 5.947798515 2.639046293
1.50695393 -2.814602272 4.600236128 3.947037802 3.747865614
4.225318277 -1.719764195 5.083967999 6.249135149 2.937736643 -
0.097099499 6.043151413 -0.640278757 5.980800449 5.203963599
4.427933253 5.440786625 6.334616049 10.67595687 0.647714861
2.768167421 0.791285607 -0.999463073 2.763305077 0.894541964
4.2911967876.846288895 3.614097006 4.587259988 -2.09905836 2.049106298
-0.168812754 3.222622314 3.533375728 -2.565379617 5.348688516
4.920479908 0.659103105 2.899733532 4.235518984 4.889035993
4.928479643 3.052219134 5.335320004 4.851454001 4.856423328
4.126124149 0.334948542 2.645634712 4.268496784 4.622611894
4.790440039 5.555146744 4.953618625 0.775873004 5.639043905
4.605805693 1.970460137 3.800324277 -0.650056677 -4.113618668
2.956607065 0.003320966 -3.243871525 -3.724552127 1.74644263
2.681544223 4.274485261 5.00142744 3.344582253 5.990678334 -
0.1714431192.890143931 4.420457146 3.674851921 2.676468779
TCGA-86-7953-01A -0.650381894 4.606707204 2.553496823 5.384491504
4.381644091 8.231414063 3.49254586 4.314026825 1.936261338
3.536999451 5.444673323 -2.3802181 3.5787241144.820050234 4.863785461
5.02546531 3.918212749 3.860240409 0.017693579 0.476351759
2.784518206 5.211251397-1.279826508 2.993285902 4.589613356
2.659985703 5.75647515 2.238940655 4.530819521 3.448785709
4.876273131 1.20013777 -3.518553848 3.503654523 -0.840988646
2.263305467 -3.862616249 5.600456968 3.932264396 0.491725242
4.69759048 -1.171297317 5.252801245 3.15877668 3.933831327 4.321958241
6.90243234 1.230624019 3.945438395 7.01345129 -5.285521229 -1.818099581
4.935695462 -2.271342413 3.345739114-4.827616044 5.146293477
1.879324499 2.360952715 3.794351981 6.05555011 2.598383453 2.65274836
-2.587373792 4.638328069 3.938255609 3.866327504 4.536419446 -

1.5173112865.079942688 6.314938621 2.8021661140.0116935615.894620405 -
0.516659348 6.137508574 5.085757814 4.699982237 5.533813968
6.32971981110.34252392 0.220997195 2.930377558 1.07564813 -0.761127324
3.525477438 0.917052476 4.234886719 6.7899202113.516620953
5.115832294-1.861563382 2.474772487 -0.243199021 3.312354768
3.737774105 -2.375389107 5.352219162 4.415989602 1.399162895
3.261275991 3.726706729 5.02261631 4.818832321 3.098188895
5.335472002 4.662740874 5.040343421 4.187422451 0.687125379
3.266399172 4.622402237 4.809724932 4.739572944 5.646513843
4.988349186 0.94025207 5.572523447 3.900934752 1.798401781
3.867103811-0.255561995 -3.717282517 3.005870509 -0.259424659 -
3.81783898 -3.78660727 1.472721665 2.738903197 4.51276806 5.485730728
3.630253735 6.161823238 -0.15748414 2.523073928 4.530086552
3.728464514 2.601484442
TCGA-86-7954-01A -0.3380602 4.681221685 2.363062531 5.393077172 4.19980305
8.238238593 3.498645028 4.244641966 1.66037995 3.530298225
5.149533993 -2.417935654 3.249425181 4.753174681 5.005572299
5.0544611924.405340675 3.792053578 0.178339668 0.401223528
2.864408836 5.048571902 -1.312592218 2.875266001 4.559174538
2.831378608 5.755698584 2.48069717 4.530552109 3.850404217
4.924973968 1.165717636 -3.580510433 3.522949492 -1.169556374
2.492677963 -3.546953647 5.595254942 3.607525388 0.500696876
4.702653086 -1.183252155 5.133814513 3.425582832 3.939265595
4.237305214 7.00854929 1.657804817 3.956683654 6.821614559 -
5.060912291 -2.00404955 4.772210193 -2.498589278 3.230689666 -4.822055303
5.0747828 2.1138557062.053903516 3.960120542 6.077724736 2.707128103
2.424900428 -2.2714784 4.69673 3.939203439 3.967622305 4.404942049 -
1.567951879 5.093441251 6.325764633 2.829027874 -0.045407298
5.834643551 -0.450121365 6.059099631 5.157521695 4.706094318
5.485641248 6.349504705 10.65859378 0.655250034 2.767807711
0.969859163 -0.818075791 3.772725687 0.956490082 4.247459704
6.842092107 3.663595583 4.994650898 -1.833484095 2.418740235 -
0.360241539 3.305161597 3.73477302 -2.240748685 5.361287491 4.31309526

1.878181917	3.417659624	3.935359842	4.970667422	4.767502843
3.20201976	5.335493629	4.744328792	5.0110455664.295486329	0.776742315
4.200026992	4.540437369	4.818126652	4.764973824	5.599768495
5.278615413	0.862237076	5.608497744	4.023126483	1.954553801
3.882893967	-0.384764952	-3.692028195	2.940551027	-0.276797853	-
3.674635702	-3.802351583	1.607917193	2.853914677	4.3661146815.445309508
3.624978556	6.202131129-0.098237909	2.831637626	4.5011009563.708659707
3.151020454
TCGA-86-7955-01A -1.258821215	4.936109643	2.140785151	5.442907898
4.684085998	8.095439053	2.993635809	4.018288822	2.242084912
3.41079244	5.048176649	-2.212377121	3.391309835	4.917408269
4.678740927	4.9749381123.854664786	3.907900267	-0.252020514
0.286621234	2.831454373	4.58230662	-1.572173538	1.585678126
4.680004745	2.819972714	5.082777345	3.414797405	4.461667698
4.260091008	4.989532701	1.322690583	-3.945369024	2.698960807	-
1.0811993242.820239651	-3.311737713	5.623023553	3.090716623	0.236926097
4.699576824	-1.205134717	5.240410161	3.242920699	3.861715329
4.084965441	7.286485652	1.224777803	4.3116907756.869207455	-5.1815055
-1.841044501	5.356085203	-2.75478816	3.537897908	-4.913841552
5.222952309	1.877420204	2.892353405	3.68839578	5.635421745
2.417197831	1.953474817	-2.505509789	4.417298549	3.936174787
4.345010278	4.238464797	-2.068832945	5.074934072	6.286185604
3.002919334	0.049033538	4.801760003	-1.406322127	6.281440383
4.961219922	5.001560967	5.470073995	6.425921277	10.72985191
0.514400432	2.668377504	0.800283445	-0.788646018	2.542874464
0.862686173	4.324893541	6.903106889	3.585300841	5.048445621	-
2.243142873	2.440915013	0.323635481	3.321072394	3.758424533	-
2.416793294	5.34954175	3.668624733	0.585031668	2.268970301	3.824671622
5.074893569	4.814660141	3.558442058	5.335376054	4.784002859
4.867612435	4.133291813	0.477647995	3.552169001	4.716309298
4.732061871	4.7113338855.858391786	4.887510241	1.03709	5.151703509
4.47717091	1.428646198	3.973502396	-2.155130033	-3.476771145
2.650812084	0.083061252	-3.967465657	-3.913907121	1.092596318

2.569895948 4.310705071 5.668523843 3.7276111636.469656455 -
1.129510271 2.436978563 4.693195634 3.629858308 2.705539129
TCGA-86-8054-01A -1.34363001 4.8028117041.432423902 5.370871504 4.083652022
8.318290625 4.427417877 4.098873596 2.757809426 4.011389404
5.326705804 -1.358331602 2.877783615 5.321985786 5.039643273
4.064859138 4.25164147 4.853636665 0.859879414 0.391986281
2.750985788 6.028486611-1.946653757 2.666226339 4.833721705
2.002006033 5.777323392 3.123491252 4.562253777 4.862403963
4.58745007 1.667273315 -3.689960366 3.40448616 -0.551428347 2.316867655
-4.407249208 5.583512445 3.399613584 0.252878889 4.682377273 -
1.130837345 5.223688333 4.123505793 3.632812356 4.414136367
7.035624788 1.706134424 4.721550379 7.42790547 -5.267538153 -
1.280972702 4.711380417-2.039090569 4.244603963 -3.939417576 5.146249117
2.174330449 3.07161112 4.400412594 5.585732796 2.89421137 2.329095251
-1.682682066 4.510455253 3.947148674 4.7386937114.289147298 -
2.677361943 5.058735297 6.649533503 2.853705556 0.441455862
5.568171476 -0.893453735 6.14190601 5.036968196 5.126373646
5.279294104 6.945627105 10.04402614 -0.411890098 3.295419973
2.154696481 -0.907733151 2.891226861 0.487018554 4.122285733
6.894987578 3.477344415 5.014603692 -1.946506241 2.765860449
0.45702725 3.396554137 4.305597629 -2.653657533 5.3319801144.9755683
1.731522621 3.430396565 3.438499533 4.987762587 4.666241268
3.880577367 5.335483881 4.709956538 4.9167259114.291585977
0.347278313 4.406968985 4.416728413 4.410299666 4.751789075
5.618038291 4.537800586 1.272471289 4.924500503 3.684469711
1.273193518 3.932292091 -0.759764741 -3.046182724 3.756651889 -
0.648155388 -3.891275594 -3.108818239 0.819363867 2.034103403
3.361422581 5.963655544 3.828676499 6.317892133 -0.487821065
1.861304514 4.490332344 3.750357829 1.711118049
TCGA-86-8055-01A -0.655321951 4.425121881 2.334990892 5.418182898
4.154903875 8.373625181 3.34282591 4.296051365 1.675466142
3.598889249 5.55414637 -2.57024338 3.358180566 4.624356903 4.583795169
5.198055431 3.809583425 3.622429234 0.539394577 0.520922835

2.7217561165.374790183	-1.251985714	2.842151396	4.55696572 2.928157671
5.578807177	1.596121232	4.568133643	3.480639175	4.929752782
1.286709402	-3.789899812	3.774399255	-0.89959089 2.564672828	-
2.994906207	5.599475298	3.809785256	1.23265332 4.712534484	-1.259455982
5.210974568	3.038904445	3.949575025	4.243414781	7.006299849
1.408643386	3.163222259	6.693043411-4.686860132	-1.787970815
5.076164317	-2.683116633	3.262101782	-4.416390242	4.957675758
1.900979445	2.444079247	3.847340743	6.146240671	2.67394101
2.417626992	-1.873709208	4.667317125	3.9428771134.070165334
4.200030502	-1.327293428	5.085899484	6.292932581	2.803097092	-
0.1125626125.764629684	-0.570218175	5.938565058	5.161490939	4.539728841
5.454795271	6.393964471	11.254373771.165701082	2.617944774
0.541624306	-0.621038324	3.167594835	1.046471445	4.258824168
6.81999595 3.62633508 4.752813815	-2.082239775	2.149994378	-0.240815891
3.323253985	3.694542712	-2.125451195	5.337218137	4.671832321
1.262868881	3.157696025	4.1113934714.934223798	4.822619226
2.940606406	5.335363472	4.967499134	5.019067605	4.126395896
0.680226483	2.975568016	4.428970641	4.784327278	4.777859828
5.586799829	5.237752655	0.674508481	5.732206659	3.851780791
2.1208319	3.795944902	-0.150209729	-3.818937389	3.163206764	-
0.12764613 -3.423077136	-3.395286762	1.670012794	2.732994719	4.442834983
4.880209777	3.25262395 6.0557611110.205987757	3.223410136	4.420900638
3.568292557	3.229564913
TCGA-86-8056-01A -0.736059703	4.654399657	3.016541205	5.330583479
4.488549142	8.281864614	3.246218218	4.284309767	1.514709726
2.918386026	5.397525803	-2.539658931	3.250213885	4.698603085
4.718656983	5.09121986 3.859406626	3.225876146	0.624586249
0.519363136	2.638249405	5.222244158	-1.298692715	2.485465418
4.529024907	3.153141514	5.482402704	1.346834839	4.393558975
2.777337139	4.837800014	0.606348187	-3.71841443 3.52510208 -1.222775495
2.692035848	-3.540723236	5.588461873	4.266922648	1.426357603
4.702756846	-1.329615272	4.892669675	3.314026257	4.0082191
4.261386183	6.912001949	1.432891218	3.850657967	6.45629262 -

4.585889259	-1.912661506	4.65367499	-2.478958544	3.1598149	-5.194032541
4.990132458	1.532824533	2.09853142	3.943587739	6.069185893
2.784919715	1.964552108	-3.074069509	4.662030948	3.944148074
3.682553661	4.315540903	-1.279819486	5.086185648	6.128477529
2.972079911-0.313745547	6.018554919	-0.557869129	6.081516593
5.202639506	4.298985471	5.45691831	6.33980258	10.63212845	0.735031298
2.603858106	0.286801049	-1.099188662	3.362377147	0.900443805
4.276806661	6.785897276	3.625976313	4.73232453	-1.915853056
2.016334516	-0.250978837	3.176209798	3.442690057	-2.109314162
5.370470687	4.678281502	1.106816163	2.863382949	4.359528744
4.944617618	4.786229454	2.794675036	5.335346369	5.100770649
4.826521334	3.933983886	0.478333213	3.563842952	4.573253695
4.714612687	4.763530029	5.5733823	5.268137694	0.618985801
5.766098209	4.453956501	2.0209135113.821758788	-0.359017368	-
3.833992778	2.421295372	-0.12272616-3.296134976	-4.246795269	1.655756397
2.894863416	4.43836997	5.252434483	3.461971044	6.157377473
0.267902162	3.27424473	4.327667643	3.601521437	2.970057064
TCGA-86-8073-01A -0.863870567	4.481797899	2.292497261	5.235590022
4.548501373	8.295084219	3.317468479	4.164009195	1.681145506
3.266413414	5.235698844	-2.321700472	3.2744711034.860005745
4.687107787	5.089318639	4.10576073	3.469654464	0.469930354
0.506801066	2.669872021	5.221457898	-1.496434833	2.674816733
4.594860703	3.139126868	5.187862036	1.96051905	4.344527292
3.015340366	4.902647992	0.988873887	-3.824050692	3.114812937-
1.023473509	2.558447292	-3.715314551	5.594729945	3.765770261
2.026296551	4.688801682	-1.484886636	5.097264505	3.468561984
3.815651753	4.184971325	6.923699315	1.410821006	3.532302855
6.924560653	-4.591346453	-1.793998665	4.827709844	-2.833589274
3.564450299	-4.712610109	5.004669901	1.494222239	2.875485945
3.856789439	6.043769456	2.783404619	1.696537113-2.508745392
4.612926926	3.931461298	3.781953578	4.196448329	-1.555111412
5.080067499	6.242669997	2.828585853	0.0391143975.530648395	-
0.847106602	6.062071217	5.094606265	4.559877562	5.341226246

6.446317334 10.77061571 0.953407703 2.731316755 0.193165971 -1.11209088 3.37027647 0.82930302 4.235658828 6.856332007 3.564754562
4.888707991 -1.930019377 1.917254873 0.064364697 3.26449308
3.766108866 -2.117297264 5.335175304 4.592052518 1.340558988
2.961337764 3.986612026 4.88036968 4.68070493 3.270707533 5.335118796
4.77716612 4.939823793 4.106791338 0.431245013 3.979315227
4.576941938 4.706544223 4.766334319 5.743667536 5.365041674
0.809537474 5.622554045 4.334649871 1.99128468 3.743281682 -0.734900877 -3.656811512 2.953462002 -0.349601382 -3.472489639 -3.844499838 1.5704481132.5118160254.2742761155.080992888 3.349898946
6.094782001 -0.053798849 2.97871751 4.284979174 3.626222584
3.114582858
TCGA-86-8074-01A -0.561889006 4.458886607 1.679418272 5.397214429
4.395799796 8.27685077 3.482907854 4.24839171 1.716606953 3.457281065
5.012046168 -2.49896966 3.496213089 4.802258859 4.813336714
4.932898093 4.131217158 3.888520973 0.488259086 0.560391412
2.64699953 5.454006535 -1.294705655 2.442606362 4.607413441
2.762225379 5.275538251 1.123553545 4.490270217 3.54846125
4.970667276 1.320150398 -3.711041243 3.467270457 -0.730732237
2.762115475-3.512994899 5.60898455 3.65205817 1.683282954 4.698966727 -1.355581361 5.256489954 3.25867935 3.851851757 4.201416952 6.783051144
1.51344641 3.551571533 6.871808481 -4.870162464 -1.953967625 5.00140196
-2.371446672 3.481697198 -4.713800988 5.007604304 1.827001465
2.859876409 3.963055478 5.93479943 2.559547243 2.087170899 -2.339130499 4.6829886 3.935770702 3.81472099 4.299500941 -1.353391569
5.073531387 6.310004371 2.775052545 0.054036837 5.362630266 -0.628665805 6.013299267 5.059128907 4.567253699 5.433029385
6.370521709 10.78467622 0.557546563 2.910627994 1.025144725 -0.752872903 3.2313141110.896638165 4.266364398 6.819823325 3.508118825
4.996748672 -1.831783591 2.178497958 -0.149186931 3.311987835
3.658310991 -1.931846617 5.338512922 4.451822705 1.270221805
3.443422658 3.7311351344.9112731184.92648836 3.178785297 5.335219639
4.560453454 5.013023705 4.151325775 0.763965206 3.380213059

4.41090154 4.759248808 4.759220929 5.502447931 5.022579417
0.703779995 5.449875156 4.046919504 2.049727465 3.784405064 -
0.051602266 -3.798264826 2.90920614 -0.13732703 -3.58988692 -3.542170548
1.459784517 2.60773994 4.291845156 5.139247137 3.471902663
6.176064223 0.459288555 2.671890396 4.333988248 3.593595596
3.381740217
TCGA-86-8075-01A -0.571149753 4.3879110831.969521275 5.332373888
4.254333059 8.338367081 3.299710174 4.278741761 1.722987415
3.568350449 5.330058046 -2.470694235 3.285466652 4.832150638
4.760261425 5.177931608 4.216577523 3.670001983 0.575309812
0.472669991 2.784726278 5.470988799 -1.32430359 2.697204654
4.571883263 2.889659806 5.533624033 2.168203728 4.517245869
3.1180100125.041232023 1.39133159 -3.778693943 3.760650573 -0.832608144
2.562407517 -3.222890202 5.603376143 3.916795734 1.498241982
4.705392615 -1.283903668 5.123583294 3.301264815 3.924423343
4.213608077 6.974886504 1.492078522 3.437082126 6.839793816 -
4.739617758 -1.727055825 5.153779598 -2.650037681 3.289853166 -
4.372881561 5.053078359 1.815580904 2.610317924 3.922491565
6.096059547 2.49414524 2.239326849 -1.476387176 4.680223521
3.938293541 4.0118039544.161752872 -1.430403356 5.075027609
6.344398884 2.802617744 0.027433307 5.640234294 -0.547112479
5.967682108 5.1198087164.683575491 5.423759981 6.406672528
10.87347522 1.031853707 2.83825945 0.628126531 -0.61921279 3.248165949
0.957452402 4.2311485186.833262861 3.5141132414.927276763 -1.947217921
2.088809633 -0.18901507 3.323558629 3.72071572 -2.088355725 5.32704406
4.556892865 1.553058261 3.452898943 3.8576245114.898321522
4.837350369 3.199630004 5.335237202 4.790048608 5.096518054
4.0411283030.693768947 3.199125006 4.47647666 4.807986635 4.771145181
5.574974636 5.24758832 0.651532596 5.635315349 3.989933375
2.063885672 3.862839881 -0.015647485 -3.590103222 3.124514041 -
0.126591011-3.471953808 -3.59200182 1.579047556 2.692622327 4.349197146
5.078763792 3.258101375 6.13168406 0.184310419 3.212415707 4.43383575
3.6401162693.607510131

TCGA-86-8076-01A -0.536553757 4.919991666 3.239262553 5.44078188 4.06466987
8.242675216 3.460001798 4.328498807 1.954754063 3.288974372
5.289585788 -2.272736721 3.2411337294.294277459 4.810154595
4.652476465 3.491660883 4.1126438450.56890105 0.469258799 2.502668583
5.263333853 -1.424462243 2.128373281 4.577351643 2.901484683
5.883761625 2.163089153 4.429482696 3.255786317 4.700014849
0.780977462 -3.699770161 3.369936636 -1.164088045 2.505531026 -
3.318325802 5.575746232 3.042595324 0.392082268 4.701839611-1.173105152
5.1139785052.922505139 4.043205074 4.339194447 6.833973476
1.380578038 3.749364252 6.744686606 -5.235897495 -1.806050641
4.543334104 -2.403049234 3.387737873 -5.119244898 5.041299249
1.892969153 2.066055071 4.055188762 5.97829096 2.6472111471.907739105
-2.643253002 4.676440475 3.93450596 3.881015016 4.335166702 -
2.071829571 5.094261069 6.375361013 2.946739677 -0.153080192
6.181939039 -0.51090507 6.083797321 5.1568799 4.543627387 5.480669009
6.394228133 10.20525877 0.577802944 2.534534839 0.841240082 -
0.7522485 3.270860231 0.875019819 4.275834029 6.881719879 3.593149994
4.669841044 -1.96739135 2.090736002 -0.616824782 3.236072698
3.905352723 -2.497091439 5.350884672 4.820218183 0.940964054
3.002610371 4.073323023 4.978248564 4.741502682 2.920594505
5.335522601 4.971050137 4.758463992 4.022330888 0.477435792
2.635432967 4.53492274 4.677198235 4.759836907 5.515747563
4.864512593 1.01825209 5.536961784 4.25661811 1.846288358 3.808115563-
0.416487655 -4.474957136 2.752662308 -0.594704454 -3.646463141 -
3.9397611771.409254388 2.794057207 4.301738685 5.233145531 3.273518647
6.012890875 -0.457584651 2.958859438 4.3511975253.703141845 2.46279113
TCGA-86-8278-01A -0.872166715 4.492519626 2.514491669 5.34467345
4.435457397 8.220536987 3.453913903 4.237769734 1.77060612
3.127067177 5.085421369 -2.491044081 3.4045110114.321863834
4.582673067 5.275928727 3.730240013 4.030187183 0.553572729
0.427036696 2.647085942 5.019230104 -1.228258403 2.227445713
4.546494674 3.00133191 5.515723549 3.005680238 4.588039826 3.48425445
5.051250165 0.954479984 -3.773294672 3.542970543 -0.904408045

3.469083933 -2.805672087 5.603009462 3.8823111931.275770753
4.710261759 -1.228804818 5.430776015 2.914910182 4.07046937 4.1829081
7.026229942 1.820076204 3.875467127 6.614793886 -4.639472322 -
1.888270106 4.884049543 -2.415667394 3.142840611-4.860454543 5.014682635
2.2220112992.529313135 3.971618191 6.070277572 2.576677607 1.81176902
-2.422119708 4.667043487 3.937561272 4.033484609 4.202574652 -
1.40164647 5.080071095 6.378741633 2.950360831 -0.181107697 5.756845813
-0.612653004 6.008479683 5.155908898 4.694388817 5.487396388
6.23007294 10.71075502 0.360764956 2.578998742 0.921748162 -
0.757021721 3.125784689 0.873694613 4.331469327 6.842583966
3.694126413 4.713136582 -2.020336088 2.283235283 -0.332179588
3.279248461 3.53054484 -2.300036823 5.354132915 4.638390104
0.859869024 3.007841273 3.8610511974.95786253 4.968929731 3.164144393
5.335431767 4.866907621 4.955095249 3.902361684 0.677908713
3.170695253 4.457108778 4.810371551 4.736697957 5.719901485
5.339493308 0.7521151855.599599058 3.994334631 1.936228024
3.979872013 -0.442919375 -4.010969211 2.673212663 0.058288981 -
3.427023536 -3.64630902 1.74470624 3.014387773 4.460285756 5.199421806
3.620584492 6.230299284 -0.09949417 3.153234378 4.439666067 3.61584625
3.085050894
TCGA-86-8279-01A -0.545054679 4.691988826 2.1121006625.36460351 4.363172949
8.137707608 3.451095423 4.121566095 1.73624987 3.323434044
5.154832613 -2.37525915 3.31609017 4.670082009 4.6811153234.959856204
4.191445343 4.066312467 -0.06689832 0.31915529 2.851492882 5.292516593
-1.272156991 1.990677049 4.578633478 2.849155407 5.2914151
2.544537289 4.581363531 4.0115684955.175567439 1.316579996 -
3.6833411473.002872532 -1.052141298 2.963380263 -3.307456777 5.598657011
3.971228248 0.669270005 4.697889477 -1.230585021 4.907652465
3.458928738 3.898344137 4.090907163 6.935278579 1.748484054
4.088317008 6.987505857 -5.040299761 -1.858058156 4.762509148 -
2.614690762 3.462499974 -4.786711552 5.196844338 2.312498072
2.463928012 3.897481413 5.937934527 2.4833441142.079428048 -
2.1164408364.668071562 3.930599555 4.1126404464.295674514 -1.613141552

5.073165951	6.418581889	2.838003783	0.097862843	5.415087149	-
0.700521385	6.143726493	5.041305946	4.942286092	5.466153131
6.363350032	10.72823785	0.351250254	2.7324831140.94565308	-0.676015351
3.062931631	0.708156993	4.304038088	6.908994921	3.498673828
4.911085402-1.865200332	2.286773682	-0.329674096	3.3064117413.67764677	-
2.143251432	5.353379052	4.139236735	1.427740808	2.847674383
3.534406161	5.012440851	4.813660604	3.530302653	5.335401444
4.593676073	4.9352173	4.072342395	0.863257102	3.895302567
4.520478876	4.870664989	4.705259635	5.584697306	5.010716313
0.904880104	5.3914211973.705533281	1.794778701	3.938185832	-
0.768072634	-3.846950768	2.7064894	-0.109591826	-3.747029978	-3.851685575
1.421414555	2.741487097	4.222351734	5.343548801	3.47199646
6.424291241	-0.032513901	2.818734716	4.412230862	3.658229624
2.643606889
TCGA-86-8280-01A	0.143062376	4.903297983	2.956396315	5.344667375
4.181862594	8.201720131	3.660569416	4.239031812	1.91408561
3.350343984	5.031836636	-2.350398026	3.635439505	4.498964195
5.100812495	5.029414709	3.7911637184.129134667	0.345739618
0.592600868	2.840727859	5.265101962	-0.985907456	2.445895858
4.57386659	3.178785843	5.724257309	2.618665156	4.610198688
3.456379365	4.946359648	1.138277729	-3.433049133	3.463320263	-
1.17129405	2.969204958	-2.976056555	5.58052799	3.122481798	1.39829133
4.72091143	-1.02336564 5.133605567	3.171773587	4.020329102	4.272784551
7.064465558	1.944139587	3.999027495	6.994456373	-4.658203637	-
1.801629156	4.538665459	-2.469122303	3.342032395	-4.588740443
5.200569352	2.240649051	2.47467715	4.021906926	6.077860329
2.709713649	1.828245863	-1.781733733	4.790101402	3.935744487
4.218282046	4.318728536	-1.142131676	5.090926445	6.439408115
2.919016894	-0.043571303	5.853519181	-0.142248769	6.033343267
5.1115759124.716623802	5.472224597	6.340407041	10.55919239
0.747324659	2.793305091	0.901145471-0.673499553	3.744246139
0.993884754	4.287202401	6.962263035	3.586274846	4.836267458	-
1.733615645	2.276603476	-0.253635961	3.27783205	3.884566263	-1.849220741

5.352660563 4.69767697 1.548843991 3.146998974 4.05214449 5.020814061
4.736446037 3.101144771 5.335606014 4.953832684 4.983751631
4.199074228 0.966224311 3.597584598 4.636337532 4.899770301
4.778569774 5.562953539 5.215712563 1.11117325 5.538187957
4.293957903 2.201324186 3.944623095 -0.40034895 -3.466401284
3.119528734 -0.082903448 -3.472427298 -3.605962369 1.651554362
3.073637203 4.49185961 5.402904384 3.237002837 6.355907513
0.276478172 3.080874804 4.53683092 3.747724705 3.359431671
TCGA-86-8358-01A -1.094460416 5.160650128 -1.013083334 4.519283116
4.351493399 8.194243769 5.130511741 3.912605083 3.479071071
5.163834504 4.305444508 -0.966021223 2.932520099 5.966040315
5.461853078 3.728078607 4.880261199 5.457172854 1.045709294
0.115399648 3.340113408 6.451601004 -1.824052524 3.070807356 4.92794183
1.44921506 5.641235058 5.201938747 4.887569003 5.675919041
4.921242995 2.260937592 -3.133146407 3.116612292 -0.346718857
1.911511804 -4.851377673 5.583204865 3.899308189 0.872133616
4.681722819 -0.834668754 5.454075101 4.428783065 3.468965514
4.494884108 7.142955558 2.251151251 5.49776013 7.524774092 -4.736471995
-0.499665644 4.580209694 -1.628032576 5.57993209 -3.516148099
5.460774201 2.807270271 3.755999294 4.651559382 5.628118451
3.022650655 1.693164548 -0.423063849 4.531989468 3.934365202
4.725689599 4.494090264 -2.788835081 5.046173235 6.94500996
2.969027566 0.652273553 4.988774299 -0.158575741 6.04125985
4.891168356 5.515799654 5.045200039 6.958159027 9.75566612 -0.024397105
3.753511187 2.869557484 -0.486812538 3.65727113 0.113422698 3.821757235
6.967907375 3.163472931 5.252489159 -1.892421417 2.984122368
1.609721675 3.535492613 4.690480752 -2.319863094 5.309924823
5.054131654 2.985159356 4.012959159 2.544760174 5.032786356
4.604161054 4.650134674 5.33569892 4.245423549 5.29336181 4.507749052
0.575879412 5.763390153 4.422906155 4.618483692 4.755586838
5.633534352 4.187719685 1.686577676 4.289877368 3.90049002
1.415180979 4.196073621 -1.65304569 -2.293658285 4.779021006 -
0.221782906 -4.007072162 -2.674079659 0.59126325 1.878469259 2.56742848

6.60052229 4.091449014 6.768856273 0.855261069 1.220385073
4.872368814 4.053615066 1.817590102
TCGA-86-8359-01A -0.68281332 4.86656637 2.708611066 5.477347885 4.256485842
8.32900878 3.287499231 4.32802111 1.942683873 3.289874749 5.631218984
-2.421688599 3.99220739 4.036661045 4.638208262 5.221177531 3.71595703
4.071796196 1.143241023 0.630424917 2.694312481 4.991406436 -
1.121303514 2.23389749 4.53827404 2.975438517 5.653042822 2.79023662
4.568433278 3.83832978 4.878385725 0.988963803 -3.81503337 3.748190091
-0.998703629 2.865483148 -2.480804036 5.602993319 3.408043519
0.52702892 4.700788587 -1.193656212 5.272786946 2.854964298 4.1477376
4.310741524 6.956239708 1.674981733 3.507052511 6.877351831 -
4.72190839 -1.978353838 4.674231625 -2.065224774 3.020006435 -4.983421526
5.166865129 2.322660667 1.966616305 3.868740142 6.098043633
2.815619887 2.203794237 -2.142822509 4.585504368 3.926934265
4.188092067 4.558870767 -1.296451962 5.085773095 6.385833412
2.942351415 -0.196977519 5.882489782 -0.703476298 6.168340102
5.176081718 4.746649268 5.581544946 6.29453486 10.60896023
1.081597421 2.355861948 0.898008038 -0.687849038 3.540353424
0.970554707 4.406093687 6.834544862 3.820083023 4.791220382 -
1.750308244 2.410597419 -0.46660729 3.282045294 3.741871389 -2.342791669
5.357669651 4.373903297 0.899491728 3.047698987 4.177585409
5.000378778 4.73155638 3.019792513 5.335421902 4.920138222
4.783258696 4.276033414 0.600954733 2.901165977 4.555630233 4.73412411
4.701491031 5.685440363 5.11855861 0.990402536 5.806797076
3.949017435 1.838620008 3.902602899 0.085259862 -3.956310311
2.474134234 -0.387238043 -3.33386566 -3.41931711 1.702091856 2.963128404
4.649909166 5.143218784 3.536711167 6.03291416 -0.455685321 3.089668726
4.425337513 3.618038163 2.573291007
TCGA-86-8585-01A -0.725685864 4.895493382 2.467798543 5.215911152
4.171784232 8.187126846 3.424774164 4.111055991 2.057658904 3.47078549
5.041786286 -2.371022471 3.652187453 4.333366666 4.675172971
4.721908461 3.843651501 4.200043506 0.176632698 0.43902953
2.778075123 5.01780476 -1.312785514 1.481197891 4.621966286 2.902225968

5.485107668 2.06603919 4.550978239 3.924275173 5.007143493
1.099179957 -3.815961679 2.373687281 -1.247505634 3.135259677 -
3.143866478 5.597089 2.844097086 0.578892337 4.702088713 -1.118630423
5.0825814 2.908385293 3.959269528 4.180148407 6.914515691
1.496706753 3.856894521 7.173366236 -4.998281732 -1.790802532
4.829932154 -2.699126999 3.656479357 -5.093554418 5.240329283
2.078218815 2.464955388 3.962087508 5.866012499 2.574935675
1.132818816 -2.048362382 4.588634653 3.91633312 4.155705803
4.260034706 -2.035189104 5.088640461 6.454522329 2.965905522
0.083075705 5.219516445 -0.715781736 6.160869823 4.99927537
4.881323466 5.395524231 6.212892187 10.25133297 1.292010827
2.479061076 0.76467014 -0.560696842 3.3862567 0.808310607 4.294446406
6.905999408 3.482944296 4.751406559 -1.926850737 2.15218761 -
0.349385484 3.278735524 4.007405359 -2.729193902 5.3445739 4.263061143
0.871414671 2.7113922543.86241179 4.990895586 4.649439025 3.380692783
5.335400661 4.787656906 4.844941486 4.216526396 0.764268112
3.166037469 4.646813422 4.774046777 4.7276115245.670827417
4.8198113391.176474128 5.227099233 4.032127044 1.622935419
3.907877561 -1.263838211 -3.998714122 2.710758272 -0.611596345 -
3.846617527 -3.62285036 1.340170108 2.586222254 4.216163971 5.357917621
3.457691266 6.102829249 -0.681423992 2.678878076 4.575880889
3.666679526 3.034248973
TCGA-86-8668-01A -0.420078946 4.656857344 2.691338085 5.211389034
4.137832718 8.337049656 3.468273304 4.267107282 1.94773241
3.443672389 5.353632729 -2.295894593 3.009270319 4.60667651
4.896399987 4.950015544 3.862918146 3.88770663 0.649938839
0.4860501182.842122415 5.508962673 -1.225396405 2.531955845
4.602010689 3.230107303 5.707955729 2.69854235 4.630488204
3.353333672 5.007154955 1.269372638 -3.466177404 3.629495916 -
0.999094827 3.048453894 -2.90016507 5.578323469 4.192652327 1.817620172
4.722096403 -1.098986974 5.245068962 3.099639058 4.029655217
4.215502124 6.99124741 1.700408726 3.852027803 6.572102206 -
4.301947009 -1.49613416 4.585103806 -2.630472956 3.53014496 -4.234157938

5.1352311572.373080289 2.5292069 4.137992161 6.105525342 2.742822818
1.932899686 -1.318211017 4.744246208 3.942499327 4.316488641
4.120226482 -1.467422701 5.09352663 6.48252291 2.994875971 -0.050851805
5.922754815 -0.290231966 5.958915982 5.149638857 4.675770827
5.420601255 6.360763699 10.6486264 0.950727581 2.762165226 0.80324375
-0.51030078 3.05614003 0.866288044 4.24249653 6.947791633 3.622887418
4.594747224 -1.958963805 2.17019925 0.1190427653.307281078 3.828272174
-2.253539182 5.3451655114.935241839 1.372945881 3.249988729 4.18643871
4.976329794 4.812635529 3.342293376 5.335645195 5.190346355
4.97303989 4.075615859 0.715206106 3.413260545 4.412063425
4.8377041164.770863669 5.606762849 5.261258758 0.944535341
5.668406819 4.152429038 2.209367591 3.935801564 -0.539159738 -
3.376084314 3.270157786 -0.058910835 -3.34049392 -3.645955418 1.53108857
2.996525247 4.339521247 5.140237807 3.195662651 6.298298794
0.648924985 3.48129683 4.461300433 3.742778127 3.304495717
TCGA-86-8669-01A -0.741139286 4.747523527 3.140740881 5.35674307
4.331421773 8.352984871 3.636890526 4.232455564 2.014706833
3.412046954 5.751916769 -2.140958016 3.319723869 4.486195806
4.848348363 5.355960485 4.188018862 4.268348575 0.745300052
0.521698207 2.980868462 5.087360983 -1.311847055 3.023960447
4.602159771 2.947541974 5.723319452 3.317225083 4.595382238
4.127347225 4.833945028 1.197156998 -3.441785396 3.647768785 -
1.108728805 2.942233319 -3.131787878 5.600598456 4.2771672 0.980697019
4.699161446 -1.230880805 5.262202621 3.708601029 4.025328005
4.238214778 7.00686221 1.679689219 4.392084445 6.688381935 -
4.568930915 -1.587704877 4.505857447 -2.201847254 3.230599564 -
4.549870826 5.084998363 2.191634997 1.730027625 3.976798404
6.1161754093.073372836 2.791887154 -1.651081025 4.623535453
3.940705991 4.386142933 4.293327497 -1.642806124 5.088684085
6.501675351 3.034833617 -0.228563092 6.085308935 -0.566925007 6.12931
5.212754604 4.723667216 5.549319712 6.394844828 10.19965345
1.451956643 2.8552611641.083779692 -0.901843708 3.363300944
0.674496663 4.376946149 6.861426044 4.010707654 4.764053716 -

1.830710369	2.5647992	-0.225210318	3.335895628	3.762505128	-2.385717205
5.366865825	4.349235134	1.447384761	3.034503309	4.327604696
5.002222524	4.707535557	3.446656835	5.335532944	5.569028102
4.7314553114.457121807	0.6118196144.184234138	4.522193099	4.63924099
4.719555972	5.97610692	5.488959599	1.1114388675.772549435	3.52286901
1.823458402	3.886814937	-0.460185489	-3.598260355	2.619043025	-
0.359314237	-3.378405752	-3.637944761	1.726641386	2.935278203
4.456947605	5.522882191	3.652423915	6.359206557	0.031693184
3.010734004	4.569231608	3.829540982	2.710312562
TCGA-86-8671-01A 0.499466804	4.934781797	2.9018883115.166140722
3.951502762	8.249920499	3.834745561	4.318994475	1.867349268
3.43957702	5.089420933	-2.44363539 3.509672934	4.249491661	5.106248418
4.92180991	3.619218908	4.1157828790.23270371	0.578565514	2.799280682
5.423587151	-0.96981133 2.914651502	4.561582433	3.173285838	5.92245294
1.694464167	4.662078451	3.218448949	5.005285158	0.905130892	-
3.191793447	3.395716137	-1.151939988	2.72639178	-3.075008671	5.566990263
3.297327781	1.093029323	4.722795388	-0.962605636	5.227379356
2.715098865	4.080633459	4.314263902	6.924628255	1.724510104
3.867396748	7.062276524	-4.663191465	-1.730864738	4.281295148	-
2.493930726	3.540584421	-4.762003182	5.255251498	2.303628964
2.1146470224.087132918	6.2100022112.699707133	1.951889263	-1.821046332
4.9157115113.926505217	4.005868525	4.335232841	-1.598131393
5.103987362	6.555699872	2.915234556	-0.080768104	6.08469868
0.198579503	5.987827442	5.087097153	4.75075245	5.501836564
6.239095561	10.50124762	0.677322399	2.846003238	0.811037361-
0.505860162	4.005583987	1.022180832	4.23531522	6.933020703	3.455708694
4.598809998	-1.729191631	2.155636535	-0.482600869	3.241305225
4.017690011-2.098601812	5.340537124	5.042220557	1.5776871163.360427976
3.951440248	5.02892029	4.737510986	3.007235454	5.3358187	5.046735013
5.062982653	4.190302366	1.06763924	3.162296486	4.657514989
4.989926441	4.780832632	5.556530602	5.162147669	1.096102374
5.561410773	4.322710644	2.273832461	3.91581428	-0.311406361	-
3.7686551123.139390685	-0.351427301	-3.563360287	-3.535387418	1.729649112

3.181609723 4.586499692 5.204515224 3.041492844 6.305692924
0.198634939 3.086020052 4.463937965 3.872077241 3.322896161
TCGA-86-8672-01A -0.850251939 4.916862331 3.131559809 5.525368727
3.899897102 8.18396476 3.393739125 4.3001160321.672221475 3.265115399
5.55171075 -2.376003237 3.914450416 4.191013246 4.689583474
5.084476423 3.839025674 3.994860143 0.16924706 0.351448877 2.54295956
4.658667689 -1.5588007 2.089930407 4.531095924 2.798813297
5.653138478 2.91247635 4.481825337 3.732193834 4.970956004
1.067668286 -3.861399941 3.389731358 -1.260163545 2.099160452 -
2.986788627 5.614990178 2.726617492 -0.024327272 4.703465792 -
1.2110828134.65859831 2.637465937 4.0086011724.1925811196.708453097
1.291991412 3.520228432 6.990913204 -5.318357567 -1.973778201
4.983689103 -2.494829892 3.103944202 -5.24267084 5.106871952
2.068757368 1.992521032 3.601262779 5.987477853 2.512572518
2.059908479 -2.174800145 4.664745289 3.929350595 3.85402416
4.389220886 -2.615846108 5.087456931 6.34898289 2.781230359 -
0.178400715 5.759616293 -0.981822794 6.232352587 4.966985791 4.76874556
5.550364031 6.080798442 10.58581439 0.927362649 2.459761771
0.451993356 -0.506070877 3.272131902 1.023982878 4.41736142
6.857897574 3.523825667 4.870919207 -2.069168735 2.352282078 -
0.865096407 3.270795528 3.917138254 -2.931310907 5.3497001113.899913362
0.815445634 2.688371928 3.782544735 5.025729413 4.724983662
2.698999727 5.335604726 4.546341504 4.853831249 4.065454158
0.710788197 2.305376758 4.56108696 4.830467478 4.699737178 5.54411093
4.964707728 1.208808753 5.384898835 4.076778909 1.482323935
3.810803584 -1.0839602 -4.724776044 2.061599897 -0.546387914 -
3.817475323 -3.615739132 1.692569805 2.774485663 4.491308031
5.021881681 3.301633924 6.002121842 -1.117754027 2.886315849
4.274313161 3.541330881 2.581956148
TCGA-86-8673-01A 0.032031216 5.052324137 2.903127653 5.39480253
4.029715023 8.200341975 3.504865103 4.270516757 2.175736148
3.453229422 5.518256324 -2.147340363 3.860760668 4.217744222
5.012129987 5.132630557 3.849271797 4.622920588 0.038385889

0.452266355	3.001903856	5.016118884-0.705600768	2.176858847	4.62477332
2.90956207 5.556782889	4.004208437	4.736434964	4.351229256
4.991833234	1.568520648	-3.562676172	3.297141533	-0.828463756
2.717755949	-2.733567744	5.600030143	2.809012259	0.251047582
4.709259565	-1.102075729	5.242783185	2.909108097	4.011105018
4.2116300826.926298836	1.561817577	4.162715275	7.297505003	-
4.977729475	-1.698867101	4.86116809 -2.522705621	3.425123675	-4.554650652
5.282498064	2.33847751 2.518109124	3.869800342	6.021612578
2.745972651	2.385050705	-1.483097552	4.610428044	3.9264373114.74527317
4.430342816	-0.130585823	5.088160886	6.510106896	2.85228189 -
0.0119013145.515139516	-0.59507772 6.232851539	4.99205236 5.060656295
5.551890015	6.413723207	10.10326822	0.953394642	2.592357005
1.129067128	-0.285064953	3.247983267	0.948540274	4.444402584
6.9431180393.572970185	5.045814895	-1.919574998	2.701137323-0.249811639
3.402637765	4.017169465	-1.641857405	5.349008686	4.109865219
0.623429773	2.98584559 3.725927288	5.1102676374.691440236	3.433641257
5.335696413	4.863813038	4.90278041 4.425221694	0.974437951
3.081610558	4.714824884	4.944734799	4.697101207	5.748760507
5.095944072	1.222682779	5.491429086	3.662680852	2.025108686
4.041309482	-0.834448595	-3.238815898	2.92788159 -0.336848702	-
3.889860342	-3.296756734	1.397708043	3.025360156	4.513959909
5.633002801	3.2731194956.300989291	-0.313794383	3.046664548
4.508836515	3.588186734	2.461960005
TCGA-86-8674-01A -1.426094592	4.812289444	3.104713667	5.473848292
4.287108952	8.196952243	3.196646134	4.2511435282.013200503
2.873016681	5.280591757	-2.150847174	3.629641306	4.240028435
4.429955631	5.242002602	3.9209113964.460931561	0.135170366
0.280881755	2.774555046	4.649180651	-1.538460428	1.800715465
4.547466987	3.109675851	5.549870372	4.0597631134.47720154 3.71111519
5.035244406	1.106630528	-4.017142731	3.299113355-1.366861282
2.787860764	-2.873540771	5.613858105	2.851547149	0.067031445
4.699097703	-1.262961673	4.874247689	3.124738468	4.022511021
4.195092483	6.952881965	1.463303621	3.994924272	6.677156596	-

5.074655527	-1.838234396	4.802149486	-2.446230385	3.184598849	-
5.177658339	5.1130996231.920410353	1.887091457	3.733321628	5.921638671
2.481265427	2.108190448	-2.45630575 4.436621348	3.928473538
4.348560439	4.148537209	-2.607682265	5.069034475	6.482102713
3.047832194	-0.216036309	5.65984283 -1.434345351	6.253474015	5.07270257
4.889293805	5.58261292 6.284607258	10.0225731 1.037269157	2.338000834
0.728739069	-0.872227867	2.701444421	0.764896861	4.469293759
6.886372425	3.822103179	4.800813113-2.129573909	2.281984999	-
0.383083571	3.323491343	3.798694914	-2.807018246	5.369066141
3.859483377	0.495565771	2.5051179183.768446899	5.055527312	4.66372239
3.568378015	5.335398336	4.906498698	4.751052787	4.447558964
0.43637955 3.326445159	4.738476171	4.72653008 4.650587121	5.968944916
5.353429573	1.270455002	5.609480556	4.060357914	1.300108263
3.980398097	-1.442422793	-4.303986936	2.311467047-0.295119331	-
3.586239253	-4.18650872 1.700317652	2.752564264	4.457499839	5.424615281
3.54208384 6.220839862	-1.339711725	2.876514869	4.548449002
3.615441838	2.15674831
TCGA-86-A456-01A-0.105865013	4.655945359	3.425780274	5.327832042
4.383264861	8.266454403	3.359021476	4.326952758	1.65722215
3.213920561	5.133547505	-2.352370239	3.752212995	4.403660893
4.870250267	5.18106394 3.459472305	3.89878884 0.472723526	0.577195785
2.750415349	5.227966032	-1.063592703	2.657541731	4.50860897 3.45172885
5.52130038 2.7711105694.47121827 3.174460292	4.908229463	0.918831175-
3.507687707	3.530363157	-1.118445398	3.010478373	-2.830677257	5.60250865
3.348653828	1.358431512	4.714608696	-1.191434929	5.273302403
2.838773978	4.095274931	4.177285543	6.9323071161.401063827
3.787512219	6.537103013	-4.501070435	-1.787025911	4.77973722 -
2.380368749	3.332085414	-4.711267139	5.046306609	1.885550843
2.2706411833.884752743	6.130040673	2.793522754	2.229052051	-
1.833478101	4.676593569	3.937653159	4.068537631	4.269291017	-
1.326642102	5.094309677	6.243969199	2.936677644	-0.121392788
5.880586078	-0.398200543	6.060702916	5.121230739	4.528309845
5.510998852	6.377387064	10.760336 1.052814316	2.725789961

0.538645991 -0.758380963 3.474004012 1.044153404 4.335834429
6.865905302 3.731066014 4.668751237 -1.85918576 2.205152775 -
0.046970328 3.245102948 3.723463199 -2.012225383 5.35795977 4.507695561
0.767016926 2.819910813 4.103190161 5.031008864 4.830146413
3.179034522 5.335526462 5.168591369 4.905124294 4.347581519
0.75687342 2.878363575 4.775160908 4.866589043 4.746789783
5.704266863 5.3211910160.932035483 5.594534004 4.344797879
2.218546917 3.878270137 -0.375738713 -3.686494341 2.914937061 -
0.050626405 -3.319765734 -3.605653911 1.908490996 3.106039392
4.645571265 5.1935211073.288833256 6.263468996 -0.691696953
3.181728291 4.501066807 3.70986241 3.543006181
TCGA-86-A4D0-01A -1.364431151 4.636516052 1.549617368 5.302525268
4.2008731118.252895977 4.09886117 4.122627709 2.359145469 3.867871128
5.68169025 -1.549784413 3.2817711285.060108378 4.817770201 4.894599652
4.482581806 4.864320053 0.187589274 0.306497034 2.911513207
5.061303751 -1.97072273 2.991709409 4.737896572 2.021674269
5.653619347 3.45893015 4.509047743 4.747619608 4.6799811151.613023763
-3.57872473 3.318122176 -0.791289924 2.04147509 -3.988550352 5.610015995
3.284369993 -0.35942158 4.664732566 -1.230953369 5.240085045
3.909543943 3.75528407 4.360720066 6.7682773111.46635753 4.789823252
7.006659823 -5.209155081 -1.345022125 4.624348718 -1.746908894
3.871426938 -4.314566524 5.0612811812.29765816 2.1166686874.095687278
5.836756171 2.980842582 3.018852617 -1.978328345 4.443317832
3.937447891 4.422958305 4.409131809 -3.335643753 5.059829781
6.594777209 2.946144547 0.081786173 5.714089407 -1.104821947
6.191792721 4.988775498 4.974331537 5.483023844 6.553572382
9.9914049 0.550823263 3.129915793 1.810467936 -0.956129276
3.219776877 0.376414255 4.210610354 6.861235398 3.710694285
5.260509542 -1.94507417 2.8994008 0.11775649 3.4325611463.985456895 -
3.085336695 5.346685315 3.893952451 1.79557393 3.32966486 3.288924481
5.03168708 4.668946954 3.957861619 5.335395951 4.762667479
4.844488596 4.594313671 0.402565588 4.449843084 4.429810731
4.474562212 4.6732111976.026084363 4.863577448 1.5117450985.065854017

3.235891474	1.102581362	3.843313421	-1.227185052	-3.768942215
3.077632375	-0.203594276	-3.916843977	-3.281945827	1.20359206
2.181322697	3.8546457	5.904234399	3.813148998	6.443495573	-
1.363370463	1.586812944	4.574003184	3.919954693	1.684247666
TCGA-86-A4JF-01A	-0.543316829	4.874748297	2.35292454	5.440601606
4.410531476	8.259177101	3.274340523	4.345530986	1.872643909
3.344300593	5.316544548	-2.135186829	4.096222202	4.453843525
4.790997008	5.200629187	3.661574086	4.160444448	0.410512217
0.585668383	2.698319673	5.033402716	-0.95375919	2.359860641
4.566875384	2.87135622	5.755666764	2.677207687	4.632087779
4.196281862	4.883688611	1.291164317	-3.535676351	3.296752133	-0.70656458
2.944581669	-2.932050068	5.607888167	3.185871654	0.9689411
4.693323317	-1.229081144	5.389624344	2.763661139	3.955761066
4.344798971	6.729540421	1.445121363	3.784116534	6.924237521	-
4.778192882	-1.890318064	4.906360772	-2.297118985	3.41272045	-4.383610217
5.083130967	2.267356609	2.50012216	3.961154472	6.058551938	2.684489492
2.208217532	-2.199164713	4.60190507	3.931211332	4.113118661	4.38236204	-
0.640214716	5.079405801	6.383377868	2.807481035	-0.048623176
5.679499328	-0.539916374	6.15715494	5.056811873	4.739235961	5.492156858
6.350125419	10.20652403	1.05649403	2.912165799	1.186294344	-
0.576121476	3.344490108	1.066197395	4.390123396	6.796491291
3.679635329	5.022938104	-1.925854005	2.712840853	-0.257068748
3.323963285	3.70459205	-1.913276402	5.351681189	4.267216452	0.564079061
3.083953118	3.889692844	4.992045215	4.771090451	3.319143068
5.335427344	4.934031231	4.962084815	4.190157777	0.694291066
2.7220179	4.647941644	4.776598978	4.732315949	5.866263638
5.179006206	1.052804331	5.568398231	4.180784565	1.937567346
3.901940401	-0.683014896	-3.564376184	2.729213279	-0.064569258	-
3.578047326	-2.885805849	1.79354003	2.897987599	4.648294903	5.3131942
3.524712596	6.122459416	-0.035969713	2.825284111	4.437963955
3.624950344	2.951175587
TCGA-86-A4P7-01A	-0.189521849	4.902279311	2.758680858	5.200989475
4.090317018	8.213098712	3.674690518	4.222790447	1.908380037

3.307992583	5.047411941-2.308157902	3.602360199	4.259904003
4.960089875	4.932036708	3.745288743	4.044265651	0.625305878
0.53095454	2.822863314	5.268693617	-1.149050489	2.294435562
4.601604366	3.224338554	5.861482693	2.23056852	4.555245366
3.365129712	4.95608614	0.88919275	-3.421502402	3.38210276	-1.232971874
3.037354174	-3.013358714	5.570731582	3.231367183	1.382537495
4.705548977	-1.104250684	5.243506386	2.9748641124.0531101064.295703037
6.849384855	1.964107809	3.878464738	6.992065595	-4.542263968	-
1.816589358	4.30098016	-2.346161669	3.465395343	-4.850303385	5.171568394
2.25682589	2.064564303	4.153486546	6.090310656	2.743301947
1.746157415	-2.111741625	4.793345775	3.930862606	4.028367037
4.297315049	-1.608209556	5.093300816	6.423050736	2.960667054	-
0.066574213	5.923848641	-0.270711756	6.057030131	5.086405497
4.682433597	5.452134613	6.354772661	10.18127146	0.741134325
2.710403961	0.884684917	-0.745550242	3.8329591140.926667512	4.26415266
6.899808546	3.535705567	4.744268866	-1.7059395	2.235757311-0.381989911
3.223856048	3.852145835	-2.074596687	5.352817373	4.860555767
1.377902148	3.134580467	4.073489654	5.00584146	4.651446349
3.163192087	5.335639278	5.124130664	4.973891952	3.984332037
0.839864996	3.484889763	4.756156686	4.875927248	4.754838633
5.613569723	5.23360547	1.052281505	5.501042855	4.243436733	2.01532294
3.944209073	-0.536012211	-3.718914111	2.765676501	-0.370901228	-
3.472677389	-3.713893528	1.558321035	3.04736998	4.390137476	5.356701651
3.230964209	6.255239676	0.345518695	3.204648263	4.360850253
3.729444803	3.27943416
TCGA-86-A4P8-01A-0.266612128	4.754651854	3.0874205115.105781744
3.863814168	8.310102921	3.67176729	4.2724348	1.735455752	3.248982874
4.847778792	-2.499302866	3.1134187774.170838722	4.890938002
4.7271109063.553691648	3.759562525	0.466505443	0.472128661
2.679782297	5.522960626	-1.171832466	2.64807885	4.5721143483.213220367
5.885598357	0.877750547	4.513536727	2.689595801	4.897521941
0.708166364	-3.535523811	3.276144238	-1.357244842	2.894747752	-
3.296479005	5.550396269	3.584778128	1.539999514	4.717925062	-

1.15430639 5.0851141022.96883765 4.03053052 4.279134078 6.793273204 1.77454267
3.548693895 6.801444873 -4.559233858 -1.744763526 4.082037557 -
2.6934400113.467599133 -4.853241988 5.0847470111.982413705 2.305072737
4.1611441486.148225664 2.754696228 1.555890131 -2.281536585
4.847049327 3.926251047 3.851488813 4.169621047 -1.575921271
5.10409234 6.54540339 2.966973754 -0.101303545 6.062476255 -0.113637692
5.912936269 5.180984982 4.542892043 5.406125797 6.326886517
10.35488546 0.731562919 2.645283844 0.629562396 -0.79666215
3.708622207 0.84053427 4.216017897 6.9322111643.46520903 4.357390668 -
1.896548474 1.747219151 -0.419540436 3.194455314 3.958633535 -
2.097385673 5.331940194 5.376928213 1.5574961123.193863374 4.086690668
4.934953812 4.721006259 3.063251305 5.335659915 5.309718368
4.957822833 3.870297409 0.76012751 3.341034225 4.6411850874.870163536
4.800282077 5.571094725 5.287986943 0.964148393 5.498527344
4.554121385 2.337059098 3.834313598 -0.439056729 -3.608808414
2.842641428 -0.511568658 -3.439339767 -3.716495046 1.611036417
3.038995358 4.312504554 4.894498295 2.92251626 6.053935123
0.0723127113.2571105414.251783771 3.724181358 3.087440363
TCGA-91-6828-01A -1.033165643 4.454753961 2.02315239 5.218441879
4.409615888 8.387312626 3.515981937 4.265141537 1.75098007
3.440276227 5.196872902 -2.393349842 3.22793417 4.737009106
4.680250675 4.992887795 3.742897408 3.665468586 1.102162061
0.47648554 2.581734377 5.434729508 -1.556876532 2.8100707114.605355463
2.733097494 5.668314668 1.183726289 4.4441133843.560571609
4.737868918 0.861448158 -3.750679815 3.620308147 -0.805321744
2.713462836 -3.515065433 5.590612742 4.285494575 1.558672774
4.698869732 -1.347986298 5.27803489 3.431837949 3.960416182 4.2977444
6.863474498 1.604344096 3.743143268 6.454798937 -4.532371522 -
1.804849366 4.6208946 -2.195146257 3.414666211-4.803483782 4.955777453
1.649830262 2.1192448424.103524588 6.021981346 2.865445081
2.266850236 -2.653770032 4.625073499 3.942274902 3.770948754
4.393170293 -2.011030743 5.082592558 6.378090399 2.973814467 -
0.170947136 5.989468635 -0.569754559 5.947794704 5.2210115774.461603534

5.430274559	6.441367126	10.51353764	0.7118530862.770539791
0.751962094	-1.041995114	3.502164446	0.713420482	4.197225763
6.795045269	3.663079351	4.692666125	-1.878327051	2.127579051	-
0.167228277	3.248944652	3.575270177	-2.530012361	5.348759373
4.917741089	1.467705129	3.282301733	3.932817543	4.879834274
4.7977781153.212734051	5.335300206	5.174796466	4.86480295 4.070277945
0.400091071	3.5506711794.373443689	4.599716973	4.770674814
5.754410914	5.036049155	0.751384645	5.750122742	4.090868487
1.926454545	3.818170403	-0.232897285	-3.685122561	2.978681419	-
0.054667313	-3.201988488	-3.859197918	1.545910266	2.696586279
4.138982343	5.179526803	3.541450305	6.086037613	0.070157146
2.694806798	4.339865141	3.698364283	2.838576479
TCGA-91-6829-01A -0.991453678	4.314483149	1.450006234	5.44095063
3.989787648	8.401291517	3.574882203	4.200385403	1.747003778
3.872568792	5.501492328	-2.346879553	2.944023653	5.051557948
4.797571353	4.720524814	4.41950188 3.613403388	0.42219577 0.383298422
2.732386843	5.483087588	-1.683724398	3.106375741	4.618603383
2.632541561	5.497653748	1.150852354	4.481084904	3.692993989
4.967663944	1.614755893	-3.914195218	3.685925104	-0.819024301
2.159125415	-3.829856002	5.586858645	4.40600549 1.421028712
4.703117963-1.377998045	5.202464454	3.6112510973.74481852 4.193535282
6.840481	1.404487459	3.2290133116.88297049 -4.971062003	-1.750935592
4.716012602	-2.929751445	3.657986541	-4.107782966	5.028681818
1.816242196	2.763191077	4.004671281	6.016335734	2.70490615
2.680967726	-1.850120781	4.6851145553.939770606	4.093662888
4.203439144	-2.170250744	5.081745048	6.48941934 2.6774547110.225887186
5.638182087	-0.858324878	5.950053058	5.100139901	4.711888982
5.393929864	6.509548929	11.127427860.749446658	2.770204223
0.835580301	-0.701209813	3.19778546 0.856288937	4.20214264 6.890457849
3.528048339	4.96295496 -2.03026059 2.110740407-0.09020373 3.387696656
3.933319087	-2.316984256	5.322684841	4.744630997	1.981985047
3.602740796	3.759654419	4.913076412	4.683549631	3.25289937 5.33540341
4.952206074	5.092899988	4.096760792	0.596991023	4.006053586

4.402800793 4.735176043 4.770130175 5.640875839 5.132315094
0.875082905 5.669627708 4.069245901 1.97814696 3.723430996 -
0.589502195 -3.386842288 3.456450483 -0.316112044 -3.686975875 -
3.526410707 1.270576498 2.461538607 4.089560406 4.76127904 3.145376906
6.05891347 0.547940316 2.831448146 4.227014371 3.5711893092.766025613
TCGA-91-6830-01A -0.863998087 4.523609365 2.6110985265.458073798
3.885567194 8.325144523 3.30684965 4.284197182 1.484051762
3.569735593 5.506319715 -2.543786494 3.033794984 4.5594993 4.60822175
4.752483927 3.973543334 3.3891137920.499505006 0.416521914
2.656127287 5.005060384 -1.498578493 2.7311086944.506829347
2.893769229 5.633420379 1.950341928 4.442386333 3.256034768
4.760613956 0.947793158 -4.049866765 3.253100347 -1.333729861
2.392765103 -3.33203177 5.57452841 3.287155362 0.577739031 4.70098247 -
1.312659097 5.047370436 2.887315484 3.932572861 4.224547837
6.944742982 1.202418064 3.058567437 6.523015004 -5.287768686 -
1.984177222 4.292526818 -3.096135582 3.281727306 -4.807504413
4.9059763111.662931098 2.266375609 3.970235713 6.122027863
2.681880042 2.139595276 -2.236260201 4.610823468 3.933047799
3.802962643 4.238011502-1.865300312 5.089804579 6.26954027 2.813290526
-0.109159864 5.938103219 -0.613812496 5.946773804 5.202607863
4.4042571175.470813697 6.233260746 10.69900076 1.390526938
2.285442691 0.322528598 -0.579013459 3.261698233 1.001937083
4.302620365 6.84108625 3.5811927134.610618983 -2.271295927 1.905832677
-0.621955843 3.280276357 3.809103737 -2.430122579 5.337449582
4.638640082 0.976304336 3.108580999 4.532407601 4.839636187
4.533994634 2.766767129 5.335176474 5.23351015 4.910105877
4.1101179480.543787205 2.849913189 4.405721965 4.719101301
4.786368445 5.624094408 5.273590719 0.617906309 5.775446106
4.335970247 1.986980308 3.675220373 -0.691386029 -4.152625454
2.746014249 -0.876826733 -3.714407801 -3.776397693 1.50583807
2.580087776 4.344968632 4.778449524 2.859994155 5.83499928 -
0.23291603 3.314253277 4.336368693 3.584232808 3.105170577
TCGA-91-6831-01A -0.519227202 4.869542789 1.467165796 5.285391303

4.032461812	8.2885581114.104162705	4.157200064	2.348636653
4.301786382	5.375201016	-1.732698177	3.11389014	5.166305867
5.084273628	4.467258624	4.452167946	4.49489923	0.332821425
0.378275029	2.871374728	5.718611699-1.515573987	2.897505305
4.752378289	2.214622471	5.945513142	2.462175704	4.677426222
4.7451794114.758137376	1.648212028	-3.486462236	3.269048576	-
0.599912321	2.077424804	-4.116764618	5.580931049	3.249687823
0.2829116924.684984721	-1.098170262	5.316303704	3.912397962
3.719155579	4.339410682	6.944326343	1.545037528	4.410467049
7.314765386	-5.475129523	-1.453607761	4.745190996	-2.44213535
4.303565277	-4.10804983	5.2130297	2.532738074	2.75189089	4.269582961
5.885257571	2.675519793	2.495569894	-1.301451531	4.727251485
3.944686048	4.544620844	4.394680927	-2.47160345	5.071564686
6.601895091	2.78144012	0.364978091	5.701570769	-0.447527183
6.070370163	5.005577171	5.1112026645.374003756	6.673247335
10.30136228	0.197992946	3.142732683	1.649073706	-0.461761702
3.467622396	0.762248222	4.134424574	6.931991283	3.354959391
5.133342495	-1.818252481	2.705599429	0.21590005	3.378416205
4.192024307	-2.323475346	5.341246767	4.701482921	2.039186982
3.484782325	3.41234735	5.018082875	4.646068324	3.617057716
5.335504731	4.705410956	5.1177675	4.345941385	0.794927628
4.097773989	4.464481806	4.709194151	4.749374593	5.579344559
4.739266793	1.3511652885.260351847	3.823239065	1.576603343
3.863279888	-0.657653165	-3.285558219	3.883809347	-0.498776553	-
4.000643989	-3.242229436	0.968926102	2.494479491	3.825234375
5.612561399	3.43131959	6.564919052	-0.041901613	2.1165462974.667466438
3.798910705	2.384161388
TCGA-91-6835-01A	-0.298081102	4.80937904	2.11792787	5.246398086	4.236301897
8.300465126	3.667973307	4.252989743	1.962856299	3.712497192
5.12879302	-2.279595945	3.149428773	4.826425544	5.052948786
4.772528153	3.95130183	3.77336494	0.856205814	0.565413263	2.746476508
5.531063271	-1.237349545	2.612444125	4.642885834	2.965373471
5.829693706	1.39448026	4.612827682	3.863738752	4.929589436	1.1946805

-3.397717671 3.444930516 -0.886202154 2.792515119-3.450265794
5.577894233 3.803624738 1.520820755 4.706545655 -1.117626865
5.485718395 3.296104085 3.927736418 4.317421825 6.962218758
1.6611671923.647072254 6.904139883 -4.665865219 -1.759558755
4.609943045 -2.53719989 3.64757946 -4.643031535 5.12193641 1.875381348
2.265331493 4.181252427 6.017472002 2.836133941 1.98005324 -
1.889783689 4.742701045 3.937207265 3.814400927 4.369455669 -
1.180656522 5.098926563 6.366550281 2.844613962 0.071253869
5.840470508 -0.121528836 5.970059803 5.098725541 4.62270685 5.37924972
6.47531737 10.65928958 0.746409446 2.871880228 0.869712945 -
0.68315347 3.8173811870.960629426 4.173180224 6.882009685 3.485757697
4.80592892 -1.712284894 2.249781881 -0.256309527 3.287821857
3.885952802 -2.041075478 5.340448149 5.01200095 1.702904651
3.547554154 4.02976097 4.927691728 4.699624596 3.262085846
5.335575821 5.032342099 5.040849498 4.046541488 0.676112851
3.767602994 4.55622445 4.794935258 4.805527277 5.518740584
5.150752914 0.787472235 5.694775344 4.300920399 2.248709563
3.865634415 -0.150607602 -3.654171915 3.359919382 -0.372480193 -
3.548339235 -3.825599752 1.466050081 2.754990223 4.1578113635.275202978
3.392890644 6.105195391 0.758669053 3.009219276 4.362304042
3.687247696 3.174358827
TCGA-91-6836-01A -0.92817294 4.836361894 1.763383841 5.4411439724.257661345
8.136924256 3.753936933 4.129872732 2.01408621 3.698517347
5.197766885 -2.211207046 3.316179868 5.048190914 4.9433611124.59230979
4.23869929 4.23777688 0.535081975 0.358289567 2.67386348 5.497945793 -
1.520623275 2.694864224 4.683420849 2.216388926 5.326272447 1.66308517
4.535745705 4.763550948 4.88361361 1.337861929 -3.718217185
3.297397148 -0.386447874 2.48230324 -4.116590194 5.618582124
3.683498139 0.61619815 4.672409035 -1.362910075 5.100348356
3.619820899 3.63877658 4.1526346116.895194681 1.279692693 4.173131684
7.190116608-5.380194066 -1.847257879 5.463631187-2.162153426 3.459293781
-4.713609078 5.122260763 1.7078131132.801273281 3.751708219
5.624217682 2.634496408 2.733097825 -2.310875832 4.571322201

3.936722949 4.016893608 4.614465602 -1.125975294 5.073569158
6.3231941142.659216568 0.2443811115.133880288 -1.017778107 6.175204688
4.885676171 5.062826663 5.353312765 6.678785581 10.74005086 -
0.515262293 3.025723254 1.412194634 -0.665874648 3.40297437 0.731978792
4.229854145 6.744916702 3.245431637 5.208077819 -1.866857622
2.557870093 -0.058988395 3.327620481 3.818751113-2.076043472
5.337505926 4.246617471 1.223008554 3.168662018 3.202345706
4.980463681 4.895046732 3.474920337 5.335310801 4.170089309
4.96532837 4.17035418 0.418617918 3.710359906 4.523687747 4.652704821
4.747504342 5.409979772 4.385867546 0.710050267 5.218490798
3.614895305 1.651395683 3.883733638 -0.395947828 -4.021450024
2.91269117 -0.238914447 -4.033195073 -3.745431057 1.135512882
2.264626377 3.862414443 5.729136185 3.907155947 6.199295508 -
0.487824318 2.174088726 4.261759579 3.451231274 2.359763993
TCGA-91-6840-01A -1.295192734 4.682963077 1.292800914 5.151108219
4.337873679 8.257223444 3.869628091 4.063229054 2.15134384
3.482515678 5.182615219 -2.253353789 3.179736153 4.958391405
4.920696666 4.504328549 4.545920183 4.07031657 0.3425112990.549231495
2.758426082 5.527746456 -1.593177191 2.607750729 4.608779622
2.661816605 5.576271684 1.896237929 4.64018822 4.476132392
4.922672478 1.278845926 -3.518620304 2.887923352 -0.758700827
2.808970121 -3.925933619 5.578300505 3.659588556 1.403856294
4.697415505 -1.160536842 5.345054024 3.333873233 3.709466675
4.1372465 6.99889574 1.663622026 4.028106277 6.973495742 -4.849192885
-1.788864923 4.389246174 -2.907165591 3.880059639 -4.66645644
5.207424785 2.164060898 2.620958417 4.106894102 5.887545178
2.791936954 1.397254652 -2.363638785 4.62143125 3.926496626
3.836049984 4.285640141 -2.455772181 5.076928378 6.508976023
2.836765069 0.419683893 5.374123606 -0.639509347 6.000522565
5.088947046 4.8783112485.31514805 6.428997858 10.22658302 0.703039155
3.040692965 1.086032975 -0.591485219 3.026854797 0.694818221
4.298179377 6.928405071 3.494555964 4.900659287 -1.786812443
2.283395749 -0.316019232 3.293441215 3.932438899 -2.764912398

5.334066205	4.467534518	1.238642455	3.38813044 4.099379424	4.867684
4.548064039	3.745832926	5.335284992	4.753603351	5.004596269
4.029300017	0.716219181	4.129816818	4.2681128674.7117430024.766850141
5.8221185355.135704649	1.195393168	5.327561729	4.076681006	1.73392824
3.897172817	-1.126730019	-3.820361463	2.939756205	-0.537538635	-
3.660410383	-3.572752115	1.348304482	2.339275372	4.076085344
5.337607098	3.412481222	6.277558275	0.885032732	2.862780351
4.5411157073.688000756	2.869556319
TCGA-91-6847-01A -1.524251718	4.523543884	0.334665146	5.332310713
4.494454053	8.184805944	3.762725373	4.069658323	2.300423197
3.664841337	5.033205466	-1.921860081	2.884715526	5.359349768
4.70767626 4.574394047	4.685088575	4.105515644	0.60758518 0.297598448
2.790217863	6.014293548	-1.876898031	1.687450287	4.821717414
2.0714721145.567408637	1.164015676	4.595783189	4.46504393 4.974800599
1.81407812 -3.767074408	2.843537616	-0.668876718	2.656089483	-
4.743149985	5.618367886	3.716291999	0.781678023	4.68119829 -1.266687848
5.046141586	3.643880801	3.494209629	4.22187067 6.927624845
1.430670484	4.331945347	7.36254359 -5.158337015	-1.33960234 5.57363803 -
2.273218035	3.937670155	-4.313397879	5.190782299	2.071615615
3.244317521	4.048910655	5.500529306	2.023295564	1.411184194-
1.877587517	4.551422472	3.941036095	4.326319198	4.179353621	-
2.323452771	5.052155662	6.464376578	2.886478768	0.513402022
4.983590132	-1.223092067	6.149652993	4.903201294	5.189744911
5.237030368	6.650146369	10.80312261	-0.433762281	3.325502164
1.535839938	-0.626644631	1.900050234	0.560918626	4.16612979
6.859017463	3.18927271 5.211731231-2.094491178	2.401827005	-0.028221189
3.358413513	3.918660523	-2.625954559	5.320858251	4.389254007
1.174423306	3.320351615	3.387878277	4.889927978	4.987141655
3.976867801	5.335223981	4.129738095	5.081263487	4.04084944
0.471973545	3.457828947	4.212387288	4.547627595	4.743025284
5.5002114664.6051115330.847091935	4.621087488	3.710519701	1.235537456
3.949409368	-0.666481763	-3.598034068	3.270244193	-0.131324581	-
3.890075838	-3.47213598 0.968375947	1.884647753	3.665254758	5.694591582

3.732624475 6.417132294 -0.121088273 1.867352198 4.503372104 3.6579487 2.758191317

TCGA-91-6848-01A -0.474735026 4.508627278 1.563924108 5.481881335 3.561445054 8.224989937 4.37894306 4.2189118492.184837135 4.843606562 5.337461192-2.27885837 2.783598121 4.705201778 4.845839978 4.260160831 4.581432878 4.394064389 0.0770115940.184178858 2.753486232 5.5494402 -1.942309441 3.912071201 4.698430038 1.864937564 5.872785472 1.021881062 4.562565296 4.6119479034.950393054 1.760854389 -3.602800459 4.056505641 -0.473763 1.033580462 -4.519294087 5.592770293 3.508597198 -0.277226091 4.680084792 -1.164625878 5.399713958 3.223999493 3.63324053 4.212796655 6.63072619 1.00906292 3.646967988 7.538684863 -6.041130149 -1.426232078 4.943534801 -2.668749858 3.781121113-4.805208111 5.026527325 2.018981641 2.512744838 3.867172358 5.93598104 2.42599767 3.466651143-1.902944966 4.924375055 3.937503849 3.960106691 4.604501026 -3.606753754 5.09785992 6.476246807 2.48765059 0.273663975 5.607969463 -0.893193138 6.069983183 4.897521502 4.907005649 5.43442255 6.64605667 11.15080037-0.612996565 2.904878459 1.402376157 -0.167013826 3.844914298 1.065968156 4.127613909 6.810702861 3.074153385 5.015548542 -2.093432597 2.582611816-0.673063091 3.431846402 4.511245687-2.779875723 5.320445174 4.937535517 1.995390353 3.389342053 3.288657226 5.078103802 4.820577667 2.76617652 5.335924678 4.50004386 5.179422843 3.8553511630.841340136 2.853328041 4.455502891 4.862390125 4.764453893 5.353755366 4.465173207 1.076064044 4.908313758 3.244087698 1.449663497 3.634542529 -0.466685396 -4.698709682 3.178221039 -1.210376954 -4.548852908 -3.389549338 0.834754999 2.417038492 3.980605973 4.83067522 3.147484092 6.369136649 -0.049886739 2.1911405564.28034418 3.553619887 2.496031569

TCGA-91-6849-01A -0.682200419 4.716691943 3.499602573 5.485543109 4.309085653 8.3371175353.241759478 4.338251617 1.876089295 3.224778128 5.325894628 -2.287287319 3.561869742 4.33056998 4.580535252 5.224897588 3.384744038 4.123259296 0.65576057 0.518924842 2.732636484 5.002490333 -1.218261544 2.327778559

4.506247235 3.37870523 5.567784941 3.404473356 4.482427133
3.260398723 4.856338223 0.876941909 -3.876498329 3.507524308 -
1.350550535 2.868069193 -2.619768052 5.597396705 2.931404886 0.95387094
4.720171754 -1.251532457 5.190865958 2.929982264 4.114080831
4.247843387 7.094339576 1.352724671 3.598483686 6.472432455 -
4.677684373 -1.707238504 4.567226672 -2.653108204 3.331352496 -
4.7011483114.980865931 1.84781199 2.157801918 3.841340284 6.12712611
2.832109499 2.214681986 -1.587149142 4.56883519 3.937675974
4.297782599 4.039743623 -1.663580154 5.085633101 6.292624976
3.032075785 -0.249312643 5.994209942 -0.665158731 6.037780416
5.214482837 4.521092939 5.521293857 6.347517784 10.61309827
1.602562291 2.409154233 0.389218098 -0.764839052 3.195652422
1.006901709 4.394393573 6.856459839 3.88908251 4.502233297 -
2.134283597 2.072586263 -0.061262408 3.283870935 3.81801655 -2.240011906
5.353905295 4.665127744 0.51902795 2.612223628 4.427678743
5.007880771 4.659844847 3.094793248 5.335410844 5.425427711
4.814014808 4.506275679 0.53661407 2.79894058 4.69453525 4.753688868
4.742646654 5.78852691 5.471858313 0.898676572 5.8185116724.380036765
1.987021092 3.89219067 -0.669257177 -3.878267237 2.986950114-0.336636967
-3.300540849 -3.604339516 1.916641784 3.003988621 4.631621778
5.077138773 3.324791712 6.15501694 -0.85738309 3.24577266 4.604574274
3.659100712 3.085017523
TCGA-91-7771-01A -1.161148514 4.574995305 2.58641395 5.437756688
4.141905161 8.408195353 3.398410101 4.325747394 1.685818299
3.27767204 5.476510671 -2.376502164 3.259163196 4.378574327 4.65814214
4.828574191 3.684127141 3.682261976 0.999140698 0.500573919
2.468954606 5.133033986 -1.650138833 2.578745995 4.538390411
2.885772842 5.867598221 1.36686373 4.336444419 3.196312425
4.567474029 0.746908602 -3.978073506 3.686310458 -1.035963964
2.590779715 -3.187030728 5.581909815 3.509069557 0.79548611
4.703722957 -1.288747722 5.182121396 3.058672133 4.02717188
4.330660408 6.9439097111.469086591 3.244761822 6.386488029 -
4.775960172 -1.918638059 4.570055582 -2.473251126 3.091581779 -

4.814419085 4.850831929 1.652362261 1.976210149 4.131258452
6.074178799 2.8614511742.119349293-2.454141391 4.582787597 3.938921019
3.858633196 4.269808885 -2.313077953 5.089989799 6.330111857
2.983830937 -0.166058832 6.216611725-0.813741232 5.960215396
5.265769944 4.323898186 5.4694257116.375737772 10.61866 1.234088601
2.457207899 0.539601396 -0.966893331 3.429146938 0.843709063
4.294383775 6.829649818 3.753507306 4.62598718 -2.044326902
1.907493303 -0.395838452 3.23268054 3.747705332 -2.777082509
5.344476317 4.875577643 1.173475407 3.2864311844.489338647
4.860224174 4.703091809 2.820259704 5.335249147 5.324505264
4.736701304 4.105199277 0.247558435 2.72843341 4.432566545
4.554515515 4.782505465 5.728175658 5.16087272 0.80451783 5.773528703
4.435847383 1.850068366 3.728462466 -0.363327718 -4.052407051
2.78003226 -0.304867999 -3.243290734 -3.715794722 1.543439024
2.666880287 4.3601183994.9534311683.273092053 5.765688778 -0.30297595
3.103975019 4.257465923 3.617709818 3.195756131
TCGA-91-8496-01A -0.580611796 4.615850628 3.280053973 5.48967393
4.340893569 8.299744203 3.330034509 4.356806763 1.929104463
3.279146252 5.367816697 -2.231180428 3.969200195 4.478081128
4.861088814 5.062610522 3.174888508 4.1150312530.610800822
0.624837125 2.5811420335.349983041 -1.23208044 2.539009333 4.535986562
3.131297124 5.738906559 2.831498537 4.48283907 3.0919811694.673780338
1.143223913 -3.835105412 3.608691039 -0.97958529 2.808087046 -
2.779762884 5.586638838 2.718081895 1.11782395 4.721024082 -1.202208367
5.371412278 2.605426917 4.03538337 4.344587202 6.99831759 1.356375719
3.426050734 6.69454738 -4.761506445 -1.736555829 4.441688025 -
2.447609384 3.55446779 -4.147232099 4.985552087 1.956955234 3.21815207
4.050167061 6.121285613 2.785463437 1.980215625 -2.210916964
4.546563636 3.937491891 4.316164532 4.217010115-1.617397787
5.079852856 6.341757618 2.960188223 -0.051481049 6.092210267 -
0.523491988 5.949194748 5.175453667 4.420613302 5.482839217
6.311599311 10.511428531.30149215 2.667128466 0.974145128 -0.689936654
3.100794616 0.972265839 4.353449287 6.892031396 3.70849029

4.707543142 -2.153521804 2.130104049 0.019084814 3.315404859
3.866770663 -2.335921726 5.339685802 4.832871654 0.347088594
3.0261188064.167109749 4.979928914 4.7256911693.0211165985.335199561
5.147384671 4.870840971 4.578774526 0.499815689 2.021522379
4.650140401 4.63978618 4.783358947 5.674949412 5.129565278
1.197538242 5.629773461 4.498105668 2.186954018 3.841443841 -
0.526154171 -3.668267856 3.122691532 -0.101131514 -3.331832599 -
2.775509634 1.852569016 2.734045676 4.630786172 5.036318496
3.093447178 5.984164409 -0.552903361 3.088230843 4.684301777
3.698575452 3.308230742
TCGA-91-8497-01A 0.188321996 5.063797899 3.261582444 5.087484876
4.057924318 8.287428389 4.029650357 4.250636815 2.320346924
3.228028026 5.121900574 -2.159791631 3.484417368 4.330502517
5.150342819 4.890461273 3.724766016 4.575995065 0.702508599
0.667876697 2.909216529 5.511115217-1.086325984 2.702473146
4.6061122953.185849216 5.870833004 3.12731845 4.646458421 3.329164143
4.839713218 0.876896875 -3.213251203 3.456769935 -1.325504358
2.952205721 -2.934272184 5.554927053 3.424990843 1.257408757
4.722866673 -0.937709077 5.101754209 3.1228989 4.129939501
4.391298407 7.043940779 1.977813283 4.392992578 6.91415854 -
4.451268757 -1.427706862 4.012202288 -2.230424679 3.616231242 -
4.539640873 5.298291889 2.584656519 1.8754114464.240523156 6.137086936
3.076723083 1.806173401 -1.686279707 4.8276691133.931316135
4.4091136164.309855082 -1.907036062 5.093256056 6.638178423
3.102556075 -0.157345119 6.216913273 0.180738581 6.020663728
5.198342013 4.837885741 5.457387383 6.4416246 9.919546687
0.943787376 2.823894984 1.086472106 -0.672854528 3.908557262
0.814369025 4.227758226 6.97489569 3.764714938 4.546504824 -
1.645819719 2.192533725 -0.166659406 3.25866563 4.117893163-2.165707633
5.351671236 5.087633534 1.731459757 3.282332409 4.34857167
5.045514913 4.623012441 3.266950309 5.3358251115.4011419694.889051206
4.265718715 0.9117495313.963260917 4.633391628 4.813736577
4.753221605 5.667600847 5.201095418 1.285270144 5.529197636

4.222121576 2.064259649 4.017672946 -0.567752545 -3.287025508
3.234909458 -0.3432427 -3.364358901 -3.4292382 1.633394582 3.208323525
4.418361703 5.586673388 3.338351486 6.448613781 0.432427145
3.137824992 4.60438281 3.968343957 2.822345772
TCGA-91-8499-01A -1.577390204 4.744468184 -0.009313114 5.364408355
4.144840185 8.049038754 4.7951270113.930913462 2.950230794
4.635605315 4.547080163 -1.142516581 2.867895968 5.347476716
5.055099109 3.64581958 4.695304433 5.377550462 -0.299593764
0.056568441 2.803758782 6.35443965 -2.247938103 2.2127102114.918542839
1.417623223 5.840456202 1.8164323114.696615434 5.739514439
4.645102071 2.17556521 -3.82137579 2.645341934 -0.090987902 2.160843081
-5.812585645 5.583083206 2.91794677 -0.370516279 4.662972402 -
1.158829792 5.641298015 3.879300868 3.354502017 4.296718967
6.838861664 1.349133786 4.548549458 7.688209982 -6.026407134 -
1.156302546 4.214248896 -2.294094825 5.229741823 -3.658351498
5.222876455 2.249571364 4.092239223 4.401386776 5.419548388
2.577049047 1.957244577 -2.339558927 4.566563518 3.927718985
4.546617062 4.40317414 -3.583840785 5.067051998 6.665792775
2.867386614 0.903009918 5.295482071 -1.135122279 6.116893704
4.874644909 5.212456966 5.221936348 6.561653809 9.707001564 -
1.061535346 3.390314554 3.052334197 -0.581392645 2.59610928 0.234493998
4.11155232 6.848154212 3.083916669 5.311425878-2.072275709 2.755328049
0.45092215 3.490150242 4.370314254 -3.428873276 5.305271418
4.9110391031.882586864 3.7918285112.481441406 4.93538792 4.702168187
4.073305891 5.335202983 4.235016085 4.959601304 4.582825259
0.168709758 4.099141083 4.274729817 4.402315028 4.775093451
5.485330805 4.2184983 1.674210949 4.278515176 3.698149399
1.070838122 3.863081259 -1.993938924 -2.995429701 4.233074065 -
0.689839456 -4.615479403 -2.85096884 0.49676609 1.554075207 3.011412435
5.624435198 3.9119511756.384679939 -0.072675317 1.052070019
4.651785802 3.939858093 1.809110477
TCGA-91-A4BC-01A 0.477435175 5.13203189 2.504769777 5.282005654
3.685575168 8.206754002 4.160602492 4.166010025 2.39686605

3.881913466 4.95384529 -2.021200813 3.455282902 4.487058442
5.298248769 4.564508017 4.023553284 4.635076138 0.245754735
0.452053286 2.883452491 5.603331508 -1.166033316 2.731565885
4.719232158 2.61459162 5.933326854 2.733309413 4.721301043
4.499184192 4.820537712 1.280786978 -3.278507114 3.406832089 -
0.843713135 2.154496481 -3.454567418 5.563616442 2.785725782
0.037918366 4.708051252 -0.919573611 5.277434394 3.102355729
3.929683857 4.344883296 7.006032346 1.919469051 4.453727017
7.563932494 -5.213029418 -1.642381416 4.40362263 -2.309091736
3.785963848 -4.59876158 5.296978912 2.699035744 2.314130457
4.254810056 5.941633658 2.76275913 2.245761981 -1.157714496
4.861355733 3.9311418854.498240822 4.467920093 -1.865696817
5.0943118556.728594776 2.836221578 0.10833706 5.798274273 -0.027477353
6.055435378 4.983943406 5.1932112085.492049423 6.56956632 10.20529537
-0.231865925 2.98674972 1.434496372 -0.44140685 3.946352343 0.829188778
4.224203366 6.990302454 3.336883998 4.901448595 -1.675144277
2.611542286-0.314323303 3.315396167 4.311765396-1.976039116 5.336170302
4.960670509 2.045744643 3.550651918 3.60891945 5.077487683
4.654763252 3.26557245 5.335900939 4.737650656 5.077531595
4.181608662 1.093452133 3.8111832094.632624566 4.855865009
4.757242966 5.473855768 4.755728158 1.4064836 5.244930286
4.006827378 1.946294472 3.98108754 -0.682888287 -3.288603877
3.466514449 -0.704086901 -3.962590955 -3.264378961 1.128044449
3.000959693 4.102676275 5.634261561 3.144390331 6.47603784 -
0.571993719 2.391965168 4.515281705 3.820576829 2.534355364
TCGA-91-A4BD-01A -0.324277328 5.11050839 3.434894954 5.472506203
4.320883636 8.236553655 3.703979975 4.3436812112.143104889
3.254863021 5.176046536 -1.967283035 4.046557022 4.435889212
4.969734641 4.939262672 3.009613744 4.5027711350.3811168830.53432985
2.65244767 5.404982214 -1.060511119 1.917794419 4.547982951
3.073078867 5.875993544 3.7145114784.619845594 3.713008354
4.770832323 0.973468777 -3.499199149 3.260499673 -1.033845428
3.03176055 -2.952496811 5.598109577 2.799442256 0.364125819

4.732473135 -0.979583447 5.223705209 2.360605816 4.049799042
4.335032521 7.214340506 1.389968472 4.089754999 6.860972029 -
4.79844312 -1.626851113 4.594617817 -2.274339004 3.693913191 -4.143521477
5.1283056112.349588776 3.067370361 4.01179723 6.081820993 2.760098383
1.59747818 -1.197427249 4.558273794 3.933171919 4.463340742
4.199588299 -1.182928005 5.088133971 6.45329063 2.99692783 -0.045969094
6.151509673 -0.146804235 6.065900886 5.136338825 4.819137053
5.47881245 6.307099289 10.25201096 0.772238375 2.837046193
1.054528802 -0.629945333 2.9110475230.939301015 4.402654578
6.916462885 3.674751419 4.599431613 -2.118076403 2.36932541
0.276535658 3.278574763 3.889310982 -2.409149006 5.343140522
4.815658587 0.266748286 2.807238027 4.185384621 5.046031087
4.833560354 3.265495018 5.33554389 5.057062909 4.858768674 4.82657297
0.642378223 1.925224971 4.684177063 4.759222769 4.774387672
5.735876607 5.1125668991.285327442 5.308534896 4.609352062
2.075990827 4.005161013 -1.181297467 -3.729556087 3.365455153 -
0.100564577 -3.492912668 -2.680573482 1.93808931 2.8993381194.587629192
5.496899656 3.42891117 6.101127259-1.184809722 2.880853765 4.787794932
3.838242518 3.121629885
TCGA-93-7347-01A -0.427028341 4.709183035 2.952641281 5.388707095
4.159737558 8.332260852 3.47456541 4.381285245 1.641838504
3.330009041 5.602097169 -2.413082835 3.686167465 4.368948021
4.873757505 5.147736534 3.447160565 3.772358022 0.262428067
0.568967678 2.6659593115.139576147 -1.195012793 2.806668121
4.470070669 3.1425261135.744909782 1.7333112884.567730261 3.188829684
4.894876542 0.89246607 -3.527239283 3.619834352 -1.07446864 2.579306853
-3.000481342 5.584272686 3.680242502 0.91668701 4.720866532 -
1.094847658 5.043104999 2.650991282 4.10751825 4.308591569 6.895305175
1.305586632 3.484561715 6.692840998 -4.784101498 -1.793483786
4.332623443 -2.583964471 3.313961085 -4.551342983 5.116090434
2.019947625 2.08661226 3.89709239 6.254133629 2.837250363 2.240445212
-2.142985852 4.710682438 3.935092809 4.021260524 4.321130305-
1.819407047 5.098751341 6.388360468 2.863668898 -0.163274496

6.269370254	-0.243188682	6.0150261135.143412428	4.548150947
5.5608211676.210281873	10.61914766	1.193004672	2.607794434
0.558174665	-0.620286713	3.626229737	1.0428116724.314064145
6.867793574	3.606429955	4.670598087	-1.933543887	2.231008154	-
0.291274372	3.273386691	3.758653679	-2.530209597	5.350098697
4.742498314	1.060392652	3.147525031	4.197445379	5.008374345
4.701227993	2.93639901	5.335633873	5.150526797	4.93706394	4.451686931
0.726821271	2.667580979	4.590932518	4.834233703	4.759020175
5.691095583	5.16965493	1.01880162	5.793994209	4.298514002	2.146433911
3.833681071	-0.578985502	-3.962635626	2.964195051	-0.230722712	-
3.421254943	-3.498824612	1.937672032	2.997665561	4.680212865
4.945930248	3.054314525	6.049748425	-0.158754203	3.176766039
4.458947091	3.770295981	2.974944486
TCGA-93-7348-01A -0.711159217	4.73712323	2.18395633	5.353198149	4.290781539
8.300064942	3.480233897	4.235670041	2.05752406	3.4783981135.21023525	-
2.263939262	3.500700868	4.396829045	4.805473177	5.1124666973.757453395
4.005619604	0.576478598	0.478922178	2.875331966	5.310206509	-
1.126400204	2.24635302	4.570472875	3.0636311095.524725581	3.256974103
4.584662888	3.406196139	4.9161931151.032288118-3.698263189	3.640315951
-1.078107676	2.741002636	-3.151718479	5.587951749	3.65014264
0.739330932	4.719683678	-1.156259867	5.276946288	2.945546419
4.0511231014.319081733	7.151338575	1.630807186	3.529385412
6.837379761	-4.643450161	-1.742891317	4.576951911-2.561172565
3.349758774	-4.281580166	5.067798285	2.145899491	2.392245913
4.066885084	6.103013437	2.817820671	1.982910997	-1.595717209
4.579079341	3.933642657	4.27282664	4.208692249	-1.107457621
5.090214851	6.423530987	2.989762197	-0.077095374	5.913358689	-
0.512633306	5.991398263	5.21754164	4.622719864	5.485089335	6.315666535
10.52845336	1.273207215	2.6008583110.830700914	-0.761826174
3.234860056	0.852328016	4.328400414	6.860243505	3.745835479
4.692229336	-2.008375977	2.152139167	-0.018262132	3.292381926
3.704314678	-2.092609664	5.343107306	4.779544023	1.200842376
3.164719516	4.125668627	4.968429393	4.743829519	3.166921486

5.335418976 5.167690273 4.920228566 4.380858989 0.586420988
3.384766969 4.51662043 4.739377374 4.762757201 5.701367773 5.27258886
0.9045110185.679651433 4.227737253 2.175980072 3.962912407 -
0.601844078 -3.422806491 3.311035818-0.195336337 -3.371378028 -3.222708903
1.56664677 2.96177036 4.474536996 5.209528213 3.374583433 6.011204018
0.1145533933.1149732214.530000145 3.745871448 3.046705339
TCGA-93-8067-01A -1.554480988 4.457700893 2.456370996 5.411978774
4.352135465 8.332549884 3.097591289 4.307336623 1.645117656
3.224845943 5.712873021 -2.276643259 3.724926091 4.274114186
4.284881804 5.279793208 3.894644162 3.963358894 0.359361872
0.39229279 2.71301039 4.913359286 -1.569336521 2.63775319 4.505064064
2.809094676 5.701814902 2.909257753 4.4850117763.538964441
4.898108596 1.118879311-3.997603148 3.517808133 -1.072484716
2.805186907 -2.942691441 5.616148545 3.372826835 0.323917761
4.69444361 -1.368755251 5.042998043 2.698480644 3.993973087
4.189399955 6.9132112841.273570484 3.406633801 6.449739998 -
4.87997201 -1.831480858 5.029226457 -2.291056215 3.173123586 -4.752009801
5.070460363 2.037044892 1.98836076 3.629234172 6.04328845 2.697490542
2.482449293 -2.222043743 4.473091315 3.924515467 4.034846349
4.334427175 -2.093079891 5.07534098 6.438169919 2.929078196 -
0.165723572 5.755287769 -1.183225738 6.10505981 5.094544936 4.637995901
5.508985479 6.26461601110.63943683 0.780004589 2.381048577
0.787435131 -0.582235477 3.04509709 0.73817757 4.388082509 6.786991752
3.656254472 4.894172891 -2.094128527 2.016993453 -0.134388811
3.310590592 3.575800885 -3.00438502 5.348490274 3.999640816 0.32767257
3.02225494 3.940153935 4.901480984 4.78778189 3.377237848 5.335111115
4.695716331 4.823057173 4.327927883 0.35559162 2.554307043
4.462818388 4.680320824 4.700523455 5.864035329 5.25198401
1.0113652895.717937545 3.625752427 1.534727234 3.867696132 -
0.637062579 -4.207714339 2.576976124 -0.142266812 -3.543497033 -
3.717065824 1.877010299 2.4143411444.474566456 5.1256116 3.647210477
5.972204274 -1.439209803 2.672916473 4.325486385 3.534400104
2.44440405

TCGA-93-A4JN-01A	-0.168689613	4.773081842	1.7117805525.047761184
3.982985343	8.235739794	3.961269094	4.196565862	1.966059996
3.531885268	4.920531591	-2.237530444	3.392432683	4.77936391
5.069413516	4.6271147044.213245171	4.166545095	0.280298652
0.483001689	2.902049873	5.788576374	-1.011036249	2.350680327
4.669798869	2.706573481	5.825253054	1.473995886	4.694501299
3.807759008	5.001974677	1.387206672	-3.284915314	2.96732983	-
0.927503075	2.84904331	-3.684327611	5.568769672	3.2879911181.431147796
4.700837425	-1.130319741	5.105171565	3.188301314	3.830969649
4.207138701	6.795844715	1.835518993	4.250019986	7.228275263	-
4.670444768	-1.609638723	4.705435678	-2.445547538	3.864724646	-
4.441655476	5.294833319	2.303282098	2.763029535	4.264151557
5.941378051	2.595776133	1.730632581	-1.532256921	4.853809653
3.928402648	4.20497101	4.31124868	-1.043035857	5.077826251	6.580287538
2.85553723	0.231290356	5.515903493	-0.174415536	6.002472838
5.002962987	4.927583959	5.374294518	6.51436025	10.13697714	-
0.0880511063.15399758	1.146440302	-0.424661577	3.558915094	0.733014754
4.204530816	6.957954647	3.300586436	4.958535008	-1.730296005
2.207763827	-0.177684091	3.292059351	3.848563602	-1.603622244
5.332478208	4.747681763	1.528506392	3.509495715	3.77913979
4.948250804	4.771488604	3.61689364	5.335521792	4.726831797
5.097689034	3.806673053	1.098600412	3.430235986	4.56255708
4.934220516	4.752505425	5.638297521	5.062376597	1.089737547
5.303772217	3.98631433	2.162400839	3.957463272	-0.208720216	-
3.126150105	3.269213408	-0.186138663	-3.727707594	-3.544741408
1.234589576	2.792068377	4.1140499835.470943579	3.126126306
6.602547399	0.208379155	2.643295607	4.39358502	3.743154464
3.028597429
TCGA-93-A4JO-01A	0.182376729	5.273504348	2.60884508	5.257118574
4.220178509	8.14739353	3.727274741	4.234587671	2.080542994	3.56800636
5.274248908	-2.162687077	3.597957389	4.317384325	5.083318137
4.915483613	3.907098751	4.197980174	0.170902666	0.457760971
2.848989878	5.436342696	-1.025264472	1.50949293	4.638467094

3.002402282 5.883837773 2.392469152 4.701479629 3.972464375
5.014376104 1.172364206 -3.415955754 2.88048404 -1.070422146
2.810012778 -2.953143474 5.581531509 3.140066746 0.55508031
4.710451661 -0.976621296 4.94019283 2.849267276 4.07220742 4.272901368
6.974096189 1.805397445 4.1101293137.27183841 -4.829437419 -1.56914745
4.616312637 -2.402932489 3.633843782 -4.747708387 5.34487141
2.500570875 2.290816282 4.064030224 6.021018261 2.611223249
1.407983084 -1.35443226 4.80099308 3.92681775 4.197623961 4.339125273 -
1.24906426 5.0993113496.535477636 2.923889598 -0.011920914 5.869185992 -
0.184759242 6.1741143865.0211884164.920495205 5.448967247 6.281671453
10.32191052 0.371334776 2.732273409 0.748607249 -0.40170007
3.517400631 0.910079252 4.29028707 6.975833094 3.384791771
4.764416277 -1.753011354 2.508386943 -0.434811438 3.259317214
3.979728951 -2.129746094 5.346758537 4.69552355 1.233760719
2.961992681 3.912390885 5.053053998 4.70878569 3.195094536
5.335729443 4.730304691 4.908273542 4.185089782 0.989140381
3.109626457 4.610730747 4.930505393 4.74688133 5.443665913
4.975171295 1.167590834 5.417458551 3.964607735 1.918024591
3.971432177 -0.679480744 -3.61603538 2.832138792 -0.331867171 -
3.724576299 -3.539537796 1.4110453063.078591314 4.26031644 5.529436356
3.438246161 6.306073385 -0.055967933 3.039678638 4.482167225
3.767735652 3.052355989
TCGA-93-A4JP-01A -0.697018788 4.622707572 2.073835741 5.177065514
4.23059441 8.300572412 3.427230259 4.201453498 1.826799382
3.288328887 4.847626325 -2.292565096 3.1159261374.560376523
4.7110677854.828178945 4.099895605 3.888096633 0.459243442
0.466594954 2.734634675 5.388432565 -1.35545703 2.293398407
4.585876369 3.1126696735.623635757 1.660042337 4.528933628
3.1143979625.055855321 0.994685137 -3.720754929 3.183544431 -
1.0861193413.161568486 -3.491227352 5.57833129 3.6511880321.631696294
4.707947734 -1.190173468 5.132745449 3.253684018 3.912155628
4.250786928 6.938174786 1.77224538 3.698915654 6.810646185 -
4.563160503 -1.850956152 4.492773705 -2.760921158 3.616969528 -

4.687677905 5.06805867 1.881293485 2.669896494 4.1844835 6.069466912
2.558783177 1.455142409 -1.948451393 4.70291014 3.92543564 3.87525997
4.013363428 -1.877229259 5.083056482 6.529207777 2.954576873 -
0.006602576 5.673539623 -0.355111838 5.921471975 5.128922957
4.657822346 5.344990203 6.323965675 10.29901921 0.797373151
2.659507184 0.744960267 -0.857828037 3.24668188 0.75581856 4.203862717
6.906427079 3.546267913 4.635091352 -1.938641055 1.784628277
0.037040567 3.253719635 3.792488622 -2.202435448 5.332141415
4.878546434 1.502172976 3.407449988 3.876395602 4.866798505
4.7323313113.404457377 5.335338072 4.961710303 5.015044557
4.007276345 0.670436952 3.834002835 4.543461691 4.789569339
4.77949444 5.792494217 5.448925456 0.849155146 5.449646976
4.579902823 2.078314706 3.880369553 -0.685459114 -3.706105967
3.041866656 -0.417503086 -3.54375321 -3.724850611 1.515702307 2.76915835
4.324195145 5.101848928 3.252286473 6.150045104 0.071866764
3.16628166 4.403420388 3.756150104 3.264438371
TCGA-93-A4JQ-01A -0.255583453 4.915674626 2.497434597 5.185070665
4.0133118088.2590280113.792866241 4.258696278 1.871089927 3.528855351
5.299190198 -2.428280963 3.270555616 4.347077798 4.929424994
4.792619865 3.823381788 3.947104728 0.310673633 0.436294056
2.683881606 5.317481396 -1.125417122 2.31759051 4.596789869 2.82144513
5.850159743 1.356658965 4.635403904 3.839793665 4.917673009
1.039346151 -3.532046786 3.189798257 -0.937638705 2.482491965 -
3.188082142 5.584893536 3.587156628 0.805964828 4.70954547 -1.030947155
5.020069087 2.783096236 4.007039415 4.259676943 6.976362814
1.589861735 3.733294687 6.935159903 -4.870158959 -1.747000817
4.791462339 -2.532291472 3.341352505 -4.73289586 5.158966525
2.223923256 2.170477076 3.998562465 6.023687217 2.616992364
1.831854134 -1.598765325 4.773150182 3.932921085 3.972736495
4.361346803 -1.252378552 5.094991282 6.435601057 2.843195597 -
0.061637902 5.854334572 -0.306929025 6.028332157 5.040354523
4.745045455 5.452036739 6.362008223 10.596611940.564413106
2.735428793 0.6403469 -0.52900698 3.579130364 0.94627278 4.225973511

6.898086709	3.4321199674.739110045-1.899100567	2.364323015	-0.46894137
3.254646187	3.913637634	-2.101963793	5.341256368	4.694728155
1.391259989	3.239203892	3.979136673	4.981294585	4.803693484
3.103049095	5.335574573	4.78043389	4.97715004	4.072008739	0.873322954
2.650872517	4.482625713	4.900804823	4.773689874	5.562015216
4.939990542	0.9324199	5.557906995	3.988933405	2.08993605	3.90568334	-
0.484047247	-3.977611423	2.942236541	-0.360905598	-3.619563575	-
3.6739113231.430481448	2.906983333	4.257096544	5.294962997	3.219208634
6.199566166	-0.15932703	2.909228406	4.378103946	3.654065476
3.075826279
TCGA-95-7039-01A	-0.740779462	4.7471112442.11983099	5.422762992	4.390101416
8.174723249	2.967688123	4.236384855	1.781749265	3.268329605
5.442219626	-2.562953219	3.8939757	4.547576289	4.72683535	5.194478645
3.5794498113.564844324	0.060955946	0.454541312	2.641034033	4.78324491
-1.350718236	2.025401592	4.520316703	2.936428978	5.319785513
2.02717114	4.491286939	3.644845951	5.08538723	1.155849665	-3.656965644
3.21737434	-0.971634776	2.28636973	-3.15019818	5.6131104393.536844392
0.968526629	4.705993788	-1.193405305	4.867647268	2.700919483
3.974226409	4.099238824	6.880291346	1.30851262	3.615663158	6.99507286
-4.939695282	-2.110103227	5.167958064	-2.56278441	3.186598443	-
4.924599391	5.206997074	1.786706228	2.513946139	3.633365962
5.958510077	2.510873793	2.051461108-2.533162011	4.59116634	3.932862703
3.784752079	4.425435533	-1.641456016	5.077053343	6.313828183
2.747903547	-0.023767155	5.531035698	-0.738615066	6.159227196
4.972140198	4.798273593	5.510032797	6.21154366710.67531294
0.998195898	2.719483701	0.495492892	-0.738626058	3.166420402
0.928651716	4.363466935	6.901769221	3.4127111215.002979998	-
1.915093177	2.286573075	-0.373750875	3.279392364	3.599251416	-
2.496992475	5.346082533	3.936535656	0.669072175	2.7115390933.797118425
5.014316273	4.779904173	3.0524215115.335390358	4.537269253
4.909449951	3.882081997	0.753269321	2.647672049	4.601460044
4.815449916	4.713516955	5.6535117794.719624098	0.974408422
5.546503452	4.034750988	1.862061345	3.882448572	-0.790766127	-

4.091005154 2.126614095 -0.09505596 -3.570083715 -3.610189326 1.654295482 2.708410511 4.409054999 5.127526063 3.21768944 6.12823225 -0.461228386 2.839094101 4.366701281 3.571244418 2.785627323

TCGA-95-7043-01A -1.212934132 4.967815832 1.441070566 5.440462294 4.606682683 8.219847416 3.672682352 4.116598633 2.457574017 4.104688696 5.162155693 -1.463749502 3.366183789 4.919309293 4.644086047 5.435715537 4.489808334 4.47943172 0.688762548 0.25550056 3.182124341 4.836162974 -1.403161494 2.072390854 4.728423758 2.763145424 5.526050312 5.069978646 4.733574185 4.915077345 5.023981183 1.595272346 -3.832526852 3.417655764 -0.811901981 2.652066849 -3.482983454 5.609026655 3.195388175 0.753943283 4.687639716 -1.147434508 5.080546887 3.730472111 3.74303569 4.251350687 7.104867747 1.679630866 4.424359115 7.261703917 -4.898199222 -1.194610248 4.61704052 -2.548686765 3.797970676 -4.10866681 5.131807344 2.319265265 3.05306577 3.943478265 5.935669719 2.893545269 1.914440943 -1.05075125 4.418340953 3.92750108 4.787661111 4.274666259 -1.174568291 5.062660587 6.533028647 2.954018052 0.138220045 5.536960583 -0.788009718 6.124122549 5.052973919 5.012195485 5.39205108 6.411458916 10.25769121 1.071614402 2.732990923 0.960868055 -0.743578815 2.821249945 0.56615115 4.410369002 6.891826299 3.693897685 4.937584207 -2.18312203 2.792361165 0.776779149 3.443147731 3.962646778 -2.283745867 5.347353566 4.324452407 1.344877976 2.796546841 3.911992057 5.01425858 4.41553896 4.092448897 5.335284548 4.864280649 4.974470306 4.733911179 0.467747038 4.563193431 4.427756018 4.678912589 4.729395703 5.862973696 5.605282367 1.336549449 5.454398955 3.844620638 1.534620156 4.044422158 -2.13770228 -3.423129617 3.144410103 -0.51956716 -3.731408486 -3.315207812 1.43074175 2.412824652 3.994353272 5.73451466 3.771870825 6.30848315 -0.578240053 2.986054407 4.720977819 3.612224207 2.184864959

TCGA-95-7562-01A -0.871667785 4.725166298 1.380841244 5.440776112 4.70164735 8.300737738 3.349640176 4.218287578 2.24254173 3.824135735 5.238673054 -1.98062761 3.816657642 5.238188493 5.095431795 4.982659853 4.402948439 4.158149486 0.733045298 0.527738484

2.771763098	5.589183675	-1.332807664	2.678994186	4.668634939
2.442450442	5.7303118332.768246558	4.725572183	4.526577663
4.981463612	1.673796531	-3.695884923	3.725825726	-0.503016976
2.872618293	-3.702560535	5.6091176253.513570193	1.467042497
4.700072566	-1.240385199	5.432663948	3.563266399	3.763412323
4.320905598	6.977770892	1.663848144	4.12835035 7.120322106	-
4.998580788	-1.651826805	5.067761701	-2.291719913	3.918174964	-
4.195829737	5.302609526	2.1193176043.329225347	3.990662253	5.921744527
2.466095575	2.148102217	-1.986480889	4.601264534	3.939142929
4.315415186	4.385532978	-1.776147849	5.063139477	6.553501826
2.8428011330.271709062	5.532319457	-0.633907129	6.102424262
5.0164701124.928674556	5.409920896	6.447898617	10.3483654 0.577795233
3.230413064	1.714277008	-0.495075938	3.038680881	0.835498194
4.262948492	6.904502573	3.418706296	5.349552315	-1.782306456
2.656887587	0.283689289	3.395185558	3.888018851	-2.116418895
5.329904656	4.327974389	1.303322715	3.643805289	3.207319972
4.976245032	4.8282583 3.625468752	5.335289602	4.467845241	5.14518811
4.379432266	0.608640704	3.649924014	4.566865272	4.689916173
4.765854049	5.757442448	4.791287632	1.235861594	5.218213649
4.360934254	1.780279618	3.944101216	-0.495886348	-3.08384978
3.367796308	0.063585016	-3.667419078	-3.119018056	1.454204824
2.545792017	4.390521922	5.567518599	3.552121638	6.431885201
0.19518535 2.419394746	4.68569139 3.733248683	3.044978693
TCGA-95-7567-01A -0.828874344	4.741918294	2.369123403	5.504294735
4.329684487	8.237867104	3.320047471	4.286005352	1.650045411
3.222871591	5.861045253	-2.315754609	3.652127668	4.438088349
4.7221156685.053691267	3.858312126	3.969826714	0.05862475 0.436090236
2.607325848	4.852620396	-1.351754261	2.275576186	4.542586817
2.916459486	5.6601157182.260370535	4.510004531	4.166738425
4.978089412	1.131674185	-3.634388525	3.328372987	-0.859952688
2.444882982	-3.267125473	5.6081107393.618195966	0.391901966
4.694823566	-1.218222158	5.1614541182.861630164	3.932255355
4.186427851	6.821970182	1.31670839 3.5935464116.914054975	-5.03281644 -

2.01812386 5.055487914 -2.560171863 3.169738078 -4.923585564 5.074029393
2.170773582 2.04816041 3.827517232 6.01016223 2.667844795 2.47653711 -
2.1789171194.598445774 3.937445493 3.934315405 4.359973198 -1.530921695
5.083099532 6.242921495 2.75206419 0.016126832 5.796730498 -
0.835276893 6.190982205 4.989918495 4.810384466 5.548635256
6.40102113610.63444125 1.078491022 2.721087214 0.754672124 -
0.668063796 3.171016998 0.990851014 4.422004067 6.841568359
3.595654936 5.006446382 -1.953124321 2.621282826 -0.612812373
3.294559499 3.686477321 -2.51729039 5.3624318 4.040563089 0.639890615
2.928334917 3.971571954 4.994756534 4.748958933 3.240644322
5.335509992 4.727995669 4.917926018 4.019956242 0.701830031
2.809157126 4.522759604 4.798403965 4.703280005 5.781061612
4.908360545 0.922573517 5.663501453 3.752239239 1.687537376
3.907627935 -0.636885804 -4.350509851 2.330071344 -0.179901287 -
3.660635891 -3.851153982 1.491548258 2.732937305 4.473068049
5.193751877 3.378725597 6.037076902 -0.103437888 2.902906139
4.422724135 3.513598525 2.642352878
TCGA-95-7944-01A 0.45176718 5.262185521 3.149226829 5.412308342
3.661589132 8.325042868 3.406582055 4.438794264 1.950779619
3.6584091175.646447785 -2.276177742 3.631884584 4.210997791
4.799912869 4.996648524 3.862895905 4.1138110960.165586744
0.381049209 2.629352614 5.008268614 -0.992867598 2.393740663
4.584003804 2.598027763 6.120786645 3.225617657 4.640260579
3.865877839 4.834810757 1.210193326 -3.55666355 3.058486457 -
0.992453669 2.025776435 -2.676087118 5.591875628 2.636712233 -
0.400438547 4.699628214 -1.09986906 4.963035419 2.618481443 4.034895224
4.28595924 6.677124323 1.19977347 3.969547456 7.129361448 -5.388702639
-1.69713548 5.170102225 -2.598140949 3.441703448 -5.027335001
5.162902324 2.444409229 2.175640082 3.780014627 6.117869514
2.343618332 2.08877621 -1.346244501 4.791005995 3.925863062
4.2208115694.308581948 -1.381143976 5.093763741 6.4735831162.787905705
-0.223857767 5.777820107 -0.379449725 6.219238063 4.987476093
4.970073682 5.462410216 6.33341253 10.32866816 0.891307762

2.650870556 0.626114527-0.006434601 3.451350594 1.162814941
4.358329325 6.9152111073.3417281134.860955104 -2.088067625 2.479434939
-0.82002052 3.28993376 4.177145077 -2.067542489 5.345318734 4.291313888
0.640026887 2.972580175 3.807848877 5.091506299 4.777442787
2.708944651 5.335762803 4.881675308 4.939897159 3.886800288
0.937247944 1.675355236 4.539951815 4.974945635 4.72671401
5.550295926 4.975144496 1.186756612 5.389669556 4.007356989
1.85977542 3.862335986 -0.549764467 -4.128165724 2.366516968 -
0.727086624 -4.073507458 -3.371850787 1.454838019 2.973502582
4.406737469 5.1165271262.960035163 6.211156042-0.835043211 2.937247126
4.458503936 3.548672556 2.58255059
TCGA-95-7947-01A -0.480086108 4.96738149 1.676481623 5.321291718
4.7093115368.19808264 3.074655372 4.156781313 2.049376494 3.318328271
5.32306832 -2.139419049 3.253460999 4.982877755 4.871362196 5.23922996
4.606038747 3.713047483 0.189803166 0.4412695 2.930697185
5.092867808 -0.871133813 1.956677716 4.664789852 2.777715036
5.568372729 2.919932539 4.655807273 3.95615792 4.972226219
1.151691067 -3.685804831 2.923738221 -0.976099604 3.162442333 -
3.449678429 5.588512805 3.8114005441.1183774794.685653633 -1.233122591
5.242837214 3.666358262 3.880404281 4.288540148 7.021570412
1.820007951 4.638635298 7.21007036 -5.049233632 -1.837431961
4.895535139 -2.769339598 3.66839638 -4.752522443 5.213817506
1.866367514 2.808585363 4.1139537946.023527581 2.494829199
1.786852558 -2.309925659 4.528333022 3.933577071 4.216916656
4.308640449 0.493075839 5.0640360116.352815272 2.940095989 -
0.148338316 5.552041837 -0.376503866 6.1611077755.1147674684.805119775
5.424348007 6.368676716 10.03615316 0.443507609 2.915274457
0.947110515-0.734636629 3.018638387 0.776512559 4.246375168
6.857900335 3.618081964 5.23711405 -1.874216049 2.575255969 -
0.017121853 3.298550508 3.56227607 -1.305874638 5.350513264 4.209323083
1.374092924 3.391681053 3.834859917 4.918148738 4.719671158
3.508321337 5.335137054 4.78748421 4.9949453114.05279556 0.763830704
4.277536962 4.7011664974.768741865 4.752730264 5.992251747

5.318628623	0.708565631	5.602910012	4.3181418111.971945437	4.03943806
-0.665247524	-3.174067538	2.47416079	-0.118765582	-3.851116495	-
3.937680209	1.344731662	2.8322911844.500757945	5.888808827	3.644420053
6.427490975	0.322288575	3.036675474	4.699376047	3.684330096
2.799655551
TCGA-95-7948-01A -0.671251271	4.915689444	2.6539119085.441754641
4.461043758	8.1135620993.481721641	4.185191056	1.9216711162.976545052
5.199425656	-2.193353778	3.033664383	4.44946599	4.658323571
4.913028719	3.819936562	4.10431114	-0.474752898	0.370697993
2.6997177115.014938577	-1.358556739	1.853800771	4.580956485
3.050915628	5.357502007	2.458251918	4.567186667	4.142776156
5.101576089	1.175369806	-3.61968559	2.686105181	-1.0652456	2.827072305
-3.601439703	5.59974792	3.6011725170.244316863	4.705380833	-1.159095698
5.035219588	3.010381591	3.873246958	4.073523246	7.063521632
1.259077994	4.305649588	6.894560387	-5.273270624	-1.848818304
4.804815831	-2.996560664	3.451751609	-4.833113833	5.139231958
1.997898247	2.3912931173.824790727	5.863252031	2.438907927
1.751837764	-2.856491197	4.648636108	3.934152264	4.148582148
4.195986133	-1.827116714	5.08253922	6.363567145	2.884267002
0.037900087	5.592155424	-0.838703201	6.193380599	5.035790052
4.893873726	5.424454643	6.357399809	10.72907103	0.1126786092.60899049
0.775998522	-0.763599541	2.446972949	0.855860082	4.333131886
6.977189675	3.602665796	4.751877098	-2.079390217	2.295791827	-
0.472184329	3.289524876	3.780822816	-2.351787802	5.366718063
4.108572984	0.437136227	2.4746811223.753035181	5.067382224
4.826302525	3.324223591	5.335513303	4.660745469	4.803816398
4.216900736	0.694555669	3.337781572	4.464243494	4.851967814
4.71669052	5.708747537	5.099337576	0.956679797	5.397906013
4.082802044	1.734150107	3.907443349	-1.476946179	-4.293005676
2.646685786	-0.281504182	-3.937166887	-3.97128606	1.380902074
2.759471026	4.368683037	5.459516951	3.5114076696.510397451	-
0.377267883	2.983409642	4.429365896	3.7089114992.173735388
TCGA-95-8039-01A -0.5581539	4.610520022	3.033404599	5.447423379

4.251331066 8.275205402 3.345328098 4.347304509 1.689519882
3.186284982 5.602956755 -2.380182562 3.566453386 4.362408848
4.735618206 5.086164099 3.465828768 3.950775291 -0.006314259
0.428754935 2.701902928 4.996755711-1.1332502 2.284756545 4.524727236
3.072712965 5.698690418 2.351366187 4.548738134 3.567830014
4.951650638 1.155401391 -3.44178555 3.231625615 -1.054073704 2.7471344
-3.163359585 5.601265618 3.428145263 0.449562529 4.711885458-
1.164079023 5.358182392 2.569341712 4.046434532 4.236606243
6.966149906 1.287061332 3.590397981 6.707012402 -4.848027047 -
1.924353921 4.68402963 -2.551101689 3.346520413 -4.663716735 5.023818102
2.089422804 2.448917655 3.8905402116.1384774 2.601620178 2.251233148
-1.940521122 4.644673071 3.934580639 4.038958336 4.217206795 -
1.179935648 5.093410404 6.320824437 2.87125816 -0.099273456 5.963991382
-0.429444244 6.045296743 5.049176442 4.684587641 5.550208561
6.316661053 10.51353995 0.846161947 2.646063539 0.764951412 -
0.689120931 3.165637876 1.030588024 4.339504832 6.845420762
3.620099273 4.809389716 -2.105134211 2.253096195 -0.306038779
3.298641073 3.710516787 -2.334771122 5.350607271 4.457612149
0.6365755 2.963264629 4.080642382 4.9949651174.814724086 3.219564611
5.335579339 4.941226364 4.96413881 4.193906127 0.73994103 2.546942072
4.637944236 4.858155805 4.736720009 5.72500158 5.321023185
0.894612305 5.754972637 4.000696081 2.089129629 3.855981009 -
0.468917367 -3.855375269 2.900560455 -0.202076217 -3.603533145 -
3.482547947 1.72341512 2.837206448 4.629214028 5.17413702 3.253553854
6.040704059 -0.320576249 2.954456129 4.443551428 3.65857623
3.040313572
TCGA-95-8494-01A 0.133540454 5.0441421182.668620042 5.440741518 4.25318528
8.059664661 3.314777697 4.265931386 1.78884494 3.233885365
5.565284651 -2.32361281 3.874485763 4.5611947655.081745278 5.164155346
3.178895646 3.980269068 -0.243834878 0.521645446 2.778208915
4.873572223 -0.893754943 1.776898696 4.587412349 2.899017608
5.284835039 3.529838402 4.633512331 4.299526909 5.005021106
1.380886486 -3.187738624 2.955174055 -0.837980582 2.616453703 -

2.769248697 5.625039194 3.024222755 0.306669945 4.721598324 -
0.97848414 4.929570871 2.2931128324.034741491 4.137847222 7.063011378
1.371880006 4.233481723 7.113656947-4.814236771 -1.969589901
5.115787936-2.245990293 3.273546541 -4.30824814 5.325887715 2.508399573
2.9129911843.53411347 5.918885069 2.549706029 2.017334791 -1.938616349
4.664934932 3.940231808 4.29151602 4.506391144-0.282897051 5.094028472
6.276049042 2.737496345 0.0111873215.385924153 -0.344848677 6.28409876
4.8425119465.003084274 5.528212598 6.281541378 10.78481676
0.566627278 2.992995413 0.967589721 -0.29549959 2.972615294
1.210627867 4.364822355 6.881502793 3.34265748 5.004315629 -
2.021463748 2.818104469 -0.030244957 3.307267136 3.79035902 -2.139570709
5.35449292 3.70372233 0.30639511 2.554496138 3.689494034 5.128019004
4.934581855 3.100999455 5.335834092 4.149149584 4.99783605
4.361810774 1.02613521 2.351200692 4.608974038 4.98649095 4.733232564
5.492512847 4.568498729 0.986627767 5.123616622 3.997288681
1.973221768 3.977462719 -1.030368675 -3.441699289 2.88807153
0.037462308 -3.791490676 -2.825189592 1.6861157532.918708156
4.558068526 5.751513838 3.288060195 6.315985993 -0.55366764
2.687738661 4.508049004 3.664771468 2.91047801
TCGA-95-A4VK-01A -0.795279984 4.773561502 3.07890814 5.156747097
3.988241002 8.24603629 3.247037685 4.278536602 1.78286436 2.970809562
5.138632615 -2.649879221 3.325767429 4.655702521 4.6311742234.85361654
3.592164029 3.7011737420.254371728 0.373738182 2.693231467
4.923807535 -1.339024779 1.262244778 4.55608333 3.093534535
5.722509165 1.724978003 4.453106317 2.8001109344.873771939
0.701686347 -3.739786421 2.853582571 -1.601296018 2.918714217 -
3.229622372 5.586970642 2.975466644 0.583309263 4.701296013 -1.118987
4.862642925 3.029632725 4.067282789 4.238795755 7.019464382
1.434949459 4.052135883 6.643184092 -4.797841639 -1.93079252
4.794586782 -2.448498782 3.248150864 -5.198722256 5.196679103
1.950454423 2.1159189263.91968628 5.888277464 2.545367719 1.690927006
-2.388220372 4.582449277 3.93031269 4.030326614 4.243050032 -
1.653190242 5.076045434 6.268337666 3.055858835 -0.208161618

5.712363241	-0.711095945	6.105408934	5.128432882	4.906929742
5.458582613	6.329573669	10.113032140.754246185	2.518802908
0.380189346	-0.954637098	3.275936831	0.781217739	4.219771843
6.860669336	3.643819827	4.675568571	-2.009911334	2.024720164	-
0.424659142	3.198570274	3.604931951	-2.432367497	5.351648598
4.277949986	0.961554562	2.731717163	4.1727607114.971304846
4.714129781	3.44732211	5.335381038	5.065738887	4.873767298
3.788624929	0.688261855	3.1252396114.665244697	4.797802052
4.707829212	5.652505307	5.150866678	0.77521524	5.707993654	4.16126701
1.723290707	4.008506074	-0.548377343	-3.981127355	2.5106514	-
0.117818769-3.489380904	-4.199847117	1.43483293	2.730202909	4.186944485
5.449654971	3.2691163086.278397757	-1.068951377	2.948313939
4.607666831	3.718060177	2.740720244
TCGA-95-A4VN-01A	-0.113355806	5.000189007	2.782923628	5.426989718
4.1785117818.2211239243.383037574	4.378381807	1.671373773	3.414848661
5.441351671	-2.3072306	3.753366634	4.475888268	4.872898909
4.873613168	3.501753466	3.829172005	0.326827957	0.43261007
2.575383206	5.210185374	-1.052892558	2.174129078	4.559207366
2.78345893	5.776864939	1.514884313	4.593997133	3.907859775
4.931288638	1.033394684	-3.476957153	3.309270483	-0.794701155
2.23493786	-3.247269043	5.606520399	3.46200252	0.5011366794.701202039	-
1.12022828	4.80470767	2.5813118774.022743084	4.273038433	6.746242565
1.32711081	3.773075006	6.988307121	-5.031107596	-2.001547842
4.957682879	-2.17528444	3.356332756	-5.023778538	5.146137233
1.920662244	2.09284651	3.767486958	6.01124687	2.367477507	2.273787965
-1.994202293	4.721489386	3.934862472	3.7437970114.527371963	-
0.96005134	5.092310687	6.343724165	2.753106429	-0.15059711	5.876937886	-
0.384700084	6.17604741	4.928530477	4.735069509	5.564973685	6.356747798
10.54310833	0.221997828	2.796124572	0.637750225	-0.552533486
3.523592392	1.049379122	4.31483249	6.817551596	3.284956516
4.919750255	-1.900352512	2.542913943	-0.383796554	3.250944583
3.741164665-2.059045966	5.356059067	4.408790455	0.9088181143.035044559
3.600053837	5.034637741	4.845609895	2.945921273	5.335636012

4.533502092	4.969603122	4.122944888	0.797818406	2.017573695
4.65716564	4.891701903	4.745630632	5.540606285	4.735604991
0.871609607	5.555544487	4.126188645	1.973438731	3.847659752	-
0.321931132	-4.249419558	2.68849052	-0.345728653	-3.6838316	-3.892913751
1.576107584	2.887830082	4.449663087	5.315711063	3.231978728
6.140649518	-0.866811342	2.679719579	4.285928988	3.610353577
2.785079363

TCGA-95-A4VP-01A	-0.538274494	4.884761248	2.476599667	5.195134505
3.708487583	8.309034557	3.907893184	4.275780739	2.126841171
3.558210068	5.505036414	-2.098525216	3.941693569	4.140149079
4.991267963	4.926949982	3.765127854	4.572372389	0.317893435
0.509562877	2.943331227	5.21456606	-1.19844173	2.560706654	4.596838582
2.897154029	5.943348044	2.795262881	4.674294359	3.795680175
4.902645326	1.276975671	-3.352452149	3.403817387	-1.255656961
2.621451125	-2.895139723	5.581186947	2.847457058	-0.09673342	4.719456105	-
0.959382442	5.102031344	2.895272716	4.076796882	4.363310406
7.009357072	1.757077105	4.13490402	7.077583546	-4.634116261	-
1.45780241	4.240072465	-2.139760606	3.457891631	-4.231978792	5.29043289
2.61855179	1.929346988	4.130745692	6.140139658	2.927357496
2.201586163	-1.15238105	4.672622167	3.920870239	4.619740978
4.308080071	-2.285069656	5.091788743	6.743017724	2.975050618	-
0.066686603	5.999269183	-0.344091185	6.105030455	5.096676163
5.105203775	5.555233051	6.365375159	10.06681529	1.048461215
2.736754203	1.189858498	-0.531162573	3.721202084	0.733271117
4.339385442	6.959077401	3.675574884	4.68248797	-1.85697639	2.36734897	-
0.301308789	3.343971023	4.027312204	-2.709637995	5.339852216
4.533212279	1.450304101	3.226079678	3.952194366	5.078904637
4.583433605	3.523230675	5.335854292	5.234661169	4.894033762
4.480144116	0.916902463	3.40118014	4.690742287	4.814413397	4.694608172
5.88465299	5.192172161	1.549678516	5.342554532	3.683048273
1.784930238	3.989392183	-0.725760331	-3.414728709	3.200885517	-
0.332111972	-3.504110655	-2.843703191	1.665525343	2.941450606	4.417424659
5.473530336	3.014394889	6.20567721	-0.848568386	2.677136741

4.596404785 3.938801824 2.631563129
TCGA-97-7546-01A -0.7971042 4.389468844 2.380669927 5.39447003 4.250547592
8.391368256 3.608608133 4.278826941 1.950901422 3.644026082
5.185035756 -2.227917244 3.038924792 4.885314272 4.811089182
4.673053932 4.0143511923.810717903 1.065008121 0.56103137 2.657492471
5.709399335 -1.582827013 2.840283948 4.602347208 2.858910909
5.667394488 1.783614554 4.423606455 3.074282325 4.75047475
1.153080911-3.786712213 3.814070325 -0.85129212 2.717541944 -3.584219481
5.57236267 3.917205173 1.871427196 4.698415064 -1.334064996
5.346432632 3.441363833 3.892787129 4.267036034 6.825094754
1.567834608 3.487580477 6.737328322 -4.738900396 -1.550162023
4.454489823 -2.593612672 3.617601437 -4.205476622 4.933077718
1.957151598 2.990690968 4.176638706 6.046461915 2.826459189
2.092958937 -2.134924053 4.684334338 3.941798645 3.971331779
4.306654715 -2.163106463 5.084293027 6.322759658 2.844706218
0.15814009 5.977727464 -0.499344698 5.869169087 5.217740653
4.410549057 5.375847727 6.520961728 10.68977207 0.679509469
2.796089686 1.032433095 -0.671210012 3.568602934 0.839703634
4.185944863 6.837093655 3.583025445 4.748479077 -1.881044563
1.907756029 0.171025986 3.286586173 3.844724734 -2.340135681
5.32609577 5.189364671 1.559487691 3.504978198 4.127854869
4.846449417 4.763538694 3.200828742 5.335243377 5.118651341
4.988665708 4.099106303 0.409714565 3.451563876 4.423807643
4.63520969 4.805817204 5.537480758 5.169604135 0.776609723
5.687772802 4.454945151 2.10538643 3.737661529 0.103623978 -
3.547999447 3.448405133 -0.377546155 -3.420447874 -3.435284752 1.45594493
2.619477931 4.262859889 4.870138136 3.109427469 6.083020016
0.5541881113.181215042 4.374846446 3.663936163 3.26081426
TCGA-97-7547-01A -0.694871216 4.684050736 2.766328425 5.573550216
4.606705495 8.315326145 3.190927574 4.345752336 1.784474148
3.030127544 5.555140507 -2.230301515 3.476929738 4.537287423
4.522038064 5.47649717 3.728576372 3.679240251 1.045251684
0.728702601 2.763548605 5.163539218 -1.119081094 2.358582497

4.483731635 3.406070242 5.328633446 2.98851818 4.528344658
3.035264341 4.995052247 1.003361189-3.713230139 3.964784635 -
1.077340699 3.315313182 -2.677681172 5.60156497 4.277333797 1.487508411
4.718335963 -1.204247627 4.933449502 3.201462299 4.130172333
4.400743148 7.022219634 1.539535775 3.621875779 6.681482597 -
4.364149914 -1.603222118 4.542418665 -2.316389825 3.074407715 -
4.219071804 5.03433548 2.149358345 2.239644652 3.961805739 6.137557913
2.93963101 2.113519622-1.967305235 4.544551052 3.940827313 4.332295854
4.367096684 -1.198767573 5.076550413 6.347217917 2.972664683 -
0.25746373 6.132723384 -0.510100788 6.06489138 5.303651854 4.517655419
5.550383904 6.260535657 11.151569361.047757619 2.523738462
0.665070861 -0.746408995 3.301865625 0.96361582 4.346533238
6.808731406 3.957920362 4.572620264 -1.941734877 2.21012252
0.106865602 3.318339552 3.460411309-2.110727093 5.367368956
4.619148831 1.212781466 2.931229789 4.419886264 4.985644282
4.791623182 3.121552992 5.335449518 5.162527443 4.82673981
4.6372191140.502639081 3.622631366 4.53935603 4.690938086 4.737923296
5.7811304595.399721665 0.700049944 5.954994439 4.368302153 2.02038184
3.882569248 -0.273108478 -3.503502283 2.886874584 0.032470128 -
3.033081649 -3.318461159 2.089760963 3.108437295 4.770784071
5.125393946 3.433877891 6.0911458040.445398632 3.656998946
4.462658026 3.769199339 2.725662308
TCGA-97-7552-01A -0.69151622 4.782406271 2.443717449 5.334927262
4.218519486 8.349574285 3.346749359 4.342230675 1.703193134
3.240501235 5.308342927 -2.591674165 3.403839659 4.607220558
4.727137847 4.93047349 3.69184317 3.4529411270.950321443 0.583594396
2.540279233 5.163937677 -1.300696261 2.328157878 4.496201818
3.008790007 5.860829864 1.297920773 4.475895455 2.838204623
4.724585817 0.553740425 -3.72538387 3.528783209 -1.270726463
2.747068784 -3.213016691 5.575017832 3.900991885 1.267303028
4.716371253 -1.159483929 5.037651394 2.787163906 4.077365223
4.380518138 6.993943734 1.493833807 3.387254022 6.476215778 -
4.599312906 -1.914554986 4.568896331 -2.468175832 3.25571645 -4.910470812

4.985973849 1.828730817 1.960803864 4.065459335 6.156425731
2.882593835 1.635839597 -2.678993057 4.678686645 3.938755159
3.5297262 4.364537075 -1.599689806 5.093444596 6.239636374
3.017099156 -0.229988876 6.235835492 -0.072261484 5.912039926
5.3311539444.219806897 5.440063797 6.089521359 10.59416572
1.173900967 2.590033864 0.522532855 -0.831834983 3.65098157 0.95709649
4.253398862 6.792301796 3.7241388114.614900042 -1.945433284
1.874867022 -0.455379971 3.183740457 3.514198113-2.495075049
5.348151781 4.908042822 1.278234653 3.303168556 4.513788716
4.855204437 4.760797366 2.587883773 5.335298664 5.245211476
4.840990933 4.049736873 0.484395402 2.753418763 4.434645626
4.672699176 4.816270994 5.622555147 5.158867403 0.693043357
5.789468637 4.738172843 2.173852252 3.798050108 -0.29919446 -
4.1170163982.707447774 -0.120814271 -3.194250089 -3.745661289 1.833706518
2.896442099 4.4412002 4.945703623 3.44529133 5.901774883 0.486770826
3.331271049 4.40871773 3.737919944 3.289286396
TCGA-97-7553-01A -0.389290113 4.644947753 2.379516289 5.34772199
4.204134386 8.290838461 3.378364697 4.287569222 1.707000248
3.505098419 5.154677814 -2.440384246 3.486720485 4.642344524
4.830931996 4.900814931 3.783100808 3.604266076 0.43891941
0.518697361 2.5880996 5.429145242 -1.270605393 2.751931859
4.558946364 2.987442942 5.807513349 1.2811638264.4801130373.066706147
4.853236441 0.921601063 -3.625575824 3.388192199 -1.017224776
2.483486007 -3.367622628 5.570024913 3.424159029 1.205987444
4.70581089 -1.250137234 5.27528867 3.017856548 3.973610405 4.315835816
6.8862911641.5779685113.423938453 6.828403921 -4.888260257 -1.935403989
4.495978469 -2.72468579 3.659872078 -4.903815941 5.044927286
1.648885534 2.53815193 4.101661303 6.1921761142.67818499 2.010801831 -
2.38952336 4.762049302 3.9327611653.677751379 4.246783466 -1.638811314
5.09019341 6.38338257 2.837416786 -0.058589721 6.000314806 -0.129956938
5.9469291185.149688954 4.4488211835.446193497 6.329390254 10.63044481
0.604304861 2.735885178 0.543833692 -0.751064326 3.748197437
1.006405176 4.223588728 6.84993686 3.433099425 4.769105664 -

1.879067765 1.942609436 -0.297062466 3.229318646 3.796302875 -
2.120209668 5.3391168015.1132606661.528867557 3.356219927 4.048618856
4.9115504984.703668184 2.7911607285.335378317 5.013499538 5.038427425
3.960554773 0.713408542 3.132671626 4.685165979 4.784842762
4.79646648 5.588076721 5.2133648110.828051013 5.655997764 4.531240109
2.177146582 3.765855183 -0.271430947 -3.783365624 3.002162377 -
0.331351703 -3.563791582 -3.732342676 1.642313775 2.830405143
4.461074404 4.9641164313.155196546 6.009527776 0.167193927
2.977653673 4.3113766343.672752554 3.355956248
TCGA-97-7554-01A -1.024336891 4.319887222 1.996568059 5.475187452
4.29733404 8.3451168873.091360692 4.3118909841.577638542 3.328427729
5.433074506 -2.605205055 3.374083181 4.69024654 4.644452638
5.130140293 3.667023987 3.425939233 0.595833975 0.513639594
2.530944337 5.146584922 -1.529470065 2.690240761 4.552254883
3.057572167 5.474486297 1.614491208 4.439577992 3.222131368
4.987917149 1.093631785 -3.856565185 3.797440233 -0.800678387
2.477750263 -3.14208486 5.599060745 3.919007855 1.552282203
4.711065349-1.331137182 5.283647304 2.885281826 3.9495841154.203480753
6.871827261 1.413378965 3.155609573 6.610131111-4.564574921 -
1.924433985 4.83238913 -2.594723711 3.169474823 -4.719191374 4.934261856
1.8501204112.462438193 3.825364991 6.073462143 2.576943096
2.245537757 -2.357556856 4.626191666 3.942235704 3.700784972
4.222526234 -2.049292956 5.081891684 6.230300529 2.794507442 -
0.101785866 5.751207429 -0.763628836 5.987462537 5.09879672 4.487386184
5.477016271 6.385873064 11.120185261.111411243 2.68286159 0.571309197 -
0.705717209 3.292452367 1.032765675 4.305362915 6.82785496 3.496036332
4.811502107-1.99143884 2.108913479 -0.324018948 3.29499738 3.654889915 -
2.615364355 5.338712203 4.613703173 0.988789406 3.188951928
3.881366896 4.912106247 4.799345497 2.996662698 5.335375729
4.748301908 5.043687827 3.967729644 0.480972153 3.073392075
4.487376133 4.749792671 4.765923462 5.5582911544.986961048
0.669349637 5.77147654 4.305297204 1.914473683 3.835062318 -
0.278167368 -3.912132675 2.790636757 -0.090705563 -3.34124378 -3.754452962

1.694451734 2.75520735 4.44585335 4.798897842 3.0418119175.892379649
0.59373923 3.20816005 4.360828608 3.506521377 3.131408468
TCGA-97-7937-01A -0.485486972 4.738158313 1.800403037 5.329951022
4.735837507 8.222280325 3.276230004 4.13214722 1.929212438
3.335785348 5.258922113-2.245043528 3.445893073 5.1108473484.754548782
5.198228664 4.077801313 3.680156283 0.686407651 0.562041718
2.733437616 5.501203852 -1.22071369 1.795074042 4.682399024
3.015309852 5.333863712 2.270525436 4.481549508 3.88183693
4.924760432 1.285803192 -3.765085236 2.846840908 -0.754551675
2.838467588 -3.189946817 5.606506415 3.730534481 1.884071942
4.693326196 -1.333084527 4.971980301 3.422538584 3.841426638
4.202533045 7.007036762 1.622082912 3.86819982 6.997023478 -
4.651671272 -1.696700123 5.370914257 -2.420492795 3.533996452 -
4.282865304 5.142810031 2.340533338 3.068361903 3.968099484
5.876940362 2.548747281 1.332307854 -1.783983663 4.569008632
3.944249844 4.13877046 4.296947992 -0.551469388 5.062227474 6.2367717
2.8449362110.130773657 5.316520157 -0.668663454 6.1173431485.051184052
4.802353553 5.347173744 6.462941639 10.96063835 0.631914028
2.993824273 0.744758426 -0.785681764 2.869336301 0.886878827
4.294107083 6.863920123 3.516173864 4.983035464 -1.853675521
2.458386579 0.1167746933.278104469 3.65521539 -1.759842572 5.349766726
4.396659795 1.028764632 2.838724605 4.004171615 4.922184629
4.82212923 3.453302966 5.335055272 4.443537762 4.977186482
4.194134153 0.623010949 3.508761072 4.4024711424.7115995184.755332398
5.586467818 4.854613653 0.792323778 5.533300043 4.288240027
1.947433419 3.921065405 -0.606343704 -3.138840782 3.100710708
0.038217017 -3.388633979 -3.439370226 1.491269532 2.655533828
4.242793272 5.424245801 3.666123863 6.308932465 0.036870915
2.846032595 4.519414825 3.596686273 3.136148897
TCGA-97-7938-01A -1.270823068 4.2171129852.496832008 5.469961544
4.533622688 8.412754459 3.00796025 4.302525732 1.382919647
2.951362418 5.375563577 -2.495137465 3.099174305 4.641985316
4.391601436 5.0572641153.5114485133.148513731 0.78723764 0.540424606

2.4161511035.292781729 -1.652430434 2.358246306 4.518785224
3.044906255 5.45540797 1.181802615 4.271638559 2.571241064
4.749300669 0.713665271 -4.078213375 3.592794243 -1.041253506
2.882601391 -3.3506501 5.592340325 4.173062051 1.48098144 4.700317124
-1.442258703 5.207714185 3.175672093 3.95135347 4.240930696
6.882238791 1.301688812 3.096748324 6.199707192 -4.674600188 -
1.914337757 4.602779974 -2.711539821 3.091346544 -4.801064261
4.805496739 1.417619427 2.375055961 3.993975441 6.049852148
2.715228805 1.896309635 -2.948883058 4.529410277 3.940126927
3.668642512 4.079225339 -1.871208238 5.074867252 6.15253444
2.926579236 -0.171563334 5.949765131 -0.920308338 5.947982868
5.254716075 4.248847031 5.427239421 6.478821661 10.88611458
0.963657386 2.437366883 0.335146091 -1.029991017 2.912573104
0.876950789 4.300101616 6.792343017 3.655749463 4.657034844 -
2.043768241 1.67259846 -0.237355383 3.213029225 3.481572451 -2.594525804
5.348326051 4.831028309 0.687492584 2.994922813 4.330593632
4.813464849 4.81481625 2.965106971 5.334984447 5.125099552
4.832736355 3.967624841 0.172881857 2.8116730464.378310316
4.595833486 4.772972316 5.603825343 5.295306168 0.491240157
5.922348039 4.497506418 1.973728883 3.732929724 -0.211258269 -
4.272776421 2.69575879 -0.185301187 -3.182140645 -4.02819483 1.765882032
2.485705819 4.384388284 4.773957344 3.212266169 5.810028552
0.063736878 3.192259782 4.201912164 3.507363536 2.92277582
TCGA-97-7941-01A -0.474498252 5.071339703 3.206914188 5.338817905
3.948488709 8.237221563 3.519175537 4.303500569 1.889796355
2.910592952 5.423228663 -2.433627278 3.518932006 4.148792578
4.826225843 4.966432323 3.540953325 3.994424542 0.401066104
0.455147287 2.70903434 5.010436574 -1.179080446 1.913150934
4.496384563 3.060052098 5.841003261 2.335804393 4.554499808
2.9455611664.897188881 0.711527293-3.533997452 3.252313735 -1.504801943
2.761612389 -3.033912986 5.577072012 3.100729374 0.52524587
4.724106504 -1.012230858 4.708861635 2.545767945 4.119098816
4.297802032 7.008577815 1.491315764 3.810883341 6.787157126 -

4.710053852 -1.797618732 4.344040982 -2.450215416 3.182762543 -
4.978658313 5.212445237 2.169745501 1.8391051183.93418569 6.085305507
2.606415623 1.495285985 -2.067097009 4.740150983 3.928184423
4.063057994 4.325046111-1.900639446 5.101369454 6.454996863
3.067247838 -0.24510565 6.196236464 -0.282377687 6.069251601
5.172015631 4.633741641 5.540302219 6.096403271 10.11227519
1.058572424 2.485734035 0.527961829 -0.64975506 3.2388393 0.904139384
4.359180339 6.904386032 3.615463674 4.535351764 -2.021101952
1.964136135 -0.641454548 3.215121331 3.700587026 -2.551681852
5.353083272 4.594299462 0.837542865 2.872200854 4.420211986
5.006830107 4.666182788 2.951797674 5.33566998 5.201749036
4.800024607 4.227386557 0.76018219 2.473120705 4.475837556
4.853419545 4.75593593 5.734100996 5.206607233 1.106654382 5.59497805
4.383772197 1.972830429 3.904699967 -0.88652005 -4.125139789
2.599884941 -0.367709268 -3.379921897 -3.768147079 1.785549997
3.085865297 4.512220172 5.184866832 3.139456619 6.172988976 -
0.606351493 3.308743446 4.506456753 3.834310148 2.905464638
TCGA-97-8171-01A -0.174996368 5.360389694 3.305680676 5.341586412
4.134588347 8.061637099 3.6339925 4.174416632 2.254165398
2.873103351 4.728618884 -2.058809844 2.912188986 4.745005466
5.128167367 4.617233449 3.748567997 4.420996132 0.315618076
0.399485018 2.549109374 5.211871545-1.349989975 1.793744305
4.690919068 2.837541356 5.746189068 2.978451771 4.478304639
4.056847828 4.776658857 1.056883246 -3.298655713 2.953777886 -
0.945299867 2.559395501 -3.75510172 5.598305264 2.943503109 1.178901958
4.692600539 -1.147298809 4.8114947123.54842393 3.813352905 4.255958096
6.902387172 1.55088156 4.862438491 6.981584021 -5.176590985 -
1.842806706 5.030405956 -1.978092825 3.258401878 -4.861441563
5.194139435 1.97198964 2.278543691 4.031900569 5.552239807
2.361569063 1.667355859 -2.270513446 4.685666495 3.942142698
4.072801057 4.264893347 -1.207862888 5.077270422 6.445791085
2.996600547 -0.156859797 5.727853873 -0.76364901 6.244573529
4.993905742 4.985077589 5.386892384 6.438547785 9.869261451 -

0.175809676 3.226128992 1.168113396-0.973060926 2.5737411820.669501345
4.265719651 6.958237525 3.556290014 4.902411409-1.974300618
2.564637688 -0.654257448 3.236595658 3.802817921 -1.759160496
5.362951522 4.223837855 0.992884033 2.7039805113.742671764
5.054284652 4.883543795 3.384617108 5.335550375 4.703536544
4.682745298 3.945528925 0.579481571 3.1936789 4.349519428
4.6611019944.744219961 5.596323439 4.676514104 1.240313927
5.173053061 4.327484743 1.847838245 3.938942484 -1.064714542 -
4.051741796 2.415493152 -0.221422398 -3.733914898 -4.08526276 1.224856692
2.804015191 3.993625573 5.84143666 3.589161771 6.592667089 -
0.51193239 2.4117464054.410494131 3.849897542 2.313141951
TCGA-97-8172-01A -0.516359092 4.725276429 2.644357273 5.093406502
4.140642649 8.287678577 3.837014758 4.206761551 1.964870626
3.2152673 4.77670952 -2.355127322 3.042936467 4.43066244 4.806454375
4.714089299 3.754205781 4.033658046 0.555772228 0.446234681
2.696607636 5.602781582 -1.341630195 2.216698976 4.61750613
3.0311968935.656346483 1.29307815 4.5032811153.166036504 4.924462439
0.783402383 -3.617433281 3.257641093 -1.090693729 2.82043437 -
3.553005149 5.565348896 3.800899621 1.415219353 4.708619868 -
1.154074565 4.948581244 3.236617846 3.9811167994.271556184 6.865954022
1.816766541 3.820522097 6.924776646 -4.606564944 -1.594391868
4.308049956 -2.458244868 3.51764902 -4.872318533 5.10301701 2.160841269
2.241952937 4.137889382 5.994090294 2.736688974 1.497115304-
2.207545576 4.775501901 3.931266677 3.898592157 4.277823418 -
1.974236236 5.091031753 6.503225045 3.014998433 -0.012894102
5.959458685 -0.313779829 5.964303247 5.141235128 4.655422408
5.385594587 6.413893313 10.19163961 0.639790371 2.685114355
0.754646903 -0.914724087 3.630484418 0.692531314 4.184933057
6.910513672 3.516266752 4.491746802 -1.787405833 1.939777582 -
0.309495884 3.2118432343.899849727 -2.348530342 5.346338612 5.130759968
1.674745074 3.107377349 3.8581189264.947460926 4.71111649 3.322264229
5.335529229 4.947777953 4.924940761 4.065341286 0.679412307
3.789764826 4.467065407 4.791953568 4.772844094 5.515365571

5.005685111 0.998904885 5.536052656 4.200445632 2.005964078 3.90018273
-0.514362285 -3.701331532 3.101544958 -0.430038007 -3.406552164 -
3.99831548 1.410798385 2.898249636 4.049323168 5.179383204 3.268272413
6.167515135 0.159925118 3.065267582 4.378469362 3.736702252
2.704588826
TCGA-97-8174-01A -1.031335597 4.524683928 3.097475856 5.37286619
3.987053677 8.337035651 3.654772005 4.250873744 1.961571364
3.21255589 5.101234747 -2.226666894 2.851587054 4.374538564
4.642376812 4.873275181 3.796023884 4.154665096 0.51894312
0.479262933 2.668122953 5.355648632 -1.625428022 2.756035803
4.562310511 3.179417296 5.822068407 2.611033667 4.439029087 2.987445833
4.86441532 1.034584509 -3.715625175 3.835504563 -1.200538465
2.851310776 -3.033519161 5.569703399 3.678147756 1.211690358
4.706176051 -1.253983871 5.30703606 3.310378895 4.016929095
4.241446425 6.848766976 1.828038056 3.668590382 6.678846486 -
4.638527684 -1.598352919 4.32473302 -2.614746074 3.324381135 -4.595492365
4.957497207 2.03681258 2.305383469 4.085131609 6.116764041 2.906501621
2.084917749 -1.891302099 4.682078336 3.93126945 4.115896848 4.109663992
-2.57687398 5.09030589 6.492104673 2.958102078 -0.104008997 6.112574843 -
0.690269107 5.928769186 5.237891119 4.637607203 5.428135916 6.444746887
10.55814359 0.892619682 2.549253295 0.859068143 -0.826046556
3.503624315 0.714336947 4.260605866 6.924792508 3.706934411
4.484934831 -1.885225056 1.848907461 -0.267393877 3.276631918
3.960748589 -2.737141906 5.333425427 5.092826593 1.510174958
3.180405623 4.088954816 4.928400252 4.752816448 3.279500289
5.335515041 5.214472548 4.857411303 4.124623319 0.453149334
3.751815295 4.561814045 4.685094997 4.756678372 5.666730425
5.43077029 1.002583128 5.670561076 4.145211445 1.897822021 3.812716212
-0.367659795 -3.824837557 3.008406689 -0.387432207 -3.380181471 -
3.76227525 1.689770299 2.72547177 4.232450653 4.895340186 3.098761012
6.034983974 0.126385423 3.359513823 4.306772864 3.713115093
2.636539435
TCGA-97-8175-01A -0.691086701 4.73600068 2.23022614 5.341236247 4.475126448

8.266874383 3.186091596 4.337737187 1.610807141 3.197385227
5.338360657 -2.578178193 4.223944055 4.624335357 4.737429441
5.276167866 3.162841545 3.485405966 0.342795792 0.471321273
2.566921443 5.246383057 -0.965418385 1.892308579 4.532094302
2.917362039 5.479202035 1.888417244 4.620175823 3.588426237
4.9581137250.932874683 -3.610329137 3.107863051 -0.860519522
2.606736919 -3.190664785 5.618740123 3.674254549 0.772473752
4.729357876 -1.254303638 5.04176487 2.276488507 4.020099545 4.2322048
7.162121012 1.200813574 3.587369973 6.693980035 -4.392892966 -
2.030502858 5.186546263 -2.240715174 3.374078882 -4.644164002
5.1179306141.877632357 2.792536903 3.65756041 6.082402979 2.441689005
1.506109641 -2.227141099 4.59823188 3.937002842 3.890428926
4.249205374 -0.325633204 5.088345549 6.2182611682.900164802 -
0.103920162 5.68159435 -0.392566225 6.063190306 5.05088231 4.675398712
5.49136265 6.197366198 10.83944442 0.96587665 2.7885298 0.51212605 -
0.760727238 2.717693048 1.01914765 4.384040667 6.772862435 3.439905947
4.809939641 -2.229258437 2.236781795 0.006846072 3.231343172
3.430196176 -2.186225639 5.348221023 4.4119893070.353209537
2.773713195 3.832614525 4.989446058 4.924441592 3.001436298
5.335448032 4.632713485 4.953897752 4.164741391 0.565132063
2.03061047 4.549894482 4.823507601 4.77519724 5.575482064 4.974983873
0.798657341 5.390509414 4.4178911122.234481586 3.940100641 -
0.91518135 -3.312094257 2.816243692 0.217058622 -3.256544941 -2.82206087
1.875209102 2.873898092 4.4741167025.201035183 3.571961585
5.993799502 -0.415930192 2.713254421 4.551875355 3.600143695
3.388535065
TCGA-97-8176-01A -1.112626183 4.971236264 3.257828463 5.514676359
3.977603088 8.210976889 3.257572328 4.325467447 1.868639515
3.156805865 5.746558666 -2.176649163 3.590817635 4.346224704
4.576251448 5.077460026 3.789099292 4.066559877 -0.075932588
0.252166377 2.734091798 4.655708331 -1.540218325 1.896043237
4.5220423112.730101067 5.761892677 3.041084331 4.461559806
3.367626533 4.934597448 1.003687681 -3.803439715 3.142651495 -

1.371922082 2.150199835 -3.143894067 5.602490596 2.74262637 -0.414265393
4.692445984 -1.129505108 4.556516923 2.780866081 4.045563876
4.267639622 6.7922910111.138835904 3.749419622 6.862008882 -
5.519088994 -1.927582825 4.693016899 -2.493596665 3.24030314 -5.109353408
5.140683215 1.879969844 1.821913292 3.662561571 5.994646444
2.38432601 2.320695177 -2.360510281 4.566359933 3.92766615 4.083986548
4.28661563 -2.488529708 5.083064823 6.435794845 2.879277365 -
0.25068721 5.95819492 -1.049652796 6.231682845 4.97399491 4.738038315
5.602541968 6.161350409 9.914561676 0.927003245 2.382470509
0.545342389 -0.61576588 3.002209878 0.917512386 4.378458246
6.834465315 3.470635391 4.915569439 -2.183838493 2.257326113-
0.604242321 3.277207512 3.84116059 -2.917625976 5.356100604 3.999340234
0.622860861 2.708452858 3.896898641 5.030578962 4.580302476
3.078106492 5.335441063 4.77194192 4.831581785 4.326792065
0.589743916 2.354048054 4.643619289 4.784375398 4.699421838
5.705546969 5.049333284 1.144742228 5.66015149 4.026140658
1.394648193 3.810445343 -1.127048123 -4.52558719 2.347034985 -
0.567330572 -3.8642387 -4.078997696 1.690949362 2.570185214 4.465876237
5.313246528 3.281992259 6.073963567 -1.730101082 2.739818933
4.374001421 3.631772197 2.248481496
TCGA-97-8177-01A -0.358819029 4.77653237 2.857780945 5.414737503
4.120002298 8.266876341 3.631575176 4.302047099 1.954518477
3.620393009 5.344470878 -2.162121346 3.906057052 4.272075142
4.851997901 4.997083859 3.695383793 4.313854194 0.443687386
0.522725213 2.856721248 5.291847067 -1.154798709 2.507459978
4.524893756 3.127032058 5.678396872 2.928310946 4.621833135
3.703493303 4.883769401 1.198868746 -3.611204476 3.464122358 -
1.0719609112.861477723 -2.912252456 5.587887134 3.102643776 0.631511945
4.724937411-1.055099304 5.188336539 2.705989586 4.029220122
4.273757293 7.081932872 1.452285252 3.536388085 6.934317716 -
4.790030774 -1.713392459 4.348124742 -2.561673418 3.653218415 -
4.226724388 5.0973118862.280318629 2.612082302 3.987584524 6.167906234
2.839734188 2.14359458 -1.522891676 4.654208435 3.930819364

4.379382737 4.2675992 -1.803155379 5.093517322 6.462079073
2.898451891 0.018401695 5.985200734 -0.334650456 6.018520075
5.142060463 4.663790712 5.510003417 6.269983428 10.44923506
1.306217442 2.629772098 0.894593012 -0.50707154 3.410759245
0.977666063 4.356239756 6.895231395 3.729530752 4.680765976 -
2.047667661 2.163707597 0.031022913 3.334655552 3.950919834 -
2.44385508 5.341420635 4.658230039 1.084869978 3.1116223944.15237533
5.01694901 4.664087687 3.161286673 5.335530547 5.199269286
4.892807279 4.666774437 0.73723859 2.968038024 4.6951134964.811945148
4.762787697 5.688596179 5.330431067 1.151068397 5.450519812
4.1196686342.0667398 3.88056161 -0.914633 -3.564356839 3.222188773 -
0.264865605 -3.528171476 -2.775417497 1.790565561 2.872440973
4.571636786 5.122445248 3.222450282 6.083487068 -0.147705294
3.167673483 4.672338898 3.784513842 3.400017593
TCGA-97-8179-01A -0.825063125 4.624014406 2.954356677 5.410111387
4.136582079 8.246312142 3.3224711424.223152689 1.828843973
3.334861821 4.975299416 -2.140328693 3.487477874 4.447991632
4.526570179 5.1104956533.8159833 4.2496114050.164007154 0.303356833
2.865665502 5.013735065 -1.416184281 1.97276713 4.612147087
3.105529812 5.396723564 3.0611357984.495198987 3.7167811645.045768951
1.283681536 -3.851933349 3.042727649 -1.359332919 2.864152564 -
3.249133807 5.60436083 2.906343649 0.818684586 4.697652428 -1.271066738
4.880054853 3.39486876 3.900430708 4.1746491146.834724408 1.613713218
3.871444515 6.818798537 -4.878873381 -1.804924657 4.65321129 -
2.434524181 3.449171619 -4.811121452 5.032058142 2.153563343 2.42609567
3.8656991 5.916747189 2.608774502 2.123392333 -1.775969135
4.595099759 3.9346611374.241954189 4.073587685 -1.882958717
5.078183887 6.432758893 2.910878982 -0.08406042 5.458367496 -
0.931421705 6.107208644 5.047579277 4.755746586 5.477131374
6.345440291 10.2515136 0.972898107 2.450364415 0.746744458 -
0.933095962 3.100880802 0.720268201 4.334103719 6.903319597 3.70487186
4.744213535 -2.099564852 2.20251956 -0.054451329 3.3510211683.870639249
-2.227224689 5.361445753 4.326880148 1.153167344 2.607159562

3.836641467 4.990255763 4.660901452 3.582214013 5.335425293 4.847797933 4.850565715 4.306282834 0.68318944 3.895927323 4.556287701 4.77237279 4.70817531 5.6941106315.386364514 1.113570077 5.4208115583.712612094 1.728984209 3.860610143 -0.972911118 -3.732795313 2.719412131 -0.408232469 -3.663818122 -3.908782034 1.534667945 2.7311364444.236908213 5.170401267 3.473885195 6.289885618 -0.942810343 2.627829553 4.497016357 3.611150865 2.548726179

TCGA-97-8547-01A -0.125539812 4.708313543 2.57811646 5.363162836 3.994197453 8.227520677 3.481315042 4.290978133 1.74411635 3.4309176115.368393333 -2.434763475 3.55688438 4.270435908 4.933388656 5.1168294833.291542064 3.9119167030.255180287 0.483196243 2.731593414 5.241373424 -1.053991311 2.306769073 4.547339727 2.997242391 5.537528412 2.825387484 4.6126888113.474293649 5.009765958 1.238384499 -3.426499397 3.571146577-0.978704398 2.627597968 -2.631571813 5.603551727 3.3115134660.776831051 4.725631911-1.051668612 5.204508277 2.552046045 4.104200383 4.234709348 6.997079651 1.563386805 3.729971023 6.714486417 -4.56911063 -1.729581046 4.601825795 -2.3696259 3.233162859 -4.400711738 5.130825446 2.376772946 2.522316421 3.91777022 6.102565376 2.575468569 2.086825961 -1.131307547 4.742829178 3.936333539 4.277083628 4.186445835 -1.176301298 5.093846142 6.438844487 2.882080917 -0.179637517 5.843273779 -0.250190101 6.010038244 5.026146569 4.80596325 5.545981751 6.300126481 10.81293616 1.126405656 2.722732003 0.785113949-0.428068834 3.1179009161.054004552 4.319597304 6.891087328 3.481473958 4.642407371 -2.059891862 2.360110987-0.175942071 3.308193498 3.77857994 -2.175436663 5.341378784 4.590080644 1.019167485 2.9487311214.059067646 5.032279808 4.849270746 3.018954912 5.33571555 4.873642736 5.026601778 4.316983004 0.941394903 2.483981095 4.471363048 4.884806294 4.749102563 5.530175817 4.981925084 0.914583407 5.530471942 3.980494077 2.177720084 3.928218136 -0.556752968 -3.724580528 3.138572813 -0.077495684 -3.403720059 -3.212605631 1.740525535 3.032743457 4.4833203

5.187353854 3.009930629 6.208028353 -0.300768526 2.999586964
4.45720974 3.742346812 3.272976477
TCGA-97-8552-01A 0.276262404 5.1140280613.216759601 5.170279314
4.079341705 8.176156969 3.894943191 4.233447039 2.197709948
3.389005295 4.880729334 -2.135762898 3.68310552 4.357913822
5.1163718814.779421853 3.468862056 4.432930573 0.346807714
0.525881791 2.973531406 5.485762614 -0.881307882 2.029027632
4.587860977 3.166127567 5.806141982 3.390423629 4.679184018
3.443037583 4.916804067 1.14153474 -3.286974993 3.060958486 -
1.340882928 3.113241071-3.096006809 5.566867806 2.967602841 0.86495079
4.729697541 -0.914589845 5.084576593 2.825057312 4.087155368
4.285800882 7.124778979 1.849434524 4.329569458 7.07779001 -
4.627318035 -1.511312122 4.178433796 -2.361945285 3.715574613 -
4.2454442115.31783481 2.464442069 2.646099801 4.1578811146.071653896
2.852128544 1.420358921 -1.406280548 4.772596359 3.92757076
4.5112873964.312575933 -1.052093828 5.097390358 6.564471896
3.048225165 0.021875761 5.9959043 0.157604032 6.030249961
5.139001587 4.828512601 5.436713319 6.337694353 10.09052427
0.578140481 2.756442236 1.108024981 -0.559994488 3.617234811
0.844259424 4.258009171 6.980338057 3.596167421 4.581982989 -
1.860106644 2.209288932 0.121917615 3.275189898 3.96175188 -1.973242949
5.349936439 4.94764888 1.293633513 2.970754802 4.1711879665.057081655
4.685980826 3.3768856115.335699182 5.09566159 4.913238052 4.510390825
0.97321366 3.180603443 4.708873864 4.903705083 4.772959806
5.594008332 5.174053641 1.28171756 5.39290209 4.331817298 2.277940538
4.019332906 -0.728622345 -3.213847353 3.412190764 -0.27086445 -
3.550988323 -3.235811607 1.566645773 3.068833065 4.353813592
5.537845521 3.282231353 6.442397515 -0.399036408 3.023378677
4.644949888 3.916570458 3.003516355
TCGA-97-A4LX-01A -0.130773922 4.719543584 2.883904813 5.282286022
4.1418718 8.2060611083.741021049 4.254267397 1.767693798 3.312990066
5.226400603 -2.466858738 3.431596871 4.330139432 5.015922557
4.94240729 3.717478235 4.0538073 0.436594503 0.444168634 2.701616545

5.184629213 -1.165570983 2.909082853 4.569467858 3.002636533
5.714823481 1.980400484 4.53852632 3.507386471 4.91360652 1.005140248
-3.49731285 3.562622957 -0.986013621 2.717945548 -3.120834795
5.588160336 3.467038893 1.342198369 4.711714304-1.159586111
5.380503319 2.926148732 4.017616123 4.201959459 6.83313717 1.64646523
3.773734551 6.78101855 -4.778961664 -1.90737071 4.508683533 -2.510021149
3.357105524 -4.865109474 5.140248218 2.106764301 2.170970057
3.95408395 6.078677084 2.707510099 2.182458352 -2.398927833
4.8111530293.93438666 3.910812986 4.458882942 -1.495015803 5.101431508
6.386958948 2.873090766 -0.083950984 5.897366879 -0.386651598
6.042913848 5.072382895 4.585722336 5.512976332 6.321715835
10.70096524 0.581326499 2.720079097 0.917190994 -0.662854922
3.824560851 0.986307502 4.283127825 6.890617822 3.535806812
4.766770942 -1.8674316 2.295042665 -0.537040869 3.271383009
3.889908491 -2.140580573 5.351082464 4.698768759 1.308048932
3.210779989 3.881202246 5.012681499 4.830364899 3.027100236
5.33564616 5.008285257 4.980486964 4.078141451 0.844381676
3.091435846 4.592700045 4.915372377 4.773282576 5.685924345
5.1411803890.95577644 5.569214789 4.183548809 2.156504341 3.870780899
-0.464949278 -3.769217837 2.928332115-0.217656824 -3.573832064 -
3.774032873 1.510604526 2.986346451 4.487740624 5.181920716
3.280043932 6.27700247 0.223942908 2.960605891 4.377246043
3.686309873 3.276725934

TCGA-97-A4M0-01A -0.265285041 4.938216604 3.20247638 5.187515009
4.172636093 8.23198688 3.536863097 4.304154995 1.815908965
2.879457527 5.008878566 -2.426003261 3.27923366 4.463656638
4.958794639 4.860395965 3.619931385 3.869478195 0.648522255
0.505476384 2.707186207 5.272293763 -1.056275616 1.828057671
4.589448648 3.076138778 5.6877068112.855698058 4.548218454
3.462432373 4.894668072 0.908731759 -3.503512941 3.141440428 -
1.219001469 3.132973757 -3.022930778 5.5892856113.80741542 1.293223744
4.703498567 -1.102632136 5.025688346 2.871230449 4.0528711514.23345749
6.805446679 1.6162114064.0401142686.642238866 -4.700199196 -1.744885429

4.581285193 -2.335172738 3.180004179 -4.704289234 5.179809393
2.39453393 2.256024674 4.030292061 5.946326882 2.60262821 1.422202202
-2.282319375 4.6411550023.934770949 4.053535656 4.431650592 -
0.73239814 5.087921893 6.380329952 2.999537934 -0.180369562 5.895624603
-0.410914197 6.1754233115.099219503 4.706464123 5.4515616 6.315419659
10.2164337 0.758545776 2.831682423 0.966935251 -0.678186529
3.177357431 0.854176293 4.283826075 6.914904437 3.603162413
4.655047497 -1.870038758 2.422342814 -0.55155133 3.234090015
3.745723866 -2.119767604 5.357249189 4.421493428 0.951332191
2.968238656 3.962478887 4.978639179 4.841492065 3.286924697
5.3354941194.88946436 4.85778607 4.172919015 0.690957736 3.179002801
4.459222267 4.835009401 4.74305976 5.576172299 4.936926147
0.940376379 5.616812244 4.257737764 2.018231222 3.964328243 -
0.604105287 -3.732431474 2.639991503 -0.206024619 -3.472252628 -
3.780807728 1.659585694 2.965930242 4.314578702 5.485806987
3.306289208 6.162832842 0.097028109 3.229081924 4.443265464
3.737793342 2.697167894
TCGA-97-A4M1-01A -0.112442706 4.8911181443.148223286 5.182625523
4.218418159 8.22277349 3.779523723 4.19908198 2.126190593 3.140659893
5.002140173 -2.144225517 3.496612334 4.3511023814.985292189
4.933943927 4.0335686 4.519693425 0.5324853 0.52073872 2.914723605
5.514777336 -1.117286411 2.150630836 4.605763971 3.317455895
5.714517576 2.904133842 4.624712454 3.465986671 5.060945868
1.159454734 -3.367889839 3.231918558 -1.250614439 3.580286087 -
2.985637869 5.575947144 3.216651483 1.593078479 4.715099552 -
1.068247282 5.188127533 3.231504568 4.016242395 4.235705479
6.900646283 1.960441278 4.238807066 7.000049294 -4.399064825 -
1.486596509 4.15916545 -2.459026145 3.760711084-4.548419599 5.220084151
2.505738724 2.59197212 4.172783248 6.051621958 2.755451992
1.432116173-1.565721063 4.764916471 3.926984346 4.424952145
4.115092448-1.908332535 5.088518424 6.61938362 3.056810821 -0.006435484
5.797619269 -0.220954568 6.037705887 5.090720988 4.851457628
5.429190158 6.35200185 10.08646363 0.705435422 2.808996775

1.083888409 -0.621284781 3.374903599 0.760298254 4.29632662
6.992637094 3.623915919 4.581762711-1.804763305 2.176307253 -
0.067673397 3.281847234 3.983993057 -2.151447313 5.345727194 4.81252961
1.302543454 3.098945489 3.983049041 5.030758654 4.703368264
3.66760328 5.335674941 5.194766713 4.940724941 4.302667536
0.922945066 3.89023131 4.678865963 4.881450416 4.740519273 5.75665996
5.470849667 1.252819419 5.341373437 4.206131321 2.046228413
3.975300369 -0.789809952 -3.364048274 2.893329071 -0.278861906 -
3.497840338 -3.43113241 1.593954906 3.013870004 4.40843961 5.368726329
3.2500043 6.431810142 0.265542146 3.281950166 4.628518445
3.832956346 3.288748431
TCGA-97-A4M2-01A 0.1194188145.084444306 3.553174437 5.152392751
3.9629119458.213213719 3.689195241 4.269438843 1.965947798
3.221595386 5.009444457 -2.257380714 3.354987856 4.154017921
4.982272005 4.764633789 3.522773817 4.154806051 0.294012497
0.453321307 2.783882926 5.312104039 -1.054818843 2.25444659
4.571435257 3.284179526 5.981619672 2.239834961 4.583263391
3.191063131 4.918601637 0.789000227 -3.421532735 3.122317002 -
1.389075844 2.942703626 -3.113874445 5.560935202 3.159929314
1.004501259 4.71940956 -1.007378497 5.102791652 2.899379913
4.1127812474.286890339 6.928859384 1.7682955 4.093372248 6.879845742
-4.764845881 -1.713471264 4.161074573 -2.502251821 3.555382547 -
4.8720311065.226653261 2.175202043 2.020487559 4.1105302756.105845041
2.747603164 1.689148064 -1.861063195 4.815847863 3.927959395
4.098917183 4.254203234 -1.55397652 5.102236776 6.539041742
3.017553401 -0.146341815 6.159614104 -0.056931591 6.038945809
5.1151308194.70922829 5.473173002 6.313562792 10.01918109 0.591924468
2.607494803 0.745154334 -0.718501615 3.6856531130.855139875
4.248451528 6.967725228 3.551917852 4.526774908 -1.815699274
2.100506987 -0.334535793 3.214344174 3.966522634 -2.153214623
5.354169618 5.02742095 1.282301485 3.026163626 4.063394087 5.03809392
4.676940893 3.210560018 5.335737514 5.234802327 4.880214309
4.174201989 0.851497844 3.077924037 4.788514796 4.921529381

4.765879577	5.666170557	5.216893405	1.184588386	5.551658853
4.414752321	2.151837667	3.955439763	-0.671153073	-3.71993981
2.909812656	-0.468476072	-3.587058053	-3.9871421	1.539798236
3.170067794	4.409621062	5.332728137	3.12962052	6.323467418	-
0.449672612	3.156526901	4.483620952	3.832742466	2.844903619
TCGA-97-A4M3-01A	-1.07151669	4.746129491	3.130546887	5.434851064
4.040604285	8.323545051	3.484802974	4.290321868	1.943269923
3.20155009	5.26384273	-2.301299316	3.259478341	4.381833199	4.631950274
5.138446987	3.794215801	4.171617695	0.716506188	0.374445042
2.784272485	5.168168447	-1.499196905	2.323306267	4.586779779
3.095380168	5.759658033	3.160371064	4.50907099	3.461679099
4.916434328	1.024429471	-3.898688999	3.525568361	-1.361421872
2.76105458	-2.802369506	5.591408141	3.19640398	0.664367446	4.704117137-
1.210714275	4.893676493	3.21313652	4.040433242	4.208473398	6.903381833
1.466440629	3.714744443	6.620671982	-4.994659833	-1.562721568
4.691542018	-2.394418113	3.175790757	-4.697278614	5.124907776
2.144881256	1.8637115443.8339906	5.9711928522.606426223	2.074545886	-
1.730302395	4.554809741	3.931388395	4.345203068	4.174014261	-
2.34550212	5.074917697	6.508583507	2.996909115-0.1602276	5.934892584	-
1.00574383	6.124466519	5.147883533	4.819372843	5.516066203	6.385485863
10.3207252	0.922159293	2.544354483	0.771808281	-0.673562735
2.976130736	0.746251867	4.384910066	6.917092363	3.692744529
4.568444445	-2.027328005	2.156769155	-0.386141014	3.300694845
3.887864811-2.578232614	5.350090349	4.433689387	0.853066442
2.787224833	3.97995742	4.995713107	4.679253152	3.493959588
5.335388079	5.150669225	4.828302981	4.384079707	0.443419613
3.275504277	4.576971571	4.749297976	4.708859958	5.736706323
5.275683218	1.089470156	5.7211186374.214792513	1.691366296
3.910682576	-0.639293808	-4.035561384	2.694761264	-0.535517951	-
3.443475429	-3.94969877	1.760179019	2.754364072	4.340083343	5.143186839
3.215229036	6.191539177	-1.094367456	3.085309613	4.449508202
3.674922132	2.430757915
TCGA-97-A4M5-01A	-0.658338209	4.846046295	3.054918299	5.271808965

3.972796853	8.265243273	3.552978998	4.275288202	1.917548038
3.032273083	5.336146999	-2.313691724	3.43963388	4.10638377	4.732651282
4.896263984	3.748028198	4.158398092	0.370852759	0.477402118
2.6801140445.153914041	-1.377994625	2.241550498	4.528861009
3.197487885	5.926028972	2.266352375	4.479954154	2.880676001
4.928235726	0.798056417	-3.673566647	3.436614882	-1.378756986
2.896208645	-2.853556707	5.569267722	2.979222787	0.776876733
4.710312061	-1.085120543	4.91200888	2.985129907	4.1146592154.295990841
6.874048695	1.728704283	3.716044142	6.781200873	-4.773331895	-
1.796287178	4.344896402	-2.50984311 3.349213368	-4.996556222	5.172732097
2.199324375	1.780998064	4.045860465	6.088741576	2.726328851
1.814049956	-2.10956806 4.732304923	3.926252496	4.067177489
4.177205858	-2.425476591	5.088728529	6.499988428	2.999795901	-
0.17034576	6.091700765	-0.502381002	6.055698158	5.144381227	4.663763358
5.481545707	6.3200353	10.13291978	0.833084062	2.439044603
0.694098011-0.698529156	3.541367392	0.847903454	4.283395343
6.924899977	3.6174412	4.596083198	-1.862665144	1.930452228	-
0.434820937	3.230277304	3.899090579	-2.568800509	5.354245348
4.701955041	1.201664775	3.068482525	4.15165576	4.988813576
4.633216018	3.161238744	5.335544174	5.11473937	4.870861994
4.0263116060.717074206	3.258589274	4.717200915	4.832018716
4.734392913	5.7411927235.347665261	1.095615945	5.581410192
4.213453462	1.806988783	3.916376806	-0.607503642	-4.030720912
2.582871959	-0.463255809	-3.500083227	-3.865906207	1.633456398
2.918903392	4.443939425	5.1817115873.1611219396.178436834	-0.366614358
3.303663801	4.406164167	3.749487797	2.772189923
TCGA-97-A4M6-01A	-0.271645124	4.597385405	3.164446535	5.230050089
4.191992131	8.161270444	3.524400821	4.259098102	1.635200357
2.934384768	4.964280106	-2.597396698	3.350331939	4.387465729
4.790813501	5.095631208	3.849729268	3.978644577	0.134413245
0.456617449	2.71939958	5.025283735	-1.20323389 2.398176615	4.537995393
3.354476612	5.720374926	1.567579462	4.497288653	3.106012651
5.017066953	0.784666777	-3.41572113 3.115980013-1.413191982	3.042592164

-3.405173877 5.58985968 3.132521987 0.895643269 4.709579235 -
1.144515168 5.290324853 3.010642069 4.016926903 4.280261059
7.001479588 1.526104251 3.838659943 6.671175376-4.745422912 -
1.977534058 4.739333523 -2.487510617 3.227885017 -5.14342791 4.988813732
1.970817369 2.017047237 4.048224054 6.0811394732.592273823
1.947491819 -2.380114077 4.746374761 3.936347864 3.812120241
4.166034026 -1.895148429 5.091017505 6.277635709 2.992056085 -
0.152839277 5.88545352 -0.402965001 6.015472085 5.102926302 4.636150397
5.5053319116.281824295 10.602119080.464905241 2.744193785 0.630246136
-1.024993846 3.5172303110.931776069 4.242478883 6.841761715
3.694823876 4.689820073 -1.943382643 2.058860794 -0.581105882
3.224316276 3.605054635 -2.194146134 5.358224862 4.525924891
1.390147134 3.068081899 4.026342046 4.992723741 4.829232926
3.229464017 5.3356111894.982226705 4.955277518 3.885770165
0.778727198 3.606273974 4.713531728 4.852341778 4.737379425
5.686940876 5.453368043 0.767370407 5.597338077 4.214346853
1.979371661 3.908299031 -0.346120822 -4.136254909 2.705812054 -
0.127378458 -3.533638541 -3.901723731 1.697645467 3.099130746
4.555584393 5.339627982 3.462316569 6.270939443 -0.360847527
2.995316365 4.472468234 3.820793583 3.425741399
TCGA-97-A4M7-01A -0.130881597 4.940841519 2.577536204 5.122145622
4.041673935 8.257845298 3.67343285 4.273860969 2.0231160413.493589894
5.131138334-2.325055725 3.50191982 4.358012757 4.91970485 4.945485604
3.579068629 4.078506714 0.432185965 0.474428651 2.914470745
5.406224061 -0.996296293 2.271484261 4.599149802 3.004610451
5.855878585 2.647215796 4.655849176 3.539449879 4.92542419
1.159924725 -3.306633549 3.24287916 -1.164805784 2.830384856 -
3.0311105225.576631809 3.483991739 1.024195301 4.717190026 -1.006465555
5.313866502 2.831297239 4.085437588 4.347572432 6.940979093
1.7741171944.004812043 6.766778759 -4.528989321 -1.650875228
4.342652988 -2.354084687 3.49807996 -4.489793491 5.137024929
2.470556676 2.299986673 4.216766699 6.139122245 2.797048107
1.74550723 -1.684346077 4.737338643 3.934434212 4.185178241

4.274842718 -1.108949152 5.097731458 6.50687035 3.013674535 -0.1078539
5.939439825 -0.064726778 5.986445409 5.138539352 4.655092534
5.467986373 6.309681405 10.14343534 1.088386816 2.752343845
0.992887625 -0.631752943 3.432942929 0.844999743 4.244183803
6.895277227 3.625132845 4.624144634 -1.907108321 2.309629254 -
0.197720053 3.274570374 3.806147894 -2.163048498 5.352255406
4.893296818 1.4098111973.156732174 4.189702289 5.007337351
4.716894081 3.2671143485.335682843 5.195178781 4.939136239 4.05216209
0.840854598 3.1195763644.543963325 4.850245468 4.767340518
5.690528393 5.18155345 1.09140693 5.580868377 4.028551482 2.164508241
3.95375396 -0.603517039 -3.488645358 3.021552663 -0.220576224 -
3.455313585 -3.351122801 1.528739819 3.042500517 4.357223666
5.396525526 3.303939364 6.22702863 0.417227637 3.074746554
4.566466316 3.785366639 3.032379032
TCGA-99-7458-01A -0.67746721 4.563992891 2.371939688 5.490281036
4.300284699 8.303969856 3.396875816 4.32143048 1.708259801
3.264856603 5.18109981 -2.500843225 3.247607728 4.744292483
4.805287105 4.731804898 3.387160586 3.589342108 1.057151343
0.572675134 2.394398747 5.445051152-1.468397386 2.5092611574.566645117
2.952991544 5.577663591 1.039062978 4.39095557 3.09286092 4.725911829
0.793474453 -3.799551577 3.835691256 -0.895823 2.676757362 -
3.158029201 5.585546476 3.6818472111.63431782 4.706624254 -1.274273639
5.330836861 2.985155337 4.00231347 4.336259598 6.847965599
1.445513948 3.33142038 6.431792638 -4.834356046 -1.867801911
4.582339546 -2.340147658 3.295282844 -4.700373323 4.911638484
1.805560426 2.459244524 4.058162243 5.999523374 2.62672407
1.999989317 -2.735142851 4.633461961 3.948142447 3.7238745 4.37843117
-1.803627385 5.086143163 6.164382892 2.90263657 -0.102739122 6.03233038
-0.457038754 5.937949128 5.180351094 4.294452698 5.461970818
6.385457159 11.072685150.5118914482.671417505 0.833895582 -0.844432179
3.419923538 1.018642675 4.209776573 6.815763439 3.524504926
4.70155115 -1.962378629 2.098530794 -0.379364574 3.227662485
3.620961969 -2.293784432 5.342554788 4.999643793 1.148510654

3.226607952 4.004677509 4.915623475 4.890073271 2.761290109
5.335342249 4.934383275 4.92331075 4.084383929 0.367570145
2.726334664 4.404045576 4.63492379 4.80302738 5.425758095 4.759837736
0.6309811215.72931088 4.604137438 2.1178750223.743132203 0.060191237 -
3.9706976113.094302902 -0.117969262 -3.280837376 -3.768460817 1.581752659
2.744909595 4.304421041 4.979149048 3.206899013 5.936659654
0.358607507 2.995527289 4.272000696 3.63474972 3.158694502
TCGA-99-8025-01A -0.671748763 4.813480314 2.529578764 5.375696253
4.108661241 8.243749913 3.201816189 4.258270021 1.825897639
3.373578549 5.359799947 -2.321433221 3.6211236944.593163125
4.714589803 4.936346686 3.700165765 3.734174372 0.446686092
0.370452236 2.746065974 5.186965353 -1.30632474 1.498074847
4.604212934 2.846294274 5.37023953 2.456215652 4.526981997
3.450199425 4.996976129 1.12258075 -3.841442629 3.114742132-1.072342829
2.563970498 -3.146308069 5.600778235 3.213536033 0.400659497
4.704218829 -1.144717796 4.743492089 2.879051559 3.97335531
4.196975671 6.944805502 1.410401369 3.635998286 7.013153207 -
4.928403157 -1.793051017 4.835748423 -2.484360129 3.390998644 -
4.738963057 5.188290025 2.069618746 2.479488521 3.775287782
5.909610664 2.418908187 1.74265632 -1.697745302 4.566173464
3.930248602 4.16539995 4.335195624 -1.491246976 5.076284884
6.373741666 2.880678995 -0.011838463 5.547625414 -0.701247155
6.1511992934.992200121 4.94363979 5.469107134 6.359712167 10.55616123
0.698743035 2.540941725 0.497313018 -0.57477223 3.081703668
0.870820704 4.314960081 6.901489751 3.372333831 4.818035167 -
2.032583126 2.19467199 -0.161408039 3.290899919 3.810624921 -2.380331856
5.338501032 4.310022859 0.970025796 2.7930341193.877973354
4.998994628 4.681454794 3.257001814 5.335398679 4.600903387
4.965745152 4.238062264 0.666067086 2.814771919 4.551786794
4.827594681 4.719104913 5.429832791 4.869866604 0.902448544
5.52187406 4.108797472 1.768532386 3.933688302 -0.842844645 -
3.91333585 2.883212395 -0.42984563 -3.626854503 -3.748217667 1.420394811
2.66204167 4.1451112445.288061072 3.11119656 6.074218107 -1.042321782

2.96981891 4.440356828 3.620778615 2.711764225

TCGA-99-8028-01A -0.006885389 4.839600038 2.980280195 5.448115115 3.926752188 8.297393027 3.678530425 4.356515353 1.872869494 3.75459108 5.358423067 -2.347206291 3.92931215 4.155982319 5.022176945 4.880964251 3.400516035 4.184134349 0.49487 0.507493076 2.71872869 5.516024921 -1.097794112 2.764925801 4.542317948 2.925476208 5.748512713 2.038924745 4.61549878 3.715773963 4.827239325 1.148030581 -3.559327998 3.619571451 -0.981879193 2.391774725 -2.90996792 5.587752556 3.06182528 0.3807189 4.722959102 -1.082217639 5.284623453 2.466012493 4.079589701 4.292296554 6.924552231 1.389589257 3.313237227 6.990085123 -4.893875244 -1.741998323 4.476347334 -2.539683645 3.5188597 -4.507128967 5.061249125 2.081468153 2.373073105 3.974876732 6.188204769 2.746359132 2.27532608 -1.44842351 4.724034483 3.931780918 4.160479896 4.383667315 -1.563706161 5.106371294 6.480671285 2.823550843 -0.038010139 6.019821138-0.188824295 6.004924866 5.096379559 4.632798069 5.550499663 6.321485447 10.81612391 0.967023893 2.642551714 0.762328198 -0.433084125 3.675091069 1.095250028 4.341301627 6.870433062 3.547350473 4.606018623 -2.00126491 2.259989142 -0.312253485 3.309094227 4.056006142 -2.240241759 5.330025484 5.003994761 1.154165642 3.21090602 4.090647781 5.020416616 4.745234942 2.76097522 5.335723329 5.149274262 4.961567349 4.430799818 0.808805752 2.254060513 4.650916607 4.838052845 4.777075983 5.5411535215.061291622 1.040790202 5.548875763 4.209276109 2.241579423 3.820076006 -0.309475982 -3.921982581 3.190486364 -0.523887613 -3.584461664 -2.848113048 1.71178535 3.042840966 4.538506991 4.9916011693.079257514 5.914564382 -0.707289509 2.98320065 4.376622375 3.681347363 3.38976655

TCGA-99-8032-01A -0.82539669 4.638819453 2.3174451195.285972697 3.884183546 8.400393071 4.205672738 4.234003702 2.31934996 3.766503235 5.704049991 -1.867073643 3.409427607 4.587338215 4.930901136 4.842717978 3.973365173 4.798871882 0.747109846 0.501560705 2.908221978 5.694118099-1.553431031 3.023037483 4.665028784 2.5547901

5.74868845 3.548281114 4.573966648 3.989836653 4.677664471 1.39288498 -
3.586437449 3.714922437 -0.972924017 2.433642616 -3.09447227 5.579286301
3.524333583 0.06881391 4.704750502 -1.128430116 5.171328147
3.303210933 4.001825605 4.370537631 7.020916407 1.693680248
4.204713463 6.997655108 -4.842820452 -1.235981232 4.342331957 -
2.131567483 3.751849474 -3.949021735 5.142186611 2.661879433 2.126434938
4.21751117 6.047770429 3.074640868 2.649696858 -1.1589299 4.599029706
3.937312342 4.755903141 4.339755866 -2.623728155 5.074596602
6.638952215 2.972630902 0.045025531 6.028065147 -0.535266245
6.06015257 5.180844723 4.962959813 5.48326499 6.672442217 10.2266767
0.863156141 2.854593522 1.54287438 -0.590557779 3.468583726
0.658609815 4.253214022 6.893664013 3.761896528 4.71904313 -
1.884512668 2.430454326 -0.002862951 3.376369857 4.162353144 -
2.68881163 5.335478834 4.750265438 1.557167478 3.327997288 3.967968237
5.01575662 4.650473956 3.574905622 5.335589782 5.24424969 4.870267834
4.547286848 0.640621085 3.707081636 4.474354522 4.606888609
4.714644309 5.769245985 4.964711338 1.250951956 5.415166249
3.511212219 1.62056374 3.936089073 -0.50794032 -3.224778274 3.507872294 -
0.490312207 -3.500452223 -2.790453187 1.392139362 2.620273606
4.076794597 5.555864484 3.429582627 6.261212052 -0.40500128
2.642766028 4.576325551 3.851730465 2.3703449
TCGA-99-8033-01A -0.621055678 4.88862525 2.910569854 5.358615124
3.939182126 8.186769733 3.183074849 4.310634315 1.750225886
3.633290615 5.580581286 -2.439029893 3.60922952 4.57246259 4.550933344
4.965469327 4.043281483 3.810232497 0.368907696 0.240806795
2.615342259 4.801128004 -1.390729781 1.890808719 4.585367675
2.653671867 6.000674238 3.054406638 4.526409013 3.93553029
4.908275787 1.177851116 -3.847650971 3.125110183 -1.139377721 2.432529771
-2.778733024 5.608985567 2.87861298 0.321851867 4.681000839 -
1.292680365 5.129678788 2.986033703 3.986769471 4.200476657
6.780767362 1.377094237 3.910258564 6.695493033 -5.173006507 -
1.888791519 5.285773524 -2.359208853 3.266381559 -5.167857768
5.052831709 2.038606407 2.44985265 3.716420738 5.989300932

2.4562413112.25192582 -1.820034418 4.624223623 3.933431337 3.982907291
4.358608748 -1.55213418 5.079332222 6.289880189 2.806543408 -
0.217401943 5.685188129 -0.859279528 6.148190579 4.975620759
4.850235896 5.475447574 6.235932776 10.53159954 0.547390914
2.495933881 0.589677938 -0.425560002 3.251967201 0.938283931
4.285271862 6.842179101 3.394367288 5.074138269 -2.124627872
2.491161623-0.480609113 3.278154257 3.716730692 -2.419786456
5.349850518 4.022441287 0.8741112052.907748842 3.663106726
5.007334245 4.790464713 3.039005526 5.335374748 4.5317255
4.909364067 3.804341515 0.634633509 2.358344459 4.709439735
4.817900869 4.704593754 5.592849664 4.970440174 0.902731585
5.503790855 3.700199525 1.616423393 3.84440834 -0.514488385 -
4.094012485 2.257748271 -0.096707763 -3.838479626 -3.723010956
1.500125088 2.709833896 4.226838992 5.3116809253.473667325
6.197810466 -1.305718049 2.766955563 4.353829613 3.497062725
2.66535198
TCGA-99-AA5R-01A -0.255564428 4.826226201 2.881394264 5.147888054
3.814178451 8.276071498 3.755714223 4.257198271 1.903217223
3.475020777 4.971362224 -2.384477407 3.400340206 4.146436176
4.92765703 4.700192917 3.594205037 4.015048158 0.554157504
0.5237916112.752728055 5.528938465 -1.168337324 2.50596011 4.578330538
3.160939795 5.863989323 1.528205338 4.585698564 3.099563006
4.928126464 0.897647755 -3.464778597 3.357601512 -1.255086499
2.94075304 -3.053448383 5.5575556113.275586097 1.224540808 4.71892513 -
1.0838073115.139190612 2.842313022 4.043556952 4.301820466 6.854841557
1.821767139 3.578887132 7.01669654 -4.560136831 -1.667767262 4.09104449
-2.612164857 3.531847057 -4.665047747 5.145925997 2.077560291
2.29832956 4.130823161 6.142519568 2.803551221 1.729243482 -
1.7861177594.799072379 3.9221198724.052768774 4.249164555 -1.665676849
5.103953988 6.562599826 2.935179076 -0.03339051 6.015427374 -
0.1159355995.939299568 5.135276166 4.723035886 5.424699066 6.335282446
10.4046787 0.813361828 2.666420337 0.720245015 -0.575883722 3.79273653
0.847138394 4.232907004 6.937340074 3.499199889 4.461836657 -

1.839168151 1.880754812 -0.441999003 3.239778691 4.010482076 -
2.271017192 5.324840403 5.19143246 1.559028781 3.248259569 4.029020663
4.9691131224.663792321 3.108924777 5.335724582 5.2372113084.971495911
4.016051554 0.795592088 3.209810206 4.739824398 4.855220598
4.781428673 5.601440372 5.212169452 1.048449717 5.454542264
4.24719325 2.218951624 3.88556571 -0.481974841 -3.718648665 3.033290137
-0.535495406 -3.497409252 -3.47593261 1.613954387 2.950678248 4.35029172
5.05702733 2.844565621 6.015761564 0.186368423 3.27044371 4.3307355
3.7760831153.104656499
TCGA-J2-8192-01A -0.621743123 4.334307078 2.262086419 5.39590043
4.271672615 8.331845425 3.234628551 4.293405188 1.626817613
3.391676383 5.073816606 -2.681679648 3.319106653 4.561521117
4.6294815115.102381331 3.415751579 3.487901752 0.671420614
0.528676784 2.584501514 5.35827942 -1.260606699 2.5599811214.548897218
3.058480304 5.467845244 1.535588191 4.475457516 2.769204273
4.877240495 0.959017725 -3.889741232 3.706106124 -1.00788674
2.815063563 -2.957923738 5.591427496 3.702866783 1.492488519
4.722168897 -1.214899904 5.321804777 2.893980432 4.027317603
4.277240493 7.067287999 1.546101398 3.0763245116.440259285 -
4.66289097 -1.882004699 4.731087632 -2.592833627 3.179856487 -4.582170285
4.9527409 1.868173559 2.707641719 3.951912025 6.121024669
2.596892471 1.85495581 -2.240753534 4.673353783 3.940995062
3.848720175 4.17796294 -1.30896583 5.086574968 6.297198432 2.901923684
-0.110111247 5.82308035 -0.363473171 5.847209008 5.212697814
4.397592278 5.464470827 6.312006196 11.166277871.1162275362.528835749
0.626809821 -0.793403884 3.284547098 1.02973468 4.227378831
6.840424031 3.583985055 4.594852877 -2.060082596 1.840367659 -
0.204337864 3.266404208 3.672400672 -2.192820107 5.336864729
4.986140605 1.330784827 3.194749049 4.022818141 4.887567566
4.902370553 2.823984215 5.3352528 4.926590204 5.034502677
4.075298981 0.605767574 2.947455157 4.410546925 4.75646068
4.809812049 5.491276164 5.127932076 0.587449163 5.662456276
4.324489827 2.267412445 3.805447446 -0.051664807 -3.753470175

3.149550322 0.04858869 -3.242141078 -3.474856871 1.768930493
2.824800074 4.44190426 4.799989749 3.187291016 5.9471185870.030698297
3.07757562 4.467710559 3.607399285 3.463385585
TCGA-J2-8194-01A -1.025966206 4.474168433 3.493464181 5.567490261
4.229484392 8.337166738 3.213295348 4.3511489581.632967649
3.094038764 5.278406564 -2.459724611 3.135663956 4.467922658
4.433995339 5.0605081133.598395244 3.874986227 0.443848 0.393860052
2.526630467 5.233836608 -1.691669901 2.40059232 4.528370014 3.03266065
5.660947467 1.857335345 4.34437676 3.144712882 4.83051862 0.745265394
-3.924691582 3.63149976 -1.280972883 2.556225753 -3.296016518
5.5993811693.432109448 0.493365773 4.695032543 -1.30969449 4.999828997
3.255891392 4.004813319 4.226464319 6.860857894 1.290350841
3.339413528 6.412731016 -5.2295515 -1.950631381 4.786284126 -
2.391731552 3.146739974 -5.398793585 4.890748205 1.641814602
1.857071039 3.754883007 6.01089067 2.459819416 2.537446655 -
2.790496098 4.599658767 3.940102496 3.788542587 4.146307639 -
2.752251873 5.077901679 6.301248681 2.94513424 -0.285069404 6.016328936
-1.028668646 6.074264495 5.165418235 4.5230563115.55811804 6.470013764
10.67498062 0.6113380912.452246391 0.494291436 -1.118612147
3.047776791 0.869463308 4.324723736 6.829066982 3.680182378
4.671778203 -2.034836846 1.954954967 -0.506287737 3.252792256
3.798312916 -2.685290798 5.35717258 4.672368526 0.922752595
2.765896736 3.985485498 4.960168322 4.8132531 3.025319692 5.33531618
5.003193308 4.807084247 4.140604512 0.349165292 2.964497887
4.542863462 4.698921885 4.725970407 5.529573672 5.178714298
0.805192327 5.75029133 4.090974303 1.658709143 3.747230878 -
0.15489883 -4.592376647 2.627617089 -0.412754517 -3.489756305 -4.449631503
1.692907297 2.650226574 4.391023324 4.9325301123.339061306
6.072376429 -1.065209918 2.895477051 4.326752867 3.613269467
2.499194557
TCGA-J2-A4AD-01A -1.191008142 4.33730298 2.154603138 5.282724391
4.519418621 8.189936089 3.172850575 4.092854891 1.669400058
3.381074935 5.237758882 -2.496205873 3.256926379 4.840463951

4.405887192	4.9302721154.060374605	3.539523958	-0.269367122
0.288698702	2.822731666	5.186466177	-1.389542684	1.678968678
4.597896954	2.992416316	5.02624093 1.38713028 4.462359139	3.534619611
4.994209173	1.282099243	-3.982609309	2.482535078	-1.252461115
2.858869696	-3.470379966	5.60875212 3.745534244	0.959927294
4.697486774	-1.392707895	5.028671552	3.087962124	3.845415341
4.060712541	7.031861413	1.184468247	3.627037194	6.651975242	-
4.898913362	-1.80979277 5.003753733	-3.024772491	3.713519898	-4.718570243
5.048464684	1.562973331	2.978124046	3.809795586	5.872336712
2.560097318	1.780454567	-2.493364169	4.468414433	3.93297177
3.987875939	4.168299376	-1.168885151	5.075689021	6.227469607
2.9055531130.026187722	5.115922261-1.090593437	6.062192219	5.065938613
4.632416449	5.4167811086.30710634 10.8718695 0.6963116122.503376386
0.321023022	-0.82630835 2.719481861	0.7911191754.244298786	6.852996048
3.616513076	4.897904298	-2.222147549	1.965383036	0.035069221
3.309121607	3.522438915	-2.174493849	5.34101919 4.1121989840.976814613
2.631719076	3.997763747	4.916275235	4.826917675	3.473820317
5.335092371	4.796505328	4.876676714	4.1361596110.556835234
3.405142448	4.603521074	4.741692228	4.726871281	5.797027155
5.1653711050.69732041 5.558956454	3.845084266	1.890553378	3.831770544
-1.277589409	-3.57603477 2.917459137	0.128488372	-3.692774084	-
4.02823735 1.408531439	2.438534387	4.20692138 5.213372496	3.570377014
6.226209011-0.689454833	2.622317839	4.437970707	3.558443138
3.041677525
TCGA-J2-A4AE-01A	-0.281761812	4.826917602	2.572579081	5.058439364
4.297351331	8.277990772	3.596236495	4.235815701	2.045060296
3.375003809	4.694528937	-2.203487559	3.1858403 4.758120613
4.942376969	4.684873777	3.694879802	3.804661932	0.703238555
0.5011650452.699297527	5.634739194	-1.085417226	1.77017689 4.642779207
2.83921866 5.698846402	1.672454512	4.557934985	3.23807474 4.816245685
0.941268038	-3.545835629	2.974282767	-1.036689044	3.022868034	-
3.490378159	5.584740813	3.5786541191.703190721	4.70573938 -1.171641995
5.26749929 3.059261014	3.963590319	4.325403957	6.979882608

1.651553013 4.4569411966.785624784 -4.508464204 -1.558142326
4.718273176 -2.318317983 3.830193119-4.551107918 5.1174796081.873727578
3.1187131614.33358291 5.958022856 2.608038325 1.286589339 -1.75826873
4.715604191 3.937973797 4.059068216 4.202693855 -0.845917098
5.082586036 6.4172719 3.037915817 -0.076389507 5.812963697 -
0.1133900385.996416783 5.107476442 4.681620733 5.351886387 6.46259355
9.930690497 0.383287171 2.996385412 0.883153353 -0.829811022
3.143259751 0.766435929 4.137142414 6.882140261 3.470857601
4.770564531 -1.869353485 2.159175001 0.269032821 3.2190071
3.687427092 -1.883799925 5.337177409 5.033715288 1.26611128
3.245452437 3.986835774 4.922628238 4.904608593 3.385924512
5.335383161 4.950837466 4.921827446 3.986000489 0.712254512
2.916856214 4.640179615 4.704164805 4.781608596 5.70088658
4.934371583 0.895933093 5.277739787 4.466231819 2.109397092
3.950172667 -0.497062123 -3.410855915 3.226902736 -0.077534193 -
3.459298047 -3.508422128 1.519834659 2.884730833 4.176600333
5.593825981 3.355521514 6.430824517 -0.748260014 2.762766019
4.5817983113.846199437 3.292953157
TCGA-J2-A4AG-01A -0.432035043 4.726447549 2.928605058 5.306487566
4.195424771 8.288617282 3.542104121 4.309013067 1.94624314
3.387932708 5.194041107-2.387794719 3.613230208 4.635073588
4.859554521 4.897036136 3.781257666 3.895647461 0.95612018
0.499415934 2.7543811885.444049752 -1.266293607 2.3677121 4.562369287
2.957760127 5.67353621 2.490542181 4.5116772573.283819703 4.860718495
1.001976902 -3.654653236 3.606426212 -1.112593351 2.807803009 -
3.228919174 5.586314197 3.815724289 1.1222465114.707718825 -1.177783928
5.169847873 3.0211993644.065484922 4.269612368 6.893388054
1.513561396 3.731064586 6.771524724 -4.710384737 -1.685542368
4.726328874 -2.21901519 3.349109575 -4.528018679 5.100007996
2.132048346 2.226651864 3.999984605 6.030097967 2.783844872
2.106054745 -2.110871617 4.651699632 3.936975091 3.951250106
4.499158386 -1.758767253 5.085953599 6.285850127 2.956350074 -
0.015935721 5.980836029 -0.49812511 6.055518577 5.18983224 4.575510331

5.46767874 6.373724532 10.46106618 0.690972762 2.76371774 0.913460413
-0.716200462 3.753656798 0.852470718 4.255496227 6.834613723
3.69847258 4.763648044 -1.830649751 2.189091986 -0.255439412 3.25972005
3.716560029 -2.30565578 5.34703881 4.651449142 1.3149711423.147002102
4.089392294 4.980973191 4.813207291 3.144409315 5.335416217
5.1141927454.885577861 4.1113161160.565827648 3.014850358 4.600936288
4.771477254 4.754968004 5.555452382 5.05387324 0.881542386 5.66653493
4.291854955 2.041922203 3.902236214 -0.032668039 -3.622178514
3.082952654 -0.155176508 -3.427182376 -3.527911569 1.571704286
2.910731335 4.364873963 5.195177444 3.393828336 6.178763119-
0.122351245 3.156587401 4.452984593 3.731905031 3.071115554
TCGA-L4-A4E5-01A 0.321515574 5.164771068 2.232385043 5.210315035
4.312053161 8.1149369693.636952563 4.155475363 2.10719089 3.202410009
5.057891281 -2.308339991 3.507320643 4.948334101 5.308559154
4.883148153 4.030761974 4.160925158 0.197965951 0.510917001
2.849983092 5.260778684 -0.880743778 2.023827661 4.682660127
2.854378666 5.46938616 2.739219289 4.636604365 4.222716294 5.03162113
1.314364749 -3.23626199 2.924026945 -0.804756458 2.595059369 -
3.52310055 5.602632792 3.38695306 1.169426268 4.700152466 -1.096418143
5.121480374 3.210237306 3.891099343 4.199644885 6.980635885
1.681937471 4.67438508 7.312628433 -4.80113197 -1.903607646 4.944337863
-2.23074894 3.584088464 -4.644996603 5.405783468 2.109439328
2.689058447 3.890819778 5.801093303 2.550444986 1.916576642 -
1.609907106 4.68220205 3.938852174 4.321747682 4.468313858 -0.08929707
5.078062121 6.404777731 2.837837838 0.098343262 5.340688144 -
0.298360795 6.24234511 4.893938532 5.155582467 5.431003373 6.476800104
10.21722554 0.146088855 3.241082929 1.10634854 -0.672341948
3.338383944 0.922443685 4.251529669 6.961765414 3.364070053
5.168168903 -1.641911723 2.898252082 -0.202628835 3.278428912
3.832432735 -1.4460109 5.349757826 4.1143760461.325082622 3.102517162
3.474042103 5.09639016 4.824866102 3.607653267 5.335622752 4.33843815
5.053974049 4.268785809 0.979282101 3.617576204 4.721936395
4.881051204 4.727847564 5.598371734 4.6076116491.039566837

5.459282315 4.212383887 2.042056137 4.083853646 -0.617134603 -
3.14378056 2.851742833 -0.151991797 -3.797124949 -3.916168436 1.194434192
2.975871978 4.168798164 5.930586199 3.480468607 6.577263629 -
0.040095893 2.458289059 4.554988627 3.73475495 2.757463256
TCGA-L4-A4E6-01A -0.025461285 4.730866054 3.4060729 5.248351192
3.989297624 8.355367207 3.513452765 4.348195839 1.745937805
3.338060092 5.084821364 -2.455331897 3.443898661 4.113071177
4.8393822115.0481104663.336654886 3.855340795 0.434762041 0.51728624
2.729606021 5.378146352 -1.002119499 2.9801521 4.521798378
3.348259731 5.939205563 2.01707883 4.55502826 2.851424188 4.908918185
0.843589893 -3.485458965 3.454281842 -1.336357 2.752240915 -
2.918335274 5.565189402 3.4254811781.306650361 4.726014841 -1.193830398
5.202012583 2.656903714 4.097149973 4.300702923 6.923196813
1.644132892 3.521285768 6.750001082 -4.557983918 -1.748892341
4.24340294 -2.675938557 3.482680686 -4.620666112 5.055509043 1.94577019
2.305677547 4.032693499 6.27927319 2.791917332 2.006395066 -
1.887686741 4.805146185 3.927269656 4.069610012 4.135430389 -
1.212200513 5.105158526 6.551800992 2.922765781 -0.21050062 6.170622686
-0.041511156 5.921968881 5.192214618 4.564967029 5.453846192
6.302419934 10.50815018 1.0132431112.63951591 0.560909185 -0.673406557
3.693071943 0.984238656 4.28878679 6.893791586 3.571328568
4.365883237 -1.996148928 1.892784099 -0.308621308 3.237873891
3.972154719 -2.029458902 5.336926314 5.302058286 1.094653916
3.098452473 4.1828908114.987051372 4.712740789 2.958874842
5.335669801 5.423976081 4.970527386 4.183291815 0.798656361
2.798869998 4.743974006 4.876702079 4.792226288 5.655530618
5.429998416 1.009034817 5.62184763 4.417208709 2.428185784 3.84797112
-0.512272538 -3.582644358 2.985096985 -0.478789326 -3.403417751 -
3.322988933 1.928089417 3.132664979 4.621507839 4.904957941
2.983987392 6.027413635 -0.198372374 3.308282454 4.364597411
3.7431102553.293294014
TCGA-L9-A443-01A -1.036944586 4.668915897 2.321369679 5.324693882
4.079392472 8.316892091 3.686503101 4.27723526 1.940678914

3.3616291195.179320481 -2.270541953 3.420393149 4.58088587 4.818906693
4.712233815 3.419534209 4.093016843 0.551591013 0.304849697
2.707036068 5.528238579 -1.437867764 1.931064465 4.602555141
2.869567397 5.449523022 1.761918384 4.508323307 3.70352859
4.869997635 0.985041122-3.783907354 3.432074762 -0.91517448 2.555946704
-3.500424046 5.596140941 3.414420695 0.39240133 4.710930108 -
1.162449009 5.186140869 2.668567637 3.944551022 4.226353732
6.978355454 1.105787143 3.432737269 6.690074842 -4.815033 -
1.71648404 4.580458857 -2.532617616 3.422825189 -4.444943709 5.015631949
1.784229765 2.292526291 3.939566198 5.909467644 2.616661626
1.961440778 -1.54200595 4.470203889 3.931912087 4.248075825
4.204455877 -1.653128161 5.087845007 6.319560275 2.989755114
0.039269866 5.77220619 -0.797346878 5.996922234 5.065444359
4.695309864 5.468649329 6.483709935 10.55700313 0.905922619
2.614010325 0.70039882 -0.888588499 2.919779546 0.79286859 4.31141664
6.85788733 3.548904465 4.578019068 -2.198941903 2.207503554 -
0.149776213 3.298038483 3.765171319 -2.503579403 5.328228279
4.676350985 0.790506653 3.037708227 3.910878439 4.98897792
4.722329094 3.462399744 5.335455712 5.028923765 4.904565723
4.499518043 0.369183436 2.6053171174.485722437 4.718360508
4.750155722 5.665648408 5.034828193 0.877602595 5.602260021
4.1124706061.981416238 3.927966716 -0.98436382-3.720363698 3.294670526
-0.413327062 -3.4917789 -3.645701164 1.346864995 2.606949688
4.012508197 5.263207269 3.346268618 5.897001592 -1.144758691
2.850140035 4.421326503 3.653065878 2.711295876
TCGA-L9-A444-01A 0.018390472 4.977729135 2.728498965 5.256208254
4.30058642 8.225490957 3.573082454 4.2491196381.831685065 3.17999098
5.039346033 -2.355233464 3.586728158 4.358823994 5.060940899
5.024334286 3.639745307 4.1261123340.207703464 0.520689261
2.732544922 5.204516634 -1.000849424 2.212498651 4.601749411
3.203175783 5.621919334 1.870019175 4.641222508 3.920415943
5.066998055 1.018254727 -3.407004474 3.290845284 -0.856447896
2.960801764 -2.966025315 5.594971042 3.592436864 1.083657624

4.717063873 -1.103779439 5.361099079 2.915914199 4.057126135
4.231777887 6.939706565 1.73046435 3.943438758 6.929403053 -
4.533271421 -1.897035266 4.605068404 -2.429016759 3.384755997 -
4.858066996 5.161562698 2.12091629 2.084328493 4.05714989 6.026523496
2.731382336 2.040512833 -1.604717639 4.760003131 3.936609001
4.090780341 4.220257408 -1.179066997 5.095777573 6.42742889
2.939968031 -0.069598148 5.704068334 -0.243887742 6.072033217
5.0112051584.835915098 5.506352392 6.334527103 10.47004832 0.78159394
2.815142703 0.790790855 -0.725765032 3.604982441 1.0041124464.33181962
6.927177245 3.54366586 4.718361546 -1.729682121 2.509712422 -
0.397532962 3.259428676 3.807540443 -1.992570996 5.35574478 4.643977949
1.237570646 3.109178094 3.781547655 5.062099157 4.806277705
3.2119366765.335756588 4.987934009 4.992676733 4.294071859
0.898628974 3.249820197 4.707571777 4.903061886 4.740224482
5.623705144 5.085889631 1.00886137 5.607342266 4.267571087
2.1108016153.997457545 -0.667773553 -3.720606817 2.7355259 -0.072128167
-3.534628972 -3.828990471 1.517895559 3.268763488 4.410658279
5.336956588 3.446591246 6.256336595 0.133264355 3.04272875
4.432100783 3.687952821 3.249191827
TCGA-L9-A50W-01A -0.109796254 4.956832053 2.778470667 5.152802127
4.207475556 8.238979287 3.46927579 4.221691939 1.87165635 2.774353532
5.461102975-2.471772807 3.64171494 4.246002771 4.978001729 5.328281564
3.5266415114.133354033 0.062641782 0.54114524 2.904156401 4.988898155
-0.662191481 2.134609814 4.525127847 3.244198227 5.54163182
3.2568149114.630404403 3.354878428 4.979437407 0.900623923 -
3.302731602 2.914675033 -1.359108134 2.923703433 -2.658319243
5.586793202 3.534863625 0.757826052 4.724307585 -0.998829359
5.028137463 2.697392046 4.081884726 4.213969196 7.21170114
1.685561814 4.246684671 6.850422503 -4.387128582 -1.83004764
4.549035227 -2.590476354 3.133314739 -4.515145541 5.326747113
2.544267141 2.17477737 3.9604784 6.158656957 2.774997359 1.575536414
-1.759771761 4.685321906 3.929955937 4.402830896 4.254108545
0.271446976 5.08936319 6.412819217 3.055680625 -0.193542577

5.834045941	-0.139023097	6.14224798	5.136037639	4.922751146	5.524175531
6.275584719	10.3236877	1.101142856	2.663422935	0.696521029	-0.755100992
3.220987425	0.891052069	4.402724454	6.928531386	3.729321246
4.744207526	-1.875868804	2.282270576	-0.363204691	3.253202009
3.687716842	-1.840427789	5.365917416	4.275955579	0.794227109
2.831134383	4.281463543	5.035380462	4.740739425	3.368429125
5.335591322	4.972587343	4.858750901	4.291038101	0.994220089
3.128908816	4.515403813	4.94333632	4.726074811	5.78138277	5.264256116
1.100777309	5.704719565	4.1197089	2.274933484	4.065738072	-
0.813264603	-3.414157913	2.843698843	-0.095716793	-3.28772742	-3.435001842
1.816962348	3.109827441	4.608732994	5.595913682	3.404539352
6.348015411	-0.258031139	3.085554273	4.62857625	3.782210385	2.783608436
TCGA-L9-A5IP-01A	-0.775217343	4.735423545	3.467142015	5.45266813
4.053517411	8.292235219	2.874220977	4.310803542	1.748782609
3.175859828	5.675057427	-2.644401284	3.471510618	4.115383217
4.167853272	5.304819672	3.836380527	3.976994077	0.383481327
0.320542608	2.602270892	4.687343539	-1.354257123	1.997787382
4.520314732	2.868607508	5.573238719	3.32674029	4.376100162
2.871221108	4.940623144	0.897032481	-4.325629315	2.927680839	-
1.649788607	2.464487038	-2.310309517	5.620704347	2.768683101
0.113640562	4.683716194	-1.346836087	4.830696401	2.858158726
4.070352454	4.155942101	6.778756093	1.085392469	3.308646453
6.481166572	-5.472302132	-1.803410869	5.447297634	-2.708570202
2.997242074	-5.472972745	5.035553729	2.03249426	1.964782542
3.449366622	6.031646432	2.210121881	1.935894953	-2.319748124
4.516611096	3.925518849	3.987780179	4.174372715	-1.823645785
5.070662359	6.313027033	2.868565528	-0.366387078	5.417503872	-
1.174074009	6.236145728	5.050762326	4.6780618	5.529392949	6.191140501
10.8845199	1.32772716	2.044891302	0.291148097	-0.345365793	2.857800422
1.056955506	4.396953026	6.789474423	3.548409164	4.747807179	-
2.281978593	2.016300156	-0.865492196	3.243636786	3.975004607	-
2.417620295	5.353343069	3.945840213	0.086067966	2.506354137
3.904449821	4.961859435	4.768474568	2.77915793	5.335033956

4.753024529	4.843732205	3.898521871	0.631672761	1.894183322
4.472159431	4.82613485 4.682891856	5.590214344	5.090553153	0.79332953
5.50051216 4.049551925	1.526186566	3.802237205	-0.343753404	-
4.739890194	2.064456877	-0.437580185	-3.84536156 -3.699268012	1.807558529
2.712777015	4.644273542	4.961059785	3.296947603	5.975443555	-
2.219852414	2.972871547	4.319537701	3.395634827	2.456927318
TCGA-L9-A743-01A	-0.019306222	4.880542126	2.797760558	5.330419776
4.02459065 8.268073401	3.519562791	4.319156172	1.788788821	3.55622882
5.305284653	-2.322158947	3.661052846	4.307433464	4.903976384
4.821554805	3.408404913	3.858405983	0.447467464	0.491692908
2.663334384	5.245261082	-1.095019749	2.261454048	4.567917193
2.882607568	5.712142856	1.8243113124.584256848	3.574867208
4.884170344	0.922773263	-3.424964346	3.294330407	-1.024562741
2.371697844	-3.249779524	5.589159653	3.296775552	0.431962764
4.709852247	-1.063458764	5.138133919	2.603061864	4.060325541
4.293485733	6.88248924 1.441278431	3.573909472	6.96547424 -4.909181976
-1.885985444	4.62385142 -2.382943102	3.43949222 -4.85759192 5.121773904
1.973053652	2.14281945 3.990578485	6.076212407	2.610021856
2.136689025	-1.692604651	4.765223012	3.930377661	3.897167089
4.424689054	-1.444432166	5.097526902	6.395685676	2.840105627	-
0.0946211555.916474781	-0.182174381	6.050052299	5.020232396	4.705071007
5.528737372	6.374001565	10.46754695	0.718028022	2.71736532
0.619669069	-0.565292519	3.771052518	1.006341956	4.280451525
6.860099861	3.407637975	4.742719174	-1.888384493	2.194444488	-
0.347602692	3.248389286	3.879606394	-2.215976533	5.340548459
4.777830772	1.17520368 3.072251061	3.991331094	5.0007216114.743456289
2.875890644	5.33563939 4.900559429	4.9611696174.04255506 0.832649088
2.366856871	4.617435144	4.872181853	4.760054372	5.467776003
4.898334391	0.894120184	5.578974668	4.173250154	2.104794003
3.842976384	-0.235881795	-4.083173499	3.047467788	-0.489627894	-
3.626788866	-3.564053153	1.5117229842.93791755 4.41567267 5.158863091
3.045092072	6.100907949	-0.594295082	2.794144356	4.310600417
3.7110299263.078783724

TCGA-L9-A7SV-01A -0.629576688 5.147367121 2.265108661 5.10381985
4.377310163 8.112638041 3.803036335 4.058455135 2.382162283
3.321084011 4.900000041 -1.870423137 2.885137835 5.076811638 5.039007307
4.529643904 4.449836885 4.256683567 0.446145573 0.441040759
2.89671089 5.356911051 -1.515514926 1.749578361 4.753857778 2.992701096
5.241941531 3.041728676 4.530093421 4.491016889 5.087980405
1.456672053 -3.306050658 2.70560692 -0.874020572 2.790529726 -
3.879585335 5.598511231 3.436235049 1.626151635 4.677281997 -1.161681814
5.128994557 3.947408449 3.736303533 4.140082476 6.7847211
1.655051554 4.594288019 7.130818437 -4.746641077 -1.534777424
4.544038794 -2.322416569 3.960960235 -4.788386039 5.240918672
1.968564849 2.764817644 4.010397855 5.63806689 2.719692012 1.79754235
-1.563809673 4.568189367 3.931315498 4.196311955 4.316318844 -
2.022217677 5.077066304 6.444660799 2.920762181 0.197617623 5.13626848
-0.790049589 6.265925463 4.855391126 5.042773997 5.306766428 6.54712476
10.09564903 0.460120814 3.187633606 0.951510404 -0.749718831
3.05870173 0.603163147 4.216107786 6.989062527 3.430598689
4.938441173 -1.703921339 2.566661574 0.109009496 3.317322381 4.01493494
-2.229419787 5.345444451 4.123700882 1.666678588 2.891426226
3.617188663 5.050895003 4.606429223 3.928512738 5.335543045
4.656908076 4.895357541 4.267419378 0.59444814 5.012862244
4.590870869 4.745918087 4.70158497 5.624990658 5.000874852
1.213980186 5.310809353 4.080071448 1.590555228 3.930489871 -
1.285414516 -3.531943618 2.82380747 -0.423857918 -3.961159762 -4.247550016
1.090474408 2.557474378 3.789399584 5.797775856 3.366744831
6.488691259 0.469236235 2.797185695 4.454068357 3.813818624
2.33030287
TCGA-L9-A8F4-01A -0.529819811 5.045634647 2.644192863 5.132835363
4.170058053 8.166435497 3.770436251 4.147821129 2.01546098 3.373923372
5.318976065 -2.359732591 3.201710226 4.53404245 4.940829827
4.609646618 4.064645823 4.151793072 0.091016624 0.526999965
2.776647345 5.295329605 -1.352818915 2.068136077 4.636458003
2.715155122 5.625016123 1.314478072 4.599345448 4.091911424

4.914372738	1.178786714	-3.521191067	2.661762068	-1.091032121
2.843557591	-3.277247224	5.579282091	3.060525585	0.977476287
4.699385719	-1.061764369	4.855923176	3.251241088	3.927124857
4.154215943	6.914447506	1.692557226	4.250557157	7.140940955	-
5.026915851	-1.791555572	4.58190775 -2.603360163	3.668512599	-5.13093695
5.384760458	1.947016874	2.182299823	3.974006687	5.853193446
2.66167422 1.748747802	-2.704995915	4.745273741	3.920449507
4.054100214	4.480380875	-1.992631892	5.093027107	6.553018209
2.893745302	0.097922542	5.558930745	-0.61627492 6.20231383 5.002648452
4.979688107	5.428336595	6.394849704	10.28373265	0.505802753
2.726098489	0.694357347	-0.572172756	3.501968973	0.743613918
4.25375367 6.977431598	3.418360404	4.853367806	-1.694932934
2.284442785	-0.644583629	3.275804406	4.04738063 -2.572499348
5.347703079	4.371524124	1.280925618	2.959499139	4.074418367
5.01318991 4.646776786	3.377770188	5.335550191	4.855296945
4.822253007	3.937075304	0.926293791	3.467977651	4.462460013
4.849225228	4.731821456	5.601685078	4.817480083	1.166587166
5.431465582	3.677270568	1.654924699	3.895615439	-0.875329692	-
4.150488598	2.91284896 -0.565475328	-3.852448082	-4.246201976	1.242380768
2.532631236	4.102456398	5.574488242	3.256937843	6.433658769	-
0.092747198	2.873393812	4.452162148	3.763808084	2.717773676
TCGA-MN-A4N1-01A	-0.177943612	5.061999669	2.1175822955.262192764
4.283949621	8.1581113793.599986188	4.146087557	2.148024147
3.330062067	5.444198939	-2.184188852	4.08051534 4.673228535
5.038289833	5.234822168	3.944175833	4.335905871	-0.245236961
0.552245784	3.069820946	5.147663905	-0.808437806	1.731639273
4.649183375	2.941341977	5.395922937	2.748880589	4.744860774
4.324808242	5.157293927	1.605304486	-3.196763818	2.897481184-
0.916599139	2.551145049-3.290534428	5.613399752	3.258533284	0.397562299
4.719348019	-0.951370028	4.923931761	2.901801823	3.968722654
4.210674198	7.067435832	1.484939853	4.413888004	7.511954028-
4.74810583 -1.688477945	5.013088786	-2.378117349	3.576821895	-4.203855872
5.470809831	2.539230379	2.600081412	3.832124374	5.936461648

2.755456749 1.664339397 -1.263041703 4.635908816 3.931520548
4.648433763 4.375759411-0.668303039 5.078220999 6.553895524
2.854527773 0.065532343 5.300528725 -0.451508369 6.280017632
4.895728014 5.215708247 5.5453257 6.319516401 10.28699502
1.009635666 3.012527333 0.994972884 -0.539015333 3.111026921
0.919091569 4.388066691 6.962833805 3.52656197 5.070059393 -
1.758940871 2.857307244 0.083143596 3.347356013 3.792797359 -
1.997430268 5.364389957 3.815635364 1.13742174 2.836639379 3.723099524
5.157500568 4.722633362 3.695276606 5.335759872 4.587388018
5.010562223 4.571762293 1.141529442 3.514332052 4.746406597
4.96841209 4.682367789 5.864868758 4.93316852 1.387492524 5.282499406
3.899217533 1.888850577 4.079205777 -1.234181327 -2.870940298
2.902178151 -0.036592667 -3.733149686 -3.078348963 1.455648162
2.940439067 4.444370547 5.692040504 3.512038781 6.53573505 -
0.44478326 2.546195015 4.754477025 3.768598107 2.453064394
TCGA-MN-A4N4-01A -0.441974064 4.732955722 2.0905071135.243520432
4.384741707 8.198974242 3.222469236 4.192008094 1.67855927
3.170174137 5.338771325 -2.647444927 3.380879969 4.227424531
4.500450256 5.179803377 4.149703818 3.736982233 -0.068749779
0.496551362 2.731329004 5.108200295 -1.078815095 1.844146774
4.537547653 3.001624784 5.313052049 1.347326829 4.593129792
3.564461325 5.136924313 1.095230555 -3.71097855 2.770880887 -
1.009376221 2.883039374 -2.958506714 5.604549904 3.58085981 0.537238754
4.703880013 -1.165891007 4.989031235 3.039351706 4.030900046
4.147856531 6.976738817 1.605535135 3.774964266 7.034297062 -
4.895005916 -1.972868279 5.08268284 -2.765622866 3.222048588 -5.208098461
5.182304336 1.97183154 2.227203337 3.9233751 6.052002834 2.564074517
1.458099776 -2.416253425 4.647922625 3.92194923 3.8564093114.244710713
-1.239181206 5.079757902 6.389098688 2.849758712 -0.054559138
5.357712111-0.569733553 6.108555497 5.081493155 4.887304146
5.454645821 6.280445238 10.85643172 1.072981603 2.496502388
0.335614126 -0.635532857 3.189801897 0.851039617 4.304751991
6.88505479 3.588432771 4.80375013 -1.847228802 2.105731642 -0.626128483

3.268267388 3.73098575 -2.165694311 5.352014375 4.195736396
1.232613756 3.029234214 3.944623669 4.955840813 4.809014266
3.278749227 5.335321663 4.604837443 4.9118498873.86510427 0.913315905
3.238736866 4.503224532 4.879852519 4.716791289 5.654668518
5.109789752 0.682962349 5.651200214 3.736699956 1.926895495
3.949358866 -0.358249407 -4.26803734 2.22307118 -0.396974717 -3.630471236
-3.824212729 1.510980932 2.800338476 4.334560871 5.218210426
3.384864513 6.185138887 -0.092898675 3.177834703 4.431654574
3.596854984 2.946116347
TCGA-MN-A4N5-01A -0.055304572 4.882464212 2.528814468 5.184227182
4.384619457 8.17840856 3.548718188 4.288196135 1.757749917
2.942959903 5.11409305 -2.437310387 3.360604133 4.455646512
4.944455871 5.057624339 3.685560525 4.137148487 0.284160919
0.515792093 2.667586719 5.099945517 -0.916494611 1.817267598
4.581425918 3.226876139 5.531677829 1.245337974 4.618715405
3.745589786 5.089576076 1.011865877-3.375442267 2.861379494 -
0.963203618 3.144700402 -3.105229121 5.609358242 3.596622506
1.513193708 4.709231661 -1.099812942 5.209024339 2.82867374
4.028646781 4.152199537 6.910433035 1.59218015 4.234708958
6.764033254 -4.403376601 -1.95793035 5.01241437 -2.28727473 3.386736388 -
4.899064354 5.205005501 2.027298625 2.132553932 3.9492117335.918459995
2.555760319 1.600305476 -2.2133416 4.760216618 3.93589467 3.872207677
4.281365038 -0.563034914 5.084935582 6.272178224 2.908945459 -
0.086471581 5.492399929 -0.365959498 6.143170799 5.0191087114.865281255
5.444645268 6.364997185 10.54903378 -0.126251717 2.86177377
0.781386254 -0.746792965 3.32920347 0.944263693 4.309786796
6.8679711973.579649243 4.763119326-1.791619943 2.500551081 -0.461189812
3.247468622 3.616821378 -1.823450392 5.361265053 4.346702214
0.602190122 2.942793559 3.885563343 5.0285411084.946525133
3.446440428 5.335601827 4.698348235 4.918477037 3.873748906
0.960419383 2.89903964 4.558188492 4.927333533 4.731598637
5.629697315 5.233578156 0.751753099 5.642299087 4.157734135
2.080639973 3.990994127 -0.175792098 -4.02992459 2.467555856 -

0.129312989	-3.465997302	-3.960595216	1.609553528	3.0115447694.348011806
5.489580643	3.619352665	6.483271045	0.0911321922.936253603
4.385145153	3.680621896	3.134244003
TCGA-MP-A4SV-01A	-0.591974674	5.074410623	2.3404118365.302432281
4.320655173	8.132736494	3.682417772	4.226574666	1.926039619
3.303702802	5.063530992	-2.2232228	3.479640573	4.6679211265.02688851
4.66859943	3.850553723	4.03387255	0.389363499	0.437144308	2.597383791
5.170583391	-1.369106779	1.991374096	4.647953651	2.668414873
5.732058416	1.470869018	4.536415173	4.220475366	4.855348722
0.979528767	-3.483156336	3.045567594	-0.782766477	2.585178232	-
3.7437113995.597498948	3.406486672	1.151464264	4.688584813	-1.119330423
4.951258288	3.166056095	3.873090026	4.221335338	6.740644199
1.542865698	4.207220523	6.974116642-5.086089202	-1.927477019
4.922923686	-2.124961221	3.328076578	-5.049178939	5.198813236
1.941537933	2.19664678	3.997489899	5.786097631	2.504863378
1.826713346	-2.660981762	4.73612857	3.937737918	3.773186935
4.529874269	-1.722073291	5.083876615	6.320803082	2.868351095
0.060140252	5.675246639	-0.59619862	6.17314688	4.942360032	4.771323764
5.444927064	6.358712564	10.14328537	0.14707289	3.005220147
1.012664039	-0.803904692	3.482733485	0.852571077	4.276140415
6.879457493	3.357725165	5.040328847	-1.758953921	2.605431121-
0.539349085	3.227297187	3.776186226	-2.289804463	5.355585858	4.32942332
1.192717339	3.005277732	3.706652233	5.002052559	4.816898217
3.17573329	5.335509074	4.539145743	4.878574412	3.865379522
0.7111842062.961575252	4.471549054	4.762478691	4.747880372
5.5311639284.569827185	1.089122393	5.349867942	4.181781558
1.717946784	3.879237915	-0.654806298	-4.10319436	2.577059431	-
0.226824194	-3.732416976	-4.15890738	1.242320774	2.737580736	4.09538235
5.5411062543.50596474	6.357253304	-0.147109397	2.476954271	4.320537128
3.655620554	2.80864077
TCGA-MP-A4SW-01A	-0.283515953	4.84592795	3.135946186	5.209526288
4.375851753	8.215893653	3.209604274	4.292732707	1.766958856
3.133559101	5.471325325	-2.388444905	3.922704004	4.401517629

4.836450647 5.394404298 3.744568179 3.729806432 0.278891135
0.601997106 2.855197727 4.846394049 -0.938467118 1.9793099
4.520499899 3.333649745 5.733509486 2.82819778 4.60265651 3.309126874
4.966479771 1.01903155 -3.445288899 3.348179633 -1.260482273
3.105233889 -2.464208666 5.600887293 3.483983303 1.182871537
4.718970637 -1.021659627 4.979594414 2.7002115624.187643534 4.24772007
7.03672305 1.772540747 3.998082171 6.856884406 -4.313970736 -
1.869104166 4.706534831 -2.233506202 3.303149194 -4.601790397
5.275897582 2.362014748 2.355769218 3.859484047 6.16169133
2.840242721 1.869539406 -1.962283244 4.646062476 3.932400427
4.1150623054.435683224 -0.93668973 5.087044245 6.34619888 2.953169059 -
0.246248572 5.950149859 -0.279741527 6.141319777 5.106065559
4.769803617 5.550781662 6.149000927 10.30890694 1.143378858
2.735059593 0.633660424 -0.672728079 3.628890874 0.945211109
4.321686581 6.894290107 3.742866971 4.905556179 -1.72015955
2.498382997 -0.0802608 3.269274796 3.570794957 -2.36077955 5.362396182
4.193657479 1.107317982 2.9813119434.136387818 5.031770766
4.782288362 3.357205422 5.335507138 4.960586459 4.910092208
4.345098921 0.927395095 3.052631084 4.848168449 4.898871095
4.724072636 5.801615205 5.21436778 1.03890902 5.790262185 4.06246577
2.012749294 4.031330441 -0.727943292 -3.603366383 2.637156139
0.167656952 -3.26429941 -3.611496636 1.981713608 3.045023944
4.696325773 5.396131356 3.306175197 6.279449233 -0.150280787
3.306694093 4.706170566 3.825289069 3.245556218
TCGA-MP-A4SY-01A -0.811698139 4.648157322 2.665522049 5.394963465
4.165889442 8.235483343 3.640328955 4.283783159 1.725776321
3.262042329 5.183196673 -2.432796001 3.302621712 4.408981852
4.707410906 4.942400421 3.3298411263.99282362 0.644970741 0.328486542
2.501643297 5.255963819 -1.491953866 2.349829609 4.565929335
2.818553553 5.423422843 2.413509401 4.493673277 3.646168178
4.994100053 1.070395514 -3.850850816 3.562603524 -0.787392467
2.552145155 -3.414222017 5.6207111833.494797387 1.155093194
4.713659993 -1.282532928 5.125126083 2.766605792 3.962539933

4.129610377 6.90655183 1.22928106 3.550227957 6.5576752 -4.743461206 -
1.8661384 5.141307547 -2.295898628 3.083243201 -4.632402831 4.992928378
1.939160843 2.396037988 3.759604405 5.842552315 2.470459731
1.973856419 -2.057372529 4.698339586 3.943726044 3.977841459
4.266436087 -1.989613319 5.0958011316.317915258 2.848188372 -
0.020777012 5.66599025 -0.962477216 6.039960696 5.0011966154.59392433
5.446346459 6.342539769 11.147790250.713034009 2.562470078
0.821958788 -0.729232875 2.945860546 0.920854436 4.293327346
6.833647741 3.4166029114.585610949 -2.205741199 2.21036743 -0.255563403
3.264683953 3.708948728 -2.3697896 5.348481257 4.55170734 0.590375256
2.783885444 3.598335795 5.012357958 4.968655439 3.04292387
5.335577784 4.658671056 4.905657261 4.074406896 0.503269372
2.350867043 4.274413313 4.802233939 4.764638047 5.571809569
4.839614334 0.790607751 5.300564356 4.129605328 1.974549379
3.834326113-0.561439467 -4.054434283 2.993703326 -0.19373159 -3.474893314
-3.458502244 1.513343689 2.873846491 4.174522296 4.959893563
3.481054738 6.187264818 -1.060186176 2.672522935 4.255879642
3.531830893 2.936098868
TCGA-MP-A4T4-01A 0.07080479 4.908201743 2.593608578 5.292166498
4.223512599 8.1760231153.659109584 4.248710842 1.702023201
3.3526461175.28546353 -2.488864852 3.732962537 4.293726314 4.954248773
5.058393952 3.673891959 4.006796721 0.359489018 0.548373086
2.671777764 5.068238775 -1.037824503 2.309136289 4.565919292
2.966970313 5.560389683 1.906972379 4.5973137113.849084103
5.015555995 1.031564922 -3.448415153 3.295780876 -0.943056962
2.762005874 -2.921124342 5.604130744 3.35847919 1.162053628
4.707029381 -1.116656432 5.102840816 2.772514306 4.014696934
4.153700852 6.8631601121.663453336 3.77440683 7.089322785 -4.633882698
-1.892595167 5.119902291-2.332238261 3.163864041 -4.917096572
5.204035903 2.066479774 2.343953812 3.803439079 6.009063874
2.678447662 1.891979503 -1.980633904 4.796127754 3.928208069
3.856604996 4.487727523 -0.994118332 5.095165896 6.296028298
2.799944464 -0.018989931 5.588245476 -0.32216112 6.122986868 4.98352971

4.742923291	5.454776786	6.283015535	10.9315926 0.59583243 2.794159409
0.691311936-0.585179367	3.829572845	1.006193764	4.306609818
6.904746409	3.412473873	4.891022453	-1.736036473	2.405164941	-
0.564068775	3.255767174	3.792627755	-1.949513891	5.344914903
4.330160555	1.0697328112.9571171543.746412503	5.020728744	4.905281189
2.943514733	5.335613535	4.597181084	4.958765305	3.904681725
0.940908994	2.678857414	4.5861911634.966639718	4.754092875
5.563308257	4.86477887 0.938075921	5.362012384	4.014294523	2.14220328
3.90017336 -0.263990585	-4.185111812	2.662963382	-0.080702924	-
3.598718114-3.652166859	1.582427804	2.937668328	4.425284804	5.280989174
3.317361274	6.293601771	-0.323621401	2.890881803	4.276247058
3.640178519	3.249482123
TCGA-MP-A4T6-01A	0.431808497	5.1958361172.640219521	4.983731177
4.387313388	8.014146258	3.881031207	4.040955558	2.262753106
2.82189148 4.454478529	-2.442226177	3.849481072	4.495614083
5.284690917	4.9311665483.845914267	4.600675497	0.267519038
0.492271017	2.959246287	5.105453991	-0.699981859	1.440162562
4.633930805	3.263356757	5.605347609	3.363345654	4.69331475
3.979972405	5.162499589	0.994174993	-3.104726732	2.826404682	-
1.395096879	3.712939717	-2.954821482	5.5839776	3.010449457	1.340322439
4.711928841-0.936182625	5.293127232	3.150777315	4.047488414
4.170170053	7.175180563	2.281313201	4.920050409	6.973135669	-
4.421469877	-1.859819477	4.717066429	-2.019647639	3.309650576	-
4.897642459	5.471067946	2.682751478	2.308662049	4.170236821
5.844394999	2.743963171	0.911923697-2.138909121	4.742939037
3.927652947	4.294755026	4.378747836	-0.29337768 5.085230938
6.3511723613.158013943	-0.006033386	5.400073964	-0.178046016
6.210148204	5.037100847	5.072927862	5.4311503446.303789702
10.04393579	-0.063045632	2.974704609	1.444321412	-0.981160721
3.660873	0.708286676	4.3258411226.984270078	3.597987809	4.902197986
-1.560701271	2.641826724	-0.335336635	3.230259842	3.577108599	-
1.498639973	5.379136997	4.159644477	1.128559519	2.801375538
3.8987111325.1120825564.841584753	3.759701753	5.335662501	4.714252445

4.858446379 3.9551139231.073763668 3.990269517 4.698849848
4.969612877 4.7112994435.639623268 5.068957623 1.269103765
5.163153177 4.1178818972.187442672 4.228453363 -0.821395819 -
3.162773937 2.629385022 0.211682166-3.494448416 -3.729549298 1.391196213
3.199787378 4.261678694 6.026924026 3.918243312 6.783728278 -
0.139396968 2.784992224 4.710452847 3.893076731 3.134256559
TCGA-MP-A4T7-01A -0.605108038 5.279487181 3.593646646 5.279370434
3.851837486 8.233577838 3.347516166 4.337743886 1.877879943
3.108912219 5.480537351 -2.209956268 3.434741574 4.155344822
4.674936367 4.853703095 3.52723404 3.964539183 -0.014302452 0.33364161
2.743013974 4.878181555 -1.160257369 1.6514110654.5326115593.032294084
6.004209742 3.033376636 4.509206816 3.268188071 4.865017421
0.720331567 -3.656769347 2.696944231 -1.636818253 2.453799114-
3.087403867 5.583848599 2.847971673 -0.23404439 4.713680285 -1.07574066
4.519449244 2.665623107 4.091797084 4.307992848 7.027309395
1.279207104 3.977951898 6.824358058 -5.028233809 -1.845315569
4.880378621 -2.590061265 3.347403197 -4.936907535 5.171812685
2.03625991 1.9711116663.925686827 6.00648043 2.582851265 1.910222126 -
1.628070473 4.628097249 3.928368544 4.298773158 4.167849949 -
1.420550051 5.091243429 6.430349992 2.981008094 -0.289040932
6.046934799 -0.65741886 6.191776986 5.067128097 4.851940164
5.489788871 6.3114929069.854571814 1.2991610112.45984439 0.387309642 -
0.567230843 3.145078996 0.891307918 4.335058924 6.869186892
3.551990812 4.635195761 -2.204453295 2.101472145 -0.262381273
3.205086252 3.86355814 -2.434108676 5.359901581 4.3823841130.527957313
2.590669812 4.322973163 5.044383888 4.637924889 3.112095976
5.335628031 5.128562849 4.733433182 4.100835761 0.653977812
2.316035268 4.777262773 4.835373019 4.720179484 5.725018646
5.0183891141.0269754115.561202984 4.266473018 1.707380106 3.982600923
-1.183093321 -3.942452605 2.431480366 -0.473119368 -3.721700821 -
3.748961023 1.573171763 2.98116336 4.435688932 5.478445227 3.192207583
6.096491258 -1.302648887 3.068127317 4.515556606 3.700888016
2.518824719

TCGA-MP-A4T8-01A -0.883309669 4.597140284 2.969549748 5.40245639
4.068345742 8.206299331 3.314401795 4.233357176 1.744803993
2.991019274 5.217672344 -2.397852736 3.4954101114.207700266
4.535602176 4.904831799 3.790123585 4.102586045 0.477328437
0.491827883 2.548831298 4.922318395 -1.643125681 2.194398237
4.593740704 2.919718318 5.43046331 2.471235024 4.376365878 3.36533986
4.937065836 0.966729476 -3.804255757 3.4011462 -1.192162571
2.562209062 -3.333688711 5.6118284212.998933925 0.259354385
4.686228577 -1.243865388 5.023793025 3.1164371763.985366498
4.196350833 6.756493365 1.432365616 3.912700962 6.900818125 -
5.18845844 -1.90034442 5.141260857 -2.165574337 3.088873144 -5.338220471
5.1185781391.9495113161.889833248 3.795160365 5.829203001 2.451600659
2.164030934 -2.506226609 4.636503108 3.927725289 3.990147692
4.356762873 -2.899893058 5.071787078 6.36199808 2.907649178 -
0.1043116595.555860591 -1.169774253 6.2332942114.995896285 4.932556061
5.499673015 6.474599831 10.61297681 0.5251177972.577440383
0.739288352 -0.773756655 3.236853135 0.794443976 4.299615251
6.886105572 3.581277578 4.8827142 -1.848679878 2.132174369 -
0.765534881 3.25188366 3.903433159 -2.77004169 5.353819838 3.945458277
0.939001814 2.769498958 3.591023523 5.012556162 4.818465645
3.191556483 5.335400422 4.572197985 4.80994393 3.850576547
0.612745003 3.145350846 4.564270892 4.699084717 4.669826781
5.551651625 4.775088712 0.99887091 5.36354646 3.781969937 1.378316983
3.858645137 -0.256329937 -4.676940226 2.287981922 -0.49508428 -
3.707997807 -4.099315863 1.378152132 2.580643105 4.264771469
5.254916488 3.197940263 6.283488204 -1.034787205 2.584557011
4.3110739193.694600053 2.367333434
TCGA-MP-A4T9-01A -0.067027603 4.912892912 2.733072549 5.355312074
4.176273226 8.195646875 3.538195946 4.24631193 1.869576377
3.280951516 5.264289472 -2.557645253 3.683624615 4.383792928
4.822973044 5.029409714 3.476140631 3.907629928 0.749251524
0.586366747 2.737137779 5.210191737 -0.942836337 2.073537472
4.578987107 3.182987247 5.702155442 1.918659937 4.654480149

3.801202412 5.009856764 1.039835078 -3.389144276 3.26877565 -
1.100921515 3.11968341 -3.111300104 5.592145159 3.30140331 1.409490968
4.715987569 -1.069316571 5.289820247 2.847845201 4.015664607
4.249153262 7.025582606 1.726540488 3.808716205 7.015979507 -
4.608239994 -1.983008652 4.743473793 -2.419777546 3.292095758 -
4.901996041 5.161566972 1.912816626 2.390733614 3.889810875
6.052049266 2.616307433 1.777092654 -2.181469003 4.72974385
3.934030038 4.079539771 4.388772634 -0.670282861 5.091848538
6.340054534 2.872516016 -0.05692219 5.762357137 -0.180235926 6.04497459
5.064896274 4.798597859 5.484521562 6.328673999 10.64596711
0.823695431 2.722515534 0.685339935 -0.682634822 3.406874071
1.045814783 4.32743802 6.8733305 3.535217926 4.729672361 -1.851293268
2.303949516 -0.549234022 3.249066927 3.728134933 -1.941423208
5.351791434 4.687534107 1.004843454 2.913992466 4.10012512
5.015418648 4.743840803 3.065937421 5.335603104 5.017601698
4.971823443 3.904126122 0.849634358 3.019328625 4.675678562
4.873449374 4.768954249 5.622345516 5.134532585 0.769704254
5.581204281 4.103697419 2.170073728 3.941600599 -0.235249923 -
3.8760495112.757261195-0.255250606 -3.456314509 -3.682345513 1.63115897
2.936667452 4.5232951135.339275979 3.179589876 6.225723765
0.477636029 3.196581526 4.443307366 3.666596555 3.175421773
TCGA-MP-A4TA-01A -0.50366665 5.179036244 2.896093678 5.304525799
3.952350412 8.139634635 3.333445348 4.2040155 1.864584096
3.307664419 5.257630018 -2.544278019 3.620423832 4.536098934
4.950725163 4.910701679 4.054478548 3.992948178 -0.339974253
0.295006626 2.828983137 4.518025127 -1.191568033 1.846430569
4.577022817 2.716020165 5.681486517 1.809984153 4.621284972
4.351542978 4.905399177 1.09654553 -3.721753667 2.566276122 -
1.405028468 2.427360498 -3.220059603 5.595732712 2.514940807 -0.3377631
4.705381315 -1.035950039 4.782829505 2.78900622 3.972283541
4.223980719 7.001297404 1.214826129 4.141835913 6.896384416 -
5.476960092 -2.101357846 5.055824407 -2.678485273 3.408922267 -
5.124138489 5.258716787 2.042084207 1.854664267 3.840062864

5.859784496 2.481873561 2.068105807 -2.443143987 4.615247463
3.93605828 4.196547997 4.393680666 -2.047328772 5.0859638 6.331323085
2.920887616 -0.120115184 5.416155932 -0.906779228 6.266832358
4.993832974 4.896062872 5.543559354 6.190190512 10.12182699
0.457521544 2.6373771 0.722744413 -0.579176181 3.149691273
1.056433381 4.301034273 6.89977321 3.568895593 5.008095091 -
2.13288952 2.736203924 -0.502416036 3.2824116943.776013467 -2.284838891
5.367462912 3.637739326 0.754436351 2.744432257 4.047793704
5.074669172 4.714034836 3.234839609 5.335583015 4.822616858
4.843393175 4.124083001 0.924081788 2.839787824 4.719719635
4.93425431 4.708051712 5.853520972 4.957826146 1.1158951375.266999083
4.291974046 1.609608441 3.919358533 -1.508584272 -3.952060035
2.172204936 -0.387368758 -4.088146447 -3.875102966 1.270969485
3.027450525 4.464647767 5.600892649 3.435567588 6.481118163-
1.434795738 2.566633426 4.597518417 3.7253511932.785977494
TCGA-MP-A4TC-01A -0.178534788 4.687524847 2.1104016165.390939959
4.268834803 8.309901936 3.426765977 4.29965953 1.709684717
3.410021373 5.352964539 -2.458974258 3.775540184 4.573297336
4.784677504 5.202935827 3.7118962463.860043137 0.090586781
0.520931207 2.6811357375.228070169 -1.056659165 2.274122564 4.54080182
2.861988529 5.371792886 2.10232245 4.593390585 3.546357789
5.123267335 1.227710216 -3.627589754 3.315572724 -0.799361611
2.311355509-3.168777596 5.610792746 3.677991464 0.599244006
4.719341977 -1.118688192 4.943565824 2.72181644 3.978895809
4.228426052 6.914204724 1.334623781 3.332371052 7.247837255 -
4.865321321 -1.820174249 5.123686049 -2.624607945 3.358937889 -
4.473774608 5.164309452 2.299942823 2.743351755 3.749727256
6.069312435 2.496963995 1.940100367 -1.689885754 4.688323386
3.932231452 4.070464019 4.300512941 -1.438988188 5.080585391
6.475500446 2.714546028 0.086065009 5.514031036 -0.41343732 6.12413069
5.007768614 4.878452167 5.500079042 6.29911155410.88262513
0.969617319 2.86066372 0.643583543 -0.47029651 3.345373562 1.094380985
4.356944408 6.891728545 3.358062261 4.804023963 -1.93093071

2.335735562 -0.299884691 3.309499952 3.87226666 -2.208357597
5.338010041 4.385735971 1.068265016 2.973043624 3.505724313
5.037544954 4.848837687 3.063137787 5.33557986 4.504714199
5.1128930224.254307781 0.894072509 2.786693594 4.51296309 4.957920814
4.738134551 5.5361135944.880573295 0.975740857 5.422075249
4.347790495 2.0311164383.890501414 -0.590994419 -3.71011397 2.968273824
-0.179339305 -3.5962391 -2.937680692 1.731672016 2.938095646
4.493739136 4.949993934 3.15506971 6.065416948 -0.368176456
2.931714262 4.46053673 3.646750566 2.980271628
TCGA-MP-A4TD-01A -0.67706124 4.730358701 2.849007402 5.204506174
3.712821907 8.299748166 3.814140902 4.244340429 2.087373844
3.535074985 5.280165758 -2.117514124 3.464177944 3.915074315
4.750619157 4.876699131 3.70886965 4.612915197 0.199491556
0.290648722 2.948027024 5.203632776 -1.389513498 2.486499201
4.636966256 2.859871259 5.624727559 3.562328175 4.609613094
3.818425669 5.0061121381.185747673 -3.586389368 3.36214825 -1.320257019
2.456877195 -3.106890168 5.583173768 3.03003629 -0.006421269
4.704712394 -1.143530122 5.09350329 2.850132056 4.027815904
4.253394429 6.844065722 1.712363906 3.896799352 7.025514759 -
4.836492127 -1.56072109 4.139073015 -2.355833181 3.560047028 -4.734099787
5.151844345 2.580258438 2.003205241 3.985013077 6.069821486
2.738754181 2.355675555 -1.374116368 4.704723793 3.922372855
4.5118505154.19943295 -2.463783672 5.098528033 6.726314181 2.979078528
-0.11514966 5.838686048 -0.673857164 6.082752052 5.0253981154.94699393
5.51169437 6.404126758 10.0150428 1.188690908 2.489038273 0.987501919
-0.614677161 3.373766915 0.686979557 4.278474026 6.941991919
3.628602618 4.632511853-2.052710107 2.208925437 -0.353383819
3.349994741 4.191920403 -2.615602759 5.335009765 4.634733449
1.421259594 3.026895582 3.712698289 5.051813648 4.597493791
3.522477378 5.335801691 5.04159536 4.919841852 4.394588855
0.780796878 3.709445842 4.561253766 4.821883508 4.698714674
5.733628862 5.15601483 1.335963437 5.392722064 3.432814772
1.7050306113.919056437 -1.019946097 -3.830325355 3.112148619-0.730810212

-3.750252332 -3.463911898 1.439466826 2.8952115014.188228155
5.266654143 3.123868351 6.219200138 -0.885027215 2.621005146
4.425407963 3.809663927 2.298345137
TCGA-MP-A4TE-01A -1.289539294 4.792495344 2.097382506 5.285402407
4.353591803 8.339581459 3.644639455 4.203626891 2.361832572
3.393779421 5.638181721 -1.851449948 3.78995847 4.422297887
4.790859263 5.23978359 3.667255535 4.78503058 0.321269383 0.391875193
2.936664783 5.075777956 -1.518809108 2.721430703 4.657223668
2.6520557 5.548729814 4.608836355 4.636806216 4.39322917 4.879123402
1.248655068 -3.574024823 3.501150475-0.986053958 2.274423294 -
3.19964276 5.612098495 3.341110609-0.405195275 4.703696007 -1.127821183
5.224097542 3.064519152 3.997292249 4.356776694 7.126249045
1.501507914 4.435876763 6.601993457 -4.81368904 -1.384861848
4.617511787-1.965918418 3.375736443 -4.344584467 5.235610636
2.595846819 1.733835685 3.974246038 5.995826088 2.97159627
2.439194284 -1.351922896 4.449836176 3.930089939 4.725584993
4.199264373 -2.558772023 5.072984224 6.704806613 3.145887352 -
0.191642731 5.763086491 -0.855993915 6.158553264 5.084021856
5.141753108 5.536692521 6.484142536 9.942929351 1.458831926
2.734316442 1.337043416 -0.871814553 2.831899261 0.564321607
4.409579146 6.887570836 3.809734467 4.824286275 -2.056624224
2.6364460110.089182518 3.377041483 3.975058619 -2.99225801 5.354540159
4.1107050220.830735096 2.857544725 3.825232933 5.073121422
4.606946267 3.779445902 5.335576272 4.993471635 4.825167032
4.648070691 0.452795427 3.76089583 4.330629088 4.573883339
4.660658035 5.964846246 4.989849954 1.561294755 5.378966014
3.571826202 1.414205767 4.066704587 -1.339199658 -3.396443155
3.206493817 -0.251675986 -3.459011169 -3.146696303 1.536748939
2.67557783 4.222445018 5.770978888 3.855407232 6.287959977 -
1.459787477 2.088403791 4.677866173 3.876858901 1.835086288
TCGA-MP-A4TF-01A -1.068535223 4.723993245 2.774706491 5.228289066
4.183083339 8.239264772 3.676599765 4.205722037 1.848760301
3.139917066 5.309236854 -2.741552183 3.290884309 4.537106109

4.593664578 4.947465784 4.03539114 4.154036772 0.481909159
0.261522505 2.621926475 4.971442815 -1.500228111 2.161152592
4.637264186 2.601338663 5.722817512 2.489761985 4.520960687
4.070259903 4.920327888 0.942126625 -4.018494903 3.001941027 -
1.24313914 2.903416837 -3.460062736 5.624917184 3.3138113460.41250311
4.70183182 -1.307272988 5.310939402 3.1164441263.883762182 4.080235967
7.0361168991.429050838 4.320244931 6.5013874 -4.771097706 -1.932870431
5.690385186 -2.23259927 3.187320622 -5.261980452 5.037789403
2.043761382 2.034407921 3.645148056 5.744624136 2.63756304
1.931876191 -2.594890713 4.622245658 3.943743096 4.016576042
4.269538592 -1.699893692 5.081496688 6.081809697 2.987318206 -
0.086785257 5.212523374 -1.185581555 6.181879346 5.100307748
4.827255219 5.357332194 6.405989917 10.86052042 0.220345651
2.570729392 1.056661773 -0.741315243 2.856450937 0.770224855
4.318683058 6.793253427 3.639638798 4.868440233 -2.102633002
2.382084117-0.600825964 3.238405664 3.705153275 -2.606832601
5.369745443 3.982560077 0.366089283 2.710636672 3.694676847
4.997278779 5.054156779 3.317669806 5.335421022 4.593000028
4.761044438 3.594043725 0.53853546 3.017831834 4.457547615
4.795451406 4.729541843 5.607915676 4.939151589 0.593609171
5.14903137 3.501797029 1.489992676 3.995396523 -0.375054998 -
3.900618404 2.045578895 -0.183741408 -3.689991661 -3.786378117
1.255367045 2.751264443 3.9915411525.538704655 4.202448641
6.353022606 -0.653041764 2.457455899 4.582884431 3.432665729
2.708920134
TCGA-MP-A4TH-01A -0.270551095 4.891472393 3.136436901 5.198893505
4.096660501 8.292541942 3.567039821 4.280008136 1.826618594
3.01349911 5.128117487-2.493787628 3.266842956 4.469641389 4.83506521
5.022989734 3.650161407 3.881001372 0.77807858 0.543507902
2.668139651 5.287768329 -1.139211178 2.146693342 4.56409318
3.174413995 5.806783464 2.166558635 4.527121604 3.173280099
4.908354454 0.727651179-3.599861422 3.361282603 -1.305452777
3.001492972 -2.969354651 5.574472243 3.715258879 1.325454003

4.716614673 -1.126879397 5.063306444 3.087146571 4.092763472
4.308232342 7.033385978 1.790976933 4.031647147 6.633318878 -
4.477498166 -1.717009916 4.58826136 -2.426084876 3.1756561 -4.856901581
5.148570574 2.10752577 1.85132155 4.060835003 6.065790135 2.823759909
1.725973628 -1.944948428 4.732369383 3.935554959 3.989606267
4.252894958 -1.216465246 5.088821709 6.404982517 3.040253756 -
0.225819708 6.091825458 -0.227060509 6.03132727 5.194588249 4.727840694
5.455562488 6.35839014 10.46244903 0.865077722 2.641364852
0.573516971 -0.844525744 3.582813948 0.808979444 4.286774751
6.906318261 3.679159901 4.575820495 -1.776037468 2.153520005 -
0.529300678 3.202526247 3.709829853 -2.17540702 5.359279139 4.814788784
1.246996414 3.046836595 4.23386273 4.979374927 4.76591372 3.132941395
5.335568624 5.208010834 4.83287048 3.935641496 0.712581207
3.222739643 4.578112927 4.795046775 4.757958558 5.643721846
5.170837834 0.885603048 5.733441175 4.252359746 2.06304875 3.995458504
-0.383605971 -3.842117188 2.751958201 -0.186686549 -3.302635883 -
4.090486791 1.658815272 3.083843326 4.331047595 5.358762837
3.287623051 6.214048558 -0.014036987 3.281936744 4.51803736
3.738126115 2.787292749
TCGA-MP-A4TI-01A -0.317265305 4.628524456 2.419332445 5.273632845
3.831230841 8.213010722 3.730458319 4.357568642 1.514661702
3.58777027 5.404188468 -2.68777909 3.655393805 4.213807251 4.817609597
4.82499842 3.589215951 3.87610034 -0.29186776 0.339957797 2.517488217
5.065580375 -1.309299584 2.694020268 4.523219374 2.683979113
6.050995182 0.488839517 4.569518429 3.57818799 4.967143624
1.040929209 -3.447765776 3.168269994 -1.023310917 1.998680692 -
3.436371369 5.598310167 3.081867827 -0.037952404 4.709888555 -
1.075828851 5.013952428 2.256547914 3.989475453 4.188088992
6.717807129 1.226542754 3.327482255 7.004894955 -5.213770361 -
2.047639774 5.111582803 -2.470406806 3.268723283 -5.243150198 5.110465708
1.993567401 2.094990233 3.723016279 6.100215685 2.335508572
2.23371747 -2.242221898 4.834914934 3.92691835 3.584930488 4.400924401
-2.130351355 5.101738074 6.408327352 2.68633899 -0.025967721

5.866282823 -0.389207005 6.056186835 4.933897833 4.726119114
5.510846164 6.217009698 10.80320639 0.446746334 2.778994244
0.656845056 -0.439987165 3.703013957 1.079629841 4.2608472116.858062
3.165634448 4.796824379 -2.034954966 2.250771007 -0.919792392
3.247943887 3.922361895 -2.65875342 5.336365924 4.506675799
0.959852454 3.144903027 3.520427636 5.010872046 4.898622528
2.769947598 5.335725972 4.435120201 5.092424687 3.796218143
0.970231561 1.781035667 4.560150733 4.992420461 4.755178028
5.489302785 4.7421105840.929358368 5.287280974 3.855171595
1.955672364 3.813331446 -0.346160397 -4.581072187 2.587270466 -
0.325883731 -3.842923252 -3.521973667 1.571269805 2.744165589
4.4070111954.904035704 3.109922066 6.037607322 -0.871998263
2.458406978 4.262817436 3.610020351 3.166358937
TCGA-MP-A4TJ-01A -0.040609097 4.952258164 2.407866016 5.177017086
4.033786024 8.286973022 3.902731099 4.265863029 2.021320853
3.772039249 4.982371977 -2.264510245 3.535017228 4.534460101
5.0117334694.691691876 3.675096548 4.063231748 0.894756476
0.495453146 2.701058139 5.603142 -1.17660915 2.392154015 4.639230636
2.719440935 5.972233925 1.837336816 4.594388781 3.591383053
4.745863073 0.918060668 -3.522936638 3.401771674 -1.034439482
2.545146182 -3.338550292 5.570746234 3.231471675 0.593085968
4.709322775 -1.052492906 5.235914845 3.0011230734.035413804
4.406507415 7.022354578 1.744908474 3.912368315 6.966616804 -
4.937366876 -1.637341203 4.496556315 -2.258748259 3.587196045 -
4.703662431 5.142333674 2.121017876 2.175143448 4.247550894
6.055571076 2.766142545 1.753859314 -1.63860435 4.767697907
3.932914385 3.991053439 4.399613541 -1.562393587 5.093101268
6.490946395 2.943451144-0.042366689 6.055449123 0.0679802 5.978027989
5.167226537 4.677341924 5.428422881 6.420996732 10.20194477
0.55940233 2.772427308 0.931007411-0.66193628 3.862882354 0.864931545
4.192629213 6.883158876 3.495688242 4.633231467 -1.790308691
2.220878644 -0.356662609 3.245844056 3.925938259 -2.14679694
5.342799095 5.123955751 1.656899327 3.341481376 4.036494677

4.955252391 4.703072061 3.019657867 5.335540465 5.104879392
4.953903163 4.143570891 0.77005759 2.99479149 4.57545789 4.77109446
4.793696089 5.5336053114.968770263 1.012247528 5.431086405
4.265919066 2.069682018 3.893823697 -0.314272231 -3.897537818
3.300549174 -0.476598281 -3.545511598 -3.493782 1.39106758 2.918862357
4.172802651 5.412551869 3.3568271156.15885669 -0.293240189 2.76051942
4.499825737 3.801300129 3.147298296

TCGA-MP-A4TK-01A -0.516655706 4.725508932 2.23293304 5.317501518
3.92290493 8.274168066 3.850109535 4.279806074 1.863556408
3.591336939 5.31702874 -2.457594349 3.774946774 4.20348416 4.89200436
4.956989661 3.515642731 4.260804716 0.22167572 0.539786877
2.706210357 5.291776896 -1.226750269 2.420217198 4.552992318
2.924980466 5.623738135 1.898234601 4.608705986 3.778139499
4.9401185551.204151605 -3.578592193 3.341809732 -0.954845682
2.635524698 -2.837625929 5.588425954 3.376216563 1.04946077
4.720801415 -1.080792226 5.131393936 2.671694726 4.027181053
4.193633548 6.953894096 1.681003886 3.373789376 7.012341464 -
4.588270507 -1.779108366 4.576158271 -2.43643637 3.347083164 -4.41839772
5.144725065 2.434379154 2.571218509 3.884043309 6.086705904
2.790350452 2.05985417 -1.651539574 4.757469363 3.933715827
4.232603851 4.324639492 -1.70785001 5.095447578 6.431968847
2.790643101 0.097221272 5.731104854-0.435546393 6.025003515 5.03091082
4.785483385 5.489808429 6.349634529 10.87321444 0.835208255
2.703369951 0.864428575 -0.475863599 3.717718782 1.011178255
4.306338187 6.886467064 3.509232383 4.758161862 -1.856345782
2.281934314 -0.320879577 3.305228538 3.950300352 -2.389274566
5.333258477 4.818836983 1.349764123 3.11876993 3.924520459
5.015717401 4.7978611382.99723748 5.335688273 4.818954762 4.990398073
4.137415209 0.908342134 2.831552707 4.575849556 4.869764371
4.746171907 5.458044533 4.942507674 1.07548262 5.517955497
3.827739463 2.017983408 3.898161256 -0.348577011 -3.714428733
3.008567358 -0.103177399 -3.497336779 -2.970042572 1.636768859
2.902054291 4.330106144 4.98318803 3.15061082 6.069793571 0.140434478

2.856533238 4.356542495 3.610154461 3.200020656
TCGA-MP-A5C7-01A -1.268887725 4.6083203112.726699291 5.358463216
4.379107053 8.160446961 3.527443805 4.136269596 2.115164608
2.833342631 5.073407211-2.181600719 2.999202108 4.557047198
4.633598163 5.060920987 3.949981646 4.464818548 -0.069291958
0.2611050862.777573381 4.902376442 -1.515354401 2.099947219
4.607816652 3.031921052 5.439149259 3.379320261 4.362175857
3.282245887 4.822362374 0.877736557 -3.755028281 3.160365616 -
1.344447103 2.531242792 -3.402818928 5.586416234 3.2735119430.57778656
4.680700777 -1.371953741 5.139645306 3.616858872 3.948228104
4.198054072 6.931903994 1.665759895 4.493150104 6.530164984 -
5.03321612 -1.7743649 4.536498525 -2.332089823 3.257659955 -5.11625173
4.994099257 1.921044651 2.104070848 4.01491051 5.865988137
2.7484211332.291790044 -2.685110436 4.516179675 3.939855622
4.175758425 4.113846839-1.86492155 5.065264823 6.332026792 3.11047794 -
0.202704624 5.77610667 -1.135866849 6.1196411925.146905964 4.714176211
5.530159995 6.415828165 9.925122161 0.273943245 2.62284676
0.995461271 -1.319983659 2.93167009 0.534386301 4.364980255
6.857659964 3.805677598 4.874172075 -2.034095844 2.231197851-
0.279505353 3.267104522 3.617949676 -2.204646562 5.372515848
4.336295206 0.987859439 2.6375319113.951351821 4.995623643
4.763295153 3.538797674 5.335262534 4.931493462 4.705802952
4.053610822 0.38764481 3.992313751 4.595101823 4.5841611934.677223776
5.797912959 5.271862901 1.13947027 5.61572426 3.914237248 1.614093156
3.93437979 -0.850035039 -3.752338449 2.410819417 -0.026813314 -
3.510981283 -4.381079292 1.334300738 2.775446143 4.149090079
5.607779666 3.731014749 6.431298754 -0.83714795 2.5061125174.448955554
3.671517705 2.077470811
TCGA-NJ-A4YF-01A -0.362250527 5.087377559 3.047320205 5.489859546
4.269380868 8.159164407 3.68858744 4.098719876 2.233151397
3.549185522 5.344637164 -1.939912911 3.6247172114.477669914
5.0211025165.10158355 4.08726384 4.6833331190.290776609 0.368334113
2.994791066 5.198483095 -1.183229943 2.307439091 4.671802204

2.7554755115.526058446 4.08368292 4.693385526 4.842958695 4.94725023
1.445879697 -3.676852368 3.240888423 -0.975954287 3.011572707-
2.858920353 5.606389399 3.140575438 0.278169108 4.704088862 -
1.14806644 5.199322478 3.465865861 3.960436289 4.167350234 7.06650407
1.918586777 4.75439911 7.113666495-5.094616048 -1.615415806 4.82481374 -
2.322503982 3.694979556 -4.479102762 5.315494174 2.642502868
2.420582133 3.956962233 5.91129877 2.678562369 2.287487518 -
1.138216299 4.608876552 3.935093709 4.905385593 4.336423527 -
1.618665621 5.070656534 6.560561254 2.945412449 -0.01468784 5.520471071
-0.793312408 6.262843479 5.008430637 5.269486672 5.517159056
6.545265519 10.38317507 0.256577493 2.782524258 1.440063012 -
0.525592671 3.001218382 0.706386728 4.3961188476.969608171 3.606464195
4.959213501 -1.920625733 2.937379888 0.221668274 3.368283321
4.001287563 -2.047472166 5.362387213 4.174286341 0.968790622
2.774578435 3.843563302 5.120539958 4.749686796 3.64510473
5.335607219 4.9136580114.838094602 4.569297602 0.894896349
3.782593249 4.610791606 4.81296834 4.67671896 5.806430188 5.016382757
1.277523086 5.145175163 3.7211736911.61870938 4.093699024 -1.160734853
-3.158725479 2.930220028 -0.425512175 -3.834308904 -3.301375972
1.345673649 2.957613632 4.319485612 5.831915916 3.583591968
6.648806841 -1.062620634 2.6104671144.636119 3.767341788 2.587525977
TCGA-NJ-A4YG-01A -0.708750559 4.883674346 3.193716084 5.428510418
4.103530366 8.221740099 3.558989972 4.35499779 1.786437575 2.963286
5.307541616 -2.3154159 3.613731275 4.082656852 4.663682549
4.925770575 3.493054656 4.196560106 0.567571753 0.359551102
2.5116083565.122219612 -1.346382183 1.702905779 4.534357733
2.973448027 5.85631327 2.284729938 4.513175262 3.72960572 4.890547549
0.956648399 -3.747469434 3.45969846 -1.076936707 2.758852663 -
3.1798853115.6011244813.254398531 0.492981639 4.707755649 -1.166556562
4.863008962 2.72899419 4.074072526 4.290142772 6.770571523
1.444442675 3.681564947 6.675463356 -4.853078033 -1.860815105
4.63516349 -2.170468157 3.119944991-4.884866386 5.0110252342.081629892
1.976076042 3.97431138 5.960722977 2.512669818 1.886648574 -

2.237813204 4.5995119793.933239913 4.026701842 4.342582906 -1.951607826
5.086882718 6.405782719 2.965672665 -0.19980766 6.168019496 -
0.766529997 6.120209207 5.086901806 4.654706782 5.546067855
6.246063605 10.28055512 0.764070447 2.530775532 0.832969218 -
0.821862442 3.019517785 0.803220992 4.361962325 6.856445179
3.668615395 4.633756306 -2.109021935 2.371970758 -0.628232126
3.2622455 3.693264559 -2.613068327 5.361533472 4.443591305
0.772191024 2.870637828 3.904065906 5.01181318 4.804113111 3.165693381
5.335516791 4.943376825 4.755076093 4.186743332 0.514485985
2.316990106 4.493448051 4.733731588 4.720665972 5.731556651
5.017896505 1.009163864 5.614746315 4.062760421 1.804611604
3.920820563 -0.741143644 -4.336275876 2.474440645 -0.273175086 -
3.459610367 -3.707060456 1.625938292 2.916609027 4.4045831125.162884886
3.307934619 6.005786673 -0.742895298 3.0457118144.335941541
3.662573001 2.542656712
TCGA-NJ-A4YI-01A -0.444272192 4.947392351 2.981316845 5.340503574
4.254759735 8.212895958 3.457916083 4.264321293 1.900804044
3.042156623 5.277661515 -2.42745965 3.596503224 4.36323411 4.981065229
5.072958079 3.597562438 4.134522913 0.562996126 0.468138889
2.732528584 5.360310715 -0.992959783 1.845023108 4.564807354
3.107320101 5.75209204 2.703683928 4.662470724 3.891713826
5.062849103 1.064198592 -3.653604473 3.463070048 -0.982633627
3.293299987 -2.688216999 5.5868201183.475901383 1.1380121124.713588322
-1.092009419 5.019289283 2.772025868 4.087717202 4.218471867
6.783863559 1.748718042 3.782687594 6.77263379 -4.716230769 -
1.742771754 4.646020704 -2.357071837 3.128434091 -4.602773625
5.254141258 2.389334589 1.982343373 3.854390681 5.952933643
2.679058655 1.80104136 -2.022303801 4.645385356 3.931594649
4.203263369 4.332690451 -0.937030611 5.087078189 6.412890134
2.943638754 -0.073468177 5.825814803 -0.51598567 6.102439369
5.076045876 4.780769237 5.493264598 6.322387074 10.32532311
0.787567466 2.626016815 1.001908593 -0.526381597 3.173842727
0.878224176 4.382356723 6.913590414 3.529005432 4.697674764 -

1.817920544 2.593182224 -0.549930616 3.277405723 3.675726137 -
2.056124648 5.358187936 4.40478685 0.480316868 2.941045176 3.878301517
5.046542607 4.770582186 3.462278661 5.335587732 4.920044472
4.927486915 4.200959184 0.747213437 2.747502823 4.550525543
4.917288287 4.737158791 5.627878845 4.979175556 0.992039813
5.610102395 4.108950798 2.100310568 4.050211053-0.559336281 -
3.600228512 2.572512153 -0.170955159 -3.427569671 -3.855679729
1.645309361 3.042648315 4.434485304 5.340175952 3.467056149
6.1760275 -0.030870585 3.262834438 4.477813082 3.566282697 2.66621323
TCGA-NJ-A4YP-01A -0.783172423 4.993039446 3.060504585 5.447228198
3.856169573 8.235429359 3.456240956 4.364383077 1.835874416
2.963184557 5.61581387 -2.280830789 3.402870774 4.004973352
4.548729421 4.762817139 3.637098877 4.131923371 0.405936592
0.384341794 2.405645615 4.902833099 -1.513910452 2.076331519
4.5643733 2.778555646 5.842958941 2.49576676 4.444791007 3.409029497
4.854132559 0.851729758 -3.799522489 3.552021511-1.065703502
2.330209731 -2.926617836 5.589854357 2.7116289020.03553514 4.694089913
-1.228403257 4.886728067 2.8588354 4.041625622 4.31477425 6.670788351
1.410399091 3.54289444 6.844475961 -5.212220906 -1.989889886
4.710488401 -2.371139685 3.097286456 -5.241327721 5.036797994
2.1141004531.827712669 3.894167559 5.928695463 2.463575877
2.127694904 -2.551039781 4.685844329 3.928181424 3.978698098
4.312311529-2.485861828 5.086073976 6.374554154 2.897015347 -
0.18015835 5.989213296 -0.974234554 6.154552417 5.047499084 4.697811567
5.506882959 6.296757044 10.25572466 0.930755402 2.44175204
0.709868588 -0.57757045 3.136223962 0.929618723 4.328898901 6.87826219
3.529268928 4.756058581 -2.092274996 2.151126162-1.030443867
3.239940506 3.921403894 -2.659798287 5.349634074 4.284697272
0.73770769 2.902739665 3.863814892 5.021324605 4.709904048
2.857434142 5.335533762 4.837878955 4.790658147 3.807797136
0.51351271 2.355272584 4.478651421 4.7551102294.716129295 5.6312906
4.987026916 1.015789796 5.559943063 3.989127595 1.568738687
3.816144043 -0.574852159 -4.679134165 2.183763663 -0.573341169 -

3.727189101 -3.906777987 1.388695727 2.771674262 4.324361055
5.024935472 3.086130619 6.058522104 -0.495151051 3.09298318 4.18656066
3.612876998 2.292992281
TCGA-NJ-A4YQ-01A -0.232489064 5.048296458 3.1156200175.352249931
4.321041461 8.120621651 3.556557024 4.192262047 1.963130784
3.347595632 5.144411071-2.29681721 3.497162463 4.429263438 4.903971466
5.08649967 3.76941334 4.196517565 0.186327346 0.428363921 2.83225832
5.028993878 -1.137493956 1.9091119214.599639712 3.133168076
5.510564221 3.14819128 4.589736636 4.08015142 4.912468247 0.978357471
-3.687671201 2.749234774 -1.233166306 3.175351098 -2.834200118
5.594502969 2.885734797 0.604481683 4.703169736 -1.10885843 5.32282591
2.997461885 4.042143987 4.19900112 7.076135629 1.554588577
4.141980079 6.844182545 -4.94961593 -1.75928566 4.681087656 -2.651723248
3.492321208 -4.851010577 5.194783799 2.178732319 2.24458089
3.875173752 5.97464667 2.774722896 1.829920942 -2.039045404 4.61416316
3.931031849 4.315361926 4.292602049 -1.030755156 5.095739754
6.277069691 2.970259411-0.069360002 5.60384535 -0.616456461 6.202271736
5.077940058 4.783033676 5.459259363 6.34630056 10.61999335
0.582038894 2.519733105 0.725234997 -0.597840041 3.338494656
0.978069685 4.3011194336.857270579 3.7075304 4.72255877 -1.981656602
2.571553042 -0.268216618 3.271487155 3.891941072 -1.978756618
5.363433903 4.359222128 0.855001943 2.7114237464.1341381165.046126139
4.718919601 3.269768727 5.335554985 4.944809494 4.842821298
4.357472146 0.736371031 3.609337161 4.677904361 4.875011177
4.744881776 5.626602956 5.316494395 0.835105307 5.537286771
4.158472639 1.899038397 3.976457321 -0.941900576 -3.763298573
2.703887976 -0.322449644 -3.781144597 -3.629141326 1.552881103
2.9485711434.462779452 5.631700539 3.771339144 6.301468129 -
0.415355367 2.979067069 4.525162179 3.684453329 3.045702286
TCGA-NJ-A55A-01A -0.406257519 4.831294258 2.557427772 5.222280738
4.033881039 8.232984252 4.028477343 4.212679398 2.094676607
3.542976399 4.732683296 -2.233632698 3.17357276 4.520279841
4.913284686 4.48453901 3.655302956 4.1991156710.381733458 0.360175962

2.787030166 5.648251792 -1.298501453 1.8061411554.632727222
2.7411486485.821979707 1.971436007 4.540678953 3.349842555
4.807370528 0.987833671 -3.577356462 3.078453028 -1.25830208
2.636934966 -3.903525072 5.566061491 3.242867217 0.609115288
4.708895463 -1.050998477 5.142751951 2.990107019 3.95028745
4.304592131 6.9500905 1.6299576 3.973570975 6.963665918 -5.088569071
-1.577105369 4.3001828 -2.471995517 3.669378666 -4.688282306
5.109326437 2.207671765 2.579533745 4.213574074 5.923420464
2.672586169 1.306130844 -1.960851472 4.736149363 3.930994856
3.992097021 4.311363563-1.597111733 5.092601473 6.437189688 2.97097445
0.1140252815.988834191 -0.1594114 5.981880796 5.149516595 4.598633791
5.385309302 6.45455444 9.951352852 0.221660741 2.68708391 1.168433019
-0.797938872 3.357031279 0.724196704 4.188602496 6.907184774
3.449665999 4.616519194 -1.892883702 2.027285361 -0.21081614
3.244033942 3.885198102 -2.226641106 5.346016216 5.102204069
1.349863395 3.104635736 3.99842589 4.931048613 4.727821374
3.320814914 5.33548063 4.948048874 4.925328147 4.1116874680.711958397
3.129078579 4.46525944 4.802610943 4.790760768 5.493544892
4.982954794 1.031731472 5.295012525 4.270128817 2.059698813
3.900574012 -0.728537398 -3.840956826 3.347894603 -0.541762443 -
3.681390692 -3.698112301 1.252006486 2.707569659 4.0311569025.387654817
3.391076398 6.238103618 -0.467859087 2.734004228 4.479455794
3.786890287 2.637928286
TCGA-NJ-A55O-01A -0.36050289 4.962740785 3.288059019 5.375490157
4.076857285 8.339315867 3.258894723 4.335456177 1.978214253
2.999987263 5.496550793 -2.352631563 3.7029112954.296503826
4.819555693 5.184462194 3.646403891 3.981408778 0.598448271
0.600237999 2.8118661115.017319274 -1.14367772 2.349979455 4.523422201
3.294868907 5.7667641153.649092122 4.489514361 2.695827628
4.901263025 0.804517219 -3.670606252 3.578561945 -1.419360129
2.750715233 -2.619231799 5.587393351 3.161995432 0.842508357
4.721360075 -1.093907115 4.975168814 2.836277454 4.1634896 4.31807959
7.073120107 1.59027221 3.902748602 6.762979004 -4.552505064 -

1.769224321 4.682725569 -2.422742997 3.223218117-4.66144893 5.224519529
2.210739249 1.944033687 3.946635304 6.138932135 2.757601192
1.907507892 -1.575782694 4.638760512 3.928834616 4.326534316
4.183798976 -1.572275393 5.083878318 6.46042724 3.050650531 -
0.312726127 6.051274964 -0.44966819 6.132844941 5.189684331 4.751289939
5.523151213 6.256707639 10.25661332 1.580526846 2.554417177
0.630075991 -0.633654729 3.391495927 0.893468104 4.377005569
6.9202115523.729804303 4.688930863 -1.909346249 2.007774528 -
0.184193787 3.240038905 3.841424579 -2.403450918 5.354015307
4.464832013 0.838662009 3.04151525 4.376775893 5.009208827
4.668525431 3.00906843 5.335468373 5.292172498 4.815255185
4.228373568 0.673722362 3.07147629 4.70009041 4.797042389 4.736596365
5.7831157565.277277924 1.051508581 5.69194735 4.455627492 1.916249679
3.988775378 -0.58959737 -3.433372195 2.651205115-0.280804542 -3.349125207
-3.54266288 1.823050672 3.046755824 4.663776138 5.330176593 3.13914879
6.138691305 -0.295554102 3.364159379 4.634380156 3.798838548
3.028327259

TCGA-NJ-A55R-01A -0.511540703 5.006880609 2.520455963 5.019589445
4.192177234 8.2953112664.406406004 4.161597006 2.788027052
3.809432261 5.144122185 -1.826328187 3.404327624 4.790833163
5.222416597 4.942460347 4.077415691 5.365702138 0.483667055
0.347856891 3.295821378 5.779281432 -1.301154964 3.06279263
4.687997731 2.693143957 5.612775922 5.416394436 4.7361448
4.013716012 4.844840849 1.364540975 -3.473494354 3.647625912 -
1.104712293 2.531501129-2.826532223 5.582608967 3.295218904 0.6735474
4.714408098 -0.979804204 4.9820341113.538251956 4.052201083
4.333671613 7.182584529 2.069467407 4.944260748 6.863150236 -
4.51585505 -0.96232465 4.357101741 -1.862264105 3.968708273 -3.931071727
5.336305374 3.139545048 2.108617834 4.227055601 5.993158984
3.212627734 2.343143459 -0.566047029 4.599957699 3.936671052
5.093834153 4.288621737 -2.260817007 5.068944766 6.811380336
3.192189417 -0.149364081 5.76607425 -0.399684589 6.043570846
5.185814633 5.10908238 5.454152862 6.591344158 9.93444073 0.868056134

2.948662719 1.68758554 -0.543283116 3.641831319 0.452138261
4.216527582 6.968670142 3.829391515 4.696583391 -1.940477488
2.579928146 0.637345576 3.41538473 4.25716417 -2.04604885 5.34577796
4.724130127 1.86837302 3.300803956 3.938762032 5.095717851
4.653622856 3.989229569 5.335649454 5.191580845 4.939656525
4.795977968 0.786827086 4.742460835 4.475570456 4.760003714
4.7074665 5.897680348 5.082898528 1.470281798 5.358880422
3.785077128 1.809039933 4.183824539 -0.914803153 -2.47440476
3.776478542 -0.080956203 -3.462749614 -3.019401633 1.397095189
2.998863727 3.941251845 6.038536703 3.868272946 6.737686521 -
0.52996091 2.436049344 4.870253504 3.99315192 2.362130112
TCGA-NJ-A7XG-01A -0.857241971 4.867609662 3.222951475 5.308678648
4.374254949 8.186930288 3.349774375 4.263614286 2.078792075
2.8703631144.927900045 -2.368766232 3.307907202 4.325380783
4.490088145 5.07507471 3.220948173 4.141843654 0.205126647
0.362746837 2.743238919 5.088063041 -1.261621205 1.007490911
4.562586254 3.153363093 5.577777747 3.47823981 4.542192792
3.595606353 5.075869805 0.976449697 -3.656783827 2.966398727 -
1.215506057 3.102603865 -3.276830835 5.616247914 3.634789279
0.561000766 4.723800593 -1.099469869 4.959526722 2.707410979
4.059998847 4.165486213 7.157236621 1.441959904 4.300373024
6.643249316 -4.589216914 -1.721641155 5.012102997 -2.22898348 3.17419561
-4.747684124 5.1162755972.346854014 2.485923488 3.8220961145.845768573
2.50120196 1.43918019 -2.068211651 4.528862461 3.938710183 4.241008102
4.185362784 -1.416758987 5.08034872 6.3811644173.062917399 -0.164818316
5.770379698 -0.781253884 6.170132571 5.1345352114.926322845
5.476075014 6.329580167 10.28420797 0.625075041 2.578569592
0.962543294 -0.8972382 2.379722413 0.72328286 4.365630842 6.881317227
3.734246129 4.524400978 -2.142236582 2.278169537 -0.090301016
3.257165346 3.591226819 -2.678254182 5.370399292 4.361231813
0.262865871 2.319857446 3.89992045 5.055030232 4.940587441
3.613153344 5.33557984 4.726165715 4.741442218 4.125012978 0.52106854
2.792051988 4.518716492 4.798766979 4.710228953 5.67715857

4.909056286 0.927248896 5.440371096 4.003724256 1.697587163
4.101028982 -1.098830598 -3.709011843 2.624603711-0.071015813 -
3.398783437 -3.564148265 1.654053139 2.988682449 4.321529682
5.564769835 3.678246798 6.249691054 -0.662000901 3.057010887
4.606004138 3.707467318 2.163200958
TCGA-O1-A52J-01A 0.910626941 4.83972217 3.278479503 5.309950418
4.252991024 8.162026966 3.436823318 4.226296633 1.959858957
3.438172467 4.663995802 -2.433613279 3.572692331 4.608725027
5.042821019 5.075733843 3.450887961 4.050679764 0.523438864
0.465797133 3.025707489 5.166413413 -0.669129562 2.532011801
4.5621133673.436734412 5.533102638 4.459866237 4.679127373
3.614635274 5.109359887 1.143500519 -3.248329596 3.333680454 -
1.1814011163.437833035 -2.922075871 5.586057934 3.361377895 2.000734942
4.727909764 -1.050125234 5.384752106 3.101239039 4.023016925
4.205657969 7.10810143 1.942337163 4.537106393 6.675440885 -
4.401305549 -1.631404198 4.366541363 -2.430864154 3.300882643 -
4.33507235 5.1139235982.601398171 2.788128085 4.015382894 6.100405588
2.7541160881.850526518 -1.087307087 4.737373124 3.940648147
4.421666631 4.195137523 -0.002332293 5.085336612 6.394920106
2.949888599 -0.064256199 5.718184871 0.170588773 5.944874393
5.144692623 4.889987822 5.477526612 6.338525894 10.49838268
0.9110346582.856213418 0.99112342 -0.494681083 3.454732787 0.921570478
4.325401249 6.9193118143.662382406 4.617837361 -2.036337511
2.392724813 0.209892376 3.313807053 3.716445548 -1.342121807
5.362910079 4.706590132 1.193510158 2.78136454 4.317357996
5.056438344 4.712530031 3.492409095 5.335713649 5.205925815
5.062391682 4.155406714 1.046107988 3.730497306 4.649993222
5.001241789 4.769460753 5.657088945 5.370297232 0.897607701
5.464339675 4.236378266 2.476361954 4.069474354 -0.721285768 -
2.917849641 3.089772754 -0.09131771 -3.4046342 -3.257104613 1.810816519
3.394564961 4.594443634 5.631908179 3.172317876 6.514741609
0.61300398 3.24797887 4.762244563 3.74663902 3.580171731
TCGA-S2-AA1A-01A 0.103160479 5.032776679 2.708539132 5.236922964

3.846108985	8.269574427	3.623812783	4.284078559	2.019112907
3.396580868	5.226703052	-2.486709812	3.563633694	4.064399583
4.974240595	5.015840057	3.56674074 3.995852413	0.509466933
0.493523946	2.780292469	5.232452866	-0.909841954	2.310348807
4.558156908	2.949989824	5.822897017	2.643646692	4.648279777
3.300546537	4.843314413	0.915640862	-3.574160633	3.522511214-1.293744484	2.494478648	-2.726408212	5.570992166	3.245902734
0.551866662	4.728030916	-1.029027676	5.058391015	2.742510723
4.151706029	4.368828823	7.087747033	1.783547434	3.83892786
6.819674174	-4.648679492	-1.697607219	4.305353604	-2.479535945
3.215113859-4.735544674	5.198621703	2.245051072	1.840816423
4.055935028	6.176745481	2.765273978	1.871047392	-1.786277707
4.806083477	3.932601246	4.220914313	4.323689497	-1.004514075
5.106588861	6.476396041	3.05056928 -0.275400484	6.112361442-0.11407453
6.048303367	5.246632923	4.6643811825.499010309	6.189158143
10.35529702	1.12855514 2.52070688 0.658578057	-0.55637895 3.47101044
0.964061299	4.320472523	6.913162363	3.710548971	4.559628822	-1.967058517	2.173958843	-0.719382774	3.261339646	3.872241493	-2.027077944	5.347071095	4.865422534	1.406090097	3.006438683
4.303812109	5.029840652	4.682683841	2.5925421125.335748373
5.273823355	4.832432421	4.058765916	0.885602195	2.819528864
4.509561644	4.843303498	4.773195622	5.562109509	5.129942327
1.088165297	5.590963914	4.138107367	2.201414399	3.942682349	-0.577959092	-3.640635104	2.940431765	-0.403787279	-3.43939366 -3.338784569
1.52900406 3.254841874	4.40759013 5.2463139	3.221067049	6.178599221
0.22136877 3.256364422	4.482379486	3.777929174	2.918017382

Table.S10. Validation of IMvigor210 cohort for immunotherapy

sample	riskscore	riskgroup	time	status	response	binaryResponse	Response
SAM0257bbbbd388	-0.101824315	Low	469.1581109	1	SD	SD/PD	CR/PR/SD
SAM025b45c27e05	-0.085017843	Low	263.1622177	1	PD	SD/PD	PD
SAM032c642382a7	-0.012936398	High	74.90759754	1	PD	SD/PD	PD

SAM0571f17f4045	-0.099589139	Low	136.0164271	1	SD	SD/PD	CR/PR/SD
SAM065890737112	-0.160506022	Low	602.2176591	0	CR	CR/PR	CR/PR/SD
SAM0684af734db1	-0.158374478	Low	697.8234086	0	PR	CR/PR	CR/PR/SD
SAM075e037d95bc	-0.104812141	Low	639.6714579	0	PR	CR/PR	CR/PR/SD
SAM07a93a28f801	-0.015328939	High	385.3798768	0	PR	CR/PR	CR/PR/SD
SAM08cce2fa88f2	-0.063328611	High	663.3264887	0	SD	SD/PD	CR/PR/SD
SAM09c84ec0cf34	-0.095290513	Low	195.1540041	1	PD	SD/PD	PD
SAM0a0f2bac4b20	-0.038931198	High	35.4825462	1	PD	SD/PD	PD
SAM0a7c2091dd56	0.024334231	High	78.85010267	1	PD	SD/PD	PD
SAM0bdb3428bd13	-0.132191822	Low	67.02258727	1	PD	SD/PD	PD
SAM0ce9c983b20f	-0.041648756	High	198.110883	1	PD	SD/PD	PD
SAM0d855cff64e6	-0.01075455	High	85.74948665	1	PD	SD/PD	PD
SAM0f956e757453	-0.116478278	Low	312.4435318	1	PD	SD/PD	PD
SAM110501d0eedb	-0.142021795	Low	128.1314168	1	PD	SD/PD	PD
SAM12502d970c10	-0.12251312	Low	221.7659138	1	SD	SD/PD	CR/PR/SD
SAM14938611a2d3	-0.151980802	Low	642.6283368	0	PR	CR/PR	CR/PR/SD
SAM14df63a65411	-0.089397335	Low	642.6283368	0	PR	CR/PR	CR/PR/SD
SAM166a419a4e5a	-0.058882124	High	161.6427105	1	PD	SD/PD	PD
SAM18039827e1b9	-0.155925761	Low	44.35318275	1	PD	SD/PD	PD
SAM181b638b8248	-0.02946372	High	326.2422998	1	PD	SD/PD	PD
SAM187e056d6a2a	-0.075112562	High	68.99383984	1	PD	SD/PD	PD
SAM18a4dabbc557	-0.015384902	High	493.798768	1	PD	SD/PD	PD
SAM18b9351e265a	-0.0773212	High	476.0574949	1	PD	SD/PD	PD
SAM18bc1078bc15	-0.062419721	High	206.9815195	1	PD	SD/PD	PD
SAM18be5b395318	-0.086055306	Low	270.0616016	1	PD	SD/PD	PD
SAM19fec8f3b3bd	-0.036129727	High	296.6735113	1	PD	SD/PD	PD
SAM1a87df750b9d	-0.018199134	High	161.6427105	1	SD	SD/PD	CR/PR/SD
SAM1ab1b28d9f2b	-0.064876782	High	63.08008214	1	PD	SD/PD	PD
SAM1abf01dd4544	-0.060677296	High	505.6262834	0	PR	CR/PR	CR/PR/SD
SAM1ac4e3dee297	-0.064050786	High	165.5852156	1	PD	SD/PD	PD
SAM1c0ecfb3eb63	-0.04276745	High	175.4414784	1	PD	SD/PD	PD
SAM1c8b086175ca	-0.076319365	High	622.9158111	1	PD	SD/PD	PD
SAM1dda30f1c5be	-0.070290381	High	637.7002053	0	PD	SD/PD	PD

SAM1e9c4d1d39ae	-0.053344359	High	372.5667351	1	PD	SD/PD	PD
SAM1f66db567eb5	0.023982545	High	497.7412731	0	PR	CR/PR	CR/PR/SD
SAM1f83ebd6be9b	-0.058775291	High	105.4620123	1	PD	SD/PD	PD
SAM1fa6bcb7fc48	-0.166031842	Low	682.0533881	0	PD	SD/PD	PD
SAM203dcf14f927	-0.139056282	Low	580.5338809	0	CR	CR/PR	CR/PR/SD
SAM2070b416069c	-0.148810659	Low	240.4928131	1	SD	SD/PD	CR/PR/SD
SAM23095936e611	-0.132531521	Low	67.02258727	1	PD	SD/PD	PD
SAM23aa15d4a0b0	-0.092558325	Low	171.4989733	1	PD	SD/PD	PD
SAM25510f300d79	-0.022207169	High	620.9445585	0	CR	CR/PR	CR/PR/SD
SAM2570ff4aae6e	-0.152344198	Low	671.211499	0	PR	CR/PR	CR/PR/SD
SAM26104d5adc89	-0.117762594	Low	161.6427105	1	SD	SD/PD	CR/PR/SD
SAM2624229effe8	-0.093074619	Low	76.8788501	1	PD	SD/PD	PD
SAM27299aed7681	-0.097052043	Low	565.7494867	0	PR	CR/PR	CR/PR/SD
SAM28687037e4ff	-0.043977129	High	647.5564682	0	SD	SD/PD	CR/PR/SD
SAM28e6031ac18b	0.020421203	High	276.9609856	1	SD	SD/PD	CR/PR/SD
SAM297c0301e861	-0.026869854	High	242.4640657	1	PD	SD/PD	PD
SAM29da928587ad	-0.043944915	High	77.86447639	1	PD	SD/PD	PD
SAM2b672f4336c7	-0.180738111	Low	459.301848	1	SD	SD/PD	CR/PR/SD
SAM2bba8cb35e48	-0.035823253	High	314.4147844	1	PD	SD/PD	PD
SAM2c9586161ce6	-0.164283709	Low	338.0698152	1	PD	SD/PD	PD
SAM2dc3f04e45e9	-0.212436423	Low	305.5441478	1	PD	SD/PD	PD
SAM2e7aa8fa0ab3	-0.12213667	Low	231.6221766	1	PD	SD/PD	PD
SAM2e9ac0b1b250	0.041692302	High	664.312115	0	CR	CR/PR	CR/PR/SD
SAM2eb07dedf07f	-0.093754854	Low	615.0308008	0	PR	CR/PR	CR/PR/SD
SAM2f228939632f	-0.026558345	High	236.550308	1	PD	SD/PD	PD
SAM30b5c6c54cf7	-0.007756291	High	277.94661191		CR	CR/PR	CR/PR/SD
SAM30cf07d4874f	-0.060017056	High	349.8973306	1	SD	SD/PD	CR/PR/SD
SAM31d9176e11fb	-0.049762844	High	513.51129360		CR	CR/PR	CR/PR/SD
SAM31f41dd0d6ca	-0.176642793	Low	248.3778234	1	SD	SD/PD	CR/PR/SD
SAM3330c03fdf00	-0.109531676	Low	575.6057495	0	PD	SD/PD	PD
SAM34430ef08e5b	-0.094114928	Low	545.0513347	0	PD	SD/PD	PD
SAM36851bc8b9ae	-0.19259702	Low	106.4476386	1	SD	SD/PD	CR/PR/SD
SAM36a9225b0222	-0.072780817	High	714.5790554	0	SD	SD/PD	CR/PR/SD

SAM36d87392593b	-0.03222791	High	710.6365503	0	SD	SD/PD	CR/PR/SD
SAM3894ac3956a5	-0.140429101	Low	63.08008214	0	PD	SD/PD	PD
SAM39eb94fa504d	-0.044862187	High	537.1663244	1	PD	SD/PD	PD
SAM3a1c9632ff7b	-0.082209676	High	480.9856263	0	SD	SD/PD	CR/PR/SD
SAM3b1066e5801b	-0.143035221	Low	469.15811090	SD	SD/PD	CR/PR/SD	
SAM3b15b4c6311d	-0.010403779	High	643.613963	0	CR	CR/PR	CR/PR/SD
SAM3cb94b0d5297	-0.071028078	High	151.7864476	1	PD	SD/PD	PD
SAM3e04eb914f3d	-0.120530091	Low	518.4394251	0	PR	CR/PR	CR/PR/SD
SAM3e8baff50d7a	-0.015404385	High	208.9527721	1	SD	SD/PD	CR/PR/SD
SAM3ee5dcd894f0	-0.078184118	High	261.1909651	1	PD	SD/PD	PD
SAM415f36ad349e	-0.044632433	High	539.137577	1	PD	SD/PD	PD
SAM4305ab968b90	-0.133839508	Low	42.38193018	1	PD	SD/PD	PD
SAM4501e41e4751	-0.023594959	High	33.511293631	PD	SD/PD	PD	
SAM45c8e6412c66	-0.012117985	High	104.476386	1	PD	SD/PD	PD
SAM468a9e1dc821	-0.033008812	High	116.30390141	PD	SD/PD	PD	
SAM47fc46c3d6be	-0.057011291	High	561.8069815	0	SD	SD/PD	CR/PR/SD
SAM49d48750e294	-0.092830311	Low	295.687885	1	PD	SD/PD	PD
SAM49f9b2e57aa5	-0.1217644	Low	223.7371663	1	PD	SD/PD	PD
SAM4b0175e8db6e	-0.117972056	Low	62.09445585	1	PD	SD/PD	PD
SAM4b7ea015fd9e	-0.121903446	Low	398.1930185	1	PD	SD/PD	PD
SAM4caabd64e7fd	0.008609278	High	471.1293634	0	PR	CR/PR	CR/PR/SD
SAM5234688806a7	-0.136721438	Low	176.4271047	1	PD	SD/PD	PD
SAM52500cabdd36	-0.153899698	Low	614.0451745	0	SD	SD/PD	CR/PR/SD
SAM52e3fa3ad574	-0.162116587	Low	238.5215606	1	PD	SD/PD	PD
SAM548551ef782c	-0.019679471	High	518.4394251	0	PR	CR/PR	CR/PR/SD
SAM54e58f1b0230	-0.079681716	High	131.0882957	1	PD	SD/PD	PD
SAM553c3c35b847	-0.145713656	Low	201.0677618	1	PD	SD/PD	PD
SAM557dde1b9f3e	-0.156520153	Low	201.0677618	1	PD	SD/PD	PD
SAM560f23d6a3ad	0.045766232	High	641.6427105	0	CR	CR/PR	CR/PR/SD
SAM563d6233dfa2	0.000265806	High	64.06570842	1	PD	SD/PD	PD
SAM568ce160abd9	-0.162120053	Low	385.3798768	1	SD	SD/PD	CR/PR/SD
SAM572f19794c96	-0.032520372	High	504.6406571	0	CR	CR/PR	CR/PR/SD
SAM5767dd75d142	-0.124138098	Low	545.0513347	0	PR	CR/PR	CR/PR/SD

SAM58e7832f4e7d	-0.123765038	Low	493.798768	0	PR	CR/PR	CR/PR/SD
SAM59f392864f5d	-0.138156735	Low	533.2238193	1	SD	SD/PD	CR/PR/SD
SAM59fda9035d1d	-0.014754463	High	112.36139631		PD	SD/PD	PD
SAM5a2347c0498a	-0.201447186	Low	146.8583162	1	SD	SD/PD	CR/PR/SD
SAM5b57e47fdcb3	-0.08117608	High	115.31827521		PD	SD/PD	PD
SAM5c139c5c1c4f	-0.068093285	High	216.8377823	1	PD	SD/PD	PD
SAM5cc2d9036053	-0.119198468	Low	170.513347	1	PD	SD/PD	PD
SAM5cfa1699bdb7	-0.069590751	High	475.0718686	0	PD	SD/PD	PD
SAM5d1dfd5207f5	-0.102205635	Low	327.2279261	1	PD	SD/PD	PD
SAM5d989c86255e	-0.05437258	High	103.4907598	1	PD	SD/PD	PD
SAM5e3bae090b8c	-0.121385882	Low	202.0533881	1	PD	SD/PD	PD
SAM5fe7a81a39dd	-0.165088093	Low	512.5256674	1	PD	SD/PD	PD
SAM6083aac8db99	-0.057913827	High	618.973306	0	PR	CR/PR	CR/PR/SD
SAM6157c8f38b72	-0.098619415	Low	497.7412731	0	PR	CR/PR	CR/PR/SD
SAM61b9d4d84c64	-0.153773339	Low	641.6427105	0	SD	SD/PD	CR/PR/SD
SAM61baf919bb01	-0.119557557	Low	468.1724846	1	SD	SD/PD	CR/PR/SD
SAM62fb1388c871	-0.06720936	High	675.1540041	0	PD	SD/PD	PD
SAM63405b04ab2d	-0.044094548	High	204.0246407	1	PD	SD/PD	PD
SAM63b2189c36d7	-0.060071152	High	119.26078031		PD	SD/PD	PD
SAM65afda25b920	-0.072884643	High	242.4640657	1	SD	SD/PD	CR/PR/SD
SAM6662f5181f87	-0.149324741	Low	578.5626283	0	PD	SD/PD	PD
SAM670649e105b5	-0.089261585	Low	662.3408624	0	CR	CR/PR	CR/PR/SD
SAM675a12a09c15	-0.012754445	High	86.735112941		PD	SD/PD	PD
SAM6780ed436b55	-0.018719454	High	187.2689938	1	PD	SD/PD	PD
SAM6792d6e98068	-0.194817229	Low	180.3696099	1	PD	SD/PD	PD
SAM681e4bf7cf85	-0.039631606	High	701.7659138	0	PR	CR/PR	CR/PR/SD
SAM6964a6d7b967	-0.078741602	High	487.8850103	1	PD	SD/PD	PD
SAM6cb230f208a8	-0.122022112	Low	696.8377823	0	PR	CR/PR	CR/PR/SD
SAM6cbc10abddb0	-0.031052917	High	178.3983573	1	PD	SD/PD	PD
SAM6d2ae0c39b96	-0.176817337	Low	663.3264887	0	PD	SD/PD	PD
SAM6dd7ad1d797d	0.017989797	High	578.5626283	1	PD	SD/PD	PD
SAM6f2a102a99df	-0.048660969	High	578.5626283	0	CR	CR/PR	CR/PR/SD
SAM6ff654a20f98	-0.168021707	Low	556.8788501	0	CR	CR/PR	CR/PR/SD

SAM7114d99032ec	-0.10376519	Low	325.2566735	1	PD	SD/PD	PD
SAM716f54e468f4	-0.089204386	Low	635.7289528	0	SD	SD/PD	CR/PR/SD
SAM727c0e92a2a7	-0.126596392	Low	561.8069815	0	PR	CR/PR	CR/PR/SD
SAM73663ee4a96e	-0.137014229	Low	637.7002053	0	CR	CR/PR	CR/PR/SD
SAM75142fcab9df	-0.07292474	High	357.7823409	1	PD	SD/PD	PD
SAM7538ad9ff524	-0.117329289	Low	573.6344969	0	SD	SD/PD	CR/PR/SD
SAM753d4bb52dbe	-0.122879456	Low	341.026694	1	SD	SD/PD	CR/PR/SD
SAM75f12d1a55fc	-0.141656275	Low	442.5462012	0	SD	SD/PD	CR/PR/SD
SAM76a431ba6ce1	-0.133959199	Low	423.8193018	1	PD	SD/PD	PD
SAM771445e92421	-0.117999488	Low	582.5051335	0	CR	CR/PR	CR/PR/SD
SAM7829a341b9f3	-0.167880913	Low	116.30390141		PD	SD/PD	PD
SAM7893196e0e89	-0.008368084	High	79.83572895	1	PD	SD/PD	PD
SAM7aa01fc49a80	-0.129179266	Low	64.06570842	1	PD	SD/PD	PD
SAM7b40007f4aa4	-0.01111442	High	649.5277207	0	PR	CR/PR	CR/PR/SD
SAM7bff231634e9	-0.046795575	High	307.5154004	1	SD	SD/PD	CR/PR/SD
SAM7c67b05aa109	-0.135662957	Low	247.3921971	1	SD	SD/PD	CR/PR/SD
SAM7d2dfba6cd84	-0.026813285	High	381.4373717	1	SD	SD/PD	CR/PR/SD
SAM7d7c54623618	-0.011211215	High	636.7145791	0	PR	CR/PR	CR/PR/SD
SAM7f0d9cc7f001	-0.063882244	High	138.973306	1	PD	SD/PD	PD
SAM7fb6987514a4	-0.073714261	High	685.0102669	0	PD	SD/PD	PD
SAM7fb7a13c096b	-0.095561077	Low	50.26694045	1	PD	SD/PD	PD
SAM80c6183220e6	-0.016525461	High	442.5462012	0	PD	SD/PD	PD
SAM81b71522417a	-0.001175766	High	64.06570842	1	PD	SD/PD	PD
SAM822b226466a1	-0.129326041	Low	518.4394251	0	SD	SD/PD	CR/PR/SD
SAM8533e5e261d6	-0.13111	Low	384.3942505	1	PR	CR/PR	CR/PR/SD
SAM85f0a3ac1c45	-0.102002775	Low	175.4414784	1	PD	SD/PD	PD
SAM87a8e18eb45b	-0.094667958	Low	82.7926078	1	PD	SD/PD	PD
SAM8884fe446d20	0.000198624	High	694.8665298	0	PR	CR/PR	CR/PR/SD
SAM8a1b0e02ee42	0.093737763	High	617.0020534	0	CR	CR/PR	CR/PR/SD
SAM8b4b8b0f9e73	-0.182308453	Low	470.1437372	0	SD	SD/PD	CR/PR/SD
SAM8e469834acc1	-0.18928753	Low	111.37577	1	PD	SD/PD	PD
SAM8e8ef2368dfa	-0.090252014	Low	135.0308008	1	PD	SD/PD	PD
SAM8f2275c36e8c	-0.188173033	Low	621.9301848	0	SD	SD/PD	CR/PR/SD

SAM91c47b054ffb	-0.097654866	Low	510.5544148	1	PR	CR/PR	CR/PR/SD
SAM9306c5c92444	-0.048849845	High	463.2443532	0	PD	SD/PD	PD
SAM9410b866974a	-0.004091698	High	202.0533881	1	SD	SD/PD	CR/PR/SD
SAM943df5cf15df	-0.0271461	High	64.06570842	1	PD	SD/PD	PD
SAM94859b440b1d	-0.093136538	Low	400.164271	1	PD	SD/PD	PD
SAM9539a4f19ebc	-0.123827233	Low	238.5215606	1	SD	SD/PD	CR/PR/SD
SAM957378bd907f	-0.114787023	Low	67.02258727	1	PD	SD/PD	PD
SAM961d04c42bd9	-0.040846521	High	176.4271047	1	PD	SD/PD	PD
SAM9725303dce0c	-0.103999618	Low	267.1047228	0	PD	SD/PD	PD
SAM978a587b207e	-0.154669404	Low	63.08008214	1	PD	SD/PD	PD
SAM97a00e0929fb	-0.033178897	High	381.4373717	0	PR	CR/PR	CR/PR/SD
SAM99a46b9eec27	-0.013144122	High	108.4188912	1	PD	SD/PD	PD
SAM9a2cf3c06fb3	-0.108912021	Low	169.5277207	1	PD	SD/PD	PD
SAM9aa6a095a9d6	-0.07934336	High	265.1334702	1	PD	SD/PD	PD
SAM9cafb905b36a	0.015318135	High	228.6652977	1	PD	SD/PD	PD
SAM9d2494119c05	0.242538769	High	43.36755647	1	PD	SD/PD	PD
SAM9daccafc18db	-0.024018845	High	493.798768	0	PD	SD/PD	PD
SAM9e11ec6bea80	-0.036995927	High	462.2587269	1	SD	SD/PD	CR/PR/SD
SAM9fb814c22bdb	-0.058521147	High	573.6344969	1	SD	SD/PD	CR/PR/SD
SAMa1871f491b02	-0.130492063	Low	52.23819302	1	PD	SD/PD	PD
SAMa1e62d323e1d	-0.091509564	Low	317.3716632	1	SD	SD/PD	CR/PR/SD
SAMa321770ac31c	-0.12093733	Low	211.90965090		PD	SD/PD	PD
SAMa424c75831b4	-0.068887314	High	106.4476386	1	PD	SD/PD	PD
SAMa535fcdf18a0	-0.072641744	High	518.4394251	0	PR	CR/PR	CR/PR/SD
SAMa90d73f8d891	-0.003098352	High	30.55441478	1	PD	SD/PD	PD
SAMa913c6139ec8	-0.163444991	Low	153.7577002	1	SD	SD/PD	CR/PR/SD
SAMa9ca8536d2b1	-0.098517378	Low	655.4414784	0	SD	SD/PD	CR/PR/SD
SAMaabf4afe4213	-0.041790575	High	78.85010267	1	PD	SD/PD	PD
SAMaaf505c36f93	-0.18037502	Low	617.0020534	0	PR	CR/PR	CR/PR/SD
SAMab8052a03398	-0.059694803	High	399.1786448	1	SD	SD/PD	CR/PR/SD
SAMabc151b01ea3	0.337191244	High	16.75564682	1	PD	SD/PD	PD
SAMad83c9c53537	-0.129039651	Low	449.4455852	0	PD	SD/PD	PD
SAMae02629a97f7	-0.031956708	High	97.57700205	1	PD	SD/PD	PD

SAMae1690469964	-0.190164511	Low	634.7433265	1	SD	SD/PD	CR/PR/SD
SAMae4da274eded	-0.188465819	Low	149.8151951	1	PD	SD/PD	PD
SAMaec7380f9ab0	-0.103961761	Low	625.8726899	0	PR	CR/PR	CR/PR/SD
SAMaf42c1541269	-0.033846992	High	165.5852156	0	SD	SD/PD	CR/PR/SD
SAMb0a83e5fbde9	-0.144015292	Low	699.7946612	0	SD	SD/PD	CR/PR/SD
SAMb0d11db9aa79	-0.066115508	High	316.386037	1	PD	SD/PD	PD
SAMb15ac6e4c4ef	-0.162539611	Low	600.2464066	0	PR	CR/PR	CR/PR/SD
SAMb15ad09d6e24	-0.080727172	High	116.30390141		PD	SD/PD	PD
SAMb2e4a082541a	-0.070944248	High	131.0882957	1	PD	SD/PD	PD
SAMb2f1d0e54ece	-0.076205036	High	342.0123203	1	PR	CR/PR	CR/PR/SD
SAMb3c02294aba7	-0.097224829	Low	672.1971253	0	PR	CR/PR	CR/PR/SD
SAMb419a8fcbfcd	-0.019964504	High	682.0533881	0	PR	CR/PR	CR/PR/SD
SAMb470eb8f04be	0.033463857	High	602.2176591	0	CR	CR/PR	CR/PR/SD
SAMb4c7a001537d	-0.093524325	Low	95.60574949	1	PD	SD/PD	PD
SAMb8070b7937e7	-0.170806512	Low	630.8008214	1	SD	SD/PD	CR/PR/SD
SAMb8101c538753	-0.030702457	High	402.1355236	1	SD	SD/PD	CR/PR/SD
SAMb963dda93cfd	-0.04373326	High	82.7926078	1	PD	SD/PD	PD
SAMba1a34b5a060	-0.049331626	High	385.3798768	1	PD	SD/PD	PD
SAMba7176afe070	-0.110703193	Low	103.4907598	0	PD	SD/PD	PD
SAMbc8dc3a7b54e	-0.079207065	High	442.5462012	1	PD	SD/PD	PD
SAMbcb07ba81cee	-0.041580228	High	75.89322382	1	PD	SD/PD	PD
SAMbcbc7957c264	-0.11344096	Low	125.174538	1	PD	SD/PD	PD
SAMbda79f955628	-0.094309338	Low	618.973306	0	SD	SD/PD	CR/PR/SD
SAMbe83eae4026e	-0.109983784	Low	81.80698152	1	PD	SD/PD	PD
SAMbf1a3ae828e6	-0.117989276	Low	621.9301848	0	CR	CR/PR	CR/PR/SD
SAMbfdffb97c446	-0.037148234	High	201.0677618	1	SD	SD/PD	CR/PR/SD
SAMc0d625a50eb8	-0.138411223	Low	398.1930185	1	SD	SD/PD	CR/PR/SD
SAMc0da5d48686d	-0.007794731	High	284.8459959	1	PD	SD/PD	PD
SAMc0ef41aa6c8b	-0.039025197	High	286.8172485	1	PD	SD/PD	PD
SAMc1251c7bfee2	-0.018998867	High	648.5420945	0	PR	CR/PR	CR/PR/SD
SAMc1b27bc16435	-0.161827558	Low	725.4209446	0	PR	CR/PR	CR/PR/SD
SAMc2a1820d4e6b	-0.064626481	High	621.9301848	0	CR	CR/PR	CR/PR/SD
SAMc57eadb2d82b	-0.123678615	Low	240.4928131	1	PD	SD/PD	PD

SAMc692536a795a	-0.076917318	High	169.5277207	1	PD	SD/PD	PD
SAMc6eff056c89a	-0.038384954	High	54.20944559	1	PD	SD/PD	PD
SAMc919aebc7fdd	-0.162875528	Low	621.9301848	0	PR	CR/PR	CR/PR/SD
SAMcabb6d58ff55	-0.030775053	High	608.1314168	0	SD	SD/PD	CR/PR/SD
SAMcb132b0cdd2c	-0.05426008	High	75.89322382	1	PD	SD/PD	PD
SAMcc4675f394a1	0.326322163	High	93.63449692	1	PD	SD/PD	PD
SAMcc7a42d87e9c	-0.098139462	Low	516.4681725	0	PR	CR/PR	CR/PR/SD
SAMce39dd79b441	-0.000304742	High	188.2546201	1	PD	SD/PD	PD
SAMcf018fee2acd	-0.160220371	Low	486.899384	1	PD	SD/PD	PD
SAMd027124354ce	-0.064337793	High	705.7084189	0	CR	CR/PR	CR/PR/SD
SAMd135d5867fe3	-0.065350533	High	108.4188912	1	PD	SD/PD	PD
SAMd1bd63734394	-0.052267296	High	58.15195072	1	PD	SD/PD	PD
SAMd215b503f99a	-0.068400473	High	51.25256674	0	SD	SD/PD	CR/PR/SD
SAMd2492b2a31bb	-0.036035442	High	235.5646817	1	PD	SD/PD	PD
SAMd35318127278	-0.101828011	Low	647.5564682	0	CR	CR/PR	CR/PR/SD
SAMd3601288319e	-0.14154358	Low	693.8809035	0	CR	CR/PR	CR/PR/SD
SAMd3bd67996035	-0.154497804	Low	624.8870637	0	CR	CR/PR	CR/PR/SD
SAMd43f8933066b	-0.098060461	Low	600.2464066	0	PR	CR/PR	CR/PR/SD
SAMd4c0837b0997	-0.061009596	High	161.6427105	1	PD	SD/PD	PD
SAMd636e3461955	-0.035345376	High	314.4147844	1	PD	SD/PD	PD
SAMd697ba701077	-0.167119503	Low	634.7433265	1	SD	SD/PD	CR/PR/SD
SAMd7d57ee3a863	-0.014404612	High	608.1314168	0	CR	CR/PR	CR/PR/SD
SAMd86389d0d768	-0.175737575	Low	66.03696099	1	PD	SD/PD	PD
SAMd98bac0a070f	-0.091116118	Low	62.09445585	1	PD	SD/PD	PD
SAMda4d892fddc8	-0.035802579	High	423.8193018	0	PD	SD/PD	PD
SAMdab9ca8fb5de	-0.105667258	Low	5.9137577	0	PD	SD/PD	PD
SAMdad5c29dc105	-0.083030836	Low	72.93634497	1	PD	SD/PD	PD
SAMdb3f50c9129c	-0.046830154	High	474.0862423	1	PD	SD/PD	PD
SAMdcae54fcd7fa	-0.033510948	High	187.2689938	1	SD	SD/PD	CR/PR/SD
SAMdee1011782cd	-0.15939803	Low	117.28952771		PD	SD/PD	PD
SAMdf3e42c8672a	-0.034539406	High	219.7946612	1	PD	SD/PD	PD
SAMe07c4560772d	-0.116627105	Low	63.08008214	0	PD	SD/PD	PD
SAMe0c49ea0df5d	-0.085126219	Low	124.18891171		SD	SD/PD	CR/PR/SD

SAMe1eb5d988760	-0.116618804	Low	631.7864476	0	CR	CR/PR	CR/PR/SD
SAMe3210d3632b4	-0.048135846	High	65.0513347	1	PD	SD/PD	PD
SAMe3d4266775a9	-0.164549919	Low	419.8767967	0	PD	SD/PD	PD
SAMe41b1e773582	-0.016848333	High	25.62628337	1	PD	SD/PD	PD
SAMe50d15fde368	-0.141468788	Low	566.73511290		PR	CR/PR	CR/PR/SD
SAMe56c96c51190	-0.041379036	High	292.7310062	1	PD	SD/PD	PD
SAMe5bc41772bc9	0.015249834	High	183.3264887	1	PD	SD/PD	PD
SAMe712352fb82a	-0.050993058	High	663.3264887	0	PR	CR/PR	CR/PR/SD
SAMe7bcab05402e	-0.123538038	Low	237.5359343	1	SD	SD/PD	CR/PR/SD
SAMe7bf6c015192	-0.022785966	High	93.63449692	1	PD	SD/PD	PD
SAMe7e4f7c076a7	-0.029255435	High	59.137577	1	PD	SD/PD	PD
SAMe9475f77504b	-0.036441554	High	559.835729	0	SD	SD/PD	CR/PR/SD
SAMe94c30c30616	-0.099106241	Low	461.2731006	1	SD	SD/PD	CR/PR/SD
SAMe97af0feefdf	-0.097948132	Low	333.1416838	1	PD	SD/PD	PD
SAMe9ae8beb82fa	-0.141046899	Low	164.5995893	1	PD	SD/PD	PD
SAMeaa477a5384b	-0.120073914	Low	647.5564682	0	CR	CR/PR	CR/PR/SD
SAMeb29625f76a5	-0.067527073	High	93.63449692	1	PD	SD/PD	PD
SAMeb587a68006b	-0.044552713	High	620.9445585	0	SD	SD/PD	CR/PR/SD
SAMee3844cc0b9f	-0.094029592	Low	96.59137577	1	PD	SD/PD	PD
SAMef0e3d2415fd	-0.103397786	Low	303.5728953	1	SD	SD/PD	CR/PR/SD
SAMeff2ce356ccb	-0.069484373	High	242.4640657	1	PD	SD/PD	PD
SAMf20b827dca51	-0.088001992	Low	131.0882957	1	PD	SD/PD	PD
SAMf275eb859a39	-0.160831482	Low	734.29158110		PR	CR/PR	CR/PR/SD
SAMf28c01545593	-0.101344911	Low	192.1971253	1	SD	SD/PD	CR/PR/SD
SAMf2aae1443f67	0.015467792	High	310.4722793	1	PD	SD/PD	PD
SAMf3a9bce50099	-0.111428552	Low	63.08008214	0	PD	SD/PD	PD
SAMf82bbdc267c8	-0.115251537	Low	93.63449692	1	PD	SD/PD	PD
SAMfb7aec7cb0e2	-0.112849318	Low	676.1396304	0	PR	CR/PR	CR/PR/SD
SAMfd947610629d	-0.140825483	Low	271.0472279	1	PD	SD/PD	PD
SAMfed609955db9	-0.117998662	Low	617.0020534	0	PD	SD/PD	PD
SAMffa5c7cad0e5	-0.147715351	Low	466.201232	1	PR	CR/PR	CR/PR/SD
